# mRNA-seq Analysis of the *Gossypium* arboreum transcriptome Reveals Tissue Selective Signaling in Response to Water Stress during Seedling Stage

**DOI:** 10.1371/journal.pone.0054762

**Published:** 2013-01-28

**Authors:** Xueyan Zhang, Dongxia Yao, Qianhua Wang, Wenying Xu, Qiang Wei, Chunchao Wang, Chuanliang Liu, Chaojun Zhang, Hong Yan, Yi Ling, Zhen Su, Fuguang Li

**Affiliations:** 1 State Key Laboratory of Cotton Biology, Institute of Cotton Research, Chinese Academy of Agriculture Sciences (CAAS), Anyang, Henan, China; 2 State Key Laboratory of Plant Physiology and Biochemistry, College of Biological Sciences, China Agricultural University, Beijing, China; New Mexico State University, United States of America

## Abstract

The cotton diploid species, *Gossypium arboreum*, shows important properties of stress tolerance and good genetic stability. In this study, through mRNA-seq, we *de novo* assembled the unigenes of multiple samples with 3h H_2_O, NaCl, or PEG treatments in leaf, stem and root tissues and successfully obtained 123,579 transcripts of *G. arboreum*, 89,128 of which were with hits through BLAST against known cotton ESTs and draft genome of *G. raimondii*. About 36,961 transcripts (including 1,958 possible transcription factor members) were identified with differential expression under water stresses. Principal component analysis of differential expression levels in multiple samples suggested tissue selective signalling responding to water stresses. Venn diagram analysis showed the specificity and intersection of transcripts’ response to NaCl and PEG treatments in different tissues. Self-organized mapping and hierarchical cluster analysis of the data also revealed strong tissue selectivity of transcripts under salt and osmotic stresses. In addition, the enriched gene ontology (GO) terms for the selected tissue groups were differed, including some unique enriched GO terms such as photosynthesis and tetrapyrrole binding only in leaf tissues, while the stem-specific genes showed unique GO terms related to plant-type cell wall biogenesis, and root-specific genes showed unique GO terms such as monooxygenase activity. Furthermore, there were multiple hormone cross-talks in response to osmotic and salt stress. In summary, our multidimensional mRNA sequencing revealed tissue selective signalling and hormone crosstalk in response to salt and osmotic stresses in *G. arboreum*. To our knowledge, this is the first such report of spatial resolution of transcriptome analysis in *G. arboreum*. Our study will potentially advance understanding of possible transcriptional networks associated with water stress in cotton and other crop species.

## Introduction

Cotton is an essential crop for producing fiber used in textiles and is also a major oil source. Cotton yield is dramatically reduced under drought and high salinity conditions [1], [2], [3], [4], [5], [6]. Water stress (mainly including both salt and drought stresses) is a major environmental stress that many plants have to cope with during their whole life cycle [7], [8], [9], [10], [11], [12]. The water stress signals stimulate leaf abscission [13], and enhance root extension into deeper and moist soil, adjusting the root system architecture (RSA) [14]. There is a functional balance between root-based water uptake and shoot-based photosynthesis [15], [16], [17]. Generally, high salinity disturbs cytoplasmic K^+^/Na^+^ homeostasis and can result in ion toxicity and osmotic stress, as well as altering growth regulation, etc [8], [9], [10], [18], [19].

Compared to stress-susceptible species such as the model plant Arabidopsis (*Arabidopsis thaliana*), cotton is moderately to fairly salt tolerant [20]. In agriculture, plant breeders normally use two tetraploid species (*Gossypium hirsutum* L. and *G. barbadense* L.) and two diploid species (*G. arboreum* L. and *G. herbaceum* L.). The diploid species, especially Asiatic desi cotton (*G. arboreum*), commonly called tree cotton, can be cultivated in severely dry and hot climates, and shows great potential against abiotic and biotic stresses, with good genetic stability and important property in stress tolerance [4], [21], [22]. *Gossypium arboreum* is an essential source of stress resistance genes [23], e.g. one heat-shock protein GHSP26 from *G. arboreum* was introduced into *G. hirsutum* and transgenic cotton plants showed an enhanced drought tolerance phenotype [24]. Recently, two research groups constructed *G. arboreum* cDNA libraries: one related to drought stress [25], and the other concerning biotic and abiotic stress up-regulated ESTs [23]. Some previous studies have reported possible mechanisms related to cotton water-stress response [15], [16], [22], [26], [27], [28], [29], [30], [31], [32], [33], [34]. However, the possible regulatory pathways involved in water stress are not well understood in cotton. When exposed to water stress, many plant genes are induced to directly protect against stress or regulate expression of other target genes. Plant transcriptome mapping studies have become a powerful way to reveal the possible mechanism involved in water stress and to dissect the water stress signal transduction pathways and predict genes with biological functions. [32], [35], [36], [37], [38], [39], [40], [41], [42], [43], [44], [45], [46], [47], [48], [49], [50].

The key aim of transcriptomics is to catalog all species of transcripts and quantify the changing expression levels of each transcript during development and under different environmental conditions. Microarrays have already become a main platform for profiling gene expression. During the past decade, ≥100 publications have used microarrays to study transcriptomic responses to water stress in about 28 plant species [37]. Our previous work showed an overview of the transcription map of cotton (*G. hirsutum*) roots under salt stress [51]. In addition, increasing number of groups have studied the spatiotemporal dynamic regulation of transcriptional responses to environmental stimuli. For example, Kreps et al. used microarrays to study the transcriptome changes for Arabidopsis in response to salt, osmotic and cold stresses in leaves and roots after 3- and 27-h stress treatments [40]. Nevertheless, the development of high-throughput technology advanced transcriptome analysis for environmental stress together with cell and developmental-stage-specific profiling, leading to identification of high-confidence transcriptional modules. For example, Dinneny et al. developed a comprehensive view of cell-type-specific abiotic stress responses [36]. Their results indicated that the cell identity mediates the abiotic stress response in Arabidopsis roots by studying the transcriptional response to high salinity and iron deprivation in different Arabidopsis root cell layers and developmental stages. Thus, during transcriptome analysis, the spatial and temporal dynamic changes should be considered.

Microarray data involves thousands of plant samples and this platform is anticipated to have a wide range of applications in future transcriptome studies. However there are some limitations during microarray-based transcriptome analysis, e.g. relatively lower intensity, lower dynamic range, higher background, some non-specific hybridization, and biases of labeling. In the meanwhile, the defined probe sets of microarrays should use existing genome sequences as reference. The recent application of massively parallel cDNA sequencing (RNA-seq) has complemented microarray-based methods for characterization and quantification of the transcriptome, providing more complete descriptions of transcriptomes and more efficient ways to measure transcriptome data with deep coverage and base-level resolution in different organisms [37], [52], [53], [54], [55], [56], [57], [58], [59], [60], [61], [62], [63], [64], [65]. RNA-seq can also be used on a much wider range of species in studies of water stress, especially for some plant species whose whole genome sequences are not finished yet.

In this study, the diploid cotton species, *G. arboreum*, was selected for transcriptome analysis due to its important properties of stress tolerance. To elucidate possible mechanisms regulating the water stress response of *G. arboreum*, we applied Illumina sequencing technology based mRNA deep sequencing (mRNA-seq) to *de novo* construct transcriptome profiling and to gain a more comprehensive understanding of transcriptional processes during water stress in cotton seedlings.

## Results

### De Novo mRNA-seq Assembly Across Different Expression Levels of Leaf, Stem and Root Tissues Under Normal and Water Stress Conditions

The cotton genotype, *G. arboreum* cv. Shixiya, was chosen for this study because of its great potential against abiotic and biotic stresses. The seedling plants were treated by 17% polyethylene glycol (PEG) and 150 mM NaCl (water as mock, CK) for 3 hours, and three tissues including root, stem (including hypocotyl), and leaf, were respectively harvested for mRNA-seq analysis. The experimental design and mRNA-seq procedures are shown in Fig. S1. The total RNA of each sample was isolated individually, and the transcriptome profiles generated through the standard Illumina protocol (detailed description in Materials and Methods). To maximize transcript coverage, we pooled the Illumina read sequences from nine biological conditions during the *G. arboretum* seedling stage: leaf, stem and root tissues treated by all of CK, PEG, and NaCl, respectively. We obtained approximately total 271.6 million clean reads (or 135.8 million paired-end reads) and total roughly 23 Gb nucleotides which passed the Illumina quality filtering (the number of clean reads for each sample is shown in Table 1).

**Table 1:** The data quality of mRNA-seq and assembly.

**Samples**	**Total reads**	**Total nucleotides (nt)**	**All contig**	**All scafford**	**All unigene**	**Length of all unigene (nt)**
**Leaf-Mock**	2.671E+07	2.404E+09	334,935	98,372	60,608	38,613,473
**Leaf-PEG**	2.756E+07	2.480E+09	484,539	111,592	66,371	35,425,037
**Leaf-NaCl**	2.673E+07	2.406E+09	481,884	109,592	65,608	37,796,363
**Stem-Mock**	2.676E+07	2.408E+09	378,039	113,832	71,548	35,658,125
**Stem-PEG**	3.852E+07	3.467E+09	415,789	126,267	76,860	38,206,259
**Stem-NaCl**	2.960E+07	2.664E+09	368,169	113,447	70,597	34,352,402
**Root-Mock**	3.229E+07	2.422E+09	180,867	96,615	57,723	29,210,215
**Root-PEG**	3.107E+07	2.330E+09	163,186	91,888	56,054	29,180,862
**Root-NaCl**	3.237E+07	2.428E+09	175,985	93,498	56,780	27,481,197

**Figure 1:**
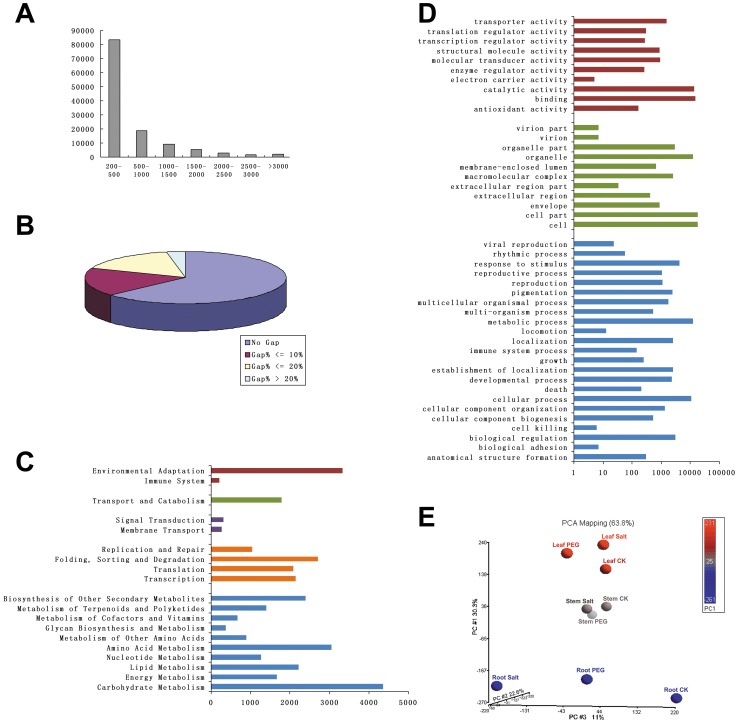
Data quality and annotation of transcripts assembled with mRNA-seq. **A:** Length distribution of transcripts. **B:** Gap distribution of transcripts. **C:** Function annotation of the transcripts based on KEGG classification. The numbers of transcripts mapped to each pathway group are shown in the bar chart. The color indicates different KEGG categories: blue for metabolism, orange for genetic information processing, purple for environmental information processing, green for cellular processes, and red for organismal systems. **D:** Gene ontology (GO) classification of the transcripts. Each bar represents the number of transcripts mapped to each GO category. The color indicates different GO categories: blue for biological process (BP), green for cellular component (CC), and red for molecular function (MF). **E:** Principle components analysis (PCA) for the samples based on the raw reads of the transcripts. The red balls represent leaf samples, the gray balls represent stem samples, and the blue balls represent root samples. The color indicates the number range in PC #1 (Principal Component 1) shown in the color bar.

The cotton whole-genome sequencing results are not publically available, thus the *de novo* assembly was carried out using SOAPdenovo, a short reads assembling program [66]. SOAPdenovo firstly combined clean reads from each sample with 29-mer overlap to form contigs; secondly connected the contigs to make scaffolds with the insertion information of the paired-end reads; then sequence clustering software (TGICL: http://sourceforge.net/projects/tgicl/) was used to connect scaffolds to unigenes which could not be extended on either end; finally, unigenes from each sample’s assembly were taken into TGICL again to acquire non-redundant All-Unigenes (here termed ‘transcripts’). The number of contigs, scaffolds, and unigenes for each sample are shown in Table 1. There were 56–76 k unigenes for each sample and the total length of assembly unigenes were 27–38 M, and the sequence depth of each sample was from 62× to 90×. In total, we got 123,579 transcripts with lengths ≥200 bp. The total length of all transcripts was approximately 76.6 Mb (we obtained sequence depth of about 300×), the N50 was 1,065 bp, and there were 21,253 transcripts of ≥1 kb in length. The length distribution of these 123,579 transcripts is shown in Fig. 1A and gap distribution in Fig. 1B. There were ≥60% transcripts without gaps (Ns), and ≤5% of transcripts with ≥20% gaps.

### Functional Annotation of the Assembled Cotton Transcripts

Following the mRNA-seq *de novo* assembly, the functional annotation process for these transcripts was mainly based on homolog search. To obtain the possible annotation and predict the sequence direction, BLAST (blastx alignment, e-value cutoff as 10^−6^) was used to search the best aligning results for transcripts against protein databases like nr (in NCBI), Swiss-Prot (in UniProt), KEGG (www.genome.jp/kegg/) and COG (http://www.ncbi.nlm.nih.gov/COG/). For transcripts with no homolog hit, ESTScan [67] was applied to predict the coding regions as well as to determine sequence direction. In the total of 123,579 transcripts, there were 81,369 sequences with determinable direction. In addition, we compared the transcripts with known cotton ESTs from NCBI and DFCI (http://compbio.dfci.harvard.edu/tgi/plant.html); there were 75,855 transcripts matching the known cotton ESTs. There were also 74,573 transcripts matching the protein-coding genes in the recently published draft genome of *G. raimondii* [68]. Take this into account, there were 34,451 remaining transcripts which may be considered as newly discovered transcripts.

**Figure 2:**
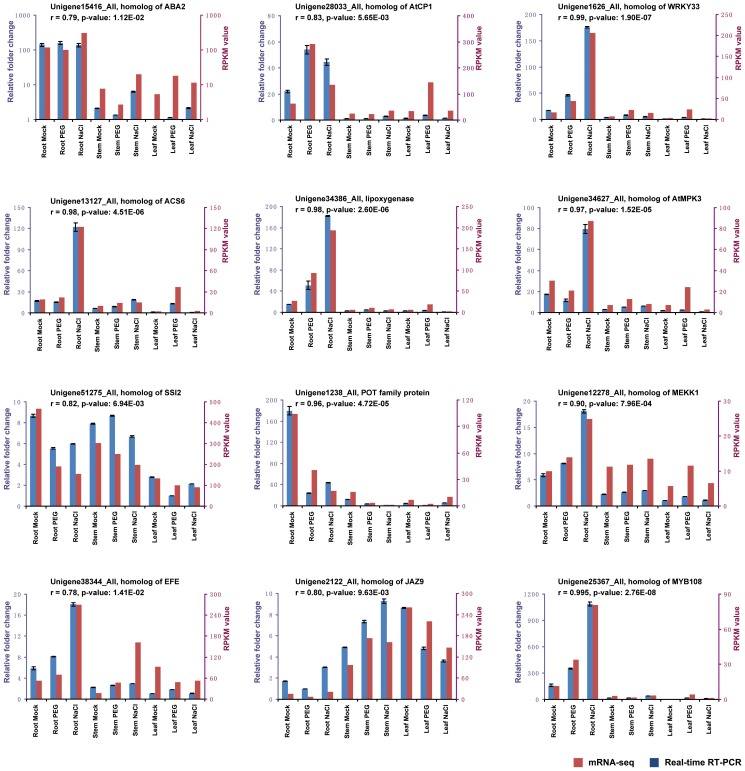
Real-time RT-PCR validation for selected transcripts. Twelve transcripts were selected for real-time RT-PCR to validate the expression patterns in different samples and treatments. The blue bars represent the relative intensity of real-time RT-PCR from independent biological replicates (using the left y-axis), the red bars represent the expression level (RPKM) of the transcript (using the right y-axis). The correlation coefficient (r) and its *P*-value between the RT-PCR values and RPKMs for each transcript are listed in each individual chart. The transcripts are: Unigene15416_All hits AT1G52340.1 (ABA2); Unigene28033_All hits AT5G49480.1 (ATCP1); Unigene1626_All hits AT2G38470.1 (WRKY33); Unigene13127_All hits AT4G11280.1 (ACS6); Unigene34386_All hits AT1G72520.1 (lipoxygenase); Unigene34627_All hits AT3G45640.1 (ATMPK3); Unigene51275_All hits AT2G43710.1 (SSI2); Unigene1238_All hits AT1G32450.1 (POT family protein); Unigene12278_All hits AT4G08500.1 (MEKK1); Unigene38344_All hits AT1G05010.1 (EFE); Unigene2122_All hits AT1G70700.3 (JAZ9); and Unigene25367_All hits AT3G06490.1 (MYB108). The real-time RT-PCR primers for each transcript are listed in Table S1.

KEGG annotation provides information of transcripts related to metabolic process and functions in cellular processes. We summarized the KEGG pathway distribution of the transcripts (Fig. 2C), which showed that 20,071 transcripts (several transcripts hit multiple pathways) mapped to 117 pathways belong to all five categories of KEGG, including metabolism, genetic information processing, environmental information processing, cellular processes, and organismal systems.

Gene ontology (GO) is an international standardized gene functional classification which can provide a biological foundation on global characterization of *de novo* assembly transcripts. With nr annotation, we used Blast2GO program [69] to obtain GO annotation of the transcripts. There were 20,008 transcripts with GO annotation (total 96,911 matches). The major categories of GO category distribution were Biological Process (BP), Molecular Function (MF), and Cellular Component (CC) (Fig. 2D).

To compare the expression level of individual transcripts in different samples, we used the *de novo* assembled sequences as a reference for short-read mapping. SOAPaligner/soap2, a short-read alignment program [66], was used to map the uniquely aligned reads on to the 123,579 transcript sequences. To eliminate the influence of different transcript length and sequencing level on the calculation, the RPKM method (Reads Per kb per Million reads) [70] was used for normalization and the result directly used for comparing the difference of gene expression between samples. Of individual samples, about 60–70% of transcripts could be detected, in which there at least one read was uniquely aligned to the transcript sequence.

### Principal Component Analysis (PCA) of Differential Expression Levels in Multiple Samples and Real-time RT-PCR Validation

During mRNA-seq transcriptome analysis, the spatial resolution of cotton response to water stress was investigated due to the tissues exhibiting different levels of gene expression. The transcripts fell along the diagonal region of pair-wise scatter plots between the nine samples (Fig. S2) indicating no major variation between the pairs; whereas some transcripts fell above or below diagonal lines, indicating their differential expression level during different tissue sample and treatment conditions. The largest difference among the nine samples was observed between tissues. The principal component analysis (PCA) for the samples, based on the raw expression level (Fig. 1E), showed the first three principal components accounted for 63.8% of the variation; all three treatments (mock, PEG, and NaCl) in each cotton tissue (leaf, stem, and root) were clustered together in different vertical planes, the distances between treatments in root samples were much greater than for leaf and stem tissues.

To validate the mRNA-seq results, we selected 12 transcripts with differential expression patterns for real-time RT-PCR analysis and made one-by-one comparisons of each transcript between real-time RT-PCR and mRNA-seq results (Fig. 2). We calculated Pearson correlation coefficient (r) between the real-time RT-PCR values and RPKMs of mRNA-seq across nine samples for each transcript. Among these 12 transcripts, the correlation coefficient range was 0.78–0.995 (*P*≤0.05), and seven transcripts had the correlation coefficient >0.9. The majority of real-time RT-PCR results matched the mRNA-seq expression patterns.

### Differential Expression Analysis of Assembled Cotton Transcripts Under NaCl and PEG Treatments in Different Tissues

We further conducted differential expression analysis for the transcripts responding to PEG and salt stresses in cotton leaf, stem, and root samples, respectively. Referring to Audic’s algorithm [71], we calculated the FDR (False Discovery Rate) based on the *P*-value which corresponds to differential expression tests of transcripts. Using “FDR ≤0.001 and the absolute value of log_2_Ratio ≥1” as the threshold, we identified total 36,961 transcripts either up- or down-regulated under 150 mM NaCl or 17% PEG treatment conditions in at least one tissue sample of cotton leaf, stem, and root. The numbers for six comparisons and the detailed information for each transcript are separately shown in Fig. S2 and Table S2. In cotton leaf samples, 8,981 transcripts were up-regulated and 5,109 transcripts down-regulated under PEG treatment; and 4,884 up-regulated and 3,692 down-regulated under salt treatment. In cotton stem samples, 1,430 transcripts were up-regulated and 1,717 down-regulated under PEG treatment; and 2,462 up-regulated and 3,859 down-regulated under salt treatment. In cotton root samples, 4,137 transcripts were up-regulated and 8,568 down-regulated under PEG treatment; and 6,303 up-regulated and 11,068 down-regulated under salt treatment. Among the three tissues, there were much greater numbers of differentially expressed transcripts under water stress in root samples, and the lowest in stem samples. In cotton root samples, more transcripts were down-regulated by PEG and salt stress than transcripts up-regulated; whereas in leaf samples, more transcripts were up-regulated by PEG and salt stress than those down-regulated.

**Figure 3:**
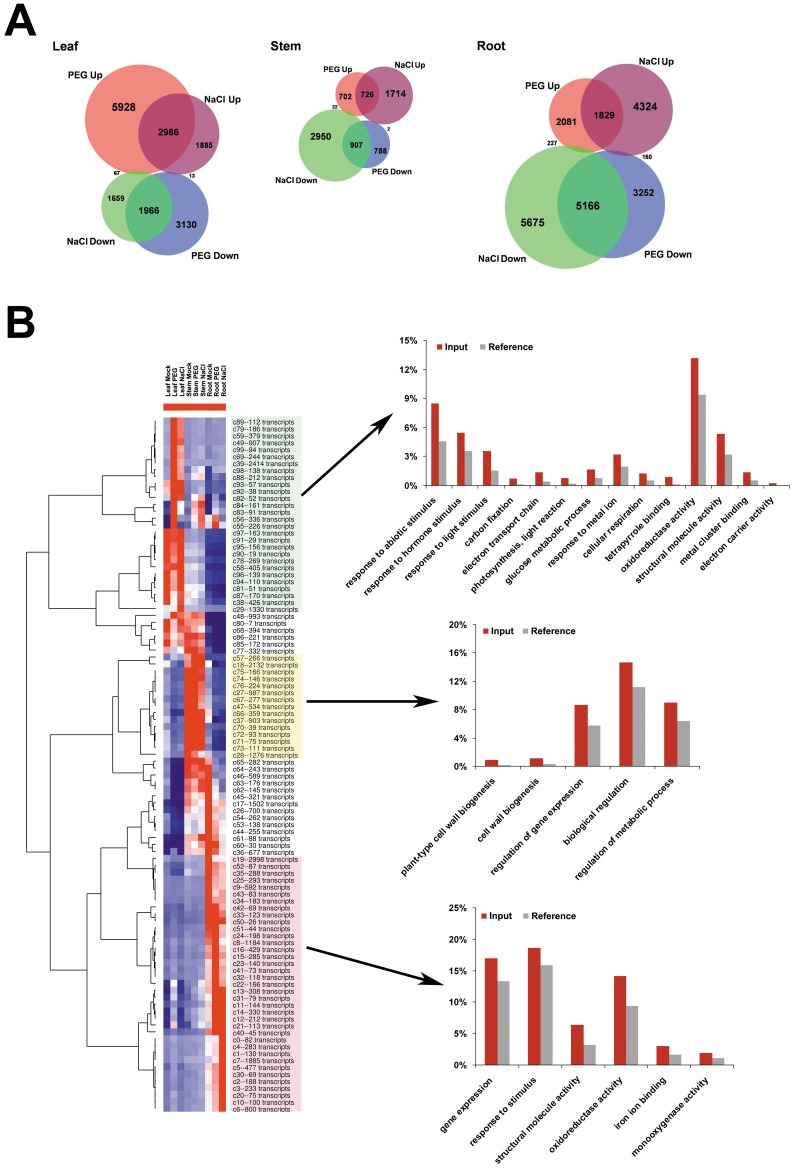
Summary of the differential expression transcripts across cotton tissue samples and treatments. **A:** Venn diagrams illustrate the differential expression transcripts under PEG and NaCl treatment in root, stem, and leaf samples. The red and blue colors represent the up-regulated and down-regulated transcripts under PEG treatment, respectively. The purple and green colors represent the up-regulated and down-regulated transcripts under NaCl treatment, respectively. **B:** Cluster and gene ontology (GO) analysis of the transcripts’ response to PEG or NaCl treatments in different cotton tissues. The overview hierarchical cluster result of the centroids of SOM cluster (listed in Fig. S4), the red (high) and blue (low) colors represent the relative expression level across the samples. The marked centroid groups represent the transcripts in these clusters preferentially expressed in the leaf (marked in light green background), stem (light yellow background), or root (light purple background) tissue. In the comparison of the enriched GO terms in the transcripts preferentially expressed in the leaf, stem, or root tissue, the red bars represent the percentage of transcripts belonging to enriched terms in the query list (Input), whereas the gray bars represent the percentage in all transcripts (Reference).

Venn diagram analysis showed the specificity and intersection of transcripts’ response to NaCl and PEG treatments among the leaf, stem, and root tissues (Fig. 3A). For the different tissues, large numbers of the transcripts’ response to NaCl and PEG treatments overlapped in the same direction. For example, in cotton root tissue, the overlap number of up-regulated transcripts was 1,829 (about 44% of the 4,137 PEG up-regulated transcripts); for the down-regulated transcripts, the overlap number was 5,166 transcripts (>60% of the 8,568 PEG down-regulated transcripts); very few numbers (227 plus 150) of transcripts responded to NaCl and PEG treatments in opposite ways. The intersection trends were similar for stem and leaf to that in root tissue.

### Clustering and GO Analysis of the Differentially Expressed Transcripts

To further identify the co-expressed transcripts with similar response patterns to the NaCl and PEG treatments in different tissues, both SOM (self-organized mapping) and hierarchical methods were used for clustering the 36,961 differentially expressed transcripts. A 10×10 SOM cluster was applied to divide the 36,961 transcripts into 100 clusters: the cluster name and the number of transcripts in each cluster were listed, the expression pattern of the centroid presented and the variances shown (Fig. S3). The SOM classified that the 36,961 transcripts into different groups with various centroids, some of which showed very similar patterns. Further classification of these centroids may provide a much clearer picture of tissue selective signalling in response to water stress in cotton. We applied hierarchical method to cluster the centroids (Fig. 3B). A heat map represented the relative expression level of the centroids in nine cotton samples. Intuitively, these centroids could be grouped into multiple clusters, for example, the top 28 centroids (marked in light green; Fig. 3B), represent the transcripts that mainly responded to NaCl or PEG treatment in leaf tissue; whereas the bottom 37 centroids (marked in light purple; Fig. 3B), representing the transcripts that mainly responded to NaCl or PEG treatment in root tissue.

In addition, we applied GO analysis (using agriGO; http://bioinfo.cau.edu.cn/agriGO/) to the selected centroids’ groups (Fig. 3B). Interestingly, many of the enriched GO terms for the selected three groups differed. For the centroids’ groups representing the transcripts that were preferentially expressed in stem tissue (marked in light yellow; Fig. 3B), the enriched GO terms were mainly related to plant cell wall (plant-type cell wall biogenesis, FDR *P*-value: 5.80E−04; and cell wall biogenesis, FDR *P*-value: 4.40E−03) and regulation of metabolic process (FDR *P*-value: 5.60E−03). Regarding the transcripts preferentially expressed in leaf tissue (the 28 clusters including 8914 transcripts), the enriched biology process type GO terms include “response to abiotic stimulus” (FDR *P*-value: 2.00E−10), “response to light stimulus” (FDR *P*-value: 3.90E−07), “response to hormone stimulus” (FDR *P*-value: 2.60E−03), “carbon fixation” (FDR *P*-value: 2.00E−04), “photosynthesis, light reaction” (FDR *P*-value: 1.40E−03), “electron transport chain” (FDR *P*-value: 2.70E−04), “glucose metabolic process” (FDR *P*-value: 1.20E−02), “response to metal ion” (FDR *P*-value: 2.20E−02), “cellular respiration” (FDR *P*-value: 2.40E−02). The enriched molecular function type GO terms include “tetrapyrrole binding” (FDR *P*-value: 1.20E−05), “oxidoreductase activity” (FDR *P*-value: 3.60E−05), “structural molecule activity” (FDR *P*-value: 2.60E−04), “metal cluster binding” (FDR *P*-value: 9.20E−03), “electron carrier activity” (FDR *P*-value: 4.20E−02). Of transcripts preferentially expressed in root tissue, the enriched GO terms mainly include “response to stimulus” (FDR *P*-value: 4.50E−02), “structural molecule activity” (FDR *P*-value: 2.30E−15), “oxidoreductase activity” (FDR *P*-value: 5.00E−14), “iron ion binding” (FDR *P*-value: 6.10E−05), and “monooxygenase activity” (FDR *P*-value: 2.20E−02), etc.

### Transcription Factors Responding to NaCl and PEG Treatments in Different Cotton Tissues

Transcription is a dynamic process and transcription factors are essential for regulation of gene expression. In this study, we also performed global transcription factor classification for differentially expressed transcripts and identified a total of 4,002 transcripts (56 transcription factor families; Table S2). About 49% of transcription factor members responded to NaCl or PEG treatment in cotton tissues. Several key regulatory gene families involved in responding to abiotic and biotic sources of stress such as AP2-EREBP (62.03%), WRKY (61.78%), ABI3VP1 (58.73%), Tify (55.88%), bHLH (55.45%), MYB (54.46%), NAC (52.48%), bZIP (52.50%), EIL (50.00%), HSF (50.00%), and C2C2-YABBY(50.00%), were largely up- or down-regulated under NaCl or PEG in at least one tissue (Table 2). Several transcription factor family genes; e.g. WRKY, NAC, and Tify (including the cotton JAZ genes) were up-regulated by salt and PEG treatments. For example, there were 64 WRKY members up-regulated versus 12 down-regulated under NaCl treatment in root tissue; there were 50 members up-regulated versus nine down-regulated under PEG treatment in leaf tissue. In some other transcription factor families, e.g. C2C2-GATA, more family members were down-regulated by NaCl and PEG treatments. For MYB in root tissue, the up- and down-regulated members were evenly balanced.

**Table 2:** Transcription factor (TF) members that responded to PEG and NaCl treatments in cotton tissues.

Transcription	Total	Changed	Change	Leaf		Stem		Root	
factor family	number	members (%)		NaCl	PEG	NaCl	PEG	NaCl	PEG
AP2-EREBP	316	196 (62.03%)	Up	23	74	37	23	90	51
			Down	32	19	19	11	21	35
WRKY	191	118 (61.78%)	Up	9	50	21	17	64	48
			Down	7	9	8	6	12	7
GRF	44	27 (61.36%)	Up	2	7	0	4	2	1
			Down	11	7	6	2	5	12
C2C2-GATA	54	33 (61.11%)	Up	1	6	1	1	0	2
			Down	10	5	7	1	15	18
CSD	18	11 (61.11%)	Up	3	4	0	1	1	3
			Down	2	2	1	0	3	3
ABI3VP1	63	37 (58.73%)	Up	3	5	1	2	5	5
			Down	4	6	5	3	11	11
DBP	12	7 (58.33%)	Up	0	4	2	1	7	3
			Down	0	0	0	0	0	0
DBB	19	11 (57.89%)	Up	0	2	1	0	6	4
			Down	1	2	0	0	1	1
zf-HD	26	15 (57.69%)	Up	0	2	0	3	4	2
			Down	2	1	5	1	0	0
Tify	34	19 (55.88%)	Up	6	9	5	2	12	1
			Down	0	0	0	0	0	4
bHLH	330	183 (55.45%)	Up	8	21	18	11	35	26
			Down	30	39	39	14	42	49
C2C2-CO-like	67	37 (55.22%)	Up	4	8	5	6	10	8
			Down	2	7	10	4	2	5
ARR-B	40	22 (55.00%)	Up	2	1	5	1	3	0
			Down	4	4	2	1	7	8
MYB	303	165 (54.46%)	Up	25	41	25	17	43	38
			Down	14	21	29	10	45	37
PLATZ	32	17 (53.13%)	Up	5	7	0	0	4	2
			Down	1	2	2	1	5	6
bZIP	160	84 (52.50%)	Up	11	26	12	8	23	23
			Down	15	16	18	12	16	5
NAC	202	106 (52.48%)	Up	15	37	18	8	38	45
			Down	9	9	15	11	20	18
C2C2-YABBY	12	6 (50.00%)	Up	0	0	0	3	0	0
			Down	2	3	0	0	0	0
EIL	22	11 (50.00%)	Up	1	9	0	0	1	2
			Down	0	0	0	0	0	0
HSF	66	33 (50.00%)	Up	8	12	5	3	15	10
			Down	4	1	3	1	4	4
C2C2-Dof	87	43 (49.43%)	Up	4	10	3	0	5	2
			Down	5	13	14	3	11	4
TCP	55	27 (49.09%)	Up	1	3	0	0	1	0
			Down	5	9	11	0	8	4
G2-like	100	48 (48.00%)	Up	3	6	1	0	15	7
			Down	4	10	8	5	12	9
SBP	55	26 (47.27%)	Up	4	4	0	1	4	2
			Down	0	3	12	1	12	1
GRAS	140	66 (47.14%)	Up	3	13	9	5	15	10
			Down	11	14	11	3	24	15
C2H2	245	114 (46.53%)	Up	10	17	9	16	34	21
			Down	15	24	18	12	36	23
HB	282	127 (45.04%)	Up	11	36	10	8	29	24
			Down	15	16	29	5	30	21
LIM	23	10 (43.48%)	Up	2	3	0	0	0	0
			Down	1	2	3	0	5	3
MADS	67	29 (43.28%)	Up	10	9	10	8	5	4
			Down	1	2	3	3	4	3
MYB-related	137	57 (41.61%)	Up	8	11	5	2	18	9
			Down	7	11	8	7	13	12
CCAAT	72	29 (40.28%)	Up	5	11	2	1	4	8
			Down	2	2	3	1	9	2
TAZ	18	6 (33.33%)	Up	1	0	1	0	5	0
			Down	0	0	0	0	0	1
CAMTA	22	7 (31.82%)	Up	0	2	0	0	4	2
			Down	0	1	0	0	1	0
E2F-DP	22	7 (31.82%)	Up	0	0	0	0	0	0
			Down	5	3	2	0	3	3
C3H	202	64 (31.68%)	Up	9	24	9	7	9	3
			Down	3	9	1	2	13	15
ARF	94	28 (29.79%)	Up	1	3	2	1	6	0
			Down	3	5	5	1	9	7
BES1	14	4 (28.57%)	Up	0	1	0	0	0	0
			Down	0	0	3	0	1	1
Trihelix	62	17 (27.42%)	Up	0	2	1	1	6	4
			Down	3	3	1	1	6	5
Other	294	111 (37.76%)	Up	7	21	6	4	25	14
			Down	13	14	21	2	28	22
**Total**	**4002**	**1958 (48.93%)**	**Up**	**205**	**501**	**224**	**165**	**548**	**384**
			**Down**	**243**	**294**	**322**	**124**	**434**	**374**

### Transcripts Related to Hormone Signaling Pathways, Responding to NaCl and PEG Treatment in Different Cotton Tissues

**Figure 4:**
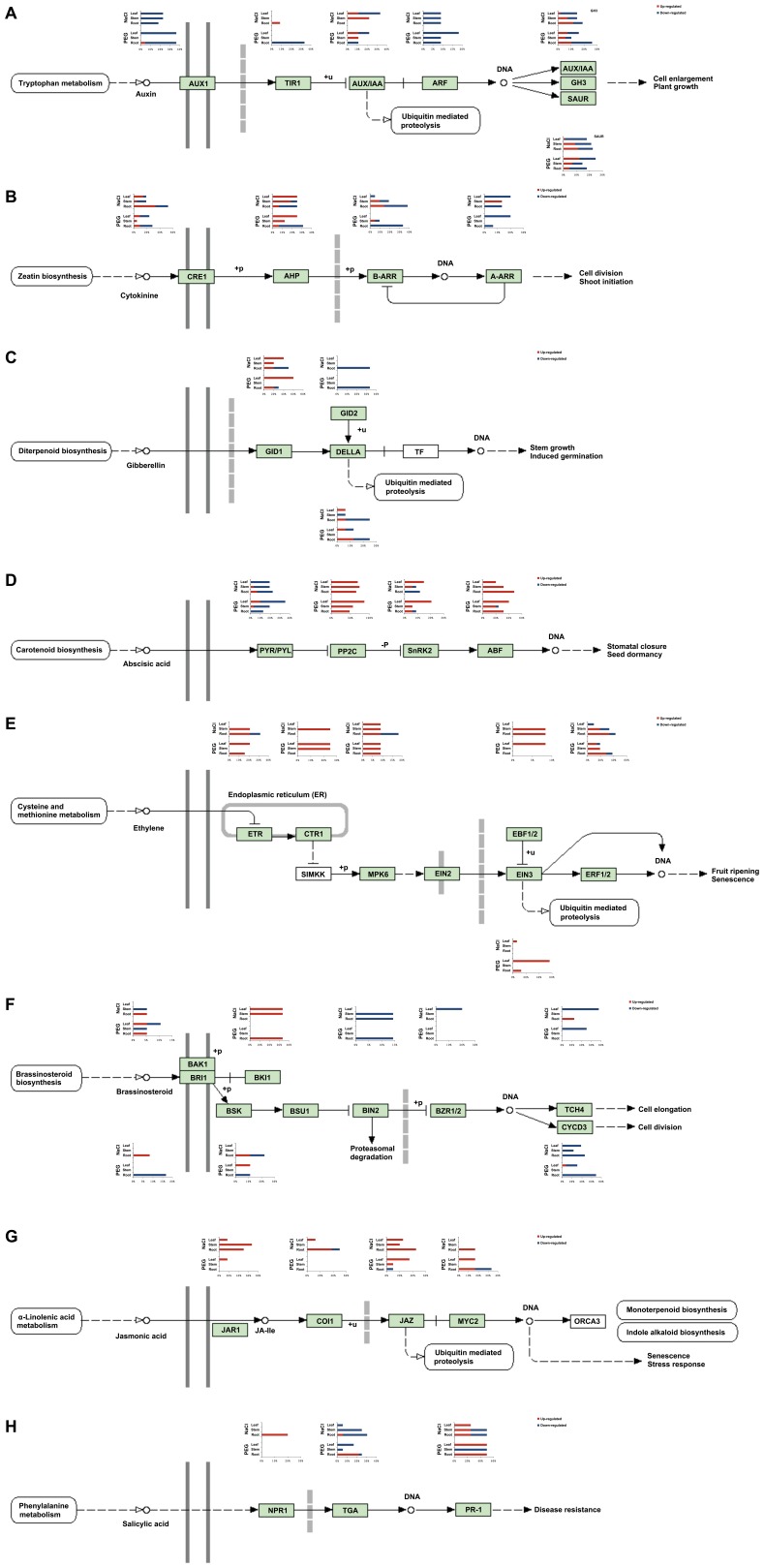
Summary of transcripts related to plant hormone signal transduction pathways and their response to PEG and NaCl treatments across cotton tissue samples. The colored bars represent the percentage of the transcripts in each bin (re-annotated to MapMan classification) whether up-regulated (red) or down-regulated (blue) under PEG or NaCl treatment in different cotton tissues. **A**: represents the differential expression transcripts related to auxin (IAA) signalling transduction pathway. **B**: represents the differential expression transcripts related to cytokinin (CK) signalling transduction pathway. **C**: represents the differential expression transcripts related to gibberellin (GA) signalling transduction pathway. **D**: represents the differential expression transcripts related to abscisic acid (ABA) signalling transduction pathway. **E**: represents the differential expression transcripts related to ethylene signalling transduction pathway. **F**: represents the differential expression transcripts related to brassinosteroid (BR) signalling transduction pathway. **G**: represents the differential expression transcripts related to jasmonate (JA) signalling transduction pathway. **H**: represents the differential expression transcripts related to salycylic acid (SA) signalling transduction pathway.

Phytohormones play essential roles in the ability of plants to adapt to water stress by mediating various adaptive responses. We mapped the assembled mRNA-seq transcripts to eight plant hormones’ signalling transduction pathways in KEGG, including auxin, cytokinine (CK), gibberellin (GA), abscisic acid (ABA), ethylene, brassinosteroid (BR), jasmonate (JA), and salycylic acid (SA). The overview of gene expression patterns under NaCl and PEG treatment in three cotton tissues (Fig. 4) showed the proportion of the transcripts related to hormone signaling up-regulated and down-regulated under NaCl and PEG treatments in cotton tissues.

ABA signaling and ABA- responsive genes were among the most studied topics in the response of plants to water stress. In the ABA signal transduction pathway, a large number of assembled cotton transcripts showed significantly differential expression under NaCl and PEG treatments with no tissue selectivity (Fig. 4D). The majority of changed *PYR*/*PYL* homologs were down-regulated, while most *PP2C* and *ABF* homologs were up-regulated under NaCl and PEG treatments in all cotton seedling tissues. Many homologs of *SnRK2* were up-regulated by NaCl and PEG in leaf tissue, and mainly down-regulated by NaCl in root tissue.

As well as the well-known stress-responsive ABA, other plant hormones are also involved in salt and osmotic stresses; and the roles of other hormones during water stress are emerging. In the auxin signal transduction pathway (Fig. 4A), we found that most homologs of *ARF* and *AUX1* were down-regulated under both NaCl and PEG treatments; transcripts for *TIR1* were up-regulated by NaCl and down-regulated by PEG in root tissue. Recently, an interactive feedback loop between auxin and CK signaling was discovered, possibly balancing CK and auxin concentration in developing root and shoot tissues. The *AHP* transcripts showed tissue selectivity during NaCl and PEG treatments, with up-regulation in leaf tissue and most down-regulation in root tissue. The majority of changed *ARR* transcripts were down-regulated (Fig. 4B). For both GA and BR signals, very few genes were differentially expressed under NaCl and PEG treatments in stem tissue. Although there were relatively lower numbers of differentially expressed transcripts in our data sets for the GA signal transduction pathway (Fig. 4C), the transcripts of *GID1* were significantly up-regulated under both NaCl and PEG treatments in leaf tissue. For the BR signal transduction pathway (Fig. 4F), the changed transcripts showed tissue selectivity and were mostly down-regulated under NaCl and PEG treatments, except that *BKI1* was up-regulated.

For the ethylene signal transduction pathway (Fig. 4E), the majority of changed transcripts were up-regulated, e.g. homologs of *ETR*, *CTR*, *MPK*, and *EIN3*. There was also tissue selectivity: e.g., the most differentially expressed *ETR* homologs were up-regulated under NaCl treatment in stem and root, but not leaf tissue; while in response to PEG treatment, the changed *ETR* homologs were only up-regulated in leaf and root tissues. Many *EIN3* homologs were up-regulated by PEG in leaf tissue, whereas they rarely responded to stresses in other tissues. In the JA signal transduction pathway (Fig. 4G), a large proportion of JA-related transcripts were up-regulated under NaCl in three tissues. However, for PEG treatment, there was tissue selectivity, especially for *JAZ*s, with most changed transcripts up-regulated in leaf and down-regulated in root tissue. Some transcripts for the SA signal transduction pathway also showed differential expression patterns (Fig. 4H), including homologs for *NPR1*, *TGA* and *PR-1*: e.g., the majority of changed *TGA* homologs were down-regulated under NaCl treatment in three tissues; while under PEG treatment, the changed *TGA* homologs were down-regulated in leaf and stem but up-regulated in root tissue. Cotton *NPR1* transcripts were only up-regulated under NaCl treatment in roots. There were up- and down-regulated *PR-1* transcripts under NaCl treatment; however, under PEG treatment, these were up-regulated in both leaf and root and down-regulated in stem tissue. Normally, ethylene, JA and SA form a complex network related to disease resistance and are mainly involved in biotic stress. Our differential expression analysis results indicated crosstalk between abiotic and biotic stresses mediating stress hormones, e.g., ABA, ethylene, JA and SA.

### Comparative Transcriptome Analysis between Cotton and Arabidopsis Transcripts Related to Hormone Signaling Pathways Under Water Stresses

In order to reveal the similarity and uniqueness of cotton drought and salt responses compared to other plant species, we conducted comparative transcriptome analysis between cotton and Arabidopsis responding to different water stresses, mainly focusing on the transcripts related to selected hormone signalling pathways. Through homolog search with BLAST tool, we mapped the cotton transcripts to Arabidopsis genes (TAIR9 version in http://www.arabidopsis.org). There were 61,631 cotton transcripts matching Arabidopsis genes with e-value cutoff as 10^−6^ (listed in Table S2).

The Arabidopsis transcriptome data were from publicly available Arabidopsis AtGenExpress project (stress treatments data were downloaded from GEO, http://www.ncbi.nlm.nih.gov/geo/and TAIR, http://www.arabidopsis.org) and we used the data sets for both root and shoot tissues under 3 h salt and osmotic treatments. The differentially expressed probe sets were calculated and mapped to plant hormone signaling pathways with MapMan. The individual gene expression patterns for the selected hormone signaling pathways (ABA, JA, IAA, and ethylene) were compared between different cotton and Arabidopsis tissues under water stresses (Fig. S4).

In the ABA signal transduction pathway (Fig. S4A), the transcripts in cotton and Arabidopsis were shown with similar proportion trend in the tissues under water stresses. The majority of changed *PYR*/*PYL* genes were down-regulated in Arabidopsis and cotton root and shoot tissues under salt and osmotic stresses. As to *PP2Cs* and *ABFs,* majority of their members in cotton and Arabidopsis were up-regulated by salt and osmotic stresses. These results indicated that the transcripts in cotton shared similar ABA signalling pathway as those in Arabidopsis responding to water stresses, while there was slight difference in the members of SnRK2, the cotton homologous were down-regulated by NaCl in root tissue but the Arabidopsis genes were not responded to salt stress in root tissue.

Like the genes involved in ABA signal transduction pathway, most cotton transcripts related to JA, auxin, and ethylene signalling pathways showed similar expression patterns as those in Arabidopsis, such as *JAZ* and *MYC2* genes in JA signal pathway, *AUX1* and *SAUR* genes in auxin signal pathway, *ETR1* and *ERF1/2* genes in ethylene pathway, etc. However, there were some significant differences compared between cotton and Arabidopsis in response to water stresses. For example, in the JA signal transduction pathway (Fig. S4B), the Arabidopsis *JAR1* and *COI1* genes were not responded to salt and osmotic stresses, whereas their homologous in cotton were mainly up-regulated under NaCl treatment. In the auxin signal transduction pathway (Fig. S4C), many *ARF* transcripts were down-regulated in cotton under water stresses, but there is no *ARF* gene with differential expression under similar conditions. Especially in the ethylene signal transduction pathways (Fig. S4D), there were a lower proportion of gene members responding to water stresses in Arabidopsis than in cotton, including *CTR1*, *MPK6*, *EBF1/2* and *EIN3* genes.

## Discussion

### *De novo* Assembly of *G. arboreum* mRNA-seq Data Sets and Tissue Selectivity of Transcripts’ Response to Water Stress During Cotton Seedling Stage

In this study, through paired-end massively parallel mRNA-seq and transcriptome *de novo* assembly, we assembled the unigenes of nine samples and successfully obtained 123,579 transcripts of *G. arboreum* (N50 = 1065 bp and length ≥200 bp) with about 300× sequence depth, ≥60% transcripts without gaps (Ns) and ≤5% transcripts with ≥20% gaps. Through BLAST against the known cotton ESTs and recently published draft genome of *G. raimondii* [68], there were 89,128 transcripts with hits, and the remaining 34,451 may be considered as novel transcripts. Due to the high proportion of short sequences, the true number of novel transcripts may be lower. Among 123,579 transcripts, there were 20,008 transcripts with GO annotation and 20,071 transcripts mapped to 117 pathways belonging to all five categories of KEGG. We obtained adequate sequence depth of coverage and acceptable assembling results and expression level detection. Our *de novo* assembly of mRNA-seq will improve genome annotation of *G. arboreum* and the specificity of the transcript signal will allow us to distinguish individual members of gene families.

During water stresses, there is osmotic adjustment in cotton leaves and roots, and the growing root tips act as dehydration sensors in soil [26]. In addition, the Arabidopsis roots and leaves were reported to display very different changes in water stress-regulated genes; also, dynamic changes occurred during 3- and 27 h of stress, with ≤5% of the changes shared by all three stresses during 3 h of acute stress response; however, by 27 h, the shared responses were reduced to ≤0.5% [40]. Thus in the present study, we considered the importance of spatial resolution and conducted tissue-specific stress transcriptome analysis [26], [36], [38], [72]. We generated transcriptome mapping for three different tissues (roots, stems (with hypocotyls) and leaves) with 3 h of acute response to salt and osmotic treatments. We applied PCA to determine the dimensionality of the mRNA-seq data set and to identify meaningful underlying expression variables of the transcripts under salt and osmotic stress in different seedling tissues (Fig. 1E). The PCA revealed that gene expression differences among leaf, stem and root tissues were much greater than differences among the three treatment conditions (i.e. mock, PEG and salt).

In addition, the results showed that tissue identity mediated water stress. PCA showed the difference in the three treatments in root samples was much greater than those in leaf and stem tissues. Statistical analysis was used to identify the differentially expressed transcripts that responded to PEG and salt stresses. Among 123,579 transcripts, about 36,961 were identified as either up- or down-regulated under NaCl or PEG treatments in different tissues. Some transcripts were selected for validation by real-time RT-PCR, and most were matched between mRNA-seq and real-time RT-PCR analysis (Fig. 2), which suggested that our *de novo* assembly transcripts were reliable and repeatable. Furthermore, Venn diagram analysis showed highly overlapping responses to salt and osmotic stresses in individual cotton tissues (Fig. 3A). There was a very similar directional trend of gene differential expression between salt and osmotic stresses. Compared to those in leaf and root, there were much lower numbers of differentially expressed transcripts under water stress in stem tissue. In leaf, relatively larger numbers of transcripts were up-regulated under PEG treatment, while in root many more transcripts were down-regulated under NaCl treatment. Both PCA and Venn diagrams indicated the strong tissue selectivity of transcripts under salt and osmotic stress in cotton seedlings. We further conducted SOM and hierarchical cluster analysis for the 36,961 differential expressed transcripts. The cluster results showed a great deal of detailed tissue selectivity in each transcript’s response to water stress. We selected several groups of clustered transcripts with preferential expression in leaf, stem and root, respectively for GO enrichment analysis, which may elucidate the possible mechanism involved. There were common enriched GO terms for the differentially expressed genes, e.g. response to stimulus and oxidoreductase activity. However, compared to stem and root, the leaf-specific group of genes showed some unique significantly enriched GO terms, including those involved in photosynthesis, light reaction, carbon fixation, glucose metabolic process and tetrapyrrole binding. Unique GO terms were shown by stem-specific genes in relation to plant-type cell wall biogenesis, and by root-specific genes for monooxygenase activity. The GO enrichment analysis for the water stress regulated tissue-specific genes suggested that there were different adaptation mechanisms and sequential effects in the different tissues responding to water stress, adjusting and balancing the whole plant for tolerance during high salinity and drought stress conditions.

### Transcription Factors and Hormone Signal Transduction Pathways Involved in Water Stress

Transcription is a dynamic process and transcription factors are essential for regulation of gene expression. In the present study, we identified a total of 4,002 transcripts (Table 2) using global transcription factor classification for the differentially expressed transcripts. About 49% of members responded to salt or osmotic stresses in different cotton tissues, including some key regulatory gene families involved in abiotic and biotic stresses, e.g. AP2-EREBP, WRKY, bHLH, MYB, bZIP, NAC, and HSF. Some developmental-related transcription factor genes were also up- or down-regulated during water stress in cotton seedlings, e.g. growth-regulating factor (GRF), regulating cell expansion in leaf and cotyledon tissues. In addition, several transcription families related to hormone signal transduction pathways were also largely regulated by water stresses, e.g. ABI3VP1 (ABA signal pathway), Tify (mainly includes the orthologs of Arabidopsis JAZ, regulating the JA signal pathway), ARR-B (CK signal pathway), and EIL (ethylene signal pathway). We also found similar results in a previous study on the transcription response to salt stress in roots of *G. hirsutum* [51].

Plant hormones are essential for plants to adapt to water stress conditions [73]. The assembled cotton genes for those hormone signal pathways showed differential expression under osmotic and salt stresses in different cotton tissues (Fig. 4). As one of the main plant hormones, ABA plays major roles in seed and bud dormancy, as well as in responses to water stress. ABA mediates the water-stress signalling transduction pathway through core signalling components [74], [75], [76], [77], [78], [79], including the core group of ABA receptor PYR/PYL/RCAR family proteins, the type 2C protein phosphatase (PP2C), and members of SNF1-related protein kinase 2 (SnRK2). During osmotic stress, the PYR/PYL/RCAR ABA-receptor-PP2C complexes control the SnRK2-AREB/ABF pathways. In cotton under salt and osmotic stresses, some ABA receptor PYR/RCAR family genes showed significantly differential expression patterns (Fig. 4D). For example, the cotton orthologs of Arabidopsis PYL4/RCAR1 were down-regulated in all seedling tissues during 17% PEG (osmotic stress) and 150mM NaCl (salt stress) treatments, respectively, while the orthologs for Arabidopsis PYL9/RCAR1 (the paralog of PYL7/RCAR2) were up-regulated under osmotic stress in cotton leaf and stem and under salt stress in root and stem tissues. The orthologs for PYR1/RCAR12 and PYL2/RCAR14 were down-regulated under osmotic stress in cotton leaf and root, and were also down-regulated in root tissue during salt stress. The homologs of PYL8/RCAR3 were down-regulated in root tissue during salt stress. In addition, the homologs for atPYL1 and atPYL11 were down-regulated in stem tissue under salt and osmotic stresses, separately. It was reported that PYL8/RCAR3 was strongly down-regulated, but PYL7/RCAR2 was up-regulated by salt and osmotic stresses [80]. Some members of PP2Cs, such as ABI1, ABI2, HAB1 and AHG3, are involved in ABA signalling through direct interaction with PYR/PYL/RCAR ABA receptors [76], [79], [81]. PP2Cs play vital roles in negatively regulating ABA response. Interestingly, our assembled *PP2C* transcripts were up-regulated under osmotic and salt stresses in all seedling tissues, similarly to the downstream *ABF* genes. The assembled cotton *SnRK2* family genes showed tissue selectivity during salt stress, e.g. the majority were up-regulated under osmotic and salt stresses in leaf and stem, but were down-regulated under salt stress in root tissue. Our results indicated a conserved crosstalk between water stress and the ABA signal transduction pathway. The comparative analysis of expression profiles responding to water stresses in the seedling tissues showed the conservation between cotton and Arabidopsis in the ABA signal transduction pathway (Fig. S4A).

Auxin controls various developmental processes, such as apical dominance, root initiation and stem elongation, etc. Auxin is transported polarly in plant shoots and roots. Our mRNA-seq based transcriptome analysis showed that a large number of genes involved in the auxin signal pathway were differentially expressed in response to water stress in cotton seedlings (Fig. 4A). The majority of changed cotton *AUX1* and *ARF* genes were down-regulated under osmotic and salt stress conditions in root, stem and leaf tissues. Some down-stream genes such as GH3 and SAUR genes showed both up- or down- regulation during water stress. Plants can adjust their RSA and direction of root growth to deal with high soil salinity and the underlying mechanism may be related to ABA-dependent repression of lateral root formation and auxin distribution in the roots during osmotic and high salt stresses [82].

In addition, many cotton JA signal transduction genes, such as members of the JAR1, JAZ, MYC2 families of genes, were also differently expressed under salt stress and osmotic stresses (Fig. 4G). In particular, a large number of JAR1 and JAZ genes were up-regulated during salt stress in root tissue, consistent with our previous report concerning salt response in JA genes of roots of upland cotton using microarray analysis [51]. The expression of the Arabidopsis JA signaling repressor JAZ1/TIFY10A was reported to be stimulated by auxin [83]. Our identified salt- induced JAZ genes may play key roles in shaping plant roots and mediating the crosstalk between auxin and JA signaling during salt stress. Some cotton *JAR1* and *JAZ* genes were also up-regulated under both salt and osmotic stresses in leaf tissue; possibly related to involvement of endogenous ABA in JA-induced stomatal closure [84]. There is crosstalk between JA and ethylene signal transduction pathways. The modulation of ethylene responses may affect plant salt-stress responses in Arabidopsis [85]. Our mRNA-seq results showed that some key regulatory genes of the ethylene signal transduction pathway (Fig. 4E), e.g. *ETR*, *CTR*, *EBF* and *ERF1/2*, were differentially expressed during osmotic and salt stresses in cotton seedlings, with the majority up-regulated. Similar results were also found for upland cotton under salt stress [51]. This suggests that there is crosstalk between ethylene signaling and water stress in cotton.

Besides ABA, auxin, JA, and ethylene-mediated signaling pathways in the response to water stress in cotton seedling stage, other hormones (e.g. CK, SA, GA, and BR) also play direct or indirect roles during water stress. CK is an antagonist to ABA and water stress results in decreased levels of CK, which is a positive regulator of auxin biosynthesis. Both CK and auxin promote stomatal opening, while ABA, JA, and SA induce stomatal closure [86]. GA and BR have many similar properties, and regulate some common biological processes, e.g. short primary roots [73]. Our mRNA-seq results indicated multiple hormone crosstalks in response to osmotic and salt stresses in different tissues of cotton seedlings. We also suggest that there are hormone crosstalks between abiotic and biotic stresses.

In summary, through mRNA-seq analysis for nine cotton samples using NaCl and PEG treatments in three cotton tissues, we tried to provide an overview of transcriptome profiling of *G. arboreum*. The whole transcriptome shotgun sequencing in *G. arboreum* will allow us to gain a broad picture of the genomic response to water stress and some interesting clues for further research. In addition, our *de novo* assembled cotton transcriptome with three tissues and three treatments will be beneficial to cotton whole genome annotation and reconstruction.

## Materials and Methods

### Plant Material and Growth Conditions

Cotton (*G. arboreum* L. cv. Shixiya) seeds were immersed in water for 1 d at 30°C and then placed for germination on sterilized soil in plates maintained under the following conditions: 28/25°C, 12/12 h of light/darkness, and relative humidity of 80%. After 3–4 d, properly germinated seeds were transferred to black plastic tanks filled with nutrient solution [51] and allowed to grow until they had produced 6–7 leaves. Seedlings showing normal growth were randomly divided into three groups, one group placed into tanks filled with a 150 mM solution of NaCl in water; another group placed into tanks filled with a 17% solution of PEG 6000 in water; and the remaining seedlings transferred to tanks filled with plain water to serve as mock. After exposing the seedlings to different solutions for 3 h, leaf, stem (including hypocotyl), and root tissues were harvested at the same time.

### Isolation of RNA and Real-time PCR

All the cotton tissue samples were homogenized in liquid nitrogen before isolation of RNA. Total RNA was isolated using a modified CTAB method and purified using Qiagen RNeasy columns (Qiagen, Hilden, Germany).

Reverse transcription was performed using an M-MLV kit (Invitrogen). The samples, 10 µl each containing 2 µg of total RNA and 20 pmol of random hexamers (Invitrogen), were maintained at 70°C for 10 min to denature the RNA and then chilled on ice for 2 min. The reaction buffer and M-MLV enzyme (20 µl of the mixture contained 500 µM dNTPs, 50 mM Tris-HCl (pH 8.3), 75 mM KCl, 3 mM MgCl2, 5 mM dithiothreitol, 200 units of M-MLV, and 20 pmol random hexamers) was added to the chilled samples and the samples maintained at 37°C for 1 h. The cDNA samples were diluted to 8 ng/µl for RT-PCR analysis.

For real-time RT-PCR, assays were performed in triplicate on 1 µl of each cDNA dilution using the SYBR Green Master Mix (PN 4309155, Applied Biosystems) with an ABI 7500 sequence detection system as prescribed in the manufacturer’s protocol (Applied Biosystems). The gene-specific primers were designed using PRIMER3 (http://frodo.wi.mit.edu/primer3/input.htm). The amplification of 18S rRNA was used as an internal control to normalize all data (forward primer, 5′-CGGCTACCACATCCAAGGAA-3′; reverse primer, 5′- TGTCACTACCTCCCCGTGTCA-3′). The gene-specific primers are listed in Table S1. The relative quantification method (ΔΔCT) was used for quantitative evaluation of the variation between replicates.

### mRNA-seq Experiment and Transcriptome *de novo* Assembly

From each cotton tissue sample, 10 µg total RNA was collected for isolate poly(A) mRNA using beads with Oligo(dT). Then the mRNA was interrupted into short fragments by fragmentation buffer. The suitable fragments were selected for the PCR amplification as templates to prepare Illumina RNA-Seq library. Each library had an insert size around 200 bp and was sequenced using Illumina HiSeq™ 2000. The read lengths were 75 bp for root, and 90 bp for leaf and stem samples.

Sequencing-received raw image data was transformed by base calling into sequence data and stored in fastq format. After filtering low quality and dirty raw reads, transcriptome *de novo* assembly was carried out with a short reads assembling program (SOAPdenovo; [66]). The first step was to combine reads with certain length of overlap to form longer fragments, which are called contigs. Next, SOAPdenovo connectted the contigs using N to represent unknown sequences between each two contigs based on the paired-end reads, and then scaffolds were made. Paired-end reads were used again for gap filling of scaffolds to get sequences with least Ns and that could not be extended on either end. Such sequences are defined as unigenes. Finally, unigenes from each sample’s assembly were used for further processes of sequence splicing and redundancy removing with sequence clustering software to acquire non-redundant unigenes (here called ‘transcripts’) that were as long as possible.

To assign the possible annotation of the transcripts, blastx alignment (e-value <0.00001) between transcripts and protein databases (e.g. nr, Swiss-Prot, KEGG and COG) was performed, and the best aligning results used to decide sequence direction of transcripts. ESTScan [67] was introduced to predict the coding regions and determine the sequence direction of transcripts without annotation. The Blast2GO program [69] was used to get GO annotation of transcripts. We also collected all cotton ESTs from NCBI and DFCI Cotton Gene Index (http://compbio.dfci.harvard.edu/tgi/plant.html) for transcripts sequence analysis.

To identify possible transcription factors in the transcripts, we used the sequence information in the PlnTFDB (http://plntfdb.bio.uni-potsdam.de/v3.0/) and PlantTFDB (http://planttfdb.cbi.pku.edu.cn) by BLAST (basic local alignment and search tool), as well as the annotation from Swiss-Prot and nr which the transcripts hit.

### Identify Differential Expression Transcripts and Functional Analysis

The expression of transcripts was calculated by RPKM method [70]; the formula is shown below:
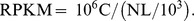
where C is the number of reads that uniquely aligned to the transcript, N is the total number of reads that uniquely aligned to all transcripts in the specific sample, and L is number of bases of the transcript. The *P*-value corresponds to differential transcript expression in two samples was determined from Audic’s algorithm [71], and FDR method was applied to determine the threshold of *P*-values in multiple tests. We use “FDR ≤0.001 and the absolute value of log_2_Ratio ≥1” as the threshold to judge the significance of gene expression difference.

GO enrichment analysis was performed for functional categorization of differentially expressed transcripts using agriGO software [87] and the *P*-values corrected by applying the FDR correction to control falsely rejected hypotheses during GO analysis.

MapMan (http://gabi.rzpd.de/projects/MapMan) was used for key regulation group analysis. The pathway analysis for plant hormone signalling were conducted using KEGG (www.genome.jp/kegg/) and the corresponding MapMan pathways were created through the mapping files for BLAST hits of transcripts to Arabidopsis TAIR9 version (www.arabidopsis.org).

## Supporting Information

Figure S1 **Workflow for sample preparation and experiment pipeline of** ***de novo*** **mRNA-seq transcriptome.** A total of nine samples were collected for mRNA-seq: leaf, stem (including hypocotyls), and root samples of cotton seedling under mock, 17% PEG, and 150 mM NaCl conditions.
(JPG)

Figure S2 **Pair-wise scatter plots for the raw reads of transcripts across all samples.**
(JPG)

Figure S3 **SOM (self-organized mapping) cluster of the transcripts response to PEG or NaCl treatments in different cotton tissue samples.** An overview of 10×10 SOM cluster for 36,961 transcripts’ response to PEG or NaCl treatments in leaf, stem, or root sample of cotton seedlings. For each cluster, the red line represents the expression pattern of the centroid, the order from left to right is: leaf-mock, leaf-PEG, leaf-NaCl, stem-mock, stem-PEG, stem-NaCl, root-mock, root-PEG, and root-NaCl. The cluster id and the number of transcripts in the cluster are also listed in the figures.
(JPG)

Figure S4 **Comparative analysis of cotton and Arabidopsis transcripts related to selected plant hormone signal transduction pathways and their response to water stresses across different tissue samples.** The colored bars represent the percentage of the transcripts in each bin whether up-regulated (red) or down-regulated (blue) under water stresses in different tissues. The charts above the pathway represent the cotton tissues and the charts below the pathway represent the Arabidopsis tissues. **A**: represents the differential expression transcripts related to abscisic acid (ABA) signalling transduction pathway. **B**: represents the differential expression transcripts related to jasmonate (JA) signalling transduction pathway. **C**: represents the differential expression transcripts related to auxin (IAA) signalling transduction pathway. **D**: represents the differential expression transcripts related to ethylene signalling transduction pathway.
(JPG)

Table S1 **Primer list of transcripts for real-time RT-PCR.**
(PDF)

Table S2 **In cotton tissues, 36,961 transcripts responded to PEG and NaCl treatments.** Including the transcript length, raw RPKM data, log_2_ ratio, FDR-value, change call, and additional annotation of each transcript
(XLS)
