# Supplementary figures and images for: mRNA-seq Analysis of the Gossypium arboreum transcriptome Reveals Tissue Selective Signaling in Response to Water Stress during Seedling Stage
Source: PLoS One. 2013 Jan 28;8(1):e54762. doi: 10.1371/journal.pone.0054762 (PMC3557298; doi:10.1371/journal.pone.0054762)

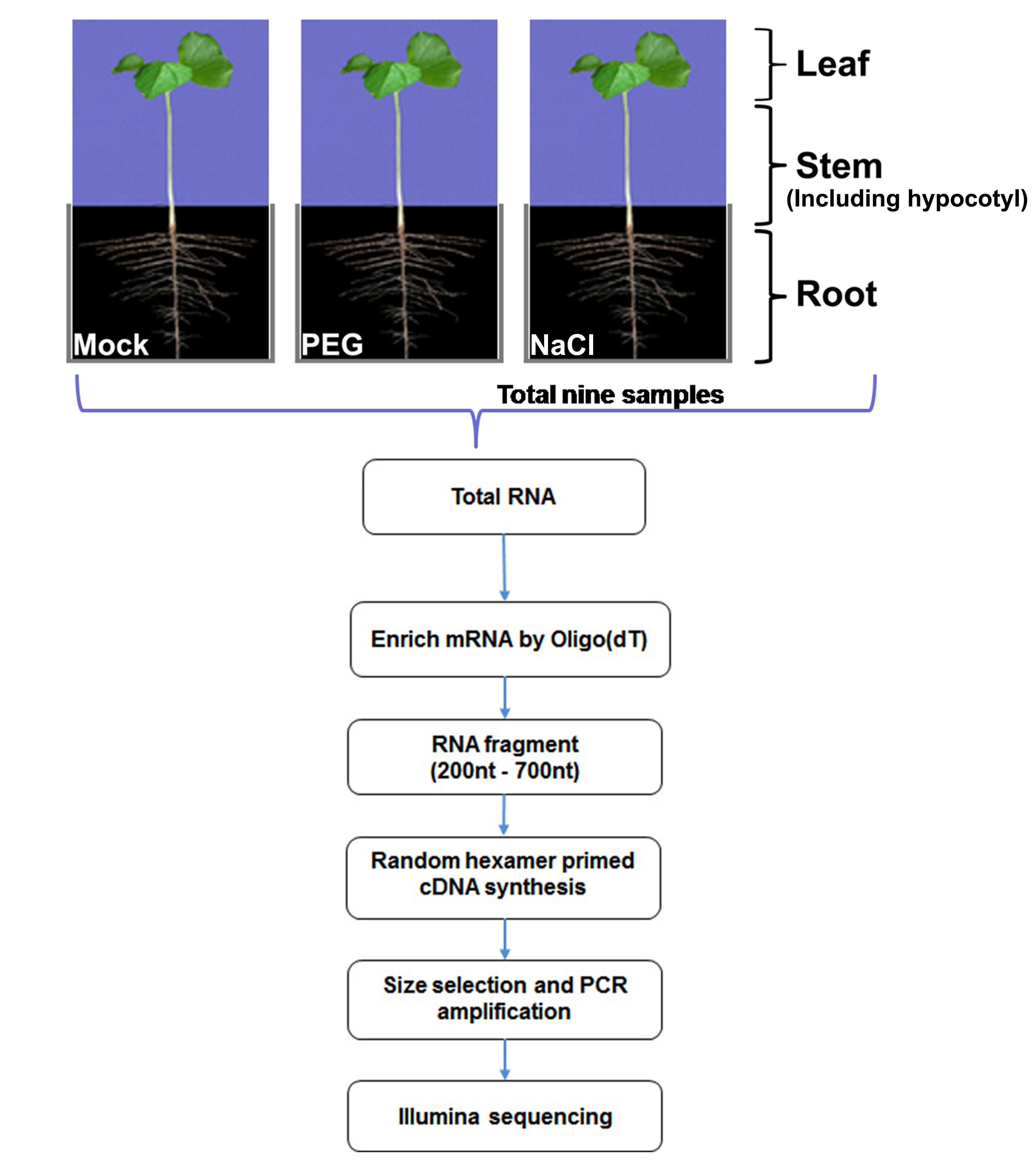

Supplement: Figure S1 — Workflow for sample preparation and experiment pipeline of de novo mRNA-seq transcriptome. A total of nine samples were collected for mRNA-seq: leaf, stem (including hypocotyls), and root samples of cotton seedling under mock, 17% PEG, and 150 mM NaCl conditions. (JPG) [file pone.0054762.s001.jpg]

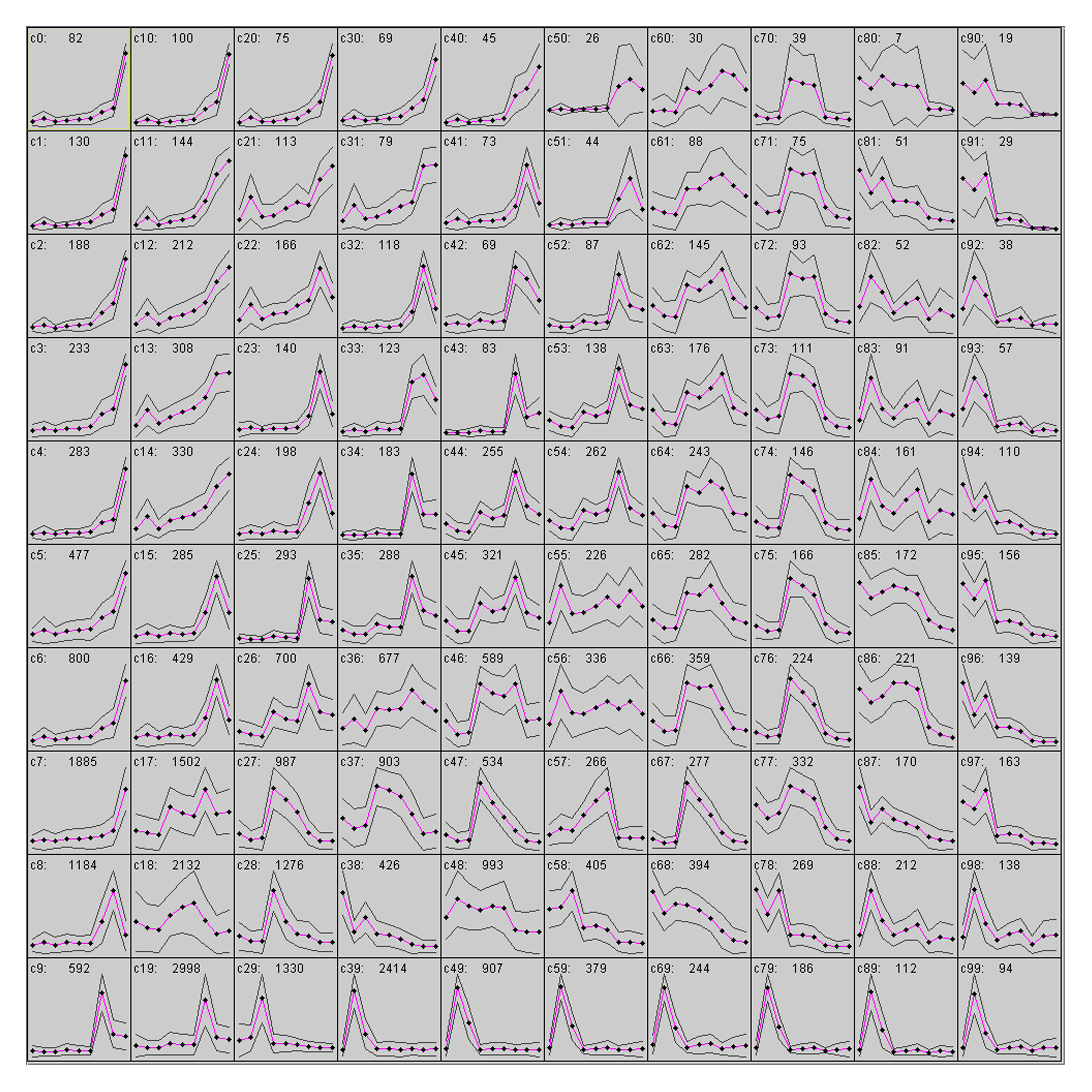

Supplement: Figure S3 — SOM (self-organized mapping) cluster of the transcripts response to PEG or NaCl treatments in different cotton tissue samples. An overview of 10×10 SOM cluster for 36,961 transcripts’ response to PEG or NaCl treatments in leaf, stem, or root sample of cotton seedlings. For each cluster, the red line represents the expression pattern of the centroid, the order from left to right is: leaf-mock, leaf-PEG, leaf-NaCl, stem-mock, stem-PEG, stem-NaCl, root-mock, root-PEG, and root-NaCl. The cluster id and the number of transcripts in the cluster are also listed in the figures. (JPG) [file pone.0054762.s003.jpg]
