# Supplementary material for: mRNA-seq Analysis of the Gossypium arboreum transcriptome Reveals Tissue Selective Signaling in Response to Water Stress during Seedling Stage
Source: PLoS One. 2013 Jan 28;8(1):e54762. doi: 10.1371/journal.pone.0054762 (PMC3557298; doi:10.1371/journal.pone.0054762)
Supplement: Table S1 — Primer list of transcripts for real-time RT-PCR. (PDF) [file pone.0054762.s005.pdf]

		RPKM									Leaf PEG vs Leaf Mock			Leaf NaCl vs Leaf Mock			Stem PEG vs Stem Mock			Stem NaCl vs Stem Mock			Root PEG vs Root Mock			Root NaCl vs Root Mock		
Unigene ID	Unigene Lengt	Leaf Mock	Leaf PEG	Leaf NaCl	Stem Mock	Stem PEG	Stem NaCl	Root Mock	Root PEG	Root NaCl	log2 ratio	FDR	Change call	log2 ratio	FDR	Change call	log2 ratio	FDR	Change call	log2 ratio	FDR	Change call	log2 ratio	FDR	Change call	log2 ratio	FDR	Change call
Unigene10_All	4154	1.32	3.51	2.09	2.91	3.12	2.66	5.23	6.19	5.83	1.41E+00	1.28E-18	Up	6.60E-01	1.16E-03		9.71E-02	5.98E-01		-1.30E-01	5.02E-01		2.42E-01	1.50E-02		1.57E-01	1.36E-01	
Unigene1000_All	2008	87.26	13.58	26.60	63.88	44.18	35.38	8.19	1.00	2.84	-2.68E+00	0.00E+00	Down	-1.71E+00	1.75E-280	Down	-5.32E-01	7.13E-38		-8.53E-01	9.16E-77		-3.04E+00	4.49E-67	Down	-1.53E+00	1.01E-29	Down
Unigene100003_All	343	0.00	0.59	0.00	1.60	1.61	0.53	1.85	0.00	0.00	9.21E+00	1.38E-01		null	null		7.90E-03	9.83E-01		-1.60E+00	1.32E-01		-1.09E+01	1.24E-04	Down	-1.09E+01	7.13E-05	Down
Unigene100006_All	344	0.00	1.77	0.93	0.00	9.01	0.00	0.00	0.00	0.00	1.08E+01	1.17E-03		9.86E+00	4.54E-02		1.31E+01	1.41E-19	Up	null	null		null	null		null	null	
Unigene100007_All	345	0.00	0.59	0.15	0.00	1.80	0.00	0.00	0.12	0.23	9.20E+00	1.38E-01		7.27E+00	6.43E-01		1.08E+01	7.90E-04	Up	null	null		6.92E+00	6.41E-01		7.85E+00	4.07E-01	
Unigene10002_All	315	0.00	0.00	2.54	0.47	0.44	2.15	0.27	0.27	0.25	null	null		1.13E+01	1.72E-04	Up	-1.18E-01	9.12E-01		2.18E+00	3.15E-02		-1.62E-02	1.02E+00		-8.58E-02	9.75E-01	
Unigene100031_All	352	0.15	0.14	0.61	3.11	0.98	0.26	1.92	0.48	0.68	-4.64E-02	9.88E-01		2.03E+00	3.62E-01		-1.67E+00	9.38E-03		-3.60E+00	5.03E-05	Down	-2.02E+00	1.98E-02		-1.50E+00	5.44E-02	
Unigene100049_All	362	0.58	1.68	0.88	1.65	5.23	6.99	0.00	0.35	0.33	1.54E+00	1.22E-01		6.10E-01	6.25E-01		1.66E+00	6.50E-04	Up	2.08E+00	2.87E-06	Up	8.44E+00	2.41E-01		8.37E+00	2.44E-01	
Unigene10005_All	326	0.00	4.82	3.43	0.00	0.00	0.00	0.00	0.00	0.00	1.22E+01	5.71E-09	Up	1.17E+01	3.44E-06	Up	null	null		null	null		null	null		null	null	
Unigene100054_All	366	0.00	0.00	0.00	0.00	19.57	0.00	0.00	0.00	0.00	null	null		null	null		1.43E+01	4.05E-46	Up	null	null		null	null		null	null	
Unigene100057_All	368	0.14	0.14	0.58	0.68	1.87	0.61	1.95	0.11	0.22	-4.64E-02	9.88E-01		2.03E+00	3.62E-01		1.47E+00	1.16E-01		-1.41E-01	9.29E-01		-4.10E+00	2.92E-04	Down	-3.17E+00	8.10E-04	Down
Unigene100060_All	369	1.84	0.00	1.15	0.27	2.05	0.61	0.46	0.45	0.54	-1.08E+01	4.10E-04	Down	-6.75E-01	4.41E-01		2.93E+00	4.37E-03		1.18E+00	5.50E-01		-1.65E-02	1.02E+00		2.36E-01	8.84E-01	
Unigene100062_All	370	0.28	0.27	0.00	0.27	2.98	0.98	0.00	0.00	0.00	-4.61E-02	9.91E-01		-8.14E+00	4.00E-01		3.47E+00	6.96E-05	Up	1.86E+00	2.20E-01		null	null		null	null	
Unigene100069_All	375	0.84	0.00	0.00	0.13	1.19	2.29	0.68	0.33	0.00	-9.71E+00	3.67E-02		-9.71E+00	4.94E-02		3.17E+00	3.95E-02		4.11E+00	3.41E-04	Up	-1.02E+00	4.96E-01		-9.40E+00	3.06E-02	
Unigene100076_All	381	0.27	2.66	1.12	0.26	0.90	0.71	0.78	0.22	0.00	3.28E+00	3.79E-04	Up	2.03E+00	1.40E-01		1.79E+00	2.58E-01		1.44E+00	4.10E-01		-1.82E+00	2.11E-01		-9.60E+00	1.59E-02	
Unigene100077_All	381	3.02	1.60	0.00	1.05	1.54	1.19	0.00	0.00	0.00	-9.21E-01	1.43E-01		-1.16E+01	2.60E-06	Down	5.55E-01	6.00E-01		1.81E-01	8.84E-01		null	null		null	null	
Unigene100078_All	381	0.00	0.13	0.42	0.52	3.16	1.19	0.00	0.11	0.63	7.06E+00	6.24E-01		8.71E+00	2.40E-01		2.60E+00	3.41E-04	Up	1.18E+00	3.38E-01		6.78E+00	6.41E-01		9.29E+00	4.41E-02	
Unigene100081_All	383	1.23	1.06	0.56	0.13	1.98	1.06	0.00	0.00	0.00	-2.16E-01	8.17E-01		-1.14E+00	3.32E-01		3.93E+00	9.79E-04	Up	3.03E+00	6.04E-02		null	null		null	null	
Unigene100083_All	384	0.00	0.26	0.00	0.00	2.24	0.00	0.00	0.00	0.00	8.04E+00	3.92E-01		null	null		1.11E+01	2.76E-05	Up	null	null		null	null		null	null	
Unigene100086_All	385	1.90	0.00	2.49	2.20	3.49	1.29	0.77	0.43	0.83	-1.09E+01	2.11E-04	Down	3.88E-01	6.08E-01		6.65E-01	2.81E-01		-7.69E-01	3.34E-01		-8.24E-01	5.41E-01		1.07E-01	9.46E-01	
Unigene100089_All	385	0.00	0.53	0.00	0.00	13.24	0.00	0.00	0.00	0.00	9.04E+00	1.38E-01		null	null		1.37E+01	1.36E-32	Up	null	null		null	null		null	null	
Unigene10009_All	669	0.00	1.51	0.24	0.07	0.62	0.20	0.76	0.94	2.14	1.06E+01	7.26E-06	Up	7.90E+00	2.40E-01		3.05E+00	5.68E-02		1.44E+00	6.41E-01		3.05E-01	7.04E-01		1.50E+00	2.97E-03	
Unigene100108_All	395	0.40	0.51	0.40	0.13	2.18	0.00	0.11	0.42	0.00	3.69E-01	8.17E-01		2.52E-02	1.00E+00		4.11E+00	2.61E-04	Up	-6.98E+00	6.47E-01		1.98E+00	3.80E-01		-6.74E+00	6.21E-01	
Unigene10011_All	387	0.00	0.00	0.14	0.00	0.18	0.23	2.29	1.51	0.10	null	null		7.10E+00	6.43E-01		7.48E+00	6.18E-01		7.87E+00	4.84E-01		-6.02E-01	3.85E-01		-4.48E+00	1.24E-05	Down
Unigene100114_All	399	0.52	0.13	1.07	0.12	1.90	0.23	0.32	0.31	0.20	-2.05E+00	3.34E-01		1.03E+00	3.97E-01		3.93E+00	9.79E-04	Up	8.59E-01	7.98E-01		-1.64E-02	1.02E+00		-6.71E-01	7.21E-01	
Unigene100117_All	401	0.00	0.63	0.00	0.00	2.15	0.68	0.00	0.31	0.10	9.30E+00	7.89E-02		null	null		1.11E+01	2.76E-05	Up	9.40E+00	7.07E-02		8.29E+00	2.41E-01		6.63E+00	6.51E-01	
Unigene100122_All	406	0.00	1.00	0.92	0.00	17.13	0.00	0.00	0.00	0.00	9.96E+00	1.36E-02		9.84E+00	2.51E-02		1.41E+01	9.15E-45	Up	null	null		null	null		null	null	
Unigene100125_All	408	0.00	0.50	0.00	2.20	3.04	0.00	0.41	0.00	0.10	8.96E+00	1.38E-01		null	null		4.67E-01	4.92E-01		-1.11E+01	1.21E-05	Down	-8.70E+00	1.35E-01		-2.09E+00	3.14E-01	
Unigene100132_All	420	1.00	0.60	0.76	0.47	3.85	0.11	0.30	0.20	0.00	-7.24E-01	5.26E-01		-3.90E-01	7.18E-01		3.02E+00	2.63E-06	Up	-2.14E+00	3.48E-01		-6.02E-01	7.68E-01		-8.24E+00	2.01E-01	
Unigene10014_All	680	0.15	0.00	0.16	0.15	0.41	0.00	3.05	0.68	1.46	-7.27E+00	3.78E-01		2.51E-02	1.00E+00		1.47E+00	4.20E-01		-7.19E+00	4.06E-01		-2.17E+00	1.96E-06	Down	-1.06E+00	6.48E-03	
Unigene100141_All	438	1.19	0.23	0.24	0.68	2.20	2.89	0.00	1.34	0.18	-2.37E+00	4.76E-02		-2.30E+00	6.77E-02		1.69E+00	2.52E-02		2.08E+00	1.96E-03		1.04E+01	2.82E-04	Up	7.51E+00	4.07E-01	
Unigene100147_All	444	0.47	0.11	0.00	1.91	1.24	0.10	0.57	1.22	0.27	-2.05E+00	3.34E-01		-8.88E+00	1.47E-01		-6.20E-01	4.13E-01		-4.23E+00	2.18E-04	Down	1.10E+00	2.36E-01		-1.09E+00	4.29E-01	
Unigene100149_All	446	0.00	0.00	0.00	0.00	3.01	0.51	0.00	0.00	0.00	null	null		null	null		1.16E+01	2.67E-08	Up	8.98E+00	1.19E-01		null	null		null	null	
Unigene10015_All	336	0.00	7.84	2.69	0.00	0.00	0.00	0.00	0.00	0.00	1.29E+01	5.45E-15	Up	1.14E+01	4.72E-05	Up	null	null		null	null		null	null		null	null	
Unigene100150_All	446	0.00	0.00	0.00	0.00	3.78	0.91	0.00	0.00	0.00	null	null		null	null		1.19E+01	1.76E-10	Up	9.83E+00	1.37E-02		null	null		null	null	
Unigene10016_All	468	0.00	14.83	3.53	0.00	0.15	0.00	0.00	0.00	0.00	1.39E+01	1.15E-39	Up	1.18E+01	4.31E-09	Up	7.20E+00	6.18E-01		null	null		null	null		null	null	
Unigene100162_All	481	0.00	0.00	0.00	0.21	1.86	0.75	0.00	0.00	0.00	null	null		null	null		3.17E+00	8.78E-04	Up	1.86E+00	2.20E-01		null	null		null	null	
Unigene100163_All	483	0.11	0.00	0.33	1.24	1.07	0.00	0.88	0.00	0.00	-6.76E+00	6.24E-01		1.61E+00	5.12E-01		-2.11E-01	8.23E-01		-1.03E+01	7.71E-04	Down	-9.77E+00	3.26E-03		-9.77E+00	2.15E-03	
Unigene100172_All	544	1.15	0.75	0.59	2.56	3.86	1.49	1.63	0.77	0.22	-6.31E-01	4.75E-01		-9.75E-01	3.16E-01		5.91E-01	2.02E-01		-7.79E-01	1.82E-01		-1.09E+00	1.07E-01		-2.89E+00	3.05E-04	Down

<table>
<tr><th>Unigene ID</th><th>Annotation</th><th>TIAR9 hit</th><th>Cotton EST hit</th><th>Transcriprion factor fa</th><th>KO pathway</th><th>GO term</th><th>Transcript direction</th></tr>
<tr><td>Unigene10_All</td><td>Unigene10_All//gi|124359633|gb|ABD32307.2|//0.00E+00//Nuclear factor related to kappa-B-binding protein, related [Medicago truncatula]</td><td>Unigene10_All//0.00E+00//AT3G45830| unknown protein</td><td>Unigene10_All//0.00E+00//ES821330</td><td></td><td></td><td></td><td>nr -</td></tr>
<tr><td>Unigene1000_All</td><td>Unigene1000_All//gi|20196907|gb|AAB67625.2|//1.00E-139//expressed protein [Arabidopsis thaliana]</td><td>Unigene1000_All//3.00E-141//AT1G29050| unknown protein</td><td>Unigene1000_All//0.00E+00//TC170634</td><td></td><td></td><td></td><td>nr -</td></tr>
<tr><td>Unigene100003_All</td><td></td><td></td><td>Unigene100003_All//7.00E-15//CO112306</td><td></td><td></td><td></td><td></td></tr>
<tr><td>Unigene100006_All</td><td></td><td></td><td></td><td></td><td></td><td></td><td></td></tr>
<tr><td>Unigene100007_All</td><td></td><td></td><td></td><td></td><td></td><td></td><td></td></tr>
<tr><td>Unigene10002_All</td><td>Unigene10002_All//gi|297817888|ref|XP_002876827.1|//3.00E-12//pentatricopeptide repeat-containing protein [Arabidopsis lyrata subsp. lyrata] >gi|297322665|gb|EFH53086.1| pentatricopeptide repeat-containing protein [Arabidopsis lyrata subsp. lyrata]</td><td>Unigene10002_All//2.00E-12//AT2G02750| pentatricopeptide (PPR) repeat-containing protein</td><td></td><td></td><td></td><td></td><td>nr +</td></tr>
<tr><td>Unigene100031_All</td><td>Unigene100031_All//gi|297598339|ref|NP_001045426.2|//6.00E-11//Os01g0953100 [Oryza sativa Japonica Group] >gi|255674080|dbj|BAF07340.2| Os01g0953100 [Oryza sativa Japonica Group]</td><td>Unigene100031_All//4.00E-13//AT5G45480| unknown protein</td><td></td><td></td><td></td><td></td><td>nr -</td></tr>
<tr><td>Unigene100049_All</td><td></td><td></td><td>Unigene100049_All//2.00E-117//EX165874</td><td></td><td></td><td></td><td>ESTscan +</td></tr>
<tr><td>Unigene10005_All</td><td>Unigene10005_All//gi|226462414|gb|EEH59706.1|//5.00E-07//predicted protein [Micromonas pusilla CCMP1545]</td><td></td><td></td><td></td><td></td><td></td><td>nr -</td></tr>
<tr><td>Unigene100054_All</td><td></td><td></td><td>Unigene100054_All//1.00E-176//TC177439</td><td></td><td></td><td></td><td></td></tr>
<tr><td>Unigene100057_All</td><td></td><td></td><td>Unigene100057_All//5.00E-13//TC176573</td><td></td><td></td><td></td><td>ESTscan +</td></tr>
<tr><td>Unigene100060_All</td><td>Unigene100060_All//gi|8778340|gb|AAF79348.1|AC007887_7//2.00E-11//F1504.13 [Arabidopsis thaliana]</td><td></td><td></td><td></td><td></td><td></td><td>nr +</td></tr>
<tr><td>Unigene100062_All</td><td>Unigene100062_All//gi|57282623|emb|CAE54309.1|//3.00E-66//peroxidase [Gossypium hirsutum]</td><td>Unigene100062_All//9.00E-37//AT4G25980| cationic peroxidase, putative</td><td>Unigene100062_All//0.00E+00//gi|21093393|gb|BQ405706.1|BQ405706</td><td></td><td>ko00940|Phenylpropanoid biosynthesis//ko00360|Phenylalanine metabolism</td><td></td><td>nr +</td></tr>
<tr><td>Unigene100069_All</td><td></td><td></td><td></td><td></td><td></td><td></td><td>ESTscan -</td></tr>
<tr><td>Unigene100076_All</td><td>Unigene100076_All//gi|75213194|sp|Q9SX85.1|SEC3A_ARATH//3.00E-32//RecName: Full=Exocyst complex component SEC3A >gi|5668806|gb|AAD46032.1|AC007519_17 Strong similarity to F16N3.17 from Arabidopsis thalian BAC gb|AC007519 [Arabidopsis thaliana]</td><td>Unigene100076_All//1.00E-33//AT1G47550| unknown protein</td><td>Unigene100076_All//0.00E+00//ES798551</td><td></td><td></td><td></td><td>nr +</td></tr>
<tr><td>Unigene100077_All</td><td>Unigene100077_All//gi|3513745|gb|AAC33961.1|//2.00E-08//contains similarity to reverse trancriptase (Pfam: rvt.hmm, score: 42.57) [Arabidopsis thaliana]</td><td>Unigene100077_All//1.00E-07//AT4G29090| reverse transcriptase, putative / RNA-dependent DNA polymerase, putative</td><td>Unigene100077_All//1.00E-25//gi|164240898|gb|ES823879.1|ES823879</td><td></td><td></td><td></td><td>nr +</td></tr>
<tr><td>Unigene100078_All</td><td>Unigene100078_All//gi|297826769|ref|XP_002881267.1|//1.00E-40//TMS membrane family protein [Arabidopsis lyrata subsp. lyrata] >gi|297327106|gb|EFH57526.1| TMS membrane family protein [Arabidopsis lyrata subsp. lyrata]</td><td>Unigene100078_All//2.00E-41//AT2G33205| FUNCTIONS IN: molecular_function unknown; INVOLVED IN: biological_process unknown; LOCATED IN: membrane; CONTAINS InterPro DOMAIN/s: TMS membrane protein/tumour differentially expressed protein (InterPro:IPR005016); BEST Arabidopsis thaliana protein match is: MEE55 (maternal effect embryo arrest 55) (TAIR:AT4G13345.1); Has 602 Blast hits to 593 proteins in 136 species: Archae - 0; Bacteria - 0; Metazoa - 380; Fungi - 90; Plants - 84; Viruses - 0; Other Eukaryotes - 48 (source: NCBI BLink).</td><td>Unigene100078_All//1.00E-72//TC144118</td><td></td><td></td><td></td><td>nr +</td></tr>
<tr><td>Unigene100081_All</td><td>Unigene100081_All//sp|P16679|PHNL_ECOLI//6.00E-14//Phosphonates transport ATP-binding protein phnL OS=Escherichia coli (strain K12) GN=phnL PE=1 SV=1</td><td>Unigene100081_All//5.00E-05//AT5G61730| ATATH11; ATPase, coupled to transmembrane movement of substances / transporter</td><td></td><td></td><td></td><td></td><td>swissprot -</td></tr>
<tr><td>Unigene100083_All</td><td>Unigene100083_All//gi|115438943|ref|NP_001043751.1|//2.00E-24//Os01g0655400 [Oryza sativa Japonica Group] >gi|55773937|dbj|BAD72325.1| eukaryotic translation initiation factor 3 subunit 10-like [Oryza sativa Japonica Group] >gi|113533282|dbj|BAF05665.1| Os01g0655400 [Oryza sativa Japonica Group]</td><td></td><td></td><td></td><td>ko03040|Spliceosome</td><td></td><td>nr -</td></tr>
<tr><td>Unigene100086_All</td><td>Unigene100086_All//gi|30684737|ref|NP_196912.2|//1.00E-32//pentatricopeptide (PPR) repeat-containing protein [Arabidopsis thaliana] >gi|223635762|sp|Q9FMU2.2|PP380_ARATH RecName: Full=Pentatricopeptide repeat-containing protein At5g14080</td><td>Unigene100086_All//6.00E-27//AT5G14080| pentatricopeptide (PPR) repeat-containing protein</td><td></td><td></td><td></td><td></td><td>nr -</td></tr>
<tr><td>Unigene100089_All</td><td>Unigene100089_All//gi|116309014|emb|CAH66131.1|//4.00E-11//OSIGBa0135L04.5 [Oryza sativa (indica cultivar-group)]</td><td>Unigene100089_All//2.00E-06//AT3G09510| nucleic acid binding</td><td>Unigene100089_All//2.00E-163//TC177439</td><td></td><td></td><td></td><td>nr +</td></tr>
<tr><td>Unigene10009_All</td><td></td><td></td><td>Unigene10009_All//8.00E-07//GE653373</td><td></td><td></td><td></td><td></td></tr>
<tr><td>Unigene100108_All</td><td></td><td></td><td></td><td></td><td></td><td></td><td></td></tr>
<tr><td>Unigene10011_All</td><td>Unigene10011_All//gi|255590973|ref|XP_002535407.1|//1.00E-11//conserved hypothetical protein [Ricinus communis] >gi|223523200|gb|EEF26976.1| conserved hypothetical protein [Ricinus communis]</td><td>Unigene10011_All//2.00E-06//AT5G53840| F-box family protein (FBL13)</td><td></td><td></td><td></td><td></td><td>nr +</td></tr>
<tr><td>Unigene100114_All</td><td></td><td></td><td>Unigene100114_All//5.00E-07//ES811668</td><td></td><td></td><td></td><td></td></tr>
<tr><td>Unigene100117_All</td><td></td><td></td><td></td><td></td><td></td><td></td><td></td></tr>
<tr><td>Unigene100122_All</td><td></td><td></td><td></td><td></td><td></td><td></td><td></td></tr>
<tr><td>Unigene100125_All</td><td>Unigene100125_All//gi|195621986|gb|ACG32823.1|//6.00E-24//BRASSINOSTEROID INSENSITIVE 1-associated receptor kinase 1 precursor [Zea mays]</td><td>Unigene100125_All//3.00E-19//AT3G26700| protein kinase family protein</td><td>Unigene100125_All//0.00E+00//CO071070</td><td></td><td>ko04626|Plant-pathogen interaction//ko00270|Cysteine and methionine metabolism</td><td></td><td>nr -</td></tr>
<tr><td>Unigene100132_All</td><td>Unigene100132_All//gi|295815790|gb|ADG36380.1|//4.00E-22//promoter-binding protein SPL9 [Vitis vinifera]</td><td></td><td>Unigene100132_All//1.00E-171//gi|78343657|gb|DT563931.1|DT563931</td><td></td><td></td><td></td><td>nr +</td></tr>
<tr><td>Unigene10014_All</td><td>Unigene10014_All//gi|9739011|gb|AAF97863.1|//5.00E-68//outward-rectifying potassium channel KCO1 [Eucalyptus camaldulensis]</td><td>Unigene10014_All//7.00E-66//AT5G55630| ATKCO1; calcium-activated potassium channel/ ion channel/ outward rectifier potassium channel</td><td>Unigene10014_All//1.00E-138//TC135368</td><td></td><td></td><td>GO:0005227//GO:0005249//GO:0006875//GO:0009845//GO:0010118//GO:0030001//GO:0031090//GO:0031224//GO:0046872//GO:0051259</td><td>nr -</td></tr>
<tr><td>Unigene100141_All</td><td>Unigene100141_All//gi|225458882|ref|XP_002283431.1|//3.00E-09//PREDICTED: hypothetical protein [Vitis vinifera] >gi|302142175|emb|CBI19378.3| unnamed protein product [Vitis vinifera]</td><td></td><td>Unigene100141_All//0.00E+00//DW512687</td><td></td><td></td><td></td><td>nr -</td></tr>
<tr><td>Unigene100147_All</td><td></td><td></td><td>Unigene100147_All//5.00E-07//DW519735</td><td></td><td></td><td></td><td></td></tr>
<tr><td>Unigene100149_All</td><td>Unigene100149_All//gi|4001781|gb|AAC94980.1|//5.00E-62//cytochrome C oxidase subunit I [Ectocarpus sp.]</td><td>Unigene100149_All//6.00E-46//ATMG01360| cytochrome c oxidase subunit 1</td><td>Unigene100149_All//2.00E-12//TC149581</td><td></td><td>ko00190|Oxidative phosphorylation</td><td></td><td>nr -</td></tr>
<tr><td>Unigene10015_All</td><td>Unigene10015_All//gi|300175133|emb|CBK20444.2|//3.00E-07//Pyruvate Dehydrogenase E1 (subunit ?) [Blastocystis hominis]</td><td>Unigene10015_All//5.00E-05//AT5G50850| MAB1 (MACCI-BOU); catalytic/ pyruvate dehydrogenase (acetyl-transferring)</td><td></td><td></td><td>ko00010|Glycolysis / Gluconeogenesis//ko00620|Pyruvate metabolism//ko00020|Citrate cycle (TCA cycle)//ko00650|Butanoate metabolism//ko00290|Valine, leucine and isoleucine biosynthesis</td><td></td><td>nr -</td></tr>
<tr><td>Unigene100150_All</td><td>Unigene100150_All//gi|260766526|gb|ACX50285.1|//1.00E-32//cytochrome oxidase subunit 1 [Guillardia theta]</td><td>Unigene100150_All//4.00E-29//ATMG01360| cytochrome c oxidase subunit 1</td><td></td><td></td><td>ko00190|Oxidative phosphorylation</td><td></td><td>nr -</td></tr>
<tr><td>Unigene10016_All</td><td></td><td></td><td></td><td></td><td></td><td></td><td>ESTscan -</td></tr>
<tr><td>Unigene100162_All</td><td></td><td></td><td></td><td></td><td></td><td></td><td></td></tr>
<tr><td>Unigene100163_All</td><td>Unigene100163_All//gi|54606737|dbj|BAD66760.1|//6.00E-06//orf120b [Beta vulgaris subsp. vulgaris] >gi|54606775|dbj|BAD66798.1| orf120b [Beta vulgaris subsp. vulgaris]</td><td></td><td>Unigene100163_All//8.00E-37//TC141826</td><td></td><td></td><td></td><td>nr +</td></tr>
<tr><td>Unigene100172_All</td><td>Unigene100172_All//gi|297799972|ref|XP_002867870.1|//3.00E-49//male sterility MS5 [Arabidopsis lyrata subsp. lyrata] >gi|297313706|gb|EFH44129.1| male sterility MS5 [Arabidopsis lyrata subsp. lyrata]</td><td>Unigene100172_All//6.00E-46//AT4G20900| MS5 (MALE-STERILE 5)</td><td></td><td></td><td></td><td></td><td>nr -</td></tr>
</table>

Unigene100173_All	559	0.00	0.73	0.00	0.00	3.08	0.73	0.00	0.00	0.00	9.50E+00	1.36E-02		null	null		1.16E+01	1.06E-10	Up	9.51E+00	1.37E-02		null	null		null	null		Unigene100173_All//gi|225703022|dbj|BAH30233.1|//5.00E-46//cytochrome c oxidase subunit II [Haptoglossa sp. SZ03]	Unigene100173_All//5.00E-23//ATMG00160| cytochrome c oxidase subunit 2			ko00190|Oxidative phosphorylation		nr +
Unigene100175_All	591	0.09	0.51	0.00	0.00	2.56	0.00	0.00	0.00	0.00	2.54E+00	1.51E-01		-6.47E+00	6.37E-01		1.13E+01	2.18E-09	Up	null	null		null	null		null	null		Unigene100175_All//gi|297841083|ref|XP_002888423.1|//8.00E-70//xanthine/uracil permease family protein [Arabidopsis lyrata subsp. lyrata] >gi|297334264|gb|EFH64682.1| xanthine/uracil permease family protein [Arabidopsis lyrata subsp. lyrata]	Unigene100175_All//5.00E-59//AT1G65550| xanthine/uracil permease family protein	Unigene100175_All//5.00E-11//TC129603				nr -
Unigene100176_All	602	1.30	0.00	0.18	5.05	2.00	0.23	0.70	0.07	0.13	-1.03E+01	1.09E-04	Down	-2.88E+00	6.35E-03		-1.33E+00	8.47E-05	Down	-4.49E+00	2.32E-15	Down	-3.34E+00	1.80E-02		-2.41E+00	3.94E-02		Unigene100176_All//gi|238909261|gb|ACR77749.1|//9.00E-77//12-oxophytodienoic acid 10,10-reductase [Astragalus sinicus]	Unigene100176_All//9.00E-75//AT1G76680| OPR1; 12-oxophytodienoate reductase	Unigene100176_All//5.00E-17//AI725636		ko00592|alpha-Linolenic acid metabolism		nr -
Unigene100180_All	665	0.00	0.00	0.00	0.00	2.74	0.54	0.00	0.00	0.00	null	null		null	null		1.14E+01	2.33E-11	Up	9.09E+00	2.38E-02		null	null		null	null		Unigene100180_All//gi|226347397|gb|ACO50109.1|//1.00E-76//elongation factor 1 alpha [Euglena longa]	Unigene100180_All//2.00E-62//AT5G60390| elongation factor 1-alpha / EF-1-alpha	Unigene100180_All//3.00E-12//TC143701				nr +
Unigene100181_All	784	0.00	0.00	0.00	0.00	2.99	0.69	0.00	0.00	0.00	null	null		null	null		1.15E+01	1.12E-14	Up	9.43E+00	2.46E-03		null	null		null	null		Unigene100181_All//gi|49147195|ref|YP_025788.1|//2.00E-23//ATP synthase F0 subunit 6 [Pseudendoclonium akinetum] >gi|33439210|gb|AAQ18747.1| ATP synthase F0 subunit 6 [Pseudendoclonium akinetum]	Unigene100181_All//2.00E-08//ATMG00410| ATPase subunit 6			ko00190|Oxidative phosphorylation		nr -
Unigene100182_All	447	0.12	0.11	0.00	0.22	0.15	0.30	1.32	0.09	0.00	-4.51E-02	9.88E-01		-6.87E+00	6.37E-01		-5.33E-01	8.20E-01		4.43E-01	8.63E-01		-3.82E+00	1.79E-03		-1.04E+01	1.42E-04	Down			Unigene100182_All//8.00E-114//ES830419				
Unigene100186_All	292	4.30	3.82	10.76	7.68	4.84	8.35	3.33	1.57	2.32	-1.72E-01	7.55E-01		1.32E+00	4.53E-04	Up	-6.67E-01	1.08E-01		1.22E-01	7.81E-01		-1.08E+00	8.88E-02		-5.22E-01	3.89E-01		Unigene100186_All//gi|213990447|gb|ACJ60637.1|//2.00E-11//glutaredoxin S12 [Populus tremula x Populus tremuloides]	Unigene100186_All//6.00E-11//AT4G28730| glutaredoxin family protein	Unigene100186_All//2.00E-119//BG445794				nr +
Unigene100191_All	491	0.43	0.00	0.33	0.41	0.49	0.37	1.12	0.00	0.41	-8.74E+00	1.23E-01		-3.89E-01	8.08E-01		2.75E-01	8.96E-01		-1.42E-01	9.36E-01		-1.01E+01	4.62E-04	Down	-1.46E+00	9.77E-02		Unigene100191_All//gi|75146761|sp|Q84K11.1|PPP5_SOLLC//2.00E-55//RecName: Full=Serine/threonine-protein phosphatase 5; AltName: Full=LePP5 >gi|28141004|gb|AAO26213.1| type 5 protein serine/threonine phosphatase 62 kDa isoform [Solanum lycopersicum] >gi|28141085|gb|AAO26215.1| type 5 protein serine/threonine phosphatase 62 kDa isoform [Solanum lycopersicum]	Unigene100191_All//5.00E-47//AT2G42810| PP5.2 (PROTEIN PHOSPHATASE 5.2); phosphoprotein phosphatase/ protein binding / protein serine/threonine phosphatase	Unigene100191_All//2.00E-167//CO074097				nr +
Unigene100195_All	374	4.62	0.00	2.71	3.20	3.22	4.59	0.45	0.56	0.43	-1.22E+01	5.34E-10	Down	-7.71E-01	1.40E-01		1.17E-02	9.99E-01		5.22E-01	3.34E-01		3.05E-01	8.37E-01		-8.58E-02	9.67E-01				Unigene100195_All//3.00E-85//BF275481				
Unigene100196_All	376	3.48	3.77	3.26	1.06	2.56	2.52	1.01	4.45	1.17	1.17E-01	8.24E-01		-9.49E-02	8.72E-01		1.27E+00	8.43E-02		1.25E+00	9.60E-02		2.14E+00	3.22E-05	Up	2.04E-01	8.26E-01				Unigene100196_All//3.00E-131//TC172967				
Unigene100224_All	206	3.81	0.00	1.03	0.48	1.84	3.51	1.85	0.81	0.19	-1.19E+01	1.09E-04	Down	-1.88E+00	4.13E-02		1.93E+00	1.94E-01		2.86E+00	7.13E-03		-1.19E+00	3.10E-01		-3.26E+00	2.22E-02								
Unigene10024_All	305	0.00	1.00	1.75	0.00	0.11	0.00	0.55	3.01	3.40	9.96E+00	4.45E-02		1.08E+01	4.05E-03		6.82E+00	8.43E-01		null	null		2.44E+00	1.33E-03		2.61E+00	2.37E-04	Up							
Unigene10025_All	446	0.00	0.00	0.00	0.00	0.00	0.00	2.28	2.44	0.00	null	null		null	null		null	null		null	null		9.88E-02	8.71E-01		-1.12E+01	1.35E-07	Down	Unigene10025_All//gi|195606812|gb|ACG25236.1|//1.00E-66//60S ribosomal protein L3 [Zea mays] >gi|195619674|gb|ACG31667.1| 60S ribosomal protein L3 [Zea mays] >gi|195626206|gb|ACG34933.1| 60S ribosomal protein L3 [Zea mays] >gi|195641988|gb|ACG40462.1| 60S ribosomal protein L3 [Zea mays]	Unigene10025_All//3.00E-60//AT1G43170| ARP1 (ARABIDOPSIS RIBOSOMAL PROTEIN 1); structural constituent of ribosome	Unigene10025_All//6.00E-13//TC135599		ko03010|Ribosome	GO:0005198//GO:0010467//GO:0015934	nr -
Unigene10028_All	568	0.00	0.00	0.00	0.44	0.55	0.32	2.90	1.47	0.35	null	null		null	null		3.15E-01	8.39E-01		-4.63E-01	7.45E-01		-9.80E-01	3.32E-02		-3.05E+00	1.31E-07	Down	Unigene10028_All//gi|32400679|gb|AAP80589.1|AF277102_1//1.00E-07//protease [Gossypium hirsutum]					GO:0003676//GO:0006259//GO:0008233	nr -
Unigene1003_All	1089	4.13	3.91	4.60	11.39	9.96	10.20	24.81	20.11	8.93	-8.02E-02	7.79E-01		1.54E-01	6.28E-01		-1.94E-01	2.78E-01		-1.59E-01	3.93E-01		-3.03E-01	1.36E-03		-1.47E+00	9.09E-46	Down	Unigene1003_All//gi|115441759|ref|NP_001045159.1|//2.00E-62//Os01g0911200 [Oryza sativa Japonica Group] >gi|113534690|dbj|BAF07073.1| Os01g0911200 [Oryza sativa Japonica Group]	Unigene1003_All//1.00E-54//AT4G21150| HAP6 (HAPLESS 6); dolichyl-diphosphooligosaccharide-protein glycotransferase	Unigene1003_All//0.00E+00//TC136242		ko04141|Protein processing in endoplasmic reticulum//ko00510|N-Glycan biosynthesis	GO:0004576//GO:0005618//GO:0005789//GO:0006487//GO:0006950//GO:0031410	nr +
Unigene100305_All	321	2.12	4.42	6.30	2.48	4.51	3.80	1.05	4.82	4.10	1.06E+00	6.14E-02		1.57E+00	1.64E-03		8.60E-01	1.31E-01		6.14E-01	3.52E-01		2.19E+00	5.11E-05	Up	1.96E+00	5.04E-04	Up	Unigene100305_All//gi|30684602|ref|NP_850606.1|//2.00E-07//ATRBL14 (ARABIDOPSIS RHOMBOID-LIKE PROTEIN 14); zinc ion binding [Arabidopsis thaliana] >gi|75247670|sp|Q8RXW0.1|Y3761_ARATH RecName: Full=Uncharacterized protein At3g17611	Unigene100305_All//3.00E-12//AT3G17611| ATRBL14 (ARABIDOPSIS RHOMBOID-LIKE PROTEIN 14); zinc ion binding	Unigene100305_All//2.00E-116//gi|78346546|gb|DT566820.1|DT566820				nr -
Unigene10031_All	443	0.00	15.56	7.21	0.00	0.00	0.00	0.00	0.00	0.00	1.39E+01	2.25E-39	Up	1.28E+01	1.15E-17	Up	null	null		null	null		null	null		null	null								
Unigene10032_All	274	13.75	4.99	9.33	16.00	23.50	19.78	96.60	86.79	79.97	-1.46E+00	1.16E-05	Down	-5.60E-01	9.29E-02		5.55E-01	1.45E-02		3.06E-01	2.81E-01		-1.54E-01	1.39E-01		-2.73E-01	3.59E-03				Unigene10032_All//2.00E-73//gi|48775848|gb|CO085214.1|CO085214				
Unigene100336_All	397	2.77	0.13	0.94	1.38	0.35	0.46	0.11	0.00	0.00	-4.44E+00	2.09E-05	Down	-1.56E+00	3.05E-02		-1.99E+00	5.13E-02		-1.60E+00	1.32E-01		-6.73E+00	6.68E-01		-6.73E+00	6.21E-01		Unigene100336_All//gi|50512303|gb|AAT77550.1|//1.00E-21//9DC3 [Solanum pimpinellifolium]	Unigene100336_All//7.00E-07//AT3G53240| AtRLP45 (Receptor Like Protein 45); protein binding	Unigene100336_All//7.00E-09//gi|78354692|gb|DT574966.1|DT574966				nr +
Unigene10034_All	519	0.00	0.00	0.00	0.00	0.00	0.00	3.34	1.61	0.15	null	null		null	null		null	null		null	null		-1.05E+00	1.87E-02		-4.44E+00	1.94E-10	Down	Unigene10034_All//gi|19908695|gb|AAM02970.1|AF421537_1//2.00E-86//beta-tubulin [Crypthecodinium cohnii]	Unigene10034_All//1.00E-70//AT1G75780| TUB1; GTP binding / GTPase/ structural molecule	Unigene10034_All//5.00E-32//TC141009		ko04145|Phagosome	GO:0007017//GO:0015630//GO:0017111//GO:0032561//GO:0032991//GO:0043623	nr +
Unigene100353_All	206	0.00	4.18	0.00	0.00	0.67	0.66	0.62	0.81	0.58	1.20E+01	4.95E-05	Up	null	null		9.39E+00	3.21E-01		9.36E+00	3.14E-01		3.99E-01	8.15E-01		-8.57E-02	9.71E-01								
Unigene100355_All	246	2.98	8.86	8.66	3.44	2.66	19.09	386.71	542.50	1048.60	1.57E+00	6.61E-04	Up	1.54E+00	1.42E-03		-3.72E-01	6.06E-01		2.47E+00	1.25E-13	Up	4.88E-01	4.82E-34		1.44E+00	0.00E+00	Up	Unigene100355_All//gi|89258498|gb|ABD65462.1|//2.00E-26//short chain alcohol dehydrogenase [Gossypium hirsutum]	Unigene100355_All//2.00E-16//AT2G47140| short-chain dehydrogenase/reductase (SDR) family protein	Unigene100355_All//2.00E-122//ES831528		ko00906|Carotenoid biosynthesis		nr +
Unigene10038_All	634	0.00	0.00	0.00	0.00	0.00	0.00	1.40	4.55	1.26	null	null		null	null		null	null		null	null		1.70E+00	1.80E-06	Up	-1.56E-01	7.94E-01		Unigene10038_All//gi|15227189|ref|NP_182322.1|//3.00E-59//RKF3 (RECEPTOR-LIKE KINASE IN IN FLOWERS 3); kinase/ receptor signaling protein serine/threonine kinase [Arabidopsis thaliana] >gi|75319082|sp|P93050.1|RKF3_ARATH RecName: Full=Probable LRR receptor-like serine/threonine-protein kinase RKF3; AltName: Full=Receptor-like kinase in flowers 3; Flags: Precursor >gi|1785621|emb|CAB06335.1| AtPK2324 [Arabidopsis thaliana] >gi|2465927|gb|AAC50045.1| receptor-like serine/threonine kinase [Arabidopsis thaliana] >gi|14335116|gb|AAK59837.1| At2g48010/T9J23.16 [Arabidopsis thaliana] >gi|23308235|gb|AAN18087.1| At2g48010/T9J23.16 [Arabidopsis thaliana]	Unigene10038_All//6.00E-59//AT2G48010| RKF3 (RECEPTOR-LIKE KINASE IN IN FLOWERS 3); kinase/ receptor signaling protein serine/threonine kinase			ko04626|Plant-pathogen interaction//ko00270|Cysteine and methionine metabolism	GO:0004674//GO:0004713//GO:0004871//GO:0006464//GO:0016020//GO:0032559	nr -
Unigene1004_All	2373	6.59	3.35	5.54	15.32	13.50	13.93	26.46	13.54	9.80	-9.76E-01	1.72E-11		-2.50E-01	1.08E-01		-1.83E-01	4.48E-02		-1.38E-01	1.74E-01		-9.66E-01	1.10E-52		-1.43E+00	9.45E-101	Down	Unigene1004_All//gi|297804040|ref|XP_002869904.1|//0.00E+00//ribophorin II family protein [Arabidopsis lyrata subsp. lyrata] >gi|297315740|gb|EFH46163.1| ribophorin II family protein [Arabidopsis lyrata subsp. lyrata]	Unigene1004_All//0.00E+00//AT4G21150| HAP6 (HAPLESS 6); dolichyl-diphosphooligosaccharide-protein glycotransferase	Unigene1004_All//0.00E+00//TC136242		ko04141|Protein processing in endoplasmic reticulum//ko00510|N-Glycan biosynthesis	GO:0004576//GO:0005618//GO:0005789//GO:0006487//GO:0006950//GO:0031410	nr +
Unigene10040_All	235	2.67	11.00	4.31	2.54	0.73	0.77	0.36	0.36	0.17	2.04E+00	4.81E-06	Up	6.88E-01	3.42E-01		-1.80E+00	5.87E-02		-1.73E+00	9.04E-02		-1.65E-02	1.02E+00		-1.09E+00	6.79E-01								
Unigene10042_All	323	1.30	0.63	0.49	3.39	1.39	0.28	1.31	0.26	0.25	-1.05E+00	3.66E-01		-1.39E+00	2.80E-01		-1.29E+00	4.07E-02		-3.60E+00	5.02E-05	Down	-2.34E+00	5.47E-02		-2.41E+00	3.94E-02		Unigene10042_All//gi|147823314|emb|CAN77450.1|//2.00E-19//hypothetical protein [Vitis vinifera]		Unigene10042_All//3.00E-14//ES792704				nr -
Unigene10043_All	524	0.00	8.80	41.46	0.00	0.00	3.45	0.00	0.00	0.00	1.31E+01	2.84E-26	Up	1.53E+01	7.30E-123	Up	null	null		1.18E+01	8.92E-11	Up	null	null		null	null		Unigene10043_All//gi|270383724|dbj|BAI53120.1|//3.00E-83//ribulose-1,5-bisphosphate carboxylase/oxygenase small subunit [Eucalyptus globulus]	Unigene10043_All//1.00E-81//AT5G38430| ribulose bisphosphate carboxylase small chain 1B / RuBisCO small subunit 1B (RBCS-1B) (ATS1B)	Unigene10043_All//1.00E-23//gi|48752358|gb|CO082877.1|CO082877		ko00710|Carbon fixation in photosynthetic organisms//ko00630|Glyoxylate and dicarboxylate metabolism	GO:0005576//GO:0005840//GO:0006950//GO:0009526//GO:0009532//GO:0009534//GO:0009639//GO:0015977//GO:0016491//GO:0016831//GO:0043094//GO:0044436//GO:0046914	nr -
Unigene10044_All	400	0.00	20.77	12.11	0.00	0.00	0.00	0.00	0.00	0.00	1.43E+01	1.52E-47	Up	1.36E+01	6.11E-27	Up	null	null		null	null		null	null		null	null		Unigene10044_All//gi|89513312|gb|ABD74515.1|//1.00E-40//ribosomal protein [Pteris vittata]	Unigene10044_All//2.00E-39//AT1G36240| 60S ribosomal protein L30 (RPL30A)			ko03010|Ribosome	GO:0005198//GO:0010467//GO:0030529	nr +
Unigene100446_All	343	22.88	18.32	11.02	27.01	24.09	28.97	46.47	20.96	28.11	-3.21E-01	1.36E-01		-1.05E+00	1.56E-06	Down	-1.65E-01	4.51E-01		1.01E-01	6.43E-01		-1.15E+00	2.74E-18	Down	-7.25E-01	3.42E-09		Unigene100446_All//gi|164564736|dbj|BAF98218.1|//7.00E-29//CM0216.350.nc [Lotus japonicus]	Unigene100446_All//1.00E-08//AT4G39200| 40S ribosomal protein S25 (RPS25E)	Unigene100446_All//7.00E-86//BF276543		ko03010|Ribosome		nr -
Unigene10047_All	390	0.00	3.77	2.18	0.00	0.00	0.00	0.00	0.00	0.00	1.19E+01	2.11E-08	Up	1.11E+01	9.04E-05	Up	null	null		null	null		null	null		null	null		Unigene10047_All//gi|224482651|gb|ACN50182.1|//5.00E-14//annexin [Annona cherimola]	Unigene10047_All//9.00E-10//AT1G68090| ANN5; calcium ion binding / calcium-dependent phospholipid binding					nr -
Unigene10048_All	592	0.00	4.02	1.71	0.00	0.00	0.00	0.00	0.00	0.00	1.20E+01	1.50E-13	Up	1.07E+01	1.28E-05	Up	null	null		null	null		null	null		null	null		Unigene10048_All//gi|300120616|emb|CBK20170.2|//4.00E-23//unnamed protein product [Blastocystis hominis]						nr +
Unigene10049_All	212	7.16	0.96	1.76	4.46	4.39	5.75	0.20	0.20	0.00	-2.90E+00	2.07E-05	Down	-2.03E+00	1.23E-03		-2.57E-02	9.50E-01		3.66E-01	6.04E-01		-1.67E-02	1.01E+00		-7.64E+00	6.21E-01				Unigene10049_All//2.00E-10//ES837561				
Unigene1005_All	1493	17.24	6.99	18.94	4.77	7.80	7.77	1.61	1.23	0.80	-1.30E+00	7.72E-29	Down	1.36E-01	2.46E-01		7.08E-01	5.86E-06		7.04E-01	1.59E-05		-3.90E-01	3.15E-01		-1.01E+00	4.32E-03		Unigene1005_All//gi|30678625|ref|NP_567175.2|//1.00E-137//ANTR2; inorganic phosphate transmembrane transporter/ organic anion transmembrane transporter [Arabidopsis thaliana] >gi|75244391|sp|Q8GX78.1|ANTR2_ARATH RecName: Full=Probable anion transporter 2, chloroplastic; AltName: Full=Phosphate transporter PHT4;4; Flags: Precursor >gi|32306495|gb|AAP78931.1| At4g00370 [Arabidopsis thaliana]	Unigene1005_All//7.00E-135//AT4G00370| ANTR2; inorganic phosphate transmembrane transporter/ organic anion transmembrane transporter	Unigene1005_All//0.00E+00//TC172647				nr -
Unigene10050_All	394	0.00	0.13	0.00	0.00	0.00	0.34	1.40	3.08	4.95	7.01E+00	6.24E-01		null	null		null	null		8.43E+00	3.14E-01		1.14E+00	4.07E-02		1.83E+00	3.51E-05	Up	Unigene10050_All//gi|224097212|ref|XP_002310878.1|//5.00E-19//1-aminocyclopropane-1-carboxylate [Populus trichocarpa] >gi|222853781|gb|EEE91328.1| 1-aminocyclopropane-1-carboxylate [Populus trichocarpa]	Unigene10050_All//7.00E-18//AT5G65800| ACS5 (ACC SYNTHASE 5); 1-aminocyclopropane-1-carboxylate synthase			ko00270|Cysteine and methionine metabolism	GO:0005515//GO:0006412//GO:0006555//GO:0009725//GO:0016846	nr +

Unigene100500_All	896	0.41	0.00	0.65	0.67	0.15	2.37	0.28	0.00	0.00	-8.68E+00	1.97E-02		6.77E-01	4.83E-01		-2.12E+00	3.06E-02		1.83E+00	1.26E-04	Up	-8.15E+00	4.07E-02		-8.15E+00	3.06E-02		Unigene100500_All//gi|5453379|gb|AAD43561.1|AF155124_1//1.00E-125//bacterial-induced peroxidase precursor [Gossypium hirsutum]	Unigene100500_All//2.00E-83//AT5G05340| peroxidase, putative	Unigene100500_All//0.00E+00//NP385887		ko00940|Phenylpropanoid biosynthesis//ko00360|Phenylalanine metabolism	GO:0003824//GO:0005506//GO:0006950//GO:0008152//GO:0016209//GO:0031410	nr +
Unigene10051_All	479	0.55	4.23	2.11	0.62	0.93	0.66	0.35	0.44	1.33	2.95E+00	4.86E-07	Up	1.95E+00	1.37E-02		5.83E-01	6.26E-01		8.09E-02	9.50E-01		3.05E-01	8.37E-01		1.91E+00	2.60E-02								
Unigene100521_All	242	0.00	0.00	0.00	0.00	0.00	0.56	0.00	0.00	3.62	null	null		null	null		null	null		9.13E+00	3.14E-01		null	null		1.18E+01	2.05E-06	Up							
Unigene10053_All	497	0.00	0.00	0.43	0.00	0.62	1.54	0.00	0.25	1.52	null	null		8.74E+00	1.42E-01		9.28E+00	4.55E-02		1.06E+01	1.27E-04	Up	7.98E+00	2.41E-01		1.06E+01	1.39E-05	Up	Unigene10053_All//gi|66792688|gb|AAY56446.1|//5.00E-39//At1g63930 [Arabidopsis thaliana] >gi|115311479|gb|ABI93920.1| At1g63930 [Arabidopsis thaliana]	Unigene10053_All//9.00E-37//AT1G63930| unknown protein	Unigene10053_All//4.00E-113//TC174086				nr +
Unigene10057_All	358	32.30	27.60	34.21	25.60	27.51	40.75	17.83	13.66	3.23	-2.27E-01	2.05E-01		8.30E-02	6.36E-01		1.04E-01	6.29E-01		6.70E-01	2.90E-06		-3.85E-01	7.45E-02		-2.47E+00	5.41E-22	Down							
Unigene10058_All	372	9.14	1.50	2.29	4.55	2.68	1.58	0.00	0.00	0.00	-2.61E+00	3.46E-10	Down	-2.00E+00	3.48E-07	Down	-7.62E-01	1.18E-01		-1.53E+00	3.34E-03		null	null		null	null		Unigene10058_All//gi|73761705|gb|AAZ83352.1|//1.00E-21//myb transcription factor MYB30 [Gossypium hirsutum]		Unigene10058_All//1.00E-19//TC145072			GO:0009725//GO:0009987	nr +
Unigene10059_All	446	0.00	0.00	0.00	0.00	0.00	0.00	3.60	0.56	1.25	null	null		null	null		null	null		null	null		-2.68E+00	2.38E-06	Down	-1.53E+00	1.16E-03								ESTscan -
Unigene100616_All	511	3.38	0.59	2.40	2.05	1.95	0.44	0.66	0.16	2.11	-2.51E+00	2.66E-05	Down	-4.95E-01	3.58E-01		-6.70E-02	9.06E-01		-2.21E+00	3.25E-03		-2.02E+00	1.37E-01		1.67E+00	6.16E-03		Unigene100616_All//gi|115452803|ref|NP_001050002.1|//1.00E-14//Os03g0328900 [Oryza sativa Japonica Group] >gi|108707942|gb|ABF95737.1| Zinc finger C-x8-C-x5-C-x3-H type family protein, expressed [Oryza sativa (japonica cultivar-group)] >gi|113548473|dbj|BAF11916.1| Os03g0328900 [Oryza sativa Japonica Group]	Unigene100616_All//6.00E-14//AT3G51950| zinc finger (CCCH-type) family protein / RNA recognition motif (RRM)-containing protein		C3H		GO:0003676//GO:0046914	nr -
Unigene10062_All	329	0.16	0.31	0.00	2.73	0.84	1.51	7.07	40.40	7.63	9.54E-01	7.28E-01		-7.31E+00	6.36E-01		-1.70E+00	1.98E-02		-8.52E-01	2.63E-01		2.51E+00	2.22E-44	Up	1.10E-01	7.55E-01				Unigene10062_All//4.00E-07//TC158719				
Unigene10065_All	297	0.00	4.44	2.87	0.00	0.00	0.00	0.00	0.00	0.00	1.21E+01	1.50E-07	Up	1.15E+01	9.04E-05	Up	null	null		null	null		null	null		null	null								ESTscan -
Unigene10066_All	248	0.00	0.00	3.01	0.60	1.39	1.27	1.02	0.67	0.16	null	null		1.16E+01	3.28E-04	Up	1.20E+00	4.26E-01		1.08E+00	4.76E-01		-6.02E-01	6.49E-01		-2.67E+00	1.15E-01								
Unigene10067_All	547	269.54	354.04	239.77	323.84	777.85	534.53	6.18	18.64	12.75	3.93E-01	4.11E-27		-1.69E-01	1.43E-04		1.26E+00	0.00E+00	Up	7.23E-01	3.30E-130		1.59E+00	3.53E-19	Up	1.04E+00	1.48E-07	Up	Unigene10067_All//gi|194466223|gb|ACF74342.1|//4.00E-34//gonadotropin beta chain [Arachis hypogaea]	Unigene10067_All//2.00E-21//AT5G14920| gibberellin-regulated family protein	Unigene10067_All//0.00E+00//TC163273				nr +
Unigene10068_All	577	0.00	28.01	7.38	0.00	0.00	0.00	0.00	0.00	0.00	1.48E+01	6.97E-93	Up	1.29E+01	1.22E-23	Up	null	null		null	null		null	null		null	null		Unigene10068_All//gi|116310923|emb|CAH67861.1|//1.00E-10//B0403H10-OSIGBa0105A11.13 [Oryza sativa (indica cultivar-group)]				ko00190|Oxidative phosphorylation//ko04145|Phagosome		nr +
Unigene10069_All	323	0.00	0.00	0.00	0.00	0.00	0.00	10.08	11.78	0.37	null	null		null	null		null	null		null	null		2.24E-01	4.72E-01		-4.77E+00	6.52E-20	Down	Unigene10069_All//gi|297829770|ref|XP_002882767.1|//4.00E-52//actin-11 [Arabidopsis lyrata subsp. lyrata] >gi|297328607|gb|EFH59026.1| actin-11 [Arabidopsis lyrata subsp. lyrata]	Unigene10069_All//2.00E-53//AT3G12110| ACT11 (actin-11); structural constituent of cytoskeleton	Unigene10069_All//6.00E-40//TC153744		ko04145|Phagosome	GO:0007010//GO:0016020//GO:0032559//GO:0043231//GO:0043232	nr -
Unigene100697_All	211	0.00	0.96	0.00	0.47	0.49	1.50	0.40	3.96	0.00	9.91E+00	1.38E-01		null	null		5.22E-02	9.69E-01		1.67E+00	3.09E-01		3.31E+00	3.10E-04	Up	-8.65E+00	3.63E-01								
Unigene10070_All	624	0.08	0.00	0.00	0.08	0.11	0.22	0.14	0.00	1.79	-6.39E+00	6.24E-01		-6.39E+00	6.36E-01		4.68E-01	9.63E-01		1.44E+00	6.41E-01		-7.08E+00	4.09E-01		3.72E+00	4.35E-06	Up	Unigene10070_All//gi|124359981|gb|ABN07997.1|//7.00E-09//contains similarity to reverse trancriptase , related [Medicago truncatula]	Unigene10070_All//6.00E-07//AT4G29090| reverse transcriptase, putative / RNA-dependent DNA polymerase, putative				GO:0005488	nr -
Unigene10071_All	379	0.28	6.82	4.36	0.39	0.36	2.74	3.79	8.71	98.49	4.63E+00	3.34E-12	Up	3.98E+00	5.16E-07	Up	-1.18E-01	9.12E-01		2.80E+00	8.13E-04	Up	1.20E+00	1.14E-04	Up	4.70E+00	8.30E-217	Up			Unigene10071_All//3.00E-159//DT461826				
Unigene100711_All	265	2.37	4.02	1.61	0.38	0.65	5.79	0.80	0.00	0.75	7.61E-01	2.48E-01		-5.60E-01	5.54E-01		7.89E-01	7.72E-01		3.95E+00	4.90E-07	Up	-9.64E+00	7.49E-02		-8.57E-02	9.62E-01		Unigene100711_All//gi|225451804|ref|XP_002281338.1|//2.00E-09//PREDICTED: brassinosteroid-6-oxidase [Vitis vinifera]	Unigene100711_All//7.00E-07//AT5G38970| BR6OX1 (BRASSINOSTEROID-6-OXIDASE 1); monooxygenase/ oxygen binding	Unigene100711_All//3.00E-103//TC166913		ko00905|Brassinosteroid biosynthesis	GO:0000226//GO:0005506//GO:0006091//GO:0009646//GO:0016129//GO:0016491//GO:0031224//GO:0032501//GO:0048878	nr +
Unigene10072_All	595	0.00	0.00	0.00	0.50	0.00	0.00	1.63	0.42	10.58	null	null		null	null		-8.97E+00	2.02E-02		-8.97E+00	4.01E-02		-1.96E+00	4.44E-03		2.69E+00	1.68E-23	Up			Unigene10072_All//0.00E+00//gi|109888809|gb|DW517778.1|DW517778				
Unigene10074_All	962	0.54	0.95	1.11	4.87	4.19	2.77	0.75	0.04	0.33	8.02E-01	2.71E-01		1.03E+00	1.43E-01		-2.17E-01	4.91E-01		-8.13E-01	3.07E-03		-4.11E+00	2.92E-04	Down	-1.17E+00	1.09E-01		Unigene10074_All//gi|39725413|emb|CAE09057.1|//2.00E-70//MYB transcription factor [Eucalyptus gunnii]	Unigene10074_All//4.00E-53//AT3G08500| MYB83 (myb domain protein 83); DNA binding / transcription factor	Unigene10074_All//1.00E-166//DW508945	MYB		GO:0001071//GO:0003676//GO:0006350//GO:0009832//GO:0010033//GO:0010468//GO:0043231	nr +
Unigene10075_All	585	0.00	9.79	5.46	0.00	0.00	0.00	0.00	0.00	0.00	1.33E+01	1.13E-32	Up	1.24E+01	1.15E-17	Up	null	null		null	null		null	null		null	null		Unigene10075_All//gi|159483465|ref|XP_001699781.1|//2.00E-51//eukaryotic initiation factor [Chlamydomonas reinhardtii] >gi|158281723|gb|EDP07477.1| eukaryotic initiation factor [Chlamydomonas reinhardtii]	Unigene10075_All//1.00E-36//AT2G04520| eukaryotic translation initiation factor 1A, putative / eIF-1A, putative / eIF-4C, putative				GO:0006412//GO:0008135	nr +
Unigene100750_All	499	0.10	0.00	0.96	1.00	1.73	1.18	2.46	0.75	0.56	-6.71E+00	6.23E-01		3.19E+00	3.48E-02		7.89E-01	3.21E-01		2.37E-01	7.99E-01		-1.70E+00	3.32E-03		-2.14E+00	3.24E-04	Down	Unigene100750_All//gi|240254302|ref|NP_176378.4|//3.00E-60//galactolipase/ phospholipase [Arabidopsis thaliana]	Unigene100750_All//2.00E-61//AT1G61850| galactolipase/ phospholipase	Unigene100750_All//6.00E-10//EV498230			GO:0003824//GO:0005488//GO:0044238	nr -
Unigene10076_All	406	0.00	0.00	0.00	0.00	0.00	0.22	4.58	1.13	2.06	null	null		null	null		null	null		7.80E+00	4.84E-01		-2.02E+00	2.13E-05	Down	-1.15E+00	5.56E-03		Unigene10076_All//gi|224114738|ref|XP_002332312.1|//9.00E-08//predicted protein [Populus trichocarpa] >gi|222832311|gb|EEE70788.1| predicted protein [Populus trichocarpa]						nr -
Unigene10078_All	346	0.00	108.37	30.63	0.00	0.00	0.00	0.00	0.00	0.00	1.67E+01	2.24E-216	Up	1.49E+01	1.66E-59	Up	null	null		null	null		null	null		null	null		Unigene10078_All//gi|195620360|gb|ACG32010.1|//2.00E-39//60S ribosomal protein L27a-3 [Zea mays]	Unigene10078_All//1.00E-26//AT1G23290| RPL27AB; structural constituent of ribosome	Unigene10078_All//3.00E-20//TC169611		ko03010|Ribosome	GO:0005198//GO:0010467//GO:0030529	nr -
Unigene100795_All	365	0.00	0.00	0.00	0.14	0.47	0.00	6.49	5.73	1.42	null	null		null	null		1.79E+00	5.22E-01		-7.09E+00	6.47E-01		-1.80E-01	6.34E-01		-2.19E+00	1.28E-07	Down	Unigene100795_All//gi|9294177|dbj|BAB02079.1|//9.00E-20//nodulin-lile protein [Arabidopsis thaliana]	Unigene100795_All//4.00E-21//AT3G25190| nodulin, putative	Unigene100795_All//5.00E-62//gi|109868191|gb|DW497167.1|DW497167				nr -
Unigene10084_All	254	0.00	4.19	1.68	0.00	0.00	0.00	0.00	0.00	0.00	1.20E+01	3.82E-06	Up	1.07E+01	1.39E-02		null	null		null	null		null	null		null	null		Unigene10084_All//gi|301111870|ref|XP_002905014.1|//6.00E-25//superoxide dismutase, mitochondrial precursor [Phytophthora infestans T30-4] >gi|262095344|gb|EEY53396.1| superoxide dismutase, mitochondrial precursor [Phytophthora infestans T30-4]	Unigene10084_All//3.00E-19//AT3G10920| MSD1 (MANGANESE SUPEROXIDE DISMUTASE 1); metal ion binding / superoxide dismutase			ko04146|Peroxisome	GO:0003824//GO:0005488//GO:0044237	nr -
Unigene100844_All	296	53.38	23.28	17.63	74.89	57.47	77.82	80.99	27.82	35.80	-1.20E+00	7.32E-16	Down	-1.60E+00	3.29E-23	Down	-3.82E-01	4.81E-04		5.53E-02	6.72E-01		-1.54E+00	3.80E-42	Down	-1.18E+00	5.17E-29	Down	Unigene100844_All//gi|15231288|ref|NP_187967.1|//7.00E-26//60S ribosomal protein L7 (RPL7D) [Arabidopsis thaliana] >gi|42572417|ref|NP_974304.1| 60S ribosomal protein L7 (RPL7D) [Arabidopsis thaliana] >gi|42572419|ref|NP_974305.1| 60S ribosomal protein L7 (RPL7D) [Arabidopsis thaliana] >gi|38503370|sp|Q9LHP1.1|RL74_ARATH RecName: Full=60S ribosomal protein L7-4 >gi|11994560|dbj|BAB02600.1| 60S ribosomal protein L7 [Arabidopsis thaliana] >gi|14532552|gb|AAK64004.1| AT3g13580/K20M4_2 [Arabidopsis thaliana] >gi|15810196|gb|AAL06999.1| AT3g13580/K20M4_2 [Arabidopsis thaliana] >gi|18655395|gb|AAL76153.1| AT3g13580/K20M4_2 [Arabidopsis thaliana]	Unigene100844_All//3.00E-27//AT3G13580| 60S ribosomal protein L7 (RPL7D)	Unigene100844_All//5.00E-74//EV497578		ko03010|Ribosome	GO:0005198//GO:0005488//GO:0009536//GO:0010467//GO:0015934//GO:0016020//GO:0030312//GO:0031981	nr -
Unigene10085_All	234	65.07	19.70	29.58	99.20	79.61	118.51	62.70	16.61	42.90	-1.72E+00	6.70E-26	Down	-1.14E+00	1.52E-13	Down	-3.17E-01	3.63E-03		2.57E-01	1.58E-02		-1.92E+00	3.80E-35	Down	-5.47E-01	1.60E-05		Unigene10085_All//gi|76160947|gb|ABA40437.1|//3.00E-11//40S ribosomal protein S7-like protein [Solanum tuberosum] >gi|77999315|gb|ABB17004.1| ribosomal protein S7-like protein [Solanum tuberosum] >gi|82623373|gb|ABB87101.1| 40S ribosomal protein S7-like protein-like [Solanum tuberosum]	Unigene10085_All//2.00E-10//AT3G02560| 40S ribosomal protein S7 (RPS7B)	Unigene10085_All//1.00E-89//gi|13355512|gb|BG445860.1|BG445860		ko03010|Ribosome	GO:0005198//GO:0010467//GO:0030529	nr -
Unigene10088_All	459	0.00	0.00	0.00	0.33	0.68	0.79	1.47	1.73	3.99	null	null		null	null		1.05E+00	5.32E-01		1.27E+00	3.82E-01		2.31E-01	7.35E-01		1.44E+00	9.58E-04	Up							
Unigene10089_All	472	0.00	0.00	0.00	0.00	0.22	0.19	3.31	0.44	1.18	null	null		null	null		7.77E+00	4.59E-01		7.58E+00	4.84E-01		-2.90E+00	1.13E-06	Down	-1.49E+00	1.74E-03		Unigene10089_All//gi|15239653|ref|NP_200262.1|//1.00E-49//DFL1 (DWARF IN LIGHT 1); indole-3-acetic acid amido synthetase [Arabidopsis thaliana] >gi|62900334|sp|Q9LSQ4.1|GH36_ARATH RecName: Full=Indole-3-acetic acid-amido synthetase GH3.6; AltName: Full=Auxin-responsive GH3-like protein 6; Short=AtGH3-6; AltName: Full=Protein DWARF IN LIGHT 1; Short=DFL-1 >gi|8885594|dbj|BAA97524.1| auxin-responsive-like protein [Arabidopsis thaliana] >gi|11041726|dbj|BAB17304.1| auxin-responsive GH3 homologue [Arabidopsis thaliana] >gi|59958336|gb|AAX12878.1| At5g54510 [Arabidopsis thaliana] >gi|209414530|gb|ACI46505.1| At5g54510 [Arabidopsis thaliana]	Unigene10089_All//7.00E-51//AT5G54510| DFL1 (DWARF IN LIGHT 1); indole-3-acetic acid amido synthetase	Unigene10089_All//1.00E-07//DW501990			GO:0000902//GO:0009314//GO:0009536//GO:0009755//GO:0016881//GO:0048878	nr -
Unigene100896_All	313	0.33	0.97	0.68	3.18	0.44	0.87	0.54	0.00	0.25	1.54E+00	3.24E-01		1.03E+00	5.93E-01		-2.85E+00	2.37E-04	Down	-1.88E+00	1.18E-02		-9.08E+00	1.35E-01		-1.09E+00	5.54E-01								
Unigene100906_All	390	0.13	1.82	0.41	0.26	0.18	1.39	16.26	6.43	81.62	3.76E+00	2.36E-03		1.61E+00	5.12E-01		-5.33E-01	8.20E-01		2.44E+00	4.42E-02		-1.34E+00	1.23E-09	Down	2.33E+00	4.31E-98	Up	Unigene100906_All//gi|75297894|sp|Q84MB3.1|ACCH1_ARATH//4.00E-33//RecName: Full=1-aminocyclopropane-1-carboxylate oxidase homolog 1 >gi|30102640|gb|AAP21238.1| At1g06620 [Arabidopsis thaliana] >gi|110743652|dbj|BAE99663.1| oxidoreductase like protein [Arabidopsis thaliana]	Unigene100906_All//2.00E-34//AT1G06620| 2-oxoglutarate-dependent dioxygenase, putative	Unigene100906_All//1.00E-133//gi|109879784|gb|DW508754.1|DW508754		ko00941|Flavonoid biosynthesis//ko00402|Benzoxazinoid biosynthesis	GO:0008152//GO:0016706//GO:0046914	nr -
Unigene100908_All	2421	0.04	0.46	0.24	0.06	0.16	14.31	0.00	0.00	0.00	3.41E+00	1.24E-04	Up	2.49E+00	3.76E-02		1.34E+00	3.40E-01		7.86E+00	2.13E-206	Up	null	null		null	null		Unigene100908_All//sp|Q16927|VIT1_AEDAE//3.00E-56//Vitellogenin-A1 OS=Aedes aegypti GN=VGA1 PE=1 SV=2						swissprot -

Unigene10091_All	904	0.00	8.02	2.71	0.00	0.00	0.00	0.00	0.00	0.00	1.30E+01	2.04E-41	Up	1.14E+01	1.64E-13	Up	null	null		null	null		null	null		null	null		Unigene10091_All//gi|301107486|ref|XP_002902825.1|//7.00E-52//long-chain-fatty-acid-CoA ligase [Phytophthora infestans T30-4] >gi|262097943|gb|EEY55995.1| long-chain-fatty-acid-CoA ligase [Phytophthora infestans T30-4]	Unigene10091_All//5.00E-17//AT4G14070| AAE15 (acyl-activating enzyme 15); long-chain-fatty-acid-[acyl-carrier-protein] ligase			ko00940|Phenylpropanoid biosynthesis//ko04146|Peroxisome//ko00360|Phenylalanine metabolism//ko00071|Fatty acid metabolism//ko00130|Ubiquinone and other terpenoid-quinone biosynthesis		nr -
Unigene10092_All	391	0.13	56.50	24.11	0.00	0.00	0.00	0.00	0.00	0.00	8.72E+00	7.66E-125	Up	7.49E+00	6.51E-51	Up	null	null		null	null		null	null		null	null								ESTscan +
Unigene10093_All	346	0.30	0.00	0.00	1.87	2.99	0.52	0.00	1.57	0.23	-8.24E+00	3.78E-01		-8.24E+00	4.00E-01		6.74E-01	3.49E-01		-1.84E+00	6.01E-02		1.06E+01	5.37E-04	Up	7.85E+00	4.07E-01				Unigene10093_All//8.00E-172//TC147034				
Unigene100964_All	213	0.00	0.00	0.00	0.47	0.32	4.45	0.20	0.98	0.00	null	null		null	null		-5.33E-01	8.20E-01		3.25E+00	5.84E-04	Up	2.31E+00	2.40E-01		-7.63E+00	6.21E-01				Unigene100964_All//7.00E-20//DR457055				
Unigene10098_All	531	0.00	0.00	0.00	0.00	0.00	0.00	2.23	9.21	1.05	null	null		null	null		null	null		null	null		2.05E+00	3.31E-13	Up	-1.09E+00	4.38E-02								
Unigene1010_All	4361	4.57	2.21	3.50	3.03	3.00	2.59	12.27	9.90	5.23	-1.05E+00	3.47E-16	Down	-3.83E-01	3.03E-03		-1.26E-02	9.52E-01		-2.26E-01	1.90E-01		-3.10E-01	1.50E-06		-1.23E+00	2.47E-68	Down	Unigene1010_All//gi|224053809|ref|XP_002297990.1|//0.00E+00//multidrug resistance protein ABC transporter family [Populus trichocarpa] >gi|222845248|gb|EEE82795.1| multidrug resistance protein ABC transporter family [Populus trichocarpa]	Unigene1010_All//0.00E+00//AT3G13080| ATMRP3; ATPase, coupled to transmembrane movement of substances / chlorophyll catabolite transporter/ glutathione S-conjugate-exporting ATPase	Unigene1010_All//3.00E-20//TC139924		ko02010|ABC transporters	GO:0006810//GO:0015405//GO:0017111//GO:0031224//GO:0032559	nr +
Unigene101002_All	382	0.00	0.00	0.00	0.52	0.09	0.12	1.77	0.11	0.52	null	null		null	null		-2.53E+00	2.34E-01		-2.14E+00	3.48E-01		-4.02E+00	5.36E-04	Down	-1.76E+00	2.80E-02		Unigene101002_All//gi|224097478|ref|XP_002310952.1|//9.00E-07//predicted protein [Populus trichocarpa] >gi|222850772|gb|EEE88319.1| predicted protein [Populus trichocarpa]	Unigene101002_All//2.00E-06//AT2G33840| tRNA synthetase class I (W and Y) family protein	Unigene101002_All//8.00E-126//gi|11204884|gb|BF273826.1|BF273826		ko00970|Aminoacyl-tRNA biosynthesis		nr -
Unigene10101_All	1285	0.00	0.00	0.00	0.00	0.00	0.00	4.71	11.84	1.58	null	null		null	null		null	null		null	null		1.33E+00	8.52E-22	Up	-1.57E+00	6.44E-12	Down							
Unigene101019_All	212	0.99	0.48	2.01	0.24	0.97	0.85	0.20	2.56	6.01	-1.05E+00	5.66E-01		1.03E+00	3.97E-01		2.05E+00	3.95E-01		1.86E+00	4.60E-01		3.68E+00	3.69E-03		4.91E+00	5.19E-08	Up			Unigene101019_All//4.00E-18//TC141091				
Unigene101039_All	270	0.39	0.38	0.20	0.92	1.79	2.01	0.16	0.93	9.74	-4.65E-02	9.91E-01		-9.75E-01	7.23E-01		9.53E-01	4.24E-01		1.12E+00	3.05E-01		2.57E+00	1.53E-01		5.96E+00	2.54E-17	Up			Unigene101039_All//2.00E-51//DT527515				
Unigene101047_All	207	2.27	1.22	7.97	6.26	3.99	0.65	0.61	0.00	0.00	-8.94E-01	3.89E-01		1.81E+00	1.76E-03		-6.48E-01	2.72E-01		-3.26E+00	1.98E-05	Down	-9.26E+00	2.37E-01		-9.26E+00	2.01E-01								
Unigene101050_All	625	0.00	0.00	0.00	0.32	0.11	0.72	1.76	0.00	0.45	null	null		null	null		-1.53E+00	4.17E-01		1.18E+00	3.38E-01		-1.08E+01	7.67E-08	Down	-1.98E+00	1.39E-03		Unigene101050_All//gi|119507465|dbj|BAF42040.1|//1.00E-90//pectin methylesterase 3 [Pyrus communis]	Unigene101050_All//6.00E-89//AT1G02810| pectinesterase family protein	Unigene101050_All//0.00E+00//TC138774		ko00500|Starch and sucrose metabolism//ko00040|Pentose and glucuronate interconversions	GO:0004091//GO:0005618//GO:0030234//GO:0071555	nr -
Unigene10106_All	383	0.96	0.26	0.83	1.30	1.17	0.59	2.21	0.11	0.31	-1.85E+00	1.87E-01		-1.97E-01	8.66E-01		-1.54E-01	8.50E-01		-1.14E+00	3.07E-01		-4.34E+00	4.56E-05	Down	-2.82E+00	5.38E-04	Down			Unigene10106_All//4.00E-07//ES837531				
Unigene10107_All	676	0.00	1.20	0.00	0.59	1.78	1.00	0.25	0.31	0.41	1.02E+01	9.39E-05	Up	null	null		1.60E+00	1.36E-02		7.66E-01	4.10E-01		3.05E-01	8.37E-01		7.21E-01	5.97E-01		Unigene10107_All//gi|297804010|ref|XP_002869889.1|//3.00E-63//At4g21420 [Arabidopsis lyrata subsp. lyrata] >gi|297315725|gb|EFH46148.1| At4g21420 [Arabidopsis lyrata subsp. lyrata]	Unigene10107_All//1.00E-08//AT5G42905| nucleic acid binding / ribonuclease H	Unigene10107_All//0.00E+00//gi|3326554|gb|AI055440.1|AI055440			GO:0005488//GO:0016891//GO:0043231	nr +
Unigene10109_All	924	0.00	12.94	4.21	0.05	0.00	0.00	0.00	0.00	0.00	1.37E+01	1.36E-68	Up	1.20E+01	1.52E-21	Up	-5.75E+00	5.67E-01		-5.75E+00	6.47E-01		null	null		null	null		Unigene10109_All//gi|297800072|ref|XP_002867920.1|//1.00E-18//glycosyl hydrolase family 18 protein [Arabidopsis lyrata subsp. lyrata] >gi|297313756|gb|EFH44179.1| glycosyl hydrolase family 18 protein [Arabidopsis lyrata subsp. lyrata]	Unigene10109_All//3.00E-17//AT4G19810| glycosyl hydrolase family 18 protein				GO:0003824	nr -
Unigene101106_All	204	0.00	0.00	0.00	0.49	0.00	0.44	27.57	49.99	76.16	null	null		null	null		-8.93E+00	3.18E-01		-1.41E-01	9.54E-01		8.59E-01	1.02E-07		1.47E+00	3.29E-26	Up			Unigene101106_All//6.00E-42//gi|31407394|gb|CD486429.1|CD486429				
Unigene10111_All	570	0.00	10.93	4.11	0.00	0.00	0.00	0.00	0.00	0.00	1.34E+01	1.38E-35	Up	1.20E+01	6.41E-13	Up	null	null		null	null		null	null		null	null		Unigene10111_All//gi|8954372|ref|NP_059361.1|//4.00E-34//NADH dehydrogenase subunit 4 [Cyanidioschyzon merolae] >gi|4115785|dbj|BAA36523.1| NADH-ubiquinone oxidoreductase chain 4 [Cyanidioschyzon merolae strain 10D]	Unigene10111_All//2.00E-14//ATMG00580| NADH dehydrogenase subunit 4			ko00190|Oxidative phosphorylation	GO:0016020//GO:0055114	nr -
Unigene10112_All	381	0.00	0.00	0.00	0.00	0.00	0.00	4.66	7.02	0.21	null	null		null	null		null	null		null	null		5.91E-01	8.97E-02		-4.48E+00	1.00E-10	Down							
Unigene10114_All	426	0.00	21.29	17.13	0.23	0.00	0.00	0.00	0.00	0.00	1.44E+01	6.32E-52	Up	1.41E+01	8.91E-41	Up	-7.87E+00	3.18E-01		-7.87E+00	4.06E-01		null	null		null	null		Unigene10114_All//gi|159483157|ref|XP_001699629.1|//4.00E-25//acidic ribosomal protein P1 [Chlamydomonas reinhardtii] >gi|266947|sp|P29763.1|RLA1_CHLRE RecName: Full=60S acidic ribosomal protein P1 >gi|18211|emb|CAA47042.1| ribosomal protein P1 [Chlamydomonas reinhardtii] >gi|158272734|gb|EDO98531.1| acidic ribosomal protein P1 [Chlamydomonas reinhardtii]	Unigene10114_All//2.00E-14//AT5G24510| 60s acidic ribosomal protein P1, putative			ko03010|Ribosome	GO:0030529	nr +
Unigene10115_All	553	3.12	1.10	0.48	0.45	0.62	0.33	0.31	0.08	0.22	-1.51E+00	3.95E-03		-2.70E+00	2.40E-05	Down	4.67E-01	7.80E-01		-4.63E-01	7.45E-01		-2.02E+00	3.69E-01		-5.01E-01	7.47E-01								
Unigene101179_All	205	0.00	0.00	0.00	0.00	0.00	0.00	12.58	0.20	0.39	null	null		null	null		null	null		null	null		-5.95E+00	9.35E-17	Down	-5.02E+00	2.78E-16	Down	Unigene101179_All//gi|18558997|gb|AAL73112.1|//1.00E-10//bacterial-induced peroxidase [Gossypium hirsutum]	Unigene101179_All//8.00E-09//AT5G39580| peroxidase, putative	Unigene101179_All//4.00E-46//TC171015		ko00940|Phenylpropanoid biosynthesis//ko00360|Phenylalanine metabolism	GO:0003824//GO:0005506//GO:0006950//GO:0016209//GO:0042743	nr -
Unigene10118_All	285	0.00	0.00	0.00	0.00	0.00	0.00	4.01	4.40	0.00	null	null		null	null		null	null		null	null		1.35E-01	8.03E-01		-1.20E+01	1.65E-08	Down	Unigene10118_All//gi|55168340|gb|AAV44205.1|//3.00E-10//unknow protein [Oryza sativa Japonica Group]						nr +
Unigene10119_All	409	0.00	0.00	0.00	0.00	0.00	0.00	1.86	4.29	0.00	null	null		null	null		null	null		null	null		1.21E+00	7.16E-03		-1.09E+01	8.89E-06	Down	Unigene10119_All//gi|297845688|ref|XP_002890725.1|//4.00E-28//60S ribosomal protein L17 [Arabidopsis lyrata subsp. lyrata] >gi|297336567|gb|EFH66984.1| 60S ribosomal protein L17 [Arabidopsis lyrata subsp. lyrata]	Unigene10119_All//3.00E-28//AT1G27400| 60S ribosomal protein L17 (RPL17A)			ko03010|Ribosome	GO:0005198//GO:0009536//GO:0010467//GO:0015934//GO:0016020//GO:0031981	nr -
Unigene1012_All	811	1.68	3.31	1.90	5.96	6.28	6.18	8.81	17.99	15.77	9.81E-01	1.13E-02		1.83E-01	7.19E-01		7.69E-02	8.39E-01		5.31E-02	8.71E-01		1.03E+00	3.51E-14	Up	8.40E-01	2.07E-09		Unigene1012_All//gi|15230596|ref|NP_187250.1|//1.00E-06//leucine-rich repeat family protein [Arabidopsis thaliana] >gi|30102730|gb|AAP21283.1| At3g05990 [Arabidopsis thaliana]		Unigene1012_All//0.00E+00//gi|109888009|gb|DW516978.1|DW516978			GO:0005488//GO:0023060	nr +
Unigene10123_All	430	0.00	6.36	5.20	0.00	0.00	0.00	0.00	0.00	0.19	1.26E+01	1.44E-15	Up	1.23E+01	2.51E-12	Up	null	null		null	null		null	null		7.53E+00	4.07E-01		Unigene10123_All//gi|192910698|gb|ACF06457.1|//5.00E-42//60S ribosomal protein L35 [Elaeis guineensis]	Unigene10123_All//4.00E-31//AT2G39390| 60S ribosomal protein L35 (RPL35B)			ko03010|Ribosome	GO:0005198//GO:0010467//GO:0030529	nr -
Unigene10124_All	399	3.15	0.76	0.00	1.12	1.29	0.34	0.00	0.21	0.00	-2.05E+00	2.33E-03		-1.16E+01	7.11E-07	Down	2.04E-01	8.59E-01		-1.73E+00	1.61E-01		7.71E+00	4.04E-01		null	null								ESTscan -
Unigene101257_All	206	0.76	3.20	0.00	0.48	1.50	4.38	0.82	0.61	1.35	2.07E+00	3.93E-02		-9.57E+00	2.47E-01		1.64E+00	3.22E-01		3.18E+00	9.74E-04	Up	-4.32E-01	7.94E-01		7.22E-01	5.97E-01								
Unigene10126_All	379	0.00	4.41	6.04	0.00	0.18	0.00	0.00	0.00	0.00	1.21E+01	1.54E-09	Up	1.26E+01	1.27E-12	Up	7.51E+00	6.17E-01		null	null		null	null		null	null		Unigene10126_All//sp|Q95339|ATPK_PIG//6.00E-11//ATP synthase subunit f, mitochondrial OS=Sus scrofa GN=ATP5J2 PE=3 SV=4						swissprot +
Unigene10127_All	398	0.66	0.76	1.07	0.00	1.90	0.45	0.85	0.42	0.50	2.17E-01	8.65E-01		7.04E-01	5.54E-01		1.09E+01	1.17E-04	Up	8.83E+00	1.96E-01		-1.02E+00	4.00E-01		-7.64E-01	5.02E-01		Unigene10127_All//gi|186511604|ref|NP_192625.4|//9.00E-37//kinase [Arabidopsis thaliana] >gi|222423559|dbj|BAH19749.1| AT4G08850 [Arabidopsis thaliana]	Unigene10127_All//5.00E-38//AT4G08850| kinase	Unigene10127_All//7.00E-117//DR462992		ko03410|Base excision repair	GO:0004672//GO:0006464//GO:0016628//GO:0032559	nr -
Unigene101271_All	330	0.63	0.15	1.61	1.51	0.83	0.68	6.41	1.77	2.90	-2.05E+00	3.34E-01		1.35E+00	2.25E-01		-8.55E-01	4.07E-01		-1.14E+00	3.07E-01		-1.85E+00	1.76E-05	Down	-1.14E+00	3.04E-03		Unigene101271_All//gi|71535005|gb|AAZ32900.1|//2.00E-18//zinc finger Glo3-like protein [Medicago sativa]	Unigene101271_All//3.00E-19//AT4G17890| AGD8 (ARF-GAP DOMAIN 8); ARF GTPase activator/ DNA binding / zinc ion binding	Unigene101271_All//4.00E-81//TC132880		ko04144|Endocytosis	GO:0005083//GO:0032012//GO:0043231//GO:0046914	nr +
Unigene10129_All	356	0.00	1.85	2.69	1.12	0.10	0.00	0.59	0.12	0.00	1.09E+01	6.26E-04	Up	1.14E+01	2.46E-05	Up	-3.53E+00	2.10E-02		-1.01E+01	1.11E-02		-2.34E+00	2.38E-01		-9.21E+00	5.84E-02		Unigene10129_All//gi|223452526|gb|ACM89590.1|//6.00E-20//disease resistance protein [Glycine max]	Unigene10129_All//7.00E-10//AT1G58190| AtRLP9 (Receptor Like Protein 9); protein binding				GO:0005488//GO:0016301	nr +
Unigene10131_All	404	0.00	15.43	4.22	0.00	0.00	0.00	0.00	0.00	0.00	1.39E+01	1.38E-35	Up	1.20E+01	2.20E-09	Up	null	null		null	null		null	null		null	null		Unigene10131_All//gi|219125548|ref|XP_002183039.1|//1.00E-20//thioredoxin h [Phaeodactylum tricornutum CCAP 1055/1] >gi|217405314|gb|EEC45257.1| thioredoxin h [Phaeodactylum tricornutum CCAP 1055/1]	Unigene10131_All//2.00E-19//AT1G19730| ATTRX4; oxidoreductase, acting on sulfur group of donors, disulfide as acceptor				GO:0005488//GO:0005737//GO:0008152//GO:0009987	nr -
Unigene101317_All	718	0.00	0.00	0.00	0.14	0.00	0.19	1.12	0.06	0.11	null	null		null	null		-7.12E+00	3.18E-01		4.43E-01	8.63E-01		-4.26E+00	8.48E-05	Down	-3.33E+00	2.47E-04	Down	Unigene101317_All//gi|15241528|ref|NP_196427.1|//1.00E-88//L-asparaginase / L-asparagine amidohydrolase [Arabidopsis thaliana] >gi|25108880|sp|P50287.2|ASPG1_ARATH RecName: Full=Isoaspartyl peptidase/L-asparaginase 1; AltName: Full=L-asparagine amidohydrolase 1; Contains: RecName: Full=Isoaspartyl peptidase/L-asparaginase 1 subunit alpha; Contains: RecName: Full=Isoaspartyl peptidase/L-asparaginase 1 subunit beta; Flags: Precursor >gi|8346547|emb|CAB93711.1| asparaginase [Arabidopsis thaliana] >gi|14517440|gb|AAK62610.1| AT5g08100/T22D6_40 [Arabidopsis thaliana] >gi|20147331|gb|AAM10379.1| AT5g08100/T22D6_40 [Arabidopsis thaliana]	Unigene101317_All//8.00E-90//AT5G08100| L-asparaginase / L-asparagine amidohydrolase	Unigene101317_All//0.00E+00//gi|78347096|gb|DT567370.1|DT567370			GO:0008242//GO:0009057//GO:0016811	nr -
Unigene101318_All	212	0.25	0.00	0.00	0.24	0.81	4.26	0.00	0.20	0.00	-7.95E+00	6.23E-01		-7.95E+00	6.36E-01		1.79E+00	5.22E-01		4.18E+00	1.95E-04	Up	7.62E+00	6.41E-01		null	null								
Unigene101319_All	232	1.13	0.00	0.00	0.00	0.00	0.00	4.92	0.36	1.03	-1.01E+01	6.73E-02		-1.01E+01	8.61E-02		null	null		null	null		-3.77E+00	3.90E-06	Down	-2.26E+00	3.40E-04	Down	Unigene101319_All//gi|16416376|dbj|BAB21610.2|//6.00E-21//mangrin [Bruguiera sexangula]	Unigene101319_All//2.00E-15//AT3G25760| AOC1 (ALLENE OXIDE CYCLASE 1); allene-oxide cyclase	Unigene101319_All//7.00E-82//DW500651		ko00592|alpha-Linolenic acid metabolism	GO:0009536//GO:0009694//GO:0009975	nr +
Unigene10132_All	820	0.00	7.54	2.73	0.00	0.00	0.00	0.00	0.00	0.00	1.29E+01	2.70E-35	Up	1.14E+01	2.51E-12	Up	null	null		null	null		null	null		null	null		Unigene10132_All//gi|115459616|ref|NP_001053408.1|//1.00E-103//Os04g0533000 [Oryza sativa Japonica Group] >gi|75327302|sp|Q7XMK8.1|RH6_ORYSJ RecName: Full=DEAD-box ATP-dependent RNA helicase 6 >gi|32489827|emb|CAE04571.1| OSJNBb0039L24.10 [Oryza sativa (japonica cultivar-group)] >gi|113564979|dbj|BAF15322.1| Os04g0533000 [Oryza sativa Japonica Group]	Unigene10132_All//1.00E-103//AT4G00660| DEAD/DEAH box helicase, putative			ko03018|RNA degradation		nr +
Unigene10135_All	433	2.18	1.64	1.48	5.64	2.94	0.83	0.98	0.39	0.00	-4.09E-01	5.67E-01		-5.60E-01	4.39E-01		-9.38E-01	1.46E-02		-2.76E+00	2.09E-08	Down	-1.34E+00	2.25E-01		-9.93E+00	2.15E-03								

Unigene101358_All	379	0.00	0.67	0.00	0.13	0.00	1.91	45.96	79.84	261.32	9.38E+00	7.89E-02		null	null		-7.04E+00	5.67E-01		3.86E+00	1.75E-03		7.97E-01	7.87E-19		2.51E+00	0.00E+00	Up	Unigene101358_All//gi|224092400|ref|XP_002309592.1|//4.00E-25//2-oxoglutarate-dependent dioxygenase [Populus trichocarpa] >gi|222855568|gb|EEE93115.1| 2-oxoglutarate-dependent dioxygenase [Populus trichocarpa]	Unigene101358_All//6.00E-18//AT1G52800| oxidoreductase, 2OG-Fe(II) oxygenase family protein	Unigene101358_All//3.00E-162//gi|31407058|gb|CD486093.1|CD486093			GO:0016491	nr -
Unigene101372_All	209	0.25	0.00	5.61	2.86	0.82	3.03	0.00	0.00	0.38	-7.97E+00	6.23E-01		4.49E+00	1.90E-05	Up	-1.80E+00	5.87E-02		8.10E-02	9.46E-01		null	null		8.57E+00	4.07E-01								
Unigene101390_All	266	0.00	0.00	0.00	0.37	0.26	6.45	0.00	0.79	0.00	null	null		null	null		-5.33E-01	8.20E-01		4.11E+00	5.03E-08	Up	9.62E+00	7.87E-02		null	null		Unigene101390_All//gi|224143857|ref|XP_002336087.1|//4.00E-22//white-brown-complex ABC transporter family [Populus trichocarpa] >gi|222871834|gb|EEF08965.1| white-brown-complex ABC transporter family [Populus trichocarpa]	Unigene101390_All//5.00E-11//AT3G21090| ABC transporter family protein	Unigene101390_All//2.00E-70//CO132421			GO:0000166//GO:0017111	nr +
Unigene1014_All	456	0.11	0.22	0.93	1.42	1.96	1.09	1.95	1.56	4.81	9.54E-01	7.28E-01		3.03E+00	5.74E-02		4.67E-01	5.72E-01		-3.82E-01	6.78E-01		-3.21E-01	6.33E-01		1.30E+00	7.66E-04	Up	Unigene1014_All//gi|224085664|ref|XP_002307656.1|//1.00E-23//chromatin remodeling complex subunit [Populus trichocarpa] >gi|222857105|gb|EEE94652.1| chromatin remodeling complex subunit [Populus trichocarpa]	Unigene1014_All//1.00E-15//AT2G18760| CHR8 (chromatin remodeling 8); ATP binding / DNA binding / helicase/ nucleic acid binding			ko03420|Nucleotide excision repair	GO:0003676//GO:0003824//GO:0006259//GO:0010212	nr -
Unigene101404_All	205	0.00	20.27	0.26	0.49	0.84	1.54	0.00	0.00	0.00	1.43E+01	1.16E-23	Up	8.02E+00	6.43E-01		7.89E-01	7.72E-01		1.67E+00	3.09E-01		null	null		null	null		Unigene101404_All//gi|297812271|ref|XP_002874019.1|//3.00E-13//glycosyl hydrolase family 17 protein [Arabidopsis lyrata subsp. lyrata] >gi|297319856|gb|EFH50278.1| glycosyl hydrolase family 17 protein [Arabidopsis lyrata subsp. lyrata]	Unigene101404_All//2.00E-14//AT5G20870| glycosyl hydrolase family 17 protein	Unigene101404_All//2.00E-11//gi|78329547|gb|DT549821.1|DT549821				nr +
Unigene10141_All	663	0.00	10.93	5.78	0.00	0.00	0.00	0.00	0.00	0.00	1.34E+01	2.04E-41	Up	1.25E+01	3.02E-21	Up	null	null		null	null		null	null		null	null								ESTscan -
Unigene10142_All	516	0.00	0.00	0.00	0.00	0.13	0.09	8.11	4.37	0.77	null	null		null	null		7.06E+00	6.17E-01		6.45E+00	7.05E-01		-8.91E-01	7.83E-04		-3.39E+00	4.51E-20	Down	Unigene10142_All//gi|254803073|gb|ACT82815.1|//8.00E-13//At1g29270 [Arabidopsis thaliana]	Unigene10142_All//4.00E-11//AT1G29270| unknown protein	Unigene10142_All//3.00E-89//gi|109881197|gb|DW510167.1|DW510167				nr +
Unigene101435_All	574	0.18	0.00	0.00	0.61	0.12	0.16	1.18	0.29	0.00	-7.51E+00	3.78E-01		-7.51E+00	4.00E-01		-2.34E+00	1.01E-01		-1.95E+00	1.96E-01		-2.02E+00	1.98E-02		-1.02E+01	3.57E-05	Down	Unigene101435_All//gi|260446978|emb|CBG76260.1|//1.00E-30//OO_Ba0005L10-OO_Ba0081K17.11 [Oryza officinalis]	Unigene101435_All//5.00E-29//AT3G19850| phototropic-responsive NPH3 family protein				GO:0005488//GO:0009314//GO:0044464//GO:0060089	nr +
Unigene10144_All	449	0.00	20.65	7.47	0.00	0.00	0.00	0.00	0.00	0.00	1.43E+01	4.29E-53	Up	1.29E+01	1.47E-18	Up	null	null		null	null		null	null		null	null								ESTscan -
Unigene10145_All	410	0.00	0.00	0.00	10.57	4.79	3.41	0.00	0.00	0.00	null	null		null	null		-1.14E+00	2.80E-05	Down	-1.63E+00	9.03E-08	Down	null	null		null	null		Unigene10145_All//gi|104295001|gb|ABF72016.1|//9.00E-36//GDSL-motif lipase/hydrolase family protein [Musa acuminata]	Unigene10145_All//3.00E-26//AT5G45670| GDSL-motif lipase/hydrolase family protein	Unigene10145_All//6.00E-170//ES847780			GO:0016787//GO:0044238	nr -
Unigene101501_All	214	0.00	0.00	0.00	0.00	0.00	0.00	0.99	6.84	1.30	null	null		null	null		null	null		null	null		2.79E+00	4.85E-06	Up	4.00E-01	7.43E-01		Unigene101501_All//gi|225449847|ref|XP_002265001.1|//4.00E-07//PREDICTED: hypothetical protein [Vitis vinifera]	Unigene101501_All//5.00E-07//AT1G62600| flavin-containing monooxygenase family protein / FMO family protein				GO:0000166//GO:0004497	nr +
Unigene10151_All	283	0.00	1.79	0.00	0.00	0.73	0.32	0.45	0.74	3.24	1.08E+01	4.05E-03		null	null		9.51E+00	1.58E-01		8.32E+00	4.83E-01		7.20E-01	6.33E-01		2.85E+00	3.18E-04	Up			Unigene10151_All//2.00E-128//CO078325				
Unigene10152_All	572	0.18	0.18	0.09	5.66	3.25	1.66	0.96	0.44	0.14	-4.57E-02	9.91E-01		-9.74E-01	7.23E-01		-8.00E-01	1.40E-02		-1.77E+00	1.26E-06	Down	-1.13E+00	2.18E-01		-2.79E+00	7.97E-03		Unigene10152_All//gi|297821166|ref|XP_002878466.1|//9.00E-87//DNAJ heat shock family protein [Arabidopsis lyrata subsp. lyrata] >gi|297324304|gb|EFH54725.1| DNAJ heat shock family protein [Arabidopsis lyrata subsp. lyrata]	Unigene10152_All//5.00E-88//AT3G62600| ATERDJ3B; heat shock protein binding / unfolded protein binding	Unigene10152_All//0.00E+00//DR456694		ko04141|Protein processing in endoplasmic reticulum	GO:0005515//GO:0016020//GO:0031410//GO:0044267//GO:0044432	nr -
Unigene101529_All	221	3.08	6.88	10.84	9.69	9.35	2.45	1.34	0.38	2.34	1.16E+00	3.43E-02		1.82E+00	9.66E-05	Up	-5.21E-02	9.02E-01		-1.98E+00	3.06E-05	Down	-1.82E+00	2.11E-01		8.07E-01	3.66E-01				Unigene101529_All//2.00E-69//DV849351				
Unigene10154_All	557	0.19	2.46	0.10	0.72	1.98	0.49	1.90	4.43	2.07	3.71E+00	7.18E-06	Up	-9.75E-01	7.23E-01		1.47E+00	3.09E-02		-5.57E-01	6.38E-01		1.22E+00	9.01E-04	Up	1.28E-01	8.11E-01								
Unigene101542_All	211	0.00	0.00	0.00	0.00	0.00	0.64	1.20	0.59	6.23	null	null		null	null		null	null		9.33E+00	3.13E-01		-1.02E+00	4.96E-01		2.37E+00	7.20E-05	Up	Unigene101542_All//gi|110738232|dbj|BAF01045.1|//1.00E-07//serine/threonine kinase - like protein [Arabidopsis thaliana]	Unigene101542_All//6.00E-09//AT4G21390| B120; ATP binding / protein kinase/ protein serine/threonine kinase/ sugar binding				GO:0004672//GO:0005488//GO:0009987	nr +
Unigene101555_All	276	7.58	5.69	5.40	5.78	5.49	5.07	3.52	0.61	1.44	-4.14E-01	3.72E-01		-4.89E-01	3.03E-01		-7.32E-02	8.77E-01		-1.87E-01	7.18E-01		-2.54E+00	6.42E-04	Down	-1.29E+00	3.62E-02		Unigene101555_All//gi|226492900|ref|NP_001148774.1|//1.00E-08//mitochondrial ribosomal protein L43 [Zea mays] >gi|195622062|gb|ACG32861.1| mitochondrial ribosomal protein L43 [Zea mays]	Unigene101555_All//7.00E-10//AT3G59650| mitochondrial ribosomal protein L51/S25/CI-B8 family protein	Unigene101555_All//5.00E-83//TC165625			GO:0030529	nr -
Unigene10156_All	300	0.00	1.86	3.20	0.17	0.34	0.15	0.00	0.00	0.00	1.09E+01	2.19E-03		1.16E+01	2.46E-05	Up	1.05E+00	8.10E-01		-1.41E-01	9.58E-01		null	null		null	null		Unigene10156_All//gi|116056517|emb|CAL52806.1|//3.00E-10//Sel1-like repeat (ISS) [Ostreococcus tauri]	Unigene10156_All//2.00E-09//AT1G70590| F-box family protein			ko04141|Protein processing in endoplasmic reticulum		nr +
Unigene10157_All	559	0.37	1.54	0.00	0.89	0.92	0.65	1.06	2.09	3.71	2.04E+00	1.59E-02		-8.55E+00	1.47E-01		5.23E-02	9.80E-01		-4.63E-01	6.59E-01		9.83E-01	8.30E-02		1.81E+00	2.23E-05	Up			Unigene10157_All//8.00E-19//TC146433				
Unigene1016_All	3371	3.21	5.05	3.60	3.50	7.80	5.55	22.75	39.28	35.54	6.53E-01	1.29E-06		1.65E-01	3.90E-01		1.16E+00	1.54E-28	Up	6.63E-01	1.05E-07		7.88E-01	4.09E-78		6.43E-01	4.77E-51		Unigene1016_All//gi|15239540|ref|NP_197965.1|//0.00E+00//leucine-rich repeat family protein / protein kinase family protein [Arabidopsis thaliana] >gi|5107831|gb|AAD40144.1|AF149413_25 contains similarity to protein kinase domains (Pfam F00069, Score=162.6, E=6.8e-45, N=1) and leucien rich repeats (Pfam PF00560, Score=210.7, E=2.2e-59, N=10) [Arabidopsis thaliana] >gi|224589685|gb|ACN59374.1| leucine-rich repeat receptor-like protein kinase [Arabidopsis thaliana]	Unigene1016_All//0.00E+00//AT5G25930| leucine-rich repeat family protein / protein kinase family protein	Unigene1016_All//0.00E+00//TC138249			GO:0004674//GO:0006464//GO:0016491//GO:0031224//GO:0032559	nr +
Unigene101614_All	786	0.00	0.00	0.00	0.06	0.00	4.54	0.00	0.00	0.00	null	null		null	null		-5.99E+00	5.67E-01		6.16E+00	6.65E-20	Up	null	null		null	null		Unigene101614_All//gi|300268585|gb|EFJ52765.1|//2.00E-11//pathogenesis-related protein 1-like protein [Volvox carteri f. nagariensis]				ko00230|Purine metabolism//ko00240|Pyrimidine metabolism//ko03020|RNA polymerase		nr +
Unigene10162_All	305	0.00	0.00	0.17	2.94	2.15	0.15	0.83	0.00	0.26	null	null		7.45E+00	6.43E-01		-4.55E-01	5.58E-01		-4.31E+00	1.14E-04	Down	-9.70E+00	4.07E-02		-1.67E+00	2.57E-01				Unigene10162_All//8.00E-144//gi|78351568|gb|DT571842.1|DT571842				
Unigene10164_All	248	2.53	8.58	1.07	0.60	0.56	0.36	0.00	0.34	0.32	1.76E+00	2.38E-04	Up	-1.24E+00	2.13E-01		-1.18E-01	9.12E-01		-7.27E-01	7.17E-01		8.40E+00	4.04E-01		8.33E+00	4.07E-01								
Unigene101652_All	409	0.00	0.00	0.00	0.12	0.00	0.22	1.65	0.10	1.36	null	null		null	null		-6.93E+00	5.66E-01		8.58E-01	7.98E-01		-4.02E+00	5.36E-04	Down	-2.78E-01	6.89E-01		Unigene101652_All//gi|224053787|ref|XP_002297979.1|//2.00E-32//AP2/ERF domain-containing transcription factor [Populus trichocarpa] >gi|222845237|gb|EEE82784.1| AP2/ERF domain-containing transcription factor [Populus trichocarpa]	Unigene101652_All//1.00E-15//AT1G64380| AP2 domain-containing transcription factor, putative	Unigene101652_All//2.00E-46//TC154225	AP2-EREBP		GO:0001071//GO:0003676//GO:0006351//GO:0009743//GO:0010468//GO:0043231	nr -
Unigene10166_All	1138	0.00	0.00	0.00	0.00	0.00	0.04	10.18	0.29	2.35	null	null		null	null		null	null		5.31E+00	7.05E-01		-5.11E+00	8.05E-70	Down	-2.12E+00	1.00E-32	Down	Unigene10166_All//gi|297793821|ref|XP_002864795.1|//1.00E-125//aldo/keto reductase family protein [Arabidopsis lyrata subsp. lyrata] >gi|297310630|gb|EFH41054.1| aldo/keto reductase family protein [Arabidopsis lyrata subsp. lyrata]	Unigene10166_All//3.00E-116//AT5G62420| aldo/keto reductase family protein			ko00010|Glycolysis / Gluconeogenesis//ko00051|Fructose and mannose metabolism//ko00561|Glycerolipid metabolism//ko00591|Linoleic acid metabolism//ko00650|Butanoate metabolism	GO:0006970//GO:0016616	nr +
Unigene101666_All	258	1.42	1.18	0.62	1.74	1.74	1.40	0.98	4.70	0.46	-2.69E-01	8.01E-01		-1.20E+00	3.96E-01		-2.20E-03	9.69E-01		-3.11E-01	7.57E-01		2.26E+00	3.35E-04	Up	-1.09E+00	4.29E-01				Unigene101666_All//2.00E-64//CK640533				
Unigene10168_All	454	0.00	0.00	0.00	0.00	0.00	0.00	4.28	2.30	0.00	null	null		null	null		null	null		null	null		-8.96E-01	3.11E-02		-1.21E+01	2.41E-14	Down	Unigene10168_All//gi|240256452|ref|NP_200624.5|//3.00E-08//actin binding [Arabidopsis thaliana]					GO:0003824//GO:0008152	nr +
Unigene1017_All	941	5.78	4.95	3.73	6.83	4.14	2.30	6.52	0.84	1.14	-2.23E-01	3.97E-01		-6.31E-01	1.83E-02		-7.24E-01	8.18E-04		-1.57E+00	1.20E-10	Down	-2.95E+00	1.12E-24	Down	-2.51E+00	1.41E-21	Down	Unigene1017_All//gi|124359162|gb|ABN05685.1|//4.00E-14//RNA-directed DNA polymerase (Reverse transcriptase) [Medicago truncatula]	Unigene1017_All//3.00E-10//AT4G29090| reverse transcriptase, putative / RNA-dependent DNA polymerase, putative	Unigene1017_All//7.00E-113//TC135736				nr -
Unigene10170_All	876	0.00	0.00	0.00	0.06	0.08	0.00	0.48	2.58	2.77	null	null		null	null		4.66E-01	9.62E-01		-5.83E+00	6.47E-01		2.42E+00	9.14E-08	Up	2.52E+00	6.53E-09	Up			Unigene10170_All//2.00E-23//DW234869				
Unigene101703_All	208	0.00	0.24	1.02	4.31	0.17	1.95	1.02	0.20	0.77	7.93E+00	6.24E-01		1.00E+01	1.42E-01		-4.70E+00	1.27E-05	Down	-1.14E+00	1.31E-01		-2.34E+00	2.37E-01		-4.08E-01	7.56E-01								
Unigene101708_All	211	9.67	0.72	2.27	1.89	1.80	0.64	0.00	0.00	0.00	-3.75E+00	1.18E-08	Down	-2.09E+00	8.44E-05	Down	-7.32E-02	9.26E-01		-1.56E+00	2.31E-01		null	null		null	null								
Unigene10172_All	201	11.97	15.63	14.84	111.03	105.02	102.46	45.44	31.81	20.81	3.84E-01	2.87E-01		3.09E-01	4.13E-01		-8.03E-02	5.79E-01		-1.16E-01	4.04E-01		-5.14E-01	2.51E-03		-1.13E+00	7.26E-11	Down	Unigene10172_All//gi|30725324|gb|AAP37684.1|//3.00E-17//At3g01680 [Arabidopsis thaliana]	Unigene10172_All//1.00E-18//AT3G01680| unknown protein	Unigene10172_All//2.00E-88//ES843933				nr +
Unigene101722_All	247	0.21	0.41	0.00	1.41	0.00	1.65	1.88	13.37	5.00	9.54E-01	7.28E-01		-7.73E+00	6.36E-01		-1.05E+01	9.46E-03		2.21E-01	8.63E-01		2.83E+00	5.32E-13	Up	1.41E+00	9.74E-03		Unigene101722_All//gi|55818553|gb|AAV66071.1|//5.00E-08//acidic glucanase [Medicago sativa]		Unigene101722_All//2.00E-42//TC142668			GO:0016798	nr +
Unigene10174_All	397	0.00	3.57	2.28	0.00	0.09	0.00	0.00	0.00	0.00	1.18E+01	4.06E-08	Up	1.12E+01	4.72E-05	Up	6.44E+00	8.42E-01		null	null		null	null		null	null				Unigene10174_All//8.00E-18//FL577438				
Unigene10175_All	473	0.00	0.00	0.00	0.42	0.00	0.00	6.08	4.68	0.51	null	null		null	null		-8.72E+00	8.49E-02		-8.72E+00	1.34E-01		-3.76E-01	2.81E-01		-3.59E+00	1.22E-14	Down	Unigene10175_All//gi|297725019|ref|NP_001174873.1|//6.00E-19//Os06g0586150 [Oryza sativa Japonica Group] >gi|255677180|dbj|BAH93601.1| Os06g0586150 [Oryza sativa Japonica Group]	Unigene10175_All//3.00E-15//AT3G47580| leucine-rich repeat transmembrane protein kinase, putative			ko04626|Plant-pathogen interaction	GO:0000166//GO:0004672//GO:0008152	nr -
Unigene10177_All	277	0.00	0.00	0.00	0.00	0.00	0.00	2.75	0.00	1.15	null	null		null	null		null	null		null	null		-1.14E+01	1.68E-05	Down	-1.26E+00	7.70E-02		Unigene10177_All//gi|42568648|ref|NP_200715.2|//5.00E-23//glycosyl transferase family 1 protein [Arabidopsis thaliana]	Unigene10177_All//2.00E-24//AT5G59070| glycosyl transferase family 1 protein				GO:0008152//GO:0016740	nr -
Unigene10179_All	239	1.31	1.27	0.89	2.50	1.73	0.00	0.00	0.00	1.17	-4.63E-02	9.85E-01		-5.60E-01	6.64E-01		-5.33E-01	5.62E-01		-1.13E+01	7.71E-04	Down	null	null		1.02E+01	2.44E-02		Unigene10179_All//gi|77999307|gb|ABB17000.1|//2.00E-13//ribosomal protein L23 family protein [Solanum tuberosum]	Unigene10179_All//5.00E-13//AT4G39880| ribosomal protein L23 family protein	Unigene10179_All//2.00E-26//TC146980			GO:0005198//GO:0005402//GO:0005488//GO:0006810//GO:0010467//GO:0012505//GO:0022613//GO:0030529//GO:0031224	nr -
Unigene1018_All	1337	50.79	20.31	31.34	68.11	35.75	16.08	10.44	4.47	2.21	-1.32E+00	9.37E-77	Down	-6.97E-01	6.92E-26		-9.30E-01	6.32E-72		-2.08E+00	1.17E-213	Down	-1.22E+00	1.04E-17	Down	-2.24E+00	3.98E-42	Down	Unigene1018_All//gi|297725701|ref|NP_001175214.1|//1.00E-11//Os07g0501200 [Oryza sativa Japonica Group] >gi|255677791|dbj|BAH93942.1| Os07g0501200 [Oryza sativa Japonica Group]	Unigene1018_All//3.00E-07//AT4G29090| reverse transcriptase, putative / RNA-dependent DNA polymerase, putative	Unigene1018_All//3.00E-91//TC135736				nr +
Unigene10181_All	827	0.00	9.44	4.25	0.00	0.00	0.00	0.00	0.00	0.00	1.32E+01	1.26E-44	Up	1.21E+01	1.88E-19	Up	null	null		null	null		null	null		null	null		Unigene10181_All//gi|226502788|ref|NP_001148944.1|//1.00E-48//nascent polypeptide-associated complex alpha subunit-like protein [Zea mays] >gi|195623512|gb|ACG33586.1| nascent polypeptide-associated complex alpha subunit-like protein [Zea mays]	Unigene10181_All//1.00E-32//AT3G49470| NACA2 (NASCENT POLYPEPTIDE-ASSOCIATED COMPLEX SUBUNIT ALPHA-LIKE PROTEIN 2)				GO:0006970	nr -
Unigene101816_All	208	0.25	0.00	1.28	1.20	0.33	1.30	0.00	2.61	0.38	-7.97E+00	6.23E-01		2.35E+00	2.40E-01		-1.85E+00	2.76E-01		1.22E-01	9.45E-01		1.14E+01	5.37E-04	Up	8.58E+00	4.07E-01								
Unigene101818_All	234	7.60	3.68	13.88	2.13	1.77	4.25	2.53	11.25	3.75	-1.05E+00	3.39E-02		8.69E-01	1.56E-02		-2.70E-01	8.02E-01		9.96E-01	1.72E-01		2.15E+00	6.92E-08	Up	5.66E-01	3.85E-01				Unigene101818_All//6.00E-42//TC156918				

Unigene10183_All	603	0.00	0.00	0.00	0.00	0.00	0.00	6.52	1.66	2.11	null	null		null	null		null	null		null	null		-1.97E+00	2.60E-10	Down	-1.62E+00	2.16E-08	Down	Unigene10183_All//gi|21322711|emb|CAD22154.1|//9.00E-53//pherophorin-dz1 protein [Volvox carteri f. nagariensis]				ko03040|Spliceosome	GO:0044464	nr +
Unigene10185_All	666	0.00	0.00	0.00	0.00	0.00	0.27	2.73	6.02	2.51	null	null		null	null		null	null		8.08E+00	1.96E-01		1.14E+00	3.76E-05	Up	-1.20E-01	7.71E-01		Unigene10185_All//gi|224096718|ref|XP_002310710.1|//6.00E-88//2-oxoglutarate-dependent dioxygenase [Populus trichocarpa] >gi|222853613|gb|EEE91160.1| 2-oxoglutarate-dependent dioxygenase [Populus trichocarpa]	Unigene10185_All//9.00E-79//AT3G50210| 2-oxoacid-dependent oxidase, putative			ko00270|Cysteine and methionine metabolism	GO:0008152//GO:0016706//GO:0031669//GO:0032502//GO:0046914	nr -
Unigene10187_All	437	1.08	0.23	1.34	6.61	5.36	2.69	0.19	0.77	1.28	-2.22E+00	7.77E-02		3.15E-01	7.19E-01		-3.03E-01	4.44E-01		-1.30E+00	4.76E-04	Down	1.98E+00	1.45E-01		2.72E+00	9.39E-03		Unigene10187_All//gi|24461861|gb|AAN62348.1|AF506028_15//5.00E-31//NBS-LRR type disease resistance protein [Citrus trifoliata]	Unigene10187_All//8.00E-22//AT5G63020| disease resistance protein (CC-NBS-LRR class), putative	Unigene10187_All//6.00E-136//DR461626		ko04626|Plant-pathogen interaction	GO:0005488//GO:0016787	nr +
Unigene10188_All	459	0.00	12.58	9.28	0.00	0.00	0.00	0.18	0.00	0.00	1.36E+01	5.79E-33	Up	1.32E+01	1.22E-23	Up	null	null		null	null		-7.53E+00	4.09E-01		-7.53E+00	3.62E-01		Unigene10188_All//gi|15229631|ref|NP_190560.1|//1.00E-48//60S ribosomal protein L26 (RPL26A) [Arabidopsis thaliana] >gi|297816242|ref|XP_002876004.1| 60S ribosomal protein L26 [Arabidopsis lyrata subsp. lyrata] >gi|27735242|sp|P51414.2|RL261_ARATH RecName: Full=60S ribosomal protein L26-1 >gi|6723436|emb|CAB66929.1| 60S RIBOSOMAL PROTEIN-like [Arabidopsis thaliana] >gi|297321842|gb|EFH52263.1| 60S ribosomal protein L26 [Arabidopsis lyrata subsp. lyrata]	Unigene10188_All//4.00E-41//AT3G49910| 60S ribosomal protein L26 (RPL26A)			ko03010|Ribosome	GO:0005198//GO:0006950//GO:0009536//GO:0010467//GO:0015934//GO:0016020//GO:0031981	nr +
Unigene10191_All	445	0.00	0.00	0.00	0.00	0.00	0.00	1.43	8.27	4.48	null	null		null	null		null	null		null	null		2.54E+00	6.95E-13	Up	1.65E+00	1.08E-04	Up							
Unigene10192_All	672	0.16	15.31	7.69	0.00	0.00	0.00	0.00	0.00	0.00	6.62E+00	2.97E-55	Up	5.63E+00	1.12E-25	Up	null	null		null	null		null	null		null	null		Unigene10192_All//gi|585238|sp|Q08864.3|H11_VOLCA//6.00E-25//RecName: Full=Histone H1-I >gi|349585|gb|AAA74723.1| histone H1-I [Volvox carteri] >gi|300267026|gb|EFJ51211.1| histone H1 [Volvox carteri f. nagariensis]	Unigene10192_All//2.00E-05//AT3G18035| HON4; DNA binding					nr +
Unigene10193_All	642	2.12	0.55	1.00	10.32	8.69	7.18	7.05	4.56	1.92	-1.94E+00	2.18E-03		-1.09E+00	7.48E-02		-2.48E-01	3.12E-01		-5.24E-01	2.22E-02		-6.29E-01	1.31E-02		-1.87E+00	2.00E-11	Down	Unigene10193_All//gi|79499196|ref|NP_195518.2|//3.00E-21//transmembrane transporter [Arabidopsis thaliana] >gi|75128428|sp|Q6SZ87.1|NAT11_ARATH RecName: Full=Nucleobase-ascorbate transporter 11; Short=AtNAT11 >gi|38350521|gb|AAR18373.1| nucleobase-ascorbate transporter 11 [Arabidopsis thaliana]	Unigene10193_All//6.00E-21//AT4G38050| transmembrane transporter				GO:0005215//GO:0006810//GO:0016020	nr +
Unigene101932_All	249	0.00	6.10	0.00	0.80	1.66	2.54	0.68	1.01	1.44	1.26E+01	1.10E-08	Up	null	null		1.05E+00	4.26E-01		1.67E+00	9.80E-02		5.68E-01	6.75E-01		1.08E+00	3.50E-01				Unigene101932_All//1.00E-58//gi|164243022|gb|ES823910.1|ES823910				
Unigene10196_All	312	1.17	12.83	4.27	5.43	3.31	2.46	2.03	1.88	2.04	3.45E+00	2.82E-15	Up	1.86E+00	4.86E-03		-7.13E-01	1.48E-01		-1.14E+00	2.50E-02		-1.16E-01	8.88E-01		7.30E-03	9.95E-01				Unigene10196_All//2.00E-11//TC153817				
Unigene101960_All	220	0.00	1.38	0.00	0.68	0.16	0.82	0.77	1.14	8.15	1.04E+01	4.45E-02		null	null		-2.12E+00	3.88E-01		2.74E-01	9.06E-01		5.68E-01	6.75E-01		3.41E+00	8.04E-09	Up	Unigene101960_All//gi|297806781|ref|XP_002871274.1|//3.00E-08//predicted protein [Arabidopsis lyrata subsp. lyrata] >gi|297317111|gb|EFH47533.1| predicted protein [Arabidopsis lyrata subsp. lyrata]	Unigene101960_All//1.00E-09//AT5G07490| unknown protein					nr -
Unigene101985_All	271	0.97	1.68	0.59	0.74	1.27	1.50	10.61	8.79	1.47	8.02E-01	4.60E-01		-7.12E-01	6.22E-01		7.89E-01	5.96E-01		1.03E+00	4.29E-01		-2.71E-01	4.47E-01		-2.85E+00	5.76E-12	Down			Unigene101985_All//3.00E-69//TC172050				
Unigene10199_All	717	0.07	12.30	7.35	0.00	0.00	0.00	0.00	0.00	0.00	7.40E+00	1.54E-48	Up	6.65E+00	1.17E-27	Up	null	null		null	null		null	null		null	null		Unigene10199_All//gi|160895838|ref|YP_001561420.1|//2.00E-65//elongation factor Tu [Delftia acidovorans SPH-1] >gi|160361422|gb|ABX33035.1| translation elongation factor Tu [Delftia acidovorans SPH-1]	Unigene10199_All//3.00E-50//AT4G20360| ATRABE1B (ARABIDOPSIS RAB GTPASE HOMOLOG E1B); GTP binding / GTPase/ translation elongation factor	Unigene10199_All//6.00E-11//EX172631		ko04626|Plant-pathogen interaction	GO:0006412//GO:0008135//GO:0009536//GO:0016772//GO:0017111//GO:0032561	nr +
Unigene102_All	1463	3.40	3.05	1.49	3.00	2.94	2.65	2.80	2.31	1.31	-1.57E-01	6.04E-01		-1.19E+00	3.56E-05	Down	-2.63E-02	9.35E-01		-1.75E-01	6.24E-01		-2.77E-01	3.52E-01		-1.10E+00	3.37E-05	Down	Unigene102_All//gi|297798410|ref|XP_002867089.1|//1.00E-150//transducin family protein [Arabidopsis lyrata subsp. lyrata] >gi|297312925|gb|EFH43348.1| transducin family protein [Arabidopsis lyrata subsp. lyrata]	Unigene102_All//4.00E-148//AT4G35140| transducin family protein / WD-40 repeat family protein	Unigene102_All//0.00E+00//gi|84164480|gb|DR453128.1|DR453128				nr +
Unigene1020_All	1402	8.84	20.38	16.75	22.28	42.42	51.90	51.39	96.86	22.42	1.20E+00	3.23E-28	Up	9.21E-01	9.14E-15		9.29E-01	3.79E-45		1.22E+00	1.31E-77	Up	9.14E-01	4.63E-103		-1.20E+00	1.37E-87	Down	Unigene1020_All//gi|79521554|ref|NP_197973.2|//3.00E-96//catalytic/ protein serine/threonine phosphatase [Arabidopsis thaliana] >gi|226739226|sp|Q9XGZ9.2|P2C72_ARATH RecName: Full=Probable protein phosphatase 2C 72; Short=AtPP2C72	Unigene1020_All//2.00E-92//AT5G26010| catalytic/ protein serine/threonine phosphatase	Unigene1020_All//0.00E+00//TC156152			GO:0003824	nr -
Unigene10200_All	523	0.00	26.26	6.52	0.00	0.40	0.00	0.00	0.00	0.00	1.47E+01	7.80E-79	Up	1.27E+01	7.39E-19	Up	8.63E+00	1.58E-01		null	null		null	null		null	null		Unigene10200_All//gi|298711973|emb|CBJ32915.1|//6.00E-25//ribosomal protein L35 [Ectocarpus siliculosus]	Unigene10200_All//7.00E-24//AT1G74270| 60S ribosomal protein L35a (RPL35aC)			ko03010|Ribosome	GO:0043231	nr -
Unigene10201_All	1255	0.25	4.44	2.72	0.40	0.58	0.43	0.44	0.23	0.32	4.15E+00	3.21E-24	Up	3.44E+00	1.30E-12	Up	5.38E-01	5.64E-01		1.22E-01	9.21E-01		-9.10E-01	3.19E-01		-4.65E-01	5.97E-01		Unigene10201_All//gi|71905515|gb|AAZ52735.1|//7.00E-94//expressed protein [Arabidopsis thaliana]	Unigene10201_All//4.00E-90//AT4G12680| unknown protein	Unigene10201_All//0.00E+00//CO131664			GO:0003006//GO:0031410	nr +
Unigene10202_All	740	0.00	0.00	0.00	0.00	0.00	0.00	2.97	0.56	3.98	null	null		null	null		null	null		null	null		-2.40E+00	1.55E-07	Down	4.23E-01	1.93E-01		Unigene10202_All//gi|224077026|ref|XP_002305098.1|//2.00E-68//AP2/ERF domain-containing transcription factor [Populus trichocarpa] >gi|222848062|gb|EEE85609.1| AP2/ERF domain-containing transcription factor [Populus trichocarpa]	Unigene10202_All//1.00E-26//AT4G18450| ethylene-responsive factor, putative	Unigene10202_All//1.00E-08//TC178583	AP2-EREBP		GO:0006350//GO:0010033//GO:0010468//GO:0043231	nr +
Unigene102075_All	420	61.41	49.95	55.28	5.81	8.77	6.77	3.22	1.69	0.76	-2.98E-01	6.26E-03		-1.52E-01	2.23E-01		5.94E-01	6.40E-02		2.21E-01	6.27E-01		-9.29E-01	7.36E-02		-2.09E+00	1.85E-04	Down	Unigene102075_All//gi|47155380|dbj|BAD18925.1|//2.00E-30//ferredoxin [Codonopsis lanceolata]	Unigene102075_All//2.00E-31//AT2G04700| ferredoxin thioredoxin reductase catalytic beta chain family protein	Unigene102075_All//4.00E-171//gi|109867808|gb|DW496784.1|DW496784			GO:0008152//GO:0009536//GO:0016731//GO:0043169//GO:0051536	nr +
Unigene10208_All	501	0.00	11.02	2.23	0.00	0.00	0.00	0.00	0.00	0.00	1.34E+01	1.66E-31	Up	1.11E+01	3.44E-06	Up	null	null		null	null		null	null		null	null		Unigene10208_All//sp|P08285|H103_CHICK//4.00E-06//Histone H1.03 OS=Gallus gallus PE=3 SV=2						swissprot +
Unigene1021_All	3759	9.24	5.65	7.00	19.40	16.65	16.18	34.99	15.68	18.60	-7.11E-01	1.30E-14		-4.01E-01	1.98E-05		-2.20E-01	1.47E-04		-2.62E-01	1.27E-05		-1.16E+00	4.04E-147	Down	-9.12E-01	3.18E-102		Unigene1021_All//gi|15228737|ref|NP_191795.1|//0.00E+00//carbohydrate binding [Arabidopsis thaliana]	Unigene1021_All//0.00E+00//AT3G62360| carbohydrate binding	Unigene1021_All//0.00E+00//TC179301			GO:0005488//GO:0005618//GO:0008238//GO:0016020//GO:0031410//GO:0043231	nr +
Unigene10210_All	778	0.40	0.91	0.14	0.00	0.22	0.00	1.03	11.66	2.15	1.18E+00	1.75E-01		-1.56E+00	3.30E-01		7.79E+00	2.28E-01		null	null		3.50E+00	3.07E-42	Up	1.06E+00	1.67E-02		Unigene10210_All//gi|229609807|gb|ACQ83519.1|//6.00E-20//CBL-interacting protein kinase 03 [Vitis vinifera]	Unigene10210_All//4.00E-15//AT4G30960| SIP3 (SOS3-INTERACTING PROTEIN 3); ATP binding / kinase/ protein kinase/ protein serine/threonine kinase	Unigene10210_All//6.00E-85//TC133167			GO:0004674//GO:0006464//GO:0006972//GO:0009926//GO:0023060//GO:0032501//GO:0032502//GO:0032559//GO:0046872	nr +
Unigene102112_All	480	1.42	0.00	0.33	4.36	1.43	0.56	0.35	0.00	0.50	-1.05E+01	4.10E-04	Down	-2.09E+00	4.38E-02		-1.60E+00	2.04E-04	Down	-2.95E+00	9.27E-08	Down	-8.46E+00	1.35E-01		4.99E-01	7.08E-01		Unigene102112_All//gi|115446245|ref|NP_001046902.1|//1.00E-14//Os02g0502500 [Oryza sativa Japonica Group] >gi|113536433|dbj|BAF08816.1| Os02g0502500 [Oryza sativa Japonica Group]	Unigene102112_All//6.00E-06//AT2G40980| LOCATED IN: plasma membrane; EXPRESSED IN: 22 plant structures; EXPRESSED DURING: 13 growth stages; CONTAINS InterPro DOMAIN/s: Protein kinase-like (InterPro:IPR011009); Has 152 Blast hits to 139 proteins in 56 species: Archae - 0; Bacteria - 74; Metazoa - 0; Fungi - 0; Plants - 60; Viruses - 0; Other Eukaryotes - 18 (source: NCBI BLink).					nr -
Unigene10213_All	796	0.00	1.02	0.13	0.06	0.04	0.11	0.85	3.10	30.03	9.99E+00	9.39E-05	Up	7.06E+00	4.03E-01		-5.32E-01	8.30E-01		8.59E-01	7.98E-01		1.87E+00	2.64E-06	Up	5.14E+00	8.58E-146	Up	Unigene10213_All//gi|224139556|ref|XP_002323167.1|//2.00E-09//ABC transporter family protein [Populus trichocarpa] >gi|222867797|gb|EEF04928.1| ABC transporter family protein [Populus trichocarpa]		Unigene10213_All//0.00E+00//DT463777				nr +
Unigene10215_All	575	0.00	0.00	0.00	0.00	0.00	0.00	2.43	1.89	0.35	null	null		null	null		null	null		null	null		-3.61E-01	5.05E-01		-2.81E+00	4.26E-06	Down	Unigene10215_All//gi|116222231|gb|ABJ80979.1|//8.00E-95//alpha tubulin [Porphyra yezoensis]	Unigene10215_All//1.00E-84//AT5G19780| TUA5; structural constituent of cytoskeleton	Unigene10215_All//5.00E-48//TC142299		ko04145|Phagosome	GO:0007017//GO:0015630//GO:0017111//GO:0032561//GO:0032991//GO:0043623	nr -
Unigene10216_All	636	0.00	0.00	0.00	0.00	0.00	0.00	4.45	4.73	0.06	null	null		null	null		null	null		null	null		8.72E-02	8.00E-01		-6.15E+00	2.65E-19	Down	Unigene10216_All//gi|297844300|ref|XP_002890031.1|//2.00E-50//eukaryotic translation initiation factor 5A-1 [Arabidopsis lyrata subsp. lyrata] >gi|297335873|gb|EFH66290.1| eukaryotic translation initiation factor 5A-1 [Arabidopsis lyrata subsp. lyrata]	Unigene10216_All//1.00E-50//AT1G13950| ELF5A-1 (EUKARYOTIC ELONGATION FACTOR 5A-1); translation initiation factor	Unigene10216_All//8.00E-07//EX164461			GO:0006448//GO:0006449//GO:0008135//GO:0010087//GO:0018205//GO:0043021	nr -
Unigene102186_All	206	0.51	1.48	0.00	2.42	0.00	2.63	0.41	0.41	0.00	1.54E+00	3.24E-01		-8.99E+00	4.00E-01		-1.12E+01	8.99E-04	Down	1.22E-01	9.21E-01		-1.66E-02	1.02E+00		-8.68E+00	3.62E-01				Unigene102186_All//2.00E-10//DW517741				
Unigene102197_All	486	0.11	0.00	0.00	0.31	0.43	2.23	0.52	0.00	2.38	-6.75E+00	6.23E-01		-6.75E+00	6.35E-01		4.67E-01	8.26E-01		2.86E+00	4.98E-04	Up	-9.03E+00	4.06E-02		2.19E+00	4.81E-04	Up	Unigene102197_All//gi|300507131|gb|ADK23941.1|//5.00E-43//KASIII [Gossypium hirsutum]	Unigene102197_All//1.00E-36//AT1G62640| KAS III (3-KETOACYL-ACYL CARRIER PROTEIN SYNTHASE III); 3-oxoacyl-[acyl-carrier-protein] synthase/ catalytic/ transferase, transferring acyl groups other than amino-acyl groups	Unigene102197_All//4.00E-116//gi|48815960|gb|CO117273.1|CO117273		ko00061|Fatty acid biosynthesis	GO:0004312//GO:0006631	nr -
Unigene1022_All	3085	4.94	4.75	8.35	12.45	17.22	9.88	1.55	6.06	1.14	-5.62E-02	7.18E-01		7.59E-01	8.80E-12		4.68E-01	2.08E-12		-3.33E-01	8.28E-05		1.97E+00	5.24E-46	Up	-4.47E-01	6.33E-02		Unigene1022_All//gi|1321686|emb|CAA66376.1|//0.00E+00//light repressible receptor protein kinase [Arabidopsis thaliana]	Unigene1022_All//2.00E-178//AT4G29990| light repressible receptor protein kinase			ko04626|Plant-pathogen interaction	GO:0000166//GO:0004672	nr +
Unigene10221_All	334	0.78	0.46	0.32	0.30	1.03	0.68	0.00	0.00	2.27	-7.83E-01	6.20E-01		-1.30E+00	4.37E-01		1.79E+00	2.58E-01		1.18E+00	5.50E-01		null	null		1.11E+01	1.39E-05	Up			Unigene10221_All//4.00E-07//TC142752				
Unigene10223_All	701	0.00	0.00	0.00	0.00	0.00	0.00	4.34	5.43	0.00	null	null		null	null		null	null		null	null		3.21E-01	2.87E-01		-1.21E+01	2.16E-22	Down	Unigene10223_All//gi|115445597|ref|NP_001046578.1|//6.00E-81//Os02g0287000 [Oryza sativa Japonica Group] >gi|113536109|dbj|BAF08492.1| Os02g0287000 [Oryza sativa Japonica Group]	Unigene10223_All//9.00E-80//AT4G34670| 40S ribosomal protein S3A (RPS3aB)			ko03010|Ribosome	GO:0005198//GO:0010467//GO:0030529	nr -

Unigene10226_All	711	0.00	71.91	36.10	0.00	0.00	0.00	0.00	0.00	0.00	1.61E+01	2.30E-295	Up	1.51E+01	2.51E-145	Up	null	null		null	null		null	null		null	null	
Unigene10227_All	473	0.77	0.32	0.00	1.90	1.89	1.81	2.68	0.35	3.54	-1.27E+00	3.36E-01		-9.60E+00	2.79E-02		-2.20E-03	9.78E-01		-6.34E-02	9.39E-01		-2.92E+00	1.46E-05	Down	4.00E-01	3.78E-01	
Unigene10229_All	254	2.88	0.00	0.42	2.55	1.90	3.56	0.33	0.49	0.16	-1.15E+01	2.11E-04	Down	-2.78E+00	1.05E-02		-4.26E-01	6.09E-01		4.80E-01	5.50E-01		5.69E-01	7.83E-01		-1.09E+00	6.78E-01	
Unigene102296_All	210	0.25	0.00	0.00	4.98	3.28	0.22	0.00	0.00	0.00	-7.96E+00	6.22E-01		-7.96E+00	6.35E-01		-6.03E-01	3.67E-01		-4.53E+00	1.60E-05	Down	null	null		null	null	
Unigene1023_All	1442	8.56	4.81	5.83	10.23	8.43	5.86	3.69	1.07	0.39	-8.31E-01	3.05E-07		-5.54E-01	9.15E-04		-2.79E-01	5.87E-02		-8.04E-01	1.66E-08		-1.78E+00	4.34E-12	Down	-3.26E+00	1.85E-24	Down
Unigene10230_All	691	0.00	53.82	27.36	0.00	0.00	0.00	0.00	0.00	0.00	1.57E+01	1.29E-214	Up	1.47E+01	8.83E-107	Up	null	null		null	null		null	null		null	null	
Unigene10232_All	521	1.41	0.97	0.00	2.87	3.04	0.35	0.89	0.16	0.00	-5.32E-01	5.13E-01		-1.05E+01	4.13E-04	Down	8.40E-02	8.96E-01		-3.05E+00	7.42E-06	Down	-2.48E+00	3.38E-02		-9.80E+00	1.10E-03	
Unigene10234_All	314	0.17	2.58	0.00	1.90	1.75	1.73	1.48	0.93	1.14	3.95E+00	7.52E-04	Up	-7.38E+00	6.35E-01		-1.18E-01	8.76E-01		-1.41E-01	8.80E-01		-6.69E-01	5.09E-01		-3.75E-01	6.65E-01	
Unigene102349_All	209	0.75	5.58	3.31	1.19	0.99	3.24	120.97	99.19	62.33	2.89E+00	2.98E-04	Up	2.14E+00	3.67E-02		-2.70E-01	8.23E-01		1.44E+00	1.28E-01		-2.86E-01	3.70E-03		-9.57E-01	5.16E-22	
Unigene10236_All	804	0.00	0.00	0.00	0.00	0.00	0.00	13.09	14.87	1.64	null	null		null	null		null	null		null	null		1.83E-01	2.62E-01		-3.00E+00	4.18E-44	Down
Unigene10239_All	698	0.00	9.15	5.04	0.00	0.00	0.00	0.00	0.00	0.00	1.32E+01	1.85E-36	Up	1.23E+01	1.88E-19	Up	null	null		null	null		null	null		null	null	
Unigene1024_All	2200	20.36	5.37	7.82	20.38	19.03	11.58	5.59	1.22	1.30	-1.92E+00	4.43E-87	Down	-1.38E+00	2.23E-52	Down	-9.85E-02	2.92E-01		-8.16E-01	3.22E-25		-2.20E+00	2.84E-35	Down	-2.10E+00	2.33E-34	Down
Unigene10241_All	790	0.00	50.22	19.75	0.00	0.00	0.00	0.00	0.00	0.00	1.56E+01	5.33E-229	Up	1.43E+01	5.59E-88	Up	null	null		null	null		null	null		null	null	
Unigene10242_All	517	0.00	12.84	6.90	0.00	0.00	0.00	0.00	0.00	0.00	1.36E+01	6.43E-38	Up	1.28E+01	9.42E-20	Up	null	null		null	null		null	null		null	null	
Unigene10243_All	275	0.38	0.37	0.00	1.45	0.88	1.48	6.61	3.34	21.88	-4.62E-02	9.91E-01		-8.57E+00	4.00E-01		-7.25E-01	5.47E-01		2.86E-02	9.85E-01		-9.83E-01	2.32E-02		1.73E+00	2.90E-13	Up
Unigene102458_All	289	7.06	4.38	2.21	13.79	9.65	9.38	32.19	33.70	26.47	-6.88E-01	1.27E-01		-1.68E+00	8.93E-04	Down	-5.15E-01	8.49E-02		-5.56E-01	7.28E-02		6.62E-02	7.27E-01		-2.82E-01	9.80E-02	
Unigene10247_All	405	0.00	4.25	1.97	0.00	0.00	0.00	0.00	0.00	0.00	1.21E+01	8.00E-10	Up	1.09E+01	1.72E-04	Up	null	null		null	null		null	null		null	null	
Unigene10248_All	675	0.00	23.50	10.33	0.00	0.00	0.00	0.00	0.00	0.00	1.45E+01	3.99E-91	Up	1.33E+01	5.73E-39	Up	null	null		null	null		null	null		null	null	
Unigene10249_All	281	0.00	0.00	0.00	0.00	0.00	0.00	12.94	4.61	7.51	null	null		null	null		null	null		null	null		-1.49E+00	9.84E-07	Down	-7.84E-01	4.67E-03	
Unigene1025_All	2394	3.74	4.66	1.40	8.57	10.66	12.04	7.05	2.62	3.74	3.17E-01	7.13E-02		-1.42E+00	2.77E-11	Down	3.14E-01	2.65E-03		4.90E-01	5.93E-07		-1.43E+00	9.40E-27	Down	-9.12E-01	6.59E-14	
Unigene10250_All	619	0.00	9.25	3.79	0.00	0.06	0.00	0.00	0.00	0.00	1.32E+01	1.13E-32	Up	1.19E+01	6.41E-13	Up	5.80E+00	8.41E-01		null	null		null	null		null	null	
Unigene10251_All	456	0.00	0.11	0.23	0.22	0.00	0.59	1.76	1.74	64.30	6.80E+00	6.23E-01		7.87E+00	4.03E-01		-7.77E+00	3.18E-01		1.44E+00	4.10E-01		-1.66E-02	1.02E+00		5.19E+00	1.37E-179	Up
Unigene102510_All	318	14.64	35.37	7.87	23.03	21.77	19.32	12.76	17.35	7.14	1.27E+00	1.57E-12	Up	-8.96E-01	2.17E-03		-8.13E-02	7.69E-01		-2.54E-01	2.93E-01		4.43E-01	5.55E-02		-8.38E-01	1.34E-03	
Unigene102517_All	339	2.47	0.15	0.47	0.29	0.81	0.27	2.37	2.10	1.65	-4.05E+00	4.66E-04	Down	-2.39E+00	1.14E-02		1.47E+00	4.20E-01		-1.41E-01	9.54E-01		-1.77E-01	7.93E-01		-5.26E-01	4.35E-01	
Unigene10252_All	455	0.00	1.89	2.11	0.00	0.00	0.00	0.00	0.00	0.00	1.09E+01	4.95E-05	Up	1.10E+01	2.46E-05	Up	null	null		null	null		null	null		null	null	
Unigene10254_All	574	0.00	2.38	3.25	0.00	0.00	0.00	0.00	0.00	0.00	1.12E+01	7.80E-08	Up	1.17E+01	2.89E-10	Up	null	null		null	null		null	null		null	null	
Unigene102563_All	403	0.26	6.16	0.26	0.00	0.17	0.90	0.00	0.00	0.00	4.57E+00	1.16E-11	Up	2.53E-02	1.00E+00		7.42E+00	6.17E-01		9.81E+00	2.38E-02		null	null		null	null	
Unigene102572_All	210	0.00	3.14	1.01	2.61	1.48	3.87	1.01	1.00	0.38	1.16E+01	6.26E-04	Up	9.99E+00	1.42E-01		-8.22E-01	3.96E-01		5.69E-01	4.96E-01		-1.67E-02	1.02E+00		-1.41E+00	3.81E-01	
Unigene102582_All	298	0.00	0.51	0.00	0.00	0.00	0.91	0.43	6.87	2.94	8.99E+00	2.37E-01		null	null		null	null		9.83E+00	7.06E-02		4.01E+00	5.29E-11	Up	2.79E+00	5.37E-04	Up
Unigene102587_All	243	0.00	0.00	0.00	0.21	0.00	0.00	5.05	0.17	0.16	null	null		null	null		-7.68E+00	5.66E-01		-7.68E+00	6.47E-01		-4.87E+00	1.49E-07	Down	-4.94E+00	6.14E-08	Down
Unigene102591_All	297	0.18	0.00	0.00	0.50	1.39	3.50	0.71	0.00	0.00	-7.46E+00	6.22E-01		-7.46E+00	6.35E-01		1.47E+00	2.80E-01		2.80E+00	8.13E-04	Up	-9.48E+00	7.48E-02		-9.48E+00	5.84E-02	
Unigene10260_All	801	0.00	0.25	0.00	0.12	0.00	0.06	3.85	0.26	2.39	7.98E+00	1.38E-01		null	null		-6.96E+00	3.18E-01		-1.14E+00	6.79E-01		-3.88E+00	4.99E-16	Down	-6.91E-01	2.33E-02	
Unigene10261_All	408	0.00	0.50	4.57	0.85	1.18	0.11	0.41	0.20	0.00	8.96E+00	1.38E-01		1.22E+01	2.89E-10	Up	4.67E-01	7.04E-01		-2.95E+00	8.31E-02		-1.02E+00	6.16E-01		-8.70E+00	1.09E-01	
Unigene102612_All	200	0.00	0.00	0.00	0.00	0.00	0.00	1.90	9.82	6.97	null	null		null	null		null	null		null	null		2.37E+00	1.05E-06	Up	1.87E+00	4.97E-04	Up
Unigene10262_All	338	0.00	5.55	2.52	0.00	0.00	0.00	0.00	0.00	0.00	1.24E+01	1.11E-10	Up	1.13E+01	9.04E-05	Up	null	null		null	null		null	null		null	null	
Unigene102629_All	363	0.00	0.00	0.00	0.00	0.09	0.25	0.23	0.00	2.63	null	null		null	null		6.57E+00	8.41E-01		7.96E+00	4.83E-01		-7.86E+00	4.08E-01		3.50E+00	4.29E-05	Up
Unigene10263_All	622	0.00	9.04	4.71	0.00	0.11	0.00	0.00	0.00	0.00	1.31E+01	4.34E-32	Up	1.22E+01	3.52E-16	Up	6.79E+00	6.17E-01		null	null		null	null		null	null	
Unigene10266_All	712	0.00	45.47	14.51	0.00	0.19	0.00	0.00	0.00	0.00	1.55E+01	9.73E-187	Up	1.38E+01	5.38E-58	Up	7.60E+00	3.21E-01		null	null		null	null		null	null	
Unigene102670_All	220	0.00	0.23	0.00	2.72	0.47	0.00	1.15	0.38	0.36	7.85E+00	6.23E-01		null	null		-2.53E+00	1.36E-02		-1.14E+01	7.71E-04	Down	-1.60E+00	3.11E-01		-1.67E+00	2.57E-01	
Unigene10269_All	558	276.98	321.18	318.55	97.66	154.09	132.66	4.85	17.08	10.71	2.14E-01	1.90E-08		2.02E-01	3.02E-07		6.58E-01	3.26E-36		4.42E-01	4.51E-14		1.82E+00	1.79E-21	Up	1.14E+00	1.66E-07	Up
Unigene10271_All	347	0.00	6.57	3.53	0.00	0.00	0.00	0.00	0.00	0.00	1.27E+01	5.64E-13	Up	1.18E+01	9.13E-07	Up	null	null		null	null		null	null		null	null	
Unigene10272_All	759	0.00	12.42	5.47	0.00	0.14	0.00	0.00	0.00	0.00	1.36E+01	5.67E-54	Up	1.24E+01	4.84E-23	Up	7.09E+00	4.59E-01		null	null		null	null		null	null	
Unigene102727_All	389	4.71	5.34	3.70	14.34	10.09	12.07	62.39	22.56	37.07	1.82E-01	6.73E-01		-3.49E-01	4.94E-01		-5.07E-01	3.80E-02		-2.48E-01	3.92E-01		-1.47E+00	1.07E-39	Down	-7.51E-01	2.64E-14	
Unigene102730_All	204	0.00	0.50	0.78	0.24	0.00	0.89	328.29	717.49	291.95	8.96E+00	3.91E-01		9.61E+00	2.40E-01		-7.93E+00	5.66E-01		1.86E+00	4.59E-01		1.13E+00	1.30E-156	Up	-1.69E-01	3.38E-03	
Unigene10274_All	310	0.34	1.14	0.17	0.00	0.00	0.29	8.73	0.94	2.31	1.76E+00	2.18E-01		-9.75E-01	7.23E-01		null	null		8.19E+00	4.83E-01		-3.21E+00	3.86E-12	Down	-1.92E+00	2.33E-07	Down
Unigene102740_All	251	0.00	1.21	1.06	1.59	1.51	1.80	44.47	489.39	10.63	1.02E+01	4.45E-02		1.01E+01	8.04E-02		-7.32E-02	9.26E-01		1.81E-01	8.84E-01		3.46E+00	0.00E+00	Up	-2.06E+00	1.46E-30	Down

Unigene10226_All	Unigene10226_All//gi|57471708|gb|AAW50985.1|//2.00E-61//ribosomal protein L18 [Triticum aestivum]	Unigene10226_All//2.00E-57//AT3G05590| RPL18 (RIBOSOMAL PROTEIN L18); structural constituent of ribosome			ko03010|Ribosome	GO:0005198//GO:0010467//GO:0030529	nr -
Unigene10227_All							
Unigene10229_All							
Unigene102296_All			Unigene102296_All//2.00E-29//gi|164354102|gb|ES849373.1|ES849373				
Unigene1023_All	Unigene1023_All//gi|297791827|ref|XP_002863798.1|//2.00E-73//binding protein [Arabidopsis lyrata subsp. lyrata] >gi|297309633|gb|EFH40057.1| binding protein [Arabidopsis lyrata subsp. lyrata]	Unigene1023_All//2.00E-59//AT5G42340| binding / ubiquitin-protein ligase	Unigene1023_All//0.00E+00//TC179266			GO:0019787//GO:0032446//GO:0043234//GO:0044424	nr +
Unigene10230_All	Unigene10230_All//gi|297812211|ref|XP_002873989.1|//2.00E-69//ribosomal protein S8 [Arabidopsis lyrata subsp. lyrata] >gi|297319826|gb|EFH50248.1| ribosomal protein S8 [Arabidopsis lyrata subsp. lyrata]	Unigene10230_All//5.00E-62//AT5G20290| 40S ribosomal protein S8 (RPS8A)			ko03010|Ribosome	GO:0005198//GO:0009536//GO:0010467//GO:0015935//GO:0016020//GO:0022613//GO:0030312//GO:0031981	nr -
Unigene10232_All			Unigene10232_All//3.00E-15//DR454672				
Unigene10234_All	Unigene10234_All//gi|17979268|gb|AAL49950.1|//3.00E-10//At1g22970/F19G10_8 [Arabidopsis thaliana] >gi|21700855|gb|AAM70551.1| At1g22970/F19G10_8 [Arabidopsis thaliana]	Unigene10234_All//1.00E-11//AT1G22970| unknown protein	Unigene10234_All//2.00E-113//CO097471				nr -
Unigene102349_All			Unigene102349_All//6.00E-45//gi|109867479|gb|DW496455.1|DW496455				
Unigene10236_All	Unigene10236_All//gi|238014838|gb|ACR38454.1|//1.00E-14//unknown [Zea mays]		Unigene10236_All//9.00E-38//TC146753				nr -
Unigene10239_All							ESTscan -
Unigene1024_All	Unigene1024_All//gi|297791827|ref|XP_002863798.1|//0.00E+00//binding protein [Arabidopsis lyrata subsp. lyrata] >gi|297309633|gb|EFH40057.1| binding protein [Arabidopsis lyrata subsp. lyrata]	Unigene1024_All//0.00E+00//AT5G42340| binding / ubiquitin-protein ligase	Unigene1024_All//0.00E+00//TC179266		ko04141|Protein processing in endoplasmic reticulum//ko04120|Ubiquitin mediated proteolysis	GO:0019787//GO:0032446//GO:0043231//GO:0043234	nr +
Unigene10241_All	Unigene10241_All//sp|P92201|LCP8_DROME//5.00E-26//Larval cuticle protein 8 OS=Drosophila melanogaster GN=Lcp65Ag1 PE=1 SV=1						swissprot -
Unigene10242_All	Unigene10242_All//sp|P56562|CUD5_SCHGR//2.00E-10//Endocuticle structural glycoprotein SgAbd-5 OS=Schistocerca gregaria PE=1 SV=1						swissprot -
Unigene10243_All			Unigene10243_All//4.00E-25//gi|164363055|gb|ES820462.1|ES820462				
Unigene102458_All	Unigene102458_All//gi|45861745|gb|AAS78671.1|//1.00E-23//actin [Capsicum annuum]	Unigene102458_All//1.00E-23//AT5G59370| ACT4 (ACTIN 4); structural constituent of cytoskeleton	Unigene102458_All//3.00E-84//TC171105		ko04145|Phagosome	GO:0032559//GO:0043232	nr +
Unigene10247_All							
Unigene10248_All	Unigene10248_All//gi|24061807|gb|AAN39804.1|//5.00E-11//transferrin-like protein IDI-100 [Dunaliella salina]						nr -
Unigene10249_All	Unigene10249_All//gi|117607067|gb|ABK42077.1|//9.00E-39//ubiquitin extension protein [Capsicum annuum]	Unigene10249_All//2.00E-21//AT3G62250| UBQ5 (ubiquitin 5); protein binding / structural constituent of ribosome	Unigene10249_All//3.00E-07//ES818268		ko03010|Ribosome	GO:0005198//GO:0005488//GO:0010467//GO:0015935//GO:0016567//GO:0019941	nr +
Unigene1025_All	Unigene1025_All//gi|297791827|ref|XP_002863798.1|//1.00E-166//binding protein [Arabidopsis lyrata subsp. lyrata] >gi|297309633|gb|EFH40057.1| binding protein [Arabidopsis lyrata subsp. lyrata]	Unigene1025_All//7.00E-154//AT5G42340| binding / ubiquitin-protein ligase	Unigene1025_All//0.00E+00//TC147579		ko04141|Protein processing in endoplasmic reticulum//ko04120|Ubiquitin mediated proteolysis		nr -
Unigene10250_All	Unigene10250_All//gi|261853464|gb|ACY00391.1|//9.00E-27//calmodulin [Polygonum sibiricum]	Unigene10250_All//5.00E-22//AT5G37780| CAM1 (CALMODULIN 1); calcium ion binding			ko04626|Plant-pathogen interaction//ko04070|Phosphatidylinositol signaling system	GO:0005488//GO:0019932	nr -
Unigene10251_All	Unigene10251_All//gi|224061172|ref|XP_002300362.1|//5.00E-26//multidrug resistance protein ABC transporter family [Populus trichocarpa] >gi|222847620|gb|EEE85167.1| multidrug resistance protein ABC transporter family [Populus trichocarpa]	Unigene10251_All//7.00E-10//AT3G13100| ATMRP7; ATPase, coupled to transmembrane movement of substances	Unigene10251_All//6.00E-96//TC157562			GO:0009607//GO:0016020//GO:0017111	nr +
Unigene102510_All			Unigene102510_All//1.00E-105//TC167368				
Unigene102517_All	Unigene102517_All//gi|226499554|ref|NP_001149976.1|//1.00E-10//SAM domain family protein [Zea mays] >gi|195635833|gb|ACG37385.1| SAM domain family protein [Zea mays]	Unigene102517_All//3.00E-10//AT1G70180| sterile alpha motif (SAM) domain-containing protein	Unigene102517_All//7.00E-95//TC167190			GO:0006464//GO:0016301//GO:0032559	nr -
Unigene10252_All							ESTscan -
Unigene10254_All	Unigene10254_All//gi|116060285|emb|CAL55621.1|//1.00E-05//Myosin class II heavy chain (ISS) [Ostreococcus tauri]	Unigene10254_All//7.00E-05//AT5G41790| CIP1 (COP1-INTERACTIVE PROTEIN 1); protein binding					nr -
Unigene102563_All	Unigene102563_All//gi|5031277|gb|AAD38144.1|AF139497_1//4.00E-26//homeobox leucine zipper protein [Prunus armeniaca]	Unigene102563_All//6.00E-25//AT2G46680| ATHB-7 (ARABIDOPSIS THALIANA HOMEOBOX 7); transcription activator/ transcription factor	Unigene102563_All//2.00E-142//ES813725	HB		GO:0001071//GO:0003677//GO:0006351//GO:0006970//GO:0009755//GO:0010468//GO:0010557//GO:0030528//GO:0043231	nr +
Unigene102572_All							
Unigene102582_All			Unigene102582_All//6.00E-46//DN799979				
Unigene102587_All	Unigene102587_All//gi|224140739|ref|XP_002323736.1|//3.00E-06//predicted protein [Populus trichocarpa] >gi|222866738|gb|EEF03869.1| predicted protein [Populus trichocarpa]		Unigene102587_All//5.00E-24//TC148715				nr +
Unigene102591_All	Unigene102591_All//gi|15809986|gb|AAL06920.1|//2.00E-34//AT3g19540/T31J18_4 [Arabidopsis thaliana] >gi|22137022|gb|AAM91356.1| At3g19540/T31J18_4 [Arabidopsis thaliana]	Unigene102591_All//9.00E-36//AT3G19540| unknown protein	Unigene102591_All//9.00E-51//gi|78336860|gb|DT557134.1|DT557134			GO:0016020	nr -
Unigene10260_All	Unigene10260_All//gi|224094005|ref|XP_002310060.1|//3.00E-45//cytochrome P450 [Populus trichocarpa] >gi|222852963|gb|EEE90510.1| cytochrome P450 [Populus trichocarpa]	Unigene10260_All//3.00E-29//AT3G26280| CYP71B4; electron carrier/ heme binding / iron ion binding / monooxygenase/ oxygen binding	Unigene10260_All//4.00E-92//CO098968		ko00945|Stilbenoid, diarylheptanoid and gingerol biosynthesis//ko00903|Limonene and pinene degradation	GO:0005506//GO:0008152//GO:0016709	nr +
Unigene10261_All			Unigene10261_All//1.00E-07//CA993990				
Unigene102612_All							
Unigene10262_All	Unigene10262_All//gi|219112151|ref|XP_002177827.1|//1.00E-33//predicted protein [Phaeodactylum tricornutum CCAP 1055/1] >gi|217410712|gb|EEC50641.1| predicted protein [Phaeodactylum tricornutum CCAP 1055/1]					GO:0003824	nr +
Unigene102629_All	Unigene102629_All//gi|75755957|gb|ABA27034.1|//1.00E-11//TO66-1 [Taraxacum officinale]	Unigene102629_All//3.00E-10//AT1G17710| phosphatase	Unigene102629_All//2.00E-58//TC169946			GO:0016791	nr -
Unigene10263_All							
Unigene10266_All	Unigene10266_All//gi|38325811|gb|AAR17078.1|//3.00E-89//heat shock protein 70-1 [Nicotiana tabacum]	Unigene10266_All//2.00E-86//AT5G02500| HSC70-1 (HEAT SHOCK COGNATE PROTEIN 70-1); ATP binding	Unigene10266_All//2.00E-13//TC161167		ko03040|Spliceosome//ko04141|Protein processing in endoplasmic reticulum//ko04144|Endocytosis	GO:0004672//GO:0006464//GO:0032559//GO:0050896	nr +
Unigene102670_All			Unigene102670_All//6.00E-45//ES832413				
Unigene10269_All	Unigene10269_All//gi|224058639|ref|XP_002299579.1|//9.00E-57//cytochrome P450 [Populus trichocarpa] >gi|222846837|gb|EEE84384.1| cytochrome P450 [Populus trichocarpa]	Unigene10269_All//2.00E-50//AT2G45550| CYP76C4; electron carrier/ heme binding / iron ion binding / monooxygenase/ oxygen binding	Unigene10269_All//0.00E+00//gi|48740564|gb|CO071083.1|CO071083		ko00945|Stilbenoid, diarylheptanoid and gingerol biosynthesis//ko00903|Limonene and pinene degradation//ko00941|Flavonoid biosynthesis//ko00944|Flavone and flavonol biosynthesis	GO:0005506//GO:0008152//GO:0016709	nr +
Unigene10271_All							
Unigene10272_All	Unigene10272_All//sp|P27878|VIT1_CERCA//2.00E-119//Vitellogenin-1 OS=Ceratitis capitata GN=VG1-GAMMA PE=2 SV=1						swissprot -
Unigene102727_All	Unigene102727_All//gi|115473585|ref|NP_001060391.1|//1.00E-07//Os07g0636000 [Oryza sativa Japonica Group] >gi|113611927|dbj|BAF22305.1| Os07g0636000 [Oryza sativa Japonica Group]		Unigene102727_All//3.00E-23//TC179304			GO:0005488	nr +
Unigene102730_All			Unigene102730_All//2.00E-57//CD485591				
Unigene10274_All			Unigene10274_All//3.00E-146//ES846200				
Unigene102740_All	Unigene102740_All//gi|294992077|gb|ABG90381.2|//6.00E-21//glutathione S-transferase [Caragana korshinskii]	Unigene102740_All//2.00E-20//AT1G78380| ATGSTU19 (GLUTATHIONE S-TRANSFERASE TAU 19); glutathione binding / glutathione transferase	Unigene102740_All//2.00E-82//gi|31407290|gb|CD486325.1|CD486325		ko00480|Glutathione metabolism	GO:0009755//GO:0016765	nr -

Unigene10276_All	868	0.00	0.00	0.00	0.00	0.00	0.00	2.78	0.39	7.48	null	null		null	null		null	null		null	null		-2.85E+00	9.15E-10	Down	1.43E+00	4.61E-11	Up	Unigene10276_All//gi|5080804|gb|AAD39313.1|AC007258_2//5.00E-54//Hypothetical protein [Arabidopsis thaliana] >gi|15010642|gb|AAK73980.1|At1g59710/T30E16_31 [Arabidopsis thaliana] >gi|22137290|gb|AAM91490.1|At1g59710/T30E16_31 [Arabidopsis thaliana]	Unigene10276_All//2.00E-61//AT3G28630| FUNCTIONS IN: molecular_function unknown; INVOLVED IN: biological_process unknown; LOCATED IN: endomembrane system; EXPRESSED IN: leaf whorl, male gametophyte, flower, pollen tube; EXPRESSED DURING: L mature pollen stage, M germinated pollen stage, 4 anthesis; CONTAINS InterPro DOMAIN/s: Protein of unknown function DUF569 (InterPro:IPR007679), Actin_cross-linking (InterPro:IPR008999); BEST Arabidopsis thaliana protein match is: unknown protein (TAIR:AT1G59710.1); Has 121 Blast hits to 110 proteins in 10 species: Archae - 0; Bacteria - 0; Metazoa - 4; Fungi - 0; Plants - 117; Viruses - 0; Other Eukaryotes - 0 (source: NCBI BLink).					nr -
Unigene10277_All	217	0.00	0.00	0.00	0.00	0.00	0.00	0.39	0.00	3.49	null	null		null	null		null	null		null	null		-8.61E+00	4.08E-01		3.16E+00	6.83E-04	Up			Unigene10277_All//4.00E-15//TC171388				
Unigene10279_All	257	0.00	0.00	0.00	0.00	0.00	0.00	1.97	5.69	0.00	null	null		null	null		null	null		null	null		1.53E+00	2.96E-03		-1.09E+01	5.56E-04	Down							
Unigene102790_All	362	0.14	0.00	0.00	3.44	1.43	0.50	0.58	1.50	7.48	-7.17E+00	6.22E-01		-7.17E+00	6.35E-01		-1.27E+00	2.92E-02		-2.79E+00	1.35E-04	Down	1.36E+00	1.44E-01		3.68E+00	8.19E-14	Up	Unigene102790_All//gi|269996964|gb|ACZ57767.1|//5.00E-35//phospholipase A1 [Nicotiana attenuata]	Unigene102790_All//2.00E-24//AT4G16820| lipase class 3 family protein	Unigene102790_All//1.00E-115//gi|78333729|gb|DT554003.1|DT554003			GO:0004091//GO:0009536//GO:0044238	nr +
Unigene102794_All	255	0.00	0.00	0.00	0.59	0.54	0.00	0.99	0.66	5.16	null	null		null	null		-1.17E-01	9.12E-01		-9.19E+00	2.35E-01		-6.02E-01	6.49E-01		2.37E+00	7.20E-05	Up	Unigene102794_All//gi|7211984|gb|AAF40455.1|AC004809_13//1.00E-11//Strong similarity to the SEN3-like tRNA-processing protein F24L7.13 from A. thaliana gi|2914700 on BAC gb|AC003974. EST gb|AA712969 comes from this gene [Arabidopsis thaliana]	Unigene102794_All//4.00E-13//AT1G04810| 26S proteasome regulatory subunit, putative	Unigene102794_All//3.00E-87//CO124331		ko03050|Proteasome	GO:0005838//GO:0009894//GO:0016020//GO:0019941//GO:0031410//GO:0043231	nr -
Unigene102797_All	210	0.00	0.00	0.00	0.71	0.66	4.30	55.77	41.99	115.72	null	null		null	null		-1.18E-01	9.12E-01		2.60E+00	3.43E-03		-4.09E-01	6.17E-03		1.05E+00	1.65E-24	Up	Unigene102797_All//gi|18766934|gb|AAL79181.1|//5.00E-13//beta-caryophyllene synthase QHS1 [Artemisia annua]	Unigene102797_All//1.00E-09//AT5G23960| TPS21 (TERPENE SYNTHASE 21); (-)-E-beta-caryophyllene synthase/ alpha-humulene synthase	Unigene102797_All//6.00E-48//CO111120		ko00904|Diterpenoid biosynthesis//ko00900|Terpenoid backbone biosynthesis//ko00902|Monoterpenoid biosynthesis	GO:0046872	nr +
Unigene10281_All	794	0.00	0.00	0.00	0.00	0.00	0.00	7.35	63.43	0.25	null	null		null	null		null	null		null	null		3.11E+00	1.39E-208	Up	-4.87E+00	1.01E-35	Down			Unigene10281_All//6.00E-08//ES813936				
Unigene10282_All	577	0.00	22.22	7.57	0.00	0.00	0.00	0.00	0.00	0.00	1.44E+01	1.45E-73	Up	1.29E+01	3.09E-24	Up	null	null		null	null		null	null		null	null		Unigene10282_All//gi|219109896|ref|XP_002176701.1|//3.00E-55//nucleoside diphosphate kinase 1 [Phaeodactylum tricornutum CCAP 1055/1] >gi|217411236|gb|EEC51164.1|nucleoside diphosphate kinase 1 [Phaeodactylum tricornutum CCAP 1055/1]	Unigene10282_All//1.00E-51//AT4G09320| NDPK1; ATP binding / nucleoside diphosphate kinase			ko00230|Purine metabolism//ko00240|Pyrimidine metabolism	GO:0007602//GO:0009206//GO:0009209//GO:0016776//GO:0023033//GO:0032559//GO:0042995//GO:0043169//GO:0044424	nr +
Unigene10283_All	674	0.00	4.66	6.95	0.00	0.00	0.07	0.13	0.00	0.12	1.22E+01	7.08E-18	Up	1.28E+01	4.86E-26	Up	null	null		6.07E+00	7.05E-01		-6.97E+00	4.08E-01		-8.65E-02	9.74E-01		Unigene10283_All//gi|40287526|gb|AAR83877.1|//4.00E-69//60S ribosomal protein L19 [Capsicum annuum]	Unigene10283_All//2.00E-50//AT4G02230| 60S ribosomal protein L19 (RPL19C)			ko03010|Ribosome	GO:0005198//GO:0010467//GO:0015934//GO:0016020//GO:0022613	nr +
Unigene102840_All	205	0.00	3.21	0.52	1.46	1.34	1.98	0.00	0.61	0.19	1.16E+01	6.26E-04	Up	9.02E+00	4.03E-01		-1.18E-01	9.01E-01		4.44E-01	7.01E-01		9.26E+00	2.41E-01		7.60E+00	6.50E-01								
Unigene102848_All	334	0.00	0.30	1.12	0.45	0.31	0.54	1.52	7.51	21.95	8.25E+00	3.91E-01		1.01E+01	2.51E-02		-5.33E-01	7.86E-01		2.74E-01	9.06E-01		2.31E+00	3.91E-08	Up	3.85E+00	1.15E-37	Up	Unigene102848_All//gi|264664459|sp|COLGJ1.1|Y1743_ARATH//3.00E-43//RecName: Full=Probable LRR receptor-like serine/threonine-protein kinase At1g74360; Flags: Precursor >gi|224589485|gb|ACN59276.1|leucine-rich repeat receptor-like protein kinase [Arabidopsis thaliana]	Unigene102848_All//1.00E-44//AT1G74360| leucine-rich repeat transmembrane protein kinase, putative	Unigene102848_All//1.00E-155//DT467067			GO:0004672//GO:0004871//GO:0006464//GO:0007167//GO:0016791//GO:0031224//GO:0031410//GO:0032559//GO:0043231	nr -
Unigene10286_All	650	0.00	0.08	0.00	0.31	0.05	0.14	0.46	1.99	1.72	6.29E+00	6.23E-01		null	null		-2.53E+00	2.34E-01		-1.14E+00	5.70E-01		2.13E+00	3.40E-04	Up	1.91E+00	1.87E-03		Unigene10286_All//gi|18416730|ref|NP_567744.1|//3.00E-21//UNE17 (UNFERTILIZED EMBRYO SAC 17); identical protein binding / serine-type endopeptidase [Arabidopsis thaliana] >gi|4539429|emb|CAB38962.1|subtilisin protease-like [Arabidopsis thaliana] >gi|7269485|emb|CAB79488.1|subtilisin protease-like [Arabidopsis thaliana]	Unigene10286_All//2.00E-22//AT4G26330| UNE17 (UNFERTILIZED EMBRYO SAC 17); identical protein binding / serine-type endopeptidase				GO:0004175//GO:0005515//GO:0009566//GO:0019538//GO:0042579//GO:0044092	nr -
Unigene102877_All	312	0.17	2.92	1.19	2.24	2.65	1.30	0.68	0.00	0.64	4.12E+00	2.34E-04	Up	2.83E+00	9.32E-02		2.45E-01	8.03E-01		-7.79E-01	3.93E-01		-9.40E+00	7.48E-02		-8.60E-02	9.61E-01								
Unigene10289_All	708	0.00	0.00	0.00	0.00	0.00	0.00	12.00	7.85	1.24	null	null		null	null		null	null		null	null		-6.12E-01	5.16E-04		-3.28E+00	7.60E-39	Down			Unigene10289_All//1.00E-11//TC165956				
Unigene10290_All	331	0.00	0.46	0.00	3.31	1.46	0.00	2.17	0.38	1.44	8.84E+00	2.37E-01		null	null		-1.18E+00	5.94E-02		-1.17E+01	7.26E-07	Down	-2.52E+00	4.69E-03		-5.88E-01	4.09E-01		Unigene10290_All//gi|224101869|ref|XP_002312452.1|//5.00E-11//predicted protein [Populus trichocarpa] >gi|222852272|gb|EEE89819.1|predicted protein [Populus trichocarpa]		Unigene10290_All//1.00E-10//DW225279			GO:0016798	nr -
Unigene10291_All	665	0.00	0.00	0.00	0.00	0.00	0.00	2.29	0.19	1.14	null	null		null	null		null	null		null	null		-3.60E+00	9.55E-08	Down	-1.01E+00	2.94E-02		Unigene10291_All//gi|225447256|ref|XP_002272957.1|//2.00E-26//PREDICTED: similar to Os04g0636200 [Vitis vinifera]						nr -
Unigene10293_All	415	0.00	0.00	0.00	0.00	0.00	0.00	2.04	0.91	0.29	null	null		null	null		null	null		null	null		-1.17E+00	9.29E-02		-2.82E+00	5.38E-04	Down	Unigene10293_All//gi|159473469|ref|XP_001694856.1|//1.00E-07//acidic ribosomal protein P2 [Chlamydomonas reinhardtii] >gi|158276235|gb|EDP02008.1|acidic ribosomal protein P2 [Chlamydomonas reinhardtii]	Unigene10293_All//7.00E-06//AT3G44590| 60S acidic ribosomal protein P2 (RPP2D)			ko03010|Ribosome	GO:0043229	nr +
Unigene10299_All	386	0.00	0.00	0.00	0.00	0.00	0.00	1.10	3.79	2.99	null	null		null	null		null	null		null	null		1.79E+00	7.25E-04	Up	1.45E+00	1.09E-02								
Unigene102998_All	284	0.00	0.00	0.38	0.18	0.12	2.86	66.25	74.17	60.88	null	null		8.55E+00	4.03E-01		-5.32E-01	8.29E-01		4.03E+00	5.91E-04	Up	1.63E-01	1.70E-01		-1.22E-01	3.39E-01		Unigene102998_All//gi|14582310|gb|AAK69446.1|//4.00E-51//proline-rich protein-2 [Gossypium hirsutum]		Unigene102998_All//3.00E-140//NP386003				nr -
Unigene10300_All	1671	0.00	0.00	0.00	0.00	0.00	0.00	8.35	12.18	0.19	null	null		null	null		null	null		null	null		5.45E-01	5.21E-07		-5.45E+00	1.23E-89	Down	Unigene10300_All//gi|299470262|emb|CBN79566.1|//1.00E-13//RecName: Full=Spherulin-4; Flags: Precursor [Ectocarpus siliculosus]						nr +
Unigene10301_All	896	0.00	5.09	2.50	0.00	0.00	0.00	0.00	0.00	0.00	1.23E+01	5.54E-26	Up	1.13E+01	2.50E-12	Up	null	null		null	null		null	null		null	null		Unigene10301_All//gi|301098633|ref|XP_002898409.1|//2.00E-06//lamin-like protein [Phytophthora infestans T30-4] >gi|262105180|gb|EEY63232.1|lamin-like protein [Phytophthora infestans T30-4]	Unigene10301_All//2.00E-05//AT2G32240| FUNCTIONS IN: molecular_function unknown; INVOLVED IN: response to cadmium ion; LOCATED IN: plasma membrane; EXPRESSED IN: 25 plant structures; EXPRESSED DURING: 13 growth stages; CONTAINS InterPro DOMAIN/s: Prefoldin (InterPro:IPR009053); BEST Arabidopsis thaliana protein match is: unknown protein (TAIR:AT1G05320.3); Has 221693 Blast hits to 95553 proteins in 2574 species: Archae - 2848; Bacteria - 44259; Metazoa - 97474; Fungi - 15490; Plants - 8386; Viruses - 1229; Other Eukaryotes - 52007 (source: NCBI BLink).			ko03440|Homologous recombination//ko03450|Non-homologous end-joining		nr +
Unigene10302_All	605	0.61	0.67	0.35	3.87	1.88	0.82	0.77	0.35	0.59	1.46E-01	8.98E-01		-7.82E-01	5.53E-01		-1.04E+00	8.02E-03		-2.24E+00	1.70E-06	Down	-1.15E+00	2.53E-01		-3.75E-01	6.65E-01		Unigene10302_All//gi|28950973|gb|AAO63410.1|//2.00E-89//At1g23340 [Arabidopsis thaliana]	Unigene10302_All//1.00E-88//AT1G10750| unknown protein	Unigene10302_All//4.00E-138//NP508247			GO:0044464	nr +
Unigene10303_All	322	0.33	1.57	3.14	0.00	0.00	0.00	0.00	0.00	0.00	2.28E+00	6.23E-02		3.27E+00	5.93E-04	Up	null	null		null	null		null	null		null	null		Unigene10303_All//gi|224138866|ref|XP_002322921.1|//4.00E-44//aldose reductase with ABC domain [Populus trichocarpa] >gi|222867551|gb|EEF04682.1|aldose reductase with ABC domain [Populus trichocarpa]	Unigene10303_All//2.00E-32//AT2G37770| aldo/keto reductase family protein	Unigene10303_All//2.00E-67//DR460967		ko00010|Glycolysis / Gluconeogenesis//ko00051|Fructose and mannose metabolism//ko00561|Glycerolipid metabolism//ko00591|Linoleic acid metabolism//ko00650|Butanoate metabolism	GO:0004033//GO:0006970//GO:0008152	nr +
Unigene10305_All	842	0.00	0.00	0.00	0.12	0.12	0.59	0.60	2.53	4.64	null	null		null	null		5.27E-02	9.69E-01		2.32E+00	6.89E-02		2.07E+00	3.04E-06	Up	2.94E+00	3.32E-16	Up							
Unigene103070_All	375	0.14	0.00	0.00	0.00	0.09	0.00	1.35	0.00	0.00	-7.12E+00	6.22E-01		-7.12E+00	6.35E-01		6.52E+00	8.41E-01		null	null		-1.04E+01	8.91E-04	Down	-1.04E+01	5.56E-04	Down	Unigene103070_All//gi|18412056|ref|NP_565185.1|//9.00E-08//POLD3; DNA-directed DNA polymerase [Arabidopsis thaliana] >gi|4836868|gb|AAD30571.1|AC007260_2 Hypothetical protein [Arabidopsis thaliana]		Unigene103070_All//1.00E-109//BG445037		ko00230|Purine metabolism//ko00240|Pyrimidine metabolism//ko03420|Nucleotide excision repair//ko03410|Base excision repair//ko03030|DNA replication//ko03440|Homologous recombination//ko03430|Mismatch repair		nr +
Unigene10309_All	376	0.00	0.00	0.00	0.27	0.00	0.00	281.57	54.91	230.87	null	null		null	null		-8.05E+00	3.18E-01		-8.05E+00	4.06E-01		-2.36E+00	0.00E+00	Down	-2.86E-01	6.87E-11		Unigene10309_All//gi|15224236|ref|NP_181844.1|//4.00E-07//proline-rich extensin-like family protein [Arabidopsis thaliana]		Unigene10309_All//0.00E+00//TC158176				nr +
Unigene1031_All	930	8.27	6.37	6.53	13.61	10.77	13.50	25.23	11.68	11.01	-3.76E-01	7.88E-02		-3.41E-01	1.38E-01		-3.37E-01	3.18E-02		-1.11E-02	9.42E-01		-1.11E+00	3.54E-25	Down	-1.20E+00	4.04E-29	Down	Unigene1031_All//gi|297842397|ref|XP_002889080.1|//1.00E-107//ribophorin I family protein [Arabidopsis lyrata subsp. lyrata] >gi|297334921|gb|EFH65339.1|ribophorin I family protein [Arabidopsis lyrata subsp. lyrata]	Unigene1031_All//3.00E-101//AT1G76400| ribophorin I family protein	Unigene1031_All//0.00E+00//gi|48813336|gb|CO114649.1|CO114649		ko04141|Protein processing in endoplasmic reticulum//ko00510|N-Glycan biosynthesis	GO:0004576//GO:0006464//GO:0031224//GO:0031410//GO:0043231	nr -
Unigene10310_All	639	0.25	1.03	0.00	2.03	0.00	0.00	2.58	0.78	0.69	2.07E+00	3.93E-02		-7.94E+00	2.47E-01		-1.10E+01	1.34E-09	Down	-1.10E+01	4.24E-08	Down	-1.72E+00	4.45E-04	Down	-1.91E+00	8.63E-05	Down	Unigene10310_All//gi|16326137|dbj|BAB70512.1|//7.00E-64//Myb [Nicotiana tabacum]	Unigene10310_All//4.00E-55//AT5G11510| MYB3R-4 (myb domain protein 3R-4); DNA binding / transcription coactivator/ transcription factor	Unigene10310_All//8.00E-07//ES830113	MYB		GO:0006350//GO:0010468//GO:0043231	nr -
Unigene103112_All	231	0.45	0.00	0.00	3.88	1.04	0.39	0.18	0.00	0.00	-8.82E+00	3.78E-01		-8.82E+00	4.00E-01		-1.90E+00	1.06E-02		-3.31E+00	5.80E-04	Down	-7.52E+00	6.65E-01		-7.52E+00	6.19E-01				Unigene103112_All//2.00E-32//DW500061				
Unigene10315_All	483	0.00	0.00	0.00	0.00	0.00	0.00	4.55	4.59	0.25	null	null		null	null		null	null		null	null		1.09E-02	1.02E+00		-4.20E+00	1.16E-12	Down	Unigene10315_All//gi|192910854|gb|ACF06535.1|//2.00E-39//60S ribosomal protein L27 [Elaeis guineensis]	Unigene10315_All//1.00E-38//AT2G32220| 60S ribosomal protein L27 (RPL27A)	Unigene10315_All//1.00E-07//gi|78337699|gb|DT557973.1|DT557973		ko03010|Ribosome	GO:0030529	nr +
Unigene10316_All	264	0.00	0.00	3.03	0.00	0.78	1.88	0.00	0.48	2.72	null	null		1.16E+01	1.72E-04	Up	9.61E+00	1.58E-01		1.09E+01	4.38E-03		8.89E+00	2.41E-01		1.14E+01	2.63E-05	Up							

Unigene103176_All	631	0.50	1.20	0.42	2.13	1.31	1.65	0.87	1.59	5.24	1.28E+00	1.28E-01		-2.38E-01	8.50E-01		-7.03E-01	2.18E-01		-3.73E-01	5.53E-01		8.68E-01	1.64E-01		2.59E+00	3.51E-12	Up	Unigene103176_All//gi|297839709|ref|XP_002887736.1|//2.00E-45//protein binding protein [Arabidopsis lyrata subsp. lyrata] >gi|297333577|gb|EFH63995.1| protein binding protein [Arabidopsis lyrata subsp. lyrata]	Unigene103176_All//1.00E-46//AT1G78420| protein binding / zinc ion binding	Unigene103176_All//3.00E-163//TC172240			GO:0003824//GO:0043231//GO:0046914	nr -
Unigene10318_All	972	0.00	37.85	14.52	0.00	0.00	0.00	0.00	0.00	0.00	1.52E+01	2.87E-212	Up	1.38E+01	1.69E-79	Up	null	null		null	null		null	null		null	null		Unigene10318_All//gi|226505998|ref|NP_001146929.1|//1.00E-115//60S ribosomal protein L7a [Zea mays] >gi|195605318|gb|ACG24489.1| 60S ribosomal protein L7a [Zea mays]	Unigene10318_All//1.00E-80//AT3G62870| 60S ribosomal protein L7A (RPL7aB)			ko03010|Ribosome	GO:0005198//GO:0009987//GO:0010467//GO:0030529//GO:0046872	nr +
Unigene1032_All	2509	11.49	9.21	11.08	21.96	22.37	24.08	33.64	19.42	12.85	-3.19E-01	1.73E-03		-5.26E-02	6.46E-01		2.68E-02	8.03E-01		1.33E-01	7.30E-02		-7.92E-01	1.33E-50		-1.39E+00	5.34E-129	Down	Unigene1032_All//gi|15223039|ref|NP_177766.1|//0.00E+00//ribophorin I family protein [Arabidopsis thaliana] >gi|22655006|gb|AAM98094.1| At1g76400/F15M4_10 [Arabidopsis thaliana]	Unigene1032_All//0.00E+00//AT1G76400| ribophorin I family protein	Unigene1032_All//0.00E+00//TC167797		ko04141|Protein processing in endoplasmic reticulum//ko00510|N-Glycan biosynthesis	GO:0004576//GO:0006464//GO:0031224//GO:0043231	nr +
Unigene10320_All	352	0.00	0.00	0.00	0.00	0.00	0.00	4.68	1.31	0.57	null	null		null	null		null	null		null	null		-1.84E+00	2.07E-04	Down	-3.05E+00	1.31E-07	Down	Unigene10320_All//gi|149392583|gb|ABR26094.1|//2.00E-10//retrotransposon protein [Oryza sativa Indica Group]		Unigene10320_All//8.00E-24//TC152783				nr +
Unigene10323_All	312	43.60	29.39	22.53	61.79	50.55	32.57	61.93	33.49	16.60	-5.69E-01	1.81E-04		-9.53E-01	2.84E-09		-2.90E-01	1.97E-02		-9.24E-01	1.13E-13		-8.87E-01	1.20E-14		-1.90E+00	6.17E-47	Down	Unigene10323_All//gi|416641|sp|P32294.1|AX22B_PHAAU//3.00E-09//RecName: Full=Auxin-induced protein 22B; AltName: Full=Indole-3-acetic acid-induced protein ARG4	Unigene10323_All//9.00E-10//AT5G43700| ATAUX2-11 (AUXIN INDUCIBLE 2-11); DNA binding / transcription factor	Unigene10323_All//2.00E-141//CB066529				nr -
Unigene103235_All	610	8.66	7.64	6.55	8.17	5.42	2.96	0.97	0.82	0.39	-1.81E-01	5.19E-01		-4.04E-01	1.44E-01		-5.92E-01	2.11E-02		-1.46E+00	1.22E-07	Down	-2.39E-01	7.65E-01		-1.31E+00	1.15E-01		Unigene103235_All//gi|158427472|gb|ABW38331.1|//7.00E-87//chloroplast fructose-1,6-bisphosphatase II [Fragaria x ananassa] >gi|190151803|gb|ACE63522.1| chloroplast fructose-1,6-bisphosphatase [Fragaria x ananassa]	Unigene103235_All//2.00E-82//AT5G64380| fructose-1,6-bisphosphatase family protein	Unigene103235_All//0.00E+00//gi|48800926|gb|CO102240.1|CO102240		ko00010|Glycolysis / Gluconeogenesis//ko00710|Carbon fixation in photosynthetic organisms//ko00051|Fructose and mannose metabolism//ko00030|Pentose phosphate pathway	GO:0005975//GO:0009536//GO:0019203	nr +
Unigene10324_All	320	15.21	6.33	14.48	12.61	8.82	3.25	0.00	0.00	0.00	-1.26E+00	7.65E-06	Down	-7.08E-02	8.06E-01		-5.15E-01	8.41E-02		-1.96E+00	3.44E-09	Down	null	null		null	null				Unigene10324_All//8.00E-30//gi|164302226|gb|ES807570.1|ES807570				
Unigene103249_All	281	0.00	0.00	1.14	0.18	0.25	2.89	0.00	0.00	0.00	null	null		1.02E+01	4.54E-02		4.67E-01	9.62E-01		4.03E+00	5.91E-04	Up	null	null		null	null				Unigene103249_All//2.00E-11//TC171826				
Unigene10326_All	341	0.00	3.86	1.25	0.00	0.00	0.00	0.00	0.00	0.00	1.19E+01	1.50E-07	Up	1.03E+01	1.39E-02		null	null		null	null		null	null		null	null								ESTscan +
Unigene10328_All	321	0.00	0.00	0.00	0.31	0.64	0.14	441.51	118.88	71.98	null	null		null	null		1.05E+00	6.13E-01		-1.14E+00	6.79E-01		-1.89E+00	0.00E+00	Down	-2.62E+00	0.00E+00	Down							ESTscan +
Unigene10329_All	929	0.00	0.38	0.11	0.00	0.00	0.00	0.27	2.92	14.32	8.58E+00	2.47E-02		6.84E+00	4.03E-01		null	null		null	null		3.42E+00	8.23E-13	Up	5.71E+00	4.04E-85	Up	Unigene10329_All//gi|224054472|ref|XP_002298277.1|//5.00E-68//NAC domain protein, IPR003441 [Populus trichocarpa] >gi|224149083|ref|XP_002336754.1| NAC domain protein, IPR003441 [Populus trichocarpa] >gi|222836661|gb|EEE75054.1| NAC domain protein, IPR003441 [Populus trichocarpa] >gi|222845535|gb|EEE83082.1| NAC domain protein, IPR003441 [Populus trichocarpa]	Unigene10329_All//6.00E-61//AT5G22380| anac090 (Arabidopsis NAC domain containing protein 90); transcription factor	Unigene10329_All//0.00E+00//TC166110	NAC		GO:0001071//GO:0009743//GO:0010468//GO:0032501	nr +
Unigene1033_All	686	12.13	17.21	19.56	20.33	15.16	42.20	50.79	109.18	86.47	5.05E-01	2.23E-03		6.90E-01	1.39E-05		-4.24E-01	2.99E-03		1.05E+00	9.65E-25	Up	1.10E+00	8.74E-78	Up	7.68E-01	9.20E-35		Unigene1033_All//gi|4263517|gb|AAD15343.1|//5.00E-58//similar to PHZF, catalyzing the hydroxylation of phenazine-1-carboxylic acid to 2-hydroxy-phenazine-1-carboxylic acid [Arabidopsis thaliana] >gi|51972158|gb|AAU15183.1| At4g02860 [Arabidopsis thaliana]	Unigene1033_All//3.00E-59//AT4G02860| catalytic	Unigene1033_All//0.00E+00//gi|164253821|gb|ES839366.1|ES839366			GO:0008152	nr -
Unigene10330_All	493	0.00	0.00	0.00	0.00	0.00	0.00	2.92	2.71	0.00	null	null		null	null		null	null		null	null		-1.04E-01	8.37E-01		-1.15E+01	1.19E-10	Down							ESTscan +
Unigene103316_All	377	0.00	0.00	0.00	1.45	0.00	0.00	0.00	0.00	0.00	null	null		null	null		-1.05E+01	4.01E-04	Down	-1.05E+01	1.52E-03		null	null		null	null		Unigene103316_All//gi|115452451|ref|NP_001049826.1|//1.00E-15//Os03g0295700 [Oryza sativa Japonica Group] >gi|108707640|gb|ABF95435.1| expressed protein [Oryza sativa (japonica cultivar-group)] >gi|113548297|dbj|BAF11740.1| Os03g0295700 [Oryza sativa Japonica Group]	Unigene103316_All//3.00E-21//AT5G58510| unknown protein	Unigene103316_All//1.00E-121//TC142475			GO:0009536	nr -
Unigene10332_All	470	0.00	43.88	21.98	0.00	0.00	0.00	0.00	0.00	0.00	1.54E+01	1.15E-118	Up	1.44E+01	5.37E-58	Up	null	null		null	null		null	null		null	null		Unigene10332_All//gi|15229845|ref|NP_187143.1|//7.00E-41//40S ribosomal protein S24 (RPS24A) [Arabidopsis thaliana] >gi|11134742|sp|Q9SS17.1|RS241_ARATH RecName: Full=40S ribosomal protein S24-1 >gi|15215672|gb|AAK91381.1| AT3g04920/T9J14_13 [Arabidopsis thaliana] >gi|20334888|gb|AAM16200.1| AT3g04920/T9J14_13 [Arabidopsis thaliana]	Unigene10332_All//4.00E-34//AT3G04920| 40S ribosomal protein S24 (RPS24A)			ko03010|Ribosome	GO:0005198//GO:0005488//GO:0009536//GO:0010467//GO:0015935//GO:0016020//GO:0022613//GO:0031981	nr -
Unigene103341_All	215	0.00	0.47	0.25	0.23	0.00	0.42	0.79	4.67	0.37	8.88E+00	3.91E-01		7.95E+00	6.42E-01		-7.86E+00	5.65E-01		8.59E-01	7.97E-01		2.57E+00	4.79E-04	Up	-1.09E+00	5.54E-01		Unigene103341_All//gi|51536426|gb|AAU05451.1|//1.00E-13//At5g19870 [Arabidopsis thaliana] >gi|51972068|gb|AAU15138.1| At5g19870 [Arabidopsis thaliana]	Unigene103341_All//4.00E-15//AT5G19870| unknown protein	Unigene103341_All//3.00E-56//CO129484			GO:0044464	nr +
Unigene10335_All	579	0.72	0.70	0.92	0.60	2.68	3.43	0.07	0.00	0.00	-4.62E-02	9.80E-01		3.47E-01	7.06E-01		2.15E+00	2.30E-04	Up	2.51E+00	3.78E-06	Up	-6.19E+00	6.65E-01		-6.19E+00	6.19E-01		Unigene10335_All//gi|297832296|ref|XP_002884030.1|//1.00E-34//tetracycline transporter [Arabidopsis lyrata subsp. lyrata] >gi|297329870|gb|EFH60289.1| tetracycline transporter [Arabidopsis lyrata subsp. lyrata]	Unigene10335_All//4.00E-32//AT2G16990| tetracycline transporter	Unigene10335_All//1.00E-66//TC151593			GO:0005215//GO:0006810//GO:0016020//GO:0031410//GO:0042221	nr +
Unigene103358_All	211	0.74	1.20	0.76	1.18	2.12	7.49	2.00	0.79	0.19	6.91E-01	6.27E-01		2.54E-02	1.00E+00		8.46E-01	5.14E-01		2.67E+00	2.71E-05	Up	-1.34E+00	2.25E-01		-3.41E+00	1.24E-02								
Unigene10338_All	545	0.00	0.00	0.00	0.46	1.01	0.00	0.39	0.84	2.19	null	null		null	null		1.15E+00	2.97E-01		-8.84E+00	7.36E-02		1.12E+00	2.74E-01		2.50E+00	1.03E-04	Up	Unigene10338_All//gi|66792688|gb|AAY56446.1|//3.00E-46//At1g63930 [Arabidopsis thaliana] >gi|115311479|gb|ABI93920.1| At1g63930 [Arabidopsis thaliana]	Unigene10338_All//2.00E-38//AT1G63930| unknown protein	Unigene10338_All//0.00E+00//TC174086			GO:0003824//GO:0008152//GO:0030554	nr -
Unigene103382_All	234	96.82	35.95	76.69	247.79	237.52	174.86	267.41	251.49	161.40	-1.43E+00	2.98E-29	Down	-3.36E-01	5.60E-03		-6.11E-02	4.84E-01		-5.03E-01	4.57E-14		-8.86E-02	2.00E-01		-7.29E-01	1.14E-33				Unigene103382_All//4.00E-80//gi|78347287|gb|DT567561.1|DT567561				
Unigene1034_All	616	1.53	2.96	1.30	0.16	1.90	3.23	1.51	7.67	11.32	9.54E-01	4.89E-02		-2.38E-01	7.23E-01		3.56E+00	2.92E-05	Up	4.32E+00	1.57E-09	Up	2.34E+00	5.50E-15	Up	2.91E+00	4.94E-28	Up	Unigene1034_All//gi|79461947|ref|NP_192195.2|//2.00E-27//catalytic [Arabidopsis thaliana]	Unigene1034_All//6.00E-29//AT4G02860| catalytic	Unigene1034_All//6.00E-140//gi|48778677|gb|CO088043.1|CO088043			GO:0008152	nr -
Unigene10343_All	513	0.00	0.00	0.00	0.00	0.00	0.00	5.69	0.65	2.95	null	null		null	null		null	null		null	null		-3.13E+00	9.74E-13	Down	-9.46E-01	2.81E-03		Unigene10343_All//gi|15236902|ref|NP_194418.1|//3.00E-33//stigma-specific Stig1 family protein [Arabidopsis thaliana] >gi|4455211|emb|CAB36534.1| STIG1 like protein [Arabidopsis thaliana] >gi|7269541|emb|CAB79543.1| STIG1 like protein [Arabidopsis thaliana]	Unigene10343_All//2.00E-34//AT4G26880| stigma-specific Stig1 family protein					nr +
Unigene103449_All	402	0.00	0.00	0.00	0.12	0.00	0.00	4.52	0.31	0.89	null	null		null	null		-6.95E+00	5.65E-01		-6.95E+00	6.46E-01		-3.86E+00	1.31E-09	Down	-2.34E+00	1.83E-06	Down							ESTscan -
Unigene10345_All	494	0.00	6.56	6.68	0.00	0.07	0.00	0.00	0.00	0.16	1.27E+01	1.88E-18	Up	1.27E+01	2.92E-18	Up	6.12E+00	8.41E-01		null	null		null	null		7.33E+00	4.06E-01		Unigene10345_All//gi|157093615|gb|ABV22462.1|//2.00E-63//ribosomal protein S23e [Oxyrrhis marina]	Unigene10345_All//8.00E-61//AT5G02960| 40S ribosomal protein S23 (RPS23B)	Unigene10345_All//8.00E-28//TC142994		ko03010|Ribosome	GO:0005198//GO:0010467//GO:0033279	nr -
Unigene103456_All	251	1.67	3.23	1.27	6.35	10.70	3.96	2.36	0.83	0.00	9.54E-01	2.27E-01		-3.90E-01	7.18E-01		7.53E-01	4.91E-02		-6.82E-01	2.07E-01		-1.50E+00	9.14E-02		-1.12E+01	1.42E-04	Down			Unigene103456_All//4.00E-80//TC163108				
Unigene10346_All	1122	0.00	16.62	0.09	0.09	0.06	0.48	0.00	0.30	4.54	1.40E+01	3.09E-107	Up	6.57E+00	4.03E-01		-5.32E-01	8.20E-01		2.44E+00	4.41E-02		8.22E+00	1.28E-02		1.22E+01	1.69E-36	Up	Unigene10346_All//gi|225436325|ref|XP_002267961.1|//2.00E-53//PREDICTED: similar to CBF-like transcription factor [Vitis vinifera] >gi|39578548|gb|AAR28676.1| CBF-like transcription factor [Vitis vinifera]	Unigene10346_All//8.00E-35//AT4G25490| CBF1 (C-REPEAT/DRE BINDING FACTOR 1); DNA binding / transcription activator/ transcription factor	Unigene10346_All//0.00E+00//TC142390	AP2-EREBP			nr +
Unigene10347_All	1014	0.36	0.10	0.00	0.20	0.75	0.27	1.33	2.39	4.13	-1.85E+00	1.87E-01		-8.50E+00	2.79E-02		1.93E+00	3.36E-02		4.43E-01	7.64E-01		8.41E-01	2.12E-02		1.63E+00	8.52E-09	Up	Unigene10347_All//gi|264664473|sp|COLGK4.1|Y2165_ARATH//6.00E-92//RecName: Full=Probable LRR receptor-like serine/threonine-protein kinase At2g16250; Flags: Precursor >gi|224589511|gb|ACN59289.1| leucine-rich repeat receptor-like protein kinase [Arabidopsis thaliana]	Unigene10347_All//8.00E-70//AT2G16250| leucine-rich repeat transmembrane protein kinase, putative	Unigene10347_All//4.00E-28//TC176578		ko04626|Plant-pathogen interaction	GO:0004672//GO:0005618//GO:0006464//GO:0007167//GO:0016020//GO:0032559	nr -
Unigene103479_All	364	0.58	0.14	0.15	0.96	0.19	2.85	0.12	0.34	1.97	-2.05E+00	3.34E-01		-1.97E+00	3.82E-01		-2.34E+00	1.01E-01		1.57E+00	2.92E-02		1.57E+00	5.46E-01		4.08E+00	2.41E-04	Up			Unigene103479_All//4.00E-10//TC158284				
Unigene10348_All	346	2.12	0.00	0.15	1.01	1.59	0.26	0.24	0.00	0.00	-1.10E+01	2.11E-04	Down	-3.78E+00	2.79E-03		6.60E-01	5.44E-01		-1.95E+00	1.96E-01		-7.93E+00	4.08E-01		-7.93E+00	3.62E-01		Unigene10348_All//gi|297839191|ref|XP_002887477.1|//2.00E-17//sugar transporter family protein [Arabidopsis lyrata subsp. lyrata] >gi|297333318|gb|EFH63736.1| sugar transporter family protein [Arabidopsis lyrata subsp. lyrata]	Unigene10348_All//7.00E-18//AT1G73220| AtOCT1 (Arabidopsis thaliana ORGANIC CATION/CARNITINE TRANSPORTER1); carbohydrate transmembrane transporter/ carnitine transporter/ transporter				GO:0006810//GO:0022891//GO:0031224//GO:0051183	nr -
Unigene103480_All	232	0.90	0.00	0.00	0.64	0.89	1.17	6.01	9.37	0.69	-9.82E+00	1.23E-01		-9.82E+00	1.47E-01		4.68E-01	8.26E-01		8.59E-01	6.32E-01		6.39E-01	1.05E-01		-3.13E+00	1.09E-06	Down			Unigene103480_All//2.00E-60//gi|109874097|gb|DW503072.1|DW503072				
Unigene10349_All	317	2.97	0.16	0.67	1.41	0.76	0.28	0.40	0.13	0.00	-4.22E+00	1.36E-04	Down	-2.14E+00	1.16E-02		-8.95E-01	4.13E-01		-2.31E+00	7.82E-02		-1.60E+00	5.36E-01		-8.64E+00	2.01E-01		Unigene10349_All//gi|15241158|ref|NP_197471.1|//5.00E-35//MATE efflux protein-related [Arabidopsis thaliana] >gi|67633808|gb|AAY78828.1| MATE efflux protein-related [Arabidopsis thaliana]	Unigene10349_All//2.00E-36//AT5G19700| MATE efflux protein-related	Unigene10349_All//2.00E-169//gi|78347917|gb|DT568191.1|DT568191			GO:0015291//GO:0015893//GO:0044464	nr -
Unigene1035_All	1492	0.18	1.87	0.43	0.40	0.78	1.15	1.16	6.42	8.41	3.41E+00	1.06E-10	Up	1.29E+00	1.87E-01		9.70E-01	1.37E-01		1.52E+00	3.74E-03		2.47E+00	1.99E-31	Up	2.86E+00	5.03E-49	Up	Unigene1035_All//gi|15218739|ref|NP_171820.1|//5.00E-23//phenazine biosynthesis PhzC/PhzF family protein [Arabidopsis thaliana] >gi|3850584|gb|AAC72124.1| ESTs gb|H37641 and gb|AA651422 come from this gene [Arabidopsis thaliana]	Unigene1035_All//6.00E-24//AT1G03210| phenazine biosynthesis PhzC/PhzF family protein	Unigene1035_All//2.00E-101//TC165972				nr -

Unigene103566_All	259	6.26	0.00	0.00	0.00	0.27	0.52	0.00	0.00	0.00	-1.26E+01	2.11E-09	Down	-1.26E+01	7.39E-09	Down	8.05E+00	6.17E-01		9.03E+00	3.13E-01		null	null		null	null		Unigene103566_All//gi|297813081|ref|XP_002874424.1|//4.00E-06//hypothetical protein ARALYDRAFT_489647 [Arabidopsis lyrata subsp. lyrata] >gi|297320261|gb|EFH50683.1| hypothetical protein ARALYDRAFT_489647 [Arabidopsis lyrata subsp. lyrata]						nr +
Unigene10357_All	413	0.00	0.00	0.00	0.00	0.00	0.00	1.64	3.04	5.98	null	null		null	null		null	null		null	null		8.90E-01	1.02E-01		1.87E+00	1.66E-06	Up	Unigene10357_All//gi|224137294|ref|XP_002327090.1|//1.00E-32//cytochrome P450 [Populus trichocarpa] >gi|222835405|gb|EEE73840.1| cytochrome P450 [Populus trichocarpa]	Unigene10357_All//6.00E-26//AT4G31940| CYP82C4; electron carrier/ heme binding / iron ion binding / monooxygenase/ oxygen binding	Unigene10357_All//3.00E-33//TC132245		ko00945|Stilbenoid, diarylheptanoid and gingerol biosynthesis//ko00903|Limonene and pinene degradation//ko00941|Flavonoid biosynthesis//ko00944|Flavone and flavonol biosynthesis	GO:0005506//GO:0016491	nr -
Unigene10358_All	631	0.17	2.25	0.00	0.71	1.75	3.44	0.87	2.25	1.01	3.76E+00	3.98E-06	Up	-7.37E+00	4.00E-01		1.30E+00	5.46E-02		2.27E+00	5.25E-06	Up	1.37E+00	7.78E-03		2.14E-01	7.73E-01		Unigene10358_All//gi|124359162|gb|ABN05685.1|//6.00E-08//RNA-directed DNA polymerase (Reverse transcriptase) [Medicago truncatula]		Unigene10358_All//1.00E-20//ES811125			GO:0005488	nr -
Unigene103595_All	326	22.63	6.68	17.48	9.32	9.72	9.28	33.72	17.44	10.14	-1.76E+00	2.80E-13	Down	-3.73E-01	1.08E-01		6.01E-02	8.86E-01		-6.10E-03	9.82E-01		-9.52E-01	1.14E-09		-1.73E+00	5.91E-24	Down	Unigene103595_All//gi|297807807|ref|XP_002871787.1|//7.00E-12//cobalamin-independent methionine synthase [Arabidopsis lyrata subsp. lyrata] >gi|297317624|gb|EFH48046.1| cobalamin-independent methionine synthase [Arabidopsis lyrata subsp. lyrata]		Unigene103595_All//4.00E-47//EX163984		ko00270|Cysteine and methionine metabolism	GO:0000097//GO:0005576//GO:0006970//GO:0008172//GO:0009526//GO:0009532//GO:0010038//GO:0016020//GO:0042579//GO:0046914	nr -
Unigene1036_All	492	1.81	3.60	0.22	1.72	0.42	4.31	4.38	11.72	16.36	9.96E-01	4.45E-02		-3.06E+00	2.25E-03		-2.04E+00	9.05E-03		1.33E+00	3.50E-03		1.42E+00	1.45E-09	Up	1.90E+00	8.59E-20	Up	Unigene1036_All//gi|4263517|gb|AAD15343.1|//3.00E-30//similar to PHZF, catalyzing the hydroxylation of phenazine-1-carboxylic acid to 2-hydroxy-phenazine-1-carboxylic acid [Arabidopsis thaliana] >gi|51972158|gb|AAU15183.1| At4g02860 [Arabidopsis thaliana]	Unigene1036_All//2.00E-31//AT4G02860| catalytic	Unigene1036_All//0.00E+00//TC131623			GO:0008152	nr +
Unigene10361_All	601	0.52	0.51	2.66	0.00	0.57	0.98	0.00	0.00	0.00	-4.60E-02	9.85E-01		2.35E+00	2.09E-04	Up	9.16E+00	3.02E-02		9.93E+00	1.37E-03		null	null		null	null		Unigene10361_All//gi|194476845|ref|YP_002049024.1|//5.00E-14//DnaB helicase:RecA bacterial DNA recombination protein [Paulinella chromatophora] >gi|171191852|gb|ACB42814.1| DnaB helicase:RecA bacterial DNA recombination protein [Paulinella chromatophora]				ko03040|Spliceosome		nr -
Unigene103677_All	1075	0.00	0.00	0.00	0.14	0.13	0.08	0.71	0.00	0.04	null	null		null	null		-1.18E-01	9.12E-01		-7.27E-01	7.17E-01		-9.47E+00	1.68E-05	Down	-4.25E+00	8.66E-05	Down	Unigene103677_All//gi|600111|emb|CAA84378.1|//1.00E-117//cysteine proteinase [Vicia sativa]	Unigene103677_All//2.00E-127//AT5G50260| cysteine proteinase, putative	Unigene103677_All//0.00E+00//TC172692				nr +
Unigene103690_All	214	3.18	2.37	0.00	5.59	0.64	3.80	2.57	1.95	4.28	-4.25E-01	5.97E-01		-1.16E+01	7.65E-04	Down	-3.12E+00	1.54E-05	Down	-5.56E-01	3.87E-01		-3.95E-01	6.34E-01		7.37E-01	2.43E-01								
Unigene1037_All	1388	7.12	11.32	8.63	11.20	8.34	26.52	57.08	102.77	76.81	6.68E-01	2.17E-06		2.77E-01	1.25E-01		-4.26E-01	1.43E-03		1.24E+00	1.12E-40	Up	8.48E-01	2.18E-95		4.28E-01	3.17E-22		Unigene1037_All//gi|4263517|gb|AAD15343.1|//1.00E-89//similar to PHZF, catalyzing the hydroxylation of phenazine-1-carboxylic acid to 2-hydroxy-phenazine-1-carboxylic acid [Arabidopsis thaliana] >gi|51972158|gb|AAU15183.1| At4g02860 [Arabidopsis thaliana]	Unigene1037_All//6.00E-91//AT4G02860| catalytic	Unigene1037_All//0.00E+00//gi|164253821|gb|ES839366.1|ES839366			GO:0008152	nr +
Unigene10371_All	634	0.00	0.08	0.00	8.17	2.28	1.28	0.33	0.86	0.57	6.32E+00	6.23E-01		null	null		-1.84E+00	3.35E-12	Down	-2.67E+00	9.28E-17	Down	1.36E+00	1.44E-01		7.62E-01	5.00E-01		Unigene10371_All//gi|51536456|gb|AAU05466.1|//1.00E-12//At5g44660 [Arabidopsis thaliana] >gi|53828621|gb|AAU94420.1| At5g44660 [Arabidopsis thaliana] >gi|60543337|gb|AAX22266.1| At5g44650 [Arabidopsis thaliana]	Unigene10371_All//5.00E-21//AT4G20190| unknown protein	Unigene10371_All//6.00E-23//gi|73859175|gb|DT461914.1|DT461914				nr +
Unigene103722_All	213	2.21	3.33	1.75	17.07	12.29	2.12	0.40	0.20	0.00	5.91E-01	4.78E-01		-3.37E-01	7.34E-01		-4.75E-01	1.41E-01		-3.01E+00	2.09E-13	Down	-1.02E+00	7.27E-01		-8.63E+00	3.62E-01								
Unigene10374_All	418	2.50	0.00	0.38	9.53	5.52	1.84	1.52	0.00	1.24	-1.13E+01	3.80E-06	Down	-2.71E+00	1.65E-03		-7.89E-01	6.01E-03		-2.38E+00	1.98E-11	Down	-1.06E+01	1.24E-04	Down	-2.92E-01	6.84E-01								
Unigene10375_All	513	0.71	0.79	0.21	1.26	2.28	1.32	4.29	5.21	9.55	1.47E-01	8.98E-01		-1.78E+00	2.34E-01		8.54E-01	1.91E-01		6.51E-02	9.50E-01		2.83E-01	4.46E-01		1.16E+00	2.07E-06	Up	Unigene10375_All//gi|297827079|ref|XP_002881422.1|//8.00E-37//abscisic acid-responsive HVA22 family protein [Arabidopsis lyrata subsp. lyrata] >gi|297327261|gb|EFH57681.1| abscisic acid-responsive HVA22 family protein [Arabidopsis lyrata subsp. lyrata]	Unigene10375_All//5.00E-38//AT2G36020| HVA22J (HVA22-LIKE PROTEIN J)	Unigene10375_All//4.00E-119//ES833630			GO:0004871//GO:0044464	nr +
Unigene10376_All	767	0.96	0.26	0.56	3.83	1.66	1.88	0.72	0.60	0.57	-1.85E+00	4.08E-02		-7.82E-01	3.80E-01		-1.21E+00	4.51E-04	Down	-1.02E+00	4.81E-03		-2.58E-01	7.60E-01		-3.27E-01	6.72E-01		Unigene10376_All//gi|38346596|emb|CAE04660.2|//2.00E-06//OSJNBa0061G20.16 [Oryza sativa (japonica cultivar-group)]						nr +
Unigene103773_All	1190	0.00	0.38	0.98	0.21	0.58	10.82	227.00	95.67	504.91	8.58E+00	7.45E-03		9.94E+00	1.77E-06	Up	1.47E+00	1.16E-01		5.69E+00	4.11E-70	Up	-1.25E+00	0.00E+00	Down	1.15E+00	0.00E+00	Up	Unigene103773_All//gi|124365601|gb|AAK50778.4|AF361893_1//1.00E-146//bacterial-induced lipoxygenase [Gossypium hirsutum]	Unigene103773_All//3.00E-127//AT3G22400| LOX5; electron carrier/ iron ion binding / lipoxygenase/ metal ion binding / oxidoreductase, acting on single donors with incorporation of molecular oxygen, incorporation of two atoms of oxygen	Unigene103773_All//0.00E+00//gi|164244850|gb|ES799168.1|ES799168		ko00592|alpha-Linolenic acid metabolism//ko00591|Linoleic acid metabolism		nr +
Unigene1038_All	1132	7.30	5.86	8.42	7.57	19.89	10.73	1.12	0.78	1.06	-3.17E-01	1.30E-01		2.05E-01	3.34E-01		1.39E+00	1.56E-32	Up	5.04E-01	1.74E-03		-5.31E-01	3.43E-01		-8.58E-02	8.72E-01		Unigene1038_All//gi|269980442|gb|ACZ56416.1|//1.00E-134//ankyrin-like protein [Vitis vinifera]	Unigene1038_All//6.00E-120//AT3G01750| ankyrin repeat family protein	Unigene1038_All//0.00E+00//gi|78354540|gb|DT574814.1|DT574814				nr +
Unigene103802_All	367	0.00	0.55	2.47	5.84	4.41	3.08	1.15	2.16	0.76	9.11E+00	1.37E-01		1.13E+01	4.71E-05	Up	-4.04E-01	3.75E-01		-9.24E-01	3.62E-02		9.09E-01	2.11E-01		-6.00E-01	5.53E-01				Unigene103802_All//6.00E-77//gi|109890213|gb|DW519177.1|DW519177				
Unigene10382_All	346	6.65	2.20	2.15	0.43	0.60	0.39	0.00	0.00	0.00	-1.60E+00	3.45E-04	Down	-1.63E+00	5.02E-04	Down	4.67E-01	8.26E-01		-1.41E-01	9.44E-01		null	null		null	null				Unigene10382_All//1.00E-07//DT048782				
Unigene103835_All	298	0.00	0.00	2.14	0.00	0.00	3.33	0.00	0.00	0.00	null	null		1.11E+01	1.16E-03		null	null		1.17E+01	6.15E-06	Up	null	null		null	null		Unigene103835_All//gi|124359981|gb|ABN07997.1|//1.00E-08//contains similarity to reverse trancriptase , related [Medicago truncatula]		Unigene103835_All//3.00E-07//gi|78324173|gb|DT544447.1|DT544447				nr +
Unigene103839_All	227	2.77	0.00	0.00	0.44	0.00	0.60	0.37	1.10	0.18	-1.14E+01	7.92E-04	Down	-1.14E+01	1.41E-03		-8.78E+00	3.18E-01		4.44E-01	8.63E-01		1.57E+00	3.27E-01		-1.09E+00	6.78E-01								
Unigene10385_All	655	0.00	0.00	0.00	0.00	0.00	0.00	5.62	1.08	0.97	null	null		null	null		null	null		null	null		-2.37E+00	5.08E-12	Down	-2.53E+00	2.37E-13	Down							ESTscan -
Unigene103855_All	317	0.33	4.00	0.17	1.26	1.52	2.28	0.13	0.13	0.00	3.60E+00	2.27E-05	Up	-9.74E-01	7.23E-01		2.75E-01	8.20E-01		8.59E-01	3.42E-01		-1.74E-02	1.01E+00		-7.06E+00	6.19E-01		Unigene103855_All//gi|115476212|ref|NP_001061702.1|//1.00E-48//Os08g0384500 [Oryza sativa Japonica Group] >gi|113623671|dbj|BAF23616.1| Os08g0384500 [Oryza sativa Japonica Group]	Unigene103855_All//5.00E-47//AT1G15520| PDR12 (PLEIOTROPIC DRUG RESISTANCE 12); ATPase, coupled to transmembrane movement of substances	Unigene103855_All//4.00E-59//TC147767		ko03040|Spliceosome//ko02010|ABC transporters	GO:0032559//GO:0042626//GO:0043231	nr +
Unigene10386_All	407	0.00	6.85	4.19	0.00	0.00	0.00	0.00	0.00	0.00	1.27E+01	7.44E-16	Up	1.20E+01	2.20E-09	Up	null	null		null	null		null	null		null	null		Unigene10386_All//sp|P17275|JUNB_HUMAN//6.00E-26//Transcription factor jun-B OS=Homo sapiens GN=JUNB PE=1 SV=1						swissprot +
Unigene10387_All	422	1.36	0.24	2.02	2.12	1.14	0.86	4.01	1.09	3.40	-2.51E+00	2.94E-02		5.66E-01	4.61E-01		-8.95E-01	2.14E-01		-1.31E+00	8.52E-02		-1.88E+00	1.33E-04	Down	-2.38E-01	5.94E-01		Unigene10387_All//gi|21901929|emb|CAC86383.1|//2.00E-11//carboxypeptidase type III [Theobroma cacao]	Unigene10387_All//2.00E-08//AT3G10410| scpl49 (serine carboxypeptidase-like 49); serine-type carboxypeptidase	Unigene10387_All//2.00E-46//gi|11206118|gb|BF275048.1|BF275048		ko00940|Phenylpropanoid biosynthesis	GO:0004180//GO:0019538//GO:0043231	nr -
Unigene10388_All	417	0.00	4.01	0.51	2.51	3.80	3.79	0.00	1.10	0.00	1.20E+01	1.54E-09	Up	9.00E+00	1.42E-01		5.99E-01	2.87E-01		5.96E-01	2.89E-01		1.01E+01	1.94E-03		null	null		Unigene10388_All//gi|255562741|ref|XP_002522376.1|//8.00E-08//conserved hypothetical protein [Ricinus communis] >gi|223538454|gb|EEF40060.1| conserved hypothetical protein [Ricinus communis]		Unigene10388_All//5.00E-16//TC168014				nr +
Unigene10390_All	676	0.31	0.00	0.00	0.81	1.02	0.00	2.13	0.19	0.77	-8.27E+00	1.23E-01		-8.27E+00	1.47E-01		3.30E-01	7.52E-01		-9.66E+00	1.52E-03		-3.52E+00	3.19E-07	Down	-1.47E+00	3.09E-03		Unigene10390_All//gi|15224831|ref|NP_181947.1|//3.00E-40//EDE1 (ENDOSPERM DEFECTIVE 1); microtubule binding [Arabidopsis thaliana] >gi|63003848|gb|AAY25453.1| At2g44190 [Arabidopsis thaliana]	Unigene10390_All//1.00E-40//AT2G44190| EDE1 (ENDOSPERM DEFECTIVE 1); microtubule binding	Unigene10390_All//0.00E+00//TC170349			GO:0003006//GO:0005874//GO:0007349//GO:0009791//GO:0009987//GO:0015631//GO:0043231	nr +
Unigene10391_All	528	0.00	125.91	50.33	0.00	0.13	0.00	0.00	0.00	0.00	1.69E+01	0.00E+00	Up	1.56E+01	1.70E-150	Up	7.03E+00	6.17E-01		null	null		null	null		null	null		Unigene10391_All//gi|223995201|ref|XP_002287284.1|//2.00E-16//RL14, ribosomal protein 14 60S large ribosomal subunit [Thalassiosira pseudonana CCMP1335] >gi|220976400|gb|EED94727.1| RL14, ribosomal protein 14 60S large ribosomal subunit [Thalassiosira pseudonana CCMP1335]	Unigene10391_All//6.00E-12//AT2G20450| 60S ribosomal protein L14 (RPL14A)			ko03010|Ribosome	GO:0044424	nr -
Unigene103930_All	395	0.00	3.98	0.13	0.13	0.26	0.11	4.07	3.39	4.24	1.20E+01	5.71E-09	Up	7.07E+00	6.42E-01		1.05E+00	8.10E-01		-1.42E-01	9.57E-01		-2.65E-01	6.15E-01		5.86E-02	9.12E-01		Unigene103930_All//gi|225436241|ref|XP_002275171.1|//8.00E-16//PREDICTED: hypothetical protein [Vitis vinifera]		Unigene103930_All//6.00E-176//gi|84174516|gb|DR463164.1|DR463164			GO:0000166	nr +
Unigene10395_All	867	0.48	16.71	0.43	0.23	1.39	0.21	0.15	0.00	0.18	5.11E+00	1.26E-70	Up	-1.67E-01	8.79E-01		2.60E+00	3.41E-04	Up	-1.41E-01	9.36E-01		-7.19E+00	2.37E-01		3.29E-01	8.52E-01		Unigene10395_All//gi|224069539|ref|XP_002326368.1|//3.00E-27//SAUR family protein [Populus trichocarpa] >gi|222833561|gb|EEE72038.1| SAUR family protein [Populus trichocarpa]	Unigene10395_All//3.00E-20//AT3G09870| auxin-responsive family protein	Unigene10395_All//0.00E+00//TC150348				nr -
Unigene103975_All	362	0.00	0.00	0.00	0.00	0.10	0.00	3.97	1.04	0.11	null	null		null	null		6.57E+00	8.40E-01		null	null		-1.93E+00	3.78E-04	Down	-5.17E+00	2.09E-09	Down	Unigene103975_All//gi|18396925|ref|NP_566232.1|//1.00E-21//DNA-binding protein-related [Arabidopsis thaliana] >gi|29028876|gb|AA064817.1| At3g04570 [Arabidopsis thaliana] >gi|119657382|tpd|FAA00290.1| TPA: AT-hook motif nuclear localized protein 19 [Arabidopsis thaliana]	Unigene103975_All//1.00E-07//AT3G04570| DNA-binding protein-related	Unigene103975_All//3.00E-57//DW502620				nr -
Unigene103977_All	503	0.10	1.01	2.12	0.59	0.89	0.99	0.00	0.00	0.08	3.28E+00	2.11E-02		4.35E+00	6.46E-05	Up	5.83E-01	6.26E-01		7.33E-01	5.16E-01		null	null		6.31E+00	6.49E-01		Unigene103977_All//gi|270342124|gb|ACZ74707.1|//2.00E-44//carbonic anhydrase [Phaseolus vulgaris]	Unigene103977_All//4.00E-44//AT5G14740| CA2 (CARBONIC ANHYDRASE 2); carbonate dehydratase/ zinc ion binding	Unigene103977_All//4.00E-162//TC141146		ko00910|Nitrogen metabolism		nr -

Unigene10398_All	1740	0.00	0.00	0.00	0.06	0.00	0.00	1.26	1.56	5.84	null	null		null	null		-5.84E+00	3.18E-01		-5.84E+00	4.06E-01		3.05E-01	3.98E-01		2.21E+00	6.82E-30	Up	Unigene10398_All//gi|115485949|ref|NP_001068118.1|//1.00E-104//Os11g0569600 [Oryza sativa Japonica Group] >gi|113645340|dbj|BAF28481.1| Os11g0569600 [Oryza sativa Japonica Group]	Unigene10398_All//2.00E-92//AT3G47570| leucine-rich repeat transmembrane protein kinase, putative	Unigene10398_All//4.00E-23//TC151383		ko04626|Plant-pathogen interaction		nr +
Unigene10399_All	542	0.00	0.00	0.00	0.00	0.00	0.00	4.91	6.40	0.00	null	null		null	null		null	null		null	null		3.81E-01	2.24E-01		-1.23E+01	1.34E-19	Down	Unigene10399_All//gi|195618110|gb|ACG30885.1|//2.00E-27//translationally-controlled tumor protein [Zea mays] >gi|195618316|gb|ACG30988.1| translationally-controlled tumor protein [Zea mays]	Unigene10399_All//7.00E-28//AT3G05540| INVOLVED IN: biological_process unknown; LOCATED IN: cytoplasm; CONTAINS InterPro DOMAIN/s: Translationally controlled tumour protein (InterPro:IPR018105), Translationally controlled tumour-associated TCTP (InterPro:IPR001983), Mss4/translationally controlled tumour-associated TCTP (InterPro:IPR011323), Translationally controlled tumour protein, conserved site (InterPro:IPR018103), Mss4-like (InterPro:IPR011057); BEST Arabidopsis thaliana protein match is: TCTP (TRANSLATIONALLY CONTROLLED TUMOR PROTEIN) (TAIR:AT3G16640.1); Has 640 Blast hits to 640 proteins in 231 species: Archae - 0; Bacteria - 0; Metazoa - 343; Fungi - 110; Plants - 105; Viruses - 0; Other Eukaryotes - 82 (source: NCBI BLink).				GO:0000904//GO:0005488//GO:0005576//GO:0006952//GO:0009536//GO:0010038//GO:0016020//GO:0016049//GO:0048528//GO:0048878	nr +
Unigene103996_All	204	0.26	1.24	0.00	0.49	0.84	2.88	5.18	1.84	0.00	2.28E+00	2.34E-01		-8.00E+00	6.34E-01		7.89E-01	7.72E-01		2.56E+00	2.87E-02		-1.49E+00	1.64E-02		-1.23E+01	6.73E-08	Down	Unigene103996_All//gi|46396756|sp|P93194.2|RPK1_IPONI//1.00E-11//RecName: Full=Receptor-like protein kinase; Flags: Precursor >gi|14495542|gb|AAB36558.2| receptor-like protein kinase INRPK1 [Ipomoea nil]	Unigene103996_All//3.00E-11//AT1G73066| protein binding			ko04626|Plant-pathogen interaction		nr +
Unigene103999_All	272	74.25	16.95	61.28	20.51	28.99	14.61	1.40	0.92	0.44	-2.13E+00	3.48E-45	Down	-2.77E-01	3.66E-02		4.99E-01	1.36E-02		-4.89E-01	5.67E-02		-6.02E-01	6.13E-01		-1.67E+00	1.41E-01		Unigene103999_All//gi|229893912|gb|ACQ90301.1|//1.00E-34//squalene epoxidase [Gynostemma pentaphyllum]	Unigene103999_All//4.00E-33//AT4G37760| SQE3 (squalene epoxidase 3); squalene monooxygenase	Unigene103999_All//6.00E-101//gi|48795641|gb|CO096955.1|CO096955		ko00100|Steroid biosynthesis		nr +
Unigene1040_All	2495	20.07	6.30	11.01	37.52	33.66	10.28	21.11	8.74	8.69	-1.67E+00	2.98E-80	Down	-8.66E-01	9.17E-28		-1.56E-01	3.07E-03		-1.87E+00	6.92E-190	Down	-1.27E+00	1.10E-68	Down	-1.28E+00	1.70E-71	Down	Unigene1040_All//gi|269980442|gb|ACZ56416.1|//0.00E+00//ankyrin-like protein [Vitis vinifera]	Unigene1040_All//0.00E+00//AT3G01750| ankyrin repeat family protein	Unigene1040_All//0.00E+00//TC151204				nr +
Unigene10400_All	551	0.38	1.10	0.10	4.34	3.87	0.25	0.15	0.83	1.23	1.54E+00	1.22E-01		-1.98E+00	3.82E-01		-1.63E-01	7.23E-01		-4.14E+00	1.49E-11	Down	2.44E+00	3.75E-02		3.00E+00	2.00E-03		Unigene10400_All//gi|15233416|ref|NP_193816.1|//9.00E-32//FAD-binding domain-containing protein [Arabidopsis thaliana] >gi|5262224|emb|CAB45850.1| reticuline oxidase-like protein [Arabidopsis thaliana] >gi|7268880|emb|CAB79084.1| reticuline oxidase-like protein [Arabidopsis thaliana] >gi|222424693|dbj|BAH20300.1| AT4G20840 [Arabidopsis thaliana]	Unigene10400_All//4.00E-41//AT4G20840| FAD-binding domain-containing protein	Unigene10400_All//0.00E+00//TC153608			GO:0005576//GO:0005618//GO:0006950//GO:0016020//GO:0043231	nr -
Unigene10401_All	349	0.00	2.61	1.07	0.00	0.00	0.00	0.00	0.00	0.00	1.14E+01	2.62E-05	Up	1.01E+01	2.51E-02		null	null		null	null		null	null		null	null		Unigene10401_All//sp|P13395|SPTCA_DROME//6.00E-61//Spectrin alpha chain OS=Drosophila melanogaster GN=alpha-Spec PE=1 SV=2	Unigene10401_All//6.00E-05//AT5G37780| CAM1 (CALMODULIN 1); calcium ion binding			ko04626|Plant-pathogen interaction		swissprot -
Unigene10405_All	618	0.25	0.33	2.84	0.00	0.67	0.07	0.00	0.00	0.00	3.69E-01	8.17E-01		3.48E+00	8.87E-07	Up	9.39E+00	1.25E-02		6.19E+00	7.04E-01		null	null		null	null		Unigene10405_All//gi|168069625|ref|XP_001786518.1|//1.00E-15//sensory histidine protein kinase [Physcomitrella patens subsp. patens] >gi|162661105|gb|EDQ48669.1| sensory histidine protein kinase [Physcomitrella patens subsp. patens]	Unigene10405_All//4.00E-09//AT5G10720| AHK5 (ARABIDOPSIS HISTIDINE KINASE 5); protein histidine kinase		Orphans		GO:0016301//GO:0044237//GO:0044238	nr -
Unigene104056_All	549	0.00	0.46	4.75	0.00	0.00	1.15	0.00	0.00	0.00	8.85E+00	7.89E-02		1.22E+01	2.12E-14	Up	null	null		1.02E+01	7.60E-04	Up	null	null		null	null		Unigene104056_All//gi|56900734|gb|AAW31667.1|//4.00E-78//ribulose-1,5-bisphosphate carboxylase/oxygenase small subunit [Ammopiptanthus mongolicus]	Unigene104056_All//4.00E-77//AT5G38430| ribulose bisphosphate carboxylase small chain 1B / RuBisCO small subunit 1B (RBCS-1B) (ATS1B)	Unigene104056_All//1.00E-20//gi|109886204|gb|DW515174.1|DW515174		ko00710|Carbon fixation in photosynthetic organisms//ko00630|Glyoxylate and dicarboxylate metabolism		nr +
Unigene10406_All	823	0.13	132.18	103.14	0.36	0.54	0.66	0.36	0.10	0.48	1.00E+01	0.00E+00	Up	9.66E+00	0.00E+00	Up	5.83E-01	6.26E-01		8.58E-01	4.23E-01		-1.82E+00	2.11E-01		4.29E-01	6.62E-01		Unigene10406_All//gi|150406463|ref|YP_001315116.1|//7.00E-56//cytochrome c oxidase subunit 2 [Chlorokybus atmophyticus] >gi|126507700|gb|ABO15097.1| cytochrome c oxidase subunit 2 [Chlorokybus atmophyticus]	Unigene10406_All//4.00E-35//ATMG00160| cytochrome c oxidase subunit 2			ko00190|Oxidative phosphorylation	GO:0016020//GO:0046872//GO:0055114	nr -
Unigene104075_All	790	29.21	19.05	21.70	14.69	11.51	6.92	2.03	1.53	0.66	-6.17E-01	6.10E-08		-4.28E-01	2.90E-04		-3.53E-01	3.16E-02		-1.09E+00	6.70E-11	Down	-4.07E-01	3.95E-01		-1.63E+00	6.02E-04	Down	Unigene104075_All//gi|297727877|ref|NP_001176302.1|//6.00E-10//Os11g0106066 [Oryza sativa Japonica Group] >gi|255679693|dbj|BAH95030.1| Os11g0106066 [Oryza sativa Japonica Group]		Unigene104075_All//1.00E-46//ES795234				nr -
Unigene10408_All	824	0.00	15.86	4.85	0.00	0.00	0.00	0.00	0.00	0.00	1.40E+01	5.01E-75	Up	1.22E+01	3.83E-22	Up	null	null		null	null		null	null		null	null		Unigene10408_All//gi|255081672|ref|XP_002508058.1|//8.00E-71//peptidylprolyl isomerase, precursor [Micromonas sp. RCC299] >gi|226523334|gb|ACO69316.1| peptidylprolyl isomerase, precursor [Micromonas sp. RCC299]	Unigene10408_All//6.00E-50//AT5G13120| peptidyl-prolyl cis-trans isomerase cyclophilin-type family protein				GO:0016853//GO:0031410//GO:0043231	nr -
Unigene104081_All	268	0.00	0.00	0.00	1.12	1.03	9.61	0.95	3.28	1.19	null	null		null	null		-1.18E-01	9.01E-01		3.11E+00	1.37E-09	Up	1.79E+00	1.27E-02		3.29E-01	7.64E-01		Unigene104081_All//gi|22329131|ref|NP_195096.2|//5.00E-18//CBS domain-containing protein [Arabidopsis thaliana] >gi|17381276|gb|AAL36056.1| AT4g33700/T16L1_190 [Arabidopsis thaliana] >gi|20856058|gb|AAM26645.1| AT4g33700/T16L1_190 [Arabidopsis thaliana]	Unigene104081_All//2.00E-19//AT4G33700| CBS domain-containing protein	Unigene104081_All//7.00E-42//TC133787				nr +
Unigene104087_All	216	1.45	10.56	1.48	1.15	2.23	1.67	10.96	12.19	49.25	2.86E+00	1.28E-07	Up	2.54E-02	1.00E+00		9.53E-01	4.24E-01		5.37E-01	6.95E-01		1.53E-01	6.76E-01		2.17E+00	1.44E-30	Up			Unigene104087_All//2.00E-63//TC139645				
Unigene10409_All	278	0.00	15.31	4.02	0.00	0.00	0.00	0.00	0.00	0.00	1.39E+01	3.04E-24	Up	1.20E+01	3.44E-06	Up	null	null		null	null		null	null		null	null								
Unigene104096_All	237	0.44	7.06	0.22	0.00	0.00	1.14	0.00	0.00	0.00	4.00E+00	2.08E-07	Up	-9.74E-01	7.23E-01		null	null		1.02E+01	7.06E-02		null	null		null	null				Unigene104096_All//3.00E-19//FL576574				
Unigene1041_All	2453	10.69	7.06	6.58	6.58	14.35	10.11	0.43	1.01	0.96	-5.97E-01	1.85E-08		-7.00E-01	1.84E-10		1.12E+00	1.75E-36	Up	6.19E-01	6.17E-09		1.22E+00	9.01E-04	Up	1.15E+00	1.66E-03		Unigene1041_All//gi|269980442|gb|ACZ56416.1|//0.00E+00//ankyrin-like protein [Vitis vinifera]	Unigene1041_All//8.00E-180//AT3G01750| ankyrin repeat family protein	Unigene1041_All//0.00E+00//TC151607				nr -
Unigene10411_All	578	2.99	0.79	3.41	0.95	0.71	0.47	1.02	1.16	0.14	-1.92E+00	4.67E-04	Down	1.90E-01	6.77E-01		-4.07E-01	6.58E-01		-1.02E+00	3.30E-01		1.76E-01	8.15E-01		-2.89E+00	4.54E-03								
Unigene10412_All	384	0.00	7.26	2.36	0.00	0.00	0.00	0.00	0.00	0.00	1.28E+01	7.43E-16	Up	1.12E+01	4.71E-05	Up	null	null		null	null		null	null		null	null		Unigene10412_All//sp|Q69566|U88_HHV6U//1.00E-09//Uncharacterized protein U88 OS=Human herpesvirus 6A (strain Uganda-1102) GN=U88 PE=4 SV=1						swissprot -
Unigene10414_All	741	0.00	0.00	0.00	0.40	0.28	0.24	7.70	2.03	1.29	null	null		null	null		-5.33E-01	6.79E-01		-7.27E-01	6.39E-01		-1.92E+00	3.41E-14	Down	-2.58E+00	9.98E-21	Down			Unigene10414_All//2.00E-13//TC166793				
Unigene10415_All	564	0.00	0.00	0.00	0.00	0.00	0.00	1.80	5.48	0.00	null	null		null	null		null	null		null	null		1.61E+00	2.00E-06	Up	-1.08E+01	1.35E-07	Down							
Unigene10418_All	385	10.60	11.84	13.42	26.27	18.60	26.16	322.98	424.80	678.29	1.60E-01	6.18E-01		3.40E-01	2.33E-01		-4.98E-01	3.42E-03		-5.80E-03	9.71E-01		3.95E-01	2.21E-28		1.07E+00	7.23E-262	Up			Unigene10418_All//1.00E-152//gi|164253602|gb|ES824425.1|ES824425				
Unigene104194_All	325	27.05	2.65	4.26	28.36	11.87	10.56	18.34	1.80	0.74	-3.35E+00	1.86E-32	Down	-2.67E+00	7.87E-25	Down	-1.26E+00	2.93E-12	Down	-1.42E+00	4.12E-13	Down	-3.35E+00	4.94E-27	Down	-4.64E+00	1.63E-35	Down	Unigene104194_All//gi|15236175|ref|NP_194362.1|//2.00E-07//AGP13 (arabinogalactan protein 13) [Arabidopsis thaliana] >gi|75207711|sp|Q9STQ3.1|AGP13_ARATH RecName: Full=Arabinogalactan peptide 13; Short=AG-peptide 13; Flags: Precursor >gi|10880503|gb|AAG24281.1|AF195894_1 arabinogalactan protein [Arabidopsis thaliana] >gi|89111816|gb|ABD60680.1| At4g26320 [Arabidopsis thaliana]		Unigene104194_All//5.00E-71//gi|21094474|gb|BQ406799.1|BQ406799				nr -
Unigene104197_All	334	0.00	0.15	0.00	0.30	0.41	0.27	6.46	46.30	1.79	7.25E+00	6.23E-01		null	null		4.67E-01	8.78E-01		-1.42E-01	9.53E-01		2.84E+00	7.50E-59	Up	-1.85E+00	8.47E-06	Down	Unigene104197_All//gi|15220954|ref|NP_174363.1|//1.00E-32//FAD-binding domain-containing protein [Arabidopsis thaliana] >gi|16323167|gb|AAL15318.1| At1g30760/T5I8_22 [Arabidopsis thaliana] >gi|28416457|gb|AAO42759.1| At1g30760/T5I8_22 [Arabidopsis thaliana]	Unigene104197_All//5.00E-34//AT1G30760| FAD-binding domain-containing protein	Unigene104197_All//6.00E-09//TC166754				nr +
Unigene1042_All	3284	6.21	62.55	13.18	8.72	35.65	50.99	29.21	161.88	260.77	3.33E+00	0.00E+00	Up	1.09E+00	7.01E-35	Up	2.03E+00	5.48E-285	Up	2.55E+00	0.00E+00	Up	2.47E+00	0.00E+00	Up	3.16E+00	0.00E+00	Up	Unigene1042_All//gi|15224213|ref|NP_179460.1|//0.00E+00//ATTPS11; transferase, transferring glycosyl groups [Arabidopsis thaliana] >gi|75216907|sp|Q9ZV48.1|TPS11_ARATH RecName: Full=Probable alpha,alpha-trehalose-phosphate synthase [UDP-forming] 11; AltName: Full=Trehalose-6-phosphate synthase 11; Short=AtTPS11	Unigene1042_All//0.00E+00//AT2G18700| ATTPS11; transferase, transferring glycosyl groups	Unigene1042_All//0.00E+00//TC176400		ko00500|Starch and sucrose metabolism	GO:0005991//GO:0019203//GO:0035251//GO:0043231	nr +

Unigene10420_All	671	0.00	0.00	0.00	0.00	0.00	0.00	1.76	4.98	0.00	null	null		null	null		null	null		null	null		1.50E+00	2.73E-06	Up	-1.08E+01	8.15E-09	Down	Unigene10420_All//gi|115469274|ref|NP_001058236.1|//3.00E-19//Os06g0652600 [Oryza sativa Japonica Group] >gi|51535038|dbj|BAD37409.1| fibroin heavy chain precursor-like [Oryza sativa Japonica Group] >gi|113596276|dbj|BAF20150.1| Os06g0652600 [Oryza sativa Japonica Group]				ko03040|Spliceosome//ko03018|RNA degradation	GO:0044464	nr +
Unigene104210_All	261	3.61	2.14	2.04	7.44	2.37	4.50	1.94	1.76	0.61	-7.57E-01	2.86E-01		-8.23E-01	2.71E-01		-1.65E+00	2.73E-04	Down	-7.26E-01	1.27E-01		-1.42E-01	8.76E-01		-1.67E+00	7.78E-02				Unigene104210_All//1.00E-74//DW498863				
Unigene10422_All	393	0.27	0.52	0.00	3.80	3.86	1.38	2.15	0.21	0.30	9.54E-01	6.23E-01		-8.06E+00	4.00E-01		1.99E-02	9.85E-01		-1.46E+00	8.90E-03		-3.34E+00	2.52E-04	Down	-2.82E+00	5.38E-04	Down	Unigene10422_All//gi|124360202|gb|ABN08215.1|//1.00E-67//Zinc finger, CCCH-type [Medicago truncatula]	Unigene10422_All//6.00E-68//AT2G41900| zinc finger (CCCH-type) family protein	Unigene10422_All//4.00E-32//TC132093	C3H		GO:0001071//GO:0010468//GO:0044424//GO:0046914	nr +
Unigene104239_All	267	3.33	10.44	7.18	13.99	13.93	11.16	9.03	7.04	10.30	1.65E+00	4.82E-05	Up	1.11E+00	2.53E-02		-6.60E-03	9.73E-01		-3.26E-01	3.52E-01		-3.58E-01	3.67E-01		1.90E-01	6.15E-01				Unigene104239_All//8.00E-88//gi|13243815|gb|BE053182.2|BE053182				
Unigene10425_All	734	0.00	5.11	1.45	0.00	0.00	0.00	0.00	0.00	0.00	1.23E+01	2.40E-21	Up	1.05E+01	6.63E-06	Up	null	null		null	null		null	null		null	null								ESTscan -
Unigene10427_All	438	0.00	0.58	0.24	0.00	0.24	0.21	0.19	2.29	0.18	9.18E+00	7.89E-02		7.93E+00	4.03E-01		7.88E+00	4.59E-01		7.69E+00	4.82E-01		3.57E+00	3.08E-05	Up	-8.62E-02	9.74E-01		Unigene10427_All//gi|267124|sp|P29449.1|TRXH1_TOBAC//2.00E-06//RecName: Full=Thioredoxin H-type 1; Short=Trx-H1 >gi|20047|emb|CAA41415.1| thioredoxin [Nicotiana tabacum]		Unigene10427_All//3.00E-33//EX167848				nr -
Unigene104270_All	402	0.00	0.00	0.00	0.00	0.09	4.27	0.00	0.00	0.00	null	null		null	null		6.42E+00	8.40E-01		1.21E+01	3.12E-10	Up	null	null		null	null		Unigene104270_All//gi|146160694|gb|ABQ08576.1|//3.00E-06//alpha-gliadin [Australopyrum retrofractum]				ko00760|Nicotinate and nicotinamide metabolism		nr -
Unigene10428_All	337	0.00	9.02	0.00	0.59	1.23	1.88	0.00	0.00	1.30	1.31E+01	2.68E-17	Up	null	null		1.05E+00	4.26E-01		1.67E+00	9.80E-02		null	null		1.03E+01	2.14E-03		Unigene10428_All//gi|2245113|emb|CAB10535.1|//9.00E-15//glycerol-3-phosphate permease like protein [Arabidopsis thaliana] >gi|7268507|emb|CAB78758.1| glycerol-3-phosphate permease like protein [Arabidopsis thaliana]	Unigene10428_All//4.00E-16//AT4G17550| transporter-related				GO:0006810//GO:0009987//GO:0043231	nr +
Unigene10429_All	980	0.11	0.31	0.33	0.81	0.70	0.23	4.27	4.65	1.22	1.54E+00	3.24E-01		1.61E+00	3.05E-01		-2.11E-01	8.06E-01		-1.82E+00	3.32E-02		1.22E-01	6.54E-01		-1.81E+00	3.56E-10	Down	Unigene10429_All//gi|44888124|sp|P93308.1|M530_ARATH//2.00E-30//RecName: Full=Uncharacterized mitochondrial protein AtMg00530; AltName: Full=ORF109	Unigene10429_All//3.00E-27//ATMG00530| hypothetical protein	Unigene10429_All//0.00E+00//gi|109854544|gb|DW483523.1|DW483523			GO:0043231	nr +
Unigene10430_All	582	0.27	0.09	0.09	0.51	0.12	0.31	1.24	2.37	4.38	-1.63E+00	4.89E-01		-1.56E+00	5.34E-01		-2.12E+00	1.66E-01		-7.26E-01	6.39E-01		9.40E-01	6.71E-02		1.83E+00	1.63E-06	Up			Unigene10430_All//9.00E-25//TC149710				
Unigene10431_All	726	0.00	0.00	0.00	0.00	0.09	0.00	0.82	1.73	5.43	null	null		null	null		6.57E+00	6.16E-01		null	null		1.08E+00	4.70E-02		2.74E+00	3.14E-15	Up	Unigene10431_All//gi|284026888|gb|ADB66335.1|//4.00E-40//CC-NBS-LRR protein [Quercus suber]	Unigene10431_All//4.00E-17//AT3G14460| disease resistance protein (NBS-LRR class), putative					nr +
Unigene104310_All	274	72.56	40.50	49.95	119.99	131.83	166.81	144.89	79.93	64.70	-8.41E-01	2.18E-11		-5.39E-01	2.39E-05		1.36E-01	1.68E-01		4.75E-01	4.07E-10		-8.58E-01	1.11E-27		-1.16E+00	1.52E-46	Down	Unigene104310_All//gi|195620934|gb|ACG32297.1|//6.00E-36//60S ribosomal protein L23 [Zea mays]	Unigene104310_All//1.00E-36//AT3G04400| emb2171 (embryo defective 2171); structural constituent of ribosome	Unigene104310_All//6.00E-135//TC154590		ko03010|Ribosome		nr -
Unigene104330_All	277	5.10	7.87	6.92	20.32	14.92	7.83	3.51	2.87	3.02	6.25E-01	1.50E-01		4.40E-01	3.86E-01		-4.46E-01	7.15E-02		-1.38E+00	7.40E-08	Down	-2.92E-01	6.31E-01		-2.17E-01	7.07E-01				Unigene104330_All//7.00E-141//TC137783				ESTscan -
Unigene104332_All	307	0.68	14.19	3.99	0.49	0.67	6.18	0.28	0.27	1.17	4.38E+00	1.10E-19	Up	2.55E+00	8.25E-04	Up	4.67E-01	8.26E-01		3.67E+00	3.48E-08	Up	-1.63E-02	1.02E+00		2.08E+00	1.01E-01		Unigene104332_All//gi|44681328|gb|AAS47604.1|//5.00E-24//At4g15670 [Arabidopsis thaliana] >gi|45773840|gb|AAS76724.1| At4g15670 [Arabidopsis thaliana]	Unigene104332_All//3.00E-25//AT5G18600| glutaredoxin family protein	Unigene104332_All//4.00E-56//gi|109866735|gb|DW495711.1|DW495711				nr -
Unigene104349_All	239	0.00	0.42	0.00	0.00	0.29	1.70	25.12	55.44	226.70	8.73E+00	3.91E-01		null	null		8.17E+00	6.16E-01		1.07E+01	1.36E-02		1.14E+00	3.57E-15	Up	3.17E+00	8.94E-233	Up	Unigene104349_All//gi|224092400|ref|XP_002309592.1|//5.00E-14//2-oxoglutarate-dependent dioxygenase [Populus trichocarpa] >gi|222855568|gb|EEE93115.1| 2-oxoglutarate-dependent dioxygenase [Populus trichocarpa]	Unigene104349_All//1.00E-13//AT1G52800| oxidoreductase, 2OG-Fe(II) oxygenase family protein	Unigene104349_All//2.00E-82//TC144167				nr -
Unigene10436_All	629	0.00	10.47	8.21	0.00	0.00	0.14	0.00	0.00	0.00	1.34E+01	1.26E-37	Up	1.30E+01	9.62E-29	Up	null	null		7.17E+00	4.82E-01		null	null		null	null		Unigene10436_All//sp|P08727|K1C19_HUMAN//3.00E-90//Keratin, type I cytoskeletal 19 OS=Homo sapiens GN=KRT19 PE=1 SV=3						swissprot -
Unigene104378_All	241	0.22	3.78	0.44	0.83	3.43	0.94	0.35	0.35	0.33	4.12E+00	2.34E-04	Up	1.03E+00	7.05E-01		2.05E+00	1.79E-02		1.81E-01	9.36E-01		-1.65E-02	1.02E+00		-8.60E-02	9.74E-01								
Unigene10439_All	750	0.00	7.30	5.40	0.00	0.00	0.00	0.00	0.00	0.00	1.28E+01	3.25E-31	Up	1.24E+01	1.92E-22	Up	null	null		null	null		null	null		null	null		Unigene10439_All//gi|300147461|gb|EFJ14125.1|//3.00E-06//hypothetical protein SELMODRAFT_446044 [Selaginella moellendorffii]	Unigene10439_All//6.00E-06//AT1G56040| binding / protein binding / ubiquitin-protein ligase					nr +
Unigene104391_All	280	0.75	0.00	0.76	0.00	0.74	2.90	0.45	0.00	0.28	-9.55E+00	1.23E-01		2.54E-02	1.00E+00		9.53E+00	1.57E-01		1.15E+01	6.96E-05	Up	-8.82E+00	2.37E-01		-6.71E-01	7.21E-01		Unigene104391_All//gi|297842799|ref|XP_002889281.1|//4.00E-23//kinase family protein [Arabidopsis lyrata subsp. lyrata] >gi|297335122|gb|EFH65540.1| kinase family protein [Arabidopsis lyrata subsp. lyrata]	Unigene104391_All//3.00E-24//AT1G80640| protein kinase family protein	Unigene104391_All//4.00E-96//TC158373		ko04626|Plant-pathogen interaction		nr -
Unigene10441_All	835	0.00	48.18	12.88	0.00	0.00	0.00	0.00	0.00	0.00	1.56E+01	3.15E-232	Up	1.37E+01	2.06E-60	Up	null	null		null	null		null	null		null	null		Unigene10441_All//gi|195611912|gb|ACG27786.1|//2.00E-79//40S ribosomal protein SA [Zea mays]	Unigene10441_All//3.00E-73//AT1G72370| P40; structural constituent of ribosome			ko03010|Ribosome	GO:0005198//GO:0010467//GO:0033279	nr +
Unigene104422_All	496	0.63	0.51	0.32	2.81	1.04	0.55	2.73	1.18	0.96	-3.09E-01	7.89E-01		-9.75E-01	5.06E-01		-1.43E+00	8.65E-03		-2.36E+00	2.38E-04	Down	-1.21E+00	2.08E-02		-1.50E+00	3.72E-03				Unigene104422_All//7.00E-127//TC158572				
Unigene10443_All	482	0.98	0.53	1.99	0.52	2.36	4.69	0.00	0.00	0.17	-8.94E-01	3.89E-01		1.03E+00	1.68E-01		2.19E+00	2.16E-03		3.18E+00	1.22E-08	Up	null	null		7.37E+00	4.06E-01		Unigene10443_All//gi|224060417|ref|XP_002300189.1|//7.00E-57//white-brown-complex ABC transporter family [Populus trichocarpa] >gi|222847447|gb|EEE84994.1| white-brown-complex ABC transporter family [Populus trichocarpa]	Unigene10443_All//8.00E-35//AT1G17840| WBC11 (WHITE-BROWN COMPLEX HOMOLOG PROTEIN 11); ATPase, coupled to transmembrane movement of substances / fatty acid transporter	Unigene10443_All//0.00E+00//gi|48880748|gb|CO131770.1|CO131770		ko03040|Spliceosome//ko02010|ABC transporters	GO:0015604//GO:0017111//GO:0032559//GO:0044464	nr +
Unigene104438_All	620	0.17	0.08	0.09	0.16	0.06	0.07	1.98	0.47	0.32	-1.05E+00	6.86E-01		-9.75E-01	7.22E-01		-1.53E+00	5.89E-01		-1.14E+00	6.79E-01		-2.07E+00	6.67E-04	Down	-2.62E+00	4.02E-05	Down	Unigene104438_All//gi|297818614|ref|XP_002877190.1|//4.00E-15//ATEXO70E1 [Arabidopsis lyrata subsp. lyrata] >gi|297323028|gb|EFH53449.1| ATEXO70E1 [Arabidopsis lyrata subsp. lyrata]	Unigene104438_All//3.00E-15//AT3G29400| ATEXO70E1 (exocyst subunit EXO70 family protein E1); protein binding	Unigene104438_All//0.00E+00//TC146233				nr -
Unigene10445_All	619	1.35	0.74	1.89	8.77	3.73	2.19	2.19	0.88	0.51	-8.76E-01	2.47E-01		4.85E-01	4.47E-01		-1.23E+00	3.00E-07	Down	-2.00E+00	1.58E-12	Down	-1.32E+00	1.25E-02		-2.09E+00	1.85E-04	Down			Unigene10445_All//3.00E-12//ES830995				
Unigene10446_All	595	0.79	0.17	0.81	0.50	3.65	0.76	1.07	0.21	0.27	-2.22E+00	7.77E-02		2.55E-02	1.00E+00		2.86E+00	5.97E-08	Up	5.96E-01	6.40E-01		-2.34E+00	1.27E-02		-1.99E+00	2.04E-02		Unigene10446_All//gi|15232013|ref|NP_187520.1|//2.00E-35//transducin family protein / WD-40 repeat family protein [Arabidopsis thaliana]	Unigene10446_All//1.00E-36//AT3G09080| transducin family protein / WD-40 repeat family protein	Unigene10446_All//0.00E+00//ES850458				nr +
Unigene10447_All	335	0.00	14.82	2.54	0.00	0.00	0.00	0.00	0.00	0.00	1.39E+01	2.63E-28	Up	1.13E+01	9.03E-05	Up	null	null		null	null		null	null		null	null		Unigene10447_All//gi|226456844|gb|EEH54144.1|//1.00E-25//histone H4 [Micromonas pusilla CCMP1545]	Unigene10447_All//6.00E-27//AT5G59970| histone H4	Unigene10447_All//1.00E-16//TC167353			GO:0000785//GO:0003676//GO:0005856//GO:0008092//GO:0034728//GO:0043231	nr -
Unigene104472_All	587	4.10	3.45	1.18	3.73	2.70	4.23	3.75	2.21	1.83	-2.48E-01	5.63E-01		-1.80E+00	1.11E-04	Down	-4.69E-01	2.94E-01		1.81E-01	6.97E-01		-7.63E-01	4.86E-02		-1.03E+00	5.87E-03		Unigene104472_All//gi|7330785|gb|AAF60252.1|AF204677_1//2.00E-59//guanylate kinase [Arabidopsis thaliana]	Unigene104472_All//3.00E-52//AT3G57550| AGK2 (GUANYLATE KINASE); guanylate kinase	Unigene104472_All//0.00E+00//EX168696		ko00230|Purine metabolism	GO:0009117//GO:0016776//GO:0032559//GO:0044424	nr +
Unigene10448_All	479	0.00	0.00	0.00	0.00	0.29	0.19	0.26	1.83	6.15	null	null		null	null		8.17E+00	3.20E-01		7.56E+00	4.82E-01		2.79E+00	6.97E-04	Up	4.54E+00	3.38E-17	Up	Unigene10448_All//gi|6017938|gb|AAF01694.1|AF181456_1//7.00E-13//dehydrin [Hordeum vulgare subsp. vulgare]						nr +
Unigene10449_All	483	0.00	0.00	0.00	0.00	0.00	0.00	5.34	0.61	2.14	null	null		null	null		null	null		null	null		-3.14E+00	2.22E-11	Down	-1.32E+00	1.79E-04	Down	Unigene10449_All//gi|55168340|gb|AAV44205.1|//3.00E-12//unknow protein [Oryza sativa Japonica Group]		Unigene10449_All//1.00E-17//TC149394				nr +
Unigene104511_All	250	0.21	0.00	0.00	0.60	0.83	0.18	3.55	0.50	0.48	-7.71E+00	6.21E-01		-7.71E+00	6.34E-01		4.67E-01	8.26E-01		-1.73E+00	5.02E-01		-2.82E+00	5.65E-04	Down	-2.89E+00	3.05E-04	Down							
Unigene104515_All	277	0.19	0.37	1.35	1.44	0.25	1.63	8.70	15.99	27.04	9.53E-01	7.28E-01		2.83E+00	9.32E-02		-2.53E+00	5.80E-02		1.81E-01	8.84E-01		8.78E-01	6.29E-04		1.64E+00	3.49E-15	Up			Unigene104515_All//2.00E-60//FL576534				
Unigene104518_All	306	0.00	0.33	0.00	0.00	0.00	2.66	0.83	0.00	0.13	8.37E+00	3.91E-01		null	null		null	null		1.14E+01	6.96E-05	Up	-9.70E+00	4.06E-02		-2.67E+00	1.15E-01								
Unigene10452_All	452	0.00	5.72	3.30	0.00	0.00	0.00	0.00	0.00	0.00	1.25E+01	1.06E-14	Up	1.17E+01	3.22E-08	Up	null	null		null	null		null	null		null	null		Unigene10452_All//gi|297798734|ref|XP_002867251.1|//1.00E-43//ATP synthase family [Arabidopsis lyrata subsp. lyrata] >gi|297313087|gb|EFH43510.1| ATP synthase family [Arabidopsis lyrata subsp. lyrata]	Unigene10452_All//1.00E-37//AT4G32260| ATP synthase family			ko00190|Oxidative phosphorylation//ko00195|Photosynthesis	GO:0006754//GO:0006818//GO:0006952//GO:0009526//GO:0009534//GO:0015077//GO:0031224//GO:0033177	nr -
Unigene10453_All	493	0.00	2.78	1.30	0.00	0.14	0.09	0.00	0.00	0.00	1.14E+01	7.80E-08	Up	1.03E+01	1.16E-03		7.13E+00	6.16E-01		6.52E+00	7.04E-01		null	null		null	null		Unigene10453_All//sp|Q5RC60|TIMP1_PONAB//2.00E-68//Metalloproteinase inhibitor 1 OS=Pongo abelii GN=TIMP1 PE=2 SV=1						swissprot -
Unigene104556_All	614	0.00	0.00	0.00	0.16	0.22	1.69	0.48	0.14	0.00	null	null		null	null		4.67E-01	8.78E-01		3.38E+00	2.04E-04	Up	-1.82E+00	2.11E-01		-8.91E+00	1.59E-02		Unigene104556_All//gi|296081092|emb|CBI18286.3|//3.00E-06//unnamed protein product [Vitis vinifera]		Unigene104556_All//4.00E-27//DT462277				nr -
Unigene104566_All	364	0.00	0.00	0.00	0.00	0.00	0.00	6.27	0.00	0.00	null	null		null	null		null	null		null	null		-1.26E+01	3.78E-16	Down	-1.26E+01	8.15E-17	Down	Unigene104566_All//gi|53828151|gb|AAU94355.1|//2.00E-31//iron reductase [Pisum sativum]	Unigene104566_All//4.00E-16//AT1G23020| FRO3; ferric-chelate reductase	Unigene104566_All//1.00E-22//CO089158			GO:0006826//GO:0008152//GO:0016723//GO:0030554//GO:0031224//GO:0046914//GO:0050664//GO:0051707//GO:0055066	nr -
Unigene104568_All	251	0.00	0.00	0.21	0.40	0.00	0.00	4.55	0.00	0.63	null	null		7.73E+00	6.42E-01		-8.63E+00	3.18E-01		-8.63E+00	4.06E-01		-1.22E+01	3.89E-08	Down	-2.84E+00	3.60E-05	Down	Unigene104568_All//gi|224120220|ref|XP_002330994.1|//3.00E-22//cytochrome P450 [Populus trichocarpa] >gi|222872924|gb|EEF10055.1| cytochrome P450 [Populus trichocarpa]	Unigene104568_All//2.00E-22//AT2G42250| CYP712A1; electron carrier/ heme binding / iron ion binding / monooxygenase/ oxygen binding	Unigene104568_All//6.00E-70//ES837654			GO:0005506//GO:0008152//GO:0016709//GO:0044464	nr +
Unigene104571_All	391	1.34	0.00	0.14	0.00	0.18	1.85	0.11	0.11	0.00	-1.04E+01	2.93E-03		-3.30E+00	2.40E-02		7.46E+00	6.16E-01		1.09E+01	2.31E-04	Up	-1.61E-02	1.01E+00		-6.76E+00	6.18E-01		Unigene104571_All//gi|9294523|dbj|BAB02785.1|//3.00E-43//protein transport protein Sec23 [Arabidopsis thaliana]	Unigene104571_All//1.00E-44//AT3G23660| transport protein, putative	Unigene104571_All//1.00E-96//TC133366			GO:0012507//GO:0015031//GO:0046914//GO:0048193	nr +
Unigene104579_All	286	3.48	9.92	2.98	2.96	3.85	4.58	0.30	0.58	0.84	1.51E+00	1.25E-04	Up	-2.23E-01	7.34E-01		3.80E-01	6.01E-01		6.29E-01	3.15E-01		9.83E-01	6.35E-01		1.50E+00	3.39E-01		Unigene104579_All//gi|196196276|gb|ACG76109.1|//2.00E-18//pathogenesis-induced protein [Arachis hypogaea]	Unigene104579_All//2.00E-16//AT1G01360| unknown protein	Unigene104579_All//6.00E-61//CO084264				nr +

Unigene10459_All	349	0.00	0.00	0.00	0.29	0.00	0.00	1.94	0.12	1.71	null	null		null	null		-8.16E+00	3.18E-01		-8.16E+00	4.06E-01		-4.02E+00	5.36E-04	Down	-1.79E-01	7.94E-01		Unigene10459_All//gi|255586731|ref|XP_002533988.1|//1.00E-36//ice binding protein, putative [Ricinus communis] >gi|223526023|gb|EEF28395.1| ice binding protein, putative [Ricinus communis]	Unigene10459_All//3.00E-22//AT5G57770| FUNCTIONS IN: molecular_function unknown; INVOLVED IN: biological_process unknown; LOCATED IN: cellular_component unknown; EXPRESSED IN: root; CONTAINS InterPro DOMAIN/s: Pleckstrin-like, plant (InterPro:IPR013666), Protein of unknown function DUF828, plant (InterPro:IPR008546); Has 104 Blast hits to 104 proteins in 9 species: Archae - 0; Bacteria - 2; Metazoa - 0; Fungi - 0; Plants - 102; Viruses - 0; Other Eukaryotes - 0 (source: NCBI BLink).					nr +
Unigene104598_All	250	0.84	0.00	0.21	6.38	2.75	1.63	0.17	0.00	0.00	-9.71E+00	1.23E-01		-1.97E+00	3.82E-01		-1.21E+00	1.52E-02		-1.97E+00	4.88E-04	Down	-7.40E+00	6.64E-01		-7.40E+00	6.18E-01				Unigene104598_All//4.00E-71//DT048741				
Unigene104611_All	258	0.00	0.00	0.00	3.67	1.87	0.18	0.33	0.00	0.00	null	null		null	null		-9.73E-01	1.60E-01		-4.39E+00	5.97E-05	Down	-8.36E+00	4.08E-01		-8.36E+00	3.62E-01		Unigene104611_All//gi|224123342|ref|XP_002319055.1|//5.00E-13//predicted protein [Populus trichocarpa] >gi|222857431|gb|EEE94978.1| predicted protein [Populus trichocarpa]		Unigene104611_All//2.00E-76//gi|3326234|gb|AI055120.1|AI055120				nr -
Unigene104622_All	224	0.00	0.00	0.71	0.22	0.46	0.81	5.10	0.93	0.53	null	null		9.48E+00	2.40E-01		1.05E+00	8.10E-01		1.86E+00	4.59E-01		-2.45E+00	2.46E-04	Down	-3.26E+00	8.94E-06	Down			Unigene104622_All//2.00E-66//TC154279				
Unigene10463_All	378	0.14	0.13	0.28	0.79	0.18	0.48	2.01	6.63	11.38	-4.66E-02	9.88E-01		1.03E+00	7.05E-01		-2.12E+00	1.66E-01		-7.26E-01	6.39E-01		1.72E+00	8.26E-06	Up	2.50E+00	4.72E-15	Up			Unigene10463_All//1.00E-10//BQ413454				
Unigene104641_All	722	0.00	0.00	0.00	3.31	5.20	2.13	1.52	0.17	0.11	null	null		null	null		6.51E-01	3.83E-02		-6.39E-01	1.31E-01		-3.13E+00	3.47E-05	Down	-3.79E+00	3.27E-06	Down	Unigene104641_All//gi|224090738|ref|XP_002309069.1|//1.00E-100//laccase 110c [Populus trichocarpa] >gi|222855045|gb|EEE92592.1| laccase 110c [Populus trichocarpa]	Unigene104641_All//1.00E-83//AT5G60020| LAC17 (laccase 17); laccase	Unigene104641_All//0.00E+00//gi|109859138|gb|DW488117.1|DW488117		ko00910|Nitrogen metabolism//ko00053|Ascorbate and aldarate metabolism	GO:0005576//GO:0009808//GO:0016682//GO:0046914	nr +
Unigene104648_All	304	1.20	2.50	0.00	0.82	1.25	6.39	0.28	0.55	0.00	1.05E+00	2.05E-01		-1.02E+01	2.79E-02		6.05E-01	6.59E-01		2.96E+00	4.85E-07	Up	9.83E-01	6.35E-01		-8.12E+00	3.62E-01				Unigene104648_All//1.00E-12//AW187099				
Unigene10467_All	469	0.45	0.65	0.57	1.49	0.59	0.67	0.99	4.55	1.10	5.39E-01	6.72E-01		3.47E-01	7.97E-01		-1.34E+00	1.09E-01		-1.14E+00	2.01E-01		2.20E+00	1.16E-06	Up	1.55E-01	8.60E-01				Unigene10467_All//0.00E+00//TC137224				
Unigene10468_All	316	0.00	0.00	0.00	0.00	0.00	0.00	1.87	3.04	0.00	null	null		null	null		null	null		null	null		7.00E-01	2.78E-01		-1.09E+01	1.42E-04	Down							ESTscan -
Unigene104697_All	359	5.98	14.26	7.71	10.27	14.96	17.99	10.36	22.12	24.97	1.25E+00	5.19E-06	Up	3.68E-01	3.75E-01		5.43E-01	3.31E-02		8.09E-01	4.14E-04		1.09E+00	7.67E-09	Up	1.27E+00	1.35E-12	Up	Unigene104697_All//gi|224058435|ref|XP_002299508.1|//6.00E-06//predicted protein [Populus trichocarpa] >gi|222846766|gb|EEE84313.1| predicted protein [Populus trichocarpa]	Unigene104697_All//4.00E-05//AT1G01490| heavy-metal-associated domain-containing protein	Unigene104697_All//9.00E-73//TC164512				nr -
Unigene10470_All	339	0.15	2.69	0.47	0.29	0.41	0.80	0.37	0.25	1.06	4.12E+00	2.34E-04	Up	1.61E+00	5.12E-01		4.67E-01	8.78E-01		1.44E+00	4.10E-01		-6.02E-01	7.68E-01		1.50E+00	2.04E-01				Unigene10470_All//4.00E-07//TC161099				
Unigene104704_All	215	1.22	0.71	5.70	0.93	2.08	6.72	0.20	1.56	0.00	-7.83E-01	6.20E-01		2.23E+00	2.23E-03		1.17E+00	3.47E-01		2.86E+00	3.35E-05	Up	2.98E+00	5.74E-02		-7.62E+00	6.18E-01				Unigene104704_All//3.00E-19//TC133986				ESTscan -
Unigene104720_All	312	12.41	5.52	17.75	3.03	2.54	3.04	0.00	0.00	0.00	-1.17E+00	2.34E-04	Down	5.16E-01	5.24E-02		-2.57E-01	7.27E-01		2.90E-03	9.98E-01		null	null		null	null		Unigene104720_All//gi|19184|emb|CAA43590.1|//6.00E-07//Type I (26 kD) CP29 polypeptide [Solanum lycopersicum]	Unigene104720_All//2.00E-05//AT4G10340| LHCB5 (LIGHT HARVESTING COMPLEX OF PHOTOSYSTEM II 5); chlorophyll binding	Unigene104720_All//3.00E-20//EX167600		ko00196|Photosynthesis - antenna proteins	GO:0044237//GO:0044464	nr -
Unigene10473_All	568	0.00	5.17	1.22	0.00	0.00	0.00	0.00	0.00	0.00	1.23E+01	1.01E-16	Up	1.03E+01	6.17E-04	Up	null	null		null	null		null	null		null	null								ESTscan +
Unigene104760_All	230	0.00	0.00	0.23	0.43	0.60	0.79	6.99	52.34	10.74	null	null		7.85E+00	6.42E-01		4.67E-01	8.78E-01		8.59E-01	6.97E-01		2.91E+00	5.45E-47	Up	6.21E-01	7.63E-02		Unigene104760_All//gi|23978432|dbj|BAC21263.1|//3.00E-12//glutathione S-transferase [Cucurbita maxima]		Unigene104760_All//1.00E-30//gi|164314276|gb|ES842964.1|ES842964		ko00480|Glutathione metabolism	GO:0016740//GO:0016846	nr +
Unigene104761_All	315	0.00	0.00	0.17	4.90	4.04	1.86	2.68	0.27	0.89	null	null		7.40E+00	6.42E-01		-2.77E-01	5.98E-01		-1.40E+00	1.06E-02		-3.34E+00	2.52E-04	Down	-1.60E+00	2.02E-02				Unigene104761_All//1.00E-46//gi|109836538|gb|DW235099.1|DW235099				
Unigene104787_All	352	1.34	5.18	1.82	0.71	1.47	1.80	1.08	1.07	1.13	1.95E+00	2.77E-04	Up	4.41E-01	6.23E-01		1.05E+00	3.49E-01		1.34E+00	1.74E-01		-1.66E-02	1.02E+00		6.63E-02	9.66E-01		Unigene104787_All//gi|6664314|gb|AAF22896.1|AC006932_13//8.00E-12//T27G7.21 [Arabidopsis thaliana]	Unigene104787_All//1.00E-15//AT1G08530| unknown protein	Unigene104787_All//2.00E-120//TC152997			GO:0043231	nr -
Unigene10480_All	291	7.01	32.21	25.25	75.66	61.06	57.58	353.35	284.38	143.88	2.20E+00	5.48E-22	Up	1.85E+00	1.64E-13	Up	-3.09E-01	6.24E-03		-3.94E-01	6.26E-04		-3.13E-01	4.91E-12		-1.30E+00	2.92E-141	Down			Unigene10480_All//2.00E-138//gi|109838778|gb|DW237339.1|DW237339				
Unigene104806_All	495	0.42	0.82	2.80	1.51	0.42	0.91	0.17	0.17	0.16	9.54E-01	4.14E-01		2.73E+00	1.78E-04	Up	-1.85E+00	2.53E-02		-7.26E-01	3.90E-01		-1.61E-02	1.02E+00		-8.52E-02	9.74E-01				Unigene104806_All//1.00E-149//gi|21096576|gb|BQ408889.1|BQ408889				
Unigene10482_All	412	0.00	0.00	0.00	0.00	0.00	0.00	6.57	3.04	0.00	null	null		null	null		null	null		null	null		-1.11E+00	1.31E-03		-1.27E+01	6.55E-20	Down	Unigene10482_All//gi|145408590|ref|YP_001152214.1|//8.00E-06//ORF124 [Pinus koraiensis] >gi|145048839|gb|ABP35456.1| ORF124 [Pinus koraiensis]		Unigene10482_All//4.00E-23//gi|11207717|gb|BF276647.1|BF276647				nr -
Unigene104832_All	298	0.53	0.34	1.25	7.36	15.25	4.55	2.84	0.00	0.00	-6.31E-01	7.29E-01		1.25E+00	3.66E-01		1.05E+00	1.21E-04	Up	-6.94E-01	1.18E-01		-1.15E+01	4.39E-06	Down	-1.15E+01	2.21E-06	Down			Unigene104832_All//3.00E-134//ES851582				
Unigene104833_All	309	34.20	13.45	37.57	23.70	22.18	10.09	0.96	0.68	0.52	-1.35E+00	6.78E-13	Down	1.35E-01	4.79E-01		-9.57E-02	7.14E-01		-1.23E+00	1.49E-08	Down	-5.02E-01	6.78E-01		-8.93E-01	4.71E-01				Unigene104833_All//2.00E-110//TC174668				
Unigene10484_All	373	0.56	2.45	4.00	8.15	8.68	6.54	0.45	0.22	0.00	2.12E+00	1.04E-02		2.83E+00	6.17E-05	Up	9.12E-02	8.23E-01		-3.17E-01	4.17E-01		-1.02E+00	6.16E-01		-8.82E+00	1.09E-01				Unigene10484_All//2.00E-67//gi|13247536|gb|BF270981.2|BF270981				
Unigene104856_All	269	0.39	0.00	0.00	0.19	0.38	0.17	2.67	0.16	0.59	-8.60E+00	3.77E-01		-8.60E+00	3.99E-01		1.05E+00	8.10E-01		-1.42E-01	9.57E-01		-4.10E+00	2.92E-04	Down	-2.17E+00	7.76E-03		Unigene104856_All//gi|19570247|dbj|BAB86283.1|//6.00E-28//kinesin-like protein NACK1 [Nicotiana tabacum]	Unigene104856_All//7.00E-27//AT1G18370| HIK (HINKEL); ATP binding / microtubule motor				GO:0000226//GO:0003774//GO:0007349//GO:0009536//GO:0015630//GO:0032559//GO:0033206//GO:0046914//GO:0048229	nr +
Unigene104865_All	266	0.39	0.00	1.20	0.00	1.42	2.89	0.95	0.00	0.60	-8.62E+00	3.77E-01		1.61E+00	3.05E-01		1.05E+01	1.96E-02		1.15E+01	1.27E-04	Up	-9.90E+00	4.06E-02		-6.71E-01	6.06E-01		Unigene104865_All//gi|186501175|ref|NP_179383.2|//8.00E-35//binding / inositol or phosphatidylinositol kinase/ phosphotransferase, alcohol group as acceptor [Arabidopsis thaliana]	Unigene104865_All//3.00E-36//AT2G17930| binding / inositol or phosphatidylinositol kinase/ phosphotransferase, alcohol group as acceptor	Unigene104865_All//2.00E-141//TC142236			GO:0016772	nr +
Unigene10487_All	286	0.00	0.00	0.00	0.00	0.00	0.00	217.46	79.21	21.17	null	null		null	null		null	null		null	null		-1.46E+00	1.65E-99	Down	-3.36E+00	6.57E-287	Down							
Unigene10489_All	362	0.87	0.14	3.97	1.24	0.00	0.00	0.00	0.00	0.00	-2.63E+00	1.31E-01		2.20E+00	8.73E-04	Up	-1.03E+01	2.00E-03		-1.03E+01	5.79E-03		null	null		null	null		Unigene10489_All//COG4784//1.00E-25//Putative Zn-dependent protease						cog -
Unigene104908_All	274	2.29	2.03	1.75	4.73	5.66	2.47	5.25	6.25	1.16	-1.72E-01	8.29E-01		-3.90E-01	6.46E-01		2.59E-01	6.64E-01		-9.35E-01	1.22E-01		2.54E-01	6.22E-01		-2.17E+00	6.89E-05	Down			Unigene104908_All//7.00E-73//ES847924				
Unigene104912_All	236	190.88	117.44	217.96	24.27	22.18	8.80	0.18	0.35	0.00	-7.01E-01	2.76E-18		1.91E-01	1.68E-02		-1.30E-01	6.34E-01		-1.46E+00	1.08E-08	Down	9.83E-01	7.33E-01		-7.49E+00	6.18E-01				Unigene104912_All//6.00E-107//gi|84145839|gb|DN760081.1|DN760081				
Unigene104917_All	446	1.06	0.68	1.19	1.90	0.77	2.23	5.97	1.78	1.79	-6.31E-01	5.54E-01		1.77E-01	8.45E-01		-1.30E+00	8.01E-02		2.31E-01	7.41E-01		-1.75E+00	2.99E-06	Down	-1.74E+00	1.78E-06	Down	Unigene104917_All//gi|225440831|ref|XP_002282071.1|//2.00E-15//PREDICTED: similar to ubiquitin fusion protein [Vitis vinifera] >gi|71040673|gb|AAZ20285.1| ubiquitin fusion protein [Arachis hypogaea] >gi|192910738|gb|ACF06477.1| ubiquitin fusion protein [Elaeis guineensis] >gi|284433784|gb|ADB85098.1| ubiquitin C variant [Jatropha curcas]	Unigene104917_All//2.00E-16//AT3G52590| UBQ1 (UBIQUITIN EXTENSION PROTEIN 1); protein binding / structural constituent of ribosome	Unigene104917_All//2.00E-154//gi|84164961|gb|DR453609.1|DR453609		ko03010|Ribosome	GO:0003006//GO:0005198//GO:0005488//GO:0010467//GO:0015934//GO:0031981//GO:0032446	nr +
Unigene10493_All	272	0.00	0.00	0.00	0.00	0.00	0.00	2.80	0.92	0.00	null	null		null	null		null	null		null	null		-1.60E+00	3.70E-02		-1.15E+01	8.89E-06	Down	Unigene10493_All//gi|159489791|ref|XP_001702878.1|//3.00E-18//ribosomal protein L38, component of cytosolic 80S ribosome and 60S large subunit [Chlamydomonas reinhardtii] >gi|27497793|gb|AAO13217.1| 60S ribosomal protein L38 [Chlamydomonas reinhardtii] >gi|158271006|gb|EDO96835.1| ribosomal protein L38 [Chlamydomonas reinhardtii]	Unigene10493_All//4.00E-19//AT3G59540| 60S ribosomal protein L38 (RPL38B)			ko03010|Ribosome	GO:0005198//GO:0010467//GO:0015934//GO:0022613	nr +
Unigene104932_All	487	0.43	3.95	0.87	0.20	0.14	2.97	1.48	3.18	1.31	3.20E+00	3.42E-07	Up	1.03E+00	3.97E-01		-5.33E-01	8.20E-01		3.86E+00	1.51E-06	Up	1.11E+00	2.15E-02		-1.73E-01	7.94E-01		Unigene104932_All//gi|109631652|gb|ABG36000.1|//3.00E-31//glutathione peroxidase [Helianthus annuus] >gi|109631654|gb|ABG36001.1| glutathione peroxidase [Helianthus annuus]	Unigene104932_All//1.00E-26//AT1G48130| ATPER1; antioxidant/ thioredoxin peroxidase	Unigene104932_All//1.00E-168//gi|109871672|gb|DW500648.1|DW500648		ko00940|Phenylpropanoid biosynthesis//ko00360|Phenylalanine metabolism	GO:0008152//GO:0016209//GO:0016705//GO:0019725//GO:0043231	nr +
Unigene10494_All	343	0.00	4.87	0.78	0.00	0.00	0.00	0.00	0.00	0.00	1.23E+01	1.54E-09	Up	9.60E+00	8.04E-02		null	null		null	null		null	null		null	null		Unigene10494_All//sp|P40092|SPI1_YEAST//7.00E-09//Uncharacterized cell wall protein SPI1 OS=Saccharomyces cerevisiae GN=SPI1 PE=2 SV=1						swissprot -
Unigene10496_All	398	0.00	0.00	0.00	0.63	0.95	0.00	2.76	1.47	0.60	null	null		null	null		6.05E-01	6.59E-01		-9.29E+00	7.36E-02		-9.10E-01	1.24E-01		-2.20E+00	5.66E-04	Down	Unigene10496_All//gi|224129122|ref|XP_002320506.1|//3.00E-19//f-box family protein [Populus trichocarpa] >gi|222861279|gb|EEE98821.1| f-box family protein [Populus trichocarpa]	Unigene10496_All//3.00E-11//AT3G07870| F-box family protein					nr -
Unigene104966_All	793	1.06	1344.50	787.60	0.38	0.09	3.82	2.03	2.27	1.51	1.03E+01	0.00E+00	Up	9.54E+00	0.00E+00	Up	-2.12E+00	1.66E-01		3.34E+00	6.20E-12	Up	1.62E-01	7.18E-01		-4.27E-01	3.59E-01		Unigene104966_All//gi|145408594|ref|YP_001152218.1|//3.00E-15//ORF82c [Pinus koraiensis] >gi|145048843|gb|ABP35459.1| ORF82c [Pinus koraiensis]		Unigene104966_All//1.00E-92//TC171825			GO:0009536	nr +
Unigene10498_All	332	0.00	3.36	3.37	0.00	0.00	0.00	0.00	0.00	0.12	1.17E+01	2.00E-06	Up	1.17E+01	3.44E-06	Up	null	null		null	null		null	null		6.91E+00	6.48E-01								
Unigene104980_All	310	11.65	9.81	15.46	20.57	19.55	20.11	44.33	25.08	18.38	-2.48E-01	4.59E-01		4.09E-01	1.62E-01		-7.32E-02	8.11E-01		-3.29E-02	9.14E-01		-8.22E-01	1.86E-09		-1.27E+00	4.74E-19	Down	Unigene104980_All//gi|225464952|ref|XP_002274218.1|//2.00E-09//PREDICTED: similar to ribosomal protein L10a [Vitis vinifera]	Unigene104980_All//5.00E-09//AT2G27530| PGY1 (PIGGYBACK1); RNA binding / structural constituent of ribosome	Unigene104980_All//7.00E-138//gi|13245690|gb|BE055045.2|BE055045		ko03010|Ribosome		nr +
Unigene104982_All	254	0.82	1.00	3.14	0.39	1.76	2.13	3.50	0.49	1.73	2.76E-01	8.44E-01		1.93E+00	3.42E-02		2.17E+00	1.06E-01		2.44E+00	4.41E-02		-2.82E+00	5.65E-04	Down	-1.02E+00	1.09E-01		Unigene104982_All//gi|26451087|dbj|BAC42648.1|//4.00E-15//GPI-anchored protein [Arabidopsis thaliana] >gi|28416735|gb|AAO42898.1| At5g63500 [Arabidopsis thaliana]	Unigene104982_All//6.00E-17//AT3G48660| unknown protein	Unigene104982_All//1.00E-21//gi|31073375|gb|CA993654.1|CA993654			GO:0031224	nr -
Unigene104988_All	443	0.24	0.23	0.24	0.00	0.86	2.24	0.48	0.94	0.36	-4.59E-02	9.91E-01		2.54E-02	1.00E+00		9.74E+00	1.96E-02		1.11E+01	6.15E-06	Up	9.83E-01	3.73E-01		-4.08E-01	7.56E-01		Unigene104988_All//gi|149391295|gb|ABR25665.1|//2.00E-54//cbs domain containing protein, expressed [Oryza sativa Indica Group]	Unigene104988_All//3.00E-55//AT2G14520| CBS domain-containing protein	Unigene104988_All//2.00E-56//TC172662			GO:0031410	nr +
Unigene104998_All	252	5.40	0.60	0.85	12.45	9.57	8.24	43.29	22.56	13.12	-3.16E+00	2.85E-05	Down	-2.68E+00	2.60E-04	Down	-3.81E-01	3.03E-01		-5.95E-01	9.66E-02		-9.40E-01	1.97E-09		-1.72E+00	1.43E-23	Down	Unigene104998_All//gi|195618310|gb|ACG30985.1|//6.00E-06//60S acidic ribosomal protein P1 [Zea mays] >gi|195625300|gb|ACG34480.1| 60S acidic ribosomal protein P1 [Zea mays]		Unigene104998_All//3.00E-133//gi|21089661|gb|BQ401986.1|BQ401986		ko03010|Ribosome		nr -
Unigene1050_All	2881	5.43	0.97	2.62	88.13	71.68	64.87	31.61	6.34	9.04	-2.49E+00	2.54E-42	Down	-1.05E+00	2.82E-12	Down	-2.98E-01	1.71E-25		-4.42E-01	3.39E-47		-2.32E+00	8.41E-274	Down	-1.81E+00	1.05E-203	Down	Unigene1050_All//gi|224130916|ref|XP_002320956.1|//0.00E+00//anion exchanger family protein [Populus trichocarpa] >gi|222861729|gb|EEE99271.1| anion exchanger family protein [Populus trichocarpa]	Unigene1050_All//0.00E+00//AT2G47160| BOR1 (REQUIRES HIGH BORON 1); anion exchanger/ boron transporter	Unigene1050_All//0.00E+00//CO074020			GO:0006811//GO:0010035//GO:0015301//GO:0015562//GO:0031224//GO:0043231//GO:0043673//GO:0044459	nr -

Unigene10500_All	428	0.00	0.00	0.00	0.00	0.00	0.00	1.78	1.86	0.00	null	null		null	null		null	null		null	null		6.14E-02	9.50E-01		-1.08E+01	8.89E-06	Down	Unigene10500_All//gi|300267444|gb|EFJ51627.1|//2.00E-23//component of cytosolic 80S ribosome and 40S small subunit [Volvox carteri f. nagariensis]	Unigene10500_All//1.00E-21//AT3G04770| RPSAb (40S ribosomal protein SA B); structural constituent of ribosome			ko03010|Ribosome	GO:0005737//GO:0030529	nr +
Unigene105022_All	596	34.41	12.33	36.54	4.01	3.18	1.21	1.21	1.68	0.27	-1.48E+00	5.85E-28	Down	8.66E-02	5.43E-01		-3.36E-01	4.41E-01		-1.73E+00	6.61E-05	Down	4.81E-01	4.46E-01		-2.17E+00	7.76E-03		Unigene105022_All//gi|87241054|gb|ABD32912.1|//5.00E-38//Ferric reductase-like transmembrane component [Medicago truncatula]	Unigene105022_All//6.00E-30//AT5G49730| ATFRO6 (FERRIC REDUCTION OXIDASE 6); ferric-chelate reductase/ oxidoreductase	Unigene105022_All//0.00E+00//DW504224			GO:0005488//GO:0009314//GO:0016020//GO:0016723	nr -
Unigene10503_All	1095	0.00	0.00	0.00	0.05	0.19	0.25	0.66	1.41	4.04	null	null		null	null		2.05E+00	3.95E-01		2.44E+00	2.22E-01		1.11E+00	2.15E-02		2.62E+00	2.98E-16	Up	Unigene10503_All//gi|14330716|emb|CAC40826.1|//1.00E-42//HcrVf2 protein [Malus floribunda]	Unigene10503_All//1.00E-29//AT2G34930| disease resistance family protein					nr +
Unigene10507_All	381	0.41	0.00	0.00	0.00	0.00	0.00	2.89	1.10	0.21	-8.69E+00	2.17E-01		-8.69E+00	2.46E-01		null	null		null	null		-1.40E+00	2.04E-02		-3.79E+00	3.27E-06	Down							
Unigene10509_All	482	0.87	1.68	0.77	3.93	3.72	1.03	0.70	0.35	0.50	9.54E-01	2.26E-01		-1.67E-01	8.79E-01		-8.02E-02	8.58E-01		-1.93E+00	1.45E-04	Down	-1.02E+00	4.00E-01		-5.01E-01	6.50E-01				Unigene10509_All//3.00E-135//DW519316				
Unigene1051_All	2081	12.22	14.88	14.25	25.28	26.58	38.07	51.54	46.48	20.01	2.84E-01	4.02E-03		2.22E-01	4.09E-02		7.22E-02	4.13E-01		5.91E-01	4.64E-25		-1.49E-01	1.21E-03		-1.37E+00	1.34E-159	Down	Unigene1051_All//gi|28380080|sp|Q9C8F1.1|IF5Y_ARATH//1.00E-170//RecName: Full=Probable eukaryotic translation initiation factor 5-1; Short=eIF-5 1	Unigene1051_All//4.00E-147//AT1G36730| eukaryotic translation initiation factor 5, putative / eIF-5, putative	Unigene1051_All//0.00E+00//TC149110			GO:0006412//GO:0008135	nr -
Unigene105108_All	298	0.18	0.00	0.00	4.18	2.20	0.76	0.00	0.00	0.27	-7.46E+00	6.21E-01		-7.46E+00	6.34E-01		-9.29E-01	1.11E-01		-2.46E+00	4.15E-04	Down	null	null		8.06E+00	4.06E-01		Unigene105108_All//gi|22329014|ref|NP_194662.2|//5.00E-24//DER1 (DERLIN-1) [Arabidopsis thaliana] >gi|75161705|sp|Q8VZU9.1|DERL1_ARATH RecName: Full=Derlin-1; AltName: Full=AtDerlin1-1	Unigene105108_All//2.00E-25//AT4G29330| DER1 (DERLIN-1)	Unigene105108_All//9.00E-08//DR459372			GO:0019538//GO:0043231//GO:0044425	nr +
Unigene10511_All	398	0.00	4.71	2.81	0.00	0.00	0.00	0.00	0.00	0.00	1.22E+01	1.11E-10	Up	1.15E+01	3.44E-06	Up	null	null		null	null		null	null		null	null								
Unigene105113_All	296	2.65	0.17	1.62	1.85	1.63	1.53	0.14	0.56	0.81	-3.95E+00	8.54E-04	Down	-7.12E-01	3.89E-01		-1.85E-01	8.25E-01		-2.79E-01	7.57E-01		1.98E+00	3.80E-01		2.50E+00	1.59E-01		Unigene105113_All//gi|18396347|ref|NP_564285.1|//2.00E-16//NPGR1 (NO POLLEN GERMINATION RELATED 1); calmodulin binding [Arabidopsis thaliana] >gi|9802536|gb|AAF99738.1|AC004557_17 F17L21.25 [Arabidopsis thaliana] >gi|16226498|gb|AAL16183.1|AF428415_1 At1g27460/F17L21_26 [Arabidopsis thaliana] >gi|33589668|gb|AAQ22600.1| At1g27460/F17L21_26 [Arabidopsis thaliana]	Unigene105113_All//7.00E-18//AT1G27460| NPGR1 (NO POLLEN GERMINATION RELATED 1); calmodulin binding	Unigene105113_All//1.00E-108//TC142974			GO:0005515//GO:0016020	nr +
Unigene10512_All	430	0.00	0.47	1.24	0.00	1.20	2.00	0.98	0.29	2.78	8.88E+00	1.37E-01		1.03E+01	4.05E-03		1.02E+01	3.22E-03		1.10E+01	3.79E-05	Up	-1.75E+00	1.24E-01		1.50E+00	7.56E-03		Unigene10512_All//gi|225432404|ref|XP_002276907.1|//2.00E-11//PREDICTED: hypothetical protein [Vitis vinifera]		Unigene10512_All//2.00E-49//gi|109864441|gb|DW493417.1|DW493417				nr +
Unigene105124_All	495	0.42	0.00	1.18	2.92	2.09	2.10	1.03	0.00	0.32	-8.72E+00	1.23E-01		1.48E+00	1.53E-01		-4.84E-01	3.92E-01		-4.76E-01	4.15E-01		-1.00E+01	8.91E-04	Down	-1.67E+00	7.78E-02		Unigene105124_All//gi|118481019|gb|ABK92463.1|//4.00E-15//unknown [Populus trichocarpa]	Unigene105124_All//4.00E-08//AT2G40480| unknown protein	Unigene105124_All//4.00E-14//TC165937				nr +
Unigene105128_All	317	0.00	0.16	0.00	0.16	0.00	1.42	0.27	0.79	2.76	7.32E+00	6.22E-01		null	null		-7.30E+00	5.64E-01		3.18E+00	3.79E-02		1.57E+00	3.27E-01		3.37E+00	1.32E-04	Up	Unigene105128_All//gi|297819958|ref|XP_002877862.1|//4.00E-21//U-box domain-containing protein [Arabidopsis lyrata subsp. lyrata] >gi|297323700|gb|EFH54121.1| U-box domain-containing protein [Arabidopsis lyrata subsp. lyrata]	Unigene105128_All//2.00E-22//AT3G52450| PUB22 (PLANT U-BOX 22); ubiquitin-protein ligase			ko04141|Protein processing in endoplasmic reticulum//ko04120|Ubiquitin mediated proteolysis	GO:0002252//GO:0006950//GO:0016567//GO:0019787//GO:0043234//GO:0044444	nr +
Unigene10514_All	478	0.00	0.00	0.00	2.29	1.80	0.38	1.06	0.26	0.67	null	null		null	null		-3.48E-01	5.97E-01		-2.60E+00	7.14E-04	Down	-2.02E+00	5.21E-02		-6.71E-01	4.49E-01		Unigene10514_All//gi|297605832|ref|NP_001057661.2|//7.00E-16//Os06g0484800 [Oryza sativa Japonica Group] >gi|255677053|dbj|BAF19575.2| Os06g0484800 [Oryza sativa Japonica Group]						nr -
Unigene10515_All	342	0.00	0.00	0.00	0.00	0.00	0.00	3.83	3.79	0.00	null	null		null	null		null	null		null	null		-1.66E-02	1.01E+00		-1.19E+01	9.86E-10	Down			Unigene10515_All//4.00E-10//TC139311				
Unigene105161_All	568	0.00	1.78	0.00	0.18	0.36	1.67	0.00	0.66	1.05	1.08E+01	7.26E-06	Up	null	null		1.05E+00	6.13E-01		3.25E+00	5.84E-04	Up	9.37E+00	6.87E-03		1.00E+01	1.76E-04	Up	Unigene105161_All//gi|224082932|ref|XP_002306897.1|//1.00E-48//AP2/ERF domain-containing transcription factor [Populus trichocarpa] >gi|222856346|gb|EEE93893.1| AP2/ERF domain-containing transcription factor [Populus trichocarpa]	Unigene105161_All//1.00E-42//AT1G75490| DNA binding / transcription factor	Unigene105161_All//3.00E-52//TC178922	AP2-EREBP		GO:0001071//GO:0003676//GO:0006351//GO:0009743//GO:0010468//GO:0043231	nr +
Unigene10517_All	250	0.21	0.00	0.00	0.00	0.28	0.00	13.53	26.75	224.85	-7.71E+00	6.21E-01		-7.71E+00	6.34E-01		8.11E+00	6.16E-01		null	null		9.83E-01	1.72E-06		4.05E+00	1.65E-296	Up	Unigene10517_All//gi|255575021|ref|XP_002528416.1|//9.00E-13//amino acid transporter, putative [Ricinus communis] >gi|223532152|gb|EEF33958.1| amino acid transporter, putative [Ricinus communis]					GO:0031224	nr +
Unigene105188_All	486	0.65	1.25	0.00	1.33	1.70	2.42	0.09	1.12	1.56	9.54E-01	3.14E-01		-9.34E+00	4.93E-02		3.52E-01	6.87E-01		8.59E-01	1.95E-01		3.68E+00	3.69E-03		4.16E+00	1.34E-04	Up	Unigene105188_All//gi|79474118|ref|NP_193133.3|//3.00E-54//zinc ion binding [Arabidopsis thaliana]	Unigene105188_All//1.00E-55//AT4G13970| zinc ion binding				GO:0046914	nr -
Unigene10519_All	394	0.27	2.44	0.81	1.52	0.52	0.11	2.15	0.95	1.21	3.20E+00	6.55E-04	Up	1.61E+00	3.05E-01		-1.53E+00	1.01E-01		-3.73E+00	4.96E-03		-1.17E+00	9.29E-02		-8.23E-01	2.07E-01				Unigene10519_All//0.00E+00//TC173046				
Unigene105205_All	567	0.00	0.00	0.19	0.79	0.97	0.40	15.36	0.96	0.49	null	null		7.55E+00	4.03E-01		2.97E-01	8.18E-01		-9.89E-01	3.94E-01		-4.00E+00	2.65E-45	Down	-4.97E+00	1.25E-53	Down	Unigene105205_All//gi|224120220|ref|XP_002330994.1|//7.00E-63//cytochrome P450 [Populus trichocarpa] >gi|222872924|gb|EEF10055.1| cytochrome P450 [Populus trichocarpa]	Unigene105205_All//1.00E-55//AT2G42250| CYP712A1; electron carrier/ heme binding / iron ion binding / monooxygenase/ oxygen binding	Unigene105205_All//2.00E-158//gi|48750763|gb|CO081282.1|CO081282		ko00945|Stilbenoid, diarylheptanoid and gingerol biosynthesis//ko00903|Limonene and pinene degradation	GO:0005506//GO:0008152//GO:0016709//GO:0044464	nr +
Unigene105206_All	340	0.92	0.00	0.31	3.52	0.81	0.80	1.74	0.25	0.00	-9.85E+00	3.67E-02		-1.56E+00	3.30E-01		-2.12E+00	8.56E-04	Down	-2.14E+00	1.81E-03		-2.82E+00	7.18E-03		-1.08E+01	1.42E-04	Down							
Unigene105230_All	327	0.48	0.16	0.00	0.61	1.05	1.66	4.91	3.07	0.85	-1.63E+00	4.89E-01		-8.91E+00	2.46E-01		7.89E-01	5.96E-01		1.44E+00	1.93E-01		-6.80E-01	1.47E-01		-2.53E+00	2.96E-06	Down			Unigene105230_All//2.00E-67//gi|18098905|gb|BM358159.1|BM358159				
Unigene105235_All	741	0.14	0.00	0.07	0.20	0.05	0.00	0.68	0.00	0.00	-7.14E+00	3.77E-01		-9.74E-01	7.22E-01		-2.12E+00	3.88E-01		-7.66E+00	2.35E-01		-9.42E+00	8.91E-04	Down	-9.42E+00	5.56E-04	Down	Unigene105235_All//gi|224140225|ref|XP_002323485.1|//5.00E-81//multidrug/pheromone exporter, MDR family, ABC transporter family [Populus trichocarpa] >gi|222868115|gb|EEF05246.1| multidrug/pheromone exporter, MDR family, ABC transporter family [Populus trichocarpa]	Unigene105235_All//8.00E-69//AT2G36910| ABCB1 (ATP BINDING CASSETTE SUBFAMILY B1); ATPase, coupled to transmembrane movement of substances / auxin efflux transmembrane transporter/ calmodulin binding			ko02010|ABC transporters	GO:0005515//GO:0009630//GO:0009639//GO:0009725//GO:0015562//GO:0022891//GO:0031224//GO:0032535//GO:0032559//GO:0042626//GO:0043231//GO:0043479//GO:0048437//GO:0051707//GO:0060918	nr -
Unigene105249_All	810	0.13	0.00	0.13	0.06	0.38	2.84	0.00	0.00	0.30	-7.01E+00	3.77E-01		2.55E-02	1.00E+00		2.64E+00	1.61E-01		5.53E+00	2.12E-12	Up	null	null		8.21E+00	4.41E-02								
Unigene105254_All	655	4.79	9.98	9.02	3.80	3.31	3.17	0.13	0.06	0.12	1.06E+00	7.00E-06	Up	9.13E-01	3.35E-04		-1.99E-01	6.31E-01		-2.62E-01	5.66E-01		-1.02E+00	7.27E-01		-8.63E-02	9.74E-01				Unigene105254_All//1.00E-131//gi|78333874|gb|DT554148.1|DT554148				
Unigene10526_All	302	1.73	7.89	7.93	0.99	1.14	0.75	2.52	0.42	0.66	2.19E+00	4.62E-06	Up	2.20E+00	7.43E-06	Up	2.04E-01	9.02E-01		-4.05E-01	7.52E-01		-2.60E+00	2.81E-03		-1.93E+00	1.14E-02				Unigene10526_All//3.00E-109//gi|21102508|gb|BQ414821.1|BQ414821				
Unigene105280_All	301	8.00	10.44	10.79	8.44	5.38	2.85	2.25	0.97	0.40	3.84E-01	2.87E-01		4.33E-01	2.39E-01		-6.51E-01	9.08E-02		-1.57E+00	1.37E-04	Down	-1.21E+00	1.34E-01		-2.50E+00	4.79E-03				Unigene105280_All//9.00E-88//TC149505				
Unigene10529_All	293	0.36	0.00	1.27	0.17	0.00	0.00	6.06	3.14	1.22	-8.48E+00	3.77E-01		1.83E+00	2.13E-01		-7.41E+00	5.64E-01		-7.41E+00	6.46E-01		-9.50E-01	3.09E-02		-2.31E+00	3.08E-06	Down	Unigene10529_All//gi|115447285|ref|NP_001047422.1|//4.00E-19//Os02g0614100 [Oryza sativa Japonica Group] >gi|113536953|dbj|BAF09336.1| Os02g0614100 [Oryza sativa Japonica Group]	Unigene10529_All//5.00E-11//AT4G32272| FUNCTIONS IN: molecular_function unknown; INVOLVED IN: biological_process unknown; LOCATED IN: cellular_component unknown; CONTAINS InterPro DOMAIN/s: Protein of unknown function DUF250 (InterPro:IPR004853); BEST Arabidopsis thaliana protein match is: UDP-glucuronic acid/UDP-N-acetylgalactosamine transporter-related (TAIR:AT4G31600.1); Has 1302 Blast hits to 1300 proteins in 174 species: Archae - 0; Bacteria - 4; Metazoa - 376; Fungi - 239; Plants - 567; Viruses - 0; Other Eukaryotes - 116 (source: NCBI BLink).	Unigene10529_All//3.00E-94//TC175087			GO:0031224	nr +
Unigene1053_All	1096	2.24	3.98	0.97	1.18	2.04	1.69	1.62	7.59	3.93	8.25E-01	4.54E-03		-1.21E+00	5.14E-03		7.89E-01	6.64E-02		5.16E-01	3.15E-01		2.23E+00	2.37E-24	Up	1.28E+00	1.51E-06	Up	Unigene1053_All//gi|90900944|gb|ABE01834.1|//1.00E-82//NAK-type protein kinase [Nicotiana tabacum]	Unigene1053_All//2.00E-54//AT2G39110| protein kinase, putative	Unigene1053_All//0.00E+00//CO113128			GO:0004672//GO:0006464//GO:0032559	nr +
Unigene10530_All	1110	0.00	0.00	0.00	0.00	0.00	0.00	4.72	1.51	0.50	null	null		null	null		null	null		null	null		-1.65E+00	9.64E-11	Down	-3.23E+00	6.29E-24	Down	Unigene10530_All//gi|27544010|dbj|BAC54829.1|//5.00E-67//vacuolar processing enzyme-2 [Nicotiana tabacum]	Unigene10530_All//2.00E-65//AT2G25940| ALPHA-VPE (alpha-vacuolar processing enzyme); cysteine-type endopeptidase				GO:0004175//GO:0016485	nr -
Unigene105308_All	361	0.72	0.84	3.69	2.48	0.86	1.25	0.23	0.00	0.00	2.17E-01	8.65E-01		2.35E+00	8.68E-04	Up	-1.53E+00	3.38E-02		-9.89E-01	1.96E-01		-7.87E+00	4.08E-01		-7.87E+00	3.62E-01								
Unigene105325_All	691	0.53	0.44	0.46	1.37	1.45	3.66	0.98	0.00	0.52	-2.69E-01	8.01E-01		-1.97E-01	8.66E-01		7.74E-02	9.37E-01		1.42E+00	5.71E-04	Up	-9.94E+00	6.35E-05	Down	-9.16E-01	2.21E-01		Unigene105325_All//gi|14334057|gb|AAK60517.1|AF332974_1//8.00E-53//P450 monooxygenase [Gossypium arboreum]	Unigene105325_All//2.00E-32//AT4G12320| CYP706A6; electron carrier/ heme binding / iron ion binding / monooxygenase/ oxygen binding	Unigene105325_All//1.00E-119//ES819299		ko00945|Stilbenoid, diarylheptanoid and gingerol biosynthesis//ko00903|Limonene and pinene degradation//ko00941|Flavonoid biosynthesis//ko00944|Flavone and flavonol biosynthesis	GO:0005506//GO:0016491	nr -
Unigene10533_All	562	0.00	0.00	0.00	0.00	0.00	0.00	3.01	2.60	0.43	null	null		null	null		null	null		null	null		-2.09E-01	6.45E-01		-2.82E+00	2.86E-07	Down	Unigene10533_All//gi|116222259|gb|ABJ80993.1|//7.00E-86//beta tubulin [Goniomonas truncata]	Unigene10533_All//1.00E-82//AT5G23860| TUB8; structural constituent of cytoskeleton	Unigene10533_All//4.00E-11//gi|48790617|gb|CO091931.1|CO091931		ko04145|Phagosome	GO:0007017//GO:0015630//GO:0017111//GO:0032561//GO:0032991//GO:0043623	nr +
Unigene105331_All	833	0.06	0.37	0.38	0.00	0.08	11.66	0.00	0.00	0.00	2.54E+00	1.51E-01		2.61E+00	1.46E-01		6.37E+00	6.16E-01		1.35E+01	3.50E-59	Up	null	null		null	null		Unigene105331_All//sp|Q7Z1M0|VIT1_SOLIN//1.00E-59//Vitellogenin-1 OS=Solenopsis invicta PE=2 SV=1						swissprot -
Unigene105333_All	535	2.25	5.78	5.08	3.63	4.31	2.79	0.40	1.02	0.30	1.36E+00	2.28E-04	Up	1.17E+00	3.80E-03		2.48E-01	6.08E-01		-3.82E-01	4.51E-01		1.36E+00	1.44E-01		-4.07E-01	7.56E-01				Unigene105333_All//1.00E-165//gi|84165968|gb|DR454616.1|DR454616				
Unigene105373_All	592	0.18	0.00	0.00	0.08	0.06	0.15	0.00	1.41	0.00	-7.47E+00	3.77E-01		-7.47E+00	3.99E-01		-5.31E-01	8.29E-01		8.60E-01	7.97E-01		1.05E+01	5.54E-06	Up	null	null		Unigene105373_All//gi|297829824|ref|XP_002882794.1|//3.00E-56//oxidoreductase [Arabidopsis lyrata subsp. lyrata] >gi|297328634|gb|EFH59053.1| oxidoreductase [Arabidopsis lyrata subsp. lyrata]	Unigene105373_All//2.00E-57//AT3G12900| oxidoreductase, 2OG-Fe(II) oxygenase family protein	Unigene105373_All//2.00E-16//TC134433		ko00941|Flavonoid biosynthesis//ko00402|Benzoxazinoid biosynthesis	GO:0008152//GO:0016701//GO:0016706	nr -
Unigene10539_All	397	0.00	0.00	0.00	0.00	0.00	0.00	2.13	1.58	0.00	null	null		null	null		null	null		null	null		-4.32E-01	5.47E-01		-1.11E+01	2.21E-06	Down	Unigene10539_All//sp|P34121|COAA_DICDI//9.00E-17//Coactosin OS=Dictyostelium discoideum GN=coaA PE=1 SV=1						swissprot +
Unigene105402_All	407	0.13	0.00	0.00	0.00	0.17	1.66	0.73	0.10	0.78	-7.01E+00	6.21E-01		-7.01E+00	6.34E-01		7.40E+00	6.16E-01		1.07E+01	4.19E-04	Up	-2.82E+00	8.87E-02		1.07E-01	9.46E-01								
Unigene105414_All	419	3.12	0.24	1.40	4.16	3.21	0.86	0.61	0.20	0.00	-3.69E+00	1.09E-05	Down	-1.16E+00	6.45E-02		-3.77E-01	4.65E-01		-2.27E+00	4.53E-05	Down	-1.60E+00	3.11E-01		-9.24E+00	3.06E-02		Unigene105414_All//gi|30678256|ref|NP_171720.2|//2.00E-08//ferredoxin-related [Arabidopsis thaliana]	Unigene105414_All//6.00E-09//AT1G02180| ferredoxin-related	Unigene105414_All//6.00E-90//BG440260				nr +
Unigene10542_All	491	0.00	0.00	0.00	0.00	0.00	0.00	5.43	1.45	3.25	null	null		null	null		null	null		null	null		-1.91E+00	6.07E-07	Down	-7.41E-01	2.52E-02				Unigene10542_All//2.00E-12//DR461796				

Unigene105426_All	754	0.07	1.21	0.57	0.26	0.27	0.78	0.67	0.11	0.11	4.12E+00	2.34E-04	Up	3.03E+00	5.74E-02		5.20E-02	9.89E-01		1.56E+00	1.36E-01		-2.60E+00	2.05E-02		-2.67E+00	1.38E-02		Unigene105426_All//gi|226499346|ref|NP_001149805.1|//1.00E-47//CUE domain containing protein [Zea mays] >gi|195634781|gb|ACG36859.1| CUE domain containing protein [Zea mays]	Unigene105426_All//6.00E-35//AT5G32440| LOCATED IN: chloroplast; EXPRESSED IN: 24 plant structures; EXPRESSED DURING: 15 growth stages; CONTAINS InterPro DOMAIN/s: Ubiquitin system component Cue (InterPro:IPR003892); BEST Arabidopsis thaliana protein match is: unknown protein (TAIR:AT1G80040.1); Has 303 Blast hits to 289 proteins in 67 species: Archae - 0; Bacteria - 2; Metazoa - 74; Fungi - 21; Plants - 93; Viruses - 0; Other Eukaryotes - 113 (source: NCBI BLink).	Unigene105426_All//0.00E+00//gi|21097417|gb|BQ409730.1|BQ409730			GO:0043231	nr +
Unigene10543_All	318	0.00	0.00	0.00	0.00	0.00	0.00	3.19	0.26	0.75	null	null		null	null		null	null		null	null		-3.60E+00	2.38E-05	Down	-2.09E+00	1.53E-03								
Unigene105439_All	454	6.57	23.77	11.26	23.59	15.02	16.31	9.22	8.75	7.55	1.86E+00	2.37E-20	Up	7.78E-01	4.78E-03		-6.52E-01	5.09E-05		-5.32E-01	1.94E-03		-7.61E-02	7.94E-01		-2.89E-01	2.89E-01				Unigene105439_All//2.00E-71//gi|164311793|gb|ES851073.1|ES851073				
Unigene10544_All	212	0.00	1.67	0.50	3.99	0.32	1.49	1.20	0.79	0.75	1.07E+01	2.47E-02		8.97E+00	4.03E-01		-3.62E+00	1.47E-04	Down	-1.42E+00	7.34E-02		-6.02E-01	6.48E-01		-6.71E-01	6.06E-01								
Unigene105450_All	482	3.15	0.84	0.33	2.07	0.29	1.22	2.28	0.87	1.41	-1.90E+00	1.25E-03		-3.25E+00	1.40E-05	Down	-2.85E+00	2.37E-04	Down	-7.63E-01	2.89E-01		-1.40E+00	2.04E-02		-6.99E-01	2.11E-01		Unigene105450_All//gi|222424070|dbj|BAH19995.1|//5.00E-19//AT4G28760 [Arabidopsis thaliana]	Unigene105450_All//7.00E-12//AT4G28760| unknown protein	Unigene105450_All//7.00E-59//gi|78343612|gb|DT563886.1|DT563886			GO:0005488	nr -
Unigene105454_All	484	0.43	0.00	0.00	0.00	0.64	1.31	0.09	0.00	0.00	-8.76E+00	1.23E-01		-8.76E+00	1.47E-01		9.32E+00	4.55E-02		1.04E+01	7.60E-04	Up	-6.45E+00	6.64E-01		-6.45E+00	6.18E-01		Unigene105454_All//gi|225458882|ref|XP_002283431.1|//4.00E-09//PREDICTED: hypothetical protein [Vitis vinifera] >gi|302142175|emb|CBI19378.3| unnamed protein product [Vitis vinifera]	Unigene105454_All//6.00E-06//AT1G10140| FUNCTIONS IN: molecular_function unknown; INVOLVED IN: biological_process unknown; LOCATED IN: plasma membrane; EXPRESSED IN: 23 plant structures; EXPRESSED DURING: 13 growth stages; CONTAINS InterPro DOMAIN/s: Uncharacterised conserved protein UCP031279 (InterPro:IPR016972); BEST Arabidopsis thaliana protein match is: unknown protein (TAIR:AT1G58420.1); Has 38 Blast hits to 38 proteins in 10 species: Archae - 0; Bacteria - 0; Metazoa - 0; Fungi - 0; Plants - 33; Viruses - 0; Other Eukaryotes - 5 (source: NCBI BLink).	Unigene105454_All//0.00E+00//DW512687				nr +
Unigene105469_All	585	0.72	0.78	0.46	0.43	0.82	1.62	0.94	0.00	0.14	1.24E-01	9.11E-01		-6.53E-01	5.94E-01		9.53E-01	4.24E-01		1.93E+00	1.48E-02		-9.88E+00	4.62E-04	Down	-2.79E+00	7.97E-03		Unigene105469_All//gi|18406084|ref|NP_565988.1|//9.00E-20//PAR1 (PHY RAPIDLY REGULATED 1) [Arabidopsis thaliana] >gi|4512668|gb|AAD21722.1| expressed protein [Arabidopsis thaliana] >gi|83776541|tpe|CAJ55345.1| TPA: helix-loop-helix protein [Arabidopsis thaliana]	Unigene105469_All//4.00E-17//AT2G42870| PAR1 (PHY RAPIDLY REGULATED 1)	Unigene105469_All//4.00E-119//TC160093				nr +
Unigene10547_All	230	0.00	6.17	2.08	0.00	0.00	0.00	0.00	0.00	0.00	1.26E+01	4.06E-08	Up	1.10E+01	7.50E-03		null	null		null	null		null	null		null	null								ESTscan -
Unigene105471_All	605	1.38	1.42	1.76	2.55	1.42	1.34	2.10	0.35	2.11	4.12E-02	9.74E-01		3.47E-01	6.29E-01		-8.43E-01	1.01E-01		-9.26E-01	8.58E-02		-2.60E+00	5.14E-05	Down	7.30E-03	9.89E-01		Unigene105471_All//gi|240254425|ref|NP_178255.4|//7.00E-42//ATP binding / kinase [Arabidopsis thaliana]	Unigene105471_All//4.00E-43//AT2G01460| ATP binding / kinase	Unigene105471_All//0.00E+00//ES849346				nr +
Unigene105490_All	200	1.31	2.03	3.46	1.00	1.55	6.10	1.27	1.67	3.98	6.32E-01	6.01E-01		1.40E+00	1.39E-01		6.37E-01	6.95E-01		2.61E+00	3.93E-04	Up	3.98E-01	7.24E-01		1.65E+00	2.32E-02				Unigene105490_All//5.00E-107//TC170854				
Unigene1055_All	367	4.28	1.52	4.21	13.98	15.95	16.61	3.80	2.73	1.09	-1.49E+00	6.73E-03		-2.35E-02	9.87E-01		1.90E-01	5.20E-01		2.49E-01	3.65E-01		-4.76E-01	3.74E-01		-1.81E+00	6.10E-04	Down	Unigene1055_All//gi|85069287|gb|ABC69715.1|//1.00E-35//auxin response factor 4 [Solanum lycopersicum]	Unigene1055_All//1.00E-22//AT5G60450| ARF4 (AUXIN RESPONSE FACTOR 4); transcription factor	Unigene1055_All//6.00E-142//gi|48807809|gb|CO109123.1|CO109123	ARF		GO:0001708//GO:0005488//GO:0006350//GO:0009725//GO:0009791//GO:0010468	nr +
Unigene105501_All	200	31.65	11.15	13.31	61.52	56.65	58.94	42.70	13.79	20.12	-1.51E+00	2.36E-09	Down	-1.25E+00	7.17E-07	Down	-1.19E-01	5.52E-01		-6.19E-02	7.36E-01		-1.63E+00	9.33E-17	Down	-1.09E+00	1.11E-09	Down			Unigene105501_All//3.00E-96//gi|13248510|gb|BF277159.2|BF277159				
Unigene10553_All	264	0.00	0.00	0.00	9.06	3.39	1.03	0.80	0.00	0.00	null	null		null	null		-1.42E+00	3.12E-04	Down	-3.14E+00	2.53E-09	Down	-9.65E+00	7.48E-02		-9.65E+00	5.84E-02		Unigene10553_All//gi|186509725|ref|NP_001118563.1|//1.00E-11//WOX11 (WUSCHEL related homeobox 11); DNA binding / transcription factor [Arabidopsis thaliana]	Unigene10553_All//5.00E-13//AT3G03660| WOX11 (WUSCHEL related homeobox 11); DNA binding / transcription factor	Unigene10553_All//7.00E-110//TC173647	HB		GO:0001071//GO:0003677//GO:0006351//GO:0009536//GO:0010468	nr -
Unigene10554_All	284	0.00	0.00	0.00	7.19	2.06	1.27	0.15	0.00	0.14	null	null		null	null		-1.80E+00	5.35E-05	Down	-2.50E+00	1.87E-06	Down	-7.22E+00	6.64E-01		-8.58E-02	9.74E-01								
Unigene10555_All	394	0.00	12.86	6.76	0.00	0.00	0.23	0.00	0.00	0.00	1.37E+01	6.88E-29	Up	1.27E+01	1.07E-14	Up	null	null		7.84E+00	4.81E-01		null	null		null	null		Unigene10555_All//gi|14423905|sp|Q9XGL4.1|RL31_CYAPA//1.00E-44//RecName: Full=60S ribosomal protein L31 >gi|5123727|emb|CAB45375.1| ribosomal protein L31 [Cyanophora paradoxa]	Unigene10555_All//1.00E-29//AT4G26230| 60S ribosomal protein L31 (RPL31B)			ko03010|Ribosome	GO:0005198//GO:0010467//GO:0030529//GO:0044267	nr +
Unigene10556_All	344	0.00	1.03	0.31	0.00	0.60	0.13	0.49	2.92	4.40	1.00E+01	2.47E-02		8.27E+00	4.03E-01		9.23E+00	1.57E-01		7.04E+00	7.03E-01		2.57E+00	4.79E-04	Up	3.16E+00	4.04E-07	Up	Unigene10556_All//gi|30679545|ref|NP_179190.2|//2.00E-13//phox (PX) domain-containing protein [Arabidopsis thaliana]	Unigene10556_All//7.00E-15//AT2G15900| phox (PX) domain-containing protein					nr +
Unigene105576_All	203	0.00	0.75	0.00	0.00	0.00	0.45	1.46	4.74	10.01	9.55E+00	2.37E-01		null	null		null	null		8.80E+00	4.81E-01		1.70E+00	1.17E-02		2.78E+00	2.59E-08	Up							
Unigene10558_All	422	0.62	3.96	1.26	2.83	2.28	1.28	0.00	0.00	0.00	2.68E+00	1.99E-05	Up	1.03E+00	3.42E-01		-3.10E-01	6.07E-01		-1.14E+00	7.15E-02		null	null		null	null		Unigene10558_All//gi|13877599|gb|AAK43877.1|AF370500_1//2.00E-32//Unknown protein [Arabidopsis thaliana] >gi|28059703|gb|AAO30085.1| Unknown protein [Arabidopsis thaliana]	Unigene10558_All//1.00E-33//AT2G40150| INVOLVED IN: biological_process unknown; EXPRESSED IN: 21 plant structures; EXPRESSED DURING: 13 growth stages; CONTAINS InterPro DOMAIN/s: Protein of unknown function DUF231, plant (InterPro:IPR004253); BEST Arabidopsis thaliana protein match is: ESK1 (ESKIMO 1) (TAIR:AT3G55990.1); Has 712 Blast hits to 693 proteins in 16 species: Archae - 0; Bacteria - 0; Metazoa - 0; Fungi - 0; Plants - 712; Viruses - 0; Other Eukaryotes - 0 (source: NCBI BLink).	Unigene10558_All//7.00E-68//EE592863			GO:0006970//GO:0009409//GO:0010557	nr +
Unigene105591_All	203	61.08	29.20	32.53	74.11	66.50	120.36	70.81	54.77	36.50	-1.06E+00	1.40E-10	Down	-9.09E-01	6.29E-08		-1.56E-01	3.32E-01		7.00E-01	8.88E-11		-3.71E-01	5.57E-03		-9.56E-01	6.86E-13		Unigene105591_All//gi|13877126|gb|AAK43709.1|AF358665_1//7.00E-07//ribosomal protein L32 [Mercurialis annua] >gi|13899053|gb|AAK48948.1|AF370436_1 ribosomal protein L32 [Mercurialis annua] >gi|13899055|gb|AAK48949.1|AF370437_1 ribosomal protein L32 [Mercurialis annua] >gi|13899057|gb|AAK48950.1|AF370438_1 ribosomal protein L32 [Mercurialis annua]		Unigene105591_All//5.00E-70//TC152244		ko03010|Ribosome		nr +
Unigene1056_All	3063	9.60	5.14	4.17	28.02	29.16	36.67	7.73	4.64	3.00	-9.00E-01	2.29E-18		-1.20E+00	5.95E-28	Down	5.76E-02	4.00E-01		3.88E-01	1.13E-16		-7.37E-01	3.90E-13		-1.36E+00	8.27E-36	Down	Unigene1056_All//gi|85069287|gb|ABC69715.1|//0.00E+00//auxin response factor 4 [Solanum lycopersicum]	Unigene1056_All//0.00E+00//AT5G60450| ARF4 (AUXIN RESPONSE FACTOR 4); transcription factor	Unigene1056_All//0.00E+00//gi|48807809|gb|CO109123.1|CO109123	ARF		GO:0001071//GO:0001708//GO:0003676//GO:0005515//GO:0006351//GO:0009755//GO:0009791//GO:0010468//GO:0043231	nr +
Unigene10562_All	1852	0.00	0.22	0.23	0.27	0.37	0.54	0.23	0.43	3.61	7.77E+00	1.36E-02		7.85E+00	1.39E-02		4.67E-01	6.12E-01		9.96E-01	1.72E-01		9.09E-01	2.11E-01		3.98E+00	2.69E-35	Up	Unigene10562_All//gi|195619326|gb|ACG31493.1|//3.00E-18//harpin-induced protein [Zea mays]	Unigene10562_All//2.00E-14//AT2G01080| FUNCTIONS IN: molecular_function unknown; INVOLVED IN: biological_process unknown; LOCATED IN: plasma membrane; EXPRESSED IN: 14 plant structures; EXPRESSED DURING: 6 growth stages; CONTAINS InterPro DOMAIN/s: Harpin-induced 1 (InterPro:IPR010847); BEST Arabidopsis thaliana protein match is: unknown protein (TAIR:AT3G54200.1); Has 388 Blast hits to 387 proteins in 21 species: Archae - 0; Bacteria - 0; Metazoa - 0; Fungi - 2; Plants - 386; Viruses - 0; Other Eukaryotes - 0 (source: NCBI BLink).	Unigene10562_All//6.00E-10//TC149098				nr -
Unigene105620_All	204	0.00	1.49	0.00	0.00	1.01	3.54	0.00	0.00	0.20	1.05E+01	4.45E-02		null	null		9.98E+00	1.57E-01		1.18E+01	2.31E-04	Up	null	null		7.61E+00	6.48E-01								
Unigene10563_All	339	1.23	0.90	1.26	2.65	2.64	0.40	2.62	0.74	0.35	-4.61E-01	6.50E-01		2.54E-02	1.00E+00		-2.10E-03	9.78E-01		-2.73E+00	2.06E-03		-1.82E+00	1.08E-02		-2.89E+00	3.05E-04	Down	Unigene10563_All//gi|297818068|ref|XP_002876917.1|//8.00E-27//nsp-interacting kinase 2 [Arabidopsis lyrata subsp. lyrata] >gi|297322755|gb|EFH53176.1| nsp-interacting kinase 2 [Arabidopsis lyrata subsp. lyrata]	Unigene10563_All//3.00E-24//AT3G25560| NIK2 (NSP-INTERACTING KINASE 2); ATP binding / protein binding / protein kinase/ protein serine/threonine kinase			ko04626|Plant-pathogen interaction	GO:0004672//GO:0006464//GO:0031224//GO:0031410//GO:0032559	nr +
Unigene105633_All	204	2.31	4.97	9.14	9.03	5.06	5.76	1.24	0.41	1.76	1.11E+00	1.13E-01		1.98E+00	3.17E-04	Up	-8.35E-01	7.02E-02		-6.50E-01	1.92E-01		-1.60E+00	3.11E-01		4.99E-01	6.28E-01		Unigene105633_All//gi|224130518|ref|XP_002328629.1|//1.00E-10//tir-nbs-lrr resistance protein [Populus trichocarpa] >gi|222838611|gb|EEE76976.1| tir-nbs-lrr resistance protein [Populus trichocarpa]		Unigene105633_All//2.00E-63//TC135081				nr +
Unigene105635_All	204	46.68	79.48	44.90	63.98	75.96	97.63	195.86	261.02	83.19	7.68E-01	3.72E-08		-5.61E-02	7.81E-01		2.48E-01	9.59E-02		6.10E-01	3.98E-07		4.14E-01	1.27E-10		-1.24E+00	1.92E-51	Down	Unigene105635_All//gi|38194890|gb|AAR13288.1|//4.00E-17//Anx1 [Gossypium hirsutum]	Unigene105635_All//1.00E-12//AT5G65020| ANNAT2 (Annexin Arabidopsis 2); calcium ion binding / calcium-dependent phospholipid binding	Unigene105635_All//2.00E-106//TC139742				nr -
Unigene10564_All	426	0.25	13.92	3.25	0.00	0.00	0.00	0.00	0.00	0.00	5.82E+00	1.27E-30	Up	3.73E+00	1.05E-05	Up	null	null		null	null		null	null		null	null		Unigene10564_All//gi|115441859|ref|NP_001045209.1|//3.00E-10//Os01g0919200 [Oryza sativa Japonica Group] >gi|57899841|dbj|BAD87625.1| fibroin heavy chain Fib-H-like [Oryza sativa Japonica Group] >gi|113534740|dbj|BAF07123.1| Os01g0919200 [Oryza sativa Japonica Group]				ko03040|Spliceosome		nr +
Unigene10568_All	276	0.00	0.37	0.00	2.35	0.12	1.15	0.31	0.91	0.43	8.52E+00	3.91E-01		null	null		-4.23E+00	5.94E-04	Down	-1.03E+00	2.68E-01		1.57E+00	3.27E-01		4.99E-01	8.09E-01								
Unigene10569_All	340	2.31	2.09	0.63	3.52	4.25	2.79	26.24	21.76	5.39	-1.46E-01	8.37E-01		-1.88E+00	4.13E-02		2.75E-01	6.54E-01		-3.34E-01	6.39E-01		-2.70E-01	1.41E-01		-2.28E+00	8.16E-28	Down			Unigene10569_All//3.00E-85//BQ402008				

<table>
<tr><th></th><th></th><th></th><th></th><th></th><th></th><th></th><th></th><th></th><th></th><th></th><th></th><th></th><th></th><th></th><th></th><th></th><th></th><th></th><th></th><th></th><th></th><th></th><th></th><th></th><th></th><th></th><th></th><th></th><th></th><th></th><th></th><th></th><th></th><th></th><th></th></tr>
<tr><td>Unigene1057_All</td><td>999</td><td>0.68</td><td>0.10</td><td>0.00</td><td>4.64</td><td>6.03</td><td>2.76</td><td>14.94</td><td>10.96</td><td>3.43</td><td>-2.75E+00</td><td>1.06E-02</td><td></td><td>-9.41E+00</td><td>7.65E-04</td><td>Down</td><td>3.79E-01</td><td>1.31E-01</td><td></td><td>-7.50E-01</td><td>6.84E-03</td><td></td><td>-4.47E-01</td><td>5.50E-04</td><td></td><td>-2.12E+00</td><td>4.64E-42</td><td>Down</td><td>Unigene1057_All//gi|224126613|ref|XP_002329598.1|//1.00E-108//cationic amino acid transporter [Populus trichocarpa] >gi|222870307|gb|EEF07438.1| cationic amino acid transporter [Populus trichocarpa]</td><td>Unigene1057_All//2.00E-63//AT5G04770| CAT6 (CATIONIC AMINO ACID TRANSPORTER 6); amino acid transmembrane transporter/ basic amino acid transmembrane transporter/ cationic amino acid transmembrane transporter</td><td>Unigene1057_All//5.00E-77//TC146944</td><td></td><td></td><td>GO:0006810//GO:0015171//GO:0016020//GO:0043231//GO:0051707</td><td>nr -</td></tr>
<tr><td>Unigene105701_All</td><td>207</td><td>21.99</td><td>8.81</td><td>14.15</td><td>15.64</td><td>13.31</td><td>14.18</td><td>44.73</td><td>9.49</td><td>8.66</td><td>-1.32E+00</td><td>7.66E-06</td><td>Down</td><td>-6.36E-01</td><td>3.20E-02</td><td></td><td>-2.33E-01</td><td>5.57E-01</td><td></td><td>-1.41E-01</td><td>6.92E-01</td><td></td><td>-2.24E+00</td><td>3.07E-27</td><td>Down</td><td>-2.37E+00</td><td>4.10E-30</td><td>Down</td><td></td><td></td><td>Unigene105701_All//1.00E-76//gi|21089134|gb|BQ401447.1|BQ401447</td><td></td><td></td><td></td><td></td></tr>
<tr><td>Unigene10572_All</td><td>301</td><td>0.00</td><td>0.00</td><td>0.00</td><td>0.17</td><td>0.00</td><td>0.00</td><td>3.09</td><td>1.67</td><td>0.40</td><td>null</td><td>null</td><td></td><td>null</td><td>null</td><td></td><td>-7.37E+00</td><td>5.64E-01</td><td></td><td>-7.37E+00</td><td>6.45E-01</td><td></td><td>-8.91E-01</td><td>1.72E-01</td><td></td><td>-2.96E+00</td><td>1.72E-04</td><td>Down</td><td>Unigene10572_All//gi|297810727|ref|XP_002873247.1|//3.00E-09//carboxy-lyase [Arabidopsis lyrata subsp. lyrata] >gi|297319084|gb|EFH49506.1| carboxy-lyase [Arabidopsis lyrata subsp. lyrata]</td><td>Unigene10572_All//2.00E-10//AT5G06300| carboxy-lyase</td><td></td><td></td><td></td><td></td><td>nr +</td></tr>
<tr><td>Unigene105733_All</td><td>209</td><td>3.25</td><td>5.33</td><td>4.33</td><td>0.95</td><td>2.31</td><td>2.81</td><td>1.01</td><td>3.40</td><td>10.29</td><td>7.13E-01</td><td>2.69E-01</td><td></td><td>4.12E-01</td><td>5.94E-01</td><td></td><td>1.27E+00</td><td>2.92E-01</td><td></td><td>1.56E+00</td><td>1.36E-01</td><td></td><td>1.75E+00</td><td>3.21E-02</td><td></td><td>3.35E+00</td><td>2.59E-10</td><td>Up</td><td></td><td></td><td>Unigene105733_All//3.00E-22//EX169656</td><td></td><td></td><td></td><td></td></tr>
<tr><td>Unigene105742_All</td><td>209</td><td>0.25</td><td>4.61</td><td>1.53</td><td>0.00</td><td>0.49</td><td>1.30</td><td>0.20</td><td>0.20</td><td>0.38</td><td>4.20E+00</td><td>1.29E-04</td><td>Up</td><td>2.61E+00</td><td>1.46E-01</td><td></td><td>8.95E+00</td><td>4.59E-01</td><td></td><td>1.03E+01</td><td>7.06E-02</td><td></td><td>-1.65E-02</td><td>1.01E+00</td><td></td><td>9.14E-01</td><td>7.52E-01</td><td></td><td>Unigene105742_All//gi|297803414|ref|XP_002869591.1|//2.00E-20//GDSL-motif lipase/hydrolase family protein [Arabidopsis lyrata subsp. lyrata] >gi|297315427|gb|EFH45850.1| GDSL-motif lipase/hydrolase family protein [Arabidopsis lyrata subsp. lyrata]</td><td>Unigene105742_All//8.00E-22//AT4G26790| GDSL-motif lipase/hydrolase family protein</td><td>Unigene105742_All//3.00E-108//CO090421</td><td></td><td></td><td></td><td>nr +</td></tr>
<tr><td>Unigene105744_All</td><td>209</td><td>65.34</td><td>36.12</td><td>30.83</td><td>98.91</td><td>74.47</td><td>105.02</td><td>242.55</td><td>117.19</td><td>98.17</td><td>-8.55E-01</td><td>2.82E-08</td><td></td><td>-1.08E+00</td><td>2.39E-11</td><td>Down</td><td>-4.10E-01</td><td>2.96E-04</td><td></td><td>8.64E-02</td><td>5.64E-01</td><td></td><td>-1.05E+00</td><td>7.81E-49</td><td>Down</td><td>-1.31E+00</td><td>8.69E-71</td><td>Down</td><td>Unigene105744_All//gi|73761683|gb|AAZ83341.1|//6.00E-36//ubiquitin extension protein [Gossypium hirsutum]</td><td>Unigene105744_All//1.00E-24//AT2G47110| UBQ6; protein binding</td><td>Unigene105744_All//2.00E-100//ES822113</td><td></td><td>ko03010|Ribosome</td><td></td><td>nr -</td></tr>
<tr><td>Unigene105748_All</td><td>209</td><td>0.25</td><td>2.18</td><td>0.00</td><td>0.00</td><td>0.49</td><td>3.67</td><td>0.00</td><td>0.20</td><td>0.19</td><td>3.12E+00</td><td>3.58E-02</td><td></td><td>-7.97E+00</td><td>6.33E-01</td><td></td><td>8.95E+00</td><td>4.59E-01</td><td></td><td>1.18E+01</td><td>1.27E-04</td><td>Up</td><td>7.64E+00</td><td>6.38E-01</td><td></td><td>7.57E+00</td><td>6.48E-01</td><td></td><td></td><td></td><td></td><td></td><td></td><td></td><td></td></tr>
<tr><td>Unigene105759_All</td><td>210</td><td>0.75</td><td>0.00</td><td>0.00</td><td>101.76</td><td>89.53</td><td>41.08</td><td>0.40</td><td>0.60</td><td>0.00</td><td>-9.55E+00</td><td>2.17E-01</td><td></td><td>-9.55E+00</td><td>2.46E-01</td><td></td><td>-1.85E-01</td><td>1.48E-01</td><td></td><td>-1.31E+00</td><td>4.01E-26</td><td>Down</td><td>5.68E-01</td><td>7.83E-01</td><td></td><td>-8.65E+00</td><td>3.61E-01</td><td></td><td>Unigene105759_All//gi|186510343|ref|NP_001078202.2|//1.00E-26//LSH4 (LIGHT SENSITIVE HYPOCOTYLS 4) [Arabidopsis thaliana]</td><td>Unigene105759_All//6.00E-28//AT3G23290| LSH4 (LIGHT SENSITIVE HYPOCOTYLS 4)</td><td>Unigene105759_All//2.00E-103//TC160625</td><td></td><td></td><td></td><td>nr +</td></tr>
<tr><td>Unigene105761_All</td><td>210</td><td>0.75</td><td>0.00</td><td>0.00</td><td>0.00</td><td>0.16</td><td>3.01</td><td>0.40</td><td>0.20</td><td>0.00</td><td>-9.55E+00</td><td>2.17E-01</td><td></td><td>-9.55E+00</td><td>2.46E-01</td><td></td><td>7.36E+00</td><td>8.40E-01</td><td></td><td>1.16E+01</td><td>7.60E-04</td><td>Up</td><td>-1.02E+00</td><td>7.27E-01</td><td></td><td>-8.65E+00</td><td>3.61E-01</td><td></td><td></td><td></td><td>Unigene105761_All//7.00E-91//AI728016</td><td></td><td></td><td></td><td>ESTscan +</td></tr>
<tr><td>Unigene105767_All</td><td>210</td><td>1.00</td><td>1.93</td><td>4.82</td><td>0.47</td><td>1.80</td><td>6.45</td><td>0.00</td><td>0.60</td><td>1.52</td><td>9.54E-01</td><td>4.14E-01</td><td></td><td>2.27E+00</td><td>5.77E-03</td><td></td><td>1.93E+00</td><td>1.94E-01</td><td></td><td>3.77E+00</td><td>4.57E-06</td><td>Up</td><td>9.22E+00</td><td>2.41E-01</td><td></td><td>1.06E+01</td><td>1.34E-02</td><td></td><td></td><td></td><td></td><td></td><td></td><td></td><td></td></tr>
<tr><td>Unigene105773_All</td><td>211</td><td>20.09</td><td>5.04</td><td>6.81</td><td>12.75</td><td>10.93</td><td>33.18</td><td>58.91</td><td>27.73</td><td>19.26</td><td>-1.99E+00</td><td>2.96E-09</td><td>Down</td><td>-1.56E+00</td><td>1.86E-06</td><td>Down</td><td>-2.22E-01</td><td>5.93E-01</td><td></td><td>1.38E+00</td><td>1.72E-09</td><td>Up</td><td>-1.09E+00</td><td>2.80E-13</td><td>Down</td><td>-1.61E+00</td><td>1.95E-24</td><td>Down</td><td></td><td></td><td>Unigene105773_All//9.00E-41//gi|78330471|gb|DT550745.1|DT550745</td><td></td><td></td><td></td><td></td></tr>
<tr><td>Unigene105778_All</td><td>211</td><td>0.25</td><td>4.08</td><td>0.50</td><td>4.25</td><td>3.10</td><td>1.50</td><td>2.40</td><td>0.79</td><td>1.32</td><td>4.04E+00</td><td>4.20E-04</td><td>Up</td><td>1.03E+00</td><td>7.05E-01</td><td></td><td>-4.55E-01</td><td>5.58E-01</td><td></td><td>-1.50E+00</td><td>5.09E-02</td><td></td><td>-1.60E+00</td><td>1.07E-01</td><td></td><td>-8.63E-01</td><td>3.39E-01</td><td></td><td></td><td></td><td>Unigene105778_All//6.00E-14//ES803332</td><td></td><td></td><td></td><td></td></tr>
<tr><td>Unigene105779_All</td><td>211</td><td>0.00</td><td>0.24</td><td>0.00</td><td>0.00</td><td>0.33</td><td>4.28</td><td>0.20</td><td>0.00</td><td>0.57</td><td>7.91E+00</td><td>6.22E-01</td><td></td><td>null</td><td>null</td><td></td><td>8.35E+00</td><td>6.16E-01</td><td></td><td>1.21E+01</td><td>2.07E-05</td><td>Up</td><td>-7.65E+00</td><td>6.63E-01</td><td></td><td>1.50E+00</td><td>5.59E-01</td><td></td><td>Unigene105779_All//gi|297809467|ref|XP_002872617.1|//5.00E-14//pentatricopeptide repeat-containing protein [Arabidopsis lyrata subsp. lyrata] >gi|297318454|gb|EFH48876.1| pentatricopeptide repeat-containing protein [Arabidopsis lyrata subsp. lyrata]</td><td>Unigene105779_All//4.00E-15//AT4G11690| pentatricopeptide (PPR) repeat-containing protein</td><td></td><td></td><td></td><td></td><td>nr +</td></tr>
<tr><td>Unigene10579_All</td><td>235</td><td>0.00</td><td>0.22</td><td>0.00</td><td>0.21</td><td>0.59</td><td>0.19</td><td>9.00</td><td>2.85</td><td>5.26</td><td>7.75E+00</td><td>6.22E-01</td><td></td><td>null</td><td>null</td><td></td><td>1.47E+00</td><td>6.20E-01</td><td></td><td>-1.42E-01</td><td>9.57E-01</td><td></td><td>-1.66E+00</td><td>7.98E-05</td><td>Down</td><td>-7.76E-01</td><td>4.06E-02</td><td></td><td></td><td></td><td>Unigene10579_All//2.00E-38//DW480207</td><td></td><td></td><td></td><td></td></tr>
<tr><td>Unigene105797_All</td><td>212</td><td>0.00</td><td>1.43</td><td>0.25</td><td>2.11</td><td>0.16</td><td>2.13</td><td>312.91</td><td>200.30</td><td>856.91</td><td>1.05E+01</td><td>4.45E-02</td><td></td><td>7.97E+00</td><td>6.41E-01</td><td></td><td>-3.70E+00</td><td>1.07E-02</td><td></td><td>1.05E-02</td><td>1.00E+00</td><td></td><td>-6.44E-01</td><td>5.14E-28</td><td></td><td>1.45E+00</td><td>8.66E-294</td><td>Up</td><td>Unigene105797_All//gi|40218371|gb|AAR83118.1|//8.00E-38//secretory laccase [Gossypium arboreum]</td><td>Unigene105797_All//7.00E-23//AT5G48100| TT10 (TRANSPARENT TESTA 10); copper ion binding / laccase</td><td>Unigene105797_All//6.00E-116//TC137114</td><td></td><td>ko00053|Ascorbate and aldarate metabolism</td><td></td><td>nr +</td></tr>
<tr><td>Unigene1058_All</td><td>2372</td><td>0.66</td><td>0.90</td><td>0.81</td><td>13.00</td><td>10.12</td><td>9.90</td><td>16.81</td><td>7.47</td><td>5.29</td><td>4.39E-01</td><td>3.33E-01</td><td></td><td>2.89E-01</td><td>5.72E-01</td><td></td><td>-3.62E-01</td><td>6.65E-05</td><td></td><td>-3.93E-01</td><td>3.44E-05</td><td></td><td>-1.17E+00</td><td>8.03E-46</td><td>Down</td><td>-1.67E+00</td><td>3.36E-80</td><td>Down</td><td>Unigene1058_All//gi|224137956|ref|XP_002326482.1|//0.00E+00//cationic amino acid transporter [Populus trichocarpa] >gi|222833804|gb|EEE72281.1| cationic amino acid transporter [Populus trichocarpa]</td><td>Unigene1058_All//2.00E-128//AT5G04770| CAT6 (CATIONIC AMINO ACID TRANSPORTER 6); amino acid transmembrane transporter/ basic amino acid transmembrane transporter/ cationic amino acid transmembrane transporter</td><td>Unigene1058_All//6.00E-60//TC146944</td><td></td><td></td><td>GO:0005275//GO:0015837//GO:0031224//GO:0043231</td><td>nr -</td></tr>
<tr><td>Unigene10580_All</td><td>287</td><td>55.60</td><td>550.48</td><td>210.59</td><td>33.33</td><td>81.95</td><td>117.08</td><td>29.46</td><td>78.20</td><td>120.21</td><td>3.31E+00</td><td>0.00E+00</td><td>Up</td><td>1.92E+00</td><td>1.20E-113</td><td>Up</td><td>1.30E+00</td><td>1.60E-30</td><td>Up</td><td>1.81E+00</td><td>2.28E-64</td><td>Up</td><td>1.41E+00</td><td>1.49E-34</td><td>Up</td><td>2.03E+00</td><td>1.50E-89</td><td>Up</td><td>Unigene10580_All//gi|210062232|gb|ACJ06165.1|//7.00E-18//HVA22E [Arabidopsis thaliana]</td><td>Unigene10580_All//3.00E-19//AT5G50720| ATHVA22E</td><td>Unigene10580_All//7.00E-104//TC166347</td><td></td><td></td><td>GO:0031224//GO:0050896</td><td>nr -</td></tr>
<tr><td>Unigene105815_All</td><td>213</td><td>259.39</td><td>338.03</td><td>364.00</td><td>402.26</td><td>333.20</td><td>396.50</td><td>311.64</td><td>248.81</td><td>130.36</td><td>3.82E-01</td><td>4.63E-10</td><td></td><td>4.89E-01</td><td>6.69E-16</td><td></td><td>-2.72E-01</td><td>1.46E-07</td><td></td><td>-2.08E-02</td><td>7.64E-01</td><td></td><td>-3.25E-01</td><td>1.41E-08</td><td></td><td>-1.26E+00</td><td>2.90E-87</td><td>Down</td><td>Unigene105815_All//gi|225427492|ref|XP_002263421.1|//3.00E-13//PREDICTED: similar to Os03g0576600 [Vitis vinifera]</td><td></td><td>Unigene105815_All//3.00E-102//gi|84164696|gb|DR453344.1|DR453344</td><td></td><td></td><td></td><td>nr +</td></tr>
<tr><td>Unigene105816_All</td><td>213</td><td>2899.75</td><td>970.33</td><td>3168.25</td><td>428.68</td><td>469.32</td><td>300.24</td><td>0.00</td><td>0.00</td><td>0.00</td><td>-1.58E+00</td><td>0.00E+00</td><td>Down</td><td>1.28E-01</td><td>6.28E-11</td><td></td><td>1.31E-01</td><td>1.34E-02</td><td></td><td>-5.14E-01</td><td>1.18E-22</td><td></td><td>null</td><td>null</td><td></td><td>null</td><td>null</td><td></td><td></td><td></td><td>Unigene105816_All//2.00E-69//gi|21093041|gb|BQ405354.1|BQ405354</td><td></td><td></td><td></td><td></td></tr>
<tr><td>Unigene10584_All</td><td>274</td><td>0.57</td><td>1.11</td><td>0.39</td><td>2.18</td><td>4.02</td><td>4.45</td><td>4.17</td><td>0.31</td><td>0.44</td><td>9.54E-01</td><td>5.04E-01</td><td></td><td>-5.60E-01</td><td>7.77E-01</td><td></td><td>8.82E-01</td><td>1.94E-01</td><td></td><td>1.03E+00</td><td>1.07E-01</td><td></td><td>-3.77E+00</td><td>3.90E-06</td><td>Down</td><td>-3.26E+00</td><td>8.94E-06</td><td>Down</td><td></td><td></td><td></td><td></td><td></td><td></td><td></td></tr>
<tr><td>Unigene105840_All</td><td>215</td><td>0.97</td><td>2.59</td><td>0.99</td><td>3.71</td><td>4.00</td><td>15.75</td><td>4.33</td><td>4.28</td><td>11.12</td><td>1.41E+00</td><td>1.69E-01</td><td></td><td>2.54E-02</td><td>1.00E+00</td><td></td><td>1.11E-01</td><td>9.04E-01</td><td></td><td>2.09E+00</td><td>3.11E-08</td><td>Up</td><td>-1.66E-02</td><td>1.02E+00</td><td></td><td>1.36E+00</td><td>2.45E-04</td><td>Up</td><td>Unigene105840_All//gi|15233476|ref|NP_192343.1|//2.00E-21//early-responsive to dehydration protein-related / ERD protein-related [Arabidopsis thaliana] >gi|30679712|ref|NP_849296.1| early-responsive to dehydration protein-related / ERD protein-related [Arabidopsis thaliana] >gi|30679715|ref|NP_849297.1| early-responsive to dehydration protein-related / ERD protein-related [Arabidopsis thaliana]</td><td>Unigene105840_All//5.00E-23//AT1G11960| FUNCTIONS IN: molecular_function unknown; INVOLVED IN: biological_process unknown; LOCATED IN: plasma membrane; EXPRESSED IN: 22 plant structures; EXPRESSED DURING: 13 growth stages; CONTAINS InterPro DOMAIN/s: Protein of unknown function DUF221 (InterPro:IPR003864); BEST Arabidopsis thaliana protein match is: early-responsive to dehydration protein-related / ERD protein-related (TAIR:AT1G62320.1); Has 746 Blast hits to 706 proteins in 111 species: Archae - 0; Bacteria - 4; Metazoa - 131; Fungi - 370; Plants - 224; Viruses - 0; Other Eukaryotes - 17 (source: NCBI BLink).</td><td>Unigene105840_All//1.00E-36//TC131090</td><td></td><td></td><td></td><td>nr -</td></tr>
<tr><td>Unigene105841_All</td><td>215</td><td>8.76</td><td>5.42</td><td>1.24</td><td>18.54</td><td>14.25</td><td>15.33</td><td>8.65</td><td>5.64</td><td>6.67</td><td>-6.93E-01</td><td>1.39E-01</td><td></td><td>-2.82E+00</td><td>5.01E-06</td><td>Down</td><td>-3.79E-01</td><td>2.27E-01</td><td></td><td>-2.74E-01</td><td>4.20E-01</td><td></td><td>-6.18E-01</td><td>1.52E-01</td><td></td><td>-3.75E-01</td><td>3.77E-01</td><td></td><td></td><td></td><td>Unigene105841_All//3.00E-99//TC132167</td><td></td><td></td><td></td><td></td></tr>
<tr><td>Unigene105844_All</td><td>215</td><td>0.00</td><td>0.00</td><td>0.50</td><td>0.00</td><td>0.16</td><td>9.45</td><td>0.00</td><td>0.00</td><td>0.00</td><td>null</td><td>null</td><td></td><td>8.95E+00</td><td>4.02E-01</td><td></td><td>7.32E+00</td><td>8.40E-01</td><td></td><td>1.32E+01</td><td>3.89E-12</td><td>Up</td><td>null</td><td>null</td><td></td><td>null</td><td>null</td><td></td><td>Unigene105844_All//sp|Q9U8M0|VIT1_PERAM//2.00E-11//Vitellogenin-1 OS=Periplaneta americana GN=VG2 PE=2 SV=1</td><td></td><td></td><td></td><td></td><td></td><td>swissprot +</td></tr>
<tr><td>Unigene105859_All</td><td>216</td><td>0.00</td><td>0.00</td><td>0.00</td><td>0.00</td><td>0.64</td><td>2.93</td><td>0.20</td><td>0.19</td><td>0.00</td><td>null</td><td>null</td><td></td><td>null</td><td>null</td><td></td><td>9.32E+00</td><td>3.20E-01</td><td></td><td>1.15E+01</td><td>7.60E-04</td><td>Up</td><td>-1.63E-02</td><td>1.01E+00</td><td></td><td>-7.61E+00</td><td>6.18E-01</td><td></td><td>Unigene105859_All//gi|15226939|ref|NP_178341.1|//3.00E-10//AtPP2-B10 (Phloem protein 2-B10); carbohydrate binding [Arabidopsis thaliana] >gi|75268076|sp|Q9ZVQ6.1|P2B10_ARATH RecName: Full=F-box protein PP2-B10; AltName: Full=Protein PHLOEM PROTEIN 2-LIKE B10; Short=AtPP2-B10 >gi|107738391|gb|ABF83689.1| At2g02360 [Arabidopsis thaliana]</td><td>Unigene105859_All//1.00E-11//AT2G02360| AtPP2-B10 (Phloem protein 2-B10); carbohydrate binding</td><td>Unigene105859_All//7.00E-54//gi|109843286|gb|DW241844.1|DW241844</td><td></td><td></td><td></td><td>nr -</td></tr>
<tr><td>Unigene10586_All</td><td>430</td><td>0.00</td><td>0.35</td><td>0.00</td><td>1.16</td><td>1.04</td><td>0.11</td><td>1.18</td><td>0.00</td><td>0.56</td><td>8.47E+00</td><td>2.37E-01</td><td></td><td>null</td><td>null</td><td></td><td>-1.54E-01</td><td>8.50E-01</td><td></td><td>-3.46E+00</td><td>1.61E-02</td><td></td><td>-1.02E+01</td><td>8.91E-04</td><td>Down</td><td>-1.09E+00</td><td>2.28E-01</td><td></td><td>Unigene10586_All//gi|297721755|ref|NP_001173241.1|//2.00E-75//Os03g0119966 [Oryza sativa Japonica Group] >gi|27452910|gb|AAO15294.1| Hypothetical protein [Oryza sativa Japonica Group] >gi|217415974|dbj|BAH03170.1| NAC protein [Oryza sativa Japonica Group] >gi|255674164|dbj|BAH91969.1| Os03g0119966 [Oryza sativa Japonica Group]</td><td>Unigene10586_All//4.00E-75//AT1G65910| anac028 (Arabidopsis NAC domain containing protein 28); transcription factor</td><td></td><td>NAC</td><td></td><td>GO:0001071//GO:0003676//GO:0010468//GO:0032501</td><td>nr +</td></tr>
<tr><td>Unigene105860_All</td><td>216</td><td>40.69</td><td>26.98</td><td>24.16</td><td>30.90</td><td>36.67</td><td>61.05</td><td>57.35</td><td>34.83</td><td>18.63</td><td>-5.93E-01</td><td>2.26E-03</td><td></td><td>-7.52E-01</td><td>1.99E-04</td><td></td><td>2.47E-01</td><td>2.86E-01</td><td></td><td>9.82E-01</td><td>2.18E-10</td><td></td><td>-7.20E-01</td><td>5.27E-07</td><td></td><td>-1.62E+00</td><td>1.50E-24</td><td>Down</td><td></td><td></td><td>Unigene105860_All//2.00E-94//TC156390</td><td></td><td></td><td></td><td></td></tr>
<tr><td>Unigene10588_All</td><td>313</td><td>199.42</td><td>160.26</td><td>218.27</td><td>6.37</td><td>14.74</td><td>4.47</td><td>0.00</td><td>0.00</td><td>0.00</td><td>-3.15E-01</td><td>2.01E-06</td><td></td><td>1.30E-01</td><td>6.83E-02</td><td></td><td>1.21E+00</td><td>9.95E-06</td><td>Up</td><td>-5.09E-01</td><td>2.94E-01</td><td></td><td>null</td><td>null</td><td></td><td>null</td><td>null</td><td></td><td></td><td></td><td>Unigene10588_All//4.00E-155//gi|164342758|gb|ES843169.1|ES843169</td><td></td><td></td><td></td><td></td></tr>
<tr><td>Unigene105883_All</td><td>217</td><td>126.82</td><td>328.53</td><td>286.37</td><td>92.28</td><td>150.91</td><td>83.46</td><td>0.00</td><td>0.96</td><td>0.00</td><td>1.37E+00</td><td>6.61E-85</td><td>Up</td><td>1.18E+00</td><td>1.22E-56</td><td>Up</td><td>7.10E-01</td><td>6.26E-16</td><td></td><td>-1.45E-01</td><td>3.13E-01</td><td></td><td>9.91E+00</td><td>7.87E-02</td><td></td><td>null</td><td>null</td><td></td><td></td><td></td><td>Unigene105883_All//5.00E-98//DW515441</td><td></td><td></td><td></td><td></td></tr>
<tr><td>Unigene10589_All</td><td>445</td><td>0.00</td><td>6.15</td><td>2.99</td><td>0.00</td><td>0.00</td><td>0.00</td><td>0.00</td><td>0.00</td><td>0.00</td><td>1.26E+01</td><td>1.44E-15</td><td>Up</td><td>1.15E+01</td><td>2.40E-07</td><td>Up</td><td>null</td><td>null</td><td></td><td>null</td><td>null</td><td></td><td>null</td><td>null</td><td></td><td>null</td><td>null</td><td></td><td></td><td></td><td></td><td></td><td></td><td></td><td></td></tr>
<tr><td>Unigene105896_All</td><td>218</td><td>764.65</td><td>322.84</td><td>795.09</td><td>112.65</td><td>160.49</td><td>55.31</td><td>0.19</td><td>0.38</td><td>0.55</td><td>-1.24E+00</td><td>1.36E-170</td><td>Down</td><td>5.63E-02</td><td>2.36E-01</td><td></td><td>5.11E-01</td><td>1.14E-09</td><td></td><td>-1.03E+00</td><td>1.88E-20</td><td>Down</td><td>9.84E-01</td><td>7.33E-01</td><td></td><td>1.50E+00</td><td>5.59E-01</td><td></td><td>Unigene105896_All//gi|1345698|sp|P27518.2|CB21_GOSHI//7.00E-28//RecName: Full=Chlorophyll a-b binding protein 151, chloroplastic; AltName: Full=LHCII type II CAB-151; Short=LHCP; Flags: Precursor >gi|452314|emb|CAA38025.1| chlorophyll ab binding protein [Gossypium hirsutum]</td><td>Unigene105896_All//3.00E-27//AT3G27690| LHCB2.3; chlorophyll binding</td><td>Unigene105896_All//4.00E-80//ES834749</td><td></td><td>ko00196|Photosynthesis - antenna proteins</td><td></td><td>nr +</td></tr>
<tr><td>Unigene1059_All</td><td>1930</td><td>0.03</td><td>0.11</td><td>0.00</td><td>2.01</td><td>1.39</td><td>1.26</td><td>5.87</td><td>3.03</td><td>1.67</td><td>1.95E+00</td><td>3.76E-01</td><td></td><td>-4.76E+00</td><td>6.33E-01</td><td></td><td>-5.33E-01</td><td>7.95E-02</td><td></td><td>-6.72E-01</td><td>3.04E-02</td><td></td><td>-9.53E-01</td><td>5.66E-10</td><td></td><td>-1.81E+00</td><td>3.45E-26</td><td>Down</td><td>Unigene1059_All//gi|224137956|ref|XP_002326482.1|//0.00E+00//cationic amino acid transporter [Populus trichocarpa] >gi|222833804|gb|EEE72281.1| cationic amino acid transporter [Populus trichocarpa]</td><td>Unigene1059_All//3.00E-142//AT5G04770| CAT6 (CATIONIC AMINO ACID TRANSPORTER 6); amino acid transmembrane transporter/ basic amino acid transmembrane transporter/ cationic amino acid transmembrane transporter</td><td>Unigene1059_All//9.00E-77//TC146944</td><td></td><td></td><td>GO:0005275//GO:0015837//GO:0031224//GO:0043231</td><td>nr +</td></tr>
<tr><td>Unigene105905_All</td><td>218</td><td>0.24</td><td>2.79</td><td>0.00</td><td>3.43</td><td>7.11</td><td>10.98</td><td>1.16</td><td>1.73</td><td>1.64</td><td>3.54E+00</td><td>7.25E-03</td><td></td><td>-7.91E+00</td><td>6.33E-01</td><td></td><td>1.05E+00</td><td>5.02E-02</td><td></td><td>1.68E+00</td><td>1.19E-04</td><td>Up</td><td>5.68E-01</td><td>6.38E-01</td><td></td><td>4.99E-01</td><td>6.28E-01</td><td></td><td></td><td></td><td></td><td></td><td></td><td></td><td></td></tr>
<tr><td>Unigene10591_All</td><td>373</td><td>0.84</td><td>1.36</td><td>6.00</td><td>3.34</td><td>2.22</td><td>2.66</td><td>0.34</td><td>0.67</td><td>0.11</td><td>6.91E-01</td><td>4.99E-01</td><td></td><td>2.83E+00</td><td>4.06E-07</td><td>Up</td><td>-5.92E-01</td><td>3.18E-01</td><td></td><td>-3.26E-01</td><td>6.41E-01</td><td></td><td>9.83E-01</td><td>5.18E-01</td><td></td><td>-1.67E+00</td><td>4.78E-01</td><td></td><td>Unigene10591_All//gi|50512300|gb|AAT77547.1|//6.00E-20//9A [Solanum pimpinellifolium]</td><td>Unigene10591_All//2.00E-17//AT3G11080| AtRLP35 (Receptor Like Protein 35); kinase/ protein binding</td><td></td><td></td><td></td><td></td><td>nr +</td></tr>
<tr><td>Unigene105912_All</td><td>219</td><td>83.38</td><td>23.60</td><td>83.16</td><td>19.79</td><td>20.28</td><td>8.46</td><td>0.00</td><td>0.00</td><td>0.18</td><td>-1.82E+00</td><td>2.44E-33</td><td>Down</td><td>-3.80E-03</td><td>1.00E+00</td><td></td><td>3.56E-02</td><td>9.25E-01</td><td></td><td>-1.23E+00</td><td>2.67E-05</td><td>Down</td><td>null</td><td>null</td><td></td><td>7.51E+00</td><td>6.48E-01</td><td></td><td></td><td></td><td>Unigene105912_All//3.00E-102//TC132557</td><td></td><td></td><td></td><td></td></tr>
<tr><td>Unigene105917_All</td><td>219</td><td>7.17</td><td>6.25</td><td>5.59</td><td>12.06</td><td>14.78</td><td>11.55</td><td>1.35</td><td>6.68</td><td>0.91</td><td>-1.98E-01</td><td>6.85E-01</td><td></td><td>-3.58E-01</td><td>5.20E-01</td><td></td><td>2.94E-01</td><td>4.54E-01</td><td></td><td>-6.20E-02</td><td>8.91E-01</td><td></td><td>2.31E+00</td><td>4.93E-05</td><td>Up</td><td>-5.71E-01</td><td>6.28E-01</td><td></td><td>Unigene105917_All//gi|195625106|gb|ACG34383.1|//2.00E-08//mannose-1-phosphate guanyltransferase [Zea mays]</td><td></td><td>Unigene105917_All//6.00E-73//TC166316</td><td></td><td></td><td></td><td>nr +</td></tr>
<tr><td>Unigene105924_All</td><td>219</td><td>0.00</td><td>0.00</td><td>0.00</td><td>0.00</td><td>0.16</td><td>12.17</td><td>0.00</td><td>0.00</td><td>0.00</td><td>null</td><td>null</td><td></td><td>null</td><td>null</td><td></td><td>7.30E+00</td><td>8.40E-01</td><td></td><td>1.36E+01</td><td>5.75E-16</td><td>Up</td><td>null</td><td>null</td><td></td><td>null</td><td>null</td><td></td><td></td><td></td><td></td><td></td><td></td><td></td><td>ESTscan -</td></tr>
<tr><td>Unigene105938_All</td><td>220</td><td>1.43</td><td>0.00</td><td>0.00</td><td>1.81</td><td>2.97</td><td>7.39</td><td>2.50</td><td>0.57</td><td>0.54</td><td>-1.05E+01</td><td>3.67E-02</td><td></td><td>-1.05E+01</td><td>4.93E-02</td><td></td><td>7.15E-01</td><td>4.65E-01</td><td></td><td>2.03E+00</td><td>3.98E-04</td><td>Up</td><td>-2.13E+00</td><td>3.32E-02</td><td></td><td>-2.20E+00</td><td>2.24E-02</td><td></td><td>Unigene105938_All//gi|297793507|ref|XP_002864638.1|//8.00E-27//protein-s-isoprenylcysteine O-methyltransferase [Arabidopsis lyrata subsp. lyrata] >gi|297310473|gb|EFH40897.1| protein-s-isoprenylcysteine O-methyltransferase [Arabidopsis lyrata subsp. lyrata]</td><td>Unigene105938_All//2.00E-28//AT5G59500| unknown protein</td><td>Unigene105938_All//2.00E-51//ES841908</td><td></td><td></td><td></td><td>nr +</td></tr>
<tr><td>Unigene10594_All</td><td>240</td><td>1.09</td><td>1.27</td><td>0.67</td><td>3.53</td><td>3.30</td><td>0.19</td><td>0.18</td><td>0.17</td><td>0.00</td><td>2.17E-01</td><td>8.65E-01</td><td></td><td>-7.12E-01</td><td>6.22E-01</td><td></td><td>-9.66E-02</td><td>8.77E-01</td><td></td><td>-4.23E+00</td><td>2.18E-04</td><td>Down</td><td>-1.73E-02</td><td>1.01E+00</td><td></td><td>-7.46E+00</td><td>6.18E-01</td><td></td><td></td><td></td><td>Unigene10594_All//1.00E-55//ES839899</td><td></td><td></td><td></td><td></td></tr>
</table>

<table>
<tr><td>Unigene105942_All</td><td>220</td><td>119.39</td><td>299.41</td><td>236.96</td><td>102.35</td><td>140.40</td><td>112.91</td><td>0.38</td><td>0.00</td><td>0.18</td><td>1.33E+00</td><td>1.79E-74</td><td>Up</td><td>9.89E-01</td><td>4.70E-36</td><td></td><td>4.56E-01</td><td>4.22E-07</td><td></td><td>1.42E-01</td><td>2.65E-01</td><td></td><td>-8.59E+00</td><td>4.07E-01</td><td></td><td>-1.09E+00</td><td>6.78E-01</td><td></td><td>Unigene105942_All//gi|15294284|gb|AAK95319.1|AF410333_1//5.00E-07//AT3g62730/F26K9_160 [Arabidopsis thaliana] >gi|20147275|gb|AAM10351.1|AT3g62730/F26K9_160 [Arabidopsis thaliana]</td><td></td><td>Unigene105942_All//4.00E-83//TC159115</td><td></td><td></td><td></td><td>nr -</td></tr>
<tr><td>Unigene105946_All</td><td>220</td><td>0.95</td><td>1.38</td><td>8.23</td><td>3.62</td><td>2.97</td><td>5.75</td><td>0.19</td><td>0.57</td><td>0.36</td><td>5.39E-01</td><td>6.72E-01</td><td></td><td>3.11E+00</td><td>2.28E-06</td><td>Up</td><td>-2.85E-01</td><td>7.21E-01</td><td></td><td>6.66E-01</td><td>2.99E-01</td><td></td><td>1.57E+00</td><td>5.46E-01</td><td></td><td>9.14E-01</td><td>7.51E-01</td><td></td><td></td><td></td><td></td><td></td><td></td><td></td><td>ESTscan +</td></tr>
<tr><td>Unigene105952_All</td><td>221</td><td>27.46</td><td>19.95</td><td>24.58</td><td>23.89</td><td>23.37</td><td>25.14</td><td>13.01</td><td>12.86</td><td>5.23</td><td>-4.61E-01</td><td>5.74E-02</td><td></td><td>-1.60E-01</td><td>5.64E-01</td><td></td><td>-3.18E-02</td><td>9.10E-01</td><td></td><td>7.32E-02</td><td>7.93E-01</td><td></td><td>-1.66E-02</td><td>9.95E-01</td><td></td><td>-1.32E+00</td><td>6.99E-05</td><td>Down</td><td></td><td></td><td>Unigene105952_All//7.00E-51//TC155713</td><td></td><td></td><td></td><td></td></tr>
<tr><td>Unigene105963_All</td><td>221</td><td>0.47</td><td>4.13</td><td>1.45</td><td>1.35</td><td>1.71</td><td>4.09</td><td>13.58</td><td>3.97</td><td>10.82</td><td>3.12E+00</td><td>1.14E-03</td><td></td><td>1.61E+00</td><td>3.05E-01</td><td></td><td>3.42E-01</td><td>8.36E-01</td><td></td><td>1.60E+00</td><td>4.34E-02</td><td></td><td>-1.77E+00</td><td>4.38E-07</td><td>Down</td><td>-3.29E-01</td><td>3.14E-01</td><td></td><td></td><td></td><td>Unigene105963_All//1.00E-67//gi|164369257|gb|ES850707.1|ES850707</td><td></td><td></td><td></td><td></td></tr>
<tr><td>Unigene10598_All</td><td>224</td><td>0.47</td><td>0.00</td><td>0.24</td><td>0.44</td><td>0.00</td><td>0.81</td><td>80.03</td><td>50.19</td><td>866.49</td><td>-8.87E+00</td><td>3.77E-01</td><td></td><td>-9.75E-01</td><td>7.22E-01</td><td></td><td>-8.80E+00</td><td>3.17E-01</td><td></td><td>8.59E-01</td><td>6.96E-01</td><td></td><td>-6.73E-01</td><td>8.76E-09</td><td></td><td>3.44E+00</td><td>0.00E+00</td><td>Up</td><td>Unigene10598_All//gi|3970689|emb|CAA77191.1|//1.00E-14//(+)-delta-cadinene synthase [Gossypium arboreum]</td><td></td><td>Unigene10598_All//3.00E-13//gi|73861227|gb|DT463966.1|DT463966</td><td></td><td></td><td>GO:0010334//GO:0046872</td><td>nr +</td></tr>
<tr><td>Unigene1060_All</td><td>1955</td><td>0.08</td><td>0.10</td><td>0.19</td><td>3.67</td><td>2.29</td><td>2.73</td><td>4.84</td><td>0.98</td><td>1.57</td><td>3.69E-01</td><td>8.17E-01</td><td></td><td>1.25E+00</td><td>3.66E-01</td><td></td><td>-6.80E-01</td><td>8.16E-04</td><td></td><td>-4.29E-01</td><td>5.54E-02</td><td></td><td>-2.30E+00</td><td>8.60E-29</td><td>Down</td><td>-1.63E+00</td><td>4.92E-19</td><td>Down</td><td>Unigene1060_All//gi|224137956|ref|XP_002326482.1|//0.00E+00//cationic amino acid transporter [Populus trichocarpa] >gi|222833804|gb|EEE72281.1|cationic amino acid transporter [Populus trichocarpa]</td><td>Unigene1060_All//9.00E-144//AT5G04770| CAT6 (CATIONIC AMINO ACID TRANSPORTER 6); amino acid transmembrane transporter/ basic amino acid transmembrane transporter/ cationic amino acid transmembrane transporter</td><td>Unigene1060_All//1.00E-72//TC146944</td><td></td><td></td><td>GO:0005275//GO:0015837//GO:0031224//GO:0043231</td><td>nr +</td></tr>
<tr><td>Unigene10600_All</td><td>453</td><td>0.00</td><td>0.11</td><td>0.00</td><td>0.55</td><td>0.15</td><td>0.30</td><td>0.09</td><td>0.28</td><td>2.11</td><td>6.81E+00</td><td>6.22E-01</td><td></td><td>null</td><td>null</td><td></td><td>-1.85E+00</td><td>2.76E-01</td><td></td><td>-8.78E-01</td><td>6.17E-01</td><td></td><td>1.57E+00</td><td>5.46E-01</td><td></td><td>4.50E+00</td><td>6.81E-06</td><td>Up</td><td></td><td></td><td>Unigene10600_All//4.00E-29//TC170757</td><td></td><td></td><td></td><td>ESTscan +</td></tr>
<tr><td>Unigene106005_All</td><td>224</td><td>201.34</td><td>86.41</td><td>212.29</td><td>26.24</td><td>32.28</td><td>13.91</td><td>0.94</td><td>0.56</td><td>1.25</td><td>-1.22E+00</td><td>3.05E-45</td><td>Down</td><td>7.64E-02</td><td>3.96E-01</td><td></td><td>2.99E-01</td><td>2.00E-01</td><td></td><td>-9.16E-01</td><td>1.41E-04</td><td></td><td>-7.54E-01</td><td>6.33E-01</td><td></td><td>4.00E-01</td><td>7.43E-01</td><td></td><td></td><td></td><td>Unigene106005_All//3.00E-87//gi|164293551|gb|ES810014.1|ES810014</td><td></td><td></td><td></td><td></td></tr>
<tr><td>Unigene106024_All</td><td>226</td><td>0.00</td><td>0.00</td><td>0.00</td><td>0.00</td><td>0.30</td><td>3.00</td><td>0.00</td><td>0.18</td><td>1.76</td><td>null</td><td>null</td><td></td><td>null</td><td>null</td><td></td><td>8.25E+00</td><td>6.16E-01</td><td></td><td>1.15E+01</td><td>4.19E-04</td><td>Up</td><td>7.53E+00</td><td>6.38E-01</td><td></td><td>1.08E+01</td><td>3.97E-03</td><td></td><td></td><td></td><td></td><td></td><td></td><td></td><td></td></tr>
<tr><td>Unigene10603_All</td><td>428</td><td>0.24</td><td>2.25</td><td>0.00</td><td>0.35</td><td>1.45</td><td>0.11</td><td>0.20</td><td>0.00</td><td>0.00</td><td>3.20E+00</td><td>6.55E-04</td><td>Up</td><td>-7.93E+00</td><td>3.99E-01</td><td></td><td>2.05E+00</td><td>5.13E-02</td><td></td><td>-1.73E+00</td><td>5.02E-01</td><td></td><td>-7.63E+00</td><td>4.07E-01</td><td></td><td>-7.63E+00</td><td>3.61E-01</td><td></td><td>Unigene10603_All//gi|300507129|gb|ADK23940.1|//4.00E-29//KASII [Gossypium hirsutum]</td><td></td><td>Unigene10603_All//2.00E-12//gi|48829336|gb|CO127726.1|CO127726</td><td></td><td>ko00061|Fatty acid biosynthesis</td><td>GO:0008152//GO:0016747</td><td>nr +</td></tr>
<tr><td>Unigene106036_All</td><td>226</td><td>0.00</td><td>0.00</td><td>0.71</td><td>0.00</td><td>0.00</td><td>4.40</td><td>0.56</td><td>0.37</td><td>0.00</td><td>null</td><td>null</td><td></td><td>9.47E+00</td><td>2.40E-01</td><td></td><td>null</td><td>null</td><td></td><td>1.21E+01</td><td>6.15E-06</td><td>Up</td><td>-6.01E-01</td><td>7.68E-01</td><td></td><td>-9.13E+00</td><td>2.01E-01</td><td></td><td>Unigene106036_All//gi|302128114|dbj|BAJ13478.1|//5.00E-28//actin 1 [Guillardia theta]</td><td>Unigene106036_All//2.00E-28//AT3G18780| ACT2 (ACTIN 2); structural constituent of cytoskeleton</td><td>Unigene106036_All//1.00E-27//gi|31406833|gb|CD485868.1|CD485868</td><td></td><td>ko04145|Phagosome</td><td></td><td>nr -</td></tr>
<tr><td>Unigene10605_All</td><td>530</td><td>0.00</td><td>0.00</td><td>0.00</td><td>0.00</td><td>0.00</td><td>0.00</td><td>2.39</td><td>4.02</td><td>0.08</td><td>null</td><td>null</td><td></td><td>null</td><td>null</td><td></td><td>null</td><td>null</td><td></td><td>null</td><td>null</td><td></td><td>7.49E-01</td><td>5.78E-02</td><td></td><td>-4.99E+00</td><td>3.13E-08</td><td>Down</td><td>Unigene10605_All//gi|195624710|gb|ACG34185.1|//7.00E-69//60S ribosomal protein L10-3 [Zea mays]</td><td>Unigene10605_All//1.00E-68//AT1G14320| SAC52 (SUPPRESSOR OF ACAULIS 52); structural constituent of ribosome</td><td>Unigene10605_All//2.00E-13//ES822876</td><td></td><td>ko03010|Ribosome</td><td>GO:0005198//GO:0010467//GO:0030529</td><td>nr -</td></tr>
<tr><td>Unigene10606_All</td><td>525</td><td>0.00</td><td>2.03</td><td>0.81</td><td>0.19</td><td>0.46</td><td>1.12</td><td>1.53</td><td>1.19</td><td>3.57</td><td>1.10E+01</td><td>3.82E-06</td><td>Up</td><td>9.66E+00</td><td>1.39E-02</td><td></td><td>1.27E+00</td><td>5.32E-01</td><td></td><td>2.56E+00</td><td>2.87E-02</td><td></td><td>-3.58E-01</td><td>6.49E-01</td><td></td><td>1.22E+00</td><td>3.68E-03</td><td></td><td></td><td></td><td>Unigene10606_All//3.00E-70//gi|78339929|gb|DT560203.1|DT560203</td><td></td><td></td><td></td><td></td></tr>
<tr><td>Unigene106066_All</td><td>228</td><td>0.92</td><td>0.22</td><td>0.47</td><td>0.22</td><td>0.15</td><td>0.59</td><td>2.60</td><td>0.00</td><td>1.22</td><td>-2.05E+00</td><td>3.34E-01</td><td></td><td>-9.75E-01</td><td>6.22E-01</td><td></td><td>-5.33E-01</td><td>8.29E-01</td><td></td><td>1.44E+00</td><td>6.40E-01</td><td></td><td>-1.13E+01</td><td>2.40E-04</td><td>Down</td><td>-1.09E+00</td><td>1.86E-01</td><td></td><td></td><td></td><td>Unigene106066_All//7.00E-119//TC175066</td><td></td><td></td><td></td><td></td></tr>
<tr><td>Unigene106097_All</td><td>230</td><td>0.23</td><td>3.97</td><td>0.00</td><td>0.65</td><td>1.20</td><td>0.79</td><td>0.55</td><td>0.18</td><td>0.35</td><td>4.12E+00</td><td>2.34E-04</td><td>Up</td><td>-7.83E+00</td><td>6.33E-01</td><td></td><td>8.82E-01</td><td>5.95E-01</td><td></td><td>2.74E-01</td><td>9.06E-01</td><td></td><td>-1.60E+00</td><td>5.35E-01</td><td></td><td>-6.71E-01</td><td>7.21E-01</td><td></td><td>Unigene106097_All//gi|15235982|ref|NP_195678.1|//4.00E-07//SAP domain-containing protein [Arabidopsis thaliana] >gi|145334269|ref|NP_001078515.1|SAP domain-containing protein [Arabidopsis thaliana]</td><td>Unigene106097_All//2.00E-08//AT4G39680| SAP domain-containing protein</td><td>Unigene106097_All//3.00E-115//TC177371</td><td></td><td></td><td></td><td>nr -</td></tr>
<tr><td>Unigene106112_All</td><td>231</td><td>0.45</td><td>3.29</td><td>1.61</td><td>0.86</td><td>1.79</td><td>0.98</td><td>91.88</td><td>11.58</td><td>15.00</td><td>2.86E+00</td><td>5.51E-03</td><td></td><td>1.83E+00</td><td>2.13E-01</td><td></td><td>1.05E+00</td><td>4.26E-01</td><td></td><td>1.81E-01</td><td>9.36E-01</td><td></td><td>-2.99E+00</td><td>4.67E-85</td><td>Down</td><td>-2.61E+00</td><td>3.35E-76</td><td>Down</td><td></td><td></td><td>Unigene106112_All//1.00E-114//gi|164365935|gb|ES812563.1|ES812563</td><td></td><td></td><td></td><td></td></tr>
<tr><td>Unigene106120_All</td><td>231</td><td>0.00</td><td>0.00</td><td>0.00</td><td>0.00</td><td>0.00</td><td>3.52</td><td>0.00</td><td>0.00</td><td>0.00</td><td>null</td><td>null</td><td></td><td>null</td><td>null</td><td></td><td>null</td><td>null</td><td></td><td>1.18E+01</td><td>6.95E-05</td><td>Up</td><td>null</td><td>null</td><td></td><td>null</td><td>null</td><td></td><td>Unigene106120_All//gi|160898912|ref|YP_001564494.1|//8.00E-06//CsbD family protein [Delftia acidovorans SPH-1] >gi|160364496|gb|ABX36109.1| CsbD family protein [Delftia acidovorans SPH-1]</td><td></td><td></td><td></td><td></td><td></td><td>nr -</td></tr>
<tr><td>Unigene10614_All</td><td>203</td><td>92.27</td><td>40.93</td><td>72.66</td><td>60.86</td><td>76.67</td><td>41.60</td><td>2.08</td><td>0.62</td><td>0.79</td><td>-1.17E+00</td><td>4.42E-18</td><td>Down</td><td>-3.45E-01</td><td>1.08E-02</td><td></td><td>3.33E-01</td><td>1.78E-02</td><td></td><td>-5.49E-01</td><td>4.96E-04</td><td></td><td>-1.75E+00</td><td>1.24E-01</td><td></td><td>-1.41E+00</td><td>1.74E-01</td><td></td><td>Unigene10614_All//gi|228481103|gb|ACQ42250.1|//2.00E-29//blue light photoreceptor [Fragaria x ananassa]</td><td>Unigene10614_All//2.00E-27//AT3G45780| PHOT1 (PHOTOTROPIN 1); FMN binding / blue light photoreceptor/ kinase/ protein binding / protein serine/threonine kinase</td><td>Unigene10614_All//3.00E-111//TC143876</td><td></td><td></td><td>GO:0004673//GO:0004674//GO:0005515//GO:0006351//GO:0006468//GO:0009416//GO:0009606//GO:0009881//GO:0009902//GO:0010118//GO:0010360//GO:0015992//GO:0023060//GO:0032559//GO:0043231//GO:0044459</td><td>nr +</td></tr>
<tr><td>Unigene106146_All</td><td>232</td><td>252.13</td><td>104.18</td><td>167.78</td><td>57.54</td><td>90.39</td><td>46.53</td><td>0.00</td><td>0.36</td><td>0.00</td><td>-1.28E+00</td><td>1.32E-62</td><td>Down</td><td>-5.88E-01</td><td>1.07E-16</td><td></td><td>6.52E-01</td><td>5.44E-09</td><td></td><td>-3.07E-01</td><td>5.64E-02</td><td></td><td>8.49E+00</td><td>4.04E-01</td><td></td><td>null</td><td>null</td><td></td><td></td><td></td><td>Unigene106146_All//2.00E-128//TC141493</td><td></td><td></td><td></td><td></td></tr>
<tr><td>Unigene106152_All</td><td>233</td><td>0.22</td><td>0.65</td><td>4.80</td><td>3.63</td><td>3.84</td><td>2.52</td><td>0.18</td><td>1.61</td><td>0.68</td><td>1.54E+00</td><td>5.32E-01</td><td></td><td>4.42E+00</td><td>3.51E-05</td><td>Up</td><td>8.03E-02</td><td>9.41E-01</td><td></td><td>-5.28E-01</td><td>5.03E-01</td><td></td><td>3.15E+00</td><td>3.41E-02</td><td></td><td>1.91E+00</td><td>3.78E-01</td><td></td><td></td><td></td><td>Unigene106152_All//8.00E-57//ES821800</td><td></td><td></td><td></td><td></td></tr>
<tr><td>Unigene106165_All</td><td>233</td><td>6.74</td><td>98.29</td><td>11.20</td><td>7.48</td><td>7.83</td><td>21.90</td><td>72.95</td><td>208.26</td><td>60.70</td><td>3.87E+00</td><td>1.63E-93</td><td>Up</td><td>7.33E-01</td><td>7.42E-02</td><td></td><td>6.60E-02</td><td>9.10E-01</td><td></td><td>1.55E+00</td><td>2.74E-08</td><td>Up</td><td>1.51E+00</td><td>4.06E-82</td><td>Up</td><td>-2.65E-01</td><td>2.80E-02</td><td></td><td>Unigene106165_All//gi|225426282|ref|XP_002266388.1|//8.00E-16//PREDICTED: similar to B2 protein [Vitis vinifera]</td><td>Unigene106165_All//4.00E-13//AT3G27090| FUNCTIONS IN: molecular_function unknown; INVOLVED IN: biological_process unknown; LOCATED IN: cellular_component unknown; EXPRESSED IN: 24 plant structures; EXPRESSED DURING: 15 growth stages; CONTAINS InterPro DOMAIN/s: Development and cell death domain (InterPro:IPR013989), Kelch related (InterPro:IPR013089); BEST Arabidopsis thaliana protein match is: unknown protein (TAIR:AT5G42050.1); Has 642 Blast hits to 564 proteins in 35 species: Archae - 0; Bacteria - 2; Metazoa - 12; Fungi - 0; Plants - 155; Viruses - 0; Other Eukaryotes - 473 (source: NCBI BLink).</td><td>Unigene106165_All//5.00E-89//DR461377</td><td></td><td></td><td></td><td>nr +</td></tr>
<tr><td>Unigene10617_All</td><td>574</td><td>1.19</td><td>0.18</td><td>0.00</td><td>0.09</td><td>0.42</td><td>0.16</td><td>2.58</td><td>1.02</td><td>1.04</td><td>-2.75E+00</td><td>1.06E-02</td><td></td><td>-1.02E+01</td><td>7.65E-04</td><td>Down</td><td>2.27E+00</td><td>3.05E-01</td><td></td><td>8.59E-01</td><td>7.97E-01</td><td></td><td>-1.34E+00</td><td>7.48E-03</td><td></td><td>-1.31E+00</td><td>6.57E-03</td><td></td><td>Unigene10617_All//gi|124359380|gb|ABN05846.1|//4.00E-06//Tetratricopeptide-like helical [Medicago truncatula]</td><td></td><td>Unigene10617_All//2.00E-13//TC136777</td><td></td><td></td><td></td><td>nr -</td></tr>
<tr><td>Unigene106174_All</td><td>234</td><td>107.55</td><td>221.73</td><td>175.68</td><td>92.82</td><td>144.95</td><td>102.68</td><td>51.13</td><td>66.98</td><td>64.35</td><td>1.04E+00</td><td>2.18E-40</td><td>Up</td><td>7.08E-01</td><td>2.27E-16</td><td></td><td>6.43E-01</td><td>6.05E-14</td><td></td><td>1.46E-01</td><td>2.57E-01</td><td></td><td>3.90E-01</td><td>2.13E-03</td><td></td><td>3.32E-01</td><td>9.08E-03</td><td></td><td>Unigene106174_All//gi|151368154|gb|ABS10812.1|//2.00E-23//DNA binding protein-like protein [Gossypium arboreum]</td><td></td><td>Unigene106174_All//7.00E-125//TC132469</td><td></td><td></td><td></td><td>nr +</td></tr>
<tr><td>Unigene106178_All</td><td>234</td><td>0.22</td><td>1.30</td><td>1.82</td><td>1.70</td><td>13.10</td><td>8.88</td><td>1.63</td><td>0.18</td><td>0.34</td><td>2.54E+00</td><td>1.51E-01</td><td></td><td>3.03E+00</td><td>5.74E-02</td><td></td><td>2.94E+00</td><td>2.00E-11</td><td>Up</td><td>2.38E+00</td><td>4.55E-06</td><td>Up</td><td>-3.19E+00</td><td>3.12E-02</td><td></td><td>-2.26E+00</td><td>6.50E-02</td><td></td><td></td><td></td><td></td><td></td><td></td><td></td><td></td></tr>
<tr><td>Unigene106195_All</td><td>235</td><td>0.22</td><td>3.67</td><td>1.13</td><td>0.21</td><td>0.29</td><td>0.96</td><td>0.18</td><td>1.07</td><td>1.19</td><td>4.04E+00</td><td>4.20E-04</td><td>Up</td><td>2.35E+00</td><td>2.40E-01</td><td></td><td>4.67E-01</td><td>9.61E-01</td><td></td><td>2.18E+00</td><td>3.23E-01</td><td></td><td>2.57E+00</td><td>1.53E-01</td><td></td><td>2.72E+00</td><td>9.95E-02</td><td></td><td></td><td></td><td>Unigene106195_All//2.00E-36//TC157213</td><td></td><td></td><td></td><td></td></tr>
<tr><td>Unigene106196_All</td><td>235</td><td>34.73</td><td>8.19</td><td>15.86</td><td>61.26</td><td>31.65</td><td>24.98</td><td>4.50</td><td>1.42</td><td>2.20</td><td>-2.08E+00</td><td>3.83E-18</td><td>Down</td><td>-1.13E+00</td><td>1.68E-07</td><td>Down</td><td>-9.53E-01</td><td>3.66E-12</td><td></td><td>-1.29E+00</td><td>1.80E-17</td><td>Down</td><td>-1.66E+00</td><td>8.55E-03</td><td></td><td>-1.03E+00</td><td>7.30E-02</td><td></td><td></td><td></td><td>Unigene106196_All//1.00E-86//TC153940</td><td></td><td></td><td></td><td>ESTscan +</td></tr>
<tr><td>Unigene106228_All</td><td>237</td><td>0.00</td><td>1.92</td><td>0.45</td><td>1.05</td><td>1.02</td><td>3.43</td><td>2.68</td><td>8.29</td><td>5.72</td><td>1.09E+01</td><td>7.44E-03</td><td></td><td>8.81E+00</td><td>4.02E-01</td><td></td><td>-4.72E-02</td><td>9.52E-01</td><td></td><td>1.71E+00</td><td>4.57E-02</td><td></td><td>1.63E+00</td><td>1.97E-04</td><td>Up</td><td>1.09E+00</td><td>2.95E-02</td><td></td><td></td><td></td><td>Unigene106228_All//2.00E-131//TC164875</td><td></td><td></td><td></td><td></td></tr>
<tr><td>Unigene106270_All</td><td>239</td><td>14.45</td><td>6.15</td><td>31.86</td><td>82.54</td><td>131.55</td><td>52.72</td><td>78.37</td><td>11.54</td><td>45.84</td><td>-1.23E+00</td><td>3.00E-04</td><td>Down</td><td>1.14E+00</td><td>3.76E-07</td><td>Up</td><td>6.72E-01</td><td>5.62E-14</td><td></td><td>-6.47E-01</td><td>7.89E-08</td><td></td><td>-2.76E+00</td><td>2.71E-69</td><td>Down</td><td>-7.74E-01</td><td>8.45E-12</td><td></td><td>Unigene106270_All//gi|50313462|gb|AAT74601.1|//4.00E-23//cyclopropane fatty acid synthase [Gossypium hirsutum]</td><td>Unigene106270_All//7.00E-17//AT3G23510| cyclopropane fatty acid synthase, putative / CPA-FA synthase, putative</td><td>Unigene106270_All//3.00E-78//TC129560</td><td></td><td></td><td></td><td>nr +</td></tr>
<tr><td>Unigene10628_All</td><td>273</td><td>0.38</td><td>0.00</td><td>0.00</td><td>2.37</td><td>0.13</td><td>1.99</td><td>0.62</td><td>0.61</td><td>0.44</td><td>-8.58E+00</td><td>3.77E-01</td><td></td><td>-8.58E+00</td><td>3.99E-01</td><td></td><td>-4.23E+00</td><td>5.94E-04</td><td>Down</td><td>-2.57E-01</td><td>7.57E-01</td><td></td><td>-1.66E-02</td><td>1.02E+00</td><td></td><td>-5.01E-01</td><td>7.47E-01</td><td></td><td>Unigene10628_All//gi|186898205|gb|ACC93947.1|//6.00E-44//heat-shock protein 70 [Hevea brasiliensis]</td><td>Unigene10628_All//5.00E-27//AT3G12580| HSP70 (heat shock protein 70); ATP binding</td><td>Unigene10628_All//7.00E-67//TC165936</td><td></td><td>ko03040|Spliceosome//ko04141|Protein processing in endoplasmic reticulum//ko04144|Endocytosis</td><td>GO:0000302//GO:0009642//GO:0010038//GO:0016020//GO:0016628//GO:0019899//GO:0030312//GO:0032446//GO:0032559//GO:0043231//GO:0051707</td><td>nr +</td></tr>
<tr><td>Unigene106295_All</td><td>241</td><td>0.00</td><td>0.00</td><td>0.00</td><td>0.83</td><td>0.00</td><td>0.75</td><td>0.00</td><td>0.69</td><td>2.98</td><td>null</td><td>null</td><td></td><td>null</td><td>null</td><td></td><td>-9.69E+00</td><td>8.48E-02</td><td></td><td>-1.41E-01</td><td>9.35E-01</td><td></td><td>9.44E+00</td><td>1.39E-01</td><td></td><td>1.15E+01</td><td>2.63E-05</td><td>Up</td><td>Unigene106295_All//gi|255587535|ref|XP_002534305.1|//2.00E-07//conserved hypothetical protein [Ricinus communis] >gi|223525538|gb|EEF28080.1|conserved hypothetical protein [Ricinus communis]</td><td></td><td>Unigene106295_All//1.00E-126//gi|109889377|gb|DW518345.1|DW518345</td><td></td><td></td><td></td><td>nr -</td></tr>
<tr><td>Unigene1063_All</td><td>1193</td><td>8.55</td><td>17.20</td><td>12.68</td><td>12.61</td><td>15.99</td><td>15.07</td><td>8.15</td><td>12.26</td><td>11.59</td><td>1.01E+00</td><td>1.48E-15</td><td>Up</td><td>5.68E-01</td><td>1.32E-04</td><td></td><td>3.43E-01</td><td>5.44E-03</td><td></td><td>2.57E-01</td><td>6.41E-02</td><td></td><td>5.89E-01</td><td>6.10E-06</td><td></td><td>5.08E-01</td><td>1.12E-04</td><td></td><td>Unigene1063_All//gi|42572315|ref|NP_974253.1|//8.00E-68//HhH-GPD base excision DNA repair family protein [Arabidopsis thaliana] >gi|114050633|gb|ABI49466.1|At3g07930 [Arabidopsis thaliana]</td><td>Unigene1063_All//4.00E-69//AT3G07930| HhH-GPD base excision DNA repair family protein</td><td>Unigene1063_All//0.00E+00//TC169474</td><td></td><td>ko03410|Base excision repair</td><td>GO:0006281</td><td>nr +</td></tr>
<tr><td>Unigene106308_All</td><td>242</td><td>33.73</td><td>13.61</td><td>14.96</td><td>64.84</td><td>64.46</td><td>79.13</td><td>357.81</td><td>147.49</td><td>194.58</td><td>-1.31E+00</td><td>9.81E-10</td><td>Down</td><td>-1.17E+00</td><td>6.59E-08</td><td>Down</td><td>-8.50E-03</td><td>9.54E-01</td><td></td><td>2.87E-01</td><td>2.81E-02</td><td></td><td>-1.28E+00</td><td>2.39E-113</td><td>Down</td><td>-8.79E-01</td><td>1.48E-63</td><td></td><td>Unigene106308_All//gi|78191410|gb|ABB29926.1|//1.00E-35//fructose-bisphosphate aldolase-like [Solanum tuberosum]</td><td>Unigene106308_All//2.00E-34//AT5G03690| fructose-bisphosphate aldolase, putative</td><td>Unigene106308_All//2.00E-134//gi|11201622|gb|BF270531.1|BF270531</td><td></td><td>ko00010|Glycolysis / Gluconeogenesis//ko00710|Carbon fixation in photosynthetic organisms//ko00051|Fructose and mannose metabolism//ko00030|Pentose phosphate pathway</td><td></td><td>nr -</td></tr>
<tr><td>Unigene106326_All</td><td>243</td><td>0.22</td><td>0.00</td><td>1.31</td><td>0.21</td><td>1.13</td><td>10.04</td><td>0.35</td><td>1.55</td><td>0.66</td><td>-7.75E+00</td><td>6.20E-01</td><td></td><td>2.61E+00</td><td>1.46E-01</td><td></td><td>2.47E+00</td><td>2.23E-01</td><td></td><td>5.61E+00</td><td>3.38E-13</td><td>Up</td><td>2.15E+00</td><td>9.46E-02</td><td></td><td>9.14E-01</td><td>6.25E-01</td><td></td><td></td><td></td><td>Unigene106326_All//3.00E-07//TC168359</td><td></td><td></td><td></td><td></td></tr>
<tr><td>Unigene10633_All</td><td>592</td><td>0.00</td><td>0.00</td><td>0.00</td><td>0.00</td><td>0.00</td><td>0.00</td><td>2.14</td><td>12.85</td><td>0.34</td><td>null</td><td>null</td><td></td><td>null</td><td>null</td><td></td><td>null</td><td>null</td><td></td><td>null</td><td>null</td><td></td><td>2.58E+00</td><td>1.82E-26</td><td>Up</td><td>-2.67E+00</td><td>2.31E-05</td><td>Down</td><td>Unigene10633_All//gi|38640722|gb|AAR25996.1|//5.00E-06//cytochrome P450 monooxygenase [Pyrus communis]</td><td></td><td>Unigene10633_All//1.00E-36//gi|73864682|gb|DT467421.1|DT467421</td><td></td><td></td><td></td><td>nr -</td></tr>
<tr><td>Unigene106358_All</td><td>246</td><td>0.85</td><td>2.47</td><td>1.08</td><td>0.41</td><td>0.84</td><td>0.37</td><td>0.69</td><td>0.34</td><td>5.18</td><td>1.54E+00</td><td>1.22E-01</td><td></td><td>3.47E-01</td><td>7.97E-01</td><td></td><td>1.05E+00</td><td>6.13E-01</td><td></td><td>-1.41E-01</td><td>9.53E-01</td><td></td><td>-1.02E+00</td><td>6.15E-01</td><td></td><td>2.91E+00</td><td>1.05E-05</td><td>Up</td><td>Unigene106358_All//gi|55168340|gb|AAV44205.1|//3.00E-06//unknow protein [Oryza sativa Japonica Group]</td><td></td><td>Unigene106358_All//1.00E-31//TC139311</td><td></td><td></td><td></td><td>nr -</td></tr>
<tr><td>Unigene10636_All</td><td>345</td><td>0.00</td><td>0.73</td><td>0.62</td><td>1.30</td><td>0.20</td><td>0.52</td><td>1.59</td><td>0.00</td><td>2.77</td><td>9.52E+00</td><td>7.89E-02</td><td></td><td>9.27E+00</td><td>1.42E-01</td><td></td><td>-2.70E+00</td><td>3.24E-02</td><td></td><td>-1.31E+00</td><td>2.73E-01</td><td></td><td>-1.06E+01</td><td>4.62E-04</td><td>Down</td><td>7.99E-01</td><td>1.95E-01</td><td></td><td></td><td></td><td>Unigene10636_All//3.00E-128//TC179309</td><td></td><td></td><td></td><td></td></tr>
<tr><td>Unigene106360_All</td><td>246</td><td>0.00</td><td>0.62</td><td>0.00</td><td>2.84</td><td>0.00</td><td>2.39</td><td>0.17</td><td>0.34</td><td>0.16</td><td>9.27E+00</td><td>2.37E-01</td><td></td><td>null</td><td>null</td><td></td><td>-1.15E+01</td><td>3.44E-05</td><td>Down</td><td>-2.48E-01</td><td>7.56E-01</td><td></td><td>9.83E-01</td><td>7.33E-01</td><td></td><td>-8.65E-02</td><td>9.74E-01</td><td></td><td></td><td></td><td></td><td></td><td></td><td></td><td></td></tr>
<tr><td>Unigene10637_All</td><td>420</td><td>0.00</td><td>2.53</td><td>1.78</td><td>0.00</td><td>0.00</td><td>0.00</td><td>0.00</td><td>0.00</td><td>0.00</td><td>1.13E+01</td><td>3.82E-06</td><td>Up</td><td>1.08E+01</td><td>3.28E-04</td><td>Up</td><td>null</td><td>null</td><td></td><td>null</td><td>null</td><td></td><td>null</td><td>null</td><td></td><td>null</td><td>null</td><td></td><td></td><td></td><td></td><td></td><td></td><td></td><td>ESTscan +</td></tr>
<tr><td>Unigene106378_All</td><td>247</td><td>0.42</td><td>640.65</td><td>459.20</td><td>0.40</td><td>0.00</td><td>2.56</td><td>0.34</td><td>0.17</td><td>0.00</td><td>1.06E+01</td><td>0.00E+00</td><td>Up</td><td>1.01E+01</td><td>0.00E+00</td><td>Up</td><td>-8.66E+00</td><td>3.17E-01</td><td></td><td>2.67E+00</td><td>1.83E-02</td><td></td><td>-1.02E+00</td><td>7.26E-01</td><td></td><td>-8.42E+00</td><td>3.61E-01</td><td></td><td></td><td></td><td></td><td></td><td></td><td></td><td></td></tr>
<tr><td>Unigene1064_All</td><td>1263</td><td>1.04</td><td>2.85</td><td>1.31</td><td>2.45</td><td>2.84</td><td>2.00</td><td>0.87</td><td>1.36</td><td>2.71</td><td>1.46E+00</td><td>2.10E-05</td><td>Up</td><td>3.36E-01</td><td>5.35E-01</td><td></td><td>2.14E-01</td><td>5.76E-01</td><td></td><td>-2.88E-01</td><td>4.68E-01</td><td></td><td>6.41E-01</td><td>1.57E-01</td><td></td><td>1.64E+00</td><td>2.03E-07</td><td>Up</td><td>Unigene1064_All//gi|42572313|ref|NP_974252.1|//2.00E-28//HhH-GPD base excision DNA repair family protein [Arabidopsis thaliana]</td><td>Unigene1064_All//1.00E-29//AT3G07930| HhH-GPD base excision DNA repair family protein</td><td>Unigene1064_All//0.00E+00//TC169474</td><td></td><td>ko03410|Base excision repair</td><td></td><td>nr -</td></tr>
<tr><td>Unigene10641_All</td><td>774</td><td>0.00</td><td>0.00</td><td>0.00</td><td>0.51</td><td>0.98</td><td>0.41</td><td>0.33</td><td>3.29</td><td>0.82</td><td>null</td><td>null</td><td></td><td>null</td><td>null</td><td></td><td>9.27E-01</td><td>2.97E-01</td><td></td><td>-3.34E-01</td><td>7.57E-01</td><td></td><td>3.33E+00</td><td>8.60E-12</td><td>Up</td><td>1.33E+00</td><td>1.00E-01</td><td></td><td>Unigene10641_All//gi|18404500|ref|NP_566763.1|//2.00E-65//auxin-responsive family protein [Arabidopsis thaliana] >gi|79313415|ref|NP_001030764.1|auxin-responsive family protein [Arabidopsis thaliana] >gi|16323200|gb|AAL15334.1|AT3g25290/MJL12_25 [Arabidopsis thaliana] >gi|25090093|gb|AAN72226.1|At3g25290/MJL12_25 [Arabidopsis thaliana]</td><td>Unigene10641_All//3.00E-63//AT3G25290| auxin-responsive family protein</td><td>Unigene10641_All//3.00E-62//gi|78325030|gb|DT545304.1|DT545304</td><td></td><td></td><td>GO:0016020</td><td>nr +</td></tr>
<tr><td>Unigene106413_All</td><td>250</td><td>0.00</td><td>0.00</td><td>0.00</td><td>1.00</td><td>0.69</td><td>1.99</td><td>0.85</td><td>3.34</td><td>4.30</td><td>null</td><td>null</td><td></td><td>null</td><td>null</td><td></td><td>-5.33E-01</td><td>7.13E-01</td><td></td><td>9.96E-01</td><td>3.94E-01</td><td></td><td>1.98E+00</td><td>8.88E-03</td><td></td><td>2.35E+00</td><td>4.46E-04</td><td>Up</td><td>Unigene106413_All//gi|115472687|ref|NP_001059942.1|//9.00E-28//Os07g0551300 [Oryza sativa Japonica Group] >gi|113611478|dbj|BAF21856.1|Os07g0551300 [Oryza sativa Japonica Group]</td><td>Unigene106413_All//7.00E-28//AT4G21390| B120; ATP binding / protein kinase/ protein serine/threonine kinase/ sugar binding</td><td>Unigene106413_All//5.00E-12//DT469086</td><td></td><td>ko04626|Plant-pathogen interaction</td><td></td><td>nr +</td></tr>
<tr><td>Unigene10642_All</td><td>482</td><td>0.00</td><td>0.32</td><td>0.00</td><td>0.00</td><td>0.00</td><td>0.00</td><td>1.75</td><td>4.77</td><td>0.91</td><td>8.30E+00</td><td>2.37E-01</td><td></td><td>null</td><td>null</td><td></td><td>null</td><td>null</td><td></td><td>null</td><td>null</td><td></td><td>1.44E+00</td><td>2.32E-04</td><td>Up</td><td>-9.48E-01</td><td>1.47E-01</td><td></td><td>Unigene10642_All//gi|7267528|emb|CAB78010.1|//2.00E-06//receptor protein kinase-like protein [Arabidopsis thaliana] >gi|7321074|emb|CAB82121.1|receptor protein kinase-like protein [Arabidopsis thaliana]</td><td>Unigene10642_All//1.00E-07//AT4G08850| kinase</td><td></td><td></td><td></td><td>GO:0004672//GO:0005488</td><td>nr -</td></tr>
</table>

Unigene10643_All	411	1.78	0.00	0.39	1.33	0.59	0.00	0.00	0.31	0.00	-1.08E+01	2.11E-04	Down	-2.20E+00	2.77E-02		-1.18E+00	2.25E-01		-1.04E+01	1.52E-03		8.25E+00	2.41E-01		null	null		Unigene10643_All//gi|225456658|ref|XP_002271524.1|//7.00E-07//PREDICTED: hypothetical protein [Vitis vinifera]		Unigene10643_All//1.00E-07//TC144614				nr -
Unigene106448_All	253	16.54	8.21	27.15	3.15	1.77	1.96	0.17	0.00	0.16	-1.01E+00	7.51E-04	Down	7.15E-01	2.12E-03		-8.32E-01	2.88E-01		-6.82E-01	4.05E-01		-7.38E+00	6.63E-01		-8.54E-02	9.74E-01		Unigene106448_All//gi|159895667|gb|ABX10444.1|//5.00E-14//phosphoethanolamine N-methyltransferase [Gossypium hirsutum]	Unigene106448_All//1.00E-12//AT1G48600| phosphoethanolamine N-methyltransferase 2, putative (NMT2)	Unigene106448_All//1.00E-52//TC162345		ko00564|Glycerophospholipid metabolism		nr -
Unigene10645_All	308	0.00	2.80	2.59	0.00	0.00	0.00	0.00	0.00	0.00	1.14E+01	4.95E-05	Up	1.13E+01	1.72E-04	Up	null	null		null	null		null	null		null	null		Unigene10645_All//sp|Q8VHX6|FLNC_MOUSE//9.00E-31//Filamin-C OS=Mus musculus GN=Flnc PE=1 SV=3						swissprot -
Unigene106472_All	255	0.00	2.98	0.63	0.59	1.35	3.01	0.33	0.00	0.00	1.15E+01	1.77E-04	Up	9.29E+00	2.40E-01		1.20E+00	4.26E-01		2.36E+00	1.34E-02		-8.37E+00	4.07E-01		-8.37E+00	3.61E-01								
Unigene106474_All	255	13.54	4.97	14.83	51.18	42.40	44.28	117.39	79.00	58.43	-1.45E+00	3.41E-05	Down	1.31E-01	6.90E-01		-2.72E-01	8.87E-02		-2.09E-01	2.32E-01		-5.71E-01	9.53E-11		-1.01E+00	4.26E-28	Down	Unigene106474_All//gi|297611781|ref|NP_001067845.2|//1.00E-30//Os11g0455500 [Oryza sativa Japonica Group] >gi|255680068|dbj|BAF28208.2|Os11g0455500 [Oryza sativa Japonica Group]	Unigene106474_All//2.00E-28//AT4G13940| MEE58 (MATERNAL EFFECT EMBRYO ARREST 58); adenosylhomocysteinase/ copper ion binding	Unigene106474_All//1.00E-101//gi|164369275|gb|EV484110.1|EV484110		ko00270|Cysteine and methionine metabolism//ko00450|Selenoamino acid metabolism		nr -
Unigene10652_All	435	0.00	3.38	2.45	0.00	0.00	0.00	0.00	0.00	0.00	1.17E+01	2.11E-08	Up	1.13E+01	6.63E-06	Up	null	null		null	null		null	null		null	null		Unigene10652_All//gi|300260006|gb|EFJ44228.1|//2.00E-14//RWP-RK domain-containing transcription factor [Volvox carteri f. nagariensis]					GO:0005488//GO:0017111//GO:0044464	nr +
Unigene106520_All	262	0.00	0.77	0.00	0.00	0.13	2.76	0.00	0.00	0.00	9.60E+00	1.37E-01		null	null		7.04E+00	8.39E-01		1.14E+01	2.31E-04	Up	null	null		null	null		Unigene106520_All//gi|15218349|ref|NP_177355.1|//3.00E-09//late embryogenesis abundant domain-containing protein / LEA domain-containing protein [Arabidopsis thaliana]						nr -
Unigene106525_All	262	0.00	42.16	1.63	0.00	0.00	0.52	0.00	0.16	0.00	1.54E+01	2.52E-63	Up	1.07E+01	1.39E-02		null	null		9.01E+00	3.12E-01		7.32E+00	6.38E-01		null	null		Unigene106525_All//gi|187610416|gb|ACD13462.1|//1.00E-23//raffionse synthase 3 [Glycine max]	Unigene106525_All//1.00E-19//AT5G40390| SIP1 (seed imbibition 1-like); galactinol-sucrose galactosyltransferase/ hydrolase, hydrolyzing O-glycosyl compounds			ko00052|Galactose metabolism		nr +
Unigene106530_All	263	6.96	0.39	3.24	8.90	6.02	5.84	0.00	0.00	0.00	-4.18E+00	2.18E-08	Down	-1.10E+00	3.06E-02		-5.64E-01	1.69E-01		-6.09E-01	1.55E-01		null	null		null	null								
Unigene106539_All	263	0.80	5.78	2.23	3.98	3.93	3.43	0.00	0.00	0.45	2.86E+00	2.69E-05	Up	1.48E+00	1.53E-01		-1.81E-02	9.61E-01		-2.12E-01	7.44E-01		null	null		8.83E+00	2.44E-01								
Unigene106542_All	264	105.24	85.41	137.97	10.00	4.83	1.37	0.64	0.32	0.00	-3.01E-01	3.97E-03		3.91E-01	2.01E-05		-1.05E+00	4.04E-03		-2.87E+00	2.04E-09	Down	-1.02E+00	6.15E-01		-9.32E+00	1.09E-01				Unigene106542_All//4.00E-133//TC140158				
Unigene106544_All	264	0.00	0.00	0.00	0.00	0.78	0.86	0.48	0.95	4.53	null	null		null	null		9.61E+00	1.57E-01		9.74E+00	1.19E-01		9.83E-01	5.18E-01		3.24E+00	7.29E-06	Up							
Unigene106548_All	264	0.00	0.00	0.20	0.00	0.65	2.57	0.00	0.16	0.00	null	null		7.66E+00	6.41E-01		9.35E+00	2.28E-01		1.13E+01	4.19E-04	Up	7.31E+00	6.38E-01		null	null		Unigene106548_All//gi|207693265|gb|ACI25287.1|//4.00E-20//ARM repeat containing protein [Populus trichocarpa]	Unigene106548_All//1.00E-20//AT5G66200| ARO2 (ARMADILLO REPEAT ONLY 2); binding	Unigene106548_All//3.00E-140//EX165874				nr -
Unigene106560_All	267	0.39	3.04	0.80	0.00	0.64	5.58	0.63	0.00	0.00	2.95E+00	3.29E-03		1.03E+00	5.92E-01		9.33E+00	2.28E-01		1.24E+01	7.04E-09	Up	-9.31E+00	1.35E-01		-9.31E+00	1.09E-01		Unigene106560_All//gi|297811437|ref|XP_002873602.1|//4.00E-17//nodulin MtN3 family protein [Arabidopsis lyrata subsp. lyrata] >gi|297319439|gb|EFH49861.1|nodulin MtN3 family protein [Arabidopsis lyrata subsp. lyrata]	Unigene106560_All//4.00E-18//AT5G13170| SAG29 (SENESCENCE-ASSOCIATED PROTEIN 29)	Unigene106560_All//1.00E-129//TC176027				nr +
Unigene106569_All	269	0.00	0.38	0.59	0.00	0.26	3.02	0.16	0.16	0.00	8.56E+00	3.91E-01		9.21E+00	2.40E-01		8.00E+00	6.16E-01		1.16E+01	6.95E-05	Up	-1.66E-02	1.01E+00		-7.30E+00	6.17E-01		Unigene106569_All//gi|15218927|ref|NP_176191.1|//9.00E-18//ALA3 (Aminophospholipid ATPase3); ATPase, coupled to transmembrane movement of ions, phosphorylative mechanism / phospholipid transporter [Arabidopsis thaliana] >gi|30316321|sp|Q9XIE6.2|ALA3_ARATH RecName: Full=Phospholipid-transporting ATPase 3; Short=AtALA3; AltName: Full=Aminophospholipid flippase 3 >gi|20147219|gb|AAM10325.1|At1g59820/F23H11_14 [Arabidopsis thaliana]	Unigene106569_All//4.00E-19//AT1G59820| ALA3 (Aminophospholipid ATPase3); ATPase, coupled to transmembrane movement of ions, phosphorylative mechanism / phospholipid transporter					nr -
Unigene106570_All	269	94.53	30.14	45.33	161.85	141.71	162.19	106.88	44.28	53.91	-1.65E+00	2.94E-40	Down	-1.06E+00	6.57E-20	Down	-1.92E-01	1.82E-02		3.00E-03	9.73E-01		-1.27E+00	8.20E-38	Down	-9.87E-01	3.32E-26				Unigene106570_All//7.00E-113//TC134569				
Unigene106571_All	269	0.19	0.38	1.58	2.96	0.00	3.02	1.10	0.00	0.30	9.54E-01	7.28E-01		3.03E+00	5.74E-02		-1.15E+01	6.49E-06	Down	2.85E-02	9.71E-01		-1.01E+01	2.19E-02		-1.89E+00	1.68E-01		Unigene106571_All//gi|255578137|ref|XP_002529938.1|//3.00E-07//conserved hypothetical protein [Ricinus communis] >gi|223530568|gb|EEF32446.1|conserved hypothetical protein [Ricinus communis]		Unigene106571_All//5.00E-89//EX164741				nr -
Unigene106575_All	270	1.94	0.75	1.78	1.29	2.17	2.84	4.85	2.01	1.33	-1.37E+00	1.97E-01		-1.27E-01	8.98E-01		7.47E-01	4.82E-01		1.14E+00	1.89E-01		-1.27E+00	1.77E-02		-1.87E+00	6.93E-04	Down			Unigene106575_All//5.00E-151//TC174841				
Unigene10658_All	785	0.00	1.03	0.00	0.00	1.32	0.86	0.00	0.05	0.00	1.00E+01	9.39E-05	Up	null	null		1.04E+01	2.35E-06	Up	9.75E+00	4.19E-04	Up	5.73E+00	6.38E-01		null	null		Unigene10658_All//gi|6686397|gb|AAF23831.1|AC007234_3//1.00E-20//F1E22.12 [Arabidopsis thaliana]	Unigene10658_All//3.00E-10//AT3G25270| nucleic acid binding				GO:0005488	nr -
Unigene106580_All	270	0.39	0.00	0.39	0.74	1.53	5.85	1.25	0.77	0.89	-8.60E+00	3.77E-01		2.51E-02	1.00E+00		1.05E+00	4.26E-01		2.99E+00	7.31E-06	Up	-6.95E-01	5.78E-01		-5.01E-01	6.50E-01				Unigene106580_All//4.00E-68//ES801598				
Unigene106586_All	271	626.50	436.95	625.25	247.42	215.00	120.66	427.95	291.95	286.07	-5.20E-01	1.88E-39		-2.90E-03	9.73E-01		-2.03E-01	1.03E-03		-1.04E+00	8.65E-56	Down	-5.52E-01	6.85E-37		-5.81E-01	1.51E-41		Unigene106586_All//gi|148807173|gb|ABR13296.1|//9.00E-07//unknown protein [Prunus dulcis]		Unigene106586_All//5.00E-83//TC144875				nr -
Unigene106593_All	272	1.15	2.79	5.48	4.76	4.94	4.98	0.78	0.31	1.90	1.28E+00	1.28E-01		2.25E+00	5.49E-04	Up	5.23E-02	9.57E-01		6.50E-02	9.34E-01		-1.34E+00	4.31E-01		1.29E+00	1.59E-01								
Unigene106597_All	272	0.58	4.47	2.15	5.68	3.42	4.65	2.18	2.77	2.64	2.95E+00	1.75E-04	Up	1.90E+00	8.48E-02		-7.32E-01	1.59E-01		-2.88E-01	6.38E-01		3.46E-01	6.20E-01		2.77E-01	6.90E-01				Unigene106597_All//2.00E-70//gi|13246583|gb|BE054988.2|BE054988				
Unigene106598_All	272	766.92	355.99	626.27	96.70	91.41	56.12	0.47	0.15	0.29	-1.11E+00	2.93E-177	Down	-2.92E-01	2.39E-16		-8.12E-02	5.52E-01		-7.85E-01	4.15E-14		-1.60E+00	5.35E-01		-6.71E-01	7.21E-01		Unigene106598_All//gi|56122690|gb|AAV74408.1|//4.00E-47//chloroplast chlorophyll A/B binding protein [Manihot esculenta]	Unigene106598_All//8.00E-46//AT2G05070| LHCB2.2; chlorophyll binding	Unigene106598_All//3.00E-152//TC135194		ko00196|Photosynthesis - antenna proteins		nr -
Unigene1066_All	819	13.61	14.54	21.00	15.02	15.64	17.37	43.21	24.29	19.07	9.55E-02	5.91E-01		6.26E-01	5.76E-06		5.81E-02	7.98E-01		2.09E-01	2.09E-01		-8.31E-01	1.76E-23		-1.18E+00	1.53E-42	Down	Unigene1066_All//gi|295687233|gb|ADG27842.1|//1.00E-126//transaldolase [Gossypium hirsutum]	Unigene1066_All//4.00E-71//AT1G12230| transaldolase, putative	Unigene1066_All//0.00E+00//TC142315		ko00030|Pentose phosphate pathway	GO:0009536//GO:0016744//GO:0044238//GO:0046914	nr -
Unigene106600_All	272	0.19	15.83	1.17	0.37	0.13	0.66	0.31	0.00	0.00	6.36E+00	6.39E-23	Up	2.61E+00	1.46E-01		-1.53E+00	5.89E-01		8.59E-01	6.96E-01		-8.28E+00	4.07E-01		-8.28E+00	3.61E-01		Unigene106600_All//gi|62420870|tpg|DAA01542.1|//5.00E-30//TPA: TPA_exp: alpha-dioxygenase 3 [Lycopersicon esculentum]	Unigene106600_All//5.00E-28//AT1G73680| pathogen-responsive alpha-dioxygenase, putative	Unigene106600_All//2.00E-125//TC167331		ko00592|alpha-Linolenic acid metabolism		nr -
Unigene106603_All	273	0.38	0.37	0.20	0.18	1.01	3.14	0.93	0.61	0.15	-4.63E-02	9.91E-01		-9.74E-01	7.22E-01		2.47E+00	2.23E-01		4.11E+00	3.41E-04	Up	-6.02E-01	6.48E-01		-2.67E+00	1.15E-01		Unigene106603_All//gi|224062025|ref|XP_002300717.1|//8.00E-41//SAUR family protein [Populus trichocarpa] >gi|222842443|gb|EEE79990.1| SAUR family protein [Populus trichocarpa]	Unigene106603_All//1.00E-39//AT1G75580| auxin-responsive protein, putative	Unigene106603_All//7.00E-141//TC157705				nr +
Unigene106609_All	273	11.88	0.00	0.39	11.50	2.65	1.65	4.03	1.53	5.11	-1.35E+01	9.73E-19	Down	-4.93E+00	3.88E-15	Down	-2.12E+00	4.75E-09	Down	-2.80E+00	7.93E-11	Down	-1.40E+00	2.04E-02		3.43E-01	5.03E-01		Unigene106609_All//gi|224115708|ref|XP_002317102.1|//7.00E-10//predicted protein [Populus trichocarpa] >gi|222860167|gb|EEE97714.1|predicted protein [Populus trichocarpa]		Unigene106609_All//4.00E-133//ES815751				nr +
Unigene10661_All	478	0.77	0.32	0.89	4.38	2.67	1.04	1.24	0.70	2.00	-1.27E+00	3.36E-01		2.18E-01	8.31E-01		-7.16E-01	9.63E-02		-2.07E+00	2.11E-05	Down	-8.24E-01	3.48E-01		6.92E-01	2.60E-01		Unigene10661_All//gi|224073274|ref|XP_002304055.1|//1.00E-30//cc-nbs-lrr resistance protein [Populus trichocarpa] >gi|222841487|gb|EEE79034.1| cc-nbs-lrr resistance protein [Populus trichocarpa]	Unigene10661_All//6.00E-18//AT3G14470| disease resistance protein (NBS-LRR class), putative	Unigene10661_All//2.00E-161//gi|109863388|gb|DW492365.1|DW492365		ko04626|Plant-pathogen interaction	GO:0005488//GO:0009987	nr +
Unigene10662_All	882	2.85	0.23	1.51	0.34	0.23	0.20	0.00	0.00	0.00	-3.63E+00	2.75E-10	Down	-9.16E-01	2.91E-02		-5.33E-01	6.79E-01		-7.27E-01	6.39E-01		null	null		null	null		Unigene10662_All//gi|224127726|ref|XP_002329162.1|//5.00E-15//tir-nbs-lrr resistance protein [Populus trichocarpa] >gi|222870943|gb|EEF08074.1| tir-nbs-lrr resistance protein [Populus trichocarpa]	Unigene10662_All//4.00E-05//AT4G19530| disease resistance protein (TIR-NBS-LRR class), putative	Unigene10662_All//6.00E-73//TC178046			GO:0005488	nr +
Unigene106629_All	277	3.40	0.00	0.00	0.36	0.62	0.82	0.46	0.75	1.44	-1.17E+01	1.46E-05	Down	-1.17E+01	3.35E-05	Down	7.89E-01	7.72E-01		1.18E+00	5.50E-01		7.20E-01	6.32E-01		1.65E+00	1.41E-01		Unigene106629_All//gi|195621024|gb|ACG32342.1|//5.00E-18//histone mRNA exonuclease 1 [Zea mays]	Unigene106629_All//1.00E-17//AT3G15140| exonuclease family protein	Unigene106629_All//2.00E-110//TC143702				nr +
Unigene10663_All	746	0.00	0.00	0.00	0.00	0.00	0.00	1.76	2.41	0.11	null	null		null	null		null	null		null	null		4.55E-01	3.20E-01		-4.04E+00	1.35E-07	Down	Unigene10663_All//gi|80973464|gb|ABB53473.1|//5.00E-86//GDP-mannose pyrophosphorylase [Malpighia glabra] >gi|161898831|gb|ABX80393.1| GDP-mannose pyrophosphorylase [Malpighia glabra]	Unigene10663_All//9.00E-86//AT2G39770| CYT1 (CYTOKINESIS DEFECTIVE 1); mannose-1-phosphate guanylyltransferase/ nucleotidyltransferase			ko00520|Amino sugar and nucleotide sugar metabolism//ko00051|Fructose and mannose metabolism	GO:0008152//GO:0008905//GO:0043231	nr -
Unigene10664_All	693	0.00	0.00	0.00	0.00	0.00	0.00	25.69	2.35	0.52	null	null		null	null		null	null		null	null		-3.45E+00	1.05E-81	Down	-5.63E+00	6.87E-116	Down	Unigene10664_All//gi|226501140|ref|NP_001152197.1|//9.00E-11//LOC100285835 [Zea mays] >gi|195653721|gb|ACG46328.1|keratin-associated protein 5-4 [Zea mays]	Unigene10664_All//2.00E-07//AT5G05660| ATNFXL2; protein binding / transcription factor/ zinc ion binding			ko00230|Purine metabolism//ko00240|Pyrimidine metabolism//ko00520|Amino sugar and nucleotide sugar metabolism//ko03020|RNA polymerase		nr +
Unigene10665_All	267	0.00	0.00	0.20	4.66	4.13	1.01	0.95	26.46	1.19	null	null		7.64E+00	6.41E-01		-1.77E-01	7.95E-01		-2.20E+00	1.10E-03		4.80E+00	3.93E-41	Up	3.29E-01	7.64E-01				Unigene10665_All//2.00E-45//gi|13244098|gb|BE053719.2|BE053719				
Unigene106654_All	283	0.18	0.36	2.07	0.00	0.00	10.21	0.00	0.00	0.00	9.54E-01	7.28E-01		3.48E+00	1.21E-02		null	null		1.33E+01	2.44E-17	Up	null	null		null	null		Unigene106654_All//sp|Q9U8M0|VIT1_PERAM//1.00E-07//Vitellogenin-1 OS=Periplaneta americana GN=VG2 PE=2 SV=1						swissprot +
Unigene10666_All	441	0.00	0.11	0.00	0.11	0.31	1.13	1.73	0.47	0.00	6.84E+00	6.22E-01		null	null		1.47E+00	6.20E-01		3.32E+00	2.31E-02		-1.86E+00	1.82E-02		-1.08E+01	8.89E-06	Down							
Unigene106662_All	284	0.00	0.00	0.00	0.00	0.12	2.54	0.60	0.29	0.56	null	null		null	null		6.92E+00	8.39E-01		1.13E+01	2.31E-04	Up	-1.02E+00	6.15E-01		-8.58E-02	9.66E-01		Unigene106662_All//gi|15237835|ref|NP_200728.1|//4.00E-36//pentatricopeptide (PPR) repeat-containing protein [Arabidopsis thaliana]	Unigene106662_All//2.00E-37//AT5G59200| pentatricopeptide (PPR) repeat-containing protein					nr -
Unigene10667_All	287	0.00	0.00	0.00	0.00	0.00	0.00	2.06	2.91	0.00	null	null		null	null		null	null		null	null		4.98E-01	4.86E-01		-1.10E+01	1.42E-04	Down	Unigene10667_All//gi|159462882|ref|XP_001689671.1|//3.00E-06//polyadenylate-binding protein RB47 [Chlamydomonas reinhardtii] >gi|158283659|gb|EDP09409.1|polyadenylate-binding protein RB47 [Chlamydomonas reinhardtii]					GO:0005488	nr -
Unigene106672_All	287	1.28	2.12	3.15	5.38	2.52	41.23	50.24	20.10	20.96	7.31E-01	4.16E-01		1.31E+00	9.77E-02		-1.09E+00	3.11E-02		2.94E+00	1.42E-40	Up	-1.32E+00	1.63E-20	Down	-1.26E+00	9.90E-20	Down			Unigene106672_All//3.00E-44//gi|78334472|gb|DT554746.1|DT554746				

<table>
<tr><td>Unigene106673_All</td><td>288</td><td>0.36</td><td>0.35</td><td>0.00</td><td>0.35</td><td>2.03</td><td>3.45</td><td>0.59</td><td>0.00</td><td>0.83</td><td>-4.60E-02</td><td>9.91E-01</td><td></td><td>-8.51E+00</td><td>3.99E-01</td><td></td><td>2.55E+00</td><td>2.84E-02</td><td></td><td>3.32E+00</td><td>3.47E-04</td><td>Up</td><td>-9.20E+00</td><td>1.35E-01</td><td></td><td>4.99E-01</td><td>7.08E-01</td><td></td><td>Unigene106673_All//gi|297836510|ref|XP_002886137.1|//4.00E-34//FAT domain-containing protein [Arabidopsis lyrata subsp. lyrata] >gi|297331977|gb|EFH62396.1| FAT domain-containing protein [Arabidopsis lyrata subsp. lyrata]</td><td>Unigene106673_All//1.00E-34//AT2G17930| binding / inositol or phosphatidylinositol kinase/ phosphotransferase, alcohol group as acceptor</td><td></td><td></td><td></td><td></td><td>nr -</td></tr>
<tr><td>Unigene106695_All</td><td>292</td><td>0.00</td><td>0.00</td><td>2.55</td><td>0.34</td><td>0.24</td><td>1.39</td><td>0.43</td><td>0.43</td><td>0.55</td><td>null</td><td>null</td><td></td><td>1.13E+01</td><td>3.28E-04</td><td>Up</td><td>-5.32E-01</td><td>8.19E-01</td><td></td><td>2.03E+00</td><td>1.49E-01</td><td></td><td>-1.67E-02</td><td>1.02E+00</td><td></td><td>3.29E-01</td><td>8.52E-01</td><td></td><td>Unigene106695_All//gi|225442485|ref|XP_002278313.1|//1.00E-44//PREDICTED: similar to PDR8/PEN3 (PLEIOTROPIC DRUG RESISTANCE8); ATPase, coupled to transmembrane movement of substances / cadmium ion transmembrane transporter [Vitis vinifera]</td><td>Unigene106695_All//4.00E-45//AT1G59870| PEN3 (PENETRATION 3); ATPase, coupled to transmembrane movement of substances / cadmium ion transmembrane transporter</td><td>Unigene106695_All//8.00E-08//gi|78354132|gb|DT574406.1|DT574406</td><td></td><td>ko02010|ABC transporters</td><td></td><td>nr -</td></tr>
<tr><td>Unigene10670_All</td><td>252</td><td>2.08</td><td>11.66</td><td>5.49</td><td>5.53</td><td>4.37</td><td>6.45</td><td>20.30</td><td>15.09</td><td>10.91</td><td>2.49E+00</td><td>2.11E-08</td><td>Up</td><td>1.40E+00</td><td>2.28E-02</td><td></td><td>-3.40E-01</td><td>5.62E-01</td><td></td><td>2.21E-01</td><td>6.77E-01</td><td></td><td>-4.28E-01</td><td>7.84E-02</td><td></td><td>-8.96E-01</td><td>1.02E-04</td><td></td><td></td><td></td><td>Unigene10670_All//1.00E-61//TC140097</td><td></td><td></td><td></td><td></td></tr>
<tr><td>Unigene106701_All</td><td>294</td><td>252.53</td><td>294.37</td><td>287.44</td><td>142.33</td><td>97.80</td><td>53.15</td><td>0.29</td><td>0.57</td><td>0.27</td><td>2.21E-01</td><td>9.00E-05</td><td></td><td>1.87E-01</td><td>1.95E-03</td><td></td><td>-5.41E-01</td><td>5.15E-13</td><td></td><td>-1.42E+00</td><td>1.99E-57</td><td>Down</td><td>9.83E-01</td><td>6.35E-01</td><td></td><td>-8.58E-02</td><td>9.74E-01</td><td></td><td></td><td></td><td>Unigene106701_All//1.00E-148//TC144944</td><td></td><td></td><td></td><td></td></tr>
<tr><td>Unigene10671_All</td><td>346</td><td>0.00</td><td>0.00</td><td>0.00</td><td>0.58</td><td>0.00</td><td>0.26</td><td>4.52</td><td>1.09</td><td>2.19</td><td>null</td><td>null</td><td></td><td>null</td><td>null</td><td></td><td>-9.17E+00</td><td>8.48E-02</td><td></td><td>-1.14E+00</td><td>5.70E-01</td><td></td><td>-2.06E+00</td><td>9.44E-05</td><td>Down</td><td>-1.05E+00</td><td>2.17E-02</td><td></td><td></td><td></td><td>Unigene10671_All//8.00E-24//DT464488</td><td></td><td></td><td></td><td></td></tr>
<tr><td>Unigene10673_All</td><td>337</td><td>0.62</td><td>1.05</td><td>0.00</td><td>2.07</td><td>3.68</td><td>2.81</td><td>1.88</td><td>0.00</td><td>0.00</td><td>7.61E-01</td><td>5.57E-01</td><td></td><td>-9.28E+00</td><td>1.47E-01</td><td></td><td>8.30E-01</td><td>1.88E-01</td><td></td><td>4.44E-01</td><td>5.74E-01</td><td></td><td>-1.09E+01</td><td>1.24E-04</td><td>Down</td><td>-1.09E+01</td><td>7.13E-05</td><td>Down</td><td>Unigene10673_All//gi|27476088|gb|AAO17019.1|//2.00E-10//Hypothetical protein [Oryza sativa Japonica Group]</td><td>Unigene10673_All//3.00E-10//AT2G22610| kinesin motor protein-related</td><td></td><td></td><td></td><td>GO:0000166//GO:0003774</td><td>nr +</td></tr>
<tr><td>Unigene106734_All</td><td>305</td><td>2.06</td><td>0.00</td><td>1.22</td><td>0.65</td><td>0.79</td><td>0.74</td><td>0.14</td><td>0.27</td><td>0.26</td><td>-1.10E+01</td><td>7.92E-04</td><td>Down</td><td>-7.52E-01</td><td>4.11E-01</td><td></td><td>2.75E-01</td><td>8.95E-01</td><td></td><td>1.81E-01</td><td>9.35E-01</td><td></td><td>9.84E-01</td><td>7.33E-01</td><td></td><td>9.14E-01</td><td>7.51E-01</td><td></td><td></td><td></td><td>Unigene106734_All//3.00E-07//DR462295</td><td></td><td></td><td></td><td></td></tr>
<tr><td>Unigene106746_All</td><td>309</td><td>0.51</td><td>0.33</td><td>0.52</td><td>0.00</td><td>0.45</td><td>0.73</td><td>6.43</td><td>0.41</td><td>1.42</td><td>-6.31E-01</td><td>7.28E-01</td><td></td><td>2.53E-02</td><td>1.00E+00</td><td></td><td>8.80E+00</td><td>3.20E-01</td><td></td><td>9.51E+00</td><td>1.19E-01</td><td></td><td>-3.99E+00</td><td>1.09E-10</td><td>Down</td><td>-2.18E+00</td><td>1.75E-06</td><td>Down</td><td>Unigene106746_All//gi|84688912|gb|ABC61505.1|//4.00E-16//AGO4-2 [Nicotiana benthamiana]</td><td>Unigene106746_All//2.00E-12//AT5G21150| PAZ domain-containing protein / piwi domain-containing protein</td><td>Unigene106746_All//8.00E-70//gi|48807247|gb|CO108561.1|CO108561</td><td></td><td></td><td></td><td>nr -</td></tr>
<tr><td>Unigene106764_All</td><td>315</td><td>0.00</td><td>0.32</td><td>0.00</td><td>0.32</td><td>2.84</td><td>1.72</td><td>0.00</td><td>0.00</td><td>0.00</td><td>8.33E+00</td><td>3.90E-01</td><td></td><td>null</td><td>null</td><td></td><td>3.17E+00</td><td>8.78E-04</td><td>Up</td><td>2.44E+00</td><td>4.41E-02</td><td></td><td>null</td><td>null</td><td></td><td>null</td><td>null</td><td></td><td></td><td>Unigene106764_All//8.00E-05//AT4G10265| wound-responsive protein, putative</td><td>Unigene106764_All//1.00E-105//gi|109876598|gb|DW505572.1|DW505572</td><td></td><td></td><td></td><td></td></tr>
<tr><td>Unigene106767_All</td><td>317</td><td>1.49</td><td>0.80</td><td>0.34</td><td>0.16</td><td>1.63</td><td>2.85</td><td>0.67</td><td>0.66</td><td>1.13</td><td>-8.94E-01</td><td>3.89E-01</td><td></td><td>-2.14E+00</td><td>1.03E-01</td><td></td><td>3.37E+00</td><td>1.82E-02</td><td></td><td>4.18E+00</td><td>1.95E-04</td><td>Up</td><td>-1.68E-02</td><td>1.02E+00</td><td></td><td>7.62E-01</td><td>5.00E-01</td><td></td><td>Unigene106767_All//gi|15228991|ref|NP_191228.1|//2.00E-17//CIP111 (CAM INTERACTING PROTEIN 111); ATPase/ calmodulin binding [Arabidopsis thaliana] >gi|9662988|emb|CAC00732.1| calmodulin-binding protein [Arabidopsis thaliana]</td><td>Unigene106767_All//8.00E-19//AT3G56690| CIP111 (CAM INTERACTING PROTEIN 111); ATPase/ calmodulin binding</td><td></td><td></td><td></td><td></td><td>nr -</td></tr>
<tr><td>Unigene106777_All</td><td>327</td><td>0.00</td><td>0.00</td><td>0.00</td><td>0.00</td><td>0.63</td><td>2.76</td><td>1.81</td><td>0.77</td><td>1.95</td><td>null</td><td>null</td><td></td><td>null</td><td>null</td><td></td><td>9.30E+00</td><td>1.57E-01</td><td></td><td>1.14E+01</td><td>2.07E-05</td><td>Up</td><td>-1.24E+00</td><td>1.54E-01</td><td></td><td>1.07E-01</td><td>9.02E-01</td><td></td><td>Unigene106777_All//gi|42565171|ref|NP_189132.2|//2.00E-17//DNA binding [Arabidopsis thaliana]</td><td>Unigene106777_All//8.00E-19//AT3G24880| DNA binding</td><td></td><td></td><td></td><td></td><td>nr +</td></tr>
<tr><td>Unigene10678_All</td><td>647</td><td>0.00</td><td>0.08</td><td>0.00</td><td>0.69</td><td>0.16</td><td>0.42</td><td>0.00</td><td>0.00</td><td>2.89</td><td>6.29E+00</td><td>6.22E-01</td><td></td><td>null</td><td>null</td><td></td><td>-2.12E+00</td><td>7.28E-02</td><td></td><td>-7.26E-01</td><td>5.33E-01</td><td></td><td>null</td><td>null</td><td></td><td>1.15E+01</td><td>1.95E-13</td><td>Up</td><td>Unigene10678_All//gi|87240526|gb|ABD32384.1|//7.00E-28//Peptidase A1, pepsin [Medicago truncatula]</td><td>Unigene10678_All//1.00E-16//AT1G03220| extracellular dermal glycoprotein, putative / EDGP, putative</td><td>Unigene10678_All//1.00E-135//TC176754</td><td></td><td></td><td>GO:0005618//GO:0006970//GO:0016020</td><td>nr -</td></tr>
<tr><td>Unigene10679_All</td><td>270</td><td>1.55</td><td>3.38</td><td>7.10</td><td>20.29</td><td>15.43</td><td>17.73</td><td>5.79</td><td>3.72</td><td>3.98</td><td>1.12E+00</td><td>1.30E-01</td><td></td><td>2.20E+00</td><td>8.21E-05</td><td>Up</td><td>-3.95E-01</td><td>1.23E-01</td><td></td><td>-1.95E-01</td><td>5.01E-01</td><td></td><td>-6.41E-01</td><td>1.82E-01</td><td></td><td>-5.40E-01</td><td>2.40E-01</td><td></td><td>Unigene10679_All//gi|147802546|emb|CAN77665.1|//4.00E-06//hypothetical protein [Vitis vinifera]</td><td></td><td>Unigene10679_All//2.00E-61//ES823904</td><td></td><td></td><td></td><td>nr +</td></tr>
<tr><td>Unigene106796_All</td><td>342</td><td>0.00</td><td>0.44</td><td>0.00</td><td>0.00</td><td>0.00</td><td>3.43</td><td>0.00</td><td>0.00</td><td>0.00</td><td>8.80E+00</td><td>2.37E-01</td><td></td><td>null</td><td>null</td><td></td><td>null</td><td>null</td><td></td><td>1.17E+01</td><td>5.32E-07</td><td>Up</td><td>null</td><td>null</td><td></td><td>null</td><td>null</td><td></td><td></td><td></td><td></td><td></td><td></td><td></td><td>ESTscan +</td></tr>
<tr><td>Unigene106799_All</td><td>346</td><td>0.00</td><td>0.00</td><td>0.00</td><td>0.00</td><td>0.00</td><td>3.13</td><td>0.00</td><td>0.00</td><td>0.00</td><td>null</td><td>null</td><td></td><td>null</td><td>null</td><td></td><td>null</td><td>null</td><td></td><td>1.16E+01</td><td>1.82E-06</td><td>Up</td><td>null</td><td>null</td><td></td><td>null</td><td>null</td><td></td><td>Unigene106799_All//gi|30696750|ref|NP_176463.2|//2.00E-07//invertase/pectin methylesterase inhibitor family protein [Arabidopsis thaliana] >gi|6630459|gb|AAF19547.1|AC007190_15 F23N19.12 [Arabidopsis thaliana]</td><td></td><td></td><td></td><td>ko00230|Purine metabolism//ko00240|Pyrimidine metabolism//ko03020|RNA polymerase</td><td></td><td>nr -</td></tr>
<tr><td>Unigene10680_All</td><td>319</td><td>0.00</td><td>0.00</td><td>0.00</td><td>0.00</td><td>0.00</td><td>0.00</td><td>0.00</td><td>4.06</td><td>1.25</td><td>null</td><td>null</td><td></td><td>null</td><td>null</td><td></td><td>null</td><td>null</td><td></td><td>null</td><td>null</td><td></td><td>1.20E+01</td><td>3.67E-09</td><td>Up</td><td>1.03E+01</td><td>3.97E-03</td><td></td><td></td><td></td><td></td><td></td><td></td><td></td><td></td></tr>
<tr><td>Unigene106801_All</td><td>346</td><td>0.00</td><td>1.76</td><td>1.23</td><td>0.58</td><td>0.30</td><td>7.70</td><td>0.12</td><td>0.00</td><td>0.46</td><td>1.08E+01</td><td>1.17E-03</td><td></td><td>1.03E+01</td><td>1.39E-02</td><td></td><td>-9.48E-01</td><td>5.88E-01</td><td></td><td>3.74E+00</td><td>1.56E-11</td><td>Up</td><td>-6.93E+00</td><td>6.63E-01</td><td></td><td>1.91E+00</td><td>3.78E-01</td><td></td><td>Unigene106801_All//gi|297807543|ref|XP_002871655.1|//5.00E-22//amino acid transporter family protein [Arabidopsis lyrata subsp. lyrata] >gi|297317492|gb|EFH47914.1| amino acid transporter family protein [Arabidopsis lyrata subsp. lyrata]</td><td>Unigene106801_All//9.00E-23//AT5G15240| amino acid transporter family protein</td><td>Unigene106801_All//5.00E-167//gi|48826851|gb|CO125241.1|CO125241</td><td></td><td></td><td></td><td>nr +</td></tr>
<tr><td>Unigene106810_All</td><td>358</td><td>7901.32</td><td>3489.26</td><td>7207.19</td><td>190.63</td><td>308.86</td><td>168.67</td><td>0.00</td><td>0.23</td><td>0.00</td><td>-1.18E+00</td><td>0.00E+00</td><td>Down</td><td>-1.33E-01</td><td>3.49E-47</td><td></td><td>6.96E-01</td><td>2.54E-51</td><td></td><td>-1.77E-01</td><td>6.91E-03</td><td></td><td>7.87E+00</td><td>4.03E-01</td><td></td><td>null</td><td>null</td><td></td><td>Unigene106810_All//gi|12620881|gb|AAG61120.1|//1.00E-65//ribulose-1,5-bisphosphate carboxylase/oxygenase activase 1 [Gossypium hirsutum]</td><td>Unigene106810_All//3.00E-54//AT2G39730| RCA (RUBISCO ACTIVASE); ADP binding / ATP binding / enzyme regulator/ ribulose-1,5-bisphosphate carboxylase/oxygenase activator</td><td>Unigene106810_All//0.00E+00//EX168151</td><td></td><td></td><td></td><td>nr +</td></tr>
<tr><td>Unigene106812_All</td><td>362</td><td>0.00</td><td>2.24</td><td>0.00</td><td>0.00</td><td>0.57</td><td>2.62</td><td>0.82</td><td>0.35</td><td>0.99</td><td>1.11E+01</td><td>9.39E-05</td><td>Up</td><td>null</td><td>null</td><td></td><td>9.16E+00</td><td>1.57E-01</td><td></td><td>1.14E+01</td><td>1.13E-05</td><td>Up</td><td>-1.24E+00</td><td>3.73E-01</td><td></td><td>2.77E-01</td><td>7.88E-01</td><td></td><td>Unigene106812_All//gi|225465575|ref|XP_002263953.1|//5.00E-08//PREDICTED: hypothetical protein [Vitis vinifera] >gi|147865101|emb|CAN79390.1| hypothetical protein [Vitis vinifera]</td><td></td><td></td><td></td><td>ko03040|Spliceosome</td><td></td><td>nr -</td></tr>
<tr><td>Unigene10682_All</td><td>491</td><td>0.00</td><td>0.00</td><td>0.00</td><td>0.00</td><td>0.00</td><td>0.00</td><td>3.10</td><td>2.21</td><td>0.00</td><td>null</td><td>null</td><td></td><td>null</td><td>null</td><td></td><td>null</td><td>null</td><td></td><td>null</td><td>null</td><td></td><td>-4.86E-01</td><td>3.35E-01</td><td></td><td>-1.16E+01</td><td>2.88E-11</td><td>Down</td><td>Unigene10682_All//gi|15241051|ref|NP_198129.1|//2.00E-37//60S ribosomal protein L22 (RPL22C) [Arabidopsis thaliana] >gi|17865557|sp|Q9FE58.1|RL223_ARATH RecName: Full=60S ribosomal protein L22-3 >gi|11692938|gb|AAG40072.1|AF324721_1 T1G16 [Arabidopsis thaliana] >gi|21554057|gb|AAM63138.1| 60S ribosomal protein L22-like [Arabidopsis thaliana] >gi|27808502|gb|AAO24531.1| At5g27770 [Arabidopsis thaliana] >gi|110736618|dbj|BAF00273.1| 60S ribosomal protein L22 - like [Arabidopsis thaliana]</td><td>Unigene10682_All//1.00E-25//AT5G27770| 60S ribosomal protein L22 (RPL22C)</td><td></td><td></td><td>ko03010|Ribosome</td><td>GO:0005198//GO:0010467//GO:0015934//GO:0016020//GO:0031981</td><td>nr +</td></tr>
<tr><td>Unigene106820_All</td><td>371</td><td>0.00</td><td>0.00</td><td>0.00</td><td>0.00</td><td>0.00</td><td>0.37</td><td>8.66</td><td>0.79</td><td>1.07</td><td>null</td><td>null</td><td></td><td>null</td><td>null</td><td></td><td>null</td><td>null</td><td></td><td>8.51E+00</td><td>3.12E-01</td><td></td><td>-3.46E+00</td><td>3.09E-15</td><td>Down</td><td>-3.01E+00</td><td>5.32E-14</td><td>Down</td><td>Unigene106820_All//gi|1743354|emb|CAA71003.1|//6.00E-35//aldehyde dehydrogenase (NAD+) [Nicotiana tabacum]</td><td>Unigene106820_All//3.00E-34//AT3G48000| ALDH2B4 (ALDEHYDE DEHYDROGENASE 2B4); 3-chloroallyl aldehyde dehydrogenase/ ATP binding / aldehyde dehydrogenase (NAD)</td><td>Unigene106820_All//4.00E-35//EG702325</td><td></td><td>ko00940|Phenylpropanoid biosynthesis//ko00010|Glycolysis / Gluconeogenesis//ko00903|Limonene and pinene degradation//ko00620|Pyruvate metabolism//ko00380|Tryptophan metabolism//ko00071|Fatty acid metabolism//ko00053|Ascorbate and aldarate metabolism//ko00330|Arginine and proline metabolism//ko00280|Valine, leucine and isoleucine degradation//ko00640|Propanoate metabolism//ko00561|Glycerolipid metabolism//ko00310|Lysine degradation//ko00410|beta-Alanine metabolism//ko00340|Histidine metabolism</td><td></td><td>nr -</td></tr>
<tr><td>Unigene106829_All</td><td>377</td><td>2.22</td><td>11.56</td><td>3.67</td><td>25.11</td><td>65.49</td><td>38.93</td><td>0.79</td><td>2.66</td><td>3.80</td><td>2.38E+00</td><td>1.47E-11</td><td>Up</td><td>7.26E-01</td><td>2.28E-01</td><td></td><td>1.38E+00</td><td>3.11E-35</td><td>Up</td><td>6.33E-01</td><td>9.30E-06</td><td></td><td>1.76E+00</td><td>7.67E-03</td><td></td><td>2.28E+00</td><td>4.94E-05</td><td>Up</td><td>Unigene106829_All//gi|15241290|ref|NP_197519.1|//3.00E-23//binding [Arabidopsis thaliana]</td><td>Unigene106829_All//1.00E-24//AT5G20190| binding</td><td>Unigene106829_All//5.00E-84//TC165464</td><td></td><td></td><td></td><td>nr -</td></tr>
<tr><td>Unigene106832_All</td><td>387</td><td>0.14</td><td>0.00</td><td>0.00</td><td>0.00</td><td>0.00</td><td>2.45</td><td>0.00</td><td>0.00</td><td>0.10</td><td>-7.08E+00</td><td>6.20E-01</td><td></td><td>-7.08E+00</td><td>6.33E-01</td><td></td><td>null</td><td>null</td><td></td><td>1.13E+01</td><td>1.13E-05</td><td>Up</td><td>null</td><td>null</td><td></td><td>6.69E+00</td><td>6.48E-01</td><td></td><td>Unigene106832_All//gi|5231127|gb|AAD41083.1|AF151961_1//5.00E-61//1-aminocyclopropane-1-carboxylic acid synthase [Vigna radiata]</td><td>Unigene106832_All//9.00E-55//AT3G49700| ACS9 (1-AMINOCYCLOPROPANE-1-CARBOXYLATE SYNTHASE 9); 1-aminocyclopropane-1-carboxylate synthase</td><td></td><td></td><td>ko00270|Cysteine and methionine metabolism</td><td></td><td>nr +</td></tr>
<tr><td>Unigene106834_All</td><td>388</td><td>0.00</td><td>0.52</td><td>0.00</td><td>0.51</td><td>1.33</td><td>3.96</td><td>0.44</td><td>0.32</td><td>0.00</td><td>9.03E+00</td><td>1.37E-01</td><td></td><td>null</td><td>null</td><td></td><td>1.37E+00</td><td>2.26E-01</td><td></td><td>2.95E+00</td><td>1.22E-05</td><td>Up</td><td>-4.32E-01</td><td>7.94E-01</td><td></td><td>-8.77E+00</td><td>1.09E-01</td><td></td><td>Unigene106834_All//gi|3121837|sp|O04940.1|CDS1_SOLTU//1.00E-53//RecName: Full=Phosphatidate cytidylyltransferase; AltName: Full=CDP-diglyceride pyrophosphorylase; AltName: Full=CDP-diglyceride synthase; AltName: Full=CDP-diacylglycerol synthase; Short=CDS; AltName: Full=CTP:phosphatidate cytidylyltransferase; AltName: Full=CDP-DG synthase; AltName: Full=CDP-DAG synthase >gi|2182104|emb|CAA63004.1| CDP-diacylglycerol synthetase [Solanum tuberosum]</td><td>Unigene106834_All//7.00E-50//AT4G22340| phosphatidate cytidylyltransferase, putative / CDP-diglyceride synthetase, putative</td><td>Unigene106834_All//1.00E-103//CO498072</td><td></td><td>ko04070|Phosphatidylinositol signaling system//ko00564|Glycerophospholipid metabolism</td><td></td><td>nr -</td></tr>
<tr><td>Unigene106836_All</td><td>389</td><td>0.81</td><td>0.00</td><td>1.10</td><td>0.38</td><td>1.68</td><td>3.25</td><td>0.00</td><td>0.11</td><td>0.10</td><td>-9.66E+00</td><td>3.67E-02</td><td></td><td>4.40E-01</td><td>6.77E-01</td><td></td><td>2.13E+00</td><td>3.78E-02</td><td></td><td>3.08E+00</td><td>6.55E-05</td><td>Up</td><td>6.75E+00</td><td>6.38E-01</td><td></td><td>6.68E+00</td><td>6.48E-01</td><td></td><td></td><td></td><td></td><td></td><td></td><td></td><td></td></tr>
<tr><td>Unigene106838_All</td><td>395</td><td>0.00</td><td>1.67</td><td>0.00</td><td>1.01</td><td>0.96</td><td>2.97</td><td>1.50</td><td>1.38</td><td>0.81</td><td>1.07E+01</td><td>6.26E-04</td><td>Up</td><td>null</td><td>null</td><td></td><td>-7.32E-02</td><td>9.26E-01</td><td></td><td>1.56E+00</td><td>1.95E-02</td><td></td><td>-1.24E-01</td><td>8.84E-01</td><td></td><td>-8.93E-01</td><td>2.72E-01</td><td></td><td></td><td></td><td>Unigene106838_All//5.00E-07//TC140528</td><td></td><td></td><td></td><td></td></tr>
<tr><td>Unigene10684_All</td><td>500</td><td>0.00</td><td>0.00</td><td>0.00</td><td>0.00</td><td>0.00</td><td>0.00</td><td>1.86</td><td>2.84</td><td>0.16</td><td>null</td><td>null</td><td></td><td>null</td><td>null</td><td></td><td>null</td><td>null</td><td></td><td>null</td><td>null</td><td></td><td>6.11E-01</td><td>2.36E-01</td><td></td><td>-3.54E+00</td><td>3.97E-05</td><td>Down</td><td>Unigene10684_All//gi|145353927|ref|XP_001421249.1|//1.00E-07//predicted protein [Ostreococcus lucimarinus CCE9901] >gi|145354001|ref|XP_001421285.1| predicted protein [Ostreococcus lucimarinus CCE9901] >gi|144581486|gb|ABO99542.1| predicted protein [Ostreococcus lucimarinus CCE9901] >gi|144581522|gb|ABO99578.1| predicted protein [Ostreococcus lucimarinus CCE9901]</td><td></td><td></td><td></td><td></td><td></td><td>nr +</td></tr>
<tr><td>Unigene106841_All</td><td>398</td><td>1.84</td><td>0.00</td><td>0.13</td><td>0.38</td><td>0.61</td><td>0.57</td><td>0.64</td><td>0.00</td><td>0.20</td><td>-1.08E+01</td><td>2.11E-04</td><td>Down</td><td>-3.78E+00</td><td>2.79E-03</td><td></td><td>6.90E-01</td><td>7.32E-01</td><td></td><td>5.96E-01</td><td>7.22E-01</td><td></td><td>-9.32E+00</td><td>4.06E-02</td><td></td><td>-1.67E+00</td><td>2.57E-01</td><td></td><td>Unigene106841_All//gi|52789958|gb|AAU87582.1|//7.00E-60//MADS9 protein [Gossypium hirsutum]</td><td>Unigene106841_All//6.00E-14//AT5G13790| AGL15 (AGAMOUS-LIKE 15); DNA binding / transcription factor</td><td>Unigene106841_All//2.00E-154//TC129654</td><td>MADS</td><td></td><td></td><td>nr -</td></tr>
<tr><td>Unigene106842_All</td><td>398</td><td>0.00</td><td>0.00</td><td>0.27</td><td>0.00</td><td>0.00</td><td>10.89</td><td>0.00</td><td>0.00</td><td>0.00</td><td>null</td><td>null</td><td></td><td>8.06E+00</td><td>4.02E-01</td><td></td><td>null</td><td>null</td><td></td><td>1.34E+01</td><td>3.67E-26</td><td>Up</td><td>null</td><td>null</td><td></td><td>null</td><td>null</td><td></td><td>Unigene106842_All//sp|Q16927|VIT1_AEDAE//2.00E-17//Vitellogenin-A1 OS=Aedes aegypti GN=VGA1 PE=1 SV=2</td><td></td><td></td><td></td><td></td><td></td><td>swissprot -</td></tr>
</table>

Unigene106844_All	406	0.52	0.50	0.52	0.12	0.59	2.89	1.15	0.21	1.57	-4.64E-02	9.89E-01		2.52E-02	1.00E+00		2.27E+00	3.05E-01		4.56E+00	6.41E-06	Up	-2.48E+00	3.38E-02		4.55E-01	5.78E-01		Unigene106844_All//gi|297841629|ref|XP_002888696.1|//4.00E-38//prolyl oligopeptidase family protein [Arabidopsis lyrata subsp. lyrata] >gi|297334537|gb|EFH64955.1| prolyl oligopeptidase family protein [Arabidopsis lyrata subsp. lyrata]	Unigene106844_All//4.00E-37//AT1G69020| prolyl oligopeptidase family protein					nr -
Unigene106847_All	411	0.00	0.00	0.00	0.12	0.34	2.09	0.51	0.71	0.29	null	null		null	null		1.47E+00	6.20E-01		4.11E+00	3.41E-04	Up	4.69E-01	7.07E-01		-8.23E-01	6.00E-01		Unigene106847_All//gi|115488208|ref|NP_001066591.1|//1.00E-10//Os12g0288000 [Oryza sativa Japonica Group] >gi|77554969|gb|ABA97765.1| Integral membrane protein DUF6 containing protein, expressed [Oryza sativa (japonica cultivar-group)] >gi|113649098|dbj|BAF29610.1| Os12g0288000 [Oryza sativa Japonica Group]	Unigene106847_All//3.00E-08//AT5G64700| nodulin MtN21 family protein	Unigene106847_All//2.00E-34//TC144438				nr -
Unigene106850_All	418	2.38	2007.38	1212.27	0.36	0.33	10.16	0.51	0.20	0.10	9.72E+00	0.00E+00	Up	8.99E+00	0.00E+00	Up	-1.18E-01	9.11E-01		4.83E+00	1.91E-21	Up	-1.34E+00	4.31E-01		-2.41E+00	1.95E-01				Unigene106850_All//9.00E-21//TC149074				
Unigene106851_All	421	0.75	0.00	0.00	0.00	0.00	7.94	0.00	0.10	0.28	-9.54E+00	3.67E-02		-9.54E+00	4.93E-02		null	null		1.30E+01	4.33E-20	Up	6.63E+00	6.38E-01		8.15E+00	2.44E-01		Unigene106851_All//gi|224127212|ref|XP_002320015.1|//8.00E-59//1-aminocyclopropane-1-carboxylate [Populus trichocarpa] >gi|222860788|gb|EEE98330.1| 1-aminocyclopropane-1-carboxylate [Populus trichocarpa]	Unigene106851_All//2.00E-57//AT5G65800| ACS5 (ACC SYNTHASE 5); 1-aminocyclopropane-1-carboxylate synthase			ko00270|Cysteine and methionine metabolism		nr -
Unigene106852_All	422	0.99	0.48	3.91	0.35	0.57	0.43	0.00	0.00	0.00	-1.05E+00	3.66E-01		1.98E+00	8.06E-04	Up	6.90E-01	7.32E-01		2.74E-01	9.06E-01		null	null		null	null		Unigene106852_All//gi|291506702|gb|ADE08783.1|//1.00E-29//phytochrome interacting factor 4 [Arabidopsis thaliana] >gi|291506704|gb|ADE08784.1| phytochrome interacting factor 4 [Arabidopsis thaliana] >gi|291506706|gb|ADE08785.1| phytochrome interacting factor 4 [Arabidopsis thaliana] >gi|291506708|gb|ADE08786.1| phytochrome interacting factor 4 [Arabidopsis thaliana] >gi|291506710|gb|ADE08787.1| phytochrome interacting factor 4 [Arabidopsis thaliana] >gi|291506712|gb|ADE08788.1| phytochrome interacting factor 4 [Arabidopsis thaliana]	Unigene106852_All//8.00E-29//AT2G43010| PIF4 (phytochrome interacting factor 4); DNA binding / protein binding / transcription factor	Unigene106852_All//5.00E-10//TC178874	bHLH	ko04626|Plant-pathogen interaction//ko04712|Circadian rhythm - plant		nr -
Unigene106856_All	432	0.00	0.23	0.49	0.92	1.91	5.85	0.00	0.00	0.00	7.87E+00	3.90E-01		8.95E+00	1.42E-01		1.05E+00	1.99E-01		2.67E+00	3.65E-08	Up	null	null		null	null		Unigene106856_All//gi|297850352|ref|XP_002893057.1|//1.00E-33//S-adenosyl-L-methionine:jasmonic acid carboxyl methyltransferase [Arabidopsis lyrata subsp. lyrata] >gi|297338899|gb|EFH69316.1| S-adenosyl-L-methionine:jasmonic acid carboxyl methyltransferase [Arabidopsis lyrata subsp. lyrata]	Unigene106856_All//1.00E-33//AT1G19640| JMT (JASMONIC ACID CARBOXYL METHYLTRANSFERASE); jasmonate O-methyltransferase			ko00592|alpha-Linolenic acid metabolism		nr +
Unigene106857_All	434	0.00	0.23	0.74	0.69	0.00	4.16	0.19	0.29	0.00	7.87E+00	3.90E-01		9.52E+00	4.54E-02		-9.43E+00	2.02E-02		2.60E+00	7.99E-06	Up	5.69E-01	7.83E-01		-7.61E+00	3.61E-01		Unigene106857_All//gi|21536895|gb|AAM61227.1|//3.00E-64//amino acid transport protein AAP2 [Arabidopsis thaliana]	Unigene106857_All//2.00E-65//AT5G09220| AAP2 (AMINO ACID PERMEASE 2); amino acid transmembrane transporter	Unigene106857_All//0.00E+00//gi|48814445|gb|CO115758.1|CO115758				nr -
Unigene106865_All	460	2.27	0.99	0.23	0.65	0.97	3.73	0.00	0.36	0.09	-1.20E+00	7.76E-02		-3.30E+00	4.59E-04	Down	5.83E-01	6.26E-01		2.52E+00	2.09E-05	Up	8.51E+00	1.39E-01		6.44E+00	6.48E-01								
Unigene106868_All	472	0.00	1.83	0.45	1.27	0.80	0.29	0.09	0.27	0.51	1.08E+01	4.95E-05	Up	8.82E+00	1.42E-01		-6.58E-01	4.78E-01		-2.14E+00	4.39E-02		1.57E+00	5.46E-01		2.50E+00	1.59E-01				Unigene106868_All//0.00E+00//gi|164288680|gb|ES800533.1|ES800533				ESTscan -
Unigene106869_All	492	0.43	5.87	2.06	0.20	0.91	4.31	0.17	0.34	0.32	3.79E+00	5.53E-12	Up	2.27E+00	5.77E-03		2.17E+00	1.06E-01		4.41E+00	2.73E-10	Up	9.83E-01	6.35E-01		9.14E-01	6.25E-01		Unigene106869_All//gi|292668975|gb|ADE41142.1|//6.00E-42//AP2 domain class transcription factor [Malus x domestica]	Unigene106869_All//3.00E-24//AT5G11590| TINY2 (TINY2); DNA binding / transcription factor	Unigene106869_All//0.00E+00//TC178420	AP2-EREBP			nr +
Unigene10687_All	465	0.00	3.92	3.78	0.00	0.00	0.00	0.00	0.00	0.00	1.19E+01	2.14E-10	Up	1.19E+01	1.12E-09	Up	null	null		null	null		null	null		null	null		Unigene10687_All//gi|15021748|gb|AAK77901.1|AF397029_1//3.00E-15//root nodule extensin [Pisum sativum]				ko03040|Spliceosome//ko04144|Endocytosis		nr +
Unigene106870_All	507	0.00	0.00	0.00	0.00	0.00	1.78	0.00	0.00	0.00	null	null		null	null		null	null		1.08E+01	2.07E-05	Up	null	null		null	null								
Unigene106871_All	507	0.00	0.00	0.00	0.00	0.27	3.83	0.00	0.00	0.00	null	null		null	null		8.09E+00	3.20E-01		1.19E+01	1.36E-11	Up	null	null		null	null		Unigene106871_All//sp|P08171|EST6_DROME//9.00E-09//Esterase-6 OS=Drosophila melanogaster GN=Est-6 PE=1 SV=2						swissprot +
Unigene106875_All	543	0.29	0.09	0.00	0.28	0.06	3.41	0.16	0.23	0.15	-1.63E+00	4.89E-01		-8.18E+00	2.46E-01		-2.12E+00	3.87E-01		3.63E+00	6.06E-08	Up	5.69E-01	7.83E-01		-8.59E-02	9.74E-01		Unigene106875_All//gi|224053975|ref|XP_002298066.1|//1.00E-38//AP2/ERF domain-containing transcription factor [Populus trichocarpa] >gi|222845324|gb|EEE82871.1| AP2/ERF domain-containing transcription factor [Populus trichocarpa]	Unigene106875_All//2.00E-26//AT1G12630| DNA binding / transcription activator/ transcription factor	Unigene106875_All//8.00E-130//TC163291	AP2-EREBP			nr -
Unigene106876_All	573	0.00	0.18	0.00	0.00	0.12	7.09	0.00	0.00	0.00	7.47E+00	3.90E-01		null	null		6.91E+00	6.15E-01		1.28E+01	1.68E-24	Up	null	null		null	null		Unigene106876_All//sp|Q05808|VIT_ANTGR//3.00E-31//Vitellogenin OS=Anthonomus grandis GN=VTG PE=4 SV=1						swissprot +
Unigene106877_All	600	0.17	1.35	0.62	0.66	1.89	3.76	0.00	0.00	0.00	2.95E+00	3.29E-03		1.83E+00	2.13E-01		1.51E+00	2.36E-02		2.50E+00	6.84E-07	Up	null	null		null	null		Unigene106877_All//gi|87241322|gb|ABD33180.1|//5.00E-29//TGF-beta receptor, type I/II extracellular region [Medicago truncatula]	Unigene106877_All//1.00E-27//AT1G33440| proton-dependent oligopeptide transport (POT) family protein	Unigene106877_All//0.00E+00//CO119167				nr +
Unigene106882_All	446	0.00	0.00	0.00	0.00	0.00	0.00	21.23	1.31	1.07	null	null		null	null		null	null		null	null		-4.02E+00	2.42E-49	Down	-4.31E+00	1.36E-53	Down							
Unigene106883_All	232	0.00	0.00	1.84	0.21	0.00	0.00	3.28	2.16	8.59	null	null		1.08E+01	1.38E-02		-7.75E+00	5.64E-01		-7.75E+00	6.45E-01		-6.02E-01	4.12E-01		1.39E+00	7.70E-04	Up	Unigene106883_All//gi|15219445|ref|NP_178085.1|//7.00E-20//RFO1 (RESISTANCE TO FUSARIUM OXYSPORUM 1); kinase [Arabidopsis thaliana] >gi|75247675|sp|Q8RY17.1|WAKLI_ARATH RecName: Full=Wall-associated receptor kinase-like 22; Flags: Precursor >gi|19310447|gb|AAL84959.1| At1g79670/F20B17_27 [Arabidopsis thaliana]	Unigene106883_All//3.00E-21//AT1G79670| RFO1 (RESISTANCE TO FUSARIUM OXYSPORUM 1); kinase	Unigene106883_All//1.00E-43//DT462731		ko04626|Plant-pathogen interaction		nr -
Unigene106884_All	641	0.00	0.00	0.00	0.00	0.00	0.00	2.84	0.72	0.44	null	null		null	null		null	null		null	null		-1.98E+00	3.39E-05	Down	-2.70E+00	1.86E-07	Down	Unigene106884_All//gi|157011451|gb|ABV00887.1|//1.00E-100//actin [Rhodomonas sp. CCMP768]	Unigene106884_All//2.00E-97//AT3G53750| ACT3 (actin 3); structural constituent of cytoskeleton	Unigene106884_All//4.00E-33//gi|5045286|gb|AI726434.1|AI726434				nr -
Unigene106886_All	379	0.00	0.00	0.00	0.00	0.00	0.00	1.78	0.00	0.00	null	null		null	null		null	null		null	null		-1.08E+01	6.35E-05	Down	-1.08E+01	3.57E-05	Down	Unigene106886_All//gi|253998300|ref|YP_003050363.1|//1.00E-08//cold-shock DNA-binding domain protein [Methylovorus sp. SIP3-4] >gi|253984979|gb|ACT49836.1| cold-shock DNA-binding domain protein [Methylovorus sp. SIP3-4]	Unigene106886_All//4.00E-07//AT4G36020| CSDP1 (cold shock domain protein 1); RNA binding / double-stranded DNA binding / nucleic acid binding / single-stranded DNA binding		CSD			nr +
Unigene106887_All	292	0.00	0.00	0.00	0.00	0.00	0.00	2.75	1.00	0.00	null	null		null	null		null	null		null	null		-1.46E+00	4.65E-02		-1.14E+01	4.43E-06	Down							
Unigene106888_All	595	0.00	0.00	0.00	0.00	0.00	0.00	5.12	0.56	0.20	null	null		null	null		null	null		null	null		-3.19E+00	1.70E-13	Down	-4.67E+00	1.91E-18	Down	Unigene106888_All//gi|300266840|gb|EFJ51026.1|//8.00E-46//component of cytosolic 80S ribosome and 40S small subunit [Volvox carteri f. nagariensis]	Unigene106888_All//5.00E-41//AT3G02560| 40S ribosomal protein S7 (RPS7B)			ko03010|Ribosome		nr -
Unigene10689_All	443	1.89	1.14	0.48	1.24	0.62	1.63	2.58	0.38	1.08	-7.24E-01	3.48E-01		-1.97E+00	2.71E-02		-9.92E-01	3.13E-01		3.99E-01	6.52E-01		-2.77E+00	7.62E-05	Down	-1.26E+00	2.42E-02								
Unigene106890_All	485	0.00	0.00	0.00	0.00	0.00	0.00	2.44	0.17	0.00	null	null		null	null		null	null		null	null		-3.82E+00	2.12E-06	Down	-1.13E+01	8.15E-09	Down							
Unigene106892_All	430	0.00	0.00	0.00	0.00	0.00	0.00	6.29	1.07	0.56	null	null		null	null		null	null		null	null		-2.56E+00	9.03E-10	Down	-3.50E+00	1.50E-13	Down							ESTscan -
Unigene106893_All	807	0.00	0.00	0.00	0.00	0.00	0.00	3.41	0.67	0.25	null	null		null	null		null	null		null	null		-2.34E+00	5.13E-09	Down	-3.79E+00	1.33E-14	Down	Unigene106893_All//gi|300267515|gb|EFJ51698.1|//4.00E-79//ADP/ATP carrier protein, probably mitochondrial [Volvox carteri f. nagariensis]	Unigene106893_All//2.00E-72//AT4G28390| AAC3 (ADP/ATP CARRIER 3); ATP:ADP antiporter/ binding	Unigene106893_All//1.00E-06//EV483520				nr -
Unigene106894_All	268	0.00	0.00	0.00	0.00	0.00	0.00	2.84	0.31	0.00	null	null		null	null		null	null		null	null		-3.19E+00	7.93E-04	Down	-1.15E+01	8.89E-06	Down	Unigene106894_All//gi|298709974|emb|CBJ31696.1|//2.00E-27//Ribosomal protein S23, component of cytosolic 80S ribosome and 40S small subunit [Ectocarpus siliculosus]	Unigene106894_All//4.00E-23//AT5G02960| 40S ribosomal protein S23 (RPS23B)	Unigene106894_All//2.00E-20//CD486290		ko03010|Ribosome		nr -
Unigene106896_All	205	0.00	0.00	0.00	0.00	0.00	0.00	3.51	0.20	0.00	null	null		null	null		null	null		null	null		-4.10E+00	2.92E-04	Down	-1.18E+01	1.78E-05	Down	Unigene106896_All//gi|116059767|emb|CAL55474.1|//6.00E-22//ribosomal protein L18 (ISS) [Ostreococcus tauri]	Unigene106896_All//1.00E-19//AT3G05590| RPL18 (RIBOSOMAL PROTEIN L18); structural constituent of ribosome			ko03010|Ribosome		nr -
Unigene106898_All	399	0.00	0.00	0.00	0.00	0.00	0.00	5.83	0.21	0.10	null	null		null	null		null	null		null	null		-4.80E+00	7.37E-14	Down	-5.87E+00	1.13E-15	Down	Unigene106898_All//gi|32400871|gb|AAP80667.1|AF479048_1//5.00E-55//ribosomal Pr 117 [Triticum aestivum]	Unigene106898_All//4.00E-56//AT3G04400| emb2171 (embryo defective 2171); structural constituent of ribosome	Unigene106898_All//5.00E-13//TC156592		ko03010|Ribosome		nr -
Unigene10690_All	593	0.00	4.53	1.62	0.00	0.00	0.00	0.00	0.00	0.00	1.21E+01	2.81E-15	Up	1.07E+01	2.46E-05	Up	null	null		null	null		null	null		null	null								ESTscan +
Unigene106901_All	269	0.00	0.00	0.00	0.00	0.00	0.00	2.67	0.47	0.00	null	null		null	null		null	null		null	null		-2.52E+00	4.69E-03		-1.14E+01	1.78E-05	Down							ESTscan +
Unigene106902_All	848	0.00	0.00	0.00	0.00	0.24	0.05	11.77	0.59	0.52	null	null		null	null		7.93E+00	1.57E-01		5.74E+00	7.03E-01		-4.31E+00	1.38E-54	Down	-4.51E+00	4.25E-58	Down	Unigene106902_All//gi|117622276|gb|ABK51384.1|//1.00E-08//IMP dehydrogenase/GMP reductase [Hevea brasiliensis]						nr -
Unigene106903_All	577	0.00	0.00	0.00	0.00	0.00	0.00	3.30	0.14	0.00	null	null		null	null		null	null		null	null		-4.51E+00	4.75E-11	Down	-1.17E+01	4.91E-14	Down	Unigene106903_All//gi|83582438|emb|CAJ45370.1|//3.00E-65//40S ribosomal protein S5 [Chlamydomonas sp. ICE-L]	Unigene106903_All//3.00E-64//AT2G37270| ATRPS5B (RIBOSOMAL PROTEIN 5B); structural constituent of ribosome			ko03010|Ribosome		nr +
Unigene106904_All	531	0.00	0.00	0.00	0.00	0.00	0.00	3.34	0.08	0.08	null	null		null	null		null	null		null	null		-5.41E+00	2.97E-11	Down	-5.48E+00	8.92E-12	Down							ESTscan -
Unigene106907_All	383	1.09	2.12	2.64	2.99	2.97	2.00	2.76	6.88	2.08	9.54E-01	2.26E-01		1.27E+00	8.36E-02		-1.19E-02	9.64E-01		-5.78E-01	3.98E-01		1.32E+00	2.33E-04	Up	-4.08E-01	4.85E-01				Unigene106907_All//2.00E-101//TC162510				
Unigene106908_All	505	0.00	0.00	0.00	0.00	0.00	0.00	2.68	1.57	0.39	null	null		null	null		null	null		null	null		-7.69E-01	1.39E-01		-2.76E+00	7.51E-06	Down	Unigene106908_All//gi|34761728|gb|AAQ82037.1|//3.00E-15//gag/pol polyprotein [Pisum sativum]		Unigene106908_All//3.00E-36//FL576545				nr +
Unigene106909_All	693	0.00	0.00	0.00	0.00	0.00	0.00	2.38	0.24	0.00	null	null		null	null		null	null		null	null		-3.30E+00	8.31E-08	Down	-1.12E+01	3.45E-12	Down							ESTscan -
Unigene106911_All	403	0.00	0.25	0.00	0.00	0.26	0.34	55.81	46.15	417.66	7.97E+00	3.90E-01		null	null		8.00E+00	4.58E-01		8.39E+00	3.12E-01		-2.74E-01	9.77E-03		2.90E+00	0.00E+00	Up	Unigene106911_All//gi|3970689|emb|CAA77191.1|//1.00E-36//(+)-delta-cadinene synthase [Gossypium arboreum]	Unigene106911_All//7.00E-13//AT5G23960| TPS21 (TERPENE SYNTHASE 21); (-)-E-beta-caryophyllene synthase/ alpha-humulene synthase	Unigene106911_All//2.00E-74//gi|84158582|gb|DN827629.1|DN827629		ko00904|Diterpenoid biosynthesis//ko00902|Monoterpenoid biosynthesis		nr -
Unigene106913_All	772	0.00	0.00	0.00	0.00	0.00	0.00	7.23	0.43	0.36	null	null		null	null		null	null		null	null		-4.06E+00	1.68E-29	Down	-4.32E+00	7.14E-32	Down	Unigene106913_All//gi|192910762|gb|ACF06489.1|//5.00E-68//ribosomal protein L10a [Elaeis guineensis]	Unigene106913_All//1.00E-61//AT1G08360| 60S ribosomal protein L10A (RPL10aA)	Unigene106913_All//6.00E-11//CD486329		ko03010|Ribosome		nr +

Unigene106914_All	597	0.00	0.00	0.00	0.00	0.00	0.00	5.03	0.00	0.40	null	null		null	null		null	null		null	null		-1.23E+01	3.14E-21	Down	-3.65E+00	1.83E-15	Down	Unigene106914_All//gi|298704815|emb|CBJ48963.1|//8.00E-74//Arf1, ARF family GTPase [Ectocarpus siliculosus]	Unigene106914_All//4.00E-72//AT3G62290| ATARFA1E (ADP-ribosylation factor A1E); GTP binding / phospholipase activator/ protein binding	Unigene106914_All//2.00E-41//gi|78322862|gb|DT543136.1|DT543136				nr -
Unigene106925_All	237	0.00	0.00	0.00	0.00	0.00	0.00	2.14	0.00	0.00	null	null		null	null		null	null		null	null		-1.11E+01	8.91E-04	Down	-1.11E+01	5.56E-04	Down							
Unigene106929_All	367	0.00	0.00	0.00	0.00	0.00	0.00	2.76	1.03	0.22	null	null		null	null		null	null		null	null		-1.43E+00	2.36E-02		-3.67E+00	1.15E-05	Down							
Unigene106932_All	383	0.00	0.26	0.00	0.00	0.00	0.00	1.77	0.11	0.21	8.05E+00	3.90E-01		null	null		null	null		null	null		-4.02E+00	5.36E-04	Down	-3.09E+00	1.45E-03								
Unigene106935_All	578	0.00	0.00	0.00	0.00	0.00	0.00	1.98	1.01	0.07	null	null		null	null		null	null		null	null		-9.64E-01	9.33E-02		-4.84E+00	2.34E-07	Down							
Unigene106940_All	361	0.58	0.00	0.00	0.00	0.00	0.00	7.26	1.62	7.06	-9.18E+00	1.23E-01		-9.18E+00	1.47E-01		null	null		null	null		-2.16E+00	6.42E-08	Down	-4.00E-02	9.25E-01								
Unigene106943_All	433	0.00	0.35	0.37	1.15	0.16	0.21	2.05	0.48	0.18	8.46E+00	2.37E-01		8.53E+00	2.40E-01		-2.85E+00	1.78E-02		-2.46E+00	4.83E-02		-2.09E+00	4.71E-03		-3.48E+00	7.34E-05	Down							
Unigene10695_All	265	0.00	6.50	3.22	0.00	0.00	0.00	0.00	0.00	0.00	1.27E+01	8.00E-10	Up	1.17E+01	9.03E-05	Up	null	null		null	null		null	null		null	null								ESTscan +
Unigene106956_All	400	0.00	0.00	0.00	0.00	0.00	0.00	2.11	0.84	0.10	null	null		null	null		null	null		null	null		-1.34E+00	5.68E-02		-4.41E+00	2.37E-05	Down							ESTscan +
Unigene106957_All	596	0.00	0.00	0.00	0.00	0.00	0.00	2.06	2.17	0.00	null	null		null	null		null	null		null	null		7.95E-02	8.90E-01		-1.10E+01	4.03E-09	Down	Unigene106957_All//gi|300266545|gb|EFJ50732.1|//9.00E-54//component of cytosolic 80S ribosome and 60S large subunit [Volvox carteri f. nagariensis]	Unigene106957_All//3.00E-42//AT3G05590| RPL18 (RIBOSOMAL PROTEIN L18); structural constituent of ribosome			ko03010|Ribosome		nr +
Unigene106959_All	1475	0.00	0.00	0.00	0.00	0.00	0.00	6.36	5.41	0.35	null	null		null	null		null	null		null	null		-2.34E-01	2.03E-01		-4.18E+00	4.76E-52	Down	Unigene106959_All//gi|226347409|gb|ACO50115.1|//0.00E+00//elongation factor 1 alpha [Peranema trichophorum]	Unigene106959_All//1.00E-130//AT5G60390| elongation factor 1-alpha / EF-1-alpha	Unigene106959_All//1.00E-72//TC166357				nr -
Unigene10696_All	451	0.00	0.00	0.00	0.00	0.00	0.00	2.81	2.22	0.35	null	null		null	null		null	null		null	null		-3.39E-01	5.58E-01		-2.99E+00	6.36E-06	Down	Unigene10696_All//gi|167859793|gb|ACA04850.1|//7.00E-14//senescence-associated protein [Picea abies]		Unigene10696_All//4.00E-17//gi|84158108|gb|DN827155.1|DN827155				nr +
Unigene106972_All	270	0.00	0.00	0.00	5.90	2.17	1.51	30.85	2.48	23.76	null	null		null	null		-1.45E+00	4.07E-03		-1.97E+00	4.88E-04	Down	-3.64E+00	2.76E-40	Down	-3.77E-01	3.26E-02				Unigene106972_All//3.00E-47//ES827343				
Unigene106979_All	236	0.89	0.86	1.35	2.53	2.19	2.87	12.36	145.40	21.44	-4.63E-02	9.89E-01		6.10E-01	6.25E-01		-2.11E-01	8.23E-01		1.81E-01	8.45E-01		3.56E+00	9.98E-161	Up	7.94E-01	5.98E-04		Unigene106979_All//gi|23978432|dbj|BAC21263.1|//7.00E-28//glutathione S-transferase [Cucurbita maxima]	Unigene106979_All//1.00E-20//AT2G29450| ATGSTU5 (ARABIDOPSIS THALIANA GLUTATHIONE S-TRANSFERASE TAU 5); glutathione binding / glutathione transferase	Unigene106979_All//2.00E-69//gi|62295949|gb|DN779339.1|DN779339		ko00480|Glutathione metabolism	GO:0016765//GO:0016846//GO:0044424	nr +
Unigene106981_All	679	0.00	0.00	0.00	0.00	0.00	0.00	3.30	0.43	0.18	null	null		null	null		null	null		null	null		-2.94E+00	2.19E-09	Down	-4.23E+00	6.02E-13	Down	Unigene106981_All//gi|226500076|ref|NP_001150681.1|//3.00E-17//LOC100284314 [Zea mays] >gi|195641032|gb|ACG39984.1| zinc finger protein hangover [Zea mays]				ko04120|Ubiquitin mediated proteolysis		nr -
Unigene106982_All	799	0.00	0.00	0.00	0.00	0.13	0.00	0.32	0.68	1.65	null	null		null	null		7.01E+00	4.58E-01		null	null		1.10E+00	2.36E-01		2.37E+00	7.20E-05	Up	Unigene106982_All//gi|224074393|ref|XP_002304363.1|//5.00E-67//acyl:coa ligase [Populus trichocarpa] >gi|222841795|gb|EEE79342.1| acyl:coa ligase [Populus trichocarpa]	Unigene106982_All//1.00E-39//AT4G19010| 4-coumarate--CoA ligase family protein / 4-coumaroyl-CoA synthase family protein	Unigene106982_All//1.00E-163//gi|13352404|gb|BG442752.1|BG442752		ko00940|Phenylpropanoid biosynthesis//ko00360|Phenylalanine metabolism//ko00592|alpha-Linolenic acid metabolism//ko00130|Ubiquinone and other terpenoid-quinone biosynthesis	GO:0016405	nr -
Unigene106986_All	288	0.00	0.00	0.00	0.00	0.00	0.00	3.38	0.00	0.00	null	null		null	null		null	null		null	null		-1.17E+01	5.84E-07	Down	-1.17E+01	2.72E-07	Down							
Unigene106989_All	288	0.00	0.00	0.00	0.00	0.00	0.00	3.08	0.44	0.00	null	null		null	null		null	null		null	null		-2.82E+00	5.65E-04	Down	-1.16E+01	1.10E-06	Down	Unigene106989_All//gi|73761683|gb|AAZ83341.1|//3.00E-33//ubiquitin extension protein [Gossypium hirsutum]	Unigene106989_All//6.00E-19//AT3G62250| UBQ5 (ubiquitin 5); protein binding / structural constituent of ribosome	Unigene106989_All//1.00E-09//gi|164316986|gb|EV492026.1|EV492026		ko03010|Ribosome	GO:0005198//GO:0005488//GO:0010467//GO:0015935//GO:0016567//GO:0019941	nr +
Unigene10699_All	274	0.19	9.06	0.78	0.18	0.88	0.49	0.31	0.15	1.02	5.57E+00	9.50E-13	Up	2.03E+00	3.62E-01		2.27E+00	3.05E-01		1.44E+00	6.39E-01		-1.02E+00	7.26E-01		1.72E+00	2.30E-01				Unigene10699_All//3.00E-29//CO111688				
Unigene1070_All	1487	10.56	4.97	8.49	12.23	13.94	15.22	6.20	6.97	14.57	-1.09E+00	1.41E-13	Down	-3.15E-01	3.77E-02		1.89E-01	1.51E-01		3.16E-01	6.81E-03		1.69E-01	3.57E-01		1.23E+00	5.20E-28	Up	Unigene1070_All//gi|90399346|emb|CAH68425.1|//2.00E-93//H0811D08.10 [Oryza sativa (indica cultivar-group)]	Unigene1070_All//1.00E-61//AT5G10790| UBP22 (UBIQUITIN-SPECIFIC PROTEASE 22); ubiquitin thiolesterase/ ubiquitin-specific protease/ zinc ion binding	Unigene1070_All//0.00E+00//gi|78338363|gb|DT558637.1|DT558637			GO:0008233//GO:0043231	nr -
Unigene10701_All	477	1.97	0.21	0.89	4.70	3.75	4.73	0.18	0.35	1.00	-3.22E+00	6.77E-04	Down	-1.14E+00	1.38E-01		-3.24E-01	4.77E-01		1.06E-02	9.82E-01		9.83E-01	6.35E-01		2.50E+00	2.52E-02		Unigene10701_All//gi|21322711|emb|CAD22154.1|//2.00E-40//pherophorin-dz1 protein [Volvox carteri f. nagariensis]				ko03040|Spliceosome		nr -
Unigene107016_All	238	0.00	0.00	0.00	0.00	0.00	0.00	2.13	0.00	0.33	null	null		null	null		null	null		null	null		-1.11E+01	8.91E-04	Down	-2.67E+00	1.37E-02								
Unigene107032_All	390	0.00	0.00	0.00	0.00	0.00	0.00	1.73	1.18	0.00	null	null		null	null		null	null		null	null		-5.57E-01	4.89E-01		-1.08E+01	3.57E-05	Down	Unigene107032_All//gi|226462262|gb|EEH59554.1|//2.00E-32//60S ribosomal protein L35 [Micromonas pusilla CCMP1545]	Unigene107032_All//3.00E-32//AT5G02610| 60S ribosomal protein L35 (RPL35D)			ko03010|Ribosome	GO:0005198//GO:0010467//GO:0030529	nr -
Unigene10704_All	297	0.00	0.68	0.00	0.00	0.00	0.15	0.00	1.97	2.68	9.41E+00	1.37E-01		null	null		null	null		7.25E+00	7.03E-01		1.09E+01	2.81E-04	Up	1.14E+01	7.36E-06	Up							
Unigene107043_All	300	0.00	0.00	0.00	0.00	0.11	0.00	2.68	0.14	0.00	null	null		null	null		6.84E+00	8.39E-01		null	null		-4.26E+00	8.48E-05	Down	-1.14E+01	4.43E-06	Down	Unigene107043_All//gi|44917549|gb|AAS49099.1|//2.00E-13//At2g37380 [Arabidopsis thaliana]	Unigene107043_All//2.00E-16//AT2G39370| unknown protein	Unigene107043_All//4.00E-10//TC160952				nr +
Unigene107048_All	552	0.00	0.00	0.00	0.00	0.00	0.00	2.53	0.08	0.14	null	null		null	null		null	null		null	null		-5.06E+00	1.10E-08	Down	-4.13E+00	3.70E-08	Down							
Unigene107050_All	326	0.48	0.78	0.00	0.46	0.63	0.83	2.46	0.00	1.10	6.91E-01	6.27E-01		-8.91E+00	2.46E-01		4.67E-01	8.26E-01		8.59E-01	6.31E-01		-1.13E+01	8.58E-06	Down	-1.16E+00	8.85E-02		Unigene107050_All//gi|297834848|ref|XP_002885306.1|//2.00E-48//kinase family protein [Arabidopsis lyrata subsp. lyrata] >gi|297331146|gb|EFH61565.1| kinase family protein [Arabidopsis lyrata subsp. lyrata]	Unigene107050_All//3.00E-40//AT3G19300| protein kinase family protein	Unigene107050_All//1.00E-179//ES800996		ko04626|Plant-pathogen interaction	GO:0004672//GO:0006464//GO:0016020//GO:0031410//GO:0032559	nr +
Unigene107061_All	257	0.00	0.00	0.00	0.00	0.00	0.00	2.14	0.00	0.31	null	null		null	null		null	null		null	null		-1.11E+01	4.62E-04	Down	-2.79E+00	7.97E-03		Unigene107061_All//gi|145345768|ref|XP_001417372.1|//2.00E-20//Ribosomal protein S17, component of cytosolic 80S ribosome and 40S small subunit [Ostreococcus lucimarinus CCE9901] >gi|144577599|gb|ABO95665.1| Ribosomal protein S17, component of cytosolic 80S ribosome and 40S small subunit [Ostreococcus lucimarinus CCE9901]	Unigene107061_All//1.00E-20//AT5G04800| 40S ribosomal protein S17 (RPS17D)			ko03010|Ribosome	GO:0005198//GO:0010467//GO:0030529	nr +
Unigene107064_All	497	0.00	0.00	0.00	0.00	0.00	0.00	2.64	0.00	0.08	null	null		null	null		null	null		null	null		-1.14E+01	2.56E-09	Down	-5.04E+00	1.60E-08	Down	Unigene107064_All//gi|209967441|gb|ACJ02349.1|//2.00E-21//60S ribosomal protein L35a [Vernicia fordii]	Unigene107064_All//2.00E-20//AT1G41880| 60S ribosomal protein L35a (RPL35aB)			ko03010|Ribosome	GO:0005198//GO:0010467//GO:0015934//GO:0022613	nr +
Unigene107068_All	207	0.00	0.00	0.00	0.00	0.00	0.00	3.06	0.20	0.00	null	null		null	null		null	null		null	null		-3.92E+00	9.81E-04	Down	-1.16E+01	7.13E-05	Down	Unigene107068_All//gi|126566038|gb|ABO20848.1|//1.00E-07//cytochrome P450-like TBP protein [Lilium longiflorum]		Unigene107068_All//9.00E-07//EV495741				nr -
Unigene10707_All	278	0.38	0.00	1.53	2.87	0.12	0.16	0.30	0.00	0.00	-8.56E+00	3.76E-01		2.03E+00	1.40E-01		-4.53E+00	6.06E-05	Down	-4.14E+00	4.13E-04	Down	-8.25E+00	4.07E-01		-8.25E+00	3.61E-01								
Unigene107071_All	284	104.64	101.70	87.56	4.03	6.79	10.02	2.38	0.74	1.26	-4.12E-02	7.09E-01		-2.57E-01	1.57E-02		7.51E-01	1.14E-01		1.31E+00	5.61E-04	Up	-1.69E+00	4.19E-02		-9.16E-01	2.21E-01		Unigene107071_All//gi|55168340|gb|AAV44205.1|//7.00E-10//unknow protein [Oryza sativa Japonica Group]		Unigene107071_All//1.00E-80//TC147077				nr -
Unigene107076_All	499	0.00	0.00	0.00	0.00	0.00	0.00	1.27	1.93	0.00	null	null		null	null		null	null		null	null		6.00E-01	3.67E-01		-1.03E+01	7.12E-05	Down	Unigene107076_All//gi|212275977|ref|NP_001130697.1|//3.00E-16//hypothetical protein LOC100191800 [Zea mays] >gi|194689862|gb|ACF79015.1| unknown [Zea mays]						nr -
Unigene10708_All	358	17.10	8.35	11.90	22.40	22.99	25.86	5.31	2.80	5.68	-1.03E+00	2.07E-05	Down	-5.23E-01	3.79E-02		3.73E-02	8.80E-01		2.07E-01	3.43E-01		-9.24E-01	2.84E-02		9.48E-02	8.12E-01				Unigene10708_All//7.00E-15//gi|109890883|gb|DW519846.1|DW519846				
Unigene107081_All	352	0.00	0.00	0.00	0.00	0.00	0.00	2.76	0.59	0.00	null	null		null	null		null	null		null	null		-2.22E+00	1.82E-03		-1.14E+01	2.72E-07	Down							
Unigene107084_All	1125	0.00	0.00	0.00	0.00	0.00	0.00	4.59	0.04	0.11	null	null		null	null		null	null		null	null		-6.95E+00	9.46E-35	Down	-5.43E+00	2.57E-33	Down	Unigene107084_All//gi|115463079|ref|NP_001055139.1|//2.00E-99//Os05g0302700 [Oryza sativa Japonica Group] >gi|113578690|dbj|BAF17053.1| Os05g0302700 [Oryza sativa Japonica Group]	Unigene107084_All//2.00E-80//AT5G13490| AAC2 (ADP/ATP carrier 2); ATP:ADP antiporter/ binding	Unigene107084_All//4.00E-13//TC167393			GO:0006810//GO:0019866//GO:0031224//GO:0043231	nr -
Unigene107088_All	462	0.00	0.00	0.00	0.00	0.00	0.00	2.10	0.54	0.00	null	null		null	null		null	null		null	null		-1.96E+00	4.44E-03		-1.10E+01	2.72E-07	Down							ESTscan -
Unigene107104_All	363	0.00	0.00	0.00	0.00	0.00	0.00	1.98	0.12	0.00	null	null		null	null		null	null		null	null		-4.10E+00	2.91E-04	Down	-1.10E+01	1.78E-05	Down	Unigene107104_All//gi|297814842|ref|XP_002875304.1|//2.00E-09//leucine-rich repeat family protein [Arabidopsis lyrata subsp. lyrata] >gi|297321142|gb|EFH51563.1| leucine-rich repeat family protein [Arabidopsis lyrata subsp. lyrata]	Unigene107104_All//2.00E-09//AT5G66330| leucine-rich repeat family protein	Unigene107104_All//8.00E-55//gi|78335416|gb|DT555690.1|DT555690				nr -
Unigene107106_All	237	0.00	0.00	0.00	0.84	0.00	0.00	2.14	0.71	0.00	null	null		null	null		-9.72E+00	8.48E-02		-9.72E+00	1.34E-01		-1.60E+00	1.07E-01		-1.11E+01	5.56E-04	Down	Unigene107106_All//gi|124360490|gb|ABN08500.1|//2.00E-13//Protein kinase [Medicago truncatula]	Unigene107106_All//3.00E-09//AT3G47570| leucine-rich repeat transmembrane protein kinase, putative			ko04626|Plant-pathogen interaction	GO:0004672//GO:0005488	nr -
Unigene107107_All	458	0.00	0.00	0.00	0.00	0.08	0.10	5.17	0.37	0.35	null	null		null	null		6.23E+00	8.39E-01		6.62E+00	7.03E-01		-3.82E+00	2.79E-12	Down	-3.89E+00	6.40E-13	Down			Unigene107107_All//2.00E-25//gi|195983575|gb|FL577381.1|FL577381				
Unigene10711_All	310	3.54	0.00	2.23	0.48	0.33	0.58	0.55	0.13	0.51	-1.18E+01	1.95E-06	Down	-6.67E-01	3.35E-01		-5.33E-01	7.86E-01		2.74E-01	9.06E-01		-2.02E+00	3.68E-01		-8.61E-02	9.66E-01								
Unigene107111_All	553	0.00	0.00	0.00	0.09	0.00	0.00	2.14	0.91	0.36	null	null		null	null		-6.49E+00	5.64E-01		-6.49E+00	6.45E-01		-1.24E+00	2.93E-02		-2.57E+00	6.98E-05	Down			Unigene107111_All//3.00E-21//TC132835				
Unigene107113_All	280	0.00	0.00	0.00	0.00	0.00	0.00	2.42	0.15	0.00	null	null		null	null		null	null		null	null		-4.02E+00	5.36E-04	Down	-1.12E+01	3.57E-05	Down	Unigene107113_All//gi|116222125|gb|ABJ80933.1|//4.00E-38//actin [Leucocryptos marina]	Unigene107113_All//3.00E-33//AT3G12110| ACT11 (actin-11); structural constituent of cytoskeleton	Unigene107113_All//1.00E-15//gi|5049214|gb|AI730362.1|AI730362			GO:0032559//GO:0043232	nr +
Unigene107114_All	272	0.00	0.00	0.00	0.00	0.00	0.00	7.46	0.00	0.88	null	null		null	null		null	null		null	null		-1.29E+01	2.32E-14	Down	-3.09E+00	2.66E-09	Down	Unigene107114_All//gi|37901017|gb|AAP46148.1|//2.00E-45//actin 2 [Pseudo-nitzschia multiseries] >gi|37935564|gb|AAP46147.1| actin 1 [Pseudo-nitzschia multiseries]	Unigene107114_All//2.00E-43//AT3G12110| ACT11 (actin-11); structural constituent of cytoskeleton	Unigene107114_All//9.00E-20//gi|31406833|gb|CD485868.1|CD485868		ko04145|Phagosome	GO:0000267//GO:0003015//GO:0005198//GO:0005884//GO:0006939//GO:0007015//GO:0008015//GO:0008092//GO:0012501//GO:0017111//GO:0030017//GO:0030239//GO:0032432//GO:0032559//GO:0048747//GO:0051707//GO:0060415	nr -
Unigene10712_All	361	0.00	4.77	1.48	0.00	0.00	0.00	0.00	0.00	0.00	1.22E+01	8.00E-10	Up	1.05E+01	4.05E-03		null	null		null	null		null	null		null	null		Unigene10712_All//gi|226530890|ref|NP_001151865.1|//1.00E-44//serine hydroxymethyltransferase [Zea mays] >gi|195650403|gb|ACG44669.1| serine hydroxymethyltransferase [Zea mays]	Unigene10712_All//2.00E-41//AT5G26780| SHM2 (SERINE HYDROXYMETHYLTRANSFERASE 2); catalytic/ glycine hydroxymethyltransferase/ pyridoxal phosphate binding			ko00460|Cyanoamino acid metabolism//ko00260|Glycine, serine and threonine metabolism//ko00670|One carbon pool by folate	GO:0009069//GO:0016742//GO:0043231//GO:0048037	nr -

Unigene107123_All	241	0.00	0.00	0.00	0.00	0.14	0.00	4.04	3.47	11.57	null	null		null	null		7.16E+00	8.39E-01		null	null		-2.18E-01	7.24E-01		1.52E+00	1.22E-05	Up	Unigene107123_All//gi|15217709|ref|NP_176647.1|//9.00E-30//ATMGL (ARABIDOPSIS THALIANA METHIONINE GAMMA-LYASE); catalytic/ methionine gamma-lyase [Arabidopsis thaliana] >gi|6633821|gb|AAF19680.1|AC009519_14 F1N19.23 [Arabidopsis thaliana] >gi|16226491|gb|AAL16181.1|AF428413_1 At1g64660/F1N19_22 [Arabidopsis thaliana] >gi|15450932|gb|AAK96737.1| similar to O-succinylhomoserine sulfhydrylase [Arabidopsis thaliana] >gi|30387533|gb|AAP31932.1| At1g64660 [Arabidopsis thaliana] >gi|110740851|dbj|BAE98522.1| similar to O-succinylhomoserine sulfhydrylase [Arabidopsis thaliana]	Unigene107123_All//4.00E-31//AT1G64660| ATMGL (ARABIDOPSIS THALIANA METHIONINE GAMMA-LYASE); catalytic/ methionine gamma-lyase	Unigene107123_All//1.00E-49//TC130861		ko00270|Cysteine and methionine metabolism//ko00450|Selenoamino acid metabolism	GO:0009087//GO:0016765//GO:0016846//GO:0044444//GO:0048037	nr -
Unigene107128_All	512	0.61	0.00	0.31	4.38	2.42	2.82	6.94	1.71	3.73	-9.26E+00	3.67E-02		-9.75E-01	5.06E-01		-8.55E-01	3.58E-02		-6.33E-01	1.48E-01		-2.02E+00	1.20E-09	Down	-8.93E-01	1.57E-03		Unigene107128_All//gi|302142868|emb|CBI20163.3|//5.00E-09//unnamed protein product [Vitis vinifera]		Unigene107128_All//2.00E-161//CO094931				nr +
Unigene10713_All	299	0.35	1.19	0.18	0.50	1.04	1.81	3.25	7.27	9.46	1.76E+00	2.18E-01		-9.75E-01	7.22E-01		1.05E+00	5.32E-01		1.86E+00	1.03E-01		1.16E+00	3.26E-03		1.54E+00	8.34E-06	Up	Unigene10713_All//gi|114199050|gb|ABI54120.1|//4.00E-24//caffeic acid O-methyltransferase [Malus x domestica]	Unigene10713_All//6.00E-24//AT5G54160| ATOMT1 (O-METHYLTRANSFERASE 1); caffeate O-methyltransferase/ myricetin 3'-O-methyltransferase/ quercetin 3-O-methyltransferase	Unigene10713_All//8.00E-36//AI055471		ko00940|Phenylpropanoid biosynthesis//ko00944|Flavone and flavonol biosynthesis	GO:0005515//GO:0008171//GO:0009699	nr +
Unigene107136_All	761	0.00	0.00	0.00	0.00	0.00	0.00	2.00	2.14	0.10	null	null		null	null		null	null		null	null		9.89E-02	8.36E-01		-4.26E+00	5.22E-09	Down	Unigene107136_All//gi|7208784|emb|CAB76914.1|//2.00E-26//60S ribosomal protein L6 [Cicer arietinum]	Unigene107136_All//9.00E-15//AT1G74060| 60S ribosomal protein L6 (RPL6B)			ko03010|Ribosome	GO:0005488//GO:0015934//GO:0016020//GO:0031981	nr +
Unigene107138_All	235	0.45	0.65	1.13	0.64	0.88	0.19	2.88	0.18	0.00	5.39E-01	7.80E-01		1.35E+00	4.09E-01		4.67E-01	8.26E-01		-1.73E+00	5.02E-01		-4.02E+00	5.36E-04	Down	-1.15E+01	3.57E-05	Down	Unigene107138_All//gi|105922786|gb|ABF81437.1|//7.00E-08//TIR-NBS-LRR-TIR type disease resistance protein [Populus trichocarpa]	Unigene107138_All//4.00E-05//AT1G27180| disease resistance protein (TIR-NBS-LRR class), putative	Unigene107138_All//9.00E-29//gi|48807031|gb|CO108345.1|CO108345			GO:0000166//GO:0006952//GO:0009987	nr -
Unigene107140_All	387	2.16	0.65	0.00	0.77	0.53	1.52	2.84	1.08	1.24	-1.72E+00	3.63E-02		-1.11E+01	1.19E-04	Down	-5.33E-01	6.79E-01		9.74E-01	3.46E-01		-1.40E+00	2.04E-02		-1.20E+00	3.41E-02				Unigene107140_All//3.00E-76//BG440255				
Unigene107141_All	255	0.00	0.00	0.00	0.00	0.00	0.00	3.15	0.00	0.00	null	null		null	null		null	null		null	null		-1.16E+01	8.58E-06	Down	-1.16E+01	4.43E-06	Down							
Unigene107156_All	507	0.21	0.10	0.63	0.20	0.27	0.36	2.34	1.48	0.16	-1.05E+00	6.86E-01		1.61E+00	3.05E-01		4.68E-01	8.78E-01		8.59E-01	6.96E-01		-6.54E-01	2.47E-01		-3.89E+00	9.21E-07	Down							
Unigene107158_All	493	0.00	0.00	0.00	0.00	0.00	0.00	4.97	0.59	1.62	null	null		null	null		null	null		null	null		-3.07E+00	1.26E-10	Down	-1.62E+00	1.55E-05	Down							
Unigene107159_All	341	0.00	0.00	0.00	0.00	0.00	0.00	2.36	1.96	0.00	null	null		null	null		null	null		null	null		-2.65E-01	7.05E-01		-1.12E+01	4.43E-06	Down			Unigene107159_All//4.00E-10//gi|13246915|gb|BE052560.2|BE052560				kegg -
Unigene107160_All	310	0.34	0.00	0.00	0.00	0.00	0.00	1.91	0.00	0.00	-8.40E+00	3.76E-01		-8.40E+00	3.99E-01		null	null		null	null		-1.09E+01	2.40E-04	Down	-1.09E+01	1.42E-04	Down							
Unigene107166_All	1000	0.16	0.15	0.11	0.60	0.55	0.77	1.06	0.17	0.20	-4.67E-02	9.90E-01		-5.60E-01	7.76E-01		-1.18E-01	8.76E-01		3.61E-01	6.80E-01		-2.66E+00	2.25E-04	Down	-2.41E+00	3.46E-04	Down	Unigene107166_All//gi|297820436|ref|XP_002878101.1|//1.00E-39//CIP111 [Arabidopsis lyrata subsp. lyrata] >gi|297323939|gb|EFH54360.1| CIP111 [Arabidopsis lyrata subsp. lyrata]	Unigene107166_All//1.00E-32//AT3G56690| CIP111 (CAM INTERACTING PROTEIN 111); ATPase/ calmodulin binding	Unigene107166_All//0.00E+00//TC173047		ko04141|Protein processing in endoplasmic reticulum	GO:0005515//GO:0009534//GO:0017111	nr -
Unigene107168_All	564	0.00	0.09	0.00	0.09	0.12	0.32	1.72	0.52	0.14	6.49E+00	6.22E-01		null	null		4.68E-01	9.61E-01		1.86E+00	4.59E-01		-1.73E+00	9.67E-03		-3.61E+00	2.14E-05	Down							
Unigene10717_All	274	102.73	130.93	103.58	147.81	170.42	157.25	59.41	61.93	134.93	3.50E-01	9.67E-05		1.19E-02	9.33E-01		2.05E-01	8.00E-03		8.93E-02	3.76E-01		6.00E-02	6.72E-01		1.18E+00	3.07E-44	Up	Unigene10717_All//gi|227204481|dbj|BAH57092.1|//6.00E-17//AT4G10970 [Arabidopsis thaliana]	Unigene10717_All//3.00E-14//AT4G10970| unknown protein	Unigene10717_All//2.00E-153//TC154089				nr +
Unigene107173_All	821	0.00	0.00	0.00	0.00	0.00	0.00	4.02	0.00	0.19	null	null		null	null		null	null		null	null		-1.20E+01	2.53E-23	Down	-4.37E+00	3.47E-19	Down							ESTscan -
Unigene107176_All	448	0.00	0.00	0.00	0.00	0.00	0.00	2.64	0.37	0.27	null	null		null	null		null	null		null	null		-2.82E+00	4.41E-05	Down	-3.31E+00	4.89E-06	Down	Unigene107176_All//gi|224004980|ref|XP_002296141.1|//7.00E-32//RL3, ribosomal protein 3, 60S large ribosomal subunit [Thalassiosira pseudonana CCMP1335] >gi|209586173|gb|ACI64858.1| RL3, ribosomal protein 3, 60S large ribosomal subunit [Thalassiosira pseudonana CCMP1335]	Unigene107176_All//1.00E-31//AT1G43170| ARP1 (ARABIDOPSIS RIBOSOMAL PROTEIN 1); structural constituent of ribosome			ko03010|Ribosome	GO:0030529	nr -
Unigene107179_All	1445	0.00	0.00	0.00	0.07	0.05	0.00	2.84	1.36	0.58	null	null		null	null		-5.31E-01	8.19E-01		-6.11E+00	4.06E-01		-1.06E+00	9.14E-05	Down	-2.29E+00	2.79E-13	Down	Unigene107179_All//gi|283132365|dbj|BAI63588.1|//1.00E-128//leucine rich repeat protein [Lotus japonicus]	Unigene107179_All//2.00E-108//AT2G19330| leucine-rich repeat family protein			ko04626|Plant-pathogen interaction		nr -
Unigene107192_All	1107	0.00	0.00	0.00	0.00	0.06	0.04	0.23	0.15	1.01	null	null		null	null		5.96E+00	6.15E-01		5.35E+00	7.03E-01		-6.02E-01	6.48E-01		2.14E+00	7.62E-04	Up	Unigene107192_All//gi|297802268|ref|XP_002869018.1|//2.00E-61//GNS1/SUR4 membrane family protein [Arabidopsis lyrata subsp. lyrata] >gi|297314854|gb|EFH45277.1| GNS1/SUR4 membrane family protein [Arabidopsis lyrata subsp. lyrata]	Unigene107192_All//2.00E-79//AT4G36830| GNS1/SUR4 membrane family protein	Unigene107192_All//3.00E-10//TC171527				nr +
Unigene107197_All	953	0.05	0.00	0.00	0.00	0.00	0.00	11.80	1.62	0.92	-5.78E+00	6.20E-01		-5.78E+00	6.33E-01		null	null		null	null		-2.86E+00	2.01E-43	Down	-3.68E+00	1.35E-56	Down	Unigene107197_All//gi|34761723|gb|AAQ82034.1|//3.00E-10//unknown [Pisum sativum]		Unigene107197_All//6.00E-70//TC161176				nr -
Unigene107201_All	391	0.00	0.00	0.00	0.00	0.00	0.00	4.87	0.21	0.00	null	null		null	null		null	null		null	null		-4.51E+00	4.75E-11	Down	-1.22E+01	4.91E-14	Down							ESTscan +
Unigene107202_All	444	0.00	0.00	0.00	0.00	0.00	0.00	1.14	0.09	0.00	null	null		null	null		null	null		null	null		-3.60E+00	5.78E-03		-1.02E+01	5.56E-04	Down	Unigene107202_All//gi|226347429|gb|ACO50125.1|//4.00E-60//elongation factor 1 alpha [Entosiphon sulcatum]	Unigene107202_All//5.00E-56//AT5G60390| elongation factor 1-alpha / EF-1-alpha				GO:0006412//GO:0008135//GO:0017111//GO:0032561//GO:0044424	nr -
Unigene107205_All	647	0.00	0.00	0.00	0.00	0.00	0.00	3.79	0.00	0.00	null	null		null	null		null	null		null	null		-1.19E+01	2.41E-17	Down	-1.19E+01	4.73E-18	Down	Unigene107205_All//gi|81157943|dbj|BAE48222.1|//1.00E-59//elongation factor 2 [Chlorella pyrenoidosa]	Unigene107205_All//4.00E-56//AT1G56070| LOS1; copper ion binding / translation elongation factor/ translation factor, nucleic acid binding				GO:0000166//GO:0016787	nr +
Unigene107206_All	334	0.00	0.00	0.00	0.00	0.00	0.00	4.05	0.00	0.12	null	null		null	null		null	null		null	null		-1.20E+01	1.30E-09	Down	-5.09E+00	8.12E-09	Down	Unigene107206_All//gi|145354676|ref|XP_001421604.1|//2.00E-13//Ribosomal protein S21, component of cytosolic 80S ribosome and 40S small subunit [Ostreococcus lucimarinus CCE9901] >gi|144581842|gb|ABO99897.1| Ribosomal protein S21, component of cytosolic 80S ribosome and 40S small subunit [Ostreococcus lucimarinus CCE9901]	Unigene107206_All//4.00E-11//AT5G27700| structural constituent of ribosome			ko03010|Ribosome	GO:0005198//GO:0010467//GO:0030529//GO:0031410	nr +
Unigene107214_All	395	0.00	0.00	0.00	0.00	0.00	0.00	2.03	0.21	0.00	null	null		null	null		null	null		null	null		-3.26E+00	4.49E-04	Down	-1.10E+01	4.43E-06	Down	Unigene107214_All//gi|223996063|ref|XP_002287705.1|//2.00E-29//RL12, ribosomal protein 11 60S large ribosomal subunit [Thalassiosira pseudonana CCMP1335] >gi|220976821|gb|EED95148.1| RL12, ribosomal protein 11 60S large ribosomal subunit [Thalassiosira pseudonana CCMP1335]	Unigene107214_All//4.00E-29//AT5G60670| 60S ribosomal protein L12 (RPL12C)			ko03010|Ribosome		nr -
Unigene107220_All	222	5.66	0.68	0.24	4.26	3.88	3.26	1.71	0.19	0.72	-3.05E+00	9.00E-05	Down	-4.56E+00	8.14E-06	Down	-1.37E-01	8.18E-01		-3.89E-01	6.28E-01		-3.19E+00	3.12E-02		-1.26E+00	2.48E-01								
Unigene107225_All	442	0.00	0.00	0.00	0.00	0.00	0.00	1.91	0.38	0.00	null	null		null	null		null	null		null	null		-2.34E+00	2.96E-03		-1.09E+01	2.21E-06	Down	Unigene107225_All//gi|195652927|gb|ACG45931.1|//4.00E-29//60S ribosomal protein L22-2 [Zea mays]	Unigene107225_All//2.00E-17//AT5G27770| 60S ribosomal protein L22 (RPL22C)			ko03010|Ribosome	GO:0015934//GO:0016020//GO:0031981	nr +
Unigene107231_All	339	0.46	0.00	0.00	0.44	0.61	0.27	2.49	0.62	0.24	-8.85E+00	2.17E-01		-8.85E+00	2.46E-01		4.68E-01	8.26E-01		-7.26E-01	7.17E-01		-2.02E+00	7.45E-03		-3.41E+00	1.35E-04	Down	Unigene107231_All//gi|27901705|gb|AAO26688.1|AF478375_1//2.00E-15//gag-pol polyprotein [Vitis vinifera]		Unigene107231_All//3.00E-20//TC140327			GO:0003676//GO:0003824//GO:0006259//GO:0046872	nr +
Unigene107239_All	528	0.00	0.00	0.00	0.00	0.00	0.00	2.40	0.16	0.00	null	null		null	null		null	null		null	null		-3.92E+00	6.20E-07	Down	-1.12E+01	1.99E-09	Down	Unigene107239_All//gi|115453465|ref|NP_001050333.1|//4.00E-20//Os03g0406200 [Oryza sativa Japonica Group] >gi|90101284|sp|Q40682.3|EF1D2_ORYSJ RecName: Full=Elongation factor 1-delta 2; Short=EF-1-delta 2; AltName: Full=Elongation factor 1B-beta 2; AltName: Full=eEF-1B beta 2 >gi|3894214|dbj|BAA34598.1| elongation factor 1 beta 2 [Oryza sativa Japonica Group] >gi|3894216|dbj|BAA34599.1| elongation factor 1 beta 2 [Oryza sativa Japonica Group] >gi|53370742|gb|AAU89237.1| elongation factor 1 beta 2 [Oryza sativa Japonica Group] >gi|108708724|gb|ABF96519.1| EF-1 guanine nucleotide exchange domain containing protein, expressed [Oryza sativa (japonica cultivar-group)] >gi|113548804|dbj|BAF12247.1| Os03g0406200 [Oryza sativa Japonica Group]	Unigene107239_All//3.00E-11//AT2G18110| elongation factor 1-beta, putative / EF-1-beta, putative				GO:0006412//GO:0008135//GO:0043234	nr +
Unigene10724_All	291	0.00	0.70	0.00	2.23	0.95	0.47	2.18	0.14	0.00	9.44E+00	1.37E-01		null	null		-1.23E+00	1.60E-01		-2.26E+00	2.76E-02		-3.92E+00	9.81E-04	Down	-1.11E+01	7.12E-05	Down			Unigene10724_All//5.00E-37//DT049498				ESTscan -
Unigene107242_All	230	0.46	0.00	2.55	2.38	2.40	1.77	49.45	35.98	23.21	-8.83E+00	3.76E-01		2.48E+00	3.76E-02		7.90E-03	9.83E-01		-4.31E-01	6.67E-01		-4.59E-01	2.36E-03		-1.09E+00	1.08E-12	Down	Unigene107242_All//gi|163943787|gb|ABY49018.1|//2.00E-07//GA2-oxidase [Leersia tisserantii]	Unigene107242_All//5.00E-07//AT1G78440| ATGA2OX1 (gibberellin 2-oxidase 1); gibberellin 2-beta-dioxygenase	Unigene107242_All//3.00E-59//TC139925		ko00904|Diterpenoid biosynthesis	GO:0016706	nr +
Unigene107248_All	439	0.00	0.00	0.00	0.00	0.00	0.00	2.02	0.57	0.18	null	null		null	null		null	null		null	null		-1.82E+00	1.08E-02		-3.48E+00	7.34E-05	Down							
Unigene10725_All	457	2.29	0.00	1.86	2.07	2.86	0.79	0.19	0.09	0.00	-1.12E+01	3.80E-06	Down	-2.97E-01	6.42E-01		4.67E-01	4.71E-01		-1.39E+00	6.08E-02		-1.02E+00	7.26E-01		-7.53E+00	3.61E-01								
Unigene107256_All	438	0.12	0.12	0.36	0.23	0.00	0.21	3.67	0.95	1.73	-4.66E-02	9.87E-01		1.61E+00	5.12E-01		-7.83E+00	3.17E-01		-1.42E-01	9.53E-01		-1.94E+00	1.42E-04	Down	-1.09E+00	1.58E-02								
Unigene107268_All	373	0.00	6.52	0.71	0.00	0.55	1.82	0.34	0.22	0.21	1.27E+01	7.73E-14	Up	9.48E+00	8.04E-02		9.11E+00	1.57E-01		1.08E+01	4.19E-04	Up	-6.02E-01	7.68E-01		-6.71E-01	7.21E-01		Unigene107268_All//gi|4336434|gb|AAD17804.1|//2.00E-09//nodule-enhanced protein phosphatase type 2C [Lotus japonicus]	Unigene107268_All//3.00E-08//AT5G59220| protein phosphatase 2C, putative / PP2C, putative				GO:0016787	nr -
Unigene107270_All	213	0.00	0.24	0.25	0.47	0.49	0.00	5.36	0.78	3.18	7.89E+00	6.22E-01		7.97E+00	6.41E-01		5.24E-02	9.68E-01		-8.87E+00	4.05E-01		-2.77E+00	7.62E-05	Down	-7.53E-01	1.68E-01				Unigene107270_All//4.00E-83//gi|73863243|gb|DT465982.1|DT465982				ESTscan -
Unigene107277_All	251	1.25	1.21	0.85	8.14	6.59	0.36	0.51	0.33	0.63	-4.63E-02	9.85E-01		-5.60E-01	6.64E-01		-3.05E-01	5.37E-01		-4.50E+00	2.02E-10	Down	-6.02E-01	7.68E-01		3.29E-01	8.52E-01				Unigene107277_All//1.00E-71//DW504295				

Unigene107278_All	239	0.00	0.00	0.00	0.00	0.14	0.38	2.30	0.52	0.00	null	null		null	null		7.17E+00	8.39E-01		8.56E+00	4.81E-01		-2.13E+00	3.32E-02		-1.12E+01	2.81E-04	Down	Unigene107278_All//gi|115472361|ref|NP_001059779.1|//4.00E-12//Os07g0516000 [Oryza sativa Japonica Group] >gi|34393670|dbj|BAC83370.1| alpha-rhamnosidase-like protein [Oryza sativa Japonica Group] >gi|50508733|dbj|BAD31309.1| alpha-rhamnosidase-like protein [Oryza sativa Japonica Group] >gi|113611315|dbj|BAF21693.1| Os07g0516000 [Oryza sativa Japonica Group]	Unigene107278_All//3.00E-12//AT5G57700| BNR/Asp-box repeat family protein	Unigene107278_All//6.00E-33//gi|78325962|gb|DT546236.1|DT546236				nr -
Unigene107279_All	287	0.00	0.00	0.00	0.00	0.00	0.00	1.77	0.44	0.00	null	null		null	null		null	null		null	null		-2.02E+00	5.21E-02		-1.08E+01	5.56E-04	Down	Unigene107279_All//sp|Q9YI97|OAZ2_DANRE//7.00E-07//Ornithine decarboxylase antizyme 2 OS=Danio rerio GN=oaz2 PE=2 SV=1						swissprot +
Unigene10728_All	308	64.04	162.54	115.49	48.04	48.19	47.66	209.34	252.95	101.28	1.34E+00	7.13E-58	Up	8.51E-01	2.40E-19		4.60E-03	9.83E-01		-1.14E-02	9.52E-01		2.73E-01	2.31E-07		-1.05E+00	1.84E-63	Down	Unigene10728_All//gi|225440392|ref|XP_002267559.1|//4.00E-16//PREDICTED: ripening-related protein-like [Vitis vinifera]	Unigene10728_All//3.00E-14//AT5G02230| haloacid dehalogenase-like hydrolase family protein	Unigene10728_All//4.00E-127//gi|73861720|gb|DT464459.1|DT464459			GO:0003824	nr -
Unigene107285_All	224	0.00	0.00	0.00	0.22	0.00	0.20	2.27	0.56	0.00	null	null		null	null		-7.80E+00	5.64E-01		-1.42E-01	9.57E-01		-2.02E+00	5.21E-02		-1.11E+01	5.56E-04	Down							
Unigene107286_All	228	0.00	0.00	0.00	0.00	0.00	0.00	2.78	1.28	0.17	null	null		null	null		null	null		null	null		-1.12E+00	1.80E-01		-3.99E+00	5.83E-04	Down	Unigene107286_All//gi|110737394|dbj|BAF00641.1|//2.00E-31//ATPase [Arabidopsis thaliana]	Unigene107286_All//7.00E-33//AT5G04930| ALA1 (aminophospholipid ATPase1); ATPase, coupled to transmembrane movement of ions, phosphorylative mechanism				GO:0005548//GO:0006869//GO:0009206//GO:0031224//GO:0032559//GO:0042625//GO:0046872	nr +
Unigene107288_All	401	0.00	0.00	0.00	0.00	0.00	0.00	1.48	1.04	0.00	null	null		null	null		null	null		null	null		-5.02E-01	5.74E-01		-1.05E+01	1.42E-04	Down	Unigene107288_All//gi|298711973|emb|CBJ32915.1|//6.00E-25//ribosomal protein L35 [Ectocarpus siliculosus]	Unigene107288_All//7.00E-26//AT1G74270| 60S ribosomal protein L35a (RPL35aC)			ko03010|Ribosome	GO:0009987//GO:0015934//GO:0043231	nr -
Unigene107296_All	477	0.00	0.00	0.00	0.00	0.00	0.00	2.84	0.44	0.00	null	null		null	null		null	null		null	null		-2.69E+00	1.77E-05	Down	-1.15E+01	4.86E-10	Down	Unigene107296_All//gi|301105230|ref|XP_002901699.1|//1.00E-51//40S ribosomal protein S11 [Phytophthora infestans T30-4] >gi|262100703|gb|EEY58755.1| 40S ribosomal protein S11 [Phytophthora infestans T30-4]	Unigene107296_All//1.00E-47//AT3G48930| EMB1080 (embryo defective 1080); structural constituent of ribosome			ko03010|Ribosome	GO:0003723//GO:0005198//GO:0009536//GO:0010467//GO:0015935//GO:0030312	nr +
Unigene107298_All	242	42.38	35.18	26.85	47.96	70.72	83.61	80.02	744.21	90.21	-2.69E-01	1.49E-01		-6.59E-01	3.72E-04		5.60E-01	8.19E-06		8.02E-01	2.85E-11		3.22E+00	0.00E+00	Up	1.73E-01	1.15E-01		Unigene107298_All//gi|297610489|ref|NP_001064615.2|//2.00E-18//Os10g0419100 [Oryza sativa Japonica Group] >gi|255679407|dbj|BAF26529.2| Os10g0419100 [Oryza sativa Japonica Group]	Unigene107298_All//2.00E-13//AT1G60690| aldo/keto reductase family protein	Unigene107298_All//3.00E-81//DR044167			GO:0004033//GO:0008152	nr +
Unigene107299_All	570	0.00	0.00	0.00	0.00	0.00	0.00	2.89	0.59	0.21	null	null		null	null		null	null		null	null		-2.30E+00	1.36E-05	Down	-3.79E+00	5.22E-09	Down	Unigene107299_All//gi|260516654|gb|ACX43954.1|//3.00E-39//cysteine protease 1 [Brachiaria hybrid cultivar] >gi|260516656|gb|ACX43955.1| cysteine protease 1 [Brachiaria hybrid cultivar] >gi|260516658|gb|ACX43956.1| cysteine protease 1 [Brachiaria hybrid cultivar] >gi|260516660|gb|ACX43957.1| cysteine protease 1 [Brachiaria hybrid cultivar] >gi|260516662|gb|ACX43958.1| cysteine protease 2 [Brachiaria hybrid cultivar] >gi|260516664|gb|ACX43959.1| cysteine protease 2 [Brachiaria hybrid cultivar] >gi|260516666|gb|ACX43960.1| cysteine protease 2 [Brachiaria hybrid cultivar] >gi|260516668|gb|ACX43961.1| cysteine protease 2 [Brachiaria hybrid cultivar] >gi|260516670|gb|ACX43962.1| cysteine protease 2 [Brachiaria hybrid cultivar]	Unigene107299_All//1.00E-37//AT5G60360| AALP (Arabidopsis aleurain-like protease); cysteine-type peptidase				GO:0005488//GO:0009725//GO:0032502//GO:0043231//GO:0070011	nr -
Unigene10730_All	503	0.52	0.10	4.66	0.20	0.89	0.00	0.00	0.00	0.00	-2.37E+00	2.14E-01		3.16E+00	3.28E-08	Up	2.17E+00	1.06E-01		-7.63E+00	4.05E-01		null	null		null	null		Unigene10730_All//gi|56130780|ref|YP_145683.1|//9.00E-12//two component response transcription regulator [Ralstonia metallidurans CH34] >gi|94152513|ref|YP_581920.1| regulator protein CopR [Cupriavidus metallidurans CH34] >gi|10047062|emb|CAC07978.1| CopR protein [Ralstonia metallidurans CH34] >gi|56068770|emb|CAI11332.1| two component response transcription regulator [Ralstonia metallidurans CH34] >gi|93358883|gb|ABF12970.1| CopR regulator from the two components sensor/regulator family involved in Cu(II)/Cu(I) resistance [Cupriavidus metallidurans CH34]					GO:0005622//GO:0006350	nr -
Unigene107300_All	502	0.00	0.00	0.00	0.00	0.00	0.00	3.87	0.17	0.95	null	null		null	null		null	null		null	null		-4.54E+00	2.49E-11	Down	-2.02E+00	7.23E-06	Down	Unigene107300_All//gi|188496902|dbj|BAG32350.1|//1.00E-12//glucan binding protein-2 [Actinophrys sol]						nr +
Unigene107302_All	244	0.00	0.00	0.00	0.00	0.00	0.00	3.29	1.03	0.33	null	null		null	null		null	null		null	null		-1.68E+00	2.46E-02		-3.33E+00	2.47E-04	Down							
Unigene10731_All	275	0.57	0.00	0.00	4.53	2.50	0.00	0.15	0.00	0.14	-9.16E+00	2.17E-01		-9.16E+00	2.46E-01		-8.55E-01	1.47E-01		-1.21E+01	8.65E-08	Down	-7.26E+00	6.63E-01		-8.51E-02	9.73E-01				Unigene10731_All//3.00E-118//TC158907				
Unigene107315_All	236	0.00	0.00	0.00	0.00	0.00	0.00	3.05	0.00	0.00	null	null		null	null		null	null		null	null		-1.16E+01	3.27E-05	Down	-1.16E+01	1.78E-05	Down							ESTscan +
Unigene107317_All	359	0.73	0.00	0.74	1.80	0.38	0.13	4.24	1.16	0.55	-9.51E+00	6.72E-02		2.53E-02	1.00E+00		-2.23E+00	1.79E-02		-3.84E+00	2.70E-03		-1.86E+00	3.50E-04	Down	-2.93E+00	7.58E-07	Down			Unigene107317_All//1.00E-90//ES850878				
Unigene107328_All	244	0.00	0.00	0.00	0.00	0.00	0.00	2.08	0.00	0.00	null	null		null	null		null	null		null	null		-1.10E+01	8.91E-04	Down	-1.10E+01	5.56E-04	Down	Unigene107328_All//sp|Q9PLI5|Y114_CHLMU//1.00E-06//Uncharacterized protein TC_0114 OS=Chlamydia muridarum GN=TC_0114 PE=4 SV=2						swissprot +
Unigene107329_All	264	0.00	0.00	0.00	0.75	0.00	0.00	4.64	5.07	0.91	null	null		null	null		-9.56E+00	8.48E-02		-9.56E+00	1.34E-01		1.25E-01	8.14E-01		-2.36E+00	1.22E-04	Down							
Unigene107333_All	257	0.00	0.00	0.00	0.39	0.00	0.35	85.22	47.00	242.75	null	null		null	null		-8.60E+00	3.17E-01		-1.41E-01	9.53E-01		-8.59E-01	1.19E-15		1.51E+00	2.99E-107	Up	Unigene107333_All//gi|40218371|gb|AAR83118.1|//2.00E-47//secretory laccase [Gossypium arboreum]	Unigene107333_All//1.00E-31//AT5G09360| LAC14 (laccase 14); laccase	Unigene107333_All//8.00E-122//TC137114		ko00910|Nitrogen metabolism//ko00053|Ascorbate and aldarate metabolism	GO:0005576//GO:0009808//GO:0016682//GO:0046914	nr +
Unigene107337_All	338	0.00	0.00	0.00	0.00	0.00	0.00	8.26	3.71	0.24	null	null		null	null		null	null		null	null		-1.15E+00	6.98E-04	Down	-5.13E+00	9.17E-18	Down	Unigene107337_All//gi|2894308|emb|CAA11269.1|//3.00E-38//polyubiquitin [Nicotiana tabacum]	Unigene107337_All//5.00E-34//AT4G05320| UBQ10 (POLYUBIQUITIN 10); protein binding	Unigene107337_All//4.00E-41//BF277514			GO:0031410//GO:0043231	nr -
Unigene107338_All	512	0.00	0.00	0.00	0.00	0.00	0.00	3.96	1.80	0.16	null	null		null	null		null	null		null	null		-1.14E+00	5.27E-03		-4.67E+00	1.83E-12	Down							
Unigene107345_All	527	0.20	0.00	0.00	0.28	1.05	0.17	4.41	0.48	0.45	-7.63E+00	3.76E-01		-7.63E+00	3.98E-01		1.88E+00	9.42E-02		-7.27E-01	7.17E-01		-3.21E+00	1.59E-10	Down	-3.28E+00	3.91E-11	Down	Unigene107345_All//gi|28466815|gb|AAO44016.1|//1.00E-08//At1g11655 [Arabidopsis thaliana]		Unigene107345_All//7.00E-22//DW518236				nr +
Unigene107348_All	497	0.21	0.41	0.21	2.00	0.14	0.36	1.70	0.59	0.64	9.54E-01	6.22E-01		2.58E-02	1.00E+00		-3.85E+00	1.61E-05	Down	-2.46E+00	2.07E-03		-1.53E+00	3.21E-02		-1.41E+00	3.70E-02		Unigene107348_All//gi|18415647|ref|NP_568185.1|//4.00E-14//mitochondrial transcription termination factor family protein / mTERF family protein [Arabidopsis thaliana]	Unigene107348_All//1.00E-15//AT5G07900| mitochondrial transcription termination factor family protein / mTERF family protein				GO:0044464	nr -
Unigene107350_All	496	0.00	0.00	0.00	0.00	0.00	0.00	1.62	0.00	0.16	null	null		null	null		null	null		null	null		-1.07E+01	8.58E-06	Down	-3.33E+00	2.47E-04	Down							
Unigene107351_All	383	0.41	0.00	0.28	0.00	0.00	0.00	4.19	0.98	4.68	-8.68E+00	2.17E-01		-5.59E-01	7.76E-01		null	null		null	null		-2.09E+00	5.87E-05	Down	1.58E-01	7.12E-01		Unigene107351_All//gi|73624747|gb|AAZ79231.1|//6.00E-14//cellulose synthase-like protein CslG [Nicotiana tabacum]	Unigene107351_All//1.00E-10//AT4G24000| ATCSLG2; cellulose synthase/ transferase/ transferase, transferring glycosyl groups				GO:0009987//GO:0016020//GO:0016757	nr +
Unigene107354_All	395	0.93	0.51	0.54	1.64	1.05	0.57	1.93	0.00	0.30	-8.54E-01	4.89E-01		-7.82E-01	5.53E-01		-6.48E-01	4.60E-01		-1.52E+00	1.12E-01		-1.09E+01	1.68E-05	Down	-2.67E+00	1.64E-03		Unigene107354_All//gi|240255876|ref|NP_193313.5|//1.00E-51//ATVAMP724 [Arabidopsis thaliana]	Unigene107354_All//3.00E-52//AT4G15780| ATVAMP724	Unigene107354_All//2.00E-169//TC163824			GO:0006810//GO:0031224	nr -
Unigene107361_All	289	0.00	0.00	0.00	0.00	0.00	0.00	2.05	0.43	0.00	null	null		null	null		null	null		null	null		-2.24E+00	2.07E-02		-1.10E+01	1.42E-04	Down	Unigene107361_All//gi|298713802|emb|CBJ27174.1|//4.00E-10//phosphatase PP1 [Ectocarpus siliculosus]	Unigene107361_All//2.00E-05//AT1G64040| TOPP3; protein serine/threonine phosphatase				GO:0016791	nr -
Unigene107362_All	499	0.00	0.00	0.00	0.00	0.00	0.00	1.69	0.34	0.24	null	null		null	null		null	null		null	null		-2.34E+00	2.96E-03		-2.82E+00	5.38E-04	Down	Unigene107362_All//gi|288310312|gb|ADC45395.1|//1.00E-43//HSP90-1 [Glycine max]	Unigene107362_All//2.00E-36//AT5G56010| HSP81-3; ATP binding / unfolded protein binding			ko04626|Plant-pathogen interaction//ko04141|Protein processing in endoplasmic reticulum	GO:0005515//GO:0006950//GO:0016020//GO:0031981//GO:0032559//GO:0044267//GO:0044444//GO:0051592	nr +
Unigene107363_All	228	0.00	0.00	0.00	0.00	0.00	0.00	2.41	0.73	0.00	null	null		null	null		null	null		null	null		-1.72E+00	7.24E-02		-1.12E+01	2.81E-04	Down	Unigene107363_All//gi|26190436|emb|CAD45251.1|//4.00E-06//barley B recombinant [Hordeum vulgare]					GO:0005488	nr -
Unigene107364_All	594	0.26	0.77	0.63	0.84	0.87	1.22	4.06	0.99	1.48	1.54E+00	1.96E-01		1.25E+00	3.66E-01		5.24E-02	9.80E-01		5.37E-01	5.61E-01		-2.04E+00	7.09E-07	Down	-1.46E+00	8.41E-05	Down	Unigene107364_All//gi|108864347|gb|ABA93307.2|//7.00E-47//expressed protein [Oryza sativa (japonica cultivar-group)]	Unigene107364_All//3.00E-41//AT5G53900| unknown protein	Unigene107364_All//1.00E-82//gi|78323562|gb|DT543836.1|DT543836				nr -
Unigene10737_All	400	0.00	3.93	2.26	0.00	0.00	0.00	0.00	0.00	0.00	1.19E+01	5.71E-09	Up	1.11E+01	4.71E-05	Up	null	null		null	null		null	null		null	null		Unigene10737_All//gi|301121760|ref|XP_002908607.1|//1.00E-30//succinyl-CoA ligase subunit alpha, mitochondrial precursor [Phytophthora infestans T30-4] >gi|262103638|gb|EEY61690.1| succinyl-CoA ligase subunit alpha, mitochondrial precursor [Phytophthora infestans T30-4]	Unigene10737_All//5.00E-27//AT5G23250| succinyl-CoA ligase (GDP-forming) alpha-chain, mitochondrial, putative / succinyl-CoA synthetase, alpha chain, putative / SCS-alpha, putative			ko00020|Citrate cycle (TCA cycle)//ko00640|Propanoate metabolism	GO:0004774//GO:0009060//GO:0043231//GO:0046912	nr +
Unigene107372_All	291	0.18	0.00	0.00	0.00	0.12	0.00	1.74	0.00	0.68	-7.49E+00	6.20E-01		-7.49E+00	6.32E-01		6.89E+00	8.39E-01		null	null		-1.08E+01	8.91E-04	Down	-1.35E+00	1.40E-01		Unigene107372_All//gi|115469532|ref|NP_001058365.1|//6.00E-16//Os06g0678800 [Oryza sativa Japonica Group] >gi|113596405|dbj|BAF20279.1| Os06g0678800 [Oryza sativa Japonica Group]	Unigene107372_All//4.00E-12//AT4G22010| sks4 (SKU5 Similar 4); copper ion binding / oxidoreductase	Unigene107372_All//8.00E-36//gi|164305746|gb|EV495235.1|EV495235		ko00053|Ascorbate and aldarate metabolism	GO:0031410	nr +
Unigene107385_All	233	0.00	0.00	0.00	0.00	0.00	0.00	2.18	0.18	0.00	null	null		null	null		null	null		null	null		-3.60E+00	5.78E-03		-1.11E+01	5.56E-04	Down							

Unigene1074_All	1273	15.08	20.26	19.70	48.09	58.32	50.24	10.50	69.47	4.13	4.26E-01	7.04E-05		3.85E-01	6.64E-04		2.78E-01	8.20E-07		6.29E-02	4.53E-01		2.73E+00	0.00E+00	Up	-1.35E+00	3.45E-20	Down	Unigene1074_All//gi|57229665|gb|AAW45741.1|//0.00E+00//sinapyl alcohol dehydrogenase-like protein [Populus tremula x Populus tremuloides]	Unigene1074_All//8.00E-116//AT4G37970| CAD6 (CINNAMYL ALCOHOL DEHYDROGENASE 6); binding / catalytic/ oxidoreductase/ zinc ion binding	Unigene1074_All//0.00E+00//TC154710		ko00940|Phenylpropanoid biosynthesis	GO:0003824//GO:0008152//GO:0046914	nr +
Unigene10740_All	416	0.00	0.00	0.00	0.12	0.00	0.00	5.89	5.63	0.86	null	null		null	null		-6.90E+00	5.64E-01		-6.90E+00	6.45E-01		-6.73E-02	8.65E-01		-2.77E+00	4.81E-10	Down			Unigene10740_All//3.00E-08//TC158843				
Unigene107402_All	735	0.00	0.00	0.00	0.00	0.00	0.00	4.60	2.45	0.92	null	null		null	null		null	null		null	null		-9.12E-01	2.38E-03		-2.32E+00	3.08E-11	Down	Unigene107402_All//gi|238014838|gb|ACR38454.1|//1.00E-07//unknown [Zea mays]		Unigene107402_All//3.00E-22//TC146753				nr +
Unigene107407_All	265	14.02	4.40	7.03	1.50	3.51	2.22	1.28	0.16	0.00	-1.67E+00	1.25E-06	Down	-9.95E-01	2.95E-03		1.22E+00	1.05E-01		5.59E-01	6.01E-01		-3.02E+00	5.31E-02		-1.03E+01	8.18E-03		Unigene107407_All//gi|18400342|ref|NP_565556.1|//7.00E-15//YCF37 (Arabidopsis homolog of Synechocystis YCF37) [Arabidopsis thaliana] >gi|3152615|gb|AAC17094.1| expressed protein [Arabidopsis thaliana] >gi|16648702|gb|AAL25543.1| At2g23670/F26B6.32 [Arabidopsis thaliana] >gi|20197003|gb|AAM14867.1| expressed protein [Arabidopsis thaliana] >gi|20334912|gb|AAM16212.1| At2g23670/F26B6.32 [Arabidopsis thaliana] >gi|206748360|gb|ACI22363.1| YCF37-like protein [Arabidopsis thaliana]	Unigene107407_All//1.00E-07//AT2G23670| YCF37 (Arabidopsis homolog of Synechocystis YCF37)	Unigene107407_All//2.00E-67//TC158076			GO:0009534	nr -
Unigene107411_All	227	0.00	2.01	3.05	1.98	2.43	1.19	0.93	0.00	0.88	1.10E+01	7.44E-03		1.16E+01	6.17E-04	Up	2.97E-01	8.18E-01		-7.26E-01	5.33E-01		-9.86E+00	7.48E-02		-8.58E-02	9.61E-01								
Unigene107413_All	221	0.95	1.83	1.69	0.68	0.47	2.25	3.25	8.89	8.83	9.54E-01	4.14E-01		8.33E-01	5.16E-01		-5.33E-01	7.86E-01		1.73E+00	1.45E-01		1.45E+00	7.26E-04	Up	1.44E+00	6.00E-04	Up			Unigene107413_All//7.00E-11//TC158642				
Unigene107416_All	509	0.62	0.40	0.00	0.10	0.14	0.00	1.74	0.00	0.08	-6.31E-01	6.22E-01		-9.27E+00	4.93E-02		4.67E-01	9.61E-01		-6.61E+00	6.45E-01		-1.08E+01	2.24E-06	Down	-4.48E+00	1.24E-05	Down	Unigene107416_All//gi|15229025|ref|NP_190449.1|//8.00E-24//sterile alpha motif (SAM) domain-containing protein [Arabidopsis thaliana]	Unigene107416_All//5.00E-25//AT3G48800| sterile alpha motif (SAM) domain-containing protein	Unigene107416_All//3.00E-129//TC176975				nr -
Unigene107417_All	388	0.00	0.00	0.00	0.00	0.00	0.00	3.92	0.75	0.00	null	null		null	null		null	null		null	null		-2.38E+00	2.15E-05	Down	-1.19E+01	2.88E-11	Down							
Unigene107424_All	295	0.00	0.00	0.00	0.00	0.00	0.00	3.58	0.57	0.00	null	null		null	null		null	null		null	null		-2.66E+00	2.25E-04	Down	-1.18E+01	6.73E-08	Down	Unigene107424_All//gi|300266716|gb|EFJ50902.1|//3.00E-14//elongation factor 2 (EF-2, EF-TU) [Volvox carteri f. nagariensis]	Unigene107424_All//2.00E-15//AT1G56070| LOS1; copper ion binding / translation elongation factor/ translation factor, nucleic acid binding				GO:0000166//GO:0006950//GO:0008135//GO:0009536//GO:0016020//GO:0016787//GO:0031981//GO:0046914	nr -
Unigene107430_All	502	0.00	0.30	0.00	0.30	0.00	0.18	1.60	1.58	6.11	8.24E+00	2.36E-01		null	null		-8.22E+00	1.66E-01		-7.27E-01	7.17E-01		-1.66E-02	1.02E+00		1.93E+00	3.29E-08	Up	Unigene107430_All//gi|115472685|ref|NP_001059941.1|//3.00E-45//Os07g0550900 [Oryza sativa Japonica Group] >gi|113611477|dbj|BAF21855.1| Os07g0550900 [Oryza sativa Japonica Group]	Unigene107430_All//2.00E-46//AT4G27300| S-locus protein kinase, putative	Unigene107430_All//2.00E-28//gi|164366409|gb|ES802621.1|ES802621		ko04626|Plant-pathogen interaction	GO:0004702//GO:0006464//GO:0008037//GO:0030246//GO:0032559	nr -
Unigene107437_All	489	0.00	0.00	0.00	0.00	0.00	0.00	1.38	0.26	0.00	null	null		null	null		null	null		null	null		-2.43E+00	7.77E-03		-1.04E+01	3.57E-05	Down							ESTscan +
Unigene107438_All	581	0.00	0.00	0.00	0.00	0.00	0.00	2.69	0.43	0.00	null	null		null	null		null	null		null	null		-2.64E+00	4.08E-06	Down	-1.14E+01	1.42E-11	Down							
Unigene107444_All	616	0.00	0.16	0.52	0.00	0.56	0.00	2.33	0.14	0.97	7.36E+00	3.90E-01		9.02E+00	4.54E-02		9.13E+00	3.02E-02		null	null		-4.10E+00	5.15E-08	Down	-1.27E+00	9.34E-03								
Unigene107449_All	339	0.00	0.00	0.00	0.00	0.00	0.00	2.49	0.12	0.00	null	null		null	null		null	null		null	null		-4.34E+00	4.56E-05	Down	-1.13E+01	2.21E-06	Down							ESTscan -
Unigene107450_All	879	0.24	0.06	0.00	0.17	0.04	0.15	2.02	0.52	0.41	-2.05E+00	3.34E-01		-7.90E+00	1.47E-01		-2.12E+00	3.87E-01		-1.42E-01	9.44E-01		-1.95E+00	5.37E-05	Down	-2.31E+00	3.08E-06	Down							ESTscan -
Unigene107451_All	867	0.00	0.00	0.00	0.00	0.00	0.00	2.39	0.05	0.05	null	null		null	null		null	null		null	null		-5.63E+00	2.86E-13	Down	-5.70E+00	7.21E-14	Down	Unigene107451_All//gi|301122631|ref|XP_002909042.1|//2.00E-63//40S ribosomal protein S6 [Phytophthora infestans T30-4] >gi|262099804|gb|EEY57856.1| 40S ribosomal protein S6 [Phytophthora infestans T30-4]	Unigene107451_All//9.00E-58//AT4G31700| RPS6 (RIBOSOMAL PROTEIN S6); structural constituent of ribosome	Unigene107451_All//3.00E-16//CD486323		ko03010|Ribosome	GO:0005198//GO:0010467//GO:0015935//GO:0016020	nr -
Unigene107452_All	837	0.00	0.00	0.00	0.12	0.29	0.11	5.66	0.85	1.05	null	null		null	null		1.28E+00	5.32E-01		-1.41E-01	9.53E-01		-2.74E+00	8.02E-18	Down	-2.43E+00	2.86E-16	Down	Unigene107452_All//gi|15222073|ref|NP_175349.1|//2.00E-56//zinc finger (C3HC4-type RING finger) family protein [Arabidopsis thaliana] >gi|68565178|sp|Q6NQG7.1|ATL1L_ARATH RecName: Full=RING-H2 finger protein ATL1L >gi|34365601|gb|AAQ65112.1| At1g49230 [Arabidopsis thaliana]	Unigene107452_All//2.00E-48//AT1G49230| zinc finger (C3HC4-type RING finger) family protein	Unigene107452_All//0.00E+00//TC137887		ko04141|Protein processing in endoplasmic reticulum//ko04120|Ubiquitin mediated proteolysis	GO:0019787//GO:0032446//GO:0046914	nr +
Unigene107455_All	610	0.00	0.00	0.00	0.00	0.00	0.00	5.13	1.30	0.98	null	null		null	null		null	null		null	null		-1.98E+00	2.16E-08	Down	-2.39E+00	8.62E-11	Down	Unigene107455_All//gi|118572295|sp|Q8LBI9.2|EDL16_ARATH//2.00E-25//RecName: Full=Sugar transporter ERD6-like 16 >gi|17381265|gb|AAL36051.1| AT5g18840/F17K4_90 [Arabidopsis thaliana] >gi|23309019|gb|AAN18269.1| At5g18840/F17K4_90 [Arabidopsis thaliana]	Unigene107455_All//1.00E-26//AT5G18840| sugar transporter, putative	Unigene107455_All//2.00E-10//TC145455			GO:0006810//GO:0016020//GO:0022891	nr -
Unigene107456_All	460	0.00	0.00	0.00	0.00	0.00	0.00	2.21	0.00	0.61	null	null		null	null		null	null		null	null		-1.11E+01	2.97E-07	Down	-1.86E+00	3.54E-03		Unigene107456_All//gi|3834323|gb|AAC83039.1|//2.00E-40//F9K20.25 [Arabidopsis thaliana]	Unigene107456_All//2.00E-41//AT1G78710| unknown protein	Unigene107456_All//1.00E-162//gi|109862280|gb|DW491257.1|DW491257				nr -
Unigene10748_All	243	30.36	927.27	324.98	95.94	221.21	295.70	0.00	0.17	0.00	4.93E+00	0.00E+00	Up	3.42E+00	2.61E-285	Up	1.21E+00	5.10E-62	Up	1.62E+00	1.01E-117	Up	7.43E+00	6.38E-01		null	null		Unigene10748_All//gi|6723687|emb|CAB67122.1|//1.00E-12//cell wall protein [Nicotiana tabacum] >gi|270313156|gb|ACZ73645.1| glycine-rich protein precursor [Nicotiana tabacum]		Unigene10748_All//1.00E-129//TC169148				nr +
Unigene107487_All	357	13.34	220.56	27.30	20.09	35.98	54.40	80.77	165.66	128.22	4.05E+00	0.00E+00	Up	1.03E+00	9.76E-08	Up	8.40E-01	1.46E-08		1.44E+00	6.41E-27	Up	1.04E+00	1.29E-55	Up	6.67E-01	2.56E-21		Unigene107487_All//gi|13161401|dbj|BAB33035.1|//5.00E-17//CPRD48 [Vigna unguiculata]	Unigene107487_All//2.00E-16//AT5G42050| FUNCTIONS IN: molecular_function unknown; EXPRESSED IN: 22 plant structures; EXPRESSED DURING: 13 growth stages; CONTAINS InterPro DOMAIN/s: Development and cell death domain (InterPro:IPR013989), Kelch related (InterPro:IPR013089); BEST Arabidopsis thaliana protein match is: unknown protein (TAIR:AT3G27090.1); Has 5084 Blast hits to 2870 proteins in 85 species: Archae - 0; Bacteria - 24; Metazoa - 270; Fungi - 58; Plants - 157; Viruses - 8; Other Eukaryotes - 4567 (source: NCBI BLink).	Unigene107487_All//2.00E-147//TC138767				nr +
Unigene107488_All	1293	0.49	0.55	0.58	0.08	0.21	0.07	0.46	0.78	1.45	1.76E-01	8.17E-01		2.48E-01	7.45E-01		1.47E+00	4.20E-01		-1.41E-01	9.53E-01		7.61E-01	2.28E-01		1.66E+00	1.72E-04	Up	Unigene107488_All//gi|15221299|ref|NP_176986.1|//3.00E-29//zinc finger (B-box type) family protein [Arabidopsis thaliana] >gi|17433041|sp|Q9C9F4.1|COLX_ARATH RecName: Full=Putative zinc finger protein At1g68190	Unigene107488_All//2.00E-31//AT1G68190| zinc finger (B-box type) family protein	Unigene107488_All//0.00E+00//TC157110	C2C2-CO-like	ko04712|Circadian rhythm - plant		nr +
Unigene10749_All	430	0.00	0.00	0.00	0.00	0.00	0.00	3.93	3.69	1.30	null	null		null	null		null	null		null	null		-9.06E-02	8.48E-01		-1.60E+00	5.13E-04	Down			Unigene10749_All//3.00E-27//ES810763				
Unigene107493_All	662	0.00	0.00	0.00	0.00	0.00	0.00	3.13	3.22	0.00	null	null		null	null		null	null		null	null		4.11E-02	9.39E-01		-1.16E+01	2.86E-15	Down	Unigene107493_All//gi|192910824|gb|ACF06520.1|//3.00E-64//60S ribosomal protein L7 [Elaeis guineensis]	Unigene107493_All//1.00E-56//AT3G13580| 60S ribosomal protein L7 (RPL7D)			ko03010|Ribosome		nr -
Unigene107494_All	233	0.00	0.00	0.00	0.00	0.00	0.00	2.54	1.44	0.00	null	null		null	null		null	null		null	null		-8.24E-01	3.48E-01		-1.13E+01	1.42E-04	Down	Unigene107494_All//gi|29539344|dbj|BAC67673.1|//6.00E-14//ribosomal protein S8 [Cyanidioschyzon merolae]	Unigene107494_All//6.00E-13//AT5G20290| 40S ribosomal protein S8 (RPS8A)			ko03010|Ribosome		nr -
Unigene107495_All	392	0.00	0.00	0.00	0.00	0.00	0.00	8.95	0.64	0.20	null	null		null	null		null	null		null	null		-3.81E+00	6.87E-18	Down	-5.46E+00	7.62E-23	Down							
Unigene107501_All	256	0.00	0.00	0.00	0.00	0.00	0.00	2.31	0.00	0.00	null	null		null	null		null	null		null	null		-1.12E+01	2.40E-04	Down	-1.12E+01	1.42E-04	Down							
Unigene107504_All	254	0.00	0.00	0.00	0.00	0.00	0.00	3.50	0.00	0.31	null	null		null	null		null	null		null	null		-1.18E+01	2.24E-06	Down	-3.48E+00	7.34E-05	Down	Unigene107504_All//gi|159471131|ref|XP_001693710.1|//3.00E-06//ribosomal protein S3a, component of cytosolic 80S ribosome and 40S small subunit [Chlamydomonas reinhardtii] >gi|269969596|sp|A8HS48.1|RS3A_CHLRE RecName: Full=40S ribosomal protein S3a >gi|158283213|gb|EDP08964.1| ribosomal protein S3a [Chlamydomonas reinhardtii]				ko03010|Ribosome		nr +
Unigene107509_All	402	0.00	0.00	0.00	0.00	0.00	0.00	2.31	1.46	0.00	null	null		null	null		null	null		null	null		-6.69E-01	3.12E-01		-1.12E+01	5.48E-07	Down	Unigene107509_All//gi|195615414|gb|ACG29537.1|//1.00E-33//60S ribosomal protein L23a [Zea mays] >gi|195641834|gb|ACG40385.1| 60S ribosomal protein L23a [Zea mays] >gi|195645532|gb|ACG42234.1| 60S ribosomal protein L23a [Zea mays]	Unigene107509_All//2.00E-30//AT3G55280| RPL23AB (RIBOSOMAL PROTEIN L23AB); RNA binding / nucleotide binding / structural constituent of ribosome	Unigene107509_All//1.00E-13//DW502458		ko03010|Ribosome		nr +
Unigene10751_All	441	1.31	0.57	0.12	3.16	1.64	0.72	0.38	0.85	0.81	-1.18E+00	2.26E-01		-3.43E+00	1.41E-02		-9.48E-01	8.16E-02		-2.14E+00	6.16E-04	Down	1.15E+00	3.28E-01		1.08E+00	3.50E-01		Unigene10751_All//gi|124359292|gb|ABD28429.2|//3.00E-52//Regulator of chromosome condensation/beta-lactamase-inhibitor protein II [Medicago truncatula]	Unigene10751_All//5.00E-45//AT3G03790| ankyrin repeat family protein / regulator of chromosome condensation (RCC1) family protein					nr +
Unigene107517_All	309	0.00	0.00	0.00	0.00	0.00	0.00	2.05	0.14	1.29	null	null		null	null		null	null		null	null		-3.92E+00	9.81E-04	Down	-6.71E-01	3.86E-01								
Unigene107518_All	267	0.00	0.00	0.00	0.00	0.00	0.00	2.38	0.31	0.00	null	null		null	null		null	null		null	null		-2.92E+00	4.20E-03		-1.12E+01	7.12E-05	Down	Unigene107518_All//gi|60543080|dbj|BAD90789.1|//8.00E-22//histone 3 [Marchantia polymorpha]	Unigene107518_All//2.00E-20//AT1G75600| histone H3.2, putative					nr +
Unigene107519_All	264	0.00	0.00	0.00	0.00	0.00	0.00	2.40	0.00	0.30	null	null		null	null		null	null		null	null		-1.12E+01	1.24E-04	Down	-2.99E+00	2.58E-03								
Unigene107521_All	444	0.00	0.00	0.00	0.00	0.08	0.00	2.10	0.09	0.63	null	null		null	null		6.28E+00	8.39E-01		null	null		-4.48E+00	1.30E-05	Down	-1.74E+00	8.55E-03				Unigene107521_All//2.00E-126//CO128086				
Unigene107532_All	369	0.00	0.00	0.00	0.00	0.00	0.00	6.07	1.02	0.54	null	null		null	null		null	null		null	null		-2.57E+00	2.90E-08	Down	-3.49E+00	2.61E-11	Down	Unigene107532_All//gi|10802935|gb|AAG23683.1|AF288091_28//2.00E-20//apocytochrome b [Thraustochytrium aureum]	Unigene107532_All//1.00E-14//ATMG00220| Mitochondrial apocytochrome b (cob) gene encodes a subunit of the ubiquinol-cytochrome c oxidoreductase and is part of a 5 kb transcript. The transcript also contains a pseudogene for ribosomal protein S14 called RPS15 and a tRNA(Ser) gene. Both the Cob and RPS15 genes are edited in the transcript.			ko00190|Oxidative phosphorylation		nr +
Unigene107537_All	259	0.81	0.00	0.00	0.19	0.13	0.35	5.22	0.32	0.77	-9.66E+00	1.23E-01		-9.66E+00	1.47E-01		-5.32E-01	8.28E-01		8.59E-01	7.97E-01		-4.02E+00	1.80E-07	Down	-2.76E+00	7.51E-06	Down			Unigene107537_All//6.00E-24//TC140227				

Unigene107544_All	240	0.00	0.00	0.00	0.00	0.00	0.00	2.99	0.17	0.00	null	null		null	null		null	null		null	null		-4.10E+00	2.91E-04	Down	-1.15E+01	1.78E-05	Down	Unigene107544_All//gi|6094014|sp|O82528.1|RL15_PETHY//5.00E-11//RecName: Full=60S ribosomal protein L15 >gi|3608479|gb|AAD13389.1| ribosomal protein L15 [Petunia x hybrida]	Unigene107544_All//1.00E-08//AT4G16720| 60S ribosomal protein L15 (RPL15A)			ko03010|Ribosome		nr -
Unigene107546_All	363	0.00	0.00	0.00	0.00	0.00	0.00	2.33	0.12	0.00	null	null		null	null		null	null		null	null		-4.34E+00	4.56E-05	Down	-1.12E+01	2.21E-06	Down	Unigene107546_All//gi|226502949|ref|NP_001149726.1|//2.00E-35//60S ribosomal protein L5-1 [Zea mays] >gi|195629822|gb|ACG36552.1| 60S ribosomal protein L5-1 [Zea mays]	Unigene107546_All//3.00E-30//AT3G25520| ATL5 (A. THALIANA RIBOSOMAL PROTEIN L5); 5S rRNA binding / structural constituent of ribosome			ko03010|Ribosome		nr -
Unigene107553_All	232	0.00	0.00	0.00	0.00	0.00	0.00	5.47	1.62	0.34	null	null		null	null		null	null		null	null		-1.75E+00	2.19E-03		-3.99E+00	2.56E-07	Down							ESTscan +
Unigene10756_All	489	0.00	9.53	4.90	0.00	0.00	0.00	0.00	0.00	0.00	1.32E+01	1.46E-26	Up	1.23E+01	3.24E-13	Up	null	null		null	null		null	null		null	null								
Unigene107564_All	372	0.00	0.00	0.00	0.00	0.00	0.00	2.50	0.00	0.00	null	null		null	null		null	null		null	null		-1.13E+01	1.15E-06	Down	-1.13E+01	5.48E-07	Down							
Unigene107569_All	273	269.46	298.45	201.69	64.23	100.53	93.30	19.67	198.57	99.82	1.47E-01	1.50E-02		-4.18E-01	1.76E-11		6.46E-01	1.86E-11		5.39E-01	2.80E-07		3.34E+00	7.83E-240	Up	2.34E+00	9.28E-85	Up	Unigene107569_All//gi|255647586|gb|ACU24256.1|//2.00E-16//unknown [Glycine max]		Unigene107569_All//3.00E-66//gi|109847166|gb|DW476146.1|DW476146				nr -
Unigene10757_All	356	0.00	39.85	18.55	0.00	0.00	0.00	0.00	0.00	0.00	1.53E+01	1.82E-81	Up	1.42E+01	7.37E-37	Up	null	null		null	null		null	null		null	null		Unigene10757_All//gi|224005945|ref|XP_002291933.1|//1.00E-18//trypsin-like serine protease [Thalassiosira pseudonana CCMP1335] >gi|220972452|gb|EED90784.1| trypsin-like serine protease [Thalassiosira pseudonana CCMP1335]					GO:0016787	nr +
Unigene107570_All	611	0.00	0.00	0.00	0.00	0.00	0.00	15.02	1.78	14.54	null	null		null	null		null	null		null	null		-3.08E+00	3.41E-38	Down	-4.65E-02	7.99E-01		Unigene107570_All//gi|15233696|ref|NP_195527.1|//6.00E-18//zinc finger (C3HC4-type RING finger) family protein [Arabidopsis thaliana] >gi|68565341|sp|Q9SZL4.1|ATL4N_ARATH RecName: Full=RING-H2 finger protein ATL4N; Flags: Precursor >gi|124301120|gb|ABN04812.1| At4g38140 [Arabidopsis thaliana]	Unigene107570_All//3.00E-19//AT4G38140| zinc finger (C3HC4-type RING finger) family protein			ko04120|Ubiquitin mediated proteolysis		nr -
Unigene107577_All	271	0.00	0.00	0.00	0.00	0.00	0.00	2.81	0.00	0.00	null	null		null	null		null	null		null	null		-1.15E+01	1.68E-05	Down	-1.15E+01	8.89E-06	Down	Unigene107577_All//gi|1256830|gb|AAB68374.1|//2.00E-06//cysteine endopeptidase 1 [Phaseolus vulgaris] >gi|2959418|emb|CAA12118.1| cysteine protease [Phaseolus vulgaris]	Unigene107577_All//5.00E-05//AT3G45310| cysteine proteinase, putative					nr -
Unigene10758_All	991	0.00	0.36	0.54	6.48	8.41	3.24	0.26	0.00	0.24	8.48E+00	2.47E-02		9.07E+00	4.05E-03		3.75E-01	6.65E-02		-1.00E+00	1.24E-05	Down	-8.00E+00	4.06E-02		-8.59E-02	9.57E-01		Unigene10758_All//gi|75296458|sp|Q7XSK0.2|BGL18_ORYSJ//3.00E-58//RecName: Full=Beta-glucosidase 18; Short=Os4bglu18; Flags: Precursor >gi|38345329|emb|CAE54546.1| OSJNBa0004N05.26 [Oryza sativa (japonica cultivar-group)] >gi|38345687|emb|CAE01910.2| OSJNBb0070J16.3 [Oryza sativa (japonica cultivar-group)] >gi|116310833|emb|CAH67620.1| OSIGBa0140J09.1 [Oryza sativa (indica cultivar-group)]	Unigene10758_All//4.00E-56//AT4G21760| BGLU47 (Beta-glucosidase 47); catalytic/ cation binding / hydrolase, hydrolyzing O-glycosyl compounds	Unigene10758_All//0.00E+00//CO109603		ko00500|Starch and sucrose metabolism//ko00940|Phenylpropanoid biosynthesis//ko00460|Cyanoamino acid metabolism	GO:0008422//GO:0009699//GO:0031410//GO:0043167	nr +
Unigene107582_All	929	0.00	0.00	0.00	0.00	0.00	0.00	4.82	1.75	0.09	null	null		null	null		null	null		null	null		-1.46E+00	6.61E-08	Down	-5.81E+00	8.77E-30	Down	Unigene107582_All//gi|115465379|ref|NP_001056289.1|//1.00E-55//Os05g0557500 [Oryza sativa Japonica Group] >gi|113579840|dbj|BAF18203.1| Os05g0557500 [Oryza sativa Japonica Group]	Unigene107582_All//5.00E-55//AT4G21700| unknown protein	Unigene107582_All//1.00E-117//CO096440				nr +
Unigene107584_All	308	0.00	0.00	0.00	0.00	0.00	0.00	1.92	0.68	0.00	null	null		null	null		null	null		null	null		-1.50E+00	9.14E-02		-1.09E+01	1.42E-04	Down	Unigene107584_All//sp|Q58A42|DD3_DICDI//2.00E-14//Protein DD3-3 OS=Dictyostelium discoideum GN=DD3-3 PE=2 SV=1						swissprot -
Unigene107585_All	227	0.00	0.00	0.00	0.00	0.00	0.00	3.54	0.18	0.35	null	null		null	null		null	null		null	null		-4.26E+00	8.48E-05	Down	-3.33E+00	2.47E-04	Down							
Unigene107598_All	252	0.00	0.00	0.00	0.00	0.00	0.00	9.06	10.12	0.16	null	null		null	null		null	null		null	null		1.59E-01	6.69E-01		-5.84E+00	2.26E-15	Down	Unigene107598_All//gi|145408590|ref|YP_001152214.1|//3.00E-14//ORF124 [Pinus koraiensis] >gi|145048839|gb|ABP35456.1| ORF124 [Pinus koraiensis]		Unigene107598_All//3.00E-38//gi|11208796|gb|BF277726.1|BF277726				nr +
Unigene1076_All	1674	0.16	3.21	1.08	4.20	6.40	4.86	2.00	14.18	0.36	4.36E+00	4.15E-24	Up	2.79E+00	8.64E-06	Up	6.09E-01	2.23E-04		2.11E-01	3.75E-01		2.83E+00	1.34E-89	Up	-2.48E+00	5.85E-12	Down	Unigene1076_All//gi|57229665|gb|AAW45741.1|//1.00E-179//sinapyl alcohol dehydrogenase-like protein [Populus tremula x Populus tremuloides]	Unigene1076_All//2.00E-111//AT4G37970| CAD6 (CINNAMYL ALCOHOL DEHYDROGENASE 6); binding / catalytic/ oxidoreductase/ zinc ion binding	Unigene1076_All//0.00E+00//TC154710		ko00940|Phenylpropanoid biosynthesis		nr +
Unigene10760_All	452	0.00	0.00	0.00	0.00	0.00	0.00	5.05	2.59	0.62	null	null		null	null		null	null		null	null		-9.64E-01	1.09E-02		-3.03E+00	3.22E-10	Down			Unigene10760_All//2.00E-19//TC149074				
Unigene107612_All	295	0.00	0.00	0.00	0.00	0.00	0.00	1.72	0.00	0.00	null	null		null	null		null	null		null	null		-1.07E+01	8.91E-04	Down	-1.07E+01	5.56E-04	Down	Unigene107612_All//gi|255085060|ref|XP_002504961.1|//7.00E-10//COMPASS/Set1C complex protein [Micromonas sp. RCC299] >gi|226520230|gb|ACO66219.1| COMPASS/Set1C complex protein [Micromonas sp. RCC299]				ko03040|Spliceosome		nr +
Unigene107615_All	252	0.00	0.00	0.00	0.20	0.14	0.00	2.35	0.00	0.16	null	null		null	null		-5.33E-01	8.28E-01		-7.63E+00	6.45E-01		-1.12E+01	2.40E-04	Down	-3.89E+00	1.10E-03		Unigene107615_All//gi|224136035|ref|XP_002322223.1|//3.00E-26//sulfate/bicarbonate/oxalate exchanger and transporter sat-1 [Populus trichocarpa] >gi|222869219|gb|EEF06350.1| sulfate/bicarbonate/oxalate exchanger and transporter sat-1 [Populus trichocarpa]	Unigene107615_All//2.00E-22//AT5G50300| xanthine/uracil/vitamin C permease family protein					nr +
Unigene107622_All	243	0.22	1.25	0.00	0.00	0.14	0.00	2.09	0.00	0.00	2.54E+00	1.51E-01		-7.75E+00	6.32E-01		7.15E+00	8.38E-01		null	null		-1.10E+01	8.91E-04	Down	-1.10E+01	5.56E-04	Down			Unigene107622_All//2.00E-14//FL577038				
Unigene10764_All	464	0.00	0.00	0.00	2.68	1.19	0.49	0.18	0.00	1.20	null	null		null	null		-1.18E+00	4.23E-02		-2.46E+00	4.15E-04	Down	-7.51E+00	4.07E-01		2.72E+00	9.38E-03		Unigene10764_All//gi|297834560|ref|XP_002885162.1|//6.00E-24//nodulin MtN3 family protein [Arabidopsis lyrata subsp. lyrata] >gi|297331002|gb|EFH61421.1| nodulin MtN3 family protein [Arabidopsis lyrata subsp. lyrata]	Unigene10764_All//3.00E-24//AT3G16690| nodulin MtN3 family protein				GO:0031224	nr -
Unigene107652_All	239	0.00	0.00	0.00	0.00	0.00	0.00	1.42	0.87	10.33	null	null		null	null		null	null		null	null		-6.95E-01	5.78E-01		2.87E+00	2.95E-10	Up							
Unigene107656_All	1286	0.00	0.00	0.00	0.00	0.00	0.00	3.95	1.27	0.31	null	null		null	null		null	null		null	null		-1.64E+00	2.49E-10	Down	-3.67E+00	8.13E-26	Down	Unigene107656_All//gi|1706590|sp|P50257.1|EF1AS_PORPU//1.00E-102//RecName: Full=Elongation factor 1-alpha S; Short=EF-1-alpha S; AltName: Full=Sporophyte-specific EF-1-alpha >gi|476149|gb|AAA61790.1| EF1-alpha [Porphyra purpurea]	Unigene107656_All//2.00E-123//AT5G60390| elongation factor 1-alpha / EF-1-alpha	Unigene107656_All//1.00E-13//BF275715				nr -
Unigene107659_All	247	37.28	8.21	21.99	7.87	10.73	6.95	1.20	0.34	0.00	-2.18E+00	1.38E-21	Down	-7.62E-01	1.10E-04		4.49E-01	2.82E-01		-1.79E-01	7.02E-01		-1.82E+00	2.11E-01		-1.02E+01	1.59E-02		Unigene107659_All//gi|210061121|gb|ACJ05633.1|//5.00E-10//squalene epoxidase 1 [Nigella sativa]	Unigene107659_All//7.00E-05//AT5G24160| SQE6 (SQUALENE MONOXYGENASE 6); FAD binding / oxidoreductase/ squalene monooxygenase	Unigene107659_All//1.00E-58//CO083658		ko00100|Steroid biosynthesis		nr +
Unigene107661_All	960	0.00	0.00	0.00	0.00	0.00	0.00	3.79	0.00	3.57	null	null		null	null		null	null		null	null		-1.19E+01	1.01E-25	Down	-8.58E-02	7.66E-01		Unigene107661_All//gi|225442485|ref|XP_002278313.1|//2.00E-86//PREDICTED: similar to PDR8/PEN3 (PLEIOTROPIC DRUG RESISTANCE8); ATPase, coupled to transmembrane movement of substances / cadmium ion transmembrane transporter [Vitis vinifera]	Unigene107661_All//5.00E-70//AT1G15210| PDR7 (PLEIOTROPIC DRUG RESISTANCE 7); ATPase, coupled to transmembrane movement of substances					nr -
Unigene107663_All	385	0.00	0.00	0.00	0.00	0.00	0.00	6.37	1.30	0.31	null	null		null	null		null	null		null	null		-2.29E+00	6.20E-08	Down	-4.36E+00	2.22E-14	Down	Unigene107663_All//gi|11466301|ref|NP_051129.1|//2.00E-20//cytochrome c oxidase subunit 3 [Cafeteria roenbergensis] >gi|6180083|gb|AAF05780.1|AF193903_3 cytochrome c oxidase subunit 3 [Cafeteria roenbergensis]	Unigene107663_All//1.00E-07//ATMG00730| Encodes cytochrome c oxidase subunit 3.			ko00190|Oxidative phosphorylation		nr -
Unigene107669_All	283	0.00	0.00	0.00	0.00	0.00	0.00	2.09	0.59	0.00	null	null		null	null		null	null		null	null		-1.82E+00	4.72E-02		-1.10E+01	1.42E-04	Down							
Unigene107673_All	426	0.00	0.36	0.00	0.35	0.16	0.21	1.99	0.20	0.75	8.48E+00	2.36E-01		null	null		-1.12E+00	5.85E-01		-7.27E-01	7.17E-01		-3.34E+00	2.52E-04	Down	-1.41E+00	3.70E-02								
Unigene107684_All	626	0.00	0.00	0.00	0.00	0.17	0.00	2.03	0.40	0.45	null	null		null	null		7.37E+00	4.58E-01		null	null		-2.34E+00	1.56E-04	Down	-2.19E+00	1.97E-04	Down	Unigene107684_All//gi|108710987|gb|ABF98782.1|//2.00E-17//expressed protein [Oryza sativa (japonica cultivar-group)]	Unigene107684_All//5.00E-33//AT5G42655| FUNCTIONS IN: molecular_function unknown; INVOLVED IN: biological_process unknown; LOCATED IN: cellular_component unknown; BEST Arabidopsis thaliana protein match is: disease resistance-responsive family protein (TAIR:AT5G42500.1); Has 121 Blast hits to 121 proteins in 14 species: Archae - 0; Bacteria - 0; Metazoa - 0; Fungi - 0; Plants - 121; Viruses - 0; Other Eukaryotes - 0 (source: NCBI BLink).	Unigene107684_All//9.00E-25//gi|21098523|gb|BQ410848.1|BQ410848				nr +
Unigene107688_All	233	0.00	0.00	0.00	0.00	0.00	0.00	6.90	2.69	0.85	null	null		null	null		null	null		null	null		-1.36E+00	4.47E-03		-3.01E+00	2.36E-07	Down	Unigene107688_All//gi|4139170|gb|AAD03711.1|//2.00E-38//elongation translation factor 1 alpha [Cyanophora paradoxa]	Unigene107688_All//5.00E-36//AT5G60390| elongation factor 1-alpha / EF-1-alpha	Unigene107688_All//1.00E-43//TC166357				nr -
Unigene10769_All	436	0.00	6.97	2.93	0.00	0.00	0.00	0.00	0.00	0.00	1.28E+01	2.67E-17	Up	1.15E+01	4.69E-07	Up	null	null		null	null		null	null		null	null		Unigene10769_All//sp|P06002|OPS1_DROME//2.00E-40//Opsin Rh1 OS=Drosophila melanogaster GN=ninaE PE=1 SV=1						swissprot -
Unigene107690_All	312	0.00	0.00	0.00	0.00	0.00	0.00	2.44	0.40	0.00	null	null		null	null		null	null		null	null		-2.60E+00	2.81E-03		-1.13E+01	8.89E-06	Down	Unigene107690_All//gi|300263896|gb|EFJ48094.1|//6.00E-33//component of cytosolic 80S ribosome and 60S large subunit [Volvox carteri f. nagariensis]	Unigene107690_All//3.00E-30//AT3G13580| 60S ribosomal protein L7 (RPL7D)	Unigene107690_All//4.00E-07//gi|21100940|gb|BQ413253.1|BQ413253		ko03010|Ribosome		nr -

Unigene107693_All	398	0.00	0.00	0.00	0.00	0.00	0.00	25.92	39.59	151.15	null	null		null	null		null	null		null	null		6.11E-01	1.04E-06		2.54E+00	4.88E-205	Up	Unigene107693_All//gi|224127083|re<br>f|XP_002329388.1|//2.00E-<br>08//predicted protein [Populus<br>trichocarpa]<br>>gi|222870438|gb|EEF07569.1| predicted protein [Populus<br>trichocarpa]		Unigene107693_All//4.00E-78//TC175720				nr +
Unigene107695_All	356	0.00	0.00	0.00	0.00	0.00	0.00	1.66	0.70	0.00	null	null		null	null		null	null		null	null		-1.24E+00	1.54E-01		-1.07E+01	1.42E-04	Down	Unigene107695_All//gi|300123728|em<br>b|CBK25000.2|//5.00E-24//Ribosomal<br>protein L18a [Blastocystis<br>hominis]<br>>gi|300175241|emb|CBK20552.2| Ribosomal protein L18a<br>[Blastocystis hominis]	Unigene107695_All//1.00E-<br>17//AT2G34480| 60S ribosomal<br>protein L18A (RPL18aB)			ko03010|Ribosome		nr -
Unigene1077_All	584	0.63	0.69	3.83	1.88	1.42	0.85	0.94	0.21	0.75	1.46E-01	8.98E-01		2.61E+00	1.40E-06	Up	-4.07E-01	5.61E-01		-1.14E+00	8.74E-02		-2.13E+00	3.32E-02		-3.27E-01	6.72E-01		Unigene1077_All//gi|224089188|ref| XP_002308654.1|//2.00E-<br>06//predicted protein [Populus<br>trichocarpa]<br>>gi|222854630|gb|EEE92177.1| predicted protein [Populus<br>trichocarpa]		Unigene1077_All//6.00E-<br>146//gi|78350462|gb|DT570736.1|DT570736				nr -
Unigene107708_All	558	0.00	1.18	0.29	0.18	0.19	0.65	0.15	0.22	0.07	1.02E+01	6.26E-04	Up	8.16E+00	2.40E-01		5.24E-02	9.68E-01		1.86E+00	2.20E-01		5.69E-01	7.83E-01		-1.09E+00	6.77E-01		Unigene107708_All//gi|224134959|re<br>f|XP_002327532.1|//6.00E-13//f-box<br>family protein [Populus<br>trichocarpa]<br>>gi|222836086|gb|EEE74507.1| f-box<br>family protein [Populus<br>trichocarpa]		Unigene107708_All//0.00E+00//TC178035				nr +
Unigene10771_All	321	0.00	0.95	1.16	0.00	0.11	0.00	115.38	321.61	385.11	9.89E+00	4.45E-02		1.02E+01	2.50E-02		6.75E+00	8.38E-01		null	null		1.48E+00	3.94E-168	Up	1.74E+00	2.87E-259	Up	Unigene10771_All//gi|197312909|gb| ACH63235.1|//2.00E-37//alcohol<br>dehydrogenase [Rheum australe]	Unigene10771_All//3.00E-<br>35//AT3G24503| ALDH2C4; 3-<br>chloroallyl aldehyde<br>dehydrogenase/ aldehyde<br>dehydrogenase (NAD)/ coniferyl-<br>aldehyde dehydrogenase	Unigene10771_All//8.00E-<br>36//gi|13355011|gb|BG445359.1|BG445359		ko00940|Phenylpropanoid<br>biosynthesis//ko00010|Glycolysis /<br>Gluconeogenesis//ko00903|Limonene<br>and pinene<br>degradation//ko00620|Pyruvate<br>metabolism//ko00380|Tryptophan<br>metabolism//ko00071|Fatty acid<br>metabolism//ko00053|Ascorbate and<br>aldarate<br>metabolism//ko00330|Arginine and<br>proline<br>metabolism//ko00280|Valine,<br>leucine and isoleucine<br>degradation//ko00640|Propanoate<br>metabolism//ko00561|Glycerolipid<br>metabolism//ko00310|Lysine<br>degradation//ko00410|beta-Alanine<br>metabolism//ko00340|Histidine<br>metabolism	GO:0004030//GO:0009698	nr -
Unigene107727_All	211	13.39	12.49	34.83	30.22	23.01	29.32	1.60	2.18	1.89	-1.01E-01	7.77E-01		1.38E+00	4.50E-09	Up	-3.93E-01	9.17E-02		-4.34E-02	8.79E-01		4.43E-01	6.32E-01		2.36E-01	8.05E-01				Unigene107727_All//3.00E-37//TC163844				
Unigene107728_All	322	0.00	0.00	0.00	0.00	0.00	0.00	2.49	0.65	0.00	null	null		null	null		null	null		null	null		-1.94E+00	1.17E-02		-1.13E+01	4.43E-06	Down							ESTscan -
Unigene10774_All	490	0.11	0.41	0.98	1.63	6.32	4.89	0.09	0.09	0.00	1.95E+00	3.76E-01		3.20E+00	3.48E-02		1.96E+00	1.92E-07	Up	1.59E+00	2.41E-04	Up	-1.68E-02	1.01E+00		-6.43E+00	6.17E-01								
Unigene107743_All	926	0.00	0.00	0.00	0.11	0.00	0.20	7.08	0.54	1.12	null	null		null	null		-6.75E+00	3.17E-01		8.59E-01	6.96E-01		-3.71E+00	2.73E-32	Down	-2.66E+00	1.61E-24	Down	Unigene107743_All//sp|Q5VU43|MYOME<br>_HUMAN//7.00E-07//Myomegalin<br>OS=Homo sapiens GN=PDE4DIP PE=2<br>SV=1		Unigene107743_All//2.00E-125//TC161176				swissprot +
Unigene107753_All	1494	0.00	0.00	0.00	0.00	0.00	0.00	4.47	1.15	0.48	null	null		null	null		null	null		null	null		-1.96E+00	6.48E-17	Down	-3.22E+00	3.45E-30	Down	Unigene107753_All//gi|8928433|sp|Q<br>9ZSW1.1|TBB1_CYAPA//0.00E+00//RecN<br>ame: Full=Tubulin beta-1 chain;<br>AltName: Full=Beta-1-tubulin<br>>gi|4139172|gb|AAD03712.1| beta 1<br>tubulin [Cyanophora paradoxa]	Unigene107753_All//0.00E+00//AT5G1<br>2250| TUB6 (BETA-6 TUBULIN);<br>structural constituent of<br>cytoskeleton	Unigene107753_All//2.00E-43//TC130221		ko04145|Phagosome		nr +
Unigene107754_All	245	0.00	2.69	4.35	6.91	34.15	44.43	0.35	0.00	0.33	1.14E+01	6.26E-04	Up	1.21E+01	6.63E-06	Up	2.30E+00	8.18E-24	Up	2.68E+00	5.27E-34	Up	-8.43E+00	4.07E-01		-8.57E-02	9.74E-01		Unigene107754_All//gi|30725324|gb| AAP37684.1|//2.00E-13//At3g01680<br>[Arabidopsis thaliana]	Unigene107754_All//9.00E-<br>15//AT3G01680| unknown protein	Unigene107754_All//6.00E-101//TC134810				nr +
Unigene107757_All	360	0.00	0.00	0.00	0.00	0.00	0.00	3.29	1.04	0.44	null	null		null	null		null	null		null	null		-1.65E+00	5.03E-03		-2.89E+00	2.03E-05	Down	Unigene107757_All//gi|29539344|dbj<br>|BAC67673.1|//8.00E-32//ribosomal<br>protein S8 [Cyanidioschyzon<br>merolae]	Unigene107757_All//1.00E-<br>32//AT5G20290| 40S ribosomal<br>protein S8 (RPS8A)			ko03010|Ribosome		nr -
Unigene10776_All	223	0.00	6.59	1.19	0.00	0.31	0.00	0.00	0.00	0.00	1.27E+01	2.11E-08	Up	1.02E+01	8.03E-02		8.27E+00	6.15E-01		null	null		null	null		null	null								
Unigene107764_All	1104	0.09	0.00	0.10	0.63	0.47	0.16	4.02	0.57	0.14	-6.57E+00	3.76E-01		2.56E-02	1.00E+00		-4.33E-01	6.06E-01		-1.95E+00	3.91E-02		-2.82E+00	2.72E-17	Down	-4.80E+00	4.46E-27	Down	Unigene107764_All//gi|224069166|re<br>f|XP_002302916.1|//1.00E-104//GRAS<br>family protein [Populus<br>trichocarpa]<br>>gi|222844642|gb|EEE82189.1| GRAS<br>family protein [Populus<br>trichocarpa]	Unigene107764_All//3.00E-<br>89//AT4G08250| scarecrow<br>transcription factor family<br>protein	Unigene107764_All//0.00E+00//gi|21099408| gb|BQ411721.1|BQ411721	GRAS			nr -
Unigene10777_All	243	0.22	0.00	0.00	6.56	2.27	0.00	0.00	0.00	0.00	-7.75E+00	6.20E-01		-7.75E+00	6.32E-01		-1.53E+00	2.44E-03		-1.27E+01	5.79E-10	Down	null	null		null	null								
Unigene107771_All	222	0.00	0.00	0.00	0.00	0.00	0.00	2.29	0.38	0.00	null	null		null	null		null	null		null	null		-2.60E+00	2.05E-02		-1.12E+01	5.56E-04	Down							ESTscan +
Unigene107772_All	300	0.00	0.00	0.00	0.00	0.00	0.00	3.95	0.70	0.00	null	null		null	null		null	null		null	null		-2.50E+00	1.47E-04	Down	-1.19E+01	8.15E-09	Down							
Unigene107775_All	241	0.65	0.63	0.88	1.45	1.86	1.31	1.75	0.35	6.45	-4.64E-02	9.90E-01		4.40E-01	7.76E-01		3.60E-01	8.07E-01		-1.41E-01	9.12E-01		-2.34E+00	5.47E-02		1.88E+00	2.07E-04	Up	Unigene107775_All//gi|212717129|gb<br>|ACJ37406.1|//9.00E-13//stress-<br>induced receptor-like kinase<br>[Glycine max]	Unigene107775_All//7.00E-<br>05//AT4G32300| SD2-5 (S-DOMAIN-2<br>5); carbohydrate binding / kinase/<br>protein kinase	Unigene107775_All//3.00E-75//ES826968				nr +
Unigene107776_All	318	0.00	0.00	0.00	0.00	0.00	0.00	1.86	1.05	0.00	null	null		null	null		null	null		null	null		-8.24E-01	3.48E-01		-1.09E+01	1.42E-04	Down	Unigene107776_All//gi|192910756|gb<br>|ACF06486.1|//6.00E-35//60S<br>ribosomal protein L11 [Elaeis<br>guineensis]<br>>gi|192910758|gb|ACF06487.1| 60S<br>ribosomal protein L11 [Elaeis<br>guineensis]	Unigene107776_All//7.00E-<br>25//AT5G45775| 60S ribosomal<br>protein L11 (RPL11D)	Unigene107776_All//1.00E-09//TC154176		ko03010|Ribosome		nr -
Unigene107779_All	238	0.00	0.00	0.00	0.00	0.00	0.00	2.84	0.00	0.00	null	null		null	null		null	null		null	null		-1.15E+01	6.35E-05	Down	-1.15E+01	3.57E-05	Down							ESTscan +
Unigene107790_All	347	0.45	1.75	0.92	0.43	0.00	0.26	4.02	0.36	0.46	1.95E+00	6.04E-02		1.03E+00	4.71E-01		-8.75E+00	1.66E-01		-7.27E-01	7.17E-01		-3.48E+00	5.80E-07	Down	-3.13E+00	1.09E-06	Down			Unigene107790_All//1.00E-47//TC138513				
Unigene107793_All	231	0.00	0.00	0.00	0.00	0.00	0.00	2.93	0.54	0.00	null	null		null	null		null	null		null	null		-2.43E+00	7.77E-03		-1.15E+01	3.57E-05	Down	Unigene107793_All//gi|15224284|ref<br>|NP_181874.1|//4.00E-15//60S<br>ribosomal protein L38 (RPL38A)<br>[Arabidopsis thaliana]<br>>gi|15231705|ref|NP_191513.1| 60S<br>ribosomal protein L38 (RPL38B)<br>[Arabidopsis thaliana]<br>>gi|297820788|ref|XP_002878277.1| 60S ribosomal protein L38<br>[Arabidopsis lyrata subsp. lyrata]<br>>gi|297828045|ref|XP_002881905.1| 60S ribosomal protein L38<br>[Arabidopsis lyrata subsp. lyrata]<br>>gi|3122724|sp|O22860.1|RL38_ARATH<br>RecName: Full=60S ribosomal<br>protein L38<br>>gi|13605720|gb|AAK32853.1|AF36184<br>1_1 AT3g59540/T16L24_90<br>[Arabidopsis thaliana]<br>>gi|2289009|gb|AAB64338.1| 60S<br>ribosomal protein L38 [Arabidopsis<br>thaliana]<br>>gi|6996290|emb|CAB75451.1| 60S<br>RIBOSOMAL PROTEIN L38-like protein<br>[Arabidopsis thaliana]<br>>gi|21593879|gb|AAM65846.1| 60S<br>RIBOSOMAL PROTEIN L38-like protein<br>[Arabidopsis thaliana]<br>>gi|23507773|gb|AAN38690.1| At3g59540/T16L24_90 [Arabidopsis	Unigene107793_All//2.00E-<br>16//AT3G59540| 60S ribosomal<br>protein L38 (RPL38B)			ko03010|Ribosome		nr -
Unigene107803_All	446	0.00	0.00	0.00	0.00	0.00	0.00	12.13	3.09	0.98	null	null		null	null		null	null		null	null		-1.97E+00	6.32E-14	Down	-3.63E+00	3.14E-27	Down	Unigene107803_All//gi|18076136|emb<br>|CAC80486.1|//5.00E-49//cytochrome<br>oxidase subunit I [Musa acuminata]	Unigene107803_All//1.00E-<br>46//ATMG01360| cytochrome c<br>oxidase subunit 1	Unigene107803_All//3.00E-08//EX172657		ko00190|Oxidative phosphorylation		nr -
Unigene10781_All	470	0.45	2.48	1.36	5.51	3.44	2.79	0.81	0.71	0.34	2.48E+00	9.80E-04	Up	1.61E+00	1.11E-01		-6.79E-01	7.37E-02		-9.84E-01	1.20E-02		-1.87E-01	8.57E-01		-1.26E+00	2.48E-01		Unigene10781_All//gi|297846424|ref<br>|XP_002891093.1|//1.00E-<br>55//phosphatidylinositol-4-<br>phosphate 5-kinase family protein<br>[Arabidopsis lyrata subsp. lyrata]<br>>gi|297336935|gb|EFH67352.1| phosphatidylinositol-4-phosphate<br>5-kinase family protein<br>[Arabidopsis lyrata subsp. lyrata]	Unigene10781_All//9.00E-<br>44//AT1G34260| phosphatidylinositol-4-phosphate<br>5-kinase family protein	Unigene10781_All//5.00E-75//CA993882		ko04145|Phagosome//ko04144|Endocyt<br>osis//ko04070|Phosphatidylinositol<br>signaling system//ko00562|Inositol<br>phosphate metabolism	GO:0016307//GO:0019538//GO:0030384<br>//GO:0032559	nr +
Unigene107810_All	442	0.00	0.00	0.00	0.00	0.00	0.10	1.53	1.42	0.09	null	null		null	null		null	null		6.68E+00	7.03E-01		-1.10E-01	8.90E-01		-4.09E+00	3.10E-04	Down							
Unigene10782_All	546	0.00	0.00	0.00	0.00	0.00	0.00	2.01	2.37	0.00	null	null		null	null		null	null		null	null		2.37E-01	6.52E-01		-1.10E+01	3.33E-08	Down	Unigene10782_All//gi|297829134|ref<br>|XP_002882449.1|//6.00E-25//dual<br>specificity protein phosphatase<br>family protein [Arabidopsis lyrata<br>subsp. lyrata]<br>>gi|297328289|gb|EFH58708.1| dual<br>specificity protein phosphatase<br>family protein [Arabidopsis lyrata<br>subsp. lyrata]	Unigene10782_All//1.00E-<br>25//AT3G06110| MKP2 (MAPK<br>PHOSPHATASE 2); MAP kinase<br>phosphatase/ protein<br>tyrosine/serine/threonine<br>phosphatase				GO:0004721//GO:0005515//GO:0016020<br>//GO:0042221//GO:0043231//GO:00434<br>05	nr +
Unigene107820_All	409	0.00	0.00	0.00	0.24	0.00	0.00	1.65	0.10	0.39	null	null		null	null		-7.93E+00	3.17E-01		-7.93E+00	4.05E-01		-4.02E+00	5.36E-04	Down	-2.09E+00	1.27E-02								
Unigene107821_All	255	0.00	0.00	0.00	0.00	0.00	0.00	2.32	0.49	0.00	null	null		null	null		null	null		null	null		-2.24E+00	2.07E-02		-1.12E+01	1.42E-04	Down							
Unigene107826_All	296	0.53	0.17	0.90	2.19	0.23	0.00	0.86	0.00	0.00	-1.63E+00	4.89E-01		7.62E-01	6.44E-01		-3.23E+00	2.49E-03		-1.11E+01	3.90E-04	Down	-9.74E+00	4.06E-02		-9.74E+00	3.06E-02		Unigene107826_All//gi|73921149|sp| Q9LV66.1|Y5848_ARATH//6.00E-<br>06//RecName: Full=Uncharacterized<br>protein At5g48480	Unigene107826_All//2.00E-<br>07//AT5G48480| unknown protein	Unigene107826_All//1.00E-77//TC151304				nr +
Unigene107828_All	269	0.78	0.00	0.00	0.19	0.00	0.00	2.04	0.00	0.00	-9.60E+00	1.23E-01		-9.60E+00	1.47E-01		-7.53E+00	5.63E-01		-7.53E+00	6.45E-01		-1.10E+01	4.62E-04	Down	-1.10E+01	2.81E-04	Down							
Unigene10783_All	469	0.00	8.21	2.38	0.00	0.00	0.00	0.00	0.00	0.00	1.30E+01	6.33E-22	Up	1.12E+01	3.44E-06	Up	null	null		null	null		null	null		null	null								ESTscan +
Unigene107837_All	230	0.00	0.00	0.00	0.00	0.00	0.00	2.76	0.73	0.00	null	null		null	null		null	null		null	null		-1.92E+00	3.10E-02		-1.14E+01	7.12E-05	Down			Unigene107837_All//7.00E-17//TC147981				
Unigene107838_All	266	0.00	0.00	0.00	0.00	0.00	0.00	2.38	0.00	0.60	null	null		null	null		null	null		null	null		-1.12E+01	1.24E-04	Down	-1.99E+00	2.04E-02								
Unigene10784_All	367	7.41	3.45	2.47	4.75	2.81	1.85	1.84	1.14	1.63	-1.10E+00	4.26E-03		-1.59E+00	1.72E-04	Down	-7.55E-01	1.15E-01		-1.36E+00	6.97E-03		-6.95E-01	3.94E-01		-1.79E-01	7.94E-01				Unigene10784_All//8.00E-166//TC171472				
Unigene10785_All	343	0.00	0.30	0.00	0.00	0.00	0.00	0.74	2.80	5.58	8.21E+00	3.90E-01		null	null		null	null		null	null		1.92E+00	5.42E-03		2.91E+00	3.25E-08	Up							
Unigene107856_All	410	0.00	0.00	0.00	0.00	0.00	0.00	1.75	0.41	0.19	null	null		null	null		null	null		null	null		-2.10E+00	1.25E-02		-3.17E+00	8.09E-04	Down	Unigene107856_All//gi|300259453|gb<br>|EFJ43680.1|//2.00E-17//component<br>of cytosolic 80S ribosome and 40S<br>small subunit [Volvox carteri f.<br>nagariensis]	Unigene107856_All//2.00E-<br>14//AT4G25740| 40S ribosomal<br>protein S10 (RPS10A)			ko03010|Ribosome		nr -
Unigene10786_All	360	2.03	1.41	1.18	1.11	0.19	1.51	0.23	6.85	2.77	-5.32E-01	5.13E-01		-7.82E-01	3.80E-01		-2.53E+00	5.80E-02		4.44E-01	6.78E-01		4.87E+00	1.04E-14	Up	3.56E+00	2.43E-05	Up	Unigene10786_All//gi|297804080|ref<br>|XP_002869924.1|//1.00E-25//FAD-<br>binding domain-containing protein<br>[Arabidopsis lyrata subsp. lyrata]<br>>gi|297315760|gb|EFH46183.1| FAD-<br>binding domain-containing protein<br>[Arabidopsis lyrata subsp. lyrata]	Unigene10786_All//1.00E-<br>22//AT4G20840| FAD-binding domain-<br>containing protein	Unigene10786_All//3.00E-23//TC163182			GO:0005576//GO:0005618//GO:0006950<br>//GO:0016020//GO:0016491//GO:00432<br>31	nr -

Unigene10787_All	402	0.00	0.00	0.00	0.00	0.00	0.00	3.37	3.64	0.20	null	null		null	null		null	null		null	null		1.13E-01	8.23E-01		-4.09E+00	7.07E-08	Down	Unigene10787_All//gi|195619738|gb|ACG31699.1|//5.00E-32//60S ribosomal protein L4 [Zea mays]	Unigene10787_All//6.00E-28//AT3G09630| 60S ribosomal protein L4/L1 (RPL4A)			ko03010|Ribosome	GO:0005622	nr -
Unigene107876_All	388	0.00	0.00	0.00	0.00	0.00	0.00	1.85	1.72	0.00	null	null		null	null		null	null		null	null		-1.04E-01	8.93E-01		-1.09E+01	1.78E-05	Down			Unigene107876_All//6.00E-145//ES842468				
Unigene107882_All	228	0.00	0.00	0.00	0.00	0.00	0.00	6.49	1.47	8.56	null	null		null	null		null	null		null	null		-2.15E+00	9.69E-05	Down	4.00E-01	3.41E-01		Unigene107882_All//gi|125887|sp|P13447.1|LAT52_SOLLC//2.00E-10//RecName: Full=Anther-specific protein LAT52; Flags: Precursor >gi|295812|emb|CAA33854.1| LAT52 [Solanum lycopersicum]	Unigene107882_All//1.00E-08//AT4G18596| pollen Ole e 1 allergen and extensin family protein	Unigene107882_All//5.00E-67//TC149258				nr +
Unigene107889_All	237	0.00	0.00	0.00	3.15	1.60	0.19	1.07	0.71	0.67	null	null		null	null		-9.80E-01	2.23E-01		-4.05E+00	7.76E-04	Down	-6.02E-01	6.48E-01		-6.71E-01	6.06E-01								
Unigene10789_All	311	0.00	15.31	11.30	0.00	0.00	0.00	0.00	0.00	0.00	1.39E+01	3.83E-27	Up	1.35E+01	1.87E-19	Up	null	null		null	null		null	null		null	null		Unigene10789_All//gi|1173043|sp|P46291.1|RL38_SOLLC//4.00E-25//RecName: Full=60S ribosomal protein L38 >gi|313027|emb|CAA49599.1| ribosomal protein L38 [Solanum lycopersicum] >gi|77745467|gb|ABB02632.1| ribosomal protein L38-like [Solanum tuberosum]	Unigene10789_All//2.00E-15//AT3G59540| 60S ribosomal protein L38 (RPL38B)			ko03010|Ribosome	GO:0005198//GO:0010467//GO:0015934//GO:0022613	nr +
Unigene107893_All	247	0.00	0.00	0.00	0.20	0.00	0.00	2.05	0.00	0.97	null	null		null	null		-7.66E+00	5.63E-01		-7.66E+00	6.45E-01		-1.10E+01	8.91E-04	Down	-1.09E+00	2.28E-01		Unigene107893_All//gi|4206110|gb|AAD11424.1|//5.00E-07//cytochrome [Mesembryanthemum crystallinum]						nr -
Unigene107897_All	448	0.00	0.00	0.00	0.00	0.00	0.00	1.60	0.00	0.09	null	null		null	null		null	null		null	null		-1.06E+01	3.27E-05	Down	-4.17E+00	1.65E-04	Down							ESTscan -
Unigene1079_All	2873	29.37	22.50	24.15	29.67	19.37	14.43	74.41	71.01	26.75	-3.84E-01	8.50E-12		-2.83E-01	1.27E-06		-6.15E-01	8.92E-33		-1.04E+00	3.27E-71	Down	-6.74E-02	5.16E-02		-1.48E+00	0.00E+00	Down	Unigene1079_All//gi|187940724|gb|ACD39463.1|//1.00E-170//BEL30 protein [Solanum etuberosum]	Unigene1079_All//5.00E-117//AT4G34610| BLH6 (BELL1-LIKE HOMEODOMAIN 6); DNA binding / transcription factor	Unigene1079_All//0.00E+00//TC144172	HB		GO:0001071//GO:0003677//GO:0006351//GO:0010468//GO:0043231	nr +
Unigene107904_All	393	0.00	0.00	0.14	0.00	0.00	0.00	2.15	0.21	0.20	null	null		7.08E+00	6.41E-01		null	null		null	null		-3.34E+00	2.52E-04	Down	-3.41E+00	1.35E-04	Down							
Unigene107918_All	273	0.00	0.00	0.00	0.18	0.00	0.33	1.86	0.00	0.15	null	null		null	null		-7.51E+00	5.63E-01		8.59E-01	7.97E-01		-1.09E+01	8.91E-04	Down	-3.67E+00	3.75E-03				Unigene107918_All//8.00E-119//TC143380				
Unigene107919_All	238	0.00	0.00	0.00	0.00	0.14	0.00	3.02	0.18	0.67	null	null		null	null		7.18E+00	8.38E-01		null	null		-4.10E+00	2.91E-04	Down	-2.17E+00	7.76E-03								
Unigene107926_All	633	0.25	0.24	0.34	0.47	0.27	0.00	1.67	0.99	0.19	-4.67E-02	9.90E-01		4.40E-01	7.76E-01		-7.96E-01	5.57E-01		-8.88E+00	4.01E-02		-7.54E-01	2.17E-01		-3.14E+00	2.97E-05	Down	Unigene107926_All//gi|108864366|gb|ABA93633.2|//8.00E-08//Protein kinase domain containing protein, expressed [Oryza sativa (japonica cultivar-group)]						nr +
Unigene107934_All	529	0.00	0.00	0.20	0.75	0.07	0.17	2.24	0.40	0.30	null	null		7.65E+00	4.02E-01		-3.53E+00	2.10E-02		-2.14E+00	1.26E-01		-2.50E+00	1.47E-04	Down	-2.89E+00	2.03E-05	Down							
Unigene10794_All	358	1.02	5.94	5.06	0.14	1.25	0.38	1.54	0.58	0.00	2.54E+00	2.15E-06	Up	2.31E+00	8.14E-05	Up	3.17E+00	3.95E-02		1.44E+00	6.39E-01		-1.40E+00	1.35E-01		-1.06E+01	2.81E-04	Down		Unigene10794_All//3.00E-05//AT3G53880| aldo/keto reductase family protein	Unigene10794_All//3.00E-14//gi|48805189|gb|CO106503.1|CO106503				
Unigene107942_All	296	0.00	0.00	0.00	0.00	0.00	0.00	2.14	0.00	0.13	null	null		null	null		null	null		null	null		-1.11E+01	1.24E-04	Down	-3.99E+00	5.83E-04	Down							
Unigene107947_All	224	0.70	0.00	0.00	1.78	0.46	0.00	2.83	0.19	0.18	-9.45E+00	2.17E-01		-9.45E+00	2.46E-01		-1.95E+00	1.19E-01		-1.08E+01	1.11E-02		-3.92E+00	9.81E-04	Down	-3.99E+00	5.83E-04	Down							
Unigene10795_All	445	0.35	0.00	0.00	5.49	4.26	0.91	1.24	0.19	0.18	-8.46E+00	2.17E-01		-8.46E+00	2.46E-01		-3.66E-01	3.91E-01		-2.59E+00	6.96E-08	Down	-2.72E+00	1.22E-02		-2.79E+00	7.97E-03		Unigene10795_All//gi|122209356|sp|Q2V3I3.1|EPFL4_ARATH//3.00E-26//RecName: Full=EPIDERMAL PATTERNING FACTOR-like protein 4; Short=EPF-like protein 4; Flags: Precursor	Unigene10795_All//1.00E-14//AT3G22820| allergen-related	Unigene10795_All//0.00E+00//CO071488			GO:0009791//GO:0009913	nr -
Unigene107956_All	336	12.77	7.54	16.48	23.42	23.78	17.88	34.48	13.68	15.41	-7.60E-01	9.33E-03		3.68E-01	1.77E-01		2.15E-02	9.45E-01		-3.90E-01	6.96E-02		-1.33E+00	7.46E-17	Down	-1.16E+00	3.51E-14	Down	Unigene107956_All//gi|47026989|gb|AAT08712.1|//9.00E-13//nucleoside diphosphate kinase [Hyacinthus orientalis]	Unigene107956_All//9.00E-13//AT4G09320| NDPK1; ATP binding / nucleoside diphosphate kinase	Unigene107956_All//6.00E-74//gi|109828864|gb|DW227483.1|DW227483		ko00230|Purine metabolism//ko00240|Pyrimidine metabolism		nr -
Unigene107957_All	551	0.00	0.00	0.00	0.45	0.06	0.00	2.46	0.76	0.58	null	null		null	null		-2.85E+00	1.37E-01		-8.82E+00	7.36E-02		-1.69E+00	1.96E-03		-2.09E+00	1.85E-04	Down	Unigene107957_All//gi|15225548|ref|NP_182094.1|//5.00E-79//sterile alpha motif (SAM) domain-containing protein [Arabidopsis thaliana]	Unigene107957_All//3.00E-80//AT2G45700| sterile alpha motif (SAM) domain-containing protein	Unigene107957_All//0.00E+00//TC179553				nr -
Unigene107962_All	733	0.00	0.00	0.00	0.00	0.00	0.00	7.15	0.11	0.76	null	null		null	null		null	null		null	null		-5.97E+00	7.48E-34	Down	-3.23E+00	6.29E-24	Down	Unigene107962_All//gi|115474639|ref|NP_001060916.1|//4.00E-46//Os08g0129600 [Oryza sativa Japonica Group] >gi|29467527|dbj|BAC66716.1| proline-rich protein family-like [Oryza sativa Japonica Group] >gi|113622885|dbj|BAF22830.1| Os08g0129600 [Oryza sativa Japonica Group]	Unigene107962_All//3.00E-36//AT3G25690| CHUP1 (CHLOROPLAST UNUSUAL POSITIONING 1)	Unigene107962_All//1.00E-70//TC132881				nr -
Unigene107984_All	249	0.00	0.00	0.00	0.60	0.55	0.00	3.40	0.17	0.16	null	null		null	null		-1.18E-01	9.11E-01		-9.23E+00	2.35E-01		-4.34E+00	4.56E-05	Down	-4.41E+00	2.37E-05	Down			Unigene107984_All//8.00E-51//TC140956				
Unigene107989_All	624	0.00	0.00	0.00	0.00	0.00	0.00	2.44	0.60	0.13	null	null		null	null		null	null		null	null		-2.02E+00	1.51E-04	Down	-4.26E+00	5.22E-09	Down			Unigene107989_All//9.00E-19//TC142576				
Unigene10799_All	266	0.00	4.38	2.80	0.00	0.00	0.00	0.00	0.00	0.00	1.21E+01	1.05E-06	Up	1.15E+01	3.28E-04	Up	null	null		null	null		null	null		null	null								
Unigene107998_All	448	0.00	0.00	0.00	0.00	0.00	0.00	1.51	0.00	0.09	null	null		null	null		null	null		null	null		-1.06E+01	6.35E-05	Down	-4.09E+00	3.10E-04	Down	Unigene107998_All//gi|222874561|gb|EEF11692.1|//5.00E-09//predicted protein [Populus trichocarpa]						nr +
Unigene10801_All	354	0.00	10.59	6.92	0.00	0.00	0.00	0.00	0.00	0.00	1.34E+01	2.40E-21	Up	1.28E+01	1.64E-13	Up	null	null		null	null		null	null		null	null		Unigene10801_All//gi|255089517|ref|XP_002506680.1|//4.00E-33//ribosomal protein L37ae [Micromonas sp. RCC299] >gi|226457652|gb|EEH54951.1| ribosomal protein L37ae [Micromonas pusilla CCMP1545] >gi|226521953|gb|AC067938.1| ribosomal protein L37ae [Micromonas sp. RCC299]	Unigene10801_All//2.00E-33//AT3G60245| 60S ribosomal protein L37a (RPL37aC)			ko03010|Ribosome	GO:0005198//GO:0010467//GO:0015934//GO:0022613//GO:0043169//GO:0043231	nr -
Unigene108015_All	404	0.00	0.00	0.00	0.25	0.26	0.00	5.02	1.14	1.08	null	null		null	null		5.23E-02	9.68E-01		-7.95E+00	4.05E-01		-2.14E+00	3.19E-06	Down	-2.21E+00	1.05E-06	Down	Unigene108015_All//gi|224112451|ref|XP_002332774.1|//3.00E-09//cc-nbs-lrr resistance protein [Populus trichocarpa] >gi|222834669|gb|EEE73132.1| cc-nbs-lrr resistance protein [Populus trichocarpa]						nr -
Unigene108016_All	458	0.00	0.00	0.00	0.00	0.00	0.00	1.94	0.09	0.17	null	null		null	null		null	null		null	null		-4.41E+00	2.44E-05	Down	-3.48E+00	7.34E-05	Down	Unigene108016_All//gi|300267685|gb|EFJ51868.1|//9.00E-14//dynein heavy chain 4 [Volvox carteri f. nagariensis]				ko04141|Protein processing in endoplasmic reticulum//ko03420|Nucleotide excision repair		nr -
Unigene108017_All	449	0.00	0.00	0.00	0.11	0.00	0.00	5.08	3.54	0.62	null	null		null	null		-6.79E+00	5.63E-01		-6.79E+00	6.45E-01		-5.24E-01	1.77E-01		-3.03E+00	3.22E-10	Down			Unigene108017_All//6.00E-22//TC149487				
Unigene108037_All	425	0.00	0.00	0.00	0.00	0.00	0.00	1.89	0.49	0.09	null	null		null	null		null	null		null	null		-1.94E+00	1.17E-02		-4.33E+00	4.54E-05	Down	Unigene108037_All//gi|9979193|sp|Q9ZRX0.1|TCTP_PSEMZ//4.00E-06//RecName: Full=Translationally-controlled tumor protein homolog; Short=TCTP >gi|3850844|emb|CAA10048.1| TCTP-like protein [Pseudotsuga menziesii]	Unigene108037_All//3.00E-05//AT3G16640| TCTP (TRANSLATIONALLY CONTROLLED TUMOR PROTEIN)					nr -
Unigene108040_All	461	0.23	0.00	0.23	0.00	0.75	0.20	1.65	0.18	0.95	-7.83E+00	3.76E-01		2.52E-02	1.00E+00		9.55E+00	3.02E-02		7.61E+00	4.81E-01		-3.19E+00	7.93E-04	Down	-7.96E-01	2.52E-01		Unigene108040_All//gi|145358569|ref|NP_198529.3|//1.00E-17//binding [Arabidopsis thaliana]	Unigene108040_All//7.00E-14//AT5G37130| binding	Unigene108040_All//2.00E-163//TC134980				nr -
Unigene108046_All	383	0.14	1.19	0.56	0.13	0.18	0.00	0.33	0.11	2.39	3.12E+00	3.58E-02		2.03E+00	3.62E-01		4.67E-01	9.61E-01		-7.02E+00	6.45E-01		-1.60E+00	5.35E-01		2.85E+00	3.18E-04	Up	Unigene108046_All//gi|15222311|ref|NP_172195.1|//1.00E-18//MAPKKK13; ATP binding / kinase/ protein kinase/ protein serine/threonine kinase [Arabidopsis thaliana] >gi|8954031|gb|AAF82205.1|AC067971_13 Strong similarity to a protein kinase homolog F23F1.4 gi|7488253 from Arabidopsis thaliana BAC F23F1 gb|AC004680. It contains a eukaryotic protein kinase domain PF|00069. ESTs gb|F13903 and gb|F13904 come from this gene	Unigene108046_All//6.00E-20//AT1G07150| MAPKKK13; ATP binding / kinase/ protein kinase/ protein serine/threonine kinase	Unigene108046_All//2.00E-142//TC161841				nr +
Unigene108050_All	491	0.00	0.00	0.00	0.00	0.00	0.00	5.34	0.26	0.65	null	null		null	null		null	null		null	null		-4.39E+00	8.13E-15	Down	-3.04E+00	1.19E-11	Down	Unigene108050_All//gi|226496173|ref|NP_001152735.1|//7.00E-06//phytosulfokines 4 [Zea mays] >gi|195659479|gb|ACG49207.1| phytosulfokines 4 precursor [Zea mays]	Unigene108050_All//2.00E-05//AT3G49780| ATPSK4 (PHYTOSULFOKINE 4 PRECURSOR); growth factor					nr -
Unigene108057_All	300	0.00	0.00	0.00	0.33	0.00	0.00	6.34	5.85	0.66	null	null		null	null		-8.38E+00	3.17E-01		-8.38E+00	4.05E-01		-1.16E-01	7.89E-01		-3.26E+00	3.59E-09	Down			Unigene108057_All//1.00E-19//TC134836				
Unigene108058_All	224	0.00	0.00	0.00	0.00	0.00	0.20	3.02	1.68	0.00	null	null		null	null		null	null		7.66E+00	7.03E-01		-8.47E-01	2.88E-01		-1.16E+01	3.57E-05	Down			Unigene108058_All//3.00E-07//EX169661				
Unigene10806_All	231	0.00	3.29	4.38	0.00	0.00	0.00	0.00	0.00	0.00	1.17E+01	1.77E-04	Up	1.21E+01	1.28E-05	Up	null	null		null	null		null	null		null	null		Unigene10806_All//sp|O00322|UPK1A_HUMAN//1.00E-12//Uroplakin-1a OS=Homo sapiens GN=UPK1A PE=2 SV=1						swissprot +
Unigene108067_All	335	0.00	0.00	0.00	0.00	0.00	0.00	2.15	0.00	0.36	null	null		null	null		null	null		null	null		-1.11E+01	3.27E-05	Down	-2.59E+00	2.81E-03		Unigene108067_All//sp|Q8BP40|PPA6_MOUSE//3.00E-06//Lysophosphatidic acid phosphatase type 6 OS=Mus musculus GN=Acp6 PE=2 SV=1						swissprot +
Unigene108076_All	333	0.00	0.00	0.00	0.00	0.00	0.00	2.67	0.38	0.00	null	null		null	null		null	null		null	null		-2.82E+00	5.65E-04	Down	-1.14E+01	1.10E-06	Down			Unigene108076_All//4.00E-07//gi|48746449|gb|CO076968.1|CO076968				
Unigene108087_All	294	0.00	0.00	0.00	0.00	0.00	0.00	2.01	0.85	0.00	null	null		null	null		null	null		null	null		-1.24E+00	1.54E-01		-1.10E+01	1.42E-04	Down	Unigene108087_All//gi|223994173|ref|XP_002286770.1|//4.00E-10//udp-d-glucose 6-dehydrogenase [Thalassiosira pseudonana CCMP1335] >gi|220978085|gb|EED96411.1| udp-d-glucose 6-dehydrogenase [Thalassiosira pseudonana CCMP1335]				ko00500|Starch and sucrose metabolism//ko00520|Amino sugar and nucleotide sugar metabolism//ko00053|Ascorbate and aldarate metabolism//ko00040|Pentose and glucuronate interconversions		nr -
Unigene108089_All	428	0.00	0.00	0.00	0.00	0.00	0.00	2.57	0.29	0.00	null	null		null	null		null	null		null	null		-3.13E+00	3.47E-05	Down	-1.13E+01	3.33E-08	Down	Unigene108089_All//gi|297829496|ref|XP_002882630.1|//1.00E-21//60S ribosomal protein L4/L1 [Arabidopsis lyrata subsp. lyrata] >gi|297328470|gb|EFH58889.1| 60S ribosomal protein L4/L1 [Arabidopsis lyrata subsp. lyrata]	Unigene108089_All//4.00E-22//AT3G09630| 60S ribosomal protein L4/L1 (RPL4A)			ko03010|Ribosome		nr -
Unigene10809_All	356	0.00	0.00	0.00	0.00	0.00	0.00	9.74	6.69	0.22	null	null		null	null		null	null		null	null		-5.41E-01	7.19E-02		-5.44E+00	1.52E-22	Down			Unigene10809_All//8.00E-27//gi|109850443|gb|DW479422.1|DW479422				
Unigene108104_All	595	0.18	0.94	0.54	0.25	0.17	0.15	3.41	1.19	0.67	2.41E+00	3.93E-02		1.61E+00	3.05E-01		-5.33E-01	7.86E-01		-7.27E-01	7.17E-01		-1.51E+00	3.50E-04	Down	-2.35E+00	3.80E-07	Down							
Unigene108108_All	662	0.00	0.00	0.00	0.00	0.00	0.00	6.20	1.77	0.00	null	null		null	null		null	null		null	null		-1.81E+00	1.21E-09	Down	-1.26E+01	3.74E-30	Down			Unigene108108_All//2.00E-35//TC175328				
Unigene108114_All	262	30.75	15.08	12.40	19.96	13.80	12.24	2.10	74.98	0.61	-1.03E+00	8.45E-07	Down	-1.31E+00	3.61E-09	Down	-5.33E-01	3.49E-02		-7.06E-01	6.29E-03		5.16E+00	3.33E-118	Up	-1.79E+00	5.07E-02		Unigene108114_All//gi|146148631|gb|ABQ02258.1|//4.00E-34//O-glucosyltransferase 3 [Vitis labrusca]	Unigene108114_All//2.00E-28//AT1G05560| UGT75B1 (UDP-GLUCOSYLTRANSFERASE 75B1); UDP-glucose:4-aminobenzoate acylglucosyltransferase/ UDP-glucosyltransferase/ UDP-glycosyltransferase/ abscisic acid glucosyltransferase/ transferase, transferring glycosyl groups	Unigene108114_All//9.00E-131//gi|109838326|gb|DW236887.1|DW236887		ko00940|Phenylpropanoid biosynthesis//ko00380|Tryptophan metabolism//ko00908|Zeatin biosynthesis//ko00966|Glucosinolate biosynthesis//ko00942|Anthocyanin biosynthesis		nr -

Unigene10812_All	332	0.00	6.72	4.49	0.00	0.00	0.00	0.00	0.00	0.00	1.27E+01	1.09E-12	Up	1.21E+01	3.22E-08	Up	null	null		null	null		null	null		null	null		Unigene10812_All//gi|297600424|ref|NP_001049175.2|//2.00E-14//Os03g0182700 [Oryza sativa Japonica Group] >gi|23396617|sp|Q94HF1.1|EIF3K_ORYSJ RecName: Full=Eukaryotic translation initiation factor 3 subunit K; Short=eIF3k; AltName: Full=eIF-3 p25 >gi|15217293|gb|AAK92637.1|AC079633_17 Unknown protein [Oryza sativa Japonica Group] >gi|108706532|gb|ABF94327.1| Eukaryotic translation initiation factor 3 subunit 11 family protein, expressed [Oryza sativa (japonica cultivar-group)] >gi|255674253|dbj|BAF11089.2| Os03g0182700 [Oryza sativa Japonica Group]	Unigene10812_All//1.00E-10//AT4G33250| EIF3K (EUKARYOTIC TRANSLATION INITIATION FACTOR 3K); translation initiation factor					nr +
Unigene108122_All	397	0.00	0.00	0.00	0.00	0.00	0.00	1.28	0.42	0.00	null	null		null	null		null	null		null	null		-1.60E+00	1.07E-01		-1.03E+01	5.56E-04	Down	Unigene108122_All//gi|225685853|dbj|BAH30213.1|//2.00E-30//methionine S-adenosyl transferase [Cylindrotheca closterium]	Unigene108122_All//4.00E-29//AT2G36880| MAT3 (methionine adenosyltransferase 3); copper ion binding / methionine adenosyltransferase	Unigene108122_All//5.00E-07//ES821717		ko00270|Cysteine and methionine metabolism//ko00450|Selenoamino acid metabolism		nr -
Unigene108123_All	405	1.03	0.00	0.26	0.49	0.34	1.56	3.45	0.10	0.20	-1.00E+01	1.05E-02		-1.97E+00	1.51E-01		-5.33E-01	7.49E-01		1.67E+00	9.80E-02		-5.06E+00	1.10E-08	Down	-4.13E+00	3.70E-08	Down	Unigene108123_All//gi|224101205|ref|XP_002312184.1|//1.00E-42//chromatin remodeling complex subunit [Populus trichocarpa] >gi|222852004|gb|EEE89551.1| chromatin remodeling complex subunit [Populus trichocarpa]	Unigene108123_All//6.00E-35//AT3G42670| CHR38 (CHROMATIN REMODELING 38); ATP binding / DNA binding / helicase/ nucleic acid binding					nr +
Unigene108131_All	949	0.00	0.00	0.00	0.00	0.00	0.00	2.76	0.18	0.17	null	null		null	null		null	null		null	null		-3.97E+00	6.69E-14	Down	-4.04E+00	1.31E-14	Down	Unigene108131_All//gi|301121570|ref|XP_002908512.1|//1.00E-39//40S ribosomal protein SA [Phytophthora infestans T30-4] >gi|262103543|gb|EEY61595.1| 40S ribosomal protein SA [Phytophthora infestans T30-4]	Unigene108131_All//6.00E-32//AT1G72370| P40; structural constituent of ribosome	Unigene108131_All//3.00E-07//gi|109823896|gb|DW008744.1|DW008744		ko03010|Ribosome		nr -
Unigene108132_All	368	0.00	0.00	0.00	0.00	0.00	0.00	3.10	0.00	0.43	null	null		null	null		null	null		null	null		-1.16E+01	3.89E-08	Down	-2.84E+00	3.60E-05	Down	Unigene108132_All//gi|300266605|gb|EFJ50792.1|//5.00E-34//component of cytosolic 80S ribosome and 60S large subunit [Volvox carteri f. nagariensis]	Unigene108132_All//9.00E-34//AT1G77940| 60S ribosomal protein L30 (RPL30B)			ko03010|Ribosome		nr +
Unigene108136_All	224	0.00	0.00	0.00	0.00	0.00	0.00	2.45	0.56	0.00	null	null		null	null		null	null		null	null		-2.13E+00	3.32E-02		-1.13E+01	2.81E-04	Down	Unigene108136_All//gi|74272607|gb|ABA01099.1|//9.00E-18//40S ribosomal protein S3 [Chlamydomonas incerta]	Unigene108136_All//3.00E-10//AT2G31610| 40S ribosomal protein S3 (RPS3A)			ko03010|Ribosome		nr -
Unigene108137_All	257	0.00	0.00	0.00	0.00	0.00	0.00	1.97	0.49	0.00	null	null		null	null		null	null		null	null		-2.02E+00	5.21E-02		-1.09E+01	5.56E-04	Down	Unigene108137_All//gi|116061096|emb|CAL56484.1|//4.00E-31//GTP-binding regulatory protein beta chain homolog-Ch (ISS) [Ostreococcus tauri]	Unigene108137_All//2.00E-28//AT1G48630| RACK1B_AT (RECEPTOR FOR ACTIVATED C KINASE 1 B); nucleotide binding					nr +
Unigene10814_All	216	0.00	5.16	1.97	0.00	0.00	0.00	0.00	0.00	0.00	1.23E+01	2.00E-06	Up	1.09E+01	1.38E-02		null	null		null	null		null	null		null	null		Unigene10814_All//gi|225469312|ref|XP_002267662.1|//6.00E-06//PREDICTED: similar to L-idonate dehydrogenase [Vitis vinifera]	Unigene10814_All//4.00E-07//AT5G51970| sorbitol dehydrogenase, putative / L-iditol 2-dehydrogenase, putative			ko00051|Fructose and mannose metabolism	GO:0008152//GO:0016491//GO:0046914	nr -
Unigene108140_All	569	0.00	0.00	0.00	0.00	0.00	0.00	1.86	0.07	0.00	null	null		null	null		null	null		null	null		-4.66E+00	1.94E-06	Down	-1.09E+01	6.73E-08	Down	Unigene108140_All//gi|189081811|sp|A8CEP3.1|CALM_SACJA//7.00E-44//RecName: Full=Calmodulin; Short=CaM >gi|157888809|dbj|BAF80878.1| calmodulin [Saccharina japonica] >gi|298713212|emb|CBJ33511.1| Calcium-binding protein [Ectocarpus siliculosus]	Unigene108140_All//2.00E-43//AT2G27030| CAM5 (CALMODULIN 5); calcium ion binding	Unigene108140_All//3.00E-15//CD485888		ko04626|Plant-pathogen interaction//ko04070|Phosphatidylinositol signaling system		nr -
Unigene108142_All	419	0.00	0.00	0.00	0.00	0.00	0.00	1.92	0.20	0.76	null	null		null	null		null	null		null	null		-3.26E+00	4.49E-04	Down	-1.33E+00	5.40E-02				Unigene108142_All//6.00E-28//gi|48805733|gb|CO107047.1|CO107047				
Unigene10815_All	330	1.59	11.52	4.68	1.96	4.38	5.20	5.12	6.84	12.80	2.86E+00	2.76E-12	Up	1.56E+00	7.60E-03		1.16E+00	3.97E-02		1.41E+00	6.85E-03		4.16E-01	3.00E-01		1.32E+00	9.76E-07	Up	Unigene10815_All//gi|297792833|ref|XP_002864301.1|//4.00E-09//homogentisate 1,2-dioxygenase [Arabidopsis lyrata subsp. lyrata] >gi|297310136|gb|EFH40560.1| homogentisate 1,2-dioxygenase [Arabidopsis lyrata subsp. lyrata]	Unigene10815_All//2.00E-10//AT5G54080| HGO (HOMOGENTISATE 1,2-DIOXYGENASE); homogentisate 1,2-dioxygenase	Unigene10815_All//5.00E-59//TC155472		ko00350|Tyrosine metabolism		nr +
Unigene108164_All	540	0.00	0.00	0.00	0.00	0.00	0.00	6.89	16.56	1.18	null	null		null	null		null	null		null	null		1.27E+00	3.27E-12	Up	-2.55E+00	1.37E-13	Down	Unigene108164_All//gi|238014838|gb|ACR38454.1|//6.00E-15//unknown [Zea mays]		Unigene108164_All//6.00E-38//TC146753				nr -
Unigene10817_All	323	1.94	0.63	16.65	3.08	0.21	0.00	0.26	0.00	0.00	-1.63E+00	9.23E-02		3.10E+00	6.25E-18	Up	-3.85E+00	1.61E-05	Down	-1.16E+01	2.97E-06	Down	-8.03E+00	4.07E-01		-8.03E+00	3.61E-01		Unigene10817_All//gi|5814093|gb|AAD52097.1|AF088885_1//3.00E-19//receptor-like kinase CHRK1 [Nicotiana tabacum]	Unigene10817_All//1.00E-15//AT4G19810| glycosyl hydrolase family 18 protein				GO:0004553//GO:0004672//GO:0006026//GO:0006464//GO:0030312//GO:0032559//GO:0043167	nr -
Unigene108171_All	514	0.00	0.00	0.00	0.00	0.00	0.00	3.04	1.79	0.47	null	null		null	null		null	null		null	null		-7.67E-01	1.07E-01		-2.71E+00	1.57E-06	Down	Unigene108171_All//gi|171452356|dbj|BAG15869.1|//2.00E-33//ankyrin repeat protein [Bruguiera gymnorhiza]	Unigene108171_All//2.00E-22//AT1G14480| protein binding					nr -
Unigene108186_All	275	0.00	0.00	0.00	0.18	0.00	0.00	2.46	0.00	0.14	null	null		null	null		-7.50E+00	5.63E-01		-7.50E+00	6.45E-01		-1.13E+01	6.34E-05	Down	-4.09E+00	3.10E-04	Down			Unigene108186_All//3.00E-07//TC175182				
Unigene108195_All	309	218.77	91.34	151.13	158.47	229.23	100.70	0.14	0.27	0.26	-1.26E+00	6.18E-71	Down	-5.34E-01	3.13E-16		5.33E-01	1.29E-20		-6.54E-01	7.21E-19		9.84E-01	7.33E-01		9.15E-01	7.51E-01		Unigene108195_All//gi|6959488|gb|AAF33112.1|AF181683_1//4.00E-38//RPT2 [Arabidopsis thaliana]	Unigene108195_All//2.00E-39//AT2G30520| RPT2 (ROOT PHOTOTROPISM 2); protein binding	Unigene108195_All//2.00E-95//EX168571				nr -
Unigene1082_All	466	4.04	0.98	3.20	4.49	6.80	4.94	2.09	1.97	1.28	-2.05E+00	1.17E-04	Down	-3.37E-01	5.04E-01		5.99E-01	8.91E-02		1.39E-01	7.56E-01		-8.08E-02	9.06E-01		-7.02E-01	2.43E-01		Unigene1082_All//gi|5733885|gb|AAD49773.1|AC007932_21//1.00E-25//ESTs gb|T22141 and gb|H37217 come from this gene [Arabidopsis thaliana]	Unigene1082_All//6.00E-12//AT3G17430| phosphate translocator-related	Unigene1082_All//1.00E-152//TC140046			GO:0031224//GO:0031410	nr -
Unigene10820_All	328	6.06	10.81	4.55	9.87	8.61	8.67	11.60	7.52	5.47	8.35E-01	1.08E-02		-4.15E-01	3.93E-01		-1.98E-01	6.03E-01		-1.87E-01	6.20E-01		-6.26E-01	2.59E-02		-1.09E+00	8.46E-05	Down			Unigene10820_All//7.00E-55//gi|109828667|gb|DW227288.1|DW227288				
Unigene108208_All	495	0.00	0.00	0.00	0.00	0.00	0.00	4.01	0.25	0.80	null	null		null	null		null	null		null	null		-3.99E+00	1.09E-10	Down	-2.32E+00	6.40E-07	Down	Unigene108208_All//gi|224000567|ref|XP_002289956.1|//3.00E-60//ubiquitin-conjugating enzyme e2-16kda, ubiquitin protein ligase [Thalassiosira pseudonana CCMP1335] >gi|220975164|gb|EED93493.1| ubiquitin-conjugating enzyme e2-16kda, ubiquitin protein ligase [Thalassiosira pseudonana CCMP1335]	Unigene108208_All//2.00E-46//AT5G56150| UBC30 (ubiquitin-conjugating enzyme 30); ubiquitin-protein ligase			ko04141|Protein processing in endoplasmic reticulum//ko04120|Ubiquitin mediated proteolysis		nr -
Unigene10821_All	386	0.00	5.51	0.83	2.19	2.77	1.17	3.07	5.52	4.13	1.24E+01	4.10E-12	Up	9.69E+00	4.54E-02		3.34E-01	6.42E-01		-9.07E-01	2.47E-01		8.48E-01	3.14E-02		4.29E-01	3.53E-01		Unigene10821_All//gi|297832174|ref|XP_002883969.1|//8.00E-18//myo-inositol oxygenase 2 [Arabidopsis lyrata subsp. lyrata] >gi|297329809|gb|EFH60228.1| myo-inositol oxygenase 2 [Arabidopsis lyrata subsp. lyrata]	Unigene10821_All//6.00E-17//AT5G56640| MIOX5; inositol oxygenase/ iron ion binding	Unigene10821_All//6.00E-99//ES827071		ko00562|Inositol phosphate metabolism//ko00053|Ascorbate and aldarate metabolism	GO:0006020//GO:0016701//GO:0044424//GO:0046914	nr -
Unigene108212_All	340	0.00	0.00	0.00	0.00	0.00	0.00	2.24	0.00	0.00	null	null		null	null		null	null		null	null		-1.11E+01	1.68E-05	Down	-1.11E+01	8.89E-06	Down	Unigene108212_All//gi|224008993|ref|XP_002293455.1|//6.00E-22//vacuolar proton pump alpha subunit [Thalassiosira pseudonana CCMP1335] >gi|220970855|gb|EED89191.1| vacuolar proton pump alpha subunit [Thalassiosira pseudonana CCMP1335]	Unigene108212_All//1.00E-13//AT1G78900| VHA-A (VACUOLAR ATP SYNTHASE SUBUNIT A); ATP binding / hydrogen ion transporting ATP synthase, rotational mechanism / hydrolase, acting on acid anhydrides, catalyzing transmembrane movement of substances / proton-transporting ATPase, rotational mechanism			ko00190|Oxidative phosphorylation//ko04145|Phagosome		nr +
Unigene108214_All	403	2.47	0.50	0.00	2.84	2.82	1.12	4.51	1.56	0.99	-2.29E+00	4.05E-03		-1.13E+01	1.77E-05	Down	-1.18E-02	9.64E-01		-1.34E+00	4.01E-02		-1.54E+00	6.84E-04	Down	-2.19E+00	5.01E-06	Down	Unigene108214_All//gi|195626560|gb|ACG35110.1|//6.00E-17//cytochrome c oxidase copper chaperone [Zea mays]	Unigene108214_All//7.00E-18//AT3G15352| ATCOX17; copper chaperone	Unigene108214_All//0.00E+00//gi|84167290|gb|DR455938.1|DR455938		ko00190|Oxidative phosphorylation		nr -
Unigene108229_All	491	0.00	0.00	0.00	0.00	0.00	0.00	1.64	0.17	0.16	null	null		null	null		null	null		null	null		-3.27E+00	4.49E-04	Down	-3.33E+00	2.47E-04	Down	Unigene108229_All//gi|29539334|dbj|BAC67668.1|//8.00E-50//elongation factor-2 [Cyanidioschyzon merolae]	Unigene108229_All//3.00E-41//AT1G56070| LOS1; copper ion binding / translation elongation factor/ translation factor, nucleic acid binding					nr -
Unigene10823_All	482	0.00	7.99	5.97	0.00	0.00	0.00	0.00	0.00	0.00	1.30E+01	6.32E-22	Up	1.25E+01	6.96E-16	Up	null	null		null	null		null	null		null	null		Unigene10823_All//gi|156554296|ref|XP_001602465.1|//4.00E-67//PREDICTED: similar to S14e ribosomal protein [Nasonia vitripennis]	Unigene10823_All//1.00E-58//AT2G36160| 40S ribosomal protein S14 (RPS14A)	Unigene10823_All//7.00E-19//TC154908		ko03010|Ribosome	GO:0003723//GO:0005198//GO:0009536//GO:0010467//GO:0030529	nr -
Unigene108237_All	576	0.00	0.00	0.00	0.52	0.18	0.00	2.72	0.15	0.14	null	null		null	null		-1.53E+00	2.98E-01		-9.02E+00	4.01E-02		-4.23E+00	7.78E-09	Down	-4.30E+00	2.71E-09	Down	Unigene108237_All//gi|25991350|gb|AAN76811.1|AF453448_1//3.00E-23//PEP carboxylase kinase [Solanum tuberosum] >gi|33329260|gb|AAQ10030.1| PEPC kinase 1a [Solanum tuberosum]	Unigene108237_All//4.00E-23//AT1G08650| PPCK1 (PHOSPHOENOLPYRUVATE CARBOXYLASE KINASE); kinase/ protein serine/threonine kinase	Unigene108237_All//1.00E-42//TC132996		ko04626|Plant-pathogen interaction		nr -
Unigene108242_All	241	0.00	0.00	0.00	0.00	0.00	0.00	2.98	0.35	0.33	null	null		null	null		null	null		null	null		-3.10E+00	1.40E-03		-3.17E+00	8.09E-04	Down	Unigene108242_All//gi|297728401|ref|NP_001176564.1|//6.00E-16//Os11g0521000 [Oryza sativa Japonica Group] >gi|77551166|gb|ABA93963.1| GDSL-like Lipase/Acylhydrolase family protein, expressed [Oryza sativa (japonica cultivar-group)] >gi|255680130|dbj|BAH95292.1| Os11g0521000 [Oryza sativa Japonica Group]	Unigene108242_All//2.00E-16//AT5G03980| GDSL-motif lipase/hydrolase family protein					nr -
Unigene108247_All	299	0.00	0.17	0.53	0.00	0.00	0.15	0.42	0.00	3.06	7.41E+00	6.21E-01		9.06E+00	2.40E-01		null	null		7.24E+00	7.03E-01		-8.73E+00	2.36E-01		2.85E+00	3.18E-04	Up	Unigene108247_All//gi|18405138|ref|NP_565911.1|//2.00E-15//aspartyl protease family protein [Arabidopsis thaliana] >gi|20196976|gb|AAB87120.2| expressed protein [Arabidopsis thaliana] >gi|20197046|gb|AAM14894.1| expressed protein [Arabidopsis thaliana]	Unigene108247_All//1.00E-16//AT2G39710| aspartyl protease family protein					nr +

Unigene10825_All	416	0.00	7.31	8.06	0.00	0.17	0.00	0.00	0.00	0.00	1.28E+01	2.67E-17	Up	1.30E+01	1.47E-18	Up	7.37E+00	6.15E-01		null	null		null	null		null	null	
Unigene108255_All	309	0.00	0.00	0.00	0.00	0.00	0.00	6.57	0.41	0.26	null	null		null	null		null	null		null	null		-4.02E+00	5.83E-11	Down	-4.67E+00	1.83E-12	Down
Unigene108256_All	248	0.63	1.84	0.21	1.61	0.69	0.55	9.89	4.38	3.05	1.54E+00	1.96E-01		-1.56E+00	5.34E-01		-1.21E+00	3.14E-01		-1.56E+00	2.31E-01		-1.17E+00	1.40E-03		-1.70E+00	7.66E-06	Down
Unigene108258_All	599	0.00	0.00	0.00	0.00	0.00	0.00	2.96	2.79	0.00	null	null		null	null		null	null		null	null		-8.71E-02	8.50E-01		-1.15E+01	4.12E-13	Down
Unigene10826_All	452	1.85	0.22	0.00	2.65	2.13	0.80	1.50	0.74	0.62	-3.05E+00	2.11E-03		-1.09E+01	1.19E-04	Down	-3.10E-01	6.07E-01		-1.73E+00	8.52E-03		-1.02E+00	2.04E-01		-1.28E+00	9.49E-02	
Unigene108265_All	246	0.00	0.00	0.00	0.00	0.00	0.00	2.06	0.00	0.00	null	null		null	null		null	null		null	null		-1.10E+01	8.91E-04	Down	-1.10E+01	5.56E-04	Down
Unigene10827_All	920	0.00	0.00	0.00	0.00	0.00	0.00	4.69	1.00	0.69	null	null		null	null		null	null		null	null		-2.23E+00	5.09E-13	Down	-2.76E+00	5.24E-17	Down
Unigene108272_All	250	0.00	0.00	0.00	0.00	0.00	0.00	1.52	6.02	22.95	null	null		null	null		null	null		null	null		1.98E+00	2.06E-04	Up	3.91E+00	6.01E-30	Up
Unigene108274_All	642	0.00	0.00	0.00	0.00	0.00	0.00	2.50	0.72	0.00	null	null		null	null		null	null		null	null		-1.81E+00	3.20E-04	Down	-1.13E+01	7.00E-12	Down
Unigene108278_All	395	0.00	0.00	0.13	0.38	0.09	0.23	2.35	0.42	0.20	null	null		7.07E+00	6.41E-01		-2.12E+00	3.87E-01		-7.26E-01	7.17E-01		-2.48E+00	1.07E-03		-3.55E+00	3.97E-05	Down
Unigene108279_All	476	0.44	0.96	1.68	0.42	0.00	0.00	2.49	1.40	0.42	1.12E+00	3.28E-01		1.93E+00	3.42E-02		-8.71E+00	8.47E-02		-8.71E+00	1.34E-01		-8.24E-01	1.42E-01		-2.57E+00	6.98E-05	Down
Unigene108285_All	439	2342.88	1085.53	2076.02	319.31	349.45	224.69	3.27	1.24	0.82	-1.11E+00	0.00E+00	Down	-1.75E-01	1.96E-29		1.30E-01	1.38E-03		-5.07E-01	1.12E-33		-1.40E+00	6.15E-03		-2.00E+00	1.70E-04	Down
Unigene10829_All	361	0.58	0.42	0.15	1.38	2.48	0.50	1.99	0.00	0.33	-4.61E-01	7.58E-01		-1.97E+00	3.82E-01		8.46E-01	2.87E-01		-1.46E+00	1.93E-01		-1.10E+01	3.27E-05	Down	-2.59E+00	2.81E-03	
Unigene108291_All	381	0.00	0.00	0.00	0.00	0.00	0.00	2.44	0.55	0.21	null	null		null	null		null	null		null	null		-2.15E+00	2.95E-03		-3.55E+00	3.97E-05	Down
Unigene10830_All	288	0.18	2.46	0.74	0.00	0.96	2.51	0.88	2.03	1.11	3.76E+00	2.36E-03		2.03E+00	3.62E-01		9.90E+00	7.03E-02		1.13E+01	2.31E-04	Up	1.21E+00	1.71E-01		3.29E-01	7.64E-01	
Unigene108301_All	449	0.00	0.00	0.00	0.00	0.00	0.00	5.56	1.68	0.80	null	null		null	null		null	null		null	null		-1.73E+00	7.81E-06	Down	-2.80E+00	2.72E-10	Down
Unigene10831_All	912	0.00	0.44	0.58	0.22	0.57	0.00	0.60	1.74	3.63	8.80E+00	1.36E-02		9.19E+00	4.05E-03		1.37E+00	2.26E-01		-7.77E+00	1.34E-01		1.53E+00	1.78E-03		2.59E+00	3.51E-12	Up
Unigene108314_All	413	0.89	0.00	0.00	0.00	0.42	0.44	1.33	0.51	0.00	-9.79E+00	1.97E-02		-9.79E+00	2.79E-02		8.70E+00	2.28E-01		8.77E+00	1.95E-01		-1.40E+00	1.35E-01		-1.04E+01	2.81E-04	Down
Unigene108316_All	394	0.00	0.00	0.00	0.00	0.00	0.00	3.43	0.74	0.40	null	null		null	null		null	null		null	null		-2.21E+00	1.58E-04	Down	-3.09E+00	1.97E-06	Down
Unigene10832_All	333	0.00	172.09	102.82	0.30	0.00	0.54	0.00	0.00	0.00	1.74E+01	0.00E+00	Up	1.66E+01	2.77E-194	Up	-8.23E+00	3.17E-01		8.59E-01	6.96E-01		null	null		null	null	
Unigene108325_All	423	0.25	0.48	0.00	0.24	0.65	0.11	2.90	1.58	0.19	9.54E-01	6.22E-01		-7.95E+00	3.98E-01		1.47E+00	4.20E-01		-1.14E+00	6.79E-01		-8.75E-01	1.13E-01		-3.94E+00	4.86E-07	Down
Unigene10833_All	367	2.85	1.38	1.89	3.66	1.97	0.49	0.23	0.00	0.22	-1.05E+00	1.20E-01		-5.96E-01	3.93E-01		-8.95E-01	1.07E-01		-2.90E+00	4.29E-05	Down	-7.85E+00	4.07E-01		-8.58E-02	9.73E-01	
Unigene108331_All	225	0.00	0.45	0.47	0.22	0.00	0.20	4.89	2.23	1.06	8.82E+00	3.90E-01		8.89E+00	4.02E-01		-7.79E+00	5.63E-01		-1.42E-01	9.56E-01		-1.13E+00	5.52E-02		-2.20E+00	5.66E-04	Down
Unigene108332_All	765	0.68	0.60	0.14	0.72	0.41	0.18	2.32	0.49	0.52	-1.98E-01	8.20E-01		-2.30E+00	6.77E-02		-8.22E-01	3.96E-01		-2.02E+00	7.06E-02		-2.24E+00	8.44E-06	Down	-2.16E+00	8.30E-06	Down
Unigene108333_All	1798	0.00	0.00	0.00	0.06	0.00	0.00	4.47	0.51	0.18	null	null		null	null		-5.79E+00	3.17E-01		-5.79E+00	4.05E-01		-3.13E+00	5.01E-34	Down	-4.66E+00	8.88E-48	Down
Unigene10834_All	763	0.82	0.00	0.07	0.13	0.27	0.12	0.11	5.92	3.24	-9.68E+00	7.92E-04	Down	-3.56E+00	8.32E-03		1.05E+00	6.13E-01		-1.42E-01	9.53E-01		5.74E+00	1.24E-28	Up	4.87E+00	5.00E-15	Up
Unigene108340_All	312	0.00	0.00	0.00	0.00	0.00	0.00	2.98	0.00	0.00	null	null		null	null		null	null		null	null		-1.15E+01	1.15E-06	Down	-1.15E+01	5.48E-07	Down
Unigene108341_All	1039	0.00	0.00	0.00	0.00	0.00	0.00	6.31	0.08	0.00	null	null		null	null		null	null		null	null		-6.29E+00	5.49E-43	Down	-1.26E+01	3.26E-48	Down
Unigene108350_All	935	0.00	0.00	0.00	0.00	0.00	0.00	2.58	0.58	1.02	null	null		null	null		null	null		null	null		-2.15E+00	2.78E-07	Down	-1.33E+00	2.65E-04	Down
Unigene108355_All	420	0.00	0.00	0.00	0.00	0.00	0.00	3.22	0.80	0.66	null	null		null	null		null	null		null	null		-2.02E+00	4.01E-04	Down	-2.28E+00	7.13E-05	Down
Unigene108360_All	355	0.00	0.00	0.00	0.28	0.00	0.13	2.38	0.24	1.01	null	null		null	null		-8.13E+00	3.17E-01		-1.14E+00	6.79E-01		-3.34E+00	2.52E-04	Down	-1.24E+00	6.26E-02	
Unigene108363_All	320	0.00	0.00	0.00	0.00	0.00	0.00	13.87	37.35	1.37	null	null		null	null		null	null		null	null		1.43E+00	3.60E-19	Up	-3.34E+00	5.76E-21	Down
Unigene108369_All	466	0.00	0.00	0.00	0.00	0.00	0.00	1.63	0.18	13.34	null	null		null	null		null	null		null	null		-3.19E+00	7.93E-04	Down	3.03E+00	4.22E-26	Up
Unigene10837_All	368	0.14	0.00	0.00	4.60	0.37	3.31	0.00	0.00	0.00	-7.15E+00	6.19E-01		-7.15E+00	6.32E-01		-3.62E+00	1.02E-08	Down	-4.74E-01	3.86E-01		null	null		null	null	
Unigene108376_All	529	0.00	0.00	0.00	0.00	0.00	0.00	2.80	1.50	0.00	null	null		null	null		null	null		null	null		-8.98E-01	6.74E-02		-1.14E+01	5.85E-11	Down
Unigene108380_All	496	0.00	0.00	0.00	0.00	0.00	0.00	1.62	0.17	0.40	null	null		null	null		null	null		null	null		-3.26E+00	4.49E-04	Down	-2.01E+00	7.11E-03	
Unigene108381_All	589	0.00	0.00	0.00	0.00	0.00	0.00	4.88	0.28	0.27	null	null		null	null		null	null		null	null		-4.10E+00	1.53E-15	Down	-4.17E+00	2.58E-16	Down
Unigene108383_All	379	0.00	0.00	0.00	0.13	0.00	0.36	2.57	0.22	0.00	null	null		null	null		-7.04E+00	5.63E-01		1.44E+00	6.39E-01		-3.54E+00	4.32E-05	Down	-1.13E+01	2.72E-07	Down

<table>
<tr><td>Unigene10825_All</td><td>Unigene10825_All//sp|P35749|MYH11_
HUMAN//6.00E-29//Myosin-11 OS=Homo
sapiens GN=MYH11 PE=1 SV=3</td><td></td><td></td><td></td><td></td><td></td><td>swissprot +</td></tr>
<tr><td>Unigene108255_All</td><td>Unigene108255_All//gi|6012952|emb|
CAB57309.1|//9.00E-15//60S
ribosomal protein L6 (YL 16 like)
[Cyanophora paradoxa]</td><td></td><td></td><td></td><td>ko03010|Ribosome</td><td></td><td>nr -</td></tr>
<tr><td>Unigene108256_All</td><td>Unigene108256_All//gi|297810879|re
f|XP_002873323.1|//5.00E-
06//predicted protein [Arabidopsis
lyrata subsp. lyrata]
>gi|297319160|gb|EFH49582.1|
predicted protein [Arabidopsis
lyrata subsp. lyrata]</td><td></td><td>Unigene108256_All//1.00E-31//ES819093</td><td></td><td></td><td></td><td>nr +</td></tr>
<tr><td>Unigene108258_All</td><td>Unigene108258_All//gi|115484761|re
f|NP_001067524.1|//2.00E-
54//Os11g0220800 [Oryza sativa
Japonica Group]
>gi|122240410|sp|Q0ITS8.1|RL101_OR
YSJ RecName: Full=60S ribosomal
protein L10-1; AltName:
Full=Putative tumor suppressor
SC34; AltName: Full=Protein QM
>gi|158513712|sp|A2ZCQ7.2|RL101_OR
YSI RecName: Full=60S ribosomal
protein L10-1; AltName:
Full=Putative tumor suppressor
SC34; AltName: Full=Protein QM
>gi|113644746|dbj|BAF27887.1|
Os11g0220800 [Oryza sativa
Japonica Group]</td><td>Unigene108258_All//1.00E-
53//AT1G66580| 60S ribosomal
protein L10 (RPL10C)</td><td>Unigene108258_All//3.00E-15//CD485839</td><td></td><td>ko03010|Ribosome</td><td></td><td>nr -</td></tr>
<tr><td>Unigene10826_All</td><td>Unigene10826_All//gi|224054508|ref
|XP_002298295.1|//2.00E-
13//predicted protein [Populus
trichocarpa]
>gi|222845553|gb|EEE83100.1|
predicted protein [Populus
trichocarpa]</td><td></td><td>Unigene10826_All//1.00E-16//ES852406</td><td></td><td></td><td></td><td>nr -</td></tr>
<tr><td>Unigene108265_All</td><td></td><td>Unigene108265_All//5.00E-
07//AT1G48590| C2 domain-
containing protein</td><td></td><td></td><td></td><td></td><td>ESTscan +</td></tr>
<tr><td>Unigene10827_All</td><td>Unigene10827_All//sp|P28770|EPD1_O
NCMY//1.00E-21//Ependymin-1
OS=Oncorhynchus mykiss GN=epd1
PE=1 SV=1</td><td></td><td></td><td></td><td></td><td></td><td>swissprot +</td></tr>
<tr><td>Unigene108272_All</td><td></td><td></td><td></td><td></td><td></td><td></td><td></td></tr>
<tr><td>Unigene108274_All</td><td>Unigene108274_All//gi|159480690|re
f|XP_001698415.1|//2.00E-07//basal
body protein [Chlamydomonas
reinhardtii]
>gi|158282155|gb|EDP07908.1| basal
body protein [Chlamydomonas
reinhardtii]</td><td>Unigene108274_All//6.00E-
06//AT1G55250| HUB2 (HISTONE MONO-
UBIQUITINATION 2); protein binding
/ zinc ion binding</td><td></td><td></td><td></td><td></td><td>nr -</td></tr>
<tr><td>Unigene108278_All</td><td>Unigene108278_All//gi|38605802|emb
|CAE05270.2|//2.00E-
06//OSJNBb0014D23.4 [Oryza sativa
(japonica cultivar-group)]</td><td></td><td></td><td></td><td></td><td></td><td>nr +</td></tr>
<tr><td>Unigene108279_All</td><td>Unigene108279_All//gi|187940732|gb
|ACD39467.1|//5.00E-06//BEL30
protein [Solanum palustre]</td><td>Unigene108279_All//2.00E-
06//AT4G34610| BLH6 (BELL1-LIKE
HOMEODOMAIN 6); DNA binding /
transcription factor</td><td>Unigene108279_All//4.00E-
17//gi|164305110|gb|ES808607.1|ES808607</td><td>HB</td><td></td><td></td><td>nr -</td></tr>
<tr><td>Unigene108285_All</td><td>Unigene108285_All//gi|5738542|emb|
CAB53034.1|//2.00E-42//photosystem
I subunit XI precursor
[Arabidopsis thaliana]</td><td>Unigene108285_All//2.00E-
43//AT4G12800| PSAL (photosystem I
subunit L)</td><td>Unigene108285_All//0.00E+00//TC136126</td><td></td><td>ko00195|Photosynthesis</td><td></td><td>nr +</td></tr>
<tr><td>Unigene10829_All</td><td></td><td></td><td></td><td></td><td></td><td></td><td>ESTscan +</td></tr>
<tr><td>Unigene108291_All</td><td>Unigene108291_All//gi|301123509|re
f|XP_002909481.1|//1.00E-27//60S
ribosomal protein L21-1
[Phytophthora infestans T30-4]
>gi|262100243|gb|EEY58295.1| 60S
ribosomal protein L21-1
[Phytophthora infestans T30-4]</td><td>Unigene108291_All//9.00E-
28//AT1G09690| 60S ribosomal
protein L21 (RPL21C)</td><td></td><td></td><td>ko03010|Ribosome</td><td></td><td>nr -</td></tr>
<tr><td>Unigene10830_All</td><td></td><td></td><td></td><td></td><td></td><td></td><td></td></tr>
<tr><td>Unigene108301_All</td><td></td><td></td><td>Unigene108301_All//5.00E-10//TC135460</td><td></td><td></td><td></td><td></td></tr>
<tr><td>Unigene10831_All</td><td></td><td></td><td>Unigene10831_All//1.00E-40//TC168132</td><td></td><td></td><td></td><td></td></tr>
<tr><td>Unigene108314_All</td><td></td><td></td><td></td><td></td><td></td><td></td><td></td></tr>
<tr><td>Unigene108316_All</td><td>Unigene108316_All//gi|34105768|gb|
AAQ62075.1|//5.00E-35//ribosomal
protein L3 [Triticum aestivum]
>gi|34105770|gb|AAQ62076.1|
ribosomal protein L3 [Triticum
aestivum]
>gi|34105774|gb|AAQ62078.1|
ribosomal protein L3 [Triticum
aestivum]
>gi|118152389|gb|ABK63938.1|
ribosomal protein L3-B3 [Triticum
aestivum]
>gi|118152400|gb|ABK63944.1|
ribosomal protein L3-B1 [Triticum
aestivum]
>gi|146141392|tpg|DAA01144.1|
TPA_inf: ribosomal protein L3B-3
[Triticum aestivum]
>gi|146141396|tpg|DAA01146.1|
TPA_inf: ribosomal protein L3B-1
[Triticum aestivum]</td><td>Unigene108316_All//2.00E-
27//AT1G61580| RPL3B (R-PROTEIN L3
B); structural constituent of
ribosome</td><td>Unigene108316_All//5.00E-
07//gi|78348789|gb|DT569063.1|DT569063</td><td></td><td>ko03010|Ribosome</td><td></td><td>nr +</td></tr>
<tr><td>Unigene10832_All</td><td></td><td></td><td></td><td></td><td></td><td></td><td></td></tr>
<tr><td>Unigene108325_All</td><td></td><td></td><td>Unigene108325_All//1.00E-50//TC154322</td><td></td><td></td><td></td><td></td></tr>
<tr><td>Unigene10833_All</td><td></td><td></td><td>Unigene10833_All//8.00E-18//ES831804</td><td></td><td></td><td></td><td></td></tr>
<tr><td>Unigene108331_All</td><td></td><td></td><td>Unigene108331_All//2.00E-14//CO123936</td><td></td><td></td><td></td><td></td></tr>
<tr><td>Unigene108332_All</td><td></td><td></td><td>Unigene108332_All//1.00E-92//TC165404</td><td></td><td></td><td></td><td></td></tr>
<tr><td>Unigene108333_All</td><td>Unigene108333_All//gi|18147592|dbj
|BAB83086.1|//0.00E+00//cycloarten
ol synthase [Betula platyphylla]</td><td>Unigene108333_All//0.00E+00//AT2G0
7050| CAS1 (cycloartenol synthase
1); cycloartenol synthase</td><td>Unigene108333_All//2.00E-25//CB350491</td><td></td><td>ko00100|Steroid biosynthesis</td><td></td><td>nr -</td></tr>
<tr><td>Unigene10834_All</td><td>Unigene10834_All//gi|75326590|sp|Q
76CU2.1|PDR1_TOBAC//1.00E-
102//RecName: Full=Pleiotropic
drug resistance protein 1;
AltName: Full=NtPDR1
>gi|41052472|dbj|BAD07483.1| PDR-
type ABC transporter 1 [Nicotiana
tabacum]</td><td>Unigene10834_All//1.00E-
101//AT1G15520| PDR12 (PLEIOTROPIC
DRUG RESISTANCE 12); ATPase,
coupled to transmembrane movement
of substances</td><td>Unigene10834_All//0.00E+00//TC147767</td><td></td><td>ko03040|Spliceosome//ko02010|ABC
transporters</td><td></td><td>nr -</td></tr>
<tr><td>Unigene108340_All</td><td>Unigene108340_All//sp|Q58A42|DD3_D
ICDI//1.00E-16//Protein DD3-3
OS=Dictyostelium discoideum
GN=DD3-3 PE=2 SV=1</td><td></td><td></td><td></td><td></td><td></td><td>swissprot -</td></tr>
<tr><td>Unigene108341_All</td><td>Unigene108341_All//gi|19698452|gb|
AAL93154.1|AF485268_1//1.00E-
137//bacterial-induced class III
peroxidase [Gossypium hirsutum]</td><td>Unigene108341_All//1.00E-
102//AT2G41480| electron carrier/
heme binding / peroxidase</td><td>Unigene108341_All//6.00E-135//TC147787</td><td></td><td>ko00940|Phenylpropanoid
biosynthesis//ko00360|Phenylalanin
e metabolism</td><td></td><td>nr +</td></tr>
<tr><td>Unigene108350_All</td><td>Unigene108350_All//gi|297806151|re
f|XP_002870959.1|//1.00E-95//amino
acid transporter family protein
[Arabidopsis lyrata subsp. lyrata]
>gi|297316796|gb|EFH47218.1| amino
acid transporter family protein
[Arabidopsis lyrata subsp. lyrata]</td><td>Unigene108350_All//9.00E-
87//AT5G02180| amino acid
transporter family protein</td><td></td><td></td><td></td><td></td><td>nr +</td></tr>
<tr><td>Unigene108355_All</td><td></td><td></td><td></td><td></td><td></td><td></td><td></td></tr>
<tr><td>Unigene108360_All</td><td></td><td></td><td></td><td></td><td></td><td></td><td></td></tr>
<tr><td>Unigene108363_All</td><td></td><td></td><td>Unigene108363_All//2.00E-08//ES810763</td><td></td><td></td><td></td><td></td></tr>
<tr><td>Unigene108369_All</td><td></td><td></td><td></td><td></td><td></td><td></td><td></td></tr>
<tr><td>Unigene10837_All</td><td>Unigene10837_All//gi|115477821|ref
|NP_001062506.1|//3.00E-
20//Os08g0560000 [Oryza sativa
Japonica Group]
>gi|113624475|dbj|BAF24420.1|
Os08g0560000 [Oryza sativa
Japonica Group]</td><td>Unigene10837_All//1.00E-
20//AT2G36690| oxidoreductase,
2OG-Fe(II) oxygenase family
protein</td><td></td><td></td><td>ko00270|Cysteine and methionine
metabolism//ko00941|Flavonoid
biosynthesis</td><td>GO:0008152//GO:0016706</td><td>nr +</td></tr>
<tr><td>Unigene108376_All</td><td>Unigene108376_All//gi|2500261|sp|P
93099.1|RL13A_CYAPA//7.00E-
41//RecName: Full=60S ribosomal
protein L13a
>gi|1743007|emb|CAA71090.1|
ribosomal protein L13a [Cyanophora
paradoxa]</td><td>Unigene108376_All//3.00E-
37//AT4G13170| 60S ribosomal
protein L13A (RPL13aC)</td><td></td><td></td><td>ko03010|Ribosome</td><td></td><td>nr +</td></tr>
<tr><td>Unigene108380_All</td><td>Unigene108380_All//gi|15242647|ref
|NP_198848.1|//1.00E-09//DC1
domain-containing protein
[Arabidopsis thaliana]
>gi|10178142|dbj|BAB11587.1| CHP-
rich zinc finger protein-like
[Arabidopsis thaliana]</td><td>Unigene108380_All//6.00E-
11//AT5G40320| DC1 domain-
containing protein</td><td>Unigene108380_All//3.00E-101//CO075261</td><td></td><td></td><td></td><td>nr +</td></tr>
<tr><td>Unigene108381_All</td><td>Unigene108381_All//gi|157503121|gb
|ABV58643.1|//2.00E-37//ubiquitin
[Catharanthus roseus]</td><td>Unigene108381_All//2.00E-
24//AT1G65350| UBQ13; protein
binding</td><td>Unigene108381_All//2.00E-25//TC164984</td><td></td><td>ko03010|Ribosome</td><td></td><td>nr +</td></tr>
<tr><td>Unigene108383_All</td><td></td><td></td><td></td><td></td><td></td><td></td><td></td></tr>
</table>

Unigene108386_All	391	0.00	0.00	0.00	0.25	0.18	0.12	1.62	0.32	0.00	null	null		null	null		-5.33E-01	8.19E-01		-1.14E+00	6.79E-01		-2.34E+00	1.27E-02		-1.07E+01	7.12E-05	Down	Unigene108386_All//gi|15233062|ref|NP_191681.1|//1.00E-24//short-chain dehydrogenase/reductase (SDR) family protein [Arabidopsis thaliana] >gi|75311801|sp|Q9M2E2.1|SDR1_ARATH RecName: Full=(+)-neomenthol dehydrogenase; AltName: Full=Menthone:neomenthol reductase; AltName: Full=Short-chain dehydrogenase/reductase 1; Short=AtSDR1	Unigene108386_All//1.00E-25//AT3G61220| short-chain dehydrogenase/reductase (SDR) family protein	Unigene108386_All//4.00E-174//ES839198				nr +
Unigene10839_All	259	0.00	11.74	2.88	0.00	0.00	0.00	0.00	0.00	0.00	1.35E+01	2.67E-17	Up	1.15E+01	3.28E-04	Up	null	null		null	null		null	null		null	null								ESTscan -
Unigene108393_All	875	0.06	0.00	0.00	0.00	0.08	0.00	9.91	1.58	0.14	-5.90E+00	6.19E-01		-5.90E+00	6.32E-01		6.30E+00	6.15E-01		null	null		-2.65E+00	5.14E-31	Down	-6.18E+00	1.58E-58	Down							
Unigene108396_All	368	0.00	0.00	0.00	0.00	0.00	0.00	1.49	1.36	0.00	null	null		null	null		null	null		null	null		-1.32E-01	8.80E-01		-1.05E+01	2.81E-04	Down							
Unigene108397_All	321	0.00	0.00	0.00	0.00	0.00	0.00	2.50	0.65	0.00	null	null		null	null		null	null		null	null		-1.94E+00	1.17E-02		-1.13E+01	4.43E-06	Down							ESTscan +
Unigene108398_All	275	8.18	2.76	4.84	17.21	14.78	19.05	7.69	3.50	8.69	-1.57E+00	5.19E-04	Down	-7.57E-01	9.06E-02		-2.20E-01	4.78E-01		1.47E-01	6.22E-01		-1.14E+00	4.42E-03		1.77E-01	6.46E-01								
Unigene10841_All	434	0.72	0.23	0.00	1.72	1.82	1.04	5.75	0.67	0.64	-1.63E+00	2.77E-01		-9.50E+00	4.93E-02		8.40E-02	9.45E-01		-7.26E-01	3.90E-01		-3.09E+00	7.09E-11	Down	-3.16E+00	1.62E-11	Down	Unigene10841_All//gi|46359653|dbj|BAD15331.1|//3.00E-42//cytochrome P450 [Panax ginseng]	Unigene10841_All//1.00E-31//AT2G45570| CYP76C2; electron carrier/ heme binding / iron ion binding / monooxygenase/ oxygen binding	Unigene10841_All//0.00E+00//TC135966		ko00940|Phenylpropanoid biosynthesis//ko00945|Stilbenoid, diarylheptanoid and gingerol biosynthesis//ko00903|Limonene and pinene degradation//ko00941|Flavonoid biosynthesis//ko00944|Flavone and flavonol biosynthesis	GO:0003676//GO:0005506//GO:0006259//GO:0016709	nr -
Unigene108423_All	523	0.00	0.00	0.00	0.00	0.00	0.00	1.86	1.36	0.00	null	null		null	null		null	null		null	null		-4.53E-01	4.90E-01		-1.09E+01	2.72E-07	Down	Unigene108423_All//gi|301095349|ref|XP_002896775.1|//6.00E-26//60S acidic ribosomal protein P0 [Phytophthora infestans T30-4] >gi|262108658|gb|EEY66710.1| 60S acidic ribosomal protein P0 [Phytophthora infestans T30-4]	Unigene108423_All//2.00E-23//AT2G40010| 60S acidic ribosomal protein P0 (RPP0A)			ko03010|Ribosome		nr +
Unigene10843_All	285	0.00	0.00	0.00	0.00	0.00	0.00	1.78	1.76	0.00	null	null		null	null		null	null		null	null		-1.66E-02	1.02E+00		-1.08E+01	5.56E-04	Down					ko00910|Nitrogen metabolism		kegg -
Unigene10844_All	360	0.73	0.00	0.30	0.00	0.19	0.50	5.29	1.97	0.66	-9.51E+00	6.72E-02		-1.30E+00	4.37E-01		7.58E+00	6.15E-01		8.97E+00	1.95E-01		-1.42E+00	1.09E-03		-2.99E+00	1.57E-08	Down							
Unigene108448_All	1989	0.11	0.00	0.00	0.05	0.03	0.00	1.51	3.13	1.12	-6.72E+00	1.23E-01		-6.72E+00	1.47E-01		-5.34E-01	8.19E-01		-5.65E+00	4.05E-01		1.05E+00	1.00E-06	Up	-4.28E-01	1.78E-01		Unigene108448_All//gi|297827551|ref|XP_002881658.1|//8.00E-22//F-box family protein [Arabidopsis lyrata subsp. lyrata] >gi|297327497|gb|EFH57917.1| F-box family protein [Arabidopsis lyrata subsp. lyrata]	Unigene108448_All//7.00E-22//AT2G39490| F-box family protein	Unigene108448_All//5.00E-23//TC177932				nr +
Unigene108452_All	341	0.00	0.00	0.00	0.00	0.00	0.00	6.08	1.72	3.04	null	null		null	null		null	null		null	null		-1.82E+00	2.74E-05	Down	-1.00E+00	9.64E-03		Unigene108452_All//gi|226453532|gb|EEH50843.1|//1.00E-10//predicted protein [Micromonas pusilla CCMP1545]		Unigene108452_All//6.00E-37//gi|45595442|gb|CK988291.1|CK988291				nr -
Unigene108457_All	237	0.00	0.00	0.00	0.00	0.00	0.00	4.64	6.88	0.00	null	null		null	null		null	null		null	null		5.68E-01	2.34E-01		-1.22E+01	3.33E-08	Down							
Unigene108459_All	527	0.00	0.00	0.00	0.00	0.00	0.00	2.65	3.09	0.00	null	null		null	null		null	null		null	null		2.24E-01	6.27E-01		-1.14E+01	2.40E-10	Down	Unigene108459_All//gi|159469668|ref|XP_001692985.1|//2.00E-65//ribosomal protein L8, component of cytosolic 80S ribosome and 60S large subunit [Chlamydomonas reinhardtii] >gi|158277787|gb|EDP03554.1| ribosomal protein L8 [Chlamydomonas reinhardtii]	Unigene108459_All//4.00E-61//AT4G36130| 60S ribosomal protein L8 (RPL8C)	Unigene108459_All//4.00E-14//ES836532		ko03010|Ribosome		nr +
Unigene108463_All	362	0.00	0.00	0.00	0.00	0.00	0.00	1.40	0.23	0.00	null	null		null	null		null	null		null	null		-2.60E+00	2.05E-02		-1.05E+01	5.56E-04	Down	Unigene108463_All//gi|38230174|gb|AAR14182.1|//6.00E-32//Rho family GTPase [Fucus distichus]	Unigene108463_All//4.00E-30//AT4G35020| ARAC3 (ARABIDOPSIS RAC-LIKE 3); GTP binding / GTPase			ko04145|Phagosome//ko04650|Natural killer cell mediated cytotoxicity	GO:0015630//GO:0016020//GO:0017111//GO:0031981//GO:0032561//GO:0035556	nr -
Unigene108466_All	429	0.00	0.00	0.00	0.00	0.00	0.00	1.18	0.97	0.00	null	null		null	null		null	null		null	null		-2.80E-01	7.49E-01		-1.02E+01	5.56E-04	Down	Unigene108466_All//gi|159468233|ref|XP_001692287.1|//3.00E-42//elongation factor EF-3 [Chlamydomonas reinhardtii] >gi|158278473|gb|EDP04237.1| elongation factor EF-3 [Chlamydomonas reinhardtii]	Unigene108466_All//4.00E-14//AT3G54540| ATGCN4; transporter	Unigene108466_All//1.00E-10//TC134087			GO:0000166//GO:0003676//GO:0017111//GO:0043232//GO:0051276	nr -
Unigene108469_All	251	0.00	0.00	0.00	0.00	0.00	0.00	2.53	2.16	0.00	null	null		null	null		null	null		null	null		-2.23E-01	7.74E-01		-1.13E+01	7.12E-05	Down	Unigene108469_All//gi|301102985|ref|XP_002900579.1|//2.00E-06//RNA polymerase-associated protein RTF1 [Phytophthora infestans T30-4] >gi|262101842|gb|EEY59894.1| RNA polymerase-associated protein RTF1 [Phytophthora infestans T30-4]					GO:0003676//GO:0006259//GO:0006351//GO:0006464//GO:0043231	nr -
Unigene108474_All	300	0.35	0.00	0.18	0.17	0.23	0.15	1.83	0.00	0.27	-8.45E+00	3.76E-01		-9.75E-01	7.22E-01		4.68E-01	9.61E-01		-1.41E-01	9.56E-01		-1.08E+01	4.62E-04	Down	-2.79E+00	7.97E-03								
Unigene10848_All	615	0.00	20.68	6.93	0.00	0.00	0.00	0.00	0.00	0.00	1.43E+01	5.60E-73	Up	1.28E+01	1.22E-23	Up	null	null		null	null		null	null		null	null		Unigene10848_All//gi|297844300|ref|XP_002890031.1|//2.00E-51//eukaryotic translation initiation factor 5A-1 [Arabidopsis lyrata subsp. lyrata] >gi|297335873|gb|EFH66290.1| eukaryotic translation initiation factor 5A-1 [Arabidopsis lyrata subsp. lyrata]	Unigene10848_All//4.00E-52//AT1G13950| ELF5A-1 (EUKARYOTIC ELONGATION FACTOR 5A-1); translation initiation factor				GO:0006448//GO:0006449//GO:0008135//GO:0010087//GO:0018205//GO:0043021	nr +
Unigene108480_All	342	0.00	0.00	0.00	0.00	0.00	0.00	3.21	0.49	0.47	null	null		null	null		null	null		null	null		-2.72E+00	1.31E-04	Down	-2.79E+00	6.37E-05	Down	Unigene108480_All//gi|9653250|ref|NP_062496.1|//1.00E-07//NADH dehydrogenase subunit 4 [Chondrus crispus] >gi|1352567|sp|P48915.1|NU4M_CHOCR RecName: Full=NADH-ubiquinone oxidoreductase chain 4; AltName: Full=NADH dehydrogenase subunit 4 >gi|1334501|emb|CAA87623.1| NADH dehydrogenase (ubiquinone), subunit 4 [Chondrus crispus]					GO:0016491//GO:0022904//GO:0043231//GO:0044425	nr -
Unigene108486_All	252	0.00	0.00	0.00	0.00	0.00	0.00	3.86	0.00	0.47	null	null		null	null		null	null		null	null		-1.19E+01	5.84E-07	Down	-3.02E+00	9.61E-05	Down							
Unigene10849_All	524	0.00	5.71	1.12	0.00	0.00	0.00	0.08	0.00	0.00	1.25E+01	5.19E-17	Up	1.01E+01	2.17E-03		null	null		null	null		-6.33E+00	6.62E-01		-6.33E+00	6.17E-01		Unigene10849_All//gi|7248459|gb|AAF43497.1|AF134579_1//4.00E-09//arabinogalactan protein [Zea mays]	Unigene10849_All//9.00E-08//AT1G21270| WAK2; ATP binding / calcium ion binding / protein kinase/ protein serine/threonine kinase				GO:0000902//GO:0005975//GO:0006884//GO:0010033//GO:0016020	nr -
Unigene108494_All	470	0.00	0.00	0.00	0.00	0.00	0.00	2.16	0.62	0.08	null	null		null	null		null	null		null	null		-1.79E+00	6.30E-03		-4.67E+00	1.72E-06	Down	Unigene108494_All//gi|247421835|gb|ACS96445.1|//1.00E-52//S18.A ribosomal protein [Jatropha curcas]	Unigene108494_All//1.00E-47//AT4G09800| RPS18C (S18 RIBOSOMAL PROTEIN); RNA binding / nucleic acid binding / structural constituent of ribosome			ko03010|Ribosome	GO:0003676//GO:0005198//GO:0006412//GO:0015935//GO:0016020//GO:0030312//GO:0031981	nr +
Unigene1085_All	3274	1.68	3.73	1.76	1.99	2.60	2.30	1.98	4.49	5.48	1.15E+00	1.08E-11	Up	6.60E-02	8.01E-01		3.82E-01	5.68E-02		2.09E-01	3.87E-01		1.19E+00	9.67E-18	Up	1.47E+00	3.08E-30	Up	Unigene1085_All//gi|15234061|ref|NP_192018.1|//2.00E-86//extra-large G-protein-related [Arabidopsis thaliana] >gi|17979014|gb|AAL47466.1| AT4g01090/F2N1_13 [Arabidopsis thaliana] >gi|20334762|gb|AAM16242.1| AT4g01090/F2N1_13 [Arabidopsis thaliana] >gi|222424660|dbj|BAH20284.1| AT4G01090 [Arabidopsis thaliana]	Unigene1085_All//5.00E-112//AT2G46380| unknown protein	Unigene1085_All//0.00E+00//gi|13351545|gb|BG441893.1|BG441893				nr -
Unigene108500_All	462	0.00	0.00	0.00	0.00	0.07	0.00	8.69	6.15	3.62	null	null		null	null		6.22E+00	8.38E-01		null	null		-4.99E-01	7.18E-02		-1.26E+00	3.83E-06	Down			Unigene108500_All//4.00E-131//gi|78352234|gb|DT572508.1|DT572508				
Unigene108501_All	598	0.35	0.17	0.09	0.25	0.17	0.00	2.26	0.77	0.33	-1.05E+00	5.65E-01		-1.98E+00	3.82E-01		-5.32E-01	7.86E-01		-7.97E+00	2.35E-01		-1.56E+00	3.85E-03		-2.76E+00	7.51E-06	Down			Unigene108501_All//6.00E-26//DW512828				
Unigene108511_All	359	0.00	0.42	0.00	0.14	0.19	0.13	2.71	0.12	0.22	8.73E+00	2.36E-01		null	null		4.67E-01	9.61E-01		-1.42E-01	9.56E-01		-4.54E+00	6.92E-06	Down	-3.61E+00	2.14E-05	Down	Unigene108511_All//gi|19715597|gb|AAL91622.1|//5.00E-16//At2g23450/F26B6.10 [Arabidopsis thaliana] >gi|23463045|gb|AAN33192.1| At2g23450/F26B6.10 [Arabidopsis thaliana]	Unigene108511_All//2.00E-17//AT2G23450| protein kinase family protein				GO:0000166//GO:0004672//GO:0008152	nr +
Unigene108513_All	570	0.00	0.00	0.00	0.00	0.00	0.00	3.63	5.28	0.56	null	null		null	null		null	null		null	null		5.39E-01	9.97E-02		-2.70E+00	2.21E-08	Down	Unigene108513_All//gi|212717149|gb|ACJ37416.1|//1.00E-31//stress-induced receptor-like kinase [Glycine max]	Unigene108513_All//5.00E-23//AT5G39020| protein kinase family protein	Unigene108513_All//2.00E-170//CO081306			GO:0004672//GO:0006464//GO:0032559	nr -
Unigene108517_All	242	0.00	0.00	0.00	0.00	0.00	0.00	2.10	0.52	0.00	null	null		null	null		null	null		null	null		-2.02E+00	5.21E-02		-1.10E+01	5.56E-04	Down							
Unigene10852_All	254	0.00	0.00	0.00	0.00	0.00	0.00	0.67	2.14	4.55	null	null		null	null		null	null		null	null		1.68E+00	7.92E-02		2.77E+00	5.09E-05	Up							
Unigene108524_All	438	0.00	0.00	0.00	0.00	0.00	0.00	2.22	0.38	1.09	null	null		null	null		null	null		null	null		-2.54E+00	6.42E-04	Down	-1.02E+00	9.02E-02		Unigene108524_All//gi|255569613|ref|XP_002525772.1|//4.00E-06//conserved hypothetical protein [Ricinus communis] >gi|255569615|ref|XP_002525773.1| conserved hypothetical protein [Ricinus communis] >gi|223534922|gb|EEF36608.1| conserved hypothetical protein [Ricinus communis] >gi|223534923|gb|EEF36609.1| conserved hypothetical protein [Ricinus communis]	Unigene108524_All//4.00E-06//AT4G10270| wound-responsive family protein	Unigene108524_All//2.00E-09//ES791233				nr -
Unigene10853_All	528	0.00	0.00	0.00	0.00	0.00	0.00	3.92	2.14	0.00	null	null		null	null		null	null		null	null		-8.76E-01	2.86E-02		-1.19E+01	2.86E-15	Down							
Unigene108533_All	274	0.00	0.00	0.00	0.00	0.00	0.00	0.31	0.00	16.72	null	null		null	null		null	null		null	null		-8.27E+00	4.06E-01		5.76E+00	1.34E-29	Up	Unigene108533_All//gi|74273615|gb|ABA01475.1|//2.00E-33//1-aminocyclopropane-1-carboxylate synthase ACS6 [Gossypium hirsutum]	Unigene108533_All//8.00E-25//AT1G01480| ACS2; 1-aminocyclopropane-1-carboxylate synthase	Unigene108533_All//8.00E-08//ES822034		ko00270|Cysteine and methionine metabolism	GO:0008152//GO:0016740//GO:0016831//GO:0016846//GO:0048037	nr +
Unigene108541_All	994	4796.46	3980.56	5932.28	912.54	1274.11	754.10	8.72	4.54	0.76	-2.69E-01	0.00E+00		3.07E-01	0.00E+00		4.82E-01	4.93E-304		-2.75E-01	6.65E-69		-9.41E-01	1.14E-07		-3.52E+00	3.85E-42	Down	Unigene108541_All//gi|224124190|ref|XP_002330127.1|//1.00E-128//light-harvesting complex I protein Lhca4 [Populus trichocarpa] >gi|222871261|gb|EEF08392.1| light-harvesting complex I protein Lhca4 [Populus trichocarpa]	Unigene108541_All//7.00E-120//AT3G47470| LHCA4 (LIGHT-HARVESTING CHLOROPHYLL-PROTEIN COMPLEX I SUBUNIT A4); chlorophyll binding	Unigene108541_All//0.00E+00//gi|48752319|gb|CO082838.1|CO082838		ko00196|Photosynthesis - antenna proteins		nr +
Unigene108545_All	442	0.00	0.00	0.00	0.00	0.00	0.00	2.20	0.09	0.00	null	null		null	null		null	null		null	null		-4.54E+00	6.92E-06	Down	-1.11E+01	2.72E-07	Down	Unigene108545_All//gi|27450759|gb|AAO14682.1|AF508263_1//2.00E-62//actin [Pyrocystis lunula]	Unigene108545_All//7.00E-54//AT3G12110| ACT11 (actin-11); structural constituent of cytoskeleton	Unigene108545_All//1.00E-26//gi|109842528|gb|DW241088.1|DW241088			GO:0032559//GO:0043232	nr +
Unigene10855_All	480	0.00	8.23	4.88	0.00	0.00	0.00	0.00	0.00	0.00	1.30E+01	1.67E-22	Up	1.23E+01	6.41E-13	Up	null	null		null	null		null	null		null	null								
Unigene108566_All	226	0.00	0.00	0.00	0.00	0.00	0.00	2.43	0.18	0.00	null	null		null	null		null	null		null	null		-3.72E+00	3.23E-03		-1.12E+01	2.81E-04	Down	Unigene108566_All//gi|73761683|gb|AAZ83341.1|//2.00E-29//ubiquitin extension protein [Gossypium hirsutum]	Unigene108566_All//7.00E-13//AT2G47110| UBQ6; protein binding	Unigene108566_All//6.00E-08//DN780054		ko03010|Ribosome	GO:0005198//GO:0010467//GO:0030529	nr +

Unigene108567_All	717	0.00	0.00	0.00	0.00	0.10	0.00	3.83	0.52	2.22	null	null		null	null		6.59E+00	6.15E-01		null	null		-2.87E+00	4.04E-11	Down	-7.86E-01	1.58E-02		Unigene108567_All//gi|42568648|ref|NP_200715.2|//3.00E-90//glycosyl transferase family 1 protein [Arabidopsis thaliana]	Unigene108567_All//3.00E-84//AT5G59070| glycosyl transferase family 1 protein					nr -
Unigene10857_All	360	1.16	0.84	2.96	2.91	1.34	0.25	0.00	0.00	0.11	-4.61E-01	6.50E-01		1.35E+00	6.01E-02		-1.12E+00	8.44E-02		-3.53E+00	9.34E-05	Down	null	null		6.79E+00	6.47E-01								
Unigene108590_All	361	0.00	0.00	0.00	0.00	0.00	0.00	10.07	1.62	0.22	null	null		null	null		null	null		null	null		-2.64E+00	2.59E-13	Down	-5.51E+00	9.56E-24	Down			Unigene108590_All//4.00E-10//TC135251				
Unigene108596_All	433	0.00	0.00	0.00	0.00	0.00	0.00	1.86	0.19	0.28	null	null		null	null		null	null		null	null		-3.26E+00	4.49E-04	Down	-2.75E+00	9.43E-04	Down	Unigene108596_All//gi|32401352|gb|AAP80848.1|//3.00E-17//protein disulfide isomerase 2 precursor [Griffithsia japonica]	Unigene108596_All//3.00E-15//AT1G77510| ATPDIL1-2 (PDI-LIKE 1-2); protein disulfide isomerase			ko04141|Protein processing in endoplasmic reticulum		nr +
Unigene108599_All	215	0.00	0.24	0.00	0.00	0.00	0.00	3.74	0.39	0.37	7.88E+00	6.21E-01		null	null		null	null		null	null		-3.26E+00	4.48E-04	Down	-3.33E+00	2.47E-04	Down							
Unigene10860_All	420	0.00	15.92	3.93	0.00	0.16	0.00	0.00	0.00	0.00	1.40E+01	3.29E-38	Up	1.19E+01	4.31E-09	Up	7.36E+00	6.15E-01		null	null		null	null		null	null								
Unigene108600_All	508	0.21	0.00	0.00	0.00	0.00	0.27	2.83	0.41	1.02	-7.69E+00	3.76E-01		-7.69E+00	3.98E-01		null	null		8.06E+00	3.12E-01		-2.78E+00	5.95E-06	Down	-1.47E+00	3.09E-03		Unigene108600_All//gi|297806927|ref|XP_002871347.1|//4.00E-50//positive transcription elongation factor/ zinc ion binding protein [Arabidopsis lyrata subsp. lyrata] >gi|297317184|gb|EFH47606.1| positive transcription elongation factor/ zinc ion binding protein [Arabidopsis lyrata subsp. lyrata]	Unigene108600_All//3.00E-51//AT5G08565| positive transcription elongation factor/ zinc ion binding	Unigene108600_All//5.00E-103//TC154168			GO:0003711//GO:0008135//GO:0010557//GO:0043231//GO:0046914	nr +
Unigene10861_All	394	0.00	0.00	0.00	0.00	0.00	0.00	4.40	8.06	0.00	null	null		null	null		null	null		null	null		8.74E-01	5.01E-03		-1.21E+01	8.37E-13	Down	Unigene10861_All//gi|66270183|gb|AAY43421.1|//6.00E-33//ribosomal protein L34 [Phytophthora infestans]	Unigene10861_All//1.00E-30//AT1G26880| 60S ribosomal protein L34 (RPL34A)			ko03010|Ribosome	GO:0043231	nr +
Unigene108618_All	245	0.00	0.00	0.00	0.00	0.00	0.00	2.42	0.00	0.00	null	null		null	null		null	null		null	null		-1.12E+01	2.40E-04	Down	-1.12E+01	1.42E-04	Down							
Unigene10862_All	342	0.31	0.00	1.09	1.60	2.52	0.00	1.98	1.71	0.00	-8.26E+00	3.76E-01		1.83E+00	2.13E-01		6.52E-01	4.25E-01		-1.06E+01	1.52E-03		-2.09E-01	7.82E-01		-1.09E+01	3.57E-05	Down			Unigene10862_All//5.00E-176//ES836338				
Unigene108620_All	459	0.00	0.00	0.00	0.22	0.00	0.00	2.86	0.27	1.39	null	null		null	null		-7.76E+00	3.17E-01		-7.76E+00	4.05E-01		-3.39E+00	1.90E-06	Down	-1.04E+00	3.96E-02		Unigene108620_All//gi|15239274|ref|NP_201416.1|//1.00E-46//glycosyl hydrolase family 38 protein [Arabidopsis thaliana] >gi|10177130|dbj|BAB10420.1| alpha-mannosidase [Arabidopsis thaliana]	Unigene108620_All//7.00E-48//AT5G66150| glycosyl hydrolase family 38 protein	Unigene108620_All//1.00E-174//TC178171		ko00511|Other glycan degradation	GO:0003676//GO:0005618//GO:0006259//GO:0015923//GO:0016020//GO:0019318//GO:0031410//GO:0043231//GO:0046914	nr +
Unigene108626_All	291	0.00	0.00	0.00	0.00	0.00	0.00	2.18	0.00	0.00	null	null		null	null		null	null		null	null		-1.11E+01	1.24E-04	Down	-1.11E+01	7.12E-05	Down							ESTscan -
Unigene108637_All	247	0.00	0.00	0.00	0.00	0.00	0.00	2.40	1.02	0.00	null	null		null	null		null	null		null	null		-1.24E+00	1.54E-01		-1.12E+01	1.42E-04	Down	Unigene108637_All//gi|145344332|ref|XP_001416689.1|//9.00E-10//Cytosolic 80S ribosomal protein L39; Cytosolic 60S large ribosomal protein L39 [Ostreococcus lucimarinus CCE9901] >gi|144576915|gb|AB094982.1| Cytosolic 80S ribosomal protein L39; Cytosolic 60S large ribosomal protein L39 [Ostreococcus lucimarinus CCE9901]	Unigene108637_All//3.00E-09//AT4G31985| 60S ribosomal protein L39 (RPL39C)			ko03010|Ribosome		nr +
Unigene10864_All	425	0.00	0.00	0.00	0.00	0.00	0.00	2.39	2.66	0.00	null	null		null	null		null	null		null	null		1.53E-01	7.89E-01		-1.12E+01	1.35E-07	Down	Unigene10864_All//gi|299472847|emb|CBN80416.1|//1.00E-07//EsV-1-116 [Ectocarpus siliculosus]						nr -
Unigene108642_All	302	59.08	20.97	50.08	27.38	35.23	24.82	2.66	0.14	0.40	-1.49E+00	9.99E-25	Down	-2.39E-01	9.88E-02		3.64E-01	3.88E-02		-1.41E-01	5.63E-01		-4.26E+00	8.48E-05	Down	-2.75E+00	9.43E-04	Down	Unigene108642_All//gi|62945915|gb|AAY22200.1|//3.00E-28//squalene monooxygenase [Datura inoxia]	Unigene108642_All//3.00E-28//AT2G22830| SQE2 (squalene epoxidase 2); FAD binding / oxidoreductase/ squalene monooxygenase	Unigene108642_All//1.00E-148//TC155314		ko00100|Steroid biosynthesis	GO:0004497//GO:0008152//GO:0030554//GO:0031224	nr +
Unigene108652_All	327	0.32	8.68	1.14	0.00	0.11	0.41	0.39	0.51	0.24	4.76E+00	1.45E-13	Up	1.83E+00	2.13E-01		6.72E+00	8.38E-01		8.69E+00	3.12E-01		3.99E-01	8.15E-01		-6.71E-01	7.21E-01		Unigene108652_All//gi|20197247|gb|AAM14995.1|//5.00E-08//Expressed protein [Arabidopsis thaliana]	Unigene108652_All//2.00E-09//AT2G38465| unknown protein				GO:0016020	nr +
Unigene108654_All	766	0.00	0.00	0.00	0.00	0.00	0.00	2.76	0.82	0.94	null	null		null	null		null	null		null	null		-1.75E+00	3.84E-05	Down	-1.56E+00	1.14E-04	Down	Unigene108654_All//gi|224072801|ref|XP_002303888.1|//4.00E-86//AP2 domain-containing transcription factor [Populus trichocarpa] >gi|222841320|gb|EEE78867.1| AP2 domain-containing transcription factor [Populus trichocarpa]	Unigene108654_All//9.00E-63//AT1G51190| PLT2 (PLETHORA 2); transcription factor	Unigene108654_All//1.00E-12//ES821430	AP2-EREBP		GO:0006350//GO:0010468//GO:0016491//GO:0043231//GO:0048513	nr +
Unigene10866_All	326	0.00	170.19	74.65	0.00	0.00	0.00	0.00	0.00	0.00	1.74E+01	0.00E+00	Up	1.62E+01	9.83E-138	Up	null	null		null	null		null	null		null	null								
Unigene108673_All	445	0.00	0.00	0.00	0.00	0.00	0.00	2.09	0.00	0.00	null	null		null	null		null	null		null	null		-1.10E+01	1.15E-06	Down	-1.10E+01	5.48E-07	Down	Unigene108673_All//gi|300294856|gb|ADJ96635.1|//1.00E-45//S-adenosyl homocysteine hydrolase [Dunaliella salina]	Unigene108673_All//1.00E-40//AT4G13940| MEE58 (MATERNAL EFFECT EMBRYO ARREST 58); adenosylhomocysteinase/ copper ion binding			ko00270|Cysteine and methionine metabolism//ko00450|Selenoamino acid metabolism	GO:0008152//GO:0016787	nr -
Unigene10868_All	270	0.39	0.00	0.39	0.37	3.83	1.34	0.31	0.31	0.15	-8.60E+00	3.76E-01		2.51E-02	1.00E+00		3.37E+00	1.64E-04	Up	1.86E+00	2.20E-01		-1.67E-02	1.02E+00		-1.09E+00	6.77E-01								
Unigene108681_All	414	0.00	0.00	0.00	0.00	0.00	0.00	2.55	1.92	0.00	null	null		null	null		null	null		null	null		-4.13E-01	5.15E-01		-1.13E+01	6.73E-08	Down							
Unigene108685_All	281	0.00	0.00	0.00	0.00	0.00	0.00	1.96	0.45	0.00	null	null		null	null		null	null		null	null		-2.13E+00	3.32E-02		-1.09E+01	2.81E-04	Down							
Unigene108686_All	248	0.00	0.00	0.00	0.80	0.14	0.18	2.73	0.17	0.32	null	null		null	null		-2.53E+00	2.34E-01		-2.14E+00	3.48E-01		-4.02E+00	5.36E-04	Down	-3.09E+00	1.45E-03								
Unigene108689_All	555	0.00	0.00	0.00	1.17	0.12	0.16	1.45	0.15	2.08	null	null		null	null		-3.23E+00	2.49E-03		-2.84E+00	1.02E-02		-3.26E+00	4.48E-04	Down	5.24E-01	3.53E-01		Unigene108689_All//gi|297727877|ref|NP_001176302.1|//6.00E-23//Os11g0106066 [Oryza sativa Japonica Group] >gi|255679693|dbj|BAH95030.1| Os11g0106066 [Oryza sativa Japonica Group]	Unigene108689_All//2.00E-05//AT1G43760| FUNCTIONS IN: molecular_function unknown; INVOLVED IN: biological_process unknown; LOCATED IN: cellular_component unknown; CONTAINS InterPro DOMAIN/s: RNA-directed DNA polymerase (reverse transcriptase), related (InterPro:IPR015706), Endonuclease/exonuclease/phosphatase (InterPro:IPR005135); BEST Arabidopsis thaliana protein match is: unknown protein (TAIR:AT1G40390.1); Has 2614 Blast hits to 2551 proteins in 44 species: Archae - 0; Bacteria - 0; Metazoa - 1517; Fungi - 2; Plants - 1086; Viruses - 0; Other Eukaryotes - 9 (source: NCBI BLink).	Unigene108689_All//2.00E-16//TC145821				nr -
Unigene108696_All	949	0.00	0.00	0.00	0.00	0.00	0.00	5.12	0.31	0.13	null	null		null	null		null	null		null	null		-4.05E+00	9.81E-26	Down	-5.35E+00	3.27E-31	Down	Unigene108696_All//gi|146216004|gb|ABQ10204.1|//3.00E-55//cysteine protease Cp6 [Actinidia deliciosa]	Unigene108696_All//2.00E-44//AT4G23520| cysteine proteinase, putative				GO:0070011	nr +
Unigene10870_All	392	0.00	0.00	0.00	0.00	0.00	0.00	2.70	2.56	0.00	null	null		null	null		null	null		null	null		-7.55E-02	9.09E-01		-1.14E+01	6.73E-08	Down	Unigene10870_All//gi|226503351|ref|NP_001150284.1|//2.00E-47//60S ribosomal protein L23a [Zea mays] >gi|195638090|gb|ACG38513.1| 60S ribosomal protein L23a [Zea mays]	Unigene10870_All//5.00E-45//AT3G55280| RPL23AB (RIBOSOMAL PROTEIN L23AB); RNA binding / nucleotide binding / structural constituent of ribosome	Unigene10870_All//2.00E-15//ES840224		ko03010|Ribosome	GO:0003723//GO:0005198//GO:0010467//GO:0030529//GO:0043231	nr +
Unigene108708_All	400	0.26	0.00	0.40	0.12	0.69	0.11	1.27	0.00	1.00	-8.03E+00	3.76E-01		6.11E-01	7.49E-01		2.47E+00	2.23E-01		-1.41E-01	9.56E-01		-1.03E+01	8.91E-04	Down	-3.49E-01	6.73E-01		Unigene108708_All//gi|22128591|gb|AAM52341.1|//2.00E-06//fertility restorer-like protein [Petunia x hybrida]	Unigene108708_All//2.00E-05//AT1G62590| pentatricopeptide (PPR) repeat-containing protein	Unigene108708_All//2.00E-30//BF268954			GO:0003676//GO:0006260//GO:0034061	nr +
Unigene10871_All	344	3.65	2.65	1.55	21.43	5.71	3.81	8.48	12.39	2.78	-4.61E-01	4.32E-01		-1.24E+00	5.53E-02		-1.91E+00	4.67E-18	Down	-2.49E+00	6.41E-22	Down	5.47E-01	3.91E-02		-1.61E+00	2.29E-06	Down			Unigene10871_All//3.00E-48//FL577497				
Unigene108718_All	300	0.00	0.00	0.00	0.00	0.00	0.00	23.54	9.89	3.85	null	null		null	null		null	null		null	null		-1.25E+00	1.17E-09	Down	-2.61E+00	7.96E-26	Down							
Unigene108722_All	725	0.00	0.00	0.00	0.00	0.00	0.00	7.70	6.05	1.65	null	null		null	null		null	null		null	null		-3.47E-01	1.41E-01		-2.22E+00	3.73E-17	Down	Unigene108722_All//gi|298706692|emb|CBJ34222.1|//6.00E-20//conserved unknown protein [Ectocarpus siliculosus] >gi|298713886|emb|CBJ33762.1| conserved unknown protein [Ectocarpus siliculosus]		Unigene108722_All//6.00E-48//gi|78352089|gb|DT572363.1|DT572363				nr -
Unigene108729_All	554	0.00	0.00	0.00	0.00	0.00	0.00	4.73	0.15	0.36	null	null		null	null		null	null		null	null		-4.97E+00	7.56E-16	Down	-3.72E+00	9.00E-14	Down	Unigene108729_All//gi|118152392|gb|ABK63940.1|//2.00E-63//ribosomal protein L3-A2-II [Triticum aestivum]	Unigene108729_All//4.00E-54//AT1G61580| RPL3B (R-PROTEIN L3 B); structural constituent of ribosome			ko03010|Ribosome	GO:0005198//GO:0010467//GO:0030529	nr +
Unigene10873_All	352	0.00	0.00	0.00	0.00	0.00	0.00	0.24	1.90	5.21	null	null		null	null		null	null		null	null		2.98E+00	2.86E-03		4.44E+00	9.60E-11	Up			Unigene10873_All//3.00E-51//TC140526				
Unigene108734_All	378	0.00	0.00	0.00	0.53	0.36	0.24	3.02	0.00	0.11	null	null		null	null		-5.33E-01	7.49E-01		-1.14E+00	5.70E-01		-1.16E+01	3.89E-08	Down	-4.84E+00	2.34E-07	Down							
Unigene108736_All	538	0.19	0.00	0.00	0.09	0.06	0.00	1.96	0.78	0.30	-7.60E+00	3.76E-01		-7.60E+00	3.98E-01		-5.33E-01	8.28E-01		-6.53E+00	6.44E-01		-1.34E+00	2.90E-02		-2.73E+00	1.12E-04	Down	Unigene108736_All//gi|297596537|ref|NP_001042723.2|//2.00E-15//Os01g0275900 [Oryza sativa Japonica Group] >gi|255673109|dbj|BAF04637.2| Os01g0275900 [Oryza sativa Japonica Group]		Unigene108736_All//8.00E-62//gi|109881497|gb|DW510467.1|DW510467				nr -
Unigene10875_All	1625	0.00	0.00	0.00	0.43	0.04	0.14	5.57	0.46	0.71	null	null		null	null		-3.34E+00	1.25E-03		-1.63E+00	7.45E-02		-3.59E+00	3.22E-43	Down	-2.97E+00	1.32E-37	Down	Unigene10875_All//gi|115447863|ref|NP_001047711.1|//1.00E-127//Os02g0673100 [Oryza sativa Japonica Group] >gi|50251239|dbj|BAD27825.1| aluminum-activated malate transporter-like [Oryza sativa Japonica Group] >gi|50253199|dbj|BAD29455.1| aluminum-activated malate transporter-like [Oryza sativa Japonica Group] >gi|113537242|dbj|BAF09625.1| Os02g0673100 [Oryza sativa Japonica Group]	Unigene10875_All//1.00E-108//AT4G00910| unknown protein	Unigene10875_All//2.00E-46//FL576764				nr +
Unigene108753_All	551	0.00	0.00	0.00	0.00	0.00	0.00	2.30	0.53	0.14	null	null		null	null		null	null		null	null		-2.12E+00	4.17E-04	Down	-3.99E+00	2.56E-07	Down	Unigene108753_All//gi|255070117|ref|XP_002507140.1|//9.00E-18//60S ribosomal protein L36 [Micromonas sp. RCC299] >gi|226522415|gb|ACO68398.1| 60S ribosomal protein L36 [Micromonas sp. RCC299]	Unigene108753_All//3.00E-17//AT2G37600| 60S ribosomal protein L36 (RPL36A)			ko03010|Ribosome	GO:0009536//GO:0010467//GO:0015934	nr -
Unigene108772_All	284	0.18	0.00	0.00	0.88	0.12	0.00	2.53	0.88	0.00	-7.53E+00	6.19E-01		-7.53E+00	6.32E-01		-2.85E+00	1.37E-01		-9.78E+00	7.36E-02		-1.52E+00	5.42E-02		-1.13E+01	1.78E-05	Down			Unigene108772_All//1.00E-31//TC167240				
Unigene108787_All	245	0.00	0.00	0.00	0.00	0.00	0.00	2.42	1.36	0.00	null	null		null	null		null	null		null	null		-8.24E-01	3.48E-01		-1.12E+01	1.42E-04	Down	Unigene108787_All//gi|192910762|gb|ACF06489.1|//1.00E-23//ribosomal protein L10a [Elaeis guineensis]	Unigene108787_All//6.00E-15//AT5G22440| 60S ribosomal protein L10A (RPL10aC)			ko03010|Ribosome	GO:0003676//GO:0005198//GO:0010467//GO:0030529	nr +
Unigene10879_All	518	6.97	14.48	2.47	29.52	30.11	55.80	110.68	79.32	214.42	1.05E+00	1.34E-06	Up	-1.50E+00	2.51E-05	Down	2.86E-02	8.72E-01		9.18E-01	8.33E-20		-4.81E-01	1.05E-14		9.54E-01	1.25E-92		Unigene10879_All//gi|45861745|gb|AAS78671.1|//1.00E-70//actin [Capsicum annuum]	Unigene10879_All//7.00E-71//AT5G09810| ACT7 (ACTIN 7); structural constituent of cytoskeleton	Unigene10879_All//0.00E+00//TC140485		ko04145|Phagosome	GO:0032559//GO:0043232	nr +
Unigene108791_All	292	0.00	0.00	0.00	0.00	0.00	0.00	2.32	0.14	0.14	null	null		null	null		null	null		null	null		-4.02E+00	5.36E-04	Down	-4.09E+00	3.10E-04	Down	Unigene108791_All//gi|75321780|sp|Q5W274.1|PDR3_TOBAC//5.00E-19//RecName: Full=Pleiotropic drug resistance protein 3; AltName: Full=NtPDR3 >gi|55056942|emb|CAH39853.1| PDR-like ABC transporter [Nicotiana tabacum]	Unigene108791_All//6.00E-19//AT1G15210| PDR7 (PLEIOTROPIC DRUG RESISTANCE 7); ATPase, coupled to transmembrane movement of substances				GO:0000166//GO:0016020//GO:0017111	nr +
Unigene108794_All	283	1.11	1.07	0.19	2.82	5.96	1.44	42.58	5.91	2.39	-4.63E-02	9.85E-01		-2.56E+00	1.59E-01		1.08E+00	3.35E-02		-9.72E-01	2.30E-01		-2.85E+00	2.89E-46	Down	-4.15E+00	2.28E-66	Down			Unigene108794_All//5.00E-09//TC156509				

Unigene108795_All	234	0.00	0.00	0.00	0.00	0.00	0.00	2.35	0.36	0.00	null	null		null	null		null	null		null	null		-2.72E+00	1.22E-02		-1.12E+01	2.81E-04	Down							ESTscan +
Unigene108804_All	246	0.00	0.00	0.00	0.00	0.14	0.00	3.27	0.00	0.32	null	null		null	null		7.13E+00	8.38E-01		null	null		-1.17E+01	8.58E-06	Down	-3.33E+00	2.47E-04	Down							
Unigene108806_All	437	0.00	0.00	0.00	0.00	0.00	0.00	2.32	3.06	0.36	null	null		null	null		null	null		null	null		3.98E-01	4.64E-01		-2.67E+00	1.94E-04	Down							
Unigene10881_All	347	0.00	0.15	1.38	0.57	0.20	0.91	46.06	135.14	215.73	7.19E+00	6.21E-01		1.04E+01	7.50E-03		-1.53E+00	4.16E-01		6.66E-01	6.48E-01		1.55E+00	2.02E-82	Up	2.23E+00	7.15E-218	Up	Unigene10881_All//gi|115467322|ref|NP_001057260.1|//1.00E-11//Os06g0239200 [Oryza sativa Japonica Group] >gi|113595300|dbj|BAF19174.1| Os06g0239200 [Oryza sativa Japonica Group]	Unigene10881_All//1.00E-10//AT3G48180| unknown protein					nr -
Unigene10882_All	293	3.57	0.35	0.36	2.04	0.59	1.08	0.58	0.14	0.00	-3.37E+00	2.13E-04	Down	-3.30E+00	4.59E-04	Down	-1.80E+00	5.87E-02		-9.19E-01	3.52E-01		-2.02E+00	3.68E-01		-9.17E+00	1.09E-01		Unigene10882_All//gi|147866156|emb|CAN84129.1|//2.00E-07//hypothetical protein [Vitis vinifera]	Unigene10882_All//2.00E-06//AT5G05330| DNA binding	Unigene10882_All//5.00E-71//ES827558				nr -
Unigene10883_All	380	3.17	0.13	3.78	1.84	2.45	1.78	0.78	0.22	0.31	-4.57E+00	5.88E-06	Down	2.57E-01	6.28E-01		4.15E-01	6.06E-01		-4.19E-02	9.45E-01		-1.82E+00	2.11E-01		-1.31E+00	3.13E-01								
Unigene108833_All	302	0.35	0.00	0.00	0.33	0.11	0.15	2.10	0.00	0.40	-8.44E+00	3.76E-01		-8.44E+00	3.98E-01		-1.53E+00	5.88E-01		-1.14E+00	6.79E-01		-1.10E+01	1.24E-04	Down	-2.41E+00	8.12E-03		Unigene108833_All//gi|297847636|ref|XP_002891699.1|//1.00E-36//glycosyl transferase family 1 protein [Arabidopsis lyrata subsp. lyrata] >gi|297337541|gb|EFH67958.1| glycosyl transferase family 1 protein [Arabidopsis lyrata subsp. lyrata]	Unigene108833_All//4.00E-35//AT1G52420| glycosyl transferase family 1 protein	Unigene108833_All//6.00E-92//TC173745			GO:0008152//GO:0009536//GO:0016740	nr -
Unigene10884_All	449	1.86	1.35	1.54	11.43	11.50	11.37	2.73	5.03	6.48	-4.61E-01	5.43E-01		-2.74E-01	7.05E-01		9.70E-03	9.80E-01		-7.70E-03	9.72E-01		8.80E-01	2.08E-02		1.25E+00	1.47E-04	Up			Unigene10884_All//1.00E-109//DW498751				ESTscan -
Unigene108849_All	634	0.00	0.00	0.00	0.00	0.00	0.00	2.07	0.00	0.88	null	null		null	null		null	null		null	null		-1.10E+01	2.56E-09	Down	-1.23E+00	1.61E-02		Unigene108849_All//gi|4559334|gb|AAD22996.1|//2.00E-50//expressed protein [Arabidopsis thaliana]	Unigene108849_All//4.00E-52//AT2G42570| unknown protein	Unigene108849_All//7.00E-180//gi|78354609|gb|DT574883.1|DT574883			GO:0031410	nr +
Unigene108850_All	396	0.00	2.94	0.00	0.00	0.26	0.11	0.43	0.42	3.12	1.15E+01	1.05E-06	Up	null	null		8.03E+00	4.58E-01		6.83E+00	7.02E-01		-1.66E-02	1.02E+00		2.87E+00	1.78E-05	Up	Unigene108850_All//gi|296081324|emb|CBI17706.3|//4.00E-07//unnamed protein product [Vitis vinifera]	Unigene108850_All//1.00E-06//AT5G55850| NOI	Unigene108850_All//8.00E-55//TC163606				nr +
Unigene108851_All	1062	0.00	0.00	0.00	0.00	0.00	0.00	13.06	0.94	0.26	null	null		null	null		null	null		null	null		-3.79E+00	6.19E-69	Down	-5.64E+00	2.30E-90	Down	Unigene108851_All//gi|149392583|gb|ABR26094.1|//8.00E-06//retrotransposon protein [Oryza sativa Indica Group]		Unigene108851_All//7.00E-27//TC146107				nr -
Unigene108867_All	348	0.00	0.00	0.00	0.00	0.00	0.00	2.55	0.72	0.46	null	null		null	null		null	null		null	null		-1.82E+00	1.08E-02		-2.48E+00	9.93E-04	Down	Unigene108867_All//gi|145349279|ref|XP_001419064.1|//8.00E-13//Ribosomal protein S30, component of cytosolic 80S ribosome and 40S small subunit [Ostreococcus lucimarinus CCE9901] >gi|144579295|gb|ABO97357.1| Ribosomal protein S30, component of cytosolic 80S ribosome and 40S small subunit [Ostreococcus lucimarinus CCE9901]	Unigene108867_All//8.00E-11//AT5G56670| 40S ribosomal protein S30 (RPS30C)			ko03010|Ribosome	GO:0005198//GO:0010467//GO:0030529	nr -
Unigene10887_All	663	0.00	5.12	3.77	0.00	0.00	0.00	0.00	0.00	0.00	1.23E+01	2.55E-19	Up	1.19E+01	8.33E-14	Up	null	null		null	null		null	null		null	null		Unigene10887_All//gi|159483795|ref|XP_001699946.1|//4.00E-07//gamete-specific transcription factor [Chlamydomonas reinhardtii] >gi|158281888|gb|EDP07642.1| gamete-specific transcription factor [Chlamydomonas reinhardtii]		Unigene10887_All//8.00E-07//gi|78326393|gb|DT546667.1|DT546667				nr -
Unigene108870_All	267	0.00	0.00	0.00	0.00	0.00	0.00	1.27	9.08	1.34	null	null		null	null		null	null		null	null		2.84E+00	9.30E-10	Up	8.42E-02	9.58E-01			Unigene108870_All//9.00E-05//AT3G13784| AtcwINV5 (Arabidopsis thaliana cell wall invertase 5); hydrolase, hydrolyzing O-glycosyl compounds	Unigene108870_All//8.00E-14//TC175966				
Unigene108872_All	408	0.00	0.00	0.00	0.00	0.00	0.00	2.07	1.43	0.00	null	null		null	null		null	null		null	null		-5.31E-01	4.45E-01		-1.10E+01	2.21E-06	Down	Unigene108872_All//gi|301105188|ref|XP_002901678.1|//3.00E-30//60S ribosomal protein L32-1 [Phytophthora infestans T30-4] >gi|262100682|gb|EEY58734.1| 60S ribosomal protein L32-1 [Phytophthora infestans T30-4]	Unigene108872_All//3.00E-29//AT4G18100| 60S ribosomal protein L32 (RPL32A)			ko03010|Ribosome	GO:0005198//GO:0010467//GO:0030529	nr +
Unigene108874_All	692	0.00	0.00	0.00	0.00	0.00	0.00	2.44	0.91	0.00	null	null		null	null		null	null		null	null		-1.43E+00	2.16E-03		-1.13E+01	1.70E-12	Down	Unigene108874_All//gi|301111866|ref|XP_002905012.1|//4.00E-40//major vault protein [Phytophthora infestans T30-4] >gi|262095342|gb|EEY53394.1| major vault protein [Phytophthora infestans T30-4]						nr +
Unigene108877_All	285	0.00	0.00	0.00	0.00	0.00	0.00	4.60	10.12	0.00	null	null		null	null		null	null		null	null		1.14E+00	6.59E-04	Up	-1.22E+01	9.85E-10	Down							
Unigene108878_All	248	0.00	0.00	0.00	0.00	0.00	0.00	2.56	0.00	0.32	null	null		null	null		null	null		null	null		-1.13E+01	1.24E-04	Down	-2.99E+00	2.58E-03								ESTscan +
Unigene108879_All	370	0.00	0.00	0.00	0.00	0.00	0.00	1.60	0.00	0.00	null	null		null	null		null	null		null	null		-1.06E+01	2.40E-04	Down	-1.06E+01	1.42E-04	Down							
Unigene10888_All	948	0.00	0.00	0.00	0.00	0.00	0.00	0.45	0.75	5.34	null	null		null	null		null	null		null	null		7.49E-01	3.41E-01		3.58E+00	9.62E-25	Up	Unigene10888_All//gi|297607204|ref|NP_001059610.2|//9.00E-11//Os07g0471900 [Oryza sativa Japonica Group] >gi|255677757|dbj|BAF21524.2| Os07g0471900 [Oryza sativa Japonica Group]	Unigene10888_All//4.00E-10//AT3G20640| ethylene-responsive protein -related	Unigene10888_All//8.00E-57//TC160843	bHLH		GO:0010468//GO:0043231	nr +
Unigene108883_All	410	0.00	0.00	0.00	0.00	0.00	0.00	3.20	0.51	0.00	null	null		null	null		null	null		null	null		-2.65E+00	3.03E-05	Down	-1.16E+01	9.85E-10	Down			Unigene108883_All//1.00E-13//DW489420				
Unigene1089_All	2875	1.64	2.15	2.22	2.34	2.37	2.98	9.51	15.06	22.61	3.93E-01	1.07E-01		4.40E-01	7.68E-02		1.98E-02	9.58E-01		3.52E-01	9.03E-02		6.63E-01	1.69E-19		1.25E+00	6.65E-84	Up	Unigene1089_All//gi|37703718|gb|AAR01224.1|//1.00E-20//cyclin T1 [Medicago truncatula]	Unigene1089_All//2.00E-06//AT5G45190| cyclin family protein	Unigene1089_All//0.00E+00//TC153765			GO:0000904//GO:0007049//GO:0009791//GO:0048827//GO:0050789//GO:0051707	nr -
Unigene108903_All	397	0.00	0.00	0.00	0.00	0.00	0.00	1.81	0.53	0.00	null	null		null	null		null	null		null	null		-1.78E+00	2.79E-02		-1.08E+01	1.78E-05	Down	Unigene108903_All//gi|301114000|ref|XP_002998770.1|//6.00E-38//cell division control protein 42 [Phytophthora infestans T30-4] >gi|262112071|gb|EEY70123.1| cell division control protein 42 [Phytophthora infestans T30-4]	Unigene108903_All//7.00E-28//AT1G20090| ROP2 (RHO-RELATED PROTEIN FROM PLANTS 2); GTP binding			ko04145|Phagosome//ko04650|Natural killer cell mediated cytotoxicity	GO:0000904//GO:0007010//GO:0009314//GO:0010118//GO:0016020//GO:0031981//GO:0032561//GO:0035556//GO:0044444	nr -
Unigene108914_All	379	4.00	0.13	1.26	2.76	3.00	2.50	14.06	6.84	3.89	-4.90E+00	1.23E-07	Down	-1.66E+00	5.58E-03		1.19E-01	8.71E-01		-1.41E-01	8.32E-01		-1.04E+00	8.91E-06	Down	-1.85E+00	3.94E-13	Down			Unigene108914_All//1.00E-118//TC162854				
Unigene108937_All	228	0.00	0.00	0.00	0.00	0.00	0.00	6.49	6.23	0.70	null	null		null	null		null	null		null	null		-5.85E-02	9.19E-01		-3.22E+00	3.28E-07	Down	Unigene108937_All//sp|Q8TGM6|TAR1_YEAST//1.00E-06//Protein TAR1 OS=Saccharomyces cerevisiae GN=TAR1 PE=2 SV=1		Unigene108937_All//1.00E-18//TC146753				swissprot -
Unigene108945_All	217	0.00	0.00	0.00	0.00	0.00	0.00	2.34	0.00	0.00	null	null		null	null		null	null		null	null		-1.12E+01	8.91E-04	Down	-1.12E+01	5.56E-04	Down	Unigene108945_All//gi|300266716|gb|EFJ50902.1|//2.00E-21//elongation factor 2 (EF-2, EF-TU) [Volvox carteri f. nagariensis]	Unigene108945_All//2.00E-21//AT1G56070| LOS1; copper ion binding / translation elongation factor/ translation factor, nucleic acid binding				GO:0008135//GO:0017111//GO:0032561	nr +
Unigene10895_All	244	43.74	12.67	73.98	105.14	152.28	116.24	175.88	45.39	62.53	-1.79E+00	2.67E-19	Down	7.58E-01	1.97E-08		5.34E-01	4.49E-11		1.45E-01	2.17E-01		-1.95E+00	4.32E-104	Down	-1.49E+00	1.98E-73	Down	Unigene10895_All//gi|50313462|gb|AAT74601.1|//9.00E-42//cyclopropane fatty acid synthase [Gossypium hirsutum]	Unigene10895_All//1.00E-26//AT3G23510| cyclopropane fatty acid synthase, putative / CPA-FA synthase, putative	Unigene10895_All//5.00E-129//TC129559			GO:0006629//GO:0008757	nr +
Unigene108954_All	231	0.00	0.00	0.00	0.00	0.00	0.00	2.93	0.72	0.00	null	null		null	null		null	null		null	null		-2.02E+00	1.98E-02		-1.15E+01	3.57E-05	Down	Unigene108954_All//gi|301105535|ref|XP_002901851.1|//2.00E-06//villin-like protein [Phytophthora infestans T30-4] >gi|262099189|gb|EEY57241.1| villin-like protein [Phytophthora infestans T30-4]	Unigene108954_All//7.00E-05//AT3G57410| VLN3 (VILLIN 3); actin binding					nr -
Unigene108955_All	360	0.00	0.00	0.00	0.00	0.00	0.00	2.58	0.93	0.00	null	null		null	null		null	null		null	null		-1.48E+00	2.77E-02		-1.13E+01	5.48E-07	Down	Unigene108955_All//gi|223994887|ref|XP_002287127.1|//3.00E-38//RS18, ribosomal protein 18 40S small ribosomal subunit [Thalassiosira pseudonana CCMP1335] >gi|220976243|gb|EED94570.1| RS18, ribosomal protein 18 40S small ribosomal subunit [Thalassiosira pseudonana CCMP1335]	Unigene108955_All//8.00E-38//AT4G09800| RPS18C (S18 RIBOSOMAL PROTEIN); RNA binding / nucleic acid binding / structural constituent of ribosome			ko03010|Ribosome	GO:0003676//GO:0005198//GO:0006412//GO:0015935//GO:0016020//GO:0030312//GO:0031981	nr +
Unigene108956_All	240	0.00	0.00	0.00	0.00	0.00	0.00	0.53	0.70	5.31	null	null		null	null		null	null		null	null		3.98E-01	8.15E-01		3.33E+00	2.39E-06	Up							
Unigene10896_All	403	2.47	0.25	2.38	3.83	3.50	2.47	0.94	0.62	2.17	-3.29E+00	3.81E-04	Down	-5.26E-02	9.54E-01		-1.29E-01	8.32E-01		-6.36E-01	2.47E-01		-6.02E-01	6.13E-01		1.20E+00	6.68E-02		Unigene10896_All//gi|225447735|ref|XP_002262698.1|//3.00E-45//PREDICTED: similar to somatic embryogenesis receptor-like kinase 3 [Vitis vinifera]	Unigene10896_All//3.00E-34//AT4G33430| BAK1 (BRI1-ASSOCIATED RECEPTOR KINASE); kinase/ protein binding / protein heterodimerization/ protein serine/threonine kinase	Unigene10896_All//6.00E-62//TC146742		ko04626|Plant-pathogen interaction	GO:0002239//GO:0004713//GO:0004871//GO:0006464//GO:0006952//GO:0009555//GO:0016628//GO:0031224//GO:0032559//GO:0032991//GO:0040007//GO:0043231//GO:0043401//GO:0046983	nr +
Unigene108961_All	621	0.00	0.00	0.00	0.00	0.00	0.00	1.36	0.67	0.06	null	null		null	null		null	null		null	null		-1.02E+00	1.41E-01		-4.41E+00	2.37E-05	Down	Unigene108961_All//gi|298715057|emb|CBJ27764.1|//2.00E-09//delta protein [Ectocarpus siliculosus]				ko00520|Amino sugar and nucleotide sugar metabolism		nr +
Unigene10897_All	316	20469.36	7123.71	20124.62	2439.64	2547.67	1786.53	3.61	2.38	0.88	-1.52E+00	0.00E+00	Down	-2.45E-02	1.82E-04		6.25E-02	2.72E-04		-4.50E-01	7.83E-147		-6.02E-01	3.08E-01		-2.03E+00	8.64E-04	Down	Unigene10897_All//gi|29468504|gb|AAO85557.1|//2.00E-39//photosystem I subunit XI [Nicotiana attenuata]	Unigene10897_All//5.00E-37//AT4G12800| PSAL (photosystem I subunit L)	Unigene10897_All//1.00E-173//TC136126		ko00195|Photosynthesis	GO:0006091//GO:0009522//GO:0009526//GO:0009534//GO:0009570//GO:0031224	nr -
Unigene108970_All	307	0.00	0.00	0.00	0.00	0.00	0.00	2.20	0.54	0.00	null	null		null	null		null	null		null	null		-2.02E+00	1.98E-02		-1.11E+01	3.57E-05	Down	Unigene108970_All//gi|4501883|ref|NP_001604.1|//2.00E-32//actin, aortic smooth muscle [Homo sapiens] >gi|6671507|ref|NP_031418.1| actin, aortic smooth muscle [Mus musculus] >gi|78045238|ref|NP_001029674.1| actin, aortic smooth muscle [Bos taurus] >gi|110625958|ref|NP_112266.1| actin, aortic smooth muscle [Rattus norvegicus] >gi|156119362|ref|NP_001095152.1| actin, aortic smooth muscle [Oryctolagus cuniculus] >gi|197097944|ref|NP_001126814.1| actin, aortic smooth muscle [Pongo abelii] >gi|213688375|ref|NP_001135417.1| actin, aortic smooth muscle [Homo sapiens] >gi|257470979|ref|NP_001158122.1| actin, aortic smooth muscle [Sus scrofa] >gi|73996085|ref|XP_534781.2| PREDICTED: similar to Actin, aortic smooth muscle (Alpha-actin 2) [Canis familiaris] >gi|114631685|ref|XP_521550.2|	Unigene108970_All//2.00E-29//AT5G09810| ACT7 (ACTIN 7); structural constituent of cytoskeleton	Unigene108970_All//4.00E-19//TC140956		ko04145|Phagosome	GO:0000267//GO:0003015//GO:0005198//GO:0005884//GO:0006939//GO:0007015//GO:0008015//GO:0008092//GO:0012501//GO:0017111//GO:0030017//GO:0030239//GO:0032432//GO:0032559//GO:0048747//GO:0051707//GO:0060415	nr +

Unigene108978_All	433	0.00	0.00	0.00	0.00	0.00	0.00	1.37	0.39	0.00	null	null		null	null		null	null		null	null		-1.82E+00	4.72E-02		-1.04E+01	1.42E-04	Down	Unigene108978_All//gi|219118192|ref|XP_002179876.1|//5.00E-09//coronin like protein [Phaeodactylum tricornutum CCAP 1055/1] >gi|217408929|gb|EEC48862.1| coronin like protein [Phaeodactylum tricornutum CCAP 1055/1]						nr +
Unigene10898_All	422	0.00	0.36	0.00	0.12	0.98	0.11	0.70	0.50	8.59	8.49E+00	2.36E-01		null	null		3.05E+00	5.68E-02		-1.41E-01	9.56E-01		-5.02E-01	6.78E-01		3.61E+00	4.95E-18	Up	Unigene10898_All//gi|224054334|ref|XP_002298208.1|//3.00E-16//amino acid transporter [Populus trichocarpa] >gi|222845466|gb|EEE83013.1| amino acid transporter [Populus trichocarpa]	Unigene10898_All//3.00E-16//AT1G80510| amino acid transporter family protein	Unigene10898_All//2.00E-09//TC168546				nr -
Unigene108980_All	418	0.25	0.36	0.13	0.72	0.16	0.86	2.63	0.40	0.57	5.39E-01	7.80E-01		-9.74E-01	7.22E-01		-2.12E+00	1.66E-01		2.74E-01	8.33E-01		-2.72E+00	1.31E-04	Down	-2.20E+00	5.66E-04	Down			Unigene108980_All//5.00E-07//EX172114				
Unigene108994_All	681	0.00	0.15	0.00	0.15	0.15	0.07	3.29	0.12	0.00	7.22E+00	3.90E-01		null	null		5.23E-02	9.68E-01		-1.14E+00	6.79E-01		-4.75E+00	2.70E-13	Down	-1.17E+01	1.66E-16	Down	Unigene108994_All//gi|224052970|ref|XP_002297642.1|//2.00E-79//2-oxoglutarate-dependent dioxygenase [Populus trichocarpa] >gi|222844900|gb|EEE82447.1| 2-oxoglutarate-dependent dioxygenase [Populus trichocarpa]	Unigene108994_All//2.00E-78//AT1G55290| oxidoreductase, 2OG-Fe(II) oxygenase family protein	Unigene108994_All//3.00E-34//TC178591		ko00270|Cysteine and methionine metabolism//ko00941|Flavonoid biosynthesis//ko00904|Diterpenoid biosynthesis//ko00402|Benzoxazinoid biosynthesis	GO:0009699//GO:0012501//GO:0016701//GO:0016706	nr -
Unigene108997_All	403	0.39	0.00	0.26	0.25	0.17	0.11	1.78	0.10	0.49	-8.61E+00	2.17E-01		-5.59E-01	7.76E-01		-5.33E-01	8.19E-01		-1.14E+00	6.79E-01		-4.10E+00	2.91E-04	Down	-1.85E+00	1.79E-02		Unigene108997_All//gi|115471355|ref|NP_001059276.1|//2.00E-47//Os07g0243200 [Oryza sativa Japonica Group] >gi|113610812|dbj|BAF21190.1| Os07g0243200 [Oryza sativa Japonica Group]	Unigene108997_All//6.00E-48//AT1G27680| APL2 (ADPGLC-PPASE LARGE SUBUNIT); glucose-1-phosphate adenylyltransferase	Unigene108997_All//2.00E-176//ES801523		ko00500|Starch and sucrose metabolism//ko00520|Amino sugar and nucleotide sugar metabolism	GO:0005977//GO:0005982//GO:0009536//GO:0070566	nr -
Unigene1090_All	981	5.97	8.94	5.81	20.26	35.45	52.30	4.05	11.12	7.47	5.81E-01	2.90E-03		-4.05E-02	8.83E-01		8.07E-01	5.35E-21		1.37E+00	2.15E-65	Up	1.46E+00	4.73E-18	Up	8.83E-01	3.31E-06		Unigene1090_All//gi|42571559|ref|NP_973870.1|//1.00E-111//pathogenesis-related thaumatin family protein [Arabidopsis thaliana] >gi|8778995|gb|AAF79910.1|AC022472_19 Contains similarity to SCUTL1 mRNA from Vitis vinifera gb|AF195653 and is a member of the thaumatin family PF|00314. EST gb|AI995819 comes from this gene [Arabidopsis thaliana]	Unigene1090_All//7.00E-111//AT1G20030| pathogenesis-related thaumatin family protein	Unigene1090_All//0.00E+00//CO118480				nr +
Unigene10900_All	285	135.85	67.38	71.37	244.35	265.33	200.30	214.66	70.69	75.20	-1.01E+00	6.14E-29	Down	-9.29E-01	3.18E-24		1.19E-01	6.43E-02		-2.87E-01	2.67E-06		-1.60E+00	1.53E-112	Down	-1.51E+00	1.52E-106	Down	Unigene10900_All//gi|295389384|dbj|BAJ06362.1|//3.00E-50//alpha-tubulin [Alopecurus aequalis]	Unigene10900_All//1.00E-51//AT4G14960| TUA6; structural constituent of cytoskeleton	Unigene10900_All//6.00E-160//EX171166		ko04145|Phagosome	GO:0007017//GO:0015630//GO:0017111//GO:0032561//GO:0032991//GO:0043623	nr +
Unigene109001_All	261	0.00	0.00	0.00	0.00	0.00	0.00	2.59	0.00	0.00	null	null		null	null		null	null		null	null		-1.13E+01	6.34E-05	Down	-1.13E+01	3.57E-05	Down							
Unigene109002_All	536	0.00	0.00	0.00	0.00	0.00	0.00	3.47	2.81	0.00	null	null		null	null		null	null		null	null		-3.06E-01	5.09E-01		-1.18E+01	9.97E-14	Down	Unigene109002_All//gi|195641182|gb|ACG40059.1|//2.00E-33//60S ribosomal protein L21 [Zea mays]	Unigene109002_All//1.00E-33//AT1G09690| 60S ribosomal protein L21 (RPL21C)			ko03010|Ribosome	GO:0005198//GO:0009536//GO:0010467//GO:0015934//GO:0031981	nr +
Unigene10901_All	360	0.00	0.00	0.00	0.00	0.00	0.00	2.35	1.74	0.00	null	null		null	null		null	null		null	null		-4.32E-01	5.47E-01		-1.12E+01	2.21E-06	Down							ESTscan +
Unigene109011_All	333	0.00	0.00	0.00	0.15	0.00	0.00	36.31	47.32	11.72	null	null		null	null		-7.23E+00	5.63E-01		-7.23E+00	6.44E-01		3.82E-01	2.56E-03		-1.63E+00	3.66E-24	Down	Unigene109011_All//gi|161778780|gb|ABX79341.1|//2.00E-36//cysteine protease [Vitis vinifera]	Unigene109011_All//2.00E-35//AT2G21430| cysteine proteinase A494, putative / thiol protease, putative	Unigene109011_All//8.00E-67//gi|109856138|gb|DW485117.1|DW485117			GO:0004175//GO:0019538	nr -
Unigene109012_All	314	0.00	0.00	0.00	0.00	0.00	0.00	2.02	0.40	0.13	null	null		null	null		null	null		null	null		-2.34E+00	1.27E-02		-3.99E+00	5.82E-04	Down	Unigene109012_All//gi|300120011|emb|CBK19565.2|//2.00E-06//unnamed protein product [Blastocystis hominis]					GO:0043231	nr -
Unigene109014_All	277	0.38	0.00	0.00	0.54	0.00	0.49	2.14	0.00	0.72	-8.56E+00	3.76E-01		-8.56E+00	3.98E-01		-9.08E+00	1.66E-01		-1.42E-01	9.44E-01		-1.11E+01	2.40E-04	Down	-1.57E+00	6.49E-02		Unigene109014_All//gi|225427898|ref|XP_002276690.1|//4.00E-07//PREDICTED: hypothetical protein [Vitis vinifera]						nr +
Unigene109019_All	586	0.09	0.26	0.18	0.26	0.53	0.00	1.52	0.00	0.34	1.54E+00	5.32E-01		1.02E+00	7.04E-01		1.05E+00	5.32E-01		-7.99E+00	2.35E-01		-1.06E+01	2.24E-06	Down	-2.16E+00	2.68E-03		Unigene109019_All//gi|297601293|ref|NP_001050632.2|//6.00E-24//Os03g0604600 [Oryza sativa Japonica Group] >gi|255674692|dbj|BAF12546.2| Os03g0604600 [Oryza sativa Japonica Group]	Unigene109019_All//2.00E-20//AT1G49870| LOCATED IN: chloroplast; EXPRESSED IN: 10 plant structures; EXPRESSED DURING: F mature embryo stage, petal differentiation and expansion stage, D bilateral stage, E expanded cotyledon stage; BEST Arabidopsis thaliana protein match is: MBD10; DNA binding / methyl-CpG binding (TAIR:AT1G15340.1); Has 5602 Blast hits to 4205 proteins in 368 species: Archae - 30; Bacteria - 406; Metazoa - 2889; Fungi - 302; Plants - 153; Viruses - 8; Other Eukaryotes - 1814 (source: NCBI BLink).				GO:0043231	nr +
Unigene10902_All	307	0.00	6.77	5.55	0.00	0.00	0.00	0.00	0.00	0.00	1.27E+01	7.95E-12	Up	1.24E+01	2.20E-09	Up	null	null		null	null		null	null		null	null		Unigene10902_All//sp|P00995|ISK1_HUMAN//7.00E-35//Pancreatic secretory trypsin inhibitor OS=Homo sapiens GN=SPINK1 PE=1 SV=2						swissprot -
Unigene109024_All	354	0.00	0.00	0.00	0.00	0.00	0.00	2.27	0.47	0.00	null	null		null	null		null	null		null	null		-2.26E+00	4.82E-03		-1.11E+01	4.43E-06	Down	Unigene109024_All//gi|15229438|ref|NP_188981.1|//2.00E-34//60S ribosomal protein L36a/L44 (RPL36aA) [Arabidopsis thaliana] >gi|15236469|ref|NP_193168.1| 60S ribosomal protein L36a/L44 (RPL36aB) [Arabidopsis thaliana] >gi|297800816|ref|XP_002868292.1| 60S ribosomal protein L36a/L44 [Arabidopsis lyrata subsp. lyrata] >gi|297831094|ref|XP_002883429.1| 60S ribosomal protein L36a/L44 [Arabidopsis lyrata subsp. lyrata] >gi|3122753|sp|O23290.3|RL36A_ARATH RecName: Full=60S ribosomal protein L36a >gi|2244789|emb|CAB10211.1| ribosomal protein [Arabidopsis thaliana] >gi|7268137|emb|CAB78474.1| ribosomal protein [Arabidopsis thaliana] >gi|11994324|dbj|BAB02283.1| 60S ribosomal protein L44-like [Arabidopsis thaliana] >gi|17065558|gb|AAL32933.1| 60S ribosomal protein L44-like [Arabidopsis thaliana] >gi|17473681|gb|AAL38297.1| ribosomal protein [Arabidopsis	Unigene109024_All//1.00E-35//AT4G14320| 60S ribosomal protein L36a/L44 (RPL36aB)			ko03010|Ribosome	GO:0005198//GO:0010467//GO:0015934	nr +
Unigene109039_All	431	0.00	0.00	0.00	0.00	0.00	0.00	4.32	1.36	2.03	null	null		null	null		null	null		null	null		-1.67E+00	2.33E-04	Down	-1.09E+00	8.60E-03								
Unigene10904_All	270	1.74	4.13	1.18	0.55	0.26	0.67	3.45	9.75	4.13	1.24E+00	6.10E-02		-5.60E-01	6.28E-01		-1.12E+00	5.85E-01		2.74E-01	9.05E-01		1.50E+00	3.94E-05	Up	2.62E-01	6.28E-01				Unigene10904_All//2.00E-51//gi|164300495|gb|ES814990.1|ES814990				
Unigene109048_All	420	0.00	0.00	0.00	0.00	0.00	0.00	3.52	0.70	0.47	null	null		null	null		null	null		null	null		-2.34E+00	3.57E-05	Down	-2.89E+00	1.35E-06	Down	Unigene109048_All//gi|226505998|ref|NP_001146929.1|//3.00E-35//60S ribosomal protein L7a [Zea mays] >gi|195605318|gb|ACG24489.1| 60S ribosomal protein L7a [Zea mays]	Unigene109048_All//1.00E-23//AT3G62870| 60S ribosomal protein L7A (RPL7aB)			ko03010|Ribosome	GO:0005198//GO:0005488//GO:0010467//GO:0022613//GO:0030529	nr -
Unigene10905_All	992	0.00	4.60	1.61	0.00	0.00	0.00	0.00	0.00	0.00	1.22E+01	5.54E-26	Up	1.07E+01	8.44E-09	Up	null	null		null	null		null	null		null	null		Unigene10905_All//gi|300267271|gb|EFJ51455.1|//7.00E-09//CCCH zinc finger protein [Volvox carteri f. nagariensis]					GO:0005488//GO:0044238//GO:0044260	nr -
Unigene109058_All	535	0.00	0.00	0.00	0.00	0.00	0.00	5.22	1.64	0.97	null	null		null	null		null	null		null	null		-1.67E+00	3.67E-06	Down	-2.43E+00	6.89E-10	Down	Unigene109058_All//gi|45477151|sp|Q8RXA8.1|NDK4_SPIOL//3.00E-31//RecName: Full=Nucleoside diphosphate kinase 4, chloroplastic; AltName: Full=Nucleoside diphosphate kinase IV; Short=NDP kinase IV; Short=NDPK IV; Short=NDK IV; AltName: Full=Nucleoside diphosphate kinase III; Flags: Precursor >gi|19699051|gb|AAL91136.1| nucleoside diphosphate kinase III [Spinacia oleracea]	Unigene109058_All//1.00E-31//AT4G23900| nucleoside diphosphate kinase 4 (NDK4)			ko00230|Purine metabolism//ko00240|Pyrimidine metabolism	GO:0009117//GO:0043231//GO:0044444//GO:0046914	nr +
Unigene109061_All	270	0.00	0.00	0.00	0.00	0.00	0.00	2.19	0.31	0.00	null	null		null	null		null	null		null	null		-2.82E+00	7.18E-03		-1.11E+01	1.42E-04	Down	Unigene109061_All//gi|301115126|ref|XP_002905292.1|//9.00E-07//RIB72 protein [Phytophthora infestans T30-4] >gi|262110081|gb|EEY68133.1| RIB72 protein [Phytophthora infestans T30-4]						nr +
Unigene10907_All	278	0.38	0.00	0.00	2.51	0.12	1.62	0.30	0.15	0.57	-8.56E+00	3.76E-01		-8.56E+00	3.98E-01		-4.34E+00	2.80E-04	Down	-6.27E-01	4.76E-01		-1.02E+00	7.26E-01		9.14E-01	6.25E-01		Unigene10907_All//gi|224102125|ref|XP_002312557.1|//2.00E-38//autoinhibited calcium ATPase [Populus trichocarpa] >gi|222852377|gb|EEE89924.1| autoinhibited calcium ATPase [Populus trichocarpa]	Unigene10907_All//7.00E-34//AT3G63380| calcium-transporting ATPase, plasma membrane-type, putative / Ca(2+)-ATPase, putative (ACA12)	Unigene10907_All//8.00E-08//TC163603			GO:0009206//GO:0019829//GO:0031224//GO:0032559//GO:0046873//GO:0070838	nr -
Unigene109072_All	372	0.00	0.00	0.00	0.13	0.00	0.00	1.59	0.00	0.00	null	null		null	null		-7.07E+00	5.63E-01		-7.07E+00	6.44E-01		-1.06E+01	2.40E-04	Down	-1.06E+01	1.42E-04	Down	Unigene109072_All//gi|55168340|gb|AAV44205.1|//3.00E-17//unknow protein [Oryza sativa Japonica Group]		Unigene109072_All//2.00E-27//TC149394				nr -
Unigene109075_All	276	0.95	2.57	3.47	16.42	3.12	3.76	3.22	2.73	0.72	1.44E+00	1.06E-01		1.87E+00	2.06E-02		-2.40E+00	5.62E-15	Down	-2.13E+00	2.40E-11	Down	-2.39E-01	7.21E-01		-2.16E+00	2.68E-03				Unigene109075_All//1.00E-86//DW499907				

Unigene10908_All	997	0.00	0.00	0.00	0.00	0.00	0.00	3.01	1.22	0.12	null	null		null	null		null	null		null	null		-1.31E+00	7.84E-05	Down	-4.65E+00	3.75E-18	Down	Unigene10908_All//gi|115474653|ref|NP_001060923.1|//1.00E-65//Os08g0130500 [Oryza sativa Japonica Group] >gi|730580|sp|P41095.3|RLA0_ORYSJ RecName: Full=60S acidic ribosomal protein P0 >gi|455401|dbj|BAA04668.1| acidic ribosomal protein P0 [Oryza sativa Japonica Group] >gi|29467534|dbj|BAC66723.1| 60S acidic ribosomal protein P0 [Oryza sativa Japonica Group] >gi|113622892|dbj|BAF22837.1| Os08g0130500 [Oryza sativa Japonica Group]	Unigene10908_All//4.00E-52//AT2G40010| 60S acidic ribosomal protein P0 (RPP0A)			ko03010|Ribosome	GO:0005198//GO:0006412//GO:0030529	nr +
Unigene109086_All	260	0.00	0.00	0.00	0.00	0.00	0.00	1.95	0.00	0.15	null	null		null	null		null	null		null	null		-1.09E+01	8.90E-04	Down	-3.67E+00	3.75E-03		Unigene109086_All//sp|Q55FT1|DPYD_DICDI//4.00E-08//Dihydropyrimidine dehydrogenase [NADP+] OS=Dictyostelium discoideum GN=pyd1 PE=2 SV=1						swissprot +
Unigene109090_All	342	0.00	0.00	0.00	0.00	0.00	0.00	1.61	0.00	0.00	null	null		null	null		null	null		null	null		-1.07E+01	4.62E-04	Down	-1.07E+01	2.81E-04	Down	Unigene109090_All//gi|94958151|gb|ABF47216.1|//1.00E-15//cathepsin B [Nicotiana benthamiana]	Unigene109090_All//3.00E-14//AT1G02300| cathepsin B-like cysteine protease, putative				GO:0008233	nr +
Unigene1091_All	801	0.39	0.63	1.20	4.29	6.88	4.85	0.42	2.56	0.80	6.91E-01	4.99E-01		1.61E+00	4.07E-02		6.81E-01	5.50E-03		1.76E-01	6.08E-01		2.60E+00	1.19E-07	Up	9.14E-01	2.47E-01		Unigene1091_All//gi|118485842|gb|ABK94768.1|//2.00E-08//unknown [Populus trichocarpa]		Unigene1091_All//1.00E-117//CO118480				nr -
Unigene10910_All	292	0.00	0.00	3.65	4.44	0.00	0.00	0.00	0.00	0.00	null	null		1.18E+01	6.63E-06	Up	-1.21E+01	1.33E-09	Down	-1.21E+01	4.24E-08	Down	null	null		null	null		Unigene10910_All//gi|548587|sp|P04284.2|PR06_SOLLC//3.00E-51//RecName: Full=Pathogenesis-related leaf protein 6; Short=P6; AltName: Full=Ethylene-induced protein P1; AltName: Full=P14; AltName: Full=P14A; AltName: Full=PR protein; Flags: Precursor >gi|19285|emb|CAA48672.1| P1(p14) protein [Solanum lycopersicum] >gi|170490|gb|AAA03616.1| pathogenesis-related protein P6 [Solanum lycopersicum] >gi|2529165|emb|CAA70042.1| PR protein [Solanum lycopersicum]	Unigene10910_All//5.00E-24//AT2G14580| ATPRB1			ko04626|Plant-pathogen interaction	GO:0001906//GO:0006952	nr -
Unigene109104_All	459	1.37	0.77	2.09	0.54	0.83	2.95	1.11	0.27	0.43	-8.24E-01	3.75E-01		6.10E-01	3.86E-01		6.05E-01	6.59E-01		2.44E+00	2.97E-04	Up	-2.02E+00	5.21E-02		-1.35E+00	1.40E-01				Unigene109104_All//1.00E-94//ES837091				
Unigene109107_All	525	0.20	0.00	0.00	0.00	0.00	0.00	2.42	0.00	0.68	-7.64E+00	3.76E-01		-7.64E+00	3.98E-01		null	null		null	null		-1.12E+01	5.06E-09	Down	-1.82E+00	1.09E-03		Unigene109107_All//gi|18265373|dbj|BAB84015.1|//3.00E-43//polyprotein [Arabidopsis thaliana]	Unigene109107_All//1.00E-28//ATMG00810| hypothetical protein	Unigene109107_All//1.00E-29//EX169483			GO:0003676	nr -
Unigene109109_All	245	0.00	0.00	0.00	0.00	0.00	0.00	3.28	0.17	0.33	null	null		null	null		null	null		null	null		-4.26E+00	8.48E-05	Down	-3.33E+00	2.47E-04	Down							
Unigene10911_All	411	0.00	2.10	2.33	0.00	0.00	0.00	0.00	0.00	0.00	1.10E+01	4.95E-05	Up	1.12E+01	2.46E-05	Up	null	null		null	null		null	null		null	null								ESTscan -
Unigene109116_All	1080	0.68	0.23	0.00	0.32	0.41	0.08	1.72	0.35	0.30	-1.53E+00	7.78E-02		-9.41E+00	4.13E-04	Down	3.60E-01	8.07E-01		-1.95E+00	1.96E-01		-2.31E+00	3.11E-06	Down	-2.55E+00	3.55E-07	Down	Unigene109116_All//gi|30694495|ref|NP_849787.1|//3.00E-40//WNK7; kinase/ protein kinase [Arabidopsis thaliana] >gi|75246559|sp|Q8LST2.1|WNK7_ARATH RecName: Full=Probable serine/threonine-protein kinase WNK7; Short=AtWNK7; AltName: Full=Protein kinase with no lysine 7 >gi|21039014|dbj|BAB92986.1| protein kinase [Arabidopsis thaliana] >gi|222423633|dbj|BAH19785.1| AT1G49160 [Arabidopsis thaliana]	Unigene109116_All//7.00E-37//AT3G18750| WNK6 (WITH NO K (=LYSINE) 6); kinase/ protein kinase	Unigene109116_All//1.00E-49//TC164044		ko04712|Circadian rhythm - plant	GO:0004702//GO:0006464//GO:0032559	nr -
Unigene10912_All	319	0.00	9.69	3.17	0.00	0.00	0.00	0.00	0.00	0.00	1.32E+01	1.38E-17	Up	1.16E+01	1.27E-05	Up	null	null		null	null		null	null		null	null		Unigene10912_All//gi|301105781|ref|XP_002901974.1|//4.00E-13//DnaJ subfamily C protein [Phytophthora infestans T30-4] >gi|262099312|gb|EEY57364.1| DnaJ subfamily C protein [Phytophthora infestans T30-4]				ko04141|Protein processing in endoplasmic reticulum	GO:0005515	nr -
Unigene109125_All	258	0.00	0.00	0.00	0.00	0.00	0.00	3.61	0.32	0.00	null	null		null	null		null	null		null	null		-3.48E+00	7.79E-05	Down	-1.18E+01	5.48E-07	Down							ESTscan +
Unigene109126_All	428	1.34	0.95	1.12	1.63	0.64	0.21	1.98	0.68	0.28	-5.06E-01	6.00E-01		-2.64E-01	7.67E-01		-1.34E+00	1.09E-01		-2.95E+00	5.84E-03		-1.53E+00	3.21E-02		-2.82E+00	5.38E-04	Down	Unigene109126_All//gi|124359162|gb|ABN05685.1|//7.00E-10//RNA-directed DNA polymerase (Reverse transcriptase) [Medicago truncatula]	Unigene109126_All//3.00E-05//AT3G09510| nucleic acid binding	Unigene109126_All//2.00E-43//ES818849				nr +
Unigene10913_All	364	0.00	2.51	5.71	0.00	0.00	0.00	0.00	0.00	0.00	1.13E+01	2.62E-05	Up	1.25E+01	1.93E-11	Up	null	null		null	null		null	null		null	null		Unigene10913_All//gi|9858814|gb|AAG01147.1|AF283816_1//3.00E-32//calreticulin [Pinus taeda]	Unigene10913_All//5.00E-27//AT1G09210| calreticulin 2 (CRT2)			ko04141|Protein processing in endoplasmic reticulum//ko04145|Phagosome	GO:0006874//GO:0031410//GO:0044432//GO:0046872	nr -
Unigene109131_All	1836	0.00	0.00	0.00	0.11	0.00	0.00	2.16	2.85	4.51	null	null		null	null		-6.76E+00	8.47E-02		-6.76E+00	1.34E-01		3.95E-01	1.03E-01		1.06E+00	5.06E-09	Up	Unigene109131_All//gi|115435462|ref|NP_001042489.1|//1.00E-142//Os01g0229700 [Oryza sativa Japonica Group] >gi|113532020|dbj|BAF04403.1| Os01g0229700 [Oryza sativa Japonica Group]	Unigene109131_All//1.00E-35//AT3G06880| nucleotide binding	Unigene109131_All//0.00E+00//gi|164312745|gb|ES848791.1|ES848791		ko03040|Spliceosome	GO:0019787//GO:0032446//GO:0043234//GO:0055086	nr -
Unigene109134_All	252	0.00	0.00	0.00	0.00	0.00	0.00	3.52	1.00	0.16	null	null		null	null		null	null		null	null		-1.82E+00	1.08E-02		-4.48E+00	1.23E-05	Down	Unigene109134_All//gi|300261259|gb|EFJ45473.1|//1.00E-25//component of cytosolic 80S ribosome and 60S large subunit [Volvox carteri f. nagariensis]	Unigene109134_All//2.00E-25//AT5G60670| 60S ribosomal protein L12 (RPL12C)			ko03010|Ribosome	GO:0003723//GO:0005198//GO:0010467//GO:0030529	nr +
Unigene109137_All	623	0.00	0.00	0.00	0.00	0.00	0.00	7.13	5.50	0.19	null	null		null	null		null	null		null	null		-3.73E-01	1.64E-01		-5.22E+00	3.22E-28	Down	Unigene109137_All//gi|253759791|ref|XP_002488947.1|//1.00E-10//hypothetical protein SORBIDRAFT_1368s002010 [Sorghum bicolor] >gi|241947119|gb|EES20264.1| hypothetical protein SORBIDRAFT_1368s002010 [Sorghum bicolor]		Unigene109137_All//4.00E-36//TC148521				nr +
Unigene109138_All	297	0.00	0.00	0.00	0.00	0.00	0.00	2.42	0.42	0.00	null	null		null	null		null	null		null	null		-2.52E+00	4.69E-03		-1.12E+01	1.78E-05	Down							
Unigene109142_All	476	0.00	0.00	0.00	0.00	0.00	0.00	1.51	0.09	0.00	null	null		null	null		null	null		null	null		-4.10E+00	2.91E-04	Down	-1.06E+01	1.78E-05	Down	Unigene109142_All//gi|297801916|ref|XP_002868842.1|//3.00E-11//patched family protein [Arabidopsis lyrata subsp. lyrata] >gi|297314678|gb|EFH45101.1| patched family protein [Arabidopsis lyrata subsp. lyrata]						nr +
Unigene109144_All	241	0.00	0.00	0.00	0.41	0.14	0.00	4.39	1.73	0.17	null	null		null	null		-1.53E+00	5.88E-01		-8.69E+00	4.05E-01		-1.34E+00	2.90E-02		-4.73E+00	8.85E-07	Down							
Unigene109146_All	395	0.00	0.00	0.00	0.00	0.00	0.00	2.68	0.00	0.10	null	null		null	null		null	null		null	null		-1.14E+01	1.51E-07	Down	-4.73E+00	8.85E-07	Down	Unigene109146_All//gi|223452518|gb|ACM89586.1|//2.00E-28//leucine-rich repeat family protein [Glycine max]	Unigene109146_All//7.00E-27//AT5G66330| leucine-rich repeat family protein	Unigene109146_All//3.00E-153//gi|78335416|gb|DT555690.1|DT555690			GO:0016491//GO:0023060	nr +
Unigene10915_All	294	3.03	0.00	3.62	0.34	0.00	0.00	0.58	0.57	0.41	-1.16E+01	2.86E-05	Down	2.60E-01	6.82E-01		-8.40E+00	3.17E-01		-8.40E+00	4.05E-01		-1.66E-02	1.02E+00		-5.01E-01	7.47E-01				Unigene10915_All//3.00E-07//TC153528				
Unigene109158_All	1047	0.00	0.00	0.00	0.10	0.00	0.09	0.44	0.40	1.98	null	null		null	null		-6.57E+00	3.17E-01		-1.42E-01	9.53E-01		-1.54E-01	8.70E-01		2.16E+00	1.54E-06	Up							
Unigene10916_All	923	0.00	0.00	0.00	0.00	0.00	0.00	6.73	1.95	0.86	null	null		null	null		null	null		null	null		-1.79E+00	4.95E-14	Down	-2.96E+00	5.25E-26	Down	Unigene10916_All//gi|3377952|emb|CAA08906.1|//1.00E-64//cysteine proteinase [Cicer arietinum]	Unigene10916_All//1.00E-60//AT4G35350| XCP1 (XYLEM CYSTEINE PEPTIDASE 1); cysteine-type endopeptidase/ cysteine-type peptidase				GO:0016787	nr +
Unigene109161_All	279	70.70	52.49	26.53	93.20	131.57	117.20	72.13	1093.73	156.93	-4.30E-01	5.39E-04		-1.41E+00	3.68E-24	Down	4.97E-01	1.13E-09		3.31E-01	3.96E-04		3.92E+00	0.00E+00	Up	1.12E+00	5.40E-48	Up	Unigene109161_All//gi|59896631|gb|AAX11684.1|//8.00E-06//perakine reductase [Rauvolfia serpentina]		Unigene109161_All//5.00E-154//TC135928			GO:0003824//GO:0008152	nr +
Unigene109163_All	397	0.00	0.00	0.00	0.00	0.00	0.00	2.34	0.00	0.00	null	null		null	null		null	null		null	null		-1.12E+01	1.15E-06	Down	-1.12E+01	5.48E-07	Down			Unigene109163_All//5.00E-07//ES792745				
Unigene109164_All	297	0.00	0.00	0.00	0.00	0.00	0.00	1.71	0.56	0.00	null	null		null	null		null	null		null	null		-1.60E+00	1.07E-01		-1.07E+01	5.56E-04	Down			Unigene109164_All//9.00E-08//TC159777				
Unigene109167_All	462	0.00	0.00	0.00	0.00	0.00	0.00	2.47	0.09	0.09	null	null		null	null		null	null		null	null		-4.77E+00	5.39E-07	Down	-4.84E+00	2.34E-07	Down							
Unigene109175_All	233	0.00	0.00	0.00	0.00	0.00	0.00	0.00	0.18	2.56	null	null		null	null		null	null		null	null		7.49E+00	6.38E-01		1.13E+01	1.76E-04	Up	Unigene109175_All//gi|15235905|ref|NP_194034.1|//2.00E-06//MATE efflux protein-related [Arabidopsis thaliana]						nr +
Unigene109177_All	431	0.00	0.00	0.00	0.00	0.00	0.00	1.37	1.26	0.00	null	null		null	null		null	null		null	null		-1.24E-01	8.84E-01		-1.04E+01	1.42E-04	Down	Unigene109177_All//gi|111379068|gb|ABH09321.1|//6.00E-11//leucine rich protein [Arachis hypogaea]		Unigene109177_All//3.00E-08//DW479423				nr -
Unigene109182_All	385	0.00	0.00	0.00	0.00	0.00	0.00	3.07	0.54	0.31	null	null		null	null		null	null		null	null		-2.50E+00	1.47E-04	Down	-3.31E+00	4.89E-06	Down	Unigene109182_All//gi|3885515|gb|AAC77928.1|//7.00E-18//similar to ribosomal protein S26 [Medicago sativa]	Unigene109182_All//2.00E-12//AT3G56340| 40S ribosomal protein S26 (RPS26C)			ko03010|Ribosome	GO:0005198//GO:0009536//GO:0010467//GO:0015935	nr -
Unigene109185_All	711	0.22	0.00	0.00	1.05	0.19	0.19	4.16	0.12	0.50	-7.79E+00	2.17E-01		-7.79E+00	2.46E-01		-2.44E+00	5.60E-03		-2.46E+00	1.02E-02		-5.15E+00	3.87E-18	Down	-3.05E+00	4.39E-13	Down			Unigene109185_All//7.00E-63//CO490797				
Unigene109186_All	403	0.00	0.00	0.00	0.00	0.00	0.00	2.62	0.52	0.20	null	null		null	null		null	null		null	null		-2.34E+00	6.78E-04	Down	-3.73E+00	6.14E-06	Down							
Unigene109189_All	288	0.00	0.00	0.00	0.00	0.00	0.00	1.17	5.81	0.00	null	null		null	null		null	null		null	null		2.31E+00	1.19E-05	Up	-1.02E+01	8.17E-03								
Unigene109196_All	285	0.00	0.00	0.00	0.00	0.00	0.00	2.67	0.00	0.84	null	null		null	null		null	null		null	null		-1.14E+01	1.68E-05	Down	-1.67E+00	2.39E-02		Unigene109196_All//gi|224141315|ref|XP_002324019.1|//2.00E-20//multidrug/pheromone exporter, MDR family, ABC transporter family [Populus trichocarpa] >gi|222867021|gb|EEF04152.1| multidrug/pheromone exporter, MDR family, ABC transporter family [Populus trichocarpa]	Unigene109196_All//2.00E-21//AT3G28390| PGP18 (P-GLYCOPROTEIN 18); ATPase, coupled to transmembrane movement of substances			ko02010|ABC transporters	GO:0006810//GO:0031224//GO:0032559//GO:0042626	nr +
Unigene109204_All	272	0.00	0.00	0.00	0.00	0.00	0.00	2.18	1.84	0.00	null	null		null	null		null	null		null	null		-2.39E-01	7.65E-01		-1.11E+01	1.42E-04	Down	Unigene109204_All//gi|297838233|ref|XP_002886998.1|//7.00E-07//expressed protein [Arabidopsis lyrata subsp. lyrata] >gi|297332839|gb|EFH63257.1| expressed protein [Arabidopsis lyrata subsp. lyrata]					GO:0016462//GO:0032559	nr -
Unigene109209_All	433	0.12	0.12	1.97	0.12	0.00	0.00	0.88	0.00	0.09	-4.61E-02	9.87E-01		4.03E+00	7.02E-04	Up	-6.85E+00	5.62E-01		-6.85E+00	6.44E-01		-9.78E+00	6.19E-03		-3.26E+00	2.22E-02		Unigene109209_All//gi|4894211|gb|AAD32302.1|//1.00E-36//ribonucleotide reductase small subunit [Glycine max]	Unigene109209_All//6.00E-37//AT3G23580| RNR2A (RIBONUCLEOTIDE REDUCTASE 2A); ribonucleoside-diphosphate reductase	Unigene109209_All//4.00E-131//gi|164251877|gb|ES849561.1|ES849561		ko00230|Purine metabolism//ko00240|Pyrimidine metabolism//ko00480|Glutathione metabolism	GO:0006259//GO:0007049//GO:0009132//GO:0016728//GO:0016757//GO:0031224//GO:0032501//GO:0043234//GO:0046872	nr +
Unigene109225_All	263	0.00	0.00	0.00	0.00	0.00	0.00	2.09	0.32	0.00	null	null		null	null		null	null		null	null		-2.72E+00	1.22E-02		-1.10E+01	2.81E-04	Down							
Unigene109231_All	324	0.32	0.00	0.00	0.00	0.74	0.14	2.48	0.52	0.37	-8.34E+00	3.76E-01		-8.34E+00	3.98E-01		9.54E+00	1.04E-01		7.12E+00	7.02E-01		-2.26E+00	4.82E-03		-2.75E+00	9.43E-04	Down							
Unigene109234_All	427	0.00	0.00	0.00	0.00	0.00	0.00	4.75	0.59	1.03	null	null		null	null		null	null		null	null		-3.02E+00	9.07E-09	Down	-2.21E+00	1.05E-06	Down			Unigene109234_All//3.00E-14//TC150240				

Unigene109243_All	545	0.00	0.00	0.00	0.00	0.00	0.00	2.95	0.00	0.15	null	null		null	null		null	null		null	null		-1.15E+01	2.17E-11	Down	-4.33E+00	1.41E-09	Down	Unigene109243_All//gi|159464339|ref|XP_001690399.1|//2.00E-06//fibrocystin-L-like protein [Chlamydomonas reinhardtii] >gi|158279899|gb|EDP05658.1| fibrocystin-L-like protein [Chlamydomonas reinhardtii]					GO:0044464	nr +
Unigene109245_All	492	0.00	0.00	0.00	0.00	0.00	0.00	2.58	1.10	0.65	null	null		null	null		null	null		null	null		-1.22E+00	2.45E-02		-1.99E+00	4.87E-04	Down	Unigene109245_All//gi|159479982|ref|XP_001698065.1|//4.00E-50//receptor of activated protein kinase C 1 [Chlamydomonas reinhardtii] >gi|121026|sp|P25387.1|GBLP_CHLRE RecName: Full=Guanine nucleotide-binding protein subunit beta-like protein >gi|158273864|gb|EDO99650.1| receptor of activated protein kinase C 1 [Chlamydomonas reinhardtii]	Unigene109245_All//4.00E-46//AT1G18080| ATARCA; nucleotide binding			ko03040|Spliceosome//ko03022|Basal transcription factors	GO:0004683//GO:0004871	nr +
Unigene109247_All	342	0.00	0.00	0.00	0.00	0.00	0.00	3.21	0.98	0.00	null	null		null	null		null	null		null	null		-1.72E+00	5.68E-03		-1.17E+01	3.33E-08	Down	Unigene109247_All//gi|13359451|dbj|BAB33421.1|//7.00E-07//putative senescence-associated protein [Pisum sativum]		Unigene109247_All//1.00E-10//gi|84157990|gb|DN827037.1|DN827037				nr -
Unigene10925_All	209	40.55	81.46	49.43	53.87	46.30	58.99	70.00	49.99	52.04	1.01E+00	5.39E-13	Up	2.86E-01	1.42E-01		-2.18E-01	2.33E-01		1.31E-01	4.98E-01		-4.86E-01	1.97E-04		-4.28E-01	7.83E-04		Unigene10925_All//gi|195641908|gb|ACG40422.1|//7.00E-07//cytochrome b5 [Zea mays]	Unigene10925_All//9.00E-07//AT5G53560| CB5-E (CYTOCHROME B5 ISOFORM E); heme binding	Unigene10925_All//8.00E-103//TC157945			GO:0005506//GO:0008152//GO:0008940//GO:0016020	nr +
Unigene109251_All	344	0.00	0.00	0.00	0.43	0.10	0.00	1.47	0.24	0.00	null	null		null	null		-2.12E+00	3.87E-01		-8.76E+00	2.35E-01		-2.60E+00	2.05E-02		-1.05E+01	5.56E-04	Down			Unigene109251_All//9.00E-70//gi|21098584|gb|BQ410897.1|BQ410897				
Unigene109252_All	369	0.00	0.00	0.00	0.27	0.00	0.00	1.72	0.34	0.00	null	null		null	null		-8.08E+00	3.17E-01		-8.08E+00	4.05E-01		-2.34E+00	1.27E-02		-1.07E+01	7.12E-05	Down	Unigene109252_All//gi|164564729|dbj|BAF98211.1|//4.00E-13//CM0216.240.nc [Lotus japonicus]	Unigene109252_All//3.00E-07//AT3G51400| unknown protein					nr +
Unigene109253_All	248	0.00	0.00	0.00	0.00	0.00	0.00	2.22	0.34	0.00	null	null		null	null		null	null		null	null		-2.72E+00	1.22E-02		-1.11E+01	2.81E-04	Down							
Unigene109256_All	645	0.00	0.39	0.33	0.31	0.32	0.63	1.97	0.39	1.24	8.62E+00	7.88E-02		8.37E+00	1.42E-01		5.23E-02	9.89E-01		1.03E+00	4.29E-01		-2.34E+00	1.56E-04	Down	-6.71E-01	1.95E-01		Unigene109256_All//gi|297800328|ref|XP_002868048.1|//1.00E-39//tRNA/rRNA methyltransferase family protein [Arabidopsis lyrata subsp. lyrata] >gi|297313884|gb|EFH44307.1| tRNA/rRNA methyltransferase family protein [Arabidopsis lyrata subsp. lyrata]	Unigene109256_All//9.00E-36//AT4G17610| tRNA/rRNA methyltransferase (SpoU) family protein				GO:0003676//GO:0008168//GO:0010467	nr +
Unigene109261_All	463	0.00	0.00	0.00	0.00	0.00	0.00	1.37	0.72	0.00	null	null		null	null		null	null		null	null		-9.24E-01	2.63E-01		-1.04E+01	7.12E-05	Down			Unigene109261_All//4.00E-17//ES794483				
Unigene109269_All	402	0.00	0.00	0.00	0.00	0.00	0.00	3.89	1.04	0.20	null	null		null	null		null	null		null	null		-1.90E+00	2.25E-04	Down	-4.30E+00	2.71E-09	Down	Unigene109269_All//sp|Q86HV8|CTNC_DICDI//1.00E-12//Countin-3 OS=Dictyostelium discoideum GN=ctnC PE=2 SV=1						swissprot -
Unigene109271_All	656	0.00	0.00	0.00	0.00	0.00	0.00	3.22	0.13	0.12	null	null		null	null		null	null		null	null		-4.66E+00	1.89E-12	Down	-4.73E+00	4.77E-13	Down	Unigene109271_All//gi|225463468|ref|XP_002276545.1|//8.00E-43//PREDICTED: similar to RPL18AA (60S RIBOSOMAL PROTEIN L18A-1); structural constituent of ribosome isoform 2 [Vitis vinifera]	Unigene109271_All//4.00E-42//AT2G34480| 60S ribosomal protein L18A (RPL18aB)			ko03010|Ribosome		nr -
Unigene109272_All	246	11.91	2.88	11.91	4.05	2.38	7.89	0.52	0.17	0.16	-2.05E+00	7.98E-07	Down	-6.00E-04	9.99E-01		-7.67E-01	2.60E-01		9.63E-01	4.20E-02		-1.60E+00	5.35E-01		-1.67E+00	4.78E-01				Unigene109272_All//7.00E-39//gi|48878782|gb|CO129804.1|CO129804				
Unigene109280_All	1083	0.05	0.28	0.00	0.18	0.25	0.08	0.90	0.00	0.52	2.54E+00	1.51E-01		-5.59E+00	6.32E-01		4.67E-01	8.17E-01		-1.14E+00	5.70E-01		-9.81E+00	5.84E-07	Down	-8.02E-01	1.83E-01		Unigene109280_All//gi|10176937|dbj|BAB10136.1|//7.00E-76//serine/threonine-specific kinase like protein [Arabidopsis thaliana]	Unigene109280_All//1.00E-74//AT5G55830| lectin protein kinase, putative	Unigene109280_All//4.00E-90//CO115789		ko04626|Plant-pathogen interaction	GO:0004702//GO:0006464//GO:0016020//GO:0030246//GO:0031410//GO:0032559//GO:0046914	nr -
Unigene109281_All	265	0.39	0.00	0.40	0.38	0.00	0.00	1.91	0.00	0.15	-8.63E+00	3.76E-01		2.53E-02	1.00E+00		-8.55E+00	3.17E-01		-8.55E+00	4.05E-01		-1.09E+01	8.90E-04	Down	-3.67E+00	3.75E-03								
Unigene109284_All	595	0.00	0.00	0.00	0.00	0.00	0.00	0.07	0.07	1.21	null	null		null	null		null	null		null	null		-1.84E-02	1.01E+00		4.08E+00	2.41E-04	Up	Unigene109284_All//gi|86155935|gb|ABC86705.1|//1.00E-14//R111 [Coffea arabica]	Unigene109284_All//9.00E-13//AT5G12010| unknown protein	Unigene109284_All//0.00E+00//TC140933			GO:0009536	nr +
Unigene109294_All	399	0.00	0.00	0.00	0.00	0.00	0.00	2.65	0.10	0.00	null	null		null	null		null	null		null	null		-4.66E+00	1.94E-06	Down	-1.14E+01	6.72E-08	Down	Unigene109294_All//gi|3059122|emb|CAA06030.1|//2.00E-50//glyeraldehyde-3-phosphate dehydrogenase [Marsilea quadrifolia]	Unigene109294_All//1.00E-49//AT1G79530| GAPCP-1 (GLYCERALDEHYDE-3-PHOSPHATE DEHYDROGENASE OF PLASTID 1); NAD or NADH binding / binding / catalytic/ glyceraldehyde-3-phosphate dehydrogenase (phosphorylating)/ glyceraldehyde-3-phosphate dehydrogenase	Unigene109294_All//1.00E-16//gi|84169252|gb|DR457900.1|DR457900		ko00010|Glycolysis / Gluconeogenesis	GO:0000166//GO:0006007//GO:0008943//GO:0043231	nr +
Unigene109295_All	386	0.00	0.00	0.00	0.00	0.00	0.00	3.51	0.11	0.00	null	null		null	null		null	null		null	null		-5.02E+00	2.11E-08	Down	-1.18E+01	4.86E-10	Down	Unigene109295_All//gi|12229935|sp|Q9SMI3.1|RS12_CYAPA//6.00E-29//RecName: Full=40S ribosomal protein S12 >gi|6012956|emb|CAB57311.1| 40s ribosomal protein S12 [Cyanophora paradoxa]	Unigene109295_All//5.00E-20//AT2G32060| 40S ribosomal protein S12 (RPS12C)			ko03010|Ribosome	GO:0030529//GO:0043229//GO:0044444	nr +
Unigene1093_All	1433	11.79	6.22	7.36	11.54	8.80	6.02	3.22	0.26	0.75	-9.22E-01	2.59E-11		-6.81E-01	1.10E-06		-3.92E-01	2.56E-03		-9.39E-01	3.96E-12		-3.61E+00	2.07E-22	Down	-2.10E+00	2.30E-13	Down	Unigene1093_All//gi|227204257|dbj|BAH56980.1|//1.00E-102//AT1G75800 [Arabidopsis thaliana]	Unigene1093_All//9.00E-103//AT1G20030| pathogenesis-related thaumatin family protein	Unigene1093_All//0.00E+00//gi|78323547|gb|DT543821.1|DT543821				nr +
Unigene10930_All	599	0.35	1.10	2.67	0.58	0.40	0.30	0.00	0.00	0.00	1.65E+00	8.14E-02		2.93E+00	2.09E-05	Up	-5.33E-01	6.46E-01		-9.49E-01	4.81E-01		null	null		null	null		Unigene10930_All//gi|255591021|ref|XP_002535422.1|//3.00E-30//conserved hypothetical protein [Ricinus communis] >gi|223523171|gb|EEF26965.1| conserved hypothetical protein [Ricinus communis]	Unigene10930_All//2.00E-05//AT4G03140| binding / catalytic/ oxidoreductase				GO:0003824	nr -
Unigene109305_All	324	0.00	0.00	0.00	0.00	0.00	0.00	10.57	5.16	14.26	null	null		null	null		null	null		null	null		-1.03E+00	5.63E-04	Down	4.32E-01	8.00E-02		Unigene109305_All//gi|15230603|ref|NP_190094.1|//9.00E-23//sulfotransferase family protein [Arabidopsis thaliana] >gi|6911846|emb|CAB72146.1| sulfotransferase-like protein [Arabidopsis thaliana] >gi|50198823|gb|AAT70444.1| At3g45080 [Arabidopsis thaliana] >gi|52421317|gb|AAU45228.1| At3g45080 [Arabidopsis thaliana]	Unigene109305_All//4.00E-24//AT3G45080| sulfotransferase family protein	Unigene109305_All//1.00E-99//gi|48804834|gb|CO106148.1|CO106148		ko00380|Tryptophan metabolism//ko00966|Glucosinolate biosynthesis	GO:0008146	nr -
Unigene109307_All	479	0.11	0.00	0.00	0.83	0.22	0.00	2.03	0.44	0.17	-6.77E+00	6.19E-01		-6.77E+00	6.32E-01		-1.95E+00	1.19E-01		-9.70E+00	1.11E-02		-2.22E+00	1.82E-03		-3.61E+00	2.14E-05	Down							
Unigene10931_All	237	0.00	0.00	0.00	0.00	0.00	0.00	0.18	1.23	2.86	null	null		null	null		null	null		null	null		2.79E+00	9.49E-02		4.00E+00	4.30E-04	Up							
Unigene109313_All	241	0.00	0.00	0.00	0.00	0.00	0.00	2.28	0.17	0.00	null	null		null	null		null	null		null	null		-3.72E+00	3.23E-03		-1.12E+01	2.81E-04	Down							
Unigene109318_All	1489	0.00	0.00	0.00	0.13	0.00	0.00	3.55	2.58	1.26	null	null		null	null		-7.06E+00	8.47E-02		-7.06E+00	1.34E-01		-4.59E-01	5.23E-02		-1.50E+00	7.58E-10	Down	Unigene109318_All//gi|225425912|ref|XP_002267341.1|//1.00E-107//PREDICTED: similar to F21J9.9 [Vitis vinifera]	Unigene109318_All//4.00E-94//AT1G24430| transferase/ transferase, transferring acyl groups other than amino-acyl groups	Unigene109318_All//0.00E+00//TC142154		ko00940|Phenylpropanoid biosynthesis//ko00945|Stilbenoid, diarylheptanoid and gingerol biosynthesis//ko00941|Flavonoid biosynthesis	GO:0016740	nr +
Unigene10932_All	328	0.00	0.77	0.00	0.30	0.52	0.55	3.22	2.04	0.49	9.59E+00	7.88E-02		null	null		7.89E-01	7.72E-01		8.59E-01	6.96E-01		-6.61E-01	2.78E-01		-2.73E+00	1.12E-04	Down							
Unigene109324_All	910	0.00	0.00	0.00	0.60	0.26	0.15	4.83	0.37	0.31	null	null		null	null		-1.18E+00	2.25E-01		-2.02E+00	7.06E-02		-3.72E+00	6.99E-22	Down	-3.98E+00	7.42E-24	Down	Unigene109324_All//gi|288559104|sp|B9GXD6.1|U497E_POPTR//1.00E-55//RecName: Full=UPF0497 membrane protein 14	Unigene109324_All//5.00E-38//AT3G16300| integral membrane family protein	Unigene109324_All//0.00E+00//TC173483			GO:0016020	nr +
Unigene109326_All	1230	0.09	0.08	0.00	0.04	0.22	0.07	6.29	0.51	3.14	-4.65E-02	9.91E-01		-6.41E+00	3.98E-01		2.47E+00	2.23E-01		8.58E-01	7.97E-01		-3.63E+00	2.38E-37	Down	-1.00E+00	7.22E-08	Down	Unigene109326_All//gi|17979520|gb|AAL50095.1|//1.00E-125//At2g41800/T11A7.10 [Arabidopsis thaliana] >gi|20453311|gb|AAM19894.1| At2g41800/T11A7.10 [Arabidopsis thaliana]	Unigene109326_All//3.00E-132//AT2G41810| FUNCTIONS IN: molecular_function unknown; INVOLVED IN: biological_process unknown; LOCATED IN: endomembrane system; EXPRESSED IN: root; CONTAINS InterPro DOMAIN/s: Protein of unknown function DUF642 (InterPro:IPR006946), Galactose-binding like (InterPro:IPR008979); BEST Arabidopsis thaliana protein match is: unknown protein (TAIR:AT2G41800.1); Has 161 Blast hits to 157 proteins in 12 species: Archae - 0; Bacteria - 2; Metazoa - 0; Fungi - 0; Plants - 159; Viruses - 0; Other Eukaryotes - 0 (source: NCBI BLink).	Unigene109326_All//0.00E+00//gi|78342877|gb|DT563151.1|DT563151			GO:0005618	nr -
Unigene109328_All	264	0.00	0.00	0.00	0.00	0.00	0.00	1.92	0.00	0.00	null	null		null	null		null	null		null	null		-1.09E+01	8.90E-04	Down	-1.09E+01	5.56E-04	Down	Unigene109328_All//gi|227059902|gb|ACP18969.1|//2.00E-08//calcium binding protein [Euglena gracilis]	Unigene109328_All//2.00E-09//AT2G41100| TCH3 (TOUCH 3); calcium ion binding			ko04626|Plant-pathogen interaction//ko04070|Phosphatidylinositol signaling system	GO:0009612//GO:0009642//GO:0016020//GO:0046872	nr +
Unigene109329_All	403	0.00	0.00	0.00	0.00	0.00	0.00	2.31	0.31	0.00	null	null		null	null		null	null		null	null		-2.89E+00	3.28E-04	Down	-1.12E+01	5.48E-07	Down							
Unigene10933_All	469	0.56	0.97	1.25	1.59	1.25	2.79	3.34	3.39	13.85	8.02E-01	4.60E-01		1.16E+00	2.47E-01		-3.52E-01	6.51E-01		8.10E-01	1.91E-01		2.18E-02	9.98E-01		2.05E+00	9.12E-18	Up							
Unigene109336_All	800	0.00	0.00	0.00	0.00	0.00	0.00	6.24	0.37	0.10	null	null		null	null		null	null		null	null		-4.09E+00	1.46E-26	Down	-5.97E+00	2.00E-33	Down	Unigene109336_All//gi|297820480|ref|XP_002878123.1|//6.00E-27//OBP3-responsive gene 3 [Arabidopsis lyrata subsp. lyrata] >gi|297323961|gb|EFH54382.1| OBP3-responsive gene 3 [Arabidopsis lyrata subsp. lyrata]	Unigene109336_All//1.00E-22//AT3G56970| BHLH038; DNA binding / transcription factor		bHLH		GO:0005488//GO:0006950//GO:0010468	nr +
Unigene10934_All	1536	0.00	0.00	0.35	0.00	0.00	0.06	0.28	0.84	6.04	null	null		8.44E+00	4.05E-03		null	null		5.88E+00	4.81E-01		1.62E+00	3.75E-03		4.46E+00	1.58E-52	Up	Unigene10934_All//gi|124360549|gb|ABD33126.2|//2.00E-24//RNA-directed DNA polymerase (Reverse transcriptase) [Medicago truncatula]	Unigene10934_All//5.00E-05//AT4G29090| reverse transcriptase, putative / RNA-dependent DNA polymerase, putative				GO:0003676//GO:0016772//GO:0044238//GO:0044260	nr -
Unigene109348_All	335	0.00	0.00	0.00	0.00	0.00	0.00	1.51	0.00	0.36	null	null		null	null		null	null		null	null		-1.06E+01	8.90E-04	Down	-2.09E+00	3.65E-02		Unigene109348_All//gi|15778156|dbj|BAB68528.1|//2.00E-29//14-3-3 protein [Nicotiana tabacum]	Unigene109348_All//2.00E-29//AT1G22300| GRF10 (GENERAL REGULATORY FACTOR 10); ATP binding / protein binding / protein phosphorylated amino acid binding	Unigene109348_All//4.00E-10//DV849765				nr -
Unigene109354_All	311	0.00	0.00	0.00	0.00	0.00	0.00	1.90	0.00	0.13	null	null		null	null		null	null		null	null		-1.09E+01	2.40E-04	Down	-3.89E+00	1.10E-03		Unigene109354_All//gi|37625025|gb|AAQ96336.1|//3.00E-20//ribosomal protein L3B [Nicotiana tabacum]	Unigene109354_All//9.00E-18//AT1G43170| ARP1 (ARABIDOPSIS RIBOSOMAL PROTEIN 1); structural constituent of ribosome	Unigene109354_All//9.00E-11//TC149894		ko03010|Ribosome	GO:0005198//GO:0010467//GO:0030529	nr -
Unigene109357_All	737	0.00	0.00	0.00	0.34	0.28	0.00	3.27	0.74	0.16	null	null		null	null		-2.70E-01	8.23E-01		-8.40E+00	7.36E-02		-2.15E+00	2.78E-07	Down	-4.33E+00	4.31E-14	Down	Unigene109357_All//gi|297835706|ref|XP_002885735.1|//9.00E-57//mate efflux family protein [Arabidopsis lyrata subsp. lyrata] >gi|297331575|gb|EFH61994.1| mate efflux family protein [Arabidopsis lyrata subsp. lyrata]	Unigene109357_All//2.00E-51//AT2G04100| MATE efflux family protein	Unigene109357_All//3.00E-151//gi|5044469|gb|AI725617.1|AI725617			GO:0006810//GO:0022857//GO:0044464	nr +

Unigene109364_All	340	0.00	0.00	0.00	0.00	0.00	0.00	1.74	0.00	0.00	null	null		null	null		null	null		null	null		-1.08E+01	2.40E-04	Down	-1.08E+01	1.42E-04	Down	Unigene109364_All//sp|Q9ZZM3|NU5M_SALSA//7.00E-33//NADH-ubiquinone oxidoreductase chain 5 OS=Salmo salar GN=MT-ND5 PE=3 SV=1						swissprot +
Unigene109376_All	341	0.00	0.00	0.00	0.00	0.00	0.00	1.74	0.00	0.00	null	null		null	null		null	null		null	null		-1.08E+01	2.40E-04	Down	-1.08E+01	1.42E-04	Down							ESTscan -
Unigene109381_All	525	0.00	0.00	0.00	0.00	0.00	0.00	1.53	0.08	0.08	null	null		null	null		null	null		null	null		-4.26E+00	8.48E-05	Down	-4.33E+00	4.54E-05	Down	Unigene109381_All//gi|159464339|ref|XP_001690399.1|//3.00E-15//fibrocystin-L-like protein [Chlamydomonas reinhardtii] >gi|158279899|gb|EDP05658.1| fibrocystin-L-like protein [Chlamydomonas reinhardtii]						nr -
Unigene109383_All	486	0.00	0.00	0.00	0.00	0.00	0.00	2.44	0.60	0.33	null	null		null	null		null	null		null	null		-2.02E+00	1.06E-03		-2.89E+00	2.03E-05	Down	Unigene109383_All//gi|157060284|dbj|BAF79872.1|//3.00E-42//actin [Lobosphaeropsis lobophora]	Unigene109383_All//3.00E-34//AT3G18780| ACT2 (ACTIN 2); structural constituent of cytoskeleton	Unigene109383_All//1.00E-07//EX164549		ko04145|Phagosome	GO:0032559//GO:0043232	nr -
Unigene109385_All	767	0.00	0.00	0.00	0.00	0.00	0.00	2.37	0.16	0.36	null	null		null	null		null	null		null	null		-3.86E+00	1.31E-09	Down	-2.70E+00	1.86E-07	Down	Unigene109385_All//gi|84508536|ref|YP_448700.1|//2.00E-95//cytochrome c oxidase subunit 1 [Dictyota dichotoma] >gi|45925704|gb|AAS79085.1| cytochrome oxidase subunit I [Dictyota dichotoma]	Unigene109385_All//4.00E-77//ATMG01360| cytochrome c oxidase subunit 1	Unigene109385_All//2.00E-07//EX167412		ko00190|Oxidative phosphorylation	GO:0005506//GO:0015002//GO:0019866//GO:0031224//GO:0043231//GO:0045333//GO:0051234	nr -
Unigene109388_All	610	0.09	0.00	0.00	0.16	0.00	0.15	0.90	0.00	1.96	-6.42E+00	6.19E-01		-6.42E+00	6.32E-01		-7.35E+00	3.17E-01		-1.41E-01	9.53E-01		-9.82E+00	4.62E-04	Down	1.12E+00	3.88E-02		Unigene109388_All//gi|147771776|emb|CAN66777.1|//6.00E-15//hypothetical protein [Vitis vinifera]						nr -
Unigene10939_All	410	2.68	6.92	5.45	3.89	3.44	2.09	2.99	2.75	2.14	1.37E+00	4.19E-04	Up	1.03E+00	2.31E-02		-1.75E-01	7.68E-01		-8.94E-01	9.15E-02		-1.20E-01	8.28E-01		-4.84E-01	3.61E-01		Unigene10939_All//gi|224993580|gb|ACN76469.1|//1.00E-08//CBL-interacting protein kinase 12 [Populus euphratica]	Unigene10939_All//3.00E-07//AT1G01140| CIPK9 (CBL-INTERACTING PROTEIN KINASE 9); ATP binding / kinase/ protein kinase/ protein serine/threonine kinase	Unigene10939_All//8.00E-86//gi|78328960|gb|DT549234.1|DT549234				nr +
Unigene109390_All	354	0.00	0.00	0.00	0.00	0.00	0.00	2.75	0.00	0.34	null	null		null	null		null	null		null	null		-1.14E+01	5.84E-07	Down	-3.02E+00	9.61E-05	Down	Unigene109390_All//gi|188496902|dbj|BAG32350.1|//1.00E-07//glucan binding protein-2 [Actinophrys sol]						nr -
Unigene109396_All	960	1.42	1.32	0.33	6.69	3.95	3.20	0.53	0.52	0.79	-1.03E-01	8.47E-01		-2.09E+00	1.95E-03		-7.63E-01	4.08E-04		-1.07E+00	3.80E-06	Down	-1.67E-02	1.02E+00		5.77E-01	4.12E-01		Unigene109396_All//gi|22326632|ref|NP_196289.2|//6.00E-70//HAT14 (HOMEOBOX FROM ARABIDOPSIS THALIANA); DNA binding / transcription factor [Arabidopsis thaliana] >gi|90101577|sp|P46665.3|HAT14_ARATH RecName: Full=Homeobox-leucine zipper protein HAT14; AltName: Full=Homeodomain-leucine zipper protein HAT14; Short=HD-ZIP protein 14 >gi|110743069|dbj|BAE99427.1| homeobox protein [Arabidopsis thaliana] >gi|167730916|emb|CAD24012.2| homeodomain-leucine zipper protein HAT14 [Arabidopsis thaliana]	Unigene109396_All//9.00E-58//AT5G06710| HAT14 (HOMEOBOX FROM ARABIDOPSIS THALIANA); DNA binding / transcription factor	Unigene109396_All//0.00E+00//ES802659	HB		GO:0001071//GO:0003677//GO:0006351//GO:0010468//GO:0030528//GO:0043231	nr -
Unigene1094_All	1254	4.59	7.03	5.18	13.63	20.79	43.61	2.80	6.37	4.70	6.15E-01	1.53E-03		1.75E-01	5.05E-01		6.09E-01	7.02E-10		1.68E+00	5.55E-94	Up	1.19E+00	5.38E-10	Up	7.49E-01	4.13E-04		Unigene1094_All//gi|42571559|ref|NP_973870.1|//1.00E-117//pathogenesis-related thaumatin family protein [Arabidopsis thaliana] >gi|8778995|gb|AAF79910.1|AC022472_19 Contains similarity to SCUTL1 mRNA from Vitis vinifera gb|AF195653 and is a member of the thaumatin family PF|00314. EST gb|AI995819 comes from this gene [Arabidopsis thaliana]	Unigene1094_All//8.00E-118//AT1G20030| pathogenesis-related thaumatin family protein	Unigene1094_All//0.00E+00//TC132485				nr +
Unigene10940_All	282	0.00	0.00	0.00	0.00	0.00	0.00	4.50	5.34	0.71	null	null		null	null		null	null		null	null		2.46E-01	6.67E-01		-2.67E+00	2.31E-05	Down			Unigene10940_All//1.00E-12//TC155558				
Unigene109400_All	918	0.63	0.00	0.12	0.22	0.00	0.30	1.43	0.73	0.09	-9.29E+00	1.52E-03		-2.43E+00	4.40E-02		-7.76E+00	8.47E-02		4.43E-01	7.64E-01		-9.71E-01	6.64E-02		-4.04E+00	1.35E-07	Down	Unigene109400_All//gi|145332393|ref|NP_001078153.1|//2.00E-12//RTFL10 (ROTUNDIFOLIA LIKE 10) [Arabidopsis thaliana] >gi|42822071|tpg|DAA02290.1| TPA_exp: DVL19 [Arabidopsis thaliana]	Unigene109400_All//4.00E-13//AT3G14362| RTFL10 (ROTUNDIFOLIA LIKE 10)				GO:0048513	nr -
Unigene109402_All	566	0.00	0.00	0.00	0.00	0.00	0.00	1.42	0.52	0.00	null	null		null	null		null	null		null	null		-1.46E+00	4.65E-02		-1.05E+01	4.43E-06	Down	Unigene109402_All//gi|21362874|sp|Q9ATF5.1|RL18A_CASSA//3.00E-29//RecName: Full=60S ribosomal protein L18a >gi|13430184|gb|AAK25759.1|AF334839_1 ribosomal protein L18a [Castanea sativa]	Unigene109402_All//6.00E-29//AT3G14600| 60S ribosomal protein L18A (RPL18aC)			ko03010|Ribosome	GO:0009987//GO:0015934	nr +
Unigene109419_All	407	0.00	0.00	0.00	0.00	0.08	0.00	0.93	0.21	3.82	null	null		null	null		6.40E+00	8.37E-01		null	null		-2.19E+00	8.77E-02		2.03E+00	8.65E-05	Up							
Unigene109420_All	292	0.00	2.43	0.00	0.00	0.00	0.00	0.58	0.14	0.14	1.12E+01	3.33E-04	Up	null	null		null	null		null	null		-2.02E+00	3.68E-01		-2.09E+00	3.14E-01								
Unigene109421_All	619	0.51	0.49	0.95	0.48	0.17	0.00	1.57	0.20	0.45	-4.63E-02	9.85E-01		9.00E-01	3.77E-01		-1.53E+00	2.98E-01		-8.92E+00	4.01E-02		-2.96E+00	1.89E-04	Down	-1.80E+00	5.53E-03		Unigene109421_All//gi|15242739|ref|NP_195957.1|//5.00E-27//acylphosphatase family [Arabidopsis thaliana]	Unigene109421_All//6.00E-28//AT5G03370| acylphosphatase family	Unigene109421_All//7.00E-115//TC154406		ko00620|Pyruvate metabolism	GO:0016787//GO:0043231	nr -
Unigene109422_All	350	0.00	0.00	0.00	0.00	0.00	0.00	2.17	1.19	0.00	null	null		null	null		null	null		null	null		-8.65E-01	2.39E-01		-1.11E+01	8.89E-06	Down	Unigene109422_All//gi|37625523|gb|AAQ96375.1|//9.00E-29//60S ribosomal protein L13 [Solanum palustre]	Unigene109422_All//2.00E-23//AT3G49010| ATBBC1 (ARABIDOPSIS THALIANA BREAST BASIC CONSERVED 1); structural constituent of ribosome			ko03010|Ribosome	GO:0030529	nr -
Unigene109425_All	255	0.00	0.00	0.00	0.00	0.14	0.18	2.32	0.66	0.00	null	null		null	null		7.08E+00	8.37E-01		7.47E+00	7.02E-01		-1.82E+00	4.72E-02		-1.12E+01	1.42E-04	Down		Unigene109425_All//7.00E-05//AT3G13870| RHD3 (ROOT HAIR DEFECTIVE 3); GTP binding	Unigene109425_All//3.00E-22//DW478609				
Unigene109427_All	244	0.00	0.00	0.00	0.00	0.00	0.00	6.93	2.74	0.00	null	null		null	null		null	null		null	null		-1.34E+00	3.80E-03		-1.28E+01	1.70E-12	Down	Unigene109427_All//gi|302128114|dbj|BAJ13478.1|//5.00E-30//actin 1 [Guillardia theta]	Unigene109427_All//6.00E-30//AT5G09810| ACT7 (ACTIN 7); structural constituent of cytoskeleton	Unigene109427_All//9.00E-29//gi|31406833|gb|CD485868.1|CD485868		ko04145|Phagosome	GO:0000267//GO:0003015//GO:0005198//GO:0005884//GO:0006939//GO:0007015//GO:0008015//GO:0008092//GO:0012501//GO:0017111//GO:0030017//GO:0030239//GO:0032432//GO:0032559//GO:0048747//GO:0051707//GO:0060415	nr -
Unigene109428_All	258	0.00	0.00	0.00	0.00	0.00	0.00	5.08	0.97	0.00	null	null		null	null		null	null		null	null		-2.39E+00	9.40E-05	Down	-1.23E+01	9.85E-10	Down	Unigene109428_All//gi|76161004|gb|ABA40465.1|//2.00E-20//ribosomal protein S27-like protein [Solanum tuberosum]	Unigene109428_All//5.00E-21//AT3G61110| ARS27A (ARABIDOPSIS RIBOSOMAL PROTEIN S27); structural constituent of ribosome			ko03010|Ribosome	GO:0005198//GO:0010467//GO:0030529//GO:0043169	nr +
Unigene109430_All	984	0.00	0.00	0.00	0.30	0.00	0.00	2.41	0.34	6.64	null	null		null	null		-8.25E+00	2.02E-02		-8.25E+00	4.01E-02		-2.82E+00	1.60E-09	Down	1.46E+00	1.70E-11	Up	Unigene109430_All//gi|158934007|emb|CAJ40777.1|//4.00E-93//alpha galactosidase precursor [Coffea arabica]	Unigene109430_All//4.00E-95//AT2G42570| unknown protein	Unigene109430_All//5.00E-80//TC144190				nr +
Unigene109437_All	315	0.00	0.00	0.00	0.00	0.00	0.00	1.74	0.00	0.00	null	null		null	null		null	null		null	null		-1.08E+01	4.62E-04	Down	-1.08E+01	2.81E-04	Down	Unigene109437_All//gi|17221648|dbj|BAB78478.1|//9.00E-15//preproMP73 [Cucurbita maxima]				ko03040|Spliceosome		nr +
Unigene109438_All	477	0.00	0.21	0.00	0.31	0.00	0.38	3.55	0.35	0.17	7.73E+00	3.90E-01		null	null		-8.29E+00	1.66E-01		2.74E-01	9.05E-01		-3.34E+00	4.60E-08	Down	-4.41E+00	3.76E-10	Down			Unigene109438_All//4.00E-20//ES817207				
Unigene109439_All	988	0.00	0.00	0.00	0.00	0.00	0.00	2.10	2.83	0.00	null	null		null	null		null	null		null	null		4.35E-01	2.16E-01		-1.10E+01	2.86E-15	Down							
Unigene109441_All	415	0.13	0.37	0.51	0.48	0.17	0.76	76.41	86.21	256.31	1.54E+00	5.32E-01		2.03E+00	3.62E-01		-1.53E+00	4.16E-01		6.66E-01	6.48E-01		1.74E-01	4.20E-02		1.75E+00	1.84E-224	Up			Unigene109441_All//1.00E-26//gi|164320070|gb|EV494852.1|EV494852				
Unigene109445_All	528	0.00	0.00	0.00	0.00	0.00	0.00	2.08	0.32	0.23	null	null		null	null		null	null		null	null		-2.72E+00	1.31E-04	Down	-3.20E+00	1.63E-05	Down	Unigene109445_All//gi|162458045|ref|NP_001105804.1|//2.00E-25//protein disulfide isomerase [Zea mays] >gi|59861273|gb|AAX09966.1| protein disulfide isomerase [Zea mays]	Unigene109445_All//3.00E-24//AT1G04980| ATPDIL2-2 (PDI-LIKE 2-2); protein disulfide isomerase	Unigene109445_All//6.00E-07//TC132722		ko04141|Protein processing in endoplasmic reticulum	GO:0016020//GO:0033554//GO:0043231//GO:0044444	nr -
Unigene109449_All	442	0.00	0.11	0.00	0.00	0.31	0.20	1.82	0.09	0.72	6.84E+00	6.21E-01		null	null		8.28E+00	3.20E-01		7.68E+00	4.81E-01		-4.26E+00	8.48E-05	Down	-1.33E+00	5.40E-02								
Unigene109464_All	594	0.18	0.00	0.18	0.25	0.12	0.15	1.85	0.28	1.81	-7.46E+00	3.76E-01		2.52E-02	1.00E+00		-1.12E+00	5.85E-01		-7.26E-01	7.17E-01		-2.72E+00	1.31E-04	Down	-3.14E-02	9.69E-01								
Unigene109467_All	256	0.00	0.00	0.00	0.00	0.00	0.00	2.81	0.82	0.00	null	null		null	null		null	null		null	null		-1.78E+00	2.79E-02		-1.15E+01	1.78E-05	Down	Unigene109467_All//gi|254933819|gb|ACT87559.1|//4.00E-13//60S ribosomal protein L22 [Karlodinium veneficum]				ko03010|Ribosome		nr +
Unigene109472_All	261	0.00	0.00	0.00	0.00	0.00	0.00	2.75	0.64	0.00	null	null		null	null		null	null		null	null		-2.10E+00	1.25E-02		-1.14E+01	1.78E-05	Down	Unigene109472_All//gi|301111866|ref|XP_002905012.1|//4.00E-13//major vault protein [Phytophthora infestans T30-4] >gi|262095342|gb|EEY53394.1| major vault protein [Phytophthora infestans T30-4]						nr +
Unigene109488_All	474	0.00	0.00	0.00	0.00	0.00	0.00	3.75	1.94	0.00	null	null		null	null		null	null		null	null		-9.50E-01	3.09E-02		-1.19E+01	4.12E-13	Down	Unigene109488_All//gi|4204761|gb|AAD11482.1|//1.00E-24//peroxidase precursor [Glycine max]	Unigene109488_All//5.00E-22//AT1G05260| RCI3 (RARE COLD INDUCIBLE GENE 3); peroxidase	Unigene109488_All//3.00E-104//DN817536		ko00940|Phenylpropanoid biosynthesis//ko00360|Phenylalanine metabolism		nr -
Unigene10949_All	288	6.54	3.87	2.96	9.17	5.86	2.82	3.82	2.61	0.55	-7.57E-01	1.10E-01		-1.14E+00	2.33E-02		-6.46E-01	8.50E-02		-1.70E+00	3.01E-05	Down	-5.47E-01	3.72E-01		-2.79E+00	6.37E-05	Down			Unigene10949_All//3.00E-07//GE653421				
Unigene109491_All	264	0.00	0.00	0.00	0.00	0.00	0.00	3.36	1.58	0.15	null	null		null	null		null	null		null	null		-1.09E+00	1.07E-01		-4.48E+00	1.23E-05	Down			Unigene109491_All//2.00E-08//gi|11208618|gb|BF277548.1|BF277548				
Unigene109492_All	456	0.00	0.00	0.00	0.00	0.00	0.00	1.67	0.00	0.00	null	null		null	null		null	null		null	null		-1.07E+01	1.68E-05	Down	-1.07E+01	8.89E-06	Down							ESTscan -
Unigene109495_All	396	0.00	0.00	0.00	1.13	1.65	2.85	1.92	0.21	0.91	null	null		null	null		5.45E-01	5.86E-01		1.33E+00	4.77E-02		-3.19E+00	7.93E-04	Down	-1.09E+00	1.21E-01								
Unigene109497_All	263	0.00	0.00	0.00	0.00	0.00	0.00	1.93	0.16	0.00	null	null		null	null		null	null		null	null		-3.60E+00	5.78E-03		-1.09E+01	5.56E-04	Down	Unigene109497_All//gi|4139170|gb|AAD03711.1|//1.00E-31//elongation translation factor 1 alpha [Cyanophora paradoxa]	Unigene109497_All//5.00E-31//AT5G60390| elongation factor 1-alpha / EF-1-alpha	Unigene109497_All//6.00E-27//TC166357				nr +
Unigene10950_All	478	0.66	0.00	0.00	4.38	2.02	1.42	0.35	0.00	0.08	-9.36E+00	3.67E-02		-9.36E+00	4.93E-02		-1.12E+00	8.02E-03		-1.63E+00	4.42E-04	Down	-8.47E+00	1.35E-01		-2.09E+00	3.14E-01		Unigene10950_All//gi|42567435|ref|NP_195315.2|//2.00E-11//FD; DNA binding / protein binding / transcription activator/ transcription factor [Arabidopsis thaliana]						nr -
Unigene109510_All	271	0.00	0.00	0.00	0.00	0.00	0.00	2.50	0.00	0.00	null	null		null	null		null	null		null	null		-1.13E+01	6.34E-05	Down	-1.13E+01	3.57E-05	Down							
Unigene109511_All	260	0.00	0.00	0.00	0.00	0.00	0.00	2.28	0.64	0.00	null	null		null	null		null	null		null	null		-1.82E+00	4.72E-02		-1.12E+01	1.42E-04	Down							ESTscan -
Unigene109513_All	284	0.00	0.00	0.00	0.00	0.12	0.00	1.94	0.00	0.42	null	null		null	null		6.92E+00	8.37E-01		null	null		-1.09E+01	4.62E-04	Down	-2.20E+00	2.24E-02		Unigene109513_All//gi|224087080|ref|XP_002308057.1|//2.00E-08//predicted protein [Populus trichocarpa] >gi|222854033|gb|EEE91580.1| predicted protein [Populus trichocarpa]	Unigene109513_All//5.00E-06//AT5G35200| epsin N-terminal homology (ENTH) domain-containing protein	Unigene109513_All//3.00E-13//TC132781				nr -
Unigene109516_All	462	0.00	0.00	0.00	0.00	0.00	0.20	2.10	0.54	0.17	null	null		null	null		null	null		7.61E+00	4.81E-01		-1.96E+00	4.44E-03		-3.61E+00	2.14E-05	Down			Unigene109516_All//3.00E-67//TC167240				

Unigene109518_All	567	0.00	0.00	0.00	0.18	0.00	0.00	2.24	0.74	0.42	null	null		null	null		-7.46E+00	3.16E-01		-7.46E+00	4.05E-01		-1.60E+00	4.42E-03		-2.41E+00	7.19E-05	Down	Unigene109518_All//gi|297610489|ref|NP_001064615.2|//5.00E-37//Os10g0419100 [Oryza sativa Japonica Group] >gi|255679407|dbj|BAF26529.2| Os10g0419100 [Oryza sativa Japonica Group]	Unigene109518_All//3.00E-25//AT1G60680| aldo/keto reductase family protein			ko00053|Ascorbate and aldarate metabolism		nr -
Unigene109528_All	217	0.00	0.00	0.00	0.00	0.00	0.00	5.26	0.00	0.00	null	null		null	null		null	null		null	null		-1.24E+01	3.89E-08	Down	-1.24E+01	1.65E-08	Down							ESTscan -
Unigene10954_All	405	6.59	7.26	5.65	1.23	0.34	0.22	0.21	0.52	2.16	1.39E-01	6.97E-01		-2.21E-01	6.16E-01		-1.85E+00	8.44E-02		-2.46E+00	4.83E-02		1.31E+00	4.48E-01		3.37E+00	1.32E-04	Up			Unigene10954_All//4.00E-66//TC170579				
Unigene109544_All	251	0.00	0.00	0.00	0.00	0.00	0.00	2.36	0.67	0.00	null	null		null	null		null	null		null	null		-1.82E+00	4.72E-02		-1.12E+01	1.42E-04	Down	Unigene109544_All//sp|O70370|CATS_MOUSE//9.00E-09//Cathepsin S OS=Mus musculus GN=Ctss PE=2 SV=1						swissprot -
Unigene109546_All	502	0.00	0.00	0.00	0.00	0.00	0.00	2.53	0.83	0.00	null	null		null	null		null	null		null	null		-1.60E+00	4.42E-03		-1.13E+01	1.99E-09	Down	Unigene109546_All//gi|226490975|ref|NP_001147262.1|//1.00E-36//60S ribosomal protein L13a-2 [Zea mays] >gi|195609234|gb|ACG26447.1| 60S ribosomal protein L13a-2 [Zea mays]	Unigene109546_All//3.00E-17//AT3G07110| 60S ribosomal protein L13A (RPL13aA)			ko03010|Ribosome		nr -
Unigene109547_All	280	0.75	1.45	0.76	1.42	3.69	3.23	3.47	0.30	3.98	9.54E-01	4.14E-01		2.54E-02	1.00E+00		1.37E+00	5.14E-02		1.18E+00	1.29E-01		-3.54E+00	4.32E-05	Down	1.98E-01	7.17E-01		Unigene109547_All//gi|225454244|ref|XP_002274479.1|//5.00E-39//PREDICTED: similar to 2-oxo acid dehydrogenase, lipoyl-binding site [Vitis vinifera]	Unigene109547_All//3.00E-31//AT4G00450| CRP (CRYPTIC PRECOCIOUS)	Unigene109547_All//6.00E-135//TC136182				nr -
Unigene109548_All	227	0.00	0.00	0.00	0.00	0.00	0.00	5.40	1.84	0.18	null	null		null	null		null	null		null	null		-1.55E+00	6.55E-03		-4.94E+00	6.14E-08	Down	Unigene109548_All//gi|297792603|ref|XP_002864186.1|//5.00E-28//heat shock protein 81-1 [Arabidopsis lyrata subsp. lyrata] >gi|297310021|gb|EFH40445.1| heat shock protein 81-1 [Arabidopsis lyrata subsp. lyrata]	Unigene109548_All//2.00E-29//AT5G52640| ATHSP90.1 (HEAT SHOCK PROTEIN 90.1); ATP binding / unfolded protein binding			ko04626|Plant-pathogen interaction//ko04141|Protein processing in endoplasmic reticulum		nr -
Unigene109552_All	276	0.00	0.00	0.00	0.18	0.00	0.00	2.14	1.21	0.00	null	null		null	null		-7.50E+00	5.62E-01		-7.50E+00	6.44E-01		-8.24E-01	3.48E-01		-1.11E+01	1.42E-04	Down	Unigene109552_All//gi|297720735|ref|NP_001172729.1|//6.00E-22//Os01g0935200 [Oryza sativa Japonica Group] >gi|148921414|dbj|BAF64446.1| HAP3 subunit of HAP complex [Oryza sativa Japonica Group] >gi|255674039|dbj|BAH91459.1| Os01g0935200 [Oryza sativa Japonica Group]	Unigene109552_All//6.00E-22//AT2G38880| NF-YB1 (NUCLEAR FACTOR Y, SUBUNIT B1); transcription factor		CCAAT			nr +
Unigene109563_All	993	0.00	0.00	0.00	0.00	0.00	0.05	2.43	0.29	0.84	null	null		null	null		null	null		5.51E+00	7.02E-01		-3.04E+00	2.24E-10	Down	-1.53E+00	4.53E-05	Down	Unigene109563_All//gi|15236579|ref|NP_194919.1|//2.00E-45//transferase family protein [Arabidopsis thaliana] >gi|15450359|gb|AAK96473.1| AT4g31910/F11C18_110 [Arabidopsis thaliana] >gi|22655040|gb|AAM98111.1| At4g31910/F11C18_110 [Arabidopsis thaliana]	Unigene109563_All//8.00E-41//AT4G31910| transferase family protein			ko00940|Phenylpropanoid biosynthesis//ko00945|Stilbenoid, diarylheptanoid and gingerol biosynthesis//ko00941|Flavonoid biosynthesis		nr -
Unigene109567_All	517	0.00	0.00	0.00	0.00	0.00	0.00	4.91	1.37	0.00	null	null		null	null		null	null		null	null		-1.84E+00	2.33E-06	Down	-1.23E+01	1.14E-18	Down							
Unigene10957_All	301	0.00	3.87	0.00	0.99	1.49	0.60	0.00	0.00	0.00	1.19E+01	1.05E-06	Up	null	null		5.83E-01	6.26E-01		-7.26E-01	6.39E-01		null	null		null	null		Unigene10957_All//gi|224103919|ref|XP_002313244.1|//6.00E-27//ZIP transporter [Populus trichocarpa] >gi|222849652|gb|EEE87199.1| ZIP transporter [Populus trichocarpa]	Unigene10957_All//3.00E-22//AT2G30080| ZIP6; cation transmembrane transporter/ metal ion transmembrane transporter	Unigene10957_All//2.00E-122//CO088688			GO:0000041//GO:0008152//GO:0015082//GO:0016628//GO:0031224//GO:0046914//GO:0051707	nr -
Unigene109574_All	270	0.00	0.00	0.00	0.00	0.00	0.00	2.66	0.00	0.00	null	null		null	null		null	null		null	null		-1.14E+01	3.27E-05	Down	-1.14E+01	1.78E-05	Down							ESTscan -
Unigene109575_All	484	0.00	0.00	0.00	0.00	0.00	0.00	7.51	0.00	0.08	null	null		null	null		null	null		null	null		-1.29E+01	1.01E-25	Down	-6.51E+00	4.29E-25	Down	Unigene109575_All//gi|289586042|gb|ADD11814.1|//6.00E-27//hybrid proline-rich protein [Cajanus cajan]	Unigene109575_All//6.00E-17//AT4G12480| pEARLI 1; lipid binding	Unigene109575_All//2.00E-25//gi|72270150|gb|DT047511.1|DT047511				nr +
Unigene109580_All	617	0.17	0.00	0.00	0.16	0.00	0.00	1.71	0.34	0.32	-7.41E+00	3.76E-01		-7.41E+00	3.98E-01		-7.34E+00	3.16E-01		-7.34E+00	4.05E-01		-2.34E+00	6.78E-04	Down	-2.41E+00	3.46E-04	Down	Unigene109580_All//gi|87240699|gb|ABD32557.1|//7.00E-11//Tetratricopeptide-like helical [Medicago truncatula]	Unigene109580_All//1.00E-11//AT2G22070| pentatricopeptide (PPR) repeat-containing protein					nr +
Unigene109588_All	265	1.38	0.00	0.60	4.51	11.70	0.85	1.28	0.16	0.00	-1.04E+01	1.97E-02		-1.20E+00	3.96E-01		1.37E+00	9.45E-05	Up	-2.40E+00	7.07E-04	Down	-3.02E+00	5.31E-02		-1.03E+01	8.17E-03		Unigene109588_All//gi|255550325|ref|XP_002516213.1|//8.00E-06//conserved hypothetical protein [Ricinus communis] >gi|223544699|gb|EEF46215.1| conserved hypothetical protein [Ricinus communis]		Unigene109588_All//1.00E-61//gi|109867016|gb|DW495992.1|DW495992				nr +
Unigene10959_All	408	0.00	17.39	12.40	0.00	0.00	0.00	0.00	0.00	0.00	1.41E+01	1.53E-40	Up	1.36E+01	3.83E-28	Up	null	null		null	null		null	null		null	null								
Unigene109593_All	539	0.00	0.00	0.00	0.00	0.00	0.00	2.27	0.54	0.89	null	null		null	null		null	null		null	null		-2.07E+00	6.67E-04	Down	-1.36E+00	1.18E-02		Unigene109593_All//gi|5302802|emb|CAB46043.1|//3.00E-41//retrotransposon like protein [Arabidopsis thaliana] >gi|7268438|emb|CAB80958.1| retrotransposon like protein [Arabidopsis thaliana]	Unigene109593_All//2.00E-05//ATMG00710| hypothetical protein					nr -
Unigene109597_All	248	0.00	0.00	0.00	0.00	0.00	0.00	3.92	1.69	0.16	null	null		null	null		null	null		null	null		-1.22E+00	5.68E-02		-4.61E+00	3.32E-06	Down	Unigene109597_All//gi|224062999|ref|XP_002300962.1|//4.00E-28//1-aminocyclopropane-1-carboxylate [Populus trichocarpa] >gi|222842688|gb|EEE80235.1| 1-aminocyclopropane-1-carboxylate [Populus trichocarpa]	Unigene109597_All//1.00E-21//AT1G77330| 1-aminocyclopropane-1-carboxylate oxidase, putative / ACC oxidase, putative			ko00270|Cysteine and methionine metabolism		nr -
Unigene10960_All	310	0.68	3.11	4.81	0.48	0.22	0.00	0.00	0.00	0.00	2.20E+00	6.58E-03		2.83E+00	6.17E-05	Up	-1.12E+00	5.85E-01		-8.91E+00	2.34E-01		null	null		null	null								
Unigene109600_All	513	0.00	0.00	0.00	0.00	0.00	0.00	1.98	1.79	0.16	null	null		null	null		null	null		null	null		-1.42E-01	8.11E-01		-3.67E+00	1.15E-05	Down	Unigene109600_All//sp|Q869S5|DOKA_DICDI//4.00E-07//Hybrid signal transduction protein dokA OS=Dictyostelium discoideum GN=dokA PE=1 SV=1						swissprot +
Unigene109601_All	255	0.00	0.00	0.00	0.00	0.00	0.00	2.16	0.00	0.00	null	null		null	null		null	null		null	null		-1.11E+01	4.62E-04	Down	-1.11E+01	2.81E-04	Down							
Unigene109604_All	372	0.00	0.00	0.00	0.00	0.00	0.00	2.16	0.90	0.00	null	null		null	null		null	null		null	null		-1.26E+00	7.98E-02		-1.11E+01	4.43E-06	Down							ESTscan +
Unigene109605_All	629	0.00	0.00	0.00	0.00	0.00	0.00	1.81	0.00	0.00	null	null		null	null		null	null		null	null		-1.08E+01	3.89E-08	Down	-1.08E+01	1.65E-08	Down	Unigene109605_All//gi|226497590|ref|NP_001148801.1|//5.00E-43//60S ribosomal protein L7a [Zea mays] >gi|195622244|gb|ACG32952.1| 60S ribosomal protein L7a [Zea mays]	Unigene109605_All//7.00E-38//AT2G47610| 60S ribosomal protein L7A (RPL7aA)			ko03010|Ribosome		nr +
Unigene10962_All	297	0.00	0.00	0.18	1.01	0.93	0.30	3.70	8.02	24.82	null	null		7.49E+00	6.40E-01		-1.18E-01	9.01E-01		-1.73E+00	2.90E-01		1.12E+00	2.75E-03		2.75E+00	6.25E-28	Up			Unigene10962_All//4.00E-28//CO103704				
Unigene109621_All	448	0.00	0.00	0.00	0.44	0.23	0.20	2.27	0.56	0.00	null	null		null	null		-9.48E-01	5.88E-01		-1.14E+00	5.70E-01		-2.02E+00	2.83E-03		-1.11E+01	1.35E-07	Down	Unigene109621_All//gi|145340316|ref|NP_193430.3|//2.00E-39//ATP binding / kinase/ protein kinase/ protein serine/threonine kinase [Arabidopsis thaliana] >gi|110738029|dbj|BAF00949.1| kinase like protein [Arabidopsis thaliana]	Unigene109621_All//1.00E-40//AT4G16970| ATP binding / kinase/ protein kinase/ protein serine/threonine kinase	Unigene109621_All//7.00E-62//ES834303				nr -
Unigene109622_All	264	0.00	0.00	0.00	0.00	0.00	0.00	1.92	0.48	0.00	null	null		null	null		null	null		null	null		-2.02E+00	5.21E-02		-1.09E+01	5.56E-04	Down			Unigene109622_All//2.00E-36//TC169484				
Unigene109623_All	204	0.00	0.00	0.00	0.00	0.00	0.00	2.90	0.41	8.98	null	null		null	null		null	null		null	null		-2.82E+00	7.18E-03		1.63E+00	2.55E-04	Up							
Unigene109625_All	472	0.00	0.00	0.00	0.00	0.07	0.00	1.88	0.00	0.00	null	null		null	null		6.19E+00	8.37E-01		null	null		-1.09E+01	2.24E-06	Down	-1.09E+01	1.10E-06	Down							
Unigene109628_All	500	0.00	0.20	0.43	0.40	0.21	0.09	1.94	2.26	0.32	7.66E+00	3.90E-01		8.73E+00	1.42E-01		-9.48E-01	5.88E-01		-2.14E+00	3.48E-01		2.15E-01	7.15E-01		-2.61E+00	3.36E-04	Down							
Unigene109636_All	224	0.00	0.00	0.00	0.67	0.77	0.40	4.72	2.99	0.36	null	null		null	null		2.04E-01	9.50E-01		-7.27E-01	7.17E-01		-6.61E-01	2.78E-01		-3.73E+00	6.14E-06	Down							
Unigene10964_All	773	0.00	6.82	2.48	0.00	0.00	0.00	0.00	0.00	0.00	1.27E+01	4.74E-30	Up	1.13E+01	1.47E-10	Up	null	null		null	null		null	null		null	null		Unigene10964_All//gi|195636372|gb|ACG37654.1|//2.00E-24//eukaryotic initiation factor 5C CG2922-PF, isoform F [Zea mays]	Unigene10964_All//7.00E-20//AT5G36230| eIF4-gamma/eIF5/eIF2-epsilon domain-containing protein				GO:0044464	nr -
Unigene10965_All	448	0.47	1.13	3.21	0.56	0.46	0.00	0.00	0.00	0.00	1.28E+00	2.31E-01		2.78E+00	1.05E-04	Up	-2.70E-01	8.23E-01		-9.12E+00	7.36E-02		null	null		null	null								ESTscan -
Unigene109651_All	508	0.00	0.00	0.00	0.00	0.00	0.00	1.50	0.16	0.08	null	null		null	null		null	null		null	null		-3.19E+00	7.93E-04	Down	-4.26E+00	8.66E-05	Down	Unigene109651_All//gi|15231294|ref|NP_187969.1|//2.00E-21//calmodulin-binding family protein [Arabidopsis thaliana]	Unigene109651_All//7.00E-23//AT3G13600| calmodulin-binding family protein					nr +
Unigene109657_All	324	0.00	0.00	0.00	0.00	0.00	0.00	2.09	0.39	0.12	null	null		null	null		null	null		null	null		-2.43E+00	7.77E-03		-4.09E+00	3.10E-04	Down	Unigene109657_All//gi|159466892|ref|XP_001691632.1|//2.00E-37//beta subunit of mitochondrial ATP synthase [Chlamydomonas reinhardtii] >gi|584796|sp|P38482.1|ATPBM_CHLRE RecName: Full=ATP synthase subunit beta, mitochondrial; Flags: Precursor >gi|18123|emb|CAA43808.1| H(+)-transporting ATP synthase; beta subunit of mitochondrial ATP synthase [Chlamydomonas reinhardtii] >gi|158278978|gb|EDP04740.1| beta subunit of mitochondrial ATP synthase [Chlamydomonas reinhardtii]	Unigene109657_All//1.00E-36//AT5G08680| ATP synthase beta chain, mitochondrial, putative			ko00190|Oxidative phosphorylation		nr -
Unigene109658_All	480	0.00	0.00	0.00	0.00	0.00	0.00	2.47	1.31	0.17	null	null		null	null		null	null		null	null		-9.17E-01	1.03E-01		-3.89E+00	9.21E-07	Down							ESTscan +
Unigene109665_All	1174	0.13	0.35	0.36	0.51	0.21	0.15	5.08	3.06	1.59	1.37E+00	2.74E-01		1.44E+00	2.53E-01		-1.31E+00	1.56E-01		-1.73E+00	9.04E-02		-7.30E-01	7.15E-04		-1.67E+00	9.54E-13	Down			Unigene109665_All//1.00E-13//TC178515				
Unigene109666_All	278	0.00	0.00	0.00	0.00	0.00	0.00	2.43	1.50	0.00	null	null		null	null		null	null		null	null		-6.95E-01	3.94E-01		-1.12E+01	3.57E-05	Down	Unigene109666_All//gi|197312883|gb|ACH63222.1|//2.00E-10//actin depolymerizing factor [Rheum australe]	Unigene109666_All//3.00E-10//AT5G59890| ADF4 (ACTIN DEPOLYMERIZING FACTOR 4); actin binding					nr -
Unigene109667_All	246	0.00	0.00	0.00	0.00	0.00	0.00	2.23	0.68	0.00	null	null		null	null		null	null		null	null		-1.72E+00	7.23E-02		-1.11E+01	2.81E-04	Down	Unigene109667_All//gi|146216004|gb|ABQ10204.1|//3.00E-10//cysteine protease Cp6 [Actinidia deliciosa]	Unigene109667_All//3.00E-09//AT4G35350| XCP1 (XYLEM CYSTEINE PEPTIDASE 1); cysteine-type endopeptidase/ cysteine-type peptidase					nr -
Unigene109668_All	312	0.00	0.00	0.17	0.16	0.11	0.00	2.03	0.00	0.89	null	null		7.42E+00	6.40E-01		-5.33E-01	8.28E-01		-7.32E+00	6.44E-01		-1.10E+01	1.24E-04	Down	-1.19E+00	1.33E-01				Unigene109668_All//6.00E-09//FL577058				
Unigene109670_All	469	0.11	0.11	0.00	1.17	0.88	0.29	2.07	0.27	0.25	-4.73E-02	9.87E-01		-6.80E+00	6.31E-01		-4.07E-01	6.58E-01		-2.02E+00	7.06E-02		-2.95E+00	1.89E-04	Down	-3.02E+00	9.61E-05	Down	Unigene109670_All//gi|224126937|ref|XP_002319964.1|//1.00E-12//predicted protein [Populus trichocarpa] >gi|222858340|gb|EEE95887.1| predicted protein [Populus trichocarpa]						nr -
Unigene109672_All	394	0.00	0.00	0.00	0.00	0.00	0.00	1.29	1.91	0.00	null	null		null	null		null	null		null	null		5.68E-01	4.47E-01		-1.03E+01	5.56E-04	Down	Unigene109672_All//gi|14423905|sp|Q9XGL4.1|RL31_CYAPA//1.00E-24//RecName: Full=60S ribosomal protein L31 >gi|5123727|emb|CAB45375.1| ribosomal protein L31 [Cyanophora paradoxa]	Unigene109672_All//2.00E-18//AT5G56710| 60S ribosomal protein L31 (RPL31C)			ko03010|Ribosome		nr +
Unigene109673_All	272	0.00	0.00	0.00	0.00	0.00	0.00	1.87	0.00	0.29	null	null		null	null		null	null		null	null		-1.09E+01	8.90E-04	Down	-2.67E+00	1.37E-02								ESTscan -

Unigene109676_All	407	0.00	0.00	0.00	0.00	0.00	0.00	1.45	0.62	0.00	null	null		null	null		null	null		null	null		-1.24E+00	1.54E-01		-1.05E+01	1.42E-04	Down	Unigene109676_All//gi|226493187|ref|NP_001148445.1|//6.00E-19//actin-depolymerizing factor [Zea mays] >gi|195619314|gb|ACG31487.1| actin-depolymerizing factor [Zea mays]	Unigene109676_All//7.00E-18//AT4G25590| ADF7 (actin depolymerizing factor 7); actin binding					nr -
Unigene109677_All	519	0.00	0.00	0.00	0.00	0.00	0.00	0.98	1.45	0.00	null	null		null	null		null	null		null	null		5.68E-01	4.47E-01		-9.93E+00	5.55E-04	Down	Unigene109677_All//gi|116056034|emb|CAL58567.1|//2.00E-15//Nucleolar GTPase/ATPase p130 (ISS) [Ostreococcus tauri]						nr +
Unigene109678_All	442	0.00	0.00	0.00	0.00	0.00	0.00	5.74	0.47	0.72	null	null		null	null		null	null		null	null		-3.60E+00	1.51E-12	Down	-2.99E+00	3.88E-11	Down	Unigene109678_All//gi|301105188|ref|XP_002901678.1|//1.00E-31//60S ribosomal protein L32-1 [Phytophthora infestans T30-4] >gi|262100682|gb|EEY58734.1| 60S ribosomal protein L32-1 [Phytophthora infestans T30-4]	Unigene109678_All//5.00E-26//AT4G18100| 60S ribosomal protein L32 (RPL32A)			ko03010|Ribosome		nr +
Unigene10968_All	357	1.61	3.12	7.31	0.56	1.16	1.14	0.24	0.00	0.00	9.54E-01	1.40E-01		2.18E+00	2.86E-06	Up	1.05E+00	4.26E-01		1.03E+00	4.29E-01		-7.89E+00	4.06E-01		-7.89E+00	3.60E-01				Unigene10968_All//0.00E+00//BQ403264				
Unigene109680_All	426	0.00	0.00	0.00	0.00	0.00	0.00	2.98	0.10	0.00	null	null		null	null		null	null		null	null		-4.92E+00	7.77E-08	Down	-1.15E+01	1.99E-09	Down	Unigene109680_All//gi|298713887|emb|CBJ33763.1|//3.00E-09//conserved unknown protein [Ectocarpus siliculosus]		Unigene109680_All//4.00E-20//TC146753				nr +
Unigene109682_All	424	0.00	0.00	0.00	0.00	0.00	0.00	1.79	0.10	0.00	null	null		null	null		null	null		null	null		-4.19E+00	1.58E-04	Down	-1.08E+01	8.89E-06	Down	Unigene109682_All//gi|110006646|gb|ABG37650.2|//3.00E-16//CCHC-type integrase [Populus trichocarpa]						nr -
Unigene109684_All	308	0.00	0.00	0.17	0.00	0.00	0.00	3.16	0.81	0.65	null	null		7.43E+00	6.40E-01		null	null		null	null		-1.96E+00	4.44E-03		-2.29E+00	9.84E-04	Down	Unigene109684_All//gi|149392539|gb|ABR26072.1|//1.00E-14//imp biosynthesis protein [Oryza sativa Indica Group]		Unigene109684_All//4.00E-50//gi|78354813|gb|DT575087.1|DT575087				nr +
Unigene109686_All	415	0.00	0.00	0.00	0.00	0.00	0.00	5.91	0.40	0.00	null	null		null	null		null	null		null	null		-3.87E+00	8.08E-13	Down	-1.25E+01	4.73E-18	Down	Unigene109686_All//gi|116057495|emb|CAL51922.1|//1.00E-43//Putative 40S ribosomal protein S17 (ISS) [Ostreococcus tauri]	Unigene109686_All//4.00E-39//AT5G04800| 40S ribosomal protein S17 (RPS17D)			ko03010|Ribosome		nr +
Unigene10969_All	473	0.00	7.28	2.59	0.00	0.00	0.00	0.00	0.00	0.00	1.28E+01	1.31E-19	Up	1.13E+01	9.13E-07	Up	null	null		null	null		null	null		null	null		Unigene10969_All//gi|56236114|gb|AAV84513.1|//3.00E-09//At5g67490 [Arabidopsis thaliana] >gi|56381905|gb|AAV85671.1| At5g67490 [Arabidopsis thaliana] >gi|114050697|gb|ABI49498.1| At5g67490 [Arabidopsis thaliana]	Unigene10969_All//2.00E-10//AT5G67490| unknown protein					nr -
Unigene109693_All	318	0.16	0.00	0.00	0.63	0.87	0.14	3.06	0.26	0.25	-7.36E+00	6.19E-01		-7.36E+00	6.31E-01		4.67E-01	8.17E-01		-2.14E+00	3.48E-01		-3.54E+00	4.32E-05	Down	-3.61E+00	2.14E-05	Down			Unigene109693_All//4.00E-07//TC138616				
Unigene109698_All	590	0.00	0.00	0.00	0.00	0.00	0.00	1.58	0.00	0.47	null	null		null	null		null	null		null	null		-1.06E+01	1.15E-06	Down	-1.74E+00	8.54E-03		Unigene109698_All//gi|119714|sp|P13983.1|EXTN_TOBAC//2.00E-09//RecName: Full=Extensin; AltName: Full=Cell wall hydroxyproline-rich glycoprotein; Flags: Precursor				ko03040|Spliceosome		nr +
Unigene109699_All	270	0.00	0.00	0.00	0.00	0.00	0.00	1.88	0.31	0.00	null	null		null	null		null	null		null	null		-2.60E+00	2.05E-02		-1.09E+01	5.55E-04	Down							
Unigene10970_All	334	0.00	19.27	9.73	0.00	0.00	0.00	0.00	0.00	0.00	1.42E+01	9.43E-37	Up	1.32E+01	5.81E-18	Up	null	null		null	null		null	null		null	null								ESTscan +
Unigene109703_All	405	0.39	0.38	0.13	0.74	0.77	0.22	1.98	0.72	0.20	-4.65E-02	9.90E-01		-1.56E+00	5.34E-01		5.22E-02	9.99E-01		-1.73E+00	2.89E-01		-1.46E+00	4.65E-02		-3.33E+00	2.47E-04	Down			Unigene109703_All//2.00E-169//TC179467				
Unigene109708_All	238	0.00	0.00	0.00	0.21	0.00	0.19	1.60	0.35	19.25	null	null		null	null		-7.71E+00	5.62E-01		-1.41E-01	9.56E-01		-2.19E+00	8.76E-02		3.59E+00	1.66E-22	Up	Unigene109708_All//gi|115472809|ref|NP_001060003.1|//4.00E-20//Os07g0564500 [Oryza sativa Japonica Group] >gi|113611539|dbj|BAF21917.1| Os07g0564500 [Oryza sativa Japonica Group]	Unigene109708_All//2.00E-20//AT2G29990| NDA2 (ALTERNATIVE NAD(P)H DEHYDROGENASE 2); FAD binding / NADH dehydrogenase/ oxidoreductase	Unigene109708_All//4.00E-80//gi|164352536|gb|EX166155.1|EX166155		ko00190|Oxidative phosphorylation		nr +
Unigene10971_All	399	0.13	0.38	0.00	2.50	0.95	0.45	2.54	0.94	0.50	1.54E+00	5.32E-01		-7.03E+00	6.31E-01		-1.40E+00	3.79E-02		-2.46E+00	2.07E-03		-1.43E+00	2.36E-02		-2.35E+00	5.84E-04	Down			Unigene10971_All//3.00E-156//AW187287				
Unigene109712_All	301	0.00	0.34	0.00	0.00	0.00	0.00	7.16	2.36	0.40	8.40E+00	3.90E-01		null	null		null	null		null	null		-1.60E+00	1.06E-04	Down	-4.17E+00	2.24E-12	Down							
Unigene109719_All	243	0.00	0.00	0.00	0.00	0.00	0.00	4.87	2.75	0.66	null	null		null	null		null	null		null	null		-8.24E-01	1.42E-01		-2.89E+00	2.03E-05	Down	Unigene109719_All//gi|20378861|gb|AAM21011.1|AF469073_1//1.00E-23//NBS resistance protein-like protein [Gossypium hirsutum]	Unigene109719_All//1.00E-12//AT4G11170| disease resistance protein (TIR-NBS-LRR class), putative	Unigene109719_All//8.00E-88//TC177530				nr +
Unigene109728_All	447	0.23	1.02	0.00	0.45	0.00	0.51	2.93	0.84	0.80	2.12E+00	9.60E-02		-7.87E+00	3.98E-01		-8.80E+00	8.47E-02		1.81E-01	9.35E-01		-1.80E+00	1.43E-03		-1.87E+00	6.93E-04	Down	Unigene109728_All//gi|224144987|ref|XP_002325485.1|//6.00E-59//cytochrome P450 [Populus trichocarpa] >gi|222862360|gb|EEE99866.1| cytochrome P450 [Populus trichocarpa]	Unigene109728_All//4.00E-36//AT5G36110| CYP716A1; electron carrier/ heme binding / iron ion binding / monooxygenase/ oxygen binding			ko00906|Carotenoid biosynthesis		nr -
Unigene10973_All	511	0.00	4.96	1.67	0.00	0.00	0.00	0.00	0.00	0.00	1.23E+01	2.05E-14	Up	1.07E+01	9.03E-05	Up	null	null		null	null		null	null		null	null		Unigene10973_All//sp|Q8VEL2|MTMRE_MOUSE//3.00E-26//Myotubularin-related protein 14 OS=Mus musculus GN=Mtmr14 PE=2 SV=2						swissprot +
Unigene109733_All	234	0.00	0.00	0.00	0.00	0.00	0.00	6.14	3.04	0.34	null	null		null	null		null	null		null	null		-1.02E+00	4.18E-02		-4.17E+00	1.93E-08	Down	Unigene109733_All//gi|66270081|gb|AAY43370.1|//7.00E-20//cathepsin-like cysteine protease [Phytophthora infestans]	Unigene109733_All//2.00E-18//AT5G60360| AALP (Arabidopsis aleurain-like protease); cysteine-type peptidase					nr -
Unigene109739_All	263	0.00	0.00	0.00	0.00	0.00	0.00	2.25	2.07	0.00	null	null		null	null		null	null		null	null		-1.24E-01	8.84E-01		-1.11E+01	1.41E-04	Down	Unigene109739_All//gi|257219572|gb|ACV50439.1|//5.00E-29//ribosomal protein L15 [Jatropha curcas]	Unigene109739_All//2.00E-19//AT4G16720| 60S ribosomal protein L15 (RPL15A)			ko03010|Ribosome		nr +
Unigene10974_All	353	0.00	0.00	0.00	0.00	0.00	0.77	0.96	2.84	4.06	null	null		null	null		null	null		9.58E+00	7.04E-02		1.57E+00	1.54E-02		2.08E+00	1.33E-04	Up							
Unigene109742_All	477	0.00	0.00	0.00	0.00	0.00	0.00	2.57	0.00	0.17	null	null		null	null		null	null		null	null		-1.13E+01	9.98E-09	Down	-3.94E+00	4.86E-07	Down	Unigene109742_All//gi|15229631|ref|NP_190560.1|//5.00E-34//60S ribosomal protein L26 (RPL26A) [Arabidopsis thaliana] >gi|297816242|ref|XP_002876004.1| 60S ribosomal protein L26 [Arabidopsis lyrata subsp. lyrata] >gi|27735242|sp|P51414.2|RL261_ARATH RecName: Full=60S ribosomal protein L26-1 >gi|6723436|emb|CAB66929.1| 60S RIBOSOMAL PROTEIN-like [Arabidopsis thaliana] >gi|297321842|gb|EFH52263.1| 60S ribosomal protein L26 [Arabidopsis lyrata subsp. lyrata]	Unigene109742_All//2.00E-35//AT3G49910| 60S ribosomal protein L26 (RPL26A)			ko03010|Ribosome		nr -
Unigene10975_All	200	1.31	0.00	0.00	0.75	0.00	1.58	0.85	5.22	0.20	-1.04E+01	6.72E-02		-1.04E+01	8.59E-02		-9.55E+00	1.66E-01		1.08E+00	4.76E-01		2.63E+00	2.86E-04	Up	-2.09E+00	3.14E-01				Unigene10975_All//3.00E-62//TC178153				
Unigene109753_All	387	0.14	0.13	0.00	0.77	0.00	0.00	2.73	1.40	0.62	-4.66E-02	9.87E-01		-7.08E+00	6.31E-01		-9.59E+00	2.02E-02		-9.59E+00	4.01E-02		-9.60E-01	1.12E-01		-2.14E+00	9.36E-04	Down	Unigene109753_All//gi|223452335|gb|ACM89495.1|//7.00E-15//protein kinase family protein [Glycine max]	Unigene109753_All//5.00E-11//AT2G18890| protein kinase family protein	Unigene109753_All//1.00E-75//TC160556		ko04626|Plant-pathogen interaction		nr -
Unigene109755_All	406	0.00	0.00	0.00	0.00	0.00	0.00	4.89	0.93	0.79	null	null		null	null		null	null		null	null		-2.40E+00	6.76E-07	Down	-2.64E+00	6.80E-08	Down							
Unigene109758_All	250	0.00	0.00	0.00	1.39	0.55	0.90	3.89	1.00	0.00	null	null		null	null		-1.34E+00	3.08E-01		-6.27E-01	6.21E-01		-1.96E+00	4.44E-03		-1.19E+01	2.72E-07	Down							
Unigene10976_All	384	0.00	0.00	0.00	0.26	1.61	1.65	21.47	9.80	1.97	null	null		null	null		2.64E+00	1.99E-02		2.67E+00	1.83E-02		-1.13E+00	1.22E-09	Down	-3.45E+00	2.06E-39	Down			Unigene10976_All//2.00E-178//DW510669				
Unigene109768_All	307	0.00	0.00	0.00	0.00	0.00	0.00	3.31	0.41	0.26	null	null		null	null		null	null		null	null		-3.02E+00	1.08E-04	Down	-3.67E+00	1.15E-05	Down	Unigene109768_All//gi|15231294|ref|NP_187969.1|//2.00E-46//calmodulin-binding family protein [Arabidopsis thaliana]	Unigene109768_All//5.00E-45//AT3G13600| calmodulin-binding family protein	Unigene109768_All//4.00E-47//gi|73861130|gb|DT463869.1|DT463869				nr +
Unigene10977_All	336	5.29	2.41	0.00	0.59	1.84	0.27	1.26	0.87	0.83	-1.13E+00	2.21E-02		-1.24E+01	1.03E-09	Down	1.64E+00	1.06E-01		-1.14E+00	5.70E-01		-5.31E-01	6.34E-01		-6.00E-01	5.53E-01		Unigene10977_All//gi|32171918|sp|Q9LXB4.1|Y5620_ARATH//3.00E-17//RecName: Full=Putative RNA methyltransferase At5g10620	Unigene10977_All//1.00E-18//AT5G10620| FUNCTIONS IN: molecular_function unknown; INVOLVED IN: biological_process unknown; LOCATED IN: chloroplast; EXPRESSED IN: 19 plant structures; EXPRESSED DURING: 11 growth stages; CONTAINS InterPro DOMAIN/s: SPOUT methyltransferase, predicted (InterPro:IPR003742); Has 3142 Blast hits to 3142 proteins in 1101 species: Archae - 27; Bacteria - 2338; Metazoa - 0; Fungi - 0; Plants - 22; Viruses - 0; Other Eukaryotes - 755 (source: NCBI BLink).	Unigene10977_All//6.00E-120//gi|164321360|gb|ES846517.1|ES846517			GO:0006364//GO:0008168//GO:0009536	nr +
Unigene109773_All	325	0.00	0.00	0.00	0.00	0.00	0.00	2.99	0.13	0.00	null	null		null	null		null	null		null	null		-4.54E+00	6.92E-06	Down	-1.15E+01	2.72E-07	Down	Unigene109773_All//gi|301120388|ref|XP_002907921.1|//1.00E-11//polyadenylate-binding protein 1-B [Phytophthora infestans T30-4] >gi|262102952|gb|EEY61004.1| polyadenylate-binding protein 1-B [Phytophthora infestans T30-4]	Unigene109773_All//1.00E-11//AT1G71770| PAB5 (POLY(A)-BINDING PROTEIN 5); RNA binding / poly(A) binding / translation initiation factor					nr -
Unigene109776_All	425	0.00	0.00	0.00	0.00	0.00	0.00	3.68	0.89	1.31	null	null		null	null		null	null		null	null		-2.06E+00	9.44E-05	Down	-1.49E+00	1.74E-03								
Unigene10978_All	345	5.16	4.70	0.62	2.60	5.29	4.84	1.23	0.00	0.00	-1.34E-01	7.71E-01		-3.06E+00	3.82E-06	Down	1.03E+00	3.41E-02		8.98E-01	8.51E-02		-1.03E+01	3.26E-03		-1.03E+01	2.15E-03		Unigene10978_All//gi|30684447|ref|NP_193591.2|//2.00E-15//proline-rich family protein [Arabidopsis thaliana] >gi|22135814|gb|AAM91093.1| AT4g18560/F28J12_220 [Arabidopsis thaliana] >gi|28416477|gb|AAO42769.1| At4g18560/F28J12_220 [Arabidopsis thaliana]		Unigene10978_All//1.00E-90//gi|78331756|gb|DT552030.1|DT552030			GO:0043231	
Unigene109780_All	257	0.41	1.18	0.00	0.00	0.13	0.00	3.62	0.00	2.33	1.54E+00	3.24E-01		-8.67E+00	3.98E-01		7.07E+00	8.37E-01		null	null		-1.18E+01	1.15E-06	Down	-6.38E-01	3.08E-01								
Unigene109781_All	279	2.81	0.00	3.05	3.21	0.74	1.30	2.27	0.60	0.00	-1.15E+01	1.09E-04	Down	1.19E-01	8.69E-01		-2.12E+00	5.22E-03		-1.31E+00	8.52E-02		-1.92E+00	3.09E-02		-1.12E+01	7.12E-05	Down	Unigene109781_All//gi|224092091|ref|XP_002309468.1|//3.00E-26//cytokinin oxidase [Populus trichocarpa] >gi|222855444|gb|EEE92991.1| cytokinin oxidase [Populus trichocarpa]	Unigene109781_All//2.00E-24//AT5G21482| CKX7 (CYTOKININ OXIDASE 7); cytokinin dehydrogenase/ oxidoreductase	Unigene109781_All//3.00E-121//ES790738		ko00908|Zeatin biosynthesis		nr +
Unigene109786_All	405	0.26	0.00	0.00	0.00	0.00	0.00	3.03	0.62	0.49	-8.01E+00	3.76E-01		-8.01E+00	3.98E-01		null	null		null	null		-2.29E+00	2.57E-04	Down	-2.62E+00	4.02E-05	Down	Unigene109786_All//gi|291197511|emb|CAZ68124.1|//3.00E-29//ATP binding / protein kinase/ protein tyrosine kinase [Arabidopsis halleri subsp. halleri]	Unigene109786_All//3.00E-24//AT2G46850| ATP binding / protein kinase/ protein tyrosine kinase					nr -
Unigene109799_All	317	0.00	0.00	0.00	0.00	0.00	0.00	1.87	1.19	0.00	null	null		null	null		null	null		null	null		-6.54E-01	4.45E-01		-1.09E+01	1.41E-04	Down	Unigene109799_All//gi|1015937|emb|CAA62943.1|//2.00E-10//extensin class 1 protein [Vigna unguiculata]				ko03040|Spliceosome		nr -
Unigene109801_All	430	0.00	0.00	0.00	0.00	0.00	0.00	2.85	0.49	0.56	null	null		null	null		null	null		null	null		-2.55E+00	8.71E-05	Down	-2.36E+00	1.22E-04	Down	Unigene109801_All//gi|297829824|ref|XP_002882794.1|//2.00E-27//oxidoreductase [Arabidopsis lyrata subsp. lyrata] >gi|297328634|gb|EFH59053.1| oxidoreductase [Arabidopsis lyrata subsp. lyrata]	Unigene109801_All//1.00E-28//AT3G12900| oxidoreductase, 2OG-Fe(II) oxygenase family protein					nr +

Unigene109811_All	606	0.00	0.00	0.00	0.00	0.00	0.00	5.51	0.14	0.07	null	null		null	null		null	null		null	null		-5.32E+00	9.93E-21	Down	-6.39E+00	5.88E-23	Down	Unigene109811_All//gi|15824574|gb|AAL09401.1|AF307336_1//2.00E-41//ribosomal protein [Petunia x hybrida]	Unigene109811_All//3.00E-42//AT1G27400| 60S ribosomal protein L17 (RPL17A)			ko03010|Ribosome		nr -
Unigene109813_All	353	0.00	0.00	0.00	0.00	0.00	0.00	1.92	0.00	0.34	null	null		null	null		null	null		null	null		-1.09E+01	6.34E-05	Down	-2.50E+00	4.79E-03		Unigene109813_All//sp|Q4JQH8|NU4M_TETNG//1.00E-26//NADH-ubiquinone oxidoreductase chain 4 OS=Tetraodon nigroviridis GN=MT-ND4 PE=3 SV=1						swissprot -
Unigene109815_All	570	0.00	0.00	0.00	0.00	0.00	0.00	3.19	1.17	0.00	null	null		null	null		null	null		null	null		-1.44E+00	1.28E-03		-1.16E+01	2.03E-13	Down							
Unigene109817_All	396	0.00	0.00	0.00	0.00	0.00	0.00	1.71	0.63	0.00	null	null		null	null		null	null		null	null		-1.43E+00	7.84E-02		-1.07E+01	3.57E-05	Down							
Unigene109820_All	492	0.00	0.00	0.00	0.00	0.00	0.00	2.23	1.61	0.00	null	null		null	null		null	null		null	null		-4.69E-01	4.31E-01		-1.11E+01	3.33E-08	Down	Unigene109820_All//gi|160330969|ref|XP_001712192.1|//2.00E-67//ef2 [Hemiselmis andersenii] >gi|159765639|gb|ABW97867.1| ef2 [Hemiselmis andersenii]	Unigene109820_All//3.00E-65//AT1G56070| LOS1; copper ion binding / translation elongation factor/ translation factor, nucleic acid binding	Unigene109820_All//1.00E-07//ES850809				nr -
Unigene109821_All	279	0.00	0.18	0.00	0.00	0.00	0.49	1.97	0.00	0.43	7.50E+00	6.21E-01		null	null		null	null		8.92E+00	3.12E-01		-1.09E+01	4.62E-04	Down	-2.20E+00	2.24E-02		Unigene109821_All//gi|30684540|ref|NP_193617.2|//8.00E-38//ATP binding / DNA binding / DNA-directed DNA polymerase/ nucleoside-triphosphatase/ nucleotide binding [Arabidopsis thaliana]	Unigene109821_All//3.00E-39//AT4G18820| ATP binding / DNA binding / DNA-directed DNA polymerase/ nucleoside-triphosphatase/ nucleotide binding					nr +
Unigene109822_All	265	0.00	0.76	0.00	0.00	0.26	0.00	3.35	0.79	0.45	9.58E+00	1.37E-01		null	null		8.02E+00	6.15E-01		null	null		-2.09E+00	4.71E-03		-2.89E+00	3.05E-04	Down	Unigene109822_All//gi|256016311|gb|ACU56981.1|//1.00E-28//importin alpha [Citrus sinensis]	Unigene109822_All//5.00E-26//AT1G09270| IMPA-4 (IMPORTIN ALPHA ISOFORM 4); binding / protein transporter	Unigene109822_All//3.00E-07//TC168025				nr +
Unigene109830_All	370	0.00	0.00	0.00	0.27	0.09	0.00	3.54	0.34	0.54	null	null		null	null		-1.53E+00	5.88E-01		-8.07E+00	4.05E-01		-3.39E+00	1.90E-06	Down	-2.72E+00	1.32E-05	Down	Unigene109830_All//gi|224126161|ref|XP_002319770.1|//1.00E-27//cytochrome P450 [Populus trichocarpa] >gi|222858146|gb|EEE95693.1| cytochrome P450 [Populus trichocarpa]	Unigene109830_All//5.00E-16//AT2G27690| CYP94C1; fatty acid (omega-1)-hydroxylase/ oxygen binding	Unigene109830_All//0.00E+00//gi|84167351|gb|DR455999.1|DR455999		ko00945|Stilbenoid, diarylheptanoid and gingerol biosynthesis//ko00903|Limonene and pinene degradation		nr -
Unigene109831_All	335	0.00	1.21	0.00	1.04	0.82	0.67	2.52	0.25	0.48	1.02E+01	1.36E-02		null	null		-3.40E-01	7.86E-01		-6.27E-01	6.21E-01		-3.34E+00	2.51E-04	Down	-2.41E+00	1.68E-03		Unigene109831_All//gi|148524775|dbj|BAF63395.1|//5.00E-22//kinetochore protein [Olimarabidopsis pumila]	Unigene109831_All//5.00E-23//AT3G54630| FUNCTIONS IN: molecular_function unknown; INVOLVED IN: biological_process unknown; LOCATED IN: cellular_component unknown; EXPRESSED IN: 11 plant structures; EXPRESSED DURING: 6 growth stages; CONTAINS InterPro DOMAIN/s: Kinetochore protein Ndc80 (InterPro:IPR005550); Has 19800 Blast hits to 12108 proteins in 845 species: Archae - 345; Bacteria - 1709; Metazoa - 11031; Fungi - 1499; Plants - 797; Viruses - 39; Other Eukaryotes - 4380 (source: NCBI BLink).	Unigene109831_All//6.00E-43//CO109061				nr +
Unigene109832_All	396	0.00	0.00	0.00	0.00	0.00	0.00	2.67	0.00	0.00	null	null		null	null		null	null		null	null		-1.14E+01	1.51E-07	Down	-1.14E+01	6.72E-08	Down	Unigene109832_All//gi|66270183|gb|AAY43421.1|//5.00E-27//ribosomal protein L34 [Phytophthora infestans]	Unigene109832_All//7.00E-19//AT1G26880| 60S ribosomal protein L34 (RPL34A)			ko03010|Ribosome		nr +
Unigene109843_All	469	0.89	0.11	0.00	0.74	0.29	0.48	2.79	0.62	0.25	-3.05E+00	4.65E-02		-9.80E+00	1.56E-02		-1.34E+00	3.08E-01		-6.27E-01	6.21E-01		-2.16E+00	2.57E-04	Down	-3.46E+00	7.84E-07	Down	Unigene109843_All//gi|224144987|ref|XP_002325485.1|//5.00E-26//cytochrome P450 [Populus trichocarpa] >gi|222862360|gb|EEE99866.1| cytochrome P450 [Populus trichocarpa]	Unigene109843_All//2.00E-07//AT2G42850| CYP718; electron carrier/ heme binding / iron ion binding / monooxygenase/ oxygen binding					nr +
Unigene109846_All	664	1.26	0.15	0.00	1.95	1.82	1.09	2.42	1.07	0.90	-3.05E+00	2.11E-03		-1.03E+01	1.19E-04	Down	-1.04E-01	8.43E-01		-8.42E-01	1.64E-01		-1.18E+00	1.24E-02		-1.43E+00	2.13E-03		Unigene109846_All//gi|30685150|ref|NP_188529.2|//5.00E-21//VRN1 (REDUCED VERNALIZATION RESPONSE 1); transcription repressor [Arabidopsis thaliana] >gi|75153628|sp|Q8L3W1.1|VRN1_ARATH RecName: Full=B3 domain-containing transcription factor VRN1; AltName: Full=Protein VERNALIZATION 1 >gi|21734794|gb|AAM76972.1|AF289051_1 reduced vernalization response 1 [Arabidopsis thaliana] >gi|21734796|gb|AAM76973.1|AF289052_1 reduced vernalization response 1 [Arabidopsis thaliana] >gi|89000959|gb|ABD59069.1| At3g18990 [Arabidopsis thaliana]	Unigene109846_All//3.00E-22//AT3G18990| VRN1 (REDUCED VERNALIZATION RESPONSE 1); transcription repressor	Unigene109846_All//0.00E+00//ES832924	ABI3VP1			nr +
Unigene109848_All	359	0.00	0.00	0.00	0.00	0.00	0.00	1.77	0.58	0.00	null	null		null	null		null	null		null	null		-1.60E+00	6.27E-02		-1.08E+01	7.12E-05	Down	Unigene109848_All//gi|226495717|ref|NP_001152730.1|//5.00E-19//60S ribosomal protein L13-2 [Zea mays] >gi|195659423|gb|ACG49179.1| 60S ribosomal protein L13-2 [Zea mays]	Unigene109848_All//2.00E-19//AT5G23900| 60S ribosomal protein L13 (RPL13D)			ko03010|Ribosome		nr +
Unigene109849_All	241	0.00	0.00	0.00	0.00	0.00	0.00	2.81	0.35	0.17	null	null		null	null		null	null		null	null		-3.02E+00	2.44E-03		-4.09E+00	3.10E-04	Down							
Unigene109851_All	337	0.00	0.00	0.00	0.00	0.00	0.00	2.76	0.74	0.35	null	null		null	null		null	null		null	null		-1.89E+00	6.92E-03		-2.96E+00	1.72E-04	Down	Unigene109851_All//gi|283795057|ref|YP_003359473.1|//2.00E-17//cytochrome c oxidase subunit I [Synedra acus] >gi|261279713|gb|ACX62021.1| cytochrome c oxidase subunit I [Synedra acus]	Unigene109851_All//3.00E-13//ATMG01360| cytochrome c oxidase subunit 1			ko00190|Oxidative phosphorylation		nr +
Unigene109852_All	358	0.00	0.00	0.00	0.00	0.00	0.00	2.13	0.35	0.00	null	null		null	null		null	null		null	null		-2.60E+00	2.81E-03		-1.11E+01	8.89E-06	Down			Unigene109852_All//2.00E-12//gi|109859885|gb|DW488864.1|DW488864				
Unigene109853_All	866	0.06	0.00	0.00	0.12	0.04	0.21	4.78	0.92	0.05	-5.92E+00	6.19E-01		-5.92E+00	6.31E-01		-1.53E+00	5.88E-01		8.59E-01	6.96E-01		-2.38E+00	1.60E-13	Down	-6.70E+00	9.12E-29	Down	Unigene109853_All//gi|257219564|gb|ACV50435.1|//2.00E-82//Al-induced protein [Jatropha curcas]	Unigene109853_All//3.00E-78//AT5G19140| AILP1	Unigene109853_All//0.00E+00//DT047684				nr +
Unigene109855_All	1188	0.00	0.00	0.00	0.00	0.00	0.00	3.24	0.00	0.00	null	null		null	null		null	null		null	null		-1.17E+01	3.21E-27	Down	-1.17E+01	2.75E-28	Down	Unigene109855_All//gi|4139170|gb|AAD03711.1|//1.00E-173//elongation translation factor 1 alpha [Cyanophora paradoxa]	Unigene109855_All//1.00E-165//AT5G60390| elongation factor 1-alpha / EF-1-alpha	Unigene109855_All//2.00E-12//TC166357				nr -
Unigene109856_All	289	0.00	0.00	0.00	0.00	0.00	0.00	2.63	0.00	0.14	null	null		null	null		null	null		null	null		-1.14E+01	1.68E-05	Down	-4.26E+00	8.66E-05	Down							
Unigene109861_All	256	0.00	0.00	0.00	0.00	0.00	0.00	2.31	0.16	0.00	null	null		null	null		null	null		null	null		-3.82E+00	1.79E-03		-1.12E+01	1.41E-04	Down							ESTscan -
Unigene109864_All	654	0.00	0.00	0.00	0.00	0.00	0.00	4.53	0.70	0.43	null	null		null	null		null	null		null	null		-2.69E+00	3.55E-11	Down	-3.41E+00	1.95E-14	Down	Unigene109864_All//gi|14029020|gb|AAK52561.1|AC079853_14//8.00E-20//Putative retroelement pol polyprotein [Oryza sativa Japonica Group]						nr +
Unigene109865_All	328	0.00	0.00	0.00	0.00	0.00	0.00	1.93	1.15	0.00	null	null		null	null		null	null		null	null		-7.54E-01	3.73E-01		-1.09E+01	7.12E-05	Down	Unigene109865_All//gi|300267215|gb|EFJ51399.1|//2.00E-17//component of cytosolic 80S ribosome and 60S large subunit [Volvox carteri f. nagariensis]	Unigene109865_All//5.00E-18//AT1G07070| 60S ribosomal protein L35a (RPL35aA)			ko03010|Ribosome		nr -
Unigene10987_All	760	5.44	1.60	0.70	0.59	1.04	0.95	0.33	0.17	0.31	-1.77E+00	8.51E-08	Down	-2.96E+00	3.32E-13	Down	8.21E-01	3.28E-01		6.89E-01	4.48E-01		-1.02E+00	4.95E-01		-8.59E-02	9.57E-01		Unigene10987_All//gi|157863714|gb|ABV90878.1|//3.00E-74//inositol-1,4,5-triphosphate-5-phosphatase [Solanum lycopersicum]	Unigene10987_All//3.00E-45//AT2G32010| endonuclease/exonuclease/phosphatase family protein	Unigene10987_All//7.00E-171//TC169894			GO:0046030	nr +
Unigene109871_All	323	0.00	0.00	0.00	0.00	0.00	0.00	2.23	2.85	0.25	null	null		null	null		null	null		null	null		3.55E-01	6.21E-01		-3.17E+00	8.09E-04	Down			Unigene109871_All//6.00E-12//TC174779				
Unigene109874_All	976	0.00	0.00	0.00	0.00	0.00	0.00	3.99	0.09	0.04	null	null		null	null		null	null		null	null		-5.54E+00	1.70E-24	Down	-6.61E+00	6.27E-27	Down	Unigene109874_All//gi|226505998|ref|NP_001146929.1|//2.00E-59//60S ribosomal protein L7a [Zea mays] >gi|195605318|gb|ACG24489.1| 60S ribosomal protein L7a [Zea mays]	Unigene109874_All//2.00E-55//AT2G47610| 60S ribosomal protein L7A (RPL7aA)			ko03010|Ribosome		nr -
Unigene109878_All	477	0.33	0.85	0.67	0.52	0.94	0.00	3.72	1.14	3.67	1.37E+00	2.74E-01		1.03E+00	4.71E-01		8.46E-01	5.14E-01		-9.03E+00	7.35E-02		-1.71E+00	2.61E-04	Down	-1.87E-02	9.70E-01				Unigene109878_All//6.00E-07//TC168693				
Unigene109897_All	339	0.00	0.00	0.00	0.00	0.00	0.00	3.37	0.99	0.00	null	null		null	null		null	null		null	null		-1.77E+00	3.71E-03		-1.17E+01	1.65E-08	Down			Unigene109897_All//4.00E-07//CO126363				
Unigene1099_All	3340	11.23	40.07	16.25	9.02	11.77	40.88	10.75	16.36	22.03	1.83E+00	1.43E-241	Up	5.33E-01	3.98E-13		3.84E-01	1.28E-06		2.18E+00	0.00E+00	Up	6.06E-01	6.51E-21		1.04E+00	1.83E-70	Up	Unigene1099_All//gi|225449372|ref|XP_002282355.1|//0.00E+00//PREDICTED: pyrroline-5-carboxylate synthetase [Vitis vinifera]	Unigene1099_All//0.00E+00//AT2G39800| P5CS1 (DELTA1-PYRROLINE-5-CARBOXYLATE SYNTHASE 1); delta1-pyrroline-5-carboxylate synthetase	Unigene1099_All//0.00E+00//TC155874		ko00330|Arginine and proline metabolism	GO:0006560//GO:0016620//GO:0016774//GO:0032559//GO:0044424	nr -
Unigene109902_All	537	0.00	0.00	0.00	0.00	0.00	0.00	5.12	0.31	0.00	null	null		null	null		null	null		null	null		-4.04E+00	1.02E-14	Down	-1.23E+01	3.21E-20	Down	Unigene109902_All//gi|247421835|gb|ACS96445.1|//3.00E-48//S18.A ribosomal protein [Jatropha curcas]	Unigene109902_All//7.00E-49//AT4G09800| RPS18C (S18 RIBOSOMAL PROTEIN); RNA binding / nucleic acid binding / structural constituent of ribosome	Unigene109902_All//4.00E-11//BM360106		ko03010|Ribosome		nr -
Unigene109904_All	388	0.00	0.00	0.00	0.00	0.00	0.00	2.94	3.45	0.00	null	null		null	null		null	null		null	null		2.29E-01	6.60E-01		-1.15E+01	1.65E-08	Down	Unigene109904_All//gi|195626686|gb|ACG35173.1|//5.00E-37//60S ribosomal protein L12 [Zea mays]	Unigene109904_All//3.00E-36//AT2G37190| 60S ribosomal protein L12 (RPL12A)			ko03010|Ribosome		nr -
Unigene109906_All	517	0.00	0.00	0.00	0.00	0.00	0.00	2.04	1.54	0.31	null	null		null	null		null	null		null	null		-4.13E-01	5.15E-01		-2.73E+00	1.12E-04	Down							
Unigene109909_All	491	0.00	0.00	0.22	1.01	0.14	0.00	1.89	0.00	0.00	null	null		7.76E+00	4.02E-01		-2.85E+00	1.78E-02		-9.99E+00	2.98E-03		-1.09E+01	1.15E-06	Down	-1.09E+01	5.48E-07	Down	Unigene109909_All//gi|297819644|ref|XP_002877705.1|//1.00E-56//U-box domain-containing protein [Arabidopsis lyrata subsp. lyrata] >gi|297323543|gb|EFH53964.1| U-box domain-containing protein [Arabidopsis lyrata subsp. lyrata]	Unigene109909_All//3.00E-53//AT3G49810| U-box domain-containing protein	Unigene109909_All//0.00E+00//gi|3325662|gb|AI054548.1|AI054548				nr +
Unigene109914_All	256	0.20	0.40	0.42	0.00	0.54	1.06	2.64	2.45	0.16	9.54E-01	7.27E-01		1.03E+00	7.04E-01		9.07E+00	3.20E-01		1.00E+01	7.04E-02		-1.10E-01	8.90E-01		-4.09E+00	3.10E-04	Down							
Unigene10992_All	431	0.61	0.00	0.00	0.46	1.36	0.73	2.55	0.78	0.46	-9.25E+00	6.72E-02		-9.25E+00	8.59E-02		1.55E+00	1.41E-01		6.66E-01	6.48E-01		-1.72E+00	5.68E-03		-2.46E+00	2.04E-04	Down			Unigene10992_All//1.00E-90//ES817369				ESTscan -
Unigene109921_All	527	0.00	0.00	0.00	0.00	0.00	0.00	1.93	1.11	0.00	null	null		null	null		null	null		null	null		-7.94E-01	2.03E-01		-1.09E+01	1.35E-07	Down	Unigene109921_All//gi|81074776|gb|ABB55374.1|//1.00E-62//ribosomal protein L2-like [Solanum tuberosum]	Unigene109921_All//7.00E-57//AT2G18020| EMB2296 (embryo defective 2296); structural constituent of ribosome			ko03010|Ribosome		nr +
Unigene109928_All	368	0.00	0.00	0.00	0.00	0.00	0.00	2.30	1.25	0.00	null	null		null	null		null	null		null	null		-8.79E-01	2.07E-01		-1.12E+01	2.21E-06	Down	Unigene109928_All//gi|42565379|gb|AAS20966.1|//1.00E-26//60s acidic ribosomal protein [Hyacinthus orientalis]	Unigene109928_All//3.00E-15//AT3G44590| 60S acidic ribosomal protein P2 (RPP2D)	Unigene109928_All//7.00E-09//CD486354		ko03010|Ribosome		nr -
Unigene10993_All	474	0.00	4.38	2.13	0.00	0.00	0.00	0.00	0.00	0.00	1.21E+01	7.95E-12	Up	1.11E+01	1.27E-05	Up	null	null		null	null		null	null		null	null		Unigene10993_All//gi|224000485|ref|XP_002289915.1|//1.00E-21//ran binding protein 7 [Thalassiosira pseudonana CCMP1335] >gi|220975123|gb|EED93452.1| ran binding protein 7 [Thalassiosira pseudonana CCMP1335]	Unigene10993_All//2.00E-21//AT2G31660| SAD2 (SUPER SENSITIVE TO ABA AND DROUGHT2); binding / protein transporter					nr +
Unigene109935_All	395	0.00	0.00	0.00	0.00	0.00	0.00	1.28	0.32	0.00	null	null		null	null		null	null		null	null		-2.02E+00	5.21E-02		-1.03E+01	5.55E-04	Down							
Unigene109937_All	900	0.12	0.11	0.24	0.11	0.00	0.00	3.81	1.49	0.80	-4.66E-02	9.91E-01		1.03E+00	5.92E-01		-6.79E+00	3.16E-01		-6.79E+00	4.05E-01		-1.36E+00	1.16E-05	Down	-2.26E+00	5.16E-11	Down	Unigene109937_All//gi|164564743|dbj|BAF98224.1|//2.00E-63//CM0216.420.nc [Lotus japonicus]	Unigene109937_All//3.00E-56//AT2G21540| SFH3 (SEC14-LIKE 3); phosphatidylinositol transporter	Unigene109937_All//0.00E+00//TC131079				nr -

Unigene109939_All	430	0.49	0.00	0.37	1.16	0.48	0.21	2.46	0.58	0.28	-8.93E+00	1.23E-01		-3.90E-01	8.08E-01		-1.27E+00	2.22E-01		-2.46E+00	4.83E-02		-2.08E+00	1.78E-03		-3.15E+00	2.97E-05	Down	Unigene109939_All//gi|124359162|gb|ABN05685.1|//4.00E-09//RNA-directed DNA polymerase (Reverse transcriptase) [Medicago truncatula]		Unigene109939_All//2.00E-77//TC176279				nr +
Unigene109940_All	291	0.00	0.00	0.55	0.17	0.00	0.31	1.74	0.00	0.27	null	null		9.10E+00	2.40E-01		-7.42E+00	5.62E-01		8.58E-01	7.96E-01		-1.08E+01	8.90E-04	Down	-2.67E+00	1.37E-02		Unigene109940_All//gi|224144987|ref|XP_002325485.1|//8.00E-11//cytochrome P450 [Populus trichocarpa] >gi|222862360|gb|EEE99866.1| cytochrome P450 [Populus trichocarpa]	Unigene109940_All//2.00E-07//AT1G12740| CYP87A2; electron carrier/ heme binding / iron ion binding / monooxygenase/ oxygen binding	Unigene109940_All//8.00E-08//gi|78336333|gb|DT556607.1|DT556607				nr -
Unigene109943_All	263	0.00	0.00	0.00	0.00	0.00	0.00	2.09	1.27	0.00	null	null		null	null		null	null		null	null		-7.17E-01	4.22E-01		-1.10E+01	2.81E-04	Down	Unigene109943_All//gi|192910912|gb|ACF06564.1|//7.00E-20//ribosomal protein L15 [Elaeis guineensis]	Unigene109943_All//8.00E-16//AT4G16720| 60S ribosomal protein L15 (RPL15A)			ko03010|Ribosome		nr +
Unigene109944_All	321	0.00	0.00	0.00	0.00	0.00	0.00	5.80	0.26	0.50	null	null		null	null		null	null		null	null		-4.48E+00	9.04E-11	Down	-3.55E+00	1.31E-09	Down	Unigene109944_All//gi|161015596|gb|ABX55852.1|//2.00E-42//actin [Spumella-like flagellate JBC/S24]	Unigene109944_All//1.00E-40//AT3G12110| ACT11 (actin-11); structural constituent of cytoskeleton	Unigene109944_All//2.00E-46//TC153744		ko04145|Phagosome		nr +
Unigene109946_All	391	0.00	0.13	0.00	0.00	0.00	0.00	3.46	1.60	0.31	7.02E+00	6.21E-01		null	null		null	null		null	null		-1.11E+00	3.31E-02		-3.50E+00	4.24E-07	Down	Unigene109946_All//gi|224096760|ref|XP_002334672.1|//7.00E-13//cytochrome P450 [Populus trichocarpa] >gi|222874188|gb|EEF11319.1| cytochrome P450 [Populus trichocarpa]	Unigene109946_All//2.00E-10//AT4G36220| FAH1 (FERULIC ACID 5-HYDROXYLASE 1); ferulate 5-hydroxylase/ monooxygenase			ko00940|Phenylpropanoid biosynthesis		nr +
Unigene109949_All	354	0.15	0.00	0.15	0.14	0.00	0.00	2.15	0.00	0.68	-7.21E+00	6.19E-01		2.52E-02	9.97E-01		-7.14E+00	5.62E-01		-7.14E+00	6.43E-01		-1.11E+01	1.68E-05	Down	-1.67E+00	2.39E-02		Unigene109949_All//gi|186513260|ref|NP_194210.2|//3.00E-17//ATP binding / DNA binding / DNA-directed DNA polymerase [Arabidopsis thaliana] >gi|186513264|ref|NP_001119046.1| ATP binding / DNA binding / DNA-directed DNA polymerase [Arabidopsis thaliana]	Unigene109949_All//1.00E-18//AT4G24790| ATP binding / DNA binding / DNA-directed DNA polymerase					nr +
Unigene109958_All	384	0.00	0.00	0.00	0.00	0.00	0.00	1.98	0.87	0.00	null	null		null	null		null	null		null	null		-1.19E+00	1.12E-01		-1.10E+01	8.88E-06	Down	Unigene109958_All//gi|195618210|gb|ACG30935.1|//4.00E-20//60S ribosomal protein L21 [Zea mays]	Unigene109958_All//4.00E-20//AT1G57860| 60S ribosomal protein L21	Unigene109958_All//4.00E-07//CD485725		ko03010|Ribosome		nr +
Unigene109972_All	587	0.00	0.00	0.00	0.00	0.00	0.00	6.27	1.57	0.07	null	null		null	null		null	null		null	null		-2.00E+00	7.24E-10	Down	-6.53E+00	2.12E-25	Down	Unigene109972_All//gi|208964722|gb|ACI31551.1|//5.00E-76//heat shock protein 90-2 [Glycine max]	Unigene109972_All//5.00E-66//AT5G52640| ATHSP90.1 (HEAT SHOCK PROTEIN 90.1); ATP binding / unfolded protein binding	Unigene109972_All//3.00E-12//TC173881		ko04626|Plant-pathogen interaction//ko04141|Protein processing in endoplasmic reticulum		nr +
Unigene109975_All	355	0.00	0.00	0.00	0.00	0.00	0.00	4.17	0.47	0.22	null	null		null	null		null	null		null	null		-3.15E+00	8.54E-07	Down	-4.22E+00	1.00E-08	Down	Unigene109975_All//gi|15230008|ref|NP_187207.1|//2.00E-23//60S ribosomal protein L22-2 (RPL22B) [Arabidopsis thaliana] >gi|42572267|ref|NP_974229.1| 60S ribosomal protein L22-2 (RPL22B) [Arabidopsis thaliana] >gi|145331980|ref|NP_001078112.1| 60S ribosomal protein L22-2 (RPL22B) [Arabidopsis thaliana] >gi|17865568|sp|Q9M9W1.1|RL222_ARATH RecName: Full=60S ribosomal protein L22-2 >gi|21595684|gb|AAM66123.1| 60S ribosomal protein L22-2 [Arabidopsis thaliana] >gi|30102826|gb|AAP21331.1| At3g05560 [Arabidopsis thaliana]	Unigene109975_All//2.00E-14//AT3G05560| 60S ribosomal protein L22-2 (RPL22B)			ko03010|Ribosome		nr +
Unigene109978_All	696	0.00	0.00	0.00	0.00	0.00	0.00	3.58	1.14	0.74	null	null		null	null		null	null		null	null		-1.65E+00	1.62E-05	Down	-2.27E+00	2.76E-08	Down							
Unigene109982_All	221	0.00	0.00	0.00	0.00	0.00	0.00	2.87	6.62	0.18	null	null		null	null		null	null		null	null		1.21E+00	1.59E-02		-3.99E+00	5.82E-04	Down							
Unigene109983_All	428	0.00	0.00	0.00	0.00	0.00	0.00	10.97	1.66	2.14	null	null		null	null		null	null		null	null		-2.72E+00	1.39E-17	Down	-2.36E+00	1.47E-15	Down							
Unigene109985_All	342	0.92	1.19	0.00	0.44	0.30	0.79	3.71	1.83	0.35	3.69E-01	7.24E-01		-9.84E+00	4.93E-02		-5.33E-01	7.86E-01		8.58E-01	6.31E-01		-1.02E+00	5.93E-02		-3.41E+00	1.45E-06	Down	Unigene109985_All//gi|42568453|ref|NP_199910.2|//7.00E-15//WD-40 repeat family protein [Arabidopsis thaliana]	Unigene109985_All//4.00E-12//AT5G50970| WD-40 repeat family protein	Unigene109985_All//2.00E-157//TC151162				nr +
Unigene109986_All	382	1.10	0.27	0.14	0.65	0.27	0.47	3.21	2.19	0.52	-2.05E+00	1.21E-01		-2.97E+00	6.46E-02		-1.27E+00	4.24E-01		-4.63E-01	7.45E-01		-5.53E-01	3.30E-01		-2.62E+00	4.02E-05	Down	Unigene109986_All//gi|297809813|ref|XP_002872790.1|//2.00E-21//S-locus lectin protein kinase family protein [Arabidopsis lyrata subsp. lyrata] >gi|297318627|gb|EFH49049.1| S-locus lectin protein kinase family protein [Arabidopsis lyrata subsp. lyrata]	Unigene109986_All//1.00E-21//AT4G03230| ATP binding / kinase/ protein kinase/ protein serine/threonine kinase/ protein tyrosine kinase/ sugar binding			ko04626|Plant-pathogen interaction		nr -
Unigene109987_All	682	0.00	0.00	0.00	0.00	0.00	0.00	2.67	0.61	0.00	null	null		null	null		null	null		null	null		-2.12E+00	1.37E-05	Down	-1.14E+01	2.03E-13	Down							ESTscan -
Unigene109988_All	582	0.00	0.00	0.00	0.00	0.00	0.00	2.83	0.14	0.41	null	null		null	null		null	null		null	null		-4.30E+00	2.19E-09	Down	-2.79E+00	5.06E-07	Down	Unigene109988_All//gi|157313320|gb|ABV32553.1|//6.00E-65//endopolygalacturonase [Prunus persica]	Unigene109988_All//1.00E-53//AT3G59850| polygalacturonase, putative / pectinase, putative	Unigene109988_All//0.00E+00//CO111186		ko00500|Starch and sucrose metabolism//ko00040|Pentose and glucuronate interconversions		nr -
Unigene109991_All	276	0.00	0.00	0.00	0.00	0.00	0.00	3.06	0.91	0.00	null	null		null	null		null	null		null	null		-1.75E+00	1.64E-02		-1.16E+01	2.21E-06	Down	Unigene109991_All//gi|192913040|gb|ACF06628.1|//2.00E-22//structural constituent of ribosome [Elaeis guineensis]	Unigene109991_All//7.00E-22//AT3G04920| 40S ribosomal protein S24 (RPS24A)			ko03010|Ribosome		nr +
Unigene109992_All	265	0.00	0.00	0.00	0.00	0.00	0.00	0.64	4.57	0.00	null	null		null	null		null	null		null	null		2.84E+00	3.39E-05	Up	-9.32E+00	1.09E-01		Unigene109992_All//gi|3426064|emb|CAA07575.1|//2.00E-10//monooxygenase [Arabidopsis thaliana] >gi|4467141|emb|CAB37510.1| monooxygenase 2 (MO2) [Arabidopsis thaliana] >gi|7270837|emb|CAB80518.1| monooxygenase 2 (MO2) [Arabidopsis thaliana]	Unigene109992_All//7.00E-12//AT4G38540| monooxygenase, putative (MO2)	Unigene109992_All//1.00E-15//DW488507				nr +
Unigene109993_All	1307	0.00	0.00	0.00	0.00	0.00	0.00	2.94	4.35	0.09	null	null		null	null		null	null		null	null		5.63E-01	1.17E-02		-5.01E+00	4.76E-24	Down	Unigene109993_All//gi|297721715|ref|NP_001173220.1|//4.00E-12//Os02g0832150 [Oryza sativa Japonica Group] >gi|255671379|dbj|BAH91949.1| Os02g0832150 [Oryza sativa Japonica Group]	Unigene109993_All//3.00E-12//AT4G32600| zinc finger (C3HC4-type RING finger) family protein					nr +
Unigene109997_All	264	0.00	0.00	0.00	0.00	0.00	0.00	1.92	0.32	0.00	null	null		null	null		null	null		null	null		-2.60E+00	2.05E-02		-1.09E+01	5.55E-04	Down							
Unigene109998_All	361	0.00	0.00	0.00	0.00	0.00	0.00	2.58	0.58	0.11	null	null		null	null		null	null		null	null		-2.15E+00	2.95E-03		-4.54E+00	6.41E-06	Down			Unigene109998_All//3.00E-82//TC142701				
Unigene11_All	3832	4.74	1.81	1.64	22.52	19.26	9.57	10.03	2.33	4.00	-1.39E+00	1.43E-22	Down	-1.53E+00	5.28E-25	Down	-2.26E-01	1.72E-05		-1.23E+00	6.11E-95	Down	-2.10E+00	1.19E-102	Down	-1.33E+00	3.72E-55	Down	Unigene11_All//gi|15231259|ref|NP_190171.1|//0.00E+00//kinesin motor protein-related [Arabidopsis thaliana] >gi|7339486|emb|CAB82809.1| kinesin-related protein-like [Arabidopsis thaliana]	Unigene11_All//0.00E+00//AT3G45850 | kinesin motor protein-related	Unigene11_All//0.00E+00//TC155062				nr +
Unigene1100_All	462	0.00	0.33	0.00	1.51	0.75	2.05	7.05	10.95	27.68	8.36E+00	2.36E-01		null	null		-1.02E+00	2.25E-01		4.44E-01	5.74E-01		6.35E-01	7.52E-03		1.97E+00	1.54E-32	Up	Unigene1100_All//gi|15237207|ref|NP_197091.1|//1.00E-49//glycosyl hydrolase family 81 protein [Arabidopsis thaliana]	Unigene1100_All//7.00E-51//AT5G15870| glycosyl hydrolase family 81 protein	Unigene1100_All//2.00E-148//gi|84168686|gb|DR457334.1|DR457334			GO:0008152//GO:0016798	nr -
Unigene110000_All	334	0.00	0.91	0.96	3.73	1.24	0.68	3.16	2.50	0.00	9.83E+00	4.45E-02		9.90E+00	4.54E-02		-1.59E+00	7.06E-03		-2.46E+00	4.15E-04	Down	-3.39E-01	6.10E-01		-1.16E+01	6.72E-08	Down	Unigene110000_All//gi|225016141|gb|ACN78965.1|//3.00E-06//Rpp4 candidate 1 [Glycine max]		Unigene110000_All//9.00E-116//ES810366				nr -
Unigene110003_All	732	0.00	0.00	0.00	0.00	0.00	0.00	2.77	0.34	0.44	null	null		null	null		null	null		null	null		-3.02E+00	9.07E-09	Down	-2.67E+00	3.89E-08	Down							
Unigene110007_All	516	0.20	0.00	0.00	0.39	0.27	0.96	2.95	0.65	1.00	-7.66E+00	3.76E-01		-7.66E+00	3.98E-01		-5.33E-01	7.49E-01		1.32E+00	2.52E-01		-2.19E+00	5.96E-05	Down	-1.56E+00	1.38E-03				Unigene110007_All//7.00E-170//AW187731				
Unigene110011_All	1620	0.65	0.06	0.07	0.15	0.09	0.08	11.98	7.56	8.75	-3.37E+00	2.13E-04	Down	-3.30E+00	4.59E-04	Down	-8.55E-01	5.74E-01		-8.79E-01	6.17E-01		-6.64E-01	3.05E-09		-4.52E-01	3.23E-05		Unigene110011_All//gi|3152598|gb|AAC17079.1|//1.00E-155//Contains similarity to C2-HC type zinc finger protein C.e-MyT1 gb|U67079 from C. elegans and to hypersensitivity-related gene 201 isolog T28M21.14 from A. thaliana BAC gb|AF002109 [Arabidopsis thaliana]	Unigene110011_All//1.00E-150//AT1G78990| transferase family protein	Unigene110011_All//0.00E+00//DW488553		ko00940|Phenylpropanoid biosynthesis//ko00945|Stilbenoid, diarylheptanoid and gingerol biosynthesis//ko00941|Flavonoid biosynthesis		nr +
Unigene110013_All	365	0.00	0.00	0.00	0.00	0.00	0.00	4.75	0.23	0.22	null	null		null	null		null	null		null	null		-4.37E+00	6.16E-10	Down	-4.44E+00	1.94E-10	Down	Unigene110013_All//gi|159465565|ref|XP_001690993.1|//2.00E-30//ribosomal protein S23, component of cytosolic 80S ribosome and 40S small subunit [Chlamydomonas reinhardtii] >gi|158279679|gb|EDP05439.1| ribosomal protein S23, component of cytosolic 80S ribosome and 40S small subunit [Chlamydomonas reinhardtii]	Unigene110013_All//5.00E-20//AT5G02960| 40S ribosomal protein S23 (RPS23B)	Unigene110013_All//7.00E-09//TC139937		ko03010|Ribosome		nr +
Unigene110016_All	302	0.00	0.00	0.00	0.33	0.00	0.00	2.38	0.14	0.40	null	null		null	null		-8.37E+00	3.16E-01		-8.37E+00	4.05E-01		-4.10E+00	2.91E-04	Down	-2.59E+00	2.81E-03		Unigene110016_All//gi|4914318|gb|AAD32866.1|AC005489_4//2.00E-07//F14N23.4 [Arabidopsis thaliana]	Unigene110016_All//2.00E-06//AT1G40390| FUNCTIONS IN: molecular_function unknown; INVOLVED IN: biological_process unknown; LOCATED IN: cellular_component unknown; CONTAINS InterPro DOMAIN/s: Endonuclease/exonuclease/phosphatase (InterPro:IPR005135), RNA-directed DNA polymerase (reverse transcriptase), related (InterPro:IPR015706); BEST Arabidopsis thaliana protein match is: unknown protein (TAIR:AT1G43760.1); Has 480 Blast hits to 479 proteins in 10 species: Archae - 0; Bacteria - 0; Metazoa - 0; Fungi - 0; Plants - 480; Viruses - 0; Other Eukaryotes - 0 (source: NCBI BLink).					nr -
Unigene110017_All	254	1.03	0.40	0.42	0.00	0.27	0.00	16.48	8.56	4.08	-1.37E+00	3.83E-01		-1.30E+00	4.37E-01		8.08E+00	6.15E-01		null	null		-9.46E-01	3.80E-04		-2.01E+00	1.26E-11	Down			Unigene110017_All//6.00E-107//ES801549				
Unigene110024_All	517	0.00	0.29	0.00	0.19	0.07	0.09	1.88	0.24	0.31	8.20E+00	2.36E-01		null	null		-1.53E+00	5.88E-01		-1.14E+00	6.79E-01		-2.96E+00	1.89E-04	Down	-2.61E+00	3.36E-04	Down	Unigene110024_All//gi|15239682|ref|NP_200273.1|//6.00E-21//ankyrin repeat family protein [Arabidopsis thaliana]	Unigene110024_All//1.00E-22//AT5G54620| ankyrin repeat family protein					nr -
Unigene11003_All	395	0.13	0.13	0.40	0.63	0.52	0.11	2.35	2.22	0.20	-4.65E-02	9.87E-01		1.61E+00	5.12E-01		-2.70E-01	8.23E-01		-2.46E+00	2.24E-01		-8.37E-02	9.05E-01		-3.55E+00	3.97E-05	Down			Unigene11003_All//4.00E-23//FL577168				

Unigene110032_All	341	0.00	0.00	0.00	0.00	0.00	0.00	3.10	0.98	0.00	null	null		null	null		null	null		null	null		-1.66E+00	8.55E-03		-1.16E+01	6.72E-08	Down	Unigene110032_All//gi|37780051|gb|AAP32198.1|//1.00E-25//cysteine protease 12 [Trifolium repens]	Unigene110032_All//2.00E-25//AT5G60360| AALP (Arabidopsis aleurain-like protease); cysteine-type peptidase					nr -
Unigene110033_All	573	0.18	0.00	0.00	0.35	0.24	0.16	2.21	0.22	0.63	-7.51E+00	3.76E-01		-7.51E+00	3.98E-01		-5.32E-01	7.49E-01		-1.14E+00	5.70E-01		-3.34E+00	3.42E-06	Down	-1.82E+00	1.09E-03		Unigene110033_All//gi|224061531|ref|XP_002300526.1|//8.00E-54//AP2/ERF domain-containing transcription factor [Populus trichocarpa] >gi|222847784|gb|EEE85331.1| AP2/ERF domain-containing transcription factor [Populus trichocarpa]	Unigene110033_All//3.00E-10//AT5G19790| RAP2.11 (related to AP2 11); DNA binding / transcription factor	Unigene110033_All//0.00E+00//gi|78329508|gb|DT549782.1|DT549782	AP2-EREBP			nr +
Unigene110034_All	251	0.00	0.00	0.00	0.00	0.00	0.00	2.36	1.33	0.00	null	null		null	null		null	null		null	null		-8.24E-01	3.48E-01		-1.12E+01	1.41E-04	Down	Unigene110034_All//gi|116061061|emb|CAL56449.1|//8.00E-38//GTP-binding protein (ISS) [Ostreococcus tauri]	Unigene110034_All//3.00E-36//AT5G20010| RAN-1; GTP binding / GTPase/ protein binding	Unigene110034_All//2.00E-08//ES831931				nr -
Unigene110035_All	654	0.00	0.00	0.00	0.00	0.00	0.00	3.56	0.38	0.61	null	null		null	null		null	null		null	null		-3.21E+00	1.59E-10	Down	-2.55E+00	8.83E-09	Down	Unigene110035_All//sp|P40414|TPM2_YEAST//7.00E-06//Tropomyosin-2 OS=Saccharomyces cerevisiae GN=TPM2 PE=1 SV=1						swissprot -
Unigene110037_All	259	0.00	0.00	0.00	0.00	0.00	0.00	3.26	0.00	0.00	null	null		null	null		null	null		null	null		-1.17E+01	4.39E-06	Down	-1.17E+01	2.21E-06	Down							
Unigene110038_All	700	0.00	0.00	0.00	0.00	0.00	0.00	2.72	0.36	0.46	null	null		null	null		null	null		null	null		-2.92E+00	4.98E-08	Down	-2.58E+00	2.06E-07	Down	Unigene110038_All//gi|224118242|ref|XP_002331505.1|//9.00E-25//NAC domain protein, IPR003441 [Populus trichocarpa] >gi|222873841|gb|EEF10972.1| NAC domain protein, IPR003441 [Populus trichocarpa]						nr -
Unigene110046_All	372	0.00	0.00	0.00	0.00	0.00	0.00	1.82	0.79	0.00	null	null		null	null		null	null		null	null		-1.21E+00	1.34E-01		-1.08E+01	3.57E-05	Down	Unigene110046_All//gi|156554296|ref|XP_001602465.1|//5.00E-28//PREDICTED: similar to S14e ribosomal protein [Nasonia vitripennis]	Unigene110046_All//2.00E-27//AT3G11510| 40S ribosomal protein S14 (RPS14B)	Unigene110046_All//7.00E-12//CD485874		ko03010|Ribosome		nr +
Unigene110051_All	528	0.00	0.00	0.00	0.00	0.00	0.00	3.12	0.16	0.00	null	null		null	null		null	null		null	null		-4.30E+00	2.19E-09	Down	-1.16E+01	3.45E-12	Down	Unigene110051_All//gi|226495867|ref|NP_001151716.1|//1.00E-12//LOC100285352 [Zea mays] >gi|195649275|gb|ACG44105.1| actin-depolymerizing factor [Zea mays]	Unigene110051_All//6.00E-13//AT5G52360| ADF10 (ACTIN DEPOLYMERIZING FACTOR 10); actin binding					nr +
Unigene110055_All	415	0.00	0.00	0.00	0.00	0.00	0.00	5.09	0.91	0.67	null	null		null	null		null	null		null	null		-2.49E+00	1.42E-07	Down	-2.92E+00	3.37E-09	Down			Unigene110055_All//5.00E-124//gi|189098583|gb|FG551735.1|FG551735				
Unigene110056_All	932	0.00	0.00	0.00	0.21	0.22	0.00	3.67	1.08	1.92	null	null		null	null		5.23E-02	9.89E-01		-7.74E+00	1.34E-01		-1.77E+00	5.77E-08	Down	-9.34E-01	1.23E-03		Unigene110056_All//gi|297810727|ref|XP_002873247.1|//1.00E-105//carboxy-lyase [Arabidopsis lyrata subsp. lyrata] >gi|297319084|gb|EFH49506.1| carboxy-lyase [Arabidopsis lyrata subsp. lyrata]	Unigene110056_All//6.00E-94//AT5G06300| carboxy-lyase	Unigene110056_All//6.00E-33//ES812867				nr +
Unigene110059_All	298	0.00	0.00	0.00	0.00	0.00	0.00	4.26	0.56	0.80	null	null		null	null		null	null		null	null		-2.92E+00	1.46E-05	Down	-2.41E+00	7.19E-05	Down							
Unigene110065_All	507	0.00	0.00	0.00	0.00	0.00	0.00	1.92	0.41	0.00	null	null		null	null		null	null		null	null		-2.22E+00	1.82E-03		-1.09E+01	2.72E-07	Down	Unigene110065_All//gi|226494632|ref|NP_001152668.1|//6.00E-48//elongation factor 1-alpha [Zea mays] >gi|195658719|gb|ACG48827.1| elongation factor 1-alpha [Zea mays]	Unigene110065_All//7.00E-45//AT5G60390| elongation factor 1-alpha / EF-1-alpha					nr -
Unigene110071_All	224	0.23	0.00	0.00	0.22	0.31	0.00	3.59	0.37	0.89	-7.87E+00	6.19E-01		-7.87E+00	6.31E-01		4.67E-01	9.61E-01		-7.80E+00	6.43E-01		-3.26E+00	4.48E-04	Down	-2.01E+00	7.11E-03				Unigene110071_All//2.00E-39//DW510835				
Unigene110073_All	498	0.00	0.00	0.00	0.00	0.00	0.00	1.27	0.76	0.00	null	null		null	null		null	null		null	null		-7.54E-01	3.73E-01		-1.03E+01	7.12E-05	Down	Unigene110073_All//gi|159465349|ref|XP_001690885.1|//3.00E-37//ribosomal protein L18, component of cytosolic 80S ribosome and 60S large subunit [Chlamydomonas reinhardtii] >gi|158279571|gb|EDP05331.1| ribosomal protein L18, component of cytosolic 80S ribosome and 60S large subunit [Chlamydomonas reinhardtii]	Unigene110073_All//2.00E-35//AT5G27850| 60S ribosomal protein L18 (RPL18C)			ko03010|Ribosome		nr -
Unigene110077_All	397	0.00	0.00	0.00	0.00	0.00	0.00	2.56	0.21	0.00	null	null		null	null		null	null		null	null		-3.60E+00	2.38E-05	Down	-1.13E+01	1.35E-07	Down							
Unigene11008_All	647	0.00	0.00	0.00	0.00	0.00	0.00	11.63	1.55	1.35	null	null		null	null		null	null		null	null		-2.91E+00	9.92E-30	Down	-3.10E+00	9.26E-33	Down	Unigene11008_All//gi|30683788|ref|NP_850114.1|//4.00E-42//FRU (FER-LIKE REGULATOR OF IRON UPTAKE); DNA binding / transcription factor [Arabidopsis thaliana] >gi|122242304|sp|Q0V7X4.1|FIT_ARATH RecName: Full=Transcription factor FER-LIKE IRON DEFICIENCY-INDUCED TRANSCRIPTION FACTOR; AltName: Full=Transcription factor EN 43; AltName: Full=Transcription factor Fe-DEFICIENCY INDUCED TRANSCRIPTION FACTOR 1; AltName: Full=FER-LIKE REGULATOR OF IRON UPTAKE; AltName: Full=bHLH transcription factor bHLH029; AltName: Full=Basic helix-loop-helix protein 29; Short=bHLH 29; Short=AtbHLH29 >gi|111074360|gb|ABH04553.1| At2g28160 [Arabidopsis thaliana]	Unigene11008_All//4.00E-26//AT2G28160| FRU (FER-LIKE REGULATOR OF IRON UPTAKE); DNA binding / transcription factor	Unigene11008_All//0.00E+00//CA994022	bHLH	ko04626|Plant-pathogen interaction	GO:0005515//GO:0006826//GO:0010038//GO:0010468//GO:0043231	nr -
Unigene110080_All	1010	0.00	0.20	0.00	0.00	0.00	0.00	4.31	1.32	1.38	7.65E+00	1.37E-01		null	null		null	null		null	null		-1.70E+00	1.94E-09	Down	-1.64E+00	2.46E-09	Down	Unigene110080_All//gi|79570100|ref|NP_181364.2|//5.00E-33//DNA binding / transcription factor [Arabidopsis thaliana]	Unigene110080_All//4.00E-35//AT2G38300| DNA binding / transcription factor		G2-like			nr +
Unigene110081_All	583	0.00	0.00	0.00	0.00	0.12	0.08	1.89	5.23	0.21	null	null		null	null		6.88E+00	6.15E-01		6.28E+00	7.02E-01		1.47E+00	1.10E-05	Up	-3.20E+00	1.63E-05	Down	Unigene110081_All//gi|186501250|ref|NP_849973.3|//4.00E-45//metal ion binding [Arabidopsis thaliana]	Unigene110081_All//7.00E-47//AT2G18196| metal ion binding	Unigene110081_All//2.00E-56//DN800214				nr +
Unigene110084_All	262	0.00	0.00	0.00	0.95	0.13	0.34	2.42	0.00	0.15	null	null		null	null		-2.86E+00	1.37E-01		-1.46E+00	4.07E-01		-1.12E+01	1.23E-04	Down	-3.99E+00	5.82E-04	Down							
Unigene110085_All	382	0.00	0.00	0.00	0.00	0.00	0.00	1.77	0.11	0.42	null	null		null	null		null	null		null	null		-4.02E+00	5.36E-04	Down	-2.09E+00	1.27E-02								
Unigene110086_All	806	0.00	0.00	0.00	0.31	0.60	0.28	13.69	0.57	0.30	null	null		null	null		9.53E-01	4.24E-01		-1.41E-01	9.29E-01		-4.59E+00	1.14E-62	Down	-5.53E+00	2.17E-71	Down	Unigene110086_All//gi|297805842|ref|XP_002870805.1|//1.00E-105//ATTAP2 [Arabidopsis lyrata subsp. lyrata] >gi|297316641|gb|EFH47064.1| ATTAP2 [Arabidopsis lyrata subsp. lyrata]	Unigene110086_All//9.00E-82//AT5G39040| ATTAP2; ATPase, coupled to transmembrane movement of substances / transporter	Unigene110086_All//7.00E-91//TC135398		ko02010|ABC transporters		nr +
Unigene110097_All	269	0.00	0.00	0.00	0.00	0.00	0.00	1.89	0.47	0.00	null	null		null	null		null	null		null	null		-2.02E+00	5.21E-02		-1.09E+01	5.55E-04	Down	Unigene110097_All//gi|15226694|ref|NP_181583.1|//2.00E-19//40S ribosomal protein S26 (RPS26A) [Arabidopsis thaliana] >gi|115502826|sp|Q8LPJ7.2|RS262_ARATH RecName: Full=40S ribosomal protein S26-2 >gi|2651314|gb|AAB87594.1| 40S ribosomal protein S26 [Arabidopsis thaliana] >gi|21555493|gb|AAM63871.1| 40S ribosomal protein S26 [Arabidopsis thaliana] >gi|56236112|gb|AAV84512.1| At2g40510 [Arabidopsis thaliana] >gi|111074312|gb|ABH04529.1| At2g40510 [Arabidopsis thaliana]	Unigene110097_All//9.00E-18//AT3G56340| 40S ribosomal protein S26 (RPS26C)			ko03010|Ribosome		nr -
Unigene110098_All	258	0.00	0.00	0.00	0.00	0.00	0.00	2.13	0.16	0.00	null	null		null	null		null	null		null	null		-3.72E+00	3.23E-03		-1.11E+01	2.81E-04	Down	Unigene110098_All//gi|156763856|emb|CAO99130.1|//5.00E-24//berberine bridge enzyme like protein [Nicotiana tabacum]	Unigene110098_All//1.00E-12//AT2G34790| MEE23 (MATERNAL EFFECT EMBRYO ARREST 23); FAD binding / catalytic/ electron carrier/ oxidoreductase					nr -
Unigene1101_All	287	0.00	0.00	0.00	0.52	0.48	0.63	2.80	5.10	9.72	null	null		null	null		-1.18E-01	9.11E-01		2.74E-01	9.05E-01		8.65E-01	8.03E-02		1.80E+00	6.57E-07	Up	Unigene1101_All//gi|81250791|gb|ABB69785.1|//6.00E-39//beta-glucan-binding protein 5 [Medicago truncatula]	Unigene1101_All//3.00E-28//AT1G18310| glycosyl hydrolase family 81 protein	Unigene1101_All//1.00E-114//gi|164309730|gb|EV483652.1|EV483652			GO:0004553//GO:0004871//GO:0009056	nr +
Unigene11010_All	635	0.00	0.00	0.00	0.00	0.00	0.00	1.20	1.38	7.65	null	null		null	null		null	null		null	null		2.06E-01	7.54E-01		2.68E+00	3.58E-18	Up	Unigene11010_All//gi|297850310|ref|XP_002893036.1|//4.00E-31//flavin-dependent monooxygenase 1 [Arabidopsis lyrata subsp. lyrata] >gi|297338878|gb|EFH69295.1| flavin-dependent monooxygenase 1 [Arabidopsis lyrata subsp. lyrata]	Unigene11010_All//6.00E-32//AT1G19250| FMO1 (FLAVIN-DEPENDENT MONOOXYGENASE 1); FAD binding / NADP or NADPH binding / electron carrier/ flavin-containing monooxygenase/ monooxygenase/ oxidoreductase			ko00380|Tryptophan metabolism	GO:0002218//GO:0005789//GO:0008152//GO:0009814//GO:0016709//GO:0030554//GO:0033554	nr +
Unigene110101_All	258	0.61	0.00	0.21	0.39	0.40	0.00	2.29	0.00	0.15	-9.25E+00	2.17E-01		-1.56E+00	5.34E-01		5.21E-02	9.68E-01		-8.59E+00	4.05E-01		-1.12E+01	2.40E-04	Down	-3.89E+00	1.10E-03				Unigene110101_All//2.00E-60//DR456209				ESTscan +
Unigene110103_All	321	0.00	0.00	0.00	0.00	0.00	0.00	4.08	0.00	0.00	null	null		null	null		null	null		null	null		-1.20E+01	2.56E-09	Down	-1.20E+01	9.85E-10	Down	Unigene110103_All//gi|51315784|sp|Q7Y1X1.1|EST_HEVBR//2.00E-10//RecName: Full=Esterase; AltName: Full=Early nodule-specific protein homolog; AltName: Full=Latex allergen Hev b 13; AltName: Allergen=Hev b 13; Flags: Precursor >gi|30909057|gb|AAP37470.1| ENSP-like protein [Hevea brasiliensis]	Unigene110103_All//3.00E-09//AT5G14450| GDSL-motif lipase/hydrolase family protein	Unigene110103_All//3.00E-57//DR453903				nr +
Unigene110106_All	285	0.00	0.00	0.00	0.00	0.00	0.00	2.08	0.29	0.00	null	null		null	null		null	null		null	null		-2.82E+00	7.18E-03		-1.10E+01	1.41E-04	Down	Unigene110106_All//gi|300262703|gb|EFJ46908.1|//2.00E-06//metalloproteinase, extracellular matrix glycoprotein VMP6 [Volvox carteri f. nagariensis]						nr -
Unigene110109_All	247	0.00	0.00	0.00	0.00	0.00	0.00	2.05	0.17	0.00	null	null		null	null		null	null		null	null		-3.60E+00	5.78E-03		-1.10E+01	5.55E-04	Down							ESTscan +
Unigene110110_All	261	0.00	0.00	0.00	0.00	0.00	0.17	1.94	0.80	0.00	null	null		null	null		null	null		7.43E+00	7.02E-01		-1.28E+00	1.86E-01		-1.09E+01	5.55E-04	Down			Unigene110110_All//3.00E-56//gi|84165786|gb|DR454434.1|DR454434				
Unigene110112_All	298	0.00	0.00	1.61	0.00	0.00	0.00	2.13	0.70	0.13	null	null		1.07E+01	7.49E-03		null	null		null	null		-1.60E+00	6.27E-02		-3.99E+00	5.82E-04	Down			Unigene110112_All//3.00E-07//DW500060				
Unigene110113_All	480	0.00	0.00	0.00	0.00	0.00	0.00	1.76	1.22	0.00	null	null		null	null		null	null		null	null		-5.31E-01	4.45E-01		-1.08E+01	2.21E-06	Down			Unigene110113_All//3.00E-24//gi|109856915|gb|DW485894.1|DW485894				
Unigene110115_All	631	0.00	0.00	0.00	0.00	0.00	0.00	0.87	2.12	0.00	null	null		null	null		null	null		null	null		1.28E+00	1.56E-02		-9.77E+00	2.81E-04	Down							

Unigene110119_All	250	0.00	0.00	0.00	0.00	0.00	0.00	2.03	1.84	0.00	null	null		null	null		null	null		null	null		-1.42E-01	8.76E-01		-1.10E+01	5.55E-04	Down	Unigene110119_All//gi|301097804|ref|XP_002897996.1|//3.00E-30//60S ribosomal protein L5 [Phytophthora infestans T30-4] >gi|262106441|gb|EEY64493.1| 60S ribosomal protein L5 [Phytophthora infestans T30-4]	Unigene110119_All//4.00E-29//AT3G25520| ATL5 (A. THALIANA RIBOSOMAL PROTEIN L5); 5S rRNA binding / structural constituent of ribosome			ko03010|Ribosome		nr +
Unigene11012_All	259	16.16	20.35	7.61	18.08	22.34	31.21	31.83	20.82	25.53	3.32E-01	2.18E-01		-1.09E+00	5.41E-04	Down	3.05E-01	2.57E-01		7.88E-01	8.89E-05		-6.13E-01	6.38E-04		-3.18E-01	7.78E-02				Unigene11012_All//4.00E-62//TC168595				
Unigene110121_All	288	0.00	0.00	0.00	0.00	0.00	0.16	1.76	0.44	0.00	null	null		null	null		null	null		7.29E+00	7.02E-01		-2.02E+00	5.21E-02		-1.08E+01	5.55E-04	Down							
Unigene110123_All	397	0.00	0.00	0.00	0.00	0.00	0.00	2.88	0.00	0.00	null	null		null	null		null	null		null	null		-1.15E+01	3.89E-08	Down	-1.15E+01	1.65E-08	Down	Unigene110123_All//gi|109939734|sp|P25776.2|ORYA_ORYSJ//5.00E-24//RecName: Full=Oryzain alpha chain; Flags: Precursor >gi|78192122|gb|ABB30151.1| oryzain alpha [Oryza sativa Japonica Group]	Unigene110123_All//9.00E-24//AT1G06260| cysteine proteinase, putative					nr -
Unigene110131_All	1894	0.00	0.05	0.00	0.00	0.00	0.00	1.16	1.54	0.17	5.74E+00	3.90E-01		null	null		null	null		null	null		4.12E-01	2.32E-01		-2.79E+00	4.04E-09	Down	Unigene110131_All//gi|115466826|ref|NP_001057012.1|//1.00E-132//Os06g0186700 [Oryza sativa Japonica Group] >gi|55773865|dbj|BAD72450.1| integral membrane transporter-like protein [Oryza sativa Japonica Group] >gi|113595052|dbj|BAF18926.1| Os06g0186700 [Oryza sativa Japonica Group]	Unigene110131_All//2.00E-125//AT5G25050| integral membrane transporter family protein					nr +
Unigene110134_All	333	2.20	2.28	0.00	1.20	1.65	0.81	10.54	7.15	8.37	5.32E-02	9.63E-01		-1.11E+01	4.13E-04	Down	4.67E-01	6.68E-01		-5.56E-01	6.38E-01		-5.59E-01	5.99E-02		-3.32E-01	2.62E-01		Unigene110134_All//gi|17933310|gb|AAL48237.1|AF446365_1//1.00E-36//At1g10950/T19D16_13 [Arabidopsis thaliana] >gi|15451172|gb|AAK96857.1| endomembrane protein EMP70 precusor isolog [Arabidopsis thaliana] >gi|20148415|gb|AAM10098.1| endomembrane protein EMP70 precusor isolog [Arabidopsis thaliana]	Unigene110134_All//6.00E-38//AT1G10950| endomembrane protein 70, putative	Unigene110134_All//1.00E-96//TC147189				nr +
Unigene11014_All	604	0.00	0.84	0.26	2.39	3.25	0.30	0.56	0.28	0.13	9.71E+00	4.05E-03		8.05E+00	2.40E-01		4.42E-01	3.82E-01		-3.00E+00	1.34E-05	Down	-1.02E+00	4.00E-01		-2.09E+00	1.07E-01		Unigene11014_All//gi|297832296|ref|XP_002884030.1|//3.00E-37//tetracycline transporter [Arabidopsis lyrata subsp. lyrata] >gi|297329870|gb|EFH60289.1| tetracycline transporter [Arabidopsis lyrata subsp. lyrata]	Unigene11014_All//1.00E-51//AT5G42210| FUNCTIONS IN: molecular_function unknown; INVOLVED IN: biological_process unknown; LOCATED IN: endomembrane system; CONTAINS InterPro DOMAIN/s: Major facilitator superfamily MFS-1 (InterPro:IPR011701), Major facilitator superfamily, general substrate transporter (InterPro:IPR016196); BEST Arabidopsis thaliana protein match is: tetracycline transporter (TAIR:AT2G16990.2); Has 985 Blast hits to 984 proteins in 348 species: Archae - 2; Bacteria - 541; Metazoa - 251; Fungi - 0; Plants - 39; Viruses - 0; Other Eukaryotes - 152 (source: NCBI BLink).	Unigene11014_All//0.00E+00//TC134017			GO:0006810//GO:0031410	nr +
Unigene110142_All	345	0.00	0.00	0.00	0.00	0.00	0.00	2.08	0.00	0.23	null	null		null	null		null	null		null	null		-1.10E+01	3.27E-05	Down	-3.17E+00	8.09E-04	Down							
Unigene110147_All	725	0.00	0.00	0.00	0.00	0.05	0.19	3.09	1.04	0.27	null	null		null	null		5.57E+00	8.37E-01		7.55E+00	3.12E-01		-1.57E+00	9.33E-05	Down	-3.49E+00	2.61E-11	Down							
Unigene11015_All	314	0.00	5.49	4.75	0.00	0.00	0.00	0.00	0.00	0.00	1.24E+01	7.99E-10	Up	1.22E+01	3.22E-08	Up	null	null		null	null		null	null		null	null		Unigene11015_All//gi|301095248|ref|XP_002896725.1|//6.00E-06//enoyl-CoA hydratase, mitochondrial precursor [Phytophthora infestans T30-4] >gi|262108786|gb|EEY66838.1| enoyl-CoA hydratase, mitochondrial precursor [Phytophthora infestans T30-4]	Unigene11015_All//3.00E-05//AT2G30660| 3-hydroxyisobutyryl-coenzyme A hydrolase, putative / CoA-thioester hydrolase, putative					nr -
Unigene110161_All	268	0.00	0.00	0.00	0.00	0.00	0.00	8.36	16.84	6.39	null	null		null	null		null	null		null	null		1.01E+00	8.10E-05	Up	-3.88E-01	3.12E-01				Unigene110161_All//2.00E-33//gi|109886409|gb|DW515379.1|DW515379				
Unigene110162_All	266	0.00	0.00	0.00	0.00	0.26	0.00	3.02	0.00	0.15	null	null		null	null		8.02E+00	6.15E-01		null	null		-1.16E+01	8.58E-06	Down	-4.33E+00	4.54E-05	Down			Unigene110162_All//6.00E-24//FL576545				ESTscan +
Unigene110170_All	404	0.00	0.00	0.00	0.00	0.00	0.22	2.41	1.97	0.20	null	null		null	null		null	null		7.80E+00	4.81E-01		-2.92E-01	6.31E-01		-3.61E+00	2.14E-05	Down			Unigene110170_All//2.00E-37//DN801068				
Unigene110172_All	300	0.00	0.00	0.00	0.00	0.00	0.00	2.96	0.42	0.00	null	null		null	null		null	null		null	null		-2.82E+00	5.65E-04	Down	-1.15E+01	1.10E-06	Down	Unigene110172_All//gi|300264920|gb|EFJ49114.1|//4.00E-06//hypothetical protein VOLCADRAFT_117426 [Volvox carteri f. nagariensis]						nr -
Unigene110175_All	240	0.00	0.00	0.00	0.00	0.14	0.00	13.39	3.66	5.81	null	null		null	null		7.16E+00	8.37E-01		null	null		-1.87E+00	4.78E-08	Down	-1.20E+00	8.77E-05	Down							
Unigene110178_All	273	0.00	0.00	0.00	0.00	0.00	0.00	2.32	0.77	0.00	null	null		null	null		null	null		null	null		-1.60E+00	6.27E-02		-1.12E+01	7.12E-05	Down							
Unigene110179_All	365	0.00	0.00	0.00	0.00	0.00	0.00	2.55	0.23	0.00	null	null		null	null		null	null		null	null		-3.48E+00	7.79E-05	Down	-1.13E+01	5.48E-07	Down	Unigene110179_All//gi|76161008|gb|ABA40467.1|//3.00E-17//glycoprotein-like protein [Solanum tuberosum]	Unigene110179_All//9.00E-18//AT4G27090| 60S ribosomal protein L14 (RPL14B)			ko03010|Ribosome		nr +
Unigene11018_All	405	1.42	3.25	2.37	1.72	2.55	2.56	3.97	1.03	3.64	1.19E+00	4.50E-02		7.36E-01	3.23E-01		5.67E-01	4.46E-01		5.75E-01	4.27E-01		-1.94E+00	1.42E-04	Down	-1.24E-01	7.77E-01		Unigene11018_All//gi|224064045|ref|XP_002301364.1|//3.00E-39//chromatin remodeling complex subunit [Populus trichocarpa] >gi|222843090|gb|EEE80637.1| chromatin remodeling complex subunit [Populus trichocarpa]	Unigene11018_All//3.00E-30//AT2G46020| transcription regulatory protein SNF2, putative				GO:0003676//GO:0006338//GO:0010160//GO:0010468//GO:0016818//GO:0043231//GO:0043234//GO:0044444	nr +
Unigene110180_All	354	0.00	0.00	0.00	0.00	0.00	0.00	2.15	0.24	0.11	null	null		null	null		null	null		null	null		-3.19E+00	7.93E-04	Down	-4.26E+00	8.66E-05	Down							ESTscan +
Unigene110181_All	261	0.00	0.00	0.00	0.00	0.00	0.00	2.11	0.00	0.00	null	null		null	null		null	null		null	null		-1.10E+01	4.62E-04	Down	-1.10E+01	2.81E-04	Down	Unigene110181_All//gi|159483211|ref|XP_001699654.1|//1.00E-20//ribosomal protein L27a, component of cytosolic 80S ribosome and 60S large subunit [Chlamydomonas reinhardtii] >gi|158281596|gb|EDP07350.1| ribosomal protein L27a, component of cytosolic 80S ribosome and 60S large subunit [Chlamydomonas reinhardtii]	Unigene110181_All//3.00E-20//AT1G70600| structural constituent of ribosome			ko03010|Ribosome		nr -
Unigene110182_All	516	0.00	0.00	0.00	0.00	0.00	0.00	2.29	0.24	0.23	null	null		null	null		null	null		null	null		-3.24E+00	1.10E-05	Down	-3.31E+00	4.89E-06	Down							
Unigene110184_All	557	0.00	0.00	0.00	0.00	0.00	0.16	0.53	0.23	3.43	null	null		null	null		null	null		7.34E+00	4.80E-01		-1.24E+00	3.73E-01		2.69E+00	1.24E-07	Up	Unigene110184_All//gi|145335895|ref|NP_173359.3|//4.00E-68//FMO1 (FLAVIN-DEPENDENT MONOOXYGENASE 1); FAD binding / NADP or NADPH binding / electron carrier/ flavin-containing monooxygenase/ monooxygenase/ oxidoreductase [Arabidopsis thaliana] >gi|75174733|sp|Q9LMA1.1|FMO1_ARATH RecName: Full=Probable flavin-containing monooxygenase 1 >gi|8954062|gb|AAF82235.1|AC069143_11 Contains similarity to a dimethylaniline monooxygenase (N-oxide forming) 2 (pulmonary flavin-containing monooxygenase 2) (FMO 2) (Dimethylaniline oxidase 2) (FMO 1B1) from Oryctolagus cuniculus gi|120434 and contains a flavin-binding monooxygenase-like PF|00743 domain [Arabidopsis thaliana]	Unigene110184_All//8.00E-70//AT1G19250| FMO1 (FLAVIN-DEPENDENT MONOOXYGENASE 1); FAD binding / NADP or NADPH binding / electron carrier/ flavin-containing monooxygenase/ monooxygenase/ oxidoreductase					nr +
Unigene110186_All	901	0.00	0.00	0.00	0.00	0.00	0.00	4.55	0.05	0.04	null	null		null	null		null	null		null	null		-6.62E+00	2.45E-27	Down	-6.69E+00	1.85E-28	Down	Unigene110186_All//gi|4139170|gb|AAD03711.1|//1.00E-129//elongation translation factor 1 alpha [Cyanophora paradoxa]	Unigene110186_All//3.00E-123//AT5G60390| elongation factor 1-alpha / EF-1-alpha	Unigene110186_All//8.00E-20//TC170429				nr -
Unigene110187_All	276	0.00	0.00	0.00	0.00	0.00	0.00	1.84	0.30	0.00	null	null		null	null		null	null		null	null		-2.60E+00	2.05E-02		-1.08E+01	5.55E-04	Down							
Unigene110193_All	476	0.00	0.00	0.00	0.00	0.00	0.00	2.22	1.05	0.00	null	null		null	null		null	null		null	null		-1.08E+00	7.50E-02		-1.11E+01	6.72E-08	Down	Unigene110193_All//gi|9188543|dbj|BAA99561.1|//2.00E-34//alpha-tubulin [Chlorella vulgaris]	Unigene110193_All//8.00E-24//AT5G19780| TUA5; structural constituent of cytoskeleton	Unigene110193_All//9.00E-09//gi|54122405|gb|CO497914.1|CO497914		ko04145|Phagosome		nr +
Unigene110196_All	509	0.00	1.19	0.21	0.00	0.34	0.35	2.33	0.41	0.63	1.02E+01	1.17E-03		7.71E+00	4.02E-01		8.40E+00	2.27E-01		8.47E+00	1.95E-01		-2.50E+00	1.47E-04	Down	-1.89E+00	1.24E-03		Unigene110196_All//gi|224077504|ref|XP_002305276.1|//3.00E-15//GRAS domain protein [Populus trichocarpa] >gi|222848240|gb|EEE85787.1| GRAS domain protein [Populus trichocarpa]		Unigene110196_All//1.00E-112//TC153520				nr -
Unigene110197_All	246	0.00	0.00	0.00	0.00	0.00	0.00	3.27	0.34	0.65	null	null		null	null		null	null		null	null		-3.26E+00	4.48E-04	Down	-2.33E+00	2.82E-03								ESTscan -
Unigene110199_All	279	0.00	0.00	0.00	0.00	0.00	0.00	1.97	0.15	0.00	null	null		null	null		null	null		null	null		-3.72E+00	3.23E-03		-1.09E+01	2.81E-04	Down	Unigene110199_All//gi|224000593|ref|XP_002289969.1|//7.00E-14//the actin binding protein cofilin-like protein [Thalassiosira pseudonana CCMP1335] >gi|220975177|gb|EED93506.1| the actin binding protein cofilin-like protein [Thalassiosira pseudonana CCMP1335]	Unigene110199_All//4.00E-12//AT2G16700| ADF5 (ACTIN DEPOLYMERIZING FACTOR 5); actin binding					nr +
Unigene110202_All	542	0.00	0.00	0.00	0.00	0.00	0.00	1.95	0.69	0.44	null	null		null	null		null	null		null	null		-1.49E+00	1.64E-02		-2.14E+00	9.36E-04	Down							ESTscan -
Unigene110205_All	353	0.74	0.00	0.00	1.27	0.39	0.00	2.40	0.95	0.23	-9.53E+00	6.72E-02		-9.53E+00	8.59E-02		-1.70E+00	1.35E-01		-1.03E+01	5.79E-03		-1.34E+00	5.68E-02		-3.41E+00	1.35E-04	Down			Unigene110205_All//4.00E-07//CO105291				
Unigene110206_All	224	0.00	0.00	0.00	0.00	0.00	0.00	2.27	0.19	0.00	null	null		null	null		null	null		null	null		-3.60E+00	5.78E-03		-1.11E+01	5.55E-04	Down	Unigene110206_All//gi|300508148|dbj|BAJ10906.1|//7.00E-23//elongation factor like [Grateloupia subpectinata]						nr +
Unigene110210_All	272	0.00	0.00	0.00	0.00	0.00	0.00	2.49	0.15	0.00	null	null		null	null		null	null		null	null		-4.02E+00	5.36E-04	Down	-1.13E+01	3.57E-05	Down	Unigene110210_All//gi|116222123|gb|ABJ80932.1|//2.00E-36//actin [Leucocryptos marina]	Unigene110210_All//7.00E-33//AT5G09810| ACT7 (ACTIN 7); structural constituent of cytoskeleton			ko04145|Phagosome		nr -

Unigene110212_All	275	0.00	0.00	0.00	0.00	0.00	0.00	1.85	1.06	0.00	null	null		null	null		null	null		null	null		-7.94E-01	4.02E-01		-1.08E+01	5.55E-04	Down	Unigene110212_All//gi|253760977|ref|XP_002489033.1|//7.00E-07//hypothetical protein SORBIDRAFT_0351s002020 [Sorghum bicolor] >gi|241947330|gb|EES20475.1| hypothetical protein SORBIDRAFT_0351s002020 [Sorghum bicolor]		Unigene110212_All//3.00E-16//EX170698				nr +
Unigene11022_All	2745	0.50	1.22	0.95	2.09	2.95	3.06	3.17	2.12	4.17	1.30E+00	2.16E-04	Up	9.40E-01	2.21E-02		4.98E-01	1.24E-02		5.52E-01	5.55E-03		-5.84E-01	7.91E-04		3.93E-01	7.68E-03		Unigene11022_All//gi|224064045|ref|XP_002301364.1|//0.00E+00//chromatin remodeling complex subunit [Populus trichocarpa] >gi|222843090|gb|EEE80637.1| chromatin remodeling complex subunit [Populus trichocarpa]	Unigene11022_All//4.00E-137//AT2G46020| transcription regulatory protein SNF2, putative	Unigene11022_All//0.00E+00//ES845478			GO:0003676//GO:0006338//GO:0010160//GO:0010468//GO:0016818//GO:0043231//GO:0043234//GO:0044444	nr +
Unigene110220_All	880	0.00	0.00	0.00	0.00	0.00	0.10	2.31	0.57	0.81	null	null		null	null		null	null		6.68E+00	4.80E-01		-2.02E+00	8.03E-06	Down	-1.50E+00	2.57E-04	Down	Unigene110220_All//gi|159138929|gb|ABW89464.1|//5.00E-89//glutamine synthetase [Gossypium hirsutum] >gi|159138931|gb|ABW89465.1| glutamine synthetase [Gossypium hirsutum]	Unigene110220_All//5.00E-86//AT5G16570| GLN1;4; glutamate-ammonia ligase	Unigene110220_All//0.00E+00//gi|78342884|gb|DT563158.1|DT563158		ko00910|Nitrogen metabolism//ko00250|Alanine, aspartate and glutamate metabolism//ko00330|Arginine and proline metabolism		nr -
Unigene110224_All	566	0.00	0.00	0.00	0.09	0.00	0.00	2.69	1.18	0.84	null	null		null	null		-6.46E+00	5.62E-01		-6.46E+00	6.43E-01		-1.19E+00	1.47E-02		-1.67E+00	7.06E-04	Down			Unigene110224_All//1.00E-54//DV849456				
Unigene11023_All	2339	1.23	2.56	2.85	4.32	4.53	4.44	5.42	3.11	6.27	1.06E+00	1.98E-05	Up	1.21E+00	6.90E-07	Up	6.88E-02	7.79E-01		3.88E-02	8.59E-01		-8.03E-01	1.79E-08		2.09E-01	1.22E-01		Unigene11023_All//gi|224064045|ref|XP_002301364.1|//0.00E+00//chromatin remodeling complex subunit [Populus trichocarpa] >gi|222843090|gb|EEE80637.1| chromatin remodeling complex subunit [Populus trichocarpa]	Unigene11023_All//0.00E+00//AT2G46020| transcription regulatory protein SNF2, putative	Unigene11023_All//3.00E-77//ES845478			GO:0003676//GO:0006338//GO:0010160//GO:0010468//GO:0017111//GO:0032559//GO:0043231//GO:0043234//GO:0044444	nr -
Unigene110233_All	463	0.00	0.00	0.00	0.00	0.00	0.00	2.74	0.90	0.00	null	null		null	null		null	null		null	null		-1.60E+00	4.42E-03		-1.14E+01	1.99E-09	Down	Unigene110233_All//gi|42569129|ref|NP_179444.2|//2.00E-14//cupin family protein [Arabidopsis thaliana]				ko03040|Spliceosome		nr -
Unigene110235_All	299	0.00	0.00	0.00	0.00	0.23	0.00	4.67	0.28	1.20	null	null		null	null		7.85E+00	6.15E-01		null	null		-4.06E+00	9.64E-08	Down	-1.96E+00	2.74E-04	Down							
Unigene110237_All	946	0.00	0.00	0.00	0.00	0.00	0.00	2.23	0.27	0.00	null	null		null	null		null	null		null	null		-3.08E+00	2.88E-09	Down	-1.11E+01	1.40E-15	Down	Unigene110237_All//gi|50508930|dbj|BAD31835.1|//2.00E-07//putative high-affinity potassium transporter [Oryza sativa Japonica Group] >gi|215694830|dbj|BAG90021.1| unnamed protein product [Oryza sativa Japonica Group]						nr -
Unigene110238_All	402	0.65	0.50	0.53	0.25	0.60	0.11	3.37	0.52	0.99	-3.68E-01	7.79E-01		-2.97E-01	8.32E-01		1.27E+00	5.32E-01		-1.14E+00	6.79E-01		-2.69E+00	1.77E-05	Down	-1.76E+00	9.57E-04	Down	Unigene110238_All//gi|145334291|ref|NP_001078527.1|//9.00E-20//DME (DEMETER); DNA N-glycosylase/ DNA-(apurinic or apyrimidinic site) lyase [Arabidopsis thaliana] >gi|108935833|sp|Q8LK56.2|DME_ARATH RecName: Full=Transcriptional activator DEMETER; AltName: Full=DNA glycosylase-related protein DME >gi|84782664|gb|ABC61677.1| DNA glycosylase DEMETER [Arabidopsis thaliana]	Unigene110238_All//1.00E-18//AT5G04560| DME (DEMETER); DNA N-glycosylase/ DNA-(apurinic or apyrimidinic site) lyase			ko03410|Base excision repair		nr -
Unigene110240_All	623	0.08	0.00	0.00	0.40	0.28	0.87	11.20	3.02	4.86	-6.39E+00	6.18E-01		-6.39E+00	6.31E-01		-5.33E-01	7.13E-01		1.12E+00	3.05E-01		-1.89E+00	8.16E-17	Down	-1.20E+00	2.30E-09	Down	Unigene110240_All//gi|224128414|ref|XP_002329156.1|//3.00E-26//nbs-lrr resistance protein [Populus trichocarpa] >gi|222869825|gb|EEF06956.1| nbs-lrr resistance protein [Populus trichocarpa]	Unigene110240_All//1.00E-09//AT4G27220| disease resistance protein (NBS-LRR class), putative	Unigene110240_All//2.00E-47//CO080429		ko04626|Plant-pathogen interaction		nr +
Unigene110242_All	1635	0.00	0.00	0.00	0.00	0.00	0.00	3.36	2.89	0.05	null	null		null	null		null	null		null	null		-2.19E-01	3.98E-01		-6.11E+00	4.43E-37	Down	Unigene110242_All//gi|115463921|ref|NP_001055560.1|//1.00E-120//Os05g0417100 [Oryza sativa Japonica Group] >gi|113579111|dbj|BAF17474.1| Os05g0417100 [Oryza sativa Japonica Group]	Unigene110242_All//3.00E-116//AT5G36950| DegP10 (DegP protease 10); catalytic/ protein binding / serine-type endopeptidase/ serine-type peptidase					nr -
Unigene110246_All	306	0.51	0.17	0.00	0.33	0.56	0.44	1.80	0.55	0.00	-1.63E+00	4.89E-01		-9.00E+00	2.46E-01		7.89E-01	7.72E-01		4.44E-01	8.62E-01		-1.72E+00	7.23E-02		-1.08E+01	2.81E-04	Down							
Unigene110253_All	448	0.00	0.00	0.00	0.00	0.00	0.00	1.23	0.00	4.36	null	null		null	null		null	null		null	null		-1.03E+01	4.62E-04	Down	1.83E+00	3.51E-05	Up	Unigene110253_All//gi|68299217|emb|CAJ13709.1|//6.00E-19//glutathione S-transferase 12 [Capsicum chinense]	Unigene110253_All//4.00E-11//AT5G62480| ATGSTU9 (ARABIDOPSIS THALIANA GLUTATHIONE S-TRANSFERASE TAU 9); glutathione transferase			ko00480|Glutathione metabolism		nr +
Unigene110256_All	422	0.00	0.00	0.50	0.47	0.00	0.00	4.21	2.87	0.85	null	null		8.98E+00	1.42E-01		-8.88E+00	8.47E-02		-8.88E+00	1.34E-01		-5.51E-01	2.24E-01		-2.31E+00	3.08E-06	Down							
Unigene110257_All	405	0.00	0.00	0.00	0.00	0.00	0.00	1.88	1.03	0.10	null	null		null	null		null	null		null	null		-8.65E-01	2.39E-01		-4.26E+00	8.66E-05	Down							
Unigene110258_All	405	0.00	0.00	0.00	0.12	0.00	0.11	5.32	1.65	21.15	null	null		null	null		-6.94E+00	5.62E-01		-1.42E-01	9.56E-01		-1.69E+00	5.26E-05	Down	1.99E+00	2.74E-22	Up			Unigene110258_All//1.00E-100//gi|195983813|gb|FL576553.1|FL576553				
Unigene110259_All	272	0.00	0.00	0.00	0.00	0.00	0.00	2.49	1.08	0.00	null	null		null	null		null	null		null	null		-1.21E+00	1.34E-01		-1.13E+01	3.57E-05	Down			Unigene110259_All//5.00E-15//DW479423				ESTscan -
Unigene110266_All	264	0.00	0.00	0.00	0.00	0.00	0.00	2.88	0.32	0.00	null	null		null	null		null	null		null	null		-3.19E+00	7.93E-04	Down	-1.15E+01	8.88E-06	Down							
Unigene110268_All	337	0.00	0.00	0.00	0.00	0.00	0.00	2.01	0.00	0.00	null	null		null	null		null	null		null	null		-1.10E+01	6.34E-05	Down	-1.10E+01	3.57E-05	Down	Unigene110268_All//gi|42569129|ref|NP_179444.2|//4.00E-11//cupin family protein [Arabidopsis thaliana]						nr -
Unigene11027_All	940	1.78	1.62	4.65	6.78	4.47	5.57	4.18	2.13	4.87	-1.39E-01	7.69E-01		1.38E+00	1.09E-05	Up	-6.02E-01	6.30E-03		-2.83E-01	2.70E-01		-9.71E-01	4.39E-04		2.21E-01	3.99E-01		Unigene11027_All//gi|186510768|ref|NP_190388.2|//5.00E-46//DNA binding / protein binding / transcription regulator [Arabidopsis thaliana] >gi|186510770|ref|NP_850669.2| DNA binding / protein binding / transcription regulator [Arabidopsis thaliana]	Unigene11027_All//1.00E-47//AT3G48050| DNA binding / protein binding / transcription regulator	Unigene11027_All//0.00E+00//gi|73866001|gb|DT468739.1|DT468739				nr -
Unigene110279_All	296	0.00	0.00	0.00	0.34	0.00	0.00	1.86	0.00	0.00	null	null		null	null		-8.39E+00	3.16E-01		-8.39E+00	4.05E-01		-1.09E+01	4.62E-04	Down	-1.09E+01	2.81E-04	Down							
Unigene110282_All	290	0.00	0.00	0.00	0.00	0.00	0.00	1.90	0.00	0.27	null	null		null	null		null	null		null	null		-1.09E+01	4.62E-04	Down	-2.79E+00	7.96E-03		Unigene110282_All//gi|15234663|ref|NP_192431.1|//6.00E-13//harpin-induced protein-related / HIN1-related / harpin-responsive protein-related [Arabidopsis thaliana]	Unigene110282_All//2.00E-14//AT4G05220| harpin-induced protein-related / HIN1-related / harpin-responsive protein-related					nr -
Unigene110284_All	509	0.00	0.00	0.00	0.00	0.00	0.00	2.82	1.07	0.16	null	null		null	null		null	null		null	null		-1.40E+00	6.15E-03		-4.17E+00	1.93E-08	Down	Unigene110284_All//gi|226505998|ref|NP_001146929.1|//1.00E-30//60S ribosomal protein L7a [Zea mays] >gi|195605318|gb|ACG24489.1| 60S ribosomal protein L7a [Zea mays]	Unigene110284_All//3.00E-26//AT2G47610| 60S ribosomal protein L7A (RPL7aA)			ko03010|Ribosome		nr -
Unigene110286_All	448	0.23	0.00	0.12	0.00	1.00	0.40	4.06	0.19	0.36	-7.87E+00	3.76E-01		-9.74E-01	7.22E-01		9.96E+00	8.02E-03		8.66E+00	1.95E-01		-4.44E+00	1.72E-10	Down	-3.51E+00	2.43E-09	Down							
Unigene110287_All	1443	0.00	0.00	0.00	0.00	0.00	0.00	11.08	0.00	0.47	null	null		null	null		null	null		null	null		-1.34E+01	9.31E-114	Down	-4.56E+00	2.00E-93	Down							
Unigene110289_All	365	0.00	0.00	0.00	0.00	0.00	0.00	2.09	0.23	0.00	null	null		null	null		null	null		null	null		-3.19E+00	7.93E-04	Down	-1.10E+01	8.88E-06	Down	Unigene110289_All//gi|205364757|gb|ACI04578.1|//2.00E-20//cysteine protease-like protein [Robinia pseudoacacia]	Unigene110289_All//2.00E-20//AT4G16190| cysteine proteinase, putative					nr -
Unigene11029_All	726	5.98	18.63	5.13	12.63	13.38	14.43	6.76	8.52	15.20	1.64E+00	1.96E-21	Up	-2.20E-01	4.94E-01		8.33E-02	7.30E-01		1.93E-01	3.48E-01		3.35E-01	1.33E-01		1.17E+00	1.38E-13	Up	Unigene11029_All//gi|186510768|ref|NP_190388.2|//1.00E-22//DNA binding / protein binding / transcription regulator [Arabidopsis thaliana] >gi|186510770|ref|NP_850669.2| DNA binding / protein binding / transcription regulator [Arabidopsis thaliana]	Unigene11029_All//1.00E-17//AT3G48050| DNA binding / protein binding / transcription regulator	Unigene11029_All//1.00E-36//AW187392			GO:0003676//GO:0006351//GO:0030528	nr +
Unigene110292_All	425	0.00	0.00	0.00	0.00	0.00	0.21	0.50	3.64	1.03	null	null		null	null		null	null		7.73E+00	4.80E-01		2.87E+00	1.64E-06	Up	1.05E+00	2.98E-01		Unigene110292_All//gi|13940209|emb|CAC37922.1|//3.00E-27//fructan 1-exohydrolase IIa [Cichorium intybus] >gi|32492892|gb|AAP85536.1| fructan 1-exohydrolase IIa [Cichorium intybus]	Unigene110292_All//4.00E-26//AT5G11920| AtcwINV6 (6-&1-fructan exohydrolase); hydrolase, hydrolyzing O-glycosyl compounds / inulinase/ levanase	Unigene110292_All//6.00E-59//TC152854		ko00500|Starch and sucrose metabolism//ko00052|Galactose metabolism		nr +
Unigene11030_All	1205	1.61	3.53	3.18	5.08	5.12	8.66	4.74	3.71	15.31	1.14E+00	1.62E-04	Up	9.86E-01	2.63E-03		8.60E-03	9.80E-01		7.68E-01	9.76E-06		-3.52E-01	1.33E-01		1.69E+00	6.59E-38	Up	Unigene11030_All//gi|186510768|ref|NP_190388.2|//8.00E-82//DNA binding / protein binding / transcription regulator [Arabidopsis thaliana] >gi|186510770|ref|NP_850669.2| DNA binding / protein binding / transcription regulator [Arabidopsis thaliana]	Unigene11030_All//2.00E-80//AT3G48050| DNA binding / protein binding / transcription regulator	Unigene11030_All//5.00E-25//AW187169			GO:0006351//GO:0008135//GO:0030528//GO:0043231	nr -
Unigene110300_All	1037	0.20	0.00	0.10	0.29	0.10	0.30	2.04	0.04	0.54	-7.66E+00	1.23E-01		-9.75E-01	6.21E-01		-1.53E+00	2.98E-01		8.13E-02	9.50E-01		-5.66E+00	1.47E-13	Down	-1.92E+00	5.97E-06	Down	Unigene110300_All//gi|115451409|ref|NP_001049305.1|//1.00E-09//Os03g0203100 [Oryza sativa Japonica Group] >gi|108706728|gb|ABF94523.1| expressed protein [Oryza sativa (japonica cultivar-group)] >gi|113547776|dbj|BAF11219.1| Os03g0203100 [Oryza sativa Japonica Group]	Unigene110300_All//4.00E-35//AT4G30150| EXPRESSED IN: 13 plant structures; EXPRESSED DURING: 6 growth stages; CONTAINS InterPro DOMAIN/s: Nucleolar 27S pre-rRNA processing, Urb2/Npa2 (InterPro:IPR018849); Has 31 Blast hits to 27 proteins in 14 species: Archae - 0; Bacteria - 2; Metazoa - 3; Fungi - 0; Plants - 19; Viruses - 3; Other Eukaryotes - 4 (source: NCBI BLink).					nr +
Unigene110302_All	267	0.39	0.00	0.00	0.93	0.00	1.01	2.38	0.00	0.75	-8.61E+00	3.76E-01		-8.61E+00	3.98E-01		-9.87E+00	4.19E-02		1.22E-01	9.44E-01		-1.12E+01	1.23E-04	Down	-1.67E+00	4.29E-02		Unigene110302_All//gi|115455297|ref|NP_001051249.1|//3.00E-22//Os03g0746400 [Oryza sativa Japonica Group] >gi|113549720|dbj|BAF13163.1| Os03g0746400 [Oryza sativa Japonica Group]	Unigene110302_All//2.00E-23//AT3G22670| pentatricopeptide (PPR) repeat-containing protein	Unigene110302_All//4.00E-145//TC174147				nr +
Unigene110307_All	267	0.20	0.00	0.00	0.00	0.00	0.00	2.38	0.00	0.15	-7.61E+00	6.18E-01		-7.61E+00	6.31E-01		null	null		null	null		-1.12E+01	1.23E-04	Down	-3.99E+00	5.82E-04	Down							
Unigene110308_All	319	0.00	0.00	0.00	0.00	0.00	0.00	1.72	0.00	0.00	null	null		null	null		null	null		null	null		-1.08E+01	4.62E-04	Down	-1.08E+01	2.81E-04	Down							
Unigene110313_All	276	0.00	0.00	0.00	0.18	0.00	0.00	4.44	0.30	1.15	null	null		null	null		-7.50E+00	5.62E-01		-7.50E+00	6.43E-01		-3.87E+00	1.15E-06	Down	-1.94E+00	7.80E-04	Down							
Unigene110315_All	360	465.51	192.26	505.14	165.36	225.55	318.53	598.26	282.01	292.37	-1.28E+00	2.16E-178	Down	1.18E-01	4.01E-03		4.48E-01	2.64E-17		9.46E-01	6.13E-81		-1.09E+00	1.60E-216	Down	-1.03E+00	2.55E-205	Down	Unigene110315_All//gi|292653525|gb|ADE34280.1|//2.00E-23//aquaporin PIP1;11 [Gossypium hirsutum]	Unigene110315_All//3.00E-23//AT2G45960| PIP1B (NAMED PLASMA MEMBRANE INTRINSIC PROTEIN 1B); water channel	Unigene110315_All//2.00E-138//TC165394				nr +
Unigene110317_All	294	0.00	0.00	0.00	0.00	0.00	0.00	1.87	0.00	0.27	null	null		null	null		null	null		null	null		-1.09E+01	4.62E-04	Down	-2.79E+00	7.96E-03								
Unigene110322_All	559	0.00	0.00	0.00	0.00	0.00	0.00	1.66	0.15	0.00	null	null		null	null		null	null		null	null		-3.48E+00	7.79E-05	Down	-1.07E+01	5.48E-07	Down							ESTscan -

Unigene110327_All	505	0.73	0.20	2.00	0.20	0.48	0.00	4.35	0.91	0.55	-1.85E+00	1.87E-01		1.47E+00	4.95E-02		1.27E+00	5.32E-01		-7.62E-01	4.05E-01		-2.26E+00	4.47E-07	Down	-2.98E+00	1.05E-09	Down	Unigene110327_All//gi|224142499|ref|XP_002324594.1|//1.00E-06//predicted protein [Populus trichocarpa] >gi|222866028|gb|EEF03159.1| predicted protein [Populus trichocarpa]		Unigene110327_All//1.00E-17//TC175191				nr +
Unigene110335_All	261	0.00	0.00	0.20	0.19	0.00	0.35	2.43	0.48	0.00	null	null		7.67E+00	6.40E-01		-7.58E+00	5.62E-01		8.58E-01	7.96E-01		-2.34E+00	1.27E-02		-1.12E+01	7.12E-05	Down			Unigene110335_All//4.00E-22//gi|164357674|gb|ES820724.1|ES820724				
Unigene110338_All	269	0.00	0.00	0.00	0.00	0.00	0.00	4.56	0.78	0.30	null	null		null	null		null	null		null	null		-2.55E+00	8.71E-05	Down	-3.94E+00	4.86E-07	Down			Unigene110338_All//2.00E-08//gi|109850443|gb|DW479422.1|DW479422				
Unigene110341_All	274	0.00	0.00	0.00	0.00	0.00	0.00	2.78	0.00	0.00	null	null		null	null		null	null		null	null		-1.14E+01	1.68E-05	Down	-1.14E+01	8.88E-06	Down							
Unigene110342_All	266	0.59	0.57	0.80	0.56	0.26	0.00	3.34	2.04	0.45	-4.62E-02	9.90E-01		4.41E-01	7.76E-01		-1.12E+00	5.85E-01		-9.13E+00	2.34E-01		-7.09E-01	2.97E-01		-2.89E+00	3.05E-04	Down							
Unigene110343_All	578	0.00	0.00	0.00	0.00	0.00	0.00	2.56	2.82	0.00	null	null		null	null		null	null		null	null		1.40E-01	7.67E-01		-1.13E+01	5.85E-11	Down	Unigene110343_All//gi|302128114|dbj|BAJ13478.1|//1.00E-59//actin 1 [Guillardia theta]	Unigene110343_All//1.00E-58//AT3G12110| ACT11 (actin-11); structural constituent of cytoskeleton	Unigene110343_All//7.00E-13//TC153744				nr +
Unigene110344_All	457	0.00	0.00	0.00	0.00	0.00	0.00	2.04	0.00	0.17	null	null		null	null		null	null		null	null		-1.10E+01	1.15E-06	Down	-3.55E+00	3.97E-05	Down							
Unigene110352_All	407	0.39	0.00	0.00	0.12	0.51	0.00	1.45	0.00	0.10	-8.59E+00	2.17E-01		-8.59E+00	2.46E-01		2.05E+00	3.95E-01		-6.94E+00	6.43E-01		-1.05E+01	2.40E-04	Down	-3.89E+00	1.10E-03		Unigene110352_All//gi|226530818|ref|NP_001148776.1|//1.00E-18//cysteine-type peptidase [Zea mays] >gi|195622072|gb|ACG32866.1| cysteine-type peptidase [Zea mays]	Unigene110352_All//9.00E-18//AT5G04250| OTU-like cysteine protease family protein	Unigene110352_All//6.00E-136//TC174360				nr -
Unigene110353_All	687	0.00	0.00	0.00	0.00	0.00	0.00	4.31	0.12	0.23	null	null		null	null		null	null		null	null		-5.15E+00	3.87E-18	Down	-4.21E+00	6.93E-17	Down	Unigene110353_All//gi|297811659|ref|XP_002873713.1|//3.00E-63//40S ribosomal protein S9 [Arabidopsis lyrata subsp. lyrata] >gi|297319550|gb|EFH49972.1| 40S ribosomal protein S9 [Arabidopsis lyrata subsp. lyrata]	Unigene110353_All//5.00E-50//AT5G15200| 40S ribosomal protein S9 (RPS9B)			ko03010|Ribosome		nr +
Unigene110356_All	351	0.00	0.00	0.00	1.70	0.78	1.03	3.98	0.60	0.34	null	null		null	null		-1.12E+00	2.26E-01		-7.26E-01	4.60E-01		-2.74E+00	1.03E-05	Down	-3.55E+00	2.28E-07	Down	Unigene110356_All//gi|297596229|ref|NP_001042217.2|//3.00E-08//Os01g0182300 [Oryza sativa Japonica Group] >gi|255672942|dbj|BAF04131.2| Os01g0182300 [Oryza sativa Japonica Group]						nr -
Unigene110357_All	225	0.00	0.00	0.24	1.55	0.46	0.40	2.25	0.19	0.00	null	null		7.89E+00	6.40E-01		-1.76E+00	1.90E-01		-1.95E+00	1.96E-01		-3.60E+00	5.78E-03		-1.11E+01	5.55E-04	Down			Unigene110357_All//5.00E-21//TC176559				
Unigene11036_All	1023	3.58	1.68	2.71	8.62	10.20	10.90	10.25	2.94	6.35	-1.09E+00	8.27E-04	Down	-4.04E-01	2.42E-01		2.43E-01	2.08E-01		3.39E-01	5.60E-02		-1.80E+00	2.43E-23	Down	-6.91E-01	6.51E-06		Unigene11036_All//gi|225438627|ref|XP_002276798.1|//1.00E-161//PREDICTED: similar to binding [Vitis vinifera]	Unigene11036_All//3.00E-147//AT2G22125| binding	Unigene11036_All//0.00E+00//TC133396				nr -
Unigene110360_All	343	0.00	0.15	0.16	1.74	0.60	0.00	1.23	0.00	0.93	7.21E+00	6.21E-01		7.28E+00	6.40E-01		-1.53E+00	1.01E-01		-1.08E+01	7.71E-04	Down	-1.03E+01	3.26E-03		-4.08E-01	6.54E-01		Unigene110360_All//gi|57834114|emb|CAE05707.2|//2.00E-24//OSJNBb0065J09.3 [Oryza sativa (japonica cultivar-group)]						nr +
Unigene110364_All	243	0.43	0.00	0.00	0.00	0.00	0.00	4.00	0.00	0.33	-8.75E+00	3.76E-01		-8.75E+00	3.98E-01		null	null		null	null		-1.20E+01	5.84E-07	Down	-3.61E+00	2.14E-05	Down	Unigene110364_All//gi|9759032|dbj|BAB09401.1|//1.00E-13//transmembrane transport protein-like [Arabidopsis thaliana]	Unigene110364_All//5.00E-15//AT5G50300| xanthine/uracil/vitamin C permease family protein					nr -
Unigene110366_All	374	0.00	0.00	0.00	0.00	0.00	0.00	2.94	0.89	0.32	null	null		null	null		null	null		null	null		-1.72E+00	5.68E-03		-3.20E+00	1.63E-05	Down							
Unigene11037_All	635	1.32	0.48	1.51	2.12	6.13	3.41	5.93	1.71	2.82	-1.46E+00	6.69E-02		1.95E-01	7.70E-01		1.53E+00	9.42E-07	Up	6.89E-01	1.28E-01		-1.79E+00	8.35E-09	Down	-1.07E+00	1.16E-04	Down	Unigene11037_All//gi|225438627|ref|XP_002276798.1|//3.00E-94//PREDICTED: similar to binding [Vitis vinifera]	Unigene11037_All//7.00E-83//AT2G22125| binding	Unigene11037_All//0.00E+00//TC133396				nr -
Unigene110370_All	326	0.32	0.78	3.10	0.61	0.53	0.28	1.82	0.77	0.49	1.28E+00	4.36E-01		3.27E+00	5.93E-04	Up	-2.11E-01	8.59E-01		-1.14E+00	5.70E-01		-1.24E+00	1.54E-01		-1.89E+00	3.22E-02		Unigene110370_All//gi|255541236|ref|XP_002511682.1|//7.00E-07//beta-tubulin cofactor d, putative [Ricinus communis] >gi|223548862|gb|EEF50351.1| beta-tubulin cofactor d, putative [Ricinus communis]	Unigene110370_All//5.00E-05//AT3G60740| TTN1 (TITAN 1); tubulin binding	Unigene110370_All//1.00E-47//gi|73847702|gb|DT456722.1|DT456722				nr +
Unigene110372_All	344	0.00	0.00	0.00	0.00	0.00	0.00	2.21	0.49	0.23	null	null		null	null		null	null		null	null		-2.19E+00	7.78E-03		-3.26E+00	4.48E-04	Down							ESTscan -
Unigene11038_All	608	0.86	0.67	0.70	2.87	2.10	4.23	3.48	0.28	0.46	-3.68E-01	6.83E-01		-2.96E-01	7.50E-01		-4.53E-01	3.73E-01		5.62E-01	1.74E-01		-3.66E+00	1.12E-10	Down	-2.92E+00	3.37E-09	Down	Unigene11038_All//gi|225438627|ref|XP_002276798.1|//2.00E-93//PREDICTED: similar to binding [Vitis vinifera]	Unigene11038_All//1.00E-78//AT2G22125| binding	Unigene11038_All//0.00E+00//TC142058			GO:0043231	nr +
Unigene110380_All	268	0.00	0.00	0.00	0.00	0.00	0.00	3.79	0.00	0.59	null	null		null	null		null	null		null	null		-1.19E+01	2.97E-07	Down	-2.67E+00	1.94E-04	Down							
Unigene110381_All	1570	0.33	0.39	1.22	0.10	0.13	0.43	1.62	1.22	0.53	2.17E-01	7.93E-01		1.87E+00	4.53E-04	Up	4.67E-01	8.26E-01		2.18E+00	3.14E-02		-4.00E-01	2.85E-01		-1.60E+00	1.31E-05	Down							
Unigene110385_All	304	513.40	1507.92	676.13	78.82	157.90	305.89	67.87	173.23	214.26	1.55E+00	0.00E+00	Up	3.97E-01	2.88E-28		1.00E+00	2.57E-41	Up	1.96E+00	7.56E-197	Up	1.35E+00	2.98E-75	Up	1.66E+00	4.06E-128	Up	Unigene110385_All//gi|73761689|gb|AAZ83344.1|//4.00E-31//ACC oxidase 3 [Gossypium hirsutum]	Unigene110385_All//1.00E-24//AT1G05010| EFE (ETHYLENE-FORMING ENZYME); 1-aminocyclopropane-1-carboxylate oxidase	Unigene110385_All//2.00E-159//TC134764		ko00270|Cysteine and methionine metabolism		nr +
Unigene110386_All	760	0.00	0.00	0.00	0.20	0.09	0.06	7.01	0.93	4.19	null	null		null	null		-1.12E+00	5.85E-01		-1.73E+00	5.02E-01		-2.91E+00	3.18E-21	Down	-7.41E-01	9.05E-04		Unigene110386_All//gi|224069338|ref|XP_002302959.1|//1.00E-13//cc-nbs-lrr resistance protein [Populus trichocarpa] >gi|222844685|gb|EEE82232.1| cc-nbs-lrr resistance protein [Populus trichocarpa]	Unigene110386_All//1.00E-06//AT1G69545| leucine-rich repeat family protein	Unigene110386_All//2.00E-85//ES801940				nr -
Unigene11039_All	1073	9.26	3.02	6.15	22.38	21.18	15.78	18.68	6.23	10.40	-1.62E+00	9.99E-16	Down	-5.90E-01	1.76E-03		-7.92E-02	5.67E-01		-5.04E-01	3.16E-06		-1.58E+00	9.88E-37	Down	-8.45E-01	1.78E-14		Unigene11039_All//gi|225438627|ref|XP_002276798.1|//1.00E-171//PREDICTED: similar to binding [Vitis vinifera]	Unigene11039_All//4.00E-150//AT2G22125| binding	Unigene11039_All//0.00E+00//TC133396				nr -
Unigene110393_All	416	0.00	0.00	0.00	0.00	0.00	0.00	2.03	0.00	0.00	null	null		null	null		null	null		null	null		-1.10E+01	4.39E-06	Down	-1.10E+01	2.21E-06	Down							ESTscan -
Unigene110399_All	314	0.00	0.00	0.00	0.00	0.00	0.00	2.29	0.53	0.00	null	null		null	null		null	null		null	null		-2.10E+00	1.25E-02		-1.12E+01	1.78E-05	Down	Unigene110399_All//gi|146454510|gb|ABQ41921.1|//3.00E-33//60S ribosomal protein large subunit 9 [Sonneratia caseolaris] >gi|156633898|gb|ABU90948.1| ribosomal protein L9 [Sonneratia caseolaris] >gi|156633900|gb|ABU90949.1| ribosomal protein L9 [Sonneratia caseolaris] >gi|156633902|gb|ABU90950.1| ribosomal protein L9 [Sonneratia caseolaris] >gi|156633904|gb|ABU90951.1| ribosomal protein L9 [Sonneratia caseolaris] >gi|156633906|gb|ABU90952.1| ribosomal protein L9 [Sonneratia caseolaris] >gi|156633908|gb|ABU90953.1| ribosomal protein L9 [Sonneratia caseolaris] >gi|156633910|gb|ABU90954.1| ribosomal protein L9 [Sonneratia caseolaris] >gi|156633912|gb|ABU90955.1| ribosomal protein L9 [Sonneratia caseolaris] >gi|156633914|gb|ABU90956.1| ribosomal protein L9 [Sonneratia	Unigene110399_All//6.00E-32//AT4G10450| 60S ribosomal protein L9 (RPL90D)			ko03010|Ribosome		nr -
Unigene11040_All	1917	2.10	2.19	1.94	5.61	5.86	6.15	4.39	1.72	2.87	6.19E-02	8.39E-01		-1.12E-01	7.18E-01		6.12E-02	8.03E-01		1.32E-01	5.05E-01		-1.35E+00	1.12E-12	Down	-6.14E-01	3.71E-04		Unigene11040_All//gi|225438627|ref|XP_002276798.1|//0.00E+00//PREDICTED: similar to binding [Vitis vinifera]	Unigene11040_All//0.00E+00//AT2G22125| binding	Unigene11040_All//0.00E+00//TC142058				nr +
Unigene110400_All	898	0.82	2.09	0.83	2.77	1.80	2.06	1.18	0.37	0.27	1.36E+00	5.88E-03		2.54E-02	9.95E-01		-6.22E-01	1.12E-01		-4.28E-01	3.21E-01		-1.66E+00	8.55E-03		-2.14E+00	9.36E-04	Down			Unigene110400_All//3.00E-161//gi|84172254|gb|DR460902.1|DR460902				
Unigene11041_All	5663	6.25	3.44	3.71	11.56	10.34	10.08	15.18	4.94	7.01	-8.64E-01	1.45E-20		-7.52E-01	1.14E-15		-1.61E-01	1.36E-02		-1.97E-01	2.74E-03		-1.62E+00	4.55E-160	Down	-1.12E+00	2.81E-93	Down	Unigene11041_All//gi|225438627|ref|XP_002276798.1|//0.00E+00//PREDICTED: similar to binding [Vitis vinifera]	Unigene11041_All//0.00E+00//AT2G22125| binding	Unigene11041_All//0.00E+00//TC133396				nr -
Unigene110411_All	858	0.00	0.00	0.19	0.00	0.04	0.00	0.79	0.15	0.05	null	null		7.54E+00	2.40E-01		5.33E+00	8.37E-01		null	null		-2.43E+00	7.77E-03		-4.09E+00	3.10E-04	Down	Unigene110411_All//gi|4249380|gb|AAD14477.1|//1.00E-36//ESTs gb|Z37637, gb|AA042498 and gb|AA042269 come from this gene [Arabidopsis thaliana]	Unigene110411_All//7.00E-38//AT1G60010| unknown protein	Unigene110411_All//0.00E+00//TC164121				nr -
Unigene110413_All	627	0.08	1.37	0.59	0.24	1.26	1.15	1.15	0.53	0.95	4.04E+00	4.20E-04	Up	2.83E+00	9.31E-02		2.41E+00	9.58E-03		2.27E+00	2.08E-02		-1.10E+00	1.49E-01		-2.66E-01	6.93E-01				Unigene110413_All//7.00E-07//DN803899				
Unigene110414_All	598	0.00	0.00	0.00	0.00	0.00	0.00	3.82	5.94	0.13	null	null		null	null		null	null		null	null		6.38E-01	2.95E-02		-4.84E+00	3.22E-14	Down	Unigene110414_All//gi|238014838|gb|ACR38454.1|//2.00E-13//unknown [Zea mays]		Unigene110414_All//4.00E-27//ES850594				nr -
Unigene110418_All	301	0.00	0.00	0.00	0.00	0.00	0.00	1.83	0.00	0.40	null	null		null	null		null	null		null	null		-1.08E+01	4.62E-04	Down	-2.20E+00	2.24E-02								
Unigene11042_All	6962	6.76	4.24	4.16	15.66	14.53	12.62	17.51	5.15	8.70	-6.75E-01	6.42E-18		-7.01E-01	2.56E-18		-1.08E-01	3.72E-02		-3.11E-01	5.86E-11		-1.77E+00	1.50E-256	Down	-1.01E+00	5.13E-112	Down	Unigene11042_All//gi|225438627|ref|XP_002276798.1|//0.00E+00//PREDICTED: similar to binding [Vitis vinifera]	Unigene11042_All//0.00E+00//AT2G22125| binding	Unigene11042_All//0.00E+00//TC133396			GO:0003824	nr -
Unigene110421_All	263	0.00	0.00	0.00	0.00	0.00	0.00	3.38	0.64	0.30	null	null		null	null		null	null		null	null		-2.41E+00	1.79E-03		-3.48E+00	7.34E-05	Down	Unigene110421_All//gi|255071745|ref|XP_002499547.1|//8.00E-29//ribosomal protein L37e [Micromonas sp. RCC299] >gi|226514809|gb|ACO60805.1| ribosomal protein L37e [Micromonas sp. RCC299]	Unigene110421_All//2.00E-28//AT1G52300| 60S ribosomal protein L37 (RPL37B)			ko03010|Ribosome		nr -
Unigene110427_All	413	0.51	0.00	0.26	0.24	0.33	0.44	1.54	0.20	0.00	-8.99E+00	1.23E-01		-9.74E-01	6.21E-01		4.68E-01	8.78E-01		8.59E-01	6.96E-01		-2.92E+00	4.20E-03		-1.06E+01	7.12E-05	Down							
Unigene110428_All	340	0.00	0.00	0.00	0.29	0.00	0.00	3.36	0.49	0.12	null	null		null	null		-8.19E+00	3.16E-01		-8.19E+00	4.04E-01		-2.77E+00	7.62E-05	Down	-4.84E+00	2.34E-07	Down	Unigene110428_All//gi|75100616|sp|O82318.1|Y2579_ARATH//2.00E-13//RecName: Full=Probably inactive leucine-rich repeat receptor-like protein kinase At2g25790; Flags: Precursor >gi|224589523|gb|ACN59295.1| leucine-rich repeat receptor-like protein kinase [Arabidopsis thaliana]	Unigene110428_All//7.00E-12//AT2G25790| leucine-rich repeat transmembrane protein kinase, putative	Unigene110428_All//7.00E-92//TC179552		ko04626|Plant-pathogen interaction		nr +
Unigene110437_All	310	0.00	0.00	0.00	0.00	0.00	0.00	2.05	0.13	0.13	null	null		null	null		null	null		null	null		-3.92E+00	9.81E-04	Down	-3.99E+00	5.82E-04	Down							
Unigene110439_All	592	0.00	0.00	0.00	0.00	0.00	0.00	3.43	1.84	0.34	null	null		null	null		null	null		null	null		-9.01E-01	2.61E-02		-3.35E+00	5.75E-10	Down							
Unigene110440_All	473	0.00	0.00	0.00	0.21	0.07	0.00	3.49	0.00	0.25	null	null		null	null		-1.53E+00	5.88E-01		-7.72E+00	4.04E-01		-1.18E+01	1.09E-11	Down	-3.79E+00	5.22E-09	Down	Unigene110440_All//gi|240255605|ref|NP_001030839.5|//2.00E-39//ETO1 (ETHYLENE OVERPRODUCER 1); protein binding, bridging [Arabidopsis thaliana]	Unigene110440_All//2.00E-40//AT3G51770| ETO1 (ETHYLENE OVERPRODUCER 1); protein binding, bridging					nr -
Unigene110441_All	407	30.34	8.59	12.82	40.88	19.12	10.43	10.39	7.70	4.60	-1.82E+00	9.37E-23	Down	-1.24E+00	1.11E-12	Down	-1.10E+00	1.51E-17	Down	-1.97E+00	1.37E-36	Down	-4.32E-01	1.13E-01		-1.18E+00	7.98E-06	Down			Unigene110441_All//3.00E-70//TC171513				
Unigene110443_All	310	0.34	0.00	0.86	0.80	0.22	0.29	1.64	0.81	0.00	-8.40E+00	3.76E-01		1.35E+00	4.09E-01		-1.85E+00	2.76E-01		-1.46E+00	4.07E-01		-1.02E+00	2.88E-01		-1.07E+01	5.55E-04	Down							

Unigene110444_All	380	0.14	0.00	0.42	3.80	2.54	1.55	9.35	4.18	1.05	-7.11E+00	6.18E-01		1.61E+00	5.12E-01		-5.83E-01	2.98E-01		-1.30E+00	2.16E-02		-1.16E+00	9.24E-05	Down	-3.16E+00	4.42E-16	Down	Unigene110444_All//gi|4733946|gb|AAD28641.1|//6.00E-17//sucrose synthase [Gossypium hirsutum]	Unigene110444_All//1.00E-14//AT4G02280| SUS3 (sucrose synthase 3); UDP-glycosyltransferase/ sucrose synthase/ transferase, transferring glycosyl groups	Unigene110444_All//3.00E-54//TC150792		ko00500|Starch and sucrose metabolism		nr -
Unigene110445_All	568	0.00	0.00	0.00	0.09	0.06	0.00	4.09	0.88	0.56	null	null		null	null		-5.33E-01	8.28E-01		-6.45E+00	6.43E-01		-2.21E+00	2.77E-07	Down	-2.87E+00	7.21E-10	Down			Unigene110445_All//2.00E-145//TC132634				
Unigene110447_All	459	0.00	0.00	0.00	0.00	0.00	0.00	1.66	0.00	0.09	null	null		null	null		null	null		null	null		-1.07E+01	1.68E-05	Down	-4.26E+00	8.66E-05	Down	Unigene110447_All//gi|300265162|gb|EFJ49355.1|//4.00E-29//component of cytosolic 80S ribosome and 60S large subunit [Volvox carteri f. nagariensis]	Unigene110447_All//3.00E-27//AT3G25520| ATL5 (A. THALIANA RIBOSOMAL PROTEIN L5); 5S rRNA binding / structural constituent of ribosome			ko03010|Ribosome		nr +
Unigene110448_All	310	1.01	0.98	0.34	0.16	0.56	0.29	2.86	0.40	0.64	-4.63E-02	9.85E-01		-1.56E+00	3.30E-01		1.79E+00	5.21E-01		8.59E-01	7.96E-01		-2.82E+00	5.65E-04	Down	-2.16E+00	2.68E-03		Unigene110448_All//gi|12322008|gb|AAG51046.1|AC069473_8//1.00E-21//gypsy/Ty-3 retroelement polyprotein; 69905-74404 [Arabidopsis thaliana] >gi|10998138|dbj|BAB03109.1| retroelement pol polyprotein [Arabidopsis thaliana]						nr -
Unigene110449_All	324	0.00	0.00	0.00	0.00	0.21	0.00	2.87	9.55	6.89	null	null		null	null		7.73E+00	6.14E-01		null	null		1.73E+00	4.69E-07	Up	1.26E+00	9.46E-04	Up		Unigene110449_All//2.00E-05//AT5G18170| GDH1 (GLUTAMATE DEHYDROGENASE 1); ATP binding / glutamate dehydrogenase [NAD(P)+]/ oxidoreductase	Unigene110449_All//8.00E-64//ES791234		ko00910|Nitrogen metabolism//ko00250|Alanine, aspartate and glutamate metabolism//ko00330|Arginine and proline metabolism		kegg -
Unigene11045_All	547	0.67	1.76	0.97	0.18	0.82	2.15	1.47	1.15	1.68	1.39E+00	5.77E-02		5.40E-01	6.10E-01		2.17E+00	1.06E-01		3.56E+00	4.10E-05	Up	-3.58E-01	6.49E-01		1.90E-01	7.54E-01		Unigene11045_All//gi|30679594|ref|NP_195851.2|//6.00E-74//PRT6 (PROTEOLYSIS 6); ubiquitin-protein ligase [Arabidopsis thaliana]	Unigene11045_All//3.00E-75//AT5G02310| PRT6 (PROTEOLYSIS 6); ubiquitin-protein ligase				GO:0019787//GO:0046914	nr -
Unigene110450_All	282	0.00	0.00	0.00	0.00	0.00	0.00	2.25	0.15	0.42	null	null		null	null		null	null		null	null		-3.92E+00	9.81E-04	Down	-2.41E+00	8.12E-03		Unigene110450_All//sp|P26262|KLKB1_MOUSE//5.00E-06//Plasma kallikrein OS=Mus musculus GN=Klkb1 PE=1 SV=1						swissprot -
Unigene110451_All	576	0.00	0.00	0.00	0.17	0.24	0.16	6.61	10.67	24.42	null	null		null	null		4.67E-01	8.78E-01		-1.42E-01	9.52E-01		6.91E-01	1.12E-03		1.89E+00	1.20E-33	Up	Unigene110451_All//gi|115483761|ref|NP_001065542.1|//6.00E-38//Os11g0106900 [Oryza sativa Japonica Group] >gi|115486902|ref|NP_001065938.1| Os12g0106200 [Oryza sativa Japonica Group] >gi|113644246|dbj|BAF27387.1| Os11g0106900 [Oryza sativa Japonica Group] >gi|113648445|dbj|BAF28957.1| Os12g0106200 [Oryza sativa Japonica Group]	Unigene110451_All//4.00E-31//AT1G07900| LBD1 (LOB DOMAIN-CONTAINING PROTEIN 1)	Unigene110451_All//0.00E+00//TC178840		ko00240|Pyrimidine metabolism//ko00250|Alanine, aspartate and glutamate metabolism		nr +
Unigene110458_All	774	0.00	0.00	0.14	0.13	0.09	0.41	5.79	0.38	2.11	null	null		7.10E+00	4.02E-01		-5.32E-01	8.19E-01		1.67E+00	3.09E-01		-3.94E+00	2.86E-23	Down	-1.46E+00	3.57E-08	Down	Unigene110458_All//gi|57222232|gb|AAW39023.1|//2.00E-78//At3g27230 [Arabidopsis thaliana] >gi|63003758|gb|AAY25408.1| At3g27230 [Arabidopsis thaliana]	Unigene110458_All//4.00E-82//AT5G40830| methyltransferase	Unigene110458_All//3.00E-56//gi|48744105|gb|CO074624.1|CO074624				nr -
Unigene110459_All	490	0.00	0.00	0.00	0.00	0.00	0.00	15.19	1.11	0.41	null	null		null	null		null	null		null	null		-3.78E+00	4.05E-37	Down	-5.22E+00	4.72E-47	Down	Unigene110459_All//gi|5080804|gb|AAD39313.1|AC007258_2//3.00E-29//Hypothetical protein [Arabidopsis thaliana] >gi|15010642|gb|AAK73980.1| At1g59710/T30E16_31 [Arabidopsis thaliana] >gi|22137290|gb|AAM91490.1| At1g59710/T30E16_31 [Arabidopsis thaliana]	Unigene110459_All//2.00E-30//AT1G59710| FUNCTIONS IN: molecular_function unknown; INVOLVED IN: biological_process unknown; LOCATED IN: cellular_component unknown; EXPRESSED IN: 23 plant structures; EXPRESSED DURING: 13 growth stages; CONTAINS InterPro DOMAIN/s: Protein of unknown function DUF569 (InterPro:IPR007679), Actin_cross-linking (InterPro:IPR008999); BEST Arabidopsis thaliana protein match is: unknown protein (TAIR:AT1G27100.1); Has 119 Blast hits to 107 proteins in 9 species: Archae - 0; Bacteria - 0; Metazoa - 0; Fungi - 0; Plants - 119; Viruses - 0; Other Eukaryotes - 0 (source: NCBI BLink).	Unigene110459_All//2.00E-59//TC162219				nr +
Unigene110461_All	241	7.82	10.72	15.02	15.50	3.57	13.87	1.23	1.39	0.66	4.56E-01	2.50E-01		9.43E-01	5.28E-03		-2.12E+00	1.11E-10	Down	-1.61E-01	6.52E-01		1.76E-01	8.81E-01		-8.93E-01	4.71E-01				Unigene110461_All//7.00E-42//gi|78341252|gb|DT561526.1|DT561526				
Unigene110465_All	613	0.00	0.00	0.00	0.00	0.00	0.00	3.10	0.48	0.00	null	null		null	null		null	null		null	null		-2.70E+00	1.87E-07	Down	-1.16E+01	4.91E-14	Down							ESTscan +
Unigene110470_All	539	0.00	0.00	0.00	0.18	0.00	0.00	3.22	0.16	0.30	null	null		null	null		-7.53E+00	3.16E-01		-7.53E+00	4.04E-01		-4.37E+00	6.16E-10	Down	-3.44E+00	8.42E-09	Down							
Unigene110474_All	270	0.00	0.00	0.00	0.00	0.00	0.00	2.35	0.46	0.00	null	null		null	null		null	null		null	null		-2.34E+00	1.27E-02		-1.12E+01	7.12E-05	Down	Unigene110474_All//gi|81237625|gb|AAX77224.2|//3.00E-17//14-3-3 protein [Dunaliella salina] >gi|157062258|gb|ABV03825.1| 14-3-3 protein [Dunaliella salina]	Unigene110474_All//1.00E-15//AT5G38480| GRF3 (GENERAL REGULATORY FACTOR 3); ATP binding / protein binding / protein phosphorylated amino acid binding	Unigene110474_All//9.00E-20//DV849765				nr -
Unigene110478_All	495	0.00	0.00	0.00	0.00	0.21	0.00	4.27	1.60	0.08	null	null		null	null		7.71E+00	4.58E-01		null	null		-1.41E+00	5.54E-04	Down	-5.73E+00	3.61E-14	Down	Unigene110478_All//gi|224365602|gb|ACN41354.1|//6.00E-30//LR34 [Triticum aestivum]	Unigene110478_All//2.00E-30//AT2G37280| PDR5 (PLEIOTROPIC DRUG RESISTANCE 5); ATPase, coupled to transmembrane movement of substances					nr +
Unigene110483_All	330	0.00	0.00	0.00	0.00	0.00	0.00	2.18	0.13	0.00	null	null		null	null		null	null		null	null		-4.10E+00	2.91E-04	Down	-1.11E+01	1.78E-05	Down	Unigene110483_All//gi|195640868|gb|ACG39902.1|//6.00E-08//36.4 kDa proline-rich protein [Zea mays]				ko04626|Plant-pathogen interaction//ko03040|Spliceosome		nr +
Unigene110484_All	617	0.00	0.00	0.00	0.00	0.00	0.00	3.84	0.41	0.13	null	null		null	null		null	null		null	null		-3.24E+00	8.82E-11	Down	-4.89E+00	8.31E-15	Down	Unigene110484_All//gi|299471658|emb|CBN76880.1|//1.00E-27//chp-1 / RAR1 homologue [Ectocarpus siliculosus]	Unigene110484_All//3.00E-13//AT5G51700| PBS2 (PPHB SUSCEPTIBLE 2); protein binding / zinc ion binding			ko04626|Plant-pathogen interaction		nr -
Unigene110487_All	250	6.91	15.00	8.95	32.88	27.00	30.71	4.40	3.51	4.14	1.12E+00	5.36E-04	Up	3.73E-01	3.96E-01		-2.84E-01	1.77E-01		-9.83E-02	6.90E-01		-3.25E-01	6.15E-01		-8.58E-02	8.84E-01				Unigene110487_All//4.00E-43//TC161899				
Unigene11049_All	4497	0.15	0.18	0.31	0.22	0.25	0.15	0.75	1.68	1.02	2.54E-01	7.26E-01		1.03E+00	8.63E-02		1.46E-01	8.37E-01		-5.57E-01	4.47E-01		1.16E+00	3.12E-09	Up	4.38E-01	7.78E-02		Unigene11049_All//gi|30679594|ref|NP_195851.2|//0.00E+00//PRT6 (PROTEOLYSIS 6); ubiquitin-protein ligase [Arabidopsis thaliana]	Unigene11049_All//0.00E+00//AT5G02310| PRT6 (PROTEOLYSIS 6); ubiquitin-protein ligase	Unigene11049_All//0.00E+00//TC138549			GO:0003824	nr -
Unigene110496_All	295	0.00	0.00	0.00	0.00	0.00	0.00	1.72	0.00	0.00	null	null		null	null		null	null		null	null		-1.07E+01	8.90E-04	Down	-1.07E+01	5.55E-04	Down	Unigene110496_All//gi|226347409|gb|ACO50115.1|//2.00E-42//elongation factor 1 alpha [Peranema trichophorum]	Unigene110496_All//4.00E-32//AT5G60390| elongation factor 1-alpha / EF-1-alpha	Unigene110496_All//6.00E-18//TC129595				nr -
Unigene110501_All	307	0.34	0.50	0.17	0.32	0.00	0.00	7.99	4.36	0.91	5.38E-01	7.80E-01		-9.74E-01	7.22E-01		-8.34E+00	3.16E-01		-8.34E+00	4.04E-01		-8.75E-01	1.57E-02		-3.14E+00	2.96E-11	Down			Unigene110501_All//7.00E-21//TC152267				
Unigene110505_All	410	0.00	0.00	0.00	0.00	0.00	0.00	2.47	3.06	0.00	null	null		null	null		null	null		null	null		3.05E-01	6.11E-01		-1.13E+01	1.35E-07	Down	Unigene110505_All//gi|20140684|sp|Q944W6.1|TCTP_BRAOL//6.00E-13//RecName: Full=Translationally-controlled tumor protein homolog; Short=TCTP >gi|16033628|gb|AAL13303.1|AF418663_1 translationally controlled tumor protein [Brassica oleracea]	Unigene110505_All//1.00E-11//AT3G16640| TCTP (TRANSLATIONALLY CONTROLLED TUMOR PROTEIN)					nr -
Unigene11051_All	1128	1.67	3.59	3.92	3.71	4.15	5.53	8.32	13.04	23.42	1.11E+00	3.41E-04	Up	1.23E+00	6.31E-05	Up	1.63E-01	6.07E-01		5.75E-01	1.56E-02		6.48E-01	6.26E-07		1.49E+00	4.13E-45	Up	Unigene11051_All//gi|190148357|gb|ACE63261.1|//0.00E+00//histidine kinase 3 [Betula pendula]	Unigene11051_All//2.00E-169//AT1G27320| AHK3 (ARABIDOPSIS HISTIDINE KINASE 3); cytokinin receptor/ osmosensor/ protein histidine kinase	Unigene11051_All//0.00E+00//TC179474	Orphans		GO:0004673//GO:0004871//GO:0006351//GO:0006468//GO:0023060//GO:0032559//GO:0044464	nr -
Unigene110515_All	765	0.00	0.00	0.00	0.00	0.00	0.00	0.88	0.66	2.40	null	null		null	null		null	null		null	null		-4.32E-01	6.12E-01		1.44E+00	9.58E-04	Up	Unigene110515_All//gi|300157379|gb|EFJ24005.1|//2.00E-62//ubiquitin-protein ligase, PUB17 [Selaginella moellendorffii]	Unigene110515_All//5.00E-48//AT3G54850| PUB14; ubiquitin-protein ligase			ko04141|Protein processing in endoplasmic reticulum//ko04120|Ubiquitin mediated proteolysis		nr -
Unigene110516_All	625	0.00	0.00	0.00	0.00	0.00	0.00	2.23	0.40	0.57	null	null		null	null		null	null		null	null		-2.48E+00	3.36E-05	Down	-1.96E+00	2.74E-04	Down	Unigene110516_All//gi|223452532|gb|ACM89593.1|//5.00E-86//leucine-rich repeat transmembrane protein kinase [Glycine max]	Unigene110516_All//7.00E-81//AT1G34110| leucine-rich repeat transmembrane protein kinase, putative			ko04626|Plant-pathogen interaction		nr +
Unigene11052_All	3040	6.06	10.78	10.44	10.67	13.99	16.61	18.26	29.52	38.27	8.32E-01	7.03E-18		7.85E-01	3.54E-15		3.91E-01	2.39E-07		6.39E-01	1.61E-18		6.93E-01	1.08E-42		1.07E+00	1.74E-116	Up	Unigene11052_All//gi|190148357|gb|ACE63261.1|//0.00E+00//histidine kinase 3 [Betula pendula]	Unigene11052_All//0.00E+00//AT1G27320| AHK3 (ARABIDOPSIS HISTIDINE KINASE 3); cytokinin receptor/ osmosensor/ protein histidine kinase	Unigene11052_All//0.00E+00//TC179474	Orphans	ko04712|Circadian rhythm - plant	GO:0004673//GO:0004888//GO:0006351//GO:0006468//GO:0006826//GO:0006952//GO:0006970//GO:0009267//GO:0009409//GO:0009737//GO:0009744//GO:0009755//GO:0009845//GO:0009888//GO:0009908//GO:0010260//GO:0015996//GO:0016020//GO:0016791//GO:0019901//GO:0023060//GO:0032559//GO:0040007//GO:2000026	nr +
Unigene110524_All	493	0.00	0.00	0.00	0.00	0.00	0.00	5.15	0.17	0.00	null	null		null	null		null	null		null	null		-4.92E+00	2.81E-15	Down	-1.23E+01	1.14E-18	Down	Unigene110524_All//gi|297824403|ref|XP_002880084.1|//9.00E-59//60S ribosomal protein L7 [Arabidopsis lyrata subsp. lyrata] >gi|297325923|gb|EFH56343.1| 60S ribosomal protein L7 [Arabidopsis lyrata subsp. lyrata]	Unigene110524_All//5.00E-60//AT2G44120| 60S ribosomal protein L7 (RPL7C)	Unigene110524_All//4.00E-08//TC131081		ko03010|Ribosome		nr +
Unigene110529_All	522	0.00	0.00	0.00	0.00	0.00	0.00	2.27	0.32	0.00	null	null		null	null		null	null		null	null		-2.82E+00	4.41E-05	Down	-1.11E+01	8.15E-09	Down	Unigene110529_All//gi|159462862|ref|XP_001689661.1|//2.00E-60//calreticulin 2, calcium-binding protein [Chlamydomonas reinhardtii] >gi|11131843|sp|Q9STD3.1|CALR_CHLRE RecName: Full=Calreticulin; Flags: Precursor >gi|5830186|emb|CAB54526.1| calreticulin [Chlamydomonas reinhardtii] >gi|158283649|gb|EDP09399.1| calreticulin 2, calcium-binding protein [Chlamydomonas reinhardtii]	Unigene110529_All//6.00E-43//AT1G09210| calreticulin 2 (CRT2)			ko04141|Protein processing in endoplasmic reticulum//ko04145|Phagosome		nr -
Unigene11053_All	4147	4.24	20.22	13.02	5.57	12.81	14.22	4.83	12.00	18.52	2.25E+00	5.43E-198	Up	1.62E+00	4.91E-80	Up	1.20E+00	5.78E-61	Up	1.35E+00	9.49E-74	Up	1.31E+00	4.72E-68	Up	1.94E+00	5.18E-187	Up	Unigene11053_All//gi|190148361|gb|ACE63263.1|//0.00E+00//histidine kinase 3B [Populus trichocarpa]	Unigene11053_All//0.00E+00//AT1G27320| AHK3 (ARABIDOPSIS HISTIDINE KINASE 3); cytokinin receptor/ osmosensor/ protein histidine kinase	Unigene11053_All//0.00E+00//ES839435	Orphans		GO:0004673//GO:0004888//GO:0006351//GO:0006468//GO:0006826//GO:0006952//GO:0006970//GO:0009267//GO:0009409//GO:0009737//GO:0009744//GO:0009755//GO:0009845//GO:0009888//GO:0009908//GO:0010260//GO:0015996//GO:0016020//GO:0016791//GO:0019901//GO:0023060//GO:0032559//GO:0040007//GO:0043231//GO:2000026	nr -
Unigene110536_All	496	0.00	0.00	0.00	0.00	0.00	0.00	2.30	0.42	0.00	null	null		null	null		null	null		null	null		-2.45E+00	2.46E-04	Down	-1.12E+01	1.65E-08	Down	Unigene110536_All//gi|186898205|gb|ACC93947.1|//4.00E-58//heat-shock protein 70 [Hevea brasiliensis]	Unigene110536_All//5.00E-55//AT3G09440| heat shock cognate 70 kDa protein 3 (HSC70-3) (HSP70-3)	Unigene110536_All//4.00E-23//ES849176		ko03040|Spliceosome//ko04141|Protein processing in endoplasmic reticulum//ko04144|Endocytosis		nr -

Unigene110537_All	360	0.00	0.00	0.00	0.00	0.00	0.00	2.00	0.00	0.00	null	null		null	null		null	null		null	null		-1.10E+01	3.27E-05	Down	-1.10E+01	1.78E-05	Down	Unigene110537_All//gi|299470507|emb|CBN78498.1|//3.00E-07//carnitine/acylcarnitine carrier protein [Ectocarpus siliculosus]	Unigene110537_All//5.00E-05//AT1G25380| mitochondrial substrate carrier family protein					nr -
Unigene110539_All	1050	0.00	0.00	0.00	0.00	0.00	0.09	6.85	0.08	0.46	null	null		null	null		null	null		6.43E+00	4.80E-01		-6.43E+00	2.00E-47	Down	-3.91E+00	3.94E-38	Down	Unigene110539_All//gi|194136583|gb|ACF33514.1|//6.00E-81//salicylic acid methyl transferase-like protein [Glycine max]	Unigene110539_All//2.00E-75//AT5G38020| S-adenosyl-L-methionine:carboxyl methyltransferase family protein			ko00592|alpha-Linolenic acid metabolism		nr -
Unigene11054_All	4216	6.17	8.81	6.64	9.23	9.62	10.71	15.08	29.40	52.60	5.14E-01	4.58E-09		1.07E-01	3.91E-01		6.03E-02	5.79E-01		2.15E-01	8.17E-03		9.63E-01	1.29E-102		1.80E+00	0.00E+00	Up	Unigene11054_All//gi|190148357|gb|ACE63261.1|//0.00E+00//histidine kinase 3 [Betula pendula]	Unigene11054_All//0.00E+00//AT1G27320| AHK3 (ARABIDOPSIS HISTIDINE KINASE 3); cytokinin receptor/ osmosensor/ protein histidine kinase	Unigene11054_All//0.00E+00//TC179474	Orphans	ko04712|Circadian rhythm - plant	GO:0004673//GO:0004888//GO:0006351//GO:0006468//GO:0006826//GO:0006952//GO:0006970//GO:0009267//GO:0009409//GO:0009737//GO:0009744//GO:0009755//GO:0009845//GO:0009888//GO:0009908//GO:0010260//GO:0015996//GO:0016020//GO:0016791//GO:0019901//GO:0023060//GO:0032559//GO:0040007//GO:2000026	nr +
Unigene110543_All	303	0.00	0.00	0.00	0.00	0.00	0.00	2.37	0.69	0.00	null	null		null	null		null	null		null	null		-1.78E+00	2.79E-02		-1.12E+01	1.78E-05	Down							
Unigene110544_All	530	0.00	0.00	0.00	0.00	0.00	0.00	3.03	2.21	0.00	null	null		null	null		null	null		null	null		-4.57E-01	3.54E-01		-1.16E+01	7.00E-12	Down	Unigene110544_All//gi|145353601|ref|XP_001421096.1|//3.00E-58//Receptor of activated protein kinase C 1A, component of 40S small ribosomal subunit [Ostreococcus lucimarinus CCE9901] >gi|145357301|ref|XP_001422858.1| Receptor of activated protein kinase C 1B, component of 40S small ribosomal subunit [Ostreococcus lucimarinus CCE9901] >gi|144581332|gb|ABO99389.1| Receptor of activated protein kinase C 1A, component of 40S small ribosomal subunit [Ostreococcus lucimarinus CCE9901] >gi|144583102|gb|ABP01217.1| Receptor of activated protein kinase C 1B, component of 40S small ribosomal subunit [Ostreococcus lucimarinus CCE9901]	Unigene110544_All//1.00E-52//AT1G48630| RACK1B_AT (RECEPTOR FOR ACTIVATED C KINASE 1 B); nucleotide binding	Unigene110544_All//2.00E-13//gi|78353144|gb|DT573418.1|DT573418		ko03040|Spliceosome//ko03022|Basal transcription factors		nr -
Unigene110545_All	642	0.00	0.00	0.00	0.00	0.00	0.00	6.72	7.62	0.74	null	null		null	null		null	null		null	null		1.81E-01	5.18E-01		-3.17E+00	1.79E-19	Down							
Unigene110546_All	416	0.00	0.00	0.00	0.00	0.00	0.11	6.20	0.10	0.29	null	null		null	null		null	null		6.76E+00	7.02E-01		-5.95E+00	9.35E-17	Down	-4.43E+00	3.02E-15	Down	Unigene110546_All//gi|224141527|ref|XP_002324121.1|//9.00E-20//ABC transporter family protein [Populus trichocarpa] >gi|222867123|gb|EEF04254.1| ABC transporter family protein [Populus trichocarpa]	Unigene110546_All//2.00E-19//AT5G39040| ATTAP2; ATPase, coupled to transmembrane movement of substances / transporter	Unigene110546_All//1.00E-10//TC135398				nr -
Unigene110549_All	441	0.83	0.00	0.00	0.23	1.09	0.00	1.73	0.00	0.90	-9.70E+00	1.97E-02		-9.70E+00	2.79E-02		2.27E+00	7.83E-02		-7.82E+00	4.04E-01		-1.08E+01	1.68E-05	Down	-9.34E-01	1.82E-01		Unigene110549_All//gi|145336239|ref|NP_174256.2|//6.00E-62//nuclease [Arabidopsis thaliana]	Unigene110549_All//4.00E-63//AT1G29630| nuclease			ko03430|Mismatch repair		nr +
Unigene110551_All	1188	0.00	0.00	0.00	0.00	0.00	0.00	3.45	0.32	0.50	null	null		null	null		null	null		null	null		-3.45E+00	3.04E-19	Down	-2.78E+00	2.48E-16	Down	Unigene110551_All//gi|297824859|ref|XP_002880312.1|//1.00E-120//pectinesterase family protein [Arabidopsis lyrata subsp. lyrata] >gi|297326151|gb|EFH56571.1| pectinesterase family protein [Arabidopsis lyrata subsp. lyrata]	Unigene110551_All//3.00E-112//AT1G02810| pectinesterase family protein	Unigene110551_All//0.00E+00//TC135702		ko00500|Starch and sucrose metabolism//ko00040|Pentose and glucuronate interconversions		nr +
Unigene110553_All	315	0.00	0.00	0.00	0.00	0.00	0.00	2.42	0.40	0.00	null	null		null	null		null	null		null	null		-2.60E+00	2.81E-03		-1.12E+01	8.88E-06	Down	Unigene110553_All//gi|160330969|ref|XP_001712192.1|//7.00E-29//ef2 [Hemiselmis andersenii] >gi|159765639|gb|ABW97867.1| ef2 [Hemiselmis andersenii]	Unigene110553_All//1.00E-27//AT1G56070| LOS1; copper ion binding / translation elongation factor/ translation factor, nucleic acid binding					nr +
Unigene11056_All	3466	102.74	46.09	56.15	159.60	157.59	105.74	45.12	12.71	16.28	-1.16E+00	0.00E+00	Down	-8.72E-01	2.89E-197		-1.83E-02	5.30E-01		-5.94E-01	4.55E-175		-1.83E+00	0.00E+00	Down	-1.47E+00	4.03E-260	Down	Unigene11056_All//gi|297825203|ref|XP_002880484.1|//0.00E+00//armadillo/beta-catenin repeat family protein [Arabidopsis lyrata subsp. lyrata] >gi|297326323|gb|EFH56743.1| armadillo/beta-catenin repeat family protein [Arabidopsis lyrata subsp. lyrata]	Unigene11056_All//0.00E+00//AT2G23140| binding / ubiquitin-protein ligase	Unigene11056_All//0.00E+00//TC140444		ko00520|Amino sugar and nucleotide sugar metabolism	GO:0016874	nr -
Unigene110561_All	249	0.00	0.00	0.00	0.00	0.00	0.00	3.40	0.34	0.32	null	null		null	null		null	null		null	null		-3.34E+00	2.51E-04	Down	-3.41E+00	1.35E-04	Down	Unigene110561_All//gi|44662866|gb|AAS47512.1|//1.00E-12//ribosomal protein L37 [Glycine max]				ko03010|Ribosome		nr -
Unigene110569_All	534	0.00	0.00	0.00	0.00	0.00	0.00	3.09	0.39	0.37	null	null		null	null		null	null		null	null		-2.98E+00	3.67E-07	Down	-3.05E+00	1.31E-07	Down	Unigene110569_All//gi|300266999|gb|EFJ51184.1|//3.00E-32//component of cytosolic 80S ribosome and 60S large subunit [Volvox carteri f. nagariensis]	Unigene110569_All//5.00E-31//AT3G09630| 60S ribosomal protein L4/L1 (RPL4A)			ko03010|Ribosome		nr +
Unigene11057_All	2242	0.68	0.59	0.81	1.78	1.61	1.47	3.15	5.33	13.93	-2.04E-01	6.81E-01		2.55E-01	6.40E-01		-1.40E-01	6.82E-01		-2.74E-01	4.20E-01		7.60E-01	2.28E-07		2.15E+00	4.10E-87	Up	Unigene11057_All//gi|297825203|ref|XP_002880484.1|//1.00E-159//armadillo/beta-catenin repeat family protein [Arabidopsis lyrata subsp. lyrata] >gi|297326323|gb|EFH56743.1| armadillo/beta-catenin repeat family protein [Arabidopsis lyrata subsp. lyrata]	Unigene11057_All//3.00E-150//AT2G23140| binding / ubiquitin-protein ligase	Unigene11057_All//0.00E+00//gi|78340263|gb|DT560537.1|DT560537				nr +
Unigene110571_All	1033	0.05	0.00	0.00	0.14	0.13	0.04	5.69	0.73	0.73	-5.66E+00	6.18E-01		-5.66E+00	6.31E-01		-1.18E-01	9.11E-01		-1.73E+00	5.02E-01		-2.97E+00	8.14E-24	Down	-2.96E+00	1.42E-24	Down	Unigene110571_All//gi|9759032|dbj|BAB09401.1|//1.00E-127//transmembrane transport protein-like [Arabidopsis thaliana]	Unigene110571_All//1.00E-110//AT5G50300| xanthine/uracil/vitamin C permease family protein					nr +
Unigene110572_All	388	0.27	0.39	0.69	1.67	0.18	0.81	1.85	0.86	0.10	5.39E-01	7.80E-01		1.35E+00	4.09E-01		-3.23E+00	2.49E-03		-1.03E+00	2.68E-01		-1.10E+00	1.49E-01		-4.17E+00	1.65E-04	Down	Unigene110572_All//gi|297836510|ref|XP_002886137.1|//2.00E-65//FAT domain-containing protein [Arabidopsis lyrata subsp. lyrata] >gi|297331977|gb|EFH62396.1| FAT domain-containing protein [Arabidopsis lyrata subsp. lyrata]	Unigene110572_All//1.00E-66//AT4G36080| FAT domain-containing protein / phosphatidylinositol 3- and 4-kinase family protein	Unigene110572_All//0.00E+00//CO110904				nr +
Unigene110575_All	668	0.00	0.00	0.00	0.00	0.00	0.00	2.22	0.44	0.42	null	null		null	null		null	null		null	null		-2.34E+00	3.57E-05	Down	-2.41E+00	1.48E-05	Down	Unigene110575_All//gi|183229550|gb|ACC60273.1|//2.00E-55//aluminum-activated malate transporter [Glycine max]	Unigene110575_All//6.00E-53//AT3G11680| unknown protein					nr -
Unigene110577_All	595	0.00	0.00	0.00	0.00	0.00	0.00	2.49	0.56	0.33	null	null		null	null		null	null		null	null		-2.15E+00	9.69E-05	Down	-2.89E+00	1.35E-06	Down	Unigene110577_All//gi|146454508|gb|ABQ41920.1|//4.00E-45//60S ribosomal protein large subunit 9 [Sonneratia alba] >gi|156633690|gb|ABU90844.1| ribosomal protein L9 [Sonneratia alba] >gi|156633692|gb|ABU90845.1| ribosomal protein L9 [Sonneratia alba] >gi|156633694|gb|ABU90846.1| ribosomal protein L9 [Sonneratia alba] >gi|156633696|gb|ABU90847.1| ribosomal protein L9 [Sonneratia alba] >gi|156633698|gb|ABU90848.1| ribosomal protein L9 [Sonneratia alba] >gi|156633700|gb|ABU90849.1| ribosomal protein L9 [Sonneratia alba] >gi|156633702|gb|ABU90850.1| ribosomal protein L9 [Sonneratia alba] >gi|156633704|gb|ABU90851.1| ribosomal protein L9 [Sonneratia alba] >gi|156633706|gb|ABU90852.1| ribosomal protein L9 [Sonneratia alba] >gi|156633708|gb|ABU90853.1| ribosomal protein L9 [Sonneratia alba] >gi|156633710|gb|ABU90854.1| ribosomal protein L9 [Sonneratia alba] >gi|156633712|gb|ABU90855.1| ribosomal protein L9 [Sonneratia alba] >gi|156633714|gb|ABU90856.1| ribosomal protein L9 [Sonneratia	Unigene110577_All//2.00E-42//AT1G33140| PGY2 (PIGGYBACK2); structural constituent of ribosome			ko03010|Ribosome		nr +
Unigene110578_All	677	0.00	2.25	0.31	0.74	0.36	0.00	1.44	0.86	0.94	1.11E+01	1.10E-08	Up	8.30E+00	1.42E-01		-1.05E+00	3.15E-01		-9.52E+00	2.98E-03		-7.33E-01	2.48E-01		-6.09E-01	3.16E-01				Unigene110578_All//1.00E-08//FL576999				
Unigene110579_All	366	2.14	2.91	1.45	3.27	3.01	3.95	8.78	16.10	18.72	4.39E-01	5.07E-01		-5.60E-01	4.96E-01		-1.18E-01	8.27E-01		2.74E-01	6.49E-01		8.75E-01	6.26E-05		1.09E+00	6.01E-08	Up			Unigene110579_All//6.00E-74//TC142193				
Unigene110582_All	448	0.00	0.00	0.00	0.11	0.00	0.00	3.87	1.96	0.44	null	null		null	null		-6.80E+00	5.62E-01		-6.80E+00	6.43E-01		-9.82E-01	2.77E-02		-3.12E+00	3.99E-08	Down			Unigene110582_All//0.00E+00//gi|109883187|gb|DW512157.1|DW512157				ESTscan +
Unigene110583_All	278	0.00	0.00	0.00	0.00	0.00	0.00	1.83	0.00	0.00	null	null		null	null		null	null		null	null		-1.08E+01	8.90E-04	Down	-1.08E+01	5.55E-04	Down	Unigene110583_All//gi|159474294|ref|XP_001695264.1|//1.00E-16//heat shock protein 90A [Chlamydomonas reinhardtii] >gi|158276198|gb|EDP01972.1| heat shock protein 90A [Chlamydomonas reinhardtii]	Unigene110583_All//3.00E-13//AT5G56030| HSP81-2 (HEAT SHOCK PROTEIN 81-2); ATP binding			ko04626|Plant-pathogen interaction//ko04141|Protein processing in endoplasmic reticulum		nr +
Unigene110597_All	438	0.00	0.00	0.00	0.00	0.00	0.00	2.32	0.00	0.00	null	null		null	null		null	null		null	null		-1.12E+01	2.97E-07	Down	-1.12E+01	1.35E-07	Down							ESTscan -
Unigene110598_All	252	0.00	0.00	0.00	0.00	0.00	0.00	1.68	13.27	1.26	null	null		null	null		null	null		null	null		2.98E+00	7.53E-14	Up	-4.08E-01	6.54E-01								
Unigene110604_All	257	0.00	0.00	0.00	0.00	0.00	0.00	4.94	4.39	0.00	null	null		null	null		null	null		null	null		-1.69E-01	7.53E-01		-1.23E+01	1.99E-09	Down	Unigene110604_All//gi|27450759|gb|AAO14682.1|AF508263_1//7.00E-20//actin [Pyrocystis lunula]	Unigene110604_All//2.00E-20//AT5G09810| ACT7 (ACTIN 7); structural constituent of cytoskeleton	Unigene110604_All//1.00E-21//gi|31406833|gb|CD485868.1|CD485868		ko04145|Phagosome		nr -
Unigene110606_All	447	0.00	0.00	0.00	0.00	0.00	0.00	1.23	0.19	0.00	null	null		null	null		null	null		null	null		-2.72E+00	1.22E-02		-1.03E+01	2.81E-04	Down	Unigene110606_All//gi|297842159|ref|XP_002888961.1|//2.00E-16//60S ribosomal protein L6 [Arabidopsis lyrata subsp. lyrata] >gi|297334802|gb|EFH65220.1| 60S ribosomal protein L6 [Arabidopsis lyrata subsp. lyrata]	Unigene110606_All//1.00E-10//AT1G74060| 60S ribosomal protein L6 (RPL6B)	Unigene110606_All//8.00E-09//TC147093		ko03010|Ribosome		nr +
Unigene110608_All	471	0.00	0.00	0.00	0.00	0.00	0.00	3.23	0.09	0.25	null	null		null	null		null	null		null	null		-5.19E+00	1.55E-09	Down	-3.67E+00	3.49E-08	Down	Unigene110608_All//gi|297812535|ref|XP_002874151.1|//5.00E-10//60S ribosomal protein L13 [Arabidopsis lyrata subsp. lyrata] >gi|297319988|gb|EFH50410.1| 60S ribosomal protein L13 [Arabidopsis lyrata subsp. lyrata]	Unigene110608_All//4.00E-11//AT5G23900| 60S ribosomal protein L13 (RPL13D)			ko03010|Ribosome		nr -

Unigene11061_All	3271	0.99	2.37	0.75	6.69	5.26	7.75	28.15	16.99	31.89	1.26E+00	1.01E-08	Up	-4.05E-01	2.71E-01		-3.45E-01	2.02E-03		2.12E-01	6.78E-02		-7.28E-01	9.34E-48		1.80E-01	6.51E-05		Unigene11061_All//gi|301119757|ref|XP_002907606.1|//1.00E-19//NUK6 [Phytophthora infestans T30-4] >gi|262106118|gb|EEY64170.1| NUK6 [Phytophthora infestans T30-4]	Unigene11061_All//4.00E-13//AT2G22795| unknown protein	Unigene11061_All//0.00E+00//TC174208				nr -
Unigene110614_All	427	0.00	0.00	0.00	0.00	0.00	0.00	1.78	0.10	0.09	null	null		null	null		null	null		null	null		-4.19E+00	1.58E-04	Down	-4.26E+00	8.66E-05	Down							
Unigene110616_All	533	0.00	0.00	0.00	0.00	0.00	0.00	4.84	0.24	0.00	null	null		null	null		null	null		null	null		-4.36E+00	1.54E-14	Down	-1.22E+01	5.57E-19	Down	Unigene110616_All//gi|301122321|ref|XP_002908887.1|//2.00E-40//40S ribosomal protein S10 [Phytophthora infestans T30-4] >gi|66270167|gb|AAY43413.1| ribosomal protein S10 [Phytophthora infestans] >gi|262099649|gb|EEY57701.1| 40S ribosomal protein S10 [Phytophthora infestans T30-4]	Unigene110616_All//2.00E-25//AT4G25740| 40S ribosomal protein S10 (RPS10A)			ko03010|Ribosome		nr -
Unigene110617_All	324	0.00	0.00	0.00	0.00	0.00	0.00	7.70	0.90	0.74	null	null		null	null		null	null		null	null		-3.09E+00	7.09E-11	Down	-3.38E+00	3.35E-12	Down							
Unigene110619_All	297	0.00	0.00	0.00	0.17	0.23	0.00	4.70	0.00	0.67	null	null		null	null		4.68E-01	9.60E-01		-7.39E+00	6.43E-01		-1.22E+01	6.58E-10	Down	-2.81E+00	4.26E-06	Down			Unigene110619_All//4.00E-13//gi|84168982|gb|DR457630.1|DR457630				
Unigene11062_All	2638	1.39	4.17	0.99	9.14	8.71	12.09	32.74	17.97	37.32	1.59E+00	8.95E-17	Up	-4.89E-01	1.48E-01		-7.00E-02	6.17E-01		4.03E-01	1.52E-05		-8.66E-01	3.59E-60		1.89E-01	4.62E-05		Unigene11062_All//gi|89357137|gb|ABD72304.1|//2.00E-24//apple fruit acidity-related protein [Malus x domestica]	Unigene11062_All//8.00E-26//AT2G22795| unknown protein	Unigene11062_All//0.00E+00//TC164203		ko03040|Spliceosome		nr +
Unigene110621_All	484	0.00	0.00	0.00	0.00	0.00	0.00	2.88	0.00	0.00	null	null		null	null		null	null		null	null		-1.15E+01	6.58E-10	Down	-1.15E+01	2.40E-10	Down	Unigene110621_All//gi|227206418|dbj|BAH57264.1|//3.00E-06//AT2G40070 [Arabidopsis thaliana]						nr +
Unigene110623_All	507	0.00	0.00	0.00	0.00	0.00	0.00	3.09	0.00	0.00	null	null		null	null		null	null		null	null		-1.16E+01	4.29E-11	Down	-1.16E+01	1.42E-11	Down	Unigene110623_All//gi|148727870|gb|ABR08570.1|//2.00E-08//proline-rich protein [Oryza sativa Japonica Group]	Unigene110623_All//5.00E-06//AT4G02270| pollen Ole e 1 allergen and extensin family protein	Unigene110623_All//6.00E-10//BG444324				nr -
Unigene110624_All	340	0.00	0.00	0.00	0.00	0.00	0.00	1.74	0.00	0.12	null	null		null	null		null	null		null	null		-1.08E+01	2.39E-04	Down	-3.89E+00	1.10E-03								ESTscan -
Unigene110625_All	313	0.00	0.00	0.00	0.00	0.00	0.00	2.16	0.27	0.00	null	null		null	null		null	null		null	null		-3.02E+00	2.43E-03		-1.11E+01	3.57E-05	Down	Unigene110625_All//gi|58801147|dbj|BAD89506.1|//5.00E-46//beta-tubulin [Protoopalina japonica]	Unigene110625_All//3.00E-38//AT5G62700| TUB3; GTP binding / GTPase/ structural molecule	Unigene110625_All//2.00E-18//gi|78323375|gb|DT543649.1|DT543649		ko04145|Phagosome		nr -
Unigene110626_All	264	11.10	1.34	3.63	13.59	15.13	8.72	4.16	2.37	1.81	-3.05E+00	2.74E-10	Down	-1.61E+00	7.17E-05	Down	1.55E-01	6.62E-01		-6.39E-01	5.07E-02		-8.10E-01	1.69E-01		-1.20E+00	3.41E-02				Unigene110626_All//5.00E-46//TC169420				
Unigene110629_All	373	0.00	0.00	0.00	0.00	0.00	0.00	2.15	0.56	0.00	null	null		null	null		null	null		null	null		-1.94E+00	1.17E-02		-1.11E+01	4.43E-06	Down			Unigene110629_All//7.00E-09//TC149074				ESTscan -
Unigene11063_All	7254	3.94	9.44	2.31	19.15	17.71	24.31	56.80	35.01	60.52	1.26E+00	1.06E-71	Up	-7.73E-01	2.39E-13		-1.13E-01	1.08E-02		3.45E-01	7.95E-21		-6.98E-01	3.61E-196		9.14E-02	2.05E-05		Unigene11063_All//gi|38344252|emb|CAE04334.2|//2.00E-41//OSJNBa0008M17.5 [Oryza sativa (japonica cultivar-group)]	Unigene11063_All//6.00E-18//AT5G40450| unknown protein	Unigene11063_All//0.00E+00//TC156877		ko03040|Spliceosome		nr +
Unigene110638_All	504	0.00	0.00	0.00	0.00	0.00	0.00	1.93	0.66	0.00	null	null		null	null		null	null		null	null		-1.54E+00	1.90E-02		-1.09E+01	2.72E-07	Down	Unigene110638_All//gi|255073061|ref|XP_002500205.1|//2.00E-23//profilin [Micromonas sp. RCC299] >gi|226515467|gb|ACO61463.1| profilin [Micromonas sp. RCC299]	Unigene110638_All//1.00E-14//AT2G19770| PRF5 (PROFILIN5); actin binding / actin monomer binding					nr +
Unigene110640_All	365	0.00	0.00	0.00	0.14	0.00	0.00	2.20	0.69	0.33	null	null		null	null		-7.09E+00	5.62E-01		-7.09E+00	6.43E-01		-1.68E+00	2.46E-02		-2.75E+00	9.43E-04	Down			Unigene110640_All//3.00E-23//TC142576				
Unigene110644_All	399	0.00	0.00	0.00	0.00	0.00	0.00	1.80	0.10	0.20	null	null		null	null		null	null		null	null		-4.10E+00	2.91E-04	Down	-3.17E+00	8.09E-04	Down	Unigene110644_All//gi|240254117|ref|NP_173393.5|//1.00E-35//CYP722A1; electron carrier/ heme binding / iron ion binding / monooxygenase/ oxygen binding [Arabidopsis thaliana]	Unigene110644_All//7.00E-37//AT1G19630| CYP722A1; electron carrier/ heme binding / iron ion binding / monooxygenase/ oxygen binding			ko00906|Carotenoid biosynthesis//ko00904|Diterpenoid biosynthesis		nr +
Unigene110647_All	316	0.00	0.00	0.00	0.00	0.00	0.00	2.41	0.00	0.00	null	null		null	null		null	null		null	null		-1.12E+01	1.68E-05	Down	-1.12E+01	8.88E-06	Down	Unigene110647_All//gi|11131946|sp|Q9ZNY3.1|CALR_EUGGR//3.00E-14//RecName: Full=Calreticulin; Flags: Precursor >gi|3970687|emb|CAA70945.1| calreticulin precursor [Euglena gracilis]	Unigene110647_All//2.00E-05//AT1G56340| CRT1 (CALRETICULIN 1); calcium ion binding / unfolded protein binding			ko04141|Protein processing in endoplasmic reticulum//ko04145|Phagosome		nr -
Unigene110648_All	571	0.00	0.00	0.00	0.00	0.00	0.00	11.18	3.73	1.88	null	null		null	null		null	null		null	null		-1.58E+00	3.20E-12	Down	-2.57E+00	5.17E-23	Down	Unigene110648_All//gi|20197696|gb|AAM15209.1|//1.00E-14//senescence-associated protein [Arabidopsis thaliana] >gi|20197726|gb|AAM15226.1| senescence-associated protein [Arabidopsis thaliana]	Unigene110648_All//3.00E-16//AT2G21045| INVOLVED IN: aging; EXPRESSED IN: hypocotyl, root; CONTAINS InterPro DOMAIN/s: Rhodanese-like (InterPro:IPR001763); BEST Arabidopsis thaliana protein match is: unknown protein (TAIR:AT5G66170.2); Has 1919 Blast hits to 1917 proteins in 542 species: Archae - 34; Bacteria - 1258; Metazoa - 17; Fungi - 21; Plants - 115; Viruses - 0; Other Eukaryotes - 474 (source: NCBI BLink).					nr +
Unigene110649_All	372	0.00	0.00	0.00	0.00	0.00	0.00	2.84	0.67	0.43	null	null		null	null		null	null		null	null		-2.08E+00	1.78E-03		-2.73E+00	1.12E-04	Down	Unigene110649_All//gi|116058914|emb|CAL54621.1|//9.00E-10//calcineurin B regulatory subunit (ISS) [Ostreococcus tauri]	Unigene110649_All//4.00E-07//AT5G37780| CAM1 (CALMODULIN 1); calcium ion binding			ko04626|Plant-pathogen interaction//ko04070|Phosphatidylinositol signaling system//ko04650|Natural killer cell mediated cytotoxicity		nr +
Unigene11065_All	1054	2.48	1.54	2.83	3.36	4.12	3.99	4.09	1.31	1.21	-6.90E-01	7.70E-02		1.89E-01	6.29E-01		2.95E-01	3.59E-01		2.48E-01	4.68E-01		-1.64E+00	6.08E-09	Down	-1.76E+00	4.29E-10	Down	Unigene11065_All//gi|115455129|ref|NP_001051165.1|//1.00E-126//Os03g0731500 [Oryza sativa Japonica Group] >gi|113549636|dbj|BAF13079.1| Os03g0731500 [Oryza sativa Japonica Group]	Unigene11065_All//2.00E-124//AT5G42620| metalloendopeptidase/ zinc ion binding	Unigene11065_All//9.00E-162//EE592770				nr -
Unigene110650_All	357	0.15	0.00	0.15	0.00	0.19	0.25	2.61	0.12	0.22	-7.20E+00	6.18E-01		2.54E-02	9.96E-01		7.59E+00	6.14E-01		7.98E+00	4.80E-01		-4.48E+00	1.30E-05	Down	-3.55E+00	3.97E-05	Down	Unigene110650_All//gi|222424691|dbj|BAH20299.1|//7.00E-26//AT5G25770 [Arabidopsis thaliana]	Unigene110650_All//9.00E-26//AT5G25770| unknown protein	Unigene110650_All//2.00E-77//gi|164318181|gb|ES792606.1|ES792606				nr +
Unigene110652_All	301	0.17	0.00	0.53	0.17	0.34	0.00	1.83	0.69	0.00	-7.44E+00	6.18E-01		1.61E+00	5.12E-01		1.05E+00	8.09E-01		-7.37E+00	6.43E-01		-1.40E+00	1.35E-01		-1.08E+01	2.81E-04	Down							
Unigene110653_All	459	0.00	0.00	0.00	0.00	0.00	0.30	1.93	0.73	0.00	null	null		null	null		null	null		8.21E+00	3.12E-01		-1.41E+00	4.01E-02		-1.09E+01	1.10E-06	Down	Unigene110653_All//gi|7768153|emb|CAB90634.1|//7.00E-41//protein phosphatase 2C (PP2C) [Fagus sylvatica]	Unigene110653_All//2.00E-40//AT5G66080| protein phosphatase 2C family protein / PP2C family protein	Unigene110653_All//7.00E-62//TC150159				nr -
Unigene110656_All	1049	0.00	0.10	0.00	0.19	0.13	0.34	6.69	2.83	1.06	6.59E+00	3.90E-01		null	null		-5.32E-01	7.49E-01		8.59E-01	5.42E-01		-1.24E+00	1.64E-09	Down	-2.65E+00	4.03E-26	Down	Unigene110656_All//gi|30690333|ref|NP_195265.2|//1.00E-84//AGF1 (AT-hook protein of GA feedback 1); transcription factor [Arabidopsis thaliana] >gi|50198777|gb|AAT70422.1| At4g35390 [Arabidopsis thaliana] >gi|53828597|gb|AAU94408.1| At4g35390 [Arabidopsis thaliana] >gi|119657394|tpd|FAA00296.1| TPA: AT-hook motif nuclear localized protein 25 [Arabidopsis thaliana]	Unigene110656_All//8.00E-44//AT4G35390| AGF1 (AT-hook protein of GA feedback 1); transcription factor	Unigene110656_All//0.00E+00//gi|78324579|gb|DT544853.1|DT544853				nr +
Unigene110660_All	349	1.20	0.58	2.75	1.28	1.97	0.91	7.87	2.16	5.37	-1.05E+00	3.66E-01		1.20E+00	1.15E-01		6.19E-01	5.30E-01		-5.04E-01	6.49E-01		-1.87E+00	5.66E-07	Down	-5.54E-01	9.22E-02		Unigene110660_All//gi|17017951|emb|CAC84117.1|//4.00E-18//3'(2'),5'-bisphosphate nucleotidase [Gossypium hirsutum]		Unigene110660_All//5.00E-90//TC158033		ko04070|Phosphatidylinositol signaling system//ko00562|Inositol phosphate metabolism//ko00920|Sulfur metabolism		nr -
Unigene110662_All	326	0.00	0.00	0.00	0.00	0.00	0.00	2.59	0.13	0.12	null	null		null	null		null	null		null	null		-4.34E+00	4.55E-05	Down	-4.41E+00	2.37E-05	Down							ESTscan +
Unigene110666_All	355	0.59	0.71	0.00	0.28	0.00	0.89	2.98	0.47	0.90	2.76E-01	8.44E-01		-9.20E+00	1.47E-01		-8.13E+00	3.16E-01		1.67E+00	3.09E-01		-2.66E+00	2.25E-04	Down	-1.73E+00	4.76E-03		Unigene110666_All//gi|224110076|ref|XP_002315406.1|//1.00E-47//aminophospholipid ATPase [Populus trichocarpa] >gi|222864446|gb|EEF01577.1| aminophospholipid ATPase [Populus trichocarpa]	Unigene110666_All//2.00E-46//AT5G04930| ALA1 (aminophospholipid ATPase1); ATPase, coupled to transmembrane movement of ions, phosphorylative mechanism					nr -
Unigene110679_All	241	0.87	9.25	5.74	0.41	0.43	1.69	0.00	0.00	0.00	3.41E+00	1.13E-08	Up	2.73E+00	1.78E-04	Up	5.24E-02	9.68E-01		2.03E+00	1.49E-01		null	null		null	null		Unigene110679_All//gi|18397470|ref|NP_564354.1|//4.00E-22//ERD4 (early-responsive to dehydration 4) [Arabidopsis thaliana]	Unigene110679_All//8.00E-19//AT1G30360| ERD4 (early-responsive to dehydration 4)	Unigene110679_All//9.00E-106//gi|109882488|gb|DW511458.1|DW511458				nr -
Unigene110681_All	217	5.06	2.57	7.61	18.59	13.17	14.57	160.74	113.64	36.35	-9.79E-01	1.34E-01		5.87E-01	2.72E-01		-4.98E-01	9.62E-02		-3.52E-01	2.86E-01		-5.00E-01	6.51E-10		-2.14E+00	1.71E-98	Down	Unigene110681_All//gi|211906506|gb|ACJ11746.1|//1.00E-12//luminal binding protein [Gossypium hirsutum]	Unigene110681_All//5.00E-14//AT5G42020| BIP2; ATP binding	Unigene110681_All//4.00E-49//ES837705		ko04141|Protein processing in endoplasmic reticulum//ko03060|Protein export		nr -
Unigene110684_All	337	0.16	0.00	0.00	0.00	0.00	0.00	3.26	0.74	0.24	-7.28E+00	6.18E-01		-7.28E+00	6.31E-01		null	null		null	null		-2.13E+00	1.11E-03		-3.79E+00	3.27E-06	Down	Unigene110684_All//gi|15226168|ref|NP_178215.1|//8.00E-11//nucleic acid binding / zinc ion binding [Arabidopsis thaliana]	Unigene110684_All//3.00E-12//AT2G01050| nucleic acid binding / zinc ion binding					nr -
Unigene110686_All	279	0.00	0.00	0.00	0.00	0.00	0.00	3.33	0.60	0.29	null	null		null	null		null	null		null	null		-2.48E+00	1.07E-03		-3.55E+00	3.97E-05	Down							ESTscan -
Unigene110688_All	356	0.00	0.00	0.00	0.00	0.00	0.00	2.26	0.00	0.00	null	null		null	null		null	null		null	null		-1.11E+01	8.58E-06	Down	-1.11E+01	4.43E-06	Down							ESTscan +
Unigene11069_All	3171	7.05	5.42	5.98	12.27	18.70	10.95	24.85	9.71	21.90	-3.79E-01	1.13E-03		-2.37E-01	6.17E-02		6.08E-01	7.50E-22		-1.64E-01	7.83E-02		-1.36E+00	6.76E-113	Down	-1.83E-01	4.89E-04		Unigene11069_All//gi|297795237|ref|XP_002865503.1|//0.00E+00//metalloendopeptidase/ metallopeptidase/ zinc ion binding protein [Arabidopsis lyrata subsp. lyrata] >gi|297311338|gb|EFH41762.1| metalloendopeptidase/ metallopeptidase/ zinc ion binding protein [Arabidopsis lyrata subsp. lyrata]	Unigene11069_All//0.00E+00//AT5G42620| metalloendopeptidase/ zinc ion binding	Unigene11069_All//0.00E+00//TC167388			GO:0004175//GO:0009987//GO:0019538//GO:0044464//GO:0046914	nr +
Unigene110694_All	287	0.00	0.00	0.00	0.00	0.00	0.00	1.77	0.29	0.00	null	null		null	null		null	null		null	null		-2.60E+00	2.05E-02		-1.08E+01	5.55E-04	Down							ESTscan +
Unigene110695_All	231	0.00	0.00	0.00	0.00	0.00	0.00	2.75	0.72	0.17	null	null		null	null		null	null		null	null		-1.92E+00	3.09E-02		-3.99E+00	5.82E-04	Down	Unigene110695_All//gi|145408590|ref|YP_001152214.1|//2.00E-07//ORF124 [Pinus koraiensis] >gi|145048839|gb|ABP35456.1| ORF124 [Pinus koraiensis]						nr +
Unigene110696_All	211	0.00	0.00	0.00	0.00	0.00	0.00	5.81	0.40	0.00	null	null		null	null		null	null		null	null		-3.87E+00	1.15E-06	Down	-1.25E+01	4.03E-09	Down	Unigene110696_All//sp|Q86IV5|CTNA_DICDI//2.00E-06//Countin-1 OS=Dictyostelium discoideum GN=ctnA PE=1 SV=1						swissprot -
Unigene110697_All	261	0.00	0.00	0.00	0.00	0.79	0.00	2.75	0.00	0.00	null	null		null	null		9.63E+00	1.57E-01		null	null		-1.14E+01	3.27E-05	Down	-1.14E+01	1.78E-05	Down			Unigene110697_All//2.00E-76//TC174148				ESTscan -
Unigene110704_All	233	0.00	0.00	0.00	0.00	0.00	0.00	4.54	3.41	0.00	null	null		null	null		null	null		null	null		-4.13E-01	5.15E-01		-1.21E+01	6.72E-08	Down			Unigene110704_All//4.00E-09//gi|78348172|gb|DT568446.1|DT568446				

Unigene110707_All	564	0.00	0.00	0.00	0.00	0.18	0.08	2.55	0.15	0.92	null	null		null	null		7.52E+00	4.58E-01		6.32E+00	7.02E-01		-4.10E+00	5.15E-08	Down	-1.47E+00	3.09E-03		Unigene110707_All//gi|115446137|ref|NP_001046848.1|//8.00E-39//Os02g0479300 [Oryza sativa Japonica Group] >gi|47848144|dbj|BAD21925.1| XTP3-transactivated protein A-like [Oryza sativa Japonica Group] >gi|47848239|dbj|BAD22064.1| XTP3-transactivated protein A-like [Oryza sativa Japonica Group] >gi|113536379|dbj|BAF08762.1| Os02g0479300 [Oryza sativa Japonica Group]	Unigene110707_All//7.00E-28//AT3G25400| FUNCTIONS IN: molecular_function unknown; INVOLVED IN: biological_process unknown; LOCATED IN: cellular_component unknown; EXPRESSED IN: 11 plant structures; EXPRESSED DURING: 6 growth stages; CONTAINS InterPro DOMAIN/s: NTP Pyrophosphohydrolase MazG-related, RS21-C6 (InterPro:IPR011394), EAR (InterPro:IPR009039), NTP pyrophosphohydrolase MazG, putative catalytic core (InterPro:IPR004518); Has 572 Blast hits to 572 proteins in 181 species: Archae - 11; Bacteria - 286; Metazoa - 71; Fungi - 1; Plants - 31; Viruses - 0; Other Eukaryotes - 172 (source: NCBI BLink).	Unigene110707_All//4.00E-39//DW519333				nr -
Unigene110708_All	392	1.33	0.00	0.68	0.64	0.18	0.00	3.24	0.64	2.54	-1.04E+01	2.93E-03		-9.75E-01	3.77E-01		-1.85E+00	2.76E-01		-9.31E+00	7.35E-02		-2.34E+00	1.56E-04	Down	-3.49E-01	5.15E-01		Unigene110708_All//gi|242199346|gb|ACS87994.1|//4.00E-11//UDP-glucosyltransferase family 1 protein [Citrus sinensis]	Unigene110708_All//2.00E-07//AT1G78270| AtUGT85A4 (UDP-glucosyl transferase 85A4); UDP-glycosyltransferase/ glucuronosyltransferase/ transferase, transferring hexosyl groups	Unigene110708_All//4.00E-60//gi|48819923|gb|CO121236.1|CO121236				nr +
Unigene110714_All	284	0.00	0.00	0.00	0.00	0.00	0.00	3.72	1.62	0.00	null	null		null	null		null	null		null	null		-1.20E+00	4.79E-02		-1.19E+01	6.72E-08	Down	Unigene110714_All//gi|116056464|emb|CAL52753.1|//4.00E-32//ribosomal protein S20 (ISS) [Ostreococcus tauri]	Unigene110714_All//1.00E-31//AT5G62300| 40S ribosomal protein S20 (RPS20C)			ko03010|Ribosome		nr -
Unigene110717_All	304	1.03	0.00	0.88	0.98	0.45	0.45	9.32	29.83	8.39	-1.00E+01	3.66E-02		-2.38E-01	8.50E-01		-1.12E+00	4.24E-01		-1.14E+00	4.58E-01		1.68E+00	1.98E-18	Up	-1.52E-01	6.44E-01				Unigene110717_All//2.00E-14//gi|3326594|gb|AI055480.1|AI055480				
Unigene110719_All	1481	0.00	0.00	0.00	0.00	0.00	0.00	2.14	1.89	0.16	null	null		null	null		null	null		null	null		-1.79E-01	6.32E-01		-3.73E+00	1.43E-16	Down	Unigene110719_All//gi|260446989|emb|CBG76271.1|//6.00E-44//OO_Ba0005L10-OO_Ba0081K17.22 [Oryza officinalis]	Unigene110719_All//1.00E-42//AT2G44570| AtGH9B12 (Arabidopsis thaliana glycosyl hydrolase 9B12); catalytic/ hydrolase, hydrolyzing O-glycosyl compounds			ko00500|Starch and sucrose metabolism		nr -
Unigene110721_All	362	0.00	0.00	0.00	0.00	0.00	0.00	1.75	0.92	0.00	null	null		null	null		null	null		null	null		-9.23E-01	2.63E-01		-1.08E+01	7.12E-05	Down	Unigene110721_All//gi|301111866|ref|XP_002905012.1|//2.00E-27//major vault protein [Phytophthora infestans T30-4] >gi|262095342|gb|EEY53394.1| major vault protein [Phytophthora infestans T30-4]						nr +
Unigene110723_All	623	0.00	0.00	0.00	0.00	0.06	0.22	1.63	0.27	0.13	null	null		null	null		5.79E+00	8.37E-01		7.76E+00	3.12E-01		-2.60E+00	3.81E-04	Down	-3.67E+00	1.15E-05	Down	Unigene110723_All//gi|292668903|gb|ADE41106.1|//3.00E-11//AP2 domain class transcription factor [Malus x domestica]	Unigene110723_All//1.00E-08//AT4G27950| CRF4 (CYTOKININ RESPONSE FACTOR 4); DNA binding / transcription factor	Unigene110723_All//2.00E-66//gi|84169116|gb|DR457764.1|DR457764	AP2-EREBP			nr +
Unigene110724_All	671	0.00	0.00	0.00	0.00	0.00	0.00	3.09	0.12	0.06	null	null		null	null		null	null		null	null		-4.63E+00	3.60E-12	Down	-5.70E+00	7.21E-14	Down	Unigene110724_All//gi|2388584|gb|AAB71465.1|//5.00E-08//EST gb|ATTS1136 comes from this gene [Arabidopsis thaliana]						nr +
Unigene110734_All	316	4.14	1.76	2.19	2.84	2.18	1.72	4.28	1.19	0.38	-1.23E+00	3.96E-02		-9.18E-01	1.38E-01		-3.81E-01	6.05E-01		-7.26E-01	3.45E-01		-1.85E+00	9.22E-04	Down	-3.50E+00	4.24E-07	Down			Unigene110734_All//4.00E-121//ES818844				
Unigene110736_All	531	0.00	0.00	0.00	0.00	0.00	0.00	3.90	0.31	0.00	null	null		null	null		null	null		null	null		-3.63E+00	2.06E-10	Down	-1.19E+01	2.86E-15	Down							
Unigene110743_All	419	0.00	0.00	0.00	0.00	0.00	0.00	2.52	0.40	0.00	null	null		null	null		null	null		null	null		-2.66E+00	2.25E-04	Down	-1.13E+01	6.72E-08	Down	Unigene110743_All//gi|224139376|ref|XP_002323082.1|//3.00E-45//cytochrome P450 [Populus trichocarpa] >gi|222867712|gb|EEF04843.1| cytochrome P450 [Populus trichocarpa]	Unigene110743_All//3.00E-36//AT5G25120| CYP71B11; electron carrier/ heme binding / iron ion binding / monooxygenase/ oxygen binding	Unigene110743_All//1.00E-118//CO115885		ko00945|Stilbenoid, diarylheptanoid and gingerol biosynthesis//ko00903|Limonene and pinene degradation		nr +
Unigene110744_All	536	0.00	0.00	0.00	0.00	0.00	0.00	2.68	0.47	0.30	null	null		null	null		null	null		null	null		-2.52E+00	2.00E-05	Down	-3.17E+00	5.96E-07	Down							
Unigene110748_All	267	0.20	0.76	0.00	0.00	0.39	0.17	18.69	3.60	3.28	1.95E+00	3.76E-01		-7.61E+00	6.31E-01		8.60E+00	4.58E-01		7.40E+00	7.02E-01		-2.38E+00	4.42E-16	Down	-2.51E+00	1.10E-17	Down							
Unigene110749_All	651	0.00	0.00	0.00	0.46	0.05	0.14	3.18	0.00	0.55	null	null		null	null		-3.12E+00	7.57E-02		-1.73E+00	2.89E-01		-1.16E+01	1.17E-14	Down	-2.53E+00	7.37E-08	Down	Unigene110749_All//gi|297824857|ref|XP_002880311.1|//1.00E-37//pollen ole e 1 allergen and extensin family protein [Arabidopsis lyrata subsp. lyrata] >gi|297326150|gb|EFH56570.1| pollen ole e 1 allergen and extensin family protein [Arabidopsis lyrata subsp. lyrata]	Unigene110749_All//6.00E-38//AT2G47540| pollen Ole e 1 allergen and extensin family protein					nr +
Unigene110752_All	571	0.00	0.00	0.00	0.00	0.00	0.08	1.41	0.00	1.60	null	null		null	null		null	null		6.31E+00	7.02E-01		-1.05E+01	8.58E-06	Down	1.90E-01	7.54E-01				Unigene110752_All//1.00E-159//DR459330				
Unigene110757_All	657	0.00	0.00	0.00	0.00	0.00	0.00	3.60	1.08	0.00	null	null		null	null		null	null		null	null		-1.74E+00	1.33E-05	Down	-1.18E+01	1.96E-17	Down	Unigene110757_All//gi|298711872|emb|CBJ32893.1|//6.00E-93//EEF1A2, eukaryotic translation elongation factor 1 alpha [Ectocarpus siliculosus] >gi|298711873|emb|CBJ32894.1| EEF1A1, eukaryotic translation elongation factor 1 alpha [Ectocarpus siliculosus]	Unigene110757_All//4.00E-88//AT5G60390| elongation factor 1-alpha / EF-1-alpha	Unigene110757_All//1.00E-24//gi|13354929|gb|BG445277.1|BG445277				nr -
Unigene110758_All	304	0.00	0.00	0.00	0.00	0.00	0.00	1.95	0.96	0.00	null	null		null	null		null	null		null	null		-1.02E+00	2.40E-01		-1.09E+01	1.41E-04	Down	Unigene110758_All//gi|256427002|gb|ACU81074.1|//4.00E-44//ubiquitin extension protein [Jatropha curcas]	Unigene110758_All//2.00E-35//AT3G62250| UBQ5 (ubiquitin 5); protein binding / structural constituent of ribosome	Unigene110758_All//2.00E-27//BM359461		ko03010|Ribosome		nr +
Unigene110762_All	500	0.00	0.00	0.00	0.00	0.00	0.00	2.62	0.08	0.00	null	null		null	null		null	null		null	null		-4.97E+00	4.05E-08	Down	-1.14E+01	9.85E-10	Down	Unigene110762_All//gi|300267685|gb|EFJ51868.1|//2.00E-09//dynein heavy chain 4 [Volvox carteri f. nagariensis]	Unigene110762_All//2.00E-05//AT2G43580| chitinase, putative					nr -
Unigene110769_All	1320	0.00	0.00	0.16	0.15	0.00	0.14	4.07	0.66	4.04	null	null		7.33E+00	1.42E-01		-7.24E+00	8.47E-02		-1.41E-01	9.35E-01		-2.61E+00	3.63E-19	Down	-8.40E-03	9.65E-01		Unigene110769_All//gi|18402992|ref|NP_566685.1|//4.00E-70//oxidoreductase, 2OG-Fe(II) oxygenase family protein [Arabidopsis thaliana] >gi|29029088|gb|AAO64923.1| At3g21420 [Arabidopsis thaliana]	Unigene110769_All//4.00E-71//AT3G21420| oxidoreductase, 2OG-Fe(II) oxygenase family protein	Unigene110769_All//1.00E-44//gi|31073529|gb|CA993808.1|CA993808		ko00270|Cysteine and methionine metabolism//ko00941|Flavonoid biosynthesis		nr +
Unigene110771_All	315	1.00	3.38	0.00	0.47	1.86	1.00	4.70	3.85	1.26	1.76E+00	1.43E-02		-9.96E+00	4.93E-02		1.97E+00	7.04E-02		1.08E+00	4.76E-01		-2.88E-01	6.00E-01		-1.89E+00	2.45E-04	Down		Unigene110771_All//2.00E-05//AT3G53570| AFC1 (ARABIDOPSIS FUS3-COMPLEMENTING GENE 1); ATP binding / kinase/ protein kinase/ protein serine/threonine kinase	Unigene110771_All//2.00E-144//gi|164309664|gb|ES844604.1|ES844604				kegg -
Unigene110772_All	275	0.00	0.00	0.00	0.00	0.00	0.00	2.15	0.76	0.00	null	null		null	null		null	null		null	null		-1.50E+00	9.14E-02		-1.11E+01	1.41E-04	Down	Unigene110772_All//gi|258598734|gb|ACV83439.1|//2.00E-29//QM-like protein [Zea mays]	Unigene110772_All//3.00E-30//AT1G66580| 60S ribosomal protein L10 (RPL10C)			ko03010|Ribosome		nr -
Unigene110778_All	456	4.93	10.67	5.14	2.51	8.38	5.05	0.09	0.28	0.35	1.11E+00	6.62E-05	Up	5.86E-02	8.94E-01		1.74E+00	7.96E-08	Up	1.01E+00	1.82E-02		1.57E+00	5.46E-01		1.91E+00	3.78E-01		Unigene110778_All//gi|194466223|gb|ACF74342.1|//2.00E-33//gonadotropin beta chain [Arachis hypogaea]	Unigene110778_All//1.00E-21//AT5G14920| gibberellin-regulated family protein	Unigene110778_All//9.00E-160//TC176495				nr +
Unigene110783_All	363	0.00	0.00	0.00	0.00	0.00	0.00	2.80	0.35	0.33	null	null		null	null		null	null		null	null		-3.02E+00	1.08E-04	Down	-3.09E+00	5.36E-05	Down	Unigene110783_All//gi|6831602|sp|Q9XHE4.1|RL37A_GOSHI//2.00E-31//RecName: Full=60S ribosomal protein L37a >gi|4741896|gb|AAD28753.1|AF127042_1 60S ribosomal protein L37a [Gossypium hirsutum]	Unigene110783_All//1.00E-31//AT3G60245| 60S ribosomal protein L37a (RPL37aC)			ko03010|Ribosome		nr -
Unigene110786_All	367	0.00	0.00	0.00	0.00	0.00	0.00	4.38	0.11	0.22	null	null		null	null		null	null		null	null		-5.26E+00	4.17E-10	Down	-4.33E+00	1.41E-09	Down	Unigene110786_All//gi|297827841|ref|XP_002881803.1|//6.00E-20//40S ribosomal protein S2 [Arabidopsis lyrata subsp. lyrata] >gi|297327642|gb|EFH58062.1| 40S ribosomal protein S2 [Arabidopsis lyrata subsp. lyrata]	Unigene110786_All//3.00E-21//AT1G58684| 40S ribosomal protein S2, putative			ko03010|Ribosome		nr +
Unigene110787_All	316	0.00	0.00	0.00	0.00	0.00	0.00	2.94	1.06	0.13	null	null		null	null		null	null		null	null		-1.48E+00	2.77E-02		-4.54E+00	6.41E-06	Down	Unigene110787_All//gi|300266840|gb|EFJ51026.1|//2.00E-23//component of cytosolic 80S ribosome and 40S small subunit [Volvox carteri f. nagariensis]	Unigene110787_All//3.00E-23//AT1G48830| 40S ribosomal protein S7 (RPS7A)			ko03010|Ribosome		nr +
Unigene110789_All	313	0.00	0.81	1.36	0.32	0.44	0.29	1.76	0.53	0.00	9.66E+00	7.88E-02		1.04E+01	1.38E-02		4.67E-01	8.78E-01		-1.41E-01	9.52E-01		-1.72E+00	7.23E-02		-1.08E+01	2.81E-04	Down	Unigene110789_All//gi|115450411|ref|NP_001048806.1|//3.00E-23//Os03g0123600 [Oryza sativa Japonica Group] >gi|113547277|dbj|BAF10720.1| Os03g0123600 [Oryza sativa Japonica Group]	Unigene110789_All//3.00E-24//AT5G41390| FUNCTIONS IN: molecular_function unknown; INVOLVED IN: biological_process unknown; CONTAINS InterPro DOMAIN/s: Protein of unknown function Cys-rich (InterPro:IPR006461); BEST Arabidopsis thaliana protein match is: proline-rich family protein (TAIR:AT1G63830.2); Has 12019 Blast hits to 5816 proteins in 448 species: Archae - 6; Bacteria - 1151; Metazoa - 4980; Fungi - 1500; Plants - 2605; Viruses - 338; Other Eukaryotes - 1439 (source: NCBI BLink).	Unigene110789_All//1.00E-114//gi|21094210|gb|BQ406523.1|BQ406523				nr -
Unigene110794_All	495	0.00	0.00	0.00	0.00	0.00	0.00	2.65	0.76	0.00	null	null		null	null		null	null		null	null		-1.80E+00	1.43E-03		-1.14E+01	9.85E-10	Down	Unigene110794_All//gi|226460767|gb|EEH58061.1|//2.00E-21//profilin [Micromonas pusilla CCMP1545]	Unigene110794_All//3.00E-10//AT2G19770| PRF5 (PROFILIN5); actin binding / actin monomer binding					nr -
Unigene110795_All	422	0.00	0.00	0.00	0.00	0.00	0.00	2.50	1.88	0.47	null	null		null	null		null	null		null	null		-4.13E-01	5.15E-01		-2.41E+00	3.46E-04	Down	Unigene110795_All//gi|115440955|ref|NP_001044757.1|//2.00E-30//Os01g0840100 [Oryza sativa Japonica Group] >gi|113534288|dbj|BAF06671.1| Os01g0840100 [Oryza sativa Japonica Group]	Unigene110795_All//2.00E-22//AT3G09440| heat shock cognate 70 kDa protein 3 (HSC70-3) (HSP70-3)			ko03040|Spliceosome//ko04141|Protein processing in endoplasmic reticulum//ko04144|Endocytosis		nr +
Unigene110803_All	369	0.00	0.00	0.00	0.00	0.00	0.00	2.64	0.11	0.00	null	null		null	null		null	null		null	null		-4.54E+00	6.92E-06	Down	-1.14E+01	2.72E-07	Down	Unigene110803_All//gi|48431269|gb|AAT44124.1|//1.00E-30//F3H-like protein [Saussurea medusa]	Unigene110803_All//6.00E-23//AT2G44800| oxidoreductase, 2OG-Fe(II) oxygenase family protein	Unigene110803_All//4.00E-112//DW479710		ko00941|Flavonoid biosynthesis		nr +
Unigene110805_All	495	0.00	0.00	0.00	0.50	0.00	0.00	2.99	0.68	0.80	null	null		null	null		-8.98E+00	4.19E-02		-8.98E+00	7.35E-02		-2.15E+00	9.69E-05	Down	-1.89E+00	2.45E-04	Down							
Unigene110807_All	339	0.00	0.00	0.00	0.00	0.00	0.00	2.25	0.37	0.24	null	null		null	null		null	null		null	null		-2.60E+00	2.81E-03		-3.26E+00	4.48E-04	Down	Unigene110807_All//gi|260766526|gb|ACX50285.1|//1.00E-20//cytochrome oxidase subunit 1 [Guillardia theta]	Unigene110807_All//9.00E-18//ATMG01360| cytochrome c oxidase subunit 1			ko00190|Oxidative phosphorylation		nr -

Unigene110808_All	251	0.00	0.00	0.00	0.00	0.00	0.00	3.03	0.33	0.00	null	null		null	null		null	null		null	null		-3.19E+00	7.93E-04	Down	-1.16E+01	8.88E-06	Down	Unigene110808_All//gi|115451757|ref|NP_001049479.1|//5.00E-27//Os03g0234200 [Oryza sativa Japonica Group] >gi|303857|dbj|BAA02154.1| ubiquitin/ribosomal polyprotein [Oryza sativa Japonica Group] >gi|13358806|dbj|BAB33149.1| ubiquitin fused to ribosomal protein L40 [Oryza sativa] >gi|13358808|dbj|BAB33150.1| ubiquitin fused to ribosomal protein L40 [Oryza sativa] >gi|52077170|dbj|BAD46215.1| ubiquitin / ribosomal protein CEP52 [Oryza sativa Japonica Group] >gi|113547950|dbj|BAF11393.1| Os03g0234200 [Oryza sativa Japonica Group]	Unigene110808_All//1.00E-22//AT3G52590| UBQ1 (UBIQUITIN EXTENSION PROTEIN 1); protein binding / structural constituent of ribosome	Unigene110808_All//2.00E-26//DW500399		ko03010|Ribosome		nr +
Unigene110813_All	251	0.00	0.00	0.00	0.00	0.00	0.00	3.20	0.50	0.48	null	null		null	null		null	null		null	null		-2.68E+00	1.67E-03		-2.75E+00	9.43E-04	Down	Unigene110813_All//gi|14423905|sp|Q9XGL4.1|RL31_CYAPA//2.00E-30//RecName: Full=60S ribosomal protein L31 >gi|5123727|emb|CAB45375.1| ribosomal protein L31 [Cyanophora paradoxa]	Unigene110813_All//2.00E-30//AT5G56710| 60S ribosomal protein L31 (RPL31C)			ko03010|Ribosome		nr +
Unigene110815_All	619	0.00	0.00	0.17	0.08	0.06	0.00	3.21	2.36	77.94	null	null		7.43E+00	4.02E-01		-5.34E-01	8.28E-01		-6.33E+00	6.43E-01		-4.42E-01	3.06E-01		4.60E+00	1.01E-276	Up							
Unigene110819_All	571	0.00	0.09	0.00	0.00	0.36	0.00	2.96	0.73	0.70	6.47E+00	6.21E-01		null	null		8.50E+00	1.57E-01		null	null		-2.02E+00	5.66E-05	Down	-2.09E+00	2.24E-05	Down	Unigene110819_All//gi|15234416|ref|NP_192948.1|//4.00E-33//SMO1-1 (STEROL-4ALPHA-METHYL OXIDASE 1-1); 4,4-dimethyl-9beta,19-cyclopropylsterol-4alpha-methyl oxidase/ C-4 methylsterol oxidase/ catalytic [Arabidopsis thaliana] >gi|21928127|gb|AAM78091.1| AT4g12110/F16J13_180 [Arabidopsis thaliana] >gi|23308291|gb|AAN18115.1| At4g12110/F16J13_180 [Arabidopsis thaliana]	Unigene110819_All//3.00E-27//AT4G12110| SMO1-1 (STEROL-4ALPHA-METHYL OXIDASE 1-1); 4,4-dimethyl-9beta,19-cyclopropylsterol-4alpha-methyl oxidase/ C-4 methylsterol oxidase/ catalytic	Unigene110819_All//0.00E+00//DW512447		ko00100|Steroid biosynthesis		nr -
Unigene110820_All	330	0.00	0.00	0.00	0.00	0.00	0.00	4.10	7.22	0.00	null	null		null	null		null	null		null	null		8.16E-01	2.69E-02		-1.20E+01	4.86E-10	Down	Unigene110820_All//gi|255084545|ref|XP_002508847.1|//4.00E-41//ribosomal protein L44 [Micromonas sp. RCC299] >gi|226524124|gb|ACO70105.1| ribosomal protein L44 [Micromonas sp. RCC299]	Unigene110820_All//3.00E-41//AT4G14320| 60S ribosomal protein L36a/L44 (RPL36aB)	Unigene110820_All//4.00E-19//TC176118		ko03010|Ribosome		nr -
Unigene110821_All	414	8.47	26.93	11.06	27.07	27.45	40.04	36.15	50.18	24.15	1.67E+00	3.15E-18	Up	3.86E-01	2.08E-01		1.99E-02	9.34E-01		5.65E-01	2.35E-05		4.73E-01	1.06E-05		-5.82E-01	3.62E-06		Unigene110821_All//gi|22327990|ref|NP_200888.2|//1.00E-07//heavy-metal-associated domain-containing protein [Arabidopsis thaliana] >gi|28416657|gb|AAO42859.1| At5g60800 [Arabidopsis thaliana]	Unigene110821_All//2.00E-06//AT5G60800| heavy-metal-associated domain-containing protein	Unigene110821_All//2.00E-120//gi|78345451|gb|DT565725.1|DT565725				nr +
Unigene110822_All	529	0.00	0.19	0.00	0.19	1.11	0.17	2.80	0.16	1.05	7.58E+00	3.90E-01		null	null		2.56E+00	2.84E-02		-1.41E-01	9.52E-01		-4.15E+00	2.75E-08	Down	-1.41E+00	3.79E-03		Unigene110822_All//gi|21929220|dbj|BAC06184.1|//2.00E-69//110 kDa 4SNc-Tudor domain protein [Pisum sativum]	Unigene110822_All//2.00E-65//AT5G07350| tudor domain-containing protein / nuclease family protein	Unigene110822_All//3.00E-144//gi|84173243|gb|DR461891.1|DR461891				nr -
Unigene110824_All	705	0.00	0.00	0.00	0.00	0.00	0.00	5.34	0.47	0.79	null	null		null	null		null	null		null	null		-3.49E+00	6.76E-18	Down	-2.75E+00	6.54E-15	Down							
Unigene110825_All	212	0.00	0.00	0.00	0.00	0.00	0.00	4.19	0.59	0.00	null	null		null	null		null	null		null	null		-2.82E+00	5.65E-04	Down	-1.20E+01	1.10E-06	Down	Unigene110825_All//gi|94692251|gb|ABF46835.1|//3.00E-33//actin [Labyrinthula terrestris]	Unigene110825_All//5.00E-32//AT5G09810| ACT7 (ACTIN 7); structural constituent of cytoskeleton	Unigene110825_All//3.00E-22//gi|5045730|gb|AI726878.1|AI726878		ko04145|Phagosome		nr +
Unigene110826_All	591	0.00	0.00	0.00	0.00	0.00	0.00	4.36	0.21	0.13	null	null		null	null		null	null		null	null		-4.36E+00	1.54E-14	Down	-5.02E+00	2.78E-16	Down	Unigene110826_All//gi|159489312|ref|XP_001702641.1|//2.00E-68//ribosomal protein L3, component of cytosolic 80S ribosome and 60S large subunit [Chlamydomonas reinhardtii] >gi|158280663|gb|EDP06420.1| ribosomal protein L3, component of cytosolic 80S ribosome and 60S large subunit [Chlamydomonas reinhardtii]	Unigene110826_All//2.00E-50//AT1G43170| ARP1 (ARABIDOPSIS RIBOSOMAL PROTEIN 1); structural constituent of ribosome	Unigene110826_All//7.00E-10//ES848354		ko03010|Ribosome		nr +
Unigene110828_All	547	0.00	0.00	0.00	0.00	0.00	0.00	1.78	0.08	0.00	null	null		null	null		null	null		null	null		-4.54E+00	6.92E-06	Down	-1.08E+01	2.72E-07	Down		Unigene110828_All//2.00E-05//AT5G15090| VDAC3 (VOLTAGE DEPENDENT ANION CHANNEL 3); voltage-gated anion channel					ESTscan +
Unigene110829_All	395	0.13	0.00	0.00	0.38	0.17	0.23	2.03	0.11	0.81	-7.05E+00	6.18E-01		-7.05E+00	6.31E-01		-1.12E+00	5.85E-01		-7.26E-01	7.16E-01		-4.26E+00	8.48E-05	Down	-1.33E+00	5.40E-02		Unigene110829_All//gi|30699453|ref|NP_850983.1|//1.00E-30//SGB1 (SUPPRESSOR OF G PROTEIN BETA1); carbohydrate transmembrane transporter/ sugar:hydrogen symporter [Arabidopsis thaliana] >gi|30699455|ref|NP_178100.3| SGB1 (SUPPRESSOR OF G PROTEIN BETA1); carbohydrate transmembrane transporter/ sugar:hydrogen symporter [Arabidopsis thaliana] >gi|117940111|sp|Q2V4B9.2|PLST3_ARATH RecName: Full=Probable plastidic glucose transporter 3 >gi|222424748|dbj|BAH20327.1| AT1G79820 [Arabidopsis thaliana]	Unigene110829_All//8.00E-32//AT1G79820| SGB1 (SUPPRESSOR OF G PROTEIN BETA1); carbohydrate transmembrane transporter/ sugar:hydrogen symporter	Unigene110829_All//2.00E-40//CO109110				nr +
Unigene110834_All	415	0.00	0.00	0.00	0.00	0.00	0.00	6.52	1.31	0.38	null	null		null	null		null	null		null	null		-2.32E+00	8.55E-09	Down	-4.09E+00	3.55E-15	Down	Unigene110834_All//gi|145408595|ref|YP_001152219.1|//1.00E-15//ORF58f [Pinus koraiensis] >gi|145048844|gb|ABP35460.1| ORF58f [Pinus koraiensis]		Unigene110834_All//4.00E-29//TC150009				nr -
Unigene110836_All	433	17.40	6.79	5.41	29.91	14.79	20.13	49.21	17.95	23.65	-1.36E+00	1.59E-09	Down	-1.69E+00	3.35E-12	Down	-1.02E+00	3.47E-12	Down	-5.71E-01	1.88E-04		-1.45E+00	1.63E-34	Down	-1.06E+00	6.71E-22	Down	Unigene110836_All//gi|73761683|gb|AAZ83341.1|//6.00E-30//ubiquitin extension protein [Gossypium hirsutum]	Unigene110836_All//5.00E-27//AT2G47110| UBQ6; protein binding	Unigene110836_All//0.00E+00//gi|164334010|gb|ES806164.1|ES806164		ko03010|Ribosome		nr +
Unigene110839_All	394	0.00	0.00	0.00	0.76	0.52	0.00	2.47	0.21	1.42	null	null		null	null		-5.33E-01	6.79E-01		-9.57E+00	4.01E-02		-3.54E+00	4.32E-05	Down	-8.02E-01	1.83E-01		Unigene110839_All//gi|115451357|ref|NP_001049279.1|//4.00E-56//Os03g0198500 [Oryza sativa Japonica Group] >gi|113547750|dbj|BAF11193.1| Os03g0198500 [Oryza sativa Japonica Group]	Unigene110839_All//4.00E-52//AT1G47740| unknown protein	Unigene110839_All//0.00E+00//TC156499				nr +
Unigene110841_All	587	2634.40	2034.29	3068.81	421.68	567.39	385.70	2.81	0.78	0.75	-3.73E-01	5.91E-192		2.20E-01	4.45E-80		4.28E-01	1.74E-64		-1.29E-01	6.20E-05		-1.84E+00	2.07E-04	Down	-1.91E+00	8.63E-05	Down	Unigene110841_All//gi|224100797|ref|XP_002312019.1|//3.00E-55//light-harvesting complex I protein Lhca1 [Populus trichocarpa] >gi|222851839|gb|EEE89386.1| light-harvesting complex I protein Lhca1 [Populus trichocarpa]	Unigene110841_All//2.00E-51//AT3G54890| LHCA1; chlorophyll binding	Unigene110841_All//0.00E+00//TC132114		ko00196|Photosynthesis - antenna proteins		nr +
Unigene110845_All	454	0.00	0.00	0.00	0.00	0.00	0.00	2.05	0.92	0.26	null	null		null	null		null	null		null	null		-1.15E+00	7.87E-02		-2.96E+00	1.72E-04	Down	Unigene110845_All//gi|4115918|gb|AAD03429.1|//2.00E-33//similar to nascent polypeptide associated complex alpha chain [Arabidopsis thaliana]	Unigene110845_All//8.00E-32//AT5G13850| NACA3 (NASCENT POLYPEPTIDE-ASSOCIATED COMPLEX SUBUNIT ALPHA-LIKE PROTEIN 3)					nr +
Unigene110847_All	271	0.00	0.00	0.00	0.00	0.00	0.00	2.50	0.00	0.00	null	null		null	null		null	null		null	null		-1.13E+01	6.34E-05	Down	-1.13E+01	3.57E-05	Down							ESTscan -
Unigene110848_All	377	0.00	0.00	0.00	0.00	0.00	0.00	6.50	1.00	0.63	null	null		null	null		null	null		null	null		-2.70E+00	1.95E-09	Down	-3.36E+00	6.21E-12	Down	Unigene110848_All//gi|238014838|gb|ACR38454.1|//8.00E-17//unknown [Zea mays]		Unigene110848_All//6.00E-28//TC143817				nr -
Unigene11085_All	725	7.14	5.73	6.17	9.69	8.64	4.73	31.55	28.59	23.96	-3.18E-01	2.39E-01		-2.12E-01	4.64E-01		-1.64E-01	5.31E-01		-1.03E+00	2.31E-06	Down	-1.42E-01	2.24E-01		-3.97E-01	6.78E-05		Unigene11085_All//gi|15233656|ref|NP_194133.1|//2.00E-36//NLP7 (NIN LIKE PROTEIN 7); transcription factor [Arabidopsis thaliana]	Unigene11085_All//1.00E-37//AT4G24020| NLP7 (NIN LIKE PROTEIN 7); transcription factor	Unigene11085_All//0.00E+00//gi|73850762|gb|DT459782.1|DT459782	RWP-RK		GO:0006950//GO:0009987//GO:0010035//GO:0042126//GO:0043231	nr -
Unigene110853_All	417	0.00	0.00	0.00	0.00	0.00	0.00	2.94	0.40	18.06	null	null		null	null		null	null		null	null		-2.87E+00	2.54E-05	Down	2.62E+00	3.95E-27	Up	Unigene110853_All//gi|28300299|gb|AAO37645.1|//6.00E-30//NBS-LRR resistance protein RGH1 [Manihot esculenta]	Unigene110853_All//1.00E-18//AT3G14470| disease resistance protein (NBS-LRR class), putative	Unigene110853_All//5.00E-87//TC177573		ko04626|Plant-pathogen interaction		nr -
Unigene110854_All	590	0.00	0.00	0.00	0.00	0.00	0.00	2.65	0.50	0.00	null	null		null	null		null	null		null	null		-2.42E+00	1.29E-05	Down	-1.14E+01	1.42E-11	Down	Unigene110854_All//gi|192910756|gb|ACF06486.1|//2.00E-57//60S ribosomal protein L11 [Elaeis guineensis] >gi|192910758|gb|ACF06487.1| 60S ribosomal protein L11 [Elaeis guineensis]	Unigene110854_All//2.00E-57//AT5G45775| 60S ribosomal protein L11 (RPL11D)			ko03010|Ribosome		nr -
Unigene110857_All	277	0.00	0.00	0.00	0.00	0.00	0.00	4.73	0.00	0.14	null	null		null	null		null	null		null	null		-1.22E+01	2.56E-09	Down	-5.04E+00	1.60E-08	Down							ESTscan +
Unigene11086_All	717	2.41	9.89	3.94	7.99	8.12	12.16	13.74	25.53	23.78	2.04E+00	2.53E-15	Up	7.09E-01	7.03E-02		2.27E-02	9.55E-01		6.06E-01	1.75E-03		8.94E-01	4.45E-14		7.92E-01	3.72E-11		Unigene11086_All//gi|297803722|ref|XP_002869745.1|//3.00E-39//RWP-RK domain-containing protein [Arabidopsis lyrata subsp. lyrata] >gi|297315581|gb|EFH46004.1| RWP-RK domain-containing protein [Arabidopsis lyrata subsp. lyrata]	Unigene11086_All//4.00E-40//AT4G24020| NLP7 (NIN LIKE PROTEIN 7); transcription factor	Unigene11086_All//0.00E+00//TC134183	RWP-RK		GO:0006950//GO:0009987//GO:0010035//GO:0042126//GO:0043231	nr +
Unigene110861_All	302	173.59	76.51	232.04	73.57	98.86	35.89	0.84	0.28	0.66	-1.18E+00	6.36E-50	Down	4.19E-01	5.18E-11		4.26E-01	3.83E-06		-1.04E+00	7.99E-19	Down	-1.60E+00	3.11E-01		-3.49E-01	7.67E-01		Unigene110861_All//gi|222422857|dbj|BAH19415.1|//2.00E-08//AT4G25080 [Arabidopsis thaliana]		Unigene110861_All//3.00E-81//TC132357				nr +
Unigene110862_All	434	0.00	0.00	0.00	0.00	0.00	0.00	3.31	0.19	0.00	null	null		null	null		null	null		null	null		-4.10E+00	5.15E-08	Down	-1.17E+01	1.19E-10	Down							
Unigene110866_All	1029	0.31	0.30	0.00	0.39	0.54	0.70	2.75	0.93	0.89	-4.66E-02	9.85E-01		-8.25E+00	4.93E-02		4.67E-01	6.68E-01		8.58E-01	3.42E-01		-1.56E+00	9.71E-06	Down	-1.63E+00	2.69E-06	Down	Unigene110866_All//gi|4971998|emb|CAB43864.1|//1.00E-100//polynucleotide phosphorylase [Arabidopsis thaliana] >gi|4972000|emb|CAB43865.1| polynucleotide phosphorylase [Arabidopsis thaliana] >gi|7573307|emb|CAB87625.1| polynucleotide phosphorylase [Arabidopsis thaliana]	Unigene110866_All//3.00E-101//AT5G14580| polyribonucleotide nucleotidyltransferase, putative	Unigene110866_All//0.00E+00//TC170267		ko00230|Purine metabolism//ko00240|Pyrimidine metabolism//ko03018|RNA degradation		nr +

<table>
<tr><td>Unigene110867_All</td><td>432</td><td>0.00</td><td>0.00</td><td>0.00</td><td>0.00</td><td>0.00</td><td>0.00</td><td>5.38</td><td>0.10</td><td>0.18</td><td>null</td><td>null</td><td></td><td>null</td><td>null</td><td></td><td>null</td><td>null</td><td></td><td>null</td><td>null</td><td></td><td>-5.80E+00</td><td>5.23E-15</td><td>Down</td><td>-4.87E+00</td><td>1.64E-14</td><td>Down</td><td>Unigene110867_All//gi|301095995|ref|XP_002897096.1|//2.00E-54//40S ribosomal protein S15a [Phytophthora infestans T30-4] >gi|262107415|gb|EEY65467.1| 40S ribosomal protein S15a [Phytophthora infestans T30-4]</td><td>Unigene110867_All//8.00E-48//AT5G59850| 40S ribosomal protein S15A (RPS15aF)</td><td></td><td></td><td>ko03010|Ribosome</td><td></td><td>nr -</td></tr>
<tr><td>Unigene110868_All</td><td>415</td><td>0.00</td><td>0.00</td><td>0.00</td><td>0.00</td><td>0.00</td><td>0.00</td><td>1.63</td><td>0.10</td><td>0.00</td><td>null</td><td>null</td><td></td><td>null</td><td>null</td><td></td><td>null</td><td>null</td><td></td><td>null</td><td>null</td><td></td><td>-4.02E+00</td><td>5.36E-04</td><td>Down</td><td>-1.07E+01</td><td>3.57E-05</td><td>Down</td><td>Unigene110868_All//gi|226493384|ref|NP_001150104.1|//8.00E-32//LOC100283733 [Zea mays] >gi|195636758|gb|ACG37847.1| xaa-Pro dipeptidase [Zea mays]</td><td>Unigene110868_All//3.00E-26//AT4G29490| aminopeptidase/ manganese ion binding</td><td></td><td></td><td></td><td></td><td>nr -</td></tr>
<tr><td>Unigene110874_All</td><td>340</td><td>0.00</td><td>0.00</td><td>0.00</td><td>0.00</td><td>0.00</td><td>0.00</td><td>3.23</td><td>0.00</td><td>0.00</td><td>null</td><td>null</td><td></td><td>null</td><td>null</td><td></td><td>null</td><td>null</td><td></td><td>null</td><td>null</td><td></td><td>-1.17E+01</td><td>7.67E-08</td><td>Down</td><td>-1.17E+01</td><td>3.33E-08</td><td>Down</td><td>Unigene110874_All//gi|223995041|ref|XP_002287204.1|//3.00E-33//v-type ATPase [Thalassiosira pseudonana CCMP1335] >gi|223996057|ref|XP_002287702.1| vacuolar ATP synthase [Thalassiosira pseudonana CCMP1335] >gi|220976320|gb|EED94647.1| v-type ATPase [Thalassiosira pseudonana CCMP1335] >gi|220976818|gb|EED95145.1| vacuolar ATP synthase [Thalassiosira pseudonana CCMP1335]</td><td>Unigene110874_All//2.00E-20//AT4G34720| AVA-P1; ATPase/ proton-transporting ATPase, rotational mechanism</td><td></td><td></td><td>ko00190|Oxidative phosphorylation//ko04145|Phagosome</td><td></td><td>nr +</td></tr>
<tr><td>Unigene110875_All</td><td>397</td><td>0.00</td><td>0.00</td><td>0.00</td><td>0.00</td><td>0.00</td><td>0.00</td><td>10.54</td><td>4.00</td><td>1.10</td><td>null</td><td>null</td><td></td><td>null</td><td>null</td><td></td><td>null</td><td>null</td><td></td><td>null</td><td>null</td><td></td><td>-1.40E+00</td><td>5.05E-07</td><td>Down</td><td>-3.26E+00</td><td>2.32E-19</td><td>Down</td><td></td><td></td><td></td><td></td><td></td><td></td><td></td></tr>
<tr><td>Unigene110876_All</td><td>399</td><td>0.00</td><td>0.00</td><td>0.00</td><td>0.00</td><td>0.00</td><td>0.00</td><td>2.01</td><td>0.00</td><td>0.00</td><td>null</td><td>null</td><td></td><td>null</td><td>null</td><td></td><td>null</td><td>null</td><td></td><td>null</td><td>null</td><td></td><td>-1.10E+01</td><td>8.58E-06</td><td>Down</td><td>-1.10E+01</td><td>4.43E-06</td><td>Down</td><td>Unigene110876_All//gi|1706586|sp|P50256.1|EF1AC_PORPU//6.00E-24//RecName: Full=Elongation factor 1-alpha C; Short=EF-1-alpha C >gi|476155|gb|AAA61793.1| EF1-alpha [Porphyra purpurea]</td><td>Unigene110876_All//3.00E-21//AT5G60390| elongation factor 1-alpha / EF-1-alpha</td><td>Unigene110876_All//5.00E-13//CD809343</td><td></td><td></td><td></td><td>nr -</td></tr>
<tr><td>Unigene110877_All</td><td>298</td><td>0.00</td><td>0.00</td><td>0.00</td><td>0.00</td><td>0.00</td><td>0.00</td><td>2.13</td><td>0.00</td><td>0.27</td><td>null</td><td>null</td><td></td><td>null</td><td>null</td><td></td><td>null</td><td>null</td><td></td><td>null</td><td>null</td><td></td><td>-1.11E+01</td><td>1.23E-04</td><td>Down</td><td>-2.99E+00</td><td>2.58E-03</td><td></td><td>Unigene110877_All//sp|P22549|IPDE_DICDI//9.00E-06//Cyclic nucleotide phosphodiesterase inhibitor OS=Dictyostelium discoideum GN=pdiA PE=1 SV=1</td><td></td><td></td><td></td><td></td><td></td><td>swissprot +</td></tr>
<tr><td>Unigene110879_All</td><td>908</td><td>0.35</td><td>0.00</td><td>0.12</td><td>0.16</td><td>0.23</td><td>0.00</td><td>4.52</td><td>0.46</td><td>6.01</td><td>-8.43E+00</td><td>3.66E-02</td><td></td><td>-1.56E+00</td><td>3.30E-01</td><td></td><td>4.67E-01</td><td>8.26E-01</td><td></td><td>-7.36E+00</td><td>2.34E-01</td><td></td><td>-3.29E+00</td><td>1.63E-18</td><td>Down</td><td>4.12E-01</td><td>6.65E-02</td><td></td><td>Unigene110879_All//gi|24461866|gb|AAN62353.1|AF506028_20//2.00E-37//NBS-LRR type disease resistance protein [Citrus trifoliata]</td><td>Unigene110879_All//2.00E-24//AT1G12290| disease resistance protein (CC-NBS-LRR class), putative</td><td>Unigene110879_All//4.00E-102//gi|48775674|gb|CO085040.1|CO085040</td><td></td><td>ko04626|Plant-pathogen interaction</td><td></td><td>nr +</td></tr>
<tr><td>Unigene110881_All</td><td>651</td><td>0.16</td><td>0.00</td><td>0.00</td><td>0.38</td><td>0.26</td><td>0.14</td><td>2.40</td><td>0.06</td><td>0.06</td><td>-7.33E+00</td><td>3.76E-01</td><td></td><td>-7.33E+00</td><td>3.98E-01</td><td></td><td>-5.33E-01</td><td>7.13E-01</td><td></td><td>-1.46E+00</td><td>4.07E-01</td><td></td><td>-5.23E+00</td><td>8.04E-10</td><td>Down</td><td>-5.30E+00</td><td>2.73E-10</td><td>Down</td><td>Unigene110881_All//gi|58294490|gb|AAW70161.1|//1.00E-33//GEX2 [Arabidopsis thaliana]</td><td>Unigene110881_All//2.00E-35//AT5G49150| GEX2 (GAMETE EXPRESSED 2)</td><td>Unigene110881_All//0.00E+00//gi|164365009|gb|ES831842.1|ES831842</td><td></td><td></td><td></td><td>nr -</td></tr>
<tr><td>Unigene110883_All</td><td>332</td><td>0.00</td><td>0.00</td><td>0.00</td><td>0.00</td><td>0.00</td><td>0.00</td><td>5.22</td><td>0.25</td><td>1.08</td><td>null</td><td>null</td><td></td><td>null</td><td>null</td><td></td><td>null</td><td>null</td><td></td><td>null</td><td>null</td><td></td><td>-4.37E+00</td><td>6.16E-10</td><td>Down</td><td>-2.27E+00</td><td>5.17E-06</td><td>Down</td><td>Unigene110883_All//gi|225444704|ref|XP_002278093.1|//6.00E-41//PREDICTED: similar to aldehyde dehydrogenase family 7 member A1 [Vitis vinifera]</td><td>Unigene110883_All//4.00E-42//AT1G54100| ALDH7B4 (Aldehyde Dehydrogenase 7B4); 3-chloroallyl aldehyde dehydrogenase/ oxidoreductase</td><td></td><td></td><td>ko00010|Glycolysis / Gluconeogenesis//ko00903|Limonene and pinene degradation//ko00620|Pyruvate metabolism//ko00380|Tryptophan metabolism//ko00071|Fatty acid metabolism//ko00250|Alanine, aspartate and glutamate metabolism//ko00053|Ascorbate and aldarate metabolism//ko00330|Arginine and proline metabolism//ko00280|Valine, leucine and isoleucine degradation//ko00640|Propanoate metabolism//ko00561|Glycerolipid metabolism//ko00310|Lysine degradation//ko00350|Tyrosine metabolism//ko00650|Butanoate metabolism//ko00410|beta-Alanine metabolism//ko00340|Histidine metabolism</td><td></td><td>nr -</td></tr>
<tr><td>Unigene110886_All</td><td>346</td><td>2.27</td><td>1.61</td><td>0.92</td><td>1.58</td><td>0.90</td><td>0.39</td><td>5.99</td><td>1.93</td><td>3.91</td><td>-4.94E-01</td><td>5.30E-01</td><td></td><td>-1.30E+00</td><td>1.38E-01</td><td></td><td>-8.22E-01</td><td>3.96E-01</td><td></td><td>-2.02E+00</td><td>7.05E-02</td><td></td><td>-1.63E+00</td><td>1.21E-04</td><td>Down</td><td>-6.13E-01</td><td>1.11E-01</td><td></td><td>Unigene110886_All//gi|18405757|ref|NP_564717.1|//8.00E-12//DNAJ heat shock N-terminal domain-containing protein [Arabidopsis thaliana] >gi|30102878|gb|AAP21357.1| At1g56300 [Arabidopsis thaliana]</td><td>Unigene110886_All//3.00E-13//AT1G56300| DNAJ heat shock N-terminal domain-containing protein</td><td>Unigene110886_All//1.00E-90//TC144885</td><td></td><td></td><td></td><td>nr -</td></tr>
<tr><td>Unigene110887_All</td><td>609</td><td>0.00</td><td>0.00</td><td>0.00</td><td>0.00</td><td>0.00</td><td>0.00</td><td>3.47</td><td>1.03</td><td>0.00</td><td>null</td><td>null</td><td></td><td>null</td><td>null</td><td></td><td>null</td><td>null</td><td></td><td>null</td><td>null</td><td></td><td>-1.75E+00</td><td>3.84E-05</td><td>Down</td><td>-1.18E+01</td><td>1.40E-15</td><td>Down</td><td></td><td></td><td></td><td></td><td></td><td></td><td></td></tr>
<tr><td>Unigene110888_All</td><td>460</td><td>0.00</td><td>0.00</td><td>0.35</td><td>0.22</td><td>0.07</td><td>0.20</td><td>2.48</td><td>0.27</td><td>0.26</td><td>null</td><td>null</td><td></td><td>8.44E+00</td><td>2.40E-01</td><td></td><td>-1.53E+00</td><td>5.88E-01</td><td></td><td>-1.41E-01</td><td>9.52E-01</td><td></td><td>-3.19E+00</td><td>1.96E-05</td><td>Down</td><td>-3.26E+00</td><td>8.94E-06</td><td>Down</td><td></td><td></td><td></td><td></td><td></td><td></td><td></td></tr>
<tr><td>Unigene110889_All</td><td>453</td><td>2.08</td><td>6.38</td><td>1.41</td><td>1.54</td><td>1.22</td><td>0.50</td><td>2.71</td><td>2.86</td><td>0.35</td><td>1.62E+00</td><td>4.40E-05</td><td>Up</td><td>-5.60E-01</td><td>4.39E-01</td><td></td><td>-3.40E-01</td><td>6.80E-01</td><td></td><td>-1.63E+00</td><td>7.45E-02</td><td></td><td>7.96E-02</td><td>8.90E-01</td><td></td><td>-2.94E+00</td><td>1.14E-05</td><td>Down</td><td></td><td></td><td>Unigene110889_All//4.00E-131//TC178548</td><td></td><td></td><td></td><td></td></tr>
<tr><td>Unigene110890_All</td><td>461</td><td>0.34</td><td>0.00</td><td>0.58</td><td>0.00</td><td>0.07</td><td>0.20</td><td>2.84</td><td>0.63</td><td>0.60</td><td>-8.41E+00</td><td>2.17E-01</td><td></td><td>7.62E-01</td><td>6.44E-01</td><td></td><td>6.22E+00</td><td>8.37E-01</td><td></td><td>7.61E+00</td><td>4.80E-01</td><td></td><td>-2.16E+00</td><td>2.57E-04</td><td>Down</td><td>-2.23E+00</td><td>1.19E-04</td><td>Down</td><td></td><td></td><td>Unigene110890_All//5.00E-07//TC178893</td><td></td><td></td><td></td><td></td></tr>
<tr><td>Unigene110891_All</td><td>276</td><td>0.00</td><td>0.00</td><td>0.00</td><td>0.18</td><td>0.25</td><td>0.16</td><td>1.84</td><td>0.30</td><td>0.00</td><td>null</td><td>null</td><td></td><td>null</td><td>null</td><td></td><td>4.67E-01</td><td>9.60E-01</td><td></td><td>-1.42E-01</td><td>9.56E-01</td><td></td><td>-2.60E+00</td><td>2.05E-02</td><td></td><td>-1.08E+01</td><td>5.55E-04</td><td>Down</td><td></td><td></td><td>Unigene110891_All//3.00E-32//gi|164346740|gb|ES822710.1|ES822710</td><td></td><td></td><td></td><td></td></tr>
<tr><td>Unigene110892_All</td><td>320</td><td>0.16</td><td>0.00</td><td>0.17</td><td>0.62</td><td>0.65</td><td>0.28</td><td>2.11</td><td>0.00</td><td>0.50</td><td>-7.35E+00</td><td>6.18E-01</td><td></td><td>2.54E-02</td><td>9.96E-01</td><td></td><td>5.23E-02</td><td>9.89E-01</td><td></td><td>-1.14E+00</td><td>5.70E-01</td><td></td><td>-1.10E+01</td><td>6.34E-05</td><td>Down</td><td>-2.09E+00</td><td>1.27E-02</td><td></td><td>Unigene110892_All//gi|195633079|gb|ACG36723.1|//5.00E-08//transcriptional activator FHA1 [Zea mays] >gi|195637976|gb|ACG38456.1| transcriptional activator FHA1 [Zea mays] >gi|195648198|gb|ACG43567.1| transcriptional activator FHA1 [Zea mays]</td><td>Unigene110892_All//6.00E-14//AT3G07220| transcriptional activator, putative</td><td>Unigene110892_All//4.00E-50//DT049758</td><td></td><td></td><td></td><td>nr +</td></tr>
<tr><td>Unigene110895_All</td><td>402</td><td>0.00</td><td>0.00</td><td>0.00</td><td>1.36</td><td>1.28</td><td>0.22</td><td>6.84</td><td>0.21</td><td>0.69</td><td>null</td><td>null</td><td></td><td>null</td><td>null</td><td></td><td>-8.52E-02</td><td>9.07E-01</td><td></td><td>-2.60E+00</td><td>2.93E-02</td><td></td><td>-5.04E+00</td><td>1.05E-16</td><td>Down</td><td>-3.30E+00</td><td>4.24E-13</td><td>Down</td><td>Unigene110895_All//gi|18411885|ref|NP_565175.1|//4.00E-17//RNA recognition motif (RRM)-containing protein [Arabidopsis thaliana] >gi|21537200|gb|AAM61541.1| RNA recognition motif-containing protein SEB-4 [Arabidopsis thaliana] >gi|124300976|gb|ABN04740.1| At1g78260 [Arabidopsis thaliana]</td><td>Unigene110895_All//2.00E-18//AT1G78260| RNA recognition motif (RRM)-containing protein</td><td>Unigene110895_All//8.00E-120//gi|78345885|gb|DT566159.1|DT566159</td><td></td><td></td><td></td><td>nr -</td></tr>
<tr><td>Unigene110896_All</td><td>448</td><td>0.00</td><td>0.00</td><td>0.00</td><td>0.00</td><td>0.00</td><td>0.00</td><td>2.27</td><td>2.24</td><td>24.10</td><td>null</td><td>null</td><td></td><td>null</td><td>null</td><td></td><td>null</td><td>null</td><td></td><td>null</td><td>null</td><td></td><td>-1.66E-02</td><td>1.02E+00</td><td></td><td>3.41E+00</td><td>5.22E-50</td><td>Up</td><td></td><td></td><td></td><td></td><td></td><td></td><td></td></tr>
<tr><td>Unigene110897_All</td><td>519</td><td>0.00</td><td>0.98</td><td>0.00</td><td>0.10</td><td>0.00</td><td>0.44</td><td>1.47</td><td>0.00</td><td>0.38</td><td>9.93E+00</td><td>4.05E-03</td><td></td><td>null</td><td>null</td><td></td><td>-6.59E+00</td><td>5.62E-01</td><td></td><td>2.18E+00</td><td>3.23E-01</td><td></td><td>-1.05E+01</td><td>1.68E-05</td><td>Down</td><td>-1.93E+00</td><td>1.14E-02</td><td></td><td></td><td></td><td></td><td></td><td></td><td></td><td></td></tr>
<tr><td>Unigene110899_All</td><td>421</td><td>0.00</td><td>0.00</td><td>0.00</td><td>0.36</td><td>0.16</td><td>0.00</td><td>2.61</td><td>0.89</td><td>0.00</td><td>null</td><td>null</td><td></td><td>null</td><td>null</td><td></td><td>-1.12E+00</td><td>5.85E-01</td><td></td><td>-8.47E+00</td><td>2.34E-01</td><td></td><td>-1.55E+00</td><td>1.12E-02</td><td></td><td>-1.14E+01</td><td>3.33E-08</td><td>Down</td><td>Unigene110899_All//gi|115462287|ref|NP_001054743.1|//9.00E-19//Os05g0164900 [Oryza sativa Japonica Group] >gi|113578294|dbj|BAF16657.1| Os05g0164900 [Oryza sativa Japonica Group]</td><td>Unigene110899_All//9.00E-22//AT5G01660| FUNCTIONS IN: molecular_function unknown; INVOLVED IN: biological_process unknown; LOCATED IN: cellular_component unknown; EXPRESSED IN: 9 plant structures; EXPRESSED DURING: 6 growth stages; CONTAINS InterPro DOMAIN/s: Kelch repeat type 1 (InterPro:IPR006652), Galactose oxidase/kelch, beta-propeller (InterPro:IPR011043), Development and cell death domain (InterPro:IPR013989), Kelch related (InterPro:IPR013089), Kelch-type beta propeller (InterPro:IPR015915); BEST Arabidopsis thaliana protein match is: unknown protein (TAIR:AT3G11000.2); Has 12390 Blast hits to 5423 proteins in 323 species: Archae - 22; Bacteria - 787; Metazoa - 8729; Fungi - 223; Plants - 966; Viruses - 639; Other Eukaryotes - 1024 (source: NCBI BLink).</td><td></td><td></td><td></td><td></td><td>nr +</td></tr>
<tr><td>Unigene1109_All</td><td>1236</td><td>3.81</td><td>1.80</td><td>4.39</td><td>5.88</td><td>5.85</td><td>7.45</td><td>19.12</td><td>25.60</td><td>14.12</td><td>-1.08E+00</td><td>1.32E-04</td><td>Down</td><td>2.06E-01</td><td>4.64E-01</td><td></td><td>-8.20E-03</td><td>9.69E-01</td><td></td><td>3.41E-01</td><td>8.70E-02</td><td></td><td>4.21E-01</td><td>8.12E-07</td><td></td><td>-4.38E-01</td><td>7.54E-06</td><td></td><td>Unigene1109_All//gi|15220976|ref|NP_173253.1|//1.00E-176//ATMPK8; MAP kinase [Arabidopsis thaliana] >gi|30685711|ref|NP_849685.1| ATMPK8; MAP kinase [Arabidopsis thaliana] >gi|110287680|sp|Q9LM33.2|MPK8_ARATH RecName: Full=Mitogen-activated protein kinase 8; Short=MAP kinase 8; Short=AtMPK8 >gi|9719729|gb|AAF97831.1|AC034107_14 Strong similarity (practically identical) to ATMPK8 gene from Arabidopsis thaliana gb|AB038693 and contains a eukaryotic protein kinase PF|00069 domain. ESTs gb|AV526779, gb|AV527934, gb|AV540522, gb|T22988, gb|R90476, gb|Z24497, gb|N97150, gb|AA713291, gb|AI100188 come from this gene</td><td>Unigene1109_All//8.00E-155//AT1G18150| ATMPK8; MAP kinase</td><td>Unigene1109_All//0.00E+00//TC176812</td><td></td><td>ko04626|Plant-pathogen interaction//ko04650|Natural killer cell mediated cytotoxicity</td><td></td><td>nr -</td></tr>
<tr><td>Unigene110900_All</td><td>347</td><td>0.00</td><td>0.00</td><td>0.00</td><td>0.00</td><td>0.00</td><td>0.13</td><td>25.59</td><td>0.36</td><td>0.00</td><td>null</td><td>null</td><td></td><td>null</td><td>null</td><td></td><td>null</td><td>null</td><td></td><td>7.02E+00</td><td>7.02E-01</td><td></td><td>-6.15E+00</td><td>9.52E-58</td><td>Down</td><td>-1.46E+01</td><td>2.26E-65</td><td>Down</td><td>Unigene110900_All//gi|84514187|gb|ABC59102.1|//1.00E-26//cytochrome P450 monooxygenase CYP706A12 [Medicago truncatula]</td><td>Unigene110900_All//6.00E-21//AT5G44620| CYP706A3; electron carrier/ heme binding / iron ion binding / monooxygenase/ oxygen binding</td><td>Unigene110900_All//3.00E-91//TC134420</td><td></td><td>ko00940|Phenylpropanoid biosynthesis//ko00945|Stilbenoid, diarylheptanoid and gingerol biosynthesis//ko00903|Limonene and pinene degradation//ko00941|Flavonoid biosynthesis//ko00360|Phenylalanine metabolism</td><td></td><td>nr -</td></tr>
<tr><td>Unigene110905_All</td><td>410</td><td>0.00</td><td>0.00</td><td>0.00</td><td>0.00</td><td>0.00</td><td>0.00</td><td>1.55</td><td>0.00</td><td>0.19</td><td>null</td><td>null</td><td></td><td>null</td><td>null</td><td></td><td>null</td><td>null</td><td></td><td>null</td><td>null</td><td></td><td>-1.06E+01</td><td>1.23E-04</td><td>Down</td><td>-2.99E+00</td><td>2.58E-03</td><td></td><td>Unigene110905_All//gi|219110933|ref|XP_002177218.1|//4.00E-34//gelsolin [Phaeodactylum tricornutum CCAP 1055/1] >gi|217411753|gb|EEC51681.1| gelsolin [Phaeodactylum tricornutum CCAP 1055/1]</td><td>Unigene110905_All//1.00E-31//AT2G41740| VLN2 (VILLIN 2); actin binding</td><td></td><td></td><td></td><td></td><td>nr -</td></tr>
</table>

Unigene110909_All	287	0.00	0.00	0.00	0.00	0.00	0.00	8.25	1.46	0.28	null	null		null	null		null	null		null	null		-2.50E+00	1.92E-08	Down	-4.89E+00	8.31E-15	Down	Unigene110909_All//gi|224085888|ref|XP_002307727.1|//1.00E-26//tubulin, beta chain [Populus trichocarpa] >gi|222857176|gb|EEE94723.1| tubulin, beta chain [Populus trichocarpa]	Unigene110909_All//9.00E-23//AT5G23860| TUB8; structural constituent of cytoskeleton	Unigene110909_All//7.00E-33//TC166231		ko04145|Phagosome		nr -
Unigene11091_All	3281	1.82	4.34	2.63	4.78	3.97	6.41	12.54	17.02	21.37	1.26E+00	2.39E-15	Up	5.32E-01	9.54E-03		-2.70E-01	5.88E-02		4.24E-01	3.33E-04		4.41E-01	2.40E-12		7.69E-01	4.72E-41		Unigene11091_All//gi|15217662|ref|NP_176634.1|//0.00E+00//RWP-RK domain-containing protein [Arabidopsis thaliana]	Unigene11091_All//0.00E+00//AT1G64530| RWP-RK domain-containing protein	Unigene11091_All//0.00E+00//TC177468	RWP-RK		GO:0006950//GO:0009987//GO:0010035//GO:0042126//GO:0043231	nr -
Unigene110911_All	383	0.00	0.00	0.00	0.00	0.00	0.00	1.66	1.31	0.10	null	null		null	null		null	null		null	null		-3.39E-01	6.63E-01		-3.99E+00	5.82E-04	Down	Unigene110911_All//gi|223992823|ref|XP_002286095.1|//3.00E-46//guanine nucleotide binding protein beta subunit-like protein [Thalassiosira pseudonana CCMP1335] >gi|220977410|gb|EED95736.1| guanine nucleotide binding protein beta subunit-like protein [Thalassiosira pseudonana CCMP1335]	Unigene110911_All//2.00E-43//AT1G18080| ATARCA; nucleotide binding			ko03022|Basal transcription factors		nr +
Unigene110915_All	363	0.00	0.00	0.00	0.00	0.00	0.00	1.98	0.23	0.00	null	null		null	null		null	null		null	null		-3.10E+00	1.40E-03		-1.10E+01	1.78E-05	Down	Unigene110915_All//gi|262360187|gb|ACY38051.2|//2.00E-19//cysteine proteinase C1A [Dactylis glomerata]	Unigene110915_All//5.00E-19//AT5G50260| cysteine proteinase, putative					nr -
Unigene110917_All	317	0.00	0.00	0.00	0.63	0.00	0.14	1.87	0.00	0.00	null	null		null	null		-9.30E+00	8.47E-02		-2.14E+00	3.48E-01		-1.09E+01	2.39E-04	Down	-1.09E+01	1.41E-04	Down	Unigene110917_All//gi|115468064|ref|NP_001057631.1|//1.00E-31//Os06g0474300 [Oryza sativa Japonica Group] >gi|113595671|dbj|BAF19545.1| Os06g0474300 [Oryza sativa Japonica Group]	Unigene110917_All//1.00E-44//AT3G05390| unknown protein					nr -
Unigene110919_All	342	0.00	0.00	0.16	0.00	0.00	0.00	5.44	0.73	0.70	null	null		7.28E+00	6.40E-01		null	null		null	null		-2.89E+00	8.76E-08	Down	-2.96E+00	2.82E-08	Down	Unigene110919_All//gi|7105717|gb|AAB84202.2|//1.00E-09//plasma membrane proton ATPase [Kosteletzkya virginica]	Unigene110919_All//4.00E-10//AT2G24520| AHA5 (Arabidopsis H(+)-ATPase 5); ATPase	Unigene110919_All//7.00E-21//CO113495		ko00190|Oxidative phosphorylation		nr -
Unigene110925_All	399	0.00	0.00	0.00	0.00	0.09	0.11	0.32	5.55	0.80	null	null		null	null		6.43E+00	8.37E-01		6.82E+00	7.02E-01		4.13E+00	4.45E-12	Up	1.33E+00	2.93E-01		Unigene110925_All//gi|224083966|ref|XP_002307190.1|//2.00E-13//predicted protein [Populus trichocarpa] >gi|222856639|gb|EEE94186.1| predicted protein [Populus trichocarpa]						nr +
Unigene110926_All	496	0.00	0.00	0.21	0.00	0.07	0.00	2.30	0.51	0.96	null	null		7.75E+00	4.02E-01		6.12E+00	8.37E-01		null	null		-2.19E+00	6.84E-04	Down	-1.26E+00	2.42E-02		Unigene110926_All//gi|79390803|ref|NP_187087.2|//4.00E-23//PDLP4 (PLASMODESMATA-LOCATED PROTEIN 4) [Arabidopsis thaliana] >gi|158563987|sp|Q6E263.2|CRR39_ARATH RecName: Full=Cysteine-rich repeat secretory protein 39; Flags: Precursor	Unigene110926_All//6.00E-19//AT3G04370| PDLP4 (PLASMODESMATA-LOCATED PROTEIN 4)					nr +
Unigene110928_All	361	0.00	0.00	0.00	0.00	0.00	0.00	6.79	3.47	2.98	null	null		null	null		null	null		null	null		-9.68E-01	7.64E-03		-1.19E+00	8.55E-04	Down			Unigene110928_All//5.00E-28//BG445269				
Unigene11093_All	4362	4.21	1.85	3.64	9.52	8.94	6.21	10.79	4.96	5.93	-1.19E+00	4.07E-18	Down	-2.11E-01	1.43E-01		-9.19E-02	3.43E-01		-6.17E-01	1.52E-14		-1.12E+00	2.33E-50	Down	-8.64E-01	5.50E-34		Unigene11093_All//gi|42570286|ref|NP_849997.2|//0.00E+00//CLASP (CLIP-ASSOCIATED PROTEIN); binding [Arabidopsis thaliana]	Unigene11093_All//0.00E+00//AT2G20190| CLASP (CLIP-ASSOCIATED PROTEIN); binding	Unigene11093_All//0.00E+00//TC176731			GO:0000087//GO:0005819//GO:0016020//GO:0031647//GO:0040007	nr -
Unigene110931_All	255	3.28	2.78	1.25	2.93	4.05	10.45	3.15	2.62	3.12	-2.39E-01	7.25E-01		-1.39E+00	9.66E-02		4.67E-01	5.51E-01		1.83E+00	1.12E-05	Up	-2.65E-01	7.05E-01		-1.18E-02	9.78E-01				Unigene110931_All//8.00E-14//DT463109				
Unigene110932_All	622	0.00	0.00	0.00	0.00	0.00	0.00	1.09	0.94	0.00	null	null		null	null		null	null		null	null		-2.09E-01	7.82E-01		-1.01E+01	3.57E-05	Down							ESTscan -
Unigene110934_All	378	0.00	0.00	0.00	0.00	0.00	0.00	2.91	0.33	0.74	null	null		null	null		null	null		null	null		-3.13E+00	3.47E-05	Down	-1.98E+00	1.39E-03		Unigene110934_All//gi|110430668|gb|ABG73458.1|//7.00E-20//AP2 domain-containing protein [Oryza brachyantha]	Unigene110934_All//2.00E-18//AT5G19790| RAP2.11 (related to AP2 11); DNA binding / transcription factor		AP2-EREBP			nr +
Unigene110937_All	379	0.00	0.00	0.00	0.00	0.18	0.48	4.46	0.77	0.95	null	null		null	null		7.51E+00	6.14E-01		8.90E+00	1.95E-01		-2.53E+00	2.71E-06	Down	-2.24E+00	8.65E-06	Down			Unigene110937_All//2.00E-68//DW515284				
Unigene110948_All	405	0.00	0.00	0.00	0.00	0.00	0.00	2.82	0.93	0.10	null	null		null	null		null	null		null	null		-1.60E+00	7.51E-03		-4.84E+00	2.34E-07	Down	Unigene110948_All//gi|2780194|emb|CAA05979.1|//2.00E-15//adenine nucleotide translocator [Lupinus albus]	Unigene110948_All//2.00E-16//AT5G13490| AAC2 (ADP/ATP carrier 2); ATP:ADP antiporter/ binding					nr +
Unigene11095_All	841	7.90	9.22	10.64	12.26	14.13	8.32	16.44	11.53	7.15	2.22E-01	3.28E-01		4.29E-01	3.57E-02		2.04E-01	2.70E-01		-5.59E-01	1.41E-03		-5.12E-01	1.40E-04		-1.20E+00	1.30E-17	Down	Unigene11095_All//gi|49523817|emb|CAF18249.1|//1.00E-148//SEU3A protein [Antirrhinum majus]	Unigene11095_All//8.00E-148//AT1G43850| SEU (seuss); DNA binding / protein binding / protein heterodimerization/ transcription cofactor	Unigene11095_All//0.00E+00//gi|84148586|gb|DN779886.1|DN779886				nr +
Unigene110952_All	294	0.00	2.24	0.18	0.85	1.29	0.77	0.43	0.14	0.00	1.11E+01	6.26E-04	Up	7.50E+00	6.40E-01		6.05E-01	6.59E-01		-1.41E-01	9.29E-01		-1.60E+00	5.35E-01		-8.75E+00	2.00E-01		Unigene110952_All//gi|15219557|ref|NP_171875.1|//1.00E-06//SOM (SOMNUS); nucleic acid binding / transcription factor [Arabidopsis thaliana] >gi|75268098|sp|Q9ZWA1.1|C3H2_ARATH RecName: Full=Zinc finger CCCH domain-containing protein 2; Short=AtC3H2; AltName: Full=Protein SOMNUS; Short=SOM >gi|4204308|gb|AAD10689.1| Hypothetical protein [Arabidopsis thaliana]	Unigene110952_All//6.00E-06//AT5G44260| zinc finger (CCCH-type) family protein	Unigene110952_All//3.00E-72//TC129629	C3H			nr +
Unigene110953_All	822	0.00	0.00	0.00	0.00	0.00	0.00	2.78	1.17	0.44	null	null		null	null		null	null		null	null		-1.25E+00	1.20E-03		-2.67E+00	4.62E-09	Down	Unigene110953_All//gi|1706740|sp|P52837.1|F4ST_FLACH//4.00E-50//RecName: Full=Flavonol 4'-sulfotransferase; Short=F4-ST >gi|168169|gb|AAA33343.1| flavonol 4'-sulfotransferase [Flaveria chloraefolia]	Unigene110953_All//1.00E-50//AT1G18590| SOT17 (SULFOTRANSFERASE 17); desulfoglucosinolate sulfotransferase/ sulfotransferase	Unigene110953_All//4.00E-12//EX167975		ko00380|Tryptophan metabolism//ko00966|Glucosinolate biosynthesis		nr -
Unigene110955_All	371	0.00	0.00	0.00	0.00	0.00	0.00	2.39	0.79	0.11	null	null		null	null		null	null		null	null		-1.60E+00	2.18E-02		-4.48E+00	1.23E-05	Down							ESTscan +
Unigene110956_All	335	0.31	0.30	0.16	0.00	1.03	0.67	1.89	0.12	0.36	-4.65E-02	9.91E-01		-9.74E-01	7.22E-01		1.00E+01	3.02E-02		9.40E+00	1.19E-01		-3.92E+00	9.81E-04	Down	-2.41E+00	8.12E-03				Unigene110956_All//2.00E-08//DR461672				
Unigene110957_All	375	0.00	0.00	0.00	0.00	0.00	0.00	5.07	0.00	0.00	null	null		null	null		null	null		null	null		-1.23E+01	1.81E-13	Down	-1.23E+01	4.91E-14	Down							ESTscan +
Unigene110958_All	218	0.00	0.00	0.00	0.91	0.00	0.00	2.72	0.00	0.00	null	null		null	null		-9.84E+00	8.47E-02		-9.84E+00	1.34E-01		-1.14E+01	2.39E-04	Down	-1.14E+01	1.41E-04	Down	Unigene110958_All//gi|297607012|ref|NP_001059355.2|//9.00E-07//Os07g0273700 [Oryza sativa Japonica Group] >gi|255677660|dbj|BAF21269.2| Os07g0273700 [Oryza sativa Japonica Group]		Unigene110958_All//2.00E-11//TC168228				nr -
Unigene110959_All	296	0.00	0.00	0.00	0.00	0.00	0.00	1.71	0.00	0.00	null	null		null	null		null	null		null	null		-1.07E+01	8.90E-04	Down	-1.07E+01	5.55E-04	Down	Unigene110959_All//gi|116056464|emb|CAL52753.1|//1.00E-21//ribosomal protein S20 (ISS) [Ostreococcus tauri]	Unigene110959_All//3.00E-21//AT5G62300| 40S ribosomal protein S20 (RPS20C)			ko03010|Ribosome		nr -
Unigene110962_All	255	0.00	0.00	0.00	0.00	0.00	0.00	6.13	1.48	1.56	null	null		null	null		null	null		null	null		-2.06E+00	9.44E-05	Down	-1.97E+00	9.58E-05	Down			Unigene110962_All//3.00E-10//TC167637				
Unigene110965_All	215	0.97	0.00	0.00	2.78	3.68	2.94	3.34	0.97	0.37	-9.93E+00	1.23E-01		-9.93E+00	1.46E-01		4.06E-01	6.32E-01		8.10E-02	9.45E-01		-1.78E+00	2.79E-02		-3.17E+00	8.09E-04	Down	Unigene110965_All//gi|224152830|ref|XP_002337280.1|//7.00E-12//BED finger-nbs-lrr resistance protein [Populus trichocarpa] >gi|222838678|gb|EEE77043.1| BED finger-nbs-lrr resistance protein [Populus trichocarpa]	Unigene110965_All//5.00E-10//AT4G27220| disease resistance protein (NBS-LRR class), putative	Unigene110965_All//4.00E-92//DT463318		ko04626|Plant-pathogen interaction	GO:0005488	nr +
Unigene110967_All	468	0.00	0.22	1.02	0.00	0.00	0.19	1.63	0.36	0.00	7.76E+00	3.90E-01		1.00E+01	7.49E-03		null	null		7.59E+00	4.80E-01		-2.19E+00	7.78E-03		-1.07E+01	8.88E-06	Down			Unigene110967_All//8.00E-145//ES806643				
Unigene110969_All	481	0.00	0.00	0.00	0.00	0.00	0.00	1.14	0.70	0.00	null	null		null	null		null	null		null	null		-7.17E-01	4.22E-01		-1.02E+01	2.81E-04	Down	Unigene110969_All//gi|40287526|gb|AAR83877.1|//6.00E-33//60S ribosomal protein L19 [Capsicum annuum]	Unigene110969_All//7.00E-26//AT3G16780| 60S ribosomal protein L19 (RPL19B)	Unigene110969_All//1.00E-07//TC132886		ko03010|Ribosome	GO:0043231	nr -
Unigene110972_All	672	0.08	0.00	0.16	0.15	0.15	0.07	10.76	0.87	1.84	-6.28E+00	6.18E-01		1.02E+00	7.04E-01		5.16E-02	9.68E-01		-1.14E+00	6.78E-01		-3.63E+00	5.99E-35	Down	-2.55E+00	9.72E-26	Down	Unigene110972_All//gi|109900626|gb|ABG47806.1|//1.00E-42//pectin methlyesterase inhibitor protein 1 [Capsicum annuum] >gi|110631514|gb|ABG81102.1| pectin methlyesterase inhibitor protein 1 [Capsicum annuum]	Unigene110972_All//2.00E-36//AT5G62360| invertase/pectin methylesterase inhibitor family protein	Unigene110972_All//1.00E-14//DW502487		ko00500|Starch and sucrose metabolism//ko00040|Pentose and glucuronate interconversions		nr -
Unigene110974_All	516	0.00	0.00	0.00	0.00	0.00	0.00	2.54	0.08	0.00	null	null		null	null		null	null		null	null		-4.97E+00	4.05E-08	Down	-1.13E+01	9.85E-10	Down	Unigene110974_All//gi|301095349|ref|XP_002896775.1|//1.00E-34//60S acidic ribosomal protein P0 [Phytophthora infestans T30-4] >gi|262108658|gb|EEY66710.1| 60S acidic ribosomal protein P0 [Phytophthora infestans T30-4]	Unigene110974_All//8.00E-31//AT2G40010| 60S acidic ribosomal protein P0 (RPP0A)			ko03010|Ribosome	GO:0005198//GO:0006412//GO:0030529	nr -
Unigene110976_All	405	0.00	0.00	0.00	0.00	0.00	0.00	3.55	0.10	0.00	null	null		null	null		null	null		null	null		-5.10E+00	5.72E-09	Down	-1.18E+01	1.19E-10	Down	Unigene110976_All//gi|6094086|sp|O65353.1|RL5_HELAN//2.00E-40//RecName: Full=60S ribosomal protein L5 >gi|3153904|gb|AAC17448.1| RPL5A-related protein [Helianthus annuus]	Unigene110976_All//1.00E-17//AT3G25520| ATL5 (A. THALIANA RIBOSOMAL PROTEIN L5); 5S rRNA binding / structural constituent of ribosome			ko03010|Ribosome	GO:0003002//GO:0005198//GO:0009536//GO:0009887//GO:0010467//GO:0015934//GO:0016020//GO:0019843//GO:0022613//GO:0031981//GO:0046914//GO:0051169//GO:0065003	nr -
Unigene110977_All	462	0.00	0.00	0.00	0.00	0.00	0.00	2.29	12.57	0.00	null	null		null	null		null	null		null	null		2.46E+00	2.75E-19	Up	-1.12E+01	6.72E-08	Down							
Unigene110978_All	707	0.00	0.00	0.00	0.00	0.00	0.00	2.81	0.77	0.11	null	null		null	null		null	null		null	null		-1.87E+00	2.98E-05	Down	-4.64E+00	3.57E-12	Down	Unigene110978_All//gi|159471131|ref|XP_001693710.1|//4.00E-78//ribosomal protein S3a, component of cytosolic 80S ribosome and 40S small subunit [Chlamydomonas reinhardtii] >gi|269969596|sp|A8HS48.1|RS3A_CHLRE RecName: Full=40S ribosomal protein S3a >gi|158283213|gb|EDP08964.1| ribosomal protein S3a [Chlamydomonas reinhardtii]	Unigene110978_All//2.00E-63//AT4G34670| 40S ribosomal protein S3A (RPS3aB)			ko03010|Ribosome	GO:0005198//GO:0010467//GO:0015935//GO:0016020//GO:0030312//GO:0031981	nr -
Unigene110980_All	1651	0.00	0.00	0.00	0.06	0.00	0.00	4.00	1.16	1.11	null	null		null	null		-5.91E+00	3.16E-01		-5.91E+00	4.04E-01		-1.78E+00	1.01E-14	Down	-1.85E+00	5.42E-16	Down	Unigene110980_All//gi|156447779|gb|ABU63668.1|//1.00E-95//CYCLOPS [Lotus japonicus]						nr -
Unigene110981_All	411	0.00	0.00	0.00	0.00	0.00	0.00	2.67	0.71	0.19	null	null		null	null		null	null		null	null		-1.91E+00	2.64E-03		-3.79E+00	3.27E-06	Down							ESTscan +
Unigene110983_All	319	0.00	0.00	0.00	0.00	0.00	0.00	2.78	0.66	0.00	null	null		null	null		null	null		null	null		-2.09E+00	4.71E-03		-1.14E+01	1.10E-06	Down	Unigene110983_All//gi|247421835|gb|ACS96445.1|//1.00E-34//S18.A ribosomal protein [Jatropha curcas]	Unigene110983_All//8.00E-30//AT4G09800| RPS18C (S18 RIBOSOMAL PROTEIN); RNA binding / nucleic acid binding / structural constituent of ribosome			ko03010|Ribosome	GO:0003676//GO:0005198//GO:0006412//GO:0015935//GO:0016020//GO:0030312//GO:0031981	nr +

Unigene110985_All	272	18.85	54.77	24.47	11.72	13.17	25.74	10.88	22.28	10.11	1.54E+00	1.56E-21	Up	3.77E-01	1.28E-01		1.68E-01	6.54E-01		1.13E+00	3.58E-07	Up	1.03E+00	2.12E-06	Up	-1.07E-01	7.47E-01		Unigene110985_All//gi|224144535|ref|XP_002325324.1|//5.00E-10//cytochrome P450 [Populus trichocarpa] >gi|222862199|gb|EEE99705.1| cytochrome P450 [Populus trichocarpa]	Unigene110985_All//3.00E-05//AT5G23190| CYP86B1; electron carrier/ heme binding / iron ion binding / monooxygenase/ oxygen binding	Unigene110985_All//9.00E-60//TC133857			GO:0005506//GO:0016491	nr -
Unigene110986_All	1899	0.00	0.00	0.00	0.10	0.00	0.00	7.95	0.04	0.04	null	null		null	null		-6.71E+00	8.47E-02		-6.71E+00	1.34E-01		-7.50E+00	3.26E-103	Down	-7.56E+00	4.12E-107	Down	Unigene110986_All//gi|224119978|ref|XP_002331107.1|//1.00E-157//cytochrome P450 [Populus trichocarpa] >gi|222872835|gb|EEF09966.1| cytochrome P450 [Populus trichocarpa]	Unigene110986_All//5.00E-139//AT4G37310| CYP81H1; electron carrier/ heme binding / iron ion binding / monooxygenase/ oxygen binding	Unigene110986_All//6.00E-16//TC178809		ko00945|Stilbenoid, diarylheptanoid and gingerol biosynthesis//ko00903|Limonene and pinene degradation		nr -
Unigene110990_All	427	0.00	0.00	0.00	0.00	0.00	0.00	3.37	0.00	0.00	null	null		null	null		null	null		null	null		-1.17E+01	3.33E-10	Down	-1.17E+01	1.19E-10	Down	Unigene110990_All//sp|P60372|KR104_HUMAN//7.00E-07//Keratin-associated protein 10-4 OS=Homo sapiens GN=KRTAP10-4 PE=2 SV=1						swissprot +
Unigene110991_All	817	0.38	0.37	0.26	0.24	0.00	0.17	1.24	2.46	5.02	-4.62E-02	9.85E-01		-5.60E-01	6.64E-01		-7.93E+00	8.47E-02		-5.57E-01	7.33E-01		9.83E-01	1.63E-02		2.02E+00	2.88E-11	Up	Unigene110991_All//gi|223452359|gb|ACM89507.1|//5.00E-89//NAK-type protein kinase [Glycine max]	Unigene110991_All//4.00E-76//AT5G47070| protein kinase, putative	Unigene110991_All//1.00E-55//ES808920		ko04626|Plant-pathogen interaction	GO:0000166//GO:0004672//GO:0016020	nr +
Unigene110994_All	332	0.00	0.00	0.00	0.00	0.00	0.00	1.91	1.51	0.00	null	null		null	null		null	null		null	null		-3.39E-01	6.63E-01		-1.09E+01	7.12E-05	Down	Unigene110994_All//gi|115436514|ref|NP_001043015.1|//2.00E-28//Os01g0358400 [Oryza sativa Japonica Group] >gi|113532546|dbj|BAF04929.1| Os01g0358400 [Oryza sativa Japonica Group] >gi|149391309|gb|ABR25672.1| 40S ribosomal protein S4 [Oryza sativa Indica Group]	Unigene110994_All//2.00E-28//AT5G58420| 40S ribosomal protein S4 (RPS4D)			ko03010|Ribosome	GO:0003723//GO:0005198//GO:0010467//GO:0030529	nr -
Unigene110995_All	497	0.00	0.00	0.00	0.00	0.00	0.00	4.59	0.00	0.64	null	null		null	null		null	null		null	null		-1.22E+01	3.78E-16	Down	-2.84E+00	1.28E-09	Down							
Unigene110996_All	439	0.00	0.00	0.00	0.00	0.00	0.00	2.70	0.38	0.09	null	null		null	null		null	null		null	null		-2.82E+00	4.41E-05	Down	-4.89E+00	1.20E-07	Down	Unigene110996_All//gi|15809990|gb|AAL06922.1|//3.00E-10//AT5g60530/muf9_180 [Arabidopsis thaliana]				ko03040|Spliceosome		nr -
Unigene110997_All	339	0.00	0.00	0.00	0.00	0.00	0.00	2.99	0.49	0.24	null	null		null	null		null	null		null	null		-2.60E+00	3.81E-04	Down	-3.67E+00	1.15E-05	Down	Unigene110997_All//gi|255103522|gb|ACH70610.1|//5.00E-49//alpha tubulin [Ulva fasciata]	Unigene110997_All//2.00E-49//AT1G50010| TUA2; structural constituent of cytoskeleton	Unigene110997_All//7.00E-12//gi|164334548|gb|EV483418.1|EV483418		ko04145|Phagosome	GO:0007017//GO:0015630//GO:0017111//GO:0032561//GO:0032991//GO:0043623	nr +
Unigene111_All	12603	1.17	5.72	2.68	2.80	4.02	4.35	8.92	22.25	23.52	2.28E+00	4.63E-173	Up	1.19E+00	1.07E-31	Up	5.20E-01	2.96E-14		6.35E-01	7.90E-20		1.32E+00	0.00E+00	Up	1.40E+00	0.00E+00	Up	Unigene111_All//gi|225432116|ref|XP_002274451.1|//0.00E+00//PREDICTED: similar to vacuolar protein sorting 13C protein-like [Vitis vinifera]	Unigene111_All//0.00E+00//AT4G17140| FUNCTIONS IN: molecular_function unknown; INVOLVED IN: biological_process unknown; LOCATED IN: vacuole; EXPRESSED IN: 24 plant structures; EXPRESSED DURING: 13 growth stages; CONTAINS InterPro DOMAIN/s: Pleckstrin homology (InterPro:IPR001849); BEST Arabidopsis thaliana protein match is: phosphoinositide binding (TAIR:AT1G48090.2); Has 1924 Blast hits to 1182 proteins in 161 species: Archae - 0; Bacteria - 16; Metazoa - 911; Fungi - 304; Plants - 271; Viruses - 0; Other Eukaryotes - 422 (source: NCBI BLink).	Unigene111_All//0.00E+00//TC169119				nr +
Unigene1110_All	1325	5.05	2.49	3.46	4.77	6.19	8.15	19.82	21.45	21.68	-1.02E+00	9.20E-06	Down	-5.48E-01	2.14E-02		3.74E-01	7.04E-02		7.71E-01	6.02E-06		1.14E-01	2.78E-01		1.30E-01	1.86E-01		Unigene1110_All//gi|15220976|ref|NP_173253.1|//0.00E+00//ATMPK8; MAP kinase [Arabidopsis thaliana] >gi|30685711|ref|NP_849685.1| ATMPK8; MAP kinase [Arabidopsis thaliana] >gi|110287680|sp|Q9LM33.2|MPK8_ARATH RecName: Full=Mitogen-activated protein kinase 8; Short=MAP kinase 8; Short=AtMPK8 >gi|9719729|gb|AAF97831.1|AC034107_14 Strong similarity (practically identical) to ATMPK8 gene from Arabidopsis thaliana gb|AB038693 and contains a eukaryotic protein kinase PF|00069 domain. ESTs gb|AV526779, gb|AV527934, gb|AV540522, gb|T22988, gb|R90476, gb|Z24497, gb|N97150, gb|AA713291, gb|AI100188 come from this gene	Unigene1110_All//4.00E-165//AT1G18150| ATMPK8; MAP kinase	Unigene1110_All//0.00E+00//TC176812		ko04626|Plant-pathogen interaction//ko04650|Natural killer cell mediated cytotoxicity	GO:0004702//GO:0006464//GO:0016020//GO:0023060//GO:0032559	nr -
Unigene11100_All	370	0.00	0.00	0.00	0.00	0.00	0.00	0.23	0.68	2.05	null	null		null	null		null	null		null	null		1.57E+00	3.27E-01		3.16E+00	6.83E-04	Up	Unigene11100_All//gi|297722553|ref|NP_001173640.1|//2.00E-27//Os03g0744650 [Oryza sativa Japonica Group] >gi|255674888|dbj|BAH92368.1| Os03g0744650 [Oryza sativa Japonica Group]	Unigene11100_All//1.00E-28//AT1G03060| WD-40 repeat family protein / beige-related	Unigene11100_All//5.00E-93//TC145957			GO:0046914	nr -
Unigene111000_All	336	0.00	0.00	0.00	0.00	0.00	0.00	2.14	0.00	0.24	null	null		null	null		null	null		null	null		-1.11E+01	3.27E-05	Down	-3.17E+00	8.09E-04	Down							ESTscan -
Unigene111002_All	305	0.00	0.00	0.00	0.00	0.00	0.00	3.60	0.69	0.26	null	null		null	null		null	null		null	null		-2.40E+00	4.11E-04	Down	-3.79E+00	3.27E-06	Down	Unigene111002_All//gi|159476204|ref|XP_001696201.1|//3.00E-18//ribosomal protein S8, component of cytosolic 80S ribosome and 40S small subunit [Chlamydomonas reinhardtii] >gi|158282426|gb|EDP08178.1| ribosomal protein S8 [Chlamydomonas reinhardtii]	Unigene111002_All//8.00E-17//AT5G59240| 40S ribosomal protein S8 (RPS8B)			ko03010|Ribosome	GO:0030529//GO:0043229//GO:0044444	nr -
Unigene111011_All	405	0.00	0.00	0.00	0.00	0.00	0.00	8.14	2.27	0.49	null	null		null	null		null	null		null	null		-1.84E+00	4.43E-08	Down	-4.05E+00	2.95E-18	Down	Unigene111011_All//gi|238014838|gb|ACR38454.1|//1.00E-21//unknown [Zea mays]		Unigene111011_All//0.00E+00//TC146753				nr -
Unigene111013_All	666	0.00	0.00	0.00	0.00	0.00	0.00	1.71	0.44	0.00	null	null		null	null		null	null		null	null		-1.96E+00	1.69E-03		-1.07E+01	1.65E-08	Down	Unigene111013_All//sp|Q58A42|DD3_DICDI//9.00E-29//Protein DD3-3 OS=Dictyostelium discoideum GN=DD3-3 PE=2 SV=1						swissprot +
Unigene111014_All	370	0.00	0.00	0.00	0.13	0.00	0.00	3.43	0.90	1.29	null	null		null	null		-7.07E+00	5.62E-01		-7.07E+00	6.43E-01		-1.92E+00	9.98E-04	Down	-1.41E+00	8.06E-03				Unigene111014_All//1.00E-133//TC138525				
Unigene111015_All	435	0.00	0.00	0.00	0.00	0.00	0.00	4.18	0.77	0.64	null	null		null	null		null	null		null	null		-2.44E+00	1.77E-06	Down	-2.70E+00	1.86E-07	Down	Unigene111015_All//gi|297838233|ref|XP_002886998.1|//7.00E-07//expressed protein [Arabidopsis lyrata subsp. lyrata] >gi|297332839|gb|EFH63257.1| expressed protein [Arabidopsis lyrata subsp. lyrata]				ko03040|Spliceosome	GO:0005488	nr +
Unigene111022_All	272	0.00	0.37	0.20	1.28	0.76	0.50	3.89	1.23	0.88	8.54E+00	3.90E-01		7.61E+00	6.40E-01		-7.55E-01	5.67E-01		-1.36E+00	3.37E-01		-1.66E+00	8.55E-03		-2.14E+00	9.36E-04	Down							
Unigene111024_All	487	0.00	0.00	0.00	0.00	0.00	0.00	2.43	0.26	0.16	null	null		null	null		null	null		null	null		-3.24E+00	1.10E-05	Down	-3.89E+00	9.21E-07	Down							
Unigene111026_All	352	0.00	0.00	0.00	0.00	0.00	0.00	3.00	0.95	0.45	null	null		null	null		null	null		null	null		-1.66E+00	8.55E-03		-2.73E+00	1.12E-04	Down							
Unigene111028_All	543	0.00	0.00	0.00	0.00	0.00	0.00	5.61	0.00	0.07	null	null		null	null		null	null		null	null		-1.25E+01	1.58E-21	Down	-6.26E+00	7.97E-21	Down							
Unigene111029_All	583	0.00	0.00	0.00	0.00	0.00	0.00	2.83	0.57	2.32	null	null		null	null		null	null		null	null		-2.30E+00	1.36E-05	Down	-2.84E-01	5.50E-01								
Unigene111030_All	296	39.42	85.93	53.25	143.89	255.71	234.06	58.42	120.59	63.80	1.12E+00	1.18E-22	Up	4.34E-01	3.09E-03		8.30E-01	1.67E-47		7.02E-01	1.18E-29		1.05E+00	2.30E-34	Up	1.27E-01	3.12E-01		Unigene111030_All//gi|148645857|gb|ABR01221.1|//2.00E-30//MYB8 [Gossypium hirsutum]	Unigene111030_All//7.00E-30//AT4G38620| MYB4; DNA binding / transcription factor	Unigene111030_All//2.00E-91//TC167380	MYB		GO:0001071//GO:0003676//GO:0006350//GO:0009411//GO:0009719//GO:0010033//GO:0010468//GO:0043231	nr +
Unigene111033_All	373	0.00	0.00	0.00	0.00	0.00	0.00	4.31	0.34	0.21	null	null		null	null		null	null		null	null		-3.68E+00	2.83E-08	Down	-4.33E+00	1.41E-09	Down	Unigene111033_All//gi|238632211|gb|ACR50977.1|//1.00E-31//60s ribosomal protein L21 [Lycoris radiata]	Unigene111033_All//9.00E-31//AT1G09690| 60S ribosomal protein L21 (RPL21C)	Unigene111033_All//3.00E-08//TC169130		ko03010|Ribosome	GO:0005198//GO:0009536//GO:0010467//GO:0015934//GO:0031981	nr +
Unigene111038_All	811	0.00	0.37	0.00	0.00	0.00	0.00	4.59	0.41	0.69	8.55E+00	4.45E-02		null	null		null	null		null	null		-3.48E+00	1.24E-17	Down	-2.74E+00	1.16E-14	Down	Unigene111038_All//gi|297825707|ref|XP_002880736.1|//1.00E-53//bet v I allergen family protein [Arabidopsis lyrata subsp. lyrata] >gi|297326575|gb|EFH56995.1| bet v I allergen family protein [Arabidopsis lyrata subsp. lyrata]	Unigene111038_All//2.00E-54//AT2G26040| Bet v I allergen family protein				GO:0004871//GO:0009755//GO:0042802	nr +
Unigene111040_All	406	0.00	0.00	0.00	0.00	0.00	0.00	4.69	0.31	0.49	null	null		null	null		null	null		null	null		-3.92E+00	3.80E-10	Down	-3.26E+00	3.59E-09	Down	Unigene111040_All//gi|238617665|gb|ACR46954.1|//6.00E-69//alpha-tubulin [Rhodochaete parvula]	Unigene111040_All//1.00E-67//AT4G14960| TUA6; structural constituent of cytoskeleton	Unigene111040_All//1.00E-47//gi|11208953|gb|BF277883.1|BF277883		ko04145|Phagosome	GO:0007017//GO:0015630//GO:0017111//GO:0032561//GO:0032991//GO:0043623	nr -
Unigene111042_All	278	0.00	0.00	0.00	0.00	0.00	0.00	2.28	0.15	0.00	null	null		null	null		null	null		null	null		-3.92E+00	9.81E-04	Down	-1.12E+01	7.12E-05	Down	Unigene111042_All//gi|124365534|gb|ABN09768.1|//1.00E-07//Cyclin-like F-box; Calcium-binding EF-hand [Medicago truncatula]					GO:0005488	nr +
Unigene111045_All	471	0.56	0.00	0.23	0.21	0.15	0.00	2.87	0.00	0.00	-9.12E+00	6.72E-02		-1.30E+00	4.37E-01		-5.33E-01	8.19E-01		-7.72E+00	4.04E-01		-1.15E+01	1.30E-09	Down	-1.15E+01	4.86E-10	Down	Unigene111045_All//gi|224094135|ref|XP_002310080.1|//2.00E-60//glycosyl hydrolase family 9 [Populus trichocarpa] >gi|222852983|gb|EEE90530.1| glycosyl hydrolase family 9 [Populus trichocarpa]	Unigene111045_All//2.00E-59//AT1G48930| AtGH9C1 (Arabidopsis thaliana glycosyl hydrolase 9C1); catalytic/ hydrolase, hydrolyzing O-glycosyl compounds	Unigene111045_All//0.00E+00//ES790572		ko00500|Starch and sucrose metabolism	GO:0004553//GO:0005488//GO:0031410//GO:0044238	nr +
Unigene111048_All	430	0.00	0.00	0.00	0.00	0.00	0.00	2.75	0.19	0.09	null	null		null	null		null	null		null	null		-3.82E+00	2.12E-06	Down	-4.89E+00	1.20E-07	Down	Unigene111048_All//gi|11131946|sp|Q9ZNY3.1|CALR_EUGGR//3.00E-06//RecName: Full=Calreticulin; Flags: Precursor >gi|3970687|emb|CAA70945.1| calreticulin precursor [Euglena gracilis]				ko04141|Protein processing in endoplasmic reticulum//ko04145|Phagosome//ko00010|Glycolysis / Gluconeogenesis	GO:0005488//GO:0005622	nr +
Unigene111053_All	483	0.22	0.42	0.00	0.52	0.29	0.19	1.93	0.95	0.33	9.54E-01	6.22E-01		-7.76E+00	3.98E-01		-8.55E-01	5.74E-01		-1.46E+00	4.07E-01		-1.02E+00	1.21E-01		-2.55E+00	5.79E-04	Down			Unigene111053_All//1.00E-10//ES839853				
Unigene111055_All	679	0.00	0.00	0.00	0.00	0.00	0.00	3.36	0.43	0.41	null	null		null	null		null	null		null	null		-2.96E+00	1.24E-09	Down	-3.03E+00	3.22E-10	Down			Unigene111055_All//2.00E-35//CF932145				
Unigene111056_All	362	0.00	0.00	0.00	0.00	0.00	0.00	4.09	0.23	0.55	null	null		null	null		null	null		null	null		-4.15E+00	2.75E-08	Down	-2.89E+00	1.35E-06	Down	Unigene111056_All//gi|226459877|gb|EEH57172.1|//3.00E-18//ribosomal protein [Micromonas pusilla CCMP1545]	Unigene111056_All//2.00E-06//AT5G56670| 40S ribosomal protein S30 (RPS30C)			ko03010|Ribosome	GO:0005198//GO:0010467//GO:0015935//GO:0031981	nr -
Unigene111060_All	617	0.00	0.00	0.00	0.00	0.00	0.00	1.71	0.07	0.00	null	null		null	null		null	null		null	null		-4.66E+00	1.94E-06	Down	-1.07E+01	6.72E-08	Down	Unigene111060_All//gi|70663983|emb|CAE04677.2|//3.00E-06//OSJNBb0018A10.6 [Oryza sativa (japonica cultivar-group)]					GO:0005488	nr -
Unigene111063_All	359	0.00	0.00	0.00	0.00	0.00	0.00	8.01	4.42	3.22	null	null		null	null		null	null		null	null		-8.56E-01	9.39E-03		-1.32E+00	6.99E-05	Down			Unigene111063_All//3.00E-23//TC142576				
Unigene111064_All	528	0.00	0.00	0.00	0.00	0.00	0.00	2.96	0.08	0.00	null	null		null	null		null	null		null	null		-5.23E+00	8.04E-10	Down	-1.15E+01	1.42E-11	Down	Unigene111064_All//gi|195605794|gb|ACG24727.1|//6.00E-30//40S ribosomal protein S26 [Zea mays] >gi|195610710|gb|ACG27185.1| 40S ribosomal protein S26 [Zea mays]	Unigene111064_All//1.00E-24//AT3G56340| 40S ribosomal protein S26 (RPS26C)			ko03010|Ribosome	GO:0005198//GO:0010467//GO:0030529	nr +
Unigene111067_All	364	0.29	0.14	0.00	0.55	0.57	0.12	2.09	0.11	0.11	-1.05E+00	6.85E-01		-8.17E+00	3.98E-01		5.23E-02	9.89E-01		-2.14E+00	3.48E-01		-4.19E+00	1.58E-04	Down	-4.26E+00	8.66E-05	Down							
Unigene111068_All	238	0.00	0.00	0.00	0.00	0.00	0.00	3.55	1.05	0.00	null	null		null	null		null	null		null	null		-1.75E+00	1.64E-02		-1.18E+01	2.21E-06	Down			Unigene111068_All//5.00E-15//gi|109848340|gb|DW477320.1|DW477320				

Unigene11107_All	990	2.11	0.72	0.91	1.56	2.09	3.01	3.25	2.07	1.69	-1.56E+00	8.94E-04	Down	-1.21E+00	1.09E-02		4.20E-01	3.93E-01		9.49E-01	9.14E-03		-6.50E-01	3.63E-02		-9.42E-01	1.69E-03		Unigene11107_All//gi|297838455|ref|XP_002887109.1|//1.00E-134//F-box family protein [Arabidopsis lyrata subsp. lyrata] >gi|297332950|gb|EFH63368.1| F-box family protein [Arabidopsis lyrata subsp. lyrata]	Unigene11107_All//7.00E-135//AT1G67190| F-box family protein	Unigene11107_All//0.00E+00//TC178032				nr +
Unigene111070_All	476	0.00	0.53	0.00	0.00	0.07	1.14	52.32	128.72	91.06	9.06E+00	7.88E-02		null	null		6.18E+00	8.37E-01		1.02E+01	2.46E-03		1.30E+00	2.93E-82	Up	8.00E-01	1.97E-27		Unigene111070_All//gi|603890|emb|CAA87073.1|//8.00E-17//pathogenesis-related protein PR-6 type [Sambucus nigra]	Unigene111070_All//2.00E-16//AT2G38870| protease inhibitor, putative				GO:0004866//GO:0006952//GO:0030312	nr -
Unigene111073_All	342	0.31	0.00	0.47	1.02	0.30	0.66	4.45	0.49	0.82	-8.26E+00	3.76E-01		6.10E-01	7.49E-01		-1.75E+00	1.90E-01		-6.27E-01	6.21E-01		-3.19E+00	4.80E-07	Down	-2.45E+00	8.71E-06	Down							
Unigene111074_All	305	0.00	0.00	0.00	0.00	0.00	0.00	2.63	0.00	0.39	null	null		null	null		null	null		null	null		-1.14E+01	8.58E-06	Down	-2.75E+00	9.43E-04	Down	Unigene111074_All//gi|300265001|gb|EFJ49194.1|//1.00E-11//molecular chaperone [Volvox carteri f. nagariensis]	Unigene111074_All//1.00E-10//AT3G44110| ATJ3; protein binding			ko04141|Protein processing in endoplasmic reticulum	GO:0005488	nr -
Unigene111077_All	238	0.88	0.00	0.00	1.26	0.29	1.14	2.84	0.18	0.33	-9.78E+00	1.23E-01		-9.78E+00	1.46E-01		-2.12E+00	1.66E-01		-1.41E-01	9.21E-01		-4.02E+00	5.36E-04	Down	-3.09E+00	1.45E-03		Unigene111077_All//gi|224058355|ref|XP_002299486.1|//1.00E-11//high mobility group family [Populus trichocarpa] >gi|222846744|gb|EEE84291.1| high mobility group family [Populus trichocarpa]		Unigene111077_All//7.00E-42//TC163360				nr +
Unigene111079_All	380	0.00	0.00	0.00	0.00	0.00	0.00	2.11	0.11	0.00	null	null		null	null		null	null		null	null		-4.26E+00	8.48E-05	Down	-1.10E+01	4.43E-06	Down	Unigene111079_All//gi|301112246|ref|XP_002905202.1|//7.00E-10//60S ribosomal protein L7 [Phytophthora infestans T30-4] >gi|262095532|gb|EEY53584.1| 60S ribosomal protein L7 [Phytophthora infestans T30-4]	Unigene111079_All//2.00E-07//AT2G01250| 60S ribosomal protein L7 (RPL7B)			ko03010|Ribosome	GO:0005488//GO:0009536//GO:0015934//GO:0016020//GO:0030312	nr +
Unigene111081_All	345	0.00	0.00	0.00	0.87	0.30	0.13	2.70	1.21	0.35	null	null		null	null		-1.53E+00	2.98E-01		-2.73E+00	1.37E-01		-1.15E+00	7.87E-02		-2.96E+00	1.72E-04	Down			Unigene111081_All//2.00E-24//TC178046				
Unigene111082_All	439	0.00	0.00	0.00	0.00	0.00	0.00	3.18	0.29	0.36	null	null		null	null		null	null		null	null		-3.48E+00	5.80E-07	Down	-3.13E+00	1.09E-06	Down	Unigene111082_All//gi|148646851|gb|ABR01233.1|//3.00E-44//60S ribosomal protein L10a [Chlorella vulgaris] >gi|148646859|gb|ABR01237.1| 60S ribosomal protein L10a [Chlorella vulgaris] >gi|148646863|gb|ABR01239.1| 60S ribosomal protein L10a [Chlorella vulgaris]	Unigene111082_All//6.00E-35//AT5G22440| 60S ribosomal protein L10A (RPL10aC)			ko03010|Ribosome	GO:0003676//GO:0005198//GO:0010467//GO:0015934//GO:0016020//GO:0031981	nr +
Unigene111085_All	1146	0.00	0.00	0.00	0.00	0.00	0.00	3.03	1.20	0.10	null	null		null	null		null	null		null	null		-1.33E+00	1.42E-05	Down	-4.86E+00	2.19E-21	Down	Unigene111085_All//gi|301111776|ref|XP_002904967.1|//1.00E-103//60S ribosomal protein L4 [Phytophthora infestans T30-4] >gi|262095297|gb|EEY53349.1| 60S ribosomal protein L4 [Phytophthora infestans T30-4]	Unigene111085_All//8.00E-95//AT3G09630| 60S ribosomal protein L4/L1 (RPL4A)	Unigene111085_All//2.00E-17//BQ405438		ko03010|Ribosome		nr -
Unigene111086_All	267	0.00	0.00	0.00	0.00	0.00	0.00	4.43	0.31	0.15	null	null		null	null		null	null		null	null		-3.82E+00	2.12E-06	Down	-4.89E+00	1.20E-07	Down	Unigene111086_All//gi|116222111|gb|ABJ80926.1|//2.00E-48//actin [Synura sphagnicola]	Unigene111086_All//4.00E-47//AT5G09810| ACT7 (ACTIN 7); structural constituent of cytoskeleton	Unigene111086_All//3.00E-47//TC135405		ko04145|Phagosome	GO:0032559//GO:0043232	nr +
Unigene111089_All	859	0.79	0.41	0.43	0.23	0.52	0.89	2.90	0.39	0.93	-9.39E-01	2.77E-01		-8.68E-01	3.51E-01		1.17E+00	3.47E-01		1.95E+00	3.23E-02		-2.90E+00	2.98E-10	Down	-1.65E+00	1.01E-05	Down	Unigene111089_All//gi|283362114|dbj|BAI65910.1|//1.00E-74//UDP-sugar:glycosyltransferase [Forsythia x intermedia]	Unigene111089_All//6.00E-60//AT3G16520| UDP-glucoronosyl/UDP-glucosyl transferase family protein	Unigene111089_All//0.00E+00//TC133049		ko00940|Phenylpropanoid biosynthesis	GO:0035251	nr -
Unigene11109_All	2523	8.09	8.52	8.23	23.69	23.64	20.26	20.44	14.58	9.38	7.43E-02	6.05E-01		2.54E-02	8.56E-01		-3.30E-03	9.63E-01		-2.26E-01	9.50E-04		-4.88E-01	1.17E-13		-1.12E+00	4.83E-57	Down	Unigene11109_All//gi|22329659|ref|NP_173305.2|//0.00E+00//DNAJ heat shock N-terminal domain-containing protein [Arabidopsis thaliana] >gi|17979007|gb|AAL47463.1| At1g18700/F6A14_19 [Arabidopsis thaliana] >gi|23506111|gb|AAN28915.1| At1g18700/F6A14_19 [Arabidopsis thaliana]	Unigene11109_All//0.00E+00//AT1G18700| DNAJ heat shock N-terminal domain-containing protein	Unigene11109_All//0.00E+00//TC153985			GO:0005515//GO:0043231//GO:0050896	nr -
Unigene111092_All	241	1.30	0.42	2.21	1.03	2.57	1.31	6.32	1.91	3.14	-1.63E+00	2.77E-01		7.62E-01	4.53E-01		1.32E+00	1.88E-01		3.44E-01	7.97E-01		-1.73E+00	7.56E-04	Down	-1.01E+00	2.94E-02				Unigene111092_All//1.00E-58//FL576420				
Unigene111096_All	388	0.13	0.00	0.00	0.26	0.00	0.00	1.96	0.11	0.10	-7.07E+00	6.18E-01		-7.07E+00	6.31E-01		-8.00E+00	3.16E-01		-8.00E+00	4.04E-01		-4.19E+00	1.58E-04	Down	-4.26E+00	8.65E-05	Down			Unigene111096_All//1.00E-07//FL577589				
Unigene111097_All	374	0.28	0.27	0.00	0.40	0.09	0.24	2.94	0.78	0.43	-4.61E-02	9.91E-01		-8.13E+00	3.98E-01		-2.12E+00	3.87E-01		-7.27E-01	7.16E-01		-1.91E+00	2.64E-03		-2.79E+00	6.37E-05	Down			Unigene111097_All//8.00E-15//EX170653				
Unigene111098_All	789	0.13	0.00	0.00	0.00	0.00	0.00	2.57	0.05	0.45	-7.05E+00	3.76E-01		-7.05E+00	3.98E-01		null	null		null	null		-5.60E+00	5.56E-13	Down	-2.50E+00	1.27E-07	Down	Unigene111098_All//gi|124360457|gb|ABN08467.1|//3.00E-40//Haem peroxidase, plant/fungal/bacterial [Medicago truncatula]	Unigene111098_All//9.00E-37//AT4G26010| peroxidase, putative	Unigene111098_All//1.00E-30//BE053812		ko00940|Phenylpropanoid biosynthesis//ko00360|Phenylalanine metabolism	GO:0003824//GO:0005618//GO:0016209//GO:0046872	nr +
Unigene1111_All	2460	3.30	2.53	2.88	6.68	3.12	2.90	9.40	2.87	3.06	-3.80E-01	6.74E-02		-1.95E-01	3.96E-01		-1.10E+00	2.16E-17	Down	-1.20E+00	8.38E-18	Down	-1.71E+00	3.78E-47	Down	-1.62E+00	5.92E-45	Down	Unigene1111_All//gi|9294059|dbj|BAB02016.1|//0.00E+00//MAP kinase [Arabidopsis thaliana]	Unigene1111_All//0.00E+00//AT3G18040| MPK9 (MAP KINASE 9); MAP kinase	Unigene1111_All//0.00E+00//TC173934		ko04626|Plant-pathogen interaction//ko04650|Natural killer cell mediated cytotoxicity	GO:0004702//GO:0006464//GO:0009755//GO:0016020//GO:0023060//GO:0032559//GO:0043231//GO:0044444	nr +
Unigene111106_All	495	0.00	0.00	0.00	0.00	0.00	0.00	3.50	0.17	0.64	null	null		null	null		null	null		null	null		-4.37E+00	6.16E-10	Down	-2.44E+00	1.80E-06	Down							
Unigene111108_All	632	0.00	0.00	0.00	0.00	0.00	0.00	2.81	0.13	0.13	null	null		null	null		null	null		null	null		-4.41E+00	3.25E-10	Down	-4.48E+00	1.00E-10	Down	Unigene111108_All//gi|2500261|sp|P93099.1|RL13A_CYAPA//2.00E-47//RecName: Full=60S ribosomal protein L13a >gi|1743007|emb|CAA71090.1| ribosomal protein L13a [Cyanophora paradoxa]	Unigene111108_All//7.00E-41//AT3G24830| 60S ribosomal protein L13A (RPL13aB)			ko03010|Ribosome	GO:0030529	nr +
Unigene111112_All	317	0.00	0.00	0.00	0.00	0.00	0.00	2.13	0.00	0.25	null	null		null	null		null	null		null	null		-1.11E+01	6.34E-05	Down	-3.09E+00	1.45E-03		Unigene111112_All//gi|18379010|ref|NP_563662.1|//3.00E-44//pectinesterase family protein [Arabidopsis thaliana] >gi|75313808|sp|Q9SRX4.1|PME7_ARATH RecName: Full=Probable pectinesterase/pectinesterase inhibitor 7; Includes: RecName: Full=Pectinesterase inhibitor 7; AltName: Full=Pectin methylesterase inhibitor 7; Includes: RecName: Full=Pectinesterase 7; Short=PE 7; AltName: Full=Pectin methylesterase 7; AltName: Full=Pectin methylesterase 1; Short=AtPME1; Flags: Precursor >gi|6056422|gb|AAF02886.1|AC009525_20 Similar to pectinesterases [Arabidopsis thaliana] >gi|133778888|gb|AB038784.1| At1g02810 [Arabidopsis thaliana]	Unigene111112_All//2.00E-39//AT1G02810| pectinesterase family protein	Unigene111112_All//5.00E-62//TC138774		ko00500|Starch and sucrose metabolism//ko00040|Pentose and glucuronate interconversions	GO:0004091//GO:0005618//GO:0030234//GO:0031410//GO:0071555	nr -
Unigene111113_All	307	0.00	0.00	0.00	0.00	0.00	0.00	2.07	0.00	0.26	null	null		null	null		null	null		null	null		-1.10E+01	1.23E-04	Down	-2.99E+00	2.58E-03								
Unigene111115_All	420	0.00	0.00	0.00	0.12	0.00	0.00	2.32	0.20	0.09	null	null		null	null		-6.89E+00	5.62E-01		-6.89E+00	6.43E-01		-3.54E+00	4.32E-05	Down	-4.61E+00	3.32E-06	Down			Unigene111115_All//7.00E-34//FL576545				
Unigene111117_All	266	0.00	0.00	0.00	0.00	0.00	0.00	4.93	0.47	0.45	null	null		null	null		null	null		null	null		-3.39E+00	1.90E-06	Down	-3.46E+00	7.84E-07	Down	Unigene111117_All//gi|302128114|dbj|BAJ13478.1|//3.00E-18//actin 1 [Guillardia theta]	Unigene111117_All//5.00E-19//AT5G09810| ACT7 (ACTIN 7); structural constituent of cytoskeleton	Unigene111117_All//2.00E-08//TC135405		ko04145|Phagosome	GO:0000267//GO:0003015//GO:0005198//GO:0005884//GO:0006939//GO:0006950//GO:0007015//GO:0008015//GO:0008092//GO:0009314//GO:0009725//GO:0009913//GO:0012501//GO:0016020//GO:0017111//GO:0030017//GO:0030239//GO:0030312//GO:0031981//GO:0032432//GO:0032559//GO:0048588//GO:0048747//GO:0051707//GO:0060415	nr -
Unigene111119_All	424	0.00	0.00	0.00	0.24	0.08	0.00	1.70	0.79	0.09	null	null		null	null		-1.53E+00	5.88E-01		-7.88E+00	4.04E-01		-1.10E+00	1.49E-01		-4.17E+00	1.65E-04	Down	Unigene111119_All//gi|145334909|ref|NP_001078800.1|//4.00E-32//TGA1; DNA binding / calmodulin binding / transcription factor [Arabidopsis thaliana]	Unigene111119_All//2.00E-33//AT5G65210| TGA1; DNA binding / calmodulin binding / transcription factor	Unigene111119_All//9.00E-18//TC148920	bZIP		GO:0001071//GO:0003677//GO:0005515//GO:0006351//GO:0006952//GO:0043231	nr +
Unigene111120_All	479	0.00	0.00	0.00	0.00	0.00	0.00	3.71	0.79	0.42	null	null		null	null		null	null		null	null		-2.24E+00	8.44E-06	Down	-3.16E+00	2.19E-08	Down	Unigene111120_All//gi|12580867|emb|CAC27142.1|//3.00E-31//60S ribosomal protein L13E [Picea abies]	Unigene111120_All//6.00E-27//AT3G49010| ATBBC1 (ARABIDOPSIS THALIANA BREAST BASIC CONSERVED 1); structural constituent of ribosome			ko03010|Ribosome	GO:0005622	nr -
Unigene111123_All	880	0.00	0.00	0.00	0.00	0.00	0.00	6.20	2.09	10.68	null	null		null	null		null	null		null	null		-1.57E+00	2.00E-10	Down	7.86E-01	1.77E-06		Unigene111123_All//gi|73624747|gb|AAZ79231.1|//1.00E-81//cellulose synthase-like protein CslG [Nicotiana tabacum]	Unigene111123_All//9.00E-75//AT4G23990| ATCSLG3; cellulose synthase/ transferase/ transferase, transferring glycosyl groups	Unigene111123_All//4.00E-12//TC160226			GO:0009987//GO:0016020//GO:0016757	nr -
Unigene111124_All	455	0.00	0.00	0.00	0.00	0.30	0.00	3.53	0.92	0.18	null	null		null	null		8.24E+00	3.20E-01		null	null		-1.94E+00	1.42E-04	Down	-4.33E+00	1.41E-09	Down	Unigene111124_All//gi|15718687|ref|NP_000996.2|//1.00E-33//40S ribosomal protein S3 [Homo sapiens] >gi|77404290|ref|NP_001029219.1| 40S ribosomal protein S3 [Bos taurus] >gi|113205854|ref|NP_001038066.1| 40S ribosomal protein S3 [Sus scrofa] >gi|109107963|ref|XP_001086020.1| PREDICTED: 40S ribosomal protein S3 [Macaca mulatta] >gi|417719|sp|P23396.2|RS3_HUMAN RecName: Full=40S ribosomal protein S3 >gi|91207641|sp|Q3T169.1|RS3_BOVIN RecName: Full=40S ribosomal protein S3 >gi|115502828|sp|Q0Z8U2.1|RS3_PIG RecName: Full=40S ribosomal protein S3 >gi|187609257|pdb|2ZKQ|CC Chain c, Structure Of A Mammalian Ribosomal 40s Subunit Within An 80s Complex Obtained By Docking Homology Models Of The Rna And Proteins Into An 8.7 A Cryo-Em Map >gi|555941|gb|AAB60336.1| ribosomal protein S3 [Homo	Unigene111124_All//5.00E-22//AT3G53870| 40S ribosomal protein S3 (RPS3B)			ko03010|Ribosome	GO:0001726//GO:0003677//GO:0003723//GO:0004518//GO:0005198//GO:0006282//GO:0006412//GO:0008134//GO:0012502//GO:0015935//GO:0016835//GO:0019900//GO:0043231//GO:0043280//GO:0043433//GO:0051540	nr +
Unigene111125_All	250	0.00	0.00	0.00	0.00	0.00	0.00	2.88	1.34	0.00	null	null		null	null		null	null		null	null		-1.10E+00	1.49E-01		-1.15E+01	1.78E-05	Down			Unigene111125_All//3.00E-16//DW479423				
Unigene111128_All	319	0.00	0.00	0.00	0.00	0.00	0.00	0.27	0.00	24.10	null	null		null	null		null	null		null	null		-8.05E+00	4.06E-01		6.51E+00	1.85E-51	Up			Unigene111128_All//4.00E-90//TC133844				
Unigene111131_All	261	0.80	0.00	1.02	1.34	0.26	0.17	6.64	1.60	1.53	-9.65E+00	1.23E-01		3.47E-01	7.97E-01		-2.34E+00	1.01E-01		-2.95E+00	8.31E-02		-2.05E+00	3.55E-05	Down	-2.12E+00	1.37E-05	Down			Unigene111131_All//2.00E-138//DW519828				
Unigene111132_All	254	0.00	0.00	0.00	0.00	0.00	0.00	3.50	0.16	0.78	null	null		null	null		null	null		null	null		-4.41E+00	2.44E-05	Down	-2.16E+00	2.68E-03								

Unigene111133_All	371	0.00	0.00	0.00	0.00	0.00	0.00	1.37	0.45	0.00	null	null		null	null		null	null		null	null		-1.60E+00	1.07E-01		-1.04E+01	5.55E-04	Down	Unigene111133_All//gi|219116705|ref|XP_002179147.1|//8.00E-06//nad-dependent epimerase/dehydratase [Phaeodactylum tricornutum CCAP 1055/1] >gi|217409038|gb|EEC48970.1| nad-dependent epimerase/dehydratase [Phaeodactylum tricornutum CCAP 1055/1]						nr -
Unigene111134_All	626	0.17	0.00	0.00	0.08	0.22	0.14	2.97	0.00	0.76	-7.39E+00	3.76E-01		-7.39E+00	3.98E-01		1.47E+00	6.20E-01		8.58E-01	7.96E-01		-1.15E+01	3.59E-13	Down	-1.96E+00	1.89E-05	Down							ESTscan +
Unigene111139_All	721	0.00	0.00	0.00	0.00	0.00	0.00	2.11	1.10	0.44	null	null		null	null		null	null		null	null		-9.39E-01	5.17E-02		-2.26E+00	2.48E-05	Down	Unigene111139_All//gi|227438273|gb|ACP30626.1|//7.00E-25//disease resistance protein [Brassica rapa subsp. pekinensis]	Unigene111139_All//4.00E-25//AT4G12010| disease resistance protein (TIR-NBS-LRR class), putative				GO:0005488//GO:0009987	nr +
Unigene111140_All	276	0.95	0.18	0.00	0.18	0.25	0.00	1.84	0.30	0.00	-2.37E+00	2.14E-01		-9.89E+00	8.59E-02		4.67E-01	9.60E-01		-7.50E+00	6.43E-01		-2.60E+00	2.05E-02		-1.08E+01	5.55E-04	Down							
Unigene111143_All	395	0.00	0.00	0.00	0.00	0.00	0.00	4.60	0.74	0.00	null	null		null	null		null	null		null	null		-2.64E+00	5.50E-07	Down	-1.22E+01	2.03E-13	Down							
Unigene111150_All	275	0.00	0.00	0.58	0.18	0.50	0.00	2.00	0.15	0.00	null	null		9.18E+00	2.40E-01		1.47E+00	6.20E-01		-7.50E+00	6.43E-01		-3.72E+00	3.23E-03		-1.10E+01	2.81E-04	Down							
Unigene111155_All	288	0.00	0.00	0.00	0.00	0.00	0.00	1.91	0.00	0.14	null	null		null	null		null	null		null	null		-1.09E+01	4.62E-04	Down	-3.79E+00	2.03E-03		Unigene111155_All//gi|4139170|gb|AAD03711.1|//5.00E-36//elongation translation factor 1 alpha [Cyanophora paradoxa]	Unigene111155_All//8.00E-33//AT5G60390| elongation factor 1-alpha / EF-1-alpha	Unigene111155_All//2.00E-08//DR462623			GO:0006412//GO:0008135//GO:0017111//GO:0032561//GO:0044424	nr +
Unigene111157_All	498	0.00	0.00	0.00	0.10	0.00	0.09	1.95	0.34	0.24	null	null		null	null		-6.64E+00	5.62E-01		-1.41E-01	9.56E-01		-2.54E+00	6.42E-04	Down	-3.02E+00	9.61E-05	Down	Unigene111157_All//gi|224589412|gb|ACN59240.1|//4.00E-62//leucine-rich repeat receptor-like protein kinase [Arabidopsis thaliana]	Unigene111157_All//2.00E-40//AT1G34110| leucine-rich repeat transmembrane protein kinase, putative			ko04626|Plant-pathogen interaction	GO:0016301	nr -
Unigene111162_All	520	0.00	0.00	0.00	0.00	0.00	0.00	2.20	0.72	0.00	null	null		null	null		null	null		null	null		-1.60E+00	7.51E-03		-1.11E+01	1.65E-08	Down	Unigene111162_All//sp|P63240|NEC1_MUSCO//3.00E-41//Neuroendocrine convertase 1 OS=Mus cookii GN=Pcsk1 PE=2 SV=1						swissprot -
Unigene111163_All	299	0.00	0.00	0.00	0.00	0.00	0.00	1.98	0.00	0.13	null	null		null	null		null	null		null	null		-1.10E+01	2.39E-04	Down	-3.89E+00	1.09E-03		Unigene111163_All//gi|224078614|ref|XP_002305575.1|//6.00E-22//predicted protein [Populus trichocarpa] >gi|222848539|gb|EEE86086.1| predicted protein [Populus trichocarpa]						nr +
Unigene111166_All	318	0.00	0.00	0.17	0.63	0.00	0.14	1.73	0.00	0.13	null	null		7.39E+00	6.40E-01		-9.29E+00	8.47E-02		-2.14E+00	3.47E-01		-1.08E+01	4.62E-04	Down	-3.79E+00	2.03E-03		Unigene111166_All//gi|260177090|gb|ACX33888.1|//3.00E-37//flavin monooxygenase-like protein [Solanum lycopersicum]	Unigene111166_All//1.00E-35//AT5G43890| YUCCA5; monooxygenase	Unigene111166_All//3.00E-20//gi|78348897|gb|DT569171.1|DT569171		ko00380|Tryptophan metabolism	GO:0009850//GO:0016709//GO:0030554	nr -
Unigene111174_All	327	0.00	0.00	0.00	0.00	0.00	0.00	1.68	0.00	0.00	null	null		null	null		null	null		null	null		-1.07E+01	4.62E-04	Down	-1.07E+01	2.81E-04	Down							ESTscan -
Unigene111175_All	319	0.16	0.00	0.33	0.78	0.32	0.57	2.39	0.13	0.25	-7.36E+00	6.18E-01		1.03E+00	7.04E-01		-1.27E+00	4.24E-01		-4.63E-01	7.45E-01		-4.19E+00	1.58E-04	Down	-3.26E+00	4.48E-04	Down							
Unigene111177_All	639	0.00	0.00	0.00	0.00	0.00	0.00	2.85	0.00	0.19	null	null		null	null		null	null		null	null		-1.15E+01	7.10E-13	Down	-3.93E+00	4.04E-10	Down	Unigene111177_All//gi|110589651|gb|ABG77330.1|//4.00E-76//Hsp90 [Entosiphon sulcatum]	Unigene111177_All//3.00E-64//AT5G56030| HSP81-2 (HEAT SHOCK PROTEIN 81-2); ATP binding			ko04626|Plant-pathogen interaction//ko04141|Protein processing in endoplasmic reticulum	GO:0005515//GO:0032559//GO:0044267//GO:0044424//GO:0050896	nr +
Unigene11118_All	398	3.16	0.64	6.96	7.38	7.44	9.08	3.61	4.10	5.00	-2.31E+00	9.22E-04	Down	1.14E+00	4.31E-03		1.10E-02	9.89E-01		2.98E-01	4.07E-01		1.81E-01	7.06E-01		4.71E-01	2.45E-01		Unigene11118_All//gi|15450488|gb|AAK96537.1|//3.00E-06//At2g36480/F1O11.11 [Arabidopsis thaliana] >gi|16974461|gb|AAL31234.1| At2g36480/F1O11.11 [Arabidopsis thaliana] >gi|20197924|gb|AAM15315.1| Expressed protein [Arabidopsis thaliana]	Unigene11118_All//1.00E-07//AT2G36485| FUNCTIONS IN: molecular_function unknown; INVOLVED IN: biological_process unknown; LOCATED IN: chloroplast; EXPRESSED IN: 14 plant structures; EXPRESSED DURING: 6 growth stages; CONTAINS InterPro DOMAIN/s: ENTH/VHS (InterPro:IPR008942); BEST Arabidopsis thaliana protein match is: PCFS4 (PCF11P-SIMILAR PROTEIN 4); zinc ion binding (TAIR:AT4G04885.1); Has 87 Blast hits to 87 proteins in 38 species: Archae - 0; Bacteria - 0; Metazoa - 30; Fungi - 33; Plants - 24; Viruses - 0; Other Eukaryotes - 0 (source: NCBI BLink).	Unigene11118_All//5.00E-152//TC177315				nr -
Unigene111180_All	613	0.00	0.25	0.00	0.00	0.00	0.00	1.66	0.41	0.26	7.95E+00	2.36E-01		null	null		null	null		null	null		-2.02E+00	2.83E-03		-2.67E+00	1.94E-04	Down			Unigene111180_All//1.00E-14//TC168901				
Unigene111181_All	305	0.00	0.00	0.00	0.00	0.00	0.00	3.74	1.51	0.00	null	null		null	null		null	null		null	null		-1.31E+00	2.45E-02		-1.19E+01	1.65E-08	Down	Unigene111181_All//gi|115477126|ref|NP_001062159.1|//4.00E-48//Os08g0500700 [Oryza sativa Japonica Group] >gi|113624128|dbj|BAF24073.1| Os08g0500700 [Oryza sativa Japonica Group]	Unigene111181_All//6.00E-40//AT5G56010| HSP81-3; ATP binding / unfolded protein binding	Unigene111181_All//9.00E-42//gi|11208934|gb|BF277864.1|BF277864		ko04626|Plant-pathogen interaction//ko04141|Protein processing in endoplasmic reticulum	GO:0005515//GO:0032559//GO:0044267//GO:0044424//GO:0050896	nr +
Unigene111189_All	310	0.00	0.00	0.00	0.00	0.00	0.00	2.18	0.00	0.00	null	null		null	null		null	null		null	null		-1.11E+01	6.34E-05	Down	-1.11E+01	3.57E-05	Down	Unigene111189_All//gi|27764533|gb|AAO23064.1|//7.00E-07//ent-kaurenoic acid oxidase [Pisum sativum]	Unigene111189_All//2.00E-06//AT1G05160| CYP88A3 (CYTOCHROME P450 88 A3); ent-kaurenoate oxidase/ oxygen binding			ko00904|Diterpenoid biosynthesis		nr -
Unigene111194_All	690	0.61	0.00	1.39	0.58	0.40	0.46	4.04	1.76	0.64	-9.24E+00	1.05E-02		1.20E+00	1.15E-01		-5.33E-01	6.16E-01		-3.34E-01	7.57E-01		-1.20E+00	4.34E-04	Down	-2.67E+00	6.57E-11	Down	Unigene111194_All//gi|222423130|dbj|BAH19544.1|//2.00E-14//AT4G21215 [Arabidopsis thaliana]	Unigene111194_All//4.00E-16//AT4G21215| unknown protein	Unigene111194_All//9.00E-16//DW236374				nr -
Unigene111195_All	794	0.00	0.26	0.13	0.00	0.09	0.00	3.51	0.00	0.30	8.00E+00	1.37E-01		7.07E+00	4.01E-01		6.44E+00	6.14E-01		null	null		-1.18E+01	9.81E-20	Down	-3.55E+00	4.30E-14	Down	Unigene111195_All//gi|38564292|gb|AAR23725.1|//2.00E-47//At5g62280 [Arabidopsis thaliana] >gi|45592914|gb|AAS68111.1| At5g62280 [Arabidopsis thaliana]	Unigene111195_All//2.00E-49//AT5G62280| unknown protein					nr +
Unigene111197_All	590	0.00	0.00	0.00	0.00	0.00	0.00	2.51	0.57	0.47	null	null		null	null		null	null		null	null		-2.15E+00	9.69E-05	Down	-2.41E+00	1.48E-05	Down	Unigene111197_All//gi|15223002|ref|NP_172848.1|//6.00E-42//ELF5A-1 (EUKARYOTIC ELONGATION FACTOR 5A-1); translation initiation factor [Arabidopsis thaliana] >gi|20138796|sp|Q9XI91.1|IF5A1_ARATH RecName: Full=Eukaryotic translation initiation factor 5A-1; Short=eIF-5A-1 >gi|5080771|gb|AAD39281.1|AC007576_4 initiation factor 5A-4 [Arabidopsis thaliana] >gi|12407785|gb|AAG53646.1|AF296082_1 eukaryotic translation initiation factor 5A [Arabidopsis thaliana]	Unigene111197_All//1.00E-43//AT1G13950| ELF5A-1 (EUKARYOTIC ELONGATION FACTOR 5A-1); translation initiation factor				GO:0006417//GO:0008135//GO:0010087	nr -
Unigene1112_All	2717	2.33	1.94	1.67	2.59	2.92	2.63	11.19	3.35	4.18	-2.65E-01	2.84E-01		-4.84E-01	5.03E-02		1.73E-01	4.79E-01		2.29E-02	9.42E-01		-1.74E+00	4.18E-63	Down	-1.42E+00	1.23E-48	Down	Unigene1112_All//gi|30689234|ref|NP_850382.1|//0.00E+00//NPG1 (no pollen germination 1); calmodulin binding [Arabidopsis thaliana] >gi|27450574|gb|AAO14644.1|AF474176_1 pollen-specific calmodulin-binding protein [Arabidopsis thaliana] >gi|209529807|gb|ACI49798.1| At2g43040 [Arabidopsis thaliana]	Unigene1112_All//0.00E+00//AT2G43040| NPG1 (no pollen germination 1); calmodulin binding	Unigene1112_All//0.00E+00//TC145967			GO:0005515//GO:0016740	nr +
Unigene111201_All	324	0.00	0.00	0.00	0.00	0.00	0.00	2.74	1.03	0.12	null	null		null	null		null	null		null	null		-1.41E+00	4.01E-02		-4.48E+00	1.23E-05	Down	Unigene111201_All//gi|81159226|gb|ABB55890.1|//3.00E-41//heat shock protein 90 [Proteomonas sulcata]	Unigene111201_All//2.00E-42//AT5G52640| ATHSP90.1 (HEAT SHOCK PROTEIN 90.1); ATP binding / unfolded protein binding			ko04626|Plant-pathogen interaction//ko04141|Protein processing in endoplasmic reticulum	GO:0005515//GO:0006952//GO:0010035//GO:0016020//GO:0030312//GO:0032559//GO:0044267//GO:0044444	nr -
Unigene111202_All	450	0.00	0.00	0.00	0.00	0.00	0.00	1.50	0.00	0.00	null	null		null	null		null	null		null	null		-1.06E+01	6.34E-05	Down	-1.06E+01	3.57E-05	Down							ESTscan +
Unigene111207_All	324	0.32	0.63	0.82	4.92	1.28	0.56	1.70	1.03	0.61	9.54E-01	6.22E-01		1.35E+00	4.09E-01		-1.95E+00	2.04E-04	Down	-3.14E+00	2.25E-06	Down	-7.17E-01	4.22E-01		-1.46E+00	9.76E-02								
Unigene111210_All	300	0.00	0.00	0.00	0.00	0.00	0.00	1.83	1.11	0.00	null	null		null	null		null	null		null	null		-7.17E-01	4.22E-01		-1.08E+01	2.81E-04	Down	Unigene111210_All//sp|P18426|SCR11_OMMSL//4.00E-06//S-crystallin SL11 OS=Ommastrephes sloanei PE=2 SV=1						swissprot +
Unigene111211_All	331	0.00	0.00	0.00	0.00	0.00	0.00	2.68	0.25	0.00	null	null		null	null		null	null		null	null		-3.41E+00	1.40E-04	Down	-1.14E+01	1.10E-06	Down	Unigene111211_All//gi|298708862|emb|CBJ30820.1|//6.00E-17//40S ribosomal protein S28 [Ectocarpus siliculosus]	Unigene111211_All//3.00E-09//AT5G03850| 40S ribosomal protein S28 (RPS28B)			ko03010|Ribosome	GO:0005198//GO:0010467//GO:0030529	nr -
Unigene111212_All	274	0.00	0.00	0.00	0.00	0.00	0.00	3.39	1.07	0.15	null	null		null	null		null	null		null	null		-1.67E+00	1.45E-02		-4.55E+00	6.41E-06	Down	Unigene111212_All//gi|288300146|gb|ADC45376.1|//5.00E-32//actin [Cleistogenes songorica]	Unigene111212_All//1.00E-30//AT5G59370| ACT4 (ACTIN 4); structural constituent of cytoskeleton	Unigene111212_All//1.00E-21//gi|5046957|gb|AI728105.1|AI728105		ko04145|Phagosome	GO:0000267//GO:0003015//GO:0005198//GO:0005884//GO:0006939//GO:0007015//GO:0008015//GO:0008092//GO:0012501//GO:0017111//GO:0030017//GO:0030239//GO:0032432//GO:0032559//GO:0042995//GO:0048747//GO:0051707//GO:0060415	nr -
Unigene111213_All	450	0.81	1.91	0.24	0.66	0.46	0.00	2.07	0.28	0.44	1.23E+00	1.12E-01		-1.78E+00	2.34E-01		-5.33E-01	6.79E-01		-9.38E+00	4.01E-02		-2.89E+00	3.28E-04	Down	-2.22E+00	1.63E-03		Unigene111213_All//gi|115478484|ref|NP_001062837.1|//3.00E-09//Os09g0311600 [Oryza sativa Japonica Group] >gi|113631070|dbj|BAF24751.1| Os09g0311600 [Oryza sativa Japonica Group]	Unigene111213_All//1.00E-07//AT4G27190| disease resistance protein (NBS-LRR class), putative	Unigene111213_All//3.00E-150//CO097771		ko04626|Plant-pathogen interaction	GO:0005488	nr -
Unigene111214_All	419	0.00	0.00	0.00	0.00	0.00	0.00	3.43	0.70	0.19	null	null		null	null		null	null		null	null		-2.30E+00	5.90E-05	Down	-4.17E+00	1.93E-08	Down	Unigene111214_All//gi|145344703|ref|XP_001416866.1|//1.00E-35//Ribosomal protein S20, component of cytosolic 80S ribosome and 40S small subunit [Ostreococcus lucimarinus CCE9901] >gi|144577092|gb|ABO95159.1| Ribosomal protein S20, component of cytosolic 80S ribosome and 40S small subunit [Ostreococcus lucimarinus CCE9901]	Unigene111214_All//4.00E-31//AT5G62300| 40S ribosomal protein S20 (RPS20C)			ko03010|Ribosome	GO:0003676//GO:0004672//GO:0005198//GO:0006464//GO:0010467//GO:0032559//GO:0033279	nr +
Unigene111216_All	278	0.00	0.00	0.00	0.00	0.00	0.00	8.82	4.36	21.64	null	null		null	null		null	null		null	null		-1.02E+00	5.18E-03		1.29E+00	5.47E-09	Up							
Unigene11122_All	972	1.99	4.22	3.94	5.53	4.61	4.46	4.31	3.74	5.41	1.08E+00	3.96E-04	Up	9.86E-01	2.63E-03		-2.65E-01	3.33E-01		-3.11E-01	2.67E-01		-2.03E-01	5.00E-01		3.29E-01	1.61E-01		Unigene11122_All//gi|115480719|ref|NP_001063953.1|//5.00E-61//Os09g0566100 [Oryza sativa Japonica Group] >gi|113632186|dbj|BAF25867.1| Os09g0566100 [Oryza sativa Japonica Group]	Unigene11122_All//1.00E-45//AT2G36480| zinc finger (C2H2-type) family protein	Unigene11122_All//0.00E+00//TC177315			GO:0046914	nr -
Unigene111220_All	333	0.00	0.00	0.00	0.00	0.00	0.00	4.19	1.88	0.96	null	null		null	null		null	null		null	null		-1.15E+00	2.40E-02		-2.13E+00	1.13E-04	Down							
Unigene111225_All	337	0.00	0.00	0.00	0.00	0.00	0.00	1.88	0.12	0.00	null	null		null	null		null	null		null	null		-3.92E+00	9.81E-04	Down	-1.09E+01	7.12E-05	Down	Unigene111225_All//gi|224103789|ref|XP_002313194.1|//6.00E-52//tubulin alpha-5 chain [Populus trichocarpa] >gi|222849602|gb|EEE87149.1| tubulin alpha-5 chain [Populus trichocarpa]	Unigene111225_All//3.00E-53//AT1G50010| TUA2; structural constituent of cytoskeleton	Unigene111225_All//1.00E-28//gi|109864583|gb|DW493559.1|DW493559		ko04145|Phagosome	GO:0006970//GO:0007017//GO:0009536//GO:0009629//GO:0010038//GO:0015630//GO:0016020//GO:0017111//GO:0030312//GO:0032561//GO:0043234//GO:0043623	nr +
Unigene111226_All	1265	0.00	0.00	0.00	0.00	0.00	0.00	2.71	0.10	0.28	null	null		null	null		null	null		null	null		-4.77E+00	3.63E-20	Down	-3.26E+00	5.76E-16	Down	Unigene111226_All//gi|298715057|emb|CBJ27764.1|//6.00E-08//delta protein [Ectocarpus siliculosus]					GO:0044464	nr +
Unigene111227_All	562	0.74	0.00	0.47	0.00	0.18	0.16	1.20	0.60	0.07	-9.54E+00	1.05E-02		-6.53E-01	5.94E-01		7.52E+00	4.58E-01		7.33E+00	4.80E-01		-1.02E+00	2.04E-01		-4.09E+00	3.10E-04	Down			Unigene111227_All//1.00E-08//ES832462				

Unigene111229_All	597	0.00	0.00	0.00	0.00	0.12	0.00	9.77	0.84	1.60	null	null		null	null		6.85E+00	6.14E-01		null	null		-3.54E+00	9.45E-28	Down	-2.61E+00	1.87E-21	Down	Unigene111229_All//gi|18418598|ref|NP_567979.1|//3.00E-56//cyclase family protein [Arabidopsis thaliana] >gi|13937204|gb|AAK50095.1|AF372956_1 AT4g35220/F23E12_220 [Arabidopsis thaliana] >gi|18491127|gb|AAL69532.1| AT4g35220/F23E12_220 [Arabidopsis thaliana]	Unigene111229_All//2.00E-57//AT4G35220| cyclase family protein	Unigene111229_All//1.00E-128//TC158981			GO:0031410	nr -
Unigene111230_All	345	1.21	2.94	1.39	1.16	1.40	0.92	1.59	5.21	2.08	1.28E+00	6.99E-02		1.95E-01	8.39E-01		2.75E-01	8.20E-01		-3.34E-01	7.57E-01		1.71E+00	2.41E-04	Up	3.84E-01	5.96E-01		Unigene111230_All//gi|224071337|ref|XP_002303411.1|//8.00E-14//oligopeptide transporter OPT family [Populus trichocarpa] >gi|222840843|gb|EEE78390.1| oligopeptide transporter OPT family [Populus trichocarpa]	Unigene111230_All//1.00E-13//AT4G10770| OPT7 (OLIGOPEPTIDE TRANSPORTER 7); oligopeptide transporter				GO:0015197//GO:0015833//GO:0044464	nr -
Unigene111231_All	301	0.00	0.00	0.00	0.00	0.00	0.00	2.39	2.08	0.13	null	null		null	null		null	null		null	null		-1.97E-01	7.84E-01		-4.17E+00	1.64E-04	Down							
Unigene111233_All	537	0.00	0.00	0.00	0.00	0.00	0.00	4.02	0.16	0.22	null	null		null	null		null	null		null	null		-4.69E+00	9.88E-13	Down	-4.17E+00	2.24E-12	Down	Unigene111233_All//gi|297833676|ref|XP_002884720.1|//4.00E-26//60S acidic ribosomal protein P0 [Arabidopsis lyrata subsp. lyrata] >gi|297330560|gb|EFH60979.1| 60S acidic ribosomal protein P0 [Arabidopsis lyrata subsp. lyrata]	Unigene111233_All//9.00E-22//AT3G11250| 60S acidic ribosomal protein P0 (RPP0C)			ko03010|Ribosome	GO:0005840//GO:0006412//GO:0006970//GO:0009536//GO:0010038//GO:0016020//GO:0031981//GO:0046914	nr -
Unigene111234_All	287	2.19	0.00	0.74	2.95	0.72	1.10	1.62	0.29	0.83	-1.11E+01	7.92E-04	Down	-1.56E+00	1.29E-01		-2.04E+00	9.05E-03		-1.42E+00	7.34E-02		-2.48E+00	3.38E-02		-9.60E-01	3.13E-01		Unigene111234_All//gi|15240353|ref|NP_200987.1|//3.00E-44//calnexin 1 (CNX1) [Arabidopsis thaliana] >gi|231683|sp|P29402.1|CALX1_ARATH RecName: Full=Calnexin homolog 1; Flags: Precursor >gi|16211|emb|CAA79144.1| calnexin homolog [Arabidopsis thaliana] >gi|10176872|dbj|BAB10079.1| calnexin homolog precursor [Arabidopsis thaliana] >gi|16649021|gb|AAL24362.1| calnexin homolog precursor [Arabidopsis thaliana] >gi|21387169|gb|AAM47988.1| calnexin-like protein precursor [Arabidopsis thaliana] >gi|21555674|gb|AAM63911.1| calnexin-like protein [Arabidopsis thaliana]	Unigene111234_All//1.00E-45//AT5G61790| calnexin 1 (CNX1)	Unigene111234_All//5.00E-154//TC161906		ko04141|Protein processing in endoplasmic reticulum//ko04145|Phagosome	GO:0005515//GO:0009536//GO:0030246//GO:0031090//GO:0031224//GO:0044267//GO:0046872	nr -
Unigene111235_All	332	0.00	0.00	0.00	0.00	0.52	0.54	1.78	0.63	0.00	null	null		null	null		9.02E+00	2.27E-01		9.09E+00	1.95E-01		-1.50E+00	9.14E-02		-1.08E+01	1.41E-04	Down	Unigene111235_All//gi|159031811|dbj|BAF91877.1|//5.00E-33//polyamine oxidase [Nicotiana benthamiana]	Unigene111235_All//2.00E-31//AT5G13700| ATPAO1 (ARABIDOPSIS THALIANA POLYAMINE OXIDASE 1); FAD binding / polyamine oxidase	Unigene111235_All//5.00E-71//gi|78327583|gb|DT547857.1|DT547857			GO:0008152//GO:0016647	nr +
Unigene111236_All	424	0.00	0.00	0.00	0.00	0.00	0.00	1.40	0.00	0.00	null	null		null	null		null	null		null	null		-1.04E+01	2.39E-04	Down	-1.04E+01	1.41E-04	Down	Unigene111236_All//sp|Q5LWL4|IF2_SILPO//4.00E-06//Translation initiation factor IF-2 OS=Silicibacter pomeroyi GN=infB PE=3 SV=1						swissprot +
Unigene111238_All	290	0.00	0.00	0.00	0.00	0.24	0.00	3.50	0.43	1.51	null	null		null	null		7.89E+00	6.14E-01		null	null		-3.02E+00	1.08E-04	Down	-1.21E+00	4.17E-02		Unigene111238_All//gi|115435928|ref|NP_001042722.1|//2.00E-09//Os01g0275600 [Oryza sativa Japonica Group] >gi|75203337|sp|Q9SDG8.1|AGO4A_ORYSJ RecName: Full=Protein argonaute 4A; Short=OsAGO4a >gi|113532253|dbj|BAF04636.1| Os01g0275600 [Oryza sativa Japonica Group]	Unigene111238_All//1.00E-08//AT5G21150| PAZ domain-containing protein / piwi domain-containing protein	Unigene111238_All//1.00E-37//gi|109838370|gb|DW236931.1|DW236931			GO:0003676	nr -
Unigene111244_All	497	0.00	0.00	0.00	0.00	0.00	0.00	8.17	0.17	0.00	null	null		null	null		null	null		null	null		-5.60E+00	1.17E-25	Down	-1.30E+01	7.66E-30	Down			Unigene111244_All//4.00E-11//ES809074				
Unigene111245_All	311	0.17	0.49	0.00	0.00	0.22	0.00	3.26	0.00	0.90	1.54E+00	5.32E-01		-7.39E+00	6.31E-01		7.79E+00	6.14E-01		null	null		-1.17E+01	2.97E-07	Down	-1.86E+00	3.54E-03		Unigene111245_All//gi|83853831|gb|ABC47863.1|//5.00E-19//Heat shock transcription factor (HSF) [Glycine max]	Unigene111245_All//3.00E-09//AT1G46264| AT-HSFB4; DNA binding / transcription factor	Unigene111245_All//2.00E-08//TC156804	HSF		GO:0001071//GO:0003677//GO:0006351//GO:0043231//GO:0050896//GO:0051301	nr +
Unigene111246_All	422	0.37	0.00	0.50	0.00	1.47	2.14	2.20	1.19	0.38	-8.54E+00	2.17E-01		4.41E-01	7.76E-01		1.05E+01	7.90E-04	Up	1.11E+01	2.07E-05	Up	-8.91E-01	1.72E-01		-2.55E+00	5.79E-04	Down			Unigene111246_All//3.00E-122//TC163108				
Unigene111248_All	326	0.00	0.00	0.00	0.00	0.00	0.00	2.85	0.13	0.12	null	null		null	null		null	null		null	null		-4.48E+00	1.30E-05	Down	-4.55E+00	6.41E-06	Down	Unigene111248_All//gi|300175324|emb|CBK20635.2|//1.00E-12//Ribosomal protein S28e [Blastocystis hominis]	Unigene111248_All//3.00E-07//AT5G64140| RPS28 (RIBOSOMAL PROTEIN S28); structural constituent of ribosome			ko03010|Ribosome	GO:0005198//GO:0010467//GO:0030529	nr -
Unigene111249_All	335	0.00	0.00	0.00	0.00	0.00	0.00	4.29	0.25	0.24	null	null		null	null		null	null		null	null		-4.10E+00	5.15E-08	Down	-4.17E+00	1.93E-08	Down							
Unigene111251_All	311	0.00	0.00	0.00	0.00	0.00	0.00	1.90	0.27	0.00	null	null		null	null		null	null		null	null		-2.82E+00	7.18E-03		-1.09E+01	1.41E-04	Down	Unigene111251_All//gi|238481396|ref|NP_198107.2|//1.00E-25//ATP binding / microtubule motor [Arabidopsis thaliana]	Unigene111251_All//1.00E-26//AT5G27550| ATP binding / microtubule motor				GO:0003774//GO:0007017//GO:0015630//GO:0032559	nr +
Unigene111252_All	406	0.00	0.00	0.00	0.00	0.00	0.00	3.96	0.51	0.00	null	null		null	null		null	null		null	null		-2.94E+00	6.45E-07	Down	-1.20E+01	7.00E-12	Down	Unigene111252_All//gi|1174592|sp|P46259.1|TBA1_PEA//6.00E-50//RecName: Full=Tubulin alpha-1 chain >gi|525332|gb|AAA79910.1| alpha-tubulin [Pisum sativum]	Unigene111252_All//2.00E-47//AT4G14960| TUA6; structural constituent of cytoskeleton	Unigene111252_All//1.00E-35//gi|84148110|gb|DN762352.1|DN762352		ko04145|Phagosome	GO:0007017//GO:0015630//GO:0017111//GO:0032561//GO:0032991//GO:0043623	nr +
Unigene111253_All	1446	0.00	0.00	0.00	0.00	0.00	0.00	26.99	3.18	4.19	null	null		null	null		null	null		null	null		-3.09E+00	4.97E-161	Down	-2.69E+00	6.47E-144	Down	Unigene111253_All//gi|297841521|ref|XP_002888642.1|//2.00E-85//S-adenosyl-L-methionine:carboxyl methyltransferase family protein [Arabidopsis lyrata subsp. lyrata] >gi|297334483|gb|EFH64901.1| S-adenosyl-L-methionine:carboxyl methyltransferase family protein [Arabidopsis lyrata subsp. lyrata]	Unigene111253_All//5.00E-85//AT1G68040| S-adenosyl-L-methionine:carboxyl methyltransferase family protein			ko00592|alpha-Linolenic acid metabolism	GO:0008168	nr +
Unigene111255_All	342	0.00	0.00	0.00	0.00	0.00	0.00	5.69	2.93	0.23	null	null		null	null		null	null		null	null		-9.55E-01	2.18E-02		-4.61E+00	6.97E-12	Down	Unigene111255_All//gi|7799051|emb|CAB90826.1|//1.00E-26//ubiquitin [Cyanidium caldarium]	Unigene111255_All//1.00E-26//AT5G03240| UBQ3 (POLYUBIQUITIN 3); protein binding	Unigene111255_All//1.00E-13//ES829082		ko03010|Ribosome	GO:0005198//GO:0010467//GO:0016209//GO:0030529//GO:0046914	nr +
Unigene111256_All	429	0.00	0.00	0.37	0.35	0.00	0.11	2.76	0.10	0.09	null	null		8.54E+00	2.40E-01		-8.44E+00	1.66E-01		-1.73E+00	5.02E-01		-4.82E+00	2.84E-07	Down	-4.89E+00	1.20E-07	Down			Unigene111256_All//2.00E-37//TC166761				
Unigene111259_All	422	0.00	0.00	0.00	0.00	0.00	0.00	10.42	1.68	1.13	null	null		null	null		null	null		null	null		-2.63E+00	6.39E-16	Down	-3.20E+00	5.31E-20	Down			Unigene111259_All//8.00E-86//CO091281				
Unigene111260_All	400	5.49	0.38	0.00	2.49	1.38	0.34	12.90	7.52	6.77	-3.85E+00	1.84E-09	Down	-1.24E+01	5.21E-12	Down	-8.55E-01	2.05E-01		-2.88E+00	6.76E-04	Down	-7.77E-01	9.16E-04		-9.29E-01	5.42E-05				Unigene111260_All//5.00E-121//gi|48749456|gb|CO079975.1|CO079975				
Unigene111262_All	376	0.00	0.00	0.00	0.53	0.00	0.00	2.81	0.33	0.21	null	null		null	null		-9.05E+00	8.47E-02		-9.05E+00	1.34E-01		-3.08E+00	6.12E-05	Down	-3.73E+00	6.14E-06	Down	Unigene111262_All//gi|224121846|ref|XP_002318687.1|//3.00E-29//cytochrome P450 [Populus trichocarpa] >gi|222859360|gb|EEE96907.1| cytochrome P450 [Populus trichocarpa]	Unigene111262_All//4.00E-19//AT3G48300| CYP71A23; electron carrier/ heme binding / iron ion binding / monooxygenase/ oxygen binding	Unigene111262_All//2.00E-49//CO115886		ko00941|Flavonoid biosynthesis//ko00380|Tryptophan metabolism//ko00944|Flavone and flavonol biosynthesis	GO:0004497//GO:0005506//GO:0016020	nr +
Unigene111263_All	357	0.00	0.00	0.00	0.00	0.00	0.00	2.37	0.59	0.00	null	null		null	null		null	null		null	null		-2.02E+00	7.45E-03		-1.12E+01	2.21E-06	Down	Unigene111263_All//gi|17221648|dbj|BAB78478.1|//2.00E-18//preproMP73 [Cucurbita maxima]				ko03040|Spliceosome		nr +
Unigene111267_All	348	0.00	0.00	0.00	0.00	0.00	0.00	2.55	0.24	0.11	null	null		null	null		null	null		null	null		-3.41E+00	1.40E-04	Down	-4.48E+00	1.23E-05	Down	Unigene111267_All//gi|30694961|ref|NP_849791.1|//4.00E-15//myosin-related [Arabidopsis thaliana]	Unigene111267_All//4.00E-09//AT1G51405| myosin-related				GO:0043231	nr +
Unigene111271_All	612	0.34	1.74	0.00	0.49	0.00	0.59	3.32	0.82	1.11	2.35E+00	2.58E-03		-8.42E+00	1.46E-01		-8.93E+00	2.02E-02		2.74E-01	8.33E-01		-2.02E+00	8.03E-06	Down	-1.58E+00	1.35E-04	Down	Unigene111271_All//gi|28950899|gb|AAO63373.1|//2.00E-51//At2g27770 [Arabidopsis thaliana]	Unigene111271_All//1.00E-52//AT2G27770| unknown protein	Unigene111271_All//0.00E+00//TC139105			GO:0005622//GO:0008152	nr +
Unigene111274_All	464	0.00	0.00	0.00	0.00	0.00	0.00	5.65	0.18	0.00	null	null		null	null		null	null		null	null		-4.97E+00	7.56E-16	Down	-1.25E+01	2.73E-19	Down	Unigene111274_All//gi|192913040|gb|ACF06628.1|//3.00E-33//structural constituent of ribosome [Elaeis guineensis]	Unigene111274_All//4.00E-24//AT3G04920| 40S ribosomal protein S24 (RPS24A)			ko03010|Ribosome	GO:0030529	nr +
Unigene111276_All	367	0.00	0.00	0.00	0.00	0.00	0.00	6.80	0.57	3.15	null	null		null	null		null	null		null	null		-3.58E+00	2.77E-12	Down	-1.11E+00	1.54E-03								
Unigene111279_All	317	0.00	0.00	0.00	0.00	0.00	0.00	2.00	0.53	0.00	null	null		null	null		null	null		null	null		-1.92E+00	3.09E-02		-1.10E+01	7.12E-05	Down	Unigene111279_All//gi|99029149|gb|ABF60862.1|//5.00E-14//GRSF [Lilium longiflorum]	Unigene111279_All//4.00E-14//AT4G24770| RBP31 (31-KDA RNA BINDING PROTEIN); RNA binding / poly(U) binding			ko03040|Spliceosome	GO:0006952//GO:0008187//GO:0009526//GO:0009532//GO:0009534//GO:0010467//GO:0016070	nr +
Unigene111281_All	326	0.00	0.00	0.00	0.00	0.00	0.42	4.67	0.51	0.73	null	null		null	null		null	null		8.70E+00	3.12E-01		-3.19E+00	4.80E-07	Down	-2.67E+00	2.74E-06	Down	Unigene111281_All//gi|13111324|dbj|BAB32793.1|//1.00E-50//110 kDa 4SNc-Tudor domain protein [Pisum sativum]	Unigene111281_All//5.00E-45//AT5G07350| tudor domain-containing protein / nuclease family protein	Unigene111281_All//3.00E-85//TC152035			GO:0005488//GO:0016787//GO:0031332//GO:0035194	nr +
Unigene111283_All	507	0.62	0.50	0.00	0.20	0.14	0.45	2.34	1.15	0.55	-3.09E-01	7.89E-01		-9.27E+00	4.93E-02		-5.33E-01	8.18E-01		1.18E+00	5.50E-01		-1.02E+00	7.15E-02		-2.09E+00	5.32E-04	Down							ESTscan -
Unigene111284_All	754	0.00	0.00	0.00	0.00	0.00	0.00	3.87	0.22	0.16	null	null		null	null		null	null		null	null		-4.13E+00	8.13E-16	Down	-4.61E+00	1.44E-17	Down	Unigene111284_All//gi|29427830|sp|Q8LPB4.1|PSKR1_DAUCA//5.00E-70//RecName: Full=Phytosulfokine receptor 1; Short=DcPSKR1; AltName: Full=Phytosulfokine LRR receptor kinase 1; Flags: Precursor >gi|21623969|dbj|BAC00995.1| phytosulfokine receptor [Daucus carota]	Unigene111284_All//3.00E-67//AT2G02220| PSKR1 (PHYTOSULFOKIN RECEPTOR 1); ATP binding / peptide receptor/ protein serine/threonine kinase				GO:0003824//GO:0004872//GO:0005488	nr +
Unigene111288_All	874	0.18	0.06	0.00	0.00	0.00	0.00	2.18	0.91	0.59	-1.63E+00	4.89E-01		-7.49E+00	2.46E-01		null	null		null	null		-1.26E+00	3.27E-03		-1.88E+00	2.70E-05	Down	Unigene111288_All//gi|224074241|ref|XP_002304316.1|//1.00E-132//GRAS family transcription factor [Populus trichocarpa] >gi|222841748|gb|EEE79295.1| GRAS family transcription factor [Populus trichocarpa]	Unigene111288_All//2.00E-91//AT3G49950| scarecrow transcription factor family protein		GRAS		GO:0010468	nr -
Unigene111292_All	378	0.00	0.00	0.00	0.00	0.00	0.00	2.01	0.33	0.11	null	null		null	null		null	null		null	null		-2.60E+00	2.81E-03		-4.26E+00	8.65E-05	Down	Unigene111292_All//gi|224002995|ref|XP_002291169.1|//7.00E-37//translation factor tu domain 2 [Thalassiosira pseudonana CCMP1335] >gi|220972945|gb|EED91276.1| translation factor tu domain 2 [Thalassiosira pseudonana CCMP1335]	Unigene111292_All//2.00E-20//AT1G56070| LOS1; copper ion binding / translation elongation factor/ translation factor, nucleic acid binding	Unigene111292_All//7.00E-12//TC148998			GO:0008135//GO:0017111//GO:0032561	nr +
Unigene111294_All	308	1.19	0.00	0.35	1.13	0.22	1.32	3.98	1.63	0.52	-1.02E+01	1.97E-02		-1.78E+00	2.34E-01		-2.34E+00	1.01E-01		2.21E-01	8.63E-01		-1.29E+00	2.09E-02		-2.94E+00	1.14E-05	Down			Unigene111294_All//3.00E-109//ES845374				
Unigene111297_All	616	0.00	0.16	0.43	0.08	0.22	0.07	4.60	1.02	1.16	7.36E+00	3.90E-01		8.76E+00	8.03E-02		1.47E+00	6.20E-01		-1.42E-01	9.56E-01		-2.18E+00	1.48E-08	Down	-1.98E+00	5.62E-08	Down	Unigene111297_All//gi|38603902|gb|AAR24696.1|//1.00E-31//At4g32870 [Arabidopsis thaliana] >gi|38603986|gb|AAR24736.1| At4g32870 [Arabidopsis thaliana]	Unigene111297_All//2.00E-33//AT4G32870| unknown protein	Unigene111297_All//0.00E+00//TC146635				nr +
Unigene111298_All	541	0.00	0.00	0.00	0.00	0.00	0.00	3.83	0.46	0.29	null	null		null	null		null	null		null	null		-3.05E+00	5.12E-09	Down	-3.70E+00	5.61E-11	Down							
Unigene111299_All	533	0.00	0.00	0.00	0.00	0.00	0.00	3.25	1.57	0.00	null	null		null	null		null	null		null	null		-1.05E+00	1.87E-02		-1.17E+01	8.37E-13	Down	Unigene111299_All//gi|116222141|gb|ABJ80944.1|//1.00E-71//Hsp90 [Cyanophora paradoxa]	Unigene111299_All//3.00E-55//AT5G52640| ATHSP90.1 (HEAT SHOCK PROTEIN 90.1); ATP binding / unfolded protein binding	Unigene111299_All//8.00E-22//EV499016		ko04626|Plant-pathogen interaction//ko04141|Protein processing in endoplasmic reticulum	GO:0005515//GO:0032559//GO:0044267//GO:0044424//GO:0050896	nr +

Unigene111300_All	568	0.00	0.00	0.00	0.00	0.00	0.00	2.53	0.15	0.21	null	null		null	null		null	null		null	null		-4.10E+00	5.15E-08	Down	-3.59E+00	1.22E-07	Down	Unigene111300_All//gi|195655421|gb|ACG47178.1|//1.00E-17//GSDL-motif lipase [Zea mays]	Unigene111300_All//2.00E-13//AT2G23540| GDSL-motif lipase/hydrolase family protein				GO:0031410	nr +
Unigene111304_All	389	0.00	0.00	0.00	0.00	0.00	0.00	2.28	0.64	0.00	null	null		null	null		null	null		null	null		-1.82E+00	1.08E-02		-1.12E+01	1.10E-06	Down	Unigene111304_All//gi|301105230|ref|XP_002901699.1|//4.00E-39//40S ribosomal protein S11 [Phytophthora infestans T30-4] >gi|262100703|gb|EEY58755.1| 40S ribosomal protein S11 [Phytophthora infestans T30-4]	Unigene111304_All//3.00E-39//AT3G48930| EMB1080 (embryo defective 1080); structural constituent of ribosome			ko03010|Ribosome	GO:0003723//GO:0005198//GO:0010467//GO:0015935//GO:0030312	nr -
Unigene111305_All	355	0.00	0.00	0.00	0.28	0.00	0.00	1.91	0.94	0.11	null	null		null	null		-8.13E+00	3.16E-01		-8.13E+00	4.04E-01		-1.02E+00	2.04E-01		-4.09E+00	3.10E-04	Down							
Unigene111306_All	957	0.00	0.00	0.00	0.00	0.00	0.00	3.49	0.52	0.46	null	null		null	null		null	null		null	null		-2.74E+00	9.26E-13	Down	-2.93E+00	3.76E-14	Down	Unigene111306_All//gi|297853194|ref|XP_002894478.1|//3.00E-83//ALDH7B4 [Arabidopsis lyrata subsp. lyrata] >gi|297340320|gb|EFH70737.1| ALDH7B4 [Arabidopsis lyrata subsp. lyrata]	Unigene111306_All//6.00E-48//AT1G54100| ALDH7B4 (Aldehyde Dehydrogenase 7B4); 3-chloroallyl aldehyde dehydrogenase/ oxidoreductase			ko00010|Glycolysis / Gluconeogenesis//ko00903|Limonene and pinene degradation//ko00620|Pyruvate metabolism//ko00380|Tryptophan metabolism//ko00071|Fatty acid metabolism//ko00250|Alanine, aspartate and glutamate metabolism//ko00053|Ascorbate and aldarate metabolism//ko00330|Arginine and proline metabolism//ko00280|Valine, leucine and isoleucine degradation//ko00640|Propanoate metabolism//ko00561|Glycerolipid metabolism//ko00310|Lysine degradation//ko00350|Tyrosine metabolism//ko00650|Butanoate metabolism//ko00410|beta-Alanine metabolism//ko00340|Histidine metabolism	GO:0004030//GO:0006970//GO:0009414//GO:0009725//GO:0044237	nr -
Unigene111307_All	353	0.00	0.00	0.00	0.00	0.00	0.00	2.28	0.95	0.00	null	null		null	null		null	null		null	null		-1.26E+00	7.98E-02		-1.12E+01	4.43E-06	Down	Unigene111307_All//gi|192912958|gb|ACF06587.1|//7.00E-20//actin depolymerizing factor [Elaeis guineensis]	Unigene111307_All//2.00E-19//AT5G52360| ADF10 (ACTIN DEPOLYMERIZING FACTOR 10); actin binding				GO:0046872	nr +
Unigene111308_All	730	0.14	0.42	0.44	0.68	0.19	0.68	6.14	1.77	3.55	1.54E+00	3.24E-01		1.61E+00	3.05E-01		-1.85E+00	8.44E-02		-4.00E-03	9.87E-01		-1.79E+00	2.48E-10	Down	-7.91E-01	1.32E-03				Unigene111308_All//5.00E-67//TC140052				
Unigene111309_All	406	0.00	0.00	0.00	0.00	0.00	0.00	1.35	0.00	0.00	null	null		null	null		null	null		null	null		-1.04E+01	4.62E-04	Down	-1.04E+01	2.81E-04	Down							
Unigene111311_All	378	0.00	0.00	0.00	0.00	0.00	0.00	3.69	0.00	0.11	null	null		null	null		null	null		null	null		-1.18E+01	6.58E-10	Down	-5.13E+00	4.13E-09	Down	Unigene111311_All//gi|226501140|ref|NP_001152197.1|//3.00E-06//LOC100285835 [Zea mays] >gi|195653721|gb|ACG46328.1| keratin-associated protein 5-4 [Zea mays]						nr +
Unigene111312_All	402	0.00	0.00	0.00	0.00	0.00	0.00	1.79	0.00	0.00	null	null		null	null		null	null		null	null		-1.08E+01	3.27E-05	Down	-1.08E+01	1.78E-05	Down	Unigene111312_All//gi|115436514|ref|NP_001043015.1|//8.00E-32//Os01g0358400 [Oryza sativa Japonica Group] >gi|113532546|dbj|BAF04929.1| Os01g0358400 [Oryza sativa Japonica Group] >gi|149391309|gb|ABR25672.1| 40S ribosomal protein S4 [Oryza sativa Indica Group]	Unigene111312_All//7.00E-29//AT5G58420| 40S ribosomal protein S4 (RPS4D)			ko03010|Ribosome	GO:0003723//GO:0005198//GO:0010467//GO:0030529	nr +
Unigene111313_All	386	0.00	0.00	0.00	0.00	0.00	0.00	2.41	0.54	0.00	null	null		null	null		null	null		null	null		-2.15E+00	2.95E-03		-1.12E+01	5.48E-07	Down	Unigene111313_All//gi|222446462|dbj|BAH20861.1|//5.00E-07//NBS-LRR disease resistance protein [Oryza sativa Japonica Group] >gi|222446464|dbj|BAH20862.1| NBS-LRR disease resistance protein [Oryza sativa Japonica Group]	Unigene111313_All//3.00E-08//AT2G17440| leucine-rich repeat family protein					nr +
Unigene111314_All	374	0.00	0.00	0.00	0.00	0.00	0.00	2.60	0.56	0.00	null	null		null	null		null	null		null	null		-2.22E+00	1.82E-03		-1.13E+01	2.72E-07	Down	Unigene111314_All//gi|301111866|ref|XP_002905012.1|//1.00E-12//major vault protein [Phytophthora infestans T30-4] >gi|262095342|gb|EEY53394.1| major vault protein [Phytophthora infestans T30-4]						nr +
Unigene111315_All	358	0.00	0.00	1.04	0.56	0.19	0.13	1.77	0.12	0.89	null	null		1.00E+01	2.50E-02		-1.53E+00	4.16E-01		-2.14E+00	3.47E-01		-3.92E+00	9.81E-04	Down	-9.93E-01	2.02E-01		Unigene111315_All//gi|240255538|ref|NP_190402.6|//2.00E-30//ATM (ATAXIA-TELANGIECTASIA MUTATED); 1-phosphatidylinositol-3-phosphate 5-kinase [Arabidopsis thaliana]	Unigene111315_All//6.00E-32//AT3G48190| ATM (ATAXIA-TELANGIECTASIA MUTATED); 1-phosphatidylinositol-3-phosphate 5-kinase				GO:0016772//GO:0032559	nr +
Unigene111316_All	351	0.30	0.43	0.00	2.27	0.69	1.80	6.38	4.41	2.61	5.39E-01	7.80E-01		-8.22E+00	3.98E-01		-1.73E+00	2.86E-02		-3.34E-01	6.86E-01		-5.35E-01	1.71E-01		-1.29E+00	6.63E-04	Down			Unigene111316_All//4.00E-112//TC178205				
Unigene111317_All	545	0.19	0.00	0.00	0.37	0.19	0.41	6.44	1.15	0.73	-7.59E+00	3.76E-01		-7.59E+00	3.98E-01		-9.47E-01	5.88E-01		1.80E-01	9.35E-01		-2.48E+00	4.26E-12	Down	-3.14E+00	8.08E-16	Down	Unigene111317_All//gi|225468852|ref|XP_002266296.1|//1.00E-06//PREDICTED: hypothetical protein isoform 2 [Vitis vinifera]		Unigene111317_All//1.00E-14//gi|48803105|gb|CO104419.1|CO104419			GO:0008152//GO:0016491	nr +
Unigene111318_All	383	0.00	0.00	0.00	0.00	0.00	0.00	2.10	0.11	0.42	null	null		null	null		null	null		null	null		-4.26E+00	8.48E-05	Down	-2.33E+00	2.82E-03								
Unigene111319_All	387	28.26	27.50	28.48	96.67	109.98	77.14	1.31	0.00	0.21	-3.94E-02	8.31E-01		1.15E-02	9.72E-01		1.86E-01	2.60E-02		-3.26E-01	1.57E-04		-1.04E+01	8.90E-04	Down	-2.67E+00	1.37E-02		Unigene111319_All//gi|297825869|ref|XP_002880817.1|//2.00E-48//nitrate transporter [Arabidopsis lyrata subsp. lyrata] >gi|297326656|gb|EFH57076.1| nitrate transporter [Arabidopsis lyrata subsp. lyrata]	Unigene111319_All//3.00E-49//AT2G26690| nitrate transporter (NTP2)	Unigene111319_All//3.00E-147//TC140606			GO:0006950//GO:0009719//GO:0015833//GO:0031224	nr -
Unigene111320_All	330	0.00	0.00	0.00	0.00	0.00	0.00	2.95	3.42	0.48	null	null		null	null		null	null		null	null		2.15E-01	7.15E-01		-2.61E+00	3.36E-04	Down			Unigene111320_All//7.00E-15//ES843311				
Unigene111322_All	391	0.00	0.00	0.00	0.00	0.00	0.00	2.05	0.11	0.41	null	null		null	null		null	null		null	null		-4.26E+00	8.48E-05	Down	-2.33E+00	2.82E-03		Unigene111322_All//gi|15242725|ref|NP_195956.1|//4.00E-12//DC1 domain-containing protein [Arabidopsis thaliana]	Unigene111322_All//9.00E-14//AT4G13992| FUNCTIONS IN: molecular_function unknown; INVOLVED IN: intracellular signaling cascade; LOCATED IN: mitochondrion; CONTAINS InterPro DOMAIN/s: Protein kinase C, phorbol ester/diacylglycerol binding (InterPro:IPR002219), DC1 (InterPro:IPR004146), C1-like (InterPro:IPR011424); BEST Arabidopsis thaliana protein match is: DC1 domain-containing protein (TAIR:AT4G16015.1); Has 1471 Blast hits to 614 proteins in 36 species: Archae - 0; Bacteria - 0; Metazoa - 87; Fungi - 0; Plants - 1295; Viruses - 0; Other Eukaryotes - 89 (source: NCBI BLink).					nr +
Unigene111327_All	387	0.00	0.00	0.00	0.00	0.00	0.00	2.19	3.56	8.13	null	null		null	null		null	null		null	null		7.06E-01	1.71E-01		1.90E+00	3.27E-08	Up							
Unigene111328_All	629	0.08	0.00	0.51	0.32	0.22	0.50	3.70	0.53	0.57	-6.38E+00	6.18E-01		2.61E+00	1.46E-01		-5.33E-01	7.49E-01		6.66E-01	6.48E-01		-2.80E+00	2.78E-09	Down	-2.70E+00	2.63E-09	Down	Unigene111328_All//gi|255582386|ref|XP_002531982.1|//9.00E-06//conserved hypothetical protein [Ricinus communis] >gi|223528379|gb|EEF30418.1| conserved hypothetical protein [Ricinus communis]		Unigene111328_All//6.00E-66//ES815114				nr -
Unigene111329_All	742	0.14	0.00	0.00	0.13	0.19	0.91	5.24	0.96	0.97	-7.14E+00	3.76E-01		-7.14E+00	3.98E-01		4.67E-01	8.78E-01		2.77E+00	1.14E-02		-2.45E+00	3.80E-13	Down	-2.44E+00	1.54E-13	Down	Unigene111329_All//gi|48310064|gb|AAT41746.1|//6.00E-27//At4g28840 [Arabidopsis thaliana] >gi|50198849|gb|AAT70453.1| At4g28840 [Arabidopsis thaliana]	Unigene111329_All//1.00E-17//AT4G28840| unknown protein	Unigene111329_All//0.00E+00//gi|109870952|gb|DW499928.1|DW499928				nr +
Unigene111333_All	547	0.00	0.00	0.00	0.00	0.00	0.00	10.05	1.60	2.84	null	null		null	null		null	null		null	null		-2.65E+00	7.13E-20	Down	-1.82E+00	3.11E-13	Down							
Unigene111335_All	852	0.00	0.00	0.00	0.00	0.00	0.00	3.62	0.34	0.37	null	null		null	null		null	null		null	null		-3.40E+00	1.87E-14	Down	-3.28E+00	1.57E-14	Down	Unigene111335_All//gi|164564727|dbj|BAF98210.1|//3.00E-66//CM0216.210.nc [Lotus japonicus]	Unigene111335_All//8.00E-36//AT2G17080| unknown protein					nr -
Unigene111336_All	424	0.00	0.72	0.38	0.24	0.00	0.11	2.09	0.20	0.56	9.49E+00	4.45E-02		8.56E+00	2.40E-01		-7.88E+00	3.16E-01		-1.14E+00	6.78E-01		-3.41E+00	1.40E-04	Down	-1.89E+00	6.34E-03		Unigene111336_All//gi|223452393|gb|ACM89524.1|//9.00E-09//FERONIA receptor-like kinase [Glycine max]	Unigene111336_All//1.00E-06//AT5G28680| protein kinase family protein					nr -
Unigene111338_All	416	0.00	0.00	0.00	0.00	0.00	0.00	2.24	1.51	0.00	null	null		null	null		null	null		null	null		-5.69E-01	4.02E-01		-1.11E+01	5.48E-07	Down	Unigene111338_All//gi|301120388|ref|XP_002907921.1|//5.00E-08//polyadenylate-binding protein 1-B [Phytophthora infestans T30-4] >gi|262102952|gb|EEY61004.1| polyadenylate-binding protein 1-B [Phytophthora infestans T30-4]	Unigene111338_All//8.00E-07//AT2G23350| PAB4 (POLY(A) BINDING PROTEIN 4); RNA binding / translation initiation factor			ko03040|Spliceosome		nr -
Unigene111339_All	385	0.00	0.00	0.00	0.00	0.00	0.00	2.64	0.00	0.00	null	null		null	null		null	null		null	null		-1.14E+01	2.97E-07	Down	-1.14E+01	1.35E-07	Down	Unigene111339_All//gi|164564735|dbj|BAF98217.1|//2.00E-12//CM0216.330.nc [Lotus japonicus]						nr -
Unigene11134_All	2325	9.56	6.73	8.66	21.79	17.58	14.88	15.11	10.30	5.93	-5.06E-01	1.24E-05		-1.44E-01	2.86E-01		-3.10E-01	7.12E-06		-5.50E-01	1.85E-14		-5.53E-01	8.37E-12		-1.35E+00	6.27E-52	Down	Unigene11134_All//gi|115445577|ref|NP_001046568.1|//0.00E+00//Os02g0283800 [Oryza sativa Japonica Group] >gi|113536099|dbj|BAF08482.1| Os02g0283800 [Oryza sativa Japonica Group]	Unigene11134_All//0.00E+00//AT5G10290| leucine-rich repeat family protein / protein kinase family protein	Unigene11134_All//0.00E+00//ES829580			GO:0004672//GO:0006464//GO:0031224//GO:0032559//GO:0043231	nr -
Unigene111341_All	396	0.00	0.00	0.00	0.00	0.00	0.00	2.67	0.00	0.00	null	null		null	null		null	null		null	null		-1.14E+01	1.51E-07	Down	-1.14E+01	6.72E-08	Down	Unigene111341_All//gi|159487004|ref|XP_001701526.1|//2.00E-08//glyoxal or galactose oxidase [Chlamydomonas reinhardtii] >gi|158271587|gb|EDO97403.1| glyoxal or galactose oxidase [Chlamydomonas reinhardtii]				ko03430|Mismatch repair		nr +
Unigene111344_All	497	0.00	0.00	0.00	0.00	0.00	0.00	1.87	4.46	0.16	null	null		null	null		null	null		null	null		1.25E+00	1.44E-03		-3.55E+00	3.97E-05	Down			Unigene111344_All//2.00E-10//TC135460				
Unigene111345_All	1743	0.06	0.00	0.00	0.00	0.00	0.10	9.53	1.06	6.51	-5.91E+00	3.76E-01		-5.91E+00	3.98E-01		null	null		6.69E+00	1.95E-01		-3.18E+00	8.83E-71	Down	-5.49E-01	3.58E-06		Unigene111345_All//gi|224120220|ref|XP_002330994.1|//1.00E-143//cytochrome P450 [Populus trichocarpa] >gi|222872924|gb|EEF10055.1| cytochrome P450 [Populus trichocarpa]	Unigene111345_All//5.00E-130//AT5G06905| CYP712A2; electron carrier/ heme binding / iron ion binding / monooxygenase/ oxygen binding	Unigene111345_All//1.00E-07//TC137811		ko00945|Stilbenoid, diarylheptanoid and gingerol biosynthesis//ko00903|Limonene and pinene degradation//ko00941|Flavonoid biosynthesis//ko00944|Flavone and flavonol biosynthesis		nr +
Unigene111346_All	409	0.00	0.00	0.00	0.00	0.00	0.00	2.69	0.92	0.10	null	null		null	null		null	null		null	null		-1.55E+00	1.12E-02		-4.79E+00	4.55E-07	Down	Unigene111346_All//gi|298711489|emb|CBJ26577.1|//5.00E-24//40S ribosomal protein S17 [Ectocarpus siliculosus]	Unigene111346_All//2.00E-19//AT5G04800| 40S ribosomal protein S17 (RPS17D)			ko03010|Ribosome	GO:0005198//GO:0010467//GO:0030529	nr -

Unigene111347_All	582	0.00	0.00	0.00	0.00	0.00	0.00	10.32	0.14	2.05	null	null		null	null		null	null		null	null		-6.17E+00	3.77E-39	Down	-2.33E+00	2.04E-19	Down	Unigene111347_All//gi|15235406|ref|NP_192992.1|//4.00E-06//AIR1; lipid binding [Arabidopsis thaliana] >gi|15294192|gb|AAK95273.1|AF410287_1 AT4g12550/T1P17_140 [Arabidopsis thaliana] >gi|20147277|gb|AAM10352.1| AT4g12550/T1P17_140 [Arabidopsis thaliana]						nr -
Unigene111348_All	791	0.00	0.00	0.00	0.00	0.00	0.00	9.78	2.64	5.09	null	null		null	null		null	null		null	null		-1.89E+00	1.55E-18	Down	-9.43E-01	3.31E-07		Unigene111348_All//gi|288559172|sp|B9HBX2.1|U4978_POPTR//2.00E-68//RecName: Full=UPF0497 membrane protein 8	Unigene111348_All//6.00E-47//AT5G06200| integral membrane family protein				GO:0031224	nr -
Unigene111350_All	589	0.09	0.00	0.18	0.85	0.47	0.31	3.73	0.57	1.08	-6.47E+00	6.18E-01		1.03E+00	7.04E-01		-8.55E-01	4.07E-01		-1.46E+00	1.93E-01		-2.72E+00	1.45E-08	Down	-1.79E+00	1.17E-05	Down	Unigene111350_All//gi|3582434|dbj|BAA33062.1|//8.00E-62//low-molecular-weight heat shock protein [Cuscuta japonica]	Unigene111350_All//1.00E-43//AT5G59720| HSP18.2 (heat shock protein 18.2)	Unigene111350_All//0.00E+00//gi|109829300|gb|DW227888.1|DW227888			GO:0050896	nr +
Unigene111351_All	431	0.49	0.00	0.00	0.12	0.08	0.00	3.04	1.84	0.65	-8.92E+00	1.23E-01		-8.92E+00	1.46E-01		-5.33E-01	8.28E-01		-6.85E+00	6.43E-01		-7.23E-01	1.73E-01		-2.23E+00	1.19E-04	Down			Unigene111351_All//1.00E-124//gi|48794438|gb|CO095752.1|CO095752				
Unigene111352_All	724	2.31	0.28	3.31	2.75	2.05	1.93	0.58	0.00	0.17	-3.05E+00	3.81E-06	Down	5.17E-01	2.32E-01		-4.28E-01	3.61E-01		-5.09E-01	2.94E-01		-9.19E+00	3.25E-03		-1.82E+00	9.22E-02				Unigene111352_All//6.00E-08//TC133829				
Unigene111355_All	437	0.24	1.39	0.49	0.80	0.55	0.83	2.52	0.67	0.36	2.54E+00	2.46E-02		1.03E+00	5.92E-01		-5.33E-01	6.46E-01		5.13E-02	9.69E-01		-1.91E+00	2.64E-03		-2.79E+00	6.37E-05	Down	Unigene111355_All//gi|297814241|ref|XP_002875004.1|//1.00E-36//transcription factor jumonji domain-containing protein [Arabidopsis lyrata subsp. lyrata] >gi|297320841|gb|EFH51263.1| transcription factor jumonji domain-containing protein [Arabidopsis lyrata subsp. lyrata]	Unigene111355_All//1.00E-36//AT4G00990| transcription factor jumonji (jmjC) domain-containing protein	Unigene111355_All//9.00E-92//CA993153			GO:0005488	nr -
Unigene111356_All	614	0.00	0.00	0.00	0.00	0.00	0.00	9.57	0.88	0.32	null	null		null	null		null	null		null	null		-3.44E+00	3.00E-27	Down	-4.88E+00	5.10E-36	Down			Unigene111356_All//2.00E-13//TC146753				
Unigene111358_All	375	0.00	0.00	0.00	0.00	0.00	0.00	9.70	0.89	3.40	null	null		null	null		null	null		null	null		-3.44E+00	4.14E-17	Down	-1.51E+00	3.92E-07	Down	Unigene111358_All//gi|300263568|gb|EFJ47768.1|//6.00E-06//hypothetical protein VOLCADRAFT_117787 [Volvox carteri f. nagariensis]						nr +
Unigene11136_All	2603	13.33	6.93	10.72	15.25	16.22	16.19	17.57	8.32	10.68	-9.43E-01	3.06E-23		-3.14E-01	1.02E-03		8.86E-02	3.71E-01		8.59E-02	3.89E-01		-1.08E+00	3.57E-46	Down	-7.18E-01	2.82E-24		Unigene11136_All//gi|297811131|ref|XP_002873449.1|//0.00E+00//leucine-rich repeat family protein [Arabidopsis lyrata subsp. lyrata] >gi|297319286|gb|EFH49708.1| leucine-rich repeat family protein [Arabidopsis lyrata subsp. lyrata]	Unigene11136_All//0.00E+00//AT5G10290| leucine-rich repeat family protein / protein kinase family protein	Unigene11136_All//0.00E+00//TC157415		ko04626|Plant-pathogen interaction	GO:0004672//GO:0006464//GO:0031224//GO:0032559//GO:0043231	nr -
Unigene111360_All	594	0.00	0.00	0.00	0.00	0.00	0.00	3.20	1.48	0.00	null	null		null	null		null	null		null	null		-1.12E+00	8.54E-03		-1.16E+01	4.91E-14	Down	Unigene111360_All//gi|195626686|gb|ACG35173.1|//7.00E-58//60S ribosomal protein L12 [Zea mays]	Unigene111360_All//2.00E-57//AT2G37190| 60S ribosomal protein L12 (RPL12A)			ko03010|Ribosome	GO:0005198//GO:0010467//GO:0030529	nr +
Unigene111362_All	485	0.00	0.00	0.00	0.00	0.00	0.00	2.18	0.09	0.00	null	null		null	null		null	null		null	null		-4.66E+00	1.94E-06	Down	-1.11E+01	6.72E-08	Down							
Unigene111363_All	448	0.00	0.00	0.00	0.67	0.08	0.20	2.55	1.21	0.44	null	null		null	null		-3.12E+00	7.57E-02		-1.73E+00	2.89E-01		-1.07E+00	6.31E-02		-2.52E+00	1.20E-04	Down							
Unigene111365_All	711	0.00	0.00	0.00	0.00	0.00	0.00	4.10	0.29	0.22	null	null		null	null		null	null		null	null		-3.80E+00	6.00E-15	Down	-4.19E+00	1.34E-16	Down	Unigene111365_All//gi|301108189|ref|XP_002903176.1|//1.00E-70//60S ribosomal protein L19-1 [Phytophthora infestans T30-4] >gi|262097548|gb|EEY55600.1| 60S ribosomal protein L19-1 [Phytophthora infestans T30-4]	Unigene111365_All//3.00E-57//AT4G02230| 60S ribosomal protein L19 (RPL19C)	Unigene111365_All//1.00E-11//gi|109861980|gb|DW490957.1|DW490957		ko03010|Ribosome	GO:0005198//GO:0010467//GO:0030529//GO:0043231	nr -
Unigene111367_All	458	0.00	0.00	0.23	0.44	0.15	0.59	1.94	0.37	0.26	null	null		7.86E+00	4.01E-01		-1.53E+00	4.16E-01		4.44E-01	7.64E-01		-2.41E+00	1.79E-03		-2.89E+00	3.05E-04	Down	Unigene111367_All//gi|224143299|ref|XP_002324908.1|//1.00E-10//predicted protein [Populus trichocarpa] >gi|222866342|gb|EEF03473.1| predicted protein [Populus trichocarpa]						nr -
Unigene111368_All	775	0.74	0.20	0.21	0.06	0.27	0.41	2.73	1.02	0.62	-1.92E+00	7.01E-02		-1.85E+00	9.62E-02		2.05E+00	3.95E-01		2.67E+00	1.45E-01		-1.41E+00	5.54E-04	Down	-2.14E+00	1.01E-06	Down			Unigene111368_All//1.00E-163//TC150789				
Unigene111369_All	699	0.00	0.00	0.00	0.00	0.00	0.00	2.66	2.03	0.00	null	null		null	null		null	null		null	null		-3.89E-01	3.99E-01		-1.14E+01	9.97E-14	Down	Unigene111369_All//gi|195622794|gb|ACG33227.1|//1.00E-48//60S ribosomal protein L9 [Zea mays]	Unigene111369_All//2.00E-48//AT4G10450| 60S ribosomal protein L9 (RPL90D)			ko03010|Ribosome	GO:0005622	nr -
Unigene11137_All	436	0.60	0.58	0.49	0.46	1.03	3.31	5.04	10.93	11.15	-4.62E-02	9.87E-01		-2.97E-01	8.32E-01		1.17E+00	3.47E-01		2.86E+00	3.35E-05	Up	1.12E+00	8.86E-06	Up	1.14E+00	2.82E-06	Up	Unigene11137_All//gi|297824921|ref|XP_002880343.1|//6.00E-46//structural constituent of ribosome [Arabidopsis lyrata subsp. lyrata] >gi|297326182|gb|EFH56602.1| structural constituent of ribosome [Arabidopsis lyrata subsp. lyrata]	Unigene11137_All//1.00E-45//AT3G23590| RFR1 (REF4-related 1)	Unigene11137_All//2.00E-95//gi|78342988|gb|DT563262.1|DT563262				nr -
Unigene111370_All	419	0.00	0.00	0.00	0.00	0.00	0.00	2.42	1.10	0.00	null	null		null	null		null	null		null	null		-1.14E+00	6.62E-02		-1.12E+01	1.35E-07	Down	Unigene111370_All//gi|135404|sp|P11481.1|TBA1_VOLCA//9.00E-66//RecName: Full=Tubulin alpha-1/alpha-2 chain >gi|22001|emb|CAA31326.1| alpha-1 tubulin [Volvox carteri f. nagariensis] >gi|404089|gb|AAA99438.1| alpha-2 tubulin [Volvox carteri] >gi|300257467|gb|EFJ41715.1| alpha tubulin [Volvox carteri f. nagariensis] >gi|300263702|gb|EFJ47901.1| alpha tubulin [Volvox carteri f. nagariensis]	Unigene111370_All//2.00E-65//AT4G14960| TUA6; structural constituent of cytoskeleton	Unigene111370_All//6.00E-22//ES832636		ko04145|Phagosome	GO:0007017//GO:0015630//GO:0017111//GO:0032561//GO:0032991//GO:0043623	nr +
Unigene111371_All	428	1.47	0.12	0.12	1.05	0.32	0.42	1.88	0.39	0.28	-3.63E+00	5.07E-03		-3.56E+00	8.32E-03		-1.70E+00	1.35E-01		-1.31E+00	2.73E-01		-2.26E+00	4.82E-03		-2.75E+00	9.43E-04	Down			Unigene111371_All//1.00E-07//DW500060				
Unigene111373_All	801	0.00	0.00	0.00	0.00	0.00	0.00	0.79	1.04	0.00	null	null		null	null		null	null		null	null		3.98E-01	5.94E-01		-9.63E+00	7.12E-05	Down	Unigene111373_All//gi|298706583|emb|CBJ29542.1|//7.00E-23//ankyrin repeat protein [Ectocarpus siliculosus]	Unigene111373_All//5.00E-19//AT5G14230| protein binding					nr +
Unigene111375_All	683	0.00	0.00	0.00	0.00	0.00	0.00	4.70	0.67	0.35	null	null		null	null		null	null		null	null		-2.81E+00	1.30E-12	Down	-3.75E+00	7.58E-17	Down	Unigene111375_All//gi|49147195|ref|YP_025788.1|//3.00E-20//ATP synthase F0 subunit 6 [Pseudendoclonium akinetum] >gi|33439210|gb|AAQ18747.1| ATP synthase F0 subunit 6 [Pseudendoclonium akinetum]				ko00190|Oxidative phosphorylation	GO:0006754//GO:0015992//GO:0016020//GO:0044424	nr +
Unigene111377_All	449	0.00	0.34	0.24	1.22	0.15	0.00	1.98	0.74	0.27	8.40E+00	2.36E-01		7.89E+00	4.01E-01		-2.99E+00	9.39E-03		-1.03E+01	1.52E-03		-1.41E+00	4.01E-02		-2.89E+00	3.04E-04	Down							ESTscan -
Unigene111378_All	376	0.42	1.08	0.57	0.53	0.82	0.12	8.88	0.67	1.27	1.37E+00	2.74E-01		4.40E-01	7.76E-01		6.37E-01	6.95E-01		-2.14E+00	3.47E-01		-3.74E+00	8.20E-17	Down	-2.80E+00	1.46E-13	Down	Unigene111378_All//gi|224139620|ref|XP_002323196.1|//4.00E-08//argonaute protein group [Populus trichocarpa] >gi|222867826|gb|EEF04957.1| argonaute protein group [Populus trichocarpa]		Unigene111378_All//2.00E-09//TC138448				nr -
Unigene11138_All	1105	0.19	0.37	0.87	0.90	2.68	3.60	6.16	8.70	14.82	9.54E-01	4.14E-01		2.20E+00	9.10E-03		1.57E+00	2.01E-05	Up	2.00E+00	4.55E-09	Up	4.98E-01	2.63E-03		1.27E+00	3.10E-22	Up	Unigene11138_All//gi|297824921|ref|XP_002880343.1|//1.00E-68//structural constituent of ribosome [Arabidopsis lyrata subsp. lyrata] >gi|297326182|gb|EFH56602.1| structural constituent of ribosome [Arabidopsis lyrata subsp. lyrata]	Unigene11138_All//1.00E-69//AT2G48110| REF4 (REDUCED EPIDERMAL FLUORESCENCE 4)	Unigene11138_All//0.00E+00//TC138318				nr +
Unigene111380_All	503	0.00	0.00	0.00	0.00	0.00	0.00	3.11	0.50	0.00	null	null		null	null		null	null		null	null		-2.64E+00	4.08E-06	Down	-1.16E+01	1.42E-11	Down	Unigene111380_All//gi|1706581|sp|P54959.1|EF1A_BLAHO//2.00E-66//RecName: Full=Elongation factor 1-alpha; Short=EF-1-alpha >gi|984790|dbj|BAA10962.1| elongation factor 1alpha [Blastocystis hominis]	Unigene111380_All//2.00E-47//AT5G60390| elongation factor 1-alpha / EF-1-alpha	Unigene111380_All//4.00E-17//TC166357			GO:0006412//GO:0008135//GO:0017111//GO:0032561//GO:0044424	nr -
Unigene111381_All	398	0.00	0.00	0.00	0.00	0.00	0.00	8.29	0.53	0.60	null	null		null	null		null	null		null	null		-3.98E+00	2.17E-17	Down	-3.79E+00	2.10E-17	Down	Unigene111381_All//gi|27923849|sp|Q945U1.1|RS15_ELAOL//7.00E-42//RecName: Full=40S ribosomal protein S15 >gi|15425963|gb|AAK97632.1| 40S ribosomal protein S15 [Elaeis oleifera] >gi|192910788|gb|ACF06502.1| 40S ribosomal protein S15 [Elaeis guineensis]	Unigene111381_All//5.00E-32//AT5G09500| 40S ribosomal protein S15 (RPS15C)	Unigene111381_All//5.00E-07//BG444152		ko03010|Ribosome	GO:0003676//GO:0005198//GO:0010467//GO:0033279	nr -
Unigene111385_All	449	0.00	0.11	0.47	1.11	1.00	0.50	3.48	1.30	0.44	6.82E+00	6.21E-01		8.89E+00	1.42E-01		-1.54E-01	8.50E-01		-1.14E+00	3.07E-01		-1.42E+00	3.64E-03		-2.97E+00	4.24E-07	Down							
Unigene111386_All	831	0.00	0.00	0.00	0.00	0.04	0.11	3.61	0.25	0.62	null	null		null	null		5.37E+00	8.37E-01		6.76E+00	4.80E-01		-3.84E+00	1.74E-15	Down	-2.54E+00	4.57E-11	Down	Unigene111386_All//gi|260401291|gb|ACX37122.1|//4.00E-06//ORF1 [Pisum sativum]		Unigene111386_All//1.00E-09//FL577667				nr -
Unigene111387_All	422	0.00	0.00	0.00	0.00	0.00	0.00	3.01	0.10	0.19	null	null		null	null		null	null		null	null		-4.92E+00	7.77E-08	Down	-3.99E+00	2.56E-07	Down	Unigene111387_All//sp|Q92954|PRG4_HUMAN//2.00E-06//Proteoglycan 4 OS=Homo sapiens GN=PRG4 PE=1 SV=2						swissprot +
Unigene111389_All	471	0.00	0.00	0.00	0.00	0.00	0.00	2.33	0.18	0.00	null	null		null	null		null	null		null	null		-3.72E+00	7.15E-06	Down	-1.12E+01	3.33E-08	Down	Unigene111389_All//gi|223995731|ref|XP_002287539.1|//7.00E-52//cyclophilin-type peptidyl-prolyl cis-trans isomerase [Thalassiosira pseudonana CCMP1335] >gi|220976655|gb|EED94982.1| cyclophilin-type peptidyl-prolyl cis-trans isomerase [Thalassiosira pseudonana CCMP1335]	Unigene111389_All//3.00E-42//AT2G29960| CYP5 (CYCLOPHILIN 5); peptidyl-prolyl cis-trans isomerase	Unigene111389_All//7.00E-25//gi|78351431|gb|DT571705.1|DT571705			GO:0016859//GO:0044267	nr +
Unigene111393_All	506	0.00	0.00	0.00	0.00	0.00	0.00	1.67	0.08	0.00	null	null		null	null		null	null		null	null		-4.34E+00	4.55E-05	Down	-1.07E+01	2.21E-06	Down	Unigene111393_All//gi|266706161|gb|ACY78390.1|//2.00E-10//protein tyrosine phosphatase 1 [Phalaenopsis amabilis]	Unigene111393_All//9.00E-08//AT1G71860| PTP1 (PROTEIN TYROSINE PHOSPHATASE 1); protein tyrosine phosphatase				GO:0016791	nr -
Unigene111395_All	1181	0.00	0.00	0.00	0.00	0.00	0.00	9.42	0.07	0.00	null	null		null	null		null	null		null	null		-7.06E+00	4.36E-75	Down	-1.32E+01	6.27E-82	Down							ESTscan -
Unigene111397_All	452	0.00	0.00	0.00	0.00	0.00	0.00	8.33	0.00	0.35	null	null		null	null		null	null		null	null		-1.30E+01	1.28E-26	Down	-4.56E+00	2.21E-22	Down	Unigene111397_All//gi|301117182|ref|XP_002906319.1|//2.00E-41//60S ribosomal protein L27 [Phytophthora infestans T30-4] >gi|262107668|gb|EEY65720.1| 60S ribosomal protein L27 [Phytophthora infestans T30-4]	Unigene111397_All//4.00E-31//AT3G22230| 60S ribosomal protein L27 (RPL27B)	Unigene111397_All//2.00E-09//gi|78337699|gb|DT557973.1|DT557973		ko03010|Ribosome	GO:0030529	nr +
Unigene111398_All	528	0.40	0.29	0.00	1.13	0.20	0.17	2.24	0.95	0.53	-4.61E-01	7.58E-01		-8.63E+00	1.46E-01		-2.53E+00	1.36E-02		-2.73E+00	1.74E-02		-1.24E+00	2.93E-02		-2.09E+00	5.32E-04	Down			Unigene111398_All//6.00E-07//TC141683				

Unigene111399_All	492	0.00	0.00	0.00	0.00	0.00	0.00	2.75	1.61	0.65	null	null		null	null		null	null		null	null		-7.69E-01	1.39E-01		-2.09E+00	1.85E-04	Down	Unigene111399_All//gi|297824773|ref|XP_002880269.1|//7.00E-32//ATP binding protein [Arabidopsis lyrata subsp. lyrata] >gi|297326108|gb|EFH56528.1| ATP binding protein [Arabidopsis lyrata subsp. lyrata]	Unigene111399_All//7.00E-31//AT2G46850| ATP binding / protein kinase/ protein tyrosine kinase				GO:0004672//GO:0006464//GO:0032559	nr -
Unigene111400_All	494	0.00	0.00	0.00	0.00	0.00	0.00	2.65	0.17	2.10	null	null		null	null		null	null		null	null		-3.97E+00	3.35E-07	Down	-3.40E-01	5.21E-01		Unigene111400_All//gi|164564727|dbj|BAF98210.1|//6.00E-33//CM0216.210.nc [Lotus japonicus]	Unigene111400_All//6.00E-19//AT4G35200| unknown protein					nr -
Unigene111401_All	1262	0.08	0.16	0.08	0.28	0.00	0.07	3.55	2.29	1.58	9.54E-01	6.22E-01		2.59E-02	1.00E+00		-8.11E+00	9.46E-03		-1.95E+00	1.96E-01		-6.36E-01	1.26E-02		-1.17E+00	4.38E-06	Down							
Unigene111402_All	817	0.00	0.00	0.00	0.00	0.00	0.00	3.57	0.46	0.39	null	null		null	null		null	null		null	null		-2.96E+00	4.23E-12	Down	-3.19E+00	1.79E-13	Down	Unigene111402_All//gi|115489150|ref|NP_001067062.1|//9.00E-95//Os12g0567700 [Oryza sativa Japonica Group] >gi|113649569|dbj|BAF30081.1| Os12g0567700 [Oryza sativa Japonica Group]	Unigene111402_All//2.00E-88//AT4G36130| 60S ribosomal protein L8 (RPL8C)	Unigene111402_All//3.00E-10//gi|78337755|gb|DT558029.1|DT558029		ko03010|Ribosome	GO:0005198//GO:0010467//GO:0015934//GO:0016020//GO:0043231	nr -
Unigene111404_All	490	0.00	0.00	0.00	0.00	0.00	0.00	6.47	1.28	1.54	null	null		null	null		null	null		null	null		-2.34E+00	2.70E-10	Down	-2.07E+00	2.80E-09	Down	Unigene111404_All//gi|224140285|ref|XP_002323513.1|//1.00E-17//amino acid transporter [Populus trichocarpa] >gi|222868143|gb|EEF05274.1| amino acid transporter [Populus trichocarpa]	Unigene111404_All//2.00E-16//AT5G02170| amino acid transporter family protein				GO:0005275//GO:0009536//GO:0016020	nr +
Unigene111405_All	575	0.00	0.00	0.00	0.26	0.12	0.16	2.43	0.07	1.52	null	null		null	null		-1.12E+00	5.85E-01		-7.26E-01	7.16E-01		-5.06E+00	1.10E-08	Down	-6.71E-01	1.68E-01		Unigene111405_All//gi|116310721|emb|CAH67518.1|//1.00E-29//OSIGBa0092E01.13 [Oryza sativa (indica cultivar-group)]	Unigene111405_All//3.00E-18//AT5G57770| FUNCTIONS IN: molecular_function unknown; INVOLVED IN: biological_process unknown; LOCATED IN: cellular_component unknown; EXPRESSED IN: root; CONTAINS InterPro DOMAIN/s: Pleckstrin-like, plant (InterPro:IPR013666), Protein of unknown function DUF828, plant (InterPro:IPR008546); Has 104 Blast hits to 104 proteins in 9 species: Archae - 0; Bacteria - 2; Metazoa - 0; Fungi - 0; Plants - 102; Viruses - 0; Other Eukaryotes - 0 (source: NCBI BLink).	Unigene111405_All//1.00E-69//CA993859				nr -
Unigene111406_All	651	0.00	0.00	0.00	0.00	0.00	0.00	2.27	0.06	0.24	null	null		null	null		null	null		null	null		-5.15E+00	2.98E-09	Down	-3.22E+00	3.28E-07	Down	Unigene111406_All//gi|15237193|ref|NP_197691.1|//1.00E-22//MYB37 (MYB DOMAIN PROTEIN 37); DNA binding / transcription factor [Arabidopsis thaliana] >gi|75333792|sp|Q9FG68.1|RAX1_ARATH RecName: Full=Transcription factor RAX1; AltName: Full=Protein REGULATOR OF AXILLARY MERISTEMS 1; AltName: Full=Myb-related protein 37; Short=AtMYB37 >gi|9758402|dbj|BAB08873.1| myb-related transcription factor-like [Arabidopsis thaliana] >gi|18072740|emb|CAC80101.1| R2R3-MYB transcription factor [Arabidopsis thaliana] >gi|41619420|gb|AAS10098.1| MYB transcription factor [Arabidopsis thaliana] >gi|51969156|dbj|BAD43270.1| myb like transcription factor (MYB37) [Arabidopsis thaliana] >gi|91806896|gb|ABE66175.1| myb family transcription factor [Arabidopsis thaliana]	Unigene111406_All//5.00E-23//AT5G23000| MYB37 (MYB DOMAIN PROTEIN 37); DNA binding / transcription factor		MYB		GO:0006350//GO:0010468	nr -
Unigene111407_All	539	0.29	0.19	1.28	0.18	0.00	0.17	4.16	1.01	0.07	-6.31E-01	7.28E-01		2.14E+00	3.67E-02		-7.53E+00	3.16E-01		-1.41E-01	9.52E-01		-2.04E+00	1.89E-06	Down	-5.81E+00	4.52E-15	Down							
Unigene111409_All	562	0.00	0.00	0.00	0.00	0.00	0.00	3.76	0.00	0.07	null	null		null	null		null	null		null	null		-1.19E+01	5.89E-15	Down	-5.73E+00	3.61E-14	Down							ESTscan -
Unigene111411_All	553	0.00	0.00	0.00	0.00	0.00	0.00	2.37	1.13	0.14	null	null		null	null		null	null		null	null		-1.06E+00	4.42E-02		-4.04E+00	1.35E-07	Down							ESTscan +
Unigene111412_All	542	0.00	0.00	0.00	0.00	0.00	0.00	2.50	0.54	0.07	null	null		null	null		null	null		null	null		-2.21E+00	1.58E-04	Down	-5.09E+00	8.12E-09	Down	Unigene111412_All//gi|297809931|ref|XP_002872849.1|//2.00E-32//60S ribosomal protein L19 [Arabidopsis lyrata subsp. lyrata] >gi|297318686|gb|EFH49108.1| 60S ribosomal protein L19 [Arabidopsis lyrata subsp. lyrata]	Unigene111412_All//1.00E-14//AT4G02230| 60S ribosomal protein L19 (RPL19C)			ko03010|Ribosome	GO:0005198//GO:0009987//GO:0010467//GO:0015934//GO:0016020//GO:0031981	nr +
Unigene111414_All	592	0.18	0.00	0.81	1.26	0.52	0.69	2.79	0.35	0.13	-7.47E+00	3.76E-01		2.19E+00	9.15E-02		-1.27E+00	1.12E-01		-8.78E-01	3.08E-01		-2.98E+00	3.67E-07	Down	-4.37E+00	7.28E-10	Down	Unigene111414_All//gi|15230954|ref|NP_189226.1|//3.00E-59//pentatricopeptide (PPR) repeat-containing protein [Arabidopsis thaliana]	Unigene111414_All//1.00E-60//AT3G25970| pentatricopeptide (PPR) repeat-containing protein					nr +
Unigene111415_All	564	0.28	0.00	0.66	8.04	5.37	3.36	10.79	2.37	5.93	-8.12E+00	2.16E-01		1.25E+00	3.66E-01		-5.81E-01	3.32E-02		-1.26E+00	1.07E-05	Down	-2.19E+00	1.13E-17	Down	-8.63E-01	3.59E-05		Unigene111415_All//gi|211906540|gb|ACJ11763.1|//1.00E-23//class III peroxidase [Gossypium hirsutum]	Unigene111415_All//6.00E-19//AT4G30170| peroxidase, putative	Unigene111415_All//1.00E-94//TC133431		ko00940|Phenylpropanoid biosynthesis//ko00360|Phenylalanine metabolism	GO:0003824//GO:0005506//GO:0006950//GO:0008152//GO:0016209//GO:0031410	nr -
Unigene111417_All	623	0.84	0.00	0.17	0.16	0.11	0.00	14.66	7.92	0.58	-9.71E+00	2.93E-03		-2.30E+00	6.77E-02		-5.33E-01	8.18E-01		-7.32E+00	4.04E-01		-8.89E-01	2.25E-07		-4.67E+00	2.38E-54	Down	Unigene111417_All//gi|18397934|ref|NP_565380.1|//8.00E-26//glycine-rich protein [Arabidopsis thaliana] >gi|15294186|gb|AAK95270.1|AF410284_1 At2g15780/F19G14.22 [Arabidopsis thaliana] >gi|5306253|gb|AAD41986.1| expressed protein [Arabidopsis thaliana] >gi|20147265|gb|AAM10346.1| At2g15780/F19G14.22 [Arabidopsis thaliana]	Unigene111417_All//4.00E-27//AT2G15780| glycine-rich protein					nr +
Unigene111418_All	1206	1.30	0.59	1.10	0.95	0.40	0.34	1.33	0.55	0.40	-1.15E+00	3.18E-02		-2.38E-01	6.42E-01		-1.25E+00	4.15E-02		-1.50E+00	2.37E-02		-1.26E+00	7.61E-03		-1.75E+00	2.97E-04	Down	Unigene111418_All//gi|297789604|ref|XP_002862749.1|//2.00E-83//myb family transcription factor [Arabidopsis lyrata subsp. lyrata] >gi|297308457|gb|EFH39007.1| myb family transcription factor [Arabidopsis lyrata subsp. lyrata]	Unigene111418_All//2.00E-58//AT5G45420| myb family transcription factor	Unigene111418_All//0.00E+00//TC143540	MYB-related		GO:0016020//GO:0043231	nr +
Unigene111420_All	603	0.00	0.00	0.00	0.00	0.00	0.00	4.07	0.07	0.00	null	null		null	null		null	null		null	null		-5.87E+00	7.01E-16	Down	-1.20E+01	4.73E-18	Down	Unigene111420_All//gi|159476204|ref|XP_001696201.1|//9.00E-48//ribosomal protein S8, component of cytosolic 80S ribosome and 40S small subunit [Chlamydomonas reinhardtii] >gi|158282426|gb|EDP08178.1| ribosomal protein S8 [Chlamydomonas reinhardtii]	Unigene111420_All//4.00E-43//AT5G59240| 40S ribosomal protein S8 (RPS8B)			ko03010|Ribosome	GO:0003723//GO:0005198//GO:0009536//GO:0010467//GO:0030529	nr -
Unigene111421_All	1158	0.00	0.00	0.00	0.00	0.00	0.00	7.74	0.07	0.38	null	null		null	null		null	null		null	null		-6.74E+00	6.86E-60	Down	-4.35E+00	4.18E-51	Down	Unigene111421_All//gi|169137009|gb|ABS86614.2|//1.00E-10//latex protein [Morus alba]	Unigene111421_All//5.00E-05//AT3G04720| PR4 (PATHOGENESIS-RELATED 4); chitin binding					nr +
Unigene111423_All	1413	0.00	0.00	0.00	0.00	0.00	0.00	3.56	0.12	0.06	null	null		null	null		null	null		null	null		-4.91E+00	6.78E-30	Down	-5.98E+00	9.91E-34	Down	Unigene111423_All//gi|298705968|emb|CBJ29089.1|//5.00E-38//MORN repeat variant family protein [Ectocarpus siliculosus]	Unigene111423_All//1.00E-29//AT1G60890| phosphatidylinositol-4-phosphate 5-kinase family protein			ko04144|Endocytosis//ko04070|Phosphatidylinositol signaling system//ko00562|Inositol phosphate metabolism	GO:0003779//GO:0016307	nr +
Unigene111425_All	735	0.00	0.00	0.00	0.00	0.00	0.00	8.46	0.11	1.14	null	null		null	null		null	null		null	null		-6.22E+00	1.26E-40	Down	-2.89E+00	2.15E-25	Down	Unigene111425_All//gi|18414906|ref|NP_568156.1|//4.00E-46//pollen Ole e 1 allergen and extensin family protein [Arabidopsis thaliana] >gi|111074158|gb|ABH04452.1| At5g05500 [Arabidopsis thaliana]	Unigene111425_All//1.00E-46//AT5G05500| pollen Ole e 1 allergen and extensin family protein				GO:0044464	nr +
Unigene111426_All	832	0.00	0.00	0.00	0.00	0.00	0.00	1.27	0.20	1.05	null	null		null	null		null	null		null	null		-2.66E+00	2.25E-04	Down	-2.70E-01	6.43E-01		Unigene111426_All//gi|15230628|ref|NP_187896.1|//2.00E-55//oxidoreductase, 2OG-Fe(II) oxygenase family protein [Arabidopsis thaliana] >gi|91806419|gb|ABE65937.1| oxidoreductase 2OG-Fe(II) oxygenase family protein [Arabidopsis thaliana]	Unigene111426_All//2.00E-50//AT3G12900| oxidoreductase, 2OG-Fe(II) oxygenase family protein	Unigene111426_All//1.00E-104//TC134433		ko00270|Cysteine and methionine metabolism	GO:0008152//GO:0016701//GO:0016706	nr +
Unigene111427_All	849	0.00	0.00	0.00	0.00	0.00	0.00	4.18	0.89	0.70	null	null		null	null		null	null		null	null		-2.24E+00	6.71E-11	Down	-2.57E+00	3.78E-13	Down	Unigene111427_All//gi|226510214|ref|NP_001149016.1|//9.00E-14//dnaJ subfamily B member 5 [Zea mays] >gi|195624008|gb|ACG33834.1| dnaJ subfamily B member 5 [Zea mays]	Unigene111427_All//2.00E-12//AT4G28480| DNAJ heat shock family protein				GO:0005515//GO:0031410	nr +
Unigene11143_All	1661	3.24	7.23	4.81	5.19	4.87	4.30	19.80	27.73	28.95	1.16E+00	1.45E-11	Up	5.68E-01	8.03E-03		-9.08E-02	7.04E-01		-2.72E-01	2.06E-01		4.86E-01	3.50E-12		5.48E-01	5.34E-16		Unigene11143_All//gi|3776567|gb|AAC64884.1|//0.00E+00//Strong similarity to F21B7.33 gi|2809264 from A. thaliana BAC gb|AC002560. EST gb|N65119 comes from this gene [Arabidopsis thaliana]	Unigene11143_All//4.00E-180//AT1G54710| AtATG18h	Unigene11143_All//0.00E+00//DN780615			GO:0006464//GO:0006950//GO:0009536//GO:0016301//GO:0032559	nr +
Unigene111430_All	1749	0.00	0.00	0.00	0.00	0.00	0.00	15.81	0.43	0.66	null	null		null	null		null	null		null	null		-5.20E+00	1.01E-167	Down	-4.58E+00	8.55E-162	Down	Unigene111430_All//gi|224113191|ref|XP_002332632.1|//0.00E+00//cytochrome P450 [Populus trichocarpa] >gi|222832859|gb|EEE71336.1| cytochrome P450 [Populus trichocarpa]	Unigene111430_All//0.00E+00//AT3G52970| CYP76G1; electron carrier/ heme binding / iron ion binding / monooxygenase/ oxygen binding			ko00940|Phenylpropanoid biosynthesis//ko00945|Stilbenoid, diarylheptanoid and gingerol biosynthesis//ko00903|Limonene and pinene degradation//ko00941|Flavonoid biosynthesis//ko00360|Phenylalanine metabolism//ko00944|Flavone and flavonol biosynthesis	GO:0005506//GO:0008152//GO:0016709	nr -
Unigene111433_All	200	0.00	0.00	0.00	0.00	0.00	0.00	4.23	0.21	1.79	null	null		null	null		null	null		null	null		-4.34E+00	4.55E-05	Down	-1.24E+00	6.26E-02								
Unigene111436_All	200	0.00	0.00	0.00	0.25	0.69	0.00	4.86	0.00	0.00	null	null		null	null		1.47E+00	6.20E-01		-7.96E+00	6.43E-01		-1.22E+01	5.84E-07	Down	-1.22E+01	2.72E-07	Down			Unigene111436_All//9.00E-13//EX169598				
Unigene11144_All	3708	2.03	8.53	2.38	3.24	2.93	3.78	17.23	32.65	52.09	2.07E+00	2.41E-67	Up	2.31E-01	2.89E-01		-1.42E-01	4.52E-01		2.22E-01	1.84E-01		9.22E-01	2.55E-93		1.60E+00	0.00E+00	Up	Unigene11144_All//gi|18405032|ref|NP_564664.1|//0.00E+00//AtATG18h [Arabidopsis thaliana]	Unigene11144_All//0.00E+00//AT1G54710| AtATG18h	Unigene11144_All//0.00E+00//DN780615			GO:0000166//GO:0006950//GO:0043231	nr +
Unigene11145_All	3079	3.31	7.85	4.00	3.64	4.42	4.08	20.35	23.81	23.43	1.24E+00	3.41E-25	Up	2.70E-01	1.31E-01		2.79E-01	7.71E-02		1.64E-01	3.93E-01		2.27E-01	3.93E-05		2.03E-01	1.98E-04		Unigene11145_All//gi|18405032|ref|NP_564664.1|//0.00E+00//AtATG18h [Arabidopsis thaliana]	Unigene11145_All//0.00E+00//AT1G54710| AtATG18h	Unigene11145_All//0.00E+00//CO083391			GO:0000166//GO:0006950//GO:0043231	nr -

Unigene111458_All	200	8.89	3.55	3.20	14.20	6.20	6.32	32.98	22.57	27.29	-1.33E+00	8.42E-03		-1.48E+00	5.78E-03		-1.20E+00	6.51E-04	Down	-1.17E+00	1.74E-03		-5.47E-01	7.52E-03		-2.73E-01	1.94E-01		Unigene111458_All//gi|15234480|ref<br>|NP_192395.1|//9.00E-31//DER2.2<br>(DERLIN-2.2) [Arabidopsis<br>thaliana]<br>>gi|75216465|sp|Q9ZS88.1|DER22_ARA<br>TH RecName: Full=Derlin-2.2;<br>AltName: Full=AtDerlin2-2<br>>gi|14488073|gb|AAK63857.1|AF38928<br>4_1 AT4g04860/T4B21_2 [Arabidopsis<br>thaliana]<br>>gi|4115936|gb|AAD03446.1| T4B21.2<br>gene product [Arabidopsis<br>thaliana]<br>>gi|20147127|gb|AAM10280.1| AT4g04860/T4B21_2 [Arabidopsis<br>thaliana]	Unigene111458_All//5.00E-<br>23//AT4G04860| DER2.2 (DERLIN-2.2)	Unigene111458_All//5.00E-107//TC135552			GO:0019538//GO:0031090//GO:0031224<br>//GO:0043231//GO:0050896	nr -
Unigene111459_All	200	0.00	0.00	0.00	0.00	0.00	0.00	3.17	0.00	0.00	null	null		null	null		null	null		null	null		-1.16E+01	1.23E-04	Down	-1.16E+01	7.12E-05	Down	Unigene111459_All//gi|300521738|gb<br>|ADK26058.1|//4.00E-15//drought<br>resistance protein [Glycine max]	Unigene111459_All//2.00E-<br>12//AT4G02270| pollen Ole e 1<br>allergen and extensin family<br>protein					nr +
Unigene111464_All	201	0.26	0.00	0.26	0.00	0.34	0.67	2.94	0.00	0.40	-8.02E+00	6.18E-01		2.53E-02	9.96E-01		8.42E+00	6.14E-01		9.40E+00	3.12E-01		-1.15E+01	2.39E-04	Down	-2.89E+00	4.54E-03				Unigene111464_All//8.00E-<br>106//gi|109884320|gb|DW513290.1|DW513290				
Unigene111466_All	201	0.00	0.00	0.00	0.00	0.00	0.00	2.94	0.00	0.00	null	null		null	null		null	null		null	null		-1.15E+01	2.39E-04	Down	-1.15E+01	1.41E-04	Down	Unigene111466_All//gi|10443488|gb| AAG17477.1|AF106846_1//3.00E-<br>20//QM protein [Oryza sativa<br>Indica Group]	Unigene111466_All//1.00E-<br>20//AT1G14320| SAC52 (SUPPRESSOR<br>OF ACAULIS 52); structural<br>constituent of ribosome			ko03010|Ribosome	GO:0005198//GO:0010467//GO:0030529	nr -
Unigene111483_All	201	0.00	0.00	0.00	0.00	0.00	0.00	3.37	0.62	0.00	null	null		null	null		null	null		null	null		-2.43E+00	7.77E-03		-1.17E+01	3.57E-05	Down	Unigene111483_All//gi|300256601|gb<br>|EFJ40863.1|//8.00E-06//component<br>of cytosolic 80S ribosome and 60S<br>large subunit [Volvox carteri f.<br>nagariensis]				ko03010|Ribosome		nr -
Unigene111485_All	201	0.00	0.00	0.00	0.00	0.00	0.00	9.05	0.00	0.20	null	null		null	null		null	null		null	null		-1.31E+01	7.10E-13	Down	-5.51E+00	4.49E-12	Down			Unigene111485_All//2.00E-07//TC142576				
Unigene111487_All	201	0.52	0.00	0.00	1.49	0.34	0.00	2.52	0.62	0.00	-9.02E+00	3.76E-01		-9.02E+00	3.98E-01		-2.12E+00	1.66E-01		-1.05E+01	4.01E-02		-2.02E+00	5.21E-02		-1.13E+01	5.55E-04	Down							
Unigene111490_All	201	0.00	0.00	0.00	0.00	0.00	0.00	16.41	1.04	0.59	null	null		null	null		null	null		null	null		-3.98E+00	2.17E-17	Down	-4.79E+00	3.31E-20	Down							
Unigene111500_All	202	0.00	0.00	0.00	0.00	0.00	0.00	2.72	0.21	0.00	null	null		null	null		null	null		null	null		-3.72E+00	3.23E-03		-1.14E+01	2.81E-04	Down							ESTscan -
Unigene111509_All	202	0.00	0.00	0.00	0.00	0.00	0.00	2.72	0.21	0.00	null	null		null	null		null	null		null	null		-3.72E+00	3.23E-03		-1.14E+01	2.81E-04	Down							ESTscan -
Unigene11151_All	593	2.82	0.17	1.98	4.20	5.05	3.20	4.28	1.76	1.68	-4.05E+00	1.45E-07	Down	-5.15E-01	3.44E-01		2.66E-01	5.31E-01		-3.93E-01	3.72E-01		-1.28E+00	4.33E-04	Down	-1.35E+00	1.51E-04	Down	Unigene11151_All//gi|2444174|gb|AA<br>B71526.1|//3.00E-<br>90//unconventional myosin<br>[Helianthus annuus]	Unigene11151_All//2.00E-<br>89//AT1G50360| VIIIA; motor	Unigene11151_All//0.00E+00//ES791939			GO:0015629//GO:0017111//GO:0032559<br>//GO:0043231	nr +
Unigene11152_All	907	2.77	0.61	0.88	5.11	5.73	2.64	2.75	1.29	2.72	-2.17E+00	2.32E-06	Down	-1.65E+00	2.09E-04	Down	1.67E-01	5.88E-01		-9.53E-01	5.76E-04		-1.09E+00	2.53E-03		-1.42E-02	9.75E-01		Unigene11152_All//gi|6491702|emb|C<br>AB61875.1|//1.00E-78//myosin<br>[Arabidopsis thaliana]	Unigene11152_All//6.00E-<br>80//AT3G19960| ATM1 (ARABIDOPSIS<br>THALIANA MYOSIN 1); motor	Unigene11152_All//0.00E+00//gi|48777324|g<br>b|CO086690.1|CO086690			GO:0015405//GO:0015629//GO:0017111<br>//GO:0030029//GO:0031224//GO:00325<br>59//GO:0043231//GO:0044444//GO:005<br>1234	nr -
Unigene111528_All	203	0.00	0.00	0.00	0.00	0.17	0.00	121.22	72.27	328.33	null	null		null	null		7.41E+00	8.36E-01		null	null		-7.46E-01	6.55E-14		1.44E+00	1.77E-106	Up	Unigene111528_All//gi|40218371|gb| AAR83118.1|//4.00E-19//secretory<br>laccase [Gossypium arboreum]	Unigene111528_All//3.00E-<br>05//AT5G03260| LAC11 (laccase 11);<br>laccase	Unigene111528_All//2.00E-63//TC137114			GO:0005576//GO:0009808//GO:0016682<br>//GO:0046914	nr +
Unigene11153_All	1756	1.52	0.87	4.03	6.07	5.26	4.09	4.43	1.40	3.56	-8.12E-01	3.53E-02		1.41E+00	4.99E-09	Up	-2.08E-01	2.81E-01		-5.70E-01	8.81E-04		-1.66E+00	1.12E-15	Down	-3.15E-01	9.23E-02		Unigene11153_All//gi|116047949|gb| ABJ53200.1|//0.00E+00//myosin<br>VIII-1 [Nicotiana benthamiana]	Unigene11153_All//0.00E+00//AT3G19<br>960| ATM1 (ARABIDOPSIS THALIANA<br>MYOSIN 1); motor	Unigene11153_All//0.00E+00//CO099686			GO:0015405//GO:0015629//GO:0017111<br>//GO:0030029//GO:0031224//GO:00325<br>59//GO:0044444//GO:0051234	nr -
Unigene11154_All	2360	8.27	3.13	5.03	12.66	14.45	10.05	10.68	6.64	4.63	-1.40E+00	1.59E-24	Down	-7.17E-01	2.88E-08		1.90E-01	4.66E-02		-3.34E-01	6.45E-04		-6.85E-01	2.29E-12		-1.21E+00	1.26E-31	Down	Unigene11154_All//gi|116047949|gb| ABJ53200.1|//0.00E+00//myosin<br>VIII-1 [Nicotiana benthamiana]	Unigene11154_All//0.00E+00//AT3G19<br>960| ATM1 (ARABIDOPSIS THALIANA<br>MYOSIN 1); motor	Unigene11154_All//0.00E+00//gi|164335094| gb|ES790869.1|ES790869			GO:0015405//GO:0015629//GO:0017111<br>//GO:0030029//GO:0031224//GO:00325<br>59//GO:0044444//GO:0051234	nr -
Unigene111549_All	204	0.00	0.00	0.00	0.00	0.00	0.66	3.52	0.61	0.39	null	null		null	null		null	null		9.38E+00	3.12E-01		-2.52E+00	4.69E-03		-3.17E+00	8.09E-04	Down							
Unigene111563_All	204	0.00	0.00	0.00	0.00	0.00	0.00	3.11	1.43	0.00	null	null		null	null		null	null		null	null		-1.12E+00	1.80E-01		-1.16E+01	7.12E-05	Down	Unigene111563_All//sp|Q6CQE5|TAR1_<br>KLULA//4.00E-18//Protein TAR1<br>OS=Kluyveromyces lactis GN=TAR1-A<br>PE=4 SV=2		Unigene111563_All//2.00E-17//TC133518				swissprot +
Unigene111581_All	205	0.00	0.00	0.00	0.24	0.17	0.00	2.68	1.43	0.00	null	null		null	null		-5.33E-01	8.28E-01		-7.92E+00	6.43E-01		-9.10E-01	3.19E-01		-1.14E+01	2.81E-04	Down							
Unigene111583_All	205	0.00	0.00	0.00	0.00	0.00	0.00	3.30	0.82	0.00	null	null		null	null		null	null		null	null		-2.02E+00	1.98E-02		-1.17E+01	3.57E-05	Down							ESTscan -
Unigene111584_All	205	0.00	0.00	0.00	0.00	0.00	0.00	3.92	0.61	0.58	null	null		null	null		null	null		null	null		-2.68E+00	1.67E-03		-2.75E+00	9.43E-04	Down							
Unigene11159_All	468	0.67	0.65	1.48	2.24	1.03	0.87	5.87	2.32	0.77	-4.63E-02	9.85E-01		1.14E+00	2.18E-01		-1.12E+00	8.44E-02		-1.36E+00	4.89E-02		-1.34E+00	1.28E-04	Down	-2.94E+00	8.38E-12	Down			Unigene11159_All//3.00E-<br>95//gi|164342476|gb|ES793425.1|ES793425				
Unigene111592_All	205	0.00	0.00	0.00	0.00	0.00	0.00	2.68	0.82	0.00	null	null		null	null		null	null		null	null		-1.72E+00	7.23E-02		-1.14E+01	2.81E-04	Down							ESTscan -
Unigene111595_All	205	0.00	0.00	0.00	0.00	0.00	0.00	2.47	0.00	0.00	null	null		null	null		null	null		null	null		-1.13E+01	8.90E-04	Down	-1.13E+01	5.55E-04	Down							
Unigene11160_All	472	6.32	2.58	4.63	1.90	1.53	0.67	3.40	1.42	0.68	-1.29E+00	5.17E-04	Down	-4.50E-01	2.44E-01		-3.10E-01	6.65E-01		-1.50E+00	5.09E-02		-1.26E+00	7.61E-03		-2.33E+00	8.81E-06	Down			Unigene11160_All//0.00E+00//gi|164350365| gb|ES847900.1|ES847900				
Unigene11161_All	3991	6.19	2.83	4.55	2.85	2.79	1.54	2.07	0.81	0.64	-1.13E+00	3.19E-22	Down	-4.44E-01	9.25E-05		-3.02E-02	8.77E-01		-8.87E-01	8.34E-08		-1.36E+00	1.54E-12	Down	-1.69E+00	1.29E-17	Down	Unigene11161_All//gi|224131798|ref<br>|XP_002321181.1|//0.00E+00//histid<br>ine kinase cytokinin receptor<br>[Populus trichocarpa]<br>>gi|222861954|gb|EEE99496.1| histidine kinase cytokinin<br>receptor [Populus trichocarpa]	Unigene11161_All//0.00E+00//AT2G01<br>830| WOL (WOODEN LEG); cytokinin<br>receptor/ osmosensor/<br>phosphoprotein phosphatase/<br>protein histidine kinase	Unigene11161_All//0.00E+00//TC168551	Orphans		GO:0006351//GO:0006468//GO:0006952<br>//GO:0009267//GO:0009744//GO:00097<br>55//GO:0009784//GO:0009845//GO:001<br>0015//GO:0010260//GO:0015698//GO:0<br>016791//GO:0019901//GO:0023060//GO<br>:0032559//GO:0044464//GO:2000026	nr +
Unigene11162_All	5162	6.69	3.61	5.02	11.92	9.29	6.82	12.98	5.57	4.92	-8.89E-01	5.17E-21		-4.13E-01	1.17E-05		-3.60E-01	3.89E-09		-8.04E-01	1.33E-33		-1.22E+00	9.04E-82	Down	-1.40E+00	1.03E-103	Down	Unigene11162_All//gi|190148355|gb| ACE63260.1|//0.00E+00//histidine<br>kinase 2 [Betula pendula]	Unigene11162_All//0.00E+00//AT5G35<br>750| AHK2 (ARABIDOPSIS HISTIDINE<br>KINASE 2); cytokinin receptor/<br>osmosensor/ protein histidine<br>kinase	Unigene11162_All//0.00E+00//TC177777	Orphans		GO:0004673//GO:0004888//GO:0006351<br>//GO:0006468//GO:0006826//GO:00069<br>52//GO:0006970//GO:0009267//GO:000<br>9409//GO:0009737//GO:0009744//GO:0<br>009755//GO:0009845//GO:0009888//GO<br>:0009908//GO:0010260//GO:0015996//<br>GO:0016020//GO:0016791//GO:0019901<br>//GO:0023060//GO:0032559//GO:00400<br>07//GO:2000026	nr -
Unigene111627_All	207	0.00	0.00	0.00	0.00	0.00	0.00	2.45	0.00	0.00	null	null		null	null		null	null		null	null		-1.13E+01	8.90E-04	Down	-1.13E+01	5.55E-04	Down	Unigene111627_All//gi|18411538|ref<br>|NP_567096.1|//1.00E-24//60S<br>ribosomal protein L37a (RPL37aC)<br>[Arabidopsis thaliana]<br>>gi|297817320|ref|XP_002876543.1| 60S ribosomal protein L37a<br>[Arabidopsis lyrata subsp. lyrata]<br>>gi|75247667|sp|Q8RXU5.1|R37A2_ARA<br>TH RecName: Full=60S ribosomal<br>protein L37a-2<br>>gi|21593754|gb|AAM65721.1| 60S<br>ribosomal protein L37a<br>[Arabidopsis thaliana]<br>>gi|297322381|gb|EFH52802.1| 60S<br>ribosomal protein L37a<br>[Arabidopsis lyrata subsp. lyrata]	Unigene111627_All//3.00E-<br>15//AT3G60245| 60S ribosomal<br>protein L37a (RPL37aC)	Unigene111627_All//9.00E-10//ES845699		ko03010|Ribosome	GO:0005198//GO:0010467//GO:0015934<br>//GO:0022613//GO:0043169	nr +
Unigene111629_All	207	0.00	0.00	0.00	0.00	0.00	0.00	4.49	0.61	0.19	null	null		null	null		null	null		null	null		-2.89E+00	3.28E-04	Down	-4.54E+00	6.41E-06	Down							
Unigene111644_All	207	0.00	0.00	0.00	0.00	0.00	0.00	2.45	0.00	0.58	null	null		null	null		null	null		null	null		-1.13E+01	8.90E-04	Down	-2.09E+00	3.65E-02								
Unigene11166_All	695	3.39	1.31	2.83	3.94	3.86	5.33	4.87	3.61	2.06	-1.37E+00	1.46E-03		-2.57E-01	5.77E-01		-2.86E-02	9.44E-01		4.35E-01	2.01E-01		-4.32E-01	1.65E-01		-1.24E+00	3.60E-05	Down	Unigene11166_All//gi|18401324|ref| NP_565636.1|//8.00E-70//FRS3<br>(FAR1-related sequence 3); zinc<br>ion binding [Arabidopsis thaliana]<br>>gi|30683396|ref|NP_850098.1| FRS3<br>(FAR1-related sequence 3); zinc<br>ion binding [Arabidopsis thaliana]<br>>gi|75216958|sp|Q9ZVC9.2|FRS3_ARAT<br>H RecName: Full=Protein FAR1-<br>RELATED SEQUENCE 3<br>>gi|15982769|gb|AAL09732.1| At2g27110/T20P8.16 [Arabidopsis<br>thaliana]<br>>gi|20197414|gb|AAC77869.2| Mutator-like transposase<br>[Arabidopsis thaliana]<br>>gi|27363374|gb|AAO11606.1| At2g27110/T20P8.16 [Arabidopsis<br>thaliana]	Unigene11166_All//4.00E-<br>71//AT2G27110| FRS3 (FAR1-related<br>sequence 3); zinc ion binding	Unigene11166_All//0.00E+00//EX166588			GO:0009416//GO:0046914	nr -
Unigene111669_All	208	0.00	0.00	0.00	0.00	0.00	0.00	17.07	14.47	0.00	null	null		null	null		null	null		null	null		-2.39E-01	4.55E-01		-1.41E+01	4.09E-26	Down							
Unigene111674_All	208	0.00	0.00	0.00	2.63	0.33	0.22	19.92	5.02	5.75	null	null		null	null		-2.99E+00	9.39E-03		-3.60E+00	8.98E-03		-1.99E+00	6.14E-11	Down	-1.79E+00	5.60E-10	Down			Unigene111674_All//4.00E-<br>80//gi|109827560|gb|DW226236.1|DW226236				
Unigene11168_All	700	0.75	2.61	0.68	0.57	1.87	1.74	1.87	2.27	4.55	1.80E+00	6.20E-04	Up	-1.27E-01	8.98E-01		1.72E+00	5.60E-03		1.61E+00	1.36E-02		2.77E-01	6.01E-01		1.28E+00	4.26E-05	Up	Unigene11168_All//gi|224057200|ref<br>|XP_002299169.1|//5.00E-<br>21//predicted protein [Populus<br>trichocarpa]<br>>gi|222846427|gb|EEE83974.1| predicted protein [Populus<br>trichocarpa]					GO:0004713//GO:0006464//GO:0032559	nr +
Unigene111685_All	209	0.00	0.00	0.00	0.00	0.00	0.00	4.65	5.00	0.00	null	null		null	null		null	null		null	null		1.04E-01	8.68E-01		-1.22E+01	2.72E-07	Down							ESTscan +
Unigene111686_All	209	0.00	0.00	0.00	0.48	0.16	0.00	2.83	0.00	0.00	null	null		null	null		-1.53E+00	5.88E-01		-8.90E+00	4.04E-01		-1.15E+01	2.39E-04	Down	-1.15E+01	1.41E-04	Down							
Unigene11169_All	602	0.17	0.00	0.00	1.49	0.80	1.20	0.42	1.32	2.91	-7.44E+00	3.76E-01		-7.44E+00	3.98E-01		-8.95E-01	2.14E-01		-3.11E-01	6.88E-01		1.65E+00	2.88E-02		2.79E+00	2.72E-07	Up	Unigene11169_All//gi|297793187|ref<br>|XP_002864478.1|//5.00E-43//kinase<br>family protein [Arabidopsis lyrata<br>subsp. lyrata]<br>>gi|297310313|gb|EFH40737.1| kinase family protein [Arabidopsis<br>lyrata subsp. lyrata]	Unigene11169_All//3.00E-<br>44//AT5G56790| protein kinase<br>family protein	Unigene11169_All//0.00E+00//AI726378		ko04626|Plant-pathogen interaction	GO:0004713	nr +
Unigene111694_All	209	0.00	0.00	0.00	0.00	0.00	0.00	41.27	28.00	0.00	null	null		null	null		null	null		null	null		-5.60E-01	1.40E-03		-1.53E+01	1.70E-63	Down	Unigene111694_All//gi|168069582|re<br>f|XP_001786502.1|//2.00E-<br>12//predicted protein<br>[Physcomitrella patens subsp.<br>patens]<br>>gi|162661153|gb|EDQ48685.1| predicted protein [Physcomitrella<br>patens subsp. patens]		Unigene111694_All//2.00E-<br>20//gi|109830702|gb|DW229261.1|DW229261				nr -
Unigene111700_All	210	0.00	0.00	0.00	0.47	0.00	0.65	5.44	1.39	1.14	null	null		null	null		-8.89E+00	3.16E-01		4.44E-01	8.62E-01		-1.96E+00	1.69E-03		-2.26E+00	3.40E-04	Down			Unigene111700_All//1.00E-09//DW520090				
Unigene11171_All	1896	0.00	0.61	0.11	0.42	0.42	0.31	0.78	0.66	1.47	9.26E+00	1.05E-06	Up	6.81E+00	1.42E-01		-9.30E-03	9.67E-01		-4.41E-01	6.14E-01		-2.39E-01	6.20E-01		9.14E-01	5.09E-03		Unigene11171_All//gi|53791527|dbj| BAD52649.1|//9.00E-44//dual-<br>specific kinase DSK1-like [Oryza<br>sativa Japonica Group]<br>>gi|53793414|dbj|BAD53117.1| dual-<br>specific kinase DSK1-like [Oryza<br>sativa Japonica Group]	Unigene11171_All//5.00E-<br>44//AT5G56790| protein kinase<br>family protein	Unigene11171_All//0.00E+00//TC170196		ko04626|Plant-pathogen interaction	GO:0004672	nr -
Unigene111714_All	210	0.00	0.00	0.00	0.00	0.00	0.00	3.22	1.59	0.19	null	null		null	null		null	null		null	null		-1.02E+00	2.04E-01		-4.09E+00	3.10E-04	Down							
Unigene111721_All	211	0.00	0.00	0.00	0.00	0.00	0.00	3.81	8.32	0.57	null	null		null	null		null	null		null	null		1.13E+00	1.16E-02		-2.75E+00	9.43E-04	Down							
Unigene111734_All	212	0.00	0.00	0.00	0.00	0.00	0.00	2.39	0.00	0.00	null	null		null	null		null	null		null	null		-1.12E+01	8.90E-04	Down	-1.12E+01	5.55E-04	Down							ESTscan -
Unigene111739_All	212	0.00	0.00	0.00	0.00	0.49	0.00	2.19	2.56	7.70	null	null		null	null		8.93E+00	4.58E-01		null	null		2.24E-01	7.92E-01		1.81E+00	2.01E-04	Up			Unigene111739_All//7.00E-54//DW519317				
Unigene111751_All	213	15.23	5.23	11.50	14.50	20.69	17.81	2.98	4.12	6.17	-1.54E+00	2.52E-05	Down	-4.05E-01	2.71E-01		5.13E-01	7.85E-02		2.97E-01	3.94E-01		4.69E-01	5.04E-01		1.05E+00	3.92E-02		Unigene111751_All//gi|227438229|gb<br>|ACP30604.1|//6.00E-06//disease<br>resistance protein [Brassica rapa<br>subsp. pekinensis]	Unigene111751_All//1.00E-<br>06//AT5G40910| disease resistance<br>protein (TIR-NBS-LRR class),<br>putative					nr +

Unigene111755_All	213	0.49	0.24	0.00	0.47	2.26	0.42	8.93	0.20	1.68	-1.05E+00	6.85E-01		-8.94E+00	3.98E-01		2.27E+00	7.83E-02		-1.41E-01	9.52E-01		-5.51E+00	4.08E-12	Down	-2.41E+00	6.35E-07	Down	Unigene111755_All//gi|297813757|ref|XP_002874762.1|//7.00E-18//At4g12320 [Arabidopsis lyrata subsp. lyrata] >gi|297320599|gb|EFH51021.1| At4g12320 [Arabidopsis lyrata subsp. lyrata]	Unigene111755_All//4.00E-09//AT4G12300| CYP706A4; electron carrier/ heme binding / iron ion binding / monooxygenase/ oxygen binding	Unigene111755_All//3.00E-93//TC134420		ko00945|Stilbenoid, diarylheptanoid and gingerol biosynthesis//ko00903|Limonene and pinene degradation//ko00941|Flavonoid biosynthesis//ko00944|Flavone and flavonol biosynthesis	GO:0004497//GO:0005506//GO:0008152//GO:0044464	nr +
Unigene111757_All	213	0.00	0.48	1.25	0.00	1.13	0.64	3.77	0.59	0.00	8.89E+00	3.90E-01		1.03E+01	8.03E-02		1.01E+01	1.04E-01		9.31E+00	3.12E-01		-2.68E+00	1.67E-03		-1.19E+01	4.43E-06	Down	Unigene111757_All//gi|224146449|ref|XP_002326011.1|//1.00E-13//predicted protein [Populus trichocarpa] >gi|222862886|gb|EEF00393.1| predicted protein [Populus trichocarpa]		Unigene111757_All//6.00E-48//TC175290			GO:0005488//GO:0016307//GO:0044237//GO:0044238	nr +
Unigene111774_All	214	0.24	0.24	0.50	0.93	0.64	0.63	2.37	0.00	0.93	-4.62E-02	9.86E-01		1.03E+00	7.04E-01		-5.33E-01	7.49E-01		-5.57E-01	7.33E-01		-1.12E+01	8.90E-04	Down	-1.35E+00	1.40E-01		Unigene111774_All//gi|15237312|ref|NP_197731.1|//5.00E-16//disease resistance family protein / LRR family protein [Arabidopsis thaliana] >gi|9759078|dbj|BAB09556.1| disease resistance protein-like [Arabidopsis thaliana]	Unigene111774_All//3.00E-13//AT5G23400| disease resistance family protein / LRR family protein	Unigene111774_All//2.00E-115//TC175205			GO:0006950//GO:0016491//GO:0023060//GO:0030312//GO:0031410	nr -
Unigene111781_All	214	0.00	0.00	0.00	0.00	0.00	0.00	2.77	0.00	0.00	null	null		null	null		null	null		null	null		-1.14E+01	2.39E-04	Down	-1.14E+01	1.41E-04	Down	Unigene111781_All//gi|116048190|gb|ABJ53283.1|//7.00E-28//S-adenosyl-L-homocysteine hydrolase [Ligularia fischeri]	Unigene111781_All//4.00E-28//AT3G23810| SAHH2 (S-ADENOSYL-L-HOMOCYSTEINE (SAH) HYDROLASE 2); adenosylhomocysteinase/ binding / catalytic	Unigene111781_All//4.00E-12//gi|5048510|gb|AI729658.1|AI729658		ko00270|Cysteine and methionine metabolism//ko00450|Selenoamino acid metabolism	GO:0016802//GO:0044237	nr -
Unigene111789_All	214	0.00	3.08	0.00	0.47	0.00	0.84	0.99	0.00	0.56	1.16E+01	6.26E-04	Up	null	null		-8.86E+00	3.16E-01		8.59E-01	6.96E-01		-9.95E+00	7.46E-02		-8.23E-01	6.00E-01		Unigene111789_All//gi|208429106|gb|ACI26722.1|//4.00E-16//receptor-like kinase [Nicotiana glutinosa]	Unigene111789_All//1.00E-05//AT4G32300| SD2-5 (S-DOMAIN-2 5); carbohydrate binding / kinase/ protein kinase	Unigene111789_All//1.00E-117//TC178969			GO:0004674//GO:0004871//GO:0006464//GO:0031410//GO:0032559	nr +
Unigene11179_All	2663	6.96	3.58	3.14	12.96	11.42	10.35	15.77	9.01	8.35	-9.59E-01	4.15E-13		-1.15E+00	1.40E-16	Down	-1.83E-01	5.25E-02		-3.26E-01	3.11E-04		-8.07E-01	2.07E-26		-9.17E-01	1.42E-33		Unigene11179_All//gi|15231445|ref|NP_190235.1|//0.00E+00//PUB13 (PLANT U-BOX 13); ubiquitin-protein ligase [Arabidopsis thaliana] >gi|75266129|sp|Q9SNC6.1|PUB13_ARATH RecName: Full=U-box domain-containing protein 13; AltName: Full=Plant U-box protein 13 >gi|6523054|emb|CAB62321.1| arm repeat containing protein homolog [Arabidopsis thaliana] >gi|14596007|gb|AAK68731.1| arm repeat containing protein homolog [Arabidopsis thaliana] >gi|22136270|gb|AAM91213.1| arm repeat containing protein homolog [Arabidopsis thaliana]	Unigene11179_All//0.00E+00//AT3G46510| PUB13 (PLANT U-BOX 13); ubiquitin-protein ligase	Unigene11179_All//0.00E+00//TC171950		ko04141|Protein processing in endoplasmic reticulum//ko04120|Ubiquitin mediated proteolysis	GO:0005488//GO:0019787//GO:0032446//GO:0043231//GO:0043234//GO:0044444	nr +
Unigene111795_All	214	6.85	2.60	7.47	12.80	12.87	20.05	51.76	14.65	15.45	-1.39E+00	1.39E-02		1.25E-01	8.06E-01		7.90E-03	9.95E-01		6.47E-01	2.87E-02		-1.82E+00	5.48E-25	Down	-1.74E+00	2.43E-24	Down	Unigene111795_All//gi|225431804|ref|XP_002272727.1|//1.00E-11//PREDICTED: similar to 60S ribosomal protein L19 [Vitis vinifera]		Unigene111795_All//3.00E-50//ES836772		ko03010|Ribosome		nr +
Unigene111798_All	215	0.00	0.00	0.00	0.00	0.00	0.00	2.56	0.00	0.00	null	null		null	null		null	null		null	null		-1.13E+01	4.62E-04	Down	-1.13E+01	2.81E-04	Down							
Unigene111802_All	215	0.00	0.00	0.00	0.00	0.00	0.00	2.36	1.17	0.00	null	null		null	null		null	null		null	null		-1.02E+00	2.88E-01		-1.12E+01	5.55E-04	Down							ESTscan -
Unigene111805_All	215	0.00	0.00	0.00	0.00	0.00	0.00	2.56	0.97	0.00	null	null		null	null		null	null		null	null		-1.40E+00	1.35E-01		-1.13E+01	2.81E-04	Down	Unigene111805_All//gi|300137372|gb|EFJ04348.1|//5.00E-11//hypothetical protein SELMODRAFT_138833 [Selaginella moellendorffii]		Unigene111805_All//1.00E-15//TC148521				nr -
Unigene111807_All	215	0.00	0.00	0.00	1.39	1.76	0.00	3.74	0.58	0.00	null	null		null	null		3.42E-01	8.36E-01		-1.04E+01	4.01E-02		-2.68E+00	1.67E-03		-1.19E+01	4.43E-06	Down			Unigene111807_All//2.00E-07//ES810950				
Unigene111809_All	215	0.49	0.24	0.00	0.93	1.44	0.21	32.45	12.64	15.01	-1.05E+00	6.85E-01		-8.93E+00	3.98E-01		6.37E-01	6.95E-01		-2.14E+00	3.47E-01		-1.36E+00	9.07E-11	Down	-1.11E+00	2.35E-08	Down	Unigene111809_All//gi|115480625|ref|NP_001063906.1|//6.00E-35//Os09g0557800 [Oryza sativa Japonica Group] >gi|113632139|dbj|BAF25820.1| Os09g0557800 [Oryza sativa Japonica Group]	Unigene111809_All//5.00E-30//AT5G25100| endomembrane protein 70, putative	Unigene111809_All//2.00E-82//TC142008			GO:0031224//GO:0031410//GO:0043231	nr -
Unigene11181_All	4217	2.43	9.29	3.75	2.67	2.28	2.15	10.03	9.92	5.73	1.93E+00	3.09E-76	Up	6.25E-01	1.56E-05		-2.29E-01	2.09E-01		-3.11E-01	8.05E-02		-1.52E-02	8.73E-01		-8.06E-01	2.68E-27		Unigene11181_All//gi|255545642|ref|XP_002513881.1|//0.00E+00//Homomeric Acetyl-CoA Carboxylase (Hom-ACCase) [Ricinus communis] >gi|223546967|gb|EEF48464.1| Homomeric Acetyl-CoA Carboxylase (Hom-ACCase) [Ricinus communis]	Unigene11181_All//0.00E+00//AT1G36180| ACC2 (ACETYL-COA CARBOXYLASE 2); acetyl-CoA carboxylase	Unigene11181_All//0.00E+00//TC150892		ko00620|Pyruvate metabolism//ko00640|Propanoate metabolism//ko00061|Fatty acid biosynthesis	GO:0003006//GO:0006633//GO:0009725//GO:0016020//GO:0016421//GO:0019842//GO:0032559//GO:0043231//GO:0048507	nr +
Unigene111810_All	215	0.00	0.00	0.00	0.00	0.00	0.00	2.36	0.00	0.00	null	null		null	null		null	null		null	null		-1.12E+01	8.90E-04	Down	-1.12E+01	5.55E-04	Down	Unigene111810_All//gi|255103522|gb|ACH70610.1|//4.00E-16//alpha tubulin [Ulva fasciata]	Unigene111810_All//4.00E-17//AT5G19780| TUA5; structural constituent of cytoskeleton	Unigene111810_All//2.00E-17//gi|54115762|gb|CO491271.1|CO491271		ko04145|Phagosome	GO:0007017//GO:0015630//GO:0017111//GO:0032561//GO:0032991//GO:0043623	nr +
Unigene111811_All	215	0.00	0.00	0.00	0.00	0.00	0.00	2.56	0.00	1.48	null	null		null	null		null	null		null	null		-1.13E+01	4.62E-04	Down	-7.86E-01	3.62E-01								
Unigene111812_All	215	3.16	1.89	3.72	5.33	3.84	0.84	7.28	2.33	12.79	-7.47E-01	3.80E-01		2.32E-01	7.49E-01		-4.71E-01	4.64E-01		-2.67E+00	4.12E-04	Down	-1.64E+00	1.02E-03		8.13E-01	1.31E-02				Unigene111812_All//4.00E-46//TC169760				
Unigene111815_All	216	3.88	2.35	0.49	2.31	2.87	1.88	4.89	0.97	1.66	-7.24E-01	3.48E-01		-2.97E+00	3.83E-03		3.15E-01	7.84E-01		-2.93E-01	7.57E-01		-2.34E+00	6.78E-04	Down	-1.56E+00	9.40E-03				Unigene111815_All//2.00E-51//TC154650				
Unigene11182_All	7246	9.35	34.07	18.01	9.88	12.26	16.22	29.23	14.67	16.89	1.87E+00	0.00E+00	Up	9.46E-01	2.90E-83		3.11E-01	8.29E-10		7.16E-01	1.10E-51		-9.95E-01	1.16E-184		-7.91E-01	5.91E-129		Unigene11182_All//gi|255545642|ref|XP_002513881.1|//0.00E+00//Homomeric Acetyl-CoA Carboxylase (Hom-ACCase) [Ricinus communis] >gi|223546967|gb|EEF48464.1| Homomeric Acetyl-CoA Carboxylase (Hom-ACCase) [Ricinus communis]	Unigene11182_All//0.00E+00//AT1G36160| ACC1 (ACETYL-COENZYME A CARBOXYLASE 1); acetyl-CoA carboxylase	Unigene11182_All//0.00E+00//TC161428		ko00620|Pyruvate metabolism//ko00640|Propanoate metabolism//ko00061|Fatty acid biosynthesis	GO:0003006//GO:0006633//GO:0009317//GO:0009725//GO:0016020//GO:0016421//GO:0016879//GO:0019842//GO:0032559//GO:0048507	nr +
Unigene111827_All	216	139.04	815.40	501.68	244.46	264.16	876.71	52.65	105.07	50.17	2.55E+00	0.00E+00	Up	1.85E+00	1.04E-193	Up	1.12E-01	1.53E-01		1.84E+00	0.00E+00	Up	9.97E-01	1.56E-20		-6.98E-02	6.70E-01				Unigene111827_All//9.00E-78//DW510023				
Unigene111833_All	216	0.00	0.00	0.00	0.00	0.00	0.00	2.35	0.19	0.00	null	null		null	null		null	null		null	null		-3.60E+00	5.77E-03		-1.12E+01	5.55E-04	Down	Unigene111833_All//gi|241865275|gb|ACS68715.1|//2.00E-23//40S RPS3B [Sonneratia alba] >gi|241865508|gb|ACS68786.1| RPS3B [Sonneratia alba]	Unigene111833_All//3.00E-17//AT5G35530| 40S ribosomal protein S3 (RPS3C)			ko03010|Ribosome	GO:0003676//GO:0005198//GO:0010467//GO:0033279	nr -
Unigene111841_All	217	9.16	5.14	5.40	27.09	25.23	25.81	44.81	46.42	12.85	-8.35E-01	6.77E-02		-7.63E-01	1.14E-01		-1.02E-01	7.31E-01		-6.99E-02	7.91E-01		5.08E-02	7.84E-01		-1.80E+00	1.98E-22	Down	Unigene111841_All//gi|57639078|gb|AAW55475.1|//2.00E-10//ER-binding protein [Malus pumila]	Unigene111841_All//1.00E-05//AT5G42020| BIP2; ATP binding	Unigene111841_All//1.00E-117//BG440344		ko04141|Protein processing in endoplasmic reticulum//ko03060|Protein export	GO:0008152//GO:0016628//GO:0032559//GO:0044432//GO:0050896	nr +
Unigene111849_All	217	0.00	0.00	0.00	0.00	0.00	0.00	4.68	2.50	0.73	null	null		null	null		null	null		null	null		-9.01E-01	1.44E-01		-2.67E+00	1.94E-04	Down			Unigene111849_All//3.00E-22//TC154893				
Unigene111852_All	217	0.24	0.47	0.74	0.23	3.65	0.42	1.95	2.31	1.47	9.54E-01	7.27E-01		1.61E+00	5.12E-01		3.99E+00	6.32E-04	Up	8.59E-01	7.96E-01		2.46E-01	7.84E-01		-4.08E-01	6.54E-01				Unigene111852_All//1.00E-86//TC155814				
Unigene111855_All	218	0.00	0.00	0.00	0.00	0.00	0.00	9.31	0.77	6.76	null	null		null	null		null	null		null	null		-3.60E+00	3.79E-10	Down	-4.61E-01	2.45E-01				Unigene111855_All//2.00E-07//TC158875				
Unigene11186_All	2915	1.87	1.32	0.90	1.73	3.24	2.54	1.29	1.45	2.35	-4.99E-01	5.24E-02		-1.06E+00	8.56E-05	Down	9.07E-01	3.15E-07		5.58E-01	9.33E-03		1.66E-01	6.01E-01		8.65E-01	1.10E-05		Unigene11186_All//gi|9294523|dbj|BAB02785.1|//0.00E+00//protein transport protein Sec23 [Arabidopsis thaliana]	Unigene11186_All//0.00E+00//AT3G23660| transport protein, putative	Unigene11186_All//0.00E+00//TC132777			GO:0012507//GO:0015031//GO:0046914//GO:0048193	nr +
Unigene111870_All	219	0.00	0.00	0.00	0.00	0.00	0.00	4.63	0.57	0.73	null	null		null	null		null	null		null	null		-3.02E+00	1.08E-04	Down	-2.67E+00	1.94E-04	Down	Unigene111870_All//gi|164521173|gb|ABY60448.1|//5.00E-15//ribosomal protein S25 [Adonis aestivalis var. palaestina]	Unigene111870_All//5.00E-06//AT4G34555| 40S ribosomal protein S25, putative			ko03010|Ribosome	GO:0030529//GO:0043231	nr -
Unigene111874_All	219	0.00	0.00	0.00	0.00	0.00	0.00	5.21	0.76	0.18	null	null		null	null		null	null		null	null		-2.77E+00	7.62E-05	Down	-4.84E+00	2.34E-07	Down			Unigene111874_All//3.00E-10//TC161862				
Unigene11189_All	1019	4.36	5.27	6.06	14.76	15.51	10.15	10.66	6.69	4.65	2.72E-01	3.23E-01		4.74E-01	5.91E-02		7.13E-02	6.90E-01		-5.41E-01	1.21E-04		-6.74E-01	1.19E-05		-1.20E+00	5.82E-14	Down	Unigene11189_All//gi|240256264|ref|NP_196285.5|//2.00E-61//ATP binding / microtubule motor [Arabidopsis thaliana]	Unigene11189_All//1.00E-56//AT5G06670| ATP binding / microtubule motor	Unigene11189_All//0.00E+00//gi|164341533|gb|ES851792.1|ES851792			GO:0003774//GO:0007017//GO:0015630//GO:0032559	nr +
Unigene111894_All	220	96.08	36.39	45.75	688.12	473.49	1052.72	144.33	121.78	64.65	-1.40E+00	1.55E-26	Down	-1.07E+00	6.62E-17	Down	-5.39E-01	9.41E-46		6.13E-01	2.60E-77		-2.45E-01	5.31E-03		-1.16E+00	3.50E-37	Down	Unigene111894_All//gi|255559090|ref|XP_002520567.1|//6.00E-09//Repetitive proline-rich cell wall protein 2 precursor, putative [Ricinus communis] >gi|223540227|gb|EEF41800.1| Repetitive proline-rich cell wall protein 2 precursor, putative [Ricinus communis]		Unigene111894_All//2.00E-100//TC134791				nr +
Unigene111900_All	221	0.00	0.00	0.00	0.00	0.00	0.00	2.30	0.57	0.00	null	null		null	null		null	null		null	null		-2.02E+00	5.21E-02		-1.12E+01	5.55E-04	Down			Unigene111900_All//2.00E-07//FL684464				
Unigene111901_All	221	0.00	0.23	0.00	0.00	0.00	0.00	2.87	0.38	0.00	7.84E+00	6.20E-01		null	null		null	null		null	null		-2.92E+00	4.20E-03		-1.15E+01	7.12E-05	Down			Unigene111901_All//3.00E-10//FL576920				
Unigene111905_All	221	0.00	0.00	0.00	0.00	0.00	0.00	3.06	0.19	0.00	null	null		null	null		null	null		null	null		-4.02E+00	5.36E-04	Down	-1.16E+01	3.57E-05	Down							ESTscan -
Unigene111908_All	221	0.00	0.00	0.00	0.00	0.00	0.00	2.30	0.57	0.00	null	null		null	null		null	null		null	null		-2.02E+00	5.21E-02		-1.12E+01	5.55E-04	Down							ESTscan -
Unigene11191_All	3593	0.95	2.06	1.63	3.24	4.34	3.23	2.27	1.74	1.92	1.12E+00	4.12E-07	Up	7.85E-01	2.10E-03		4.20E-01	1.97E-03		-6.10E-03	9.69E-01		-3.80E-01	4.06E-02		-2.44E-01	1.95E-01		Unigene11191_All//gi|22331006|ref|NP_187809.2|//0.00E+00//kinesin motor protein-related [Arabidopsis thaliana]	Unigene11191_All//0.00E+00//AT3G12020| kinesin motor protein-related	Unigene11191_All//0.00E+00//gi|78340671|gb|DT560945.1|DT560945			GO:0003774//GO:0007017//GO:0015630//GO:0032559	nr +
Unigene111911_All	221	0.00	0.00	0.00	0.00	0.00	0.00	2.68	0.00	0.00	null	null		null	null		null	null		null	null		-1.14E+01	2.39E-04	Down	-1.14E+01	1.41E-04	Down							
Unigene111913_All	222	18.85	41.08	14.87	43.53	49.17	38.86	94.46	147.60	226.11	1.12E+00	1.31E-08	Up	-3.42E-01	2.90E-01		1.76E-01	3.81E-01		-1.64E-01	4.54E-01		6.44E-01	2.94E-14		1.26E+00	8.64E-66	Up	Unigene111913_All//gi|294987233|gb|ADF56051.1|//5.00E-23//homeobox-leucine zipper protein [Cucumis sativus]	Unigene111913_All//1.00E-08//AT2G22800| HAT9; DNA binding / transcription factor	Unigene111913_All//1.00E-117//TC143756	HB		GO:0001071//GO:0003677//GO:0006351//GO:0009725//GO:0010468//GO:0043231	nr +
Unigene111924_All	222	12.49	11.87	9.11	18.62	9.00	14.44	18.66	18.26	8.97	-7.38E-02	8.43E-01		-4.55E-01	2.54E-01		-1.05E+00	1.71E-04	Down	-3.67E-01	2.52E-01		-3.14E-02	9.30E-01		-1.06E+00	5.80E-05	Down			Unigene111924_All//3.00E-59//gi|21091992|gb|BQ404305.1|BQ404305				
Unigene111934_All	223	0.00	0.00	0.00	0.00	0.00	0.00	2.84	0.56	0.18	null	null		null	null		null	null		null	null		-2.34E+00	1.27E-02		-3.99E+00	5.82E-04	Down	Unigene111934_All//gi|297600719|ref|NP_001049719.2|//6.00E-22//Os03g0277300 [Oryza sativa Japonica Group] >gi|255674402|dbj|BAF11633.2| Os03g0277300 [Oryza sativa Japonica Group]	Unigene111934_All//1.00E-11//AT3G09440| heat shock cognate 70 kDa protein 3 (HSC70-3) (HSP70-3)			ko03040|Spliceosome//ko04141|Protein processing in endoplasmic reticulum//ko04144|Endocytosis	GO:0000302//GO:0009642//GO:0010038//GO:0016020//GO:0016628//GO:0019899//GO:0030312//GO:0032446//GO:0032559//GO:0043231//GO:0051707	nr +
Unigene11194_All	699	6.21	3.91	3.58	11.62	4.98	8.21	10.65	8.85	7.01	-6.66E-01	2.17E-02		-7.95E-01	8.80E-03		-1.22E+00	2.02E-10	Down	-5.01E-01	1.38E-02		-2.67E-01	1.97E-01		-6.03E-01	1.10E-03		Unigene11194_All//gi|113208238|dbj|BAF03523.1|//5.00E-44//CGS1 mRNA stability 1 [Arabidopsis thaliana]	Unigene11194_All//2.00E-33//AT2G25320| LOCATED IN: chloroplast; CONTAINS InterPro DOMAIN/s: TRAF-like (InterPro:IPR008974), MATH (InterPro:IPR002083); BEST Arabidopsis thaliana protein match is: meprin and TRAF homology domain-containing protein / MATH domain-containing protein (TAIR:AT2G25330.1); Has 59993 Blast hits to 32072 proteins in 1426 species: Archae - 584; Bacteria - 5166; Metazoa - 31897; Fungi - 4070; Plants - 2329; Viruses - 207; Other Eukaryotes - 15740 (source: NCBI BLink).	Unigene11194_All//0.00E+00//gi|164338975|gb|EX172487.1|EX172487			GO:0001071//GO:0006351//GO:0031406//GO:0043231//GO:0048037	nr +
Unigene111944_All	224	0.00	0.00	0.00	0.00	0.00	0.00	3.40	0.56	0.00	null	null		null	null		null	null		null	null		-2.60E+00	2.81E-03		-1.17E+01	8.88E-06	Down	Unigene111944_All//gi|116222089|gb|ABJ80915.1|//7.00E-36//actin [Goniomonas truncata]	Unigene111944_All//1.00E-36//AT3G12110| ACT11 (actin-11); structural constituent of cytoskeleton	Unigene111944_All//2.00E-39//TC153744		ko04145|Phagosome	GO:0032559//GO:0043232	nr +

Unigene111948_All	224	0.00	0.00	0.00	0.00	0.00	0.00	2.27	0.75	0.00	null	null		null	null		null	null		null	null		-1.60E+00	1.07E-01		-1.11E+01	5.55E-04	Down	Unigene111948_All//gi|2894308|emb|CAA11269.1|//4.00E-33//polyubiquitin [Nicotiana tabacum]	Unigene111948_All//2.00E-33//AT5G03240| UBQ3 (POLYUBIQUITIN 3); protein binding	Unigene111948_All//1.00E-30//gi|109826718|gb|DW225428.1|DW225428			GO:0043231	nr +
Unigene111954_All	225	0.00	0.00	0.00	0.00	0.00	0.00	3.38	1.30	0.00	null	null		null	null		null	null		null	null		-1.38E+00	6.73E-02		-1.17E+01	8.88E-06	Down	Unigene111954_All//gi|299470875|emb|CBN78824.1|//6.00E-09//Protein for assembly of pre-ribosomal particles containing 18S rRNA [Ectocarpus siliculosus]	Unigene111954_All//9.00E-05//AT5G50250| 31 kDa ribonucleoprotein, chloroplast, putative / RNA-binding protein RNP-T, putative / RNA-binding protein 1/2/3, putative / RNA-binding protein cp31, putative				GO:0005488	nr -
Unigene111958_All	225	0.00	0.00	0.00	0.00	0.00	0.00	3.19	0.00	0.18	null	null		null	null		null	null		null	null		-1.16E+01	3.27E-05	Down	-4.17E+00	1.64E-04	Down	Unigene111958_All//gi|17979018|gb|AAL49957.1|//8.00E-06//GTP cyclohydrolase I [Solanum lycopersicum]	Unigene111958_All//9.00E-05//AT3G07270| GTP cyclohydrolase I			ko00790|Folate biosynthesis	GO:0003933	nr -
Unigene111960_All	225	0.00	0.00	0.00	0.89	0.15	0.40	3.01	0.37	0.18	null	null		null	null		-2.53E+00	2.34E-01		-1.14E+00	5.70E-01		-3.02E+00	2.43E-03		-4.09E+00	3.10E-04	Down			Unigene111960_All//5.00E-18//gi|78329277|gb|DT549551.1|DT549551				
Unigene111967_All	226	340.55	183.17	81.05	225.71	195.64	123.90	208.22	52.52	53.94	-8.95E-01	2.06E-46		-2.07E+00	4.11E-159	Down	-2.06E-01	4.50E-03		-8.65E-01	1.32E-31		-1.99E+00	1.23E-116	Down	-1.95E+00	4.35E-117	Down			Unigene111967_All//1.00E-120//gi|21098593|gb|BQ410906.1|BQ410906				
Unigene11197_All	805	0.13	0.63	0.46	1.61	1.28	1.18	0.84	1.56	3.51	2.28E+00	6.23E-02		1.83E+00	2.13E-01		-3.26E-01	5.86E-01		-4.50E-01	4.84E-01		8.90E-01	1.02E-01		2.06E+00	3.11E-08	Up	Unigene11197_All//gi|297812493|ref|XP_002874130.1|//2.00E-21//transducin family protein [Arabidopsis lyrata subsp. lyrata] >gi|297319967|gb|EFH50389.1| transducin family protein [Arabidopsis lyrata subsp. lyrata]	Unigene11197_All//1.00E-22//AT5G23430| transducin family protein / WD-40 repeat family protein				GO:0004468//GO:0005488//GO:0031461	nr +
Unigene111971_All	226	0.93	0.00	0.00	0.22	0.00	0.00	5.43	1.11	1.06	-9.85E+00	1.23E-01		-9.85E+00	1.46E-01		-7.78E+00	5.61E-01		-7.78E+00	6.43E-01		-2.29E+00	2.57E-04	Down	-2.36E+00	1.22E-04	Down			Unigene111971_All//7.00E-17//TC142701				
Unigene111973_All	226	0.00	0.00	0.00	0.00	0.00	0.00	3.18	0.37	0.35	null	null		null	null		null	null		null	null		-3.10E+00	1.40E-03		-3.17E+00	8.09E-04	Down							
Unigene111987_All	227	0.46	0.00	0.94	0.00	0.00	0.00	2.79	0.74	0.18	-8.85E+00	3.75E-01		1.03E+00	5.92E-01		null	null		null	null		-1.92E+00	3.09E-02		-3.99E+00	5.82E-04	Down							
Unigene111991_All	227	0.00	0.00	0.00	0.00	0.00	0.00	18.44	1.29	1.23	null	null		null	null		null	null		null	null		-3.84E+00	2.28E-21	Down	-3.91E+00	1.91E-22	Down							
Unigene111995_All	227	42.87	20.31	20.88	37.09	33.07	64.86	85.49	45.85	33.35	-1.08E+00	1.25E-08	Down	-1.04E+00	9.21E-08	Down	-1.65E-01	4.71E-01		8.06E-01	1.89E-08		-8.99E-01	5.01E-15		-1.36E+00	2.39E-29	Down	Unigene111995_All//gi|81076822|gb|ABB55398.1|//3.00E-17//40S ribosomal protein S10-like [Solanum tuberosum]	Unigene111995_All//1.00E-15//AT5G52650| 40S ribosomal protein S10 (RPS10C)	Unigene111995_All//8.00E-103//TC134619		ko03010|Ribosome	GO:0030529	nr -
Unigene1120_All	2739	2.18	1.46	2.53	2.66	3.91	3.10	3.75	1.85	3.62	-5.75E-01	1.77E-02		2.15E-01	3.87E-01		5.58E-01	7.59E-04		2.23E-01	3.23E-01		-1.02E+00	3.87E-10	Down	-5.06E-02	7.67E-01		Unigene1120_All//gi|297796671|ref|XP_002866220.1|//0.00E+00//mitochondrial bifunctional diaminopelargonate synthetase [Arabidopsis lyrata subsp. lyrata] >gi|297312055|gb|EFH42479.1| mitochondrial bifunctional diaminopelargonate synthetase [Arabidopsis lyrata subsp. lyrata]	Unigene1120_All//6.00E-137//AT5G57590| BIO1 (biotin auxotroph 1); adenosylmethionine-8-amino-7-oxononanoate transaminase	Unigene1120_All//0.00E+00//gi|164345713|gb|ES826238.1|ES826238		ko00780|Biotin metabolism	GO:0016769//GO:0048037	nr -
Unigene11200_All	2538	1.63	1.90	1.93	4.14	5.06	3.10	6.03	2.72	2.70	2.20E-01	4.43E-01		2.45E-01	3.97E-01		2.89E-01	7.56E-02		-4.20E-01	1.83E-02		-1.15E+00	1.26E-17	Down	-1.16E+00	2.12E-18	Down	Unigene11200_All//gi|297812493|ref|XP_002874130.1|//1.00E-160//transducin family protein [Arabidopsis lyrata subsp. lyrata] >gi|297319967|gb|EFH50389.1| transducin family protein [Arabidopsis lyrata subsp. lyrata]	Unigene11200_All//4.00E-79//AT5G23430| transducin family protein / WD-40 repeat family protein	Unigene11200_All//0.00E+00//EX167732				nr -
Unigene112000_All	228	0.00	3.11	0.00	0.87	0.00	0.40	2.41	1.83	0.17	1.16E+01	3.33E-04	Up	null	null		-9.77E+00	8.47E-02		-1.14E+00	5.70E-01		-3.95E-01	6.34E-01		-3.79E+00	2.03E-03				Unigene112000_All//3.00E-07//BF270264				
Unigene112003_All	228	0.00	0.00	0.00	0.00	0.00	0.00	3.34	0.00	0.17	null	null		null	null		null	null		null	null		-1.17E+01	1.68E-05	Down	-4.26E+00	8.65E-05	Down							
Unigene112008_All	228	0.00	0.00	0.00	1.75	0.00	0.00	2.23	0.00	0.00	null	null		null	null		-1.08E+01	4.39E-03		-1.08E+01	1.11E-02		-1.11E+01	8.90E-04	Down	-1.11E+01	5.55E-04	Down							ESTscan +
Unigene112010_All	228	0.00	0.00	0.00	0.00	0.00	0.00	3.15	0.18	1.05	null	null		null	null		null	null		null	null		-4.10E+00	2.91E-04	Down	-1.59E+00	3.62E-02		Unigene112010_All//gi|112455658|gb|ABI18985.1|//4.00E-12//molecular chaperone DjA2 [Allium ampeloprasum]	Unigene112010_All//4.00E-12//AT5G22060| ATJ2; protein binding			ko04141|Protein processing in endoplasmic reticulum	GO:0005488	nr -
Unigene112011_All	228	0.00	0.00	0.00	0.00	0.00	0.00	2.60	0.00	0.00	null	null		null	null		null	null		null	null		-1.13E+01	2.39E-04	Down	-1.13E+01	1.41E-04	Down	Unigene112011_All//gi|283136138|gb|ADB11388.1|//5.00E-08//gamma class glutathione transferase EF1Bgamma3 [Populus trichocarpa]	Unigene112011_All//1.00E-05//AT1G57720| elongation factor 1B-gamma, putative / eEF-1B gamma, putative					nr +
Unigene11202_All	687	2.67	3.61	1.24	4.13	4.31	3.68	3.63	0.61	1.80	4.39E-01	2.84E-01		-1.10E+00	3.06E-02		6.07E-02	8.91E-01		-1.67E-01	6.96E-01		-2.58E+00	3.91E-09	Down	-1.01E+00	3.56E-03		Unigene11202_All//gi|75221490|sp|Q43715.1|TOC75_PEA//2.00E-90//RecName: Full=Protein TOC75, chloroplastic; AltName: Full=75 kDa translocon at the outer-envelope membrane of chloroplasts; AltName: Full=Import intermediate-associated protein of 75 kDa; AltName: Full=75 kDa chloroplast outer envelope protein; Flags: Precursor >gi|576507|gb|AAA53275.1| outer membrane protein [Pisum sativum] >gi|633607|emb|CAA58720.1| chloroplastic outer envelope membrane protein (OEP75) [Pisum sativum]	Unigene11202_All//2.00E-64//AT3G46740| TOC75-III (TRANSLOCON AT THE OUTER ENVELOPE MEMBRANE OF CHLOROPLASTS 75-III); P-P-bond-hydrolysis-driven protein transmembrane transporter	Unigene11202_All//3.00E-154//gi|78347599|gb|DT567873.1|DT567873			GO:0006605//GO:0008320//GO:0009653//GO:0009657//GO:0009707//GO:0031224	nr -
Unigene112022_All	229	2.74	8.41	4.88	3.48	2.86	4.34	9.79	12.59	3.48	1.62E+00	1.18E-03		8.33E-01	2.23E-01		-2.85E-01	7.21E-01		3.18E-01	6.73E-01		3.64E-01	3.01E-01		-1.49E+00	1.21E-04	Down			Unigene112022_All//2.00E-66//DW499434				
Unigene112024_All	229	0.23	0.00	0.00	0.87	0.60	0.00	2.77	0.18	0.52	-7.84E+00	6.18E-01		-7.84E+00	6.30E-01		-5.33E-01	7.49E-01		-9.77E+00	1.34E-01		-3.92E+00	9.81E-04	Down	-2.41E+00	8.12E-03		Unigene112024_All//gi|224119534|ref|XP_002318098.1|//6.00E-11//predicted protein [Populus trichocarpa] >gi|222858771|gb|EEE96318.1| predicted protein [Populus trichocarpa]		Unigene112024_All//7.00E-122//gi|109870617|gb|DW499593.1|DW499593			GO:0003824	nr -
Unigene112025_All	229	0.00	0.00	0.00	0.00	0.00	0.00	2.77	0.00	0.35	null	null		null	null		null	null		null	null		-1.14E+01	1.23E-04	Down	-2.99E+00	2.58E-03		Unigene112025_All//gi|222424342|dbj|BAH20127.1|//2.00E-09//AT5G35530 [Arabidopsis thaliana]				ko03010|Ribosome	GO:0005198//GO:0006970//GO:0010467//GO:0015935//GO:0031981	nr +
Unigene112026_All	230	0.00	0.00	0.00	0.00	0.00	0.00	2.39	0.00	0.00	null	null		null	null		null	null		null	null		-1.12E+01	4.61E-04	Down	-1.12E+01	2.81E-04	Down	Unigene112026_All//gi|6012952|emb|CAB57309.1|//4.00E-11//60S ribosomal protein L6 (YL 16 like) [Cyanophora paradoxa]	Unigene112026_All//7.00E-07//AT1G18540| 60S ribosomal protein L6 (RPL6A)			ko03010|Ribosome	GO:0005488//GO:0009536//GO:0015934//GO:0016020//GO:0031981	nr -
Unigene112027_All	230	0.00	0.00	0.00	0.00	0.00	0.00	2.76	0.00	0.35	null	null		null	null		null	null		null	null		-1.14E+01	1.23E-04	Down	-2.99E+00	2.58E-03								
Unigene11203_All	2343	17.95	11.20	8.45	11.01	11.74	11.58	13.52	6.07	8.48	-6.81E-01	2.64E-16		-1.09E+00	6.80E-34	Down	9.26E-02	4.67E-01		7.30E-02	5.96E-01		-1.16E+00	6.87E-36	Down	-6.72E-01	2.75E-15		Unigene11203_All//gi|15232625|ref|NP_190258.1|//0.00E+00//TOC75-III (TRANSLOCON AT THE OUTER ENVELOPE MEMBRANE OF CHLOROPLASTS 75-III); P-P-bond-hydrolysis-driven protein transmembrane transporter [Arabidopsis thaliana] >gi|75207662|sp|Q9STE8.1|TC753_ARATH RecName: Full=Protein TOC75-3, chloroplastic; AltName: Full=75 kDa translocon at the outer-envelope-membrane of chloroplasts 3; Short=AtTOC75-III; Flags: Precursor >gi|5541685|emb|CAB51191.1| chloroplast import-associated channel homolog [Arabidopsis thaliana] >gi|22022564|gb|AAM83239.1| AT3g46740/T6H20_230 [Arabidopsis thaliana] >gi|30102496|gb|AAP21166.1| At3g46740/T6H20_230 [Arabidopsis thaliana]	Unigene11203_All//0.00E+00//AT3G46740| TOC75-III (TRANSLOCON AT THE OUTER ENVELOPE MEMBRANE OF CHLOROPLASTS 75-III); P-P-bond-hydrolysis-driven protein transmembrane transporter	Unigene11203_All//0.00E+00//TC163188			GO:0006605//GO:0008320//GO:0009653//GO:0009657//GO:0009707//GO:0031224	nr -
Unigene112039_All	231	0.00	0.00	0.00	0.00	0.00	0.00	2.75	0.00	0.00	null	null		null	null		null	null		null	null		-1.14E+01	1.23E-04	Down	-1.14E+01	7.12E-05	Down	Unigene112039_All//gi|297820548|ref|XP_002878157.1|//1.00E-28//40S ribosomal protein S2 [Arabidopsis lyrata subsp. lyrata] >gi|297323995|gb|EFH54416.1| 40S ribosomal protein S2 [Arabidopsis lyrata subsp. lyrata]	Unigene112039_All//4.00E-30//AT3G57490| 40S ribosomal protein S2 (RPS2D)			ko03010|Ribosome	GO:0003676//GO:0005198//GO:0009536//GO:0010467//GO:0015935//GO:0016020//GO:0030312//GO:0031981	nr +
Unigene11204_All	2815	11.62	4.84	4.39	13.68	14.06	15.69	12.48	5.45	6.62	-1.26E+00	9.17E-35	Down	-1.40E+00	2.44E-39	Down	3.92E-02	7.42E-01		1.98E-01	1.79E-02		-1.20E+00	7.02E-42	Down	-9.14E-01	4.40E-28		Unigene11204_All//gi|15232625|ref|NP_190258.1|//0.00E+00//TOC75-III (TRANSLOCON AT THE OUTER ENVELOPE MEMBRANE OF CHLOROPLASTS 75-III); P-P-bond-hydrolysis-driven protein transmembrane transporter [Arabidopsis thaliana] >gi|75207662|sp|Q9STE8.1|TC753_ARATH RecName: Full=Protein TOC75-3, chloroplastic; AltName: Full=75 kDa translocon at the outer-envelope-membrane of chloroplasts 3; Short=AtTOC75-III; Flags: Precursor >gi|5541685|emb|CAB51191.1| chloroplast import-associated channel homolog [Arabidopsis thaliana] >gi|22022564|gb|AAM83239.1| AT3g46740/T6H20_230 [Arabidopsis thaliana] >gi|30102496|gb|AAP21166.1| At3g46740/T6H20_230 [Arabidopsis thaliana]	Unigene11204_All//0.00E+00//AT3G46740| TOC75-III (TRANSLOCON AT THE OUTER ENVELOPE MEMBRANE OF CHLOROPLASTS 75-III); P-P-bond-hydrolysis-driven protein transmembrane transporter	Unigene11204_All//0.00E+00//TC144595			GO:0006605//GO:0008320//GO:0009653//GO:0009657//GO:0009707//GO:0031224	nr -
Unigene112040_All	231	118.91	53.96	77.92	161.09	149.67	169.90	178.82	107.11	104.51	-1.14E+00	4.86E-25	Down	-6.10E-01	1.04E-08		-1.06E-01	3.05E-01		7.68E-02	4.81E-01		-7.39E-01	1.92E-22		-7.75E-01	5.42E-25		Unigene112040_All//gi|192912962|gb|ACF06589.1|//2.00E-31//ribosomal protein [Elaeis guineensis]	Unigene112040_All//3.00E-32//AT1G67430| 60S ribosomal protein L17 (RPL17B)	Unigene112040_All//3.00E-118//TC164010		ko03010|Ribosome	GO:0005198//GO:0010467//GO:0016787//GO:0019538//GO:0033279	nr -
Unigene112045_All	231	1.36	1.10	0.46	1.29	1.34	0.78	9.88	5.97	2.76	-3.09E-01	7.89E-01		-1.56E+00	3.30E-01		5.22E-02	9.99E-01		-7.27E-01	6.39E-01		-7.27E-01	5.48E-02		-1.84E+00	4.73E-06	Down			Unigene112045_All//9.00E-109//gi|109861542|gb|DW490519.1|DW490519				
Unigene11205_All	2883	7.19	4.83	3.80	8.10	7.13	6.89	5.87	2.29	3.98	-5.72E-01	2.02E-06		-9.18E-01	5.46E-13		-1.85E-01	1.26E-01		-2.34E-01	5.00E-02		-1.36E+00	7.18E-25	Down	-5.60E-01	1.89E-06		Unigene11205_All//gi|297819256|ref|XP_002877511.1|//0.00E+00//translocon outer membrane complex 75-III [Arabidopsis lyrata subsp. lyrata] >gi|297323349|gb|EFH53770.1| translocon outer membrane complex 75-III [Arabidopsis lyrata subsp. lyrata]	Unigene11205_All//0.00E+00//AT3G46740| TOC75-III (TRANSLOCON AT THE OUTER ENVELOPE MEMBRANE OF CHLOROPLASTS 75-III); P-P-bond-hydrolysis-driven protein transmembrane transporter	Unigene11205_All//0.00E+00//TC144595			GO:0006605//GO:0008320//GO:0009653//GO:0009657//GO:0009707//GO:0031224	nr +
Unigene112054_All	231	0.00	0.00	0.00	0.00	0.00	0.00	4.21	0.18	0.00	null	null		null	null		null	null		null	null		-4.54E+00	6.92E-06	Down	-1.20E+01	2.72E-07	Down			Unigene112054_All//6.00E-110//CK640449				
Unigene112068_All	232	0.00	0.00	0.00	0.00	0.00	0.00	2.19	0.72	0.00	null	null		null	null		null	null		null	null		-1.60E+00	1.07E-01		-1.11E+01	5.55E-04	Down	Unigene112068_All//gi|297735100|emb|CBI17462.3|//2.00E-23//unnamed protein product [Vitis vinifera]	Unigene112068_All//1.00E-05//AT1G19630| CYP722A1; electron carrier/ heme binding / iron ion binding / monooxygenase/ oxygen binding				GO:0005506//GO:0008152//GO:0016491	nr -

Unigene112070_All	233	0.00	0.00	0.00	0.00	0.00	0.00	2.72	0.18	0.68	null	null		null	null		null	null		null	null		-3.92E+00	9.81E-04	Down	-1.99E+00	2.04E-02		Unigene112070_All//gi|224085587|ref|XP_002307628.1|//4.00E-21//glycosyltransferase, CAZy family GT8 [Populus trichocarpa] >gi|222857077|gb|EEE94624.1| glycosyltransferase, CAZy family GT8 [Populus trichocarpa]	Unigene112070_All//2.00E-19//AT5G47780| GAUT4 (Galacturonosyltransferase 4); polygalacturonate 4-alpha-galacturonosyltransferase/ transferase, transferring glycosyl groups	Unigene112070_All//2.00E-26//TC169308			GO:0016740	nr +
Unigene112076_All	233	416.10	307.93	398.58	83.38	126.80	87.42	29.22	46.10	67.20	-4.34E-01	1.18E-16		-6.21E-02	3.42E-01		6.05E-01	5.41E-11		6.83E-02	6.53E-01		6.58E-01	2.12E-05		1.20E+00	1.09E-19	Up	Unigene112076_All//gi|115450991|ref|NP_001049096.1|//8.00E-35//Os03g0169100 [Oryza sativa Japonica Group] >gi|109940150|sp|Q9ZTP5.2|RPE_ORYSJ RecName: Full=Ribulose-phosphate 3-epimerase, chloroplastic; AltName: Full=Pentose-5-phosphate 3-epimerase; Short=PPE; AltName: Full=R5P3E; Short=RPE; Flags: Precursor >gi|19071636|gb|AAL84303.1|AC073556_20 ribulose-5-phosphate-3-epimerase [Oryza sativa Japonica Group] >gi|113547567|dbj|BAF11010.1| Os03g0169100 [Oryza sativa Japonica Group]	Unigene112076_All//8.00E-35//AT5G61410| RPE; catalytic/ ribulose-phosphate 3-epimerase	Unigene112076_All//5.00E-129//gi|164350992|gb|ES831405.1|ES831405		ko00710|Carbon fixation in photosynthetic organisms//ko00040|Pentose and glucuronate interconversions//ko00030|Pentose phosphate pathway	GO:0003006//GO:0005488//GO:0005576//GO:0006007//GO:0006950//GO:0009526//GO:0009532//GO:0009534//GO:0015977//GO:0016857//GO:0051707	nr -
Unigene112080_All	233	0.00	0.00	0.00	0.00	0.00	0.00	3.27	1.79	0.00	null	null		null	null		null	null		null	null		-8.65E-01	2.39E-01		-1.17E+01	8.88E-06	Down	Unigene112080_All//sp|P42312|NUPG_BACSU//4.00E-08//Purine nucleoside transport protein nupG OS=Bacillus subtilis GN=nupG PE=1 SV=2						swissprot -
Unigene112081_All	233	23.80	26.31	12.11	16.68	17.29	10.47	98.53	48.79	37.79	1.45E-01	5.93E-01		-9.75E-01	2.41E-04		5.23E-02	8.88E-01		-6.72E-01	3.04E-02		-1.01E+00	2.31E-21	Down	-1.38E+00	1.74E-35	Down			Unigene112081_All//4.00E-114//TC163662				
Unigene112084_All	233	4.72	0.65	3.66	2.35	0.44	0.58	2.36	5.38	0.68	-2.85E+00	4.77E-04	Down	-3.67E-01	6.01E-01		-2.41E+00	2.44E-02		-2.02E+00	7.05E-02		1.19E+00	3.01E-02		-1.79E+00	5.07E-02		Unigene112084_All//gi|2677828|gb|AAB97142.1|//5.00E-11//cysteine protease [Prunus armeniaca]	Unigene112084_All//1.00E-11//AT5G60360| AALP (Arabidopsis aleurain-like protease); cysteine-type peptidase	Unigene112084_All//7.00E-54//TC140087			GO:0004175//GO:0005488//GO:0009725//GO:0019538//GO:0032502//GO:0043231	nr +
Unigene112093_All	234	0.00	0.00	0.00	0.00	0.00	0.00	4.52	1.25	0.00	null	null		null	null		null	null		null	null		-1.85E+00	4.09E-03		-1.21E+01	6.72E-08	Down							
Unigene112099_All	234	0.00	1.08	1.37	2.34	0.00	1.74	2.53	2.86	0.68	1.01E+01	7.88E-02		1.04E+01	4.53E-02		-1.12E+01	4.01E-04	Down	-4.31E-01	6.67E-01		1.76E-01	8.15E-01		-1.89E+00	3.22E-02				Unigene112099_All//3.00E-07//TC148696				
Unigene11210_All	826	31.23	10.61	21.47	3.02	5.42	3.61	2.56	1.52	2.65	-1.56E+00	1.27E-37	Down	-5.41E-01	9.32E-07		8.46E-01	2.16E-03		2.59E-01	5.30E-01		-7.54E-01	5.72E-02		5.17E-02	9.10E-01		Unigene11210_All//gi|259130061|gb|ACV95483.1|//1.00E-73//TCP4 [Citrus trifoliata]	Unigene11210_All//8.00E-57//AT3G15030| TCP4 (TCP family transcription factor 4); transcription factor	Unigene11210_All//0.00E+00//TC147925	TCP		GO:0003006//GO:0003676//GO:0009887//GO:0010468//GO:0040034//GO:0048827//GO:0048869	nr -
Unigene112100_All	234	0.00	0.00	0.00	0.00	0.00	0.00	3.79	0.89	0.17	null	null		null	null		null	null		null	null		-2.09E+00	4.71E-03		-4.48E+00	1.23E-05	Down	Unigene112100_All//gi|297833728|ref|XP_002884746.1|//1.00E-30//40S ribosomal protein S23 [Arabidopsis lyrata subsp. lyrata] >gi|297330586|gb|EFH61005.1| 40S ribosomal protein S23 [Arabidopsis lyrata subsp. lyrata]	Unigene112100_All//5.00E-32//AT5G02960| 40S ribosomal protein S23 (RPS23B)	Unigene112100_All//7.00E-11//DV850314		ko03010|Ribosome	GO:0005198//GO:0010467//GO:0033279	nr -
Unigene112117_All	235	0.00	0.00	0.00	0.00	0.00	0.00	3.42	0.71	0.00	null	null		null	null		null	null		null	null		-2.26E+00	4.82E-03		-1.17E+01	4.43E-06	Down	Unigene112117_All//gi|1545805|dbj|BAA10929.1|//8.00E-09//cytochrome P450 like_TBP [Nicotiana tabacum]		Unigene112117_All//4.00E-09//EV495741				nr +
Unigene112118_All	235	0.00	0.00	0.00	0.00	0.00	0.00	4.14	0.00	0.00	null	null		null	null		null	null		null	null		-1.20E+01	5.84E-07	Down	-1.20E+01	2.72E-07	Down							
Unigene11212_All	2264	15.25	8.75	12.37	1.01	1.16	1.00	2.00	0.35	0.33	-8.02E-01	9.81E-18		-3.02E-01	1.67E-03		1.92E-01	6.70E-01		-2.11E-02	9.50E-01		-2.51E+00	1.45E-15	Down	-2.58E+00	1.34E-16	Down	Unigene11212_All//gi|295841589|dbj|BAJ07175.1|//1.00E-119//MdTCP4A [Malus x domestica]	Unigene11212_All//5.00E-82//AT3G15030| TCP4 (TCP family transcription factor 4); transcription factor	Unigene11212_All//0.00E+00//TC144631	TCP		GO:0009887//GO:0040034//GO:0048827//GO:0048869	nr -
Unigene112121_All	236	0.00	0.00	0.00	0.42	0.00	0.00	2.33	0.89	0.00	null	null		null	null		-8.72E+00	3.16E-01		-8.72E+00	4.04E-01		-1.40E+00	1.35E-01		-1.12E+01	2.81E-04	Down							
Unigene112123_All	236	0.00	0.00	0.00	0.00	0.00	0.00	2.51	0.35	0.00	null	null		null	null		null	null		null	null		-2.82E+00	7.18E-03		-1.13E+01	1.41E-04	Down	Unigene112123_All//gi|159465349|ref|XP_001690885.1|//2.00E-24//ribosomal protein L18, component of cytosolic 80S ribosome and 60S large subunit [Chlamydomonas reinhardtii] >gi|158279571|gb|EDP05331.1| ribosomal protein L18, component of cytosolic 80S ribosome and 60S large subunit [Chlamydomonas reinhardtii]	Unigene112123_All//7.00E-20//AT3G05590| RPL18 (RIBOSOMAL PROTEIN L18); structural constituent of ribosome			ko03010|Ribosome	GO:0005198//GO:0010467//GO:0030529	nr -
Unigene11213_All	2023	21.62	10.29	13.35	2.29	3.32	1.88	1.90	1.57	1.20	-1.07E+00	1.04E-35	Down	-6.96E-01	7.27E-17		5.36E-01	1.59E-02		-2.88E-01	3.57E-01		-2.77E-01	3.72E-01		-6.63E-01	1.35E-02		Unigene11213_All//gi|259130061|gb|ACV95483.1|//1.00E-123//TCP4 [Citrus trifoliata]	Unigene11213_All//9.00E-80//AT3G15030| TCP4 (TCP family transcription factor 4); transcription factor	Unigene11213_All//0.00E+00//TC147925	TCP		GO:0009887//GO:0010468//GO:0040034//GO:0048827//GO:0048869	nr +
Unigene11214_All	2261	62.29	22.77	41.99	6.15	5.74	3.44	4.60	2.42	2.22	-1.45E+00	8.24E-183	Down	-5.69E-01	3.15E-37		-9.84E-02	5.82E-01		-8.39E-01	1.27E-08		-9.26E-01	8.10E-09		-1.05E+00	4.82E-11	Down	Unigene11214_All//gi|37783285|gb|AAO43102.1|//1.00E-112//mutant cincinnata [Antirrhinum majus]	Unigene11214_All//2.00E-86//AT3G15030| TCP4 (TCP family transcription factor 4); transcription factor	Unigene11214_All//0.00E+00//TC136853	TCP		GO:0003006//GO:0003676//GO:0009887//GO:0040034//GO:0048827//GO:0048869	nr +
Unigene112141_All	237	0.00	0.00	0.00	0.00	0.00	0.00	8.21	0.71	0.67	null	null		null	null		null	null		null	null		-3.54E+00	1.27E-09	Down	-3.61E+00	3.72E-10	Down							ESTscan -
Unigene112143_All	237	0.00	0.00	0.00	0.00	0.15	0.00	3.21	1.06	0.00	null	null		null	null		7.18E+00	8.36E-01		null	null		-1.60E+00	3.70E-02		-1.16E+01	8.88E-06	Down	Unigene112143_All//gi|18404062|ref|NP_565836.1|//1.00E-17//ubiquitin extension protein 2 (UBQ2) / 60S ribosomal protein L40 (RPL40A) [Arabidopsis thaliana] >gi|18409639|ref|NP_566969.1| UBQ1 (UBIQUITIN EXTENSION PROTEIN 1); protein binding / structural constituent of ribosome [Arabidopsis thaliana] >gi|166930|gb|AAA32904.1| ubiquitin extension protein (UBQ1) [Arabidopsis thaliana] >gi|166932|gb|AAA32905.1| ubiquitin extension protein (UBQ2) [Arabidopsis thaliana] >gi|4886268|emb|CAB43405.1| ubiquitin / ribosomal protein CEP52 [Arabidopsis thaliana] >gi|20198116|gb|AAM15407.1| ubiquitin extension protein (UBQ2) [Arabidopsis thaliana] >gi|21553955|gb|AAM63036.1| ubiquitin extension protein UBQ1 [Arabidopsis thaliana] >gi|222424333|dbj|BAH20123.1| AT2G36170 [Arabidopsis thaliana]	Unigene112143_All//5.00E-19//AT3G52590| UBQ1 (UBIQUITIN EXTENSION PROTEIN 1); protein binding / structural constituent of ribosome			ko03010|Ribosome	GO:0003006//GO:0005198//GO:0005488//GO:0010467//GO:0015934//GO:0031981//GO:0032446	nr -
Unigene112147_All	237	0.00	0.00	0.00	0.00	0.00	0.00	2.50	0.00	0.00	null	null		null	null		null	null		null	null		-1.13E+01	2.39E-04	Down	-1.13E+01	1.41E-04	Down	Unigene112147_All//sp|Q35920|ATP6_SALSA//1.00E-07//ATP synthase subunit a OS=Salmo salar GN=mt-atp6 PE=3 SV=3						swissprot -
Unigene11215_All	966	5.25	2.99	2.98	7.37	4.85	5.75	10.11	5.49	3.84	-8.13E-01	2.22E-03		-8.20E-01	3.14E-03		-6.05E-01	3.30E-03		-3.59E-01	1.21E-01		-8.80E-01	1.04E-07		-1.40E+00	7.45E-16	Down	Unigene11215_All//gi|15221423|ref|NP_172112.1|//1.00E-123//MEE5 (MATERNAL EFFECT EMBRYO ARREST 5); GTP binding / GTPase/ translation elongation factor/ translation factor, nucleic acid binding [Arabidopsis thaliana] >gi|30679607|ref|NP_849600.1| MEE5 (MATERNAL EFFECT EMBRYO ARREST 5); GTP binding / GTPase/ translation elongation factor/ translation factor, nucleic acid binding [Arabidopsis thaliana] >gi|8844127|gb|AAF80219.1|AC025290_8 Contains similarity to an U5 snRNP-specific protein 116 kD from Homo sapiens gi|4759280 and contains elongation factor G C-terminus PF|00679 and is a member of the elongation factor Tu family PF|00009 [Arabidopsis thaliana] >gi|110741512|dbj|BAE98706.1| elongation factor like protein [Arabidopsis thaliana]	Unigene11215_All//7.00E-125//AT1G06220| MEE5 (MATERNAL EFFECT EMBRYO ARREST 5); GTP binding / GTPase/ translation elongation factor/ translation factor, nucleic acid binding	Unigene11215_All//0.00E+00//TC153558		ko03040|Spliceosome		nr +
Unigene112150_All	237	0.00	0.00	0.00	0.00	0.00	0.00	17.13	1.06	0.34	null	null		null	null		null	null		null	null		-4.02E+00	2.01E-21	Down	-5.67E+00	9.26E-27	Down			Unigene112150_All//4.00E-09//CD809343				
Unigene11216_All	994	8.95	5.35	3.11	9.67	6.93	8.81	15.70	8.75	6.61	-7.41E-01	1.24E-04		-1.53E+00	1.77E-12	Down	-4.81E-01	6.23E-03		-1.34E-01	5.50E-01		-8.44E-01	4.45E-11		-1.25E+00	6.34E-21	Down	Unigene11216_All//gi|15221423|ref|NP_172112.1|//1.00E-127//MEE5 (MATERNAL EFFECT EMBRYO ARREST 5); GTP binding / GTPase/ translation elongation factor/ translation factor, nucleic acid binding [Arabidopsis thaliana] >gi|30679607|ref|NP_849600.1| MEE5 (MATERNAL EFFECT EMBRYO ARREST 5); GTP binding / GTPase/ translation elongation factor/ translation factor, nucleic acid binding [Arabidopsis thaliana] >gi|8844127|gb|AAF80219.1|AC025290_8 Contains similarity to an U5 snRNP-specific protein 116 kD from Homo sapiens gi|4759280 and contains elongation factor G C-terminus PF|00679 and is a member of the elongation factor Tu family PF|00009 [Arabidopsis thaliana] >gi|110741512|dbj|BAE98706.1| elongation factor like protein [Arabidopsis thaliana]	Unigene11216_All//1.00E-128//AT1G06220| MEE5 (MATERNAL EFFECT EMBRYO ARREST 5); GTP binding / GTPase/ translation elongation factor/ translation factor, nucleic acid binding	Unigene11216_All//0.00E+00//gi|48778046|gb|CO087412.1|CO087412		ko03040|Spliceosome		nr -
Unigene11217_All	1411	1.97	2.01	1.85	2.47	3.64	3.55	5.33	2.22	2.88	3.32E-02	9.46E-01		-8.78E-02	8.19E-01		5.57E-01	3.32E-02		5.24E-01	5.63E-02		-1.26E+00	2.14E-10	Down	-8.89E-01	1.88E-06		Unigene11217_All//gi|110741074|dbj|BAE98631.1|//0.00E+00//elongation factor like protein [Arabidopsis thaliana]	Unigene11217_All//0.00E+00//AT1G06220| MEE5 (MATERNAL EFFECT EMBRYO ARREST 5); GTP binding / GTPase/ translation elongation factor/ translation factor, nucleic acid binding	Unigene11217_All//0.00E+00//DW481638		ko03040|Spliceosome	GO:0003006//GO:0003723//GO:0016020//GO:0016604//GO:0017111//GO:0030312//GO:0032561//GO:0032991//GO:0045595	nr +
Unigene112172_All	238	0.00	0.00	0.00	1.05	0.14	0.38	3.38	0.00	0.50	null	null		null	null		-2.85E+00	1.37E-01		-1.46E+00	4.07E-01		-1.17E+01	8.58E-06	Down	-2.75E+00	9.43E-04	Down							
Unigene112173_All	238	0.00	0.00	0.00	0.00	0.00	0.00	2.31	0.35	0.00	null	null		null	null		null	null		null	null		-2.72E+00	1.22E-02		-1.12E+01	2.81E-04	Down							

<table>
<tr><td>Unigene11218_All</td><td>2243</td><td>4.76</td><td>4.04</td><td>2.71</td><td>5.29</td><td>5.34</td><td>7.55</td><td>12.03</td><td>4.23</td><td>5.12</td><td>-2.35E-01</td><td>2.09E-01</td><td></td><td>-8.14E-01</td><td>7.29E-06</td><td></td><td>1.54E-02</td><td>9.53E-01</td><td></td><td>5.15E-01</td><td>1.00E-04</td><td></td><td>-1.51E+00</td><td>8.28E-46</td><td>Down</td><td>-1.23E+00</td><td>6.19E-35</td><td>Down</td><td>Unigene11218_All//gi|15221423|ref|NP_172112.1|//0.00E+00//MEE5 (MATERNAL EFFECT EMBRYO ARREST 5); GTP binding / GTPase/ translation elongation factor/ translation factor, nucleic acid binding [Arabidopsis thaliana] >gi|30679607|ref|NP_849600.1| MEE5 (MATERNAL EFFECT EMBRYO ARREST 5); GTP binding / GTPase/ translation elongation factor/ translation factor, nucleic acid binding [Arabidopsis thaliana] >gi|8844127|gb|AAF80219.1|AC025290_8 Contains similarity to an U5 snRNP-specific protein 116 kD from Homo sapiens gi|4759280 and contains elongation factor G C-terminus PF|00679 and is a member of the elongation factor Tu family PF|00009 [Arabidopsis thaliana] >gi|110741512|dbj|BAE98706.1| elongation factor like protein [Arabidopsis thaliana]</td><td>Unigene11218_All//0.00E+00//AT1G06220| MEE5 (MATERNAL EFFECT EMBRYO ARREST 5); GTP binding / GTPase/ translation elongation factor/ translation factor, nucleic acid binding</td><td>Unigene11218_All//0.00E+00//TC158929</td><td></td><td>ko03040|Spliceosome</td><td>GO:0003006//GO:0008135//GO:0016020//GO:0016604//GO:0017111//GO:0030312//GO:0032561//GO:0032991//GO:0045595</td><td>nr +</td></tr>
<tr><td>Unigene112180_All</td><td>239</td><td>0.00</td><td>0.00</td><td>0.00</td><td>0.00</td><td>0.00</td><td>0.00</td><td>4.60</td><td>1.22</td><td>0.00</td><td>null</td><td>null</td><td></td><td>null</td><td>null</td><td></td><td>null</td><td>null</td><td></td><td>null</td><td>null</td><td></td><td>-1.91E+00</td><td>2.64E-03</td><td></td><td>-1.22E+01</td><td>3.33E-08</td><td>Down</td><td></td><td>Unigene112180_All//5.00E-05//AT2G40935| unknown protein</td><td></td><td></td><td></td><td></td><td>ESTscan +</td></tr>
<tr><td>Unigene112181_All</td><td>239</td><td>0.00</td><td>0.00</td><td>0.00</td><td>0.00</td><td>0.00</td><td>0.00</td><td>2.48</td><td>0.00</td><td>0.17</td><td>null</td><td>null</td><td></td><td>null</td><td>null</td><td></td><td>null</td><td>null</td><td></td><td>null</td><td>null</td><td></td><td>-1.13E+01</td><td>2.39E-04</td><td>Down</td><td>-3.89E+00</td><td>1.09E-03</td><td></td><td>Unigene112181_All//gi|50843988|gb|AAT84169.1|//3.00E-23//60S ribosomal protein L27 [Chara globularis]</td><td>Unigene112181_All//1.00E-15//AT4G15000| 60S ribosomal protein L27 (RPL27C)</td><td></td><td></td><td>ko03010|Ribosome</td><td>GO:0005198//GO:0010467//GO:0030529</td><td>nr -</td></tr>
<tr><td>Unigene112189_All</td><td>240</td><td>0.00</td><td>0.00</td><td>0.00</td><td>0.21</td><td>0.00</td><td>0.00</td><td>5.81</td><td>1.39</td><td>1.83</td><td>null</td><td>null</td><td></td><td>null</td><td>null</td><td></td><td>-7.70E+00</td><td>5.61E-01</td><td></td><td>-7.70E+00</td><td>6.42E-01</td><td></td><td>-2.06E+00</td><td>2.51E-04</td><td>Down</td><td>-1.67E+00</td><td>1.27E-03</td><td></td><td></td><td></td><td></td><td></td><td></td><td></td><td></td></tr>
<tr><td>Unigene11219_All</td><td>2177</td><td>5.96</td><td>3.84</td><td>3.35</td><td>6.45</td><td>5.33</td><td>7.78</td><td>10.78</td><td>3.76</td><td>5.62</td><td>-6.34E-01</td><td>4.86E-05</td><td></td><td>-8.31E-01</td><td>3.43E-07</td><td></td><td>-2.76E-01</td><td>7.05E-02</td><td></td><td>2.70E-01</td><td>5.84E-02</td><td></td><td>-1.52E+00</td><td>2.31E-40</td><td>Down</td><td>-9.40E-01</td><td>6.27E-20</td><td></td><td>Unigene11219_All//gi|110741074|dbj|BAE98631.1|//0.00E+00//elongation factor like protein [Arabidopsis thaliana]</td><td>Unigene11219_All//0.00E+00//AT1G06220| MEE5 (MATERNAL EFFECT EMBRYO ARREST 5); GTP binding / GTPase/ translation elongation factor/ translation factor, nucleic acid binding</td><td>Unigene11219_All//0.00E+00//DW481638</td><td></td><td>ko03040|Spliceosome</td><td>GO:0003006//GO:0008135//GO:0016020//GO:0016604//GO:0017111//GO:0030312//GO:0032561//GO:0032991//GO:0045595</td><td>nr -</td></tr>
<tr><td>Unigene1122_All</td><td>2099</td><td>12.69</td><td>20.40</td><td>15.12</td><td>1.69</td><td>4.02</td><td>4.86</td><td>78.90</td><td>44.86</td><td>79.92</td><td>6.85E-01</td><td>1.01E-16</td><td></td><td>2.53E-01</td><td>1.36E-02</td><td></td><td>1.25E+00</td><td>1.31E-10</td><td>Up</td><td>1.53E+00</td><td>1.00E-15</td><td>Up</td><td>-8.15E-01</td><td>2.35E-103</td><td></td><td>1.86E-02</td><td>6.60E-01</td><td></td><td>Unigene1122_All//gi|56157408|gb|AAV80420.1|//4.00E-62//Prf interactor 30137 [Solanum lycopersicum]</td><td>Unigene1122_All//2.00E-41//AT2G27230| LHW (LONESOME HIGHWAY); protein homodimerization/ transcription activator/ transcription factor</td><td>Unigene1122_All//2.00E-114//TC150417</td><td>bHLH</td><td></td><td></td><td>nr +</td></tr>
<tr><td>Unigene112208_All</td><td>241</td><td>0.00</td><td>4.20</td><td>0.00</td><td>0.62</td><td>0.57</td><td>1.87</td><td>3.33</td><td>3.47</td><td>1.65</td><td>1.20E+01</td><td>7.26E-06</td><td>Up</td><td>null</td><td>null</td><td></td><td>-1.18E-01</td><td>9.11E-01</td><td></td><td>1.60E+00</td><td>2.05E-01</td><td></td><td>5.74E-02</td><td>9.54E-01</td><td></td><td>-1.01E+00</td><td>1.35E-01</td><td></td><td></td><td></td><td></td><td></td><td></td><td></td><td></td></tr>
<tr><td>Unigene112209_All</td><td>241</td><td>0.00</td><td>0.00</td><td>0.00</td><td>0.00</td><td>0.00</td><td>0.00</td><td>2.46</td><td>0.00</td><td>0.00</td><td>null</td><td>null</td><td></td><td>null</td><td>null</td><td></td><td>null</td><td>null</td><td></td><td>null</td><td>null</td><td></td><td>-1.13E+01</td><td>2.39E-04</td><td>Down</td><td>-1.13E+01</td><td>1.41E-04</td><td>Down</td><td>Unigene112209_All//gi|115454357|ref|NP_001050779.1|//2.00E-23//Os03g0648400 [Oryza sativa Japonica Group] >gi|29367357|gb|AAO72551.1| DNAJ-like protein [Oryza sativa Japonica Group] >gi|113549250|dbj|BAF12693.1| Os03g0648400 [Oryza sativa Japonica Group] >gi|169244473|gb|ACA50510.1| DnaJ protein [Oryza sativa Japonica Group]</td><td>Unigene112209_All//1.00E-23//AT5G22060| ATJ2; protein binding</td><td></td><td></td><td>ko04141|Protein processing in endoplasmic reticulum</td><td>GO:0005515//GO:0006950//GO:0032559//GO:0043169//GO:0044267//GO:0044424</td><td>nr +</td></tr>
<tr><td>Unigene112214_All</td><td>241</td><td>0.00</td><td>0.00</td><td>0.00</td><td>0.00</td><td>0.00</td><td>0.00</td><td>2.98</td><td>0.17</td><td>0.17</td><td>null</td><td>null</td><td></td><td>null</td><td>null</td><td></td><td>null</td><td>null</td><td></td><td>null</td><td>null</td><td></td><td>-4.10E+00</td><td>2.91E-04</td><td>Down</td><td>-4.17E+00</td><td>1.64E-04</td><td>Down</td><td>Unigene112214_All//gi|226507440|ref|NP_001152131.1|//1.00E-23//LOC100285769 [Zea mays] >gi|195653083|gb|ACG46009.1| 60S ribosomal protein L30 [Zea mays]</td><td>Unigene112214_All//2.00E-22//AT3G18740| 60S ribosomal protein L30 (RPL30C)</td><td></td><td></td><td>ko03010|Ribosome</td><td>GO:0005198//GO:0010467//GO:0030529</td><td>nr -</td></tr>
<tr><td>Unigene11222_All</td><td>986</td><td>8.49</td><td>5.24</td><td>6.59</td><td>12.02</td><td>14.95</td><td>16.12</td><td>19.98</td><td>9.07</td><td>9.90</td><td>-6.96E-01</td><td>4.92E-04</td><td></td><td>-3.66E-01</td><td>8.85E-02</td><td></td><td>3.14E-01</td><td>3.25E-02</td><td></td><td>4.23E-01</td><td>2.37E-03</td><td></td><td>-1.14E+00</td><td>3.95E-22</td><td>Down</td><td>-1.01E+00</td><td>5.31E-19</td><td>Down</td><td>Unigene11222_All//gi|146286094|sp|Q0WW26.2|COPG_ARATH//1.00E-140//RecName: Full=Coatomer subunit gamma; AltName: Full=Gamma-coat protein; Short=Gamma-COP >gi|222424705|dbj|BAH20306.1| AT4G34450 [Arabidopsis thaliana]</td><td>Unigene11222_All//5.00E-136//AT4G34450| coatomer gamma-2 subunit, putative / gamma-2 coat protein, putative / gamma-2 COP, putative</td><td>Unigene11222_All//0.00E+00//gi|78337737|gb|DT558011.1|DT558011</td><td></td><td></td><td></td><td>nr +</td></tr>
<tr><td>Unigene11223_All</td><td>1525</td><td>4.87</td><td>2.86</td><td>3.04</td><td>10.91</td><td>7.07</td><td>7.26</td><td>16.50</td><td>8.55</td><td>7.78</td><td>-7.70E-01</td><td>3.23E-04</td><td></td><td>-6.81E-01</td><td>2.19E-03</td><td></td><td>-6.26E-01</td><td>5.32E-07</td><td></td><td>-5.89E-01</td><td>8.86E-06</td><td></td><td>-9.48E-01</td><td>5.92E-21</td><td></td><td>-1.08E+00</td><td>1.09E-26</td><td>Down</td><td>Unigene11223_All//gi|146286094|sp|Q0WW26.2|COPG_ARATH//0.00E+00//RecName: Full=Coatomer subunit gamma; AltName: Full=Gamma-coat protein; Short=Gamma-COP >gi|222424705|dbj|BAH20306.1| AT4G34450 [Arabidopsis thaliana]</td><td>Unigene11223_All//0.00E+00//AT4G34450| coatomer gamma-2 subunit, putative / gamma-2 coat protein, putative / gamma-2 COP, putative</td><td>Unigene11223_All//0.00E+00//gi|109854135|gb|DW483114.1|DW483114</td><td></td><td></td><td>GO:0005515//GO:0009536//GO:0015031//GO:0030120</td><td>nr -</td></tr>
<tr><td>Unigene112235_All</td><td>243</td><td>0.43</td><td>0.00</td><td>0.00</td><td>1.23</td><td>0.71</td><td>0.19</td><td>3.65</td><td>1.55</td><td>0.66</td><td>-8.75E+00</td><td>3.75E-01</td><td></td><td>-8.75E+00</td><td>3.97E-01</td><td></td><td>-7.96E-01</td><td>5.57E-01</td><td></td><td>-2.73E+00</td><td>1.37E-01</td><td></td><td>-1.24E+00</td><td>6.79E-02</td><td></td><td>-2.48E+00</td><td>9.93E-04</td><td>Down</td><td></td><td></td><td></td><td></td><td></td><td></td><td></td></tr>
<tr><td>Unigene112236_All</td><td>243</td><td>0.00</td><td>0.00</td><td>0.00</td><td>0.00</td><td>0.00</td><td>0.00</td><td>2.78</td><td>0.52</td><td>0.00</td><td>null</td><td>null</td><td></td><td>null</td><td>null</td><td></td><td>null</td><td>null</td><td></td><td>null</td><td>null</td><td></td><td>-2.43E+00</td><td>7.77E-03</td><td></td><td>-1.14E+01</td><td>3.57E-05</td><td>Down</td><td></td><td></td><td>Unigene112236_All//2.00E-11//ES843311</td><td></td><td></td><td></td><td></td></tr>
<tr><td>Unigene112238_All</td><td>244</td><td>0.00</td><td>0.00</td><td>0.00</td><td>0.00</td><td>0.00</td><td>0.00</td><td>3.47</td><td>0.17</td><td>0.49</td><td>null</td><td>null</td><td></td><td>null</td><td>null</td><td></td><td>null</td><td>null</td><td></td><td>null</td><td>null</td><td></td><td>-4.34E+00</td><td>4.55E-05</td><td>Down</td><td>-2.82E+00</td><td>5.38E-04</td><td>Down</td><td></td><td></td><td>Unigene112238_All//1.00E-12//CD486305</td><td></td><td></td><td></td><td></td></tr>
<tr><td>Unigene11224_All</td><td>1408</td><td>11.37</td><td>6.55</td><td>5.22</td><td>24.27</td><td>21.86</td><td>18.93</td><td>27.48</td><td>15.14</td><td>12.17</td><td>-7.96E-01</td><td>1.49E-08</td><td></td><td>-1.12E+00</td><td>5.97E-14</td><td>Down</td><td>-1.51E-01</td><td>1.30E-01</td><td></td><td>-3.59E-01</td><td>6.67E-05</td><td></td><td>-8.60E-01</td><td>4.37E-27</td><td></td><td>-1.18E+00</td><td>4.04E-46</td><td>Down</td><td>Unigene11224_All//gi|146286094|sp|Q0WW26.2|COPG_ARATH//0.00E+00//RecName: Full=Coatomer subunit gamma; AltName: Full=Gamma-coat protein; Short=Gamma-COP >gi|222424705|dbj|BAH20306.1| AT4G34450 [Arabidopsis thaliana]</td><td>Unigene11224_All//0.00E+00//AT4G34450| coatomer gamma-2 subunit, putative / gamma-2 coat protein, putative / gamma-2 COP, putative</td><td>Unigene11224_All//0.00E+00//TC140277</td><td></td><td></td><td>GO:0005515//GO:0009536//GO:0015031//GO:0030120</td><td>nr +</td></tr>
<tr><td>Unigene112247_All</td><td>245</td><td>0.00</td><td>0.00</td><td>0.00</td><td>0.00</td><td>0.00</td><td>0.00</td><td>2.59</td><td>0.00</td><td>0.16</td><td>null</td><td>null</td><td></td><td>null</td><td>null</td><td></td><td>null</td><td>null</td><td></td><td>null</td><td>null</td><td></td><td>-1.13E+01</td><td>1.23E-04</td><td>Down</td><td>-3.99E+00</td><td>5.82E-04</td><td>Down</td><td>Unigene112247_All//gi|260516678|gb|ACX43965.1|//4.00E-24//cysteine protease 1 [Brachiaria hybrid cultivar]</td><td>Unigene112247_All//1.00E-23//AT5G43060| cysteine proteinase, putative / thiol protease, putative</td><td></td><td></td><td></td><td>GO:0004175//GO:0005488//GO:0006970//GO:0019538//GO:0043231</td><td>nr -</td></tr>
<tr><td>Unigene11225_All</td><td>2015</td><td>17.89</td><td>10.84</td><td>8.96</td><td>33.62</td><td>26.83</td><td>26.25</td><td>34.56</td><td>19.81</td><td>13.86</td><td>-7.23E-01</td><td>1.40E-15</td><td></td><td>-9.98E-01</td><td>2.13E-25</td><td></td><td>-3.26E-01</td><td>2.29E-08</td><td></td><td>-3.57E-01</td><td>5.84E-09</td><td></td><td>-8.03E-01</td><td>7.30E-43</td><td></td><td>-1.32E+00</td><td>1.94E-98</td><td>Down</td><td>Unigene11225_All//gi|195613770|gb|ACG28715.1|//0.00E+00//coatomer subunit gamma [Zea mays]</td><td>Unigene11225_All//0.00E+00//AT4G34450| coatomer gamma-2 subunit, putative / gamma-2 coat protein, putative / gamma-2 COP, putative</td><td>Unigene11225_All//0.00E+00//TC149588</td><td></td><td></td><td>GO:0005515//GO:0009536//GO:0015031//GO:0030120</td><td>nr -</td></tr>
<tr><td>Unigene112252_All</td><td>245</td><td>0.00</td><td>0.00</td><td>0.00</td><td>0.41</td><td>0.28</td><td>0.00</td><td>2.24</td><td>0.00</td><td>0.65</td><td>null</td><td>null</td><td></td><td>null</td><td>null</td><td></td><td>-5.33E-01</td><td>8.18E-01</td><td></td><td>-8.67E+00</td><td>4.04E-01</td><td></td><td>-1.11E+01</td><td>4.61E-04</td><td>Down</td><td>-1.79E+00</td><td>5.07E-02</td><td></td><td>Unigene112252_All//gi|159025723|emb|CAN88862.1|//6.00E-22//D5-type cyclin [Populus trichocarpa]</td><td>Unigene112252_All//6.00E-06//AT2G22490| CYCD2;1 (Cyclin D2;1); cyclin-dependent protein kinase regulator/ protein binding</td><td>Unigene112252_All//4.00E-09//ES821171</td><td></td><td></td><td>GO:0009987//GO:0043231</td><td>nr +</td></tr>
<tr><td>Unigene112253_All</td><td>245</td><td>0.00</td><td>0.00</td><td>0.00</td><td>0.00</td><td>0.00</td><td>0.00</td><td>4.49</td><td>0.17</td><td>0.33</td><td>null</td><td>null</td><td></td><td>null</td><td>null</td><td></td><td>null</td><td>null</td><td></td><td>null</td><td>null</td><td></td><td>-4.72E+00</td><td>1.02E-06</td><td>Down</td><td>-3.79E+00</td><td>3.27E-06</td><td>Down</td><td>Unigene112253_All//gi|116059767|emb|CAL55474.1|//6.00E-32//ribosomal protein L18 (ISS) [Ostreococcus tauri]</td><td>Unigene112253_All//4.00E-28//AT3G05590| RPL18 (RIBOSOMAL PROTEIN L18); structural constituent of ribosome</td><td></td><td></td><td>ko03010|Ribosome</td><td>GO:0005198//GO:0010467//GO:0030529</td><td>nr +</td></tr>
<tr><td>Unigene112254_All</td><td>245</td><td>0.00</td><td>0.00</td><td>0.00</td><td>0.81</td><td>2.53</td><td>0.74</td><td>23.30</td><td>1.19</td><td>0.49</td><td>null</td><td>null</td><td></td><td>null</td><td>null</td><td></td><td>1.64E+00</td><td>1.06E-01</td><td></td><td>-1.41E-01</td><td>9.35E-01</td><td></td><td>-4.29E+00</td><td>2.81E-31</td><td>Down</td><td>-5.58E+00</td><td>3.08E-37</td><td>Down</td><td>Unigene112254_All//gi|22327455|ref|NP_198720.2|//1.00E-32//ATTAP2; ATPase, coupled to transmembrane movement of substances / transporter [Arabidopsis thaliana] >gi|122246555|sp|Q0WML0.1|AB27B_ARATH RecName: Full=ABC transporter B family member 27; Short=ABC transporter ABCB.27; Short=AtABCB27; AltName: Full=Antigen peptide transporter-like 2; AltName: Full=Transporter associated with antigen processing-like protein 2; Short=AtTAP2; AltName: Full=Aluminum tolerance-related ATP-binding cassette transporter >gi|109690059|gb|ABG37923.1| aluminum tolerance-related ABC transporter [Arabidopsis thaliana] >gi|110739463|dbj|BAF01641.1| transporter associated with antigen processing-like protein [Arabidopsis thaliana]</td><td>Unigene112254_All//5.00E-34//AT5G39040| ATTAP2; ATPase, coupled to transmembrane movement of substances / transporter</td><td>Unigene112254_All//2.00E-45//TC135398</td><td></td><td>ko02010|ABC transporters</td><td>GO:0006810//GO:0031224//GO:0032559//GO:0042626//GO:0043234</td><td>nr +</td></tr>
<tr><td>Unigene11226_All</td><td>3116</td><td>10.36</td><td>5.46</td><td>6.19</td><td>19.90</td><td>14.81</td><td>15.15</td><td>24.48</td><td>12.80</td><td>11.28</td><td>-9.23E-01</td><td>6.23E-21</td><td></td><td>-7.44E-01</td><td>4.38E-14</td><td></td><td>-4.27E-01</td><td>1.86E-12</td><td></td><td>-3.94E-01</td><td>8.69E-10</td><td></td><td>-9.36E-01</td><td>1.45E-60</td><td></td><td>-1.12E+00</td><td>3.24E-83</td><td>Down</td><td>Unigene11226_All//gi|146286094|sp|Q0WW26.2|COPG_ARATH//0.00E+00//RecName: Full=Coatomer subunit gamma; AltName: Full=Gamma-coat protein; Short=Gamma-COP >gi|222424705|dbj|BAH20306.1| AT4G34450 [Arabidopsis thaliana]</td><td>Unigene11226_All//0.00E+00//AT4G34450| coatomer gamma-2 subunit, putative / gamma-2 coat protein, putative / gamma-2 COP, putative</td><td>Unigene11226_All//0.00E+00//TC166622</td><td></td><td></td><td>GO:0005515//GO:0009536//GO:0015031//GO:0030120</td><td>nr +</td></tr>
<tr><td>Unigene112263_All</td><td>246</td><td>0.00</td><td>0.00</td><td>0.00</td><td>0.00</td><td>0.00</td><td>0.00</td><td>5.84</td><td>1.53</td><td>0.81</td><td>null</td><td>null</td><td></td><td>null</td><td>null</td><td></td><td>null</td><td>null</td><td></td><td>null</td><td>null</td><td></td><td>-1.93E+00</td><td>3.78E-04</td><td>Down</td><td>-2.85E+00</td><td>2.40E-06</td><td>Down</td><td></td><td></td><td></td><td></td><td></td><td></td><td>ESTscan -</td></tr>
<tr><td>Unigene112270_All</td><td>247</td><td>0.00</td><td>0.00</td><td>0.00</td><td>0.00</td><td>0.00</td><td>0.00</td><td>2.05</td><td>0.00</td><td>0.00</td><td>null</td><td>null</td><td></td><td>null</td><td>null</td><td></td><td>null</td><td>null</td><td></td><td>null</td><td>null</td><td></td><td>-1.10E+01</td><td>8.90E-04</td><td>Down</td><td>-1.10E+01</td><td>5.55E-04</td><td>Down</td><td>Unigene112270_All//gi|219124680|ref|XP_002182626.1|//2.00E-13//14-3-3-like protein [Phaeodactylum tricornutum CCAP 1055/1] >gi|217405972|gb|EEC45913.1| 14-3-3-like protein [Phaeodactylum tricornutum CCAP 1055/1]</td><td>Unigene112270_All//3.00E-13//AT2G42590| GRF9 (GENERAL REGULATORY FACTOR 9); calcium ion binding / protein binding / protein phosphorylated amino acid binding</td><td></td><td></td><td></td><td>GO:0005515//GO:0009532//GO:0016020//GO:0046872</td><td>nr -</td></tr>
<tr><td>Unigene112272_All</td><td>247</td><td>0.00</td><td>0.00</td><td>0.00</td><td>0.20</td><td>0.14</td><td>0.37</td><td>2.05</td><td>0.17</td><td>0.00</td><td>null</td><td>null</td><td></td><td>null</td><td>null</td><td></td><td>-5.33E-01</td><td>8.27E-01</td><td></td><td>8.58E-01</td><td>7.96E-01</td><td></td><td>-3.60E+00</td><td>5.77E-03</td><td></td><td>-1.10E+01</td><td>5.55E-04</td><td>Down</td><td>Unigene112272_All//gi|224082258|ref|XP_002306621.1|//9.00E-31//GRAS family transcription factor [Populus trichocarpa] >gi|222856070|gb|EEE93617.1| GRAS family transcription factor [Populus trichocarpa]</td><td>Unigene112272_All//5.00E-11//AT4G08250| scarecrow transcription factor family protein</td><td></td><td>GRAS</td><td></td><td>GO:0005634//GO:0010468</td><td>nr +</td></tr>
<tr><td>Unigene11228_All</td><td>742</td><td>12.13</td><td>8.60</td><td>9.26</td><td>26.65</td><td>26.45</td><td>26.23</td><td>17.78</td><td>13.01</td><td>6.82</td><td>-4.95E-01</td><td>1.02E-02</td><td></td><td>-3.90E-01</td><td>5.73E-02</td><td></td><td>-1.08E-02</td><td>9.51E-01</td><td></td><td>-2.29E-02</td><td>8.91E-01</td><td></td><td>-4.50E-01</td><td>1.16E-03</td><td></td><td>-1.38E+00</td><td>9.26E-21</td><td>Down</td><td>Unigene11228_All//gi|240255711|ref|NP_192030.4|//1.00E-70//transferase, transferring glycosyl groups [Arabidopsis thaliana]</td><td>Unigene11228_All//2.00E-63//AT4G01210| transferase, transferring glycosyl groups</td><td>Unigene11228_All//0.00E+00//TC156636</td><td></td><td></td><td>GO:0003824//GO:0008152</td><td>nr -</td></tr>
<tr><td>Unigene112280_All</td><td>247</td><td>0.00</td><td>0.00</td><td>0.00</td><td>0.00</td><td>0.00</td><td>0.00</td><td>2.23</td><td>0.00</td><td>0.00</td><td>null</td><td>null</td><td></td><td>null</td><td>null</td><td></td><td>null</td><td>null</td><td></td><td>null</td><td>null</td><td></td><td>-1.11E+01</td><td>4.61E-04</td><td>Down</td><td>-1.11E+01</td><td>2.81E-04</td><td>Down</td><td>Unigene112280_All//gi|6831610|sp|O48513.1|RL13_CHLSW//2.00E-21//RecName: Full=60S ribosomal protein L13; AltName: Full=BBC1 protein homolog >gi|2662188|dbj|BAA23724.1| BBC1 protein [Chlamydomonas sp. W80]</td><td>Unigene112280_All//5.00E-22//AT3G49010| ATBBC1 (ARABIDOPSIS THALIANA BREAST BASIC CONSERVED 1); structural constituent of ribosome</td><td></td><td></td><td>ko03010|Ribosome</td><td>GO:0005198//GO:0010467//GO:0015934//GO:0022613</td><td>nr +</td></tr>
<tr><td>Unigene112283_All</td><td>248</td><td>0.00</td><td>0.00</td><td>0.00</td><td>0.00</td><td>0.00</td><td>0.00</td><td>2.73</td><td>0.34</td><td>0.00</td><td>null</td><td>null</td><td></td><td>null</td><td>null</td><td></td><td>null</td><td>null</td><td></td><td>null</td><td>null</td><td></td><td>-3.02E+00</td><td>2.43E-03</td><td></td><td>-1.14E+01</td><td>3.57E-05</td><td>Down</td><td>Unigene112283_All//gi|6525111|gb|AAF15333.1|AF181949_1//2.00E-15//cytochrome oxidase subunit 2 [Lespedeza formosa]</td><td>Unigene112283_All//2.00E-14//ATMG00160| cytochrome c oxidase subunit 2</td><td></td><td></td><td>ko00190|Oxidative phosphorylation</td><td>GO:0005506//GO:0015002//GO:0019866//GO:0022900//GO:0031224//GO:0043231//GO:0051234</td><td>nr -</td></tr>
<tr><td>Unigene112285_All</td><td>248</td><td>0.00</td><td>0.00</td><td>0.00</td><td>0.00</td><td>0.00</td><td>0.00</td><td>3.07</td><td>1.85</td><td>0.00</td><td>null</td><td>null</td><td></td><td>null</td><td>null</td><td></td><td>null</td><td>null</td><td></td><td>null</td><td>null</td><td></td><td>-7.27E-01</td><td>3.32E-01</td><td></td><td>-1.16E+01</td><td>8.88E-06</td><td>Down</td><td>Unigene112285_All//gi|298712420|emb|CBJ33198.1|//2.00E-17//histone H4 [Ectocarpus siliculosus]</td><td>Unigene112285_All//3.00E-08//AT5G59970| histone H4</td><td>Unigene112285_All//2.00E-23//BQ408984</td><td></td><td></td><td>GO:0000785//GO:0003676//GO:0034728//GO:0043231</td><td>nr +</td></tr>
<tr><td>Unigene112286_All</td><td>248</td><td>0.00</td><td>0.00</td><td>0.21</td><td>0.40</td><td>0.14</td><td>0.36</td><td>4.43</td><td>0.84</td><td>1.77</td><td>null</td><td>null</td><td></td><td>7.75E+00</td><td>6.40E-01</td><td></td><td>-1.53E+00</td><td>5.88E-01</td><td></td><td>-1.41E-01</td><td>9.52E-01</td><td></td><td>-2.40E+00</td><td>4.11E-04</td><td>Down</td><td>-1.33E+00</td><td>2.07E-02</td><td></td><td></td><td></td><td></td><td></td><td></td><td></td><td></td></tr>
</table>

Unigene11229_All	3570	1.38	0.79	1.48	2.53	2.00	1.70	2.87	1.44	0.79	-7.94E-01	3.37E-03		1.00E-01	7.16E-01		-3.39E-01	7.96E-02		-5.75E-01	2.35E-03		-9.93E-01	1.16E-09		-1.86E+00	1.78E-24	Down	Unigene11229_All//gi|240255711|ref|NP_192030.4|//0.00E+00//transferase, transferring glycosyl groups [Arabidopsis thaliana]	Unigene11229_All//0.00E+00//AT4G01210| transferase, transferring glycosyl groups	Unigene11229_All//0.00E+00//gi|73943845|gb|DT527153.1|DT527153				nr -
Unigene1123_All	1141	5.18	2.93	3.50	7.03	4.98	3.88	18.12	14.03	8.03	-8.22E-01	7.50E-04		-5.66E-01	2.68E-02		-4.97E-01	1.10E-02		-8.58E-01	1.95E-05		-3.69E-01	6.74E-04		-1.17E+00	5.04E-25	Down	Unigene1123_All//gi|62321656|dbj|BAD95276.1|//1.00E-147//DNA ligase like protein [Arabidopsis thaliana]	Unigene1123_All//8.00E-133//AT1G08130| ATLIG1 (ARABIDOPSIS THALIANA DNA LIGASE 1); ATP binding / DNA binding / DNA ligase (ATP)	Unigene1123_All//0.00E+00//TC162781		ko03420|Nucleotide excision repair//ko03410|Base excision repair//ko03030|DNA replication//ko03430|Mismatch repair		nr +
Unigene112300_All	249	1.05	0.20	2.14	0.80	0.41	0.00	2.21	0.00	0.64	-2.37E+00	2.14E-01		1.03E+00	3.42E-01		-9.48E-01	5.88E-01		-9.64E+00	1.34E-01		-1.11E+01	4.61E-04	Down	-1.79E+00	5.07E-02				Unigene112300_All//2.00E-82//CO110844				
Unigene112301_All	249	0.00	0.00	0.00	0.00	0.00	0.00	2.38	0.00	0.00	null	null		null	null		null	null		null	null		-1.12E+01	2.39E-04	Down	-1.12E+01	1.41E-04	Down	Unigene112301_All//gi|195623470|gb|ACG33565.1|//1.00E-28//40S ribosomal protein S24 [Zea mays]	Unigene112301_All//2.00E-26//AT3G04920| 40S ribosomal protein S24 (RPS24A)			ko03010|Ribosome	GO:0005198//GO:0005488//GO:0010467//GO:0030529	nr -
Unigene112302_All	250	0.00	0.00	0.21	0.00	0.00	0.36	3.04	0.17	0.00	null	null		7.73E+00	6.40E-01		null	null		8.50E+00	4.80E-01		-4.19E+00	1.58E-04	Down	-1.16E+01	8.88E-06	Down							
Unigene112304_All	250	59.23	31.21	24.71	99.83	75.62	51.31	44.99	22.74	40.95	-9.24E-01	4.83E-10		-1.26E+00	2.00E-15	Down	-4.01E-01	7.92E-05		-9.60E-01	1.14E-18		-9.84E-01	2.11E-10		-1.36E-01	4.10E-01		Unigene112304_All//gi|15235668|ref|NP_193070.1|//1.00E-13//leucine-rich repeat family protein / extensin family protein [Arabidopsis thaliana] >gi|75337952|sp|Q9T0K5.1|LRX3_ARATH RecName: Full=Leucine-rich repeat extensin-like protein 3; Short=LRR/EXTENSIN3; Short=AtLRX3; AltName: Full=Cell wall hydroxyproline-rich glycoprotein; Flags: Precursor >gi|4584539|emb|CAB40769.1| extensin-like protein [Arabidopsis thaliana] >gi|7268037|emb|CAB78376.1| extensin-like protein [Arabidopsis thaliana]		Unigene112304_All//4.00E-99//BQ407414			GO:0005198//GO:0005488//GO:0005618//GO:0016491//GO:0031410	nr +
Unigene112305_All	250	0.00	0.00	0.00	0.00	0.00	0.00	3.21	0.00	0.16	null	null		null	null		null	null		null	null		-1.16E+01	8.58E-06	Down	-4.33E+00	4.54E-05	Down							ESTscan -
Unigene112309_All	250	0.00	0.00	0.00	0.00	0.00	0.00	57.16	1.34	0.96	null	null		null	null		null	null		null	null		-5.42E+00	1.98E-88	Down	-5.90E+00	8.40E-95	Down	Unigene112309_All//gi|224084948|ref|XP_002307453.1|//5.00E-26//nramp transporter [Populus trichocarpa] >gi|222856902|gb|EEE94449.1| nramp transporter [Populus trichocarpa]	Unigene112309_All//5.00E-18//AT1G80830| NRAMP1 (NATURAL RESISTANCE-ASSOCIATED MACROPHAGE PROTEIN 1); inorganic anion transmembrane transporter/ manganese ion transmembrane transporter/ metal ion transmembrane transporter	Unigene112309_All//6.00E-33//DN804497			GO:0044464//GO:0051234	nr +
Unigene112310_All	250	0.00	0.00	0.00	0.00	0.00	0.00	2.03	0.50	0.00	null	null		null	null		null	null		null	null		-2.02E+00	5.21E-02		-1.10E+01	5.55E-04	Down							ESTscan +
Unigene112311_All	250	0.00	0.00	0.00	0.40	0.41	0.18	4.23	0.67	0.96	null	null		null	null		5.23E-02	9.68E-01		-1.14E+00	6.78E-01		-2.66E+00	2.25E-04	Down	-2.14E+00	9.36E-04	Down			Unigene112311_All//3.00E-07//TC173050				
Unigene112312_All	250	0.63	0.61	0.85	1.00	0.69	0.72	2.71	0.00	0.96	-4.62E-02	9.89E-01		4.41E-01	7.76E-01		-5.33E-01	7.13E-01		-4.63E-01	7.45E-01		-1.14E+01	6.34E-05	Down	-1.50E+00	5.44E-02								
Unigene112314_All	250	0.00	0.00	0.00	0.00	0.00	0.00	3.21	0.00	0.00	null	null		null	null		null	null		null	null		-1.16E+01	8.58E-06	Down	-1.16E+01	4.43E-06	Down	Unigene112314_All//gi|115456397|ref|NP_001051799.1|//2.00E-22//Os03g0832200 [Oryza sativa Japonica Group] >gi|113550270|dbj|BAF13713.1| Os03g0832200 [Oryza sativa Japonica Group]	Unigene112314_All//8.00E-24//AT1G56340| CRT1 (CALRETICULIN 1); calcium ion binding / unfolded protein binding			ko04141|Protein processing in endoplasmic reticulum//ko04145|Phagosome	GO:0005515//GO:0030246//GO:0031410//GO:0033554//GO:0042742//GO:0044267//GO:0044432//GO:0046872	nr +
Unigene112316_All	250	0.00	0.00	0.00	0.00	0.00	0.00	2.88	1.50	0.00	null	null		null	null		null	null		null	null		-9.34E-01	2.26E-01		-1.15E+01	1.78E-05	Down							
Unigene112319_All	250	0.00	0.00	0.00	0.00	0.00	0.00	2.03	0.33	0.00	null	null		null	null		null	null		null	null		-2.60E+00	2.05E-02		-1.10E+01	5.55E-04	Down	Unigene112319_All//gi|257815167|gb|ACV69975.1|//2.00E-20//14-3-3 protein [Solanum chacoense]	Unigene112319_All//1.00E-20//AT5G65430| GRF8 (GENERAL REGULATORY FACTOR 8); protein binding / protein phosphorylated amino acid binding					nr +
Unigene112322_All	251	0.00	0.00	0.00	0.00	0.00	0.00	2.02	0.00	0.00	null	null		null	null		null	null		null	null		-1.10E+01	8.90E-04	Down	-1.10E+01	5.55E-04	Down							ESTscan -
Unigene112324_All	251	0.00	0.00	0.00	0.00	0.00	0.00	9.26	9.82	0.95	null	null		null	null		null	null		null	null		8.47E-02	8.26E-01		-3.28E+00	3.91E-11	Down			Unigene112324_All//2.00E-08//TC142106				
Unigene112326_All	251	61.49	47.24	39.25	43.26	42.12	36.53	42.95	26.98	19.52	-3.81E-01	8.02E-03		-6.48E-01	1.02E-05		-3.88E-02	8.38E-01		-2.44E-01	1.98E-01		-6.71E-01	1.39E-05		-1.14E+00	7.43E-13	Down	Unigene112326_All//gi|238837063|gb|ACR61637.1|//3.00E-41//heteromeric acetyl-CoA biotin carboxylase [Jatropha curcas]	Unigene112326_All//2.00E-41//AT5G35360| CAC2; acetyl-CoA carboxylase/ biotin carboxylase	Unigene112326_All//3.00E-130//gi|164367339|gb|ES801692.1|ES801692		ko00280|Valine, leucine and isoleucine degradation	GO:0006631//GO:0009526//GO:0009532//GO:0016421//GO:0016879//GO:0019842//GO:0032559//GO:0043169	nr -
Unigene112327_All	251	0.63	0.00	0.00	0.99	0.00	0.54	2.02	0.00	0.16	-9.29E+00	2.16E-01		-9.29E+00	2.46E-01		-9.95E+00	4.19E-02		-8.78E-01	6.17E-01		-1.10E+01	8.90E-04	Down	-3.67E+00	3.75E-03		Unigene112327_All//gi|34365551|gb|AAQ65087.1|//1.00E-29//At4g14850 [Arabidopsis thaliana]	Unigene112327_All//9.00E-23//AT4G14850| LOI1 (lovastatin insensitive 1)				GO:0006066//GO:0006084//GO:0006090//GO:0019222//GO:0043231//GO:0048513//GO:0070717	nr +
Unigene112328_All	251	0.00	0.00	0.00	0.00	0.55	0.00	2.19	0.50	16.98	null	null		null	null		9.10E+00	3.20E-01		null	null		-2.13E+00	3.31E-02		2.96E+00	1.08E-17	Up			Unigene112328_All//1.00E-105//FL577603				
Unigene112336_All	252	0.00	0.00	0.00	0.00	0.00	0.00	2.35	0.17	0.00	null	null		null	null		null	null		null	null		-3.82E+00	1.79E-03		-1.12E+01	1.41E-04	Down	Unigene112336_All//gi|301122571|ref|XP_002909012.1|//2.00E-06//rho GDP-dissociation inhibitor [Phytophthora infestans T30-4] >gi|262099774|gb|EEY57826.1| rho GDP-dissociation inhibitor [Phytophthora infestans T30-4]	Unigene112336_All//4.00E-05//AT1G12070| Rho GDP-dissociation inhibitor family protein					nr -
Unigene112337_All	252	0.00	0.00	0.00	0.00	0.00	0.00	2.18	0.33	0.00	null	null		null	null		null	null		null	null		-2.72E+00	1.22E-02		-1.11E+01	2.81E-04	Down							
Unigene112339_All	252	0.00	0.00	0.00	0.00	0.00	0.00	4.19	0.50	0.16	null	null		null	null		null	null		null	null		-3.08E+00	6.12E-05	Down	-4.73E+00	8.85E-07	Down	Unigene112339_All//gi|30683788|ref|NP_850114.1|//1.00E-13//FRU (FER-LIKE REGULATOR OF IRON UPTAKE); DNA binding / transcription factor [Arabidopsis thaliana] >gi|122242304|sp|Q0V7X4.1|FIT_ARATH RecName: Full=Transcription factor FER-LIKE IRON DEFICIENCY-INDUCED TRANSCRIPTION FACTOR; AltName: Full=Transcription factor EN 43; AltName: Full=Transcription factor Fe-DEFICIENCY INDUCED TRANSCRIPTION FACTOR 1; AltName: Full=FER-LIKE REGULATOR OF IRON UPTAKE; AltName: Full=bHLH transcription factor bHLH029; AltName: Full=Basic helix-loop-helix protein 29; Short=bHLH 29; Short=AtbHLH29 >gi|111074360|gb|ABH04553.1| At2g28160 [Arabidopsis thaliana]	Unigene112339_All//1.00E-05//AT2G28160| FRU (FER-LIKE REGULATOR OF IRON UPTAKE); DNA binding / transcription factor	Unigene112339_All//4.00E-34//CA994022	bHLH		GO:0005515//GO:0006826//GO:0010038//GO:0010468//GO:0043231	nr -
Unigene11234_All	1174	9.63	17.44	11.20	24.06	28.04	36.74	14.41	16.45	35.22	8.57E-01	5.20E-12		2.19E-01	2.13E-01		2.21E-01	1.99E-02		6.11E-01	9.98E-15		1.91E-01	1.19E-01		1.29E+00	2.15E-56	Up	Unigene11234_All//gi|297807481|ref|XP_002871624.1|//3.00E-44//ATP binding protein [Arabidopsis lyrata subsp. lyrata] >gi|297317461|gb|EFH47883.1| ATP binding protein [Arabidopsis lyrata subsp. lyrata]	Unigene11234_All//3.00E-44//AT5G14610| ATP binding / ATP-dependent helicase/ helicase/ nucleic acid binding / protein binding	Unigene11234_All//0.00E+00//TC179191		ko03040|Spliceosome	GO:0000166//GO:0016887	nr +
Unigene112347_All	253	0.00	0.00	0.00	0.00	0.00	0.00	2.34	0.00	0.00	null	null		null	null		null	null		null	null		-1.12E+01	2.39E-04	Down	-1.12E+01	1.41E-04	Down	Unigene112347_All//gi|99903571|gb|ABF68589.1|//5.00E-11//chloroplast sedoheptulose-1,7-bisphosphatase [Prymnesium parvum]	Unigene112347_All//5.00E-08//AT3G55800| SBPASE (sedoheptulose-bisphosphatase); phosphoric ester hydrolase/ sedoheptulose-bisphosphatase			ko00710|Carbon fixation in photosynthetic organisms	GO:0016787	nr +
Unigene112350_All	253	0.00	0.60	0.00	0.39	0.14	0.18	2.01	0.00	0.79	9.23E+00	2.36E-01		null	null		-1.53E+00	5.88E-01		-1.14E+00	6.78E-01		-1.10E+01	8.90E-04	Down	-1.35E+00	1.40E-01				Unigene112350_All//7.00E-110//EE592576				
Unigene11236_All	3977	1.63	1.58	1.22	12.58	24.19	12.63	11.12	3.07	10.42	-4.63E-02	8.47E-01		-4.21E-01	8.73E-02		9.44E-01	6.87E-75		6.00E-03	9.57E-01		-1.86E+00	3.30E-100	Down	-9.41E-02	2.37E-01		Unigene11236_All//gi|224140965|ref|XP_002323847.1|//0.00E+00//multidrug/pheromone exporter, MDR family, ABC transporter family [Populus trichocarpa] >gi|222866849|gb|EEF03980.1| multidrug/pheromone exporter, MDR family, ABC transporter family [Populus trichocarpa]	Unigene11236_All//0.00E+00//AT3G28860| ABCB19; ATPase, coupled to transmembrane movement of substances / auxin efflux transmembrane transporter	Unigene11236_All//0.00E+00//TC144381		ko02010|ABC transporters	GO:0009630//GO:0009639//GO:0009725//GO:0009926//GO:0015562//GO:0031224//GO:0031410//GO:0032535//GO:0032559//GO:0042626//GO:0043234//GO:0043479//GO:0048437//GO:0048528	nr +
Unigene112360_All	254	0.00	0.00	0.00	0.00	0.00	0.00	2.33	0.33	0.00	null	null		null	null		null	null		null	null		-2.82E+00	7.18E-03		-1.12E+01	1.41E-04	Down	Unigene112360_All//gi|119638443|gb|ABL85034.1|//4.00E-09//ribosomal subunit 8E protein [Brachypodium sylvaticum]	Unigene112360_All//2.00E-06//AT5G20290| 40S ribosomal protein S8 (RPS8A)			ko03010|Ribosome	GO:0044424	nr +
Unigene112365_All	254	0.00	0.00	0.00	0.00	0.00	0.00	4.49	0.00	0.00	null	null		null	null		null	null		null	null		-1.21E+01	3.89E-08	Down	-1.21E+01	1.65E-08	Down							
Unigene11237_All	4421	1.60	0.73	0.73	9.15	11.18	6.62	19.27	5.00	17.97	-1.12E+00	7.12E-07	Down	-1.12E+00	1.70E-06	Down	2.89E-01	4.95E-05		-4.67E-01	8.21E-09		-1.95E+00	5.79E-205	Down	-1.01E-01	5.89E-02		Unigene11237_All//gi|162280537|gb|ABX82929.1|//0.00E+00//LO4 [Solanum pennellii]	Unigene11237_All//0.00E+00//AT3G28860| ABCB19; ATPase, coupled to transmembrane movement of substances / auxin efflux transmembrane transporter	Unigene11237_All//0.00E+00//TC150081		ko02010|ABC transporters	GO:0009630//GO:0009639//GO:0009725//GO:0009926//GO:0015562//GO:0031224//GO:0031410//GO:0032535//GO:0032559//GO:0042626//GO:0043234//GO:0043479//GO:0048437//GO:0048528	nr +
Unigene112374_All	255	0.00	0.00	0.00	0.00	0.00	0.00	2.65	0.49	0.00	null	null		null	null		null	null		null	null		-2.43E+00	7.77E-03		-1.14E+01	3.56E-05	Down							
Unigene11238_All	568	2.12	2.32	1.69	3.86	2.55	4.45	5.73	2.35	4.49	1.31E-01	8.13E-01		-3.28E-01	6.27E-01		-6.00E-01	1.60E-01		2.07E-01	6.41E-01		-1.28E+00	4.79E-05	Down	-3.53E-01	2.51E-01		Unigene11238_All//gi|27923772|sp|Q9C641.1|EFGM_ARATH//7.00E-68//RecName: Full=Elongation factor G, mitochondrial; Short=EF-Gmt; AltName: Full=Elongation factor G 1, mitochondrial; Short=mEF-G 1; AltName: Full=Elongation factor G1; Flags: Precursor	Unigene11238_All//1.00E-69//AT2G45030| mitochondrial elongation factor, putative	Unigene11238_All//0.00E+00//ES825080			GO:0006412//GO:0008135//GO:0010038//GO:0017111//GO:0032559//GO:0032561//GO:0043231	nr -
Unigene112382_All	255	0.00	0.00	0.00	0.00	0.00	0.00	2.32	0.33	0.00	null	null		null	null		null	null		null	null		-2.82E+00	7.17E-03		-1.12E+01	1.41E-04	Down	Unigene112382_All//gi|109676342|gb|ABG37653.1|//5.00E-14//integrase [Populus trichocarpa]					GO:0003676	nr +
Unigene112385_All	256	0.00	0.00	0.00	0.00	0.00	0.00	2.48	1.31	0.16	null	null		null	null		null	null		null	null		-9.24E-01	2.63E-01		-3.99E+00	5.82E-04	Down	Unigene112385_All//gi|18404062|ref|NP_565836.1|//7.00E-39//ubiquitin extension protein 2 (UBQ2) / 60S ribosomal protein L40 (RPL40A) [Arabidopsis thaliana] >gi|18409639|ref|NP_566969.1| UBQ1 (UBIQUITIN EXTENSION PROTEIN 1); protein binding / structural constituent of ribosome [Arabidopsis thaliana] >gi|166930|gb|AAA32904.1| ubiquitin extension protein (UBQ1) [Arabidopsis thaliana] >gi|166932|gb|AAA32905.1| ubiquitin extension protein (UBQ2) [Arabidopsis thaliana] >gi|4886268|emb|CAB43405.1| ubiquitin / ribosomal protein CEP52 [Arabidopsis thaliana] >gi|20198116|gb|AAM15407.1| ubiquitin extension protein (UBQ2) [Arabidopsis thaliana] >gi|21553955|gb|AAM63036.1| ubiquitin extension protein UBQ1 [Arabidopsis thaliana] >gi|222424333|dbj|BAH20123.1| AT2G36170 [Arabidopsis thaliana]	Unigene112385_All//3.00E-40//AT3G52590| UBQ1 (UBIQUITIN EXTENSION PROTEIN 1); protein binding / structural constituent of ribosome	Unigene112385_All//3.00E-10//TC154845		ko03010|Ribosome	GO:0003006//GO:0005198//GO:0005488//GO:0010467//GO:0015934//GO:0031981//GO:0032446	nr -
Unigene112386_All	256	0.00	0.00	0.00	0.00	0.00	0.00	4.29	0.00	0.16	null	null		null	null		null	null		null	null		-1.21E+01	7.67E-08	Down	-4.79E+00	4.55E-07	Down							

Unigene112391_All	256	0.41	1.98	0.83	0.19	0.00	0.00	10.40	4.57	1.40	2.28E+00	6.23E-02		1.03E+00	5.92E-01		-7.60E+00	5.61E-01		-7.60E+00	6.42E-01		-1.19E+00	7.13E-04	Down	-2.89E+00	2.70E-11	Down			Unigene112391_All//3.00E-10//FL576427				
Unigene112392_All	257	0.00	0.00	0.00	0.00	0.00	0.00	2.30	1.46	0.00	null	null		null	null		null	null		null	null		-6.54E-01	4.45E-01		-1.12E+01	1.41E-04	Down							ESTscan -
Unigene112394_All	257	0.00	0.00	0.00	0.00	0.00	0.00	16.95	0.81	9.15	null	null		null	null		null	null		null	null		-4.38E+00	2.65E-24	Down	-8.90E-01	4.13E-04		Unigene112394_All//gi|297746450|emb|CBI16506.3|//8.00E-06//unnamed protein product [Vitis vinifera]	Unigene112394_All//4.00E-06//AT1G55290| oxidoreductase, 2OG-Fe(II) oxygenase family protein					nr +
Unigene112396_All	257	0.00	0.00	0.00	0.00	0.00	0.00	2.30	0.16	0.00	null	null		null	null		null	null		null	null		-3.82E+00	1.79E-03		-1.12E+01	1.41E-04	Down	Unigene112396_All//gi|301122571|ref|XP_002909012.1|//7.00E-10//rho GDP-dissociation inhibitor [Phytophthora infestans T30-4] >gi|262099774|gb|EEY57826.1| rho GDP-dissociation inhibitor [Phytophthora infestans T30-4]	Unigene112396_All//8.00E-09//AT3G07880| Rho GDP-dissociation inhibitor family protein				GO:0005488//GO:0005737//GO:0007265//GO:0008104//GO:0010769//GO:0031344//GO:0044459	nr -
Unigene112399_All	258	0.00	0.00	0.00	0.00	0.00	0.00	1.97	0.49	0.00	null	null		null	null		null	null		null	null		-2.02E+00	5.21E-02		-1.09E+01	5.55E-04	Down	Unigene112399_All//gi|8778393|gb|AAF79401.1|AC068197_11//5.00E-11//F16A14.17 [Arabidopsis thaliana]	Unigene112399_All//2.00E-12//AT1G13950| ELF5A-1 (EUKARYOTIC ELONGATION FACTOR 5A-1); translation initiation factor				GO:0006417//GO:0008135//GO:0010087	nr +
Unigene112401_All	258	0.41	0.79	0.00	0.00	0.80	0.00	4.26	0.81	0.77	9.54E-01	6.22E-01		-8.66E+00	3.97E-01		9.65E+00	1.57E-01		null	null		-2.40E+00	4.11E-04	Down	-2.46E+00	2.04E-04	Down			Unigene112401_All//2.00E-20//TC163295				
Unigene112402_All	258	0.00	0.00	0.00	0.00	0.00	0.00	3.61	1.13	0.31	null	null		null	null		null	null		null	null		-1.67E+00	1.45E-02		-3.55E+00	3.97E-05	Down	Unigene112402_All//gi|300254105|gb|EFJ39080.1|//2.00E-14//hypothetical protein VOLCADRAFT_85069 [Volvox carteri f. nagariensis]		Unigene112402_All//1.00E-18//gi|109830702|gb|DW229261.1|DW229261				nr +
Unigene112405_All	258	0.00	0.00	0.00	0.00	0.00	0.00	2.29	0.32	0.00	null	null		null	null		null	null		null	null		-2.82E+00	7.17E-03		-1.12E+01	1.41E-04	Down	Unigene112405_All//gi|297794223|ref|XP_002864996.1|//3.00E-22//60S ribosomal protein L26 [Arabidopsis lyrata subsp. lyrata] >gi|297310831|gb|EFH41255.1| 60S ribosomal protein L26 [Arabidopsis lyrata subsp. lyrata]	Unigene112405_All//2.00E-22//AT5G67510| 60S ribosomal protein L26 (RPL26B)			ko03010|Ribosome	GO:0005198//GO:0005488//GO:0010467//GO:0015934//GO:0043231	nr +
Unigene112407_All	259	0.00	0.00	0.00	0.00	0.00	0.00	3.43	0.00	0.00	null	null		null	null		null	null		null	null		-1.17E+01	2.24E-06	Down	-1.17E+01	1.10E-06	Down							
Unigene112408_All	259	0.00	0.00	0.00	1.54	0.27	0.52	2.78	0.32	0.00	null	null		null	null		-2.53E+00	5.80E-02		-1.56E+00	2.31E-01		-3.10E+00	1.40E-03		-1.14E+01	1.78E-05	Down			Unigene112408_All//5.00E-123//EV495313				
Unigene112409_All	259	0.00	0.00	0.00	0.00	0.00	0.00	3.26	0.32	0.31	null	null		null	null		null	null		null	null		-3.34E+00	2.51E-04	Down	-3.41E+00	1.35E-04	Down							ESTscan -
Unigene11241_All	1026	5.86	16.59	7.11	8.35	5.24	9.77	12.28	23.79	18.06	1.50E+00	1.20E-23	Up	2.78E-01	2.44E-01		-6.74E-01	2.32E-04		2.27E-01	2.65E-01		9.54E-01	1.55E-20		5.56E-01	7.03E-07		Unigene11241_All//gi|18391384|ref|NP_563905.1|//1.00E-133//KEU (keule); protein transporter [Arabidopsis thaliana] >gi|150421587|sp|Q9C5X3.2|KEULE_ARATH RecName: Full=SNARE-interacting protein KEULE	Unigene11241_All//1.00E-129//AT1G12360| KEU (keule); protein transporter	Unigene11241_All//1.00E-102//TC156564				nr -
Unigene112413_All	259	0.00	0.00	0.00	0.19	0.00	0.00	3.10	0.00	0.31	null	null		null	null		-7.59E+00	5.61E-01		-7.59E+00	6.42E-01		-1.16E+01	8.58E-06	Down	-3.33E+00	2.47E-04	Down	Unigene112413_All//gi|297596074|ref|NP_001041976.2|//6.00E-21//Os01g0139200 [Oryza sativa Japonica Group] >gi|255672859|dbj|BAF03890.2| Os01g0139200 [Oryza sativa Japonica Group]	Unigene112413_All//3.00E-20//AT3G18230| octicosapeptide/Phox/Bem1p (PB1) domain-containing protein				GO:0044444	nr -
Unigene112416_All	259	0.00	0.00	0.00	0.00	0.00	0.00	31.18	0.00	0.00	null	null		null	null		null	null		null	null		-1.49E+01	2.66E-57	Down	-1.49E+01	1.93E-59	Down	Unigene112416_All//gi|145408590|ref|YP_001152214.1|//3.00E-06//ORF124 [Pinus koraiensis] >gi|145048839|gb|ABP35456.1| ORF124 [Pinus koraiensis]		Unigene112416_All//3.00E-16//BF275966				nr +
Unigene112419_All	259	0.00	0.00	0.00	0.38	0.27	0.35	3.43	0.97	268.87	null	null		null	null		-5.33E-01	8.18E-01		-1.41E-01	9.52E-01		-1.82E+00	1.08E-02		6.29E+00	0.00E+00	Up	Unigene112419_All//gi|114145390|dbj|BAF30984.1|//2.00E-09//basic helix-loop-helix protein [Nicotiana tabacum]	Unigene112419_All//1.00E-07//AT5G43650| basic helix-loop-helix (bHLH) family protein	Unigene112419_All//2.00E-141//DT465287	bHLH			nr -
Unigene112420_All	259	0.00	0.00	0.00	0.00	0.00	0.00	5.55	0.00	0.00	null	null		null	null		null	null		null	null		-1.24E+01	3.33E-10	Down	-1.24E+01	1.19E-10	Down	Unigene112420_All//gi|223997350|ref|XP_002288348.1|//5.00E-14//RL24, ribosomal protein 24 60S large ribosomal subunit [Thalassiosira pseudonana CCMP1335] >gi|220975456|gb|EED93784.1| RL24, ribosomal protein 24 60S large ribosomal subunit [Thalassiosira pseudonana CCMP1335]	Unigene112420_All//3.00E-09//AT3G53020| STV1 (SHORT VALVE1); structural constituent of ribosome			ko03010|Ribosome	GO:0009536//GO:0009755//GO:0015934//GO:0016020//GO:0031981//GO:0048438	nr +
Unigene112427_All	260	0.00	0.00	0.00	0.00	0.00	0.00	1.95	0.00	0.00	null	null		null	null		null	null		null	null		-1.09E+01	8.90E-04	Down	-1.09E+01	5.55E-04	Down							
Unigene112431_All	260	0.00	0.00	0.00	0.00	0.00	0.17	125.54	585.30	258.34	null	null		null	null		null	null		7.44E+00	7.01E-01		2.22E+00	0.00E+00	Up	1.04E+00	5.85E-65	Up	Unigene112431_All//gi|300077149|gb|ADJ66851.1|//2.00E-35//O-methyltransferase [Vitis vinifera]	Unigene112431_All//4.00E-29//AT1G51990| O-methyltransferase family 2 protein	Unigene112431_All//4.00E-102//TC157783		ko00940|Phenylpropanoid biosynthesis//ko00944|Flavone and flavonol biosynthesis//ko00402|Benzoxazinoid biosynthesis	GO:0005515//GO:0008171	nr -
Unigene112436_All	260	0.00	0.00	0.00	0.00	0.00	0.69	3.41	0.80	0.15	null	null		null	null		null	null		9.44E+00	1.95E-01		-2.09E+00	4.71E-03		-4.48E+00	1.23E-05	Down			Unigene112436_All//3.00E-44//TC149496				
Unigene11244_All	763	8.50	4.52	8.65	14.56	11.96	10.18	7.81	2.90	5.90	-9.13E-01	8.80E-05		2.54E-02	9.31E-01		-2.84E-01	1.05E-01		-5.16E-01	2.18E-03		-1.43E+00	5.78E-10	Down	-4.05E-01	5.77E-02		Unigene11244_All//gi|297845632|ref|XP_002890697.1|//7.00E-62//At1g68060/T23K23_9 [Arabidopsis lyrata subsp. lyrata] >gi|297336539|gb|EFH66956.1| At1g68060/T23K23_9 [Arabidopsis lyrata subsp. lyrata]	Unigene11244_All//1.00E-52//AT1G24764| ATMAP70-2 (microtubule-associated proteins 70-2); microtubule binding	Unigene11244_All//0.00E+00//gi|78338135|gb|DT558409.1|DT558409			GO:0003002//GO:0006996//GO:0015631//GO:0016020//GO:0042546//GO:0055028	nr -
Unigene112442_All	261	0.20	0.97	0.82	0.19	0.53	1.73	2.43	0.96	0.15	2.28E+00	2.34E-01		2.03E+00	3.62E-01		1.47E+00	6.19E-01		3.18E+00	3.79E-02		-1.34E+00	1.13E-01		-3.99E+00	5.82E-04	Down			Unigene112442_All//1.00E-18//TC174009				
Unigene112449_All	262	0.00	0.00	0.00	0.00	0.00	0.00	2.90	2.39	0.00	null	null		null	null		null	null		null	null		-2.80E-01	6.96E-01		-1.15E+01	8.88E-06	Down							ESTscan -
Unigene11245_All	1365	6.17	3.93	4.17	15.73	10.32	7.38	13.85	4.10	5.63	-6.49E-01	1.10E-03		-5.64E-01	6.74E-03		-6.08E-01	1.92E-08		-1.09E+00	8.15E-20	Down	-1.75E+00	5.00E-40	Down	-1.30E+00	1.03E-26	Down	Unigene11245_All//gi|297849904|ref|XP_002892833.1|//1.00E-123//atmap70-4 [Arabidopsis lyrata subsp. lyrata] >gi|297338675|gb|EFH69092.1| atmap70-4 [Arabidopsis lyrata subsp. lyrata]	Unigene11245_All//7.00E-86//AT1G68060| ATMAP70-1 (microtubule-associated proteins 70-1); microtubule binding	Unigene11245_All//0.00E+00//TC135367				nr -
Unigene112454_All	263	0.00	0.00	0.00	0.00	0.00	0.00	3.70	0.32	0.61	null	null		null	null		null	null		null	null		-3.54E+00	4.32E-05	Down	-2.61E+00	3.36E-04	Down	Unigene112454_All//gi|292606435|gb|ADE34119.1|//9.00E-23//Superman-like protein FRASUP5 [Fragaria virginiana subsp. virginiana]	Unigene112454_All//9.00E-20//AT2G37740| ZFP10 (ZINC-FINGER PROTEIN 10); nucleic acid binding / transcription factor/ zinc ion binding	Unigene112454_All//3.00E-13//TC130397	C2H2		GO:0003824//GO:0008152//GO:0044464//GO:0046914	nr +
Unigene112457_All	263	0.00	0.00	0.00	0.00	0.00	0.00	2.57	0.00	0.00	null	null		null	null		null	null		null	null		-1.13E+01	6.34E-05	Down	-1.13E+01	3.56E-05	Down	Unigene112457_All//gi|59859758|gb|AAX09601.1|//5.00E-39//elongation factor 1 alpha [Mallomonas rasilis]	Unigene112457_All//3.00E-34//AT5G60390| elongation factor 1-alpha / EF-1-alpha	Unigene112457_All//1.00E-28//EV494515			GO:0006412//GO:0008135//GO:0017111//GO:0032561//GO:0044424	nr -
Unigene112458_All	263	0.00	0.00	0.00	2.84	0.39	0.17	2.09	0.16	0.15	null	null		null	null		-2.85E+00	2.09E-03		-4.05E+00	7.75E-04	Down	-3.72E+00	3.23E-03		-3.79E+00	2.03E-03								
Unigene11246_All	1565	16.75	11.56	11.57	45.10	38.44	26.72	39.23	17.12	24.18	-5.35E-01	4.31E-07		-5.34E-01	1.03E-06		-2.31E-01	8.76E-05		-7.55E-01	1.53E-34		-1.20E+00	1.22E-72	Down	-6.98E-01	1.02E-30		Unigene11246_All//gi|240254161|ref|NP_173873.5|//0.00E+00//ATMAP70-2 (microtubule-associated proteins 70-2); microtubule binding [Arabidopsis thaliana] >gi|22135818|gb|AAM91095.1| At1g68060/T23K23_9 [Arabidopsis thaliana] >gi|23463063|gb|AAN33201.1| At1g68060/T23K23_9 [Arabidopsis thaliana]	Unigene11246_All//4.00E-162//AT1G24764| ATMAP70-2 (microtubule-associated proteins 70-2); microtubule binding	Unigene11246_All//0.00E+00//TC135873			GO:0003002//GO:0006996//GO:0015631//GO:0016020//GO:0042546//GO:0043231//GO:0055028	nr -
Unigene112462_All	263	0.00	0.00	0.00	0.00	0.00	0.00	1.93	0.00	0.30	null	null		null	null		null	null		null	null		-1.09E+01	8.90E-04	Down	-2.67E+00	1.37E-02								ESTscan -
Unigene112463_All	263	0.00	0.00	0.00	0.00	0.00	0.00	3.54	0.95	0.30	null	null		null	null		null	null		null	null		-1.89E+00	6.92E-03		-3.55E+00	3.97E-05	Down	Unigene112463_All//gi|116734005|gb|ABK20083.1|//7.00E-26//elongation factor 2 [Amphiroa fragilissima]	Unigene112463_All//2.00E-23//AT1G56070| LOS1; copper ion binding / translation elongation factor/ translation factor, nucleic acid binding				GO:0000166	nr -
Unigene112468_All	264	3.57	2.11	2.42	2.08	3.13	1.20	8.81	5.70	3.47	-7.57E-01	2.86E-01		-5.60E-01	4.39E-01		5.93E-01	4.93E-01		-7.94E-01	4.44E-01		-6.28E-01	9.58E-02		-1.34E+00	3.20E-04	Down			Unigene112468_All//4.00E-108//TC172388				
Unigene112470_All	264	0.00	1.73	1.41	1.89	0.26	1.03	2.88	0.95	0.30	1.08E+01	7.44E-03		1.05E+01	2.50E-02		-2.85E+00	1.78E-02		-8.78E-01	4.20E-01		-1.60E+00	3.70E-02		-3.26E+00	4.48E-04	Down							ESTscan +
Unigene112471_All	264	0.00	0.00	0.00	0.00	0.00	0.00	2.24	0.95	0.00	null	null		null	null		null	null		null	null		-1.24E+00	1.54E-01		-1.11E+01	1.41E-04	Down	Unigene112471_All//gi|226509811|ref|NP_001147421.1|//2.00E-11//60 ribosomal protein L14 [Zea mays] >gi|195611252|gb|ACG27456.1| 60 ribosomal protein L14 [Zea mays]	Unigene112471_All//6.00E-09//AT2G20450| 60S ribosomal protein L14 (RPL14A)			ko03010|Ribosome	GO:0030529//GO:0043229//GO:0044444	nr -
Unigene112473_All	265	0.39	0.19	0.00	1.88	0.00	0.34	2.39	0.47	1.05	-1.05E+00	6.85E-01		-8.63E+00	3.97E-01		-1.09E+01	8.99E-04	Down	-2.46E+00	4.83E-02		-2.34E+00	1.27E-02		-1.19E+00	1.33E-01								
Unigene112475_All	265	0.00	0.00	0.00	0.19	0.00	0.00	4.31	1.58	0.00	null	null		null	null		-7.55E+00	5.61E-01		-7.55E+00	6.42E-01		-1.45E+00	1.40E-02		-1.21E+01	1.65E-08	Down							
Unigene112477_All	265	0.00	0.00	0.00	0.00	0.00	0.00	2.39	0.16	0.00	null	null		null	null		null	null		null	null		-3.92E+00	9.81E-04	Down	-1.12E+01	7.12E-05	Down	Unigene112477_All//gi|32401334|gb|AAP80839.1|//1.00E-15//ribosomal S29-like protein [Griffithsia japonica]	Unigene112477_All//3.00E-16//AT4G33865| 40S ribosomal protein S29 (RPS29C)			ko03010|Ribosome	GO:0005198//GO:0010467//GO:0015935	nr -
Unigene112478_All	265	0.00	0.00	0.00	0.00	0.00	0.00	2.71	0.00	0.00	null	null		null	null		null	null		null	null		-1.14E+01	3.27E-05	Down	-1.14E+01	1.78E-05	Down							
Unigene11248_All	654	0.56	2.17	1.22	0.84	1.95	2.28	4.40	3.64	11.03	1.95E+00	1.68E-03		1.12E+00	1.80E-01		1.22E+00	4.61E-02		1.44E+00	1.11E-02		-2.71E-01	4.47E-01		1.33E+00	5.65E-11	Up	Unigene11248_All//gi|224071385|ref|XP_002303434.1|//8.00E-66//jumonji domain protein [Populus trichocarpa] >gi|222840866|gb|EEE78413.1| jumonji domain protein [Populus trichocarpa]	Unigene11248_All//5.00E-44//AT5G46910| transcription factor jumonji (jmj) family protein	Unigene11248_All//0.00E+00//TC133454			GO:0009743//GO:0010468//GO:0043231	nr -
Unigene112484_All	266	0.00	0.00	0.00	0.00	0.00	0.00	2.38	0.16	0.00	null	null		null	null		null	null		null	null		-3.92E+00	9.81E-04	Down	-1.12E+01	7.12E-05	Down	Unigene112484_All//gi|297812013|ref|XP_002873890.1|//8.00E-30//40S ribosomal protein S16 [Arabidopsis lyrata subsp. lyrata] >gi|297319727|gb|EFH50149.1| 40S ribosomal protein S16 [Arabidopsis lyrata subsp. lyrata]	Unigene112484_All//1.00E-29//AT5G18380| 40S ribosomal protein S16 (RPS16C)	Unigene112484_All//8.00E-08//CD486142		ko03010|Ribosome	GO:0005198//GO:0010467//GO:0030529	nr +
Unigene112485_All	266	0.00	0.00	0.00	0.00	0.00	0.00	3.66	0.00	0.00	null	null		null	null		null	null		null	null		-1.18E+01	5.83E-07	Down	-1.18E+01	2.72E-07	Down	Unigene112485_All//gi|297812013|ref|XP_002873890.1|//3.00E-30//40S ribosomal protein S16 [Arabidopsis lyrata subsp. lyrata] >gi|297319727|gb|EFH50149.1| 40S ribosomal protein S16 [Arabidopsis lyrata subsp. lyrata]	Unigene112485_All//5.00E-30//AT5G18380| 40S ribosomal protein S16 (RPS16C)			ko03010|Ribosome	GO:0005198//GO:0010467//GO:0030529	nr +
Unigene11249_All	3651	1.62	2.97	2.42	5.83	7.55	5.83	7.85	5.38	20.57	8.75E-01	5.93E-07		5.80E-01	4.00E-03		3.75E-01	1.23E-04		1.00E-04	1.00E+00		-5.45E-01	1.47E-09		1.39E+00	6.58E-114	Up	Unigene11249_All//gi|224138208|ref|XP_002326545.1|//0.00E+00//jumonji domain protein [Populus trichocarpa] >gi|222833867|gb|EEE72344.1| jumonji domain protein [Populus trichocarpa]	Unigene11249_All//0.00E+00//AT5G46910| transcription factor jumonji (jmj) family protein	Unigene11249_All//0.00E+00//TC168243			GO:0001071//GO:0009743//GO:0010468	nr +
Unigene112495_All	268	0.39	0.38	0.99	0.00	0.26	0.67	2.05	1.09	0.00	-4.66E-02	9.90E-01		1.35E+00	4.09E-01		8.01E+00	6.14E-01		9.40E+00	1.95E-01		-9.10E-01	3.19E-01		-1.10E+01	2.81E-04	Down	Unigene112495_All//gi|15233100|ref|NP_188799.1|//1.00E-40//early-responsive to dehydration protein-related / ERD protein-related [Arabidopsis thaliana]	Unigene112495_All//5.00E-42//AT3G21620| early-responsive to dehydration protein-related / ERD protein-related	Unigene112495_All//2.00E-20//TC156729			GO:0016020//GO:0031410	nr +
Unigene112499_All	268	0.00	0.00	0.00	0.00	0.00	0.00	2.52	0.00	0.00	null	null		null	null		null	null		null	null		-1.13E+01	6.34E-05	Down	-1.13E+01	3.56E-05	Down							

Unigene11250_All	3721	2.80	3.94	2.13	5.05	4.66	3.67	18.12	24.95	92.36	4.92E-01	7.76E-04		-3.92E-01	3.76E-02		-1.14E-01	4.47E-01		-4.61E-01	2.18E-04		4.61E-01	1.29E-21		2.35E+00	0.00E+00	Up	Unigene11250_All//gi|224138208|ref|XP_002326545.1|//0.00E+00//jumonji domain protein [Populus trichocarpa] >gi|222833867|gb|EEE72344.1| jumonji domain protein [Populus trichocarpa]	Unigene11250_All//0.00E+00//AT5G46910| transcription factor jumonji (jmj) family protein	Unigene11250_All//0.00E+00//TC179033			GO:0001071//GO:0009743//GO:0010468//GO:0043231	nr -
Unigene112505_All	268	0.00	0.00	0.00	0.00	0.26	0.00	2.05	0.00	0.45	null	null		null	null		8.01E+00	6.14E-01		null	null		-1.10E+01	4.61E-04	Down	-2.20E+00	2.24E-02								
Unigene112506_All	268	0.00	0.00	0.00	0.00	0.00	0.00	5.21	0.31	0.00	null	null		null	null		null	null		null	null		-4.06E+00	9.64E-08	Down	-1.23E+01	2.40E-10	Down	Unigene112506_All//gi|300264478|gb|EFJ48674.1|//3.00E-18//component of cytosolic 80S ribosome and 60S large subunit [Volvox carteri f. nagariensis]	Unigene112506_All//7.00E-17//AT3G06680| 60S ribosomal protein L29 (RPL29B)			ko03010|Ribosome	GO:0005198//GO:0010467//GO:0030529	nr -
Unigene112511_All	269	0.00	0.00	0.59	1.11	0.38	1.01	3.14	0.78	0.44	null	null		9.21E+00	2.39E-01		-1.53E+00	2.98E-01		-1.41E-01	9.21E-01		-2.02E+00	7.45E-03		-2.82E+00	5.38E-04	Down							
Unigene112515_All	270	0.19	0.00	0.00	0.00	0.00	0.00	2.19	0.00	0.00	-7.60E+00	6.17E-01		-7.60E+00	6.30E-01		null	null		null	null		-1.11E+01	2.39E-04	Down	-1.11E+01	1.41E-04	Down							
Unigene112518_All	270	0.00	0.00	0.00	0.00	0.00	0.00	5.32	1.86	0.15	null	null		null	null		null	null		null	null		-1.52E+00	3.35E-03		-5.17E+00	2.09E-09	Down							
Unigene112525_All	271	0.00	0.00	0.00	0.00	0.00	0.00	2.18	0.15	0.00	null	null		null	null		null	null		null	null		-3.82E+00	1.79E-03		-1.11E+01	1.41E-04	Down							
Unigene112528_All	271	0.19	0.37	0.39	0.92	0.25	0.00	1.87	0.00	0.59	9.53E-01	7.27E-01		1.03E+00	7.04E-01		-1.85E+00	2.75E-01		-9.84E+00	7.35E-02		-1.09E+01	8.90E-04	Down	-1.67E+00	7.78E-02				Unigene112528_All//9.00E-97//ES791525				
Unigene112534_All	272	0.00	0.00	0.00	1.47	0.38	0.33	2.95	0.31	1.03	null	null		null	null		-1.95E+00	1.19E-01		-2.14E+00	1.26E-01		-3.26E+00	4.48E-04	Down	-1.53E+00	3.04E-02								
Unigene112535_All	272	0.77	0.00	0.00	0.18	0.25	0.17	1.87	0.31	0.00	-9.59E+00	1.22E-01		-9.59E+00	1.46E-01		4.68E-01	9.60E-01		-1.41E-01	9.55E-01		-2.60E+00	2.05E-02		-1.09E+01	5.55E-04	Down	Unigene112535_All//gi|34365659|gb|AAQ65141.1|//9.00E-26//At5g37070 [Arabidopsis thaliana]	Unigene112535_All//4.00E-27//AT5G37070| unknown protein	Unigene112535_All//2.00E-23//gi|164248196|gb|ES810211.1|ES810211				nr +
Unigene112536_All	272	0.00	0.00	0.00	0.00	0.00	0.00	2.18	0.00	0.29	null	null		null	null		null	null		null	null		-1.11E+01	2.39E-04	Down	-2.89E+00	4.53E-03		Unigene112536_All//gi|87162909|gb|ABD28704.1|//6.00E-06//WD40-like [Medicago truncatula]	Unigene112536_All//5.00E-07//AT5G28350| FUNCTIONS IN: molecular_function unknown; INVOLVED IN: biological_process unknown; EXPRESSED IN: cultured cell; CONTAINS InterPro DOMAIN/s: Protein of unknown function DUF1339 (InterPro:IPR009771), WD40 repeat (InterPro:IPR001680), WD40/YVTN repeat-like (InterPro:IPR015943), Quinoprotein amine dehydrogenase, beta chain-like (InterPro:IPR011044); BEST Arabidopsis thaliana protein match is: unknown protein (TAIR:AT3G61480.1); Has 271 Blast hits to 226 proteins in 106 species: Archae - 0; Bacteria - 9; Metazoa - 133; Fungi - 64; Plants - 30; Viruses - 0; Other Eukaryotes - 35 (source: NCBI BLink).	Unigene112536_All//4.00E-22//BF275526				nr -
Unigene112538_All	272	0.00	0.00	0.00	0.00	0.00	0.00	2.80	0.00	0.15	null	null		null	null		null	null		null	null		-1.15E+01	1.68E-05	Down	-4.26E+00	8.65E-05	Down	Unigene112538_All//gi|186510913|ref|NP_190687.2|//5.00E-27//structural constituent of ribosome [Arabidopsis thaliana] >gi|67633686|gb|AAY78767.1| 60S ribosomal protein L8 [Arabidopsis thaliana]	Unigene112538_All//2.00E-28//AT3G51190| structural constituent of ribosome			ko03010|Ribosome	GO:0005198//GO:0010467//GO:0015934	nr +
Unigene112539_All	272	0.19	0.75	0.00	0.18	0.63	0.17	2.18	0.00	0.00	1.95E+00	3.76E-01		-7.59E+00	6.30E-01		1.79E+00	5.21E-01		-1.41E-01	9.55E-01		-1.11E+01	2.39E-04	Down	-1.11E+01	1.41E-04	Down			Unigene112539_All//8.00E-11//DW512877				
Unigene11254_All	652	2.57	2.88	2.61	9.78	8.03	3.32	1.56	0.06	1.65	1.63E-01	7.23E-01		2.54E-02	9.79E-01		-2.85E-01	2.51E-01		-1.56E+00	1.83E-10	Down	-4.60E+00	3.67E-06	Down	8.42E-02	8.95E-01		Unigene11254_All//gi|3540180|gb|AAC34330.1|//4.00E-07//Unknown protein [Arabidopsis thaliana]	Unigene11254_All//2.00E-08//AT1G09980| INVOLVED IN: biological_process unknown; LOCATED IN: mitochondrion; EXPRESSED IN: 22 plant structures; EXPRESSED DURING: 13 growth stages; CONTAINS InterPro DOMAIN/s: Protein of unknown function DUF676, hydrolase-like (InterPro:IPR007751); BEST Arabidopsis thaliana protein match is: ZW18 (TAIR:AT1G58350.2); Has 342 Blast hits to 275 proteins in 70 species: Archae - 0; Bacteria - 0; Metazoa - 214; Fungi - 0; Plants - 45; Viruses - 0; Other Eukaryotes - 83 (source: NCBI BLink).	Unigene11254_All//1.00E-17//DW500858				nr -
Unigene112545_All	273	0.00	0.00	0.00	0.00	0.00	0.00	9.45	0.31	0.00	null	null		null	null		null	null		null	null		-4.95E+00	1.46E-15	Down	-1.32E+01	5.57E-19	Down							ESTscan +
Unigene112546_All	273	0.00	0.00	0.00	0.00	0.00	0.00	2.79	1.84	0.00	null	null		null	null		null	null		null	null		-6.02E-01	4.12E-01		-1.14E+01	8.88E-06	Down	Unigene112546_All//gi|27923848|sp|O65059.1|RS15_PICMA//4.00E-34//RecName: Full=40S ribosomal protein S15 >gi|2982268|gb|AAC32121.1| probable 40S ribosomal protein S15 [Picea mariana]	Unigene112546_All//4.00E-33//AT5G09510| 40S ribosomal protein S15 (RPS15D)			ko03010|Ribosome	GO:0003676//GO:0005198//GO:0010467//GO:0033279	nr -
Unigene112548_All	273	0.00	0.93	0.00	2.19	0.76	0.33	4.18	0.00	0.58	9.86E+00	7.88E-02		null	null		-1.53E+00	1.01E-01		-2.73E+00	1.74E-02		-1.20E+01	3.89E-08	Down	-2.84E+00	3.60E-05	Down			Unigene112548_All//2.00E-42//gi|78347071|gb|DT567345.1|DT567345				
Unigene112550_All	274	0.00	0.00	0.00	0.00	0.00	0.00	4.01	0.31	0.29	null	null		null	null		null	null		null	null		-3.72E+00	7.15E-06	Down	-3.79E+00	3.27E-06	Down							ESTscan +
Unigene112552_All	274	0.00	0.00	0.00	0.00	0.00	0.00	2.31	0.15	0.00	null	null		null	null		null	null		null	null		-3.92E+00	9.81E-04	Down	-1.12E+01	7.12E-05	Down	Unigene112552_All//sp|P08016|EGGS_SCHMA//5.00E-07//Putative eggshell protein (Fragment) OS=Schistosoma mansoni PE=2 SV=1						swissprot +
Unigene112553_All	274	0.00	0.74	0.00	0.00	0.25	0.00	160.33	695.12	375.85	9.53E+00	1.37E-01		null	null		7.97E+00	6.14E-01		null	null		2.12E+00	0.00E+00	Up	1.23E+00	3.58E-129	Up	Unigene112553_All//gi|224103575|ref|XP_002334037.1|//5.00E-06//alkaloid o-methyltransferase related [Populus trichocarpa] >gi|224124568|ref|XP_002319364.1| alkaloid o-methyltransferase related [Populus trichocarpa] >gi|222839673|gb|EEE77996.1| alkaloid o-methyltransferase related [Populus trichocarpa] >gi|222857740|gb|EEE95287.1| alkaloid o-methyltransferase related [Populus trichocarpa]		Unigene112553_All//5.00E-46//TC157783				nr +
Unigene112554_All	274	0.00	2.40	0.00	0.00	0.00	0.00	2.78	0.15	0.00	1.12E+01	6.26E-04	Up	null	null		null	null		null	null		-4.19E+00	1.58E-04	Down	-1.14E+01	8.88E-06	Down			Unigene112554_All//1.00E-09//FL576816				
Unigene112555_All	274	0.00	0.00	0.00	0.00	0.00	0.00	1.85	0.00	0.15	null	null		null	null		null	null		null	null		-1.09E+01	8.90E-04	Down	-3.67E+00	3.75E-03								ESTscan -
Unigene112557_All	274	0.00	0.00	0.00	0.00	0.00	0.00	2.31	0.00	0.29	null	null		null	null		null	null		null	null		-1.12E+01	1.23E-04	Down	-2.99E+00	2.58E-03		Unigene112557_All//gi|1352831|sp|P48413.1|VATB_CYACA//2.00E-15//RecName: Full=V-type proton ATPase subunit B; Short=V-ATPase subunit B; AltName: Full=Vacuolar proton pump subunit B >gi|576661|gb|AAA85821.1| V-ATPase B subunit [Cyanidium caldarium]	Unigene112557_All//5.00E-16//AT4G38510| vacuolar ATP synthase subunit B, putative / V-ATPase B subunit, putative / vacuolar proton pump B subunit, putative / V-ATPase 57 kDa subunit, putative			ko00190|Oxidative phosphorylation//ko04145|Phagosome	GO:0015078//GO:0015986//GO:0016469//GO:0016820	nr -
Unigene112558_All	274	0.00	0.00	0.00	0.00	0.00	0.00	4.63	1.07	0.00	null	null		null	null		null	null		null	null		-2.12E+00	4.17E-04	Down	-1.22E+01	1.99E-09	Down	Unigene112558_All//gi|297816820|ref|XP_002876293.1|//5.00E-14//60S ribosomal protein L23A [Arabidopsis lyrata subsp. lyrata] >gi|297322131|gb|EFH52552.1| 60S ribosomal protein L23A [Arabidopsis lyrata subsp. lyrata]	Unigene112558_All//8.00E-13//AT3G55280| RPL23AB (RIBOSOMAL PROTEIN L23AB); RNA binding / nucleotide binding / structural constituent of ribosome			ko03010|Ribosome	GO:0005488//GO:0043231	nr -
Unigene112559_All	275	0.00	0.00	0.77	0.00	0.63	0.16	2.61	0.30	0.29	null	null		9.60E+00	1.42E-01		9.29E+00	2.27E-01		7.36E+00	7.01E-01		-3.10E+00	1.40E-03		-3.17E+00	8.09E-04	Down	Unigene112559_All//gi|21952815|dbj|BAC06231.1|//4.00E-06//pentatricopeptide (PPR) repeat-containing protein-like [Oryza sativa Japonica Group]	Unigene112559_All//3.00E-07//AT3G49140| EXPRESSED IN: 23 plant structures; EXPRESSED DURING: 13 growth stages; CONTAINS InterPro DOMAIN/s: Pentatricopeptide repeat (InterPro:IPR002885); BEST Arabidopsis thaliana protein match is: EMB2758 (embryo defective 2758) (TAIR:AT4G33990.1); Has 45368 Blast hits to 16108 proteins in 635 species: Archae - 60; Bacteria - 10333; Metazoa - 8211; Fungi - 3499; Plants - 14806; Viruses - 492; Other Eukaryotes - 7967 (source: NCBI BLink).	Unigene112559_All//1.00E-151//DW008051			GO:0043231	nr +
Unigene11256_All	3070	0.44	0.56	0.36	0.76	1.30	1.00	0.85	0.14	0.53	3.41E-01	5.01E-01		-2.83E-01	6.05E-01		7.71E-01	9.48E-03		3.92E-01	3.12E-01		-2.65E+00	7.82E-10	Down	-6.83E-01	4.13E-02		Unigene11256_All//gi|18406260|ref|NP_564732.1|//0.00E+00//ZW18 [Arabidopsis thaliana] >gi|186491769|ref|NP_001117514.1| ZW18 [Arabidopsis thaliana] >gi|8979939|gb|AAF82253.1|AC008051_4 Identical to gene ZW18 from Arabidopsis thaliana gb|AB028199 >gi|6520167|dbj|BAA87940.1| ZW18 [Arabidopsis thaliana]	Unigene11256_All//0.00E+00//AT1G58350| ZW18	Unigene11256_All//0.00E+00//TC163281				nr -
Unigene112560_All	275	0.00	0.00	0.00	0.00	0.00	0.00	3.84	0.46	0.00	null	null		null	null		null	null		null	null		-3.08E+00	6.12E-05	Down	-1.19E+01	6.72E-08	Down	Unigene112560_All//gi|83582438|emb|CAJ45370.1|//7.00E-15//40S ribosomal protein S5 [Chlamydomonas sp. ICE-L]	Unigene112560_All//1.00E-12//AT3G11940| ATRPS5A (RIBOSOMAL PROTEIN 5A); structural constituent of ribosome			ko03010|Ribosome	GO:0003676//GO:0005198//GO:0010467//GO:0033279	nr +
Unigene112562_All	275	0.00	0.00	0.00	0.00	0.00	0.00	1.85	0.46	0.00	null	null		null	null		null	null		null	null		-2.02E+00	5.21E-02		-1.08E+01	5.55E-04	Down							ESTscan +
Unigene112566_All	276	0.00	0.00	0.00	0.72	0.62	0.82	2.60	0.15	2.31	null	null		null	null		-2.11E-01	8.59E-01		1.81E-01	9.35E-01		-4.10E+00	2.91E-04	Down	-1.73E-01	7.94E-01		Unigene112566_All//gi|296087024|emb|CBI33287.3|//1.00E-15//unnamed protein product [Vitis vinifera]						nr +
Unigene112567_All	276	0.00	0.00	0.00	0.00	0.00	0.00	1.99	0.00	0.14	null	null		null	null		null	null		null	null		-1.10E+01	4.61E-04	Down	-3.79E+00	2.03E-03								ESTscan -
Unigene112569_All	276	0.00	0.00	0.00	0.00	0.00	0.00	2.30	0.30	0.00	null	null		null	null		null	null		null	null		-2.92E+00	4.20E-03		-1.12E+01	7.12E-05	Down	Unigene112569_All//gi|8096269|dbj|BAA95789.1|//3.00E-12//KED [Nicotiana tabacum]				ko03040|Spliceosome	GO:0005488	nr +
Unigene112572_All	277	0.00	0.00	0.00	0.00	0.00	0.00	11.45	0.00	0.29	null	null		null	null		null	null		null	null		-1.35E+01	2.00E-22	Down	-5.31E+00	1.91E-20	Down							
Unigene112573_All	277	0.00	0.00	0.00	0.00	0.00	0.00	2.75	0.00	0.00	null	null		null	null		null	null		null	null		-1.14E+01	1.68E-05	Down	-1.14E+01	8.88E-06	Down	Unigene112573_All//gi|162459555|ref|NP_001105685.1|//7.00E-10//cysteine proteinase 1 precursor [Zea mays] >gi|1706260|sp|Q10716.1|CYSP1_MAIZE RecName: Full=Cysteine proteinase 1; Flags: Precursor >gi|643597|dbj|BAA08244.1| cysteine proteinase [Zea mays]	Unigene112573_All//9.00E-10//AT4G16190| cysteine proteinase, putative				GO:0008233//GO:0031410//GO:0043231	nr +

Unigene112575_All	277	0.00	0.00	0.00	0.00	0.00	0.00	2.44	0.60	0.14	null	null		null	null		null	null		null	null		-2.02E+00	1.98E-02		-4.09E+00	3.10E-04	Down	Unigene112575_All//gi|159479072|ref|XP_001697622.1|//4.00E-09//cysteine endopeptidase [Chlamydomonas reinhardtii] >gi|158274232|gb|EDP00016.1| cysteine endopeptidase [Chlamydomonas reinhardtii]	Unigene112575_All//1.00E-08//AT3G19390| cysteine proteinase, putative / thiol protease, putative				GO:0016787	nr -
Unigene112576_All	277	0.00	0.00	0.00	0.00	0.00	0.00	2.59	0.15	0.00	null	null		null	null		null	null		null	null		-4.10E+00	2.91E-04	Down	-1.13E+01	1.78E-05	Down							
Unigene112577_All	277	0.19	0.55	0.00	0.90	0.12	0.82	2.90	0.91	0.29	1.54E+00	5.32E-01		-7.56E+00	6.30E-01		-2.85E+00	1.37E-01		-1.42E-01	9.29E-01		-1.68E+00	2.46E-02		-3.33E+00	2.47E-04	Down							
Unigene112579_All	277	0.00	0.18	1.73	0.18	0.25	0.00	2.14	0.60	0.00	7.51E+00	6.20E-01		1.08E+01	7.49E-03		4.67E-01	9.60E-01		-7.49E+00	6.42E-01		-1.82E+00	4.72E-02		-1.11E+01	1.41E-04	Down			Unigene112579_All//3.00E-07//TC132286				
Unigene112586_All	278	0.00	0.00	0.00	0.00	0.00	0.00	5.78	0.00	0.72	null	null		null	null		null	null		null	null		-1.25E+01	2.17E-11	Down	-3.01E+00	2.36E-07	Down	Unigene112586_All//gi|145340168|ref|NP_567399.2|//9.00E-10//EDA21 (embryo sac development arrest 21) [Arabidopsis thaliana] >gi|254767752|sp|Q56XC2.2|DEF37_ARATH RecName: Full=Defensin-like protein 37; AltName: Full=Protein EMBRYO SAC DEVELOPMENT ARREST 21; Flags: Precursor	Unigene112586_All//4.00E-11//AT4G13235| EDA21 (embryo sac development arrest 21)					nr +
Unigene112587_All	278	0.00	0.00	0.00	0.00	0.00	0.00	4.26	0.75	0.72	null	null		null	null		null	null		null	null		-2.50E+00	1.47E-04	Down	-2.57E+00	6.98E-05	Down	Unigene112587_All//gi|13235641|emb|CAC33768.1|//6.00E-12//S-adenosyl-L-methionine:salicylic acid carboxyl methyltransferase [Stephanotis floribunda]	Unigene112587_All//2.00E-12//AT5G38020| S-adenosyl-L-methionine:carboxyl methyltransferase family protein			ko00592|alpha-Linolenic acid metabolism	GO:0016740	nr +
Unigene112588_All	279	0.00	0.00	0.00	0.00	0.00	0.00	2.27	0.60	0.14	null	null		null	null		null	null		null	null		-1.92E+00	3.09E-02		-3.99E+00	5.82E-04	Down							
Unigene112589_All	279	0.19	0.00	0.00	0.54	0.62	0.16	2.73	2.10	0.29	-7.55E+00	6.17E-01		-7.55E+00	6.30E-01		2.04E-01	9.50E-01		-1.73E+00	5.02E-01		-3.79E-01	6.35E-01		-3.26E+00	4.48E-04	Down							
Unigene112590_All	279	0.00	0.00	0.19	0.00	0.49	0.00	2.58	0.45	0.14	null	null		7.58E+00	6.40E-01		8.95E+00	3.20E-01		null	null		-2.52E+00	4.69E-03		-4.17E+00	1.64E-04	Down							
Unigene112592_All	279	0.00	0.00	0.00	0.00	0.00	0.00	3.49	0.60	0.57	null	null		null	null		null	null		null	null		-2.54E+00	6.42E-04	Down	-2.61E+00	3.35E-04	Down	Unigene112592_All//gi|11465865|ref|NP_066414.1|//3.00E-13//cytochrome c oxidase subunit 2 [Ochromonas danica] >gi|10505198|gb|AAG18380.1|AF287134_5 cytochrome c oxidase subunit 2 [Ochromonas danica]				ko00190|Oxidative phosphorylation	GO:0016020//GO:0046872//GO:0055114	nr +
Unigene112599_All	280	0.00	0.00	0.00	0.00	0.00	0.00	2.27	0.00	0.00	null	null		null	null		null	null		null	null		-1.11E+01	1.23E-04	Down	-1.11E+01	7.12E-05	Down							ESTscan +
Unigene1126_All	302	23.56	11.58	19.22	18.80	16.76	25.12	15.82	9.69	5.67	-1.03E+00	4.41E-06	Down	-2.94E-01	2.29E-01		-1.66E-01	5.60E-01		4.18E-01	5.56E-02		-7.08E-01	3.87E-03		-1.48E+00	7.60E-09	Down	Unigene1126_All//gi|145910320|gb|ABP98813.1|//1.00E-18//chloroplast biotin carboxylase [Gossypium hirsutum]	Unigene1126_All//4.00E-08//AT5G35360| CAC2; acetyl-CoA carboxylase/ biotin carboxylase	Unigene1126_All//8.00E-113//ES836895			GO:0006631//GO:0009526//GO:0009532//GO:0016421//GO:0016879//GO:0019842//GO:0032559//GO:0043169	nr -
Unigene112600_All	280	0.00	0.00	0.00	0.00	0.00	0.00	2.27	1.04	0.14	null	null		null	null		null	null		null	null		-1.12E+00	1.80E-01		-3.99E+00	5.82E-04	Down	Unigene112600_All//gi|192910710|gb|ACF06463.1|//3.00E-20//actin depolymerizing factor [Elaeis guineensis]	Unigene112600_All//2.00E-20//AT3G46010| ADF1 (ACTIN DEPOLYMERIZING FACTOR 1); actin binding				GO:0008092//GO:0030036//GO:0044464	nr +
Unigene112603_All	280	0.19	0.00	0.00	0.00	0.12	0.65	2.57	0.60	0.28	-7.55E+00	6.17E-01		-7.55E+00	6.30E-01		6.94E+00	8.36E-01		9.33E+00	1.95E-01		-2.10E+00	1.25E-02		-3.17E+00	8.09E-04	Down							
Unigene112605_All	280	0.37	0.00	0.00	0.18	0.00	0.00	2.57	2.39	0.28	-8.55E+00	3.75E-01		-8.55E+00	3.97E-01		-7.47E+00	5.61E-01		-7.47E+00	6.42E-01		-1.04E-01	8.93E-01		-3.17E+00	8.09E-04	Down			Unigene112605_All//6.00E-24//FL577251				
Unigene112608_All	280	0.00	0.00	0.00	0.00	0.00	0.00	2.72	1.19	0.00	null	null		null	null		null	null		null	null		-1.19E+00	1.12E-01		-1.14E+01	8.88E-06	Down	Unigene112608_All//gi|115462177|ref|NP_001054688.1|//1.00E-26//Os05g0155100 [Oryza sativa Japonica Group] >gi|54291850|gb|AAV32218.1| cytoplasmic ribosomal protein L18 [Oryza sativa Japonica Group] >gi|113578239|dbj|BAF16602.1| Os05g0155100 [Oryza sativa Japonica Group] >gi|149392184|gb|ABR25945.1| 60S ribosomal protein l18 [Oryza sativa Indica Group]	Unigene112608_All//2.00E-26//AT5G27850| 60S ribosomal protein L18 (RPL18C)			ko03010|Ribosome	GO:0030529	nr +
Unigene112610_All	281	0.00	0.00	0.00	0.00	0.00	0.00	1.96	0.00	0.00	null	null		null	null		null	null		null	null		-1.09E+01	4.61E-04	Down	-1.09E+01	2.81E-04	Down							
Unigene112615_All	281	0.00	0.00	0.00	0.00	0.00	0.00	2.11	0.15	0.00	null	null		null	null		null	null		null	null		-3.82E+00	1.79E-03		-1.10E+01	1.41E-04	Down	Unigene112615_All//gi|116055471|emb|CAL58139.1|//7.00E-23//Ribosomal protein RPL1/RPL2/RL4L4 (ISS) [Ostreococcus tauri]	Unigene112615_All//5.00E-22//AT3G09630| 60S ribosomal protein L4/L1 (RPL4A)			ko03010|Ribosome	GO:0030529	nr +
Unigene112618_All	282	0.00	0.00	0.00	0.00	0.00	0.00	1.95	0.89	0.00	null	null		null	null		null	null		null	null		-1.13E+00	2.18E-01		-1.09E+01	2.81E-04	Down	Unigene112618_All//gi|112253589|gb|ABI14381.1|//5.00E-06//ribosomal protein S30 [Pfiesteria piscicida]						nr +
Unigene112619_All	282	0.00	0.00	0.00	0.00	0.00	0.00	3.00	0.30	0.14	null	null		null	null		null	null		null	null		-3.34E+00	2.51E-04	Down	-4.41E+00	2.37E-05	Down	Unigene112619_All//gi|192910698|gb|ACF06457.1|//2.00E-25//60S ribosomal protein L35 [Elaeis guineensis]	Unigene112619_All//8.00E-25//AT5G02610| 60S ribosomal protein L35 (RPL35D)			ko03010|Ribosome	GO:0005198//GO:0010467//GO:0015934//GO:0016020//GO:0022613	nr +
Unigene11262_All	254	1.03	3.59	0.42	7.26	4.20	3.38	10.15	3.95	2.82	1.80E+00	2.33E-02		-1.30E+00	4.37E-01		-7.88E-01	8.70E-02		-1.10E+00	2.24E-02		-1.36E+00	1.76E-04	Down	-1.85E+00	9.39E-07	Down			Unigene11262_All//1.00E-138//gi|164363932|gb|ES810591.1|ES810591				
Unigene112620_All	282	0.37	0.36	0.00	0.88	0.49	0.16	4.50	1.19	0.28	-4.62E-02	9.90E-01		-8.54E+00	3.97E-01		-8.55E-01	5.74E-01		-2.46E+00	2.24E-01		-1.92E+00	9.98E-04	Down	-3.99E+00	2.56E-07	Down							
Unigene112621_All	282	9.09	2.52	6.99	14.66	8.79	23.22	15.59	6.97	7.20	-1.85E+00	2.00E-05	Down	-3.80E-01	3.81E-01		-7.38E-01	8.92E-03		6.64E-01	3.67E-03		-1.16E+00	1.03E-05	Down	-1.11E+00	1.33E-05	Down	Unigene112621_All//gi|71370259|gb|AAZ30377.1|//6.00E-20//PHB2 [Nicotiana benthamiana]	Unigene112621_All//1.00E-08//AT4G28510| ATPHB1 (PROHIBITIN 1)	Unigene112621_All//7.00E-101//TC153736			GO:0030964//GO:0043231//GO:0050896	nr +
Unigene112622_All	282	0.00	0.00	0.00	0.00	0.12	0.32	1.95	0.00	0.28	null	null		null	null		6.93E+00	8.36E-01		8.32E+00	4.80E-01		-1.09E+01	4.61E-04	Down	-2.79E+00	7.96E-03								
Unigene112623_All	282	0.00	0.00	0.00	0.00	0.00	0.00	3.15	0.00	0.57	null	null		null	null		null	null		null	null		-1.16E+01	2.24E-06	Down	-2.48E+00	9.93E-04	Down	Unigene112623_All//gi|297796871|ref|XP_002866320.1|//1.00E-29//glycosyl transferase family 1 protein [Arabidopsis lyrata subsp. lyrata] >gi|297312155|gb|EFH42579.1| glycosyl transferase family 1 protein [Arabidopsis lyrata subsp. lyrata]	Unigene112623_All//5.00E-31//AT5G59070| glycosyl transferase family 1 protein				GO:0008152//GO:0016740	nr -
Unigene112624_All	283	0.18	0.00	0.38	0.88	0.49	0.00	5.23	1.48	1.97	-7.53E+00	6.17E-01		1.03E+00	7.04E-01		-8.55E-01	5.74E-01		-9.78E+00	7.35E-02		-1.82E+00	5.43E-04	Down	-1.41E+00	3.79E-03				Unigene112624_All//1.00E-120//CO122161				
Unigene112628_All	283	0.37	0.36	0.00	0.18	0.24	0.00	3.29	0.44	0.00	-4.64E-02	9.90E-01		-8.53E+00	3.97E-01		4.68E-01	9.60E-01		-7.46E+00	6.42E-01		-2.89E+00	3.28E-04	Down	-1.17E+01	5.48E-07	Down			Unigene112628_All//4.00E-16//TC150436				
Unigene112630_All	283	0.00	0.00	0.00	0.00	0.00	0.00	3.29	0.00	0.00	null	null		null	null		null	null		null	null		-1.17E+01	1.15E-06	Down	-1.17E+01	5.48E-07	Down							
Unigene112631_All	283	0.00	0.00	0.00	0.00	0.00	0.00	1.79	1.62	0.00	null	null		null	null		null	null		null	null		-1.42E-01	8.76E-01		-1.08E+01	5.55E-04	Down			Unigene112631_All//8.00E-11//gi|109856915|gb|DW485894.1|DW485894				
Unigene112633_All	283	0.00	0.00	0.00	0.00	0.00	0.00	6.27	0.89	0.00	null	null		null	null		null	null		null	null		-2.82E+00	2.67E-07	Down	-1.26E+01	4.12E-13	Down	Unigene112633_All//gi|223996813|ref|XP_002288080.1|//1.00E-13//RS4, ribosomal protein 4 [Thalassiosira pseudonana CCMP1335] >gi|220977196|gb|EED95523.1| RS4, ribosomal protein 4 [Thalassiosira pseudonana CCMP1335]	Unigene112633_All//9.00E-13//AT5G07090| 40S ribosomal protein S4 (RPS4B)			ko03010|Ribosome	GO:0003676//GO:0030529	nr -
Unigene112634_All	284	0.00	0.00	0.00	0.00	0.00	0.00	2.68	0.00	0.00	null	null		null	null		null	null		null	null		-1.14E+01	1.68E-05	Down	-1.14E+01	8.88E-06	Down	Unigene112634_All//gi|115439475|ref|NP_001044017.1|//2.00E-13//Os01g0706500 [Oryza sativa Japonica Group] >gi|113533548|dbj|BAF05931.1| Os01g0706500 [Oryza sativa Japonica Group]	Unigene112634_All//2.00E-11//AT2G19730| 60S ribosomal protein L28 (RPL28A)			ko03010|Ribosome		nr +
Unigene112638_All	284	0.18	0.71	0.00	0.18	0.00	0.32	3.28	0.29	0.42	1.95E+00	3.75E-01		-7.53E+00	6.30E-01		-7.45E+00	5.61E-01		8.59E-01	7.96E-01		-3.48E+00	7.79E-05	Down	-2.96E+00	1.72E-04	Down			Unigene112638_All//9.00E-85//ES804248				
Unigene112641_All	284	0.00	0.00	0.00	0.00	0.00	0.00	2.08	0.29	0.00	null	null		null	null		null	null		null	null		-2.82E+00	7.17E-03		-1.10E+01	1.41E-04	Down	Unigene112641_All//gi|195637410|gb|ACG38173.1|//1.00E-38//40S ribosomal protein S3 [Zea mays]	Unigene112641_All//2.00E-38//AT5G35530| 40S ribosomal protein S3 (RPS3C)			ko03010|Ribosome	GO:0001726//GO:0003677//GO:0003723//GO:0004518//GO:0005198//GO:0006282//GO:0006412//GO:0008134//GO:0012502//GO:0015935//GO:0016835//GO:0019900//GO:0043231//GO:0043280//GO:0043433//GO:0051540	nr +
Unigene112645_All	285	0.00	0.53	0.00	1.75	0.00	0.32	1.78	0.44	0.14	9.06E+00	2.36E-01		null	null		-1.08E+01	8.99E-04	Down	-2.46E+00	4.83E-02		-2.02E+00	5.21E-02		-3.67E+00	3.75E-03				Unigene112645_All//3.00E-07//gi|21091495|gb|BQ403808.1|BQ403808				
Unigene112646_All	285	0.00	0.00	0.00	0.35	0.36	0.48	1.78	0.00	0.00	null	null		null	null		5.23E-02	9.68E-01		4.43E-01	8.62E-01		-1.08E+01	8.90E-04	Down	-1.08E+01	5.55E-04	Down	Unigene112646_All//gi|297795265|ref|XP_002865517.1|//3.00E-30//pentatricopeptide repeat-containing protein [Arabidopsis lyrata subsp. lyrata] >gi|297311352|gb|EFH41776.1| pentatricopeptide repeat-containing protein [Arabidopsis lyrata subsp. lyrata]	Unigene112646_All//4.00E-30//AT5G42450| pentatricopeptide (PPR) repeat-containing protein					nr +
Unigene112653_All	286	0.00	0.00	0.00	0.35	0.00	0.00	11.09	3.65	0.14	null	null		null	null		-8.44E+00	3.16E-01		-8.44E+00	4.04E-01		-1.60E+00	1.50E-06	Down	-6.31E+00	9.72E-22	Down			Unigene112653_All//9.00E-14//gi|109850443|gb|DW479422.1|DW479422				
Unigene112659_All	287	0.00	2.47	0.00	0.69	0.24	0.00	1.77	0.29	0.00	1.13E+01	3.33E-04	Up	null	null		-1.53E+00	4.16E-01		-9.44E+00	1.34E-01		-2.60E+00	2.05E-02		-1.08E+01	5.55E-04	Down	Unigene112659_All//gi|8493588|gb|AAF75811.1|AC011000_14//2.00E-11//F16P17.15 [Arabidopsis thaliana]	Unigene112659_All//3.00E-15//AT4G11950| unknown protein	Unigene112659_All//4.00E-56//AI726683			GO:0044464	nr +
Unigene112664_All	287	0.00	0.71	0.00	2.43	0.60	0.31	2.95	1.46	0.28	9.46E+00	1.37E-01		null	null		-2.02E+00	2.15E-02		-2.95E+00	5.84E-03		-1.02E+00	1.41E-01		-3.41E+00	1.35E-04	Down							ESTscan -
Unigene112665_All	288	0.00	0.00	0.00	0.00	0.00	0.00	2.64	0.29	0.69	null	null		null	null		null	null		null	null		-3.19E+00	7.93E-04	Down	-1.93E+00	1.14E-02								
Unigene112666_All	288	0.36	0.88	0.00	0.69	0.12	0.47	2.20	1.74	0.00	1.28E+00	4.35E-01		-8.51E+00	3.97E-01		-2.53E+00	2.34E-01		-5.56E-01	7.33E-01		-3.39E-01	6.63E-01		-1.11E+01	7.12E-05	Down			Unigene112666_All//3.00E-07//ES798867				
Unigene112667_All	288	0.00	0.00	0.00	0.00	0.00	0.00	4.40	1.02	0.00	null	null		null	null		null	null		null	null		-2.12E+00	4.17E-04	Down	-1.21E+01	1.99E-09	Down	Unigene112667_All//gi|116057495|emb|CAL51922.1|//4.00E-33//Putative 40S ribosomal protein S17 (ISS) [Ostreococcus tauri]	Unigene112667_All//4.00E-34//AT5G04800| 40S ribosomal protein S17 (RPS17D)			ko03010|Ribosome	GO:0005198//GO:0010467//GO:0015935	nr -
Unigene112669_All	288	0.00	0.00	0.00	0.00	0.00	0.00	2.20	0.00	0.00	null	null		null	null		null	null		null	null		-1.11E+01	1.23E-04	Down	-1.11E+01	7.12E-05	Down	Unigene112669_All//gi|157093587|gb|ABV22448.1|//6.00E-17//60S ribosomal protein L21 [Oxyrrhis marina] >gi|157093591|gb|ABV22450.1| ribosomal protein L21 [Oxyrrhis marina]	Unigene112669_All//3.00E-16//AT1G57860| 60S ribosomal protein L21			ko03010|Ribosome	GO:0005622	nr -
Unigene112671_All	289	0.36	0.00	0.00	0.00	0.24	0.00	2.34	1.30	0.00	-8.50E+00	3.75E-01		-8.50E+00	3.97E-01		7.90E+00	6.14E-01		null	null		-8.47E-01	2.88E-01		-1.12E+01	3.56E-05	Down							
Unigene112672_All	289	0.00	0.00	0.00	0.00	0.00	0.00	2.63	0.58	0.00	null	null		null	null		null	null		null	null		-2.19E+00	7.78E-03		-1.14E+01	8.88E-06	Down							ESTscan +
Unigene112675_All	289	0.00	0.00	1.11	1.55	0.48	0.78	2.34	0.14	0.55	null	null		1.01E+01	4.53E-02		-1.70E+00	1.35E-01		-9.89E-01	3.94E-01		-4.02E+00	5.36E-04	Down	-2.09E+00	1.27E-02		Unigene112675_All//gi|225442190|ref|XP_002274489.1|//2.00E-27//PREDICTED: similar to midasin-related [Vitis vinifera]	Unigene112675_All//5.00E-22//AT1G67120| ATP binding / ATPase/ nucleoside-triphosphatase/ nucleotide binding / transcription factor binding				GO:0005515//GO:0016043//GO:0017111//GO:0030554//GO:0043231//GO:0044238//GO:0044260	nr +
Unigene112677_All	290	0.00	0.00	0.00	0.00	0.00	0.00	2.48	1.44	0.00	null	null		null	null		null	null		null	null		-7.82E-01	3.11E-01		-1.13E+01	1.78E-05	Down	Unigene112677_All//gi|289065194|ref|YP_003434246.1|//1.00E-19//ATP synthase F0 subunit 9 [Chattonella marina] >gi|288871906|dbj|BAI70593.1| ATP synthase F0 subunit 9 [Chattonella marina]	Unigene112677_All//2.00E-06//ATMG01080| subunit 9 of mitochondrial F0-ATPase			ko00190|Oxidative phosphorylation	GO:0000166//GO:0005739//GO:0006754//GO:0006818//GO:0015078//GO:0031224//GO:0033177	nr -
Unigene112681_All	290	0.00	0.00	0.00	0.00	0.00	0.00	3.35	0.58	0.69	null	null		null	null		null	null		null	null		-2.54E+00	6.41E-04	Down	-2.29E+00	9.84E-04	Down							ESTscan -
Unigene112682_All	290	0.18	0.00	0.00	0.17	0.00	0.00	4.08	2.02	0.55	-7.50E+00	6.17E-01		-7.50E+00	6.30E-01		-7.42E+00	5.61E-01		-7.42E+00	6.42E-01		-1.02E+00	7.15E-02		-2.89E+00	2.03E-05	Down							
Unigene112684_All	290	0.00	0.00	0.00	0.00	0.00	0.00	2.19	0.00	0.69	null	null		null	null		null	null		null	null		-1.11E+01	1.23E-04	Down	-1.67E+00	4.29E-02								ESTscan -
Unigene112688_All	291	0.36	0.00	0.00	0.00	0.00	0.00	4.36	0.72	3.15	-8.49E+00	3.75E-01		-8.49E+00	3.97E-01		null	null		null	null		-2.60E+00	5.14E-05	Down	-4.69E-01	3.69E-01								
Unigene112690_All	291	0.00	0.00	0.00	0.00	0.00	0.00	3.63	1.29	0.00	null	null		null	null		null	null		null	null		-1.49E+00	1.64E-02		-1.18E+01	6.72E-08	Down	Unigene112690_All//gi|112253669|gb|ABI14419.1|//3.00E-27//heat shock protein 90 [Karlodinium micrum]	Unigene112690_All//3.00E-21//AT5G52640| ATHSP90.1 (HEAT SHOCK PROTEIN 90.1); ATP binding / unfolded protein binding			ko04626|Plant-pathogen interaction//ko04141|Protein processing in endoplasmic reticulum	GO:0005488	nr -

Unigene112692_All	292	0.00	0.00	0.00	0.00	0.00	0.00	2.61	0.00	0.00	null	null		null	null		null	null		null	null		-1.13E+01	1.68E-05	Down	-1.13E+01	8.88E-06	Down	Unigene112692_All//gi|297833766|ref|XP_002884765.1|//4.00E-18//pollen ole e 1 allergen and extensin family protein [Arabidopsis lyrata subsp. lyrata] >gi|297330605|gb|EFH61024.1| pollen ole e 1 allergen and extensin family protein [Arabidopsis lyrata subsp. lyrata]	Unigene112692_All//2.00E-18//AT3G09925| pollen Ole e 1 allergen and extensin family protein				GO:0044464	nr -
Unigene112694_All	292	0.00	0.00	0.00	0.00	0.00	0.00	4.05	0.57	0.27	null	null		null	null		null	null		null	null		-2.82E+00	4.41E-05	Down	-3.89E+00	9.21E-07	Down	Unigene112694_All//gi|117940159|sp|P93051.2|ERDL7_ARATH//6.00E-25//RecName: Full=Sugar transporter ERD6-like 7	Unigene112694_All//2.00E-26//AT2G48020| sugar transporter, putative				GO:0006810//GO:0009536//GO:0016020//GO:0016491//GO:0022891	nr +
Unigene112698_All	293	0.00	0.00	0.00	0.00	0.00	0.00	3.90	0.43	0.14	null	null		null	null		null	null		null	null		-3.19E+00	1.96E-05	Down	-4.84E+00	2.34E-07	Down	Unigene112698_All//gi|255076213|ref|XP_002501781.1|//8.00E-27//histone H2B [Micromonas sp. RCC299] >gi|226517045|gb|ACO63039.1| histone H2B [Micromonas sp. RCC299]	Unigene112698_All//8.00E-24//AT3G09480| histone H2B, putative				GO:0000785//GO:0003676//GO:0034728//GO:0043231	nr +
Unigene112699_All	293	0.00	0.00	0.00	0.00	0.00	0.00	1.88	0.00	0.00	null	null		null	null		null	null		null	null		-1.09E+01	4.61E-04	Down	-1.09E+01	2.81E-04	Down							ESTscan +
Unigene1127_All	952	21.93	18.26	23.94	20.62	22.10	23.10	27.58	13.17	13.56	-2.65E-01	3.37E-02		1.27E-01	3.57E-01		1.00E-01	5.07E-01		1.64E-01	2.19E-01		-1.07E+00	2.48E-26	Down	-1.02E+00	1.85E-25	Down	Unigene1127_All//gi|145910320|gb|ABP98813.1|//1.00E-115//chloroplast biotin carboxylase [Gossypium hirsutum]	Unigene1127_All//3.00E-89//AT5G35360| CAC2; acetyl-CoA carboxylase/ biotin carboxylase	Unigene1127_All//0.00E+00//gi|78323863|gb|DT544137.1|DT544137		ko00280|Valine, leucine and isoleucine degradation	GO:0006631//GO:0009526//GO:0009532//GO:0016421//GO:0016879//GO:0019842//GO:0032559//GO:0043169	nr -
Unigene11270_All	317	2.64	0.32	0.00	1.73	0.33	1.42	10.54	2.24	3.77	-3.05E+00	2.11E-03		-1.14E+01	1.19E-04	Down	-2.41E+00	2.44E-02		-2.79E-01	7.57E-01		-2.23E+00	2.92E-10	Down	-1.48E+00	1.86E-06	Down			Unigene11270_All//8.00E-70//TC154667		ko00230|Purine metabolism		kegg -
Unigene112700_All	293	0.00	0.00	0.00	0.00	0.00	0.00	1.73	0.29	0.00	null	null		null	null		null	null		null	null		-2.60E+00	2.05E-02		-1.08E+01	5.55E-04	Down	Unigene112700_All//gi|224004780|ref|XP_002296041.1|//1.00E-40//F-type H-ATPase beta subunit [Thalassiosira pseudonana CCMP1335] >gi|209586073|gb|ACI64758.1| F-type H-ATPase beta subunit [Thalassiosira pseudonana CCMP1335]	Unigene112700_All//3.00E-39//AT5G08690| ATP synthase beta chain 2, mitochondrial			ko00190|Oxidative phosphorylation	GO:0005746//GO:0005753//GO:0008187//GO:0009526//GO:0010038//GO:0015078//GO:0015662//GO:0015986//GO:0019829//GO:0031981//GO:0032559//GO:0033178//GO:0046914	nr -
Unigene112701_All	293	0.71	0.00	1.82	1.36	1.88	0.31	2.89	0.29	1.22	-9.48E+00	1.22E-01		1.35E+00	2.25E-01		4.67E-01	6.68E-01		-2.14E+00	1.26E-01		-3.34E+00	2.51E-04	Down	-1.24E+00	6.26E-02								
Unigene112702_All	293	2.50	4.50	0.91	7.65	4.00	6.78	9.38	614.53	56.29	8.47E-01	1.46E-01		-1.46E+00	1.12E-01		-9.37E-01	2.06E-02		-1.74E-01	6.85E-01		6.03E+00	0.00E+00	Up	2.59E+00	9.83E-58	Up	Unigene112702_All//gi|297242517|gb|ADI24922.1|//2.00E-31//pirin [Carica papaya]	Unigene112702_All//6.00E-24//AT1G50590| pirin, putative	Unigene112702_All//4.00E-121//DW507929			GO:0005515//GO:0043231	nr +
Unigene112703_All	293	0.00	0.00	0.00	0.00	0.00	0.00	3.46	0.00	0.68	null	null		null	null		null	null		null	null		-1.18E+01	2.97E-07	Down	-2.35E+00	5.84E-04	Down							
Unigene112705_All	293	0.71	0.35	0.00	1.02	0.71	0.77	2.89	0.00	0.14	-1.05E+00	5.65E-01		-9.48E+00	1.46E-01		-5.33E-01	6.79E-01		-4.05E-01	7.52E-01		-1.15E+01	4.39E-06	Down	-4.41E+00	2.37E-05	Down	Unigene112705_All//gi|15231488|ref|NP_187419.1|//2.00E-36//exostosin family protein [Arabidopsis thaliana] >gi|75207507|sp|Q9SSE8.1|GLYT1_ARATH RecName: Full=Probable glycosyltransferase At3g07620	Unigene112705_All//1.00E-37//AT3G07620| exostosin family protein				GO:0008375//GO:0044464	nr +
Unigene112706_All	293	0.00	0.00	0.00	0.00	0.00	0.00	1.88	0.00	0.00	null	null		null	null		null	null		null	null		-1.09E+01	4.61E-04	Down	-1.09E+01	2.81E-04	Down	Unigene112706_All//gi|297826751|ref|XP_002881258.1|//1.00E-17//gamma subunit of mitochondrial F1-ATPase [Arabidopsis lyrata subsp. lyrata] >gi|297327097|gb|EFH57517.1| gamma subunit of mitochondrial F1-ATPase [Arabidopsis lyrata subsp. lyrata]	Unigene112706_All//8.00E-16//AT2G33040| ATP synthase gamma chain, mitochondrial (ATPC)			ko00190|Oxidative phosphorylation	GO:0006754//GO:0006818//GO:0015078//GO:0019829//GO:0032559//GO:0033178//GO:0043231	nr +
Unigene112707_All	294	0.00	0.00	0.00	0.00	0.00	0.00	2.01	0.57	0.00	null	null		null	null		null	null		null	null		-1.82E+00	4.72E-02		-1.10E+01	1.41E-04	Down	Unigene112707_All//gi|226457105|gb|EEH54405.1|//2.00E-10//MIF4G and MA3 domain-containing protein [Micromonas pusilla CCMP1545]					GO:0003676//GO:0004672//GO:0006464//GO:0032559//GO:0043231	nr -
Unigene112708_All	294	0.00	0.00	0.00	0.68	0.12	0.00	1.73	0.14	0.00	null	null		null	null		-2.53E+00	2.34E-01		-9.40E+00	1.34E-01		-3.60E+00	5.77E-03		-1.08E+01	5.55E-04	Down							
Unigene112709_All	294	0.00	0.00	0.00	0.00	0.00	0.00	2.44	0.57	0.00	null	null		null	null		null	null		null	null		-2.10E+00	1.25E-02		-1.13E+01	1.78E-05	Down	Unigene112709_All//gi|57115501|gb|AAW33059.1|//1.00E-30//apocytochrome B [Plantago rugelii]	Unigene112709_All//7.00E-26//ATMG00220| Mitochondrial apocytochrome b (cob) gene encodes a subunit of the ubiquinol-cytochrome c oxidoreductase and is part of a 5 kb transcript. The transcript also contains a pseudogene for ribosomal protein S14 called RPS15 and a tRNA(Ser) gene. Both the Cob and RPS15 genes are edited in the transcript.			ko00190|Oxidative phosphorylation	GO:0003824//GO:0022900//GO:0043231//GO:0044260//GO:0044425//GO:0090304	nr -
Unigene11271_All	665	3.62	0.84	0.72	5.54	2.07	2.78	7.82	2.70	3.77	-2.11E+00	6.17E-06	Down	-2.33E+00	3.17E-06	Down	-1.42E+00	3.29E-06	Down	-9.93E-01	1.72E-03		-1.53E+00	1.13E-09	Down	-1.05E+00	5.78E-06	Down	Unigene11271_All//gi|186495325|ref|NP_177566.3|//3.00E-50//PUR4 (purine biosynthesis 4); ATP binding / catalytic/ phosphoribosylformylglycinamidine synthase [Arabidopsis thaliana]	Unigene11271_All//2.00E-51//AT1G74260| PUR4 (purine biosynthesis 4); ATP binding / catalytic/ phosphoribosylformylglycinamidine synthase			ko00230|Purine metabolism	GO:0006188//GO:0016884//GO:0043231	nr +
Unigene112710_All	294	0.00	0.00	0.00	0.00	0.00	0.00	2.16	0.00	0.00	null	null		null	null		null	null		null	null		-1.11E+01	1.23E-04	Down	-1.11E+01	7.11E-05	Down	Unigene112710_All//gi|74272615|gb|ABA01103.1|//3.00E-33//mitochondrial ADP/ATP translocator [Chlamydomonas incerta]	Unigene112710_All//5.00E-32//AT5G13490| AAC2 (ADP/ATP carrier 2); ATP:ADP antiporter/ binding	Unigene112710_All//3.00E-10//EX172555			GO:0006862//GO:0009526//GO:0009987//GO:0015300//GO:0019866//GO:0030312//GO:0031224//GO:0031981//GO:0046914	nr -
Unigene112712_All	294	0.00	0.00	0.00	0.00	0.00	0.00	2.44	0.14	0.54	null	null		null	null		null	null		null	null		-4.10E+00	2.91E-04	Down	-2.17E+00	7.76E-03								ESTscan +
Unigene112714_All	294	0.00	0.00	0.00	0.00	0.00	0.00	1.87	0.85	0.00	null	null		null	null		null	null		null	null		-1.13E+00	2.18E-01		-1.09E+01	2.81E-04	Down	Unigene112714_All//gi|164652934|gb|ABY65000.1|//5.00E-36//14-3-3a protein [Gossypium hirsutum]	Unigene112714_All//6.00E-35//AT1G22300| GRF10 (GENERAL REGULATORY FACTOR 10); ATP binding / protein binding / protein phosphorylated amino acid binding	Unigene112714_All//2.00E-18//gi|48792798|gb|CO094112.1|CO094112			GO:0005515	nr +
Unigene112715_All	294	0.00	0.00	0.00	0.00	0.23	0.00	5.61	1.56	1.90	null	null		null	null		7.87E+00	6.14E-01		null	null		-1.84E+00	2.07E-04	Down	-1.56E+00	7.74E-04	Down							
Unigene112718_All	295	0.00	0.00	0.00	0.00	0.00	0.00	3.01	1.70	0.00	null	null		null	null		null	null		null	null		-8.24E-01	2.24E-01		-1.16E+01	1.10E-06	Down	Unigene112718_All//gi|159470055|ref|XP_001693175.1|//2.00E-15//ribosomal protein L29, component of cytosolic 80S ribosome and 60S large subunit [Chlamydomonas reinhardtii] >gi|158277433|gb|EDP03201.1| ribosomal protein L29 [Chlamydomonas reinhardtii]	Unigene112718_All//1.00E-12//AT3G06700| 60S ribosomal protein L29 (RPL29A)			ko03010|Ribosome	GO:0005198//GO:0010467//GO:0015934//GO:0022613//GO:0031981	nr -
Unigene11272_All	1299	4.35	2.46	1.97	5.60	4.75	5.35	13.18	7.05	7.12	-8.24E-01	9.97E-04		-1.14E+00	1.68E-05	Down	-2.39E-01	3.18E-01		-6.44E-02	7.94E-01		-9.04E-01	1.82E-13		-8.90E-01	1.49E-13		Unigene11272_All//gi|225449428|ref|XP_002277973.1|//0.00E+00//PREDICTED: similar to phosphoribosylformylglycinamidine synthase [Vitis vinifera]	Unigene11272_All//0.00E+00//AT1G74260| PUR4 (purine biosynthesis 4); ATP binding / catalytic/ phosphoribosylformylglycinamidine synthase	Unigene11272_All//0.00E+00//TC136476		ko00230|Purine metabolism	GO:0006188//GO:0016884//GO:0043231	nr -
Unigene112720_All	295	0.00	0.00	0.00	0.00	0.00	0.00	2.72	0.28	0.00	null	null		null	null		null	null		null	null		-3.26E+00	4.48E-04	Down	-1.14E+01	4.43E-06	Down							
Unigene112722_All	296	0.00	0.00	0.00	0.00	0.00	0.00	2.71	0.00	0.00	null	null		null	null		null	null		null	null		-1.14E+01	8.58E-06	Down	-1.14E+01	4.43E-06	Down							ESTscan +
Unigene112725_All	296	0.00	0.00	0.00	0.00	0.00	0.00	1.86	0.71	0.00	null	null		null	null		null	null		null	null		-1.40E+00	1.34E-01		-1.09E+01	2.81E-04	Down	Unigene112725_All//gi|297843614|ref|XP_002889688.1|//2.00E-29//60S ribosomal protein L10A [Arabidopsis lyrata subsp. lyrata] >gi|297335530|gb|EFH65947.1| 60S ribosomal protein L10A [Arabidopsis lyrata subsp. lyrata]	Unigene112725_All//7.00E-31//AT5G22440| 60S ribosomal protein L10A (RPL10aC)			ko03010|Ribosome	GO:0003002//GO:0003676//GO:0005198//GO:0010467//GO:0015934//GO:0016020//GO:0031981//GO:0048513	nr -
Unigene112727_All	297	0.00	0.00	0.00	0.00	0.00	0.00	1.71	2.39	0.00	null	null		null	null		null	null		null	null		4.86E-01	5.42E-01		-1.07E+01	5.55E-04	Down	Unigene112727_All//gi|297836874|ref|XP_002886319.1|//1.00E-08//expressed protein [Arabidopsis lyrata subsp. lyrata] >gi|297332159|gb|EFH62578.1| expressed protein [Arabidopsis lyrata subsp. lyrata]		Unigene112727_All//1.00E-12//DW506003				nr +
Unigene112728_All	297	0.00	0.00	0.00	0.00	0.00	0.00	2.14	0.00	0.00	null	null		null	null		null	null		null	null		-1.11E+01	1.23E-04	Down	-1.11E+01	7.11E-05	Down	Unigene112728_All//gi|260401292|gb|ACX37123.1|//2.00E-22//gag-pro [Pisum sativum]					GO:0003676//GO:0003824//GO:0043170//GO:0044238	nr -
Unigene11273_All	1135	4.10	0.49	1.78	3.99	2.73	3.34	6.89	1.55	2.28	-3.06E+00	4.37E-16	Down	-1.20E+00	5.57E-05	Down	-5.49E-01	4.55E-02		-2.57E-01	4.17E-01		-2.16E+00	4.09E-22	Down	-1.59E+00	1.88E-15	Down	Unigene11273_All//gi|225449428|ref|XP_002277973.1|//1.00E-180//PREDICTED: similar to phosphoribosylformylglycinamidine synthase [Vitis vinifera]	Unigene11273_All//2.00E-162//AT1G74260| PUR4 (purine biosynthesis 4); ATP binding / catalytic/ phosphoribosylformylglycinamidine synthase	Unigene11273_All//0.00E+00//TC136476		ko00230|Purine metabolism		nr +
Unigene112731_All	297	0.00	0.00	0.00	0.00	0.00	0.30	1.71	0.99	0.00	null	null		null	null		null	null		8.25E+00	4.80E-01		-7.94E-01	4.02E-01		-1.07E+01	5.55E-04	Down	Unigene112731_All//gi|63334283|gb|AAY40471.1|//4.00E-14//LITAF-domain-containing protein [Pisum sativum]	Unigene112731_All//2.00E-14//AT5G13190| INVOLVED IN: biological_process unknown; LOCATED IN: cellular_component unknown; EXPRESSED IN: 24 plant structures; EXPRESSED DURING: 15 growth stages; CONTAINS InterPro DOMAIN/s: LPS-induced tumor necrosis factor alpha factor (InterPro:IPR006629); Has 21 Blast hits to 21 proteins in 10 species: Archae - 0; Bacteria - 0; Metazoa - 2; Fungi - 0; Plants - 19; Viruses - 0; Other Eukaryotes - 0 (source: NCBI BLink).	Unigene112731_All//3.00E-26//ES802320				nr +
Unigene112732_All	298	0.00	0.00	0.00	0.00	0.00	0.00	2.84	0.00	0.00	null	null		null	null		null	null		null	null		-1.15E+01	4.39E-06	Down	-1.15E+01	2.21E-06	Down							ESTscan -
Unigene112737_All	298	0.00	0.00	0.00	0.00	0.00	0.00	3.41	0.28	0.94	null	null		null	null		null	null		null	null		-3.60E+00	2.38E-05	Down	-1.86E+00	3.54E-03								
Unigene112738_All	298	0.00	0.00	0.00	0.00	0.00	0.00	3.12	0.00	0.27	null	null		null	null		null	null		null	null		-1.16E+01	1.15E-06	Down	-3.55E+00	3.97E-05	Down							
Unigene11274_All	4903	3.44	2.10	2.60	7.52	6.38	7.32	11.50	5.59	6.69	-7.12E-01	1.59E-07		-4.05E-01	4.34E-03		-2.36E-01	6.35E-03		-3.80E-02	7.19E-01		-1.04E+00	1.67E-53	Down	-7.83E-01	2.31E-34		Unigene11274_All//gi|186495325|ref|NP_177566.3|//0.00E+00//PUR4 (purine biosynthesis 4); ATP binding / catalytic/ phosphoribosylformylglycinamidine synthase [Arabidopsis thaliana]	Unigene11274_All//0.00E+00//AT1G74260| PUR4 (purine biosynthesis 4); ATP binding / catalytic/ phosphoribosylformylglycinamidine synthase	Unigene11274_All//0.00E+00//TC136476		ko00230|Purine metabolism	GO:0006188//GO:0016884//GO:0043231	nr +
Unigene112742_All	299	0.18	0.34	0.00	1.17	0.23	0.00	2.97	0.28	0.13	9.54E-01	7.27E-01		-7.45E+00	6.30E-01		-2.34E+00	1.01E-01		-1.02E+01	2.13E-02		-3.41E+00	1.40E-04	Down	-4.48E+00	1.23E-05	Down			Unigene112742_All//6.00E-18//CO119622				
Unigene112743_All	300	0.00	0.00	0.00	0.00	0.00	0.00	2.54	0.00	0.00	null	null		null	null		null	null		null	null		-1.13E+01	1.68E-05	Down	-1.13E+01	8.88E-06	Down	Unigene112743_All//gi|25453222|sp|Q9SY33.1|PER7_ARATH//1.00E-23//RecName: Full=Peroxidase 7; Short=Atperox P7; AltName: Full=ATP30; Flags: Precursor >gi|4926834|gb|AAD32944.1|AC004135_19 T17H7.19 [Arabidopsis thaliana] >gi|9755387|gb|AAF98194.1|AC000107_17 F17F8.26 [Arabidopsis thaliana]	Unigene112743_All//4.00E-25//AT1G30870| cationic peroxidase, putative			ko00940|Phenylpropanoid biosynthesis//ko00360|Phenylalanine metabolism	GO:0003824//GO:0016209//GO:0031410//GO:0046872	nr -
Unigene112745_All	300	0.00	0.00	0.00	0.00	0.00	0.00	2.11	0.28	0.00	null	null		null	null		null	null		null	null		-2.92E+00	4.20E-03		-1.10E+01	7.11E-05	Down							ESTscan +
Unigene112746_All	300	0.00	0.00	0.00	0.00	0.00	0.00	2.54	1.11	0.00	null	null		null	null		null	null		null	null		-1.19E+00	1.12E-01		-1.13E+01	8.88E-06	Down	Unigene112746_All//gi|226460767|gb|EEH58061.1|//8.00E-14//profilin [Micromonas pusilla CCMP1545]	Unigene112746_All//2.00E-12//AT2G19770| PRF5 (PROFILIN5); actin binding / actin monomer binding					nr +
Unigene112747_All	300	0.17	0.00	0.71	0.66	0.11	0.15	3.81	0.70	0.40	-7.45E+00	6.17E-01		2.03E+00	3.62E-01		-2.53E+00	2.34E-01		-2.14E+00	3.47E-01		-2.45E+00	2.46E-04	Down	-3.26E+00	8.94E-06	Down							

Unigene11275_All	607	2.50	3.34	1.93	4.10	3.69	4.76	10.24	13.08	4.20	4.18E-01	3.77E-01		-3.73E-01	5.09E-01		-1.54E-01	7.36E-01		2.15E-01	6.36E-01		3.54E-01	6.38E-02		-1.29E+00	2.98E-09	Down	Unigene11275_All//gi|15450511|gb|AAK96548.1|//3.00E-27//At2g46420/F11C10.11 [Arabidopsis thaliana] >gi|20197824|gb|AAD23044.2| expressed protein [Arabidopsis thaliana] >gi|27363256|gb|AAO11547.1| At2g46420/F11C10.11 [Arabidopsis thaliana]	Unigene11275_All//2.00E-28//AT2G46420| unknown protein	Unigene11275_All//0.00E+00//TC179077				nr -
Unigene112750_All	300	0.00	0.00	0.00	0.00	0.00	0.00	3.81	1.39	0.80	null	null		null	null		null	null		null	null		-1.45E+00	1.40E-02		-2.26E+00	3.40E-04	Down	Unigene112750_All//gi|87241351|gb|ABD33209.1|//1.00E-08//Ribonuclease H [Medicago truncatula]					GO:0003824	nr +
Unigene112751_All	301	0.00	0.00	0.00	0.00	0.00	0.00	2.39	0.00	0.00	null	null		null	null		null	null		null	null		-1.12E+01	3.27E-05	Down	-1.12E+01	1.78E-05	Down							ESTscan -
Unigene112753_All	301	0.00	0.00	0.00	0.00	0.23	0.30	4.49	0.42	0.79	null	null		null	null		7.84E+00	6.14E-01		8.23E+00	4.80E-01		-3.43E+00	1.05E-06	Down	-2.50E+00	2.46E-05	Down							
Unigene112755_All	301	0.00	0.84	0.35	0.33	0.34	0.15	2.67	0.28	0.66	9.72E+00	7.88E-02		8.47E+00	4.01E-01		5.22E-02	9.68E-01		-1.14E+00	6.78E-01		-3.26E+00	4.48E-04	Down	-2.01E+00	7.11E-03		Unigene112755_All//gi|51968638|dbj|BAD43011.1|//8.00E-33//peroxidase ATP23a [Arabidopsis thaliana]	Unigene112755_All//3.00E-34//AT1G68850| peroxidase, putative	Unigene112755_All//2.00E-162//gi|31073240|gb|CA993519.1|CA993519		ko00940|Phenylpropanoid biosynthesis//ko00360|Phenylalanine metabolism	GO:0003824//GO:0005506//GO:0006950//GO:0008152//GO:0016209//GO:0044464	nr -
Unigene112757_All	302	0.00	0.00	0.00	0.00	0.00	0.00	2.80	0.42	0.13	null	null		null	null		null	null		null	null		-2.75E+00	9.73E-04	Down	-4.41E+00	2.37E-05	Down	Unigene112757_All//gi|6688812|emb|CAB65281.1|//5.00E-40//L3 Ribosomal protein [Medicago sativa subsp. x varia]	Unigene112757_All//7.00E-41//AT1G43170| ARP1 (ARABIDOPSIS RIBOSOMAL PROTEIN 1); structural constituent of ribosome	Unigene112757_All//2.00E-08//TC135599		ko03010|Ribosome	GO:0003006//GO:0005198//GO:0010467//GO:0015934//GO:0016020//GO:0019219//GO:0030312//GO:0031981	nr +
Unigene112758_All	302	0.00	0.00	0.00	0.00	0.00	0.00	1.68	0.28	0.00	null	null		null	null		null	null		null	null		-2.60E+00	2.05E-02		-1.07E+01	5.54E-04	Down			Unigene112758_All//3.00E-07//DW479710				
Unigene11276_All	1253	16.95	6.11	7.65	11.13	6.98	6.27	6.95	3.10	2.77	-1.47E+00	1.04E-28	Down	-1.15E+00	6.11E-19	Down	-6.73E-01	1.06E-06		-8.28E-01	1.84E-08		-1.16E+00	1.47E-10	Down	-1.33E+00	3.07E-13	Down	Unigene11276_All//gi|113205376|gb|AAU93592.2|//1.00E-144//uncharacterized plant-specific domain TIGR01589 family protein [Solanum demissum]	Unigene11276_All//9.00E-136//AT2G46420| unknown protein	Unigene11276_All//0.00E+00//TC145959				nr -
Unigene112760_All	302	0.00	0.00	0.00	0.00	0.00	0.00	2.24	0.28	0.00	null	null		null	null		null	null		null	null		-3.02E+00	2.43E-03		-1.11E+01	3.56E-05	Down	Unigene112760_All//gi|1173256|sp|P46299.1|RS4_GOSHI//3.00E-36//RecName: Full=40S ribosomal protein S4 >gi|488739|emb|CAA55882.1| ribosomal protein, small subunit 4e (RS4e) [Gossypium hirsutum]	Unigene112760_All//2.00E-37//AT5G07090| 40S ribosomal protein S4 (RPS4B)			ko03010|Ribosome	GO:0003723//GO:0005198//GO:0009536//GO:0010467//GO:0015935//GO:0016020//GO:0031981	nr +
Unigene112761_All	303	0.00	0.00	0.00	0.00	0.00	0.00	2.51	0.83	0.13	null	null		null	null		null	null		null	null		-1.60E+00	3.70E-02		-4.26E+00	8.65E-05	Down							
Unigene112763_All	303	0.00	0.00	0.00	0.00	0.00	0.00	9.21	0.28	0.13	null	null		null	null		null	null		null	null		-5.06E+00	5.44E-17	Down	-6.13E+00	5.32E-19	Down	Unigene112763_All//gi|300261783|gb|EFJ45994.1|//7.00E-18//component of cytosolic 80S ribosome and 40S small subunit [Volvox carteri f. nagariensis]	Unigene112763_All//5.00E-16//AT4G39200| 40S ribosomal protein S25 (RPS25E)			ko03010|Ribosome	GO:0010467//GO:0015935	nr -
Unigene112766_All	304	0.00	0.00	0.00	0.00	0.00	0.00	2.36	0.00	0.39	null	null		null	null		null	null		null	null		-1.12E+01	3.27E-05	Down	-2.59E+00	2.81E-03								
Unigene112767_All	304	0.00	0.00	0.00	0.00	0.00	0.00	2.36	0.14	1.18	null	null		null	null		null	null		null	null		-4.10E+00	2.91E-04	Down	-1.00E+00	1.66E-01								ESTscan +
Unigene112768_All	305	0.00	0.00	0.00	0.00	0.00	0.00	2.22	0.00	0.52	null	null		null	null		null	null		null	null		-1.11E+01	6.34E-05	Down	-2.09E+00	1.27E-02		Unigene112768_All//gi|9256636|ref|NP_061348.1|//5.00E-07//60S ribosomal protein L41 [Mus musculus] >gi|10863875|ref|NP_066927.1| 60S ribosomal protein L41 [Homo sapiens] >gi|20806115|ref|NP_620783.1| 60S ribosomal protein L41 [Rattus norvegicus] >gi|78217384|ref|NP_001030344.1| 60S ribosomal protein L41 [Homo sapiens] >gi|114326422|ref|NP_001041622.1| 60S ribosomal protein L41 [Felis catus] >gi|197100322|ref|NP_001124641.1| 60S ribosomal protein L41 [Pongo abelii] >gi|159476032|ref|XP_001696118.1| ribosomal protein L41, component of cytosolic 80S ribosome and 60S large subunit [Chlamydomonas reinhardtii] >gi|51701777|sp|Q6YLX4.1|RL41_CYPCA RecName: Full=60S ribosomal protein L41 >gi|51701779|sp|Q71H53.1|RL41_EPICO RecName: Full=60S ribosomal protein L41				ko03010|Ribosome		nr -
Unigene11277_All	1094	11.96	6.48	4.92	5.60	5.57	3.76	7.19	3.67	2.37	-8.83E-01	2.32E-08		-1.28E+00	4.89E-14	Down	-7.60E-03	9.67E-01		-5.76E-01	1.56E-02		-9.71E-01	2.32E-07		-1.60E+00	1.24E-15	Down	Unigene11277_All//gi|113205376|gb|AAU93592.2|//1.00E-111//uncharacterized plant-specific domain TIGR01589 family protein [Solanum demissum]	Unigene11277_All//5.00E-102//AT2G46420| unknown protein	Unigene11277_All//0.00E+00//TC129816				nr +
Unigene112772_All	306	0.00	0.00	0.00	0.00	0.00	0.00	3.18	0.00	0.00	null	null		null	null		null	null		null	null		-1.16E+01	5.83E-07	Down	-1.16E+01	2.72E-07	Down	Unigene112772_All//gi|159469668|ref|XP_001692985.1|//3.00E-41//ribosomal protein L8, component of cytosolic 80S ribosome and 60S large subunit [Chlamydomonas reinhardtii] >gi|158277787|gb|EDP03554.1| ribosomal protein L8 [Chlamydomonas reinhardtii]	Unigene112772_All//2.00E-38//AT4G36130| 60S ribosomal protein L8 (RPL8C)	Unigene112772_All//6.00E-09//ES817671		ko03010|Ribosome	GO:0005198//GO:0010467//GO:0030529	nr -
Unigene112773_All	306	0.00	0.00	0.00	0.00	0.00	0.00	3.18	1.09	0.39	null	null		null	null		null	null		null	null		-1.54E+00	1.90E-02		-3.02E+00	9.61E-05	Down	Unigene112773_All//gi|298709870|emb|CBJ26210.1|//3.00E-36//Ribosomal protein S27 [Ectocarpus siliculosus]	Unigene112773_All//2.00E-35//AT3G61110| ARS27A (ARABIDOPSIS RIBOSOMAL PROTEIN S27); structural constituent of ribosome			ko03010|Ribosome	GO:0005198//GO:0010467//GO:0015935//GO:0016020//GO:0022613//GO:0030312//GO:0043169	nr -
Unigene112776_All	306	0.00	0.00	0.00	0.00	0.00	0.00	3.04	0.00	0.00	null	null		null	null		null	null		null	null		-1.16E+01	1.15E-06	Down	-1.16E+01	5.48E-07	Down	Unigene112776_All//gi|6687299|emb|CAB64903.1|//2.00E-29//40S ribosomal protein S9 [Cyanophora paradoxa]	Unigene112776_All//6.00E-17//AT5G15200| 40S ribosomal protein S9 (RPS9B)			ko03010|Ribosome	GO:0003723//GO:0005198//GO:0010467//GO:0033279	nr -
Unigene112779_All	307	0.00	0.00	0.00	0.00	0.00	0.00	3.17	0.00	0.00	null	null		null	null		null	null		null	null		-1.16E+01	5.83E-07	Down	-1.16E+01	2.72E-07	Down							ESTscan -
Unigene112780_All	307	0.00	0.00	0.00	0.00	0.00	0.00	2.62	0.54	0.13	null	null		null	null		null	null		null	null		-2.26E+00	4.82E-03		-4.33E+00	4.54E-05	Down	Unigene112780_All//gi|219119576|ref|XP_002180545.1|//2.00E-18//ornithine decarboxylase 2 [Phaeodactylum tricornutum CCAP 1055/1] >gi|217408018|gb|EEC47953.1| ornithine decarboxylase 2 [Phaeodactylum tricornutum CCAP 1055/1]				ko00480|Glutathione metabolism//ko00330|Arginine and proline metabolism	GO:0006595//GO:0016831	nr -
Unigene112783_All	308	0.00	0.00	0.00	0.00	0.00	0.29	33.63	2.17	2.59	null	null		null	null		null	null		8.20E+00	4.80E-01		-3.95E+00	2.66E-53	Down	-3.70E+00	2.15E-52	Down			Unigene112783_All//6.00E-46//gi|109887275|gb|DW516244.1|DW516244				
Unigene112784_All	308	0.00	0.00	0.00	0.00	0.00	0.00	3.29	0.14	0.00	null	null		null	null		null	null		null	null		-4.60E+00	3.67E-06	Down	-1.17E+01	1.35E-07	Down	Unigene112784_All//gi|226454387|gb|EEH51693.1|//6.00E-16//predicted protein [Micromonas pusilla CCMP1545]						nr -
Unigene112785_All	309	0.00	0.00	0.00	0.00	0.00	0.00	2.33	0.54	0.00	null	null		null	null		null	null		null	null		-2.10E+00	1.25E-02		-1.12E+01	1.78E-05	Down	Unigene112785_All//gi|145408590|ref|YP_001152214.1|//4.00E-06//ORF124 [Pinus koraiensis] >gi|145048839|gb|ABP35456.1| ORF124 [Pinus koraiensis]		Unigene112785_All//6.00E-15//TC178045				nr +
Unigene112787_All	309	0.00	0.00	0.00	0.00	0.00	0.00	3.28	0.68	0.13	null	null		null	null		null	null		null	null		-2.28E+00	1.11E-03		-4.67E+00	1.72E-06	Down							
Unigene112788_All	309	0.34	0.33	0.00	1.29	0.33	0.00	3.83	0.81	0.39	-4.59E-02	9.90E-01		-8.40E+00	3.97E-01		-1.95E+00	1.19E-01		-1.03E+01	1.11E-02		-2.24E+00	4.21E-04	Down	-3.31E+00	4.89E-06	Down			Unigene112788_All//4.00E-07//TC177115				
Unigene11279_All	1853	1.07	2.79	0.66	1.64	3.03	4.02	1.73	2.48	3.40	1.38E+00	7.08E-07	Up	-6.99E-01	1.44E-01		8.85E-01	2.29E-04		1.29E+00	3.47E-09	Up	5.17E-01	4.21E-02		9.70E-01	3.16E-06		Unigene11279_All//gi|15450511|gb|AAK96548.1|//1.00E-143//At2g46420/F11C10.11 [Arabidopsis thaliana] >gi|20197824|gb|AAD23044.2| expressed protein [Arabidopsis thaliana] >gi|27363256|gb|AAO11547.1| At2g46420/F11C10.11 [Arabidopsis thaliana]	Unigene11279_All//1.00E-142//AT2G46420| unknown protein	Unigene11279_All//0.00E+00//TC145959				nr -
Unigene112791_All	309	0.00	0.00	0.00	0.00	0.00	0.00	2.05	0.00	0.00	null	null		null	null		null	null		null	null		-1.10E+01	1.23E-04	Down	-1.10E+01	7.11E-05	Down	Unigene112791_All//gi|298710912|emb|CBJ49265.1|//6.00E-35//3-ketoacyl-CoA thiolase [Ectocarpus siliculosus]	Unigene112791_All//6.00E-32//AT1G04710| PKT4 (PEROXISOMAL 3-KETOACYL-COA THIOLASE 4); acetyl-CoA C-acyltransferase/ catalytic			ko04146|Peroxisome//ko00592|alpha-Linolenic acid metabolism//ko00071|Fatty acid metabolism//ko00280|Valine, leucine and isoleucine degradation//ko01040|Biosynthesis of unsaturated fatty acids	GO:0006950//GO:0009062//GO:0009536//GO:0009657//GO:0009694//GO:0016020//GO:0016408//GO:0031981//GO:0042579	nr -
Unigene112793_All	310	0.00	0.00	0.00	0.00	0.00	0.00	4.50	0.94	0.64	null	null		null	null		null	null		null	null		-2.25E+00	9.69E-05	Down	-2.81E+00	4.26E-06	Down	Unigene112793_All//gi|10802935|gb|AAG23683.1|AF288091_28//4.00E-29//apocytochrome b [Thraustochytrium aureum]	Unigene112793_All//2.00E-18//ATMG00220| Mitochondrial apocytochrome b (cob) gene encodes a subunit of the ubiquinol-cytochrome c oxidoreductase and is part of a 5 kb transcript. The transcript also contains a pseudogene for ribosomal protein S14 called RPS15 and a tRNA(Ser) gene. Both the Cob and RPS15 genes are edited in the transcript.			ko00190|Oxidative phosphorylation	GO:0003824//GO:0005488//GO:0022900//GO:0043231//GO:0044425	nr +
Unigene112794_All	310	1.18	0.82	0.86	0.96	2.11	0.87	4.23	3.24	1.16	-5.32E-01	6.27E-01		-4.60E-01	6.97E-01		1.13E+00	2.38E-01		-1.41E-01	9.21E-01		-3.86E-01	4.87E-01		-1.87E+00	6.93E-04	Down	Unigene112794_All//gi|147857564|emb|CAN82884.1|//4.00E-08//hypothetical protein [Vitis vinifera]	Unigene112794_All//2.00E-05//AT3G60360| EDA14 (EMBRYO SAC DEVELOPMENT ARREST 14)	Unigene112794_All//4.00E-118//ES793613			GO:0030684//GO:0042254	nr -
Unigene112795_All	311	0.00	0.00	0.00	0.00	0.00	0.00	1.90	0.27	0.00	null	null		null	null		null	null		null	null		-2.82E+00	7.17E-03		-1.09E+01	1.41E-04	Down	Unigene112795_All//gi|226531524|ref|NP_001148960.1|//3.00E-20//ATP-dependent RNA helicase dhh1 [Zea mays] >gi|195623636|gb|ACG33648.1| ATP-dependent RNA helicase dhh1 [Zea mays]	Unigene112795_All//3.00E-21//AT4G00660| DEAD/DEAH box helicase, putative			ko03018|RNA degradation	GO:0032559//GO:0042623	nr -
Unigene112796_All	312	0.00	0.00	0.00	0.00	0.00	0.00	2.17	0.00	0.38	null	null		null	null		null	null		null	null		-1.11E+01	6.34E-05	Down	-2.50E+00	4.78E-03		Unigene112796_All//gi|301105535|ref|XP_002901851.1|//1.00E-10//villin-like protein [Phytophthora infestans T30-4] >gi|262099189|gb|EEY57241.1| villin-like protein [Phytophthora infestans T30-4]	Unigene112796_All//1.00E-07//AT3G57410| VLN3 (VILLIN 3); actin binding					nr -

<table>
<tr><td>Unigene112798_All</td><td>313</td><td>1144.87</td><td>389.65</td><td>570.44</td><td>2096.33</td><td>2781.45</td><td>1844.92</td><td>2.43</td><td>0.27</td><td>0.51</td><td>-1.55E+00</td><td>0.00E+00</td><td>Down</td><td>-1.01E+00</td><td>5.23E-252</td><td>Down</td><td>4.08E-01</td><td>4.46E-154</td><td></td><td>-1.84E-01</td><td>2.00E-23</td><td></td><td>-3.19E+00</td><td>7.93E-04</td><td>Down</td><td>-2.26E+00</td><td>4.68E-03</td><td></td><td>Unigene112798_All//gi|155966601|gb|ABU41235.1|//9.00E-45//xyloglucan endotransglycosylase [Gossypium hirsutum] >gi|166343773|gb|ABY86634.1| xyloglucan endotransglycosylase/hydrolase [Gossypium arboreum]</td><td>Unigene112798_All//2.00E-35//AT5G65730| xyloglucan:xyloglucosyl transferase, putative / xyloglucan endotransglycosylase, putative / endo-xyloglucan transferase, putative</td><td>Unigene112798_All//3.00E-174//gi|11202308|gb|BF271313.1|BF271313</td><td></td><td></td><td>GO:0005576//GO:0016758//GO:0016798//GO:0030312//GO:0044042</td><td>nr -</td></tr>
<tr><td>Unigene1128_All</td><td>1260</td><td>48.58</td><td>40.94</td><td>54.52</td><td>26.88</td><td>32.47</td><td>28.39</td><td>32.41</td><td>20.00</td><td>13.15</td><td>-2.47E-01</td><td>2.75E-04</td><td></td><td>1.66E-01</td><td>1.57E-02</td><td></td><td>2.72E-01</td><td>6.84E-04</td><td></td><td>7.86E-02</td><td>4.79E-01</td><td></td><td>-6.97E-01</td><td>4.44E-20</td><td></td><td>-1.30E+00</td><td>5.97E-57</td><td>Down</td><td>Unigene1128_All//gi|145910320|gb|ABP98813.1|//0.00E+00//chloroplast biotin carboxylase [Gossypium hirsutum]</td><td>Unigene1128_All//3.00E-162//AT5G35360| CAC2; acetyl-CoA carboxylase/ biotin carboxylase</td><td>Unigene1128_All//0.00E+00//TC158265</td><td></td><td>ko00280|Valine, leucine and isoleucine degradation</td><td>GO:0003824//GO:0019842//GO:0032559</td><td>nr -</td></tr>
<tr><td>Unigene11280_All</td><td>1603</td><td>3.75</td><td>4.11</td><td>3.72</td><td>4.79</td><td>4.19</td><td>3.27</td><td>2.72</td><td>0.81</td><td>1.39</td><td>1.31E-01</td><td>6.20E-01</td><td></td><td>-1.27E-02</td><td>9.83E-01</td><td></td><td>-1.92E-01</td><td>4.19E-01</td><td></td><td>-5.50E-01</td><td>8.47E-03</td><td></td><td>-1.75E+00</td><td>9.14E-10</td><td>Down</td><td>-9.65E-01</td><td>1.41E-04</td><td></td><td>Unigene11280_All//gi|113205376|gb|AAU93592.2|//1.00E-144//uncharacterized plant-specific domain TIGR01589 family protein [Solanum demissum]</td><td>Unigene11280_All//2.00E-141//AT2G46420| unknown protein</td><td>Unigene11280_All//0.00E+00//TC145959</td><td></td><td></td><td></td><td>nr -</td></tr>
<tr><td>Unigene112802_All</td><td>313</td><td>0.00</td><td>0.00</td><td>0.00</td><td>0.00</td><td>0.00</td><td>0.00</td><td>49.71</td><td>0.00</td><td>0.89</td><td>null</td><td>null</td><td></td><td>null</td><td>null</td><td></td><td>null</td><td>null</td><td></td><td>null</td><td>null</td><td></td><td>-1.56E+01</td><td>9.81E-111</td><td>Down</td><td>-5.80E+00</td><td>1.55E-102</td><td>Down</td><td></td><td></td><td></td><td></td><td></td><td></td><td></td></tr>
<tr><td>Unigene112803_All</td><td>314</td><td>0.00</td><td>0.00</td><td>0.00</td><td>0.00</td><td>0.00</td><td>0.00</td><td>1.89</td><td>0.27</td><td>0.00</td><td>null</td><td>null</td><td></td><td>null</td><td>null</td><td></td><td>null</td><td>null</td><td></td><td>null</td><td>null</td><td></td><td>-2.82E+00</td><td>7.17E-03</td><td></td><td>-1.09E+01</td><td>1.41E-04</td><td>Down</td><td></td><td></td><td></td><td></td><td></td><td></td><td>ESTscan -</td></tr>
<tr><td>Unigene112804_All</td><td>314</td><td>0.00</td><td>0.00</td><td>0.00</td><td>0.00</td><td>0.00</td><td>0.00</td><td>3.23</td><td>0.00</td><td>0.00</td><td>null</td><td>null</td><td></td><td>null</td><td>null</td><td></td><td>null</td><td>null</td><td></td><td>null</td><td>null</td><td></td><td>-1.17E+01</td><td>2.97E-07</td><td>Down</td><td>-1.17E+01</td><td>1.35E-07</td><td>Down</td><td>Unigene112804_All//gi|115477976|ref|NP_001062583.1|//2.00E-26//Os09g0115400 [Oryza sativa Japonica Group] >gi|113630816|dbj|BAF24497.1| Os09g0115400 [Oryza sativa Japonica Group] >gi|169244485|gb|ACA50516.1| poly(A)-binding protein [Oryza sativa Japonica Group]</td><td>Unigene112804_All//7.00E-25//AT4G34110| PAB2 (POLY(A) BINDING 2); RNA binding / translation initiation factor</td><td></td><td></td><td></td><td>GO:0003723//GO:0006970</td><td>nr -</td></tr>
<tr><td>Unigene112805_All</td><td>314</td><td>0.33</td><td>0.97</td><td>0.34</td><td>0.00</td><td>0.66</td><td>0.00</td><td>3.37</td><td>0.53</td><td>0.89</td><td>1.54E+00</td><td>3.24E-01</td><td></td><td>2.53E-02</td><td>1.00E+00</td><td></td><td>9.36E+00</td><td>1.57E-01</td><td></td><td>null</td><td>null</td><td></td><td>-2.66E+00</td><td>2.25E-04</td><td>Down</td><td>-1.92E+00</td><td>2.21E-03</td><td></td><td></td><td></td><td></td><td></td><td></td><td></td><td></td></tr>
<tr><td>Unigene112806_All</td><td>314</td><td>0.00</td><td>0.00</td><td>0.00</td><td>0.00</td><td>0.00</td><td>0.00</td><td>1.89</td><td>1.06</td><td>0.00</td><td>null</td><td>null</td><td></td><td>null</td><td>null</td><td></td><td>null</td><td>null</td><td></td><td>null</td><td>null</td><td></td><td>-8.24E-01</td><td>3.48E-01</td><td></td><td>-1.09E+01</td><td>1.41E-04</td><td>Down</td><td>Unigene112806_All//gi|298707517|emb|CBJ30119.1|//9.00E-23//14-3-3-like protein [Ectocarpus siliculosus]</td><td>Unigene112806_All//5.00E-23//AT5G38480| GRF3 (GENERAL REGULATORY FACTOR 3); ATP binding / protein binding / protein phosphorylated amino acid binding</td><td></td><td></td><td></td><td>GO:0009536//GO:0016020//GO:0030312//GO:0032559</td><td>nr -</td></tr>
<tr><td>Unigene112807_All</td><td>315</td><td>0.00</td><td>0.00</td><td>0.00</td><td>0.00</td><td>0.00</td><td>0.00</td><td>4.30</td><td>0.53</td><td>0.51</td><td>null</td><td>null</td><td></td><td>null</td><td>null</td><td></td><td>null</td><td>null</td><td></td><td>null</td><td>null</td><td></td><td>-3.02E+00</td><td>4.74E-06</td><td>Down</td><td>-3.09E+00</td><td>1.97E-06</td><td>Down</td><td>Unigene112807_All//gi|260516674|gb|ACX43964.1|//2.00E-22//cysteine protease 4 [Brachiaria hybrid cultivar]</td><td>Unigene112807_All//1.00E-20//AT3G19390| cysteine proteinase, putative / thiol protease, putative</td><td></td><td></td><td></td><td>GO:0008233</td><td>nr +</td></tr>
<tr><td>Unigene112808_All</td><td>315</td><td>0.00</td><td>0.00</td><td>0.00</td><td>0.00</td><td>0.00</td><td>0.00</td><td>3.22</td><td>0.00</td><td>0.00</td><td>null</td><td>null</td><td></td><td>null</td><td>null</td><td></td><td>null</td><td>null</td><td></td><td>null</td><td>null</td><td></td><td>-1.17E+01</td><td>2.97E-07</td><td>Down</td><td>-1.17E+01</td><td>1.35E-07</td><td>Down</td><td>Unigene112808_All//gi|260516678|gb|ACX43965.1|//3.00E-24//cysteine protease 1 [Brachiaria hybrid cultivar]</td><td>Unigene112808_All//4.00E-23//AT1G09850| XBCP3 (xylem bark cysteine peptidase 3); cysteine-type endopeptidase/ cysteine-type peptidase</td><td></td><td></td><td></td><td>GO:0008233</td><td>nr +</td></tr>
<tr><td>Unigene112809_All</td><td>315</td><td>0.00</td><td>0.00</td><td>0.00</td><td>0.00</td><td>0.00</td><td>0.00</td><td>15.17</td><td>0.00</td><td>3.04</td><td>null</td><td>null</td><td></td><td>null</td><td>null</td><td></td><td>null</td><td>null</td><td></td><td>null</td><td>null</td><td></td><td>-1.39E+01</td><td>7.94E-34</td><td>Down</td><td>-2.32E+00</td><td>1.49E-15</td><td>Down</td><td></td><td></td><td>Unigene112809_All//2.00E-15//TC148479</td><td></td><td></td><td></td><td></td></tr>
<tr><td>Unigene11281_All</td><td>3463</td><td>1.78</td><td>1.73</td><td>2.69</td><td>74.01</td><td>72.43</td><td>64.01</td><td>10.77</td><td>8.50</td><td>5.10</td><td>-4.63E-02</td><td>8.52E-01</td><td></td><td>5.94E-01</td><td>2.38E-03</td><td></td><td>-3.11E-02</td><td>4.45E-01</td><td></td><td>-2.09E-01</td><td>5.14E-12</td><td></td><td>-3.42E-01</td><td>1.28E-05</td><td></td><td>-1.08E+00</td><td>6.81E-39</td><td>Down</td><td>Unigene11281_All//gi|254554078|gb|ACT67415.1|//0.00E+00//cellulose synthase [Shorea parvifolia subsp. parvifolia]</td><td>Unigene11281_All//0.00E+00//AT5G17420| IRX3 (IRREGULAR XYLEM 3); cellulose synthase</td><td>Unigene11281_All//0.00E+00//TC170081</td><td></td><td></td><td>GO:0009250//GO:0009536//GO:0009832//GO:0010395//GO:0016759//GO:0031224//GO:0042545//GO:0046914</td><td>nr +</td></tr>
<tr><td>Unigene112810_All</td><td>316</td><td>0.17</td><td>0.00</td><td>0.17</td><td>0.79</td><td>1.31</td><td>0.00</td><td>1.61</td><td>0.00</td><td>0.25</td><td>-7.37E+00</td><td>6.17E-01</td><td></td><td>2.50E-02</td><td>9.96E-01</td><td></td><td>7.30E-01</td><td>5.87E-01</td><td></td><td>-9.62E+00</td><td>7.35E-02</td><td></td><td>-1.06E+01</td><td>8.89E-04</td><td>Down</td><td>-2.67E+00</td><td>1.37E-02</td><td></td><td>Unigene112810_All//gi|224129022|ref|XP_002320481.1|//7.00E-41//nucleobase ascorbate transporter [Populus trichocarpa] >gi|222861254|gb|EEE98796.1| nucleobase ascorbate transporter [Populus trichocarpa]</td><td>Unigene112810_All//3.00E-41//AT2G05760| xanthine/uracil permease family protein</td><td>Unigene112810_All//3.00E-57//gi|78348668|gb|DT568942.1|DT568942</td><td></td><td></td><td>GO:0005215//GO:0006810//GO:0044464</td><td>nr +</td></tr>
<tr><td>Unigene112814_All</td><td>318</td><td>0.00</td><td>0.00</td><td>0.00</td><td>0.31</td><td>0.22</td><td>0.00</td><td>2.39</td><td>0.00</td><td>0.38</td><td>null</td><td>null</td><td></td><td>null</td><td>null</td><td></td><td>-5.33E-01</td><td>8.18E-01</td><td></td><td>-8.29E+00</td><td>4.04E-01</td><td></td><td>-1.12E+01</td><td>1.68E-05</td><td>Down</td><td>-2.67E+00</td><td>1.64E-03</td><td></td><td>Unigene112814_All//gi|115468064|ref|NP_001057631.1|//2.00E-13//Os06g0474300 [Oryza sativa Japonica Group] >gi|113595671|dbj|BAF19545.1| Os06g0474300 [Oryza sativa Japonica Group]</td><td>Unigene112814_All//2.00E-29//AT3G05390| unknown protein</td><td></td><td></td><td></td><td>GO:0031410//GO:0043231</td><td>nr -</td></tr>
<tr><td>Unigene112816_All</td><td>318</td><td>0.00</td><td>0.00</td><td>0.00</td><td>0.00</td><td>0.22</td><td>0.85</td><td>2.53</td><td>0.39</td><td>0.13</td><td>null</td><td>null</td><td></td><td>null</td><td>null</td><td></td><td>7.76E+00</td><td>6.14E-01</td><td></td><td>9.73E+00</td><td>7.04E-02</td><td></td><td>-2.68E+00</td><td>1.67E-03</td><td></td><td>-4.33E+00</td><td>4.54E-05</td><td>Down</td><td>Unigene112816_All//gi|224064756|ref|XP_002301547.1|//1.00E-48//multidrug/pheromone exporter, MDR family, ABC transporter family [Populus trichocarpa] >gi|222843273|gb|EEE80820.1| multidrug/pheromone exporter, MDR family, ABC transporter family [Populus trichocarpa]</td><td>Unigene112816_All//3.00E-49//AT2G47000| ABCB4 (ATP BINDING CASSETTE SUBFAMILY B4); ATPase, coupled to transmembrane movement of substances / xenobiotic-transporting ATPase</td><td>Unigene112816_All//3.00E-91//TC135162</td><td></td><td>ko02010|ABC transporters</td><td>GO:0009606//GO:0009734//GO:0009926//GO:0015114//GO:0031224//GO:0032559//GO:0042626//GO:0048588</td><td>nr -</td></tr>
<tr><td>Unigene112817_All</td><td>318</td><td>0.00</td><td>0.00</td><td>0.00</td><td>0.00</td><td>0.00</td><td>0.00</td><td>2.66</td><td>0.00</td><td>0.00</td><td>null</td><td>null</td><td></td><td>null</td><td>null</td><td></td><td>null</td><td>null</td><td></td><td>null</td><td>null</td><td></td><td>-1.14E+01</td><td>4.39E-06</td><td>Down</td><td>-1.14E+01</td><td>2.21E-06</td><td>Down</td><td>Unigene112817_All//gi|301105230|ref|XP_002901699.1|//7.00E-34//40S ribosomal protein S11 [Phytophthora infestans T30-4] >gi|262100703|gb|EEY58755.1| 40S ribosomal protein S11 [Phytophthora infestans T30-4]</td><td>Unigene112817_All//1.00E-28//AT4G30800| 40S ribosomal protein S11 (RPS11B)</td><td></td><td></td><td>ko03010|Ribosome</td><td>GO:0003723//GO:0005198//GO:0009536//GO:0010467//GO:0015935//GO:0030312</td><td>nr +</td></tr>
<tr><td>Unigene112818_All</td><td>318</td><td>0.00</td><td>0.00</td><td>0.00</td><td>0.16</td><td>0.00</td><td>0.00</td><td>2.26</td><td>0.00</td><td>0.13</td><td>null</td><td>null</td><td></td><td>null</td><td>null</td><td></td><td>-7.29E+00</td><td>5.61E-01</td><td></td><td>-7.29E+00</td><td>6.42E-01</td><td></td><td>-1.11E+01</td><td>3.27E-05</td><td>Down</td><td>-4.17E+00</td><td>1.64E-04</td><td>Down</td><td></td><td></td><td></td><td></td><td></td><td></td><td></td></tr>
<tr><td>Unigene112819_All</td><td>318</td><td>0.00</td><td>0.00</td><td>0.00</td><td>0.00</td><td>0.00</td><td>0.00</td><td>5.05</td><td>0.26</td><td>0.38</td><td>null</td><td>null</td><td></td><td>null</td><td>null</td><td></td><td>null</td><td>null</td><td></td><td>null</td><td>null</td><td></td><td>-4.26E+00</td><td>4.13E-09</td><td>Down</td><td>-3.75E+00</td><td>9.85E-09</td><td>Down</td><td></td><td></td><td></td><td></td><td></td><td></td><td></td></tr>
<tr><td>Unigene11282_All</td><td>2868</td><td>2.23</td><td>2.54</td><td>4.44</td><td>66.04</td><td>59.23</td><td>62.80</td><td>17.47</td><td>8.79</td><td>7.60</td><td>1.93E-01</td><td>3.95E-01</td><td></td><td>9.96E-01</td><td>2.38E-09</td><td></td><td>-1.57E-01</td><td>6.40E-06</td><td></td><td>-7.25E-02</td><td>8.16E-02</td><td></td><td>-9.91E-01</td><td>5.14E-44</td><td></td><td>-1.20E+00</td><td>1.58E-61</td><td>Down</td><td>Unigene11282_All//gi|67003911|gb|AAY60845.1|//0.00E+00//cellulose synthase 3 [Eucalyptus grandis]</td><td>Unigene11282_All//0.00E+00//AT5G17420| IRX3 (IRREGULAR XYLEM 3); cellulose synthase</td><td>Unigene11282_All//0.00E+00//TC172422</td><td></td><td></td><td>GO:0009250//GO:0009536//GO:0009832//GO:0010395//GO:0016759//GO:0031224//GO:0042545//GO:0046914</td><td>nr +</td></tr>
<tr><td>Unigene112821_All</td><td>319</td><td>0.00</td><td>0.00</td><td>0.00</td><td>0.00</td><td>0.00</td><td>0.00</td><td>1.86</td><td>0.00</td><td>0.00</td><td>null</td><td>null</td><td></td><td>null</td><td>null</td><td></td><td>null</td><td>null</td><td></td><td>null</td><td>null</td><td></td><td>-1.09E+01</td><td>2.39E-04</td><td>Down</td><td>-1.09E+01</td><td>1.41E-04</td><td>Down</td><td>Unigene112821_All//gi|38230174|gb|AAR14182.1|//1.00E-53//Rho family GTPase [Fucus distichus]</td><td>Unigene112821_All//5.00E-45//AT4G35020| ARAC3 (ARABIDOPSIS RAC-LIKE 3); GTP binding / GTPase</td><td></td><td></td><td>ko04145|Phagosome//ko04650|Natural killer cell mediated cytotoxicity</td><td>GO:0032561//GO:0035556//GO:0044424</td><td>nr +</td></tr>
<tr><td>Unigene112822_All</td><td>319</td><td>0.00</td><td>0.00</td><td>0.00</td><td>0.00</td><td>0.00</td><td>0.00</td><td>2.65</td><td>0.13</td><td>0.12</td><td>null</td><td>null</td><td></td><td>null</td><td>null</td><td></td><td>null</td><td>null</td><td></td><td>null</td><td>null</td><td></td><td>-4.34E+00</td><td>4.55E-05</td><td>Down</td><td>-4.41E+00</td><td>2.37E-05</td><td>Down</td><td>Unigene112822_All//gi|159483149|ref|XP_001699625.1|//1.00E-07//membrane protein [Chlamydomonas reinhardtii] >gi|158272730|gb|EDO98527.1| membrane protein [Chlamydomonas reinhardtii]</td><td>Unigene112822_All//9.00E-05//AT1G14880| unknown protein</td><td></td><td></td><td></td><td></td><td>nr +</td></tr>
<tr><td>Unigene112823_All</td><td>319</td><td>0.00</td><td>0.00</td><td>0.00</td><td>0.00</td><td>0.00</td><td>0.00</td><td>4.64</td><td>0.79</td><td>0.00</td><td>null</td><td>null</td><td></td><td>null</td><td>null</td><td></td><td>null</td><td>null</td><td></td><td>null</td><td>null</td><td></td><td>-2.56E+00</td><td>1.18E-05</td><td>Down</td><td>-1.22E+01</td><td>5.85E-11</td><td>Down</td><td></td><td></td><td></td><td></td><td></td><td></td><td>ESTscan +</td></tr>
<tr><td>Unigene112824_All</td><td>319</td><td>0.00</td><td>0.00</td><td>0.00</td><td>0.00</td><td>0.00</td><td>0.00</td><td>2.25</td><td>0.00</td><td>0.12</td><td>null</td><td>null</td><td></td><td>null</td><td>null</td><td></td><td>null</td><td>null</td><td></td><td>null</td><td>null</td><td></td><td>-1.11E+01</td><td>3.27E-05</td><td>Down</td><td>-4.17E+00</td><td>1.64E-04</td><td>Down</td><td></td><td></td><td></td><td></td><td></td><td></td><td>ESTscan +</td></tr>
<tr><td>Unigene112826_All</td><td>320</td><td>0.00</td><td>0.00</td><td>0.00</td><td>0.00</td><td>0.00</td><td>0.00</td><td>2.25</td><td>0.13</td><td>0.12</td><td>null</td><td>null</td><td></td><td>null</td><td>null</td><td></td><td>null</td><td>null</td><td></td><td>null</td><td>null</td><td></td><td>-4.10E+00</td><td>2.91E-04</td><td>Down</td><td>-4.17E+00</td><td>1.64E-04</td><td>Down</td><td></td><td></td><td></td><td></td><td></td><td></td><td>ESTscan -</td></tr>
<tr><td>Unigene112827_All</td><td>320</td><td>0.00</td><td>0.00</td><td>0.00</td><td>0.00</td><td>0.00</td><td>0.00</td><td>2.25</td><td>1.44</td><td>0.00</td><td>null</td><td>null</td><td></td><td>null</td><td>null</td><td></td><td>null</td><td>null</td><td></td><td>null</td><td>null</td><td></td><td>-6.45E-01</td><td>4.01E-01</td><td></td><td>-1.11E+01</td><td>1.78E-05</td><td>Down</td><td>Unigene112827_All//gi|75330275|sp|Q8LPC4.1|EF1A_PORYE//3.00E-47//RecName: Full=Elongation factor 1-alpha; Short=EF-1-alpha >gi|21320172|dbj|BAB96818.1| elongation factor 1-alpha [Porphyra yezoensis] >gi|31745024|dbj|BAC77640.1| elongation factor-1a [Porphyra yezoensis]</td><td>Unigene112827_All//1.00E-43//AT5G60390| elongation factor 1-alpha / EF-1-alpha</td><td>Unigene112827_All//4.00E-10//EX171978</td><td></td><td></td><td>GO:0006412//GO:0008135//GO:0017111//GO:0032561//GO:0044424</td><td>nr +</td></tr>
<tr><td>Unigene112829_All</td><td>320</td><td>0.00</td><td>0.00</td><td>0.00</td><td>0.00</td><td>0.00</td><td>0.00</td><td>3.30</td><td>0.00</td><td>0.12</td><td>null</td><td>null</td><td></td><td>null</td><td>null</td><td></td><td>null</td><td>null</td><td></td><td>null</td><td>null</td><td></td><td>-1.17E+01</td><td>1.51E-07</td><td>Down</td><td>-4.73E+00</td><td>8.85E-07</td><td>Down</td><td></td><td></td><td></td><td></td><td></td><td></td><td>ESTscan +</td></tr>
<tr><td>Unigene112834_All</td><td>322</td><td>0.00</td><td>0.00</td><td>0.00</td><td>0.00</td><td>0.00</td><td>0.00</td><td>3.94</td><td>0.26</td><td>0.25</td><td>null</td><td>null</td><td></td><td>null</td><td>null</td><td></td><td>null</td><td>null</td><td></td><td>null</td><td>null</td><td></td><td>-3.92E+00</td><td>6.20E-07</td><td>Down</td><td>-3.99E+00</td><td>2.56E-07</td><td>Down</td><td>Unigene112834_All//sp|Q55CE0|MKCF_DICDI//1.00E-06//Probable serine/threonine-protein kinase mkcF OS=Dictyostelium discoideum GN=mkcF PE=3 SV=1</td><td></td><td></td><td></td><td></td><td></td><td>swissprot +</td></tr>
<tr><td>Unigene112835_All</td><td>322</td><td>0.00</td><td>0.00</td><td>0.00</td><td>0.00</td><td>0.00</td><td>0.00</td><td>1.97</td><td>0.00</td><td>0.00</td><td>null</td><td>null</td><td></td><td>null</td><td>null</td><td></td><td>null</td><td>null</td><td></td><td>null</td><td>null</td><td></td><td>-1.09E+01</td><td>1.23E-04</td><td>Down</td><td>-1.09E+01</td><td>7.11E-05</td><td>Down</td><td>Unigene112835_All//gi|14275758|emb|CAC40035.1|//4.00E-16//P-type ATPase [Hordeum vulgare]</td><td>Unigene112835_All//9.00E-15//AT3G22910| calcium-transporting ATPase, plasma membrane-type, putative / Ca(2+)-ATPase, putative (ACA13)</td><td></td><td></td><td></td><td>GO:0006464//GO:0006812//GO:0016020//GO:0016301//GO:0032559//GO:0042625//GO:0043231//GO:0046873</td><td>nr +</td></tr>
<tr><td>Unigene112836_All</td><td>322</td><td>0.00</td><td>0.00</td><td>0.00</td><td>0.00</td><td>0.00</td><td>0.00</td><td>4.33</td><td>0.26</td><td>0.00</td><td>null</td><td>null</td><td></td><td>null</td><td>null</td><td></td><td>null</td><td>null</td><td></td><td>null</td><td>null</td><td></td><td>-4.06E+00</td><td>9.64E-08</td><td>Down</td><td>-1.21E+01</td><td>2.40E-10</td><td>Down</td><td></td><td></td><td></td><td></td><td></td><td></td><td></td></tr>
<tr><td>Unigene112837_All</td><td>322</td><td>0.00</td><td>0.00</td><td>0.00</td><td>0.00</td><td>0.00</td><td>0.00</td><td>2.23</td><td>0.26</td><td>0.25</td><td>null</td><td>null</td><td></td><td>null</td><td>null</td><td></td><td>null</td><td>null</td><td></td><td>null</td><td>null</td><td></td><td>-3.10E+00</td><td>1.40E-03</td><td></td><td>-3.17E+00</td><td>8.09E-04</td><td>Down</td><td>Unigene112837_All//gi|301016726|dbj|BAJ11784.1|//2.00E-07//dehydration responsive protein [Corchorus olitorius]</td><td></td><td>Unigene112837_All//3.00E-38//TC171825</td><td></td><td></td><td></td><td>nr +</td></tr>
<tr><td>Unigene112838_All</td><td>322</td><td>0.00</td><td>0.00</td><td>0.00</td><td>0.00</td><td>0.00</td><td>0.00</td><td>2.10</td><td>0.00</td><td>0.00</td><td>null</td><td>null</td><td></td><td>null</td><td>null</td><td></td><td>null</td><td>null</td><td></td><td>null</td><td>null</td><td></td><td>-1.10E+01</td><td>6.34E-05</td><td>Down</td><td>-1.10E+01</td><td>3.56E-05</td><td>Down</td><td>Unigene112838_All//gi|297600719|ref|NP_001049719.2|//3.00E-45//Os03g0277300 [Oryza sativa Japonica Group] >gi|255674402|dbj|BAF11633.2| Os03g0277300 [Oryza sativa Japonica Group]</td><td>Unigene112838_All//8.00E-35//AT3G12580| HSP70 (heat shock protein 70); ATP binding</td><td>Unigene112838_All//4.00E-13//EX164279</td><td></td><td>ko03040|Spliceosome//ko04141|Protein processing in endoplasmic reticulum//ko04144|Endocytosis</td><td>GO:0008152//GO:0016628//GO:0032559//GO:0050896</td><td>nr +</td></tr>
<tr><td>Unigene11284_All</td><td>1162</td><td>0.77</td><td>1.31</td><td>1.10</td><td>1.76</td><td>3.67</td><td>3.58</td><td>5.09</td><td>2.73</td><td>7.23</td><td>7.73E-01</td><td>1.48E-01</td><td></td><td>5.23E-01</td><td>3.99E-01</td><td></td><td>1.06E+00</td><td>1.83E-04</td><td>Up</td><td>1.02E+00</td><td>6.01E-04</td><td>Up</td><td>-8.98E-01</td><td>3.86E-05</td><td></td><td>5.06E-01</td><td>3.54E-03</td><td></td><td>Unigene11284_All//gi|297797804|ref|XP_002866786.1|//1.00E-166//guanine nucleotide exchange family protein [Arabidopsis lyrata subsp. lyrata] >gi|297312622|gb|EFH43045.1| guanine nucleotide exchange family protein [Arabidopsis lyrata subsp. lyrata]</td><td>Unigene11284_All//2.00E-161//AT4G38200| guanine nucleotide exchange family protein</td><td>Unigene11284_All//1.00E-22//gi|13244082|gb|BE053450.2|BE053450</td><td></td><td>ko04626|Plant-pathogen interaction</td><td></td><td>nr -</td></tr>
<tr><td>Unigene112842_All</td><td>324</td><td>0.32</td><td>0.00</td><td>0.00</td><td>1.38</td><td>0.43</td><td>0.14</td><td>3.78</td><td>0.00</td><td>0.00</td><td>-8.34E+00</td><td>3.75E-01</td><td></td><td>-8.34E+00</td><td>3.97E-01</td><td></td><td>-1.70E+00</td><td>1.35E-01</td><td></td><td>-3.31E+00</td><td>2.84E-02</td><td></td><td>-1.19E+01</td><td>9.98E-09</td><td>Down</td><td>-1.19E+01</td><td>4.03E-09</td><td>Down</td><td>Unigene112842_All//gi|302141968|emb|CBI19171.3|//1.00E-06//unnamed protein product [Vitis vinifera]</td><td>Unigene112842_All//7.00E-07//AT1G78260| RNA recognition motif (RRM)-containing protein</td><td>Unigene112842_All//1.00E-158//gi|78336615|gb|DT556889.1|DT556889</td><td></td><td></td><td></td><td>nr +</td></tr>
<tr><td>Unigene112843_All</td><td>324</td><td>0.00</td><td>0.00</td><td>0.00</td><td>0.00</td><td>0.00</td><td>0.00</td><td>1.57</td><td>0.39</td><td>0.00</td><td>null</td><td>null</td><td></td><td>null</td><td>null</td><td></td><td>null</td><td>null</td><td></td><td>null</td><td>null</td><td></td><td>-2.02E+00</td><td>5.21E-02</td><td></td><td>-1.06E+01</td><td>5.54E-04</td><td>Down</td><td></td><td></td><td></td><td></td><td></td><td></td><td>ESTscan -</td></tr>
<tr><td>Unigene112844_All</td><td>324</td><td>0.00</td><td>0.00</td><td>0.00</td><td>0.00</td><td>0.00</td><td>0.00</td><td>3.13</td><td>1.16</td><td>0.00</td><td>null</td><td>null</td><td></td><td>null</td><td>null</td><td></td><td>null</td><td>null</td><td></td><td>null</td><td>null</td><td></td><td>-1.43E+00</td><td>2.36E-02</td><td></td><td>-1.16E+01</td><td>1.35E-07</td><td>Down</td><td>Unigene112844_All//gi|40809693|dbj|BAD07266.1|//2.00E-51//beta-tubulin [Opalina sp. Rs1]</td><td>Unigene112844_All//1.00E-49//AT5G23860| TUB8; structural constituent of cytoskeleton</td><td>Unigene112844_All//9.00E-08//TC147024</td><td></td><td>ko04145|Phagosome</td><td>GO:0007017//GO:0015630//GO:0017111//GO:0032561//GO:0032991//GO:0043623</td><td>nr +</td></tr>
<tr><td>Unigene112849_All</td><td>325</td><td>0.00</td><td>0.00</td><td>0.00</td><td>0.92</td><td>0.42</td><td>0.00</td><td>2.60</td><td>2.31</td><td>0.37</td><td>null</td><td>null</td><td></td><td>null</td><td>null</td><td></td><td>-1.12E+00</td><td>4.24E-01</td><td></td><td>-9.84E+00</td><td>4.01E-02</td><td></td><td>-1.69E-01</td><td>7.99E-01</td><td></td><td>-2.82E+00</td><td>5.38E-04</td><td>Down</td><td></td><td></td><td></td><td></td><td></td><td></td><td></td></tr>
<tr><td>Unigene112851_All</td><td>326</td><td>0.00</td><td>0.00</td><td>0.00</td><td>0.00</td><td>0.00</td><td>0.00</td><td>3.63</td><td>2.05</td><td>0.00</td><td>null</td><td>null</td><td></td><td>null</td><td>null</td><td></td><td>null</td><td>null</td><td></td><td>null</td><td>null</td><td></td><td>-8.24E-01</td><td>1.42E-01</td><td></td><td>-1.18E+01</td><td>8.15E-09</td><td>Down</td><td>Unigene112851_All//gi|145353890|ref|XP_001421232.1|//5.00E-43//Ribosomal protein L27aA, component of cytosolic 80S ribosome and 60S large subunit [Ostreococcus lucimarinus CCE9901] >gi|145353965|ref|XP_001421267.1| Ribosomal protein L27aB, component of cytosolic 80S ribosome and 60S large subunit [Ostreococcus lucimarinus CCE9901] >gi|144581469|gb|ABO99525.1| Ribosomal protein L27aA, component of cytosolic 80S ribosome and 60S large subunit [Ostreococcus lucimarinus CCE9901] >gi|144581504|gb|ABO99560.1| Ribosomal protein L27aB, component of cytosolic 80S ribosome and 60S large subunit [Ostreococcus lucimarinus CCE9901]</td><td>Unigene112851_All//3.00E-35//AT1G70600| structural constituent of ribosome</td><td>Unigene112851_All//6.00E-12//CD486164</td><td></td><td>ko03010|Ribosome</td><td>GO:0005198//GO:0010467//GO:0030529</td><td>nr -</td></tr>
<tr><td>Unigene112854_All</td><td>327</td><td>0.00</td><td>0.00</td><td>0.00</td><td>0.00</td><td>0.00</td><td>0.00</td><td>3.23</td><td>0.26</td><td>0.00</td><td>null</td><td>null</td><td></td><td>null</td><td>null</td><td></td><td>null</td><td>null</td><td></td><td>null</td><td>null</td><td></td><td>-3.66E+00</td><td>1.31E-05</td><td>Down</td><td>-1.17E+01</td><td>6.72E-08</td><td>Down</td><td></td><td></td><td></td><td></td><td></td><td></td><td>ESTscan -</td></tr>
</table>

Unigene112855_All	328	0.00	0.00	0.00	0.00	0.00	0.00	2.19	0.00	0.12	null	null		null	null		null	null		null	null		-1.11E+01	3.27E-05	Down	-4.17E+00	1.64E-04	Down	Unigene112855_All//gi|192910898|gb<br>|ACF06557.1|//1.00E-<br>12//translationally controlled<br>tumor protein [Elaeis guineensis]	Unigene112855_All//2.00E-<br>13//AT3G05540| INVOLVED IN:<br>biological_process unknown;<br>LOCATED IN: cytoplasm; CONTAINS<br>InterPro DOMAIN/s: Translationally<br>controlled tumour protein<br>(InterPro:IPR018105),<br>Translationally controlled tumour-<br>associated TCTP<br>(InterPro:IPR001983),<br>Mss4/translationally controlled<br>tumour-associated TCTP<br>(InterPro:IPR011323),<br>Translationally controlled tumour<br>protein, conserved site<br>(InterPro:IPR018103), Mss4-like<br>(InterPro:IPR011057); BEST<br>Arabidopsis thaliana protein match<br>is: TCTP (TRANSLATIONALLY<br>CONTROLLED TUMOR PROTEIN)<br>(TAIR:AT3G16640.1); Has 640 Blast<br>hits to 640 proteins in 231<br>species: Archae - 0; Bacteria - 0;<br>Metazoa - 343; Fungi - 110; Plants<br>- 105; Viruses - 0; Other<br>Eukaryotes - 82 (source: NCBI<br>BLink).					nr +
Unigene112857_All	328	0.00	0.00	0.00	0.00	0.00	0.28	1.29	1.78	7.89	null	null		null	null		null	null		8.11E+00	4.80E-01		4.69E-01	6.13E-01		2.61E+00	8.54E-10	Up			Unigene112857_All//4.00E-07//BQ402741				
Unigene112859_All	328	0.00	0.00	0.00	0.00	0.00	0.00	4.64	1.66	1.09	null	null		null	null		null	null		null	null		-1.49E+00	2.91E-03		-2.09E+00	6.46E-05	Down	Unigene112859_All//gi|297811659|re<br>f|XP_002873713.1|//7.00E-41//40S<br>ribosomal protein S9 [Arabidopsis<br>lyrata subsp. lyrata]<br>>gi|297319550|gb|EFH49972.1| 40S<br>ribosomal protein S9 [Arabidopsis<br>lyrata subsp. lyrata]	Unigene112859_All//7.00E-<br>42//AT5G15200| 40S ribosomal<br>protein S9 (RPS9B)			ko03010|Ribosome	GO:0003723//GO:0005198//GO:0009536<br>//GO:0010467//GO:0015935//GO:00160<br>20//GO:0030312//GO:0031981	nr +
Unigene112860_All	329	0.00	0.00	0.00	0.00	0.00	0.00	2.06	0.00	0.00	null	null		null	null		null	null		null	null		-1.10E+01	6.34E-05	Down	-1.10E+01	3.56E-05	Down	Unigene112860_All//gi|115445077|re<br>f|NP_001046318.1|//2.00E-<br>17//Os02g0220600 [Oryza sativa<br>Japonica Group]<br>>gi|13626515|sp|Q9ZRI7.1|EF1G1_ORY<br>SJ RecName: Full=Elongation factor<br>1-gamma 1; Short=EF-1-gamma 1;<br>AltName: Full=eEF-1B gamma 1<br>>gi|3868758|dbj|BAA34206.1| elongation factor 1B gamma [Oryza<br>sativa]<br>>gi|46806491|dbj|BAD17615.1| Elongation factor 1-gamma [Oryza<br>sativa Japonica Group]<br>>gi|113535849|dbj|BAF08232.1| Os02g0220600 [Oryza sativa<br>Japonica Group]	Unigene112860_All//1.00E-<br>07//AT1G57720| elongation factor<br>1B-gamma, putative / eEF-1B gamma,<br>putative				GO:0006412	nr -
Unigene112861_All	330	0.00	0.00	0.00	0.00	0.00	0.00	2.43	0.76	0.00	null	null		null	null		null	null		null	null		-1.68E+00	2.46E-02		-1.12E+01	4.43E-06	Down	Unigene112861_All//gi|297842161|re<br>f|XP_002888962.1|//3.00E-25//60S<br>ribosomal protein L6 [Arabidopsis<br>lyrata subsp. lyrata]<br>>gi|297334803|gb|EFH65221.1| 60S<br>ribosomal protein L6 [Arabidopsis<br>lyrata subsp. lyrata]	Unigene112861_All//7.00E-<br>26//AT1G74060| 60S ribosomal<br>protein L6 (RPL6B)			ko03010|Ribosome	GO:0005198//GO:0005488//GO:0009536<br>//GO:0010467//GO:0015934//GO:00160<br>20//GO:0031981	nr +
Unigene112862_All	330	20.45	7.06	21.46	1.36	0.94	6.30	0.64	0.25	0.24	-1.53E+00	3.57E-10	Down	6.95E-02	7.70E-01		-5.33E-01	5.96E-01		2.21E+00	1.30E-05	Up	-1.34E+00	4.30E-01		-1.41E+00	3.80E-01		Unigene112862_All//gi|297794051|re<br>f|XP_002864910.1|//3.00E-<br>15//C/VIF2 [Arabidopsis lyrata<br>subsp. lyrata]<br>>gi|297310745|gb|EFH41169.1| C/VIF2 [Arabidopsis lyrata subsp.<br>lyrata]	Unigene112862_All//5.00E-<br>15//AT5G64620| C/VIF2 (CELL WALL /<br>VACUOLAR INHIBITOR OF FRUCTOSIDASE<br>2); enzyme inhibitor/<br>pectinesterase/ pectinesterase<br>inhibitor	Unigene112862_All//7.00E-89//TC166594			GO:0004091//GO:0004857//GO:0031410	nr -
Unigene112863_All	330	0.00	0.00	0.00	0.00	0.00	0.00	2.05	0.00	0.00	null	null		null	null		null	null		null	null		-1.10E+01	6.34E-05	Down	-1.10E+01	3.56E-05	Down			Unigene112863_All//3.00E-63//TC161176				ESTscan -
Unigene112865_All	331	0.00	0.00	0.00	0.00	0.00	0.00	2.17	0.38	0.00	null	null		null	null		null	null		null	null		-2.52E+00	4.69E-03		-1.11E+01	1.78E-05	Down	Unigene112865_All//gi|2500261|sp|P<br>93099.1|RL13A_CYAPA//2.00E-<br>23//RecName: Full=60S ribosomal<br>protein L13a<br>>gi|1743007|emb|CAA71090.1| ribosomal protein L13a [Cyanophora<br>paradoxa]	Unigene112865_All//2.00E-<br>19//AT3G07110| 60S ribosomal<br>protein L13A (RPL13aA)			ko03010|Ribosome	GO:0009536//GO:0015934//GO:0031981	nr -
Unigene112866_All	331	0.00	0.00	0.00	0.75	0.62	0.14	2.04	0.00	0.24	null	null		null	null		-2.70E-01	8.23E-01		-2.46E+00	2.24E-01		-1.10E+01	6.34E-05	Down	-3.09E+00	1.45E-03		Unigene112866_All//gi|18411885|ref<br>|NP_565175.1|//1.00E-31//RNA<br>recognition motif (RRM)-containing<br>protein [Arabidopsis thaliana]<br>>gi|21537200|gb|AAM61541.1| RNA<br>recognition motif-containing<br>protein SEB-4 [Arabidopsis<br>thaliana]<br>>gi|124300976|gb|ABN04740.1| At1g78260 [Arabidopsis thaliana]	Unigene112866_All//6.00E-<br>22//AT1G22330| RNA binding /<br>nucleic acid binding / nucleotide<br>binding	Unigene112866_All//0.00E+00//EE592791			GO:0003676	nr +
Unigene112868_All	331	2.37	0.00	0.00	0.30	0.00	0.00	2.94	0.38	0.72	-1.12E+01	1.09E-04	Down	-1.12E+01	2.22E-04	Down	-8.23E+00	3.16E-01		-8.23E+00	4.04E-01		-2.96E+00	1.89E-04	Down	-2.02E+00	2.47E-03		Unigene112868_All//gi|15219665|ref<br>|NP_171911.1|//2.00E-29//C2<br>domain-containing protein<br>[Arabidopsis thaliana]<br>>gi|3142295|gb|AAC16746.1| Strong<br>similarity to<br>phosphoribosylanthranilate<br>transferase gb|D86180 from Pisum<br>sativum [Arabidopsis thaliana]	Unigene112868_All//9.00E-<br>31//AT1G04150| C2 domain-<br>containing protein	Unigene112868_All//3.00E-<br>180//gi|109832103|gb|DW230662.1|DW230662			GO:0016740//GO:0044464	nr -
Unigene112869_All	331	0.00	0.00	0.00	0.00	0.00	0.00	2.04	0.25	0.00	null	null		null	null		null	null		null	null		-3.02E+00	2.43E-03		-1.10E+01	3.56E-05	Down	Unigene112869_All//gi|58801147|dbj<br>|BAD89506.1|//3.00E-52//beta-<br>tubulin [Protoopalina japonica]	Unigene112869_All//3.00E-<br>51//AT1G20010| TUB5; structural<br>constituent of cytoskeleton	Unigene112869_All//1.00E-<br>16//gi|13350074|gb|BG440423.1|BG440423		ko04145|Phagosome	GO:0007017//GO:0015630//GO:0017111<br>//GO:0032561//GO:0032991//GO:00436<br>23	nr +
Unigene112870_All	331	1.11	0.00	1.61	0.00	0.62	0.27	3.19	1.01	0.60	-1.01E+01	1.97E-02		5.40E-01	6.10E-01		9.29E+00	1.57E-01		8.09E+00	4.80E-01		-1.66E+00	8.55E-03		-2.41E+00	3.46E-04	Down							
Unigene112874_All	333	0.00	0.00	0.00	0.00	0.00	0.00	1.65	0.00	0.00	null	null		null	null		null	null		null	null		-1.07E+01	4.61E-04	Down	-1.07E+01	2.81E-04	Down	Unigene112874_All//gi|224054418|re<br>f|XP_002298250.1|//6.00E-<br>36//histidine phosphotransfer<br>protein [Populus trichocarpa]<br>>gi|222845508|gb|EEE83055.1| histidine phosphotransfer protein<br>[Populus trichocarpa]	Unigene112874_All//1.00E-<br>30//AT1G80100| AHP6 (ARABIDOPSIS<br>HISTIDINE PHOSPHOTRANSFER PROTEIN<br>6); histidine phosphotransfer<br>kinase/ transferase, transferring<br>phosphorus-containing groups				GO:0004871//GO:0008152//GO:0009736<br>//GO:0010087//GO:0016628//GO:00167<br>40//GO:0023060//GO:0043231	nr +
Unigene112875_All	333	0.00	0.00	0.00	0.00	0.00	0.00	3.17	0.13	0.96	null	null		null	null		null	null		null	null		-4.66E+00	1.94E-06	Down	-1.73E+00	4.76E-03		Unigene112875_All//gi|297838323|re<br>f|XP_002887043.1|//2.00E-21//basic<br>helix-loop-helix family protein<br>[Arabidopsis lyrata subsp. lyrata]<br>>gi|297332884|gb|EFH63302.1| basic<br>helix-loop-helix family protein<br>[Arabidopsis lyrata subsp. lyrata]	Unigene112875_All//9.00E-<br>23//AT1G66470| basic helix-loop-<br>helix (bHLH) family protein		bHLH		GO:0010468//GO:0043231	nr -
Unigene112876_All	333	0.00	0.00	0.00	0.00	0.00	0.27	2.67	0.25	0.00	null	null		null	null		null	null		8.08E+00	4.80E-01		-3.41E+00	1.40E-04	Down	-1.14E+01	1.10E-06	Down	Unigene112876_All//gi|15231435|ref<br>|NP_187383.1|//1.00E-23//GTP<br>cyclohydrolase I [Arabidopsis<br>thaliana]<br>>gi|6642638|gb|AAF20219.1|AC012395<br>_6 GTP cyclohydrolase I<br>[Arabidopsis thaliana]<br>>gi|20466832|gb|AAM20733.1| GTP<br>cyclohydrolase I [Arabidopsis<br>thaliana]<br>>gi|30387557|gb|AAP31944.1| At3g07270 [Arabidopsis thaliana]	Unigene112876_All//6.00E-<br>25//AT3G07270| GTP cyclohydrolase<br>I	Unigene112876_All//4.00E-38//TC145472		ko00790|Folate biosynthesis	GO:0003933//GO:0009396//GO:0044424	nr +
Unigene112878_All	334	0.00	0.00	0.00	0.00	0.00	0.00	1.77	0.50	0.00	null	null		null	null		null	null		null	null		-1.82E+00	4.72E-02		-1.08E+01	1.41E-04	Down	Unigene112878_All//gi|226505998|re<br>f|NP_001146929.1|//4.00E-39//60S<br>ribosomal protein L7a [Zea mays]<br>>gi|195605318|gb|ACG24489.1| 60S<br>ribosomal protein L7a [Zea mays]	Unigene112878_All//7.00E-<br>36//AT2G47610| 60S ribosomal<br>protein L7A (RPL7aA)			ko03010|Ribosome	GO:0022613//GO:0030529//GO:0046914	nr -
Unigene11288_All	1363	3.72	2.23	3.52	6.51	6.82	4.71	7.60	4.17	3.57	-7.39E-01	5.36E-03		-8.27E-02	7.70E-01		6.84E-02	7.99E-01		-4.67E-01	1.63E-02		-8.66E-01	6.20E-08		-1.09E+00	1.20E-11	Down	Unigene11288_All//gi|5834248|gb|AA<br>D53877.1|AF175124_1//1.00E-<br>175//SINAH1 protein [Gossypium<br>hirsutum]	Unigene11288_All//2.00E-<br>156//AT4G27880| seven in absentia<br>(SINA) family protein	Unigene11288_All//0.00E+00//TC132499		ko04120|Ubiquitin mediated<br>proteolysis	GO:0019787//GO:0019941//GO:0032446<br>//GO:0032501//GO:0043231//GO:00469<br>14	nr +
Unigene112883_All	336	0.00	0.00	0.00	0.00	0.10	0.00	3.52	0.12	0.47	null	null		null	null		6.68E+00	8.36E-01		null	null		-4.82E+00	2.84E-07	Down	-2.89E+00	2.03E-05	Down	Unigene112883_All//gi|113205247|gb<br>|AAT39965.2|//2.00E-<br>06//hypothetical protein<br>SDM1_29t00005 [Solanum demissum]		Unigene112883_All//1.00E-161//FL577203				nr -
Unigene112884_All	336	0.00	0.00	0.00	0.00	0.00	0.00	4.91	0.37	0.00	null	null		null	null		null	null		null	null		-3.72E+00	1.53E-08	Down	-1.23E+01	3.45E-12	Down							ESTscan -
Unigene112885_All	337	0.00	0.00	0.00	0.00	0.00	0.00	3.14	0.12	0.00	null	null		null	null		null	null		null	null		-4.66E+00	1.94E-06	Down	-1.16E+01	6.72E-08	Down	Unigene112885_All//gi|20340229|gb| AAM19701.1|AF499714_1//5.00E-<br>40//14-3-3-like protein<br>[Thellungiella halophila]	Unigene112885_All//6.00E-<br>41//AT4G09000| 14-3-3-like protein<br>GF14 chi / general regulatory<br>factor 1 (GRF1)	Unigene112885_All//4.00E-10//ES846360			GO:0005576//GO:0009536//GO:0010038<br>//GO:0016020//GO:0051219	nr +
Unigene112886_All	337	0.00	0.00	0.00	0.00	0.00	0.00	3.26	0.37	0.00	null	null		null	null		null	null		null	null		-3.13E+00	3.47E-05	Down	-1.17E+01	3.33E-08	Down	Unigene112886_All//gi|15222073|ref<br>|NP_175349.1|//7.00E-26//zinc<br>finger (C3HC4-type RING finger)<br>family protein [Arabidopsis<br>thaliana]<br>>gi|68565178|sp|Q6NQG7.1|ATL1L_ARA<br>TH RecName: Full=RING-H2 finger<br>protein ATL1L<br>>gi|34365601|gb|AAQ65112.1| At1g49230 [Arabidopsis thaliana]	Unigene112886_All//3.00E-<br>27//AT1G49230| zinc finger (C3HC4-<br>type RING finger) family protein	Unigene112886_All//8.00E-<br>98//gi|109874979|gb|DW503953.1|DW503953			GO:0019787//GO:0032446//GO:0046872	nr -
Unigene112888_All	338	0.00	0.00	0.00	0.00	0.00	0.00	3.00	1.98	0.00	null	null		null	null		null	null		null	null		-6.02E-01	3.45E-01		-1.16E+01	1.35E-07	Down							ESTscan -
Unigene112894_All	339	0.00	0.00	0.00	0.00	0.10	0.00	2.37	0.00	0.00	null	null		null	null		6.67E+00	8.36E-01		null	null		-1.12E+01	8.58E-06	Down	-1.12E+01	4.43E-06	Down	Unigene112894_All//gi|297804022|re<br>f|XP_002869895.1|//2.00E-<br>33//pentatricopeptide repeat-<br>containing protein [Arabidopsis<br>lyrata subsp. lyrata]<br>>gi|297315731|gb|EFH46154.1| pentatricopeptide repeat-<br>containing protein [Arabidopsis<br>lyrata subsp. lyrata]	Unigene112894_All//8.00E-<br>35//AT4G21300| pentatricopeptide<br>(PPR) repeat-containing protein				GO:0043231	nr +
Unigene112895_All	339	0.00	0.00	0.00	0.00	0.00	0.00	2.74	0.74	0.00	null	null		null	null		null	null		null	null		-1.89E+00	6.92E-03		-1.14E+01	5.48E-07	Down	Unigene112895_All//gi|301111866|re<br>f|XP_002905012.1|//3.00E-29//major<br>vault protein [Phytophthora<br>infestans T30-4]<br>>gi|262095342|gb|EEY53394.1| major<br>vault protein [Phytophthora<br>infestans T30-4]						nr -
Unigene112896_All	340	0.00	0.00	0.00	0.00	0.00	0.00	1.74	0.00	0.00	null	null		null	null		null	null		null	null		-1.08E+01	2.39E-04	Down	-1.08E+01	1.41E-04	Down							

<table>
<tr><td>Unigene112898_All</td><td>341</td><td>0.15</td><td>0.00</td><td>0.00</td><td>0.29</td><td>0.50</td><td>0.26</td><td>5.83</td><td>1.10</td><td>0.35</td><td>-7.26E+00</td><td>6.17E-01</td><td></td><td>-7.26E+00</td><td>6.30E-01</td><td></td><td>7.89E-01</td><td>7.71E-01</td><td></td><td>-1.42E-01</td><td>9.52E-01</td><td></td><td>-2.40E+00</td><td>6.76E-07</td><td>Down</td><td>-4.06E+00</td><td>3.04E-11</td><td>Down</td><td></td><td></td><td>Unigene112898_All//4.00E-13//gi|164295307|gb|ES844568.1|ES844568</td><td></td><td></td><td></td><td></td></tr>
<tr><td>Unigene112899_All</td><td>341</td><td>0.00</td><td>0.00</td><td>0.00</td><td>0.15</td><td>0.10</td><td>0.13</td><td>3.97</td><td>1.59</td><td>1.05</td><td>null</td><td>null</td><td></td><td>null</td><td>null</td><td></td><td>-5.33E-01</td><td>8.27E-01</td><td></td><td>-1.42E-01</td><td>9.55E-01</td><td></td><td>-1.32E+00</td><td>1.25E-02</td><td></td><td>-1.92E+00</td><td>4.37E-04</td><td>Down</td><td></td><td></td><td>Unigene112899_All//3.00E-14//gi|21103053|gb|BQ415366.1|BQ415366</td><td></td><td></td><td></td><td></td></tr>
<tr><td>Unigene112900_All</td><td>342</td><td>0.00</td><td>0.00</td><td>0.00</td><td>0.00</td><td>0.00</td><td>0.00</td><td>4.82</td><td>0.00</td><td>0.23</td><td>null</td><td>null</td><td></td><td>null</td><td>null</td><td></td><td>null</td><td>null</td><td></td><td>null</td><td>null</td><td></td><td>-1.22E+01</td><td>1.09E-11</td><td>Down</td><td>-4.37E+00</td><td>7.28E-10</td><td>Down</td><td>Unigene112900_All//gi|224140765|ref|XP_002323749.1|//2.00E-06//predicted protein [Populus trichocarpa] >gi|222866751|gb|EEF03882.1| predicted protein [Populus trichocarpa]</td><td></td><td>Unigene112900_All//5.00E-142//CA992640</td><td></td><td></td><td></td><td>nr -</td></tr>
<tr><td>Unigene112901_All</td><td>342</td><td>0.00</td><td>0.00</td><td>0.00</td><td>0.00</td><td>0.00</td><td>0.00</td><td>3.09</td><td>0.12</td><td>0.35</td><td>null</td><td>null</td><td></td><td>null</td><td>null</td><td></td><td>null</td><td>null</td><td></td><td>null</td><td>null</td><td></td><td>-4.66E+00</td><td>1.94E-06</td><td>Down</td><td>-3.14E+00</td><td>2.97E-05</td><td>Down</td><td>Unigene112901_All//gi|164652938|gb|ABY65002.1|//7.00E-31//14-3-3c protein [Gossypium hirsutum]</td><td>Unigene112901_All//2.00E-26//AT2G42590| GRF9 (GENERAL REGULATORY FACTOR 9); calcium ion binding / protein binding / protein phosphorylated amino acid binding</td><td></td><td></td><td></td><td>GO:0009532//GO:0016020//GO:0046872</td><td>nr -</td></tr>
<tr><td>Unigene112902_All</td><td>343</td><td>0.00</td><td>0.00</td><td>0.00</td><td>0.00</td><td>0.00</td><td>0.00</td><td>2.47</td><td>1.58</td><td>0.00</td><td>null</td><td>null</td><td></td><td>null</td><td>null</td><td></td><td>null</td><td>null</td><td></td><td>null</td><td>null</td><td></td><td>-6.38E-01</td><td>3.70E-01</td><td></td><td>-1.13E+01</td><td>2.21E-06</td><td>Down</td><td></td><td></td><td></td><td></td><td></td><td></td><td></td></tr>
<tr><td>Unigene112903_All</td><td>343</td><td>0.00</td><td>0.00</td><td>0.00</td><td>0.00</td><td>0.00</td><td>0.00</td><td>1.97</td><td>0.24</td><td>0.00</td><td>null</td><td>null</td><td></td><td>null</td><td>null</td><td></td><td>null</td><td>null</td><td></td><td>null</td><td>null</td><td></td><td>-3.02E+00</td><td>2.43E-03</td><td></td><td>-1.09E+01</td><td>3.56E-05</td><td>Down</td><td></td><td></td><td></td><td></td><td></td><td></td><td></td></tr>
<tr><td>Unigene112904_All</td><td>344</td><td>0.00</td><td>0.00</td><td>0.00</td><td>0.00</td><td>0.00</td><td>0.00</td><td>1.60</td><td>0.00</td><td>0.00</td><td>null</td><td>null</td><td></td><td>null</td><td>null</td><td></td><td>null</td><td>null</td><td></td><td>null</td><td>null</td><td></td><td>-1.06E+01</td><td>4.61E-04</td><td>Down</td><td>-1.06E+01</td><td>2.81E-04</td><td>Down</td><td></td><td></td><td></td><td></td><td></td><td></td><td></td></tr>
<tr><td>Unigene112906_All</td><td>344</td><td>0.46</td><td>0.00</td><td>0.00</td><td>0.00</td><td>0.40</td><td>0.00</td><td>2.83</td><td>0.97</td><td>0.46</td><td>-8.83E+00</td><td>2.16E-01</td><td></td><td>-8.83E+00</td><td>2.46E-01</td><td></td><td>8.65E+00</td><td>3.20E-01</td><td></td><td>null</td><td>null</td><td></td><td>-1.54E+00</td><td>1.90E-02</td><td></td><td>-2.61E+00</td><td>3.35E-04</td><td>Down</td><td></td><td></td><td></td><td></td><td></td><td></td><td></td></tr>
<tr><td>Unigene112907_All</td><td>346</td><td>0.00</td><td>0.29</td><td>0.00</td><td>0.29</td><td>0.90</td><td>0.00</td><td>3.30</td><td>0.72</td><td>1.61</td><td>8.19E+00</td><td>3.90E-01</td><td></td><td>null</td><td>null</td><td></td><td>1.64E+00</td><td>3.22E-01</td><td></td><td>-8.17E+00</td><td>4.04E-01</td><td></td><td>-2.19E+00</td><td>6.84E-04</td><td>Down</td><td>-1.03E+00</td><td>5.95E-02</td><td></td><td></td><td></td><td></td><td></td><td></td><td></td><td></td></tr>
<tr><td>Unigene112908_All</td><td>346</td><td>0.00</td><td>0.00</td><td>0.00</td><td>0.72</td><td>0.70</td><td>0.00</td><td>2.08</td><td>0.48</td><td>0.23</td><td>null</td><td>null</td><td></td><td>null</td><td>null</td><td></td><td>-4.73E-02</td><td>9.52E-01</td><td></td><td>-9.49E+00</td><td>7.35E-02</td><td></td><td>-2.10E+00</td><td>1.25E-02</td><td></td><td>-3.17E+00</td><td>8.09E-04</td><td>Down</td><td></td><td></td><td></td><td></td><td></td><td></td><td></td></tr>
<tr><td>Unigene112909_All</td><td>346</td><td>0.00</td><td>0.00</td><td>0.00</td><td>0.00</td><td>0.00</td><td>0.00</td><td>2.69</td><td>0.00</td><td>0.00</td><td>null</td><td>null</td><td></td><td>null</td><td>null</td><td></td><td>null</td><td>null</td><td></td><td>null</td><td>null</td><td></td><td>-1.14E+01</td><td>1.15E-06</td><td>Down</td><td>-1.14E+01</td><td>5.48E-07</td><td>Down</td><td></td><td></td><td></td><td></td><td></td><td></td><td>ESTscan -</td></tr>
<tr><td>Unigene112910_All</td><td>346</td><td>0.00</td><td>0.00</td><td>0.00</td><td>0.00</td><td>0.00</td><td>0.00</td><td>10.39</td><td>0.00</td><td>0.92</td><td>null</td><td>null</td><td></td><td>null</td><td>null</td><td></td><td>null</td><td>null</td><td></td><td>null</td><td>null</td><td></td><td>-1.33E+01</td><td>2.02E-25</td><td>Down</td><td>-3.50E+00</td><td>9.26E-18</td><td>Down</td><td>Unigene112910_All//gi|115480900|ref|NP_001064043.1|//2.00E-16//Os10g0113000 [Oryza sativa Japonica Group] >gi|73621239|sp|Q7G764.1|NADO1_ORYSJ RecName: Full=Probable NAD(P)H-dependent oxidoreductase 1 >gi|14029046|gb|AAK52587.1|AC079685_18 Putative NADPH-dependent oxidoreductase [Oryza sativa Japonica Group] >gi|21263197|gb|AAM44874.1|AC098694_13 Putative NADPH-dependent oxidoreductase [Oryza sativa Japonica Group] >gi|113638652|dbj|BAF25957.1| Os10g0113000 [Oryza sativa Japonica Group]</td><td>Unigene112910_All//5.00E-12//AT1G59960| aldo/keto reductase, putative</td><td>Unigene112910_All//8.00E-67//BQ410950</td><td></td><td>ko00010|Glycolysis / Gluconeogenesis//ko00051|Fructose and mannose metabolism//ko00561|Glycerolipid metabolism//ko00591|Linoleic acid metabolism//ko00650|Butanoate metabolism</td><td>GO:0008152//GO:0016747</td><td>nr +</td></tr>
<tr><td>Unigene112913_All</td><td>347</td><td>0.00</td><td>0.00</td><td>0.00</td><td>0.14</td><td>0.20</td><td>1.17</td><td>2.56</td><td>0.00</td><td>0.69</td><td>null</td><td>null</td><td></td><td>null</td><td>null</td><td></td><td>4.67E-01</td><td>9.60E-01</td><td></td><td>3.03E+00</td><td>6.03E-02</td><td></td><td>-1.13E+01</td><td>2.24E-06</td><td>Down</td><td>-1.89E+00</td><td>6.34E-03</td><td></td><td>Unigene112913_All//gi|297609225|ref|NP_001062856.2|//1.00E-15//Os09g0321200 [Oryza sativa Japonica Group] >gi|255678772|dbj|BAF24770.2| Os09g0321200 [Oryza sativa Japonica Group]</td><td></td><td></td><td></td><td></td><td>GO:0046872</td><td>nr -</td></tr>
<tr><td>Unigene112914_All</td><td>347</td><td>0.00</td><td>0.00</td><td>0.00</td><td>0.00</td><td>0.00</td><td>0.00</td><td>5.00</td><td>0.36</td><td>0.46</td><td>null</td><td>null</td><td></td><td>null</td><td>null</td><td></td><td>null</td><td>null</td><td></td><td>null</td><td>null</td><td></td><td>-3.79E+00</td><td>4.50E-09</td><td>Down</td><td>-3.44E+00</td><td>8.41E-09</td><td>Down</td><td>Unigene112914_All//sp|O15162|PLS1_HUMAN//3.00E-06//Phospholipid scramblase 1 OS=Homo sapiens GN=PLSCR1 PE=1 SV=1</td><td></td><td></td><td></td><td></td><td></td><td>swissprot +</td></tr>
<tr><td>Unigene112915_All</td><td>348</td><td>0.00</td><td>0.00</td><td>0.00</td><td>0.00</td><td>0.00</td><td>0.00</td><td>2.55</td><td>1.08</td><td>0.00</td><td>null</td><td>null</td><td></td><td>null</td><td>null</td><td></td><td>null</td><td>null</td><td></td><td>null</td><td>null</td><td></td><td>-1.24E+00</td><td>6.79E-02</td><td></td><td>-1.13E+01</td><td>1.10E-06</td><td>Down</td><td>Unigene112915_All//gi|195621288|gb|ACG32474.1|//2.00E-27//eukaryotic translation initiation factor 5A-1/2 [Zea mays]</td><td>Unigene112915_All//6.00E-27//AT1G26630| FBR12 (FUMONISIN B1-RESISTANT12); translation initiation factor</td><td></td><td></td><td></td><td>GO:0006448//GO:0006449//GO:0006952//GO:0008135//GO:0010038//GO:0012501//GO:0018205//GO:0043021//GO:0043231//GO:0051707</td><td>nr -</td></tr>
<tr><td>Unigene112916_All</td><td>348</td><td>0.00</td><td>0.00</td><td>0.00</td><td>0.00</td><td>0.00</td><td>0.00</td><td>2.55</td><td>0.60</td><td>0.11</td><td>null</td><td>null</td><td></td><td>null</td><td>null</td><td></td><td>null</td><td>null</td><td></td><td>null</td><td>null</td><td></td><td>-2.09E+00</td><td>4.71E-03</td><td></td><td>-4.48E+00</td><td>1.23E-05</td><td>Down</td><td></td><td></td><td></td><td></td><td></td><td></td><td>ESTscan +</td></tr>
<tr><td>Unigene112918_All</td><td>349</td><td>0.00</td><td>0.00</td><td>0.00</td><td>0.00</td><td>0.00</td><td>0.00</td><td>3.39</td><td>1.68</td><td>0.68</td><td>null</td><td>null</td><td></td><td>null</td><td>null</td><td></td><td>null</td><td>null</td><td></td><td>null</td><td>null</td><td></td><td>-1.02E+00</td><td>7.15E-02</td><td></td><td>-2.31E+00</td><td>2.04E-04</td><td>Down</td><td></td><td></td><td></td><td></td><td></td><td></td><td></td></tr>
<tr><td>Unigene112919_All</td><td>349</td><td>0.00</td><td>0.00</td><td>0.00</td><td>0.00</td><td>0.00</td><td>0.00</td><td>2.79</td><td>0.00</td><td>0.00</td><td>null</td><td>null</td><td></td><td>null</td><td>null</td><td></td><td>null</td><td>null</td><td></td><td>null</td><td>null</td><td></td><td>-1.14E+01</td><td>5.83E-07</td><td>Down</td><td>-1.14E+01</td><td>2.72E-07</td><td>Down</td><td>Unigene112919_All//gi|298715853|emb|CBJ28318.1|//5.00E-06//hypothetical protein [Ectocarpus siliculosus]</td><td></td><td></td><td></td><td></td><td></td><td>nr +</td></tr>
<tr><td>Unigene112920_All</td><td>351</td><td>0.00</td><td>0.00</td><td>0.00</td><td>0.00</td><td>0.00</td><td>0.00</td><td>3.61</td><td>1.91</td><td>0.23</td><td>null</td><td>null</td><td></td><td>null</td><td>null</td><td></td><td>null</td><td>null</td><td></td><td>null</td><td>null</td><td></td><td>-9.24E-01</td><td>8.68E-02</td><td></td><td>-3.99E+00</td><td>2.56E-07</td><td>Down</td><td>Unigene112920_All//gi|145324164|ref|NP_001077671.1|//3.00E-38//ARP1 (ARABIDOPSIS RIBOSOMAL PROTEIN 1); structural constituent of ribosome [Arabidopsis thaliana]</td><td>Unigene112920_All//1.00E-39//AT1G43170| ARP1 (ARABIDOPSIS RIBOSOMAL PROTEIN 1); structural constituent of ribosome</td><td>Unigene112920_All//4.00E-10//gi|11200269|gb|BF269274.1|BF269274</td><td></td><td>ko03010|Ribosome</td><td>GO:0003006//GO:0005198//GO:0010467//GO:0015934//GO:0016020//GO:0019219//GO:0030312//GO:0031981</td><td>nr -</td></tr>
<tr><td>Unigene112921_All</td><td>351</td><td>0.00</td><td>0.00</td><td>0.00</td><td>0.00</td><td>0.00</td><td>0.00</td><td>2.77</td><td>0.00</td><td>0.34</td><td>null</td><td>null</td><td></td><td>null</td><td>null</td><td></td><td>null</td><td>null</td><td></td><td>null</td><td>null</td><td></td><td>-1.14E+01</td><td>5.83E-07</td><td>Down</td><td>-3.02E+00</td><td>9.61E-05</td><td>Down</td><td></td><td></td><td></td><td></td><td></td><td></td><td>ESTscan -</td></tr>
<tr><td>Unigene112923_All</td><td>351</td><td>0.00</td><td>1.30</td><td>2.12</td><td>0.43</td><td>0.10</td><td>0.00</td><td>1.93</td><td>1.07</td><td>0.45</td><td>1.03E+01</td><td>7.44E-03</td><td></td><td>1.11E+01</td><td>3.28E-04</td><td>Up</td><td>-2.12E+00</td><td>3.87E-01</td><td></td><td>-8.73E+00</td><td>2.34E-01</td><td></td><td>-8.47E-01</td><td>2.88E-01</td><td></td><td>-2.09E+00</td><td>1.27E-02</td><td></td><td></td><td></td><td>Unigene112923_All//1.00E-22//TC145384</td><td></td><td></td><td></td><td></td></tr>
<tr><td>Unigene112925_All</td><td>353</td><td>0.00</td><td>0.00</td><td>0.00</td><td>0.00</td><td>0.00</td><td>0.00</td><td>2.04</td><td>0.83</td><td>0.11</td><td>null</td><td>null</td><td></td><td>null</td><td>null</td><td></td><td>null</td><td>null</td><td></td><td>null</td><td>null</td><td></td><td>-1.30E+00</td><td>9.45E-02</td><td></td><td>-4.17E+00</td><td>1.64E-04</td><td>Down</td><td>Unigene112925_All//gi|192910698|gb|ACF06457.1|//6.00E-22//60S ribosomal protein L35 [Elaeis guineensis]</td><td>Unigene112925_All//7.00E-18//AT5G02610| 60S ribosomal protein L35 (RPL35D)</td><td></td><td></td><td>ko03010|Ribosome</td><td>GO:0005198//GO:0010467//GO:0015934//GO:0016020//GO:0022613</td><td>nr -</td></tr>
<tr><td>Unigene112926_All</td><td>353</td><td>97.97</td><td>67.89</td><td>51.89</td><td>94.27</td><td>69.85</td><td>20.21</td><td>63.24</td><td>13.14</td><td>29.46</td><td>-5.29E-01</td><td>6.34E-09</td><td></td><td>-9.17E-01</td><td>2.99E-21</td><td></td><td>-4.33E-01</td><td>4.22E-07</td><td></td><td>-2.22E+00</td><td>6.46E-85</td><td>Down</td><td>-2.27E+00</td><td>9.71E-66</td><td>Down</td><td>-1.10E+00</td><td>1.94E-24</td><td>Down</td><td>Unigene112926_All//gi|87241442|gb|ABD33300.1|//5.00E-11//Gibberellin regulated protein [Medicago truncatula]</td><td>Unigene112926_All//1.00E-11//AT5G15230| GASA4 (GAST1 PROTEIN HOMOLOG 4)</td><td>Unigene112926_All//8.00E-135//TC175750</td><td></td><td></td><td></td><td>nr +</td></tr>
<tr><td>Unigene11293_All</td><td>1005</td><td>2.60</td><td>3.28</td><td>2.65</td><td>3.57</td><td>2.54</td><td>2.20</td><td>5.68</td><td>2.66</td><td>3.29</td><td>3.32E-01</td><td>3.58E-01</td><td></td><td>2.54E-02</td><td>9.67E-01</td><td></td><td>-4.93E-01</td><td>1.26E-01</td><td></td><td>-6.97E-01</td><td>3.23E-02</td><td></td><td>-1.09E+00</td><td>1.39E-06</td><td>Down</td><td>-7.88E-01</td><td>2.56E-04</td><td></td><td>Unigene11293_All//gi|28950779|gb|AAO63313.1|//3.00E-98//At4g16060 [Arabidopsis thaliana]</td><td>Unigene11293_All//5.00E-96//AT4G16060| unknown protein</td><td>Unigene11293_All//0.00E+00//TC177353</td><td></td><td></td><td>GO:0043231</td><td>nr -</td></tr>
<tr><td>Unigene112930_All</td><td>354</td><td>0.00</td><td>0.00</td><td>0.00</td><td>0.00</td><td>0.00</td><td>0.00</td><td>4.18</td><td>0.47</td><td>0.00</td><td>null</td><td>null</td><td></td><td>null</td><td>null</td><td></td><td>null</td><td>null</td><td></td><td>null</td><td>null</td><td></td><td>-3.15E+00</td><td>8.54E-07</td><td>Down</td><td>-1.20E+01</td><td>5.85E-11</td><td>Down</td><td></td><td></td><td>Unigene112930_All//7.00E-15//DR455073</td><td></td><td></td><td></td><td></td></tr>
<tr><td>Unigene112932_All</td><td>355</td><td>0.00</td><td>0.00</td><td>0.00</td><td>0.00</td><td>0.00</td><td>0.00</td><td>3.57</td><td>0.00</td><td>0.22</td><td>null</td><td>null</td><td></td><td>null</td><td>null</td><td></td><td>null</td><td>null</td><td></td><td>null</td><td>null</td><td></td><td>-1.18E+01</td><td>5.06E-09</td><td>Down</td><td>-3.99E+00</td><td>2.56E-07</td><td>Down</td><td></td><td></td><td></td><td></td><td></td><td></td><td>ESTscan -</td></tr>
<tr><td>Unigene112933_All</td><td>355</td><td>0.29</td><td>0.00</td><td>0.00</td><td>1.12</td><td>0.49</td><td>0.51</td><td>1.67</td><td>0.35</td><td>0.00</td><td>-8.20E+00</td><td>3.75E-01</td><td></td><td>-8.20E+00</td><td>3.97E-01</td><td></td><td>-1.21E+00</td><td>3.14E-01</td><td></td><td>-1.14E+00</td><td>3.80E-01</td><td></td><td>-2.24E+00</td><td>2.07E-02</td><td></td><td>-1.07E+01</td><td>1.41E-04</td><td>Down</td><td></td><td></td><td></td><td></td><td></td><td></td><td></td></tr>
<tr><td>Unigene112935_All</td><td>356</td><td>0.00</td><td>0.00</td><td>0.00</td><td>0.00</td><td>0.00</td><td>0.00</td><td>2.73</td><td>1.41</td><td>0.45</td><td>null</td><td>null</td><td></td><td>null</td><td>null</td><td></td><td>null</td><td>null</td><td></td><td>null</td><td>null</td><td></td><td>-9.55E-01</td><td>1.35E-01</td><td></td><td>-2.61E+00</td><td>3.35E-04</td><td>Down</td><td></td><td></td><td></td><td></td><td></td><td></td><td></td></tr>
<tr><td>Unigene112936_All</td><td>357</td><td>0.00</td><td>0.00</td><td>0.00</td><td>0.00</td><td>0.00</td><td>0.00</td><td>5.57</td><td>1.17</td><td>0.78</td><td>null</td><td>null</td><td></td><td>null</td><td>null</td><td></td><td>null</td><td>null</td><td></td><td>null</td><td>null</td><td></td><td>-2.25E+00</td><td>1.94E-06</td><td>Down</td><td>-2.83E+00</td><td>1.91E-08</td><td>Down</td><td></td><td></td><td></td><td></td><td></td><td></td><td></td></tr>
<tr><td>Unigene112938_All</td><td>357</td><td>0.00</td><td>0.00</td><td>0.00</td><td>0.00</td><td>0.00</td><td>0.00</td><td>1.78</td><td>0.82</td><td>0.00</td><td>null</td><td>null</td><td></td><td>null</td><td>null</td><td></td><td>null</td><td>null</td><td></td><td>null</td><td>null</td><td></td><td>-1.12E+00</td><td>1.80E-01</td><td></td><td>-1.08E+01</td><td>7.11E-05</td><td>Down</td><td></td><td></td><td>Unigene112938_All//3.00E-08//TC178941</td><td></td><td></td><td></td><td>ESTscan -</td></tr>
<tr><td>Unigene112939_All</td><td>358</td><td>0.00</td><td>0.00</td><td>0.00</td><td>0.28</td><td>0.00</td><td>0.00</td><td>3.07</td><td>0.35</td><td>0.89</td><td>null</td><td>null</td><td></td><td>null</td><td>null</td><td></td><td>-8.12E+00</td><td>3.16E-01</td><td></td><td>-8.12E+00</td><td>4.04E-01</td><td></td><td>-3.13E+00</td><td>3.47E-05</td><td>Down</td><td>-1.79E+00</td><td>3.08E-03</td><td></td><td>Unigene112939_All//gi|187940311|gb|ACD39386.1|//2.00E-53//NAC domain protein [Glycine max]</td><td>Unigene112939_All//3.00E-49//AT1G26870| FEZ (FEZ); transcription factor</td><td>Unigene112939_All//1.00E-10//DW224083</td><td>NAC</td><td></td><td>GO:0001071//GO:0003676//GO:0009725//GO:0009786//GO:0010468//GO:0017145//GO:0043231//GO:0048856</td><td>nr +</td></tr>
<tr><td>Unigene11294_All</td><td>1452</td><td>0.86</td><td>2.65</td><td>0.62</td><td>3.47</td><td>3.58</td><td>3.11</td><td>2.42</td><td>1.76</td><td>2.66</td><td>1.62E+00</td><td>1.62E-06</td><td>Up</td><td>-4.72E-01</td><td>4.42E-01</td><td></td><td>4.75E-02</td><td>8.80E-01</td><td></td><td>-1.56E-01</td><td>6.38E-01</td><td></td><td>-4.61E-01</td><td>1.29E-01</td><td></td><td>1.39E-01</td><td>6.43E-01</td><td></td><td>Unigene11294_All//gi|22328699|ref|NP_567482.2|//1.00E-172//lipase class 3 family protein [Arabidopsis thaliana]</td><td>Unigene11294_All//2.00E-147//AT4G16070| lipase class 3 family protein</td><td>Unigene11294_All//0.00E+00//gi|78338930|gb|DT559204.1|DT559204</td><td></td><td></td><td>GO:0004091//GO:0006629</td><td>nr +</td></tr>
<tr><td>Unigene112940_All</td><td>358</td><td>0.00</td><td>0.00</td><td>0.00</td><td>0.00</td><td>0.00</td><td>0.00</td><td>2.36</td><td>0.35</td><td>0.00</td><td>null</td><td>null</td><td></td><td>null</td><td>null</td><td></td><td>null</td><td>null</td><td></td><td>null</td><td>null</td><td></td><td>-2.75E+00</td><td>9.73E-04</td><td>Down</td><td>-1.12E+01</td><td>2.21E-06</td><td>Down</td><td>Unigene112940_All//gi|301111414|ref|XP_002904786.1|//1.00E-41//ribosomal protein L27 [Phytophthora infestans T30-4] >gi|66270187|gb|AAY43423.1| ribosomal protein L27 [Phytophthora infestans] >gi|262095116|gb|EEY53168.1| ribosomal protein L27 [Phytophthora infestans T30-4]</td><td>Unigene112940_All//2.00E-39//AT1G70600| structural constituent of ribosome</td><td>Unigene112940_All//7.00E-12//DW504561</td><td></td><td>ko03010|Ribosome</td><td>GO:0005198//GO:0010467//GO:0030529</td><td>nr -</td></tr>
<tr><td>Unigene112941_All</td><td>358</td><td>1.02</td><td>1.13</td><td>0.60</td><td>1.11</td><td>0.19</td><td>0.00</td><td>4.96</td><td>1.17</td><td>0.89</td><td>1.47E-01</td><td>8.98E-01</td><td></td><td>-7.82E-01</td><td>5.53E-01</td><td></td><td>-2.53E+00</td><td>5.80E-02</td><td></td><td>-1.01E+01</td><td>1.11E-02</td><td></td><td>-2.09E+00</td><td>2.21E-05</td><td>Down</td><td>-2.48E+00</td><td>1.05E-06</td><td>Down</td><td>Unigene112941_All//gi|31559433|emb|CAD33533.1|//5.00E-14//major latex protein homologue [Datisca glomerata]</td><td>Unigene112941_All//7.00E-15//AT1G23120| major latex protein-related / MLP-related</td><td>Unigene112941_All//0.00E+00//gi|109883067|gb|DW512037.1|DW512037</td><td></td><td></td><td></td><td>nr +</td></tr>
<tr><td>Unigene112944_All</td><td>360</td><td>0.00</td><td>0.00</td><td>0.30</td><td>0.00</td><td>0.00</td><td>0.00</td><td>3.76</td><td>0.58</td><td>0.33</td><td>null</td><td>null</td><td></td><td>8.21E+00</td><td>4.01E-01</td><td></td><td>null</td><td>null</td><td></td><td>null</td><td>null</td><td></td><td>-2.69E+00</td><td>1.77E-05</td><td>Down</td><td>-3.50E+00</td><td>4.24E-07</td><td>Down</td><td>Unigene112944_All//gi|119507465|dbj|BAF42040.1|//2.00E-52//pectin methylesterase 3 [Pyrus communis]</td><td>Unigene112944_All//1.00E-49//AT4G02330| ATPMEPCRB; pectinesterase</td><td>Unigene112944_All//3.00E-60//TC138774</td><td></td><td>ko00500|Starch and sucrose metabolism//ko00040|Pentose and glucuronate interconversions</td><td>GO:0004091//GO:0005618//GO:0030234//GO:0071555</td><td>nr -</td></tr>
<tr><td>Unigene112947_All</td><td>362</td><td>0.00</td><td>0.00</td><td>0.00</td><td>0.00</td><td>0.00</td><td>0.00</td><td>2.57</td><td>0.00</td><td>0.00</td><td>null</td><td>null</td><td></td><td>null</td><td>null</td><td></td><td>null</td><td>null</td><td></td><td>null</td><td>null</td><td></td><td>-1.13E+01</td><td>1.14E-06</td><td>Down</td><td>-1.13E+01</td><td>5.48E-07</td><td>Down</td><td></td><td></td><td></td><td></td><td></td><td></td><td>ESTscan -</td></tr>
<tr><td>Unigene112949_All</td><td>362</td><td>0.00</td><td>0.00</td><td>0.00</td><td>0.00</td><td>0.00</td><td>0.00</td><td>3.15</td><td>0.35</td><td>0.00</td><td>null</td><td>null</td><td></td><td>null</td><td>null</td><td></td><td>null</td><td>null</td><td></td><td>null</td><td>null</td><td></td><td>-3.19E+00</td><td>1.96E-05</td><td>Down</td><td>-1.16E+01</td><td>1.65E-08</td><td>Down</td><td>Unigene112949_All//gi|226510343|ref|NP_001151730.1|//4.00E-46//40S ribosomal protein S15 [Zea mays] >gi|195649383|gb|ACG44159.1| 40S ribosomal protein S15 [Zea mays]</td><td>Unigene112949_All//6.00E-36//AT5G09500| 40S ribosomal protein S15 (RPS15C)</td><td>Unigene112949_All//2.00E-09//BG444152</td><td></td><td>ko03010|Ribosome</td><td>GO:0003676//GO:0005198//GO:0010467//GO:0033279</td><td>nr -</td></tr>
<tr><td>Unigene11295_All</td><td>759</td><td>0.69</td><td>1.13</td><td>1.05</td><td>0.98</td><td>1.54</td><td>2.26</td><td>2.73</td><td>0.72</td><td>1.47</td><td>7.19E-01</td><td>3.48E-01</td><td></td><td>6.11E-01</td><td>4.41E-01</td><td></td><td>6.48E-01</td><td>3.26E-01</td><td></td><td>1.20E+00</td><td>1.97E-02</td><td></td><td>-1.93E+00</td><td>1.21E-05</td><td>Down</td><td>-8.93E-01</td><td>2.02E-02</td><td></td><td>Unigene11295_All//gi|115463405|ref|NP_001055302.1|//8.00E-21//Os05g0358400 [Oryza sativa Japonica Group] >gi|113578853|dbj|BAF17216.1| Os05g0358400 [Oryza sativa Japonica Group]</td><td>Unigene11295_All//1.00E-16//AT4G16060| unknown protein</td><td>Unigene11295_All//3.00E-90//TC177353</td><td></td><td></td><td>GO:0043231</td><td>nr -</td></tr>
<tr><td>Unigene112950_All</td><td>365</td><td>0.00</td><td>0.69</td><td>1.02</td><td>0.55</td><td>0.19</td><td>0.87</td><td>3.01</td><td>0.69</td><td>0.22</td><td>9.44E+00</td><td>7.88E-02</td><td></td><td>1.00E+01</td><td>2.50E-02</td><td></td><td>-1.53E+00</td><td>4.16E-01</td><td></td><td>6.66E-01</td><td>6.48E-01</td><td></td><td>-2.13E+00</td><td>1.11E-03</td><td></td><td>-3.79E+00</td><td>3.27E-06</td><td>Down</td><td></td><td></td><td>Unigene112950_All//4.00E-07//TC164828</td><td></td><td></td><td></td><td></td></tr>
<tr><td>Unigene112952_All</td><td>368</td><td>0.00</td><td>0.00</td><td>0.00</td><td>0.54</td><td>0.00</td><td>0.00</td><td>1.84</td><td>0.00</td><td>0.22</td><td>null</td><td>null</td><td></td><td>null</td><td>null</td><td></td><td>-9.08E+00</td><td>8.46E-02</td><td></td><td>-9.08E+00</td><td>1.34E-01</td><td></td><td>-1.08E+01</td><td>6.34E-05</td><td>Down</td><td>-3.09E+00</td><td>1.45E-03</td><td></td><td>Unigene112952_All//gi|224078614|ref|XP_002305575.1|//6.00E-64//predicted protein [Populus trichocarpa] >gi|222848539|gb|EEE86086.1| predicted protein [Populus trichocarpa]</td><td></td><td></td><td></td><td></td><td></td><td>nr -</td></tr>
<tr><td>Unigene112953_All</td><td>368</td><td>0.28</td><td>0.00</td><td>0.00</td><td>0.27</td><td>0.09</td><td>0.12</td><td>2.30</td><td>0.11</td><td>0.87</td><td>-8.15E+00</td><td>3.75E-01</td><td></td><td>-8.15E+00</td><td>3.97E-01</td><td></td><td>-1.53E+00</td><td>5.88E-01</td><td></td><td>-1.14E+00</td><td>6.78E-01</td><td></td><td>-4.34E+00</td><td>4.55E-05</td><td>Down</td><td>-1.41E+00</td><td>3.70E-02</td><td></td><td>Unigene112953_All//gi|15233503|ref|NP_193195.1|//1.00E-30//emb2739 (embryo defective 2739) [Arabidopsis thaliana]</td><td>Unigene112953_All//5.00E-32//AT4G14590| emb2739 (embryo defective 2739)</td><td>Unigene112953_All//3.00E-91//ES802105</td><td></td><td></td><td>GO:0003006</td><td>nr -</td></tr>
<tr><td>Unigene112954_All</td><td>369</td><td>0.00</td><td>0.00</td><td>0.00</td><td>0.00</td><td>0.00</td><td>0.00</td><td>4.93</td><td>1.25</td><td>0.43</td><td>null</td><td>null</td><td></td><td>null</td><td>null</td><td></td><td>null</td><td>null</td><td></td><td>null</td><td>null</td><td></td><td>-1.98E+00</td><td>3.39E-05</td><td>Down</td><td>-3.51E+00</td><td>2.43E-09</td><td>Down</td><td></td><td></td><td></td><td></td><td></td><td></td><td></td></tr>
<tr><td>Unigene112955_All</td><td>369</td><td>0.43</td><td>0.82</td><td>0.29</td><td>1.49</td><td>0.65</td><td>0.61</td><td>3.21</td><td>0.57</td><td>1.30</td><td>9.54E-01</td><td>5.04E-01</td><td></td><td>-5.60E-01</td><td>7.76E-01</td><td></td><td>-1.18E+00</td><td>2.25E-01</td><td></td><td>-1.28E+00</td><td>2.24E-01</td><td></td><td>-2.50E+00</td><td>1.47E-04</td><td>Down</td><td>-1.31E+00</td><td>1.69E-02</td><td></td><td>Unigene112955_All//gi|22327146|ref|NP_198205.2|//2.00E-55//FRS10 (FAR1-related sequence 10); zinc ion binding [Arabidopsis thaliana] >gi|257051024|sp|Q9LKR4.2|FRS10_ARATH RecName: Full=Putative protein FAR1-RELATED SEQUENCE 10</td><td>Unigene112955_All//1.00E-56//AT5G28530| FRS10 (FAR1-related sequence 10); zinc ion binding</td><td></td><td></td><td></td><td>GO:0046914</td><td>nr +</td></tr>
<tr><td>Unigene112958_All</td><td>371</td><td>0.00</td><td>0.00</td><td>0.00</td><td>0.00</td><td>0.00</td><td>0.00</td><td>2.05</td><td>0.11</td><td>0.00</td><td>null</td><td>null</td><td></td><td>null</td><td>null</td><td></td><td>null</td><td>null</td><td></td><td>null</td><td>null</td><td></td><td>-4.19E+00</td><td>1.57E-04</td><td>Down</td><td>-1.10E+01</td><td>8.88E-06</td><td>Down</td><td>Unigene112958_All//gi|116057156|emb|CAL51583.1|//1.00E-36//ribosomal L9-like protein (ISS) [Ostreococcus tauri]</td><td>Unigene112958_All//3.00E-33//AT1G33140| PGY2 (PIGGYBACK2); structural constituent of ribosome</td><td>Unigene112958_All//5.00E-13//TC152661</td><td></td><td>ko03010|Ribosome</td><td>GO:0003723//GO:0005198//GO:0010467//GO:0030529</td><td>nr +</td></tr>
<tr><td>Unigene112959_All</td><td>372</td><td>0.14</td><td>0.00</td><td>0.00</td><td>0.00</td><td>0.37</td><td>0.73</td><td>2.73</td><td>1.12</td><td>0.54</td><td>-7.14E+00</td><td>6.17E-01</td><td></td><td>-7.14E+00</td><td>6.30E-01</td><td></td><td>8.53E+00</td><td>3.20E-01</td><td></td><td>9.51E+00</td><td>7.04E-02</td><td></td><td>-1.28E+00</td><td>4.06E-02</td><td></td><td>-2.35E+00</td><td>5.84E-04</td><td>Down</td><td></td><td></td><td></td><td></td><td></td><td></td><td></td></tr>
<tr><td>Unigene112961_All</td><td>373</td><td>0.00</td><td>0.00</td><td>0.00</td><td>0.00</td><td>0.46</td><td>0.00</td><td>5.10</td><td>0.78</td><td>0.85</td><td>null</td><td>null</td><td></td><td>null</td><td>null</td><td></td><td>8.85E+00</td><td>2.27E-01</td><td></td><td>null</td><td>null</td><td></td><td>-2.70E+00</td><td>1.87E-07</td><td>Down</td><td>-2.58E+00</td><td>2.06E-07</td><td>Down</td><td></td><td></td><td>Unigene112961_All//3.00E-125//TC172393</td><td></td><td></td><td></td><td></td></tr>
<tr><td>Unigene112962_All</td><td>373</td><td>0.00</td><td>0.00</td><td>0.00</td><td>0.40</td><td>0.46</td><td>0.00</td><td>3.51</td><td>0.90</td><td>0.75</td><td>null</td><td>null</td><td></td><td>null</td><td>null</td><td></td><td>2.04E-01</td><td>9.50E-01</td><td></td><td>-8.65E+00</td><td>2.34E-01</td><td></td><td>-1.97E+00</td><td>6.34E-04</td><td>Down</td><td>-2.23E+00</td><td>1.19E-04</td><td>Down</td><td></td><td></td><td></td><td></td><td></td><td></td><td></td></tr>
<tr><td>Unigene112963_All</td><td>375</td><td>0.00</td><td>0.00</td><td>0.57</td><td>0.00</td><td>0.00</td><td>0.00</td><td>2.48</td><td>0.22</td><td>0.00</td><td>null</td><td>null</td><td></td><td>9.15E+00</td><td>1.42E-01</td><td></td><td>null</td><td>null</td><td></td><td>null</td><td>null</td><td></td><td>-3.48E+00</td><td>7.79E-05</td><td>Down</td><td>-1.13E+01</td><td>5.48E-07</td><td>Down</td><td>Unigene112963_All//gi|225461437|ref|XP_002282367.1|//1.00E-08//PREDICTED: hypothetical protein [Vitis vinifera]</td><td></td><td>Unigene112963_All//3.00E-73//TC147141</td><td></td><td></td><td></td><td>nr +</td></tr>
<tr><td>Unigene112964_All</td><td>375</td><td>0.14</td><td>0.27</td><td>0.00</td><td>0.53</td><td>0.73</td><td>0.60</td><td>2.03</td><td>0.67</td><td>0.21</td><td>9.54E-01</td><td>7.27E-01</td><td></td><td>-7.12E+00</td><td>6.30E-01</td><td></td><td>4.67E-01</td><td>8.17E-01</td><td></td><td>1.80E-01</td><td>9.35E-01</td><td></td><td>-1.60E+00</td><td>3.70E-02</td><td></td><td>-3.26E+00</td><td>4.48E-04</td><td>Down</td><td>Unigene112964_All//gi|115461308|ref|NP_001054254.1|//2.00E-24//Os04g0676200 [Oryza sativa Japonica Group] >gi|113565825|dbj|BAF16168.1| Os04g0676200 [Oryza sativa Japonica Group]</td><td>Unigene112964_All//4.00E-16//AT1G03540| pentatricopeptide (PPR) repeat-containing protein</td><td></td><td></td><td></td><td>GO:0043231</td><td>nr +</td></tr>
<tr><td>Unigene112965_All</td><td>375</td><td>0.00</td><td>0.00</td><td>0.00</td><td>0.00</td><td>0.00</td><td>0.00</td><td>4.51</td><td>0.22</td><td>1.81</td><td>null</td><td>null</td><td></td><td>null</td><td>null</td><td></td><td>null</td><td>null</td><td></td><td>null</td><td>null</td><td></td><td>-4.34E+00</td><td>1.16E-09</td><td>Down</td><td>-1.32E+00</td><td>3.08E-03</td><td></td><td></td><td></td><td></td><td></td><td></td><td></td><td>ESTscan +</td></tr>
<tr><td>Unigene112966_All</td><td>376</td><td>0.56</td><td>0.27</td><td>0.00</td><td>0.00</td><td>0.27</td><td>0.12</td><td>4.27</td><td>0.89</td><td>0.42</td><td>-1.05E+00</td><td>5.65E-01</td><td></td><td>-9.12E+00</td><td>1.46E-01</td><td></td><td>8.10E+00</td><td>4.58E-01</td><td></td><td>6.91E+00</td><td>7.01E-01</td><td></td><td>-2.26E+00</td><td>2.23E-05</td><td>Down</td><td>-3.33E+00</td><td>5.33E-08</td><td>Down</td><td></td><td></td><td>Unigene112966_All//4.00E-07//TC164796</td><td></td><td></td><td></td><td></td></tr>
<tr><td>Unigene112968_All</td><td>377</td><td>0.00</td><td>0.00</td><td>0.00</td><td>0.00</td><td>0.00</td><td>0.00</td><td>5.27</td><td>0.11</td><td>0.53</td><td>null</td><td>null</td><td></td><td>null</td><td>null</td><td></td><td>null</td><td>null</td><td></td><td>null</td><td>null</td><td></td><td>-5.57E+00</td><td>1.08E-12</td><td>Down</td><td>-3.32E+00</td><td>1.06E-09</td><td>Down</td><td></td><td></td><td>Unigene112968_All//5.00E-13//TC149394</td><td></td><td></td><td></td><td></td></tr>
<tr><td>Unigene112971_All</td><td>382</td><td>0.00</td><td>0.00</td><td>0.00</td><td>0.00</td><td>0.00</td><td>0.00</td><td>2.10</td><td>1.53</td><td>0.31</td><td>null</td><td>null</td><td></td><td>null</td><td>null</td><td></td><td>null</td><td>null</td><td></td><td>null</td><td>null</td><td></td><td>-4.57E-01</td><td>5.36E-01</td><td></td><td>-2.75E+00</td><td>9.43E-04</td><td>Down</td><td>Unigene112971_All//gi|164449276|gb|ABY56104.1|//8.00E-51//ribosomal protein [Cucumis sativus]</td><td>Unigene112971_All//1.00E-51//AT3G04400| emb2171 (embryo defective 2171); structural constituent of ribosome</td><td>Unigene112971_All//4.00E-07//TC164532</td><td></td><td>ko03010|Ribosome</td><td>GO:0003006//GO:0005198//GO:0010467//GO:0015934//GO:0031981</td><td>nr +</td></tr>
</table>

Unigene112972_All	382	0.00	0.00	0.00	0.00	0.00	0.00	2.55	0.22	0.00	null	null		null	null		null	null		null	null		-3.54E+00	4.32E-05	Down	-1.13E+01	2.72E-07	Down	Unigene112972_All//gi|159471131|ref|XP_001693710.1|//2.00E-44//ribosomal protein S3a, component of cytosolic 80S ribosome and 40S small subunit [Chlamydomonas reinhardtii] >gi|269969596|sp|A8HS48.1|RS3A_CHLRE RecName: Full=40S ribosomal protein S3a >gi|158283213|gb|EDP08964.1| ribosomal protein S3a [Chlamydomonas reinhardtii]	Unigene112972_All//2.00E-39//AT4G34670| 40S ribosomal protein S3A (RPS3aB)			ko03010|Ribosome	GO:0005198//GO:0010467//GO:0030529	nr +
Unigene112973_All	384	0.00	0.00	0.00	0.00	0.00	0.00	7.27	1.52	0.52	null	null		null	null		null	null		null	null		-2.25E+00	8.87E-09	Down	-3.81E+00	6.99E-15	Down	Unigene112973_All//gi|74325167|ref|YP_316586.1|//9.00E-31//cytochrome oxidase subunit I [Thalassiosira pseudonana] >gi|74100233|gb|AAZ99394.1| cytochrome oxidase subunit I [Thalassiosira pseudonana]	Unigene112973_All//3.00E-19//ATMG01360| cytochrome c oxidase subunit 1			ko00190|Oxidative phosphorylation	GO:0005506//GO:0006091//GO:0016491//GO:0043231//GO:0044425	nr -
Unigene112975_All	385	0.00	0.00	0.00	0.00	0.00	0.00	1.65	0.33	0.00	null	null		null	null		null	null		null	null		-2.34E+00	1.27E-02		-1.07E+01	7.11E-05	Down	Unigene112975_All//gi|57471702|gb|AAW50982.1|//8.00E-51//ribosomal protein L10A [Triticum aestivum]	Unigene112975_All//1.00E-38//AT5G22440| 60S ribosomal protein L10A (RPL10aC)	Unigene112975_All//1.00E-07//TC157648		ko03010|Ribosome	GO:0003676//GO:0005198//GO:0010467//GO:0015934//GO:0016020	nr -
Unigene112976_All	386	0.00	0.00	0.00	0.00	0.00	0.00	2.41	1.73	0.41	null	null		null	null		null	null		null	null		-4.76E-01	4.76E-01		-2.55E+00	5.79E-04	Down			Unigene112976_All//7.00E-37//CO075999				
Unigene112977_All	386	0.81	0.00	0.14	1.55	0.36	0.70	2.52	0.00	0.93	-9.67E+00	3.66E-02		-2.56E+00	1.59E-01		-2.12E+00	3.06E-02		-1.14E+00	2.43E-01		-1.13E+01	5.83E-07	Down	-1.44E+00	2.09E-02		Unigene112977_All//gi|297606758|ref|NP_001058915.2|//3.00E-11//Os07g0154400 [Oryza sativa Japonica Group] >gi|255677527|dbj|BAF20829.2| Os07g0154400 [Oryza sativa Japonica Group]	Unigene112977_All//5.00E-20//AT4G14970| unknown protein					nr -
Unigene112978_All	387	0.00	0.00	0.00	0.00	0.00	0.00	3.50	0.32	1.13	null	null		null	null		null	null		null	null		-3.43E+00	1.05E-06	Down	-1.63E+00	1.95E-03				Unigene112978_All//1.00E-13//TC134836				
Unigene112980_All	388	0.00	0.00	0.00	0.77	0.53	0.00	1.74	0.00	0.31	null	null		null	null		-5.33E-01	6.79E-01		-9.59E+00	4.01E-02		-1.08E+01	6.34E-05	Down	-2.50E+00	4.78E-03		Unigene112980_All//sp|Q8CF90|MAFA_MOUSE//7.00E-06//Transcription factor MafA OS=Mus musculus GN=Mafa PE=1 SV=1		Unigene112980_All//6.00E-65//TC151886				swissprot -
Unigene112981_All	390	0.00	0.00	0.00	0.00	0.00	0.00	6.29	0.21	0.20	null	null		null	null		null	null		null	null		-4.87E+00	1.04E-14	Down	-4.94E+00	2.14E-15	Down	Unigene112981_All//gi|300258120|gb|EFJ42360.1|//4.00E-45//component of cytosolic 80S ribosome and 60S large subunit [Volvox carteri f. nagariensis]	Unigene112981_All//1.00E-42//AT1G23290| RPL27AB; structural constituent of ribosome	Unigene112981_All//1.00E-13//TC169611		ko03010|Ribosome	GO:0005198//GO:0010467//GO:0030529	nr -
Unigene112982_All	391	0.00	0.00	0.00	0.00	0.00	0.00	3.14	0.43	0.20	null	null		null	null		null	null		null	null		-2.87E+00	2.54E-05	Down	-3.94E+00	4.86E-07	Down	Unigene112982_All//gi|301119741|ref|XP_002907598.1|//1.00E-29//60S ribosomal protein L23a [Phytophthora infestans T30-4] >gi|262106110|gb|EEY64162.1| 60S ribosomal protein L23a [Phytophthora infestans T30-4]	Unigene112982_All//1.00E-28//AT3G55280| RPL23AB (RIBOSOMAL PROTEIN L23AB); RNA binding / nucleotide binding / structural constituent of ribosome	Unigene112982_All//7.00E-09//BG441496		ko03010|Ribosome	GO:0001071//GO:0003723//GO:0005198//GO:0006351//GO:0030529//GO:0043231	nr -
Unigene112983_All	392	0.00	0.00	0.00	0.00	0.00	0.00	3.34	0.43	0.00	null	null		null	null		null	null		null	null		-2.97E+00	8.34E-06	Down	-1.17E+01	9.85E-10	Down							
Unigene112984_All	398	0.00	0.00	0.00	0.00	0.00	0.00	32.83	1.47	0.00	null	null		null	null		null	null		null	null		-4.48E+00	4.33E-73	Down	-1.50E+01	2.58E-96	Down	Unigene112984_All//gi|224125106|ref|XP_002329895.1|//3.00E-28//cytochrome P450 [Populus trichocarpa] >gi|222871132|gb|EEF08263.1| cytochrome P450 [Populus trichocarpa]	Unigene112984_All//2.00E-26//AT4G22690| CYP706A1; electron carrier/ heme binding / iron ion binding / monooxygenase/ oxygen binding	Unigene112984_All//2.00E-86//TC137030		ko00940|Phenylpropanoid biosynthesis//ko00945|Stilbenoid, diarylheptanoid and gingerol biosynthesis//ko00903|Limonene and pinene degradation//ko00941|Flavonoid biosynthesis//ko00360|Phenylalanine metabolism//ko00944|Flavone and flavonol biosynthesis	GO:0004497//GO:0005506//GO:0016020	nr +
Unigene112985_All	399	0.00	0.00	0.00	0.00	0.00	0.00	4.45	0.73	0.00	null	null		null	null		null	null		null	null		-2.60E+00	9.40E-07	Down	-1.21E+01	4.12E-13	Down			Unigene112985_All//8.00E-18//ES794483				
Unigene112986_All	399	0.00	0.00	0.00	0.00	0.00	0.00	4.87	1.05	0.70	null	null		null	null		null	null		null	null		-2.22E+00	3.19E-06	Down	-2.80E+00	3.40E-08	Down							
Unigene112987_All	399	0.00	0.00	0.00	0.00	0.00	0.00	3.28	0.21	0.00	null	null		null	null		null	null		null	null		-3.97E+00	3.35E-07	Down	-1.17E+01	9.85E-10	Down							ESTscan -
Unigene112988_All	402	0.00	0.00	0.00	0.00	0.00	0.00	3.68	2.29	0.20	null	null		null	null		null	null		null	null		-6.87E-01	1.64E-01		-4.22E+00	1.00E-08	Down			Unigene112988_All//9.00E-21//ES825902				
Unigene112990_All	402	0.00	0.00	0.00	0.00	0.00	0.00	3.58	0.73	0.00	null	null		null	null		null	null		null	null		-2.30E+00	5.89E-05	Down	-1.18E+01	1.19E-10	Down	Unigene112990_All//gi|255977225|dbj|BAH97104.1|//1.00E-57//heat shock protein Hsp70 [Glaucocystis nostochinearum]	Unigene112990_All//6.00E-55//AT1G16030| Hsp70b (heat shock protein 70B); ATP binding	Unigene112990_All//9.00E-21//TC169169		ko03040|Spliceosome//ko04141|Protein processing in endoplasmic reticulum//ko04144|Endocytosis	GO:0008152//GO:0016628//GO:0032559//GO:0050896	nr -
Unigene112991_All	403	0.00	0.00	0.00	0.00	0.00	0.00	1.78	0.00	0.69	null	null		null	null		null	null		null	null		-1.08E+01	3.27E-05	Down	-1.37E+00	6.51E-02		Unigene112991_All//gi|55831356|gb|AAV66577.1|//2.00E-29//lipase [Ricinus communis]	Unigene112991_All//2.00E-25//AT5G42930| triacylglycerol lipase				GO:0016787	nr +
Unigene112992_All	403	0.00	0.00	0.00	0.00	0.00	0.00	1.57	0.00	0.00	null	null		null	null		null	null		null	null		-1.06E+01	1.23E-04	Down	-1.06E+01	7.11E-05	Down	Unigene112992_All//gi|4139170|gb|AAD03711.1|//5.00E-46//elongation translation factor 1 alpha [Cyanophora paradoxa]	Unigene112992_All//8.00E-44//AT5G60390| elongation factor 1-alpha / EF-1-alpha				GO:0006412//GO:0008135//GO:0017111//GO:0032561//GO:0044424	nr +
Unigene112994_All	406	0.00	0.00	0.00	0.00	0.00	0.00	2.92	0.00	0.00	null	null		null	null		null	null		null	null		-1.15E+01	1.97E-08	Down	-1.15E+01	8.15E-09	Down	Unigene112994_All//gi|301101263|ref|XP_002899720.1|//9.00E-37//40S ribosomal protein S4 [Phytophthora infestans T30-4] >gi|262102722|gb|EEY60774.1| 40S ribosomal protein S4 [Phytophthora infestans T30-4]	Unigene112994_All//1.00E-35//AT5G58420| 40S ribosomal protein S4 (RPS4D)			ko03010|Ribosome	GO:0003676//GO:0005198//GO:0010467//GO:0030529	nr +
Unigene112995_All	408	0.00	0.00	0.00	0.00	0.00	0.00	7.36	0.82	0.68	null	null		null	null		null	null		null	null		-3.17E+00	3.04E-13	Down	-3.43E+00	1.05E-14	Down	Unigene112995_All//gi|225429199|ref|XP_002276272.1|//3.00E-06//PREDICTED: hypothetical protein [Vitis vinifera] >gi|147769903|emb|CAN65538.1| hypothetical protein [Vitis vinifera] >gi|297736408|emb|CBI25131.3| unnamed protein product [Vitis vinifera]	Unigene112995_All//9.00E-06//AT3G49780| ATPSK4 (PHYTOSULFOKINE 4 PRECURSOR); growth factor					nr -
Unigene112996_All	408	0.00	0.00	0.00	0.00	0.00	0.00	2.59	0.20	0.20	null	null		null	null		null	null		null	null		-3.66E+00	1.31E-05	Down	-3.73E+00	6.14E-06	Down							
Unigene112997_All	408	0.00	0.00	0.00	0.24	0.00	0.00	6.63	1.64	1.07	null	null		null	null		-7.93E+00	3.16E-01		-7.93E+00	4.04E-01		-2.02E+00	1.61E-07	Down	-2.63E+00	2.01E-10	Down							
Unigene112999_All	411	0.00	0.00	0.00	0.00	0.00	0.00	3.39	0.00	0.00	null	null		null	null		null	null		null	null		-1.17E+01	6.58E-10	Down	-1.17E+01	2.40E-10	Down	Unigene112999_All//gi|54039313|sp|P62302.1|RS13_SOYBN//7.00E-44//RecName: Full=40S ribosomal protein S13 >gi|44662862|gb|AAS47510.1| ribosomal protein S13 [Glycine max] >gi|161367348|gb|ABX71080.1| ribosomal protein S13 [Glycine max]	Unigene112999_All//2.00E-43//AT4G00100| ATRPS13A (ARABIDOPSIS THALIANA RIBOSOMAL PROTEIN S13A); structural constituent of ribosome	Unigene112999_All//5.00E-10//TC137925		ko03010|Ribosome	GO:0000904//GO:0005198//GO:0009887//GO:0010467//GO:0015935//GO:0031981//GO:0033205	nr -
Unigene113001_All	411	0.00	0.00	0.00	0.97	0.59	0.44	4.01	0.10	0.68	null	null		null	null		-7.25E-01	5.47E-01		-1.14E+00	3.80E-01		-5.30E+00	2.16E-10	Down	-2.56E+00	1.72E-06	Down	Unigene113001_All//gi|115470965|ref|NP_001059081.1|//8.00E-43//Os07g0188700 [Oryza sativa Japonica Group] >gi|113610617|dbj|BAF20995.1| Os07g0188700 [Oryza sativa Japonica Group]	Unigene113001_All//1.00E-39//AT5G03795| LOCATED IN: membrane; EXPRESSED IN: embryo, sepal, flower; EXPRESSED DURING: C globular stage, petal differentiation and expansion stage; CONTAINS InterPro DOMAIN/s: Exostosin-like (InterPro:IPR004263); BEST Arabidopsis thaliana protein match is: exostosin family protein (TAIR:AT3G07620.1); Has 866 Blast hits to 860 proteins in 87 species: Archae - 0; Bacteria - 9; Metazoa - 265; Fungi - 4; Plants - 504; Viruses - 0; Other Eukaryotes - 84 (source: NCBI BLink).				GO:0008375//GO:0044464	nr +
Unigene113002_All	412	0.00	0.00	0.00	0.00	0.00	0.00	4.10	1.22	0.00	null	null		null	null		null	null		null	null		-1.75E+00	2.90E-04	Down	-1.20E+01	1.70E-12	Down	Unigene113002_All//gi|192910698|gb|ACF06457.1|//5.00E-37//60S ribosomal protein L35 [Elaeis guineensis]	Unigene113002_All//6.00E-36//AT3G55170| 60S ribosomal protein L35 (RPL35C)			ko03010|Ribosome	GO:0005198//GO:0010467//GO:0015934//GO:0022613//GO:0031981	nr +
Unigene113003_All	414	0.00	0.00	0.00	0.00	0.00	0.00	3.68	0.00	0.10	null	null		null	null		null	null		null	null		-1.18E+01	8.51E-11	Down	-5.26E+00	5.39E-10	Down							
Unigene113004_All	418	0.00	0.00	0.00	0.00	0.00	0.00	1.62	0.00	0.00	null	null		null	null		null	null		null	null		-1.07E+01	6.34E-05	Down	-1.07E+01	3.56E-05	Down							ESTscan +
Unigene113005_All	418	0.00	0.00	0.00	0.00	0.00	0.00	4.25	0.10	0.38	null	null		null	null		null	null		null	null		-5.41E+00	2.97E-11	Down	-3.48E+00	4.53E-09	Down							ESTscan +
Unigene113006_All	418	0.00	0.00	0.00	0.00	0.00	0.00	6.07	2.10	0.57	null	null		null	null		null	null		null	null		-1.53E+00	4.18E-05	Down	-3.41E+00	1.81E-12	Down	Unigene113006_All//gi|300262307|gb|EFJ46514.1|//3.00E-17//hypothetical protein VOLCADRAFT_93060 [Volvox carteri f. nagariensis]						nr +
Unigene113007_All	419	0.12	0.00	0.00	0.24	0.25	0.00	3.33	0.20	0.48	-6.96E+00	6.17E-01		-6.96E+00	6.30E-01		5.24E-02	9.68E-01		-7.89E+00	4.04E-01		-4.06E+00	9.64E-08	Down	-2.81E+00	4.25E-06	Down	Unigene113007_All//gi|240254545|ref|NP_180360.4|//2.00E-46//binding [Arabidopsis thaliana]	Unigene113007_All//1.00E-47//AT2G27930| binding	Unigene113007_All//7.00E-111//TC179204	PLATZ			nr -
Unigene113009_All	419	0.50	0.73	1.27	0.36	0.82	0.00	2.72	1.10	0.29	5.39E-01	6.72E-01		1.35E+00	2.25E-01		1.20E+00	4.26E-01		-8.48E+00	2.34E-01		-1.31E+00	2.45E-02		-3.26E+00	8.94E-06	Down			Unigene113009_All//1.00E-127//TC163926				
Unigene113010_All	421	0.00	0.00	0.00	0.47	0.16	0.21	3.31	0.79	0.47	null	null		null	null		-1.53E+00	4.16E-01		-1.14E+00	5.70E-01		-2.06E+00	2.51E-04	Down	-2.81E+00	4.25E-06	Down	Unigene113010_All//gi|115464229|ref|NP_001055714.1|//8.00E-08//Os05g0453300 [Oryza sativa Japonica Group] >gi|113579265|dbj|BAF17628.1| Os05g0453300 [Oryza sativa Japonica Group]	Unigene113010_All//2.00E-13//AT4G02830| unknown protein					nr +
Unigene113011_All	422	0.00	0.00	0.00	0.00	0.00	0.00	3.31	0.69	0.19	null	null		null	null		null	null		null	null		-2.25E+00	9.69E-05	Down	-4.13E+00	3.70E-08	Down	Unigene113011_All//gi|116055804|emb|CAL57889.1|//2.00E-10//Protein required for actin cytoskeleton organization and cell cycle progression (ISS) [Ostreococcus tauri]				ko03040|Spliceosome	GO:0005488//GO:0016787	nr -
Unigene113012_All	425	0.00	0.00	0.00	0.00	0.00	0.11	2.19	0.20	0.66	null	null		null	null		null	null		6.73E+00	7.01E-01		-3.48E+00	7.79E-05	Down	-1.74E+00	8.54E-03		Unigene113012_All//gi|15223264|ref|NP_172335.1|//1.00E-29//CLAVATA1 receptor kinase (CLV1) [Arabidopsis thaliana] >gi|9802553|gb|AAF99755.1|AC003981_5 F22013.7 [Arabidopsis thaliana] >gi|224589384|gb|ACN59226.1| leucine-rich repeat receptor-like protein kinase [Arabidopsis thaliana]	Unigene113012_All//7.00E-31//AT1G08590| CLAVATA1 receptor kinase (CLV1)			ko04626|Plant-pathogen interaction	GO:0000166//GO:0004672//GO:0008152//GO:0016020	nr -
Unigene113013_All	426	0.00	0.00	0.00	0.00	0.00	0.00	3.18	1.28	0.00	null	null		null	null		null	null		null	null		-1.32E+00	1.25E-02		-1.16E+01	4.86E-10	Down	Unigene113013_All//gi|299472847|emb|CBN80416.1|//3.00E-22//EsV-1-116 [Ectocarpus siliculosus]						nr -
Unigene113014_All	429	0.00	0.00	0.00	0.00	0.00	0.00	4.04	12.57	0.19	null	null		null	null		null	null		null	null		1.64E+00	6.75E-11	Up	-4.44E+00	1.94E-10	Down	Unigene113014_All//gi|145334271|ref|NP_001078516.1|//6.00E-57//histone H3.2 [Arabidopsis thaliana]	Unigene113014_All//4.00E-58//AT4G40030| histone H3.2	Unigene113014_All//8.00E-09//CD485766			GO:0000785//GO:0003676//GO:0009536//GO:0034728	nr +
Unigene113015_All	429	0.00	0.00	0.00	0.00	0.00	0.00	6.01	0.00	0.74	null	null		null	null		null	null		null	null		-1.26E+01	3.07E-18	Down	-3.02E+00	2.15E-11	Down							

Unigene113016_All	430	0.00	0.00	0.00	0.00	0.00	0.00	2.26	0.29	0.37	null	null		null	null		null	null		null	null		-2.96E+00	1.89E-04	Down	-2.61E+00	3.35E-04	Down	Unigene113016_All//gi|41203466|ref|NP_957726.1|//3.00E-28//NADH dehydrogenase subunit 4 [Emiliania huxleyi] >gi|33114152|gb|AAP94708.1| NADH dehydrogenase subunit 4 [Emiliania huxleyi]	Unigene113016_All//3.00E-22//ATMG00580| NADH dehydrogenase subunit 4			ko00190|Oxidative phosphorylation	GO:0016020//GO:0055114	nr -
Unigene113017_All	430	0.00	0.00	0.00	0.00	0.00	0.00	3.74	0.19	1.11	null	null		null	null		null	null		null	null		-4.26E+00	4.13E-09	Down	-1.75E+00	2.97E-04	Down							
Unigene113018_All	431	0.00	0.00	0.00	0.00	0.00	0.00	5.30	0.19	0.00	null	null		null	null		null	null		null	null		-4.77E+00	1.41E-13	Down	-1.24E+01	8.15E-17	Down	Unigene113018_All//gi|300265437|gb|EFJ49628.1|//6.00E-48//component of cytosolic 80S ribosome and 40S small subunit [Volvox carteri f. nagariensis]	Unigene113018_All//6.00E-45//AT4G34670| 40S ribosomal protein S3A (RPS3aB)			ko03010|Ribosome	GO:0005198//GO:0010467//GO:0030529	nr -
Unigene113019_All	431	0.00	0.00	0.00	0.00	0.00	0.00	2.16	0.00	0.00	null	null		null	null		null	null		null	null		-1.11E+01	1.14E-06	Down	-1.11E+01	5.48E-07	Down							
Unigene113020_All	434	0.00	0.00	0.00	0.00	0.16	0.00	3.02	0.19	0.55	null	null		null	null		7.31E+00	6.14E-01		null	null		-3.97E+00	3.35E-07	Down	-2.45E+00	4.22E-05	Down			Unigene113020_All//4.00E-103//TC165744				
Unigene113022_All	437	0.00	0.00	0.00	0.00	0.00	0.00	2.42	0.57	0.00	null	null		null	null		null	null		null	null		-2.08E+00	1.78E-03		-1.12E+01	6.72E-08	Down							ESTscan -
Unigene113025_All	440	0.00	0.00	0.00	0.00	0.00	0.00	3.84	0.19	0.00	null	null		null	null		null	null		null	null		-4.34E+00	1.16E-09	Down	-1.19E+01	1.70E-12	Down	Unigene113025_All//gi|18087805|ref|NP_032529.2|//2.00E-51//40S ribosomal protein S2 [Mus musculus] >gi|78126159|ref|NP_114026.3| 40S ribosomal protein S2 [Rattus norvegicus] >gi|133914|sp|P27952.1|RS2_RAT RecName: Full=40S ribosomal protein S2 >gi|46397787|sp|P25444.3|RS2_MOUSE RecName: Full=40S ribosomal protein S2; AltName: Full=40S ribosomal protein S4; AltName: Full=Protein LLRep3 >gi|10179940|gb|AAG13953.1|AF283559_1 ribosomal protein S2 [Mus musculus] >gi|57718|emb|CAA40679.1| ribosomal protein S2 [Rattus rattus] >gi|12805427|gb|AAH02186.1| Ribosomal protein S2 [Mus musculus] >gi|56971737|gb|AAH87956.1| Ribosomal protein S2 [Mus musculus] >gi|61402217|gb|AAH91730.1| Ribosomal protein S2 [Mus musculus]	Unigene113025_All//1.00E-48//AT1G58684| 40S ribosomal protein S2, putative	Unigene113025_All//4.00E-17//DW519163		ko03010|Ribosome	GO:0003676//GO:0005198//GO:0006413//GO:0009536//GO:0015935//GO:0016020//GO:0017134//GO:0030312//GO:0031981//GO:0042257	nr +
Unigene113026_All	446	0.00	0.00	0.00	0.00	0.00	0.00	4.46	0.00	0.54	null	null		null	null		null	null		null	null		-1.21E+01	4.61E-14	Down	-3.06E+00	4.82E-09	Down	Unigene113026_All//gi|114432130|gb|ABI74673.1|//4.00E-08//suppressor of necrosis 1 [Phytophthora infestans]				ko03040|Spliceosome		nr +
Unigene113027_All	446	0.00	0.00	0.00	0.00	0.00	0.00	9.95	0.56	0.00	null	null		null	null		null	null		null	null		-4.15E+00	6.72E-24	Down	-1.33E+01	1.22E-32	Down			Unigene113027_All//3.00E-08//gi|11207717|gb|BF276647.1|BF276647				
Unigene113028_All	446	0.00	0.00	0.00	0.00	0.00	0.00	3.32	0.84	1.25	null	null		null	null		null	null		null	null		-1.98E+00	2.39E-04	Down	-1.41E+00	3.79E-03		Unigene113028_All//gi|115466618|ref|NP_001056908.1|//6.00E-36//Os06g0165500 [Oryza sativa Japonica Group] >gi|113594948|dbj|BAF18822.1| Os06g0165500 [Oryza sativa Japonica Group]	Unigene113028_All//3.00E-14//AT1G78830| curculin-like (mannose-binding) lectin family protein				GO:0000166//GO:0004672//GO:0009987	nr -
Unigene113030_All	449	0.00	0.00	0.00	0.00	0.00	0.00	2.17	0.19	0.18	null	null		null	null		null	null		null	null		-3.54E+00	4.32E-05	Down	-3.61E+00	2.14E-05	Down	Unigene113030_All//gi|5669638|gb|AAD46404.1|AF096248_1//3.00E-34//ethylene-responsive RNA helicase [Solanum lycopersicum]	Unigene113030_All//1.00E-34//AT3G06480| DEAD box RNA helicase, putative			ko03040|Spliceosome	GO:0032559//GO:0042623//GO:0043231	nr +
Unigene113031_All	449	0.00	0.00	0.00	0.00	0.00	0.00	2.64	0.09	0.53	null	null		null	null		null	null		null	null		-4.82E+00	2.83E-07	Down	-2.31E+00	2.04E-04	Down	Unigene113031_All//gi|2388584|gb|AAB71465.1|//5.00E-09//EST gb|ATTS1136 comes from this gene [Arabidopsis thaliana]	Unigene113031_All//4.00E-07//AT4G33740| unknown protein				GO:0003824	nr +
Unigene113032_All	450	0.23	0.00	0.00	0.22	0.00	0.20	4.04	0.00	1.42	-7.86E+00	3.75E-01		-7.86E+00	3.97E-01		-7.79E+00	3.16E-01		-1.42E-01	9.52E-01		-1.20E+01	7.10E-13	Down	-1.51E+00	5.47E-04	Down	Unigene113032_All//gi|30687119|ref|NP_181275.2|//2.00E-26//heavy-metal-associated domain-containing protein [Arabidopsis thaliana]	Unigene113032_All//1.00E-27//AT2G37390| heavy-metal-associated domain-containing protein	Unigene113032_All//2.00E-126//TC177472			GO:0006812//GO:0043169	nr -
Unigene113033_All	451	0.00	0.00	0.00	0.00	0.00	0.00	5.72	0.19	0.00	null	null		null	null		null	null		null	null		-4.95E+00	1.46E-15	Down	-1.25E+01	5.57E-19	Down	Unigene113033_All//gi|2500354|sp|P93847.1|RL10_SOLME//3.00E-57//RecName: Full=60S ribosomal protein L10; AltName: Full=EQM >gi|1902894|dbj|BAA19462.1| QM family protein [Solanum melongena]	Unigene113033_All//5.00E-54//AT1G66580| 60S ribosomal protein L10 (RPL10C)	Unigene113033_All//4.00E-11//TC145515		ko03010|Ribosome	GO:0005198//GO:0010467//GO:0016787//GO:0030529//GO:0044238//GO:0045229	nr -
Unigene113034_All	457	0.57	0.00	0.00	1.64	1.51	0.20	3.24	0.82	0.52	-9.16E+00	6.72E-02		-9.16E+00	8.59E-02		-1.18E-01	8.62E-01		-3.05E+00	3.33E-03		-1.98E+00	2.39E-04	Down	-2.63E+00	4.77E-06	Down			Unigene113034_All//2.00E-77//TC158640				
Unigene113035_All	463	0.34	0.00	0.12	0.54	0.00	0.00	6.76	0.99	0.52	-8.41E+00	2.16E-01		-1.56E+00	5.34E-01		-9.07E+00	4.19E-02		-9.07E+00	7.35E-02		-2.77E+00	3.94E-12	Down	-3.71E+00	2.72E-16	Down							
Unigene113036_All	465	0.00	0.00	0.00	0.00	0.00	0.00	4.18	0.72	0.51	null	null		null	null		null	null		null	null		-2.54E+00	3.67E-07	Down	-3.02E+00	8.70E-09	Down	Unigene113036_All//gi|115448105|ref|NP_001047832.1|//1.00E-44//Os02g0699600 [Oryza sativa Japonica Group] >gi|113537363|dbj|BAF09746.1| Os02g0699600 [Oryza sativa Japonica Group]	Unigene113036_All//5.00E-44//AT2G37190| 60S ribosomal protein L12 (RPL12A)			ko03010|Ribosome	GO:0005198//GO:0006950//GO:0009536//GO:0010467//GO:0015934//GO:0022613//GO:0031981//GO:0046914	nr -
Unigene113037_All	466	0.00	0.00	0.00	0.00	0.00	0.00	2.18	0.00	0.09	null	null		null	null		null	null		null	null		-1.11E+01	2.97E-07	Down	-4.67E+00	1.72E-06	Down	Unigene113037_All//gi|224009247|ref|XP_002293582.1|//2.00E-15//calcium transporting rt-atpase [Thalassiosira pseudonana CCMP1335] >gi|220970982|gb|EED89318.1| calcium transporting rt-atpase [Thalassiosira pseudonana CCMP1335]	Unigene113037_All//5.00E-14//AT5G57110| ACA8 (AUTOINHIBITED CA2+ -ATPASE, ISOFORM 8); calcium-transporting ATPase/ calmodulin binding / protein self-association				GO:0005515//GO:0006810//GO:0016020//GO:0019829//GO:0051707	nr +
Unigene113038_All	468	0.56	0.00	0.68	0.21	0.15	0.00	4.88	1.61	1.19	-9.13E+00	6.72E-02		2.88E-01	8.10E-01		-5.32E-01	8.18E-01		-7.73E+00	4.04E-01		-1.60E+00	6.24E-05	Down	-2.03E+00	8.82E-07	Down			Unigene113038_All//1.00E-10//BQ405485				
Unigene113039_All	469	0.00	0.00	0.00	0.00	0.00	0.00	2.88	1.34	0.00	null	null		null	null		null	null		null	null		-1.11E+00	3.31E-02		-1.15E+01	4.86E-10	Down	Unigene113039_All//gi|115452955|ref|NP_001050078.1|//3.00E-32//Os03g0343500 [Oryza sativa Japonica Group] >gi|42733478|dbj|BAD11336.1| BRI1-KD interacting protein 108 [Oryza sativa Japonica Group] >gi|113548549|dbj|BAF11992.1| Os03g0343500 [Oryza sativa Japonica Group]	Unigene113039_All//3.00E-29//AT3G05560| 60S ribosomal protein L22-2 (RPL22B)			ko03010|Ribosome	GO:0005622	nr -
Unigene113040_All	470	0.56	0.65	0.23	1.48	0.88	0.19	2.34	1.07	0.51	2.17E-01	8.65E-01		-1.30E+00	4.37E-01		-7.55E-01	3.69E-01		-2.95E+00	5.84E-03		-1.13E+00	5.52E-02		-2.20E+00	5.66E-04	Down			Unigene113040_All//6.00E-07//ES810824				
Unigene113041_All	471	0.00	0.00	0.00	0.00	0.00	0.00	2.60	0.44	0.00	null	null		null	null		null	null		null	null		-2.55E+00	8.70E-05	Down	-1.13E+01	4.03E-09	Down	Unigene113041_All//gi|15219950|ref|NP_173692.1|//8.00E-59//PFL (POINTED FIRST LEAVES); RNA binding / nucleic acid binding / structural constituent of ribosome [Arabidopsis thaliana] >gi|15234790|ref|NP_192718.1| RPS18C (S18 RIBOSOMAL PROTEIN); RNA binding / nucleic acid binding / structural constituent of ribosome [Arabidopsis thaliana] >gi|18399100|ref|NP_564434.1| 40S ribosomal protein S18 (RPS18B) [Arabidopsis thaliana] >gi|464707|sp|P34788.1|RS18_ARATH RecName: Full=40S ribosomal protein S18 >gi|10086474|gb|AAG12534.1|AC015446_15 ribosomal protein S18 [Arabidopsis thaliana] >gi|10092450|gb|AAG12853.1|AC079286_10 40S ribosomal protein S18; 25853-24673 [Arabidopsis thaliana] >gi|14423408|gb|AAK62386.1|AF386941_1 S18.A ribosomal protein [Arabidopsis thaliana] >gi|15724158|gb|AAL06471.1|AF411781_1 At1g22780/T22J18_5 [Arabidopsis thaliana] >gi|405613|emb|CAA80684.1|	Unigene113041_All//7.00E-51//AT4G09800| RPS18C (S18 RIBOSOMAL PROTEIN); RNA binding / nucleic acid binding / structural constituent of ribosome			ko03010|Ribosome	GO:0003676//GO:0005198//GO:0006412//GO:0015935//GO:0016020//GO:0030312//GO:0031981	nr +
Unigene113042_All	472	0.00	0.00	0.00	0.00	0.00	0.00	4.30	0.35	0.84	null	null		null	null		null	null		null	null		-3.60E+00	3.79E-10	Down	-2.35E+00	3.80E-07	Down	Unigene113042_All//gi|293336485|ref|NP_001170475.1|//2.00E-63//heat shock protein 90 [Zea mays] >gi|225903795|gb|ACO35045.1| heat shock protein 90 [Zea mays]	Unigene113042_All//1.00E-40//AT5G52640| ATHSP90.1 (HEAT SHOCK PROTEIN 90.1); ATP binding / unfolded protein binding	Unigene113042_All//4.00E-23//gi|164289322|gb|ES826064.1|ES826064		ko04626|Plant-pathogen interaction//ko04141|Protein processing in endoplasmic reticulum	GO:0005515//GO:0032559//GO:0044267//GO:0044424//GO:0050896	nr +
Unigene113043_All	479	0.00	0.00	0.00	0.00	0.00	0.00	4.06	1.22	0.75	null	null		null	null		null	null		null	null		-1.73E+00	1.00E-04	Down	-2.44E+00	3.73E-07	Down							ESTscan -
Unigene113045_All	485	0.00	0.00	0.00	0.00	0.00	0.00	1.92	0.17	0.00	null	null		null	null		null	null		null	null		-3.48E+00	7.79E-05	Down	-1.09E+01	5.48E-07	Down	Unigene113045_All//gi|15239409|ref|NP_200875.1|//4.00E-52//60S ribosomal protein L12 (RPL12C) [Arabidopsis thaliana] >gi|75309019|sp|Q9FF52.1|RL123_ARATH RecName: Full=60S ribosomal protein L12-3 >gi|9759331|dbj|BAB09840.1| 60S ribosomal protein L12 [Arabidopsis thaliana] >gi|14517558|gb|AAK62669.1| AT5g60670/mup24_80 [Arabidopsis thaliana] >gi|18700232|gb|AAL77726.1| AT5g60670/mup24_80 [Arabidopsis thaliana] >gi|21593741|gb|AAM65708.1| 60S ribosomal protein L12-like [Arabidopsis thaliana]	Unigene113045_All//3.00E-53//AT5G60670| 60S ribosomal protein L12 (RPL12C)			ko03010|Ribosome	GO:0005198//GO:0010467//GO:0015934//GO:0022613	nr +
Unigene113046_All	492	0.11	0.00	0.00	0.00	0.28	2.02	1.63	0.76	0.89	-6.73E+00	6.17E-01		-6.73E+00	6.30E-01		8.13E+00	3.20E-01		1.10E+01	6.15E-06	Up	-1.09E+00	1.29E-01		-8.74E-01	1.96E-01								
Unigene113048_All	495	0.00	0.00	0.00	0.00	0.00	0.00	1.88	0.17	0.24	null	null		null	null		null	null		null	null		-3.48E+00	7.79E-05	Down	-2.96E+00	1.72E-04	Down							
Unigene113049_All	501	0.00	0.00	0.00	0.00	0.00	0.00	4.05	0.42	1.03	null	null		null	null		null	null		null	null		-3.28E+00	2.01E-09	Down	-1.97E+00	6.60E-06	Down	Unigene113049_All//gi|32400971|gb|AAP80691.1|//2.00E-69//ubiquitin-conjugating enzyme [Griffithsia japonica]	Unigene113049_All//1.00E-59//AT3G08690| UBC11 (UBIQUITIN-CONJUGATING ENZYME 11); ubiquitin-protein ligase	Unigene113049_All//2.00E-10//ES828592		ko04141|Protein processing in endoplasmic reticulum//ko04120|Ubiquitin mediated proteolysis	GO:0006464//GO:0019787//GO:0019941//GO:0060255	nr +

Unigene11305_All	3343	9.31	19.43	11.99	26.72	27.60	27.55	32.14	52.46	94.44	1.06E+00	9.55E-52	Up	3.65E-01	2.76E-05		4.66E-02	5.15E-01		4.41E-02	5.35E-01		7.07E-01	1.25E-85		1.56E+00	0.00E+00	Up	Unigene11305_All//gi|47834701|gb|AAT39004.1|//0.00E+00//AML1 [Citrus unshiu]	Unigene11305_All//0.00E+00//AT1G29400| AML5 (ARABIDOPSIS MEI2-LIKE PROTEIN 5); RNA binding / nucleic acid binding / nucleotide binding	Unigene11305_All//0.00E+00//TC150073		ko03040|Spliceosome	GO:0003676	nr +
Unigene113050_All	501	0.00	0.00	0.00	0.00	0.00	0.00	1.69	0.00	0.00	null	null		null	null		null	null		null	null		-1.07E+01	4.39E-06	Down	-1.07E+01	2.21E-06	Down	Unigene113050_All//gi|57472005|gb|AAW51128.1|//2.00E-11//minus agglutinin [Chlamydomonas incerta]	Unigene113050_All//3.00E-12//AT3G44100| MD-2-related lipid recognition domain-containing protein / ML domain-containing protein			ko00230|Purine metabolism//ko00240|Pyrimidine metabolism//ko03020|RNA polymerase	GO:0003824//GO:0016020//GO:0030312//GO:0043231	nr -
Unigene113051_All	510	0.00	0.00	0.00	0.00	0.00	0.00	2.24	0.57	0.00	null	null		null	null		null	null		null	null		-1.96E+00	1.69E-03		-1.11E+01	1.65E-08	Down	Unigene113051_All//gi|152003979|gb|ABS19813.1|//9.00E-49//eukaryotic initiation factor 4A [Lolium perenne]	Unigene113051_All//4.00E-42//AT3G13920| EIF4A1 (EUKARYOTIC TRANSLATION INITIATION FACTOR 4A1); ATP-dependent helicase/ translation initiation factor				GO:0003676//GO:0032559//GO:0042623	nr +
Unigene113052_All	518	0.51	0.78	0.31	0.96	0.53	0.35	3.59	4.68	1.23	6.32E-01	6.01E-01		-7.12E-01	6.22E-01		-8.55E-01	4.07E-01		-1.46E+00	1.93E-01		3.82E-01	3.27E-01		-1.55E+00	3.64E-04	Down			Unigene113052_All//6.00E-07//TC155745				
Unigene113053_All	519	0.00	0.10	0.41	0.96	0.07	0.78	4.48	0.97	1.07	6.61E+00	6.20E-01		8.68E+00	1.42E-01		-3.86E+00	5.32E-03		-2.93E-01	7.57E-01		-2.21E+00	2.77E-07	Down	-2.06E+00	5.41E-07	Down							
Unigene113054_All	524	0.00	0.00	0.00	0.19	0.00	0.00	2.58	0.48	1.29	null	null		null	null		-7.57E+00	3.16E-01		-7.57E+00	4.04E-01		-2.43E+00	5.63E-05	Down	-9.98E-01	4.39E-02		Unigene113054_All//gi|297830832|ref|XP_002883298.1|//3.00E-17//basic helix-loop-helix family protein [Arabidopsis lyrata subsp. lyrata] >gi|297329138|gb|EFH59557.1| basic helix-loop-helix family protein [Arabidopsis lyrata subsp. lyrata]	Unigene113054_All//2.00E-18//AT3G21330| basic helix-loop-helix (bHLH) family protein		bHLH		GO:0001071//GO:0010468//GO:0043231	nr -
Unigene113055_All	525	0.00	0.00	0.00	0.00	0.00	0.00	5.07	0.32	0.30	null	null		null	null		null	null		null	null		-3.99E+00	3.57E-14	Down	-4.06E+00	6.84E-15	Down	Unigene113055_All//gi|61656129|gb|AAX49367.1|//4.00E-31//At2g38640 [Arabidopsis thaliana]	Unigene113055_All//1.00E-30//AT5G41590| unknown protein					nr -
Unigene113056_All	525	0.20	0.29	1.01	0.28	0.33	1.29	2.98	0.16	0.91	5.39E-01	7.80E-01		2.35E+00	5.88E-02		2.04E-01	9.50E-01		2.18E+00	3.14E-02		-4.23E+00	7.78E-09	Down	-1.71E+00	4.59E-04	Down	Unigene113056_All//gi|297812057|ref|XP_002873912.1|//4.00E-38//EMB3013 [Arabidopsis lyrata subsp. lyrata] >gi|297319749|gb|EFH50171.1| EMB3013 [Arabidopsis lyrata subsp. lyrata]	Unigene113056_All//9.00E-38//AT5G18700| EMB3013 (embryo defective 3013); ATP binding / binding / kinase/ protein kinase/ protein serine/threonine kinase	Unigene113056_All//0.00E+00//TC152167			GO:0004672//GO:0005488	nr -
Unigene113058_All	536	0.20	1.13	0.00	1.12	0.90	0.25	2.76	0.55	0.67	2.54E+00	2.46E-02		-7.61E+00	3.97E-01		-3.10E-01	7.41E-01		-2.14E+00	4.39E-02		-2.34E+00	3.57E-05	Down	-2.05E+00	1.05E-04	Down							
Unigene113059_All	538	0.00	0.00	0.00	0.00	0.00	0.00	3.22	0.78	0.00	null	null		null	null		null	null		null	null		-2.05E+00	3.55E-05	Down	-1.17E+01	8.37E-13	Down	Unigene113059_All//gi|37625023|gb|AAQ96335.1|//7.00E-66//ribosomal protein L3A [Nicotiana tabacum]	Unigene113059_All//3.00E-65//AT1G61580| RPL3B (R-PROTEIN L3 B); structural constituent of ribosome	Unigene113059_All//6.00E-07//TC149894		ko03010|Ribosome	GO:0005198//GO:0010467//GO:0030529	nr -
Unigene11306_All	1990	6.52	1.94	4.04	10.89	10.28	12.23	19.46	13.97	25.99	-1.75E+00	1.09E-22	Down	-6.90E-01	2.02E-05		-8.32E-02	5.68E-01		1.68E-01	1.79E-01		-4.79E-01	3.95E-10		4.17E-01	9.27E-11		Unigene11306_All//gi|115478695|ref|NP_001062941.1|//1.00E-141//Os09g0346700 [Oryza sativa Japonica Group] >gi|113631174|dbj|BAF24855.1| Os09g0346700 [Oryza sativa Japonica Group]	Unigene11306_All//0.00E+00//AT2G25800| INVOLVED IN: biological_process unknown; EXPRESSED IN: 23 plant structures; EXPRESSED DURING: 13 growth stages; CONTAINS InterPro DOMAIN/s: Munc13 homology 1 (InterPro:IPR014770), Protein of unknown function DUF810 (InterPro:IPR008528), Munc13 homology 2 (InterPro:IPR014772); BEST Arabidopsis thaliana protein match is: unknown protein (TAIR:AT2G20010.2); Has 242 Blast hits to 156 proteins in 35 species: Archae - 0; Bacteria - 8; Metazoa - 36; Fungi - 18; Plants - 94; Viruses - 0; Other Eukaryotes - 86 (source: NCBI BLink).	Unigene11306_All//0.00E+00//TC135767				nr +
Unigene113060_All	547	0.48	0.65	0.19	0.18	0.19	0.33	3.17	1.15	1.09	4.39E-01	7.01E-01		-1.30E+00	4.37E-01		5.29E-02	9.68E-01		8.59E-01	6.96E-01		-1.47E+00	1.48E-03		-1.54E+00	6.50E-04	Down	Unigene113060_All//gi|297833840|ref|XP_002884802.1|//6.00E-68//glycosyl transferase family 1 protein [Arabidopsis lyrata subsp. lyrata] >gi|297330642|gb|EFH61061.1| glycosyl transferase family 1 protein [Arabidopsis lyrata subsp. lyrata]	Unigene113060_All//9.00E-68//AT3G10630| glycosyl transferase family 1 protein	Unigene113060_All//0.00E+00//ES809782			GO:0008152//GO:0016740//GO:0043231	nr -
Unigene113061_All	555	0.19	0.00	0.00	0.00	0.00	0.00	4.65	1.05	0.86	-7.56E+00	3.75E-01		-7.56E+00	3.97E-01		null	null		null	null		-2.14E+00	1.05E-07	Down	-2.43E+00	3.32E-09	Down	Unigene113061_All//gi|53370641|gb|AAU89136.1|//5.00E-07//F-box domain containing protein [Oryza sativa Japonica Group]		Unigene113061_All//1.00E-51//ES797781			GO:0043231	nr -
Unigene113062_All	556	0.00	0.00	0.00	0.00	0.00	0.00	2.89	0.45	0.14	null	null		null	null		null	null		null	null		-2.68E+00	2.38E-06	Down	-4.33E+00	1.41E-09	Down	Unigene113062_All//gi|300175513|emb|CBK20824.2|//2.00E-15//unnamed protein product [Blastocystis hominis]						nr +
Unigene113063_All	556	0.00	0.00	0.00	0.00	0.00	0.00	3.57	0.75	0.07	null	null		null	null		null	null		null	null		-2.25E+00	1.94E-06	Down	-5.64E+00	2.87E-13	Down	Unigene113063_All//sp|P51880|FABP7_MOUSE//1.00E-22//Fatty acid-binding protein, brain OS=Mus musculus GN=Fabp7 PE=1 SV=2						swissprot +
Unigene113064_All	561	0.00	0.00	0.00	0.00	0.00	0.00	2.49	0.00	0.00	null	null		null	null		null	null		null	null		-1.13E+01	6.58E-10	Down	-1.13E+01	2.40E-10	Down	Unigene113064_All//gi|159459822|gb|ABW96308.1|//8.00E-79//heat shock protein 90 [Vitis pseudoreticulata]	Unigene113064_All//1.00E-57//AT5G52640| ATHSP90.1 (HEAT SHOCK PROTEIN 90.1); ATP binding / unfolded protein binding			ko04626|Plant-pathogen interaction//ko04141|Protein processing in endoplasmic reticulum	GO:0005515//GO:0032559//GO:0044267//GO:0044424//GO:0050896	nr -
Unigene113065_All	562	0.00	0.00	0.00	0.00	0.00	0.00	3.46	2.45	0.07	null	null		null	null		null	null		null	null		-4.96E-01	2.44E-01		-5.61E+00	5.71E-13	Down	Unigene113065_All//gi|119713|sp|P24152.1|EXTN_SORBI//1.00E-06//RecName: Full=Extensin; AltName: Full=Proline-rich glycoprotein; Flags: Precursor >gi|21627|emb|CAA39485.1| hydroxyproline-rich glycoprotein [Sorghum bicolor]						nr -
Unigene113066_All	572	0.00	0.00	0.00	0.00	0.24	0.00	5.47	0.73	0.00	null	null		null	null		7.91E+00	3.20E-01		null	null		-2.90E+00	1.02E-12	Down	-1.24E+01	5.19E-23	Down							
Unigene113067_All	578	0.00	0.00	0.00	0.00	0.00	0.16	4.46	0.00	0.14	null	null		null	null		null	null		7.29E+00	4.80E-01		-1.21E+01	3.06E-18	Down	-5.02E+00	2.78E-16	Down			Unigene113067_All//7.00E-07//gi|21098785|gb|BQ411098.1|BQ411098				
Unigene113068_All	580	0.00	0.00	0.00	0.00	0.24	0.00	6.12	0.00	0.00	null	null		null	null		7.89E+00	3.20E-01		null	null		-1.26E+01	4.04E-25	Down	-1.26E+01	4.09E-26	Down							
Unigene113069_All	586	0.00	0.00	0.00	0.00	0.00	0.00	2.89	1.14	0.14	null	null		null	null		null	null		null	null		-1.34E+00	3.80E-03		-4.41E+00	3.76E-10	Down	Unigene113069_All//gi|6012952|emb|CAB57309.1|//4.00E-35//60S ribosomal protein L6 (YL 16 like) [Cyanophora paradoxa]	Unigene113069_All//6.00E-31//AT1G18540| 60S ribosomal protein L6 (RPL6A)			ko03010|Ribosome	GO:0009536//GO:0015934//GO:0016020	nr -
Unigene113070_All	599	0.00	0.00	0.00	0.00	0.00	0.00	2.54	0.28	0.20	null	null		null	null		null	null		null	null		-3.19E+00	4.80E-07	Down	-3.67E+00	3.49E-08	Down	Unigene113070_All//gi|116056635|emb|CAL52924.1|//2.00E-16//CCCH-type Zn-finger protein (ISS) [Ostreococcus tauri]	Unigene113070_All//9.00E-13//AT1G66810| zinc finger (CCCH-type) family protein		C3H		GO:0046872	nr +
Unigene113071_All	604	0.00	0.00	0.00	0.00	0.00	0.00	28.77	0.90	0.07	null	null		null	null		null	null		null	null		-5.00E+00	2.16E-103	Down	-8.77E+00	6.55E-126	Down	Unigene113071_All//gi|297807629|ref|XP_002871698.1|//1.00E-46//3-oxo-5-alpha-steroid 4-dehydrogenase family protein [Arabidopsis lyrata subsp. lyrata] >gi|297317535|gb|EFH47957.1| 3-oxo-5-alpha-steroid 4-dehydrogenase family protein [Arabidopsis lyrata subsp. lyrata]	Unigene113071_All//9.00E-45//AT5G16010| 3-oxo-5-alpha-steroid 4-dehydrogenase family protein / steroid 5-alpha-reductase family protein	Unigene113071_All//2.00E-16//ES794235			GO:0009526//GO:0016020	nr +
Unigene113072_All	612	0.00	0.00	0.35	0.00	0.00	0.00	13.68	0.41	0.91	null	null		8.44E+00	1.42E-01		null	null		null	null		-5.06E+00	2.82E-50	Down	-3.91E+00	2.72E-44	Down	Unigene113072_All//gi|8778875|gb|AAF79874.1|AC000348_27//3.00E-49//T7N9.16 [Arabidopsis thaliana]	Unigene113072_All//2.00E-50//AT1G27100| FUNCTIONS IN: molecular_function unknown; INVOLVED IN: biological_process unknown; LOCATED IN: vacuole; EXPRESSED IN: 23 plant structures; EXPRESSED DURING: 13 growth stages; CONTAINS InterPro DOMAIN/s: Protein of unknown function DUF569 (InterPro:IPR007679), Actin_cross-linking (InterPro:IPR008999); BEST Arabidopsis thaliana protein match is: unknown protein (TAIR:AT1G69900.1); Has 184 Blast hits to 118 proteins in 20 species: Archae - 0; Bacteria - 2; Metazoa - 3; Fungi - 8; Plants - 166; Viruses - 0; Other Eukaryotes - 5 (source: NCBI BLink).	Unigene113072_All//3.00E-89//gi|109860727|gb|DW489706.1|DW489706			GO:0043231	nr +
Unigene113073_All	614	0.09	0.00	0.00	0.00	0.11	0.00	2.34	0.00	0.91	-6.41E+00	6.17E-01		-6.41E+00	6.30E-01		6.81E+00	6.14E-01		null	null		-1.12E+01	3.33E-10	Down	-1.37E+00	5.50E-03		Unigene113073_All//gi|224085587|ref|XP_002307628.1|//3.00E-82//glycosyltransferase, CAZy family GT8 [Populus trichocarpa] >gi|222857077|gb|EEE94624.1| glycosyltransferase, CAZy family GT8 [Populus trichocarpa]	Unigene113073_All//2.00E-75//AT1G06780| GAUT6 (Galacturonosyltransferase 6); polygalacturonate 4-alpha-galacturonosyltransferase/ transferase, transferring glycosyl groups	Unigene113073_All//0.00E+00//gi|164343548|gb|ES825706.1|ES825706			GO:0008194//GO:0043231//GO:0045489	nr -
Unigene113074_All	625	0.00	0.00	0.00	0.00	0.11	0.07	2.71	0.00	0.06	null	null		null	null		6.78E+00	6.14E-01		6.18E+00	7.01E-01		-1.14E+01	5.52E-12	Down	-5.41E+00	3.52E-11	Down	Unigene113074_All//gi|300521738|gb|ADK26058.1|//9.00E-14//drought resistance protein [Glycine max]	Unigene113074_All//8.00E-13//AT2G47540| pollen Ole e 1 allergen and extensin family protein	Unigene113074_All//0.00E+00//BG444324				nr +
Unigene113075_All	630	0.00	0.00	0.00	0.00	0.00	0.00	4.03	0.13	0.70	null	null		null	null		null	null		null	null		-4.92E+00	2.81E-15	Down	-2.53E+00	1.83E-09	Down	Unigene113075_All//gi|168009836|ref|XP_001757611.1|//2.00E-06//argonaute family member [Physcomitrella patens subsp. patens] >gi|162691305|gb|EDQ77668.1| argonaute family member [Physcomitrella patens subsp. patens]						nr +
Unigene113076_All	630	0.00	0.00	0.00	0.00	0.00	0.00	2.68	0.07	0.00	null	null		null	null		null	null		null	null		-5.34E+00	1.11E-10	Down	-1.14E+01	1.70E-12	Down	Unigene113076_All//gi|70663909|emb|CAE01636.3|//2.00E-49//OSJNBa0029H02.21 [Oryza sativa (japonica cultivar-group)]	Unigene113076_All//5.00E-45//AT3G55280| RPL23AB (RIBOSOMAL PROTEIN L23AB); RNA binding / nucleotide binding / structural constituent of ribosome			ko03010|Ribosome	GO:0003676//GO:0005198//GO:0010467//GO:0030529//GO:0043231	nr +
Unigene113077_All	631	0.00	0.00	0.00	0.16	0.00	0.00	5.23	0.60	0.38	null	null		null	null		-7.30E+00	3.16E-01		-7.30E+00	4.04E-01		-3.13E+00	2.40E-14	Down	-3.79E+00	2.10E-17	Down			Unigene113077_All//5.00E-119//AI728318				
Unigene113078_All	643	0.00	0.00	0.00	0.00	0.00	0.00	7.36	0.26	0.25	null	null		null	null		null	null		null	null		-4.82E+00	6.79E-28	Down	-4.89E+00	3.81E-29	Down	Unigene113078_All//gi|301095349|ref|XP_002896775.1|//3.00E-49//60S acidic ribosomal protein P0 [Phytophthora infestans T30-4] >gi|262108658|gb|EEY66710.1| 60S acidic ribosomal protein P0 [Phytophthora infestans T30-4]	Unigene113078_All//3.00E-42//AT2G40010| 60S acidic ribosomal protein P0 (RPP0A)			ko03010|Ribosome	GO:0005198//GO:0006412//GO:0030529	nr +
Unigene113079_All	652	0.00	0.00	0.00	0.00	0.00	0.00	2.40	0.90	0.00	null	null		null	null		null	null		null	null		-1.42E+00	3.64E-03		-1.12E+01	1.42E-11	Down							ESTscan -

Unigene113080_All	673	0.23	0.00	0.08	0.37	0.00	0.40	3.58	1.43	1.36	-7.87E+00	2.16E-01		-1.56E+00	5.34E-01		-8.53E+00	4.19E-02		1.21E-01	9.44E-01		-1.33E+00	4.24E-04	Down	-1.40E+00	1.51E-04	Down	Unigene113080_All//gi|140053482|gb|ABO80459.1|//7.00E-07//RNA-directed DNA polymerase (Reverse transcriptase); Ribonuclease H [Medicago truncatula]	Unigene113080_All//8.00E-09//AT4G29090| reverse transcriptase, putative / RNA-dependent DNA polymerase, putative					nr +
Unigene113081_All	674	0.00	0.00	0.00	0.00	0.00	0.00	3.45	0.06	1.12	null	null		null	null		null	null		null	null		-5.80E+00	5.23E-15	Down	-1.62E+00	2.77E-05	Down	Unigene113081_All//gi|157093097|gb|ABV22203.1|//1.00E-33//skp1 family protein [Karlodinium micrum]	Unigene113081_All//7.00E-26//AT4G34210| ASK11 (ARABIDOPSIS SKP1-LIKE 11); protein binding / ubiquitin-protein ligase	Unigene113081_All//8.00E-07//TC174480		ko04141|Protein processing in endoplasmic reticulum//ko04120|Ubiquitin mediated proteolysis		nr +
Unigene113082_All	683	0.00	0.00	0.00	0.00	0.00	0.00	5.32	0.86	0.00	null	null		null	null		null	null		null	null		-2.64E+00	2.59E-13	Down	-1.24E+01	9.77E-27	Down	Unigene113082_All//gi|223996425|ref|XP_002287886.1|//1.00E-11//predicted protein [Thalassiosira pseudonana CCMP1335] >gi|220977002|gb|EED95329.1| predicted protein [Thalassiosira pseudonana CCMP1335]						nr -
Unigene113083_All	725	0.87	0.00	0.00	0.82	0.57	1.12	2.86	0.81	0.00	-9.76E+00	7.92E-04	Down	-9.76E+00	1.41E-03		-5.33E-01	5.62E-01		4.44E-01	6.21E-01		-1.82E+00	2.74E-05	Down	-1.15E+01	2.86E-15	Down	Unigene113083_All//gi|124359343|gb|ABD28505.2|//1.00E-34//RNA-directed DNA polymerase (Reverse transcriptase); Polynucleotidyl transferase, Ribonuclease H fold [Medicago truncatula]	Unigene113083_All//6.00E-10//AT3G24255| FUNCTIONS IN: molecular_function unknown; INVOLVED IN: biological_process unknown; LOCATED IN: cellular_component unknown; CONTAINS InterPro DOMAIN/s: RNA-directed DNA polymerase (reverse transcriptase), related (InterPro:IPR015706); BEST Arabidopsis thaliana protein match is: unknown protein (TAIR:AT3G23910.1); Has 2302 Blast hits to 2235 proteins in 74 species: Archae - 4; Bacteria - 15; Metazoa - 871; Fungi - 12; Plants - 1295; Viruses - 0; Other Eukaryotes - 105 (source: NCBI BLink).					nr -
Unigene113084_All	736	0.07	0.00	0.72	0.47	0.14	0.00	4.54	0.74	0.70	-6.15E+00	6.17E-01		3.35E+00	2.06E-02		-1.75E+00	1.90E-01		-8.89E+00	2.13E-02		-2.62E+00	3.28E-12	Down	-2.69E+00	5.32E-13	Down							
Unigene113085_All	773	0.00	0.00	0.00	0.00	0.00	0.00	11.81	0.00	0.00	null	null		null	null		null	null		null	null		-1.35E+01	7.72E-65	Down	-1.35E+01	3.03E-67	Down							ESTscan +
Unigene113086_All	847	0.00	0.00	0.00	0.00	0.00	0.00	3.94	0.35	0.33	null	null		null	null		null	null		null	null		-3.51E+00	5.04E-16	Down	-3.58E+00	6.98E-17	Down	Unigene113086_All//gi|159147901|dbj|BAF92024.1|//6.00E-29//sedolisin-like peptidase [Actinophrys sol]				ko00230|Purine metabolism//ko00240|Pyrimidine metabolism//ko03020|RNA polymerase	GO:0003824	nr -
Unigene113087_All	852	0.55	0.54	0.56	0.88	0.61	0.32	2.73	0.59	0.98	-4.64E-02	9.78E-01		2.54E-02	1.00E+00		-5.33E-01	5.26E-01		-1.46E+00	9.04E-02		-2.21E+00	2.77E-07	Down	-1.47E+00	1.01E-04	Down	Unigene113087_All//gi|224111738|ref|XP_002315960.1|//1.00E-125//NAC domain protein, IPR003441 [Populus trichocarpa] >gi|222865000|gb|EEF02131.1| NAC domain protein, IPR003441 [Populus trichocarpa]	Unigene113087_All//2.00E-106//AT1G25580| ANAC008 (Arabidopsis NAC domain containing protein 8); transcription factor	Unigene113087_All//0.00E+00//TC139349	NAC		GO:0003676//GO:0010468//GO:0043231	nr +
Unigene113088_All	876	0.00	0.00	0.00	0.00	0.00	0.00	33.01	0.86	0.91	null	null		null	null		null	null		null	null		-5.26E+00	1.85E-176	Down	-5.18E+00	3.91E-181	Down							
Unigene113089_All	888	0.00	0.00	0.00	0.00	0.00	0.00	22.38	0.38	0.63	null	null		null	null		null	null		null	null		-5.89E+00	3.34E-127	Down	-5.15E+00	3.15E-124	Down			Unigene113089_All//5.00E-15//TC171825				
Unigene113090_All	1053	0.00	0.00	0.00	0.00	0.20	0.00	9.92	0.32	0.49	null	null		null	null		7.62E+00	1.57E-01		null	null		-4.97E+00	4.98E-62	Down	-4.33E+00	2.86E-59	Down	Unigene113090_All//gi|224080564|ref|XP_002306164.1|//1.00E-119//aquaporin, MIP family, PIP subfamily [Populus trichocarpa] >gi|222849128|gb|EEE86675.1| aquaporin, MIP family, PIP subfamily [Populus trichocarpa]	Unigene113090_All//9.00E-22//AT1G17810| BETA-TIP (BETA-TONOPLAST INTRINSIC PROTEIN); water channel	Unigene113090_All//6.00E-15//gi|48791108|gb|CO092422.1|CO092422				nr -
Unigene113092_All	243	0.00	0.00	0.00	0.00	0.00	0.00	1.39	5.50	0.00	null	null		null	null		null	null		null	null		1.98E+00	5.28E-04	Up	-1.04E+01	8.15E-03								
Unigene113096_All	443	0.00	0.00	0.00	0.00	0.00	0.00	0.19	2.83	0.00	null	null		null	null		null	null		null	null		3.89E+00	8.57E-07	Up	-7.58E+00	3.60E-01		Unigene113096_All//gi|32401039|gb|AAP80725.1|//5.00E-51//histone H3.3 protein [Griffithsia japonica]	Unigene113096_All//2.00E-51//AT5G65360| histone H3				GO:0000785//GO:0003676//GO:0006810//GO:0009536//GO:0031224//GO:0034728	nr -
Unigene113097_All	1249	0.25	0.45	0.30	0.08	0.36	0.00	1.46	4.99	0.83	8.28E-01	3.81E-01		2.48E-01	8.23E-01		2.17E+00	1.06E-01		-6.32E+00	4.04E-01		1.78E+00	6.94E-14	Up	-8.12E-01	5.01E-02		Unigene113097_All//gi|8777580|dbj|BAA97098.1|//1.00E-07//unnamed protein product [Arabidopsis thaliana]		Unigene113097_All//4.00E-07//FL576859				nr -
Unigene113098_All	441	0.00	0.00	0.00	0.00	0.00	0.31	0.86	3.89	2.17	null	null		null	null		null	null		8.26E+00	3.12E-01		2.17E+00	2.00E-05	Up	1.33E+00	3.49E-02		Unigene113098_All//gi|115474457|ref|NP_001060825.1|//3.00E-11//Os08g0111200 [Oryza sativa Japonica Group] >gi|113622794|dbj|BAF22739.1| Os08g0111200 [Oryza sativa Japonica Group]	Unigene113098_All//3.00E-12//AT1G33700| catalytic/ glucosylceramidase				GO:0004553//GO:0006678//GO:0016020//GO:0043231	nr +
Unigene113100_All	364	0.00	0.00	0.00	0.00	0.00	0.00	0.00	1.84	0.22	null	null		null	null		null	null		null	null		1.08E+01	7.64E-05	Up	7.77E+00	4.06E-01		Unigene113100_All//gi|13486671|dbj|BAB39908.1|//1.00E-12//P0028E10.12 [Oryza sativa Japonica Group]	Unigene113100_All//7.00E-14//AT2G04865| unknown protein					nr +
Unigene113103_All	350	0.00	0.00	0.00	0.00	0.00	0.00	0.85	3.58	0.00	null	null		null	null		null	null		null	null		2.08E+00	5.41E-04	Up	-9.72E+00	1.58E-02		Unigene113103_All//gi|161015606|gb|ABX55855.1|//8.00E-56//actin [Spumella-like flagellate 1006]	Unigene113103_All//2.00E-54//AT5G09810| ACT7 (ACTIN 7); structural constituent of cytoskeleton	Unigene113103_All//8.00E-27//gi|31406833|gb|CD485868.1|CD485868		ko04145|Phagosome	GO:0032559//GO:0043232	nr +
Unigene113106_All	421	0.12	0.24	0.63	0.24	0.33	0.11	0.50	3.18	0.47	9.53E-01	7.27E-01		2.35E+00	2.40E-01		4.68E-01	8.77E-01		-1.14E+00	6.78E-01		2.66E+00	2.41E-05	Up	-8.58E-02	9.61E-01		Unigene113106_All//gi|297834090|ref|XP_002884927.1|//6.00E-30//short-chain dehydrogenase/reductase family protein [Arabidopsis lyrata subsp. lyrata] >gi|297330767|gb|EFH61186.1| short-chain dehydrogenase/reductase family protein [Arabidopsis lyrata subsp. lyrata]	Unigene113106_All//1.00E-24//AT3G12800| SDRB (SHORT-CHAIN DEHYDROGENASE-REDUCTASE B); binding / catalytic/ oxidoreductase	Unigene113106_All//6.00E-53//gi|48880901|gb|CO131923.1|CO131923		ko04146|Peroxisome	GO:0008152//GO:0016020//GO:0016491//GO:0042579	nr -
Unigene113107_All	571	0.27	0.44	0.09	0.00	1.09	0.47	1.04	2.56	0.56	6.91E-01	6.27E-01		-1.56E+00	5.34E-01		1.01E+01	7.90E-04	Up	8.89E+00	7.04E-02		1.31E+00	9.56E-03		-8.93E-01	2.72E-01		Unigene113107_All//gi|15187172|gb|AAK91322.1|AC090441_4//9.00E-39//Putative transposase protein [Oryza sativa Japonica Group]		Unigene113107_All//2.00E-87//ES819408				nr +
Unigene113110_All	231	0.00	0.00	0.00	0.00	0.60	0.00	1.10	6.15	0.86	null	null		null	null		9.22E+00	3.20E-01		null	null		2.49E+00	2.76E-05	Up	-3.49E-01	7.67E-01								
Unigene113111_All	412	0.25	0.98	0.00	0.24	0.00	0.11	0.00	3.45	0.10	1.95E+00	1.44E-01		-7.99E+00	3.97E-01		-7.92E+00	3.16E-01		-1.14E+00	6.78E-01		1.18E+01	4.93E-10	Up	6.60E+00	6.47E-01		Unigene113111_All//gi|18396059|ref|NP_564263.1|//1.00E-21//zinc finger (C3HC4-type RING finger) family protein [Arabidopsis thaliana] >gi|9295734|gb|AAF87040.1|AC006535_18 T24P13.19 [Arabidopsis thaliana] >gi|13605625|gb|AAK32806.1|AF361638_1 At1g26800/T24P13_21 [Arabidopsis thaliana] >gi|15450675|gb|AAK96609.1| At1g26800/T24P13_21 [Arabidopsis thaliana] >gi|15777873|gb|AAL05897.1| At1g26800/T24P13_21 [Arabidopsis thaliana]	Unigene113111_All//3.00E-21//AT1G26800| zinc finger (C3HC4-type RING finger) family protein	Unigene113111_All//0.00E+00//DW498401			GO:0046914	nr -
Unigene113112_All	809	0.00	0.00	0.00	0.00	0.00	0.22	1.41	7.39	39.15	null	null		null	null		null	null		7.80E+00	1.95E-01		2.39E+00	3.45E-19	Up	4.79E+00	4.18E-186	Up	Unigene113112_All//gi|224143055|ref|XP_002324836.1|//1.00E-22//2-oxoglutarate-dependent dioxygenase [Populus trichocarpa] >gi|222866270|gb|EEF03401.1| 2-oxoglutarate-dependent dioxygenase [Populus trichocarpa]	Unigene113112_All//2.00E-17//AT4G03070| AOP1; oxidoreductase, acting on paired donors, with incorporation or reduction of molecular oxygen, 2-oxoglutarate as one donor, and incorporation of one atom each of oxygen into both donors	Unigene113112_All//0.00E+00//TC137085		ko00904|Diterpenoid biosynthesis	GO:0016491//GO:0044464	nr -
Unigene113115_All	291	216.12	143.48	234.23	172.89	197.14	152.87	10.03	11.49	21.22	-5.91E-01	7.76E-19		1.16E-01	1.11E-01		1.89E-01	6.23E-03		-1.78E-01	2.37E-02		1.97E-01	5.74E-01		1.08E+00	3.78E-07	Up			Unigene113115_All//6.00E-132//TC136859				
Unigene113122_All	223	0.00	0.00	0.00	0.00	0.00	0.00	7.02	4.31	1.25	null	null		null	null		null	null		null	null		-7.03E-01	1.40E-01		-2.49E+00	5.08E-06	Down			Unigene113122_All//1.00E-06//ES844746				ESTscan +
Unigene113132_All	380	0.00	0.00	0.00	0.00	0.00	0.00	0.11	2.09	0.00	null	null		null	null		null	null		null	null		4.23E+00	1.05E-04	Up	-6.80E+00	6.16E-01								
Unigene113139_All	358	10.67	0.28	2.97	37.43	18.37	24.47	0.83	1.63	0.45	-5.24E+00	3.06E-19	Down	-1.84E+00	2.98E-07	Down	-1.03E+00	8.30E-13	Down	-6.13E-01	4.21E-05		9.83E-01	2.59E-01		-8.93E-01	4.71E-01				Unigene113139_All//8.00E-64//TC162673				
Unigene113147_All	417	0.00	0.00	0.00	0.00	0.00	0.00	0.00	4.11	0.10	null	null		null	null		null	null		null	null		1.20E+01	4.48E-12	Up	6.58E+00	6.47E-01				Unigene113147_All//2.00E-27//TC153523				
Unigene113174_All	224	0.00	0.68	0.00	0.22	0.00	0.20	0.00	2.61	0.18	9.41E+00	2.36E-01		null	null		-7.80E+00	5.61E-01		-1.42E-01	9.55E-01		1.14E+01	2.81E-04	Up	7.47E+00	6.47E-01		Unigene113174_All//gi|260513724|gb|ACX42574.1|//8.00E-24//CONSTANS-like zinc finger protein [Glycine max]	Unigene113174_All//4.00E-14//AT3G21880| zinc finger (B-box type) family protein		C2C2-CO-like	ko04712|Circadian rhythm - plant	GO:0044464//GO:0046914	nr +
Unigene113185_All	304	0.00	0.00	0.00	0.00	0.00	0.00	2.50	6.74	2.75	null	null		null	null		null	null		null	null		1.43E+00	6.34E-04	Up	1.37E-01	8.36E-01		Unigene113185_All//gi|55168340|gb|AAV44205.1|//4.00E-14//unknow protein [Oryza sativa Japonica Group]		Unigene113185_All//4.00E-22//TC160492				nr -
Unigene113186_All	737	0.00	0.00	0.00	0.00	0.00	0.00	0.00	5.90	0.00	null	null		null	null		null	null		null	null		1.25E+01	1.33E-30	Up	null	null		Unigene113186_All//gi|8250119|emb|CAB93512.1|//4.00E-07//HSP17.7-a protein [Brassica oleracea var. alboglabra]	Unigene113186_All//1.00E-06//AT5G12030| AT-HSP17.6A (ARABIDOPSIS THALIANA HEAT SHOCK PROTEIN 17.6A); unfolded protein binding					nr -
Unigene113189_All	553	0.00	0.00	0.00	0.00	0.00	0.00	0.54	2.49	0.00	null	null		null	null		null	null		null	null		2.22E+00	1.31E-04	Up	-9.06E+00	1.58E-02		Unigene113189_All//gi|15237805|ref|NP_197756.1|//4.00E-40//LCB2; protein binding / serine C-palmitoyltransferase [Arabidopsis thaliana] >gi|79328575|ref|NP_001031932.1| LCB2; protein binding / serine C-palmitoyltransferase [Arabidopsis thaliana] >gi|8809693|dbj|BAA97234.1| serine palmitoyltransferase [Arabidopsis thaliana]	Unigene113189_All//2.00E-41//AT5G23670| LCB2; protein binding / serine C-palmitoyltransferase	Unigene113189_All//4.00E-08//gi|84169650|gb|DR458298.1|DR458298		ko00600|Sphingolipid metabolism	GO:0005488//GO:0006670//GO:0009639//GO:0016454//GO:0043231//GO:0048229	nr -
Unigene11319_All	4363	16.50	7.51	10.62	25.23	20.95	16.48	13.15	9.96	24.40	-1.13E+00	4.80E-64	Down	-6.36E-01	2.00E-23		-2.69E-01	2.35E-09		-6.15E-01	2.14E-37		-4.01E-01	9.65E-11		8.92E-01	2.24E-79		Unigene11319_All//gi|224128678|ref|XP_002329063.1|//0.00E+00//aminophospholipid ATPase [Populus trichocarpa] >gi|222839734|gb|EEE78057.1| aminophospholipid ATPase [Populus trichocarpa]	Unigene11319_All//0.00E+00//AT1G17500| ATPase, coupled to transmembrane movement of ions, phosphorylative mechanism	Unigene11319_All//0.00E+00//TC144897			GO:0005548//GO:0006869//GO:0009206//GO:0031224//GO:0032559//GO:0042625//GO:0043231//GO:0046914	nr +
Unigene113199_All	206	27.94	15.74	21.46	45.95	40.95	52.18	99.13	48.09	59.76	-8.28E-01	8.33E-04		-3.81E-01	1.50E-01		-1.66E-01	4.39E-01		1.84E-01	3.71E-01		-1.04E+00	5.52E-20	Down	-7.30E-01	1.20E-11		Unigene113199_All//gi|209967441|gb|ACJ02349.1|//2.00E-12//60S ribosomal protein L35a [Vernicia fordii]	Unigene113199_All//1.00E-13//AT1G74270| 60S ribosomal protein L35a (RPL35aC)	Unigene113199_All//1.00E-79//TC155009		ko03010|Ribosome	GO:0005198//GO:0010467//GO:0015934//GO:0022613	nr +

Unigene11320_All	1165	2.47	1.87	5.58	6.88	7.86	5.58	3.19	1.44	2.39	-4.01E-01	2.87E-01		1.17E+00	1.82E-06	Up	1.92E-01	3.84E-01		-3.02E-01	1.71E-01		-1.15E+00	6.53E-05	Down	-4.16E-01	1.38E-01		Unigene11320_All//gi|160892401|gb|ABX52080.1|//4.00E-84//RRP6-like protein 2 [Arabidopsis thaliana]	Unigene11320_All//6.00E-70//AT5G35910| 3'-5' exonuclease domain-containing protein / helicase and RNase D C-terminal domain-containing protein / HRDC domain-containing protein	Unigene11320_All//0.00E+00//gi|109857934|gb|DW486913.1|DW486913		ko03018|RNA degradation	GO:0004527//GO:0005488//GO:0034641//GO:0043231	nr -
Unigene113207_All	350	0.00	0.00	0.00	0.00	0.00	0.00	0.24	4.54	0.80	null	null		null	null		null	null		null	null		4.23E+00	6.26E-09	Up	1.72E+00	2.30E-01		Unigene113207_All//gi|145408595|ref|YP_001152219.1|//4.00E-10//ORF58f [Pinus koraiensis] >gi|145048844|gb|ABP35460.1| ORF58f [Pinus koraiensis]		Unigene113207_All//8.00E-24//TC150009			GO:0009536	nr +
Unigene113210_All	230	0.00	0.00	0.00	0.00	0.00	0.00	0.00	2.91	0.69	null	null		null	null		null	null		null	null		1.15E+01	7.64E-05	Up	9.44E+00	1.40E-01		Unigene113210_All//gi|54399516|gb|AAV34148.1|//5.00E-11//EF-1 alpha-like protein [Helicosporidium sp. ex Simulium jonesi]					GO:0008135//GO:0017111//GO:0032561	nr -
Unigene113211_All	246	0.00	0.00	0.00	0.00	0.00	0.00	0.17	2.89	0.81	null	null		null	null		null	null		null	null		4.07E+00	3.51E-04	Up	2.24E+00	2.49E-01								
Unigene113212_All	464	0.00	0.00	0.00	0.00	0.00	0.00	2.73	6.76	0.09	null	null		null	null		null	null		null	null		1.31E+00	5.51E-05	Up	-4.99E+00	3.13E-08	Down	Unigene113212_All//gi|300137035|gb|EFJ04209.1|//2.00E-17//hypothetical protein SELMODRAFT_139061 [Selaginella moellendorffii]		Unigene113212_All//1.00E-41//TC148521				nr +
Unigene11323_All	1486	7.50	9.31	9.53	15.29	13.49	6.44	8.08	6.27	3.54	3.12E-01	4.44E-02		3.46E-01	2.94E-02		-1.81E-01	1.44E-01		-1.25E+00	9.83E-26	Down	-3.66E-01	1.39E-02		-1.19E+00	3.12E-15	Down	Unigene11323_All//gi|6056391|gb|AAF02855.1|AC009324_4//2.00E-22//Similar to retrotransposon proteins [Arabidopsis thaliana]	Unigene11323_All//3.00E-05//AT5G48050| unknown protein	Unigene11323_All//2.00E-86//TC176628				nr -
Unigene113231_All	380	0.00	0.53	0.84	0.52	0.00	0.00	0.22	6.49	6.08	9.06E+00	1.37E-01		9.72E+00	4.53E-02		-9.03E+00	8.46E-02		-9.03E+00	1.34E-01		4.87E+00	1.04E-14	Up	4.77E+00	6.01E-14	Up							
Unigene113237_All	270	0.00	0.00	0.00	0.00	0.00	0.00	0.78	3.25	4.28	null	null		null	null		null	null		null	null		2.05E+00	5.68E-03		2.45E+00	1.69E-04	Up							
Unigene11324_All	278	3.76	8.75	16.09	10.57	8.79	6.01	1.37	1.95	0.72	1.22E+00	3.60E-03		2.10E+00	7.43E-10	Up	-2.66E-01	5.14E-01		-8.15E-01	2.52E-02		5.14E-01	5.89E-01		-9.34E-01	3.80E-01				Unigene11324_All//4.00E-16//TC176628				
Unigene113240_All	456	0.00	0.00	0.00	0.11	0.00	0.00	0.19	2.20	0.61	null	null		null	null		-6.77E+00	5.61E-01		-6.77E+00	6.42E-01		3.57E+00	3.08E-05	Up	1.72E+00	2.30E-01				Unigene113240_All//1.00E-87//gi|73848529|gb|DT457549.1|DT457549				
Unigene113251_All	425	0.00	0.00	0.00	0.00	0.00	0.00	3.28	14.26	1.97	null	null		null	null		null	null		null	null		2.12E+00	6.79E-17	Up	-7.38E-01	1.28E-01		Unigene113251_All//gi|238014838|gb|ACR38454.1|//1.00E-13//unknown [Zea mays]		Unigene113251_All//4.00E-23//ES850594				nr +
Unigene11326_All	5065	3.06	6.24	4.46	6.16	5.28	4.13	6.89	7.53	5.05	1.03E+00	2.34E-24	Up	5.44E-01	4.55E-06		-2.21E-01	2.26E-02		-5.77E-01	6.88E-10		1.28E-01	1.39E-01		-4.48E-01	1.65E-08		Unigene11326_All//gi|7444442|pir||T02087//0.00E+00//gag/pol polyprotein - maize retrotransposon Hopscotch >gi|531389|gb|AAA57005.1| copia-like retrotransposon Hopscotch polyprotein [Zea mays]	Unigene11326_All//2.00E-82//AT4G23160| protein kinase family protein	Unigene11326_All//0.00E+00//TC165869				nr -
Unigene113268_All	219	0.00	0.23	0.00	0.00	0.00	0.00	0.77	6.49	0.73	7.85E+00	6.20E-01		null	null		null	null		null	null		3.07E+00	2.15E-06	Up	-8.58E-02	9.66E-01				Unigene113268_All//2.00E-07//TC165900				
Unigene11327_All	484	3.68	7.43	3.41	8.34	8.18	7.56	7.86	6.48	3.79	1.02E+00	1.97E-03		-1.08E-01	8.24E-01		-2.70E-02	9.36E-01		-1.41E-01	6.90E-01		-2.80E-01	3.68E-01		-1.05E+00	1.29E-04	Down	Unigene11327_All//gi|41056592|gb|AAR98738.1|//2.00E-55//signal peptide peptidase [Galega orientalis]	Unigene11327_All//3.00E-55//AT2G03120| ATSPP (ARABIDOPSIS SIGNAL PEPTIDE PEPTIDASE); aspartic-type endopeptidase	Unigene11327_All//0.00E+00//TC140648			GO:0004175//GO:0031224//GO:0031410	nr -
Unigene113277_All	207	0.00	0.00	0.00	0.24	0.00	0.00	4.09	7.47	0.38	null	null		null	null		-7.91E+00	5.61E-01		-7.91E+00	6.42E-01		8.71E-01	6.92E-02		-3.41E+00	1.35E-04	Down							
Unigene11328_All	500	1.05	2.33	1.28	1.20	1.93	1.45	18.27	20.23	8.29	1.16E+00	7.15E-02		2.89E-01	7.25E-01		6.90E-01	3.61E-01		2.74E-01	7.43E-01		1.47E-01	4.24E-01		-1.14E+00	4.61E-11	Down	Unigene11328_All//gi|41056592|gb|AAR98738.1|//5.00E-37//signal peptide peptidase [Galega orientalis]	Unigene11328_All//3.00E-33//AT2G03120| ATSPP (ARABIDOPSIS SIGNAL PEPTIDE PEPTIDASE); aspartic-type endopeptidase	Unigene11328_All//0.00E+00//TC131602			GO:0004175//GO:0031224//GO:0031410	nr +
Unigene113286_All	220	0.24	0.69	0.48	0.45	1.88	1.64	0.77	5.13	0.54	1.54E+00	5.32E-01		1.03E+00	7.04E-01		2.05E+00	1.47E-01		1.86E+00	2.20E-01		2.74E+00	9.94E-05	Up	-5.01E-01	7.47E-01								
Unigene11329_All	599	1.75	2.37	1.78	1.75	3.33	2.04	7.34	4.81	2.46	4.39E-01	4.26E-01		2.55E-02	9.89E-01		9.33E-01	4.16E-02		2.21E-01	7.22E-01		-6.09E-01	1.86E-02		-1.58E+00	6.31E-09	Down	Unigene11329_All//gi|297814610|ref|XP_002875188.1|//3.00E-62//signal peptide peptidase family protein [Arabidopsis lyrata subsp. lyrata] >gi|297321026|gb|EFH51447.1| signal peptide peptidase family protein [Arabidopsis lyrata subsp. lyrata]	Unigene11329_All//4.00E-55//AT2G03120| ATSPP (ARABIDOPSIS SIGNAL PEPTIDE PEPTIDASE); aspartic-type endopeptidase	Unigene11329_All//0.00E+00//TC140648			GO:0004175//GO:0031224//GO:0031410	nr -
Unigene113295_All	228	0.00	0.00	0.00	0.00	0.00	0.00	21.51	1.28	0.00	null	null		null	null		null	null		null	null		-4.07E+00	5.20E-26	Down	-1.44E+01	4.60E-36	Down	Unigene113295_All//gi|15237245|ref|NP_197105.1|//1.00E-08//3-oxo-5-alpha-steroid 4-dehydrogenase family protein / steroid 5-alpha-reductase family protein [Arabidopsis thaliana] >gi|14190513|gb|AAK55737.1|AF380656_1 AT5g16010/F1N13_150 [Arabidopsis thaliana] >gi|9755647|emb|CAC01800.1| steroid 5alpha-reductase-like protein [Arabidopsis thaliana] >gi|24797018|gb|AAN64521.1| At5g16010/F1N13_150 [Arabidopsis thaliana]	Unigene113295_All//8.00E-10//AT5G16010| 3-oxo-5-alpha-steroid 4-dehydrogenase family protein / steroid 5-alpha-reductase family protein					nr +
Unigene11330_All	1427	0.81	2.24	1.27	4.01	3.35	2.72	4.80	3.95	1.65	1.47E+00	6.16E-05	Up	6.53E-01	2.02E-01		-2.59E-01	3.26E-01		-5.61E-01	2.42E-02		-2.80E-01	1.95E-01		-1.54E+00	4.89E-13	Down	Unigene11330_All//gi|18395487|ref|NP_565294.1|//1.00E-161//ATSPP (ARABIDOPSIS SIGNAL PEPTIDE PEPTIDASE); aspartic-type endopeptidase [Arabidopsis thaliana] >gi|3548818|gb|AAC34490.1| expressed protein [Arabidopsis thaliana]	Unigene11330_All//7.00E-154//AT2G03120| ATSPP (ARABIDOPSIS SIGNAL PEPTIDE PEPTIDASE); aspartic-type endopeptidase	Unigene11330_All//0.00E+00//TC140648				nr +
Unigene113310_All	265	0.00	0.00	0.00	0.38	0.65	0.17	0.16	3.15	1.05	null	null		null	null		7.89E-01	7.71E-01		-1.14E+00	6.78E-01		4.31E+00	5.68E-05	Up	2.72E+00	9.95E-02				Unigene113310_All//1.00E-09//TC152940				
Unigene113312_All	217	0.00	0.00	0.00	0.00	0.00	0.00	0.00	3.47	0.00	null	null		null	null		null	null		null	null		1.18E+01	2.07E-05	Up	null	null		Unigene113312_All//gi|297794223|ref|XP_002864996.1|//3.00E-13//60S ribosomal protein L26 [Arabidopsis lyrata subsp. lyrata] >gi|297310831|gb|EFH41255.1| 60S ribosomal protein L26 [Arabidopsis lyrata subsp. lyrata]	Unigene113312_All//4.00E-13//AT5G67510| 60S ribosomal protein L26 (RPL26B)			ko03010|Ribosome	GO:0005198//GO:0010467//GO:0033279//GO:0043231	nr -
Unigene113315_All	370	0.00	0.68	0.00	0.67	0.00	0.37	1.26	6.10	2.26	9.42E+00	7.88E-02		null	null		-9.39E+00	4.19E-02		-8.78E-01	6.17E-01		2.28E+00	2.69E-07	Up	8.47E-01	2.03E-01		Unigene113315_All//gi|254212161|gb|ACT65738.1|//6.00E-14//QM [Nicotiana benthamiana]	Unigene113315_All//3.00E-12//AT1G26910| 60S ribosomal protein L10 (RPL10B)	Unigene113315_All//4.00E-112//CO093073		ko03010|Ribosome	GO:0005198//GO:0010467//GO:0030529	nr +
Unigene11333_All	2747	4.42	4.91	4.89	10.16	9.25	10.74	33.38	24.22	14.00	1.51E-01	3.68E-01		1.45E-01	4.00E-01		-1.35E-01	2.44E-01		8.03E-02	5.20E-01		-4.63E-01	1.59E-21		-1.25E+00	8.64E-120	Down	Unigene11333_All//gi|15215736|gb|AAK91413.1|//0.00E+00//AT5g19690/T29J13_110 [Arabidopsis thaliana] >gi|16974315|gb|AAL31142.1| AT5g19690/T29J13_110 [Arabidopsis thaliana]	Unigene11333_All//0.00E+00//AT5G19690| STT3A (STAUROSPORIN AND TEMPERATURE SENSITIVE 3-LIKE A); oligosaccharyl transferase	Unigene11333_All//0.00E+00//gi|73862681|gb|DT465420.1|DT465420		ko04141|Protein processing in endoplasmic reticulum//ko00510|N-Glycan biosynthesis	GO:0004576//GO:0006464//GO:0006970//GO:0016020//GO:0043231	nr +
Unigene113337_All	212	0.00	0.00	0.00	0.00	0.00	0.00	0.40	3.94	0.00	null	null		null	null		null	null		null	null		3.31E+00	3.10E-04	Up	-8.64E+00	3.60E-01		Unigene113337_All//sp|P23403|RS20_XENLA//2.00E-07//40S ribosomal protein S20 OS=Xenopus laevis GN=rps20 PE=3 SV=1	Unigene113337_All//9.00E-05//AT5G62300| 40S ribosomal protein S20 (RPS20C)			ko03010|Ribosome		swissprot +
Unigene113341_All	244	0.00	0.00	0.00	0.00	0.00	0.00	2.77	1.03	11.10	null	null		null	null		null	null		null	null		-1.43E+00	7.84E-02		2.00E+00	1.20E-07	Up							
Unigene113354_All	544	0.00	0.00	0.00	0.00	0.00	0.00	1.01	3.30	0.00	null	null		null	null		null	null		null	null		1.71E+00	2.41E-04	Up	-9.98E+00	2.81E-04	Down	Unigene113354_All//gi|301117174|ref|XP_002906315.1|//1.00E-32//60S ribosomal protein L35a-4 [Phytophthora infestans T30-4] >gi|66270185|gb|AAY43422.1| ribosomal protein L35 [Phytophthora infestans] >gi|262107664|gb|EEY65716.1| 60S ribosomal protein L35a-4 [Phytophthora infestans T30-4]	Unigene113354_All//1.00E-29//AT3G55750| 60S ribosomal protein L35a (RPL35aD)			ko03010|Ribosome	GO:0005198//GO:0010467//GO:0015934//GO:0022613	nr -
Unigene11336_All	2972	1.43	1.18	2.19	3.08	2.39	2.77	8.28	5.74	2.45	-2.78E-01	3.63E-01		6.16E-01	1.05E-02		-3.70E-01	4.93E-02		-1.57E-01	4.76E-01		-5.29E-01	6.36E-08		-1.76E+00	1.74E-53	Down	Unigene11336_All//gi|18419993|ref|NP_568380.1|//0.00E+00//STT3A (STAUROSPORIN AND TEMPERATURE SENSITIVE 3-LIKE A); oligosaccharyl transferase [Arabidopsis thaliana]	Unigene11336_All//0.00E+00//AT5G19690| STT3A (STAUROSPORIN AND TEMPERATURE SENSITIVE 3-LIKE A); oligosaccharyl transferase	Unigene11336_All//0.00E+00//TC142353		ko04141|Protein processing in endoplasmic reticulum//ko00510|N-Glycan biosynthesis	GO:0004576//GO:0006464//GO:0006970//GO:0016020//GO:0043231	nr +
Unigene113364_All	289	0.18	0.18	0.37	0.00	0.00	0.00	2.19	3.18	0.00	-4.62E-02	9.86E-01		1.03E+00	7.04E-01		null	null		null	null		5.36E-01	4.18E-01		-1.11E+01	7.11E-05	Down							
Unigene113367_All	254	0.00	0.00	0.00	0.00	0.00	0.00	2.50	2.63	0.00	null	null		null	null		null	null		null	null		7.65E-02	9.37E-01		-1.13E+01	7.11E-05	Down	Unigene113367_All//gi|2894308|emb|CAA11269.1|//1.00E-40//polyubiquitin [Nicotiana tabacum]	Unigene113367_All//6.00E-41//AT5G03240| UBQ3 (POLYUBIQUITIN 3); protein binding	Unigene113367_All//2.00E-42//BM359461			GO:0043231	nr -
Unigene113373_All	427	0.00	0.00	0.00	0.00	0.00	0.00	1.49	1.66	0.00	null	null		null	null		null	null		null	null		1.64E-01	8.24E-01		-1.05E+01	7.11E-05	Down							ESTscan -
Unigene113377_All	535	0.00	0.00	0.00	0.00	0.00	0.00	0.55	2.58	0.00	null	null		null	null		null	null		null	null		2.22E+00	1.31E-04	Up	-9.11E+00	1.58E-02		Unigene113377_All//gi|301092807|ref|XP_002997255.1|//4.00E-34//Mitochondrial Carrier (MC) Family [Phytophthora infestans T30-4] >gi|262111436|gb|EEY69488.1| Mitochondrial Carrier (MC) Family [Phytophthora infestans T30-4]	Unigene113377_All//1.00E-32//AT5G14040| mitochondrial phosphate transporter				GO:0016020	nr -
Unigene113384_All	505	0.00	0.00	0.00	0.00	0.00	0.00	1.09	2.48	0.00	null	null		null	null		null	null		null	null		1.19E+00	3.01E-02		-1.01E+01	2.81E-04	Down	Unigene113384_All//gi|159477048|ref|XP_001696623.1|//6.00E-11//serine/threonine phosphatase, family 2C [Chlamydomonas reinhardtii] >gi|158282848|gb|EDP08600.1| serine/threonine phosphatase, family 2C [Chlamydomonas reinhardtii]	Unigene113384_All//2.00E-09//AT4G33500| protein phosphatase 2C-related / PP2C-related				GO:0003824//GO:0009536	nr +
Unigene11341_All	2782	22.46	84.20	51.49	25.34	41.21	53.18	23.10	26.04	34.15	1.91E+00	0.00E+00	Up	1.20E+00	4.44E-134	Up	7.02E-01	6.33E-54		1.07E+00	1.08E-127	Up	1.73E-01	2.40E-03		5.64E-01	2.40E-32		Unigene11341_All//gi|30689342|ref|NP_850393.1|//1.00E-142//INT1 (INOSITOL TRANSPORTER 1); carbohydrate transmembrane transporter/ myo-inositol:hydrogen symporter/ sugar:hydrogen symporter [Arabidopsis thaliana] >gi|75331205|sp|Q8VZR6.1|INT1_ARATH RecName: Full=Probable inositol transporter 1 >gi|84617967|emb|CAJ00303.1| inositol transporter 1 [Arabidopsis thaliana]	Unigene11341_All//9.00E-113//AT2G43330| INT1 (INOSITOL TRANSPORTER 1); carbohydrate transmembrane transporter/ myo-inositol:hydrogen symporter/ sugar:hydrogen symporter	Unigene11341_All//0.00E+00//TC168474			GO:0006810//GO:0022857//GO:0031224	nr -
Unigene113412_All	844	0.00	0.00	0.00	0.00	0.00	0.00	2.50	5.30	0.66	null	null		null	null		null	null		null	null		1.08E+00	3.06E-05	Up	-1.92E+00	5.97E-06	Down	Unigene113412_All//gi|253761439|ref|XP_002489117.1|//3.00E-14//hypothetical protein SORBIDRAFT_0057s002150 [Sorghum bicolor] >gi|241947368|gb|EES20513.1| hypothetical protein SORBIDRAFT_0057s002150 [Sorghum bicolor]		Unigene113412_All//5.00E-27//ES850594				nr +
Unigene113414_All	1149	0.41	0.09	0.00	0.00	0.15	0.16	1.40	3.64	1.04	-2.22E+00	7.77E-02		-8.68E+00	8.66E-03		7.23E+00	2.27E-01		7.30E+00	1.95E-01		1.38E+00	7.10E-07	Up	-4.27E-01	3.59E-01								

Unigene113417_All	290	0.00	0.00	0.00	0.00	0.00	0.00	0.15	2.16	2.20	null	null		null	null		null	null		null	null		3.89E+00	1.15E-03		3.91E+00	7.66E-04	Up	Unigene113417_All//gi|297610014|ref|NP_001064026.2|//4.00E-09//Os10g0106900 [Oryza sativa Japonica Group] >gi|255679164|dbj|BAF25940.2| Os10g0106900 [Oryza sativa Japonica Group]					GO:0003676//GO:0006259//GO:0006810//GO:0016020//GO:0016209//GO:0046872	nr +
Unigene113423_All	280	0.19	0.36	0.00	0.00	0.00	0.00	333.71	379.00	754.80	9.53E-01	7.27E-01		-7.55E+00	6.30E-01		null	null		null	null		1.84E-01	5.52E-05		1.18E+00	4.65E-247	Up	Unigene113423_All//gi|242070913|ref|XP_002450733.1|//4.00E-16//hypothetical protein SORBIDRAFT_05g016477 [Sorghum bicolor] >gi|241936576|gb|EES09721.1| hypothetical protein SORBIDRAFT_05g016477 [Sorghum bicolor]		Unigene113423_All//4.00E-56//ES810795				nr -
Unigene113427_All	367	0.00	0.00	0.00	0.00	0.00	0.00	1.61	2.73	0.00	null	null		null	null		null	null		null	null		7.61E-01	2.28E-01		-1.07E+01	1.41E-04	Down	Unigene113427_All//gi|160330969|ref|XP_001712192.1|//2.00E-37//ef2 [Hemiselmis andersenii] >gi|159765639|gb|ABW97867.1| ef2 [Hemiselmis andersenii]	Unigene113427_All//1.00E-24//AT1G56070| LOS1; copper ion binding / translation elongation factor/ translation factor, nucleic acid binding	Unigene113427_All//1.00E-07//gi|78340849|gb|DT561123.1|DT561123			GO:0008135//GO:0017111//GO:0032561	nr +
Unigene11343_All	1871	2.68	1.68	1.59	6.04	6.13	3.81	5.31	3.02	2.17	-6.77E-01	1.12E-02		-7.52E-01	7.07E-03		2.02E-02	9.32E-01		-6.64E-01	5.52E-05		-8.16E-01	5.44E-07		-1.29E+00	2.45E-14	Down	Unigene11343_All//gi|83319215|dbj|BAE53712.1|//2.00E-72//At3g21480 [Arabidopsis thaliana]	Unigene11343_All//8.00E-73//AT3G21480| FUNCTIONS IN: molecular_function unknown; INVOLVED IN: biological_process unknown; LOCATED IN: intracellular; CONTAINS InterPro DOMAIN/s: BRCT (InterPro:IPR001357); BEST Arabidopsis thaliana protein match is: transcription coactivator (TAIR:AT4G03130.1); Has 489 Blast hits to 455 proteins in 115 species: Archae - 2; Bacteria - 37; Metazoa - 270; Fungi - 30; Plants - 45; Viruses - 0; Other Eukaryotes - 105 (source: NCBI BLink).	Unigene11343_All//0.00E+00//ES830964				nr -
Unigene113433_All	503	0.00	0.00	0.00	0.00	0.00	0.09	0.50	11.72	0.32	null	null		null	null		null	null		6.49E+00	7.01E-01		4.54E+00	2.65E-33	Up	-6.71E-01	6.06E-01		Unigene113433_All//gi|224085625|ref|XP_002307640.1|//6.00E-42//AP2/ERF domain-containing transcription factor [Populus trichocarpa] >gi|222857089|gb|EEE94636.1| AP2/ERF domain-containing transcription factor [Populus trichocarpa]	Unigene113433_All//8.00E-20//AT3G23230| ethylene-responsive factor, putative	Unigene113433_All//1.00E-171//DT461789	AP2-EREBP		GO:0000160//GO:0001071//GO:0006351//GO:0009743//GO:0010468//GO:0043231	nr -
Unigene11344_All	3341	2.27	1.71	1.43	3.03	3.28	3.26	3.80	1.81	1.63	-4.06E-01	5.91E-02		-6.63E-01	2.59E-03		1.15E-01	5.83E-01		1.06E-01	6.38E-01		-1.07E+00	3.96E-13	Down	-1.22E+00	1.48E-16	Down	Unigene11344_All//gi|83319215|dbj|BAE53712.1|//1.00E-55//At3g21480 [Arabidopsis thaliana]	Unigene11344_All//2.00E-48//AT3G21480| FUNCTIONS IN: molecular_function unknown; INVOLVED IN: biological_process unknown; LOCATED IN: intracellular; CONTAINS InterPro DOMAIN/s: BRCT (InterPro:IPR001357); BEST Arabidopsis thaliana protein match is: transcription coactivator (TAIR:AT4G03130.1); Has 489 Blast hits to 455 proteins in 115 species: Archae - 2; Bacteria - 37; Metazoa - 270; Fungi - 30; Plants - 45; Viruses - 0; Other Eukaryotes - 105 (source: NCBI BLink).	Unigene11344_All//0.00E+00//ES825416				nr -
Unigene113444_All	479	0.00	0.00	0.00	0.00	0.14	0.00	19.77	4.89	5.49	null	null		null	null		7.17E+00	6.14E-01		null	null		-2.02E+00	1.69E-24	Down	-1.85E+00	1.28E-22	Down	Unigene113444_All//gi|124361039|gb|ABN09011.1|//2.00E-36//General substrate transporter; Sugar transporter superfamily [Medicago truncatula]	Unigene113444_All//2.00E-37//AT4G04750| carbohydrate transmembrane transporter/ sugar:hydrogen symporter	Unigene113444_All//4.00E-125//gi|48820844|gb|CO122157.1|CO122157			GO:0005215//GO:0006810	nr -
Unigene11345_All	1563	8.80	6.39	6.95	14.37	13.79	14.74	15.69	6.12	4.82	-4.63E-01	2.26E-03		-3.41E-01	3.55E-02		-5.96E-02	6.78E-01		3.60E-02	7.89E-01		-1.36E+00	9.82E-36	Down	-1.70E+00	3.78E-51	Down	Unigene11345_All//gi|186528371|ref|NP_198994.2|//9.00E-62//CIP1 (COP1-INTERACTIVE PROTEIN 1); protein binding [Arabidopsis thaliana]	Unigene11345_All//5.00E-63//AT5G41790| CIP1 (COP1-INTERACTIVE PROTEIN 1); protein binding	Unigene11345_All//0.00E+00//gi|84146467|gb|DN760709.1|DN760709				nr -
Unigene113454_All	406	0.00	0.00	0.00	0.00	0.00	0.00	1.56	1.54	0.00	null	null		null	null		null	null		null	null		-1.66E-02	1.02E+00		-1.06E+01	7.11E-05	Down							ESTscan +
Unigene11346_All	320	0.00	0.32	0.17	0.16	0.22	0.28	2.91	1.31	0.37	8.31E+00	3.89E-01		7.38E+00	6.39E-01		4.67E-01	9.60E-01		8.59E-01	7.96E-01		-1.15E+00	7.87E-02		-2.96E+00	1.72E-04	Down	Unigene11346_All//gi|186528371|ref|NP_198994.2|//2.00E-11//CIP1 (COP1-INTERACTIVE PROTEIN 1); protein binding [Arabidopsis thaliana]	Unigene11346_All//7.00E-13//AT5G41790| CIP1 (COP1-INTERACTIVE PROTEIN 1); protein binding	Unigene11346_All//2.00E-122//gi|21101346|gb|BQ413659.1|BQ413659			GO:0010038//GO:0016020//GO:0043231	nr -
Unigene113460_All	673	0.00	0.00	0.00	0.00	0.00	0.00	0.38	2.98	0.89	null	null		null	null		null	null		null	null		2.98E+00	1.47E-08	Up	1.24E+00	1.39E-01		Unigene113460_All//gi|55168340|gb|AAV44205.1|//5.00E-28//unknow protein [Oryza sativa Japonica Group]		Unigene113460_All//3.00E-28//TC149394				nr -
Unigene113461_All	220	0.00	0.00	0.00	0.00	0.00	0.00	11.34	1.33	0.00	null	null		null	null		null	null		null	null		-3.09E+00	7.09E-11	Down	-1.35E+01	2.32E-18	Down			Unigene113461_All//1.00E-15//TC134836				
Unigene113464_All	528	0.00	0.00	0.00	0.00	0.00	0.00	3.52	12.19	0.00	null	null		null	null		null	null		null	null		1.79E+00	1.77E-14	Up	-1.18E+01	9.96E-14	Down							
Unigene11347_All	627	0.42	1.13	3.31	4.45	7.52	7.06	3.30	2.27	2.41	1.44E+00	1.06E-01		2.99E+00	5.58E-07	Up	7.58E-01	4.71E-03		6.66E-01	2.14E-02		-5.44E-01	1.85E-01		-4.53E-01	2.48E-01		Unigene11347_All//gi|186528371|ref|NP_198994.2|//5.00E-27//CIP1 (COP1-INTERACTIVE PROTEIN 1); protein binding [Arabidopsis thaliana]	Unigene11347_All//2.00E-22//AT5G41790| CIP1 (COP1-INTERACTIVE PROTEIN 1); protein binding	Unigene11347_All//4.00E-70//TC144925			GO:0016020//GO:0043231	nr +
Unigene113474_All	236	12.86	27.70	11.73	92.66	63.33	24.11	1.07	2.30	0.84	1.11E+00	2.90E-06	Up	-1.32E-01	7.19E-01		-5.49E-01	3.12E-07		-1.94E+00	4.84E-47	Down	1.10E+00	2.36E-01		-3.49E-01	7.67E-01		Unigene113474_All//gi|42571085|ref|NP_973616.1|//2.00E-11//ROT4 (ROTUNDIFOLIA4) [Arabidopsis thaliana] >gi|42822065|tpg|DAA02287.1| TPA_exp: DVL16 [Arabidopsis thaliana] >gi|109134191|gb|ABG25093.1| At2g36985 [Arabidopsis thaliana]	Unigene113474_All//6.00E-13//AT2G36985| ROT4 (ROTUNDIFOLIA4)	Unigene113474_All//4.00E-34//ES850629			GO:0008283//GO:0016020//GO:0048513	nr -
Unigene11348_All	2930	10.84	20.27	11.74	11.43	11.80	10.65	19.90	9.71	6.44	9.03E-01	2.10E-36		1.15E-01	2.84E-01		4.66E-02	7.01E-01		-1.01E-01	3.76E-01		-1.03E+00	9.26E-55	Down	-1.63E+00	8.25E-113	Down	Unigene11348_All//gi|186528371|ref|NP_198994.2|//1.00E-113//CIP1 (COP1-INTERACTIVE PROTEIN 1); protein binding [Arabidopsis thaliana]	Unigene11348_All//2.00E-87//AT5G41790| CIP1 (COP1-INTERACTIVE PROTEIN 1); protein binding	Unigene11348_All//0.00E+00//gi|164252827|gb|ES843361.1|ES843361			GO:0044464	nr +
Unigene113484_All	238	0.00	4.05	1.34	0.42	0.00	0.00	0.00	2.11	2.34	1.20E+01	1.38E-05	Up	1.04E+01	4.53E-02		-8.71E+00	3.16E-01		-8.71E+00	4.04E-01		1.10E+01	1.02E-03		1.12E+01	3.28E-04	Up							
Unigene11349_All	4757	6.09	4.78	4.75	8.86	9.06	9.67	13.42	5.84	3.91	-3.50E-01	5.41E-04		-3.60E-01	5.87E-04		3.17E-02	7.93E-01		1.26E-01	1.58E-01		-1.20E+00	7.38E-76	Down	-1.78E+00	4.26E-140	Down	Unigene11349_All//gi|186528371|ref|NP_198994.2|//1.00E-95//CIP1 (COP1-INTERACTIVE PROTEIN 1); protein binding [Arabidopsis thaliana]	Unigene11349_All//4.00E-85//AT5G41790| CIP1 (COP1-INTERACTIVE PROTEIN 1); protein binding	Unigene11349_All//0.00E+00//TC146762			GO:0044464	nr -
Unigene11350_All	208	0.00	0.00	0.00	0.96	0.66	0.43	2.03	20.09	6.51	null	null		null	null		-5.33E-01	7.49E-01		-1.14E+00	5.70E-01		3.31E+00	7.69E-19	Up	1.68E+00	1.63E-03		Unigene11350_All//gi|224054398|ref|XP_002298240.1|//1.00E-26//ABC transporter family, pleiotropic drug resistance protein [Populus trichocarpa] >gi|222845498|gb|EEE83045.1| ABC transporter family, pleiotropic drug resistance protein [Populus trichocarpa]	Unigene11350_All//1.00E-22//AT1G15210| PDR7 (PLEIOTROPIC DRUG RESISTANCE 7); ATPase, coupled to transmembrane movement of substances	Unigene11350_All//8.00E-103//gi|73859385|gb|DT462124.1|DT462124				nr +
Unigene113507_All	406	0.00	0.00	0.00	0.00	0.00	0.00	0.42	2.57	0.00	null	null		null	null		null	null		null	null		2.63E+00	2.86E-04	Up	-8.70E+00	1.09E-01		Unigene113507_All//gi|247421835|gb|ACS96445.1|//2.00E-27//S18.A ribosomal protein [Jatropha curcas]	Unigene113507_All//1.00E-28//AT4G09800| RPS18C (S18 RIBOSOMAL PROTEIN); RNA binding / nucleic acid binding / structural constituent of ribosome	Unigene113507_All//5.00E-10//ES845901		ko03010|Ribosome	GO:0003676//GO:0005198//GO:0006412//GO:0015935//GO:0016020//GO:0030312//GO:0031981	nr +
Unigene11351_All	281	0.19	0.72	1.52	0.18	1.96	0.96	12.04	41.80	31.47	1.95E+00	3.75E-01		3.03E+00	5.73E-02		3.47E+00	1.22E-02		2.44E+00	2.22E-01		1.80E+00	6.75E-26	Up	1.39E+00	3.96E-14	Up	Unigene11351_All//gi|224054398|ref|XP_002298240.1|//2.00E-43//ABC transporter family, pleiotropic drug resistance protein [Populus trichocarpa] >gi|222845498|gb|EEE83045.1| ABC transporter family, pleiotropic drug resistance protein [Populus trichocarpa]	Unigene11351_All//4.00E-43//AT1G15210| PDR7 (PLEIOTROPIC DRUG RESISTANCE 7); ATPase, coupled to transmembrane movement of substances			ko03040|Spliceosome//ko02010|ABC transporters	GO:0000041//GO:0009526//GO:0009814//GO:0015082//GO:0015604//GO:0015893//GO:0016020//GO:0019762//GO:0032559//GO:0042626//GO:0050832//GO:0052482//GO:0071365	nr -
Unigene11352_All	463	1.24	0.77	1.38	1.08	3.27	4.97	187.29	236.24	557.83	-6.98E-01	4.50E-01		1.51E-01	8.56E-01		1.60E+00	4.07E-03		2.21E+00	3.75E-06	Up	3.35E-01	2.42E-14		1.57E+00	0.00E+00	Up	Unigene11352_All//gi|256538309|gb|ACU82514.1|//2.00E-72//pleiotropic drug resistance protein [Cucumis sativus]	Unigene11352_All//5.00E-73//AT1G15210| PDR7 (PLEIOTROPIC DRUG RESISTANCE 7); ATPase, coupled to transmembrane movement of substances			ko03040|Spliceosome//ko02010|ABC transporters	GO:0000041//GO:0009526//GO:0009814//GO:0015082//GO:0015604//GO:0015893//GO:0016020//GO:0017111//GO:0019762//GO:0032559//GO:0050832//GO:0052482//GO:0071365	nr +
Unigene11353_All	877	0.18	0.58	0.30	0.45	1.45	2.37	136.96	174.52	354.91	1.69E+00	1.34E-01		7.62E-01	6.44E-01		1.68E+00	7.61E-03		2.38E+00	4.55E-06	Up	3.50E-01	2.55E-21		1.37E+00	0.00E+00	Up	Unigene11353_All//gi|225442485|ref|XP_002278313.1|//1.00E-153//PREDICTED: similar to PDR8/PEN3 (PLEIOTROPIC DRUG RESISTANCE8); ATPase, coupled to transmembrane movement of substances / cadmium ion transmembrane transporter [Vitis vinifera]	Unigene11353_All//1.00E-144//AT1G15210| PDR7 (PLEIOTROPIC DRUG RESISTANCE 7); ATPase, coupled to transmembrane movement of substances	Unigene11353_All//0.00E+00//TC177387		ko03040|Spliceosome//ko02010|ABC transporters		nr +
Unigene113535_All	339	0.31	2.69	0.00	1.32	0.61	0.93	1.75	7.77	1.41	3.12E+00	1.14E-03		-8.27E+00	3.97E-01		-1.12E+00	3.15E-01		-5.04E-01	6.49E-01		2.15E+00	6.92E-08	Up	-3.08E-01	6.79E-01				Unigene113535_All//3.00E-51//TC160704				
Unigene113539_All	669	0.00	0.00	0.00	0.15	0.31	0.00	0.13	2.94	0.54	null	null		null	null		1.05E+00	6.13E-01		-7.22E+00	4.03E-01		4.54E+00	2.20E-11	Up	2.08E+00	1.01E-01			Unigene113539_All//1.00E-05//AT4G29090| reverse transcriptase, putative / RNA-dependent DNA polymerase, putative					
Unigene11354_All	4943	2.89	5.29	2.68	5.22	5.25	6.41	101.23	169.58	248.74	8.72E-01	1.47E-15		-1.07E-01	5.44E-01		8.90E-03	9.62E-01		2.97E-01	1.99E-03		7.44E-01	0.00E+00		1.30E+00	0.00E+00	Up	Unigene11354_All//gi|225442485|ref|XP_002278313.1|//0.00E+00//PREDICTED: similar to PDR8/PEN3 (PLEIOTROPIC DRUG RESISTANCE8); ATPase, coupled to transmembrane movement of substances / cadmium ion transmembrane transporter [Vitis vinifera]	Unigene11354_All//0.00E+00//AT1G59870| PEN3 (PENETRATION 3); ATPase, coupled to transmembrane movement of substances / cadmium ion transmembrane transporter	Unigene11354_All//0.00E+00//TC136716		ko03040|Spliceosome//ko02010|ABC transporters	GO:0000041//GO:0009526//GO:0009814//GO:0015082//GO:0015893//GO:0016020//GO:0016887//GO:0019762//GO:0050832//GO:0052482//GO:0071365	nr +
Unigene11355_All	2113	0.10	0.05	0.15	3.35	2.40	4.02	12.39	6.17	27.00	-1.04E+00	5.65E-01		6.11E-01	6.25E-01		-4.83E-01	2.28E-02		2.63E-01	2.32E-01		-1.01E+00	6.73E-24	Down	1.12E+00	1.61E-62	Up	Unigene11355_All//gi|253740260|gb|ACT34764.1|//0.00E+00//subtilisin-like protease preproenzyme [Nicotiana tabacum]	Unigene11355_All//2.00E-146//AT5G67090| subtilase family protein	Unigene11355_All//0.00E+00//TC139051			GO:0008152//GO:0016491//GO:0031410//GO:0070011	nr -
Unigene113550_All	456	0.11	0.33	0.00	0.44	0.91	0.00	0.74	2.02	4.19	1.54E+00	5.32E-01		-6.84E+00	6.30E-01		1.05E+00	4.26E-01		-8.77E+00	1.34E-01		1.44E+00	3.26E-02		2.50E+00	4.21E-07	Up			Unigene113550_All//5.00E-07//TC179376				
Unigene113558_All	492	0.00	0.00	0.00	0.00	0.00	0.00	0.00	2.63	0.00	null	null		null	null		null	null		null	null		1.14E+01	3.67E-09	Up	null	null		Unigene113558_All//gi|1350517|gb|AAB01561.1|//2.00E-06//heat shock protein 17.0 [Picea glauca]	Unigene113558_All//2.00E-05//AT1G07400| 17.8 kDa class I heat shock protein (HSP17.8-CI)					nr -

<table>
<tr><td>Unigene11356_All</td><td>2618</td><td>184.46</td><td>32.38</td><td>88.80</td><td>44.79</td><td>56.48</td><td>34.40</td><td>55.96</td><td>113.00</td><td>65.11</td><td>-2.51E+00</td><td>0.00E+00</td><td>Down</td><td>-1.05E+00</td><td>0.00E+00</td><td>Down</td><td>3.35E-01</td><td>2.60E-18</td><td></td><td>-3.81E-01</td><td>7.00E-17</td><td></td><td>1.01E+00</td><td>1.18E-265</td><td>Up</td><td>2.19E-01</td><td>1.78E-10</td><td></td><td>Unigene11356_All//gi|253740260|gb|ACT34764.1|//0.00E+00//subtilisin-like protease preproenzyme [Nicotiana tabacum]</td><td>Unigene11356_All//3.00E-167//AT5G67090| subtilase family protein</td><td>Unigene11356_All//0.00E+00//TC139051</td><td></td><td></td><td>GO:0004175//GO:0005515//GO:0016628//GO:0019538//GO:0044092</td><td>nr -</td></tr>
<tr><td>Unigene113563_All</td><td>361</td><td>0.00</td><td>0.00</td><td>0.00</td><td>0.00</td><td>0.00</td><td>0.00</td><td>0.23</td><td>5.79</td><td>0.66</td><td>null</td><td>null</td><td></td><td>null</td><td>null</td><td></td><td>null</td><td>null</td><td></td><td>null</td><td>null</td><td></td><td>4.63E+00</td><td>3.28E-12</td><td>Up</td><td>1.50E+00</td><td>3.39E-01</td><td></td><td>Unigene113563_All//gi|145408582|ref|YP_001152206.1|//1.00E-10//ORF137 [Pinus koraiensis] >gi|145048831|gb|ABP35449.1| ORF137 [Pinus koraiensis]</td><td></td><td>Unigene113563_All//1.00E-25//gi|109823694|gb|DW008546.1|DW008546</td><td></td><td></td><td>GO:0009536</td><td>nr +</td></tr>
<tr><td>Unigene113569_All</td><td>261</td><td>246.77</td><td>101.92</td><td>139.96</td><td>363.20</td><td>545.56</td><td>413.57</td><td>455.52</td><td>174.87</td><td>180.11</td><td>-1.28E+00</td><td>6.41E-69</td><td>Down</td><td>-8.18E-01</td><td>2.45E-32</td><td></td><td>5.87E-01</td><td>6.56E-49</td><td></td><td>1.87E-01</td><td>1.59E-04</td><td></td><td>-1.38E+00</td><td>5.46E-175</td><td>Down</td><td>-1.34E+00</td><td>1.54E-171</td><td>Down</td><td>Unigene113569_All//gi|223469631|gb|ACM90156.1|//5.00E-15//40S ribosomal protein S15-like protein [Jatropha curcas]</td><td>Unigene113569_All//1.00E-12//AT5G09500| 40S ribosomal protein S15 (RPS15C)</td><td>Unigene113569_All//4.00E-99//TC171659</td><td></td><td>ko03010|Ribosome</td><td>GO:0003676//GO:0005198//GO:0010467//GO:0033279</td><td>nr -</td></tr>
<tr><td>Unigene11357_All</td><td>804</td><td>0.26</td><td>0.76</td><td>0.99</td><td>1.18</td><td>1.37</td><td>0.90</td><td>1.58</td><td>1.35</td><td>3.62</td><td>1.54E+00</td><td>1.22E-01</td><td></td><td>1.93E+00</td><td>3.42E-02</td><td></td><td>2.19E-01</td><td>7.93E-01</td><td></td><td>-3.89E-01</td><td>6.28E-01</td><td></td><td>-2.23E-01</td><td>6.77E-01</td><td></td><td>1.20E+00</td><td>2.46E-04</td><td>Up</td><td>Unigene11357_All//gi|22328551|ref|NP_192745.2|//2.00E-21//KH domain-containing protein [Arabidopsis thaliana]</td><td>Unigene11357_All//1.00E-11//AT4G10070| KH domain-containing protein</td><td>Unigene11357_All//1.00E-169//gi|78338319|gb|DT558593.1|DT558593</td><td></td><td></td><td>GO:0003676</td><td>nr +</td></tr>
<tr><td>Unigene113578_All</td><td>236</td><td>0.00</td><td>0.00</td><td>0.00</td><td>0.00</td><td>0.00</td><td>0.00</td><td>0.00</td><td>2.83</td><td>0.00</td><td>null</td><td>null</td><td></td><td>null</td><td>null</td><td></td><td>null</td><td>null</td><td></td><td>null</td><td>null</td><td></td><td>1.15E+01</td><td>7.64E-05</td><td>Up</td><td>null</td><td>null</td><td></td><td></td><td></td><td></td><td></td><td></td><td></td><td></td></tr>
<tr><td>Unigene113579_All</td><td>667</td><td>0.00</td><td>0.00</td><td>0.00</td><td>0.00</td><td>0.00</td><td>0.00</td><td>0.89</td><td>2.13</td><td>0.00</td><td>null</td><td>null</td><td></td><td>null</td><td>null</td><td></td><td>null</td><td>null</td><td></td><td>null</td><td>null</td><td></td><td>1.26E+00</td><td>1.33E-02</td><td></td><td>-9.79E+00</td><td>1.41E-04</td><td>Down</td><td>Unigene113579_All//gi|115464309|ref|NP_001055754.1|//2.00E-84//Os05g0460000 [Oryza sativa Japonica Group] >gi|113579305|dbj|BAF17668.1| Os05g0460000 [Oryza sativa Japonica Group]</td><td>Unigene113579_All//8.00E-83//AT1G56410| ERD2 (EARLY-RESPONSIVE TO DEHYDRATION 2); ATP binding</td><td>Unigene113579_All//8.00E-07//TC142196</td><td></td><td>ko03040|Spliceosome//ko04141|Protein processing in endoplasmic reticulum//ko04144|Endocytosis</td><td>GO:0008152//GO:0016628//GO:0032559//GO:0050896</td><td>nr +</td></tr>
<tr><td>Unigene113594_All</td><td>489</td><td>0.54</td><td>0.93</td><td>0.22</td><td>1.43</td><td>0.77</td><td>0.09</td><td>0.43</td><td>2.31</td><td>1.47</td><td>8.02E-01</td><td>4.60E-01</td><td></td><td>-1.30E+00</td><td>4.37E-01</td><td></td><td>-8.81E-01</td><td>3.00E-01</td><td></td><td>-3.95E+00</td><td>1.46E-03</td><td></td><td>2.42E+00</td><td>3.17E-04</td><td>Up</td><td>1.76E+00</td><td>2.48E-02</td><td></td><td></td><td></td><td>Unigene113594_All//6.00E-07//AI729024</td><td></td><td></td><td></td><td></td></tr>
<tr><td>Unigene113608_All</td><td>310</td><td>0.00</td><td>0.00</td><td>0.00</td><td>0.00</td><td>0.00</td><td>0.00</td><td>0.00</td><td>2.83</td><td>0.00</td><td>null</td><td>null</td><td></td><td>null</td><td>null</td><td></td><td>null</td><td>null</td><td></td><td>null</td><td>null</td><td></td><td>1.15E+01</td><td>2.86E-06</td><td>Up</td><td>null</td><td>null</td><td></td><td></td><td></td><td></td><td></td><td></td><td></td><td>ESTscan -</td></tr>
<tr><td>Unigene113622_All</td><td>259</td><td>67.88</td><td>59.67</td><td>41.12</td><td>61.16</td><td>58.23</td><td>61.55</td><td>15.51</td><td>7.26</td><td>12.61</td><td>-1.86E-01</td><td>1.95E-01</td><td></td><td>-7.23E-01</td><td>1.27E-07</td><td></td><td>-7.08E-02</td><td>6.80E-01</td><td></td><td>9.20E-03</td><td>9.50E-01</td><td></td><td>-1.09E+00</td><td>7.04E-05</td><td>Down</td><td>-2.98E-01</td><td>2.83E-01</td><td></td><td></td><td></td><td>Unigene113622_All//5.00E-92//gi|21093919|gb|BQ406232.1|BQ406232</td><td></td><td></td><td></td><td></td></tr>
<tr><td>Unigene11363_All</td><td>2366</td><td>3.38</td><td>2.48</td><td>1.60</td><td>7.54</td><td>8.43</td><td>6.28</td><td>8.20</td><td>5.79</td><td>8.18</td><td>-4.46E-01</td><td>3.15E-02</td><td></td><td>-1.08E+00</td><td>6.51E-07</td><td>Down</td><td>1.61E-01</td><td>2.48E-01</td><td></td><td>-2.63E-01</td><td>5.64E-02</td><td></td><td>-5.01E-01</td><td>6.69E-06</td><td></td><td>-3.30E-03</td><td>9.73E-01</td><td></td><td>Unigene11363_All//gi|115466596|ref|NP_001056897.1|//0.00E+00//Os06g0163500 [Oryza sativa Japonica Group] >gi|113594937|dbj|BAF18811.1| Os06g0163500 [Oryza sativa Japonica Group]</td><td>Unigene11363_All//0.00E+00//AT5G19660| S1P (SITE-1 PROTEASE); endopeptidase/ serine-type endopeptidase</td><td>Unigene11363_All//0.00E+00//gi|164284100|gb|ES816654.1|ES816654</td><td></td><td>ko04141|Protein processing in endoplasmic reticulum</td><td>GO:0004175//GO:0006972//GO:0019538//GO:0043231</td><td>nr +</td></tr>
<tr><td>Unigene113631_All</td><td>243</td><td>0.00</td><td>0.00</td><td>0.00</td><td>0.00</td><td>0.00</td><td>0.00</td><td>2.26</td><td>1.20</td><td>0.00</td><td>null</td><td>null</td><td></td><td>null</td><td>null</td><td></td><td>null</td><td>null</td><td></td><td>null</td><td>null</td><td></td><td>-9.10E-01</td><td>3.19E-01</td><td></td><td>-1.11E+01</td><td>2.81E-04</td><td>Down</td><td></td><td></td><td></td><td></td><td></td><td></td><td>ESTscan +</td></tr>
<tr><td>Unigene113645_All</td><td>277</td><td>0.00</td><td>0.00</td><td>0.00</td><td>0.00</td><td>0.00</td><td>0.00</td><td>3.36</td><td>11.92</td><td>4.89</td><td>null</td><td>null</td><td></td><td>null</td><td>null</td><td></td><td>null</td><td>null</td><td></td><td>null</td><td>null</td><td></td><td>1.83E+00</td><td>5.46E-08</td><td>Up</td><td>5.42E-01</td><td>2.85E-01</td><td></td><td></td><td></td><td>Unigene113645_All//5.00E-49//TC142576</td><td></td><td></td><td></td><td></td></tr>
<tr><td>Unigene11365_All</td><td>1046</td><td>11.00</td><td>8.43</td><td>9.93</td><td>41.72</td><td>32.69</td><td>31.69</td><td>18.67</td><td>8.83</td><td>6.89</td><td>-3.85E-01</td><td>2.35E-02</td><td></td><td>-1.49E-01</td><td>4.30E-01</td><td></td><td>-3.52E-01</td><td>2.19E-06</td><td></td><td>-3.97E-01</td><td>3.66E-07</td><td></td><td>-1.08E+00</td><td>3.26E-20</td><td>Down</td><td>-1.44E+00</td><td>4.07E-32</td><td>Down</td><td>Unigene11365_All//gi|17978924|gb|AAL47429.1|//1.00E-85//AT5g45290/K9E15_7 [Arabidopsis thaliana]</td><td>Unigene11365_All//3.00E-84//AT5G45290| zinc finger (C3HC4-type RING finger) family protein</td><td>Unigene11365_All//0.00E+00//TC133491</td><td></td><td></td><td>GO:0009536//GO:0046914</td><td>nr -</td></tr>
<tr><td>Unigene113660_All</td><td>1389</td><td>0.00</td><td>0.00</td><td>0.00</td><td>0.00</td><td>0.00</td><td>0.00</td><td>0.03</td><td>5.99</td><td>0.00</td><td>null</td><td>null</td><td></td><td>null</td><td>null</td><td></td><td>null</td><td>null</td><td></td><td>null</td><td>null</td><td></td><td>7.62E+00</td><td>9.90E-57</td><td>Up</td><td>-4.93E+00</td><td>6.16E-01</td><td></td><td>Unigene113660_All//gi|253759874|ref|XP_002488951.1|//2.00E-14//hypothetical protein SORBIDRAFT_1292s002010 [Sorghum bicolor] >gi|241947058|gb|EES20203.1| hypothetical protein SORBIDRAFT_1292s002010 [Sorghum bicolor]</td><td></td><td>Unigene113660_All//4.00E-41//gi|78352089|gb|DT572363.1|DT572363</td><td></td><td></td><td></td><td>nr +</td></tr>
<tr><td>Unigene113664_All</td><td>223</td><td>0.00</td><td>0.00</td><td>0.00</td><td>0.00</td><td>0.00</td><td>0.00</td><td>9.86</td><td>1.12</td><td>3.93</td><td>null</td><td>null</td><td></td><td>null</td><td>null</td><td></td><td>null</td><td>null</td><td></td><td>null</td><td>null</td><td></td><td>-3.13E+00</td><td>9.10E-10</td><td>Down</td><td>-1.33E+00</td><td>5.61E-04</td><td>Down</td><td>Unigene113664_All//gi|15232607|ref|NP_187533.1|//3.00E-09//LAC7 (laccase 7); laccase [Arabidopsis thaliana] >gi|75337548|sp|Q9SR40.1|LAC7_ARATH RecName: Full=Laccase-7; AltName: Full=Benzenediol:oxygen oxidoreductase 7; AltName: Full=Urishiol oxidase 7; AltName: Full=Diphenol oxidase 7; Flags: Precursor</td><td>Unigene113664_All//3.00E-09//AT3G09220| LAC7 (laccase 7); laccase</td><td></td><td></td><td></td><td>GO:0005576//GO:0009808//GO:0016682//GO:0031410//GO:0046914</td><td>nr -</td></tr>
<tr><td>Unigene113668_All</td><td>374</td><td>0.14</td><td>0.81</td><td>0.00</td><td>0.00</td><td>0.28</td><td>0.60</td><td>0.57</td><td>2.79</td><td>0.96</td><td>2.54E+00</td><td>1.51E-01</td><td></td><td>-7.13E+00</td><td>6.30E-01</td><td></td><td>8.11E+00</td><td>4.58E-01</td><td></td><td>9.24E+00</td><td>1.19E-01</td><td></td><td>2.31E+00</td><td>8.56E-04</td><td>Up</td><td>7.62E-01</td><td>5.00E-01</td><td></td><td>Unigene113668_All//gi|20245|emb|CAA38315.1|//5.00E-21//Glycine-rich protein [Oryza sativa (indica cultivar-group)] >gi|1167557|gb|AAA85863.1| glycine-rich cell wall protein [Oryza sativa]</td><td></td><td></td><td></td><td>ko03040|Spliceosome</td><td>GO:0031410</td><td>nr +</td></tr>
<tr><td>Unigene113669_All</td><td>534</td><td>0.20</td><td>1.33</td><td>0.80</td><td>0.47</td><td>0.19</td><td>0.34</td><td>1.35</td><td>5.40</td><td>0.97</td><td>2.76E+00</td><td>9.15E-03</td><td></td><td>2.03E+00</td><td>1.40E-01</td><td></td><td>-1.27E+00</td><td>4.24E-01</td><td></td><td>-4.63E-01</td><td>7.45E-01</td><td></td><td>2.00E+00</td><td>6.86E-08</td><td>Up</td><td>-4.73E-01</td><td>5.20E-01</td><td></td><td>Unigene113669_All//gi|18650616|gb|AAL75907.1|//7.00E-35//AT4g03420/F9H3_4 [Arabidopsis thaliana] >gi|20453247|gb|AAM19862.1| AT4g03420/F9H3_4 [Arabidopsis thaliana]</td><td>Unigene113669_All//3.00E-29//AT4G03420| unknown protein</td><td>Unigene113669_All//1.00E-69//TC143805</td><td></td><td></td><td></td><td>nr +</td></tr>
<tr><td>Unigene113673_All</td><td>448</td><td>0.00</td><td>0.00</td><td>0.00</td><td>0.00</td><td>0.00</td><td>0.00</td><td>0.00</td><td>1.31</td><td>0.00</td><td>null</td><td>null</td><td></td><td>null</td><td>null</td><td></td><td>null</td><td>null</td><td></td><td>null</td><td>null</td><td></td><td>1.04E+01</td><td>2.81E-04</td><td>Up</td><td>null</td><td>null</td><td></td><td>Unigene113673_All//gi|219119111|ref|XP_002180322.1|//2.00E-41//glutathione reductase [Phaeodactylum tricornutum CCAP 1055/1] >gi|217408579|gb|EEC48513.1| glutathione reductase [Phaeodactylum tricornutum CCAP 1055/1]</td><td>Unigene113673_All//2.00E-23//AT3G54660| GR (GLUTATHIONE REDUCTASE); ATP binding / glutathione-disulfide reductase</td><td></td><td></td><td>ko00240|Pyrimidine metabolism//ko00480|Glutathione metabolism</td><td>GO:0006790//GO:0015038//GO:0016651//GO:0019725//GO:0030554//GO:0044424</td><td>nr +</td></tr>
<tr><td>Unigene113677_All</td><td>209</td><td>0.00</td><td>0.00</td><td>0.00</td><td>4.77</td><td>0.82</td><td>0.00</td><td>0.81</td><td>1.80</td><td>0.19</td><td>null</td><td>null</td><td></td><td>null</td><td>null</td><td></td><td>-2.53E+00</td><td>6.75E-04</td><td>Down</td><td>-1.22E+01</td><td>2.97E-06</td><td>Down</td><td>1.15E+00</td><td>3.28E-01</td><td></td><td>-2.09E+00</td><td>3.14E-01</td><td></td><td></td><td></td><td>Unigene113677_All//2.00E-23//DW517725</td><td></td><td></td><td></td><td></td></tr>
<tr><td>Unigene113681_All</td><td>372</td><td>0.00</td><td>0.00</td><td>0.00</td><td>0.00</td><td>0.00</td><td>0.00</td><td>0.00</td><td>2.47</td><td>0.00</td><td>null</td><td>null</td><td></td><td>null</td><td>null</td><td></td><td>null</td><td>null</td><td></td><td>null</td><td>null</td><td></td><td>1.13E+01</td><td>1.48E-06</td><td>Up</td><td>null</td><td>null</td><td></td><td></td><td></td><td></td><td></td><td></td><td></td><td></td></tr>
<tr><td>Unigene113685_All</td><td>244</td><td>3.86</td><td>0.21</td><td>0.87</td><td>1.02</td><td>0.56</td><td>0.93</td><td>0.52</td><td>0.34</td><td>0.16</td><td>-4.22E+00</td><td>1.36E-04</td><td>Down</td><td>-2.14E+00</td><td>1.16E-02</td><td></td><td>-8.55E-01</td><td>5.74E-01</td><td></td><td>-1.41E-01</td><td>9.29E-01</td><td></td><td>-6.01E-01</td><td>7.67E-01</td><td></td><td>-1.67E+00</td><td>4.78E-01</td><td></td><td>Unigene113685_All//gi|222424875|dbj|BAH20389.1|//2.00E-16//AT3G53950 [Arabidopsis thaliana]</td><td>Unigene113685_All//6.00E-29//AT3G53950| glyoxal oxidase-related</td><td>Unigene113685_All//3.00E-50//ES799287</td><td></td><td></td><td>GO:0044464</td><td>nr +</td></tr>
<tr><td>Unigene113689_All</td><td>230</td><td>26.16</td><td>6.39</td><td>10.42</td><td>38.55</td><td>34.88</td><td>57.93</td><td>82.72</td><td>28.35</td><td>46.42</td><td>-2.03E+00</td><td>3.69E-13</td><td>Down</td><td>-1.33E+00</td><td>3.82E-07</td><td>Down</td><td>-1.44E-01</td><td>5.36E-01</td><td></td><td>5.87E-01</td><td>1.02E-04</td><td></td><td>-1.55E+00</td><td>1.04E-33</td><td>Down</td><td>-8.34E-01</td><td>1.74E-13</td><td></td><td>Unigene113689_All//gi|38327504|gb|AAR17783.1|//2.00E-27//ribosomal protein L3 [Solanum lycopersicum]</td><td>Unigene113689_All//3.00E-28//AT1G61580| RPL3B (R-PROTEIN L3 B); structural constituent of ribosome</td><td>Unigene113689_All//3.00E-127//gi|13352685|gb|BG443033.1|BG443033</td><td></td><td>ko03010|Ribosome</td><td>GO:0005198//GO:0010467//GO:0015934</td><td>nr +</td></tr>
<tr><td>Unigene113692_All</td><td>228</td><td>27.31</td><td>19.56</td><td>19.62</td><td>16.39</td><td>21.14</td><td>38.82</td><td>20.21</td><td>23.10</td><td>28.66</td><td>-4.82E-01</td><td>4.37E-02</td><td></td><td>-4.77E-01</td><td>5.69E-02</td><td></td><td>3.68E-01</td><td>2.07E-01</td><td></td><td>1.24E+00</td><td>3.35E-10</td><td>Up</td><td>1.93E-01</td><td>4.62E-01</td><td></td><td>5.04E-01</td><td>1.20E-02</td><td></td><td>Unigene113692_All//gi|149390510|gb|ABR25316.1|//7.00E-09//splicing factor [Oryza sativa Indica Group]</td><td>Unigene113692_All//7.00E-10//AT3G53500| zinc knuckle (CCHC-type) family protein</td><td>Unigene113692_All//8.00E-100//gi|78328880|gb|DT549154.1|DT549154</td><td></td><td>ko03040|Spliceosome</td><td>GO:0000398//GO:0016604//GO:0046914</td><td>nr -</td></tr>
<tr><td>Unigene1137_All</td><td>4133</td><td>4.05</td><td>9.95</td><td>6.56</td><td>3.63</td><td>5.26</td><td>5.18</td><td>9.05</td><td>17.74</td><td>28.87</td><td>1.30E+00</td><td>3.41E-45</td><td>Up</td><td>6.95E-01</td><td>1.11E-10</td><td></td><td>5.35E-01</td><td>1.05E-06</td><td></td><td>5.14E-01</td><td>9.02E-06</td><td></td><td>9.70E-01</td><td>9.60E-62</td><td></td><td>1.67E+00</td><td>4.90E-237</td><td>Up</td><td>Unigene1137_All//gi|3201682|gb|AAC19353.1|//0.00E+00//extra-large G-protein [Arabidopsis thaliana]</td><td>Unigene1137_All//0.00E+00//AT2G23460| XLG1 (EXTRA-LARGE G-PROTEIN 1); guanyl nucleotide binding / signal transducer</td><td>Unigene1137_All//0.00E+00//TC178714</td><td></td><td>ko00945|Stilbenoid, diarylheptanoid and gingerol biosynthesis//ko00903|Limonene and pinene degradation//ko00941|Flavonoid biosynthesis//ko00944|Flavone and flavonol biosynthesis</td><td>GO:0009725//GO:0009746//GO:0019001//GO:0023052//GO:0034285//GO:0043231</td><td>nr -</td></tr>
<tr><td>Unigene113707_All</td><td>223</td><td>0.00</td><td>0.00</td><td>0.00</td><td>0.00</td><td>0.00</td><td>0.41</td><td>0.00</td><td>2.62</td><td>1.25</td><td>null</td><td>null</td><td></td><td>null</td><td>null</td><td></td><td>null</td><td>null</td><td></td><td>8.66E+00</td><td>4.80E-01</td><td></td><td>1.14E+01</td><td>2.81E-04</td><td>Up</td><td>1.03E+01</td><td>2.44E-02</td><td></td><td></td><td></td><td></td><td></td><td></td><td></td><td></td></tr>
<tr><td>Unigene113728_All</td><td>366</td><td>0.29</td><td>0.00</td><td>0.00</td><td>2.45</td><td>1.32</td><td>1.48</td><td>0.35</td><td>3.20</td><td>0.54</td><td>-8.16E+00</td><td>3.75E-01</td><td></td><td>-8.16E+00</td><td>3.97E-01</td><td></td><td>-8.95E-01</td><td>2.14E-01</td><td></td><td>-7.26E-01</td><td>3.45E-01</td><td></td><td>3.21E+00</td><td>1.47E-05</td><td>Up</td><td>6.51E-01</td><td>6.66E-01</td><td></td><td>Unigene113728_All//gi|209970618|gb|ACJ03070.1|//1.00E-21//M18-S3Bp [Malus floribunda]</td><td>Unigene113728_All//2.00E-11//AT5G23400| disease resistance family protein / LRR family protein</td><td>Unigene113728_All//9.00E-107//ES844034</td><td></td><td></td><td>GO:0003824</td><td>nr -</td></tr>
<tr><td>Unigene113731_All</td><td>460</td><td>0.00</td><td>0.00</td><td>0.00</td><td>0.22</td><td>0.15</td><td>0.00</td><td>0.64</td><td>5.27</td><td>0.95</td><td>null</td><td>null</td><td></td><td>null</td><td>null</td><td></td><td>-5.33E-01</td><td>8.18E-01</td><td></td><td>-7.76E+00</td><td>4.03E-01</td><td></td><td>3.03E+00</td><td>2.27E-10</td><td>Up</td><td>5.66E-01</td><td>5.78E-01</td><td></td><td></td><td></td><td>Unigene113731_All//3.00E-08//DT468975</td><td></td><td></td><td></td><td></td></tr>
<tr><td>Unigene113732_All</td><td>208</td><td>0.00</td><td>0.00</td><td>0.00</td><td>0.00</td><td>0.00</td><td>0.00</td><td>0.41</td><td>4.22</td><td>0.00</td><td>null</td><td>null</td><td></td><td>null</td><td>null</td><td></td><td>null</td><td>null</td><td></td><td>null</td><td>null</td><td></td><td>3.38E+00</td><td>1.75E-04</td><td>Up</td><td>-8.67E+00</td><td>3.60E-01</td><td></td><td></td><td></td><td></td><td></td><td></td><td></td><td></td></tr>
<tr><td>Unigene113754_All</td><td>236</td><td>0.00</td><td>0.00</td><td>0.00</td><td>0.00</td><td>0.00</td><td>0.00</td><td>9.85</td><td>1.06</td><td>9.79</td><td>null</td><td>null</td><td></td><td>null</td><td>null</td><td></td><td>null</td><td>null</td><td></td><td>null</td><td>null</td><td></td><td>-3.21E+00</td><td>1.59E-10</td><td>Down</td><td>-9.20E-03</td><td>9.73E-01</td><td></td><td></td><td></td><td></td><td></td><td></td><td></td><td></td></tr>
<tr><td>Unigene113755_All</td><td>381</td><td>0.00</td><td>0.00</td><td>0.00</td><td>0.00</td><td>0.00</td><td>0.00</td><td>0.00</td><td>2.85</td><td>0.00</td><td>null</td><td>null</td><td></td><td>null</td><td>null</td><td></td><td>null</td><td>null</td><td></td><td>null</td><td>null</td><td></td><td>1.15E+01</td><td>1.04E-07</td><td>Up</td><td>null</td><td>null</td><td></td><td>Unigene113755_All//gi|301120398|ref|XP_002907926.1|//4.00E-16//aldehyde dehydrogenase, mitochondrial precursor [Phytophthora infestans T30-4] >gi|262102957|gb|EEY61009.1| aldehyde dehydrogenase, mitochondrial precursor [Phytophthora infestans T30-4]</td><td>Unigene113755_All//2.00E-11//AT3G24503| ALDH2C4; 3-chloroallyl aldehyde dehydrogenase/ aldehyde dehydrogenase (NAD)/ coniferyl-aldehyde dehydrogenase</td><td></td><td></td><td>ko00940|Phenylpropanoid biosynthesis//ko00010|Glycolysis / Gluconeogenesis//ko00903|Limonene and pinene degradation//ko00620|Pyruvate metabolism//ko00380|Tryptophan metabolism//ko00071|Fatty acid metabolism//ko00053|Ascorbate and aldarate metabolism//ko00330|Arginine and proline metabolism//ko00280|Valine, leucine and isoleucine degradation//ko00640|Propanoate metabolism//ko00561|Glycerolipid metabolism//ko00310|Lysine degradation//ko00410|beta-Alanine metabolism//ko00340|Histidine metabolism</td><td>GO:0009698//GO:0016620</td><td>nr -</td></tr>
<tr><td>Unigene113756_All</td><td>224</td><td>0.00</td><td>0.00</td><td>0.00</td><td>0.00</td><td>0.00</td><td>0.00</td><td>3.02</td><td>10.82</td><td>0.00</td><td>null</td><td>null</td><td></td><td>null</td><td>null</td><td></td><td>null</td><td>null</td><td></td><td>null</td><td>null</td><td></td><td>1.84E+00</td><td>4.09E-06</td><td>Up</td><td>-1.16E+01</td><td>3.56E-05</td><td>Down</td><td>Unigene113756_All//gi|49532956|dbj|BAD26579.1|//1.00E-10//cytochrome P450 like_TBP [Citrullus lanatus]</td><td></td><td>Unigene113756_All//1.00E-36//gi|5050563|gb|AI731711.1|AI731711</td><td></td><td></td><td></td><td>nr +</td></tr>
<tr><td>Unigene113765_All</td><td>217</td><td>0.72</td><td>0.47</td><td>0.49</td><td>1.15</td><td>0.16</td><td>0.00</td><td>4.29</td><td>7.32</td><td>10.46</td><td>-6.31E-01</td><td>7.28E-01</td><td></td><td>-5.60E-01</td><td>7.76E-01</td><td></td><td>-2.85E+00</td><td>1.37E-01</td><td></td><td>-1.02E+01</td><td>7.35E-02</td><td></td><td>7.72E-01</td><td>1.02E-01</td><td></td><td>1.29E+00</td><td>6.77E-04</td><td>Up</td><td>Unigene113765_All//gi|37906506|gb|AAP49697.1|//9.00E-07//cytochrome P-450-like protein [Vitis vinifera]</td><td>Unigene113765_All//4.00E-07//AT2G25160| CYP82F1; electron carrier/ heme binding / iron ion binding / monooxygenase/ oxygen binding</td><td>Unigene113765_All//4.00E-37//ES834213</td><td></td><td></td><td>GO:0005506//GO:0016709</td><td>nr -</td></tr>
<tr><td>Unigene113792_All</td><td>301</td><td>0.00</td><td>0.00</td><td>0.00</td><td>0.00</td><td>0.00</td><td>0.15</td><td>6.18</td><td>0.00</td><td>0.93</td><td>null</td><td>null</td><td></td><td>null</td><td>null</td><td></td><td>null</td><td>null</td><td></td><td>7.23E+00</td><td>7.01E-01</td><td></td><td>-1.26E+01</td><td>3.59E-13</td><td>Down</td><td>-2.74E+00</td><td>1.06E-07</td><td>Down</td><td>Unigene113792_All//gi|15232058|ref|NP_186768.1|//1.00E-27//peroxidase 27 (PER27) (P27) (PRXR7) [Arabidopsis thaliana] >gi|25453197|sp|Q43735.1|PER27_ARATH RecName: Full=Peroxidase 27; Short=Atperox P27; AltName: Full=PRXR7; AltName: Full=ATP12a; Flags: Precursor >gi|1402916|emb|CAA66963.1| peroxidase [Arabidopsis thaliana] >gi|1429217|emb|CAA67311.1| peroxidase ATP12a [Arabidopsis thaliana]</td><td>Unigene113792_All//8.00E-24//AT1G05250| peroxidase, putative</td><td></td><td></td><td>ko00940|Phenylpropanoid biosynthesis//ko00360|Phenylalanine metabolism</td><td>GO:0003824//GO:0005506//GO:0006950//GO:0008152//GO:0016209//GO:0044464</td><td>nr +</td></tr>
<tr><td>Unigene113795_All</td><td>339</td><td>0.00</td><td>0.00</td><td>0.00</td><td>0.00</td><td>0.00</td><td>0.00</td><td>0.12</td><td>2.34</td><td>0.00</td><td>null</td><td>null</td><td></td><td>null</td><td>null</td><td></td><td>null</td><td>null</td><td></td><td>null</td><td>null</td><td></td><td>4.23E+00</td><td>1.05E-04</td><td>Up</td><td>-6.96E+00</td><td>6.16E-01</td><td></td><td>Unigene113795_All//gi|299473653|emb|CBN78047.1|//1.00E-19//flagellar associated protein, quinone reductase-like protein [Ectocarpus siliculosus]</td><td>Unigene113795_All//2.00E-17//AT5G54500| FQR1 (FLAVODOXIN-LIKE QUINONE REDUCTASE 1); FMN binding / oxidoreductase, acting on NADH or NADPH, quinone or similar compound as acceptor</td><td></td><td></td><td></td><td>GO:0016491</td><td>nr +</td></tr>
<tr><td>Unigene1138_All</td><td>375</td><td>1.40</td><td>12.03</td><td>3.41</td><td>2.92</td><td>4.87</td><td>8.19</td><td>1.35</td><td>1.11</td><td>2.12</td><td>3.11E+00</td><td>1.19E-15</td><td>Up</td><td>1.29E+00</td><td>4.48E-02</td><td></td><td>7.36E-01</td><td>1.37E-01</td><td></td><td>1.49E+00</td><td>5.72E-05</td><td>Up</td><td>-2.80E-01</td><td>7.49E-01</td><td></td><td>6.51E-01</td><td>3.53E-01</td><td></td><td>Unigene1138_All//gi|18409320|ref|NP_566948.1|//2.00E-07//aspartyl protease family protein [Arabidopsis thaliana]</td><td>Unigene1138_All//1.00E-07//AT3G51330| aspartyl protease family protein</td><td>Unigene1138_All//8.00E-89//TC146833</td><td></td><td></td><td></td><td>nr -</td></tr>
<tr><td>Unigene113800_All</td><td>640</td><td>1.14</td><td>0.87</td><td>1.41</td><td>2.96</td><td>1.61</td><td>2.05</td><td>3.50</td><td>0.85</td><td>1.37</td><td>-3.94E-01</td><td>6.11E-01</td><td></td><td>3.06E-01</td><td>6.57E-01</td><td></td><td>-8.74E-01</td><td>5.25E-02</td><td></td><td>-5.31E-01</td><td>2.87E-01</td><td></td><td>-2.04E+00</td><td>1.89E-06</td><td>Down</td><td>-1.35E+00</td><td>3.87E-04</td><td>Down</td><td></td><td></td><td>Unigene113800_All//0.00E+00//TC142481</td><td></td><td></td><td></td><td></td></tr>
</table>

<table>
<tr><td>Unigene113802_All</td><td>782</td><td>0.00</td><td>0.00</td><td>0.00</td><td>0.00</td><td>0.00</td><td>0.00</td><td>1.51</td><td>2.51</td><td>0.10</td><td>null</td><td>null</td><td></td><td>null</td><td>null</td><td></td><td>null</td><td>null</td><td></td><td>null</td><td>null</td><td></td><td>7.31E-01</td><td>7.85E-02</td><td></td><td>-3.89E+00</td><td>9.21E-07</td><td>Down</td><td>Unigene113802_All//gi|301112246|ref|XP_002905202.1|//7.00E-64//60S ribosomal protein L7 [Phytophthora infestans T30-4] >gi|262095532|gb|EEY53584.1| 60S ribosomal protein L7 [Phytophthora infestans T30-4]</td><td>Unigene113802_All//1.00E-54//AT3G13580| 60S ribosomal protein L7 (RPL7D)</td><td></td><td></td><td>ko03010|Ribosome</td><td>GO:0005840</td><td>nr -</td></tr>
<tr><td>Unigene113806_All</td><td>426</td><td>0.00</td><td>0.00</td><td>0.00</td><td>0.00</td><td>0.00</td><td>0.00</td><td>0.00</td><td>3.14</td><td>0.00</td><td>null</td><td>null</td><td></td><td>null</td><td>null</td><td></td><td>null</td><td>null</td><td></td><td>null</td><td>null</td><td></td><td>1.16E+01</td><td>1.88E-09</td><td>Up</td><td>null</td><td>null</td><td></td><td></td><td></td><td></td><td></td><td></td><td></td><td>ESTscan -</td></tr>
<tr><td>Unigene113808_All</td><td>354</td><td>0.00</td><td>0.00</td><td>0.00</td><td>0.00</td><td>0.19</td><td>0.00</td><td>0.00</td><td>2.95</td><td>0.56</td><td>null</td><td>null</td><td></td><td>null</td><td>null</td><td></td><td>7.60E+00</td><td>6.14E-01</td><td></td><td>null</td><td>null</td><td></td><td>1.15E+01</td><td>2.02E-07</td><td>Up</td><td>9.14E+00</td><td>7.91E-02</td><td></td><td>Unigene113808_All//gi|297831428|ref|XP_002883596.1|//2.00E-39//oxidative signal-inducible1 [Arabidopsis lyrata subsp. lyrata] >gi|297329436|gb|EFH59855.1| oxidative signal-inducible1 [Arabidopsis lyrata subsp. lyrata]</td><td>Unigene113808_All//2.00E-40//AT4G13000| protein kinase family protein</td><td>Unigene113808_All//8.00E-169//TC142650</td><td></td><td></td><td>GO:0004674//GO:0006464//GO:0032559</td><td>nr -</td></tr>
<tr><td>Unigene11382_All</td><td>2567</td><td>2.91</td><td>2.94</td><td>3.84</td><td>7.08</td><td>6.33</td><td>5.44</td><td>4.73</td><td>2.46</td><td>2.10</td><td>1.31E-02</td><td>9.75E-01</td><td></td><td>3.97E-01</td><td>4.14E-02</td><td></td><td>-1.62E-01</td><td>2.68E-01</td><td></td><td>-3.82E-01</td><td>3.17E-03</td><td></td><td>-9.43E-01</td><td>1.90E-10</td><td></td><td>-1.17E+00</td><td>4.74E-15</td><td>Down</td><td>Unigene11382_All//gi|116008248|gb|ABJ51934.1|//0.00E+00//sucrose transporter 2A [Hevea brasiliensis]</td><td>Unigene11382_All//0.00E+00//AT2G02860| SUT2 (SUCROSE TRANSPORTER 2); carbohydrate transmembrane transporter/ sucrose transmembrane transporter/ sucrose:hydrogen symporter/ sugar:hydrogen symporter</td><td>Unigene11382_All//0.00E+00//gi|164252069|gb|ES790405.1|ES790405</td><td></td><td></td><td>GO:0005351//GO:0006950//GO:0015154//GO:0015766//GO:0016021</td><td>nr -</td></tr>
<tr><td>Unigene113824_All</td><td>580</td><td>0.00</td><td>0.00</td><td>0.00</td><td>0.09</td><td>0.30</td><td>0.00</td><td>0.51</td><td>2.74</td><td>3.30</td><td>null</td><td>null</td><td></td><td>null</td><td>null</td><td></td><td>1.79E+00</td><td>5.21E-01</td><td></td><td>-6.42E+00</td><td>6.42E-01</td><td></td><td>2.42E+00</td><td>1.10E-05</td><td>Up</td><td>2.69E+00</td><td>1.24E-07</td><td>Up</td><td></td><td></td><td></td><td></td><td></td><td></td><td></td></tr>
<tr><td>Unigene113826_All</td><td>356</td><td>6.91</td><td>1.00</td><td>2.69</td><td>20.15</td><td>23.12</td><td>9.90</td><td>9.03</td><td>3.99</td><td>1.68</td><td>-2.79E+00</td><td>4.57E-08</td><td>Down</td><td>-1.36E+00</td><td>1.89E-03</td><td></td><td>1.98E-01</td><td>3.93E-01</td><td></td><td>-1.03E+00</td><td>2.04E-06</td><td>Down</td><td>-1.18E+00</td><td>1.84E-04</td><td>Down</td><td>-2.43E+00</td><td>2.96E-11</td><td>Down</td><td></td><td></td><td>Unigene113826_All//2.00E-52//TC175539</td><td></td><td></td><td></td><td></td></tr>
<tr><td>Unigene11384_All</td><td>2951</td><td>7.64</td><td>9.58</td><td>7.98</td><td>11.33</td><td>12.63</td><td>25.63</td><td>12.09</td><td>15.48</td><td>19.99</td><td>3.26E-01</td><td>1.53E-03</td><td></td><td>6.18E-02</td><td>6.23E-01</td><td></td><td>1.57E-01</td><td>9.62E-02</td><td></td><td>1.18E+00</td><td>2.15E-76</td><td>Up</td><td>3.56E-01</td><td>3.60E-07</td><td></td><td>7.25E-01</td><td>2.05E-31</td><td></td><td>Unigene11384_All//gi|297851224|ref|XP_002893493.1|//0.00E+00//U-box domain-containing protein [Arabidopsis lyrata subsp. lyrata] >gi|297339335|gb|EFH69752.1| U-box domain-containing protein [Arabidopsis lyrata subsp. lyrata]</td><td>Unigene11384_All//0.00E+00//AT1G24330| armadillo/beta-catenin repeat family protein / U-box domain-containing family protein</td><td>Unigene11384_All//0.00E+00//DT460925</td><td></td><td></td><td>GO:0005488//GO:0019787//GO:0032446//GO:0043234</td><td>nr -</td></tr>
<tr><td>Unigene113845_All</td><td>830</td><td>0.00</td><td>0.00</td><td>0.00</td><td>0.00</td><td>0.00</td><td>0.00</td><td>1.27</td><td>3.12</td><td>0.00</td><td>null</td><td>null</td><td></td><td>null</td><td>null</td><td></td><td>null</td><td>null</td><td></td><td>null</td><td>null</td><td></td><td>1.29E+00</td><td>3.28E-04</td><td>Up</td><td>-1.03E+01</td><td>6.72E-08</td><td>Down</td><td>Unigene113845_All//gi|301121570|ref|XP_002908512.1|//3.00E-87//40S ribosomal protein SA [Phytophthora infestans T30-4] >gi|262103543|gb|EEY61595.1| 40S ribosomal protein SA [Phytophthora infestans T30-4]</td><td>Unigene113845_All//1.00E-70//AT3G04770| RPSAb (40S ribosomal protein SA B); structural constituent of ribosome</td><td></td><td></td><td>ko03010|Ribosome</td><td>GO:0005198//GO:0010467//GO:0033279</td><td>nr +</td></tr>
<tr><td>Unigene113859_All</td><td>298</td><td>0.00</td><td>0.00</td><td>0.00</td><td>0.00</td><td>0.00</td><td>0.00</td><td>1.99</td><td>3.23</td><td>0.00</td><td>null</td><td>null</td><td></td><td>null</td><td>null</td><td></td><td>null</td><td>null</td><td></td><td>null</td><td>null</td><td></td><td>7.00E-01</td><td>2.78E-01</td><td></td><td>-1.10E+01</td><td>1.41E-04</td><td>Down</td><td>Unigene113859_All//gi|145340549|ref|XP_001415385.1|//3.00E-29//Ribosomal protein L3, component of cytosolic 80S ribosome and 60S large subunit [Ostreococcus lucimarinus CCE9901] >gi|144575608|gb|ABO93677.1| Ribosomal protein L3, component of cytosolic 80S ribosome and 60S large subunit [Ostreococcus lucimarinus CCE9901]</td><td>Unigene113859_All//1.00E-27//AT1G61580| RPL3B (R-PROTEIN L3 B); structural constituent of ribosome</td><td></td><td></td><td>ko03010|Ribosome</td><td>GO:0005198//GO:0010467//GO:0015934</td><td>nr -</td></tr>
<tr><td>Unigene11386_All</td><td>1938</td><td>0.84</td><td>0.84</td><td>0.49</td><td>2.93</td><td>1.63</td><td>1.35</td><td>2.97</td><td>4.46</td><td>4.71</td><td>-5.00E-04</td><td>9.97E-01</td><td></td><td>-7.59E-01</td><td>1.59E-01</td><td></td><td>-8.42E-01</td><td>2.74E-04</td><td></td><td>-1.12E+00</td><td>6.85E-06</td><td>Down</td><td>5.89E-01</td><td>7.47E-04</td><td></td><td>6.66E-01</td><td>5.92E-05</td><td></td><td>Unigene11386_All//gi|297795477|ref|XP_002865623.1|//1.00E-109//zinc finger (B-box type) family protein [Arabidopsis lyrata subsp. lyrata] >gi|297311458|gb|EFH41882.1| zinc finger (B-box type) family protein [Arabidopsis lyrata subsp. lyrata]</td><td>Unigene11386_All//1.00E-48//AT5G48250| zinc finger (B-box type) family protein</td><td>Unigene11386_All//0.00E+00//gi|78341395|gb|DT561669.1|DT561669</td><td>C2C2-CO-like</td><td>ko04712|Circadian rhythm - plant</td><td>GO:0001071//GO:0010468//GO:0043231//GO:0048581</td><td>nr +</td></tr>
<tr><td>Unigene113862_All</td><td>449</td><td>0.00</td><td>0.00</td><td>0.00</td><td>0.00</td><td>0.00</td><td>0.00</td><td>1.51</td><td>1.77</td><td>0.00</td><td>null</td><td>null</td><td></td><td>null</td><td>null</td><td></td><td>null</td><td>null</td><td></td><td>null</td><td>null</td><td></td><td>2.31E-01</td><td>7.35E-01</td><td></td><td>-1.06E+01</td><td>3.56E-05</td><td>Down</td><td>Unigene113862_All//gi|299472847|emb|CBN80416.1|//5.00E-14//EsV-1-116 [Ectocarpus siliculosus]</td><td></td><td></td><td></td><td></td><td></td><td>nr +</td></tr>
<tr><td>Unigene113863_All</td><td>398</td><td>0.00</td><td>0.00</td><td>0.00</td><td>0.00</td><td>0.00</td><td>0.00</td><td>0.42</td><td>2.73</td><td>0.00</td><td>null</td><td>null</td><td></td><td>null</td><td>null</td><td></td><td>null</td><td>null</td><td></td><td>null</td><td>null</td><td></td><td>2.68E+00</td><td>1.69E-04</td><td>Up</td><td>-8.73E+00</td><td>1.09E-01</td><td></td><td>Unigene113863_All//gi|729618|sp|Q03682.1|BIP2_TOBAC//3.00E-26//RecName: Full=Luminal-binding protein 2; Short=BiP 2; AltName: Full=78 kDa glucose-regulated protein homolog 2; Short=GRP-78-2 >gi|19807|emb|CAA42661.1| luminal binding protein (BiP) [Nicotiana tabacum]</td><td>Unigene113863_All//2.00E-22//AT5G28540| BIP1; ATP binding</td><td>Unigene113863_All//1.00E-10//TC157469</td><td></td><td>ko04141|Protein processing in endoplasmic reticulum//ko03060|Protein export</td><td>GO:0000166//GO:0005783</td><td>nr -</td></tr>
<tr><td>Unigene113871_All</td><td>248</td><td>0.21</td><td>0.00</td><td>0.00</td><td>0.40</td><td>0.00</td><td>0.00</td><td>0.17</td><td>3.20</td><td>0.48</td><td>-7.72E+00</td><td>6.17E-01</td><td></td><td>-7.72E+00</td><td>6.30E-01</td><td></td><td>-8.65E+00</td><td>3.15E-01</td><td></td><td>-8.65E+00</td><td>4.03E-01</td><td></td><td>4.23E+00</td><td>1.05E-04</td><td>Up</td><td>1.50E+00</td><td>5.59E-01</td><td></td><td>Unigene113871_All//gi|224115116|ref|XP_002316944.1|//5.00E-09//predicted protein [Populus trichocarpa] >gi|222860009|gb|EEE97556.1| predicted protein [Populus trichocarpa]</td><td>Unigene113871_All//7.00E-05//AT4G21380| ARK3 (A. THALIANA RECEPTOR KINASE 3); kinase/ transmembrane receptor protein serine/threonine kinase</td><td></td><td></td><td></td><td>GO:0004713//GO:0006464//GO:0008037//GO:0030246//GO:0032559</td><td>nr -</td></tr>
<tr><td>Unigene1139_All</td><td>1880</td><td>33.20</td><td>114.19</td><td>35.32</td><td>38.53</td><td>44.95</td><td>68.39</td><td>13.74</td><td>13.87</td><td>15.55</td><td>1.78E+00</td><td>0.00E+00</td><td>Up</td><td>8.93E-02</td><td>2.42E-01</td><td></td><td>2.22E-01</td><td>3.30E-05</td><td></td><td>8.28E-01</td><td>1.52E-72</td><td></td><td>1.37E-02</td><td>9.20E-01</td><td></td><td>1.79E-01</td><td>5.31E-02</td><td></td><td>Unigene1139_All//gi|30680102|ref|NP_849967.1|//1.00E-176//aspartyl protease family protein [Arabidopsis thaliana] >gi|22655368|gb|AAM98276.1| At2g17760/At2g17760 [Arabidopsis thaliana]</td><td>Unigene1139_All//8.00E-169//AT2G17760| aspartyl protease family protein</td><td>Unigene1139_All//0.00E+00//TC149049</td><td></td><td></td><td>GO:0008233</td><td>nr +</td></tr>
<tr><td>Unigene113902_All</td><td>336</td><td>0.16</td><td>1.66</td><td>0.79</td><td>0.59</td><td>0.61</td><td>2.02</td><td>0.25</td><td>4.35</td><td>1.07</td><td>3.41E+00</td><td>1.24E-02</td><td></td><td>2.35E+00</td><td>2.40E-01</td><td></td><td>5.23E-02</td><td>9.89E-01</td><td></td><td>1.77E+00</td><td>6.95E-02</td><td></td><td>4.11E+00</td><td>4.02E-08</td><td>Up</td><td>2.08E+00</td><td>1.01E-01</td><td></td><td></td><td></td><td></td><td></td><td></td><td></td><td></td></tr>
<tr><td>Unigene113915_All</td><td>283</td><td>0.18</td><td>0.36</td><td>0.00</td><td>1.23</td><td>0.12</td><td>0.32</td><td>1.94</td><td>2.51</td><td>0.00</td><td>9.54E-01</td><td>7.27E-01</td><td></td><td>-7.53E+00</td><td>6.30E-01</td><td></td><td>-3.34E+00</td><td>4.07E-02</td><td></td><td>-1.95E+00</td><td>1.96E-01</td><td></td><td>3.70E-01</td><td>6.67E-01</td><td></td><td>-1.09E+01</td><td>2.81E-04</td><td>Down</td><td></td><td></td><td>Unigene113915_All//1.00E-111//gi|164273610|gb|ES842137.1|ES842137</td><td></td><td></td><td></td><td></td></tr>
<tr><td>Unigene11392_All</td><td>543</td><td>0.00</td><td>0.00</td><td>0.00</td><td>1.38</td><td>0.00</td><td>0.67</td><td>0.00</td><td>0.54</td><td>0.29</td><td>null</td><td>null</td><td></td><td>null</td><td>null</td><td></td><td>-1.04E+01</td><td>1.50E-05</td><td>Down</td><td>-1.05E+00</td><td>2.21E-01</td><td></td><td>9.07E+00</td><td>2.36E-02</td><td></td><td>8.20E+00</td><td>1.40E-01</td><td></td><td>Unigene11392_All//gi|224130846|ref|XP_002320939.1|//1.00E-46//multidrug/pheromone exporter, MDR family, ABC transporter family [Populus trichocarpa] >gi|222861712|gb|EEE99254.1| multidrug/pheromone exporter, MDR family, ABC transporter family [Populus trichocarpa]</td><td>Unigene11392_All//5.00E-34//AT1G02520| PGP11 (P-GLYCOPROTEIN 11); ATPase, coupled to transmembrane movement of substances</td><td>Unigene11392_All//5.00E-23//TC138934</td><td></td><td>ko02010|ABC transporters</td><td>GO:0006810//GO:0031224//GO:0032559//GO:0042626</td><td>nr +</td></tr>
<tr><td>Unigene113920_All</td><td>440</td><td>0.12</td><td>0.00</td><td>0.00</td><td>0.00</td><td>0.00</td><td>0.00</td><td>0.38</td><td>8.64</td><td>0.91</td><td>-6.89E+00</td><td>6.17E-01</td><td></td><td>-6.89E+00</td><td>6.30E-01</td><td></td><td>null</td><td>null</td><td></td><td>null</td><td>null</td><td></td><td>4.49E+00</td><td>1.65E-21</td><td>Up</td><td>1.24E+00</td><td>2.51E-01</td><td></td><td></td><td></td><td></td><td></td><td></td><td></td><td></td></tr>
<tr><td>Unigene113921_All</td><td>225</td><td>0.00</td><td>0.00</td><td>0.00</td><td>0.00</td><td>0.00</td><td>0.00</td><td>0.19</td><td>2.97</td><td>0.35</td><td>null</td><td>null</td><td></td><td>null</td><td>null</td><td></td><td>null</td><td>null</td><td></td><td>null</td><td>null</td><td></td><td>3.98E+00</td><td>6.38E-04</td><td>Up</td><td>9.14E-01</td><td>7.51E-01</td><td></td><td>Unigene113921_All//gi|29028782|gb|AAO64770.1|//9.00E-07//At5g02580 [Arabidopsis thaliana]</td><td>Unigene113921_All//2.00E-06//AT5G02580| unknown protein</td><td>Unigene113921_All//7.00E-14//TC157651</td><td></td><td></td><td></td><td>nr -</td></tr>
<tr><td>Unigene113922_All</td><td>476</td><td>0.22</td><td>0.53</td><td>1.12</td><td>0.00</td><td>0.94</td><td>0.38</td><td>0.36</td><td>4.13</td><td>0.25</td><td>1.28E+00</td><td>4.35E-01</td><td></td><td>2.35E+00</td><td>5.88E-02</td><td></td><td>9.88E+00</td><td>8.01E-03</td><td></td><td>8.57E+00</td><td>1.95E-01</td><td></td><td>3.54E+00</td><td>1.14E-09</td><td>Up</td><td>-5.01E-01</td><td>7.47E-01</td><td></td><td>Unigene113922_All//gi|159138935|gb|ABW89467.1|//2.00E-57//low molecular weight heat shock protein [Gossypium hirsutum]</td><td>Unigene113922_All//4.00E-40//AT2G29500| 17.6 kDa class I small heat shock protein (HSP17.6B-CI)</td><td>Unigene113922_All//2.00E-111//TC144617</td><td></td><td></td><td>GO:0044424//GO:0050896</td><td>nr -</td></tr>
<tr><td>Unigene113923_All</td><td>300</td><td>0.35</td><td>0.68</td><td>0.36</td><td>3.82</td><td>1.03</td><td>0.00</td><td>0.28</td><td>0.56</td><td>0.27</td><td>9.54E-01</td><td>6.22E-01</td><td></td><td>2.54E-02</td><td>1.00E+00</td><td></td><td>-1.89E+00</td><td>3.06E-03</td><td></td><td>-1.19E+01</td><td>3.58E-07</td><td>Down</td><td>9.83E-01</td><td>6.34E-01</td><td></td><td>-8.59E-02</td><td>9.73E-01</td><td></td><td></td><td></td><td>Unigene113923_All//6.00E-21//ES821095</td><td></td><td></td><td></td><td></td></tr>
<tr><td>Unigene11393_All</td><td>1169</td><td>0.49</td><td>0.35</td><td>1.09</td><td>3.79</td><td>1.38</td><td>2.28</td><td>54.29</td><td>88.38</td><td>201.54</td><td>-5.05E-01</td><td>6.00E-01</td><td></td><td>1.15E+00</td><td>6.95E-02</td><td></td><td>-1.45E+00</td><td>1.33E-07</td><td>Down</td><td>-7.35E-01</td><td>9.99E-03</td><td></td><td>7.03E-01</td><td>2.70E-50</td><td></td><td>1.89E+00</td><td>0.00E+00</td><td>Up</td><td>Unigene11393_All//gi|224130846|ref|XP_002320939.1|//1.00E-168//multidrug/pheromone exporter, MDR family, ABC transporter family [Populus trichocarpa] >gi|222861712|gb|EEE99254.1| multidrug/pheromone exporter, MDR family, ABC transporter family [Populus trichocarpa]</td><td>Unigene11393_All//3.00E-156//AT1G02520| PGP11 (P-GLYCOPROTEIN 11); ATPase, coupled to transmembrane movement of substances</td><td>Unigene11393_All//4.00E-87//gi|109856091|gb|DW485070.1|DW485070</td><td></td><td>ko02010|ABC transporters</td><td></td><td>nr +</td></tr>
<tr><td>Unigene113933_All</td><td>227</td><td>0.00</td><td>0.45</td><td>0.47</td><td>0.00</td><td>0.00</td><td>0.00</td><td>0.00</td><td>2.39</td><td>1.23</td><td>8.80E+00</td><td>3.89E-01</td><td></td><td>8.87E+00</td><td>4.01E-01</td><td></td><td>null</td><td>null</td><td></td><td>null</td><td>null</td><td></td><td>1.12E+01</td><td>5.37E-04</td><td>Up</td><td>1.03E+01</td><td>2.44E-02</td><td></td><td></td><td></td><td></td><td></td><td></td><td></td><td></td></tr>
<tr><td>Unigene113934_All</td><td>269</td><td>0.00</td><td>0.00</td><td>0.00</td><td>0.00</td><td>0.00</td><td>0.00</td><td>0.00</td><td>0.78</td><td>2.07</td><td>null</td><td>null</td><td></td><td>null</td><td>null</td><td></td><td>null</td><td>null</td><td></td><td>null</td><td>null</td><td></td><td>9.60E+00</td><td>7.87E-02</td><td></td><td>1.10E+01</td><td>3.28E-04</td><td>Up</td><td>Unigene113934_All//gi|224138866|ref|XP_002322921.1|//9.00E-10//aldose reductase with ABC domain [Populus trichocarpa] >gi|222867551|gb|EEF04682.1| aldose reductase with ABC domain [Populus trichocarpa]</td><td></td><td>Unigene113934_All//4.00E-34//DR460967</td><td></td><td></td><td></td><td>nr -</td></tr>
<tr><td>Unigene113935_All</td><td>239</td><td>0.00</td><td>0.00</td><td>0.89</td><td>0.42</td><td>0.00</td><td>0.00</td><td>0.88</td><td>3.32</td><td>16.67</td><td>null</td><td>null</td><td></td><td>9.80E+00</td><td>1.42E-01</td><td></td><td>-8.70E+00</td><td>3.15E-01</td><td></td><td>-8.70E+00</td><td>4.03E-01</td><td></td><td>1.91E+00</td><td>1.37E-02</td><td></td><td>4.24E+00</td><td>4.01E-22</td><td>Up</td><td></td><td></td><td></td><td></td><td></td><td></td><td></td></tr>
<tr><td>Unigene11394_All</td><td>1843</td><td>0.88</td><td>2.67</td><td>1.04</td><td>8.89</td><td>3.94</td><td>3.77</td><td>145.70</td><td>308.63</td><td>416.00</td><td>1.60E+00</td><td>5.40E-08</td><td>Up</td><td>2.41E-01</td><td>6.24E-01</td><td></td><td>-1.17E+00</td><td>2.83E-19</td><td>Down</td><td>-1.24E+00</td><td>1.71E-18</td><td>Down</td><td>1.08E+00</td><td>0.00E+00</td><td>Up</td><td>1.51E+00</td><td>0.00E+00</td><td>Up</td><td>Unigene11394_All//gi|224130858|ref|XP_002320942.1|//0.00E+00//multidrug/pheromone exporter, MDR family, ABC transporter family [Populus trichocarpa] >gi|222861715|gb|EEE99257.1| multidrug/pheromone exporter, MDR family, ABC transporter family [Populus trichocarpa]</td><td>Unigene11394_All//0.00E+00//AT3G62150| PGP21 (P-GLYCOPROTEIN 21); ATPase, coupled to transmembrane movement of substances</td><td>Unigene11394_All//0.00E+00//TC168072</td><td></td><td>ko02010|ABC transporters</td><td>GO:0000166//GO:0009606//GO:0009734//GO:0009926//GO:0016020//GO:0042626//GO:0048588</td><td>nr +</td></tr>
<tr><td>Unigene113947_All</td><td>536</td><td>1.17</td><td>0.57</td><td>1.09</td><td>1.58</td><td>2.38</td><td>5.06</td><td>0.79</td><td>0.94</td><td>0.15</td><td>-1.05E+00</td><td>2.50E-01</td><td></td><td>-1.00E-01</td><td>9.15E-01</td><td></td><td>5.89E-01</td><td>3.55E-01</td><td></td><td>1.68E+00</td><td>3.55E-05</td><td>Up</td><td>2.46E-01</td><td>7.84E-01</td><td></td><td>-2.41E+00</td><td>3.94E-02</td><td></td><td></td><td></td><td>Unigene113947_All//7.00E-90//DW510486</td><td></td><td></td><td></td><td></td></tr>
<tr><td>Unigene11395_All</td><td>3602</td><td>0.26</td><td>0.68</td><td>1.05</td><td>11.01</td><td>9.42</td><td>9.19</td><td>11.60</td><td>28.53</td><td>21.87</td><td>1.37E+00</td><td>1.23E-03</td><td></td><td>2.01E+00</td><td>5.43E-08</td><td>Up</td><td>-2.25E-01</td><td>6.87E-03</td><td></td><td>-2.60E-01</td><td>2.28E-03</td><td></td><td>1.30E+00</td><td>1.63E-137</td><td>Up</td><td>9.15E-01</td><td>1.58E-61</td><td></td><td>Unigene11395_All//gi|224064756|ref|XP_002301547.1|//0.00E+00//multidrug/pheromone exporter, MDR family, ABC transporter family [Populus trichocarpa] >gi|222843273|gb|EEE80820.1| multidrug/pheromone exporter, MDR family, ABC transporter family [Populus trichocarpa]</td><td>Unigene11395_All//0.00E+00//AT2G47000| ABCB4 (ATP BINDING CASSETTE SUBFAMILY B4); ATPase, coupled to transmembrane movement of substances / xenobiotic-transporting ATPase</td><td>Unigene11395_All//0.00E+00//TC168072</td><td></td><td>ko02010|ABC transporters</td><td>GO:0009606//GO:0009734//GO:0009926//GO:0015114//GO:0031224//GO:0032559//GO:0042626//GO:0048588</td><td>nr +</td></tr>
<tr><td>Unigene113951_All</td><td>694</td><td>0.00</td><td>0.00</td><td>0.00</td><td>0.00</td><td>0.00</td><td>0.00</td><td>1.40</td><td>4.58</td><td>0.11</td><td>null</td><td>null</td><td></td><td>null</td><td>null</td><td></td><td>null</td><td>null</td><td></td><td>null</td><td>null</td><td></td><td>1.71E+00</td><td>4.29E-07</td><td>Up</td><td>-3.61E+00</td><td>2.14E-05</td><td>Down</td><td></td><td></td><td>Unigene113951_All//9.00E-13//ES848646</td><td></td><td></td><td></td><td></td></tr>
<tr><td>Unigene113952_All</td><td>399</td><td>0.00</td><td>0.00</td><td>0.00</td><td>0.00</td><td>0.00</td><td>0.00</td><td>4.03</td><td>7.86</td><td>0.50</td><td>null</td><td>null</td><td></td><td>null</td><td>null</td><td></td><td>null</td><td>null</td><td></td><td>null</td><td>null</td><td></td><td>9.64E-01</td><td>2.19E-03</td><td></td><td>-3.01E+00</td><td>2.36E-07</td><td>Down</td><td></td><td></td><td>Unigene113952_All//3.00E-20//TC159777</td><td></td><td></td><td></td><td></td></tr>
<tr><td>Unigene113955_All</td><td>383</td><td>6.97</td><td>7.67</td><td>14.46</td><td>5.98</td><td>5.12</td><td>2.48</td><td>0.33</td><td>0.65</td><td>0.21</td><td>1.39E-01</td><td>6.97E-01</td><td></td><td>1.05E+00</td><td>7.79E-05</td><td>Up</td><td>-2.23E-01</td><td>6.06E-01</td><td></td><td>-1.27E+00</td><td>2.82E-03</td><td></td><td>9.83E-01</td><td>5.18E-01</td><td></td><td>-6.71E-01</td><td>7.20E-01</td><td></td><td></td><td></td><td>Unigene113955_All//5.00E-127//TC169561</td><td></td><td></td><td></td><td></td></tr>
<tr><td>Unigene113967_All</td><td>212</td><td>0.00</td><td>0.00</td><td>0.75</td><td>0.00</td><td>0.00</td><td>0.00</td><td>16.55</td><td>10.05</td><td>0.19</td><td>null</td><td>null</td><td></td><td>9.56E+00</td><td>2.39E-01</td><td></td><td>null</td><td>null</td><td></td><td>null</td><td>null</td><td></td><td>-7.19E-01</td><td>1.42E-02</td><td></td><td>-6.46E+00</td><td>3.54E-24</td><td>Down</td><td>Unigene113967_All//gi|116222089|gb|ABJ80915.1|//1.00E-34//actin [Goniomonas truncata]</td><td>Unigene113967_All//1.00E-35//AT5G59370| ACT4 (ACTIN 4); structural constituent of cytoskeleton</td><td>Unigene113967_All//2.00E-66//TC153744</td><td></td><td>ko04145|Phagosome</td><td>GO:0032559//GO:0043232</td><td>nr -</td></tr>
<tr><td>Unigene113969_All</td><td>1063</td><td>0.00</td><td>0.00</td><td>0.00</td><td>0.00</td><td>0.00</td><td>0.00</td><td>0.00</td><td>4.40</td><td>0.00</td><td>null</td><td>null</td><td></td><td>null</td><td>null</td><td></td><td>null</td><td>null</td><td></td><td>null</td><td>null</td><td></td><td>1.21E+01</td><td>5.77E-33</td><td>Up</td><td>null</td><td>null</td><td></td><td>Unigene113969_All//gi|298704815|emb|CBJ48963.1|//6.00E-61//Arf1, ARF family GTPase [Ectocarpus siliculosus]</td><td>Unigene113969_All//5.00E-61//AT3G62290| ATARFA1E (ADP-ribosylation factor A1E); GTP binding / phospholipase activator/ protein binding</td><td>Unigene113969_All//5.00E-12//TC157765</td><td></td><td></td><td>GO:0015031//GO:0032561//GO:0035556//GO:0043231</td><td>nr +</td></tr>
<tr><td>Unigene113973_All</td><td>206</td><td>0.00</td><td>0.00</td><td>0.00</td><td>0.97</td><td>0.67</td><td>1.97</td><td>0.00</td><td>4.06</td><td>0.19</td><td>null</td><td>null</td><td></td><td>null</td><td>null</td><td></td><td>-5.33E-01</td><td>7.49E-01</td><td></td><td>1.03E+00</td><td>4.29E-01</td><td></td><td>1.20E+01</td><td>5.54E-06</td><td>Up</td><td>7.60E+00</td><td>6.47E-01</td><td></td><td></td><td></td><td>Unigene113973_All//8.00E-75//TC157651</td><td></td><td></td><td></td><td></td></tr>
<tr><td>Unigene113980_All</td><td>235</td><td>0.22</td><td>0.00</td><td>0.00</td><td>0.00</td><td>0.00</td><td>0.38</td><td>114.79</td><td>66.34</td><td>231.74</td><td>-7.80E+00</td><td>6.17E-01</td><td></td><td>-7.80E+00</td><td>6.30E-01</td><td></td><td>null</td><td>null</td><td></td><td>8.59E+00</td><td>4.80E-01</td><td></td><td>-7.91E-01</td><td>1.07E-16</td><td></td><td>1.01E+00</td><td>1.33E-50</td><td>Up</td><td>Unigene113980_All//gi|5802576|gb|AAD51718.1|//1.00E-23//(+)-delta-cadinene sythase [Gossypium arboreum]</td><td>Unigene113980_All//2.00E-05//AT5G23960| TPS21 (TERPENE SYNTHASE 21); (-)-E-beta-caryophyllene synthase/ alpha-humulene synthase</td><td>Unigene113980_All//2.00E-72//ES834162</td><td></td><td></td><td>GO:0010334//GO:0046872</td><td>nr -</td></tr>
<tr><td>Unigene113992_All</td><td>1553</td><td>0.00</td><td>0.00</td><td>0.00</td><td>0.00</td><td>0.00</td><td>0.00</td><td>1.33</td><td>4.17</td><td>0.10</td><td>null</td><td>null</td><td></td><td>null</td><td>null</td><td></td><td>null</td><td>null</td><td></td><td>null</td><td>null</td><td></td><td>1.64E+00</td><td>5.07E-13</td><td>Up</td><td>-3.70E+00</td><td>5.61E-11</td><td>Down</td><td>Unigene113992_All//gi|115463921|ref|NP_001055560.1|//1.00E-123//Os05g0417100 [Oryza sativa Japonica Group] >gi|113579111|dbj|BAF17474.1| Os05g0417100 [Oryza sativa Japonica Group]</td><td>Unigene113992_All//1.00E-117//AT5G36950| DegP10 (DegP protease 10); catalytic/ protein binding / serine-type endopeptidase/ serine-type peptidase</td><td></td><td></td><td></td><td></td><td>nr -</td></tr>
<tr><td>Unigene113997_All</td><td>295</td><td>0.00</td><td>0.34</td><td>0.00</td><td>0.00</td><td>0.00</td><td>0.00</td><td>0.14</td><td>2.69</td><td>0.54</td><td>8.42E+00</td><td>3.89E-01</td><td></td><td>null</td><td>null</td><td></td><td>null</td><td>null</td><td></td><td>null</td><td>null</td><td></td><td>4.23E+00</td><td>1.05E-04</td><td>Up</td><td>1.91E+00</td><td>3.78E-01</td><td></td><td></td><td></td><td></td><td></td><td></td><td></td><td></td></tr>
</table>

Unigene1140_All	1470	4.24	4.24	3.33	7.96	9.14	6.36	18.29	9.10	11.25	1.40E-03	9.96E-01		-3.46E-01	1.78E-01		1.98E-01	2.46E-01		-3.24E-01	6.03E-02		-1.01E+00	1.24E-24	Down	-7.02E-01	4.29E-14		Unigene1140_All//gi|71727712|gb|AAZ39949.1|//1.00E-175//serine/threonine protein kinase [Gossypium hirsutum]	Unigene1140_All//8.00E-155//AT5G08160| ATPK3; ATP binding / kinase/ protein kinase/ protein serine/threonine kinase	Unigene1140_All//0.00E+00//TC134593				nr -
Unigene114003_All	206	0.00	0.00	0.00	0.00	0.00	0.00	11.70	5.68	0.00	null	null		null	null		null	null		null	null		-1.04E+00	4.61E-03		-1.35E+01	9.62E-18	Down	Unigene114003_All//gi|158529884|gb|ABW71681.1|//2.00E-27//actin [Glycyrrhiza uralensis]	Unigene114003_All//9.00E-29//AT5G59370| ACT4 (ACTIN 4); structural constituent of cytoskeleton	Unigene114003_All//4.00E-24//TC153744		ko04145|Phagosome	GO:0032559//GO:0043232	nr +
Unigene114010_All	401	0.00	0.00	0.00	0.00	0.00	0.00	0.11	1.04	3.78	null	null		null	null		null	null		null	null		3.31E+00	2.00E-02		5.16E+00	1.25E-09	Up	Unigene114010_All//gi|145335895|ref|NP_173359.3|//3.00E-53//FMO1 (FLAVIN-DEPENDENT MONOOXYGENASE 1); FAD binding / NADP or NADPH binding / electron carrier/ flavin-containing monooxygenase/ monooxygenase/ oxidoreductase [Arabidopsis thaliana] >gi|75174733|sp|Q9LMA1.1|FMO1_ARATH RecName: Full=Probable flavin-containing monooxygenase 1 >gi|8954062|gb|AAF82235.1|AC069143_11 Contains similarity to a dimethylaniline monooxygenase (N-oxide forming) 2 (pulmonary flavin-containing monooxygenase 2) (FMO 2) (Dimethylaniline oxidase 2) (FMO 1B1) from Oryctolagus cuniculus gi|120434 and contains a flavin-binding monooxygenase-like PF|00743 domain [Arabidopsis thaliana]	Unigene114010_All//2.00E-54//AT1G19250| FMO1 (FLAVIN-DEPENDENT MONOOXYGENASE 1); FAD binding / NADP or NADPH binding / electron carrier/ flavin-containing monooxygenase/ monooxygenase/ oxidoreductase				GO:0002218//GO:0005789//GO:0008152//GO:0009814//GO:0016709//GO:0030554//GO:0033554	nr +
Unigene114022_All	348	0.00	0.00	0.00	0.00	0.00	0.00	19.07	12.13	2.86	null	null		null	null		null	null		null	null		-6.53E-01	1.29E-03		-2.74E+00	1.44E-25	Down							ESTscan +
Unigene114025_All	216	5.33	5.86	7.89	11.07	20.88	4.18	67.34	34.25	190.34	1.38E-01	8.05E-01		5.66E-01	2.83E-01		9.16E-01	8.40E-04		-1.40E+00	8.07E-04	Down	-9.75E-01	5.04E-13		1.50E+00	3.46E-70	Up	Unigene114025_All//gi|115338597|gb|ABI94388.1|//1.00E-14//isopentyl diphosphate isomerase [Gossypium barbadense]		Unigene114025_All//4.00E-52//gi|73848414|gb|DT457434.1|DT457434			GO:0006720//GO:0016863	nr -
Unigene114030_All	210	0.25	1.45	0.00	0.00	0.00	0.00	9.87	22.29	0.57	2.54E+00	1.51E-01		-7.96E+00	6.30E-01		null	null		null	null		1.18E+00	4.06E-06	Up	-4.12E+00	8.26E-12	Down	Unigene114030_All//gi|168068200|ref|XP_001785975.1|//2.00E-21//predicted protein [Physcomitrella patens subsp. patens] >gi|162662338|gb|EDQ49215.1| predicted protein [Physcomitrella patens subsp. patens]		Unigene114030_All//2.00E-35//gi|78348172|gb|DT568446.1|DT568446				nr -
Unigene114049_All	338	0.00	0.00	0.00	0.59	1.02	0.27	0.00	5.69	0.24	null	null		null	null		7.89E-01	5.96E-01		-1.14E+00	5.70E-01		1.25E+01	1.55E-13	Up	7.88E+00	4.06E-01				Unigene114049_All//8.00E-98//DW509472				
Unigene114053_All	506	0.31	1.50	0.53	0.20	1.02	0.80	1.25	4.30	1.50	2.28E+00	1.60E-02		7.62E-01	6.44E-01		2.37E+00	5.64E-02		2.03E+00	1.49E-01		1.78E+00	2.47E-05	Up	2.55E-01	7.07E-01				Unigene114053_All//6.00E-07//TC133360				
Unigene114063_All	218	0.00	0.00	0.00	0.00	0.00	0.00	0.58	4.22	0.00	null	null		null	null		null	null		null	null		2.86E+00	4.09E-04	Up	-9.18E+00	2.00E-01		Unigene114063_All//gi|1350664|sp|P49627.1|RL13_TOBAC//3.00E-07//RecName: Full=60S ribosomal protein L13; AltName: Full=Clone 6.2.1 protein	Unigene114063_All//2.00E-06//AT3G49010| ATBBC1 (ARABIDOPSIS THALIANA BREAST BASIC CONSERVED 1); structural constituent of ribosome			ko03010|Ribosome	GO:0005622	nr -
Unigene114066_All	497	0.00	21.92	11.46	0.10	0.07	0.18	2.81	3.87	3.45	1.44E+01	1.89E-62	Up	1.35E+01	9.59E-32	Up	-5.32E-01	8.27E-01		8.59E-01	7.96E-01		4.63E-01	2.89E-01		2.96E-01	5.24E-01		Unigene114066_All//gi|145408580|ref|YP_001152204.1|//1.00E-19//ORF64d [Pinus koraiensis] >gi|145048829|gb|ABP35447.1| ORF64d [Pinus koraiensis]		Unigene114066_All//4.00E-57//TC160492			GO:0009536	nr +
Unigene114068_All	296	0.00	0.00	0.00	0.00	0.00	0.00	1.71	1.98	0.00	null	null		null	null		null	null		null	null		2.06E-01	8.00E-01		-1.07E+01	5.54E-04	Down	Unigene114068_All//gi|297812013|ref|XP_002873890.1|//6.00E-33//40S ribosomal protein S16 [Arabidopsis lyrata subsp. lyrata] >gi|297319727|gb|EFH50149.1| 40S ribosomal protein S16 [Arabidopsis lyrata subsp. lyrata]	Unigene114068_All//1.00E-32//AT5G18380| 40S ribosomal protein S16 (RPS16C)			ko03010|Ribosome	GO:0005198//GO:0010467//GO:0030529	nr -
Unigene11407_All	1956	5.08	10.18	6.07	9.17	8.34	9.63	27.17	27.78	28.90	1.00E+00	5.61E-15	Up	2.56E-01	1.54E-01		-1.36E-01	3.69E-01		7.06E-02	6.56E-01		3.19E-02	6.88E-01		8.91E-02	1.97E-01		Unigene11407_All//gi|62857010|dbj|BAD95890.1|//0.00E+00//Ser/Thr protein kinase [Lotus japonicus]	Unigene11407_All//0.00E+00//AT4G24400| CIPK8 (CBL-INTERACTING PROTEIN KINASE 8); kinase/ protein kinase	Unigene11407_All//0.00E+00//TC164614		ko04140|Regulation of autophagy	GO:0004674//GO:0006464//GO:0009746//GO:0010035//GO:0016020//GO:0023060//GO:0032559//GO:0048513	nr -
Unigene114075_All	305	0.00	0.00	0.00	0.00	0.00	0.30	2.08	8.91	96.66	null	null		null	null		null	null		8.21E+00	4.80E-01		2.10E+00	6.99E-08	Up	5.54E+00	5.62E-186	Up	Unigene114075_All//gi|115442351|ref|NP_001045455.1|//2.00E-13//Os01g0958700 [Oryza sativa Japonica Group] >gi|113534986|dbj|BAF07369.1| Os01g0958700 [Oryza sativa Japonica Group]	Unigene114075_All//8.00E-14//AT4G24290| FUNCTIONS IN: molecular_function unknown; INVOLVED IN: biological_process unknown; LOCATED IN: plasma membrane; EXPRESSED IN: 18 plant structures; EXPRESSED DURING: 9 growth stages; CONTAINS InterPro DOMAIN/s: Membrane attack complex component/perforin/complement C9 (InterPro:IPR001862); BEST Arabidopsis thaliana protein match is: NSL1 (necrotic spotted lesions 1) (TAIR:AT1G28380.1); Has 124 Blast hits to 124 proteins in 27 species: Archae - 0; Bacteria - 0; Metazoa - 41; Fungi - 0; Plants - 78; Viruses - 0; Other Eukaryotes - 5 (source: NCBI BLink).	Unigene114075_All//4.00E-93//DT466716			GO:0016020	nr -
Unigene11408_All	1688	0.37	0.42	0.28	1.15	1.39	1.95	2.63	0.79	1.53	1.76E-01	8.17E-01		-3.90E-01	6.46E-01		2.69E-01	5.74E-01		7.63E-01	2.66E-02		-1.73E+00	8.27E-10	Down	-7.78E-01	1.69E-03		Unigene11408_All//gi|115448237|ref|NP_001047898.1|//0.00E+00//Os02g0710900 [Oryza sativa Japonica Group] >gi|113537429|dbj|BAF09812.1| Os02g0710900 [Oryza sativa Japonica Group]	Unigene11408_All//0.00E+00//AT4G16660| heat shock protein 70, putative / HSP70, putative			ko04141|Protein processing in endoplasmic reticulum	GO:0008152//GO:0009536//GO:0016020//GO:0016628//GO:0031410//GO:0032559//GO:0050896	nr -
Unigene114080_All	583	0.00	0.00	0.00	0.00	0.00	0.00	0.51	2.22	0.00	null	null		null	null		null	null		null	null		2.13E+00	3.40E-04	Up	-8.99E+00	1.58E-02								ESTscan +
Unigene114083_All	638	0.00	0.00	0.00	0.00	0.00	0.00	2.12	2.10	0.37	null	null		null	null		null	null		null	null		-1.66E-02	1.01E+00		-2.50E+00	2.46E-05	Down	Unigene114083_All//sp|Q86XX4|FRAS1_HUMAN//3.00E-06//Extracellular matrix protein FRAS1 OS=Homo sapiens GN=FRAS1 PE=2 SV=1						swissprot -
Unigene114085_All	401	0.26	3.16	1.46	4.10	3.61	3.27	1.16	0.94	1.09	3.60E+00	2.27E-05	Up	2.48E+00	3.75E-02		-1.85E-01	7.46E-01		-3.28E-01	5.64E-01		-3.06E-01	7.36E-01		-8.58E-02	9.34E-01				Unigene114085_All//8.00E-126//gi|18100915|gb|BM360169.1|BM360169				
Unigene11409_All	914	1.60	1.61	1.22	6.92	3.99	5.73	9.25	3.61	4.23	4.40E-03	9.94E-01		-3.90E-01	5.00E-01		-7.93E-01	2.63E-04		-2.72E-01	2.98E-01		-1.36E+00	7.81E-13	Down	-1.13E+00	3.60E-10	Down	Unigene11409_All//gi|110739797|dbj|BAF01805.1|//1.00E-67//HSP like protein [Arabidopsis thaliana]	Unigene11409_All//4.00E-59//AT4G16660| heat shock protein 70, putative / HSP70, putative	Unigene11409_All//0.00E+00//gi|11201306|gb|BF270311.1|BF270311		ko03040|Spliceosome//ko04141|Protein processing in endoplasmic reticulum//ko04144|Endocytosis//ko03060|Protein export	GO:0009536//GO:0016020//GO:0016491//GO:0031410//GO:0032559	nr +
Unigene114097_All	330	0.00	0.00	0.00	0.00	0.00	0.00	1.67	3.29	0.00	null	null		null	null		null	null		null	null		9.83E-01	9.75E-02		-1.07E+01	2.81E-04	Down			Unigene114097_All//2.00E-09//TC154839				
Unigene1141_All	1545	6.84	14.10	9.03	13.51	11.28	13.71	23.23	22.16	13.82	1.04E+00	2.19E-17	Up	4.01E-01	1.15E-02		-2.61E-01	3.06E-02		2.12E-02	9.00E-01		-6.85E-02	4.93E-01		-7.49E-01	1.18E-20		Unigene1141_All//gi|71727712|gb|AAZ39949.1|//0.00E+00//serine/threonine protein kinase [Gossypium hirsutum]	Unigene1141_All//4.00E-178//AT5G08160| ATPK3; ATP binding / kinase/ protein kinase/ protein serine/threonine kinase	Unigene1141_All//0.00E+00//TC134693			GO:0004672//GO:0006464//GO:0032559	nr +
Unigene114103_All	568	0.00	0.00	0.00	0.00	0.00	0.00	0.00	2.21	0.00	null	null		null	null		null	null		null	null		1.11E+01	7.17E-09	Up	null	null		Unigene114103_All//gi|297719953|ref|NP_001172338.1|//3.00E-06//Os01g0363550 [Oryza sativa Japonica Group] >gi|255673227|dbj|BAH91068.1| Os01g0363550 [Oryza sativa Japonica Group]						nr -
Unigene114108_All	515	0.00	0.00	0.00	0.00	0.00	0.00	1.40	1.06	0.00	null	null		null	null		null	null		null	null		-4.04E-01	6.23E-01		-1.04E+01	1.78E-05	Down	Unigene114108_All//sp|Q869S5|DOKA_DICDI//4.00E-07//Hybrid signal transduction protein dokA OS=Dictyostelium discoideum GN=dokA PE=1 SV=1						swissprot +
Unigene11411_All	5682	6.51	5.23	4.43	5.40	5.62	7.34	6.54	7.22	19.18	-3.17E-01	3.42E-04		-5.55E-01	8.64E-10		5.70E-02	6.26E-01		4.42E-01	3.30E-08		1.43E-01	7.84E-02		1.55E+00	2.47E-194	Up	Unigene11411_All//gi|297791025|ref|XP_002863397.1|//0.00E+00//pentatricopeptide repeat-containing protein [Arabidopsis lyrata subsp. lyrata] >gi|297309232|gb|EFH39656.1| pentatricopeptide repeat-containing protein [Arabidopsis lyrata subsp. lyrata]	Unigene11411_All//0.00E+00//AT5G46580| pentatricopeptide (PPR) repeat-containing protein	Unigene11411_All//0.00E+00//TC154377		ko00230|Purine metabolism//ko00240|Pyrimidine metabolism//ko03020|RNA polymerase	GO:0009536//GO:0016787	nr -
Unigene114113_All	255	4.51	36.76	15.24	15.82	12.96	10.27	9.95	20.65	6.41	3.03E+00	3.88E-31	Up	1.76E+00	5.72E-07	Up	-2.88E-01	3.81E-01		-6.23E-01	4.08E-02		1.05E+00	8.10E-06	Up	-6.35E-01	6.30E-02				Unigene114113_All//1.00E-92//gi|164267296|gb|ES813703.1|ES813703				
Unigene114119_All	554	0.00	0.00	0.00	0.00	0.12	0.33	0.61	2.64	0.58	null	null		null	null		6.96E+00	6.13E-01		8.35E+00	1.95E-01		2.11E+00	1.32E-04	Up	-8.57E-02	9.47E-01				Unigene114119_All//7.00E-07//TC155021				
Unigene11412_All	1387	0.87	4.79	2.00	1.22	0.92	0.91	6.37	16.84	10.71	2.46E+00	5.97E-18	Up	1.20E+00	2.76E-03		-4.11E-01	4.28E-01		-4.22E-01	4.36E-01		1.40E+00	9.98E-36	Up	7.50E-01	4.85E-09				Unigene11412_All//0.00E+00//TC161569				
Unigene114126_All	866	0.06	0.35	0.18	0.17	0.32	0.00	0.98	2.90	0.64	2.54E+00	1.51E-01		1.61E+00	5.11E-01		8.82E-01	5.95E-01		-7.43E+00	2.34E-01		1.57E+00	3.40E-05	Up	-6.00E-01	3.58E-01		Unigene114126_All//gi|158828300|gb|ABW81175.1|//7.00E-12//non-LTR retrotransposon transposase [Arabidopsis cebennensis]						nr +
Unigene11413_All	2503	5.71	13.40	10.53	5.06	8.46	10.50	10.34	21.14	25.24	1.23E+00	3.73E-34	Up	8.84E-01	2.00E-15		7.43E-01	2.79E-11		1.05E+00	1.47E-22	Up	1.03E+00	1.65E-49	Up	1.29E+00	1.61E-85	Up	Unigene11413_All//gi|297843108|ref|XP_002889435.1|//1.00E-166//3-methylcrotonyl-CoA carboxylase 1 [Arabidopsis lyrata subsp. lyrata] >gi|297335277|gb|EFH65694.1| 3-methylcrotonyl-CoA carboxylase 1 [Arabidopsis lyrata subsp. lyrata]	Unigene11413_All//3.00E-167//AT1G03090| MCCA; methylcrotonoyl-CoA carboxylase	Unigene11413_All//0.00E+00//TC164785		ko00280|Valine, leucine and isoleucine degradation		nr -
Unigene114135_All	264	0.00	0.00	0.00	0.00	0.00	0.00	0.00	2.53	0.00	null	null		null	null		null	null		null	null		1.13E+01	7.64E-05	Up	null	null								
Unigene11414_All	3143	10.27	29.52	20.81	8.73	11.48	17.13	28.01	61.16	50.17	1.52E+00	9.93E-129	Up	1.02E+00	2.53E-47	Up	3.95E-01	2.03E-06		9.72E-01	5.52E-40		1.13E+00	4.09E-205	Up	8.41E-01	3.50E-106		Unigene11414_All//gi|297843108|ref|XP_002889435.1|//0.00E+00//3-methylcrotonyl-CoA carboxylase 1 [Arabidopsis lyrata subsp. lyrata] >gi|297335277|gb|EFH65694.1| 3-methylcrotonyl-CoA carboxylase 1 [Arabidopsis lyrata subsp. lyrata]	Unigene11414_All//0.00E+00//AT1G03090| MCCA; methylcrotonoyl-CoA carboxylase	Unigene11414_All//0.00E+00//TC164785		ko00280|Valine, leucine and isoleucine degradation	GO:0005840//GO:0006551//GO:0016421//GO:0019842//GO:0031980//GO:0032559//GO:0046914	nr -
Unigene114144_All	464	0.00	0.00	0.00	0.00	0.00	0.00	1.09	2.25	0.00	null	null		null	null		null	null		null	null		1.04E+00	8.75E-02		-1.01E+01	5.54E-04	Down	Unigene114144_All//gi|301122631|ref|XP_002909042.1|//5.00E-42//40S ribosomal protein S6 [Phytophthora infestans T30-4] >gi|262099804|gb|EEY57856.1| 40S ribosomal protein S6 [Phytophthora infestans T30-4]	Unigene114144_All//4.00E-28//AT4G31700| RPS6 (RIBOSOMAL PROTEIN S6); structural constituent of ribosome			ko03010|Ribosome	GO:0005198//GO:0010467//GO:0030529	nr -
Unigene114158_All	216	0.00	0.00	0.00	0.00	0.00	0.00	0.00	4.06	0.00	null	null		null	null		null	null		null	null		1.20E+01	2.86E-06	Up	null	null								
Unigene11417_All	436	32.40	14.76	23.69	49.24	28.99	22.58	2.72	3.26	4.20	-1.13E+00	3.76E-13	Down	-4.52E-01	3.83E-03		-7.65E-01	2.06E-12		-1.12E+00	9.30E-21	Down	2.64E-01	6.49E-01		6.30E-01	1.31E-01				Unigene11417_All//5.00E-121//gi|164344748|gb|EX166055.1|EX166055				ESTscan +
Unigene114170_All	246	0.00	0.00	0.00	0.00	0.00	0.18	0.17	3.40	0.00	null	null		null	null		null	null		7.52E+00	7.00E-01		4.31E+00	5.68E-05	Up	-7.43E+00	6.15E-01				Unigene114170_All//3.00E-127//DW498401				ESTscan -
Unigene11418_All	743	14.65	13.03	13.76	13.61	8.48	12.46	3.24	8.21	6.38	-1.69E-01	3.65E-01		-9.01E-02	6.28E-01		-6.82E-01	3.95E-05		-1.27E-01	5.65E-01		1.34E+00	2.65E-09	Up	9.76E-01	6.05E-05		Unigene11418_All//gi|53370641|gb|AAU89136.1|//7.00E-11//F-box domain containing protein [Oryza sativa Japonica Group]		Unigene11418_All//0.00E+00//DW498283				nr +

Unigene114182_All	427	0.00	0.00	0.00	0.00	0.00	0.00	0.20	2.25	0.00	null	null		null	null		null	null		null	null		3.51E+00	5.50E-05	Up	-7.63E+00	3.60E-01		Unigene114182_All//gi|297814478|ref|XP_002875122.1|//6.00E-06//hypothetical protein ARALYDRAFT_904467 [Arabidopsis lyrata subsp. lyrata] >gi|297320960|gb|EFH51381.1| hypothetical protein ARALYDRAFT_904467 [Arabidopsis lyrata subsp. lyrata]	Unigene114182_All//7.00E-07//AT5G41210| ATGSTT1 (GLUTATHIONE S-TRANSFERASE THETA 1); glutathione transferase			ko00350|Tyrosine metabolism		nr -
Unigene114185_All	577	0.00	0.00	0.00	0.00	0.00	0.00	0.00	3.62	0.00	null	null		null	null		null	null		null	null		1.18E+01	1.05E-14	Up	null	null		Unigene114185_All//gi|150421627|sp|Q9ZW82.3|PIRL2_ARATH//5.00E-27//RecName: Full=Pirin-like protein At2g43120 >gi|119360079|gb|ABL66768.1| At2g43120 [Arabidopsis thaliana]	Unigene114185_All//1.00E-28//AT2G43120| pirin, putative				GO:0005515//GO:0016020//GO:0043231	nr +
Unigene114186_All	327	0.00	0.00	0.00	0.00	0.00	0.00	2.97	3.45	0.24	null	null		null	null		null	null		null	null		2.15E-01	7.15E-01		-3.61E+00	2.14E-05	Down	Unigene114186_All//gi|257219570|gb|ACV50438.1|//9.00E-23//ribosomal protein L37 [Jatropha curcas]	Unigene114186_All//2.00E-08//AT1G52300| 60S ribosomal protein L37 (RPL37B)			ko03010|Ribosome	GO:0003723//GO:0005198//GO:0009987//GO:0010467//GO:0030529//GO:0043169//GO:0043231//GO:0043232	nr -
Unigene114188_All	257	0.00	0.00	0.00	0.00	0.00	0.00	0.00	2.60	0.78	null	null		null	null		null	null		null	null		1.13E+01	7.64E-05	Up	9.60E+00	7.91E-02								
Unigene11419_All	805	13.84	12.65	15.74	25.50	27.16	19.19	7.83	5.50	2.13	-1.30E-01	4.91E-01		1.86E-01	3.06E-01		9.14E-02	5.47E-01		-4.10E-01	6.09E-04		-5.08E-01	1.63E-02		-1.88E+00	1.21E-15	Down	Unigene11419_All//gi|297724159|ref|NP_001174443.1|//6.00E-06//Os05g0434000 [Oryza sativa Japonica Group] >gi|255676397|dbj|BAH93171.1| Os05g0434000 [Oryza sativa Japonica Group]		Unigene11419_All//0.00E+00//BM357993				nr -
Unigene114201_All	387	0.00	0.00	0.00	0.00	0.00	0.00	0.00	1.62	0.00	null	null		null	null		null	null		null	null		1.07E+01	1.47E-04	Up	null	null								ESTscan +
Unigene114202_All	217	2.89	0.00	1.23	1.38	1.27	0.62	0.00	0.00	0.18	-1.15E+01	7.92E-04	Down	-1.24E+00	2.13E-01		-1.18E-01	9.01E-01		-1.14E+00	4.58E-01		null	null		7.52E+00	6.47E-01		Unigene114202_All//gi|3860321|emb|CAA10128.1|//1.00E-13//beta-galactosidase [Cicer arietinum]	Unigene114202_All//3.00E-10//AT4G26140| BGAL12 (beta-galactosidase 12); beta-galactosidase/ catalytic/ cation binding	Unigene114202_All//1.00E-49//CO496844		ko00052|Galactose metabolism//ko00600|Sphingolipid metabolism//ko00511|Other glycan degradation	GO:0031410	nr +
Unigene114204_All	437	0.00	0.00	0.00	0.00	0.00	0.00	1.16	3.06	0.00	null	null		null	null		null	null		null	null		1.40E+00	9.02E-03		-1.02E+01	5.54E-04	Down							ESTscan +
Unigene114209_All	245	14.74	2.28	6.09	14.84	18.13	15.30	28.65	11.43	24.88	-2.70E+00	3.87E-11	Down	-1.28E+00	2.41E-04	Down	2.89E-01	3.66E-01		4.38E-02	9.19E-01		-1.33E+00	1.95E-10	Down	-2.04E-01	3.36E-01		Unigene114209_All//gi|297606737|ref|NP_001058899.2|//2.00E-10//Os07g0150200 [Oryza sativa Japonica Group] >gi|255677517|dbj|BAF20813.2| Os07g0150200 [Oryza sativa Japonica Group]	Unigene114209_All//6.00E-08//AT2G32060| 40S ribosomal protein S12 (RPS12C)	Unigene114209_All//4.00E-136//TC162854		ko03010|Ribosome	GO:0005198//GO:0010467//GO:0030529	nr -
Unigene11421_All	928	2.09	3.55	4.48	4.46	5.12	3.65	1.09	1.35	0.60	7.67E-01	2.46E-02		1.10E+00	4.78E-04	Up	2.01E-01	5.52E-01		-2.88E-01	3.98E-01		3.05E-01	6.11E-01		-8.64E-01	1.40E-01		Unigene11421_All//gi|115442463|ref|NP_001045511.1|//3.00E-06//Os01g0967700 [Oryza sativa Japonica Group] >gi|113535042|dbj|BAF07425.1| Os01g0967700 [Oryza sativa Japonica Group]	Unigene11421_All//3.00E-05//AT4G29090| reverse transcriptase, putative / RNA-dependent DNA polymerase, putative	Unigene11421_All//0.00E+00//TC129954				nr +
Unigene114225_All	469	0.00	0.00	0.00	0.00	0.00	0.00	0.18	2.23	0.00	null	null		null	null		null	null		null	null		3.63E+00	1.71E-05	Up	-7.49E+00	3.60E-01		Unigene114225_All//sp|P15214|GST_PROMI//6.00E-16//Glutathione S-transferase GST-6.0 OS=Proteus mirabilis GN=gstB PE=1 SV=2						swissprot +
Unigene114236_All	793	0.00	0.00	0.00	0.00	0.00	0.00	0.43	3.74	0.00	null	null		null	null		null	null		null	null		3.13E+00	6.28E-13	Up	-8.74E+00	8.15E-03		Unigene114236_All//gi|192822681|gb|ACF06186.1|//2.00E-13//heat shock protein 20 [Fucus serratus]	Unigene114236_All//2.00E-13//AT1G07400| 17.8 kDa class I heat shock protein (HSP17.8-CI)				GO:0006950	nr -
Unigene11424_All	1185	0.71	0.64	1.12	2.23	2.91	2.78	6.21	2.96	5.04	-1.39E-01	8.39E-01		6.69E-01	2.69E-01		3.83E-01	2.92E-01		3.21E-01	3.94E-01		-1.07E+00	5.70E-08	Down	-3.00E-01	1.21E-01		Unigene11424_All//gi|9743334|gb|AAF97958.1|AC000103_8//8.00E-31//F21J9.12 [Arabidopsis thaliana]	Unigene11424_All//8.00E-25//AT1G24460| unknown protein					nr +
Unigene114247_All	271	2.70	3.37	3.54	11.95	10.55	4.17	15.91	11.72	14.26	3.16E-01	6.32E-01		3.88E-01	6.08E-01		-1.80E-01	6.16E-01		-1.52E+00	1.86E-05	Down	-4.41E-01	9.99E-02		-1.58E-01	5.86E-01		Unigene114247_All//gi|3281851|emb|CAA19746.1|//1.00E-09//RNA lariat debranching enzyme - like protein [Arabidopsis thaliana] >gi|7270080|emb|CAB79895.1| RNA lariat debranching enzyme-like protein [Arabidopsis thaliana]	Unigene114247_All//5.00E-11//AT4G31770| calcineurin-like phosphoesterase family protein	Unigene114247_All//7.00E-110//TC145739			GO:0003824	nr +
Unigene114252_All	239	0.00	0.00	0.00	0.00	0.00	0.00	1.59	5.77	0.00	null	null		null	null		null	null		null	null		1.86E+00	7.81E-04	Up	-1.06E+01	4.20E-03		Unigene114252_All//sp|P0C5Q0|YL54F_YEAST//7.00E-06//Uncharacterized protein YLR154W-F OS=Saccharomyces cerevisiae GN=YLR154W-F PE=2 SV=1		Unigene114252_All//8.00E-23//ES844801				swissprot -
Unigene114259_All	571	0.00	0.00	0.00	0.00	0.00	0.00	0.00	5.20	0.00	null	null		null	null		null	null		null	null		1.23E+01	7.12E-21	Up	null	null								ESTscan +
Unigene114260_All	379	0.00	0.00	0.00	0.39	0.64	0.00	0.00	2.10	0.00	null	null		null	null		6.90E-01	7.32E-01		-8.62E+00	2.34E-01		1.10E+01	1.07E-05	Up	null	null				Unigene114260_All//9.00E-30//TC155768				
Unigene114266_All	388	0.00	0.00	0.00	0.00	0.00	0.00	1.74	4.31	0.10	null	null		null	null		null	null		null	null		1.31E+00	5.02E-03		-4.09E+00	3.10E-04	Down	Unigene114266_All//gi|116222125|gb|ABJ80933.1|//2.00E-71//actin [Leucocryptos marina]	Unigene114266_All//8.00E-72//AT3G12110| ACT11 (actin-11); structural constituent of cytoskeleton	Unigene114266_All//2.00E-64//TC153744		ko04145|Phagosome	GO:0032559//GO:0043232	nr +
Unigene114270_All	350	0.00	0.00	0.00	0.28	0.00	0.00	0.60	2.99	1.25	null	null		null	null		-8.15E+00	3.15E-01		-8.15E+00	4.03E-01		2.31E+00	8.56E-04	Up	1.05E+00	2.98E-01		Unigene114270_All//gi|92897781|gb|ABE93893.1|//1.00E-16//Heavy metal transport/detoxification protein [Medicago truncatula]	Unigene114270_All//2.00E-08//AT1G01490| heavy-metal-associated domain-containing protein	Unigene114270_All//4.00E-07//EX165152			GO:0006812//GO:0043169	nr +
Unigene114278_All	330	0.00	0.00	0.00	0.00	0.00	0.00	0.26	2.53	0.12	null	null		null	null		null	null		null	null		3.31E+00	3.10E-04	Up	-1.09E+00	6.77E-01								
Unigene114285_All	227	12.22	4.46	15.25	16.68	27.46	16.12	6.71	4.23	2.46	-1.45E+00	2.37E-04	Down	3.20E-01	3.86E-01		7.19E-01	1.50E-03		-4.95E-02	8.98E-01		-6.63E-01	1.73E-01		-1.45E+00	2.57E-03				Unigene114285_All//2.00E-48//TC134423				
Unigene114288_All	755	0.14	0.00	0.00	0.40	0.41	0.66	0.95	3.49	0.95	-7.11E+00	3.75E-01		-7.11E+00	3.97E-01		5.22E-02	9.98E-01		7.33E-01	5.16E-01		1.87E+00	1.03E-06	Up	-3.30E-03	9.90E-01				Unigene114288_All//6.00E-08//gi|3325657|gb|AI054543.1|AI054543				
Unigene114294_All	388	0.00	0.00	0.00	0.00	0.00	0.00	1.85	1.18	0.10	null	null		null	null		null	null		null	null		-6.45E-01	4.01E-01		-4.17E+00	1.64E-04	Down			Unigene114294_All//2.00E-30//ES828348				
Unigene114296_All	490	1.71	1.65	1.85	0.51	0.28	0.46	1.21	5.20	0.81	-4.63E-02	9.64E-01		1.13E-01	8.72E-01		-8.55E-01	5.74E-01		-1.41E-01	9.29E-01		2.11E+00	1.80E-07	Up	-5.71E-01	4.78E-01		Unigene114296_All//gi|34014756|gb|AAQ56194.1|//2.00E-17//aminotransferase 1 [Cucumis melo]	Unigene114296_All//2.00E-11//AT2G13360| AGT (ALANINE:GLYOXYLATE AMINOTRANSFERASE); alanine-glyoxylate transaminase/ serine-glyoxylate transaminase/ serine-pyruvate transaminase	Unigene114296_All//2.00E-170//gi|48798267|gb|CO099581.1|CO099581		ko00250|Alanine, aspartate and glutamate metabolism//ko00260|Glycine, serine and threonine metabolism	GO:0008483//GO:0048037	nr +
Unigene1143_All	3423	3.38	5.46	4.56	14.49	17.19	21.92	8.70	4.05	4.06	6.93E-01	6.70E-08		4.32E-01	3.42E-03		2.46E-01	1.67E-04		5.96E-01	3.60E-24		-1.10E+00	2.72E-31	Down	-1.10E+00	4.31E-32	Down	Unigene1143_All//gi|224058639|ref|XP_002299579.1|//1.00E-126//cytochrome P450 [Populus trichocarpa] >gi|222846837|gb|EEE84384.1| cytochrome P450 [Populus trichocarpa]	Unigene1143_All//2.00E-106//AT2G45570| CYP76C2; electron carrier/ heme binding / iron ion binding / monooxygenase/ oxygen binding	Unigene1143_All//0.00E+00//TC134926		ko00945|Stilbenoid, diarylheptanoid and gingerol biosynthesis//ko00903|Limonene and pinene degradation		nr -
Unigene11430_All	1542	6.58	3.15	7.29	2.62	2.68	1.20	0.05	0.05	0.10	-1.06E+00	9.19E-09	Down	1.47E-01	4.43E-01		3.44E-02	9.35E-01		-1.12E+00	2.00E-04	Down	-1.59E-02	1.01E+00		9.15E-01	6.25E-01		Unigene11430_All//gi|297814053|ref|XP_002874910.1|//3.00E-52//TOC159 [Arabidopsis lyrata subsp. lyrata] >gi|297320747|gb|EFH51169.1| TOC159 [Arabidopsis lyrata subsp. lyrata]	Unigene11430_All//5.00E-42//AT4G02510| TOC159 (TRANSLOCON AT THE OUTER ENVELOPE MEMBRANE OF CHLOROPLASTS 159); transmembrane receptor	Unigene11430_All//0.00E+00//TC142232			GO:0006605//GO:0009527//GO:0017111//GO:0032555	nr +
Unigene114302_All	453	0.00	0.00	0.00	0.00	0.00	0.00	1.40	2.49	0.00	null	null		null	null		null	null		null	null		8.31E-01	1.51E-01		-1.05E+01	7.11E-05	Down	Unigene114302_All//gi|195605616|gb|ACG24638.1|//1.00E-06//translationally-controlled tumor protein [Zea mays] >gi|195622768|gb|ACG33214.1| translationally-controlled tumor protein [Zea mays]						nr +
Unigene114311_All	360	0.00	0.00	0.00	0.00	0.00	0.00	0.70	4.06	0.55	null	null		null	null		null	null		null	null		2.53E+00	1.65E-05	Up	-3.49E-01	7.67E-01								
Unigene114319_All	477	0.00	0.00	0.00	0.10	0.29	0.38	0.18	2.19	0.00	null	null		null	null		1.47E+00	6.19E-01		1.86E+00	4.59E-01		3.63E+00	1.71E-05	Up	-7.47E+00	3.60E-01								
Unigene11433_All	2843	5.23	4.76	2.55	196.96	202.97	199.61	30.53	37.24	15.69	-1.35E-01	3.86E-01		-1.04E+00	1.75E-11	Down	4.33E-02	5.70E-02		1.92E-02	4.86E-01		2.87E-01	1.45E-10		-9.60E-01	8.38E-74		Unigene11433_All//gi|258489633|gb|ACV72640.1|//0.00E+00//sucrose synthase 1 [Gossypium hirsutum]	Unigene11433_All//0.00E+00//AT3G43190| SUS4; UDP-glycosyltransferase/ sucrose synthase/ transferase, transferring glycosyl groups	Unigene11433_All//0.00E+00//TC132647		ko00500|Starch and sucrose metabolism	GO:0005984//GO:0035251//GO:0044403	nr -
Unigene11435_All	2524	22.76	6.69	7.30	26.53	12.84	14.39	15.38	33.30	12.63	-1.77E+00	2.13E-99	Down	-1.64E+00	1.69E-86	Down	-1.05E+00	1.54E-64	Down	-8.83E-01	1.00E-42		1.11E+00	1.57E-88	Up	-2.84E-01	1.63E-04		Unigene11435_All//gi|211906466|gb|ACJ11726.1|//1.00E-173//serine hydroxymethyltransferase [Gossypium hirsutum]	Unigene11435_All//2.00E-161//AT4G13930| SHM4 (serine hydroxymethyltransferase 4); catalytic/ glycine hydroxymethyltransferase/ pyridoxal phosphate binding	Unigene11435_All//0.00E+00//gi|78340525|gb|DT560799.1|DT560799		ko00460|Cyanoamino acid metabolism//ko00260|Glycine, serine and threonine metabolism//ko00670|One carbon pool by folate		nr -
Unigene114350_All	276	0.00	0.00	0.00	0.00	0.00	0.00	1.99	1.82	0.00	null	null		null	null		null	null		null	null		-1.32E-01	8.80E-01		-1.10E+01	2.81E-04	Down			Unigene114350_All//6.00E-27//FL576679				
Unigene114365_All	233	0.00	0.43	0.00	0.21	0.59	0.39	0.73	5.56	1.71	8.76E+00	3.89E-01		null	null		1.47E+00	6.19E-01		8.59E-01	7.96E-01		2.94E+00	1.14E-05	Up	1.24E+00	2.51E-01		Unigene114365_All//gi|255540151|ref|XP_002511140.1|//4.00E-07//conserved hypothetical protein [Ricinus communis] >gi|223550255|gb|EEF51742.1| conserved hypothetical protein [Ricinus communis]						nr -
Unigene114372_All	228	0.00	0.00	0.00	0.00	0.00	0.00	0.37	4.03	0.00	null	null		null	null		null	null		null	null		3.44E+00	9.84E-05	Up	-8.53E+00	3.60E-01								ESTscan -
Unigene114374_All	461	0.00	0.00	0.00	0.00	0.00	0.00	19.26	390.03	0.95	null	null		null	null		null	null		null	null		4.34E+00	0.00E+00	Up	-4.34E+00	1.59E-50	Down	Unigene114374_All//gi|1545805|dbj|BAA10929.1|//4.00E-12//cytochrome P450 like_TBP [Nicotiana tabacum]		Unigene114374_All//5.00E-41//gi|73864682|gb|DT467421.1|DT467421			GO:0016491//GO:0043231	nr -
Unigene114378_All	521	0.20	0.00	0.00	0.57	0.00	0.00	0.32	9.31	1.76	-7.65E+00	3.75E-01		-7.65E+00	3.97E-01		-9.16E+00	2.02E-02		-9.16E+00	4.00E-02		4.84E+00	1.77E-28	Up	2.44E+00	1.06E-03		Unigene114378_All//gi|297849980|ref|XP_002892871.1|//6.00E-72//ATPDR12/PDR12 [Arabidopsis lyrata subsp. lyrata] >gi|297338713|gb|EFH69130.1| ATPDR12/PDR12 [Arabidopsis lyrata subsp. lyrata]	Unigene114378_All//6.00E-60//AT1G15520| PDR12 (PLEIOTROPIC DRUG RESISTANCE 12); ATPase, coupled to transmembrane movement of substances	Unigene114378_All//0.00E+00//TC164825		ko03040|Spliceosome	GO:0032559//GO:0042626//GO:0043231	nr -
Unigene11438_All	580	2.26	1.31	2.57	6.79	3.62	2.34	12.83	13.55	6.11	-7.83E-01	1.81E-01		1.89E-01	7.21E-01		-9.06E-01	1.64E-03		-1.54E+00	1.36E-06	Down	7.85E-02	7.15E-01		-1.07E+00	2.31E-08	Down			Unigene11438_All//5.00E-122//DR461849		ko04146|Peroxisome//ko00071|Fatty acid metabolism		kegg -
Unigene11439_All	674	5.05	6.69	8.22	8.65	4.19	6.16	4.01	3.16	3.01	4.07E-01	1.61E-01		7.04E-01	7.51E-03		-1.05E+00	4.84E-06	Down	-4.88E-01	5.04E-02		-3.44E-01	3.54E-01		-4.13E-01	2.22E-01				Unigene11439_All//6.00E-125//TC160702				
Unigene114400_All	709	0.00	0.00	0.00	0.00	0.00	0.00	0.00	3.12	0.00	null	null		null	null		null	null		null	null		1.16E+01	1.39E-15	Up	null	null								ESTscan -
Unigene114411_All	686	0.00	0.00	0.00	0.00	0.00	0.00	0.92	2.19	0.00	null	null		null	null		null	null		null	null		1.25E+00	1.16E-02		-9.85E+00	7.11E-05	Down	Unigene114411_All//gi|298706692|emb|CBJ34222.1|//5.00E-06//conserved unknown protein [Ectocarpus siliculosus] >gi|298713886|emb|CBJ33762.1| conserved unknown protein [Ectocarpus siliculosus]		Unigene114411_All//8.00E-07//ES794483				nr -
Unigene114416_All	296	0.00	0.00	0.00	0.00	0.00	0.00	1.71	5.65	0.00	null	null		null	null		null	null		null	null		1.72E+00	4.01E-04	Up	-1.07E+01	5.54E-04	Down							ESTscan +
Unigene114417_All	365	0.00	0.00	0.00	0.00	0.00	0.00	0.00	2.40	0.00	null	null		null	null		null	null		null	null		1.12E+01	2.86E-06	Up	null	null								ESTscan +
Unigene11442_All	2287	3.41	4.85	2.98	8.30	6.23	5.67	14.72	14.93	6.43	5.09E-01	2.93E-03		-1.94E-01	3.94E-01		-4.13E-01	5.18E-04		-5.50E-01	7.85E-06		2.09E-02	8.37E-01		-1.20E+00	2.71E-41	Down	Unigene11442_All//gi|62321026|dbj|BAD94085.1|//2.00E-10//A6 anther-specific protein [Arabidopsis thaliana]	Unigene11442_All//1.00E-11//AT4G14070| AAE15 (acyl-activating enzyme 15); long-chain-fatty-acid-[acyl-carrier-protein] ligase	Unigene11442_All//0.00E+00//gi|11204532|gb|BF273462.1|BF273462		ko04146|Peroxisome//ko00071|Fatty acid metabolism	GO:0006633//GO:0009526//GO:0015645	nr -

<table>
<tr><td>Unigene114421_All</td><td>373</td><td>0.00</td><td>0.00</td><td>0.00</td><td>0.13</td><td>0.00</td><td>0.00</td><td>0.11</td><td>1.90</td><td>0.00</td><td>null</td><td>null</td><td></td><td>null</td><td>null</td><td></td><td>-7.06E+00</td><td>5.60E-01</td><td></td><td>-7.06E+00</td><td>6.41E-01</td><td></td><td>4.07E+00</td><td>3.51E-04</td><td>Up</td><td>-6.83E+00</td><td>6.15E-01</td><td></td><td>Unigene114421_All//gi|224064011|ref|XP_002301347.1|//5.00E-37//white-brown-complex ABC transporter family [Populus trichocarpa]
>gi|222843073|gb|EEE80620.1| white-brown-complex ABC transporter family [Populus trichocarpa]</td><td>Unigene114421_All//4.00E-38//AT3G53510| ABC transporter family protein</td><td></td><td></td><td>ko03040|Spliceosome</td><td>GO:0000166//GO:0015405//GO:0016020//GO:0017111//GO:0051707</td><td>nr +</td></tr>
<tr><td>Unigene114427_All</td><td>330</td><td>1.90</td><td>1.23</td><td>6.29</td><td>1.66</td><td>2.40</td><td>1.64</td><td>4.74</td><td>2.15</td><td>2.17</td><td>-6.31E-01</td><td>4.75E-01</td><td></td><td>1.73E+00</td><td>5.70E-04</td><td>Up</td><td>5.31E-01</td><td>5.40E-01</td><td></td><td>-1.59E-02</td><td>9.75E-01</td><td></td><td>-1.14E+00</td><td>1.72E-02</td><td></td><td>-1.13E+00</td><td>1.41E-02</td><td></td><td>Unigene114427_All//gi|30695682|ref|NP_191845.2|//1.00E-17//esterase/lipase/thioesterase family protein [Arabidopsis thaliana]</td><td>Unigene114427_All//6.00E-19//AT3G62860| esterase/lipase/thioesterase family protein</td><td>Unigene114427_All//2.00E-138//CO099437</td><td></td><td></td><td>GO:0004091//GO:0016020</td><td>nr +</td></tr>
<tr><td>Unigene114430_All</td><td>323</td><td>0.00</td><td>0.00</td><td>0.00</td><td>0.00</td><td>0.00</td><td>0.00</td><td>2.23</td><td>9.32</td><td>0.37</td><td>null</td><td>null</td><td></td><td>null</td><td>null</td><td></td><td>null</td><td>null</td><td></td><td>null</td><td>null</td><td></td><td>2.07E+00</td><td>1.69E-08</td><td>Up</td><td>-2.59E+00</td><td>2.81E-03</td><td></td><td>Unigene114430_All//gi|298706692|emb|CBJ34222.1|//2.00E-17//conserved unknown protein [Ectocarpus siliculosus]
>gi|298713886|emb|CBJ33762.1| conserved unknown protein [Ectocarpus siliculosus]</td><td></td><td>Unigene114430_All//6.00E-40//gi|78348172|gb|DT568446.1|DT568446</td><td></td><td></td><td></td><td>nr -</td></tr>
<tr><td>Unigene114431_All</td><td>264</td><td>0.00</td><td>0.00</td><td>0.00</td><td>0.00</td><td>0.00</td><td>0.00</td><td>2.88</td><td>2.69</td><td>0.00</td><td>null</td><td>null</td><td></td><td>null</td><td>null</td><td></td><td>null</td><td>null</td><td></td><td>null</td><td>null</td><td></td><td>-9.91E-02</td><td>8.96E-01</td><td></td><td>-1.15E+01</td><td>8.87E-06</td><td>Down</td><td></td><td></td><td>Unigene114431_All//1.00E-15//TC134836</td><td></td><td></td><td></td><td></td></tr>
<tr><td>Unigene114437_All</td><td>437</td><td>0.00</td><td>0.00</td><td>0.00</td><td>0.00</td><td>0.00</td><td>0.00</td><td>1.84</td><td>3.35</td><td>0.00</td><td>null</td><td>null</td><td></td><td>null</td><td>null</td><td></td><td>null</td><td>null</td><td></td><td>null</td><td>null</td><td></td><td>8.65E-01</td><td>8.03E-02</td><td></td><td>-1.08E+01</td><td>4.43E-06</td><td>Down</td><td>Unigene114437_All//gi|29539332|dbj|BAC67667.1|//1.00E-57//elongation factor-1alpha [Cyanidioschyzon merolae]</td><td>Unigene114437_All//9.00E-36//AT5G60390| elongation factor 1-alpha / EF-1-alpha</td><td>Unigene114437_All//4.00E-54//gi|11208772|gb|BF277702.1|BF277702</td><td></td><td></td><td>GO:0006412//GO:0008135//GO:0017111//GO:0032561//GO:0044424</td><td>nr -</td></tr>
<tr><td>Unigene114438_All</td><td>552</td><td>0.00</td><td>0.00</td><td>0.00</td><td>0.00</td><td>0.00</td><td>0.00</td><td>3.75</td><td>4.85</td><td>0.43</td><td>null</td><td>null</td><td></td><td>null</td><td>null</td><td></td><td>null</td><td>null</td><td></td><td>null</td><td>null</td><td></td><td>3.69E-01</td><td>3.16E-01</td><td></td><td>-3.12E+00</td><td>1.46E-09</td><td>Down</td><td>Unigene114438_All//gi|4501883|ref|NP_001604.1|//4.00E-82//actin, aortic smooth muscle [Homo sapiens]
>gi|6671507|ref|NP_031418.1| actin, aortic smooth muscle [Mus musculus]
>gi|78045238|ref|NP_001029674.1| actin, aortic smooth muscle [Bos taurus]
>gi|110625958|ref|NP_112266.1| actin, aortic smooth muscle [Rattus norvegicus]
>gi|156119362|ref|NP_001095152.1| actin, aortic smooth muscle [Oryctolagus cuniculus]
>gi|197097944|ref|NP_001126814.1| actin, aortic smooth muscle [Pongo abelii]
>gi|213688375|ref|NP_001135417.1| actin, aortic smooth muscle [Homo sapiens]
>gi|257470979|ref|NP_001158122.1| actin, aortic smooth muscle [Sus scrofa]
>gi|73996085|ref|XP_534781.2| PREDICTED: similar to Actin, aortic smooth muscle (Alpha-actin 2) [Canis familiaris]
>gi|114631685|ref|XP_521550.2|</td><td>Unigene114438_All//5.00E-76//AT3G12110| ACT11 (actin-11); structural constituent of cytoskeleton</td><td>Unigene114438_All//7.00E-16//TC155341</td><td></td><td>ko04145|Phagosome</td><td>GO:0000267//GO:0003015//GO:0005198//GO:0005884//GO:0006939//GO:0007015//GO:0008015//GO:0008092//GO:0012501//GO:0017111//GO:0030017//GO:0030239//GO:0032432//GO:0032559//GO:0048747//GO:0051707//GO:0060415</td><td>nr -</td></tr>
<tr><td>Unigene11444_All</td><td>933</td><td>8.08</td><td>4.45</td><td>5.59</td><td>7.80</td><td>9.49</td><td>10.65</td><td>6.80</td><td>16.93</td><td>15.63</td><td>-8.59E-01</td><td>5.68E-05</td><td></td><td>-5.30E-01</td><td>1.73E-02</td><td></td><td>2.83E-01</td><td>1.68E-01</td><td></td><td>4.50E-01</td><td>1.41E-02</td><td></td><td>1.32E+00</td><td>3.06E-22</td><td>Up</td><td>1.20E+00</td><td>2.21E-18</td><td>Up</td><td>Unigene11444_All//gi|13937171|gb|AAK50079.1|AF372939_1//9.00E-24//At1g63420/F2K11_19 [Arabidopsis thaliana]
>gi|21700873|gb|AAM70560.1| At1g63420/F2K11_19 [Arabidopsis thaliana]</td><td>Unigene11444_All//9.00E-26//AT3G48980| FUNCTIONS IN: molecular_function unknown; INVOLVED IN: biological_process unknown; LOCATED IN: cellular_component unknown; CONTAINS InterPro DOMAIN/s: Lipopolysaccharide-modifying protein (InterPro:IPR006598), Protein of unknown function DUF821, CAP10-like (InterPro:IPR008539); BEST Arabidopsis thaliana protein match is: unknown protein (TAIR:AT5G23850.1); Has 462 Blast hits to 458 proteins in 90 species: Archae - 0; Bacteria - 36; Metazoa - 203; Fungi - 74; Plants - 116; Viruses - 2; Other Eukaryotes - 31 (source: NCBI BLink).</td><td></td><td></td><td></td><td></td><td>nr +</td></tr>
<tr><td>Unigene114441_All</td><td>242</td><td>0.00</td><td>0.00</td><td>0.00</td><td>0.00</td><td>0.00</td><td>0.00</td><td>0.35</td><td>3.28</td><td>0.00</td><td>null</td><td>null</td><td></td><td>null</td><td>null</td><td></td><td>null</td><td>null</td><td></td><td>null</td><td>null</td><td></td><td>3.23E+00</td><td>5.44E-04</td><td>Up</td><td>-8.45E+00</td><td>3.60E-01</td><td></td><td>Unigene114441_All//gi|74272639|gb|ABA01115.1|//4.00E-08//mitochondrial carrier protein CR057 [Chlamydomonas incerta]</td><td>Unigene114441_All//1.00E-07//AT5G14040| mitochondrial phosphate transporter</td><td></td><td></td><td></td><td>GO:0016020//GO:0043231</td><td>nr +</td></tr>
<tr><td>Unigene11445_All</td><td>1477</td><td>3.33</td><td>1.30</td><td>4.65</td><td>5.70</td><td>6.81</td><td>9.88</td><td>5.47</td><td>10.13</td><td>9.28</td><td>-1.35E+00</td><td>1.86E-06</td><td>Down</td><td>4.82E-01</td><td>4.18E-02</td><td></td><td>2.56E-01</td><td>1.88E-01</td><td></td><td>7.93E-01</td><td>3.68E-08</td><td></td><td>8.90E-01</td><td>1.42E-11</td><td></td><td>7.63E-01</td><td>1.23E-08</td><td></td><td>Unigene11445_All//gi|48310551|gb|AAT41837.1|//1.00E-100//At5g23850 [Arabidopsis thaliana]</td><td>Unigene11445_All//7.00E-105//AT3G61280| FUNCTIONS IN: molecular_function unknown; INVOLVED IN: biological_process unknown; LOCATED IN: cellular_component unknown; CONTAINS InterPro DOMAIN/s: Lipopolysaccharide-modifying protein (InterPro:IPR006598), Protein of unknown function DUF821, CAP10-like (InterPro:IPR008539); BEST Arabidopsis thaliana protein match is: unknown protein (TAIR:AT3G61290.1); Has 467 Blast hits to 463 proteins in 98 species: Archae - 0; Bacteria - 38; Metazoa - 201; Fungi - 82; Plants - 115; Viruses - 4; Other Eukaryotes - 27 (source: NCBI BLink).</td><td>Unigene11445_All//0.00E+00//TC155101</td><td></td><td></td><td></td><td>nr +</td></tr>
<tr><td>Unigene114450_All</td><td>278</td><td>0.00</td><td>0.00</td><td>0.00</td><td>0.00</td><td>0.00</td><td>0.00</td><td>0.00</td><td>2.71</td><td>0.00</td><td>null</td><td>null</td><td></td><td>null</td><td>null</td><td></td><td>null</td><td>null</td><td></td><td>null</td><td>null</td><td></td><td>1.14E+01</td><td>2.07E-05</td><td>Up</td><td>null</td><td>null</td><td></td><td></td><td></td><td></td><td></td><td></td><td></td><td>ESTscan +</td></tr>
<tr><td>Unigene114451_All</td><td>396</td><td>0.00</td><td>0.00</td><td>0.00</td><td>0.00</td><td>0.00</td><td>0.00</td><td>0.11</td><td>1.79</td><td>0.00</td><td>null</td><td>null</td><td></td><td>null</td><td>null</td><td></td><td>null</td><td>null</td><td></td><td>null</td><td>null</td><td></td><td>4.07E+00</td><td>3.51E-04</td><td>Up</td><td>-6.74E+00</td><td>6.15E-01</td><td></td><td></td><td></td><td></td><td></td><td></td><td></td><td>ESTscan -</td></tr>
<tr><td>Unigene114454_All</td><td>362</td><td>0.00</td><td>0.00</td><td>0.00</td><td>0.00</td><td>0.00</td><td>0.00</td><td>0.12</td><td>2.08</td><td>0.00</td><td>null</td><td>null</td><td></td><td>null</td><td>null</td><td></td><td>null</td><td>null</td><td></td><td>null</td><td>null</td><td></td><td>4.15E+00</td><td>1.92E-04</td><td>Up</td><td>-6.87E+00</td><td>6.15E-01</td><td></td><td></td><td></td><td></td><td></td><td></td><td></td><td>ESTscan +</td></tr>
<tr><td>Unigene11446_All</td><td>4064</td><td>12.42</td><td>3.70</td><td>13.93</td><td>11.01</td><td>7.03</td><td>4.35</td><td>4.81</td><td>15.21</td><td>7.55</td><td>-1.75E+00</td><td>5.63E-86</td><td>Down</td><td>1.65E-01</td><td>3.28E-02</td><td></td><td>-6.46E-01</td><td>5.87E-19</td><td></td><td>-1.34E+00</td><td>3.43E-56</td><td>Down</td><td>1.66E+00</td><td>1.53E-119</td><td>Up</td><td>6.51E-01</td><td>5.05E-14</td><td></td><td>Unigene11446_All//gi|48310551|gb|AAT41837.1|//1.00E-155//At5g23850 [Arabidopsis thaliana]</td><td>Unigene11446_All//1.00E-156//AT5G23850| FUNCTIONS IN: molecular_function unknown; INVOLVED IN: biological_process unknown; LOCATED IN: cellular_component unknown; EXPRESSED IN: 23 plant structures; EXPRESSED DURING: 13 growth stages; CONTAINS InterPro DOMAIN/s: Lipopolysaccharide-modifying protein (InterPro:IPR006598), Protein of unknown function DUF821, CAP10-like (InterPro:IPR008539); BEST Arabidopsis thaliana protein match is: unknown protein (TAIR:AT3G48980.1); Has 588 Blast hits to 567 proteins in 103 species: Archae - 2; Bacteria - 34; Metazoa - 301; Fungi - 69; Plants - 115; Viruses - 3; Other Eukaryotes - 64 (source: NCBI BLink).</td><td>Unigene11446_All//0.00E+00//TC155101</td><td></td><td></td><td></td><td>nr +</td></tr>
<tr><td>Unigene11447_All</td><td>395</td><td>5.17</td><td>1.67</td><td>2.70</td><td>11.60</td><td>9.42</td><td>5.83</td><td>10.70</td><td>2.43</td><td>6.76</td><td>-1.63E+00</td><td>6.88E-04</td><td>Down</td><td>-9.38E-01</td><td>4.84E-02</td><td></td><td>-3.01E-01</td><td>3.10E-01</td><td></td><td>-9.93E-01</td><td>3.71E-04</td><td></td><td>-2.14E+00</td><td>3.68E-12</td><td>Down</td><td>-6.64E-01</td><td>8.98E-03</td><td></td><td>Unigene11447_All//gi|16930439|gb|AAL31905.1|AF419573_1//3.00E-25//AT4g27720/T29A15_210 [Arabidopsis thaliana]
>gi|15912261|gb|AAL08264.1| AT4g27720/T29A15_210 [Arabidopsis thaliana]
>gi|27764934|gb|AAO23588.1| At4g27720/T29A15_210 [Arabidopsis thaliana]</td><td>Unigene11447_All//5.00E-21//AT4G27720| LOCATED IN: plasma membrane; EXPRESSED IN: 21 plant structures; EXPRESSED DURING: 13 growth stages; CONTAINS InterPro DOMAIN/s: Protein of unknown function DUF791 (InterPro:IPR008509), Major facilitator superfamily, general substrate transporter (InterPro:IPR016196); BEST Arabidopsis thaliana protein match is: unknown protein (TAIR:AT1G64650.1); Has 496 Blast hits to 491 proteins in 183 species: Archae - 5; Bacteria - 234; Metazoa - 75; Fungi - 33; Plants - 96; Viruses - 0; Other Eukaryotes - 53 (source: NCBI BLink).</td><td>Unigene11447_All//2.00E-108//gi|48813868|gb|CO115181.1|CO115181</td><td></td><td></td><td></td><td>nr +</td></tr>
<tr><td>Unigene114477_All</td><td>285</td><td>0.00</td><td>0.00</td><td>0.00</td><td>0.00</td><td>0.48</td><td>0.16</td><td>0.74</td><td>11.88</td><td>1.40</td><td>null</td><td>null</td><td></td><td>null</td><td>null</td><td></td><td>8.92E+00</td><td>3.20E-01</td><td></td><td>7.31E+00</td><td>7.00E-01</td><td></td><td>4.00E+00</td><td>7.88E-18</td><td>Up</td><td>9.14E-01</td><td>3.86E-01</td><td></td><td></td><td></td><td></td><td></td><td></td><td></td><td></td></tr>
<tr><td>Unigene114479_All</td><td>357</td><td>0.00</td><td>0.00</td><td>0.00</td><td>0.00</td><td>0.00</td><td>0.00</td><td>0.83</td><td>3.75</td><td>0.11</td><td>null</td><td>null</td><td></td><td>null</td><td>null</td><td></td><td>null</td><td>null</td><td></td><td>null</td><td>null</td><td></td><td>2.18E+00</td><td>2.12E-04</td><td>Up</td><td>-2.89E+00</td><td>6.77E-02</td><td></td><td>Unigene114479_All//gi|29539332|dbj|BAC67667.1|//2.00E-56//elongation factor-1alpha [Cyanidioschyzon merolae]</td><td>Unigene114479_All//5.00E-54//AT5G60390| elongation factor 1-alpha / EF-1-alpha</td><td>Unigene114479_All//9.00E-36//DR462623</td><td></td><td></td><td>GO:0006412//GO:0008135//GO:0017111//GO:0032561//GO:0044424</td><td>nr +</td></tr>
</table>

Unigene11448_All	1539	6.80	3.52	3.18	22.20	18.86	17.40	20.47	7.28	9.32	-9.49E-01	1.26E-07		-1.09E+00	5.52E-09	Down	-2.35E-01	9.38E-03		-3.52E-01	9.62E-05		-1.49E+00	1.52E-52	Down	-1.14E+00	1.02E-35	Down	Unigene11448_All//gi|16930439|gb|AAL31905.1|AF419573_1//1.00E-136//AT4g27720/T29A15_210 [Arabidopsis thaliana] >gi|15912261|gb|AAL08264.1| AT4g27720/T29A15_210 [Arabidopsis thaliana] >gi|27764934|gb|AAO23588.1| At4g27720/T29A15_210 [Arabidopsis thaliana]	Unigene11448_All//6.00E-124//AT4G27720| LOCATED IN: plasma membrane; EXPRESSED IN: 21 plant structures; EXPRESSED DURING: 13 growth stages; CONTAINS InterPro DOMAIN/s: Protein of unknown function DUF791 (InterPro:IPR008509), Major facilitator superfamily, general substrate transporter (InterPro:IPR016196); BEST Arabidopsis thaliana protein match is: unknown protein (TAIR:AT1G64650.1); Has 496 Blast hits to 491 proteins in 183 species: Archae - 5; Bacteria - 234; Metazoa - 75; Fungi - 33; Plants - 96; Viruses - 0; Other Eukaryotes - 53 (source: NCBI BLink).	Unigene11448_All//0.00E+00//TC154478				nr -
Unigene114480_All	376	0.00	0.00	0.00	0.00	0.00	0.00	0.00	2.56	0.00	null	null		null	null		null	null		null	null		1.13E+01	7.61E-07	Up	null	null		Unigene114480_All//gi|193878342|gb|ACF25911.1|//5.00E-37//MLO-like protein 6 [Vitis vinifera]	Unigene114480_All//1.00E-23//AT2G39200| MLO12 (MILDEW RESISTANCE LOCUS O 12); calmodulin binding				GO:0009987//GO:0031224//GO:0050896	nr -
Unigene114488_All	211	0.74	1.44	0.00	0.24	0.65	0.64	42.88	12.08	13.97	9.54E-01	5.04E-01		-9.54E+00	2.46E-01		1.47E+00	6.19E-01		1.44E+00	6.39E-01		-1.83E+00	1.22E-20	Down	-1.62E+00	4.32E-18	Down			Unigene114488_All//2.00E-60//TC176131				
Unigene11449_All	678	6.10	4.86	4.95	22.63	21.18	23.11	10.54	4.32	6.40	-3.28E-01	2.92E-01		-3.01E-01	3.68E-01		-9.56E-02	5.88E-01		3.06E-02	8.65E-01		-1.29E+00	3.45E-10	Down	-7.19E-01	1.49E-04		Unigene11449_All//gi|16930439|gb|AAL31905.1|AF419573_1//1.00E-68//AT4g27720/T29A15_210 [Arabidopsis thaliana] >gi|15912261|gb|AAL08264.1| AT4g27720/T29A15_210 [Arabidopsis thaliana] >gi|27764934|gb|AAO23588.1| At4g27720/T29A15_210 [Arabidopsis thaliana]	Unigene11449_All//1.00E-62//AT4G27720| LOCATED IN: plasma membrane; EXPRESSED IN: 21 plant structures; EXPRESSED DURING: 13 growth stages; CONTAINS InterPro DOMAIN/s: Protein of unknown function DUF791 (InterPro:IPR008509), Major facilitator superfamily, general substrate transporter (InterPro:IPR016196); BEST Arabidopsis thaliana protein match is: unknown protein (TAIR:AT1G64650.1); Has 496 Blast hits to 491 proteins in 183 species: Archae - 5; Bacteria - 234; Metazoa - 75; Fungi - 33; Plants - 96; Viruses - 0; Other Eukaryotes - 53 (source: NCBI BLink).	Unigene11449_All//0.00E+00//gi|13352221|gb|BG442569.1|BG442569				nr -
Unigene114498_All	690	0.00	0.07	0.00	0.00	0.00	0.00	1.04	1.64	3.06	6.20E+00	6.20E-01		null	null		null	null		null	null		6.51E-01	2.66E-01		1.55E+00	1.37E-04	Up							
Unigene11450_All	1312	0.48	1.20	0.32	2.09	2.15	2.07	4.83	1.50	1.70	1.32E+00	1.50E-02		-5.59E-01	5.54E-01		4.36E-02	9.32E-01		-1.58E-02	9.59E-01		-1.69E+00	2.85E-13	Down	-1.51E+00	9.39E-12	Down	Unigene11450_All//gi|16930439|gb|AAL31905.1|AF419573_1//1.00E-147//AT4g27720/T29A15_210 [Arabidopsis thaliana] >gi|15912261|gb|AAL08264.1| AT4g27720/T29A15_210 [Arabidopsis thaliana] >gi|27764934|gb|AAO23588.1| At4g27720/T29A15_210 [Arabidopsis thaliana]	Unigene11450_All//3.00E-136//AT4G27720| LOCATED IN: plasma membrane; EXPRESSED IN: 21 plant structures; EXPRESSED DURING: 13 growth stages; CONTAINS InterPro DOMAIN/s: Protein of unknown function DUF791 (InterPro:IPR008509), Major facilitator superfamily, general substrate transporter (InterPro:IPR016196); BEST Arabidopsis thaliana protein match is: unknown protein (TAIR:AT1G64650.1); Has 496 Blast hits to 491 proteins in 183 species: Archae - 5; Bacteria - 234; Metazoa - 75; Fungi - 33; Plants - 96; Viruses - 0; Other Eukaryotes - 53 (source: NCBI BLink).	Unigene11450_All//0.00E+00//TC154478				nr -
Unigene114501_All	389	0.00	0.00	0.00	0.00	0.00	0.00	0.00	3.65	0.00	null	null		null	null		null	null		null	null		1.18E+01	4.93E-10	Up	null	null		Unigene114501_All//gi|297832702|ref|XP_002884233.1|//2.00E-08//expressed protein [Arabidopsis lyrata subsp. lyrata] >gi|297836884|ref|XP_002886324.1| expressed protein [Arabidopsis lyrata subsp. lyrata] >gi|297836886|ref|XP_002886325.1| expressed protein [Arabidopsis lyrata subsp. lyrata] >gi|297836888|ref|XP_002886326.1| expressed protein [Arabidopsis lyrata subsp. lyrata] >gi|297330073|gb|EFH60492.1| expressed protein [Arabidopsis lyrata subsp. lyrata] >gi|297332164|gb|EFH62583.1| expressed protein [Arabidopsis lyrata subsp. lyrata] >gi|297332165|gb|EFH62584.1| expressed protein [Arabidopsis lyrata subsp. lyrata] >gi|297332166|gb|EFH62585.1| expressed protein [Arabidopsis lyrata subsp. lyrata]					GO:0043231	nr -
Unigene114519_All	209	0.00	0.00	0.00	0.00	0.49	0.00	0.20	4.20	0.57	null	null		null	null		8.95E+00	4.57E-01		null	null		4.38E+00	3.08E-05	Up	1.50E+00	5.59E-01								
Unigene114520_All	366	0.00	0.00	0.00	0.00	0.00	0.00	1.39	2.85	0.00	null	null		null	null		null	null		null	null		1.04E+00	8.74E-02		-1.04E+01	5.54E-04	Down	Unigene114520_All//gi|300266999|gb|EFJ51184.1|//3.00E-24//component of cytosolic 80S ribosome and 60S large subunit [Volvox carteri f. nagariensis]	Unigene114520_All//5.00E-10//AT5G02870| 60S ribosomal protein L4/L1 (RPL4D)			ko03010|Ribosome	GO:0005622	nr +
Unigene114522_All	351	0.00	0.43	0.61	0.99	1.47	0.90	6.38	4.05	2.27	8.76E+00	2.36E-01		9.25E+00	1.42E-01		5.67E-01	6.04E-01		-1.41E-01	9.12E-01		-6.57E-01	8.80E-02		-1.49E+00	1.21E-04	Down			Unigene114522_All//3.00E-42//ES840947				
Unigene114524_All	221	0.00	0.00	0.00	0.00	0.00	0.00	2.10	8.13	0.00	null	null		null	null		null	null		null	null		1.95E+00	4.90E-05	Up	-1.10E+01	1.09E-03								ESTscan -
Unigene114527_All	341	0.00	0.00	0.00	0.00	0.00	0.00	1.98	2.94	0.00	null	null		null	null		null	null		null	null		5.68E-01	3.79E-01		-1.10E+01	3.56E-05	Down	Unigene114527_All//gi|297842159|ref|XP_002888961.1|//5.00E-13//60S ribosomal protein L6 [Arabidopsis lyrata subsp. lyrata] >gi|297334802|gb|EFH65220.1| 60S ribosomal protein L6 [Arabidopsis lyrata subsp. lyrata]	Unigene114527_All//2.00E-06//AT1G18540| 60S ribosomal protein L6 (RPL6A)			ko03010|Ribosome	GO:0005488//GO:0009536//GO:0015934//GO:0016020//GO:0031981	nr -
Unigene11454_All	1642	30.56	15.83	19.07	37.10	44.67	41.12	25.67	7.00	9.92	-9.49E-01	1.33E-33		-6.80E-01	1.63E-18		2.68E-01	2.65E-06		1.48E-01	2.91E-02		-1.87E+00	9.40E-97	Down	-1.37E+00	1.27E-63	Down	Unigene11454_All//gi|166343837|gb|ABY86661.1|//0.00E+00//beta-tubulin 15 [Gossypium hirsutum]	Unigene11454_All//0.00E+00//AT5G23860| TUB8; structural constituent of cytoskeleton	Unigene11454_All//0.00E+00//TC130257		ko04145|Phagosome	GO:0006970//GO:0007017//GO:0015630//GO:0017111//GO:0032561//GO:0043234//GO:0043623	nr +
Unigene11455_All	1710	9.49	6.22	6.66	18.47	24.31	19.07	9.10	2.54	5.06	-6.08E-01	1.04E-05		-5.09E-01	3.79E-04		3.96E-01	2.35E-07		4.61E-02	6.75E-01		-1.84E+00	2.86E-35	Down	-8.48E-01	1.70E-11		Unigene11455_All//gi|166343837|gb|ABY86661.1|//0.00E+00//beta-tubulin 15 [Gossypium hirsutum]	Unigene11455_All//0.00E+00//AT5G23860| TUB8; structural constituent of cytoskeleton	Unigene11455_All//0.00E+00//TC130257		ko04145|Phagosome	GO:0006970//GO:0007017//GO:0015630//GO:0017111//GO:0032561//GO:0043234//GO:0043623	nr -
Unigene114553_All	283	0.00	0.00	0.00	0.00	0.00	0.00	0.00	3.99	0.28	null	null		null	null		null	null		null	null		1.20E+01	5.32E-08	Up	8.14E+00	4.06E-01		Unigene114553_All//gi|55168340|gb|AAV44205.1|//4.00E-09//unknow protein [Oryza sativa Japonica Group]		Unigene114553_All//1.00E-12//DN780954				nr -
Unigene11456_All	1247	1.09	1.63	1.92	3.00	2.82	3.04	0.85	0.07	1.09	5.75E-01	2.12E-01		8.17E-01	5.74E-02		-8.90E-02	8.18E-01		2.21E-02	9.43E-01		-3.66E+00	1.31E-05	Down	3.58E-01	4.90E-01		Unigene11456_All//gi|166343837|gb|ABY86661.1|//1.00E-128//beta-tubulin 15 [Gossypium hirsutum]	Unigene11456_All//1.00E-118//AT5G23860| TUB8; structural constituent of cytoskeleton	Unigene11456_All//0.00E+00//TC178061		ko04145|Phagosome		nr -
Unigene11457_All	1726	113.19	24.81	47.82	52.53	72.44	51.26	24.77	6.01	12.33	-2.19E+00	0.00E+00	Down	-1.24E+00	1.53E-193	Down	4.64E-01	3.60E-28		-3.52E-02	6.07E-01		-2.04E+00	5.57E-110	Down	-1.01E+00	9.56E-40	Down	Unigene11457_All//gi|166343837|gb|ABY86661.1|//0.00E+00//beta-tubulin 15 [Gossypium hirsutum]	Unigene11457_All//0.00E+00//AT5G23860| TUB8; structural constituent of cytoskeleton	Unigene11457_All//0.00E+00//TC160116		ko04145|Phagosome	GO:0006970//GO:0007017//GO:0015630//GO:0017111//GO:0032561//GO:0043234//GO:0043623	nr -
Unigene114570_All	594	0.97	1.62	0.27	2.43	0.70	1.06	0.14	0.63	0.74	7.42E-01	2.94E-01		-1.85E+00	9.62E-02		-1.81E+00	9.96E-04	Down	-1.19E+00	3.43E-02		2.15E+00	9.45E-02		2.37E+00	4.05E-02		Unigene114570_All//gi|15229185|ref|NP_190534.1|//6.00E-43//kinesin motor protein-related [Arabidopsis thaliana] >gi|6561965|emb|CAB62469.1| kinesin-like protein [Arabidopsis thaliana]	Unigene114570_All//1.00E-44//AT3G49650| kinesin motor protein-related	Unigene114570_All//4.00E-39//TC168718			GO:0003774//GO:0007017//GO:0015630//GO:0032559	nr -
Unigene114571_All	656	0.00	0.00	0.00	0.00	0.05	0.00	3.67	1.15	0.12	null	null		null	null		5.71E+00	8.35E-01		null	null		-1.68E+00	1.82E-05	Down	-4.92E+00	4.21E-15	Down	Unigene114571_All//gi|297851686|ref|XP_002893724.1|//4.00E-69//proton-dependent oligopeptide transport family protein [Arabidopsis lyrata subsp. lyrata] >gi|297339566|gb|EFH69983.1| proton-dependent oligopeptide transport family protein [Arabidopsis lyrata subsp. lyrata]	Unigene114571_All//1.00E-60//AT1G32450| NRT1.5 (NITRATE TRANSPORTER 1.5); nitrate transmembrane transporter/ transporter	Unigene114571_All//6.00E-29//TC160811			GO:0004871//GO:0015833//GO:0044464	nr -
Unigene114572_All	541	0.00	0.00	0.00	0.00	0.00	0.00	0.55	2.47	1.40	null	null		null	null		null	null		null	null		2.18E+00	2.12E-04	Up	1.35E+00	6.25E-02				Unigene114572_All//1.00E-137//ES822355				
Unigene114577_All	1680	0.00	0.00	0.00	0.06	0.00	0.00	0.91	4.98	0.66	null	null		null	null		-5.89E+00	3.15E-01		-5.89E+00	4.03E-01		2.46E+00	1.96E-27	Up	-4.48E-01	3.48E-01		Unigene114577_All//gi|145333011|ref|NP_001078371.1|//1.00E-51//ankyrin repeat family protein [Arabidopsis thaliana]	Unigene114577_All//5.00E-53//AT4G10720| ankyrin repeat family protein				GO:0010033	nr +
Unigene114586_All	282	0.00	0.00	0.00	0.00	0.00	0.00	0.15	3.26	0.00	null	null		null	null		null	null		null	null		4.44E+00	1.66E-05	Up	-7.23E+00	6.15E-01								
Unigene114593_All	260	0.00	0.19	0.00	0.00	0.13	0.17	0.16	2.09	2.60	7.61E+00	6.20E-01		null	null		7.05E+00	8.35E-01		7.44E+00	7.00E-01		3.68E+00	3.69E-03		4.00E+00	4.30E-04	Up			Unigene114593_All//4.00E-31//AI055173				
Unigene114605_All	385	0.00	0.00	0.28	0.26	0.18	0.00	0.22	2.71	1.03	null	null		8.11E+00	4.01E-01		-5.33E-01	8.18E-01		-8.02E+00	4.03E-01		3.63E+00	1.71E-05	Up	2.24E+00	6.43E-02				Unigene114605_All//4.00E-100//DR462925				
Unigene114608_All	452	0.00	0.00	0.00	0.00	0.00	0.00	0.37	2.13	0.18	null	null		null	null		null	null		null	null		2.51E+00	8.00E-04	Up	-1.09E+00	5.53E-01								
Unigene114618_All	962	0.00	0.00	0.00	0.00	0.00	0.00	2.77	4.69	0.08	null	null		null	null		null	null		null	null		7.61E-01	2.68E-03		-5.06E+00	7.12E-17	Down	Unigene114618_All//gi|4139170|gb|AAD03711.1|//1.00E-151//elongation translation factor 1 alpha [Cyanophora paradoxa]	Unigene114618_All//1.00E-146//AT5G60390| elongation factor 1-alpha / EF-1-alpha	Unigene114618_All//3.00E-32//TC166357				nr +
Unigene114629_All	943	0.11	0.00	0.00	2.91	3.87	3.98	0.58	1.86	0.34	-6.79E+00	3.75E-01		-6.79E+00	3.97E-01		4.14E-01	2.26E-01		4.52E-01	1.79E-01		1.68E+00	3.64E-04	Up	-7.86E-01	3.62E-01		Unigene114629_All//gi|30725324|gb|AAP37684.1|//2.00E-73//At3g01680 [Arabidopsis thaliana]	Unigene114629_All//2.00E-69//AT3G01680| unknown protein	Unigene114629_All//2.00E-66//TC158531				nr +
Unigene114642_All	376	0.00	0.00	0.28	0.00	0.00	0.00	3.37	2.11	0.42	null	null		8.15E+00	4.01E-01		null	null		null	null		-6.76E-01	2.18E-01		-2.99E+00	6.36E-06	Down			Unigene114642_All//8.00E-21//TC149074				
Unigene114644_All	247	0.85	3.08	2.80	17.95	30.11	13.35	14.38	13.03	6.45	1.86E+00	3.73E-02		1.73E+00	7.68E-02		7.47E-01	2.41E-04		-4.27E-01	1.56E-01		-1.42E-01	6.46E-01		-1.16E+00	6.35E-05	Down			Unigene114644_All//3.00E-56//gi|13248487|gb|BF277051.2|BF277051				
Unigene114653_All	237	0.00	0.00	0.00	0.00	0.00	0.00	0.71	4.23	0.00	null	null		null	null		null	null		null	null		2.57E+00	4.79E-04	Up	-9.48E+00	1.09E-01								
Unigene114657_All	420	0.00	0.00	0.00	0.00	0.00	0.00	2.52	2.49	0.57	null	null		null	null		null	null		null	null		-1.67E-02	1.01E+00		-2.14E+00	9.36E-04	Down							
Unigene11466_All	2335	4.71	2.91	3.95	7.64	7.89	6.48	6.05	3.22	2.76	-6.94E-01	5.12E-05		-2.54E-01	1.81E-01		4.69E-02	8.06E-01		-2.37E-01	9.12E-02		-9.09E-01	2.41E-11		-1.13E+00	2.18E-16	Down	Unigene11466_All//gi|73761697|gb|AAZ83348.1|//0.00E+00//CDPK-related protein kinase [Gossypium hirsutum]	Unigene11466_All//0.00E+00//AT3G50530| CRK (CDPK-related kinase); ATP binding / calcium ion binding / calcium-dependent protein serine/threonine phosphatase/ kinase/ protein kinase/ protein serine/threonine kinase	Unigene11466_All//0.00E+00//TC129566			GO:0000904//GO:0004674//GO:0004722//GO:0006464//GO:0008092//GO:0008154//GO:0015629//GO:0016020//GO:0016049//GO:0032559//GO:0046872	nr -

Unigene114669_All	215	0.00	0.00	0.00	0.00	0.00	0.00	20.06	17.88	0.37	null	null		null	null		null	null		null	null		-1.66E-01	5.95E-01		-5.76E+00	1.43E-28	Down	Unigene114669_All//gi|238617627|gb|ACR46935.1|//1.00E-34//actin [Katablepharis japonica]	Unigene114669_All//3.00E-34//AT3G12110| ACT11 (actin-11); structural constituent of cytoskeleton	Unigene114669_All//8.00E-32//TC153744		ko04145|Phagosome	GO:0005198//GO:0015629//GO:0017111//GO:0032559	nr +
Unigene114679_All	733	0.00	0.00	0.00	0.00	0.00	0.00	1.50	0.91	0.16	null	null		null	null		null	null		null	null		-7.17E-01	2.30E-01		-3.20E+00	1.63E-05	Down	Unigene114679_All//gi|253759874|ref|XP_002488951.1|//5.00E-09//hypothetical protein SORBIDRAFT_1292s002010 [Sorghum bicolor] >gi|241947058|gb|EES20203.1| hypothetical protein SORBIDRAFT_1292s002010 [Sorghum bicolor]		Unigene114679_All//5.00E-30//TC148521				nr +
Unigene114682_All	440	0.00	0.00	0.00	0.00	0.00	0.00	0.19	1.71	0.00	null	null		null	null		null	null		null	null		3.15E+00	9.55E-04	Up	-7.59E+00	3.60E-01		Unigene114682_All//gi|145344920|ref|XP_001416972.1|//4.00E-06//NCS1 family transporter: cytosine/purines/uracil/thiamine/allantoin [Ostreococcus lucimarinus CCE9901] >gi|144577198|gb|ABO95265.1| NCS1 family transporter: cytosine/purines/uracil/thiamine/allantoin [Ostreococcus lucimarinus CCE9901]						nr -
Unigene114697_All	825	0.13	0.12	0.13	0.36	1.00	0.00	0.82	0.51	0.00	-4.62E-02	9.90E-01		2.59E-02	1.00E+00		1.47E+00	7.56E-02		-8.50E+00	4.00E-02		-6.95E-01	3.94E-01		-9.68E+00	3.56E-05	Down	Unigene114697_All//gi|18398046|ref|NP_566318.1|//1.00E-112//3’ exoribonuclease family domain 1-containing protein [Arabidopsis thaliana] >gi|30680427|ref|NP_850537.1| 3’ exoribonuclease family domain 1-containing protein [Arabidopsis thaliana] >gi|11762184|gb|AAG40370.1|AF325018_1 AT3g07750 [Arabidopsis thaliana] >gi|56744222|gb|AAW28551.1| At3g07750 [Arabidopsis thaliana]	Unigene114697_All//7.00E-107//AT3G07750| 3’ exoribonuclease family domain 1-containing protein	Unigene114697_All//5.00E-166//DT467685		ko03018|RNA degradation	GO:0003676//GO:0008408//GO:0010467	nr -
Unigene114706_All	1012	0.00	0.00	0.00	0.00	0.00	0.00	0.13	3.35	0.00	null	null		null	null		null	null		null	null		4.74E+00	8.91E-20	Up	-6.97E+00	2.00E-01		Unigene114706_All//gi|240254556|ref|NP_180668.4|//1.00E-30//triacylglycerol lipase [Arabidopsis thaliana]	Unigene114706_All//5.00E-32//AT2G31100| triacylglycerol lipase					nr -
Unigene114713_All	322	0.65	0.00	0.00	0.77	0.32	0.70	0.92	5.45	0.74	-9.34E+00	1.22E-01		-9.34E+00	1.46E-01		-1.27E+00	4.24E-01		-1.41E-01	9.29E-01		2.57E+00	1.41E-06	Up	-3.08E-01	7.75E-01				Unigene114713_All//4.00E-56//gi|109883973|gb|DW512943.1|DW512943				
Unigene11472_All	2242	11.48	2.62	3.75	25.82	15.42	28.42	25.44	8.13	8.24	-2.13E+00	1.98E-57	Down	-1.61E+00	5.02E-38	Down	-7.44E-01	3.29E-31		1.39E-01	5.14E-02		-1.65E+00	4.48E-109	Down	-1.63E+00	2.80E-110	Down	Unigene11472_All//gi|9758212|dbj|BAB08657.1|//0.00E+00//nucleolar protein-like [Arabidopsis thaliana]	Unigene11472_All//7.00E-180//AT4G26600| nucleolar protein, putative	Unigene11472_All//0.00E+00//TC151464			GO:0003676//GO:0008168//GO:0009887//GO:0031981//GO:0042254	nr -
Unigene114722_All	505	0.00	0.00	0.00	0.00	0.00	0.00	0.17	1.41	3.00	null	null		null	null		null	null		null	null		3.07E+00	1.67E-03		4.16E+00	1.21E-08	Up	Unigene114722_All//gi|38228693|emb|CAE54083.1|//1.00E-14//zinc finger protein [Fagus sylvatica]	Unigene114722_All//8.00E-16//AT2G17730| zinc finger (C3HC4-type RING finger) family protein				GO:0009743//GO:0046872	nr +
Unigene114747_All	801	0.00	0.00	0.00	0.00	0.00	0.00	0.00	6.05	0.00	null	null		null	null		null	null		null	null		1.26E+01	3.79E-34	Up	null	null		Unigene114747_All//gi|301100738|ref|XP_002899458.1|//2.00E-59//flavodoxin-like protein [Phytophthora infestans T30-4] >gi|262103766|gb|EEY61818.1| flavodoxin-like protein [Phytophthora infestans T30-4]	Unigene114747_All//2.00E-39//AT4G27270| quinone reductase family protein					nr -
Unigene114751_All	387	0.00	0.00	0.00	0.00	0.00	0.00	0.11	1.73	0.00	null	null		null	null		null	null		null	null		3.98E+00	6.38E-04	Up	-6.77E+00	6.15E-01		Unigene114751_All//gi|224000193|ref|XP_002289769.1|//5.00E-20//RL11A, ribosomal protein 11A 60S large ribosomal subunit [Thalassiosira pseudonana CCMP1335] >gi|220974977|gb|EED93306.1| RL11A, ribosomal protein 11A 60S large ribosomal subunit [Thalassiosira pseudonana CCMP1335]	Unigene114751_All//1.00E-17//AT5G45775| 60S ribosomal protein L11 (RPL11D)			ko03010|Ribosome	GO:0030529	nr +
Unigene114768_All	496	0.21	0.00	0.32	0.40	0.28	0.64	0.34	3.37	0.32	-7.72E+00	3.75E-01		6.10E-01	7.49E-01		-5.33E-01	7.49E-01		6.66E-01	6.48E-01		3.31E+00	6.98E-08	Up	-8.59E-02	9.66E-01								
Unigene11477_All	2260	12.80	8.92	8.58	32.51	28.49	38.01	64.73	26.39	28.72	-5.21E-01	2.08E-07		-5.78E-01	2.32E-08		-1.90E-01	1.26E-03		2.25E-01	4.80E-05		-1.29E+00	9.11E-195	Down	-1.17E+00	1.97E-171	Down	Unigene11477_All//gi|31711944|gb|AAP68328.1|//0.00E+00//At1g18070 [Arabidopsis thaliana]	Unigene11477_All//0.00E+00//AT1G18070| EF-1-alpha-related GTP-binding protein, putative	Unigene11477_All//0.00E+00//TC130151			GO:0004779//GO:0006412//GO:0008079//GO:0017111//GO:0032561//GO:0043231	nr +
Unigene114770_All	2705	0.00	0.00	0.00	0.00	0.00	0.00	0.30	1.16	0.21	null	null		null	null		null	null		null	null		1.96E+00	2.59E-08	Up	-5.26E-01	4.35E-01		Unigene114770_All//gi|240256087|ref|NP_194459.4|//0.00E+00//ATP binding / protein kinase/ protein serine/threonine kinase/ protein tyrosine kinase/ sugar binding [Arabidopsis thaliana]	Unigene114770_All//0.00E+00//AT4G27290| ATP binding / protein kinase/ protein serine/threonine kinase/ protein tyrosine kinase/ sugar binding	Unigene114770_All//9.00E-10//TC151245		ko04626|Plant-pathogen interaction	GO:0000166//GO:0004672//GO:0009987	nr -
Unigene114771_All	446	0.00	0.00	0.00	0.00	0.00	0.00	0.00	2.91	0.00	null	null		null	null		null	null		null	null		1.15E+01	3.67E-09	Up	null	null		Unigene114771_All//gi|150421627|sp|Q9ZW82.3|PIRL2_ARATH//2.00E-16//RecName: Full=Pirin-like protein At2g43120 >gi|119360079|gb|ABL66768.1| At2g43120 [Arabidopsis thaliana]	Unigene114771_All//1.00E-17//AT2G43120| pirin, putative				GO:0005515//GO:0016020//GO:0043231	nr +
Unigene114776_All	460	0.00	0.00	0.00	0.00	0.00	0.00	2.76	4.54	0.00	null	null		null	null		null	null		null	null		7.20E-01	7.28E-02		-1.14E+01	1.99E-09	Down	Unigene114776_All//gi|159470597|ref|XP_001693443.1|//3.00E-12//histone H1 [Chlamydomonas reinhardtii] >gi|158282946|gb|EDP08697.1| histone H1 [Chlamydomonas reinhardtii]					GO:0043229	nr +
Unigene11478_All	1350	2.44	4.43	0.55	4.98	5.25	5.42	4.64	8.24	9.27	8.59E-01	4.17E-04		-2.14E+00	1.39E-07	Down	7.70E-02	8.07E-01		1.22E-01	6.29E-01		8.29E-01	6.64E-08		9.99E-01	4.81E-12		Unigene11478_All//gi|297610410|ref|NP_001064488.2|//2.00E-95//Os10g0382300 [Oryza sativa Japonica Group] >gi|255679365|dbj|BAF26402.2| Os10g0382300 [Oryza sativa Japonica Group]	Unigene11478_All//2.00E-103//AT5G66810| FUNCTIONS IN: molecular_function unknown; INVOLVED IN: biological_process unknown; LOCATED IN: cellular_component unknown; EXPRESSED IN: 23 plant structures; EXPRESSED DURING: 15 growth stages; CONTAINS InterPro DOMAIN/s: CTLH, C-terminal to LisH motif (InterPro:IPR006595); BEST Arabidopsis thaliana protein match is: unknown protein (TAIR:AT1G61150.6); Has 214 Blast hits to 160 proteins in 56 species: Archae - 0; Bacteria - 0; Metazoa - 87; Fungi - 27; Plants - 86; Viruses - 0; Other Eukaryotes - 14 (source: NCBI BLink).	Unigene11478_All//0.00E+00//TC164435			GO:0043231	nr -
Unigene114788_All	245	0.00	0.00	0.00	0.00	0.00	0.00	1.04	7.17	0.00	null	null		null	null		null	null		null	null		2.79E+00	4.04E-07	Up	-1.00E+01	3.04E-02				Unigene114788_All//1.00E-27//ES810763				ESTscan -
Unigene114790_All	463	0.00	0.00	0.00	0.00	0.00	0.00	0.82	4.24	0.60	null	null		null	null		null	null		null	null		2.37E+00	1.05E-06	Up	-4.49E-01	6.41E-01		Unigene114790_All//gi|15222073|ref|NP_175349.1|//1.00E-46//zinc finger (C3HC4-type RING finger) family protein [Arabidopsis thaliana] >gi|68565178|sp|Q6NQG7.1|ATL1L_ARATH RecName: Full=RING-H2 finger protein ATL1L >gi|34365601|gb|AAQ65112.1| At1g49230 [Arabidopsis thaliana]	Unigene114790_All//3.00E-44//AT1G49230| zinc finger (C3HC4-type RING finger) family protein				GO:0019787//GO:0032446//GO:0046914	nr -
Unigene114796_All	340	0.00	0.00	0.00	0.00	0.00	0.00	0.00	2.46	0.00	null	null		null	null		null	null		null	null		1.13E+01	5.54E-06	Up	null	null		Unigene114796_All//gi|301108545|ref|XP_002903354.1|//2.00E-10//mucin-like protein [Phytophthora infestans T30-4] >gi|262097726|gb|EEY55778.1| mucin-like protein [Phytophthora infestans T30-4]				ko03040|Spliceosome//ko04141|Protein processing in endoplasmic reticulum//ko03022|Basal transcription factors	GO:0003824	nr -
Unigene11480_All	2294	1.05	3.51	1.74	1.74	2.51	3.19	4.48	14.98	12.00	1.74E+00	3.64E-14	Up	7.31E-01	2.21E-02		5.29E-01	3.08E-02		8.77E-01	4.00E-05		1.74E+00	2.99E-71	Up	1.42E+00	9.26E-44	Up	Unigene11480_All//gi|297610410|ref|NP_001064488.2|//0.00E+00//Os10g0382300 [Oryza sativa Japonica Group] >gi|255679365|dbj|BAF26402.2| Os10g0382300 [Oryza sativa Japonica Group]	Unigene11480_All//0.00E+00//AT5G66810| FUNCTIONS IN: molecular_function unknown; INVOLVED IN: biological_process unknown; LOCATED IN: cellular_component unknown; EXPRESSED IN: 23 plant structures; EXPRESSED DURING: 15 growth stages; CONTAINS InterPro DOMAIN/s: CTLH, C-terminal to LisH motif (InterPro:IPR006595); BEST Arabidopsis thaliana protein match is: unknown protein (TAIR:AT1G61150.6); Has 214 Blast hits to 160 proteins in 56 species: Archae - 0; Bacteria - 0; Metazoa - 87; Fungi - 27; Plants - 86; Viruses - 0; Other Eukaryotes - 14 (source: NCBI BLink).	Unigene11480_All//0.00E+00//TC164435			GO:0043231	nr +
Unigene114802_All	349	0.00	0.00	0.00	0.00	0.00	0.00	2.67	2.63	0.34	null	null		null	null		null	null		null	null		-1.66E-02	1.02E+00		-2.96E+00	1.72E-04	Down			Unigene114802_All//4.00E-10//TC142576				
Unigene11481_All	601	4.09	5.23	3.63	7.63	7.91	8.12	4.85	2.02	3.98	3.53E-01	3.35E-01		-1.72E-01	6.75E-01		5.23E-02	8.77E-01		8.99E-02	7.62E-01		-1.27E+00	1.57E-04	Down	-2.88E-01	3.88E-01				Unigene11481_All//0.00E+00//TC141993				ESTscan +
Unigene114811_All	311	0.00	0.00	0.00	0.00	0.00	0.00	5.03	4.84	0.00	null	null		null	null		null	null		null	null		-5.61E-02	9.21E-01		-1.23E+01	1.42E-11	Down	Unigene114811_All//gi|37901017|gb|AAP46148.1|//9.00E-53//actin 2 [Pseudo-nitzschia multiseries] >gi|37935564|gb|AAP46147.1| actin 1 [Pseudo-nitzschia multiseries]	Unigene114811_All//6.00E-51//AT3G12110| ACT11 (actin-11); structural constituent of cytoskeleton	Unigene114811_All//6.00E-46//TC153744		ko04145|Phagosome	GO:0000267//GO:0003015//GO:0005198//GO:0005884//GO:0006939//GO:0007015//GO:0008015//GO:0008092//GO:0012501//GO:0017111//GO:0030017//GO:0030239//GO:0032432//GO:0032559//GO:0048747//GO:0051707//GO:0060415	nr +
Unigene114813_All	324	0.00	0.00	0.00	0.00	0.00	0.00	1.17	9.55	1.60	null	null		null	null		null	null		null	null		3.02E+00	5.11E-13	Up	4.45E-01	6.12E-01				Unigene114813_All//4.00E-07//DW479423				
Unigene114821_All	242	0.22	0.00	0.88	0.41	0.28	0.93	0.35	5.70	0.49	-7.76E+00	6.17E-01		2.03E+00	3.62E-01		-5.33E-01	8.18E-01		1.18E+00	5.50E-01		4.03E+00	1.38E-07	Up	4.99E-01	8.08E-01				Unigene114821_All//4.00E-111//DW514353				
Unigene114825_All	260	0.80	0.97	0.82	1.92	1.72	1.04	8.13	5.30	3.06	2.76E-01	8.44E-01		2.54E-02	1.00E+00		-1.54E-01	8.50E-01		-8.78E-01	4.20E-01		-6.16E-01	1.26E-01		-1.41E+00	3.88E-04	Down	Unigene114825_All//gi|154146804|emb|CAM84089.1|//3.00E-13//phosphoenolpyruvate carboxylase [Brachypodium pinnatum]	Unigene114825_All//1.00E-13//AT2G42600| ATPPC2 (PHOSPHOENOLPYRUVATE CARBOXYLASE 2); catalytic/ phosphoenolpyruvate carboxylase	Unigene114825_All//2.00E-54//ES821747		ko00620|Pyruvate metabolism//ko00710|Carbon fixation in photosynthetic organisms	GO:0004611//GO:0009060//GO:0044424//GO:0071704	nr -

Unigene114826_All	247	25.84	25.44	21.34	9.08	11.85	12.80	11.64	33.50	35.16	-2.28E-02	9.41E-01		-2.76E-01	2.84E-01		3.85E-01	3.24E-01		4.96E-01	1.81E-01		1.53E+00	8.39E-15	Up	1.59E+00	7.03E-17	Up	Unigene114826_All//gi|15223919|ref|NP_172939.1|//5.00E-14//atnudt18 (Arabidopsis thaliana Nudix hydrolase homolog 18); hydrolase >gi|68565943|sp|Q9LQU5.1|NUD18_ARATH RecName: Full=Nudix hydrolase 18, mitochondrial; Short=AtNUDT18; Flags: Precursor >gi|8778216|gb|AAF79225.1|AC006917_10 F10B6.26 [Arabidopsis thaliana]	Unigene114826_All//2.00E-15//AT1G14860| atnudt18 (Arabidopsis thaliana Nudix hydrolase homolog 18); hydrolase	Unigene114826_All//6.00E-73//gi|109889180|gb|DW518148.1|DW518148			GO:0016462	nr +
Unigene114828_All	244	0.43	0.00	0.00	0.82	0.42	0.00	0.17	0.51	3.10	-8.74E+00	3.75E-01		-8.74E+00	3.97E-01		-9.48E-01	5.88E-01		-9.67E+00	1.34E-01		1.57E+00	5.46E-01		4.16E+00	1.34E-04	Up							
Unigene114832_All	282	19.85	10.96	12.27	10.42	8.79	13.61	8.25	3.85	2.97	-8.57E-01	6.69E-04		-6.94E-01	8.13E-03		-2.45E-01	5.56E-01		3.85E-01	2.52E-01		-1.10E+00	3.58E-03		-1.47E+00	1.01E-04	Down	Unigene114832_All//gi|21780187|gb|AAM77651.1|AF522274_1//4.00E-17//cp10-like protein [Gossypium hirsutum]	Unigene114832_All//2.00E-07//AT5G20720| CPN20 (CHAPERONIN 20); calmodulin binding	Unigene114832_All//5.00E-74//gi|21092565|gb|BQ404878.1|BQ404878			GO:0005576//GO:0006950//GO:0009526//GO:0009532//GO:0009534//GO:0010038//GO:0032559//GO:0044267//GO:0046914	nr +
Unigene114839_All	255	23.80	6.16	7.10	120.53	69.82	37.02	28.52	13.11	19.53	-1.95E+00	1.35E-12	Down	-1.75E+00	2.07E-10	Down	-7.88E-01	1.71E-18		-1.70E+00	4.58E-55	Down	-1.12E+00	1.79E-08	Down	-5.46E-01	3.54E-03				Unigene114839_All//2.00E-70//TC167712				
Unigene11484_All	743	5.42	3.07	9.10	3.22	4.68	5.05	9.84	13.73	4.61	-8.21E-01	6.84E-03		7.47E-01	1.43E-03		5.41E-01	1.08E-01		6.49E-01	4.33E-02		4.80E-01	2.79E-03		-1.09E+00	1.64E-08	Down	Unigene11484_All//gi|15222119|ref|NP_172755.1|//1.00E-71//MATE efflux family protein [Arabidopsis thaliana] >gi|66792652|gb|AAY56428.1| At1g12950 [Arabidopsis thaliana]	Unigene11484_All//5.00E-73//AT1G12950| MATE efflux family protein	Unigene11484_All//0.00E+00//TC140107			GO:0015291//GO:0015893//GO:0016020//GO:0043231//GO:0051707	nr +
Unigene114840_All	588	0.00	0.00	0.00	0.00	0.00	0.00	1.37	2.20	0.00	null	null		null	null		null	null		null	null		6.90E-01	2.02E-01		-1.04E+01	4.43E-06	Down							ESTscan +
Unigene11485_All	1420	7.37	4.53	7.65	8.45	5.77	6.81	20.54	33.82	6.90	-7.01E-01	6.89E-05		5.40E-02	7.76E-01		-5.51E-01	2.87E-04		-3.13E-01	6.81E-02		7.19E-01	9.71E-25		-1.57E+00	3.09E-54	Down	Unigene11485_All//gi|297818202|ref|XP_002876984.1|//1.00E-165//mate efflux family protein [Arabidopsis lyrata subsp. lyrata] >gi|297322822|gb|EFH53243.1| mate efflux family protein [Arabidopsis lyrata subsp. lyrata]	Unigene11485_All//8.00E-162//AT3G26590| MATE efflux family protein	Unigene11485_All//0.00E+00//TC140107			GO:0015291//GO:0015893//GO:0044464	nr +
Unigene114856_All	455	0.00	0.00	0.00	0.00	0.00	0.00	4.46	5.05	0.09	null	null		null	null		null	null		null	null		1.80E-01	6.43E-01		-5.67E+00	1.44E-13	Down	Unigene114856_All//gi|6683481|dbj|BAA89231.1|//3.00E-46//wrp15a [Citrullus lanatus]	Unigene114856_All//8.00E-47//AT5G59850| 40S ribosomal protein S15A (RPS15aF)			ko03010|Ribosome	GO:0005198//GO:0010467//GO:0015935//GO:0016020//GO:0030312//GO:0043231	nr +
Unigene11486_All	1112	6.73	4.37	6.85	9.00	6.19	6.50	16.08	24.99	6.41	-6.21E-01	3.52E-03		2.54E-02	9.23E-01		-5.40E-01	1.44E-03		-4.71E-01	9.21E-03		6.36E-01	5.60E-12		-1.33E+00	3.54E-26	Down	Unigene11486_All//gi|297818202|ref|XP_002876984.1|//2.00E-71//mate efflux family protein [Arabidopsis lyrata subsp. lyrata] >gi|297322822|gb|EFH53243.1| mate efflux family protein [Arabidopsis lyrata subsp. lyrata]	Unigene11486_All//6.00E-67//AT1G12950| MATE efflux family protein	Unigene11486_All//3.00E-78//gi|48778270|gb|CO087636.1|CO087636			GO:0015291//GO:0015893//GO:0016020//GO:0043231//GO:0051707	nr -
Unigene114861_All	374	0.00	0.00	0.00	0.13	0.00	0.48	0.00	2.46	0.21	null	null		null	null		-7.06E+00	5.60E-01		1.86E+00	4.59E-01		1.13E+01	1.48E-06	Up	7.73E+00	4.06E-01		Unigene114861_All//gi|296149177|gb|ADG96403.1|//4.00E-26//S-locus glycoprotein [Olea europaea]	Unigene114861_All//2.00E-20//AT4G27290| ATP binding / protein kinase/ protein serine/threonine kinase/ protein tyrosine kinase/ sugar binding				GO:0004674//GO:0004871//GO:0006464//GO:0008037//GO:0030246//GO:0032559	nr +
Unigene114864_All	254	0.00	0.00	0.00	0.00	0.00	0.00	2.00	2.14	0.00	null	null		null	null		null	null		null	null		9.89E-02	9.21E-01		-1.10E+01	5.54E-04	Down	Unigene114864_All//gi|300256410|gb|EFJ40676.1|//2.00E-31//component of cytosolic 80S ribosome and 40S small subunit [Volvox carteri f. nagariensis]	Unigene114864_All//2.00E-23//AT5G39850| 40S ribosomal protein S9 (RPS9C)			ko03010|Ribosome	GO:0003723//GO:0005198//GO:0009536//GO:0010467//GO:0015935	nr -
Unigene11488_All	1980	7.45	3.38	4.09	10.49	6.94	10.70	10.85	4.96	7.87	-1.14E+00	7.86E-14	Down	-8.66E-01	1.26E-08		-5.96E-01	6.40E-08		2.81E-02	8.57E-01		-1.13E+00	1.05E-23	Down	-4.64E-01	6.75E-06		Unigene11488_All//gi|13623892|dbj|BAB41076.1|//0.00E+00//MAR-binding protein [Nicotiana tabacum]	Unigene11488_All//0.00E+00//AT3G05060| SAR DNA-binding protein, putative	Unigene11488_All//0.00E+00//gi|164274934|gb|ES818124.1|ES818124		ko03040|Spliceosome	GO:0003676//GO:0031981	nr -
Unigene114880_All	425	0.25	0.24	1.50	0.35	0.32	0.00	0.30	3.05	0.47	-4.64E-02	9.90E-01		2.61E+00	2.33E-02		-1.18E-01	9.11E-01		-8.46E+00	2.34E-01		3.35E+00	2.62E-06	Up	6.51E-01	6.66E-01								
Unigene114887_All	346	0.00	0.00	0.00	0.43	0.00	0.13	0.24	2.30	0.46	null	null		null	null		-8.75E+00	1.66E-01		-1.73E+00	5.02E-01		3.23E+00	5.44E-04	Up	9.14E-01	6.25E-01								
Unigene114888_All	294	0.18	0.52	0.00	0.34	0.23	0.61	0.00	0.85	2.17	1.54E+00	5.32E-01		-7.48E+00	6.29E-01		-5.33E-01	8.18E-01		8.59E-01	6.96E-01		9.74E+00	4.32E-02		1.11E+01	9.35E-05	Up			Unigene114888_All//2.00E-27//gi|84152253|gb|DN801608.1|DN801608				
Unigene11489_All	2084	16.07	7.83	9.22	27.70	21.89	33.68	56.56	31.61	31.62	-1.04E+00	3.74E-26	Down	-8.01E-01	1.76E-16		-3.40E-01	9.05E-08		2.82E-01	3.91E-06		-8.40E-01	1.13E-77		-8.39E-01	9.75E-80		Unigene11489_All//gi|13623892|dbj|BAB41076.1|//0.00E+00//MAR-binding protein [Nicotiana tabacum]	Unigene11489_All//0.00E+00//AT3G05060| SAR DNA-binding protein, putative	Unigene11489_All//0.00E+00//gi|164243931|gb|ES808355.1|ES808355		ko03040|Spliceosome	GO:0003676//GO:0031981	nr -
Unigene114890_All	427	0.00	0.00	0.00	0.00	0.00	0.00	2.97	2.06	0.19	null	null		null	null		null	null		null	null		-5.31E-01	3.43E-01		-3.99E+00	2.56E-07	Down	Unigene114890_All//gi|9188543|dbj|BAA99561.1|//2.00E-31//alpha-tubulin [Chlorella vulgaris]	Unigene114890_All//3.00E-23//AT5G19780| TUA5; structural constituent of cytoskeleton	Unigene114890_All//1.00E-26//gi|164280667|gb|ES792179.1|ES792179		ko04145|Phagosome	GO:0007017//GO:0015630//GO:0017111//GO:0032561//GO:0032991//GO:0043623	nr -
Unigene114891_All	471	0.00	0.00	0.00	0.00	0.00	0.00	0.00	2.04	0.00	null	null		null	null		null	null		null	null		1.10E+01	7.61E-07	Up	null	null		Unigene114891_All//gi|13516922|dbj|BAB40340.1|//1.00E-17//12-oxophytodienoic acid 10, 11-reductase [Pisum sativum] >gi|44917012|dbj|BAD12185.1| 12-oxophytodienoic acid 10,11-reductase [Pisum sativum]	Unigene114891_All//2.00E-17//AT1G17990| 12-oxophytodienoate reductase, putative			ko00592|alpha-Linolenic acid metabolism	GO:0003824	nr +
Unigene114892_All	357	0.00	0.43	0.15	0.28	0.48	0.13	0.12	1.99	0.33	8.73E+00	2.36E-01		7.22E+00	6.39E-01		7.89E-01	7.71E-01		-1.14E+00	6.78E-01		4.07E+00	3.51E-04	Up	1.50E+00	5.59E-01		Unigene114892_All//gi|15235767|ref|NP_193381.1|//7.00E-34//INT4 (INOSITOL TRANSPORTER 4); carbohydrate transmembrane transporter/ myo-inositol:hydrogen symporter/ sugar:hydrogen symporter [Arabidopsis thaliana] >gi|75318122|sp|O23492.1|INT4_ARATH RecName: Full=Inositol transporter 4; AltName: Full=Myo-inositol-proton symporter INT4; AltName: Full=Protein INOSITOL TRANSPORTER 4 >gi|2245004|emb|CAB10424.1| membrane transporter like protein [Arabidopsis thaliana] >gi|7268398|emb|CAB78690.1| membrane transporter like protein [Arabidopsis thaliana] >gi|84617973|emb|CAJ00306.1| inositol transporter 4 [Arabidopsis thaliana]	Unigene114892_All//3.00E-35//AT4G16480| INT4 (INOSITOL TRANSPORTER 4); carbohydrate transmembrane transporter/ myo-inositol:hydrogen symporter/ sugar:hydrogen symporter				GO:0005365//GO:0008152//GO:0015791//GO:0016628//GO:0031224	nr +
Unigene1149_All	1362	3.65	1.90	2.31	6.91	5.64	4.01	5.03	0.37	2.25	-9.44E-01	4.94E-04		-6.62E-01	1.87E-02		-2.94E-01	1.28E-01		-7.85E-01	1.78E-05		-3.77E+00	3.54E-34	Down	-1.16E+00	1.02E-08	Down	Unigene1149_All//gi|15228248|ref|NP_187640.1|//1.00E-149//phosphate translocator-related [Arabidopsis thaliana]	Unigene1149_All//7.00E-140//AT3G10290| phosphate translocator-related	Unigene1149_All//0.00E+00//gi|48820927|gb|CO122240.1|CO122240				nr -
Unigene11490_All	407	1.80	6.72	13.87	2.94	5.67	3.99	1.66	1.95	2.06	1.90E+00	7.24E-06	Up	2.95E+00	6.60E-18	Up	9.49E-01	2.28E-02		4.44E-01	4.29E-01		2.31E-01	7.35E-01		3.07E-01	6.28E-01		Unigene11490_All//gi|297843644|ref|XP_002889703.1|//2.00E-09//thioredoxin family protein [Arabidopsis lyrata subsp. lyrata] >gi|297335545|gb|EFH65962.1| thioredoxin family protein [Arabidopsis lyrata subsp. lyrata]	Unigene11490_All//3.00E-10//AT1G08570| ACHT4 (ATYPICAL CYS HIS RICH THIOREDOXIN 4); oxidoreductase, acting on sulfur group of donors, disulfide as acceptor	Unigene11490_All//3.00E-54//TC132912				nr -
Unigene114901_All	333	0.00	0.00	0.00	0.00	0.00	0.00	0.13	2.51	0.00	null	null		null	null		null	null		null	null		4.31E+00	5.68E-05	Up	-6.99E+00	6.15E-01		Unigene114901_All//gi|168070939|ref|XP_001786992.1|//3.00E-08//predicted protein [Physcomitrella patens subsp. patens] >gi|162659991|gb|EDQ48194.1| predicted protein [Physcomitrella patens subsp. patens]						nr -
Unigene114909_All	550	0.00	0.00	0.00	0.00	0.00	0.00	1.77	2.28	0.00	null	null		null	null		null	null		null	null		3.67E-01	5.29E-01		-1.08E+01	2.72E-07	Down							ESTscan -
Unigene11491_All	4312	21.19	49.76	32.34	38.87	42.82	35.88	22.99	12.24	19.37	1.23E+00	3.41E-215	Up	6.10E-01	8.31E-42		1.40E-01	1.09E-04		-1.16E-01	4.87E-03		-9.09E-01	7.70E-75		-2.47E-01	5.82E-08		Unigene11491_All//gi|18402209|ref|NP_564528.1|//0.00E+00//RKL1; ATP binding / kinase/ protein serine/threonine kinase [Arabidopsis thaliana] >gi|75335279|sp|Q9LP77.1|Y1848_ARATH RecName: Full=Probable inactive receptor kinase At1g48480; Flags: Precursor >gi|8778688|gb|AAF79696.1|AC020889_4 T1N15.9 [Arabidopsis thaliana] >gi|209529789|gb|ACI49789.1| At1g48480 [Arabidopsis thaliana] >gi|224589420|gb|ACN59244.1| leucine-rich repeat receptor-like protein kinase [Arabidopsis thaliana]	Unigene11491_All//0.00E+00//AT1G48480| RKL1; ATP binding / kinase/ protein serine/threonine kinase	Unigene11491_All//0.00E+00//TC145693			GO:0004672//GO:0006464//GO:0007167//GO:0016491//GO:0031224//GO:0031410//GO:0032559	nr +
Unigene114910_All	217	0.00	0.00	0.00	0.00	0.00	0.00	0.00	12.33	0.92	null	null		null	null		null	null		null	null		1.36E+01	8.14E-19	Up	9.84E+00	7.90E-02				Unigene114910_All//1.00E-12//DT049550				
Unigene114914_All	239	0.00	0.00	0.00	0.00	0.00	0.00	1.24	7.52	0.50	null	null		null	null		null	null		null	null		2.60E+00	8.33E-07	Up	-1.31E+00	3.13E-01				Unigene114914_All//2.00E-51//gi|109883973|gb|DW512943.1|DW512943				
Unigene114919_All	216	134.92	113.54	56.21	247.23	118.29	201.98	484.26	351.59	112.14	-2.49E-01	1.59E-02		-1.26E+00	6.55E-30	Down	-1.06E+00	7.08E-53	Down	-2.92E-01	3.89E-05		-4.62E-01	2.38E-24		-2.11E+00	4.66E-288	Down			Unigene114919_All//9.00E-38//TC175475				
Unigene114921_All	318	0.00	0.00	0.00	0.00	0.00	0.00	0.00	1.84	0.00	null	null		null	null		null	null		null	null		1.08E+01	2.81E-04	Up	null	null								
Unigene114922_All	750	0.00	0.00	0.00	0.00	0.00	0.00	0.00	3.51	0.00	null	null		null	null		null	null		null	null		1.18E+01	1.60E-18	Up	null	null		Unigene114922_All//gi|298710310|emb|CBJ31931.1|//5.00E-12//putative transmembrane protein [Ectocarpus siliculosus]						nr +
Unigene114930_All	220	0.24	3.92	0.73	0.23	0.47	1.23	0.58	3.42	1.63	4.04E+00	4.20E-04	Up	1.61E+00	5.11E-01		1.05E+00	8.09E-01		2.44E+00	2.22E-01		2.57E+00	3.32E-03		1.50E+00	2.04E-01		Unigene114930_All//gi|18542927|gb|AAK00417.2|//6.00E-14//Putative inositol 1,3,4-trisphosphate 5/6-kinase [Oryza sativa Japonica Group]	Unigene114930_All//5.00E-14//AT4G08170| inositol 1,3,4-trisphosphate 5/6-kinase family protein	Unigene114930_All//2.00E-60//TC178237		ko04070|Phosphatidylinositol signaling system//ko00562|Inositol phosphate metabolism	GO:0006950//GO:0032559//GO:0043231//GO:0043647//GO:0046872//GO:0051765//GO:0051766	nr +
Unigene114937_All	257	3.66	7.10	5.59	2.33	3.89	4.92	2.30	6.83	2.64	9.54E-01	4.89E-02		6.10E-01	2.96E-01		7.40E-01	3.10E-01		1.08E+00	8.18E-02		1.57E+00	7.26E-04	Up	1.94E-01	7.87E-01			Unigene114937_All//5.00E-07//AT1G06210| VHS domain-containing protein / GAT domain-containing protein	Unigene114937_All//1.00E-65//ES843618				
Unigene114946_All	1172	0.09	0.09	0.00	0.09	0.00	0.00	1.08	3.71	2.82	-4.60E-02	9.90E-01		-6.48E+00	3.97E-01		-6.41E+00	3.15E-01		-6.41E+00	4.03E-01		1.78E+00	6.35E-10	Up	1.38E+00	8.62E-06	Up	Unigene114946_All//gi|238478527|ref|NP_001154349.1|//3.00E-22//ATP binding / kinase/ protein kinase/ protein serine/threonine kinase/ protein tyrosine kinase [Arabidopsis thaliana] >gi|259016409|sp|P0C5E2.2|Y1839_ARATH RecName: Full=Probable serine/threonine-protein kinase At1g18390; Flags: Precursor	Unigene114946_All//1.00E-23//AT1G18390| ATP binding / kinase/ protein kinase/ protein serine/threonine kinase/ protein tyrosine kinase				GO:0004672//GO:0016020	nr -
Unigene114949_All	1050	0.00	0.00	0.00	0.00	0.00	0.04	0.40	2.07	0.49	null	null		null	null		null	null		5.43E+00	7.00E-01		2.36E+00	2.52E-07	Up	2.93E-01	7.27E-01		Unigene114949_All//gi|224124808|ref|XP_002319427.1|//1.00E-110//cytochrome P450 [Populus trichocarpa] >gi|222857803|gb|EEE95350.1| cytochrome P450 [Populus trichocarpa]	Unigene114949_All//4.00E-80//AT4G39950| CYP79B2; electron carrier/ heme binding / iron ion binding / monooxygenase/ oxygen binding	Unigene114949_All//5.00E-09//TC176629		ko00460|Cyanoamino acid metabolism	GO:0002239//GO:0004497//GO:0005506//GO:0006091//GO:0006568//GO:0009536//GO:0009683//GO:0009700//GO:0044425//GO:0052482	nr -
Unigene114953_All	328	0.00	0.00	0.00	0.00	0.00	0.00	0.00	2.93	0.00	null	null		null	null		null	null		null	null		1.15E+01	7.61E-07	Up	null	null								
Unigene114958_All	400	0.00	0.00	0.00	0.00	0.00	0.00	0.00	1.78	0.00	null	null		null	null		null	null		null	null		1.08E+01	3.98E-05	Up	null	null								

<table>
<tr><td>Unigene11496_All</td><td>2646</td><td>3.74</td><td>1.24</td><td>3.02</td><td>12.93</td><td>10.58</td><td>9.81</td><td>4.15</td><td>1.33</td><td>0.72</td><td>-1.59E+00</td><td>2.99E-15</td><td>Down</td><td>-3.08E-01</td><td>1.23E-01</td><td></td><td>-2.90E-01</td><td>9.28E-04</td><td></td><td>-3.98E-01</td><td>8.31E-06</td><td></td><td>-1.65E+00</td><td>1.22E-21</td><td>Down</td><td>-2.52E+00</td><td>2.75E-38</td><td>Down</td><td>Unigene11496_All//gi|87240917|gb|ABD32775.1|//0.00E+00//Protein kinase [Medicago truncatula]</td><td>Unigene11496_All//3.00E-128//AT2G01820| leucine-rich repeat protein kinase, putative</td><td>Unigene11496_All//0.00E+00//TC159514</td><td></td><td></td><td>GO:0004672//GO:0006464//GO:0031224//GO:0032559</td><td>nr -</td></tr>
<tr><td>Unigene114962_All</td><td>746</td><td>0.00</td><td>0.00</td><td>0.00</td><td>0.00</td><td>0.00</td><td>0.00</td><td>3.06</td><td>15.74</td><td>0.21</td><td>null</td><td>null</td><td></td><td>null</td><td>null</td><td></td><td>null</td><td>null</td><td></td><td>null</td><td>null</td><td></td><td>2.36E+00</td><td>1.41E-36</td><td>Up</td><td>-3.84E+00</td><td>2.31E-12</td><td>Down</td><td>Unigene114962_All//gi|238014838|gb|ACR38454.1|//3.00E-14//unknown [Zea mays]</td><td></td><td>Unigene114962_All//4.00E-49//TC146753</td><td></td><td></td><td></td><td>nr -</td></tr>
<tr><td>Unigene11497_All</td><td>3060</td><td>4.07</td><td>2.37</td><td>5.26</td><td>7.98</td><td>8.00</td><td>8.06</td><td>4.15</td><td>1.54</td><td>1.34</td><td>-7.81E-01</td><td>1.20E-06</td><td></td><td>3.69E-01</td><td>1.20E-02</td><td></td><td>4.40E-03</td><td>9.76E-01</td><td></td><td>1.47E-02</td><td>9.31E-01</td><td></td><td>-1.43E+00</td><td>2.96E-20</td><td>Down</td><td>-1.63E+00</td><td>3.05E-25</td><td>Down</td><td>Unigene11497_All//gi|87240917|gb|ABD32775.1|//0.00E+00//Protein kinase [Medicago truncatula]</td><td>Unigene11497_All//0.00E+00//AT1G66150| TMK1 (TRANSMEMBRANE KINASE 1); transmembrane receptor protein serine/threonine kinase</td><td>Unigene11497_All//0.00E+00//gi|164239875|gb|ES827735.1|ES827735</td><td></td><td></td><td>GO:0004674//GO:0004871//GO:0006464//GO:0016020//GO:0032559</td><td>nr +</td></tr>
<tr><td>Unigene114978_All</td><td>451</td><td>0.23</td><td>0.00</td><td>0.00</td><td>0.11</td><td>0.15</td><td>0.00</td><td>0.38</td><td>2.78</td><td>0.44</td><td>-7.86E+00</td><td>3.75E-01</td><td></td><td>-7.86E+00</td><td>3.97E-01</td><td></td><td>4.67E-01</td><td>9.60E-01</td><td></td><td>-6.79E+00</td><td>6.41E-01</td><td></td><td>2.89E+00</td><td>1.97E-05</td><td>Up</td><td>2.36E-01</td><td>8.84E-01</td><td></td><td></td><td></td><td>Unigene114978_All//4.00E-128//ES801465</td><td></td><td></td><td></td><td></td></tr>
<tr><td>Unigene11498_All</td><td>1225</td><td>1.75</td><td>4.22</td><td>4.22</td><td>5.08</td><td>3.68</td><td>3.91</td><td>7.42</td><td>8.09</td><td>5.66</td><td>1.27E+00</td><td>3.74E-06</td><td>Up</td><td>1.27E+00</td><td>7.23E-06</td><td>Up</td><td>-4.65E-01</td><td>4.35E-02</td><td></td><td>-3.79E-01</td><td>1.28E-01</td><td></td><td>1.24E-01</td><td>5.28E-01</td><td></td><td>-3.91E-01</td><td>1.95E-02</td><td></td><td></td><td></td><td>Unigene11498_All//0.00E+00//TC179542</td><td></td><td></td><td></td><td></td></tr>
<tr><td>Unigene114989_All</td><td>238</td><td>0.22</td><td>0.00</td><td>0.00</td><td>2.09</td><td>0.00</td><td>0.57</td><td>0.18</td><td>1.93</td><td>0.00</td><td>-7.78E+00</td><td>6.17E-01</td><td></td><td>-7.78E+00</td><td>6.29E-01</td><td></td><td>-1.10E+01</td><td>8.99E-04</td><td>Down</td><td>-1.88E+00</td><td>1.08E-01</td><td></td><td>3.44E+00</td><td>1.16E-02</td><td></td><td>-7.47E+00</td><td>6.15E-01</td><td></td><td></td><td></td><td>Unigene114989_All//4.00E-46//CO132540</td><td></td><td></td><td></td><td></td></tr>
<tr><td>Unigene11499_All</td><td>1231</td><td>2.08</td><td>4.65</td><td>3.81</td><td>4.17</td><td>6.10</td><td>4.37</td><td>6.49</td><td>6.79</td><td>10.62</td><td>1.16E+00</td><td>5.95E-06</td><td>Up</td><td>8.70E-01</td><td>2.74E-03</td><td></td><td>5.49E-01</td><td>7.87E-03</td><td></td><td>6.69E-02</td><td>8.20E-01</td><td></td><td>6.50E-02</td><td>7.49E-01</td><td></td><td>7.10E-01</td><td>2.12E-07</td><td></td><td>Unigene11499_All//gi|297812189|ref|XP_002873978.1|//3.00E-46//agenet domain-containing protein [Arabidopsis lyrata subsp. lyrata] >gi|297319815|gb|EFH50237.1| agenet domain-containing protein [Arabidopsis lyrata subsp. lyrata]</td><td>Unigene11499_All//4.00E-33//AT5G20030| agenet domain-containing protein</td><td>Unigene11499_All//5.00E-173//TC156483</td><td></td><td></td><td></td><td>nr +</td></tr>
<tr><td>Unigene1150_All</td><td>1368</td><td>3.48</td><td>1.07</td><td>1.67</td><td>4.73</td><td>4.10</td><td>2.71</td><td>5.72</td><td>1.34</td><td>2.80</td><td>-1.70E+00</td><td>1.99E-08</td><td>Down</td><td>-1.06E+00</td><td>2.88E-04</td><td>Down</td><td>-2.06E-01</td><td>4.24E-01</td><td></td><td>-8.06E-01</td><td>3.83E-04</td><td></td><td>-2.09E+00</td><td>2.84E-21</td><td>Down</td><td>-1.03E+00</td><td>2.65E-08</td><td>Down</td><td>Unigene1150_All//gi|15228248|ref|NP_187640.1|//1.00E-151//phosphate translocator-related [Arabidopsis thaliana]</td><td>Unigene1150_All//3.00E-131//AT5G04160| phosphate translocator-related</td><td>Unigene1150_All//0.00E+00//TC139348</td><td></td><td></td><td></td><td>nr +</td></tr>
<tr><td>Unigene115001_All</td><td>331</td><td>0.00</td><td>0.00</td><td>0.00</td><td>0.00</td><td>0.00</td><td>0.00</td><td>0.26</td><td>3.16</td><td>0.00</td><td>null</td><td>null</td><td></td><td>null</td><td>null</td><td></td><td>null</td><td>null</td><td></td><td>null</td><td>null</td><td></td><td>3.63E+00</td><td>1.71E-05</td><td>Up</td><td>-8.00E+00</td><td>3.60E-01</td><td></td><td>Unigene115001_All//gi|255070295|ref|XP_002507229.1|//2.00E-08//H+-or Na+-translocating f-type, v-type and A-type ATPase superfamily [Micromonas sp. RCC299] >gi|226522504|gb|ACO68487.1| H+-or Na+-translocating f-type, v-type and A-type ATPase superfamily [Micromonas sp. RCC299]</td><td>Unigene115001_All//3.00E-06//ATCG00480| chloroplast-encoded gene for beta subunit of ATP synthase</td><td></td><td></td><td>ko00190|Oxidative phosphorylation//ko00195|Photosynthesis</td><td>GO:0009534//GO:0015078//GO:0015662//GO:0015986//GO:0019829//GO:0032559//GO:0033178</td><td>nr -</td></tr>
<tr><td>Unigene115007_All</td><td>332</td><td>0.00</td><td>0.00</td><td>0.00</td><td>0.00</td><td>0.00</td><td>0.00</td><td>2.29</td><td>2.52</td><td>0.00</td><td>null</td><td>null</td><td></td><td>null</td><td>null</td><td></td><td>null</td><td>null</td><td></td><td>null</td><td>null</td><td></td><td>1.35E-01</td><td>8.42E-01</td><td></td><td>-1.12E+01</td><td>8.87E-06</td><td>Down</td><td></td><td></td><td></td><td></td><td></td><td></td><td></td></tr>
<tr><td>Unigene11502_All</td><td>1271</td><td>1.56</td><td>0.96</td><td>1.89</td><td>3.02</td><td>3.20</td><td>2.10</td><td>4.36</td><td>2.37</td><td>2.04</td><td>-7.09E-01</td><td>1.20E-01</td><td></td><td>2.69E-01</td><td>5.47E-01</td><td></td><td>8.32E-02</td><td>8.15E-01</td><td></td><td>-5.26E-01</td><td>1.02E-01</td><td></td><td>-8.80E-01</td><td>9.99E-05</td><td></td><td>-1.10E+00</td><td>1.11E-06</td><td>Down</td><td>Unigene11502_All//gi|63087714|emb|CAI93172.1|//1.00E-122//beta-1,3-glucuronosyltransferase [Gossypium raimondii]</td><td>Unigene11502_All//1.00E-102//AT1G27600| glycosyl transferase family 43 protein</td><td>Unigene11502_All//0.00E+00//TC129656</td><td></td><td></td><td>GO:0015020//GO:0044464</td><td>nr +</td></tr>
<tr><td>Unigene115024_All</td><td>241</td><td>0.00</td><td>0.00</td><td>0.00</td><td>0.00</td><td>0.00</td><td>0.00</td><td>0.00</td><td>2.60</td><td>0.00</td><td>null</td><td>null</td><td></td><td>null</td><td>null</td><td></td><td>null</td><td>null</td><td></td><td>null</td><td>null</td><td></td><td>1.13E+01</td><td>1.47E-04</td><td>Up</td><td>null</td><td>null</td><td></td><td></td><td></td><td></td><td></td><td></td><td></td><td></td></tr>
<tr><td>Unigene115027_All</td><td>442</td><td>0.36</td><td>0.23</td><td>0.00</td><td>0.23</td><td>0.00</td><td>0.00</td><td>0.67</td><td>2.84</td><td>1.26</td><td>-6.31E-01</td><td>7.28E-01</td><td></td><td>-8.47E+00</td><td>2.46E-01</td><td></td><td>-7.82E+00</td><td>3.15E-01</td><td></td><td>-7.82E+00</td><td>4.03E-01</td><td></td><td>2.08E+00</td><td>5.41E-04</td><td>Up</td><td>9.14E-01</td><td>2.89E-01</td><td></td><td></td><td></td><td>Unigene115027_All//8.00E-43//CO125212</td><td></td><td></td><td></td><td></td></tr>
<tr><td>Unigene11503_All</td><td>1139</td><td>3.86</td><td>3.96</td><td>3.32</td><td>7.13</td><td>6.32</td><td>6.82</td><td>5.75</td><td>2.20</td><td>4.48</td><td>3.71E-02</td><td>9.10E-01</td><td></td><td>-2.17E-01</td><td>4.98E-01</td><td></td><td>-1.74E-01</td><td>4.48E-01</td><td></td><td>-6.39E-02</td><td>7.81E-01</td><td></td><td>-1.39E+00</td><td>1.98E-10</td><td>Down</td><td>-3.62E-01</td><td>7.55E-02</td><td></td><td>Unigene11503_All//gi|63087714|emb|CAI93172.1|//0.00E+00//beta-1,3-glucuronosyltransferase [Gossypium raimondii]</td><td>Unigene11503_All//5.00E-98//AT1G27600| glycosyl transferase family 43 protein</td><td>Unigene11503_All//0.00E+00//TC129656</td><td></td><td></td><td>GO:0015020//GO:0044464</td><td>nr -</td></tr>
<tr><td>Unigene115030_All</td><td>363</td><td>0.00</td><td>0.00</td><td>0.00</td><td>0.00</td><td>0.00</td><td>0.00</td><td>0.00</td><td>1.61</td><td>0.00</td><td>null</td><td>null</td><td></td><td>null</td><td>null</td><td></td><td>null</td><td>null</td><td></td><td>null</td><td>null</td><td></td><td>1.07E+01</td><td>2.81E-04</td><td>Up</td><td>null</td><td>null</td><td></td><td>Unigene115030_All//gi|18407264|ref|NP_566095.1|//8.00E-24//UBQ6; protein binding [Arabidopsis thaliana] >gi|14030651|gb|AAK53000.1|AF375416_1 At2g47110/F14M4.6 [Arabidopsis thaliana] >gi|166936|gb|AAA32907.1| ubiquitin extension protein (UBQ6) [Arabidopsis thaliana] >gi|3522953|gb|AAC34235.1| ubiquitin extension protein (UBQ6) [Arabidopsis thaliana] >gi|21593980|gb|AAM65909.1| ubiquitin extension protein (UBQ6) [Arabidopsis thaliana] >gi|22655410|gb|AAM98297.1| At2g47110/F14M4.6 [Arabidopsis thaliana] >gi|23505779|gb|AAN28749.1| At2g47110/F14M4.6 [Arabidopsis thaliana]</td><td>Unigene115030_All//4.00E-13//AT2G47110| UBQ6; protein binding</td><td></td><td></td><td>ko03010|Ribosome</td><td>GO:0015935//GO:0044267</td><td>nr +</td></tr>
<tr><td>Unigene115034_All</td><td>392</td><td>0.00</td><td>0.00</td><td>0.00</td><td>0.38</td><td>0.09</td><td>0.23</td><td>0.22</td><td>2.24</td><td>0.00</td><td>null</td><td>null</td><td></td><td>null</td><td>null</td><td></td><td>-2.12E+00</td><td>3.87E-01</td><td></td><td>-7.26E-01</td><td>7.16E-01</td><td></td><td>3.38E+00</td><td>1.75E-04</td><td>Up</td><td>-7.75E+00</td><td>3.60E-01</td><td></td><td>Unigene115034_All//gi|186532568|ref|NP_200663.2|//7.00E-18//EOL2 (ETO1-LIKE 2); binding / protein binding [Arabidopsis thaliana]</td><td>Unigene115034_All//4.00E-19//AT5G58550| EOL2 (ETO1-LIKE 2); binding / protein binding</td><td>Unigene115034_All//4.00E-41//TC171012</td><td></td><td></td><td>GO:0005488</td><td>nr +</td></tr>
<tr><td>Unigene115045_All</td><td>469</td><td>0.00</td><td>0.43</td><td>0.00</td><td>0.11</td><td>0.00</td><td>0.10</td><td>0.18</td><td>1.96</td><td>0.25</td><td>8.76E+00</td><td>1.37E-01</td><td></td><td>null</td><td>null</td><td></td><td>-6.73E+00</td><td>5.60E-01</td><td></td><td>-1.41E-01</td><td>9.55E-01</td><td></td><td>3.44E+00</td><td>9.84E-05</td><td>Up</td><td>4.99E-01</td><td>8.08E-01</td><td></td><td>Unigene115045_All//gi|255559276|ref|XP_002520658.1|//3.00E-14//conserved hypothetical protein [Ricinus communis] >gi|223540043|gb|EEF41620.1| conserved hypothetical protein [Ricinus communis]</td><td></td><td></td><td></td><td></td><td>GO:0005488</td><td>nr +</td></tr>
<tr><td>Unigene11505_All</td><td>1265</td><td>2.44</td><td>1.04</td><td>1.05</td><td>2.84</td><td>2.18</td><td>3.07</td><td>2.61</td><td>1.72</td><td>2.87</td><td>-1.23E+00</td><td>7.17E-04</td><td>Down</td><td>-1.21E+00</td><td>1.33E-03</td><td></td><td>-3.81E-01</td><td>2.58E-01</td><td></td><td>1.15E-01</td><td>7.31E-01</td><td></td><td>-6.02E-01</td><td>4.98E-02</td><td></td><td>1.37E-01</td><td>6.40E-01</td><td></td><td>Unigene11505_All//gi|63087714|emb|CAI93172.1|//1.00E-133//beta-1,3-glucuronosyltransferase [Gossypium raimondii]</td><td>Unigene11505_All//1.00E-112//AT1G27600| glycosyl transferase family 43 protein</td><td>Unigene11505_All//0.00E+00//TC167504</td><td></td><td></td><td>GO:0015020//GO:0044464</td><td>nr -</td></tr>
<tr><td>Unigene115050_All</td><td>401</td><td>0.00</td><td>0.13</td><td>0.00</td><td>0.37</td><td>0.00</td><td>1.13</td><td>0.21</td><td>2.92</td><td>0.10</td><td>6.98E+00</td><td>6.20E-01</td><td></td><td>null</td><td>null</td><td></td><td>-8.54E+00</td><td>1.66E-01</td><td></td><td>1.60E+00</td><td>2.05E-01</td><td></td><td>3.79E+00</td><td>2.86E-06</td><td>Up</td><td>-1.09E+00</td><td>6.77E-01</td><td></td><td></td><td></td><td>Unigene115050_All//9.00E-92//gi|109878692|gb|DW507662.1|DW507662</td><td></td><td></td><td></td><td></td></tr>
<tr><td>Unigene115056_All</td><td>258</td><td>0.41</td><td>0.00</td><td>0.00</td><td>0.97</td><td>0.67</td><td>0.00</td><td>0.66</td><td>4.70</td><td>2.32</td><td>-8.66E+00</td><td>3.75E-01</td><td></td><td>-8.66E+00</td><td>3.97E-01</td><td></td><td>-5.33E-01</td><td>7.13E-01</td><td></td><td>-9.92E+00</td><td>7.35E-02</td><td></td><td>2.84E+00</td><td>3.39E-05</td><td>Up</td><td>1.82E+00</td><td>3.95E-02</td><td></td><td></td><td></td><td>Unigene115056_All//2.00E-131//DW511260</td><td></td><td></td><td></td><td>ESTscan -</td></tr>
<tr><td>Unigene11506_All</td><td>1603</td><td>2.48</td><td>1.33</td><td>1.73</td><td>3.92</td><td>3.20</td><td>2.93</td><td>2.95</td><td>1.96</td><td>1.44</td><td>-9.02E-01</td><td>3.29E-03</td><td></td><td>-5.22E-01</td><td>1.08E-01</td><td></td><td>-2.91E-01</td><td>2.42E-01</td><td></td><td>-4.18E-01</td><td>8.56E-02</td><td></td><td>-5.95E-01</td><td>1.63E-02</td><td></td><td>-1.04E+00</td><td>2.14E-05</td><td>Down</td><td>Unigene11506_All//gi|63087714|emb|CAI93172.1|//1.00E-113//beta-1,3-glucuronosyltransferase [Gossypium raimondii]</td><td>Unigene11506_All//1.00E-68//AT1G27600| glycosyl transferase family 43 protein</td><td>Unigene11506_All//0.00E+00//gi|78324411|gb|DT544685.1|DT544685</td><td></td><td></td><td>GO:0015020//GO:0044464</td><td>nr -</td></tr>
<tr><td>Unigene115063_All</td><td>417</td><td>0.00</td><td>0.00</td><td>0.00</td><td>0.00</td><td>0.00</td><td>0.00</td><td>1.42</td><td>4.21</td><td>0.00</td><td>null</td><td>null</td><td></td><td>null</td><td>null</td><td></td><td>null</td><td>null</td><td></td><td>null</td><td>null</td><td></td><td>1.57E+00</td><td>7.26E-04</td><td>Up</td><td>-1.05E+01</td><td>1.41E-04</td><td>Down</td><td>Unigene115063_All//gi|214003725|gb|ACJ60905.1|//2.00E-74//alpha tubulin [Betula pendula]</td><td>Unigene115063_All//1.00E-73//AT4G14960| TUA6; structural constituent of cytoskeleton</td><td>Unigene115063_All//6.00E-93//TC130219</td><td></td><td>ko04145|Phagosome</td><td>GO:0007017//GO:0015630//GO:0017111//GO:0032561//GO:0032991//GO:0043623</td><td>nr -</td></tr>
<tr><td>Unigene115067_All</td><td>296</td><td>4.77</td><td>5.48</td><td>5.58</td><td>4.54</td><td>2.91</td><td>6.71</td><td>6.28</td><td>9.18</td><td>23.82</td><td>1.99E-01</td><td>6.82E-01</td><td></td><td>2.25E-01</td><td>6.52E-01</td><td></td><td>-6.44E-01</td><td>2.63E-01</td><td></td><td>5.63E-01</td><td>2.43E-01</td><td></td><td>5.46E-01</td><td>1.18E-01</td><td></td><td>1.92E+00</td><td>1.05E-17</td><td>Up</td><td></td><td></td><td>Unigene115067_All//2.00E-110//ES846306</td><td></td><td></td><td></td><td></td></tr>
<tr><td>Unigene11507_All</td><td>725</td><td>12.34</td><td>4.19</td><td>2.50</td><td>16.56</td><td>15.91</td><td>15.51</td><td>15.69</td><td>9.34</td><td>8.30</td><td>-1.56E+00</td><td>1.60E-13</td><td>Down</td><td>-2.31E+00</td><td>1.97E-21</td><td>Down</td><td>-5.75E-02</td><td>7.98E-01</td><td></td><td>-9.43E-02</td><td>6.24E-01</td><td></td><td>-7.48E-01</td><td>6.72E-07</td><td></td><td>-9.19E-01</td><td>8.92E-10</td><td></td><td>Unigene11507_All//gi|297834468|ref|XP_002885116.1|//5.00E-68//glycosyl transferase family 1 protein [Arabidopsis lyrata subsp. lyrata] >gi|297330956|gb|EFH61375.1| glycosyl transferase family 1 protein [Arabidopsis lyrata subsp. lyrata]</td><td>Unigene11507_All//2.00E-68//AT3G15940| glycosyl transferase family 1 protein</td><td>Unigene11507_All//0.00E+00//TC142889</td><td></td><td>ko00561|Glycerolipid metabolism</td><td>GO:0008152//GO:0009536//GO:0016740</td><td>nr +</td></tr>
<tr><td>Unigene115074_All</td><td>524</td><td>0.00</td><td>0.19</td><td>0.00</td><td>0.19</td><td>0.72</td><td>0.52</td><td>0.48</td><td>5.34</td><td>1.67</td><td>7.60E+00</td><td>3.89E-01</td><td></td><td>null</td><td>null</td><td></td><td>1.93E+00</td><td>1.94E-01</td><td></td><td>1.44E+00</td><td>4.09E-01</td><td></td><td>3.46E+00</td><td>2.53E-13</td><td>Up</td><td>1.79E+00</td><td>1.04E-02</td><td></td><td>Unigene115074_All//gi|1732406|gb|AAB38748.1|//9.00E-44//S25-XP1 DNA binding protein [Nicotiana tabacum]</td><td>Unigene115074_All//8.00E-39//AT3G23240| ERF1 (ETHYLENE RESPONSE FACTOR 1); DNA binding / transcription activator/ transcription factor</td><td>Unigene115074_All//3.00E-24//ES803928</td><td>AP2-EREBP</td><td></td><td>GO:0001071//GO:0003676//GO:0006351//GO:0010468//GO:0043231</td><td>nr -</td></tr>
<tr><td>Unigene115075_All</td><td>285</td><td>0.00</td><td>0.00</td><td>0.00</td><td>0.00</td><td>0.00</td><td>0.00</td><td>68.54</td><td>8.07</td><td>59.55</td><td>null</td><td>null</td><td></td><td>null</td><td>null</td><td></td><td>null</td><td>null</td><td></td><td>null</td><td>null</td><td></td><td>-3.09E+00</td><td>6.98E-81</td><td>Down</td><td>-2.03E-01</td><td>7.72E-02</td><td></td><td>Unigene115075_All//gi|791146|emb|CAA60020.1|//2.00E-44//extensin-like protein [Vigna unguiculata]</td><td></td><td></td><td></td><td></td><td></td><td>nr -</td></tr>
<tr><td>Unigene115077_All</td><td>362</td><td>0.00</td><td>0.00</td><td>0.00</td><td>0.00</td><td>0.00</td><td>0.00</td><td>2.57</td><td>4.85</td><td>0.44</td><td>null</td><td>null</td><td></td><td>null</td><td>null</td><td></td><td>null</td><td>null</td><td></td><td>null</td><td>null</td><td></td><td>9.16E-01</td><td>3.87E-02</td><td></td><td>-2.55E+00</td><td>5.79E-04</td><td>Down</td><td>Unigene115077_All//gi|145408590|ref|YP_001152214.1|//1.00E-07//ORF124 [Pinus koraiensis] >gi|145048839|gb|ABP35456.1| ORF124 [Pinus koraiensis]</td><td></td><td>Unigene115077_All//3.00E-17//TC149074</td><td></td><td></td><td></td><td>nr -</td></tr>
<tr><td>Unigene115078_All</td><td>276</td><td>0.00</td><td>0.00</td><td>0.00</td><td>0.00</td><td>0.00</td><td>0.00</td><td>0.31</td><td>2.73</td><td>0.58</td><td>null</td><td>null</td><td></td><td>null</td><td>null</td><td></td><td>null</td><td>null</td><td></td><td>null</td><td>null</td><td></td><td>3.15E+00</td><td>9.54E-04</td><td>Up</td><td>9.14E-01</td><td>6.25E-01</td><td></td><td></td><td></td><td></td><td></td><td></td><td></td><td></td></tr>
<tr><td>Unigene115079_All</td><td>576</td><td>0.00</td><td>0.00</td><td>0.00</td><td>0.00</td><td>0.00</td><td>0.00</td><td>0.00</td><td>1.74</td><td>1.04</td><td>null</td><td>null</td><td></td><td>null</td><td>null</td><td></td><td>null</td><td>null</td><td></td><td>null</td><td>null</td><td></td><td>1.08E+01</td><td>3.93E-07</td><td>Up</td><td>1.00E+01</td><td>1.76E-04</td><td>Up</td><td>Unigene115079_All//gi|121077603|gb|ABM47305.1|//4.00E-06//nucleic acid binding protein [Volvox carteri f. nagariensis] >gi|300269119|gb|EFJ53299.1| nucleic acid binding protein [Volvox carteri f. nagariensis]</td><td>Unigene115079_All//1.00E-05//AT2G21060| ATGRP2B (GLYCINE-RICH PROTEIN 2B); DNA binding / nucleic acid binding / zinc ion binding</td><td></td><td>CSD</td><td></td><td></td><td>nr +</td></tr>
<tr><td>Unigene11508_All</td><td>2484</td><td>3.22</td><td>3.16</td><td>1.97</td><td>6.96</td><td>7.50</td><td>6.02</td><td>11.63</td><td>5.74</td><td>6.88</td><td>-2.76E-02</td><td>9.09E-01</td><td></td><td>-7.09E-01</td><td>8.87E-04</td><td></td><td>1.08E-01</td><td>4.99E-01</td><td></td><td>-2.10E-01</td><td>1.52E-01</td><td></td><td>-1.02E+00</td><td>7.35E-27</td><td>Down</td><td>-7.57E-01</td><td>4.56E-17</td><td></td><td>Unigene11508_All//gi|15233237|ref|NP_188215.1|//1.00E-121//glycosyl transferase family 1 protein [Arabidopsis thaliana] >gi|9294599|dbj|BAB02880.1| glycosyl transferases-like protein [Arabidopsis thaliana] >gi|20147191|gb|AAM10311.1| AT3g15940/MVC8_7 [Arabidopsis thaliana]</td><td>Unigene11508_All//2.00E-119//AT3G15940| glycosyl transferase family 1 protein</td><td>Unigene11508_All//0.00E+00//ES848925</td><td></td><td>ko00561|Glycerolipid metabolism</td><td></td><td>nr +</td></tr>
<tr><td>Unigene115080_All</td><td>229</td><td>0.00</td><td>0.44</td><td>0.00</td><td>0.22</td><td>0.00</td><td>0.39</td><td>8.68</td><td>13.69</td><td>2.78</td><td>8.79E+00</td><td>3.89E-01</td><td></td><td>null</td><td>null</td><td></td><td>-7.76E+00</td><td>5.60E-01</td><td></td><td>8.59E-01</td><td>7.96E-01</td><td></td><td>6.58E-01</td><td>3.70E-02</td><td></td><td>-1.64E+00</td><td>1.05E-04</td><td>Down</td><td>Unigene115080_All//gi|224111880|ref|XP_002316010.1|//2.00E-10//cytochrome P450 [Populus trichocarpa] >gi|222865050|gb|EEF02181.1| cytochrome P450 [Populus trichocarpa]</td><td>Unigene115080_All//3.00E-08//AT3G14690| CYP72A15; electron carrier/ heme binding / iron ion binding / monooxygenase/ oxygen binding</td><td>Unigene115080_All//1.00E-73//gi|109890708|gb|DW519672.1|DW519672</td><td></td><td></td><td>GO:0005506//GO:0008152//GO:0016634</td><td>nr -</td></tr>
<tr><td>Unigene115083_All</td><td>204</td><td>9.49</td><td>3.73</td><td>2.87</td><td>13.43</td><td>12.49</td><td>15.50</td><td>74.40</td><td>40.77</td><td>32.22</td><td>-1.35E+00</td><td>4.99E-03</td><td></td><td>-1.72E+00</td><td>9.79E-04</td><td>Down</td><td>-1.05E-01</td><td>8.15E-01</td><td></td><td>2.07E-01</td><td>6.32E-01</td><td></td><td>-8.68E-01</td><td>2.73E-11</td><td></td><td>-1.21E+00</td><td>2.07E-19</td><td>Down</td><td>Unigene115083_All//gi|8778823|gb|AAF79822.1|AC026875_2//5.00E-15//T6D22.2 [Arabidopsis thaliana]</td><td>Unigene115083_All//4.00E-11//AT5G60390| elongation factor 1-alpha / EF-1-alpha</td><td>Unigene115083_All//5.00E-64//gi|78351725|gb|DT571999.1|DT571999</td><td></td><td></td><td>GO:0006412//GO:0008135//GO:0016772//GO:0017111//GO:0032561//GO:0044424</td><td>nr -</td></tr>
<tr><td>Unigene115089_All</td><td>272</td><td>0.00</td><td>0.00</td><td>0.00</td><td>0.00</td><td>0.00</td><td>0.00</td><td>0.31</td><td>3.07</td><td>1.17</td><td>null</td><td>null</td><td></td><td>null</td><td>null</td><td></td><td>null</td><td>null</td><td></td><td>null</td><td>null</td><td></td><td>3.31E+00</td><td>3.10E-04</td><td>Up</td><td>1.91E+00</td><td>1.54E-01</td><td></td><td></td><td></td><td>Unigene115089_All//3.00E-16//CO092801</td><td></td><td></td><td></td><td></td></tr>
<tr><td>Unigene11509_All</td><td>5030</td><td>3.18</td><td>1.88</td><td>1.93</td><td>8.69</td><td>6.92</td><td>5.63</td><td>6.88</td><td>1.85</td><td>3.60</td><td>-7.57E-01</td><td>6.59E-08</td><td></td><td>-7.24E-01</td><td>5.08E-07</td><td></td><td>-3.28E-01</td><td>1.17E-05</td><td></td><td>-6.26E-01</td><td>1.19E-15</td><td></td><td>-1.89E+00</td><td>1.16E-80</td><td>Down</td><td>-9.37E-01</td><td>7.97E-29</td><td></td><td>Unigene11509_All//gi|15233237|ref|NP_188215.1|//0.00E+00//glycosyl transferase family 1 protein [Arabidopsis thaliana] >gi|9294599|dbj|BAB02880.1| glycosyl transferases-like protein [Arabidopsis thaliana] >gi|20147191|gb|AAM10311.1| AT3g15940/MVC8_7 [Arabidopsis thaliana]</td><td>Unigene11509_All//0.00E+00//AT3G15940| glycosyl transferase family 1 protein</td><td>Unigene11509_All//0.00E+00//TC158791</td><td></td><td>ko00561|Glycerolipid metabolism</td><td>GO:0008152//GO:0016740</td><td>nr -</td></tr>
<tr><td>Unigene1151_All</td><td>2003</td><td>63.89</td><td>140.45</td><td>83.37</td><td>103.28</td><td>104.27</td><td>132.81</td><td>109.70</td><td>328.02</td><td>179.01</td><td>1.14E+00</td><td>5.13E-248</td><td>Up</td><td>3.84E-01</td><td>1.12E-21</td><td></td><td>1.37E-02</td><td>8.07E-01</td><td></td><td>3.63E-01</td><td>3.40E-34</td><td></td><td>1.58E+00</td><td>0.00E+00</td><td>Up</td><td>7.06E-01</td><td>4.82E-178</td><td></td><td>Unigene1151_All//gi|225446893|ref|XP_002284064.1|//0.00E+00//PREDICTED: similar to Citrate synthase, glyoxysomal [Vitis vinifera]</td><td>Unigene1151_All//0.00E+00//AT3G58750| CSY2 (citrate synthase 2); citrate (SI)-synthase</td><td>Unigene1151_All//0.00E+00//TC133360</td><td></td><td>ko00020|Citrate cycle (TCA cycle)//ko00630|Glyoxylate and dicarboxylate metabolism</td><td>GO:0005777//GO:0009060//GO:0009062//GO:0016747//GO:0044262//GO:0046912</td><td>nr -</td></tr>
<tr><td>Unigene11510_All</td><td>1408</td><td>0.93</td><td>1.66</td><td>1.32</td><td>3.15</td><td>2.37</td><td>3.43</td><td>11.86</td><td>12.17</td><td>59.39</td><td>8.33E-01</td><td>4.59E-02</td><td></td><td>5.11E-01</td><td>3.12E-01</td><td></td><td>-4.09E-01</td><td>1.61E-01</td><td></td><td>1.24E-01</td><td>6.78E-01</td><td></td><td>3.71E-02</td><td>7.92E-01</td><td></td><td>2.32E+00</td><td>3.25E-256</td><td>Up</td><td>Unigene11510_All//gi|15226985|ref|NP_180455.1|//1.00E-121//PLL4 (POLTERGEIST LIKE 4); catalytic/ protein serine/threonine phosphatase [Arabidopsis thaliana] >gi|75216896|sp|Q9ZV25.1|P2C23_ARATH RecName: Full=Probable protein phosphatase 2C 23; Short=AtPP2C23; AltName: Full=Protein phosphatase 2C PLL4; Short=PP2C PLL4; AltName: Full=Protein POLTERGEIST-LIKE 4</td><td>Unigene11510_All//3.00E-96//AT1G07630| PLL5; catalytic/ protein serine/threonine phosphatase</td><td>Unigene11510_All//0.00E+00//TC139366</td><td></td><td></td><td></td><td>nr +</td></tr>
</table>

Unigene11511_All	2796	8.94	9.97	6.80	17.85	16.50	13.60	28.02	29.00	57.54	1.56E-01	1.61E-01		-3.96E-01	4.81E-04		-1.13E-01	1.72E-01		-3.92E-01	5.96E-08		4.96E-02	4.40E-01		1.04E+00	1.12E-153	Up	Unigene11511_All//gi|18390789|ref|NP_563791.1|//0.00E+00//PLL5; catalytic/ protein serine/threonine phosphatase [Arabidopsis thaliana] >gi|75180175|sp|Q9LQN6.1|P2C04_ARATH RecName: Full=Probable protein phosphatase 2C 4; Short=AtPP2C04; AltName: Full=Protein phosphatase 2C PLL5; Short=PP2C PLL5; AltName: Full=Protein POLTERGEIST-LIKE 5 >gi|8439909|gb|AAF75095.1|AC007583_31 It contains protein phosphatase 2C domain PF|00481. ESTs gb|H36120 and gb|36519 come from this gene [Arabidopsis thaliana]	Unigene11511_All//0.00E+00//AT1G07630| PLL5; catalytic/ protein serine/threonine phosphatase	Unigene11511_All//0.00E+00//gi|78354240|gb|DT574514.1|DT574514			GO:0004721//GO:0016020//GO:0048827	nr -
Unigene115112_All	296	0.00	0.00	0.00	0.00	0.00	0.00	12.43	2.26	2.15	null	null		null	null		null	null		null	null		-2.46E+00	1.66E-12	Down	-2.53E+00	2.37E-13	Down	Unigene115112_All//gi|20197696|gb|AAM15209.1|//1.00E-24//senescence-associated protein [Arabidopsis thaliana] >gi|20197726|gb|AAM15226.1| senescence-associated protein [Arabidopsis thaliana]	Unigene115112_All//5.00E-26//AT2G21045| INVOLVED IN: aging; EXPRESSED IN: hypocotyl, root; CONTAINS InterPro DOMAIN/s: Rhodanese-like (InterPro:IPR001763); BEST Arabidopsis thaliana protein match is: unknown protein (TAIR:AT5G66170.2); Has 1919 Blast hits to 1917 proteins in 542 species: Archae - 34; Bacteria - 1258; Metazoa - 17; Fungi - 21; Plants - 115; Viruses - 0; Other Eukaryotes - 474 (source: NCBI BLink).				GO:0031410//GO:0032502	nr -
Unigene115115_All	339	0.00	0.00	0.00	0.00	0.00	0.00	11.35	14.06	30.44	null	null		null	null		null	null		null	null		3.09E-01	2.39E-01		1.42E+00	5.84E-17	Up	Unigene115115_All//gi|147791048|emb|CAN65763.1|//7.00E-07//hypothetical protein [Vitis vinifera]		Unigene115115_All//1.00E-65//ES810763				nr +
Unigene11512_All	444	0.59	1.03	1.92	1.23	2.02	1.73	4.57	1.60	2.60	8.02E-01	4.60E-01		1.70E+00	4.53E-02		7.08E-01	3.74E-01		4.87E-01	5.91E-01		-1.51E+00	3.50E-04	Down	-8.13E-01	3.63E-02		Unigene11512_All//gi|18402856|ref|NP_565734.1|//5.00E-33//integral membrane transporter family protein [Arabidopsis thaliana] >gi|20197619|gb|AAD15400.2| expressed protein [Arabidopsis thaliana]	Unigene11512_All//2.00E-32//AT2G32040| integral membrane transporter family protein	Unigene11512_All//0.00E+00//TC177047			GO:0008517//GO:0009526//GO:0051234	nr -
Unigene115123_All	286	0.00	0.00	0.00	0.00	0.00	1.26	0.00	4.09	0.42	null	null		null	null		null	null		1.03E+01	2.37E-02		1.20E+01	2.73E-08	Up	8.71E+00	2.43E-01		Unigene115123_All//gi|224096008|ref|XP_002310518.1|//2.00E-11//cation proton exchanger [Populus trichocarpa] >gi|222853421|gb|EEE90968.1| cation proton exchanger [Populus trichocarpa]	Unigene115123_All//2.00E-11//AT2G13620| ATCHX15; monovalent cation:proton antiporter/ sodium:hydrogen antiporter				GO:0006811//GO:0015078//GO:0015298//GO:0031224	nr -
Unigene115128_All	424	0.00	0.00	0.00	0.00	0.00	0.00	0.20	1.87	0.00	null	null		null	null		null	null		null	null		3.23E+00	5.44E-04	Up	-7.64E+00	3.60E-01		Unigene115128_All//gi|145408590|ref|YP_001152214.1|//9.00E-07//ORF124 [Pinus koraiensis] >gi|145048839|gb|ABP35456.1| ORF124 [Pinus koraiensis]		Unigene115128_All//1.00E-07//TC134836				nr +
Unigene115129_All	385	0.00	0.00	0.00	0.00	0.00	0.00	0.55	3.15	0.00	null	null		null	null		null	null		null	null		2.52E+00	1.15E-04	Up	-9.10E+00	5.81E-02		Unigene115129_All//gi|159138763|gb|ABW89384.1|//6.00E-37//40S ribosomal protein S16 [Helianthus annuus] >gi|159138765|gb|ABW89385.1| 40S ribosomal protein S16 [Helianthus annuus] >gi|159138767|gb|ABW89386.1| 40S ribosomal protein S16 [Helianthus annuus] >gi|159138769|gb|ABW89387.1| 40S ribosomal protein S16 [Helianthus annuus] >gi|159138771|gb|ABW89388.1| 40S ribosomal protein S16 [Helianthus annuus] >gi|159138773|gb|ABW89389.1| 40S ribosomal protein S16 [Helianthus annuus] >gi|159138775|gb|ABW89390.1| 40S ribosomal protein S16 [Helianthus annuus] >gi|159138777|gb|ABW89391.1| 40S ribosomal protein S16 [Helianthus annuus] >gi|159138779|gb|ABW89392.1| 40S ribosomal protein S16 [Helianthus annuus] >gi|159138781|gb|ABW89393.1| 40S ribosomal protein S16 [Helianthus	Unigene115129_All//4.00E-37//AT2G09990| 40S ribosomal protein S16 (RPS16A)			ko03010|Ribosome	GO:0005198//GO:0010467//GO:0030529	nr +
Unigene115131_All	263	0.00	0.00	0.00	0.57	0.65	0.52	1.29	7.79	0.76	null	null		null	null		2.04E-01	9.50E-01		-1.41E-01	9.44E-01		2.60E+00	1.19E-07	Up	-7.64E-01	5.01E-01				Unigene115131_All//8.00E-48//TC144723				
Unigene115139_All	1059	0.00	0.38	0.20	0.47	0.49	0.34	1.52	6.59	0.71	8.58E+00	1.36E-02		7.65E+00	1.42E-01		5.24E-02	9.80E-01		-4.63E-01	6.59E-01		2.12E+00	2.36E-19	Up	-1.09E+00	1.58E-02		Unigene115139_All//gi|18404500|ref|NP_566763.1|//1.00E-95//auxin-responsive family protein [Arabidopsis thaliana] >gi|79313415|ref|NP_001030764.1| auxin-responsive family protein [Arabidopsis thaliana] >gi|16323200|gb|AAL15334.1| AT3g25290/MJL12_25 [Arabidopsis thaliana] >gi|25090093|gb|AAN72226.1| At3g25290/MJL12_25 [Arabidopsis thaliana]	Unigene115139_All//2.00E-81//AT3G25290| auxin-responsive family protein	Unigene115139_All//3.00E-81//TC146057			GO:0016020	nr +
Unigene115144_All	449	0.23	0.34	0.00	0.00	0.31	0.00	0.00	9.40	0.53	5.38E-01	7.80E-01		-7.86E+00	3.97E-01		8.26E+00	3.20E-01		null	null		1.32E+01	1.02E-29	Up	9.06E+00	4.40E-02		Unigene115144_All//gi|159138935|gb|ABW89467.1|//6.00E-42//low molecular weight heat shock protein [Gossypium hirsutum]	Unigene115144_All//3.00E-32//AT1G07400| 17.8 kDa class I heat shock protein (HSP17.8-CI)	Unigene115144_All//0.00E+00//DT465134			GO:0044424//GO:0050896	nr -
Unigene115149_All	658	0.00	0.00	0.00	0.00	0.00	0.00	0.00	2.48	0.00	null	null		null	null		null	null		null	null		1.13E+01	1.71E-11	Up	null	null			Unigene115149_All//6.00E-05//AT3G18485| ILR2 (IAA-LEUCINE RESISTANT 2)					
Unigene115159_All	246	0.00	0.00	0.00	0.20	0.00	0.37	7.91	2.55	16.03	null	null		null	null		-7.66E+00	5.60E-01		8.59E-01	7.96E-01		-1.63E+00	2.06E-04	Down	1.02E+00	1.64E-04	Up			Unigene115159_All//2.00E-32//ES835202				
Unigene115162_All	371	0.56	0.14	0.57	1.88	0.74	0.00	3.30	2.37	2.15	-2.05E+00	3.34E-01		2.54E-02	1.00E+00		-1.34E+00	1.09E-01		-1.09E+01	1.96E-04	Down	-4.82E-01	4.08E-01		-6.22E-01	2.39E-01				Unigene115162_All//0.00E+00//TC141163				
Unigene115163_All	240	0.00	0.00	0.00	0.62	1.15	0.19	0.18	3.13	0.00	null	null		null	null		8.82E-01	5.95E-01		-1.73E+00	5.02E-01		4.15E+00	1.92E-04	Up	-7.46E+00	6.15E-01								
Unigene115167_All	460	0.23	0.00	0.00	0.32	0.00	0.29	0.18	1.73	0.95	-7.83E+00	3.75E-01		-7.83E+00	3.97E-01		-8.34E+00	1.66E-01		-1.42E-01	9.44E-01		3.23E+00	5.44E-04	Up	2.37E+00	4.05E-02								
Unigene115178_All	469	0.00	0.00	0.00	0.00	0.00	0.00	0.00	5.35	0.17	null	null		null	null		null	null		null	null		1.24E+01	1.22E-17	Up	7.41E+00	4.05E-01		Unigene115178_All//sp|P0A9D2|GST_ECOLI//2.00E-09//Glutathione S-transferase OS=Escherichia coli (strain K12) GN=gst PE=1 SV=1						swissprot +
Unigene11518_All	306	0.17	0.00	0.00	0.49	1.80	3.39	1.24	0.27	0.65	-7.42E+00	6.17E-01		-7.42E+00	6.29E-01		1.88E+00	9.42E-02		2.80E+00	8.13E-04	Up	-2.19E+00	8.76E-02		-9.34E-01	3.80E-01		Unigene11518_All//gi|115452841|ref|NP_001050021.1|//6.00E-19//Os03g0333100 [Oryza sativa Japonica Group] >gi|113548492|dbj|BAF11935.1| Os03g0333100 [Oryza sativa Japonica Group]	Unigene11518_All//8.00E-16//AT1G06720| INVOLVED IN: ribosome biogenesis; LOCATED IN: nucleus; EXPRESSED IN: 21 plant structures; EXPRESSED DURING: 13 growth stages; CONTAINS InterPro DOMAIN/s: AARP2CN (InterPro:IPR012948), Protein of unknown function DUF663 (InterPro:IPR007034); BEST Arabidopsis thaliana protein match is: unknown protein (TAIR:AT1G42440.1); Has 7944 Blast hits to 5342 proteins in 373 species: Archae - 33; Bacteria - 667; Metazoa - 2609; Fungi - 1045; Plants - 414; Viruses - 80; Other Eukaryotes - 3096 (source: NCBI BLink).	Unigene11518_All//4.00E-136//gi|109853645|gb|DW482624.1|DW482624			GO:0017111//GO:0032555	nr +
Unigene115181_All	247	0.21	0.41	0.22	0.00	1.39	0.73	0.00	2.54	2.10	9.54E-01	7.27E-01		2.57E-02	9.96E-01		1.04E+01	3.02E-02		9.51E+00	1.95E-01		1.13E+01	1.47E-04	Up	1.10E+01	6.14E-04	Up	Unigene115181_All//gi|59668640|emb|CAI53674.1|//3.00E-22//glutamate dehydrogenase 2 [Glycine max]	Unigene115181_All//4.00E-23//AT5G07440| GDH2 (GLUTAMATE DEHYDROGENASE 2); ATP binding / glutamate dehydrogenase [NAD(P)+]/ glutamate dehydrogenase/ oxidoreductase	Unigene115181_All//2.00E-79//gi|164272922|gb|EV489392.1|EV489392		ko00910|Nitrogen metabolism//ko00250|Alanine, aspartate and glutamate metabolism//ko00330|Arginine and proline metabolism	GO:0006519//GO:0006970//GO:0010038//GO:0016020//GO:0016639//GO:0019222//GO:0032559//GO:0043231//GO:0046914	nr +
Unigene115182_All	447	0.00	1.25	0.00	0.00	0.46	0.20	0.09	2.71	2.50	1.03E+01	2.19E-03		null	null		8.85E+00	1.57E-01		7.66E+00	4.80E-01		4.84E+00	2.06E-07	Up	4.72E+00	6.01E-07	Up			Unigene115182_All//5.00E-143//TC173956				
Unigene115185_All	573	0.00	0.00	0.00	0.00	0.00	0.00	1.18	0.95	0.00	null	null		null	null		null	null		null	null		-3.16E-01	6.74E-01		-1.02E+01	3.56E-05	Down	Unigene115185_All//gi|29539332|dbj|BAC67667.1|//4.00E-85//elongation factor-1alpha [Cyanidioschyzon merolae]	Unigene115185_All//3.00E-80//AT5G60390| elongation factor 1-alpha / EF-1-alpha	Unigene115185_All//4.00E-70//TC166357			GO:0006412//GO:0008135//GO:0017111//GO:0032561//GO:0044424	nr +
Unigene115189_All	795	0.13	0.70	0.40	0.75	0.48	0.74	0.27	0.26	1.35	2.41E+00	3.93E-02		1.61E+00	3.05E-01		-6.58E-01	4.78E-01		-2.59E-02	9.64E-01		-1.64E-02	1.02E+00		2.35E+00	4.46E-04	Up	Unigene115189_All//gi|15229025|ref|NP_190449.1|//6.00E-36//sterile alpha motif (SAM) domain-containing protein [Arabidopsis thaliana]	Unigene115189_All//1.00E-32//AT3G48800| sterile alpha motif (SAM) domain-containing protein	Unigene115189_All//0.00E+00//TC156752				nr -
Unigene11519_All	1229	2.60	0.70	1.43	6.44	4.48	5.73	5.95	2.72	4.89	-1.89E+00	1.03E-06	Down	-8.61E-01	1.81E-02		-5.24E-01	7.58E-03		-1.69E-01	4.78E-01		-1.13E+00	1.30E-08	Down	-2.82E-01	1.50E-01		Unigene11519_All//gi|7523707|gb|AAF63146.1|AC011001_16//1.00E-125//Putative membrane protein [Arabidopsis thaliana]	Unigene11519_All//9.00E-98//AT1G06720| INVOLVED IN: ribosome biogenesis; LOCATED IN: nucleus; EXPRESSED IN: 21 plant structures; EXPRESSED DURING: 13 growth stages; CONTAINS InterPro DOMAIN/s: AARP2CN (InterPro:IPR012948), Protein of unknown function DUF663 (InterPro:IPR007034); BEST Arabidopsis thaliana protein match is: unknown protein (TAIR:AT1G42440.1); Has 7944 Blast hits to 5342 proteins in 373 species: Archae - 33; Bacteria - 667; Metazoa - 2609; Fungi - 1045; Plants - 414; Viruses - 80; Other Eukaryotes - 3096 (source: NCBI BLink).	Unigene11519_All//4.00E-90//TC137970		ko04120|Ubiquitin mediated proteolysis		nr +

Unigene115191_All	455	0.00	0.56	0.00	0.00	0.00	0.00	0.28	2.20	0.18	9.12E+00	7.88E-02		null	null		null	null		null	null		2.98E+00	1.37E-04	Up	-6.71E-01	7.20E-01								
Unigene115193_All	263	0.00	0.00	0.00	0.00	0.00	0.00	1.93	2.86	0.00	null	null		null	null		null	null		null	null		5.68E-01	4.47E-01		-1.09E+01	5.54E-04	Down							
Unigene115194_All	397	0.00	0.00	0.00	0.00	0.00	0.00	1.28	4.74	1.81	null	null		null	null		null	null		null	null		1.89E+00	4.67E-05	Up	4.99E-01	5.01E-01								
Unigene115200_All	612	0.00	0.00	0.00	0.00	0.11	0.00	2.00	4.71	1.95	null	null		null	null		6.81E+00	6.13E-01		null	null		1.23E+00	2.50E-04	Up	-3.69E-02	9.60E-01		Unigene115200_All//gi|147765928|emb|CAN68977.1|//3.00E-07//hypothetical protein [Vitis vinifera]		Unigene115200_All//2.00E-07//FL576891				nr -
Unigene115204_All	508	0.00	0.00	0.00	0.00	0.00	0.00	0.58	2.72	2.12	null	null		null	null		null	null		null	null		2.22E+00	1.31E-04	Up	1.86E+00	2.85E-03								
Unigene115206_All	324	0.00	0.00	0.00	0.00	0.00	0.00	0.00	3.48	1.11	null	null		null	null		null	null		null	null		1.18E+01	5.32E-08	Up	1.01E+01	7.30E-03		Unigene115206_All//gi|30013669|gb|AAP03877.1|//5.00E-16//Avr9/Cf-9 rapidly elicited protein 141 [Nicotiana tabacum]	Unigene115206_All//8.00E-14//AT1G42540| ATGLR3.3; intracellular ligand-gated ion channel				GO:0004872//GO:0005215//GO:0031410//GO:0043231	nr -
Unigene115208_All	224	0.00	0.00	0.00	0.00	0.00	0.00	37.56	8.96	11.38	null	null		null	null		null	null		null	null		-2.07E+00	1.50E-22	Down	-1.72E+00	2.22E-18	Down	Unigene115208_All//gi|57867865|gb|AAW57312.1|//2.00E-29//alpha-tubulin [Ceratopteris richardii]	Unigene115208_All//3.00E-17//AT4G14960| TUA6; structural constituent of cytoskeleton	Unigene115208_All//1.00E-36//ES845326		ko04145|Phagosome	GO:0007017//GO:0015630//GO:0017111//GO:0032561//GO:0032991//GO:0043623	nr +
Unigene115209_All	296	1.06	3.59	0.00	0.34	0.23	1.07	0.86	10.59	2.96	1.76E+00	1.43E-02		-1.01E+01	4.93E-02		-5.33E-01	8.18E-01		1.67E+00	3.09E-01		3.63E+00	2.13E-15	Up	1.79E+00	1.04E-02		Unigene115209_All//gi|89276799|gb|ABD66598.1|//6.00E-06//iron-binding protein [Pyrus pyrifolia]	Unigene115209_All//9.00E-07//AT3G11050| ATFER2 (ferritin 2); binding / ferric iron binding / oxidoreductase/ transition metal ion binding	Unigene115209_All//7.00E-33//gi|8381944|gb|BE054899.1|BE054899			GO:0046914	nr +
Unigene115212_All	284	0.00	0.00	0.00	0.00	0.00	0.00	0.00	2.65	0.00	null	null		null	null		null	null		null	null		1.14E+01	2.07E-05	Up	null	null								
Unigene115214_All	319	0.00	0.00	0.00	0.00	0.00	0.00	0.00	3.67	0.00	null	null		null	null		null	null		null	null		1.18E+01	2.73E-08	Up	null	null		Unigene115214_All//gi|300269136|gb|EFJ53316.1|//9.00E-07//hypothetical protein VOLCADRAFT_86475 [Volvox carteri f. nagariensis]					GO:0005488//GO:0017111	nr +
Unigene115217_All	352	0.00	0.00	0.00	0.00	0.00	0.00	1.44	2.02	0.00	null	null		null	null		null	null		null	null		4.86E-01	5.42E-01		-1.05E+01	5.54E-04	Down							ESTscan -
Unigene11522_All	1021	2.66	0.60	2.87	3.17	1.69	1.33	0.91	0.74	0.55	-2.16E+00	8.60E-07	Down	1.06E-01	7.77E-01		-9.11E-01	4.87E-03		-1.26E+00	2.89E-04	Down	-3.06E-01	6.21E-01		-7.38E-01	2.33E-01		Unigene11522_All//gi|240254315|ref|NP_001117543.4|//1.00E-107//peptidase M1 family protein [Arabidopsis thaliana]	Unigene11522_All//1.00E-107//AT1G63770| peptidase M1 family protein	Unigene11522_All//3.00E-106//gi|48812159|gb|CO113472.1|CO113472		ko00480|Glutathione metabolism	GO:0005576//GO:0009526//GO:0009532//GO:0010038//GO:0016020//GO:0070011	nr +
Unigene115233_All	327	0.00	0.00	0.00	0.00	0.00	0.00	0.00	2.05	0.00	null	null		null	null		null	null		null	null		1.10E+01	7.64E-05	Up	null	null		Unigene115233_All//sp|P73623|Y1773_SYNY3//2.00E-08//Pirin-like protein sll1773 OS=Synechocystis sp. (strain PCC 6803) GN=sll1773 PE=3 SV=1						swissprot -
Unigene115242_All	617	0.00	0.00	0.00	0.00	0.00	0.00	0.14	2.10	0.26	null	null		null	null		null	null		null	null		3.94E+00	4.68E-07	Up	9.14E-01	6.25E-01		Unigene115242_All//gi|2773251|gb|AAC15417.1|//2.00E-14//early nodulin [Maackia amurensis]				ko00230|Purine metabolism//ko00240|Pyrimidine metabolism//ko03020|RNA polymerase		nr +
Unigene115243_All	541	0.00	0.00	0.00	0.00	0.00	0.00	0.00	3.71	0.00	null	null		null	null		null	null		null	null		1.19E+01	4.04E-14	Up	null	null		Unigene115243_All//gi|159487004|ref|XP_001701526.1|//3.00E-10//glyoxal or galactose oxidase [Chlamydomonas reinhardtii] >gi|158271587|gb|EDO97403.1| glyoxal or galactose oxidase [Chlamydomonas reinhardtii]					GO:0003824//GO:0005488	nr -
Unigene115254_All	610	0.09	0.50	0.09	0.16	0.11	0.00	0.14	2.60	0.59	2.54E+00	1.51E-01		2.50E-02	9.96E-01		-5.33E-01	8.18E-01		-7.35E+00	4.03E-01		4.23E+00	6.26E-09	Up	2.08E+00	1.01E-01		Unigene115254_All//gi|42566766|ref|NP_193135.2|//7.00E-48//exostosin family protein [Arabidopsis thaliana]	Unigene115254_All//4.00E-48//AT4G13990| exostosin family protein				GO:0016740//GO:0030312//GO:0031410	nr -
Unigene115259_All	680	0.00	0.00	0.00	0.00	0.00	0.07	0.37	2.03	1.23	null	null		null	null		null	null		6.05E+00	7.00E-01		2.44E+00	4.59E-05	Up	1.72E+00	1.56E-02		Unigene115259_All//gi|288310302|gb|ADC45390.1|//1.00E-45//branched chain amino acid transaminase [Cucumis melo]	Unigene115259_All//7.00E-42//AT3G49680| BCAT3 (BRANCHED-CHAIN AMINOTRANSFERASE 3); branched-chain-amino-acid transaminase/ catalytic	Unigene115259_All//3.00E-65//CO076800		ko00280|Valine, leucine and isoleucine degradation//ko00290|Valine, leucine and isoleucine biosynthesis//ko00966|Glucosinolate biosynthesis//ko00770|Pantothenate and CoA biosynthesis	GO:0008152//GO:0008483//GO:0009536	nr -
Unigene11526_All	671	1.87	4.98	6.51	9.80	7.54	10.63	3.28	1.93	2.91	1.41E+00	6.99E-05	Up	1.80E+00	5.51E-08	Up	-3.77E-01	1.02E-01		1.18E-01	6.49E-01		-7.63E-01	4.86E-02		-1.72E-01	6.47E-01		Unigene11526_All//gi|124359654|gb|ABN06026.1|//4.00E-49//Peptidase, trypsin-like serine and cysteine proteases [Medicago truncatula]	Unigene11526_All//6.00E-41//AT5G45030| catalytic	Unigene11526_All//5.00E-153//TC159414			GO:0016787	nr -
Unigene115267_All	495	1.27	19.35	1.83	1.11	1.53	1.19	0.94	6.67	6.84	3.93E+00	8.66E-40	Up	5.28E-01	4.79E-01		4.67E-01	6.07E-01		9.96E-02	9.36E-01		2.83E+00	5.32E-13	Up	2.86E+00	8.82E-14	Up	Unigene115267_All//gi|297804110|ref|XP_002869939.1|//4.00E-12//CTP synthase [Arabidopsis lyrata subsp. lyrata] >gi|297315775|gb|EFH46198.1| CTP synthase [Arabidopsis lyrata subsp. lyrata]	Unigene115267_All//3.00E-12//AT4G20320| CTP synthase/ catalytic	Unigene115267_All//6.00E-81//DN817495		ko00240|Pyrimidine metabolism		nr +
Unigene115270_All	269	0.39	0.00	0.40	0.00	0.00	0.17	1.26	9.79	1.93	-8.60E+00	3.75E-01		2.54E-02	1.00E+00		null	null		7.39E+00	7.00E-01		2.96E+00	5.81E-11	Up	6.15E-01	4.94E-01				Unigene115270_All//6.00E-21//BF268625				
Unigene115271_All	432	0.00	0.00	0.00	0.00	0.00	0.00	2.25	1.16	0.09	null	null		null	null		null	null		null	null		-9.55E-01	1.35E-01		-4.61E+00	3.32E-06	Down							
Unigene115274_All	293	0.00	0.00	0.00	0.00	0.00	0.00	0.00	1.85	0.00	null	null		null	null		null	null		null	null		1.09E+01	5.37E-04	Up	null	null		Unigene115274_All//gi|116059710|emb|CAL55417.1|//1.00E-13//Pirin (ISS) [Ostreococcus tauri]	Unigene115274_All//3.00E-13//AT3G59260| pirin, putative				GO:0005515//GO:0016020	nr +
Unigene115275_All	702	0.00	0.00	0.00	0.00	0.00	0.00	1.26	2.20	0.11	null	null		null	null		null	null		null	null		8.01E-01	9.44E-02		-3.48E+00	7.34E-05	Down	Unigene115275_All//gi|57999636|dbj|BAD88438.1|//5.00E-31//60S ribosomal protein L6 CgRPL6 [Chara globularis]	Unigene115275_All//3.00E-17//AT1G74060| 60S ribosomal protein L6 (RPL6B)			ko03010|Ribosome	GO:0005488//GO:0015934//GO:0016020//GO:0031981	nr -
Unigene115279_All	203	1.29	7.49	1.57	1.72	2.04	2.22	2.50	3.29	2.55	2.54E+00	9.21E-05	Up	2.88E-01	8.10E-01		2.45E-01	8.53E-01		3.73E-01	7.32E-01		3.98E-01	6.48E-01		2.97E-02	9.70E-01				Unigene115279_All//2.00E-07//TC168767				
Unigene115281_All	432	0.00	0.00	0.00	0.00	0.00	0.00	1.27	1.26	0.00	null	null		null	null		null	null		null	null		-1.67E-02	1.02E+00		-1.03E+01	2.81E-04	Down	Unigene115281_All//gi|226453529|gb|EEH50840.1|//3.00E-06//predicted protein [Micromonas pusilla CCMP1545]		Unigene115281_All//8.00E-12//ES794483				nr -
Unigene115283_All	580	1.08	0.00	0.46	0.17	0.12	0.31	0.87	4.68	0.89	-1.01E+01	7.92E-04	Down	-1.24E+00	2.13E-01		-5.33E-01	8.18E-01		8.59E-01	6.96E-01		2.42E+00	3.13E-09	Up	2.95E-02	9.70E-01								
Unigene115285_All	283	11.65	28.83	19.95	21.65	27.01	17.40	148.35	161.72	159.35	1.31E+00	1.16E-09	Up	7.76E-01	2.88E-03		3.19E-01	1.53E-01		-3.16E-01	2.24E-01		1.24E-01	1.12E-01		1.03E-01	1.84E-01				Unigene115285_All//2.00E-67//DW505711				
Unigene115289_All	276	14.03	54.89	49.01	14.08	15.72	21.11	6.74	11.36	29.73	1.97E+00	1.47E-30	Up	1.80E+00	1.19E-23	Up	1.59E-01	6.29E-01		5.84E-01	1.82E-02		7.53E-01	1.64E-02		2.14E+00	2.04E-23	Up			Unigene115289_All//2.00E-82//gi|48827491|gb|CO125881.1|CO125881				
Unigene115298_All	214	0.00	0.00	0.00	0.00	0.00	0.00	28.06	18.36	11.36	null	null		null	null		null	null		null	null		-6.12E-01	4.48E-03		-1.30E+00	3.72E-09	Down	Unigene115298_All//gi|255641066|gb|ACU20812.1|//3.00E-07//unknown [Glycine max]	Unigene115298_All//5.00E-05//AT5G06730| peroxidase, putative	Unigene115298_All//4.00E-40//TC166012				nr +
Unigene115302_All	685	0.00	0.00	0.00	0.00	0.00	0.00	0.86	2.93	0.23	null	null		null	null		null	null		null	null		1.76E+00	6.27E-05	Up	-1.89E+00	3.22E-02		Unigene115302_All//gi|224124808|ref|XP_002319427.1|//6.00E-80//cytochrome P450 [Populus trichocarpa] >gi|222857803|gb|EEE95350.1| cytochrome P450 [Populus trichocarpa]	Unigene115302_All//2.00E-75//AT4G39950| CYP79B2; electron carrier/ heme binding / iron ion binding / monooxygenase/ oxygen binding			ko00380|Tryptophan metabolism//ko00460|Cyanoamino acid metabolism//ko00966|Glucosinolate biosynthesis	GO:0002239//GO:0005506//GO:0006568//GO:0009536//GO:0009683//GO:0009700//GO:0016491//GO:0031224//GO:0052482	nr -
Unigene115305_All	626	0.00	0.00	0.00	0.00	0.00	0.00	0.00	4.54	0.00	null	null		null	null		null	null		null	null		1.21E+01	5.42E-20	Up	null	null		Unigene115305_All//gi|301100736|ref|XP_002899457.1|//1.00E-48//flavodoxin-like protein [Phytophthora infestans T30-4] >gi|262103765|gb|EEY61817.1| flavodoxin-like protein [Phytophthora infestans T30-4]	Unigene115305_All//9.00E-42//AT4G27270| quinone reductase family protein					nr +
Unigene115312_All	252	0.00	0.00	0.00	0.00	0.00	0.00	0.00	2.65	0.00	null	null		null	null		null	null		null	null		1.14E+01	7.64E-05	Up	null	null								ESTscan +
Unigene115313_All	364	0.00	0.00	0.00	0.00	0.00	0.00	1.86	4.48	0.00	null	null		null	null		null	null		null	null		1.27E+00	7.03E-03		-1.09E+01	3.56E-05	Down	Unigene115313_All//gi|33590374|gb|AAQ22726.1|//4.00E-17//40S ribosomal protein S25 [Glycine max]	Unigene115313_All//2.00E-19//AT4G34555| 40S ribosomal protein S25, putative			ko03010|Ribosome	GO:0009536//GO:0010467//GO:0015935//GO:0016020//GO:0031981	nr -
Unigene115317_All	492	0.00	0.00	0.00	0.61	0.14	0.00	9.71	5.27	0.40	null	null		null	null		-2.12E+00	1.66E-01		-9.25E+00	4.00E-02		-8.83E-01	3.32E-04		-4.58E+00	2.33E-28	Down	Unigene115317_All//gi|11994591|dbj|BAB02646.1|//9.00E-16//Ac transposase-like protein [Arabidopsis thaliana]		Unigene115317_All//2.00E-84//TC156042			GO:0003676	nr +
Unigene115318_All	241	0.00	0.00	0.00	0.00	0.14	0.00	2.11	0.35	0.00	null	null		null	null		7.16E+00	8.35E-01		null	null		-2.60E+00	2.05E-02		-1.10E+01	5.54E-04	Down							
Unigene115319_All	461	0.00	0.00	0.00	0.00	0.00	0.00	4.31	10.34	0.26	null	null		null	null		null	null		null	null		1.26E+00	7.63E-07	Up	-4.06E+00	3.04E-11	Down	Unigene115319_All//gi|238014838|gb|ACR38454.1|//8.00E-15//unknown [Zea mays]		Unigene115319_All//2.00E-16//TC152959				nr +
Unigene11532_All	1972	13.48	28.83	20.68	20.21	23.75	27.73	13.29	14.18	9.74	1.10E+00	7.67E-48	Up	6.18E-01	6.58E-13		2.33E-01	2.08E-03		4.57E-01	3.48E-11		9.31E-02	3.95E-01		-4.49E-01	1.18E-06		Unigene11532_All//gi|18409750|ref|NP_566977.1|//0.00E+00//SRPK4 (Ser/Arg-rich protein kinase 4); kinase/ protein kinase [Arabidopsis thaliana]	Unigene11532_All//0.00E+00//AT3G53030| SRPK4 (Ser/Arg-rich protein kinase 4); kinase/ protein kinase	Unigene11532_All//0.00E+00//gi|78336218|gb|DT556492.1|DT556492			GO:0004672//GO:0006464//GO:0006950//GO:0032559	nr +
Unigene115327_All	378	118.34	30.96	43.11	203.34	162.06	196.42	32.55	21.56	25.08	-1.93E+00	1.25E-87	Down	-1.46E+00	1.04E-56	Down	-3.27E-01	1.64E-09		-4.99E-02	5.12E-01		-5.94E-01	3.37E-05		-3.76E-01	7.76E-03		Unigene115327_All//gi|297842159|ref|XP_002888961.1|//1.00E-47//60S ribosomal protein L6 [Arabidopsis lyrata subsp. lyrata] >gi|297334802|gb|EFH65220.1| 60S ribosomal protein L6 [Arabidopsis lyrata subsp. lyrata]	Unigene115327_All//6.00E-40//AT1G74050| 60S ribosomal protein L6 (RPL6C)	Unigene115327_All//0.00E+00//gi|13247383|gb|BF270238.2|BF270238		ko03010|Ribosome	GO:0005198//GO:0005488//GO:0009536//GO:0010467//GO:0015934//GO:0016020//GO:0031981	nr +
Unigene115333_All	371	309.98	101.61	174.25	475.18	407.28	465.15	362.52	156.26	243.86	-1.61E+00	1.02E-173	Down	-8.31E-01	6.19E-59		-2.22E-01	2.26E-10		-3.08E-02	5.50E-01		-1.21E+00	4.61E-162	Down	-5.72E-01	1.03E-46		Unigene115333_All//gi|52783264|sp|Q9FUL4.1|RL24_PRUAV//2.00E-46//RecName: Full=60S ribosomal protein L24 >gi|10180025|gb|AAG13986.1|AF298827_1 60S ribosomal protein L24 [Prunus avium]	Unigene115333_All//6.00E-44//AT3G53020| STV1 (SHORT VALVE1); structural constituent of ribosome	Unigene115333_All//1.00E-179//gi|109866646|gb|DW495622.1|DW495622		ko03010|Ribosome	GO:0005198//GO:0010467//GO:0030529//GO:0043231	nr +
Unigene115334_All	609	0.00	0.00	0.00	0.33	0.00	0.00	0.42	2.13	2.09	null	null		null	null		-8.35E+00	8.46E-02		-8.35E+00	1.34E-01		2.35E+00	1.25E-04	Up	2.33E+00	1.17E-04	Up	Unigene115334_All//gi|297849980|ref|XP_002892871.1|//3.00E-71//ATPDR12/PDR12 [Arabidopsis lyrata subsp. lyrata] >gi|297338713|gb|EFH69130.1| ATPDR12/PDR12 [Arabidopsis lyrata subsp. lyrata]	Unigene115334_All//1.00E-70//AT1G15520| PDR12 (PLEIOTROPIC DRUG RESISTANCE 12); ATPase, coupled to transmembrane movement of substances	Unigene115334_All//0.00E+00//DT465573		ko03040|Spliceosome//ko02010|ABC transporters	GO:0009725//GO:0015893//GO:0016020//GO:0022891//GO:0030001//GO:0032559//GO:0042626//GO:0046864	nr -
Unigene115339_All	527	0.00	0.00	0.00	0.00	0.00	0.00	34.66	14.91	16.03	null	null		null	null		null	null		null	null		-1.22E+00	9.33E-23	Down	-1.11E+00	1.88E-20	Down			Unigene115339_All//1.00E-14//TC142576				
Unigene11534_All	2017	1.69	3.04	1.56	2.17	3.11	2.71	0.69	1.91	1.17	8.50E-01	3.94E-04		-1.14E-01	7.40E-01		5.16E-01	2.69E-02		3.18E-01	2.53E-01		1.46E+00	6.71E-07	Up	7.53E-01	3.61E-02		Unigene11534_All//gi|18409750|ref|NP_566977.1|//0.00E+00//SRPK4 (Ser/Arg-rich protein kinase 4); kinase/ protein kinase [Arabidopsis thaliana]	Unigene11534_All//0.00E+00//AT3G53030| SRPK4 (Ser/Arg-rich protein kinase 4); kinase/ protein kinase	Unigene11534_All//0.00E+00//TC175776			GO:0004672//GO:0006464//GO:0006950//GO:0032559	nr +
Unigene115350_All	343	0.00	0.00	0.00	0.00	0.00	0.00	0.62	5.00	1.28	null	null		null	null		null	null		null	null		3.02E+00	1.78E-07	Up	1.05E+00	2.98E-01		Unigene115350_All//gi|126566038|gb|ABO20848.1|//1.00E-20//cytochrome P450-like TBP protein [Lilium longiflorum]		Unigene115350_All//2.00E-36//gi|84158108|gb|DN827155.1|DN827155				nr +
Unigene115366_All	422	0.12	0.00	0.00	0.35	0.08	0.21	0.20	1.78	0.19	-6.95E+00	6.16E-01		-6.95E+00	6.29E-01		-2.12E+00	3.87E-01		-7.27E-01	7.16E-01		3.15E+00	9.54E-04	Up	-8.60E-02	9.73E-01								
Unigene115369_All	317	0.00	0.00	0.00	0.00	0.11	0.00	4.93	18.72	5.91	null	null		null	null		6.76E+00	8.35E-01		null	null		1.92E+00	1.07E-14	Up	2.59E-01	5.61E-01		Unigene115369_All//gi|75311691|sp|Q9LY51.1|PLCD7_ARATH//1.00E-09//RecName: Full=Phosphoinositide phospholipase C 7; AltName: Full=Phosphoinositide phospholipase PLC7; Short=PI-PLC7; Short=AtPLC7; Short=AtPLC10 >gi|7573489|emb|CAB87848.1| phosphoinositide-specific phospholipase C-like protein [Arabidopsis thaliana]	Unigene115369_All//6.00E-11//AT3G55940| phosphoinositide-specific phospholipase C, putative	Unigene115369_All//2.00E-24//TC146670		ko04070|Phosphatidylinositol signaling system//ko00562|Inositol phosphate metabolism		nr -
Unigene115379_All	589	0.36	0.52	1.18	0.76	0.29	0.31	0.36	6.24	0.95	5.39E-01	6.72E-01		1.73E+00	7.68E-02		-1.38E+00	2.16E-01		-1.31E+00	2.73E-01		4.12E+00	1.01E-19	Up	1.40E+00	1.12E-01		Unigene115379_All//gi|18652398|gb|AAL77102.1|AF472608_1//5.00E-54//carbohydrate oxidase [Lactuca sativa]	Unigene115379_All//8.00E-38//AT4G20800| FAD-binding domain-containing protein				GO:0016491	nr -

Unigene115394_All	403	0.00	0.00	0.00	0.12	0.34	0.67	0.73	3.11	0.20	null	null		null	null		1.47E+00	6.19E-01		2.44E+00	2.22E-01		2.08E+00	5.41E-04	Up	-1.89E+00	1.68E-01		Unigene115394_All//gi|15230158|ref|NP_188497.1|//9.00E-10//ankyrin repeat family protein [Arabidopsis thaliana]	Unigene115394_All//5.00E-11//AT5G04730| unknown protein	Unigene115394_All//6.00E-25//TC178375				nr +
Unigene115397_All	260	0.00	0.00	0.00	0.00	0.00	0.00	11.55	3.54	0.61	null	null		null	null		null	null		null	null		-1.71E+00	9.31E-07	Down	-4.24E+00	3.58E-17	Down			Unigene115397_All//3.00E-10//TC146753				
Unigene115408_All	327	0.00	0.00	0.00	0.00	0.00	0.00	0.52	5.50	0.12	null	null		null	null		null	null		null	null		3.41E+00	1.21E-08	Up	-2.09E+00	3.14E-01								ESTscan +
Unigene115409_All	316	0.00	0.00	0.00	0.00	0.00	0.00	0.00	1.72	0.00	null	null		null	null		null	null		null	null		1.07E+01	5.37E-04	Up	null	null		Unigene115409_All//gi|220966698|gb|ACL97363.1|//3.00E-38//elongation factor-1 alpha [Ignatius tetrasporus]	Unigene115409_All//2.00E-36//AT5G60390| elongation factor 1-alpha / EF-1-alpha	Unigene115409_All//6.00E-49//CD809343			GO:0006412//GO:0008135//GO:0017111//GO:0032561//GO:0044424	nr +
Unigene115417_All	441	0.00	0.00	0.00	0.00	0.00	0.00	1.05	4.55	0.00	null	null		null	null		null	null		null	null		2.11E+00	4.88E-06	Up	-1.00E+01	1.09E-03		Unigene115417_All//gi|1527001|gb|AAB07724.1|//1.00E-20//Pn47p [Ipomoea nil]	Unigene115417_All//1.00E-20//AT4G18550| lipase class 3 family protein					nr +
Unigene115418_All	679	0.00	0.00	0.00	0.00	0.00	0.00	1.87	4.74	0.00	null	null		null	null		null	null		null	null		1.34E+00	2.74E-05	Up	-1.09E+01	1.99E-09	Down	Unigene115418_All//gi|116056034|emb|CAL58567.1|//3.00E-28//Nucleolar GTPase/ATPase p130 (ISS) [Ostreococcus tauri]						nr -
Unigene11542_All	5013	1.80	0.87	1.53	17.34	16.44	13.53	11.69	11.35	12.52	-1.05E+00	1.07E-07	Down	-2.31E-01	2.82E-01		-7.71E-02	2.33E-01		-3.58E-01	2.44E-11		-4.29E-02	6.08E-01		9.86E-02	1.24E-01		Unigene11542_All//gi|28268680|dbj|BAC56865.1|//0.00E+00//respiratory burst oxidase homolog [Nicotiana benthamiana]	Unigene11542_All//0.00E+00//AT5G51060| RHD2 (ROOT HAIR DEFECTIVE 2); NAD(P)H oxidase	Unigene11542_All//0.00E+00//gi|164283610|gb|ES818781.1|ES818781		ko04626|Plant-pathogen interaction	GO:0006950//GO:0008152//GO:0009913//GO:0010033//GO:0016021//GO:0016209//GO:0030554//GO:0046914//GO:0050664	nr -
Unigene115422_All	280	0.00	0.00	0.00	0.00	0.00	0.00	0.00	2.84	0.28	null	null		null	null		null	null		null	null		1.15E+01	1.07E-05	Up	8.15E+00	4.05E-01								ESTscan +
Unigene115423_All	352	0.00	0.00	0.00	0.00	0.00	0.00	3.48	14.60	0.00	null	null		null	null		null	null		null	null		2.07E+00	5.06E-14	Up	-1.18E+01	4.03E-09	Down							
Unigene115426_All	236	2.44	4.08	1.13	2.32	2.48	3.25	3.76	9.74	7.93	7.42E-01	2.94E-01		-1.11E+00	2.79E-01		9.54E-02	9.51E-01		4.87E-01	5.91E-01		1.37E+00	4.17E-04	Up	1.08E+00	9.46E-03				Unigene115426_All//1.00E-30//TC179380				
Unigene115442_All	326	20.06	86.57	76.44	60.05	52.81	63.73	29.96	39.23	21.87	2.11E+00	1.15E-61	Up	1.93E+00	2.64E-47	Up	-1.85E-01	1.65E-01		8.57E-02	5.81E-01		3.89E-01	6.35E-03		-4.54E-01	4.40E-03		Unigene115442_All//gi|224136362|ref|XP_002326841.1|//1.00E-06//predicted protein [Populus trichocarpa] >gi|118485001|gb|ABK94365.1| unknown [Populus trichocarpa] >gi|222835156|gb|EEE73591.1| predicted protein [Populus trichocarpa]		Unigene115442_All//5.00E-145//gi|164240695|gb|ES802406.1|ES802406				nr -
Unigene115448_All	343	1.98	5.91	3.73	2.47	3.41	2.37	2.84	8.65	4.65	1.58E+00	1.08E-03		9.10E-01	1.46E-01		4.67E-01	5.14E-01		-5.89E-02	9.44E-01		1.61E+00	3.33E-06	Up	7.13E-01	1.15E-01		Unigene115448_All//gi|297808579|ref|XP_002872173.1|//5.00E-15//zinc finger family protein [Arabidopsis lyrata subsp. lyrata] >gi|297318010|gb|EFH48432.1| zinc finger family protein [Arabidopsis lyrata subsp. lyrata]	Unigene115448_All//7.00E-16//AT5G25560| zinc finger (C3HC4-type RING finger) family protein	Unigene115448_All//1.00E-41//EX167266		ko04120|Ubiquitin mediated proteolysis	GO:0046914	nr -
Unigene11545_All	2699	1.22	0.62	0.45	1.46	1.57	1.82	2.33	2.06	2.61	-9.79E-01	4.27E-03		-1.43E+00	1.22E-04	Down	1.06E-01	7.90E-01		3.23E-01	2.79E-01		-1.81E-01	4.45E-01		1.63E-01	4.47E-01		Unigene11545_All//gi|268044007|gb|ACY92092.1|//0.00E+00//HOS1 [Citrus trifoliata]	Unigene11545_All//1.00E-178//AT2G39810| HOS1; ubiquitin-protein ligase	Unigene11545_All//0.00E+00//CO096048			GO:0006350//GO:0006950//GO:0019787//GO:0032446//GO:0043231	nr -
Unigene115453_All	208	0.00	0.00	0.00	0.00	0.00	0.00	3.66	1.41	0.19	null	null		null	null		null	null		null	null		-1.38E+00	6.73E-02		-4.26E+00	8.65E-05	Down							
Unigene115455_All	526	0.00	0.00	0.00	1.04	0.39	0.09	0.64	13.75	1.51	null	null		null	null		-1.41E+00	1.53E-01		-3.60E+00	8.98E-03		4.42E+00	4.15E-40	Up	1.24E+00	7.69E-02		Unigene115455_All//gi|297242517|gb|ADI24922.1|//8.00E-06//pirin [Carica papaya]	Unigene115455_All//7.00E-07//AT1G50590| pirin, putative	Unigene115455_All//3.00E-18//DW507929				nr -
Unigene115456_All	1145	0.00	0.00	0.00	0.00	0.00	0.00	0.11	2.92	0.07	null	null		null	null		null	null		null	null		4.72E+00	1.69E-19	Up	-6.71E-01	7.20E-01		Unigene115456_All//gi|226495425|ref|NP_001148706.1|//6.00E-67//cysteine protease 1 [Zea mays] >gi|195621544|gb|ACG32602.1| cysteine protease 1 precursor [Zea mays]	Unigene115456_All//3.00E-64//AT5G43060| cysteine proteinase, putative / thiol protease, putative				GO:0016787	nr -
Unigene115457_All	713	0.00	0.00	0.00	0.00	0.00	0.00	1.25	3.11	0.00	null	null		null	null		null	null		null	null		1.32E+00	8.45E-04	Up	-1.03E+01	1.10E-06	Down							ESTscan -
Unigene115458_All	599	0.00	0.00	0.00	0.00	0.00	0.00	0.00	4.19	0.00	null	null		null	null		null	null		null	null		1.20E+01	1.22E-17	Up	null	null		Unigene115458_All//gi|226508392|ref|NP_001151699.1|//3.00E-07//LOC100285335 [Zea mays] >gi|195649099|gb|ACG44017.1| ABA induced plasma membrane protein PM 19 [Zea mays]	Unigene115458_All//1.00E-06//AT5G18970| AWPM-19-like membrane family protein					nr -
Unigene115460_All	636	1.07	0.00	0.67	0.94	1.14	2.41	1.40	1.05	0.13	-1.01E+01	4.10E-04	Down	-6.75E-01	4.41E-01		2.75E-01	8.01E-01		1.36E+00	1.43E-02		-4.09E-01	5.62E-01		-3.48E+00	7.34E-05	Down	Unigene115460_All//gi|18401257|ref|NP_566559.1|//3.00E-20//APUM24 (Arabidopsis Pumilio 24); RNA binding / binding [Arabidopsis thaliana] >gi|15010674|gb|AAK73996.1| AT3g16810/K20I9_3 [Arabidopsis thaliana]	Unigene115460_All//2.00E-21//AT3G16810| APUM24 (Arabidopsis Pumilio 24); RNA binding / binding	Unigene115460_All//2.00E-170//CO097303			GO:0031981	nr +
Unigene11547_All	1120	2.85	0.54	1.47	2.36	1.72	2.74	2.45	1.79	1.92	-2.39E+00	9.11E-09	Down	-9.51E-01	9.11E-03		-4.53E-01	2.52E-01		2.18E-01	6.14E-01		-4.54E-01	1.98E-01		-3.53E-01	3.02E-01		Unigene11547_All//gi|15218495|ref|NP_177391.1|//9.00E-90//ATEXO70D1 (exocyst subunit EXO70 family protein D1); protein binding [Arabidopsis thaliana]	Unigene11547_All//3.00E-70//AT1G72470| ATEXO70D1 (exocyst subunit EXO70 family protein D1); protein binding	Unigene11547_All//0.00E+00//ES830101			GO:0016192//GO:0043231//GO:0043234//GO:0044444	nr -
Unigene115471_All	374	0.00	0.00	0.00	0.00	0.00	0.00	1.70	6.26	1.28	null	null		null	null		null	null		null	null		1.88E+00	4.35E-06	Up	-4.08E-01	6.19E-01				Unigene115471_All//2.00E-15//TC152786				
Unigene115474_All	278	0.00	0.00	0.00	0.00	0.00	0.00	2.28	2.10	0.14	null	null		null	null		null	null		null	null		-1.16E-01	8.88E-01		-3.99E+00	5.82E-04	Down			Unigene115474_All//1.00E-15//TC144001				
Unigene115477_All	254	0.00	0.00	0.00	0.00	0.14	0.00	46.94	33.07	103.83	null	null		null	null		7.08E+00	8.35E-01		null	null		-5.05E-01	5.48E-04		1.15E+00	3.07E-30	Up	Unigene115477_All//gi|147791048|emb|CAN65763.1|//1.00E-06//hypothetical protein [Vitis vinifera]		Unigene115477_All//3.00E-47//gi|73862963|gb|DT465702.1|DT465702				nr -
Unigene115479_All	414	0.00	0.00	0.00	0.24	0.17	0.00	0.10	1.41	2.79	null	null		null	null		-5.32E-01	8.18E-01		-7.91E+00	4.03E-01		3.79E+00	2.07E-03		4.77E+00	3.27E-07	Up							
Unigene115496_All	599	0.00	0.00	0.00	0.00	0.00	0.00	0.35	2.02	0.60	null	null		null	null		null	null		null	null		2.52E+00	1.15E-04	Up	7.62E-01	5.00E-01		Unigene115496_All//gi|115474815|ref|NP_001061004.1|//9.00E-29//Os08g0152500 [Oryza sativa Japonica Group] >gi|113622973|dbj|BAF22918.1| Os08g0152500 [Oryza sativa Japonica Group]	Unigene115496_All//1.00E-43//AT2G39170| unknown protein	Unigene115496_All//8.00E-19//TC170512				nr +
Unigene1155_All	734	0.29	0.00	0.15	0.61	1.13	1.78	4.38	1.08	2.66	-8.16E+00	1.22E-01		-9.74E-01	6.21E-01		8.82E-01	2.93E-01		1.55E+00	1.29E-02		-2.02E+00	8.50E-09	Down	-7.19E-01	1.56E-02		Unigene1155_All//gi|15912193|gb|AAL08230.1|//1.00E-113//AT4g22990/F7H19_170 [Arabidopsis thaliana]	Unigene1155_All//1.00E-112//AT4G22990| SPX (SYG1/Pho81/XPR1) domain-containing protein	Unigene1155_All//9.00E-121//CA994138				nr -
Unigene115509_All	452	0.00	0.56	0.47	0.00	0.08	0.00	0.37	2.22	1.23	9.13E+00	7.88E-02		8.88E+00	1.42E-01		6.25E+00	8.35E-01		null	null		2.57E+00	4.79E-04	Up	1.72E+00	5.92E-02				Unigene115509_All//4.00E-14//DR459422				
Unigene11551_All	1634	1.79	0.16	0.52	2.10	2.07	1.27	2.10	0.61	0.73	-3.53E+00	1.17E-11	Down	-1.78E+00	1.71E-05	Down	-2.64E-02	9.43E-01		-7.26E-01	2.91E-02		-1.77E+00	5.77E-08	Down	-1.52E+00	8.31E-07	Down	Unigene11551_All//gi|115469330|ref|NP_001058264.1|//1.00E-149//Os06g0659400 [Oryza sativa Japonica Group] >gi|51536173|dbj|BAD38346.1| lustrin A-like [Oryza sativa Japonica Group] >gi|52077362|dbj|BAD46402.1| lustrin A-like [Oryza sativa Japonica Group] >gi|113596304|dbj|BAF20178.1| Os06g0659400 [Oryza sativa Japonica Group]	Unigene11551_All//2.00E-167//AT5G20590| unknown protein	Unigene11551_All//0.00E+00//TC152473				nr -
Unigene115528_All	504	0.00	0.00	0.00	0.20	0.00	0.09	2.27	0.00	0.16	null	null		null	null		-7.63E+00	3.15E-01		-1.14E+00	6.78E-01		-1.11E+01	3.88E-08	Down	-3.84E+00	1.74E-06	Down	Unigene115528_All//gi|15233459|ref|NP_194643.1|//7.00E-53//MATE efflux protein-related [Arabidopsis thaliana]	Unigene115528_All//1.00E-42//AT4G29140| MATE efflux protein-related	Unigene115528_All//3.00E-104//TC170491				nr +
Unigene11553_All	596	1.14	4.51	2.59	2.26	1.79	4.17	60.94	57.22	46.26	1.98E+00	4.79E-06	Up	1.18E+00	3.71E-02		-3.33E-01	5.81E-01		8.85E-01	2.99E-02		-9.07E-02	3.46E-01		-3.98E-01	2.90E-07		Unigene11553_All//gi|225437164|ref|XP_002274787.1|//1.00E-77//PREDICTED: similar to type IIB calcium ATPase [Vitis vinifera]	Unigene11553_All//9.00E-64//AT3G63380| calcium-transporting ATPase, plasma membrane-type, putative / Ca(2+)-ATPase, putative (ACA12)	Unigene11553_All//0.00E+00//gi|78342403|gb|DT562677.1|DT562677			GO:0009206//GO:0019829//GO:0031224//GO:0032559//GO:0046873//GO:0070838	nr +
Unigene115532_All	437	0.00	0.00	0.00	0.00	0.00	0.00	0.10	1.82	0.18	null	null		null	null		null	null		null	null		4.23E+00	1.05E-04	Up	9.13E-01	7.51E-01		Unigene115532_All//gi|227908932|gb|ACO50134.2|//7.00E-57//elongation factor-like [Petalomonas cantuscygni]	Unigene115532_All//5.00E-18//AT5G60390| elongation factor 1-alpha / EF-1-alpha					nr -
Unigene115535_All	376	0.00	0.00	0.00	0.00	0.00	0.00	0.00	3.67	0.11	null	null		null	null		null	null		null	null		1.18E+01	9.64E-10	Up	6.73E+00	6.47E-01								ESTscan +
Unigene115539_All	293	0.00	0.00	0.00	0.00	0.00	0.00	2.31	2.43	0.14	null	null		null	null		null	null		null	null		7.08E-02	9.42E-01		-4.09E+00	3.10E-04	Down	Unigene115539_All//gi|8928433|sp|Q9ZSW1.1|TBB1_CYAPA//7.00E-34//RecName: Full=Tubulin beta-1 chain; AltName: Full=Beta-1-tubulin >gi|4139172|gb|AAD03712.1| beta 1 tubulin [Cyanophora paradoxa]	Unigene115539_All//1.00E-30//AT1G75780| TUB1; GTP binding / GTPase/ structural molecule	Unigene115539_All//1.00E-19//TC141009		ko04145|Phagosome		nr -
Unigene11554_All	4024	3.39	9.29	4.10	3.39	4.63	11.28	68.93	72.43	57.19	1.45E+00	8.84E-49	Up	2.74E-01	6.63E-02		4.49E-01	2.12E-04		1.73E+00	1.19E-81	Up	7.15E-02	1.24E-02		-2.69E-01	3.90E-24		Unigene11554_All//gi|225437164|ref|XP_002274787.1|//0.00E+00//PREDICTED: similar to type IIB calcium ATPase [Vitis vinifera]	Unigene11554_All//0.00E+00//AT3G63380| calcium-transporting ATPase, plasma membrane-type, putative / Ca(2+)-ATPase, putative (ACA12)	Unigene11554_All//0.00E+00//TC168651			GO:0005515//GO:0009206//GO:0019829//GO:0031224//GO:0032559//GO:0046873//GO:0070838	nr +
Unigene11555_All	2380	6.13	20.37	8.17	10.88	17.13	15.94	10.34	11.63	11.23	1.73E+00	3.70E-81	Up	4.13E-01	1.54E-03		6.54E-01	3.46E-17		5.51E-01	6.46E-11		1.69E-01	9.14E-02		1.20E-01	2.46E-01		Unigene11555_All//gi|115473603|ref|NP_001060400.1|//1.00E-115//Os07g0637200 [Oryza sativa Japonica Group] >gi|113611936|dbj|BAF22314.1| Os07g0637200 [Oryza sativa Japonica Group]	Unigene11555_All//8.00E-153//AT3G62200| FUNCTIONS IN: molecular_function unknown; INVOLVED IN: biological_process unknown; LOCATED IN: cellular_component unknown; CONTAINS InterPro DOMAIN/s: Protein of unknown function DUF537 (InterPro:IPR007491); BEST Arabidopsis thaliana protein match is: EDA32 (embryo sac development arrest 32) (TAIR:AT3G62210.1); Has 3380 Blast hits to 2820 proteins in 272 species: Archae - 0; Bacteria - 93; Metazoa - 1555; Fungi - 720; Plants - 473; Viruses - 42; Other Eukaryotes - 497 (source: NCBI BLink).	Unigene11555_All//0.00E+00//TC161332			GO:0000741	nr +
Unigene115551_All	625	0.00	0.00	0.00	0.00	0.00	0.00	1.62	2.74	0.00	null	null		null	null		null	null		null	null		7.56E-01	9.34E-02		-1.07E+01	1.35E-07	Down	Unigene115551_All//gi|166343843|gb|ABY86664.1|//1.00E-105//beta-tubulin 18 [Gossypium hirsutum]	Unigene115551_All//2.00E-86//AT5G23860| TUB8; structural constituent of cytoskeleton	Unigene115551_All//2.00E-41//TC148779		ko04145|Phagosome		nr -
Unigene115554_All	284	90.09	30.33	21.00	62.27	59.41	43.41	70.72	17.66	37.03	-1.57E+00	8.69E-38	Down	-2.10E+00	5.13E-54	Down	-6.77E-02	6.76E-01		-5.20E-01	5.05E-05		-2.00E+00	1.21E-50	Down	-9.33E-01	5.48E-17		Unigene115554_All//gi|166343845|gb|ABY86665.1|//1.00E-26//beta-tubulin 19 [Gossypium hirsutum]	Unigene115554_All//3.00E-26//AT5G62700| TUB3; GTP binding / GTPase/ structural molecule	Unigene115554_All//4.00E-124//gi|21094044|gb|BQ406369.1|BQ406369		ko04145|Phagosome		nr -
Unigene115555_All	308	0.00	0.00	0.00	0.00	0.00	0.00	0.82	3.94	0.00	null	null		null	null		null	null		null	null		2.26E+00	3.35E-04	Up	-9.69E+00	3.04E-02		Unigene115555_All//gi|13359451|dbj|BAB33421.1|//6.00E-06//putative senescence-associated protein [Pisum sativum]		Unigene115555_All//4.00E-07//TC143156				nr -
Unigene115558_All	415	0.00	0.00	0.00	0.00	0.00	0.00	0.61	3.32	1.44	null	null		null	null		null	null		null	null		2.44E+00	4.59E-05	Up	1.24E+00	1.39E-01				Unigene115558_All//5.00E-13//DT455935				
Unigene115569_All	222	0.00	12.10	0.72	1.57	5.74	0.20	1.71	5.27	8.08	1.36E+01	2.81E-15	Up	9.49E+00	2.39E-01		1.87E+00	3.47E-03		-2.95E+00	8.31E-02		1.62E+00	6.24E-03		2.24E+00	5.44E-06	Up			Unigene115569_All//2.00E-29//EX167763		ko00562|Inositol phosphate metabolism//ko00053|Ascorbate and aldarate metabolism		kegg +
Unigene115570_All	523	0.00	0.00	0.00	0.00	0.00	0.00	0.97	1.44	0.00	null	null		null	null		null	null		null	null		5.68E-01	4.47E-01		-9.92E+00	5.54E-04	Down	Unigene115570_All//gi|12000413|gb|AAG40108.1|//1.00E-59//elongation factor 2 [Porphyra yezoensis]	Unigene115570_All//3.00E-54//AT1G56070| LOS1; copper ion binding / translation elongation factor/ translation factor, nucleic acid binding					nr -

Unigene115574_All	530	1.28	0.29	1.31	2.54	0.91	1.45	7.58	3.71	1.58	-2.16E+00	2.84E-02		2.54E-02	9.96E-01		-1.48E+00	8.15E-03		-8.09E-01	1.73E-01		-1.03E+00	1.66E-04	Down	-2.26E+00	7.89E-13	Down	Unigene115574_All//gi|297824001|ref|XP_002879883.1|//2.00E-22//tRNA-binding region domain-containing protein [Arabidopsis lyrata subsp. lyrata] >gi|297325722|gb|EFH56142.1| tRNA-binding region domain-containing protein [Arabidopsis lyrata subsp. lyrata]	Unigene115574_All//8.00E-23//AT2G40660| tRNA-binding region domain-containing protein	Unigene115574_All//0.00E+00//gi|21097965|gb|BQ410278.1|BQ410278		ko00450|Selenoamino acid metabolism//ko00970|Aminoacyl-tRNA biosynthesis		nr +
Unigene115576_All	295	0.00	0.17	0.00	0.84	0.23	0.00	0.43	0.57	5.13	7.42E+00	6.19E-01		null	null		-1.85E+00	2.75E-01		-9.72E+00	7.35E-02		3.98E-01	8.15E-01		3.58E+00	7.96E-08	Up			Unigene115576_All//4.00E-90//DR461288				
Unigene115577_All	1161	0.00	0.00	0.05	0.04	0.03	0.12	3.02	1.37	1.44	null	null		5.52E+00	6.39E-01		-5.31E-01	8.27E-01		1.44E+00	6.39E-01		-1.14E+00	1.27E-04	Down	-1.07E+00	2.11E-04	Down	Unigene115577_All//gi|3287835|sp|Q43714.1|DCS3_GOSAR//1.00E-108//RecName: Full=(+)-delta-cadinene synthase isozyme A; Short=D-cadinene synthase A >gi|1002523|gb|AAB41259.1| (+)-delta-cadinene synthase isozyme A [Gossypium arboreum] >gi|1217956|emb|CAA65289.1| (+)-delta-cadinene synthase [Gossypium arboreum]	Unigene115577_All//7.00E-67//AT5G23960| TPS21 (TERPENE SYNTHASE 21); (-)-E-beta-caryophyllene synthase/ alpha-humulene synthase	Unigene115577_All//0.00E+00//TC142314		ko00904|Diterpenoid biosynthesis		nr -
Unigene115578_All	431	8.25	9.29	10.38	2.54	2.40	4.19	2.75	7.47	7.12	1.70E-01	6.21E-01		3.30E-01	2.89E-01		-8.52E-02	8.79E-01		7.21E-01	1.51E-01		1.44E+00	8.47E-06	Up	1.37E+00	2.17E-05	Up	Unigene115578_All//gi|85068668|gb|ABC69414.1|//7.00E-23//CYP72A57 [Nicotiana tabacum]	Unigene115578_All//2.00E-20//AT3G14690| CYP72A15; electron carrier/ heme binding / iron ion binding / monooxygenase/ oxygen binding	Unigene115578_All//9.00E-166//TC139184		ko00908|Zeatin biosynthesis//ko00591|Linoleic acid metabolism		nr +
Unigene11558_All	1662	16.09	7.99	8.30	19.69	19.81	18.80	25.69	11.42	12.63	-1.01E+00	3.62E-20	Down	-9.55E-01	1.10E-17		8.40E-03	9.58E-01		-6.65E-02	5.92E-01		-1.17E+00	4.77E-49	Down	-1.02E+00	7.81E-41	Down	Unigene11558_All//gi|297799222|ref|XP_002867495.1|//1.00E-150//band 7 family protein [Arabidopsis lyrata subsp. lyrata] >gi|297313331|gb|EFH43754.1| band 7 family protein [Arabidopsis lyrata subsp. lyrata]	Unigene11558_All//1.00E-144//AT4G27585| band 7 family protein	Unigene11558_All//0.00E+00//gi|78323484|gb|DT543758.1|DT543758				nr +
Unigene115580_All	241	0.00	0.00	0.00	0.00	0.00	0.00	272.10	40.06	3.97	null	null		null	null		null	null		null	null		-2.76E+00	2.32E-241	Down	-6.10E+00	0.00E+00	Down			Unigene115580_All//1.00E-12//TC142576				
Unigene115581_All	688	0.00	0.00	0.00	0.00	0.00	0.00	0.00	0.97	0.46	null	null		null	null		null	null		null	null		9.92E+00	7.64E-05	Up	8.86E+00	1.34E-02		Unigene115581_All//gi|296081204|emb|CBI18230.3|//3.00E-08//unnamed protein product [Vitis vinifera]		Unigene115581_All//0.00E+00//ES831270				nr -
Unigene115582_All	484	0.00	0.00	0.00	0.00	0.00	0.00	3.76	6.30	0.00	null	null		null	null		null	null		null	null		7.47E-01	1.92E-02		-1.19E+01	2.03E-13	Down	Unigene115582_All//gi|226503551|ref|NP_001148898.1|//2.00E-30//actin-depolymerizing factor [Zea mays] >gi|195623028|gb|ACG33344.1| actin-depolymerizing factor [Zea mays]	Unigene115582_All//2.00E-28//AT5G59890| ADF4 (ACTIN DEPOLYMERIZING FACTOR 4); actin binding					nr -
Unigene115588_All	339	0.00	0.00	0.00	0.00	0.00	0.00	0.00	1.85	0.00	null	null		null	null		null	null		null	null		1.09E+01	1.47E-04	Up	null	null		Unigene115588_All//gi|148524155|gb|ABQ81936.1|//5.00E-24//elongation factor 1-alpha [Chara australis]	Unigene115588_All//4.00E-21//AT5G60390| elongation factor 1-alpha / EF-1-alpha	Unigene115588_All//1.00E-07//CD809343				nr +
Unigene115595_All	305	0.34	0.00	0.35	0.00	0.00	0.00	0.00	3.84	0.00	-8.42E+00	3.75E-01		2.54E-02	1.00E+00		null	null		null	null		1.19E+01	2.73E-08	Up	null	null		Unigene115595_All//gi|41393668|gb|AAS02074.1|//3.00E-48//auxin and ethylene responsive GH3-like protein [Capsicum chinense]	Unigene115595_All//5.00E-44//AT1G59500| GH3.4; indole-3-acetic acid amido synthetase	Unigene115595_All//2.00E-85//ES831098				nr +
Unigene115598_All	444	61.75	26.48	46.17	48.80	53.20	44.35	13.05	5.18	6.19	-1.22E+00	8.06E-28	Down	-4.19E-01	9.22E-05		1.25E-01	3.40E-01		-1.38E-01	3.19E-01		-1.33E+00	8.01E-09	Down	-1.08E+00	9.33E-07	Down			Unigene115598_All//8.00E-83//gi|11206634|gb|BF275564.1|BF275564				
Unigene1156_All	1618	76.96	107.70	86.36	78.32	75.47	91.16	39.28	114.82	77.73	4.85E-01	1.20E-35		1.66E-01	2.88E-04		-5.36E-02	3.27E-01		2.19E-01	9.24E-08		1.55E+00	0.00E+00	Up	9.85E-01	2.02E-110		Unigene1156_All//gi|18416049|ref|NP_567674.1|//0.00E+00//SPX (SYG1/Pho81/XPR1) domain-containing protein [Arabidopsis thaliana]	Unigene1156_All//0.00E+00//AT4G22990| SPX (SYG1/Pho81/XPR1) domain-containing protein	Unigene1156_All//0.00E+00//TC138350			GO:0006810//GO:0031090	nr -
Unigene115606_All	436	0.00	0.00	0.00	0.00	0.00	0.00	0.39	4.03	0.00	null	null		null	null		null	null		null	null		3.38E+00	2.17E-08	Up	-8.60E+00	1.08E-01		Unigene115606_All//gi|226347409|gb|ACO50115.1|//5.00E-63//elongation factor 1 alpha [Peranema trichophorum]	Unigene115606_All//9.00E-61//AT5G60390| elongation factor 1-alpha / EF-1-alpha	Unigene115606_All//1.00E-19//CD809343				nr +
Unigene115609_All	242	8.22	21.98	12.10	9.88	8.82	13.25	9.09	6.56	3.29	1.42E+00	2.42E-07	Up	5.59E-01	1.44E-01		-1.63E-01	7.23E-01		4.23E-01	2.54E-01		-4.69E-01	2.41E-01		-1.46E+00	1.79E-04	Down	Unigene115609_All//gi|4731573|gb|AAD28506.1|AF123265_1//6.00E-06//remorin 1 [Solanum lycopersicum]	Unigene115609_All//4.00E-07//AT2G45820| DNA-binding protein, putative	Unigene115609_All//3.00E-115//TC156235				nr -
Unigene11561_All	4843	352.40	168.44	344.81	128.34	158.34	134.87	13.22	10.29	7.87	-1.06E+00	0.00E+00	Down	-3.14E-02	2.10E-02		3.03E-01	7.11E-78		7.17E-02	3.11E-04		-3.62E-01	5.72E-10		-7.48E-01	5.67E-36		Unigene11561_All//gi|291419594|gb|ADE05291.1|//0.00E+00//magnesium chelatase H subunit [Vitis vinifera]	Unigene11561_All//0.00E+00//AT5G13630| GUN5 (GENOMES UNCOUPLED 5); magnesium chelatase	Unigene11561_All//0.00E+00//TC140684		ko00860|Porphyrin and chlorophyll metabolism	GO:0003676//GO:0006779//GO:0008408//GO:0009528//GO:0010467//GO:0043234//GO:0051003	nr -
Unigene115610_All	334	0.00	0.00	0.00	0.00	0.00	0.00	0.89	4.38	0.00	null	null		null	null		null	null		null	null		2.31E+00	4.93E-05	Up	-9.79E+00	1.58E-02		Unigene115610_All//gi|242039819|ref|XP_002467304.1|//6.00E-12//hypothetical protein SORBIDRAFT_01g024221 [Sorghum bicolor] >gi|241921158|gb|EER94302.1| hypothetical protein SORBIDRAFT_01g024221 [Sorghum bicolor]		Unigene115610_All//5.00E-25//TC176575				nr -
Unigene115611_All	412	7.24	8.85	8.66	15.36	14.04	13.26	36.64	15.62	18.28	2.91E-01	3.85E-01		2.59E-01	4.60E-01		-1.29E-01	6.15E-01		-2.11E-01	4.30E-01		-1.23E+00	3.02E-19	Down	-1.00E+00	1.53E-14	Down	Unigene115611_All//gi|12229923|sp|Q9M4T8.1|PSA5_SOYBN//5.00E-43//RecName: Full=Proteasome subunit alpha type-5; AltName: Full=20S proteasome alpha subunit E; AltName: Full=20S proteasome subunit alpha-5 >gi|7839485|gb|AAF70292.1|AF255338_1 20S proteasome subunit [Glycine max]	Unigene115611_All//1.00E-43//AT3G14290| PAE2; endopeptidase/ peptidase/ threonine-type endopeptidase	Unigene115611_All//4.00E-171//TC164836		ko03050|Proteasome		nr +
Unigene115613_All	606	0.35	3.09	0.35	1.07	1.08	0.67	0.70	4.21	0.66	3.16E+00	5.97E-07	Up	2.53E-02	1.00E+00		1.49E-02	9.92E-01		-6.72E-01	4.78E-01		2.59E+00	2.41E-09	Up	-8.58E-02	9.39E-01				Unigene115613_All//1.00E-14//TC177549				
Unigene115624_All	521	0.00	1.75	0.00	0.86	1.39	0.87	0.41	3.53	0.84	1.08E+01	2.62E-05	Up	null	null		6.90E-01	4.47E-01		1.07E-02	1.00E+00		3.12E+00	3.27E-08	Up	1.05E+00	2.98E-01		Unigene115624_All//gi|224093616|ref|XP_002309943.1|//2.00E-15//predicted protein [Populus trichocarpa] >gi|222852846|gb|EEE90393.1| predicted protein [Populus trichocarpa]						nr +
Unigene11563_All	3216	10.72	7.66	24.12	41.65	53.02	46.16	29.74	23.59	33.91	-4.86E-01	9.92E-08		1.17E+00	3.73E-70	Up	3.48E-01	1.11E-22		1.48E-01	4.78E-04		-3.34E-01	1.31E-12		1.89E-01	1.54E-05		Unigene11563_All//gi|22795064|gb|AAN05433.1|//0.00E+00//phospholipase D delta isoform 1a [Gossypium hirsutum]	Unigene11563_All//0.00E+00//AT4G35790| ATPLDDELTA; phospholipase D	Unigene11563_All//0.00E+00//TC131269		ko04144|Endocytosis//ko00564|Glycerophospholipid metabolism//ko00565|Ether lipid metabolism	GO:0004620//GO:0006650//GO:0044464//GO:0046872	nr -
Unigene115630_All	599	0.09	0.00	0.00	0.33	0.00	0.30	0.00	3.07	0.13	-6.45E+00	6.16E-01		-6.45E+00	6.29E-01		-8.38E+00	8.46E-02		-1.41E-01	9.35E-01		1.16E+01	5.95E-13	Up	7.06E+00	4.05E-01								
Unigene115631_All	416	0.00	0.00	0.13	0.00	0.00	0.00	0.00	2.11	0.19	null	null		7.00E+00	6.39E-01		null	null		null	null		1.10E+01	2.86E-06	Up	7.58E+00	4.05E-01				Unigene115631_All//1.00E-53//FL576735				
Unigene115633_All	236	0.00	0.00	0.00	0.00	0.00	0.00	1.25	6.38	0.17	null	null		null	null		null	null		null	null		2.35E+00	3.01E-05	Up	-2.89E+00	6.77E-02				Unigene115633_All//3.00E-19//TC146753				
Unigene115637_All	593	0.00	0.00	0.00	0.00	0.00	0.00	1.21	2.04	0.00	null	null		null	null		null	null		null	null		7.54E-01	1.77E-01		-1.02E+01	1.78E-05	Down							
Unigene115638_All	532	0.00	0.00	0.00	0.00	0.00	0.00	0.08	2.51	0.00	null	null		null	null		null	null		null	null		4.98E+00	3.03E-08	Up	-6.31E+00	6.15E-01								
Unigene115645_All	302	0.00	0.00	0.00	0.00	0.00	0.00	1.96	1.52	0.00	null	null		null	null		null	null		null	null		-3.65E-01	6.49E-01		-1.09E+01	1.41E-04	Down	Unigene115645_All//gi|300268240|gb|EFJ52421.1|//1.00E-17//component of cytosolic 80S ribosome and 60S large subunit [Volvox carteri f. nagariensis]	Unigene115645_All//6.00E-19//AT5G46430| 60S ribosomal protein L32 (RPL32B)			ko03010|Ribosome		nr -
Unigene115649_All	739	0.00	0.41	0.00	0.00	0.00	0.49	2.35	11.37	1.19	8.68E+00	4.44E-02		null	null		null	null		8.93E+00	2.37E-02		2.28E+00	3.08E-25	Up	-9.84E-01	2.16E-02		Unigene115649_All//gi|159488530|ref|XP_001702260.1|//2.00E-12//DEAH-box RNA helicase [Chlamydomonas reinhardtii] >gi|158271237|gb|EDO97061.1| DEAH-box RNA helicase [Chlamydomonas reinhardtii]		Unigene115649_All//7.00E-26//EX168464		ko03040|Spliceosome		nr -
Unigene11565_All	1076	6.27	5.13	0.99	20.18	24.19	10.41	9.23	6.37	5.26	-2.89E-01	2.21E-01		-2.66E+00	3.87E-19	Down	2.61E-01	1.40E-02		-9.55E-01	3.47E-16		-5.36E-01	9.35E-04		-8.13E-01	3.43E-07		Unigene11565_All//gi|224124160|ref|XP_002319260.1|//1.00E-132//GH3 family protein [Populus trichocarpa] >gi|222857636|gb|EEE95183.1| GH3 family protein [Populus trichocarpa]	Unigene11565_All//3.00E-131//AT5G54510| DFL1 (DWARF IN LIGHT 1); indole-3-acetic acid amido synthetase	Unigene11565_All//0.00E+00//TC142322				nr -
Unigene115651_All	480	0.00	0.00	0.00	0.00	0.00	0.00	1.23	2.18	0.00	null	null		null	null		null	null		null	null		8.20E-01	1.79E-01		-1.03E+01	1.41E-04	Down	Unigene115651_All//gi|224003875|ref|XP_002291609.1|//2.00E-52//calreticulin-like protein [Thalassiosira pseudonana CCMP1335] >gi|220973385|gb|EED91716.1| calreticulin-like protein [Thalassiosira pseudonana CCMP1335]	Unigene115651_All//8.00E-39//AT1G56340| CRT1 (CALRETICULIN 1); calcium ion binding / unfolded protein binding			ko04141|Protein processing in endoplasmic reticulum//ko04145|Phagosome		nr -
Unigene115653_All	798	0.00	0.00	0.00	0.00	0.00	0.00	1.27	3.09	0.10	null	null		null	null		null	null		null	null		1.28E+00	5.37E-04	Up	-3.67E+00	1.15E-05	Down	Unigene115653_All//gi|300264012|gb|EFJ48210.1|//1.00E-102//component of cytosolic 80S ribosome and 60S large subunit [Volvox carteri f. nagariensis]	Unigene115653_All//8.00E-91//AT4G36130| 60S ribosomal protein L8 (RPL8C)	Unigene115653_All//2.00E-08//EV498577		ko03010|Ribosome		nr -
Unigene115654_All	489	4.81	7.15	10.45	13.04	14.15	6.74	27.32	43.16	12.38	5.70E-01	8.47E-02		1.12E+00	6.63E-05	Up	1.18E-01	6.54E-01		-9.52E-01	3.46E-05		6.60E-01	7.35E-10		-1.14E+00	8.38E-16	Down	Unigene115654_All//gi|166865124|gb|ABZ01817.1|//1.00E-33//cinnamyl alcohol dehydrogenase [Gossypium hirsutum]	Unigene115654_All//8.00E-29//AT4G34230| ATCAD5 (CINNAMYL ALCOHOL DEHYDROGENASE 5); cinnamyl-alcohol dehydrogenase	Unigene115654_All//4.00E-88//CO087836		ko00940|Phenylpropanoid biosynthesis		nr -
Unigene115655_All	293	0.00	0.00	0.00	0.00	0.00	0.00	0.87	4.71	0.00	null	null		null	null		null	null		null	null		2.44E+00	4.59E-05	Up	-9.76E+00	3.04E-02				Unigene115655_All//9.00E-17//ES843311				
Unigene11566_All	2066	6.99	7.11	1.73	24.55	27.55	13.93	24.23	19.48	13.63	2.51E-02	8.82E-01		-2.02E+00	4.22E-29	Down	1.67E-01	1.96E-02		-8.18E-01	1.41E-28		-3.15E-01	2.49E-06		-8.30E-01	9.59E-34		Unigene11566_All//gi|224124160|ref|XP_002319260.1|//0.00E+00//GH3 family protein [Populus trichocarpa] >gi|222857636|gb|EEE95183.1| GH3 family protein [Populus trichocarpa]	Unigene11566_All//0.00E+00//AT5G54510| DFL1 (DWARF IN LIGHT 1); indole-3-acetic acid amido synthetase	Unigene11566_All//0.00E+00//TC142322			GO:0000902//GO:0009314//GO:0009536//GO:0009755//GO:0016881//GO:0048878	nr -
Unigene115661_All	471	0.00	0.00	0.00	0.00	0.00	0.00	0.54	8.52	0.00	null	null		null	null		null	null		null	null		3.98E+00	5.65E-21	Up	-9.07E+00	3.04E-02								
Unigene115663_All	411	0.00	0.00	0.00	0.12	0.00	0.00	0.31	9.15	0.10	null	null		null	null		-6.92E+00	5.60E-01		-6.92E+00	6.41E-01		4.89E+00	2.71E-22	Up	-1.67E+00	4.78E-01				Unigene115663_All//6.00E-173//gi|109835103|gb|DW233668.1|DW233668				
Unigene115666_All	565	0.00	0.00	0.19	0.00	0.00	0.00	0.45	3.25	0.00	null	null		7.56E+00	4.01E-01		null	null		null	null		2.86E+00	1.36E-07	Up	-8.81E+00	3.04E-02		Unigene115666_All//gi|224115056|ref|XP_002332226.1|//1.00E-46//GH3 family protein [Populus trichocarpa] >gi|222831839|gb|EEE70316.1| GH3 family protein [Populus trichocarpa]	Unigene115666_All//1.00E-44//AT2G23170| GH3.3; indole-3-acetic acid amido synthetase	Unigene115666_All//1.00E-63//ES805542				nr +

Unigene115669_All	375	0.28	1.35	0.00	0.00	0.00	0.24	0.34	3.34	0.21	2.28E+00	6.23E-02		-8.12E+00	3.97E-01		null	null		7.91E+00	4.79E-01		3.31E+00	4.67E-06	Up	-6.70E-01	7.20E-01		Unigene115669_All//gi|8778289|gb|AAF79298.1|AC068602_21//1.00E-07//F14D16.29 [Arabidopsis thaliana]	Unigene115669_All//7.00E-08//AT1G19130| FUNCTIONS IN: molecular_function unknown; INVOLVED IN: biological_process unknown; LOCATED IN: cellular_component unknown; EXPRESSED IN: 23 plant structures; EXPRESSED DURING: 13 growth stages; CONTAINS InterPro DOMAIN/s: Protein of unknown function DUF985 (InterPro:IPR009327), RmlC-like jelly roll fold (InterPro:IPR014710); Has 778 Blast hits to 778 proteins in 280 species: Archae - 8; Bacteria - 513; Metazoa - 2; Fungi - 16; Plants - 22; Viruses - 0; Other Eukaryotes - 217 (source: NCBI BLink).	Unigene115669_All//1.00E-72//AW187833				nr +
Unigene115671_All	611	0.09	0.17	0.70	0.57	0.17	0.37	1.04	3.28	0.13	9.55E-01	7.27E-01		3.03E+00	5.73E-02		-1.75E+00	1.90E-01		-6.27E-01	6.21E-01		1.66E+00	1.31E-04	Up	-2.99E+00	2.58E-03		Unigene115671_All//gi|17065180|gb|AAL32744.1|//8.00E-26//Unknown protein [Arabidopsis thaliana]	Unigene115671_All//2.00E-27//AT5G24460| unknown protein	Unigene115671_All//2.00E-10//DN801452				nr +
Unigene115674_All	300	0.00	0.00	0.00	0.00	0.00	0.00	0.28	2.79	0.00	null	null		null	null		null	null		null	null		3.31E+00	3.10E-04	Up	-8.14E+00	3.59E-01								
Unigene115680_All	510	0.00	0.00	0.00	0.00	0.00	0.00	1.16	1.15	0.00	null	null		null	null		null	null		null	null		-1.66E-02	1.02E+00		-1.02E+01	1.41E-04	Down	Unigene115680_All//gi|255071427|ref|XP_002499387.1|//4.00E-51//sterol methytransferase [Micromonas sp. RCC299] >gi|226514650|gb|ACO60646.1| sterol methytransferase [Micromonas sp. RCC299]	Unigene115680_All//2.00E-49//AT5G13710| SMT1 (STEROL METHYLTRANSFERASE 1); sterol 24-C-methyltransferase	Unigene115680_All//4.00E-08//TC162556		ko00100|Steroid biosynthesis		nr +
Unigene115684_All	450	0.00	0.00	0.00	0.00	0.00	0.00	15.69	11.52	2.83	null	null		null	null		null	null		null	null		-4.46E-01	2.52E-02		-2.47E+00	2.82E-24	Down	Unigene115684_All//gi|149941236|emb|CAO02550.1|//8.00E-07//putative ribosomal protein S3 [Vigna unguiculata]		Unigene115684_All//2.00E-25//TC146107				nr -
Unigene11569_All	2621	55.36	93.76	87.85	22.24	37.54	36.81	11.11	16.09	22.89	7.60E-01	7.65E-111		6.66E-01	2.57E-80		7.55E-01	1.36E-52		7.27E-01	1.17E-43		5.34E-01	3.58E-13		1.04E+00	3.16E-58	Up	Unigene11569_All//gi|297797507|ref|XP_002866638.1|//0.00E+00//ATGCN5 [Arabidopsis lyrata subsp. lyrata] >gi|297312473|gb|EFH42897.1| ATGCN5 [Arabidopsis lyrata subsp. lyrata]	Unigene11569_All//0.00E+00//AT5G64840| ATGCN5 (A. THALIANA GENERAL CONTROL NON-REPRESSIBLE 5); transporter	Unigene11569_All//0.00E+00//TC149541			GO:0016887//GO:0032559//GO:0043231	nr +
Unigene115693_All	443	0.00	0.00	0.00	0.00	0.00	0.00	1.24	3.49	0.00	null	null		null	null		null	null		null	null		1.49E+00	2.61E-03		-1.03E+01	2.81E-04	Down	Unigene115693_All//gi|224000193|ref|XP_002289769.1|//2.00E-46//RL11A, ribosomal protein 11A 60S large ribosomal subunit [Thalassiosira pseudonana CCMP1335] >gi|220974977|gb|EED93306.1| RL11A, ribosomal protein 11A 60S large ribosomal subunit [Thalassiosira pseudonana CCMP1335]	Unigene115693_All//9.00E-47//AT5G45775| 60S ribosomal protein L11 (RPL11D)			ko03010|Ribosome		nr +
Unigene115694_All	566	0.00	0.00	0.00	0.00	0.00	0.00	1.87	10.86	3.45	null	null		null	null		null	null		null	null		2.54E+00	4.24E-21	Up	8.85E-01	2.73E-02								
Unigene1157_All	727	0.00	0.00	0.07	0.14	0.28	0.37	13.20	5.75	7.40	null	null		6.19E+00	6.39E-01		1.05E+00	6.13E-01		1.44E+00	4.09E-01		-1.20E+00	4.45E-12	Down	-8.36E-01	2.83E-07		Unigene1157_All//gi|147826806|emb|CAN63950.1|//3.00E-10//hypothetical protein [Vitis vinifera]		Unigene1157_All//8.00E-72//TC171648			GO:0003676//GO:0004175//GO:0006260//GO:0006325//GO:0008168//GO:0008213//GO:0034061//GO:0043231//GO:0044427//GO:0046914	nr +
Unigene11570_All	2075	3.40	1.81	2.26	42.59	23.91	11.71	25.51	4.41	7.81	-9.14E-01	3.88E-05		-5.92E-01	1.01E-02		-8.33E-01	8.35E-58		-1.86E+00	1.13E-178	Down	-2.53E+00	8.23E-177	Down	-1.71E+00	8.99E-110	Down	Unigene11570_All//gi|224482639|gb|ACN50176.1|//0.00E+00//pectinesterase [Annona cherimola]	Unigene11570_All//0.00E+00//AT1G76160| sks5 (SKU5 Similar 5); copper ion binding / oxidoreductase	Unigene11570_All//0.00E+00//TC161343		ko00910|Nitrogen metabolism//ko00053|Ascorbate and aldarate metabolism	GO:0004091//GO:0005576//GO:0005618//GO:0008152//GO:0016682//GO:0046914	nr +
Unigene115700_All	303	0.00	0.00	0.00	0.00	0.00	0.00	0.98	10.48	3.16	null	null		null	null		null	null		null	null		3.42E+00	6.83E-15	Up	1.69E+00	9.76E-03		Unigene115700_All//gi|195646322|gb|ACG42629.1|//2.00E-17//pirin-like protein [Zea mays]	Unigene115700_All//6.00E-14//AT2G43120| pirin, putative	Unigene115700_All//6.00E-83//TC141238				nr -
Unigene115703_All	794	0.26	1.47	1.01	1.32	0.39	0.00	0.80	9.37	1.05	2.48E+00	9.80E-04	Up	1.93E+00	3.42E-02		-1.76E+00	8.43E-03		-1.04E+01	1.47E-06	Down	3.55E+00	3.34E-35	Up	4.00E-01	5.65E-01		Unigene115703_All//gi|15222705|ref|NP_173966.1|//2.00E-66//FAD-binding domain-containing protein [Arabidopsis thaliana] >gi|9797756|gb|AAF98574.1|AC013427_17 Contains weak similarity to berberine bridge enzyme (bbe1) from Berberis stolonifera gb|AF049347 and contains a FAD binding PF|01565 domain [Arabidopsis thaliana]	Unigene115703_All//9.00E-68//AT1G26420| FAD-binding domain-containing protein	Unigene115703_All//2.00E-180//TC130360				nr +
Unigene115705_All	413	0.00	0.00	0.00	0.00	0.00	0.00	0.10	2.23	0.00	null	null		null	null		null	null		null	null		4.44E+00	1.66E-05	Up	-6.68E+00	6.15E-01								ESTscan -
Unigene11571_All	2213	18.72	5.61	5.49	19.34	17.47	29.18	43.29	5.40	17.19	-1.74E+00	2.95E-70	Down	-1.77E+00	1.46E-69	Down	-1.46E-01	9.20E-02		5.94E-01	9.88E-21		-3.00E+00	0.00E+00	Down	-1.33E+00	2.20E-137	Down	Unigene11571_All//gi|224061929|ref|XP_002300669.1|//0.00E+00//multicopper oxidase [Populus trichocarpa] >gi|222842395|gb|EEE79942.1| multicopper oxidase [Populus trichocarpa]	Unigene11571_All//0.00E+00//AT1G76160| sks5 (SKU5 Similar 5); copper ion binding / oxidoreductase	Unigene11571_All//0.00E+00//TC155372		ko00910|Nitrogen metabolism//ko00053|Ascorbate and aldarate metabolism	GO:0004091//GO:0005576//GO:0005618//GO:0008152//GO:0016682//GO:0046914	nr +
Unigene115711_All	429	0.00	0.00	0.00	0.00	0.00	0.00	0.20	2.34	0.00	null	null		null	null		null	null		null	null		3.57E+00	3.08E-05	Up	-7.62E+00	3.59E-01								ESTscan +
Unigene115714_All	491	0.00	0.00	0.00	0.00	0.00	0.00	0.00	1.19	0.00	null	null		null	null		null	null		null	null		1.02E+01	2.81E-04	Up	null	null		Unigene115714_All//sp|Q9GPH3|ATFC_BOMMO//1.00E-06//Activating transcription factor of chaperone OS=Bombyx mori GN=ATFC PE=2 SV=1						swissprot +
Unigene115716_All	797	0.00	0.00	0.00	0.00	0.00	0.00	1.38	6.14	0.60	null	null		null	null		null	null		null	null		2.15E+00	4.89E-14	Up	-1.20E+00	3.41E-02				Unigene115716_All//6.00E-08//TC160492				
Unigene115718_All	511	0.10	0.00	0.00	0.00	0.47	1.24	0.17	0.08	0.08	-6.68E+00	6.16E-01		-6.68E+00	6.29E-01		8.88E+00	1.04E-01		1.03E+01	7.60E-04	Up	-1.02E+00	7.26E-01		-1.09E+00	6.77E-01		Unigene115718_All//gi|212960417|gb|ACJ38666.1|//2.00E-78//cellulose synthase [Betula luminifera]	Unigene115718_All//2.00E-65//AT5G44030| CESA4 (CELLULOSE SYNTHASE A4); cellulose synthase/ transferase, transferring glycosyl groups	Unigene115718_All//4.00E-39//TC129590				nr +
Unigene115721_All	491	0.00	0.00	0.00	0.00	0.00	0.00	2.15	2.89	0.16	null	null		null	null		null	null		null	null		4.27E-01	4.07E-01		-3.73E+00	6.13E-06	Down	Unigene115721_All//gi|261887565|gb|ACY05960.1|//1.00E-82//alpha-tubulin [Haematococcus pluvialis]	Unigene115721_All//1.00E-69//AT4G14960| TUA6; structural constituent of cytoskeleton	Unigene115721_All//4.00E-17//ES829891		ko04145|Phagosome		nr +
Unigene115723_All	306	0.00	0.00	0.00	0.00	0.00	0.00	0.00	2.73	0.00	null	null		null	null		null	null		null	null		1.14E+01	5.54E-06	Up	null	null			Unigene115723_All//9.00E-05//AT5G37640| UBQ9; protein binding					
Unigene115725_All	915	0.00	0.00	0.00	0.00	0.00	0.00	0.05	3.29	0.00	null	null		null	null		null	null		null	null		6.15E+00	1.28E-19	Up	-5.53E+00	6.15E-01		Unigene115725_All//gi|301115724|ref|XP_002905591.1|//8.00E-19//kinesin-like protein [Phytophthora infestans T30-4] >gi|262110380|gb|EEY68432.1| kinesin-like protein [Phytophthora infestans T30-4]	Unigene115725_All//1.00E-17//AT3G49650| kinesin motor protein-related					nr +
Unigene115733_All	813	0.26	0.12	0.33	0.12	0.30	0.22	0.88	4.73	1.27	-1.05E+00	5.65E-01		3.48E-01	7.97E-01		1.28E+00	5.32E-01		8.59E-01	6.96E-01		2.42E+00	9.00E-13	Up	5.27E-01	3.75E-01				Unigene115733_All//7.00E-23//TC153927				
Unigene115735_All	326	0.00	0.00	0.00	0.00	0.00	0.00	0.13	2.05	0.00	null	null		null	null		null	null		null	null		3.98E+00	6.38E-04	Up	-7.02E+00	6.15E-01		Unigene115735_All//gi|89276295|gb|ABD66504.1|//2.00E-18//actin depolymerizing factor 8 [Gossypium hirsutum] >gi|119388970|gb|AAY88048.2| actin depolymerizing factor [Gossypium hirsutum]	Unigene115735_All//8.00E-19//AT5G52360| ADF10 (ACTIN DEPOLYMERIZING FACTOR 10); actin binding					nr -
Unigene115737_All	614	0.00	0.00	0.00	0.00	0.00	0.00	1.38	10.55	0.26	null	null		null	null		null	null		null	null		2.94E+00	7.41E-26	Up	-2.41E+00	1.68E-03		Unigene115737_All//gi|186501250|ref|NP_849973.3|//9.00E-59//metal ion binding [Arabidopsis thaliana]	Unigene115737_All//5.00E-60//AT2G18196| metal ion binding	Unigene115737_All//2.00E-56//DN800214				nr +
Unigene115739_All	610	0.00	0.00	0.00	0.00	0.00	0.00	1.25	3.77	0.26	null	null		null	null		null	null		null	null		1.59E+00	6.35E-05	Up	-2.26E+00	4.68E-03		Unigene115739_All//gi|297842307|ref|XP_002889035.1|//7.00E-32//leucine-rich repeat family protein [Arabidopsis lyrata subsp. lyrata] >gi|297334876|gb|EFH65294.1| leucine-rich repeat family protein [Arabidopsis lyrata subsp. lyrata]	Unigene115739_All//1.00E-28//AT1G75640| leucine-rich repeat family protein / protein kinase family protein			ko04626|Plant-pathogen interaction		nr +
Unigene115742_All	846	909.12	761.18	1391.68	169.87	218.74	174.14	1.15	0.84	0.05	-2.56E-01	2.06E-47		6.14E-01	0.00E+00		3.65E-01	9.09E-27		3.58E-02	5.17E-01		-4.53E-01	4.90E-01		-4.61E+00	3.32E-06	Down	Unigene115742_All//gi|224124190|ref|XP_002330127.1|//1.00E-127//light-harvesting complex I protein Lhca4 [Populus trichocarpa] >gi|222871261|gb|EEF08392.1| light-harvesting complex I protein Lhca4 [Populus trichocarpa]	Unigene115742_All//4.00E-122//AT3G47470| LHCA4 (LIGHT-HARVESTING CHLOROPHYLL-PROTEIN COMPLEX I SUBUNIT A4); chlorophyll binding	Unigene115742_All//0.00E+00//TC130501		ko00196|Photosynthesis - antenna proteins		nr -
Unigene11575_All	1936	13.11	12.30	8.50	28.56	27.39	23.72	30.64	15.70	13.85	-9.16E-02	4.53E-01		-6.25E-01	1.99E-08		-6.03E-02	5.10E-01		-2.68E-01	1.33E-04		-9.65E-01	9.69E-50		-1.15E+00	1.82E-67	Down	Unigene11575_All//gi|222423108|dbj|BAH19533.1|//1.00E-108//AT4G08310 [Arabidopsis thaliana]	Unigene11575_All//5.00E-78//AT4G08310| unknown protein	Unigene11575_All//0.00E+00//TC146538				nr -
Unigene115753_All	316	0.00	1.28	0.00	0.47	0.65	0.00	2.01	1.32	0.13	1.03E+01	1.36E-02		null	null		4.67E-01	8.25E-01		-8.89E+00	2.34E-01		-6.02E-01	4.67E-01		-3.99E+00	5.82E-04	Down							
Unigene115754_All	527	0.00	0.00	0.00	0.00	0.00	0.00	1.12	4.36	0.60	null	null		null	null		null	null		null	null		1.96E+00	2.90E-06	Up	-8.93E-01	2.72E-01				Unigene115754_All//3.00E-09//gi|84155251|gb|DN804606.1|DN804606				
Unigene115758_All	505	0.00	1.00	0.11	0.30	0.55	0.54	0.50	2.48	0.16	9.97E+00	4.05E-03		6.72E+00	6.39E-01		8.83E-01	5.95E-01		8.59E-01	6.31E-01		2.31E+00	2.06E-04	Up	-1.67E+00	2.56E-01								
Unigene115759_All	545	0.00	0.00	0.00	0.00	0.00	0.00	1.32	2.76	0.00	null	null		null	null		null	null		null	null		1.07E+00	2.90E-02		-1.04E+01	1.78E-05	Down	Unigene115759_All//gi|168056108|ref|XP_001780064.1|//4.00E-45//ATP-binding cassette transporter, subfamily G, member 3, group WBC protein PpABCG3 [Physcomitrella patens subsp. patens] >gi|162668562|gb|EDQ55167.1| ATP-binding cassette transporter, subfamily G, member 3, group WBC protein PpABCG3 [Physcomitrella patens subsp. patens]	Unigene115759_All//9.00E-44//AT2G39350| ABC transporter family protein			ko03040|Spliceosome//ko02010|ABC transporters		nr -
Unigene115760_All	737	0.00	0.00	0.00	0.14	0.51	0.74	1.84	3.01	5.35	null	null		null	null		1.93E+00	1.94E-01		2.44E+00	4.41E-02		7.11E-01	6.62E-02		1.54E+00	8.71E-08	Up			Unigene115760_All//0.00E+00//BM358110				
Unigene115765_All	547	0.00	0.00	0.00	0.00	0.00	0.00	0.70	2.98	0.00	null	null		null	null		null	null		null	null		2.10E+00	5.14E-05	Up	-9.44E+00	4.20E-03								
Unigene115766_All	678	0.00	0.00	0.00	0.00	0.00	0.00	1.00	2.22	0.00	null	null		null	null		null	null		null	null		1.15E+00	1.87E-02		-9.96E+00	3.56E-05	Down							
Unigene115768_All	334	0.00	0.00	0.00	0.00	0.00	0.00	1.52	2.75	0.00	null	null		null	null		null	null		null	null		8.58E-01	1.95E-01		-1.06E+01	5.54E-04	Down	Unigene115768_All//gi|159465411|ref|XP_001690916.1|//7.00E-10//extracellular matrix protein [Chlamydomonas reinhardtii] >gi|158279602|gb|EDP05362.1| extracellular matrix protein [Chlamydomonas reinhardtii]				ko04141|Protein processing in endoplasmic reticulum//ko03420|Nucleotide excision repair		nr -
Unigene11577_All	406	15.46	28.08	11.67	1.47	1.70	2.67	0.62	6.28	1.08	8.61E-01	4.34E-07		-4.06E-01	1.08E-01		2.04E-01	8.21E-01		8.59E-01	2.17E-01		3.33E+00	8.60E-12	Up	7.89E-01	4.23E-01		Unigene11577_All//gi|224979415|gb|ACN72639.1|//3.00E-20//nitrate transporter [Malus hupehensis]	Unigene11577_All//5.00E-11//AT5G62680| proton-dependent oligopeptide transport (POT) family protein	Unigene11577_All//0.00E+00//gi|21100916|gb|BQ413229.1|BQ413229			GO:0015833//GO:0016020	nr +
Unigene115771_All	369	0.00	0.00	0.00	0.27	0.00	0.00	0.57	3.06	3.89	null	null		null	null		-8.08E+00	3.15E-01		-8.08E+00	4.03E-01		2.42E+00	3.17E-04	Up	2.76E+00	4.81E-06	Up			Unigene115771_All//2.00E-160//TC178436				
Unigene115777_All	368	0.00	0.00	0.00	0.00	0.00	0.00	2.18	2.95	0.00	null	null		null	null		null	null		null	null		4.36E-01	4.77E-01		-1.11E+01	4.43E-06	Down			Unigene115777_All//1.00E-28//gi|48805733|gb|CO107047.1|CO107047				
Unigene115786_All	370	0.00	0.00	0.00	0.00	0.00	0.00	0.00	3.28	0.00	null	null		null	null		null	null		null	null		1.17E+01	1.40E-08	Up	null	null								ESTscan -

Unigene115789_All	337	0.31	0.00	0.32	0.30	0.20	0.27	0.38	3.10	1.89	-8.28E+00	3.75E-01		2.53E-02	1.00E+00		-5.32E-01	8.18E-01		-1.41E-01	9.52E-01		3.04E+00	7.88E-05	Up	2.33E+00	1.05E-02		Unigene115789_All//gi|297829424|ref|XP_002882594.1|//2.00E-32//DNAJ heat shock N-terminal domain-containing protein [Arabidopsis lyrata subsp. lyrata] >gi|297328434|gb|EFH58853.1| DNAJ heat shock N-terminal domain-containing protein [Arabidopsis lyrata subsp. lyrata]	Unigene115789_All//2.00E-33//AT3G08970| ATERDJ3A; oxidoreductase	Unigene115789_All//6.00E-80//TC178503				nr -
Unigene11579_All	683	9.12	14.84	16.76	3.65	5.34	15.41	1.49	7.83	7.47	7.03E-01	9.34E-05		8.79E-01	5.15E-07		5.51E-01	8.96E-02		2.08E+00	2.03E-24	Up	2.40E+00	2.50E-17	Up	2.33E+00	2.27E-16	Up	Unigene11579_All//gi|11933400|dbj|BAB19757.1|//8.00E-33//nitrate transporter NRT1-2 [Glycine max]	Unigene11579_All//3.00E-31//AT1G18880| proton-dependent oligopeptide transport (POT) family protein	Unigene11579_All//1.00E-150//TC162742			GO:0015833//GO:0016020	nr -
Unigene115790_All	555	0.00	0.00	0.00	0.72	1.24	0.98	0.30	5.12	0.43	null	null		null	null		7.89E-01	4.04E-01		4.44E-01	6.78E-01		4.07E+00	3.20E-15	Up	4.99E-01	7.08E-01				Unigene115790_All//7.00E-07//ES824204				
Unigene115791_All	345	0.00	0.00	0.00	0.00	0.00	0.00	2.08	2.79	0.00	null	null		null	null		null	null		null	null		4.20E-01	5.31E-01		-1.10E+01	1.78E-05	Down	Unigene115791_All//gi|159473469|ref|XP_001694856.1|//3.00E-23//acidic ribosomal protein P2 [Chlamydomonas reinhardtii] >gi|158276235|gb|EDP02008.1| acidic ribosomal protein P2 [Chlamydomonas reinhardtii]	Unigene115791_All//5.00E-16//AT2G27710| 60S acidic ribosomal protein P2 (RPP2B)			ko03010|Ribosome		nr -
Unigene115796_All	378	0.00	0.00	0.00	0.00	0.00	0.00	0.45	2.76	0.21	null	null		null	null		null	null		null	null		2.63E+00	2.86E-04	Up	-1.09E+00	5.53E-01								
Unigene115799_All	994	0.00	0.00	0.00	0.00	0.00	0.00	0.04	3.45	0.00	null	null		null	null		null	null		null	null		6.34E+00	1.66E-22	Up	-5.41E+00	6.15E-01		Unigene115799_All//gi|71143066|gb|AAZ23924.1|//6.00E-06//At2g40070 [Arabidopsis thaliana] >gi|110742086|dbj|BAE98974.1| En/Spm-like transposon protein [Arabidopsis thaliana]						nr -
Unigene115800_All	362	0.00	0.00	0.00	0.00	0.00	0.00	0.23	2.08	0.00	null	null		null	null		null	null		null	null		3.15E+00	9.54E-04	Up	-7.87E+00	3.59E-01								
Unigene115803_All	323	2.27	0.00	0.99	1.39	1.71	0.00	6.15	5.05	2.71	-1.11E+01	2.11E-04	Down	-1.20E+00	1.80E-01		2.97E-01	8.18E-01		-1.04E+01	5.79E-03		-2.86E-01	5.28E-01		-1.18E+00	3.26E-03				Unigene115803_All//2.00E-46//gi|109857439|gb|DW486418.1|DW486418				
Unigene115804_All	571	0.00	0.00	0.00	0.00	0.00	0.00	1.18	3.00	0.00	null	null		null	null		null	null		null	null		1.34E+00	3.54E-03		-1.02E+01	3.56E-05	Down	Unigene115804_All//gi|300266840|gb|EFJ51026.1|//2.00E-46//component of cytosolic 80S ribosome and 40S small subunit [Volvox carteri f. nagariensis]	Unigene115804_All//3.00E-42//AT1G48830| 40S ribosomal protein S7 (RPS7A)			ko03010|Ribosome		nr -
Unigene115805_All	815	0.00	0.00	0.00	0.00	0.00	0.00	0.00	4.56	0.00	null	null		null	null		null	null		null	null		1.22E+01	3.56E-26	Up	null	null		Unigene115805_All//gi|294987218|gb|ADF56046.1|//9.00E-37//glutathione S-transferase [Grimmia pilifera]	Unigene115805_All//3.00E-07//AT2G30870| GSTF10 (HALIANA GLUTATHIONE S-TRANSFERASE PHI 10); copper ion binding / glutathione binding / glutathione transferase			ko00480|Glutathione metabolism		nr -
Unigene115809_All	372	0.00	0.00	0.00	0.00	0.00	0.00	0.68	3.82	0.32	null	null		null	null		null	null		null	null		2.49E+00	2.76E-05	Up	-1.09E+00	4.28E-01		Unigene115809_All//gi|300253702|gb|EFJ38959.1|//7.00E-12//hypothetical protein VOLCADRAFT_78580 [Volvox carteri f. nagariensis]		Unigene115809_All//4.00E-32//TC146753				nr -
Unigene11581_All	2304	23.50	30.00	26.35	10.27	14.83	29.64	4.20	25.63	17.93	3.52E-01	1.99E-08		1.65E-01	2.61E-02		5.30E-01	4.55E-10		1.53E+00	1.46E-102	Up	2.61E+00	2.14E-203	Up	2.09E+00	1.75E-111	Up	Unigene11581_All//gi|11933414|dbj|BAB19760.1|//0.00E+00//nitrate transporter NRT1-5 [Glycine max]	Unigene11581_All//1.00E-165//AT5G62680| proton-dependent oligopeptide transport (POT) family protein	Unigene11581_All//0.00E+00//TC159080				nr +
Unigene115811_All	500	0.00	0.00	0.00	0.00	0.00	0.72	15.05	10.11	75.61	null	null		null	null		null	null		9.50E+00	2.37E-02		-5.74E-01	2.46E-03		2.33E+00	1.87E-116	Up	Unigene115811_All//gi|224052970|ref|XP_002297642.1|//2.00E-47//2-oxoglutarate-dependent dioxygenase [Populus trichocarpa] >gi|222844900|gb|EEE82447.1| 2-oxoglutarate-dependent dioxygenase [Populus trichocarpa]	Unigene115811_All//4.00E-42//AT3G13610| oxidoreductase, 2OG-Fe(II) oxygenase family protein	Unigene115811_All//2.00E-65//FL577006		ko00941|Flavonoid biosynthesis		nr -
Unigene115823_All	690	0.38	0.29	0.15	0.51	0.10	0.46	3.00	18.17	7.79	-3.68E-01	7.79E-01		-1.30E+00	4.37E-01		-2.34E+00	1.01E-01		-1.41E-01	9.12E-01		2.60E+00	1.92E-43	Up	1.38E+00	7.77E-09	Up	Unigene115823_All//gi|297610489|ref|NP_001064615.2|//6.00E-06//Os10g0419100 [Oryza sativa Japonica Group] >gi|255679407|dbj|BAF26529.2| Os10g0419100 [Oryza sativa Japonica Group]	Unigene115823_All//5.00E-05//AT1G60690| aldo/keto reductase family protein	Unigene115823_All//0.00E+00//CD809346				nr +
Unigene115824_All	397	0.00	1.02	0.00	0.00	0.17	0.23	0.53	2.84	0.10	1.00E+01	1.36E-02		null	null		7.44E+00	6.13E-01		7.83E+00	4.79E-01		2.42E+00	3.17E-04	Up	-2.41E+00	1.95E-01								
Unigene115825_All	607	0.00	0.00	0.00	0.00	0.00	0.00	1.60	3.72	0.00	null	null		null	null		null	null		null	null		1.21E+00	1.71E-03		-1.06E+01	2.72E-07	Down	Unigene115825_All//gi|29691166|gb|AA089237.1|//1.00E-66//adenosylhomocysteinase [Medicago truncatula]	Unigene115825_All//2.00E-66//AT4G13940| MEE58 (MATERNAL EFFECT EMBRYO ARREST 58); adenosylhomocysteinase/ copper ion binding	Unigene115825_All//7.00E-07//gi|164343927|gb|ES810489.1|ES810489		ko00270|Cysteine and methionine metabolism//ko00450|Selenoamino acid metabolism		nr +
Unigene115826_All	413	0.25	0.37	0.13	0.24	0.17	0.33	0.41	2.43	0.58	5.39E-01	7.80E-01		-9.75E-01	7.22E-01		-5.32E-01	8.18E-01		4.44E-01	8.62E-01		2.57E+00	4.79E-04	Up	4.99E-01	7.08E-01				Unigene115826_All//5.00E-07//TC175259				
Unigene115829_All	395	0.00	0.90	0.54	0.00	0.00	0.00	3.21	5.08	0.10	9.81E+00	2.47E-02		9.07E+00	1.42E-01		null	null		null	null		6.61E-01	1.09E-01		-4.99E+00	3.13E-08	Down	Unigene115829_All//gi|113289|sp|P26182.1|ACT_ACHBI//7.00E-61//RecName: Full=Actin >gi|2290|emb|CAA42559.1| actin [Achlya bisexualis] >gi|78057820|gb|ABB17316.1| actin A [Peronospora viciae]	Unigene115829_All//8.00E-57//AT5G09810| ACT7 (ACTIN 7); structural constituent of cytoskeleton	Unigene115829_All//6.00E-71//TC153744		ko04145|Phagosome		nr +
Unigene115830_All	399	0.00	0.76	0.27	0.00	0.00	1.02	0.42	2.51	0.00	9.57E+00	4.44E-02		8.06E+00	4.01E-01		null	null		9.99E+00	1.36E-02		2.57E+00	4.79E-04	Up	-8.73E+00	1.08E-01				Unigene115830_All//5.00E-19//TC175987				
Unigene115832_All	539	0.00	0.00	0.00	0.00	0.00	0.00	0.00	2.56	0.00	null	null		null	null		null	null		null	null		1.13E+01	9.64E-10	Up	null	null		Unigene115832_All//gi|269315884|gb|ACZ37068.1|//4.00E-16//thioredoxin h4 [Medicago truncatula]	Unigene115832_All//1.00E-10//AT1G59730| ATH7 (thioredoxin H-type 7)					nr +
Unigene115833_All	380	1.93	0.00	1.26	6.55	2.27	0.71	5.23	11.33	1.89	-1.09E+01	2.11E-04	Down	-6.12E-01	4.70E-01		-1.53E+00	7.55E-05	Down	-3.20E+00	7.46E-10	Down	1.12E+00	2.70E-05	Up	-1.47E+00	3.81E-04	Down	Unigene115833_All//gi|30697277|ref|NP_851226.1|//4.00E-31//MYB59 (MYB DOMAIN PROTEIN 59); DNA binding / transcription factor [Arabidopsis thaliana] >gi|68480112|gb|AAY97895.1| MYB transcription factor MYB59-2 [Arabidopsis thaliana]	Unigene115833_All//2.00E-32//AT5G59780| MYB59 (MYB DOMAIN PROTEIN 59); DNA binding / transcription factor	Unigene115833_All//1.00E-72//TC178283	MYB-related			nr +
Unigene115835_All	675	0.00	0.00	0.16	0.30	0.36	0.33	0.44	2.66	2.36	null	null		7.30E+00	4.01E-01		2.75E-01	8.95E-01		1.80E-01	9.35E-01		2.60E+00	8.33E-07	Up	2.43E+00	7.10E-06	Up	Unigene115835_All//gi|7671450|emb|CAB89390.1|//1.00E-55//lectin-like protein kinase-like [Arabidopsis thaliana] >gi|91806848|gb|ABE66151.1| lectin protein kinase [Arabidopsis thaliana]	Unigene115835_All//8.00E-57//AT5G10530| lectin protein kinase, putative	Unigene115835_All//1.00E-73//CA994027		ko04626|Plant-pathogen interaction		nr +
Unigene115836_All	629	0.33	0.81	0.25	0.00	0.11	0.36	0.27	3.06	0.57	1.28E+00	2.31E-01		-3.89E-01	8.08E-01		6.77E+00	6.13E-01		8.49E+00	1.19E-01		3.51E+00	2.06E-09	Up	1.08E+00	3.50E-01		Unigene115836_All//gi|15222705|ref|NP_173966.1|//7.00E-33//FAD-binding domain-containing protein [Arabidopsis thaliana] >gi|9797756|gb|AAF98574.1|AC013427_17 Contains weak similarity to berberine bridge enzyme (bbe1) from Berberis stolonifera gb|AF049347 and contains a FAD binding PF|01565 domain [Arabidopsis thaliana]	Unigene115836_All//1.00E-34//AT1G26420| FAD-binding domain-containing protein	Unigene115836_All//1.00E-73//TC130360				nr +
Unigene115837_All	600	0.00	0.00	0.00	0.00	0.06	0.00	0.99	3.41	1.59	null	null		null	null		5.84E+00	8.35E-01		null	null		1.79E+00	4.09E-05	Up	6.92E-01	2.60E-01				Unigene115837_All//0.00E+00//DN804072				
Unigene115840_All	794	0.26	0.00	0.00	0.06	0.09	0.00	1.33	3.05	0.20	-8.04E+00	1.22E-01		-8.04E+00	1.46E-01		4.68E-01	9.60E-01		-5.97E+00	6.41E-01		1.20E+00	1.25E-03		-2.73E+00	1.12E-04	Down							
Unigene115841_All	733	0.00	0.00	0.00	0.00	0.00	0.00	1.62	2.74	0.00	null	null		null	null		null	null		null	null		7.61E-01	6.33E-02		-1.07E+01	8.15E-09	Down	Unigene115841_All//gi|297829496|ref|XP_002882630.1|//2.00E-66//60S ribosomal protein L4/L1 [Arabidopsis lyrata subsp. lyrata] >gi|297328470|gb|EFH58889.1| 60S ribosomal protein L4/L1 [Arabidopsis lyrata subsp. lyrata]	Unigene115841_All//7.00E-46//AT3G09630| 60S ribosomal protein L4/L1 (RPL4A)			ko03010|Ribosome		nr -
Unigene115842_All	1184	0.00	0.00	0.00	0.00	0.00	0.00	0.79	1.24	0.07	null	null		null	null		null	null		null	null		6.53E-01	1.93E-01		-3.55E+00	3.97E-05	Down	Unigene115842_All//gi|297721715|ref|NP_001173220.1|//4.00E-12//Os02g0832150 [Oryza sativa Japonica Group] >gi|255671379|dbj|BAH91949.1| Os02g0832150 [Oryza sativa Japonica Group]	Unigene115842_All//3.00E-12//AT4G32600| zinc finger (C3HC4-type RING finger) family protein					nr -
Unigene115845_All	420	0.00	0.00	0.00	0.00	0.00	0.00	0.70	3.28	0.00	null	null		null	null		null	null		null	null		2.22E+00	1.31E-04	Up	-9.46E+00	1.58E-02		Unigene115845_All//gi|301121610|ref|XP_002908532.1|//2.00E-53//60S ribosomal protein L23 [Phytophthora infestans T30-4] >gi|66270163|gb|AAY43411.1| ribosomal protein L23 [Phytophthora infestans] >gi|262103563|gb|EEY61615.1| 60S ribosomal protein L23 [Phytophthora infestans T30-4]	Unigene115845_All//2.00E-52//AT3G04400| emb2171 (embryo defective 2171); structural constituent of ribosome	Unigene115845_All//2.00E-18//TC174626		ko03010|Ribosome		nr -
Unigene115846_All	405	0.00	0.00	0.00	0.00	0.00	0.00	0.10	2.79	0.00	null	null		null	null		null	null		null	null		4.74E+00	7.27E-07	Up	-6.71E+00	6.15E-01		Unigene115846_All//gi|160331695|ref|XP_001712554.1|//1.00E-19//kin(snf2) [Hemiselmis andersenii] >gi|159766003|gb|ABW98229.1| kin(snf2) [Hemiselmis andersenii]	Unigene115846_All//6.00E-18//AT1G78290| serine/threonine protein kinase, putative			ko04140|Regulation of autophagy		nr +
Unigene115847_All	726	0.00	0.70	0.00	0.41	0.09	0.37	1.34	5.18	1.10	9.45E+00	4.05E-03		null	null		-2.12E+00	1.66E-01		-1.41E-01	9.21E-01		1.95E+00	9.38E-10	Up	-2.88E-01	6.35E-01		Unigene115847_All//gi|226713182|sp|A7R333.1|U497A_VITVI//5.00E-46//RecName: Full=UPF0497 membrane protein 10	Unigene115847_All//6.00E-34//AT4G03540| integral membrane family protein					nr +
Unigene115848_All	872	0.18	0.17	0.00	0.40	0.04	0.78	1.21	7.43	1.87	-4.64E-02	9.89E-01		-7.49E+00	2.46E-01		-3.34E+00	4.07E-02		9.58E-01	3.09E-01		2.62E+00	6.16E-23	Up	6.28E-01	1.61E-01		Unigene115848_All//gi|115187640|gb|ABI84264.1|//2.00E-07//ubiquitin-conjugating enzyme [Arachis hypogaea]	Unigene115848_All//2.00E-07//AT3G55380| UBC14 (ubiquitin-conjugating enzyme 14); ubiquitin-protein ligase	Unigene115848_All//0.00E+00//gi|72273404|gb|DT049137.1|DT049137		ko04141|Protein processing in endoplasmic reticulum//ko04120|Ubiquitin mediated proteolysis		nr -
Unigene115850_All	420	0.00	0.00	0.00	0.00	0.00	0.00	0.00	2.19	0.00	null	null		null	null		null	null		null	null		1.11E+01	1.48E-06	Up	null	null		Unigene115850_All//gi|299473653|emb|CBN78047.1|//1.00E-21//flagellar associated protein, quinone reductase-like protein [Ectocarpus siliculosus]	Unigene115850_All//7.00E-17//AT5G54500| FQR1 (FLAVODOXIN-LIKE QUINONE REDUCTASE 1); FMN binding / oxidoreductase, acting on NADH or NADPH, quinone or similar compound as acceptor					nr +
Unigene115851_All	437	0.00	0.00	0.00	0.00	0.00	0.21	0.10	5.16	6.66	null	null		null	null		null	null		7.69E+00	4.79E-01		5.74E+00	1.88E-14	Up	6.10E+00	2.88E-19	Up							
Unigene115852_All	671	0.86	0.15	0.24	0.00	0.51	0.00	0.50	3.05	1.37	-2.51E+00	2.94E-02		-1.85E+00	9.62E-02		9.00E+00	3.02E-02		null	null		2.60E+00	1.19E-07	Up	1.44E+00	2.77E-02		Unigene115852_All//gi|124484395|dbj|BAF46308.1|//4.00E-07//leucine-rich repeat transmembrane protein kinase [Ipomoea nil]	Unigene115852_All//4.00E-08//AT5G01950| ATP binding / kinase/ protein serine/threonine kinase	Unigene115852_All//2.00E-35//gi|31072574|gb|CA992853.1|CA992853				nr -
Unigene115854_All	447	0.00	0.23	0.00	0.00	0.23	0.00	0.57	3.09	0.45	7.82E+00	3.89E-01		null	null		7.85E+00	4.57E-01		null	null		2.44E+00	4.59E-05	Up	-3.49E-01	7.67E-01				Unigene115854_All//4.00E-140//TC141652				
Unigene115859_All	438	0.00	0.00	0.00	0.00	0.00	0.00	0.00	1.62	0.00	null	null		null	null		null	null		null	null		1.07E+01	3.98E-05	Up	null	null		Unigene115859_All//gi|258598734|gb|ACV83439.1|//7.00E-54//QM-like protein [Zea mays]	Unigene115859_All//6.00E-53//AT1G66580| 60S ribosomal protein L10 (RPL10C)	Unigene115859_All//2.00E-58//CD485839		ko03010|Ribosome		nr -

Unigene115861_All	424	0.00	0.00	0.00	0.00	0.00	0.00	0.00	3.25	0.00	null	null		null	null		null	null		null	null		1.17E+01	9.64E-10	Up	null	null		Unigene115861_All//gi|159467345|ref|XP_001691852.1|//3.00E-10//DEAD/DEAH box helicase [Chlamydomonas reinhardtii] >gi|158278579|gb|EDP04342.1| DEAD/DEAH box helicase [Chlamydomonas reinhardtii]						nr +
Unigene115862_All	459	0.00	0.00	1.04	0.22	0.15	0.20	0.83	3.37	0.78	null	null		1.00E+01	7.49E-03		-5.33E-01	8.18E-01		-1.42E-01	9.52E-01		2.02E+00	1.30E-04	Up	-8.59E-02	9.43E-01								
Unigene115863_All	430	0.00	0.00	0.00	0.00	0.00	0.00	0.79	4.08	1.30	null	null		null	null		null	null		null	null		2.38E+00	4.38E-06	Up	7.22E-01	3.99E-01		Unigene115863_All//gi|42567541|ref|NP_195692.2|//2.00E-16//binding [Arabidopsis thaliana] >gi|53749172|gb|AAU90071.1| At4g39820 [Arabidopsis thaliana]	Unigene115863_All//2.00E-16//AT4G39820| binding	Unigene115863_All//6.00E-22//gi|21090904|gb|BQ403217.1|BQ403217				nr -
Unigene115864_All	454	0.00	0.00	0.00	0.00	0.00	0.00	0.56	3.50	0.00	null	null		null	null		null	null		null	null		2.65E+00	3.44E-06	Up	-9.13E+00	3.04E-02		Unigene115864_All//sp|Q5UQX7|YL881_MIMIV//4.00E-07//Uncharacterized protein L881 OS=Acanthamoeba polyphaga mimivirus GN=MIMI_L881 PE=4 SV=1						swissprot +
Unigene115865_All	790	0.00	0.00	0.00	0.00	0.00	0.00	1.34	3.54	0.00	null	null		null	null		null	null		null	null		1.41E+00	5.57E-05	Up	-1.04E+01	6.72E-08	Down	Unigene115865_All//gi|81074776|gb|ABB55374.1|//2.00E-92//ribosomal protein L2-like [Solanum tuberosum]	Unigene115865_All//1.00E-80//AT4G36130| 60S ribosomal protein L8 (RPL8C)	Unigene115865_All//2.00E-20//gi|78337755|gb|DT558029.1|DT558029		ko03010|Ribosome		nr -
Unigene115867_All	467	0.00	0.00	0.00	0.00	0.00	0.00	0.18	2.24	0.00	null	null		null	null		null	null		null	null		3.63E+00	1.71E-05	Up	-7.50E+00	3.59E-01								ESTscan -
Unigene115871_All	761	0.00	0.87	0.00	0.72	1.00	0.47	0.56	2.31	1.83	9.76E+00	6.26E-04	Up	null	null		4.67E-01	6.07E-01		-6.01E-01	5.66E-01		2.05E+00	3.20E-05	Up	1.72E+00	1.09E-03				Unigene115871_All//0.00E+00//CA993885				
Unigene115873_All	539	0.00	0.00	0.00	0.00	0.00	0.00	1.49	2.79	0.00	null	null		null	null		null	null		null	null		9.05E-01	6.27E-02		-1.05E+01	4.43E-06	Down	Unigene115873_All//gi|226501140|ref|NP_001152197.1|//3.00E-10//LOC100285835 [Zea mays] >gi|195653721|gb|ACG46328.1| keratin-associated protein 5-4 [Zea mays]						nr +
Unigene115874_All	493	0.00	0.00	0.00	0.00	0.00	0.00	2.14	5.34	0.16	null	null		null	null		null	null		null	null		1.32E+00	2.33E-04	Up	-3.73E+00	6.13E-06	Down	Unigene115874_All//gi|115467796|ref|NP_001057497.1|//2.00E-10//Os06g0316800 [Oryza sativa Japonica Group] >gi|113595537|dbj|BAF19411.1| Os06g0316800 [Oryza sativa Japonica Group]				ko03040|Spliceosome		nr +
Unigene115875_All	775	0.00	0.00	0.00	0.00	0.00	0.00	0.11	2.64	0.00	null	null		null	null		null	null		null	null		4.60E+00	6.19E-12	Up	-6.77E+00	3.59E-01								ESTscan +
Unigene115876_All	515	0.20	0.49	0.00	0.00	0.07	0.18	0.33	1.79	4.87	1.28E+00	4.35E-01		-7.67E+00	3.97E-01		6.06E+00	8.35E-01		7.45E+00	4.79E-01		2.44E+00	1.33E-03		3.89E+00	2.29E-13	Up	Unigene115876_All//gi|297720115|ref|NP_001172419.1|//1.00E-08//Os01g0558700 [Oryza sativa Japonica Group] >gi|255673363|dbj|BAH91149.1| Os01g0558700 [Oryza sativa Japonica Group]		Unigene115876_All//0.00E+00//CO102469				nr +
Unigene115877_All	474	0.00	0.00	0.67	0.74	0.07	1.05	0.45	12.79	1.26	null	null		9.40E+00	4.53E-02		-3.34E+00	4.07E-02		5.11E-01	6.41E-01		4.84E+00	1.65E-35	Up	1.50E+00	7.88E-02		Unigene115877_All//gi|87241148|gb|ABD33006.1|//8.00E-22//TNFR/CD27/30/40/95 cysteine-rich region [Medicago truncatula]	Unigene115877_All//9.00E-14//AT1G12064| unknown protein	Unigene115877_All//1.00E-10//TC168397				nr +
Unigene115879_All	516	0.00	0.00	0.00	0.00	0.00	0.00	0.66	2.59	0.54	null	null		null	null		null	null		null	null		1.98E+00	5.28E-04	Up	-2.79E-01	7.81E-01		Unigene115879_All//gi|157093563|gb|ABV22436.1|//1.00E-36//cysteine proteinase [Oxyrrhis marina]	Unigene115879_All//7.00E-33//AT5G43060| cysteine proteinase, putative / thiol protease, putative					nr -
Unigene115880_All	1142	0.00	0.00	0.00	0.00	0.00	0.00	0.00	4.28	0.00	null	null		null	null		null	null		null	null		1.21E+01	1.92E-34	Up	null	null		Unigene115880_All//gi|1155068|emb|CAA64425.1|//3.00E-07//cell wall-plasma membrane linker protein [Brassica napus]						nr -
Unigene115881_All	548	0.00	0.00	0.00	0.00	0.00	0.00	0.00	3.05	0.00	null	null		null	null		null	null		null	null		1.16E+01	8.76E-12	Up	null	null		Unigene115881_All//gi|38325811|gb|AAR17078.1|//5.00E-71//heat shock protein 70-1 [Nicotiana tabacum]	Unigene115881_All//5.00E-41//AT3G12580| HSP70 (heat shock protein 70); ATP binding	Unigene115881_All//6.00E-07//EX170481		ko03040|Spliceosome//ko04141|Protein processing in endoplasmic reticulum//ko04144|Endocytosis		nr +
Unigene115882_All	3348	0.00	0.00	0.00	0.00	0.00	0.00	0.43	0.10	0.77	null	null		null	null		null	null		null	null		-2.10E+00	1.56E-04	Down	8.49E-01	1.25E-02		Unigene115882_All//gi|115435462|ref|NP_001042489.1|//0.00E+00//Os01g0229700 [Oryza sativa Japonica Group] >gi|113532020|dbj|BAF04403.1| Os01g0229700 [Oryza sativa Japonica Group]	Unigene115882_All//1.00E-46//AT3G06880| nucleotide binding	Unigene115882_All//0.00E+00//gi|164312745|gb|ES848791.1|ES848791		ko03040|Spliceosome//ko04141|Protein processing in endoplasmic reticulum//ko04120|Ubiquitin mediated proteolysis		nr -
Unigene115886_All	564	0.00	0.00	0.00	0.00	0.00	0.00	0.60	2.74	0.07	null	null		null	null		null	null		null	null		2.19E+00	5.11E-05	Up	-3.09E+00	3.92E-02								
Unigene115887_All	530	0.00	0.00	0.00	0.00	0.00	0.00	0.56	3.39	0.00	null	null		null	null		null	null		null	null		2.60E+00	8.32E-07	Up	-9.13E+00	1.58E-02		Unigene115887_All//gi|298711872|emb|CBJ32893.1|//3.00E-72//EEF1A2, eukaryotic translation elongation factor 1 alpha [Ectocarpus siliculosus] >gi|298711873|emb|CBJ32894.1| EEF1A1, eukaryotic translation elongation factor 1 alpha [Ectocarpus siliculosus]	Unigene115887_All//1.00E-65//AT5G60390| elongation factor 1-alpha / EF-1-alpha	Unigene115887_All//3.00E-18//TC173271				nr -
Unigene115889_All	1158	0.00	0.00	0.00	0.00	0.00	0.00	0.62	0.00	0.00	null	null		null	null		null	null		null	null		-9.28E+00	3.27E-05	Down	-9.28E+00	1.78E-05	Down	Unigene115889_All//gi|297841521|ref|XP_002888642.1|//9.00E-80//S-adenosyl-L-methionine:carboxyl methyltransferase family protein [Arabidopsis lyrata subsp. lyrata] >gi|297334483|gb|EFH64901.1| S-adenosyl-L-methionine:carboxyl methyltransferase family protein [Arabidopsis lyrata subsp. lyrata]	Unigene115889_All//1.00E-67//AT5G37990| S-adenosylmethionine-dependent methyltransferase/ methyltransferase			ko00592|alpha-Linolenic acid metabolism		nr -
Unigene115890_All	689	0.00	0.00	0.00	0.00	0.00	0.00	0.43	1.82	0.00	null	null		null	null		null	null		null	null		2.08E+00	5.41E-04	Up	-8.75E+00	1.58E-02		Unigene115890_All//gi|5902592|gb|AAD55566.1|AF110784_1//1.00E-36//protein disulfide isomerase precursor [Volvox carteri f. nagariensis] >gi|300257635|gb|EFJ41881.1| protein disulfide isomerase 1 [Volvox carteri f. nagariensis]	Unigene115890_All//7.00E-37//AT5G60640| ATPDIL1-4 (PDI-LIKE 1-4); protein disulfide isomerase			ko04141|Protein processing in endoplasmic reticulum		nr -
Unigene115892_All	686	0.00	0.00	0.00	0.00	0.00	0.00	1.85	2.13	0.00	null	null		null	null		null	null		null	null		2.06E-01	6.81E-01		-1.09E+01	1.99E-09	Down							
Unigene115893_All	756	0.00	0.00	0.00	0.00	0.00	0.00	0.78	3.48	0.00	null	null		null	null		null	null		null	null		2.15E+00	6.92E-08	Up	-9.61E+00	1.41E-04	Down	Unigene115893_All//gi|30687814|ref|NP_849698.1|//8.00E-74//GRF10 (GENERAL REGULATORY FACTOR 10); ATP binding / protein binding / protein phosphorylated amino acid binding [Arabidopsis thaliana] >gi|9392684|gb|AAF87261.1|AC068562_8 Identical to 14-3-3 protein GF14 epsilon (GRF10) from Arabidopsis thaliana gb|AF145302 and contains a 14-3-3 protein PF|00244 domain. ESTs gb|H37302, gb|T43075, gb|T88323, gb|T41936, gb|R87021, gb|N37965, gb|AI994245, gb|Z46557, gb|T20402, gb|T44175, gb|T88028 come from this gene >gi|222423202|dbj|BAH19578.1| AT1G22300 [Arabidopsis thaliana]	Unigene115893_All//2.00E-57//AT1G22300| GRF10 (GENERAL REGULATORY FACTOR 10); ATP binding / protein binding / protein phosphorylated amino acid binding					nr +
Unigene115894_All	776	0.00	0.00	0.00	0.00	0.00	0.00	0.11	5.22	0.10	null	null		null	null		null	null		null	null		5.58E+00	1.78E-25	Up	-8.59E-02	9.73E-01								ESTscan -
Unigene115895_All	834	0.00	0.00	0.00	0.00	0.00	0.00	0.15	3.46	0.00	null	null		null	null		null	null		null	null		4.51E+00	1.91E-16	Up	-7.25E+00	2.00E-01								ESTscan +
Unigene115896_All	830	0.00	0.00	0.00	0.00	0.00	0.00	0.87	1.36	0.00	null	null		null	null		null	null		null	null		6.51E-01	2.66E-01		-9.76E+00	1.78E-05	Down	Unigene115896_All//gi|299470262|emb|CBN79566.1|//5.00E-14//RecName: Full=Spherulin-4; Flags: Precursor [Ectocarpus siliculosus]						nr +
Unigene115897_All	1249	0.00	0.00	0.00	0.00	0.00	0.00	1.02	8.37	1.40	null	null		null	null		null	null		null	null		3.04E+00	8.82E-43	Up	4.67E-01	2.88E-01		Unigene115897_All//gi|15242438|ref|NP_196515.1|//2.00E-94//hydroxyproline-rich glycoprotein family protein [Arabidopsis thaliana] >gi|7671436|emb|CAB89377.1| periaxin-like protein [Arabidopsis thaliana]	Unigene115897_All//6.00E-05//AT1G10390| nucleoporin family protein	Unigene115897_All//4.00E-53//DW500838		ko03040|Spliceosome//ko03018|RNA degradation		nr +
Unigene1159_All	2546	0.49	0.08	0.08	1.15	0.95	2.00	8.57	5.91	8.23	-2.63E+00	3.16E-04	Down	-2.56E+00	6.99E-04	Down	-2.86E-01	4.66E-01		7.96E-01	2.52E-03		-5.36E-01	2.88E-07		-5.81E-02	6.35E-01		Unigene1159_All//gi|4235644|gb|AAD13304.1|//5.00E-51//polyprotein [Solanum lycopersicum]		Unigene1159_All//0.00E+00//TC171648			GO:0003676//GO:0006259//GO:0016740//GO:0019538//GO:0043229//GO:0046872	nr -
Unigene11590_All	2947	0.27	1.03	0.63	0.73	0.65	0.97	2.01	1.76	2.46	1.95E+00	1.22E-06	Up	1.25E+00	1.41E-02		-1.51E-01	7.68E-01		4.10E-01	3.09E-01		-1.92E-01	4.28E-01		2.93E-01	1.38E-01		Unigene11590_All//gi|15239902|ref|NP_199169.1|//1.00E-108//meprin and TRAF homology domain-containing protein / MATH domain-containing protein [Arabidopsis thaliana] >gi|30694322|ref|NP_851125.1| meprin and TRAF homology domain-containing protein / MATH domain-containing protein [Arabidopsis thaliana] >gi|19310439|gb|AAL84956.1| AT5g43560/K9D7_6 [Arabidopsis thaliana]	Unigene11590_All//3.00E-133//AT5G43560| meprin and TRAF homology domain-containing protein / MATH domain-containing protein	Unigene11590_All//0.00E+00//TC176246				nr -
Unigene115938_All	201	0.00	0.00	0.00	0.00	0.00	0.00	29.45	9.98	4.96	null	null		null	null		null	null		null	null		-1.56E+00	3.54E-11	Down	-2.57E+00	2.08E-21	Down							
Unigene115957_All	202	0.78	0.50	0.26	0.00	0.34	1.12	11.51	8.69	0.99	-6.31E-01	7.28E-01		-1.56E+00	5.34E-01		8.41E+00	6.13E-01		1.01E+01	1.19E-01		-4.06E-01	3.06E-01		-3.55E+00	7.48E-12	Down			Unigene115957_All//1.00E-101//gi|164354782|gb|ES805273.1|ES805273				
Unigene115964_All	202	0.00	0.00	0.00	0.00	0.00	0.00	23.02	3.72	0.99	null	null		null	null		null	null		null	null		-2.63E+00	8.61E-17	Down	-4.55E+00	1.75E-27	Down							
Unigene115965_All	202	0.00	0.50	0.79	0.25	0.00	0.00	2.09	8.90	3.75	8.97E+00	3.89E-01		9.63E+00	2.39E-01		-7.95E+00	5.60E-01		-7.95E+00	6.41E-01		2.09E+00	2.01E-05	Up	8.40E-01	2.38E-01				Unigene115965_All//1.00E-12//TC164872				
Unigene115967_All	202	0.00	0.00	0.00	0.00	0.00	0.00	2.72	0.83	0.00	null	null		null	null		null	null		null	null		-1.72E+00	7.23E-02		-1.14E+01	2.81E-04	Down	Unigene115967_All//gi|298706692|emb|CBJ34222.1|//1.00E-07//conserved unknown protein [Ectocarpus siliculosus] >gi|298713886|emb|CBJ33762.1| conserved unknown protein [Ectocarpus siliculosus]		Unigene115967_All//1.00E-15//TC148521				nr +
Unigene1160_All	1238	14.41	38.10	20.60	9.29	11.63	13.39	8.88	7.97	22.30	1.40E+00	3.20E-58	Up	5.16E-01	3.33E-06		3.23E-01	2.96E-02		5.27E-01	8.33E-05		-1.56E-01	3.84E-01		1.33E+00	1.15E-39	Up	Unigene1160_All//gi|15451126|gb|AAK96834.1|//1.00E-107//Unknown protein [Arabidopsis thaliana]	Unigene1160_All//1.00E-108//AT1G70480| unknown protein	Unigene1160_All//0.00E+00//gi|78336205|gb|DT556479.1|DT556479			GO:0005488	nr -
Unigene116005_All	204	0.00	0.00	0.00	1.95	3.38	1.99	2.28	8.40	6.05	null	null		null	null		7.89E-01	4.04E-01		2.85E-02	9.85E-01		1.88E+00	1.20E-04	Up	1.41E+00	9.74E-03		Unigene116005_All//gi|6651292|gb|AAF22256.1|//8.00E-24//myb-related transcription factor [Pimpinella brachycarpa]	Unigene116005_All//1.00E-24//AT4G22680| MYB85 (myb domain protein 85); DNA binding / transcription activator/ transcription factor	Unigene116005_All//2.00E-51//gi|78326281|gb|DT546555.1|DT546555	MYB			nr -
Unigene116009_All	204	0.00	0.00	0.00	0.00	0.00	0.00	1.04	31.96	0.78	null	null		null	null		null	null		null	null		4.95E+00	1.31E-38	Up	-4.08E-01	7.56E-01								
Unigene116016_All	204	0.00	0.00	0.00	0.73	1.01	0.00	0.00	3.28	0.59	null	null		null	null		4.67E-01	8.25E-01		-9.52E+00	2.34E-01		1.17E+01	7.64E-05	Up	9.19E+00	2.43E-01				Unigene116016_All//1.00E-49//ES809411				
Unigene116041_All	205	0.00	0.00	0.00	0.00	0.00	0.00	59.81	15.09	2.53	null	null		null	null		null	null		null	null		-1.99E+00	6.50E-31	Down	-4.57E+00	8.01E-72	Down			Unigene116041_All//2.00E-07//TC171709				

Unigene11605_All	2133	0.93	1.00	0.62	0.68	1.42	1.61	2.62	2.02	3.94	9.81E-02	8.17E-01		-5.79E-01	2.40E-01		1.07E+00	2.31E-03		1.25E+00	2.28E-04	Up	-3.75E-01	1.10E-01		5.91E-01	6.41E-04		Unigene11605_All//gi|18390971|ref|NP_563834.1|//1.00E-169//SNF4 (HOMOLOG OF YEAST SUCROSE NONFERMENTING 4); protein kinase activator [Arabidopsis thaliana] >gi|16612255|gb|AAL27498.1|AF439826_1 At1g09020/F7G19_11 [Arabidopsis thaliana] >gi|23308443|gb|AAN18191.1| At1g09020/F7G19_11 [Arabidopsis thaliana] >gi|75037070|gb|ABA12450.1| AKINbetagamma [Arabidopsis thaliana]	Unigene11605_All//9.00E-171//AT1G09020| SNF4 (HOMOLOG OF YEAST SUCROSE NONFERMENTING 4); protein kinase activator	Unigene11605_All//0.00E+00//TC145866				nr +
Unigene116050_All	206	0.00	0.00	0.00	0.00	0.33	0.00	0.00	4.06	0.39	null	null		null	null		8.39E+00	6.13E-01		null	null		1.20E+01	5.54E-06	Up	8.60E+00	4.05E-01				Unigene116050_All//2.00E-07//FL576731				
Unigene11606_All	662	5.85	5.05	6.68	11.89	12.28	9.14	10.03	6.06	4.21	-2.11E-01	5.23E-01		1.91E-01	5.57E-01		4.62E-02	8.49E-01		-3.79E-01	7.74E-02		-7.26E-01	3.45E-04		-1.25E+00	1.88E-09	Down			Unigene11606_All//2.00E-90//DW504126				
Unigene116060_All	206	123.44	59.03	109.86	30.71	41.12	33.76	0.41	1.62	0.58	-1.06E+00	7.19E-21	Down	-1.68E-01	1.68E-01		4.21E-01	3.36E-02		1.37E-01	6.32E-01		1.98E+00	1.45E-01		4.99E-01	8.08E-01		Unigene116060_All//gi|21616072|emb|CAC87810.2|//1.00E-06//Tic62 protein [Pisum sativum]						nr +
Unigene116066_All	207	1.01	0.73	0.77	2.41	2.16	1.53	4.90	2.22	0.19	-4.61E-01	7.58E-01		-3.90E-01	8.08E-01		-1.54E-01	8.50E-01		-6.56E-01	5.50E-01		-1.14E+00	6.62E-02		-4.67E+00	1.72E-06	Down			Unigene116066_All//3.00E-93//gi|109889353|gb|DW518321.1|DW518321				
Unigene11609_All	1581	5.66	1.06	1.68	17.39	12.22	8.00	26.37	11.95	19.20	-2.42E+00	1.09E-23	Down	-1.75E+00	4.09E-15	Down	-5.09E-01	7.16E-08		-1.12E+00	2.80E-26	Down	-1.14E+00	4.33E-46	Down	-4.58E-01	2.28E-10		Unigene11609_All//gi|224092698|ref|XP_002309702.1|//2.00E-38//cc-nbs-lrr resistance protein [Populus trichocarpa] >gi|222855678|gb|EEE93225.1| cc-nbs-lrr resistance protein [Populus trichocarpa]	Unigene11609_All//5.00E-07//AT1G69545| leucine-rich repeat family protein	Unigene11609_All//0.00E+00//TC130797				nr -
Unigene116093_All	208	0.00	0.49	0.00	0.00	0.00	0.00	1.22	6.23	31.41	8.93E+00	3.89E-01		null	null		null	null		null	null		2.35E+00	1.25E-04	Up	4.69E+00	3.16E-38	Up	Unigene116093_All//gi|15234693|ref|NP_193934.1|//5.00E-08//F-box family protein [Arabidopsis thaliana]	Unigene116093_All//2.00E-10//AT4G04480| LOCATED IN: chloroplast; EXPRESSED IN: flower, pollen tube; EXPRESSED DURING: petal differentiation and expansion stage; BEST Arabidopsis thaliana protein match is: F-box family protein (TAIR:AT4G22030.1); Has 51 Blast hits to 51 proteins in 4 species: Archae - 0; Bacteria - 0; Metazoa - 0; Fungi - 0; Plants - 51; Viruses - 0; Other Eukaryotes - 0 (source: NCBI BLink).					nr -
Unigene1161_All	1516	14.98	43.02	18.44	7.52	14.17	15.91	6.89	9.70	19.47	1.52E+00	1.47E-90	Up	3.00E-01	5.71E-03		9.14E-01	4.64E-16		1.08E+00	1.56E-21	Up	4.94E-01	1.42E-04		1.50E+00	1.23E-50	Up	Unigene1161_All//gi|15451126|gb|AAK96834.1|//1.00E-103//Unknown protein [Arabidopsis thaliana]	Unigene1161_All//6.00E-95//AT1G70480| unknown protein	Unigene1161_All//0.00E+00//TC162925			GO:0005488	nr +
Unigene11610_All	963	7.93	0.89	3.26	17.95	13.70	8.07	23.09	11.54	13.32	-3.15E+00	1.03E-26	Down	-1.28E+00	1.87E-08	Down	-3.90E-01	2.06E-03		-1.15E+00	1.67E-17	Down	-1.00E+00	2.80E-20	Down	-7.94E-01	1.99E-14		Unigene11610_All//gi|224069142|ref|XP_002302910.1|//2.00E-20//cc-nbs-lrr resistance protein [Populus trichocarpa] >gi|222844636|gb|EEE82183.1| cc-nbs-lrr resistance protein [Populus trichocarpa]	Unigene11610_All//6.00E-06//AT1G69545| leucine-rich repeat family protein	Unigene11610_All//0.00E+00//TC130797				nr +
Unigene116102_All	209	0.00	0.00	0.00	0.00	0.00	0.00	47.34	14.00	0.76	null	null		null	null		null	null		null	null		-1.76E+00	2.31E-21	Down	-5.96E+00	6.24E-66	Down							
Unigene116116_All	209	0.00	0.00	0.00	0.24	0.00	0.00	1.42	8.40	1.72	null	null		null	null		-7.90E+00	5.60E-01		-7.90E+00	6.41E-01		2.57E+00	1.41E-06	Up	2.77E-01	7.87E-01				Unigene116116_All//2.00E-85//TC141238				
Unigene11612_All	2168	3.23	1.43	2.24	10.80	7.78	6.37	7.82	6.01	6.39	-1.18E+00	2.46E-07	Down	-5.33E-01	2.19E-02		-4.73E-01	5.52E-06		-7.61E-01	5.51E-12		-3.79E-01	1.81E-03		-2.90E-01	1.55E-02		Unigene11612_All//gi|224092702|ref|XP_002309704.1|//9.00E-96//nbs-lrr resistance protein [Populus trichocarpa] >gi|222855680|gb|EEE93227.1| nbs-lrr resistance protein [Populus trichocarpa]	Unigene11612_All//4.00E-40//AT3G14460| disease resistance protein (NBS-LRR class), putative	Unigene11612_All//0.00E+00//TC177573		ko04626|Plant-pathogen interaction		nr -
Unigene11613_All	3840	20.11	43.32	19.84	57.87	79.86	67.38	48.34	71.30	115.88	1.11E+00	7.63E-141	Up	-1.93E-02	7.84E-01		4.65E-01	5.27E-69		2.20E-01	3.53E-13		5.61E-01	1.71E-88		1.26E+00	0.00E+00	Up	Unigene11613_All//gi|224106445|ref|XP_002314168.1|//0.00E+00//GRAS family transcription factor [Populus trichocarpa] >gi|222850576|gb|EEE88123.1| GRAS family transcription factor [Populus trichocarpa]	Unigene11613_All//1.00E-149//AT3G46600| scarecrow transcription factor family protein	Unigene11613_All//0.00E+00//TC170524	GRAS	ko03018|RNA degradation		nr +
Unigene116131_All	210	0.00	0.00	0.00	0.00	0.00	0.00	0.20	4.58	2.47	null	null		null	null		null	null		null	null		4.51E+00	8.92E-06	Up	3.61E+00	4.19E-03				Unigene116131_All//2.00E-07//TC177400				
Unigene116147_All	211	0.00	0.00	0.00	0.00	0.00	0.00	0.00	3.96	0.57	null	null		null	null		null	null		null	null		1.20E+01	5.54E-06	Up	9.15E+00	2.43E-01				Unigene116147_All//2.00E-07//EX166575				
Unigene116157_All	212	10.86	10.99	11.05	9.40	6.01	11.08	4.19	11.63	8.46	1.79E-02	9.86E-01		2.54E-02	9.71E-01		-6.45E-01	1.53E-01		2.37E-01	6.36E-01		1.47E+00	9.56E-05	Up	1.01E+00	1.67E-02				Unigene116157_All//3.00E-65//TC145750				
Unigene116169_All	212	0.00	0.00	0.00	0.00	0.00	0.00	0.00	4.34	0.00	null	null		null	null		null	null		null	null		1.21E+01	1.48E-06	Up	null	null								
Unigene116179_All	213	0.00	0.00	0.00	0.00	0.00	0.21	1.39	2.94	6.73	null	null		null	null		null	null		7.73E+00	7.00E-01		1.08E+00	1.99E-01		2.28E+00	4.94E-05	Up			Unigene116179_All//7.00E-51//TC150953				
Unigene11618_All	1427	3.63	2.59	2.05	37.32	32.63	28.90	7.02	2.28	3.02	-4.86E-01	6.66E-02		-8.23E-01	2.70E-03		-1.94E-01	6.74E-03		-3.69E-01	1.36E-07		-1.62E+00	2.35E-19	Down	-1.22E+00	2.59E-13	Down	Unigene11618_All//gi|297833894|ref|XP_002884829.1|//1.00E-164//steroid hormone receptor/ transcription factor [Arabidopsis lyrata subsp. lyrata] >gi|297330669|gb|EFH61088.1| steroid hormone receptor/ transcription factor [Arabidopsis lyrata subsp. lyrata]	Unigene11618_All//0.00E+00//AT2G40320| unknown protein	Unigene11618_All//0.00E+00//TC173302				nr -
Unigene11619_All	1555	2.22	2.93	3.32	23.29	20.77	15.68	8.24	2.53	4.87	4.01E-01	1.65E-01		5.81E-01	3.56E-02		-1.65E-01	7.87E-02		-5.70E-01	3.54E-11		-1.71E+00	2.50E-26	Down	-7.59E-01	4.10E-08		Unigene11619_All//gi|297833894|ref|XP_002884829.1|//1.00E-143//steroid hormone receptor/ transcription factor [Arabidopsis lyrata subsp. lyrata] >gi|297330669|gb|EFH61088.1| steroid hormone receptor/ transcription factor [Arabidopsis lyrata subsp. lyrata]	Unigene11619_All//4.00E-149//AT2G40320| unknown protein	Unigene11619_All//0.00E+00//TC135572				nr -
Unigene116197_All	214	0.49	0.00	0.50	1.86	0.64	1.06	91.87	61.13	18.43	-8.93E+00	3.75E-01		2.54E-02	1.00E+00		-1.53E+00	2.06E-01		-8.20E-01	5.07E-01		-5.88E-01	1.11E-07		-2.32E+00	1.97E-61	Down	Unigene116197_All//gi|211906506|gb|ACJ11746.1|//1.00E-11//luminal binding protein [Gossypium hirsutum]	Unigene116197_All//5.00E-13//AT5G42020| BIP2; ATP binding	Unigene116197_All//1.00E-42//TC165456		ko04141|Protein processing in endoplasmic reticulum//ko03060|Protein export		nr -
Unigene116211_All	215	0.00	0.94	0.00	1.62	2.72	0.42	1.38	4.67	6.49	9.88E+00	1.37E-01		null	null		7.47E-01	4.81E-01		-1.95E+00	1.96E-01		1.76E+00	7.67E-03		2.24E+00	7.92E-05	Up			Unigene116211_All//3.00E-105//CO103526				
Unigene116213_All	215	0.00	0.00	0.00	0.00	0.00	0.00	117.01	294.90	122.67	null	null		null	null		null	null		null	null		1.33E+00	1.32E-88	Up	6.81E-02	5.52E-01				Unigene116213_All//8.00E-29//TC159659				ESTscan -
Unigene116222_All	216	0.73	1.41	0.49	0.00	0.00	4.60	1.17	3.68	0.00	9.54E-01	5.04E-01		-5.60E-01	7.76E-01		null	null		1.22E+01	6.15E-06	Up	1.65E+00	2.88E-02		-1.02E+01	3.04E-02								
Unigene116224_All	216	0.00	0.00	0.00	0.00	0.00	0.00	4.89	0.77	0.18	null	null		null	null		null	null		null	null		-2.66E+00	2.25E-04	Down	-4.73E+00	8.85E-07	Down							
Unigene116226_All	216	0.00	0.00	0.00	0.00	0.00	0.00	0.00	4.64	0.00	null	null		null	null		null	null		null	null		1.22E+01	3.93E-07	Up	null	null								
Unigene11624_All	4341	5.60	12.94	9.54	5.34	7.64	7.84	12.56	17.83	19.04	1.21E+00	4.71E-55	Up	7.68E-01	6.55E-19		5.18E-01	2.08E-09		5.56E-01	5.00E-10		5.05E-01	2.56E-21		6.00E-01	1.86E-31		Unigene11624_All//gi|186529839|ref|NP_001119383.1|//1.00E-110//NLI interacting factor (NIF) family protein [Arabidopsis thaliana]	Unigene11624_All//1.00E-111//AT5G46410| NLI interacting factor (NIF) family protein	Unigene11624_All//0.00E+00//TC167376				nr -
Unigene116240_All	217	0.96	3.97	0.25	3.21	2.38	2.50	2.34	3.27	8.08	2.04E+00	1.59E-02		-1.97E+00	3.82E-01		-4.33E-01	6.06E-01		-3.64E-01	6.81E-01		4.86E-01	5.42E-01		1.79E+00	1.28E-04	Up			Unigene116240_All//2.00E-20//AW186738				
Unigene116252_All	217	0.48	7.01	1.23	1.61	0.79	0.62	1.95	3.08	1.29	3.86E+00	1.23E-06	Up	1.35E+00	4.09E-01		-1.02E+00	4.23E-01		-1.36E+00	3.37E-01		6.61E-01	4.02E-01		-6.00E-01	5.53E-01		Unigene116252_All//gi|111038269|gb|ABH03531.1|//1.00E-11//class II knotted-like homeobox protein [Prunus persica] >gi|215983122|gb|ACJ71731.1| class II knotted-like homeobox transcription factor [Prunus persica]	Unigene116252_All//5.00E-13//AT5G25220| KNAT3 (KNOTTED1-LIKE HOMEOBOX GENE 3); transcription activator/ transcription factor	Unigene116252_All//4.00E-55//gi|11201779|gb|BF270784.1|BF270784	HB			nr +
Unigene116278_All	220	0.00	2.30	0.00	0.45	0.00	0.21	0.96	4.75	0.91	1.12E+01	4.05E-03		null	null		-8.82E+00	3.15E-01		-1.14E+00	6.78E-01		2.31E+00	8.56E-04	Up	-8.58E-02	9.61E-01								
Unigene116279_All	220	16.89	4.61	8.47	49.36	61.67	29.97	26.52	8.93	28.79	-1.87E+00	1.28E-07	Down	-9.95E-01	2.95E-03		3.21E-01	3.73E-02		-7.20E-01	1.94E-05		-1.57E+00	4.03E-11	Down	1.19E-01	6.04E-01				Unigene116279_All//3.00E-65//TC129604				
Unigene116297_All	220	0.00	0.00	0.00	0.00	0.00	0.00	0.38	6.84	4.71	null	null		null	null		null	null		null	null		4.15E+00	2.17E-08	Up	3.61E+00	1.38E-05	Up							
Unigene1163_All	3626	2.40	0.88	1.60	4.40	3.41	3.18	8.12	3.82	4.38	-1.44E+00	7.73E-12	Down	-5.82E-01	4.37E-03		-3.67E-01	6.26E-03		-4.69E-01	6.29E-04		-1.09E+00	2.08E-30	Down	-8.89E-01	1.33E-22		Unigene1163_All//gi|83423282|emb|CAI64810.1|//0.00E+00//nucleoporin [Lotus japonicus] >gi|83423284|emb|CAI64811.1| nucleoporin [Lotus japonicus]	Unigene1163_All//0.00E+00//AT2G05120| LOCATED IN: chloroplast; EXPRESSED IN: 16 plant structures; EXPRESSED DURING: 9 growth stages; CONTAINS InterPro DOMAIN/s: Nup133, N-terminal (InterPro:IPR014908); Has 103 Blast hits to 103 proteins in 31 species: Archae - 0; Bacteria - 0; Metazoa - 79; Fungi - 0; Plants - 19; Viruses - 0; Other Eukaryotes - 5 (source: NCBI BLink).	Unigene1163_All//0.00E+00//TC168837			GO:0005506//GO:0016491	nr +
Unigene116313_All	221	0.24	1.60	0.48	0.45	0.00	0.20	0.00	1.89	4.33	2.76E+00	9.60E-02		1.03E+00	7.04E-01		-8.82E+00	3.15E-01		-1.14E+00	6.78E-01		1.09E+01	3.65E-03		1.21E+01	5.69E-07	Up							
Unigene116319_All	222	0.00	0.00	0.00	0.67	0.31	0.20	530.41	113.53	188.43	null	null		null	null		-1.12E+00	5.84E-01		-1.73E+00	5.02E-01		-2.22E+00	0.00E+00	Down	-1.49E+00	2.26E-200	Down	Unigene116319_All//gi|87240949|gb|ABD32807.1|//5.00E-36//Major intrinsic protein [Medicago truncatula]	Unigene116319_All//2.00E-37//AT5G60660| PIP2;4 (PLASMA MEMBRANE INTRINSIC PROTEIN 2;4); water channel	Unigene116319_All//4.00E-120//gi|48792282|gb|CO093596.1|CO093596				nr -
Unigene11632_All	2894	0.49	1.54	1.80	1.95	2.09	1.50	1.74	2.53	2.52	1.66E+00	1.15E-07	Up	1.89E+00	5.24E-10	Up	1.07E-01	7.30E-01		-3.77E-01	1.54E-01		5.40E-01	5.19E-03		5.35E-01	4.36E-03		Unigene11632_All//gi|267631890|gb|ACY78680.1|//0.00E+00//calcium-dependent protein kinase 1 [Panax ginseng]	Unigene11632_All//3.00E-179//AT4G04720| CPK21; ATP binding / calcium ion binding / calmodulin-dependent protein kinase/ kinase/ protein kinase/ protein serine/threonine kinase	Unigene11632_All//0.00E+00//TC130026		ko04626|Plant-pathogen interaction	GO:0004674//GO:0006464//GO:0016020//GO:0032559//GO:0046872	nr -
Unigene116324_All	222	31.35	19.40	33.82	44.65	18.92	46.38	1.52	1.88	1.26	-6.92E-01	1.79E-03		1.10E-01	6.24E-01		-1.24E+00	7.32E-13	Down	5.49E-02	7.95E-01		3.05E-01	7.58E-01		-2.78E-01	7.81E-01				Unigene116324_All//3.00E-47//CO099551				
Unigene116329_All	222	0.00	0.00	0.00	0.00	0.16	0.00	0.57	21.46	0.36	null	null		null	null		7.28E+00	8.34E-01		null	null		5.23E+00	4.46E-29	Up	-6.71E-01	7.20E-01		Unigene116329_All//gi|255543735|ref|XP_002512930.1|//5.00E-07//ribonuclease t2, putative [Ricinus communis] >gi|223547941|gb|EEF49433.1| ribonuclease t2, putative [Ricinus communis]	Unigene116329_All//2.00E-06//AT1G14220| ribonuclease T2 family protein					nr -
Unigene11633_All	1770	2.51	2.18	0.93	17.53	15.76	18.01	6.35	4.16	4.25	-2.08E-01	4.94E-01		-1.43E+00	5.11E-06	Down	-1.54E-01	1.44E-01		3.90E-02	7.32E-01		-6.13E-01	4.78E-05		-5.79E-01	8.08E-05		Unigene11633_All//gi|224083155|ref|XP_002306955.1|//0.00E+00//calcium dependent protein kinase 27 [Populus trichocarpa] >gi|222856404|gb|EEE93951.1| calcium dependent protein kinase 27 [Populus trichocarpa]	Unigene11633_All//0.00E+00//AT1G76040| CPK29; ATP binding / calcium ion binding / calmodulin-dependent protein kinase/ kinase/ protein kinase/ protein serine/threonine kinase/ protein tyrosine kinase	Unigene11633_All//0.00E+00//ES824808		ko04626|Plant-pathogen interaction	GO:0004674//GO:0006464//GO:0016020//GO:0032559//GO:0046872	nr -
Unigene11638_All	1197	27.62	54.99	26.83	31.54	27.50	31.81	39.07	31.63	12.65	9.93E-01	4.34E-47		-4.24E-02	6.99E-01		-1.98E-01	2.46E-02		1.19E-02	9.30E-01		-3.04E-01	1.18E-05		-1.63E+00	1.11E-90	Down	Unigene11638_All//gi|195608970|gb|ACG26315.1|//3.00E-75//calcyclin-binding protein [Zea mays]	Unigene11638_All//3.00E-73//AT1G30070| SGS domain-containing protein	Unigene11638_All//0.00E+00//gi|84149129|gb|DN780429.1|DN780429			GO:0016020	nr -
Unigene116384_All	226	0.00	0.22	0.00	0.00	0.00	0.00	85.31	22.19	5.29	7.81E+00	6.19E-01		null	null		null	null		null	null		-1.94E+00	8.63E-47	Down	-4.01E+00	2.79E-103	Down	Unigene116384_All//gi|48675345|dbj|BAD22820.1|//2.00E-27//nitrate transporter [Prunus persica]	Unigene116384_All//4.00E-11//AT1G12110| NRT1.1; nitrate transmembrane transporter/ transporter	Unigene116384_All//4.00E-77//TC165574				nr -
Unigene11639_All	1506	11.95	8.34	12.09	15.94	17.67	13.02	16.56	5.02	9.13	-5.18E-01	6.77E-05		1.70E-02	9.19E-01		1.49E-01	2.09E-01		-2.93E-01	9.77E-03		-1.72E+00	3.25E-51	Down	-8.60E-01	2.66E-18		Unigene11639_All//gi|222423637|dbj|BAH19787.1|//1.00E-168//AT2G34560 [Arabidopsis thaliana] >gi|222423678|dbj|BAH19806.1| AT2G34560 [Arabidopsis thaliana]	Unigene11639_All//5.00E-169//AT2G34560| katanin, putative	Unigene11639_All//0.00E+00//EX164211			GO:0032559//GO:0042623	nr +

<table>
<tr><td>Unigene116399_All</td><td>227</td><td>14827.08</td><td>6527.21</td><td>17871.35</td><td>3686.03</td><td>4388.91</td><td>3285.39</td><td>0.56</td><td>0.92</td><td>0.18</td><td>-1.18E+00</td><td>0.00E+00</td><td>Down</td><td>2.69E-01</td><td>7.59E-265</td><td></td><td>2.52E-01</td><td>3.71E-71</td><td></td><td>-1.66E-01</td><td>2.33E-24</td><td></td><td>7.20E-01</td><td>6.32E-01</td><td></td><td>-1.67E+00</td><td>4.78E-01</td><td></td><td>Unigene116399_All//gi|224072580|ref|XP_002303791.1|//4.00E-25//light-harvesting complex I protein Lhca2 [Populus trichocarpa] >gi|222841223|gb|EEE78770.1| light-harvesting complex I protein Lhca2 [Populus trichocarpa]</td><td>Unigene116399_All//2.00E-09//AT3G61470| LHCA2; chlorophyll binding</td><td>Unigene116399_All//2.00E-125//TC131837</td><td></td><td>ko00196|Photosynthesis - antenna proteins</td><td></td><td>nr -</td></tr>
<tr><td>Unigene11640_All</td><td>2476</td><td>8.14</td><td>12.52</td><td>9.25</td><td>9.64</td><td>8.57</td><td>9.36</td><td>10.62</td><td>4.36</td><td>3.01</td><td>6.22E-01</td><td>1.58E-10</td><td></td><td>1.85E-01</td><td>1.47E-01</td><td></td><td>-1.70E-01</td><td>1.61E-01</td><td></td><td>-4.25E-02</td><td>7.48E-01</td><td></td><td>-1.29E+00</td><td>2.81E-35</td><td>Down</td><td>-1.82E+00</td><td>5.25E-60</td><td>Down</td><td>Unigene11640_All//gi|56201862|dbj|BAD73312.1|//1.00E-159//vacuolar protein sorting factor 4B-like [Oryza sativa Japonica Group] >gi|56201915|dbj|BAD73365.1| vacuolar protein sorting factor 4B-like [Oryza sativa Japonica Group]</td><td>Unigene11640_All//8.00E-159//AT2G34560| katanin, putative</td><td>Unigene11640_All//0.00E+00//gi|78333495|gb|DT553769.1|DT553769</td><td></td><td></td><td>GO:0032559//GO:0042623</td><td>nr +</td></tr>
<tr><td>Unigene11641_All</td><td>1428</td><td>3.22</td><td>3.16</td><td>3.10</td><td>125.27</td><td>106.34</td><td>96.84</td><td>102.15</td><td>262.36</td><td>60.04</td><td>-3.00E-02</td><td>9.30E-01</td><td></td><td>-5.90E-02</td><td>8.43E-01</td><td></td><td>-2.36E-01</td><td>9.50E-12</td><td></td><td>-3.71E-01</td><td>2.70E-24</td><td></td><td>1.36E+00</td><td>0.00E+00</td><td>Up</td><td>-7.67E-01</td><td>2.07E-84</td><td></td><td>Unigene11641_All//gi|224134154|ref|XP_002327769.1|//0.00E+00//coniferylaldehyde 5- hydroxylase [Populus trichocarpa] >gi|222836854|gb|EEE75247.1| coniferylaldehyde 5- hydroxylase [Populus trichocarpa]</td><td>Unigene11641_All//0.00E+00//AT4G36220| FAH1 (FERULIC ACID 5-HYDROXYLASE 1); ferulate 5-hydroxylase/ monooxygenase</td><td>Unigene11641_All//0.00E+00//DW225229</td><td></td><td>ko00940|Phenylpropanoid biosynthesis//ko00941|Flavonoid biosynthesis//ko00944|Flavone and flavonol biosynthesis</td><td>GO:0005506//GO:0008152//GO:0016491</td><td>nr +</td></tr>
<tr><td>Unigene116417_All</td><td>228</td><td>0.00</td><td>0.44</td><td>0.00</td><td>0.00</td><td>0.00</td><td>0.00</td><td>0.74</td><td>4.03</td><td>4.19</td><td>8.80E+00</td><td>3.89E-01</td><td></td><td>null</td><td>null</td><td></td><td>null</td><td>null</td><td></td><td>null</td><td>null</td><td></td><td>2.44E+00</td><td>1.33E-03</td><td></td><td>2.50E+00</td><td>6.42E-04</td><td>Up</td><td>Unigene116417_All//gi|297737079|emb|CBI26280.3|//1.00E-10//unnamed protein product [Vitis vinifera]</td><td></td><td>Unigene116417_All//2.00E-26//gi|54121950|gb|CO497459.1|CO497459</td><td></td><td></td><td></td><td>nr +</td></tr>
<tr><td>Unigene116418_All</td><td>228</td><td>0.00</td><td>0.00</td><td>0.00</td><td>0.00</td><td>0.00</td><td>0.00</td><td>0.00</td><td>10.27</td><td>0.17</td><td>null</td><td>null</td><td></td><td>null</td><td>null</td><td></td><td>null</td><td>null</td><td></td><td>null</td><td>null</td><td></td><td>1.33E+01</td><td>1.83E-16</td><td>Up</td><td>7.45E+00</td><td>6.46E-01</td><td></td><td></td><td></td><td></td><td></td><td></td><td></td><td></td></tr>
<tr><td>Unigene11642_All</td><td>1863</td><td>3.43</td><td>4.13</td><td>2.77</td><td>79.95</td><td>70.44</td><td>63.35</td><td>6.29</td><td>1.97</td><td>27.71</td><td>2.71E-01</td><td>2.20E-01</td><td></td><td>-3.06E-01</td><td>2.39E-01</td><td></td><td>-1.83E-01</td><td>3.21E-06</td><td></td><td>-3.36E-01</td><td>6.26E-17</td><td></td><td>-1.67E+00</td><td>1.80E-23</td><td>Down</td><td>2.14E+00</td><td>3.89E-143</td><td>Up</td><td>Unigene11642_All//gi|47933890|gb|AAT39511.1|//0.00E+00//ferulate 5-hydroxylase [Camptotheca acuminata]</td><td>Unigene11642_All//0.00E+00//AT4G36220| FAH1 (FERULIC ACID 5-HYDROXYLASE 1); ferulate 5-hydroxylase/ monooxygenase</td><td>Unigene11642_All//0.00E+00//TC152108</td><td></td><td>ko00940|Phenylpropanoid biosynthesis//ko00941|Flavonoid biosynthesis//ko00944|Flavone and flavonol biosynthesis</td><td>GO:0005506//GO:0008152//GO:0016491</td><td>nr -</td></tr>
<tr><td>Unigene116432_All</td><td>229</td><td>0.00</td><td>0.00</td><td>0.00</td><td>0.00</td><td>0.00</td><td>0.00</td><td>0.00</td><td>2.74</td><td>0.00</td><td>null</td><td>null</td><td></td><td>null</td><td>null</td><td></td><td>null</td><td>null</td><td></td><td>null</td><td>null</td><td></td><td>1.14E+01</td><td>1.47E-04</td><td>Up</td><td>null</td><td>null</td><td></td><td>Unigene116432_All//gi|114159828|gb|ABI53721.1|//2.00E-18//polyubiquitin [Porphyra yezoensis]</td><td>Unigene116432_All//2.00E-19//AT5G03240| UBQ3 (POLYUBIQUITIN 3); protein binding</td><td>Unigene116432_All//1.00E-30//CD486028</td><td></td><td>ko03010|Ribosome</td><td></td><td>nr -</td></tr>
<tr><td>Unigene11644_All</td><td>2059</td><td>0.81</td><td>1.65</td><td>0.16</td><td>1.11</td><td>1.05</td><td>0.92</td><td>9.75</td><td>21.37</td><td>37.30</td><td>1.02E+00</td><td>2.66E-03</td><td></td><td>-2.39E+00</td><td>1.21E-04</td><td>Down</td><td>-7.90E-02</td><td>8.45E-01</td><td></td><td>-2.73E-01</td><td>5.68E-01</td><td></td><td>1.13E+00</td><td>7.16E-48</td><td>Up</td><td>1.94E+00</td><td>1.27E-186</td><td>Up</td><td>Unigene11644_All//gi|297818990|ref|XP_002877378.1|//0.00E+00//phosphatidate cytidylyltransferase family protein [Arabidopsis lyrata subsp. lyrata] >gi|297323216|gb|EFH53637.1| phosphatidate cytidylyltransferase family protein [Arabidopsis lyrata subsp. lyrata]</td><td>Unigene11644_All//0.00E+00//AT3G45040| phosphatidate cytidylyltransferase family protein</td><td>Unigene11644_All//0.00E+00//TC157004</td><td></td><td>ko00510|N-Glycan biosynthesis</td><td>GO:0016301//GO:0031224</td><td>nr -</td></tr>
<tr><td>Unigene116447_All</td><td>230</td><td>0.00</td><td>1.98</td><td>0.00</td><td>0.00</td><td>0.75</td><td>0.20</td><td>0.37</td><td>2.91</td><td>5.89</td><td>1.10E+01</td><td>7.44E-03</td><td></td><td>null</td><td>null</td><td></td><td>9.55E+00</td><td>2.27E-01</td><td></td><td>7.62E+00</td><td>6.99E-01</td><td></td><td>2.98E+00</td><td>2.86E-03</td><td></td><td>4.00E+00</td><td>1.31E-07</td><td>Up</td><td></td><td></td><td>Unigene116447_All//1.00E-104//CO108092</td><td></td><td></td><td></td><td></td></tr>
<tr><td>Unigene116461_All</td><td>231</td><td>1.81</td><td>0.88</td><td>0.23</td><td>4.10</td><td>0.60</td><td>2.54</td><td>1.28</td><td>3.26</td><td>0.17</td><td>-1.05E+00</td><td>3.66E-01</td><td></td><td>-2.97E+00</td><td>6.46E-02</td><td></td><td>-2.78E+00</td><td>4.58E-04</td><td>Down</td><td>-6.89E-01</td><td>3.58E-01</td><td></td><td>1.35E+00</td><td>7.61E-02</td><td></td><td>-2.89E+00</td><td>6.77E-02</td><td></td><td>Unigene116461_All//gi|32362295|gb|AAP80175.1|//2.00E-12//At3g55350 [Arabidopsis thaliana]</td><td>Unigene116461_All//8.00E-14//AT3G55350| unknown protein</td><td></td><td></td><td></td><td></td><td>nr -</td></tr>
<tr><td>Unigene116463_All</td><td>231</td><td>0.00</td><td>0.00</td><td>0.00</td><td>0.00</td><td>0.00</td><td>0.00</td><td>0.00</td><td>4.52</td><td>0.00</td><td>null</td><td>null</td><td></td><td>null</td><td>null</td><td></td><td>null</td><td>null</td><td></td><td>null</td><td>null</td><td></td><td>1.21E+01</td><td>2.02E-07</td><td>Up</td><td>null</td><td>null</td><td></td><td></td><td></td><td></td><td></td><td></td><td></td><td></td></tr>
<tr><td>Unigene116465_All</td><td>232</td><td>0.45</td><td>10.05</td><td>5.51</td><td>0.86</td><td>1.48</td><td>3.11</td><td>2.37</td><td>5.58</td><td>1.37</td><td>4.48E+00</td><td>7.48E-11</td><td>Up</td><td>3.61E+00</td><td>3.41E-05</td><td>Up</td><td>7.89E-01</td><td>5.96E-01</td><td></td><td>1.86E+00</td><td>4.71E-02</td><td></td><td>1.24E+00</td><td>2.18E-02</td><td></td><td>-7.86E-01</td><td>3.62E-01</td><td></td><td></td><td></td><td>Unigene116465_All//7.00E-91//TC144883</td><td></td><td></td><td></td><td></td></tr>
<tr><td>Unigene116466_All</td><td>232</td><td>0.00</td><td>0.00</td><td>0.00</td><td>0.00</td><td>0.00</td><td>0.00</td><td>4.37</td><td>7.03</td><td>0.69</td><td>null</td><td>null</td><td></td><td>null</td><td>null</td><td></td><td>null</td><td>null</td><td></td><td>null</td><td>null</td><td></td><td>6.84E-01</td><td>1.42E-01</td><td></td><td>-2.67E+00</td><td>1.94E-04</td><td>Down</td><td></td><td></td><td></td><td></td><td></td><td></td><td></td></tr>
<tr><td>Unigene116467_All</td><td>232</td><td>0.68</td><td>1.97</td><td>0.92</td><td>0.00</td><td>0.15</td><td>0.00</td><td>4.01</td><td>10.09</td><td>1.37</td><td>1.54E+00</td><td>1.96E-01</td><td></td><td>4.40E-01</td><td>7.76E-01</td><td></td><td>7.21E+00</td><td>8.34E-01</td><td></td><td>null</td><td>null</td><td></td><td>1.33E+00</td><td>5.14E-04</td><td>Up</td><td>-1.55E+00</td><td>1.68E-02</td><td></td><td></td><td></td><td>Unigene116467_All//5.00E-126//TC173535</td><td></td><td></td><td></td><td></td></tr>
<tr><td>Unigene116472_All</td><td>232</td><td>0.00</td><td>0.87</td><td>0.23</td><td>1.50</td><td>0.30</td><td>0.19</td><td>8.75</td><td>18.38</td><td>24.56</td><td>9.77E+00</td><td>1.37E-01</td><td></td><td>7.84E+00</td><td>6.39E-01</td><td></td><td>-2.34E+00</td><td>1.01E-01</td><td></td><td>-2.95E+00</td><td>8.31E-02</td><td></td><td>1.07E+00</td><td>5.70E-05</td><td>Up</td><td>1.49E+00</td><td>2.31E-10</td><td>Up</td><td>Unigene116472_All//gi|224130846|ref|XP_002320939.1|//1.00E-13//multidrug/pheromone exporter, MDR family, ABC transporter family [Populus trichocarpa] >gi|222861712|gb|EEE99254.1| multidrug/pheromone exporter, MDR family, ABC transporter family [Populus trichocarpa]</td><td>Unigene116472_All//8.00E-08//AT3G62150| PGP21 (P-GLYCOPROTEIN 21); ATPase, coupled to transmembrane movement of substances</td><td>Unigene116472_All//4.00E-77//gi|73864444|gb|DT467183.1|DT467183</td><td></td><td>ko02010|ABC transporters</td><td></td><td>nr +</td></tr>
<tr><td>Unigene116477_All</td><td>232</td><td>0.00</td><td>0.00</td><td>0.00</td><td>0.00</td><td>0.00</td><td>0.00</td><td>3.83</td><td>2.70</td><td>0.52</td><td>null</td><td>null</td><td></td><td>null</td><td>null</td><td></td><td>null</td><td>null</td><td></td><td>null</td><td>null</td><td></td><td>-5.02E-01</td><td>4.60E-01</td><td></td><td>-2.89E+00</td><td>3.04E-04</td><td>Down</td><td>Unigene116477_All//gi|168068591|ref|XP_001786133.1|//4.00E-09//predicted protein [Physcomitrella patens subsp. patens] >gi|162662081|gb|EDQ49057.1| predicted protein [Physcomitrella patens subsp. patens]</td><td></td><td>Unigene116477_All//1.00E-15//gi|109830702|gb|DW229261.1|DW229261</td><td></td><td></td><td></td><td>nr -</td></tr>
<tr><td>Unigene116484_All</td><td>232</td><td>0.00</td><td>0.66</td><td>0.00</td><td>0.21</td><td>0.89</td><td>0.97</td><td>0.91</td><td>5.95</td><td>0.69</td><td>9.36E+00</td><td>2.36E-01</td><td></td><td>null</td><td>null</td><td></td><td>2.05E+00</td><td>3.95E-01</td><td></td><td>2.18E+00</td><td>3.23E-01</td><td></td><td>2.71E+00</td><td>1.42E-05</td><td>Up</td><td>-4.08E-01</td><td>7.56E-01</td><td></td><td></td><td></td><td>Unigene116484_All//2.00E-125//DW484004</td><td></td><td></td><td></td><td>ESTscan +</td></tr>
<tr><td>Unigene11649_All</td><td>536</td><td>0.00</td><td>0.38</td><td>2.19</td><td>0.84</td><td>0.58</td><td>0.76</td><td>1.34</td><td>0.86</td><td>0.22</td><td>8.56E+00</td><td>1.37E-01</td><td></td><td>1.11E+01</td><td>1.77E-06</td><td>Up</td><td>-5.33E-01</td><td>5.96E-01</td><td></td><td>-1.41E-01</td><td>9.00E-01</td><td></td><td>-6.45E-01</td><td>4.01E-01</td><td></td><td>-2.59E+00</td><td>2.81E-03</td><td></td><td>Unigene11649_All//gi|6739629|gb|AAF27340.1|AF186020_1//3.00E-15//abscisic acid-activated protein kinase [Vicia faba]</td><td>Unigene11649_All//5.00E-16//AT5G66880| SNRK2.3 (SUCROSE NONFERMENTING 1(SNF1)-RELATED PROTEIN KINASE 2.3); kinase/ protein kinase</td><td>Unigene11649_All//7.00E-121//EX169027</td><td></td><td></td><td>GO:0004672//GO:0006464//GO:0006970//GO:0009738//GO:0009845//GO:0032559//GO:0043231//GO:0044444</td><td>nr +</td></tr>
<tr><td>Unigene116497_All</td><td>233</td><td>0.45</td><td>0.43</td><td>0.00</td><td>2.35</td><td>4.88</td><td>2.33</td><td>4.72</td><td>10.40</td><td>11.63</td><td>-4.64E-02</td><td>9.90E-01</td><td></td><td>-8.81E+00</td><td>3.96E-01</td><td></td><td>1.05E+00</td><td>1.10E-01</td><td></td><td>-1.59E-02</td><td>9.75E-01</td><td></td><td>1.14E+00</td><td>2.01E-03</td><td></td><td>1.30E+00</td><td>1.53E-04</td><td>Up</td><td></td><td></td><td>Unigene116497_All//1.00E-33//CO131853</td><td></td><td></td><td></td><td></td></tr>
<tr><td>Unigene116500_All</td><td>233</td><td>1250.76</td><td>522.35</td><td>580.49</td><td>1552.79</td><td>1446.73</td><td>1499.50</td><td>351.67</td><td>142.79</td><td>206.72</td><td>-1.26E+00</td><td>8.13E-304</td><td>Down</td><td>-1.11E+00</td><td>3.62E-240</td><td>Down</td><td>-1.02E-01</td><td>5.64E-05</td><td></td><td>-5.04E-02</td><td>9.66E-02</td><td></td><td>-1.30E+00</td><td>3.85E-110</td><td>Down</td><td>-7.67E-01</td><td>8.58E-48</td><td></td><td>Unigene116500_All//gi|32401253|gb|AAP80800.1|//2.00E-17//class VII chitinase precursor [Gossypium hirsutum] >gi|32401255|gb|AAP80801.1| class VII chitinase precursor [Gossypium hirsutum]</td><td>Unigene116500_All//1.00E-09//AT1G05850| POM1 (POM-POM1); chitinase</td><td>Unigene116500_All//1.00E-126//gi|21100178|gb|BQ412491.1|BQ412491</td><td></td><td></td><td></td><td>nr +</td></tr>
<tr><td>Unigene116504_All</td><td>234</td><td>2.68</td><td>0.87</td><td>1.82</td><td>2.98</td><td>2.06</td><td>1.16</td><td>33.07</td><td>13.22</td><td>10.04</td><td>-1.63E+00</td><td>9.23E-02</td><td></td><td>-5.60E-01</td><td>5.54E-01</td><td></td><td>-5.33E-01</td><td>5.45E-01</td><td></td><td>-1.36E+00</td><td>1.31E-01</td><td></td><td>-1.32E+00</td><td>2.15E-11</td><td>Down</td><td>-1.72E+00</td><td>6.36E-17</td><td>Down</td><td></td><td></td><td>Unigene116504_All//3.00E-56//gi|21093336|gb|BQ405649.1|BQ405649</td><td></td><td></td><td></td><td></td></tr>
<tr><td>Unigene11651_All</td><td>374</td><td>39.59</td><td>80.20</td><td>47.41</td><td>35.16</td><td>37.20</td><td>41.30</td><td>59.92</td><td>67.50</td><td>66.89</td><td>1.02E+00</td><td>9.48E-23</td><td>Up</td><td>2.60E-01</td><td>7.05E-02</td><td></td><td>8.11E-02</td><td>6.61E-01</td><td></td><td>2.32E-01</td><td>1.33E-01</td><td></td><td>1.72E-01</td><td>1.04E-01</td><td></td><td>1.59E-01</td><td>1.21E-01</td><td></td><td>Unigene11651_All//gi|186516051|ref|NP_001119111.1|//2.00E-61//OST1 (OPEN STOMATA 1); calcium-dependent protein serine/threonine kinase/ kinase/ protein kinase [Arabidopsis thaliana]</td><td>Unigene11651_All//8.00E-63//AT4G33950| OST1 (OPEN STOMATA 1); calcium-dependent protein serine/threonine kinase/ kinase/ protein kinase</td><td>Unigene11651_All//0.00E+00//TC142675</td><td></td><td></td><td>GO:0004674//GO:0005984//GO:0006464//GO:0006633//GO:0006641//GO:0006800//GO:0006952//GO:0006970//GO:0009738//GO:0009845//GO:0010118//GO:0032559//GO:0043231//GO:0044444//GO:0048827</td><td>nr +</td></tr>
<tr><td>Unigene116512_All</td><td>234</td><td>0.00</td><td>0.00</td><td>0.46</td><td>2.55</td><td>2.35</td><td>1.93</td><td>1.26</td><td>16.08</td><td>1.19</td><td>null</td><td>null</td><td></td><td>8.83E+00</td><td>4.01E-01</td><td></td><td>-1.18E-01</td><td>8.76E-01</td><td></td><td>-4.04E-01</td><td>6.73E-01</td><td></td><td>3.67E+00</td><td>1.56E-18</td><td>Up</td><td>-8.58E-02</td><td>9.52E-01</td><td></td><td></td><td></td><td></td><td></td><td></td><td></td><td></td></tr>
<tr><td>Unigene116524_All</td><td>235</td><td>0.00</td><td>1.08</td><td>0.00</td><td>0.00</td><td>0.73</td><td>0.38</td><td>0.18</td><td>1.78</td><td>2.88</td><td>1.01E+01</td><td>7.88E-02</td><td></td><td>null</td><td>null</td><td></td><td>9.52E+00</td><td>2.27E-01</td><td></td><td>8.59E+00</td><td>4.79E-01</td><td></td><td>3.31E+00</td><td>2.00E-02</td><td></td><td>4.00E+00</td><td>4.30E-04</td><td>Up</td><td></td><td></td><td>Unigene116524_All//3.00E-07//TC148620</td><td></td><td></td><td></td><td></td></tr>
<tr><td>Unigene116526_All</td><td>235</td><td>159.41</td><td>136.92</td><td>164.51</td><td>165.34</td><td>189.91</td><td>173.16</td><td>195.03</td><td>104.76</td><td>51.20</td><td>-2.20E-01</td><td>1.53E-02</td><td></td><td>4.54E-02</td><td>6.45E-01</td><td></td><td>2.00E-01</td><td>1.25E-02</td><td></td><td>6.66E-02</td><td>5.34E-01</td><td></td><td>-8.97E-01</td><td>3.16E-34</td><td></td><td>-1.93E+00</td><td>1.44E-112</td><td>Down</td><td>Unigene116526_All//gi|18403820|ref|NP_566732.1|//4.00E-24//dienelactone hydrolase family protein [Arabidopsis thaliana] >gi|17381244|gb|AAL36041.1| AT3g23600/MDB19_9 [Arabidopsis thaliana] >gi|20453365|gb|AAM19921.1| AT3g23600/MDB19_9 [Arabidopsis thaliana] >gi|21536848|gb|AAM61180.1| contains similarity to endo-1,3-1,4-beta-D-glucanase [Arabidopsis thaliana]</td><td>Unigene116526_All//1.00E-25//AT3G23600| dienelactone hydrolase family protein</td><td>Unigene116526_All//8.00E-128//TC162002</td><td></td><td></td><td>GO:0004091//GO:0005576//GO:0006970//GO:0016020//GO:0043231</td><td>nr -</td></tr>
<tr><td>Unigene11654_All</td><td>3138</td><td>33.75</td><td>15.84</td><td>27.00</td><td>15.48</td><td>17.28</td><td>16.12</td><td>10.25</td><td>9.36</td><td>8.43</td><td>-1.09E+00</td><td>3.72E-88</td><td>Down</td><td>-3.22E-01</td><td>3.24E-10</td><td></td><td>1.59E-01</td><td>3.18E-02</td><td></td><td>5.86E-02</td><td>5.45E-01</td><td></td><td>-1.31E-01</td><td>1.70E-01</td><td></td><td>-2.83E-01</td><td>7.33E-04</td><td></td><td>Unigene11654_All//gi|297796141|ref|XP_002865955.1|//0.00E+00//FTSH11 [Arabidopsis lyrata subsp. lyrata] >gi|297311790|gb|EFH42214.1| FTSH11 [Arabidopsis lyrata subsp. lyrata]</td><td>Unigene11654_All//0.00E+00//AT5G53170| FTSH11 (FtsH protease 11); ATP-dependent peptidase/ ATPase/ metallopeptidase</td><td>Unigene11654_All//0.00E+00//TC146914</td><td></td><td></td><td>GO:0004175//GO:0006950//GO:0009536//GO:0009642//GO:0032559//GO:0042623//GO:0044257</td><td>nr +</td></tr>
<tr><td>Unigene116545_All</td><td>237</td><td>0.00</td><td>0.00</td><td>0.00</td><td>0.00</td><td>0.00</td><td>0.00</td><td>0.36</td><td>4.06</td><td>0.00</td><td>null</td><td>null</td><td></td><td>null</td><td>null</td><td></td><td>null</td><td>null</td><td></td><td>null</td><td>null</td><td></td><td>3.51E+00</td><td>5.50E-05</td><td>Up</td><td>-8.48E+00</td><td>3.59E-01</td><td></td><td></td><td></td><td></td><td></td><td></td><td></td><td></td></tr>
<tr><td>Unigene116547_All</td><td>237</td><td>0.00</td><td>0.00</td><td>0.00</td><td>0.00</td><td>0.00</td><td>0.00</td><td>0.18</td><td>3.53</td><td>0.50</td><td>null</td><td>null</td><td></td><td>null</td><td>null</td><td></td><td>null</td><td>null</td><td></td><td>null</td><td>null</td><td></td><td>4.31E+00</td><td>5.68E-05</td><td>Up</td><td>1.50E+00</td><td>5.58E-01</td><td></td><td></td><td></td><td></td><td></td><td></td><td></td><td>ESTscan -</td></tr>
<tr><td>Unigene116549_All</td><td>237</td><td>0.00</td><td>0.00</td><td>0.00</td><td>0.00</td><td>0.00</td><td>0.00</td><td>0.00</td><td>2.29</td><td>0.00</td><td>null</td><td>null</td><td></td><td>null</td><td>null</td><td></td><td>null</td><td>null</td><td></td><td>null</td><td>null</td><td></td><td>1.12E+01</td><td>5.37E-04</td><td>Up</td><td>null</td><td>null</td><td></td><td>Unigene116549_All//gi|145334857|ref|NP_001078774.1|//3.00E-09//AALP (Arabidopsis aleurain-like protease); cysteine-type peptidase [Arabidopsis thaliana]</td><td>Unigene116549_All//1.00E-10//AT5G60360| AALP (Arabidopsis aleurain-like protease); cysteine-type peptidase</td><td></td><td></td><td></td><td>GO:0005488//GO:0009725//GO:0032502//GO:0043231//GO:0070011</td><td>nr +</td></tr>
<tr><td>Unigene11655_All</td><td>1713</td><td>4.95</td><td>4.44</td><td>3.23</td><td>9.22</td><td>12.66</td><td>10.31</td><td>12.12</td><td>2.37</td><td>4.51</td><td>-1.57E-01</td><td>4.65E-01</td><td></td><td>-6.14E-01</td><td>2.97E-03</td><td></td><td>4.58E-01</td><td>3.43E-05</td><td></td><td>1.61E-01</td><td>2.94E-01</td><td></td><td>-2.36E+00</td><td>4.22E-64</td><td>Down</td><td>-1.43E+00</td><td>1.18E-33</td><td>Down</td><td>Unigene11655_All//gi|115480615|ref|NP_001063901.1|//2.00E-74//Os09g0556800 [Oryza sativa Japonica Group] >gi|113632134|dbj|BAF25815.1| Os09g0556800 [Oryza sativa Japonica Group]</td><td></td><td>Unigene11655_All//0.00E+00//TC135786</td><td></td><td></td><td></td><td>nr -</td></tr>
<tr><td>Unigene116552_All</td><td>237</td><td>0.00</td><td>0.00</td><td>0.00</td><td>0.00</td><td>0.00</td><td>0.00</td><td>2.85</td><td>1.94</td><td>0.00</td><td>null</td><td>null</td><td></td><td>null</td><td>null</td><td></td><td>null</td><td>null</td><td></td><td>null</td><td>null</td><td></td><td>-5.57E-01</td><td>4.89E-01</td><td></td><td>-1.15E+01</td><td>3.56E-05</td><td>Down</td><td></td><td></td><td></td><td></td><td></td><td></td><td>ESTscan -</td></tr>
<tr><td>Unigene116559_All</td><td>237</td><td>0.00</td><td>0.00</td><td>0.00</td><td>0.00</td><td>0.00</td><td>0.00</td><td>12.31</td><td>5.64</td><td>1.34</td><td>null</td><td>null</td><td></td><td>null</td><td>null</td><td></td><td>null</td><td>null</td><td></td><td>null</td><td>null</td><td></td><td>-1.13E+00</td><td>6.78E-04</td><td>Down</td><td>-3.19E+00</td><td>1.79E-13</td><td>Down</td><td>Unigene116559_All//gi|219118863|ref|XP_002180198.1|//2.00E-36//ubiquitin extension protein 4 [Phaeodactylum tricornutum CCAP 1055/1] >gi|217408455|gb|EEC48389.1| ubiquitin extension protein 4 [Phaeodactylum tricornutum CCAP 1055/1]</td><td>Unigene116559_All//3.00E-37//AT5G03240| UBQ3 (POLYUBIQUITIN 3); protein binding</td><td>Unigene116559_All//2.00E-08//DW238310</td><td></td><td></td><td>GO:0044424</td><td>nr +</td></tr>
<tr><td>Unigene11656_All</td><td>323</td><td>1.94</td><td>12.08</td><td>3.13</td><td>2.62</td><td>2.45</td><td>5.87</td><td>3.27</td><td>6.34</td><td>3.33</td><td>2.64E+00</td><td>1.32E-11</td><td>Up</td><td>6.88E-01</td><td>3.42E-01</td><td></td><td>-9.66E-02</td><td>8.77E-01</td><td></td><td>1.16E+00</td><td>1.59E-02</td><td></td><td>9.54E-01</td><td>1.83E-02</td><td></td><td>2.52E-02</td><td>9.76E-01</td><td></td><td>Unigene11656_All//gi|195606966|gb|ACG25313.1|//6.00E-25//zinc finger, C3HC4 type family protein [Zea mays]</td><td>Unigene11656_All//5.00E-19//AT5G45290| zinc finger (C3HC4-type RING finger) family protein</td><td>Unigene11656_All//6.00E-120//DR455518</td><td></td><td></td><td>GO:0043231//GO:0046914</td><td>nr -</td></tr>
<tr><td>Unigene11657_All</td><td>3891</td><td>11.19</td><td>53.78</td><td>26.47</td><td>12.84</td><td>22.25</td><td>20.72</td><td>11.43</td><td>17.00</td><td>13.25</td><td>2.27E+00</td><td>0.00E+00</td><td>Up</td><td>1.24E+00</td><td>2.91E-102</td><td>Up</td><td>7.93E-01</td><td>2.58E-50</td><td></td><td>6.90E-01</td><td>1.94E-33</td><td></td><td>5.73E-01</td><td>7.83E-23</td><td></td><td>2.13E-01</td><td>1.18E-03</td><td></td><td>Unigene11657_All//gi|115480615|ref|NP_001063901.1|//4.00E-89//Os09g0556800 [Oryza sativa Japonica Group] >gi|113632134|dbj|BAF25815.1| Os09g0556800 [Oryza sativa Japonica Group]</td><td>Unigene11657_All//2.00E-29//AT5G45290| zinc finger (C3HC4-type RING finger) family protein</td><td>Unigene11657_All//0.00E+00//TC158702</td><td></td><td></td><td>GO:0046914</td><td>nr +</td></tr>
<tr><td>Unigene116580_All</td><td>238</td><td>14.95</td><td>18.73</td><td>14.10</td><td>32.65</td><td>17.80</td><td>25.81</td><td>30.73</td><td>23.88</td><td>10.38</td><td>3.26E-01</td><td>2.74E-01</td><td></td><td>-8.48E-02</td><td>8.01E-01</td><td></td><td>-8.76E-01</td><td>5.58E-06</td><td></td><td>-3.39E-01</td><td>1.26E-01</td><td></td><td>-3.64E-01</td><td>6.87E-02</td><td></td><td>-1.57E+00</td><td>3.77E-14</td><td>Down</td><td></td><td></td><td>Unigene116580_All//3.00E-50//TC168990</td><td></td><td></td><td></td><td></td></tr>
<tr><td>Unigene116588_All</td><td>239</td><td>0.00</td><td>0.00</td><td>0.00</td><td>0.00</td><td>0.00</td><td>0.00</td><td>0.00</td><td>2.27</td><td>0.00</td><td>null</td><td>null</td><td></td><td>null</td><td>null</td><td></td><td>null</td><td>null</td><td></td><td>null</td><td>null</td><td></td><td>1.12E+01</td><td>5.37E-04</td><td>Up</td><td>null</td><td>null</td><td></td><td>Unigene116588_All//gi|299473653|emb|CBN78047.1|//1.00E-13//flagellar associated protein, quinone reductase-like protein [Ectocarpus siliculosus]</td><td>Unigene116588_All//1.00E-07//AT4G27270| quinone reductase family protein</td><td></td><td></td><td></td><td></td><td>nr +</td></tr>
<tr><td>Unigene116592_All</td><td>239</td><td>0.00</td><td>0.00</td><td>0.00</td><td>0.00</td><td>0.00</td><td>0.00</td><td>0.00</td><td>5.25</td><td>0.00</td><td>null</td><td>null</td><td></td><td>null</td><td>null</td><td></td><td>null</td><td>null</td><td></td><td>null</td><td>null</td><td></td><td>1.24E+01</td><td>7.17E-09</td><td>Up</td><td>null</td><td>null</td><td></td><td>Unigene116592_All//sp|Q9RAJ1|DHAR_MYCSX//2.00E-10//HTH-type transcriptional repressor dhaR OS=Mycobacterium sp. (strain GP1) GN=dhaR PE=4 SV=1</td><td></td><td></td><td></td><td></td><td></td><td>swissprot -</td></tr>
<tr><td>Unigene116593_All</td><td>239</td><td>0.22</td><td>0.00</td><td>0.00</td><td>0.00</td><td>0.29</td><td>0.38</td><td>2.30</td><td>16.79</td><td>1.83</td><td>-7.77E+00</td><td>6.16E-01</td><td></td><td>-7.77E+00</td><td>6.28E-01</td><td></td><td>8.17E+00</td><td>6.13E-01</td><td></td><td>8.56E+00</td><td>4.79E-01</td><td></td><td>2.87E+00</td><td>7.07E-16</td><td>Up</td><td>-3.27E-01</td><td>6.72E-01</td><td></td><td></td><td></td><td>Unigene116593_All//3.00E-07//CD809346</td><td></td><td></td><td></td><td></td></tr>
</table>

Unigene11660_All	564	6.12	2.07	1.61	10.07	6.53	6.17	13.34	12.45	10.81	-1.57E+00	9.76E-06	Down	-1.93E+00	5.28E-07	Down	-6.24E-01	8.10E-03		-7.08E-01	4.04E-03		-1.00E-01	6.31E-01		-3.04E-01	1.11E-01		Unigene11660_All//gi|47497095|dbj|BAD19146.1|//2.00E-58//ankyrin repeat protein-like [Oryza sativa Japonica Group]	Unigene11660_All//5.00E-69//AT3G04470| FUNCTIONS IN: molecular_function unknown; INVOLVED IN: biological_process unknown; LOCATED IN: plasma membrane; EXPRESSED IN: 25 plant structures; EXPRESSED DURING: 15 growth stages; CONTAINS InterPro DOMAIN/s: Ankyrin (InterPro:IPR002110); BEST Arabidopsis thaliana protein match is: ankyrin repeat family protein (TAIR:AT1G04780.1); Has 842 Blast hits to 642 proteins in 95 species: Archae - 0; Bacteria - 6; Metazoa - 523; Fungi - 26; Plants - 165; Viruses - 4; Other Eukaryotes - 118 (source: NCBI BLink).	Unigene11660_All//0.00E+00//CO113709			GO:0016020	nr -
Unigene11663_All	637	1.31	1.51	2.17	1.96	3.51	1.56	2.12	6.30	4.50	2.02E-01	7.54E-01		7.26E-01	2.28E-01		8.46E-01	4.73E-02		-3.26E-01	6.41E-01		1.57E+00	7.47E-08	Up	1.08E+00	8.63E-04	Up	Unigene11663_All//gi|163644184|dbj|BAF95742.1|//7.00E-41//ethylene receptor homolog [Physcomitrella patens]	Unigene11663_All//2.00E-30//AT1G66340| ETR1 (ETHYLENE RESPONSE 1); ethylene binding / protein histidine kinase/ two-component response regulator	Unigene11663_All//2.00E-103//gi|109853628|gb|DW482607.1|DW482607	Orphans		GO:0004871//GO:0005488//GO:0016020//GO:0016301//GO:0023060//GO:0044237	nr -
Unigene116630_All	242	0.00	0.00	0.00	0.00	0.00	0.00	198.65	28.84	1.98	null	null		null	null		null	null		null	null		-2.78E+00	2.04E-178	Down	-6.65E+00	0.00E+00	Down							
Unigene116642_All	243	0.00	0.00	0.00	0.00	0.00	0.00	2.78	2.58	0.00	null	null		null	null		null	null		null	null		-1.10E-01	8.90E-01		-1.14E+01	3.56E-05	Down			Unigene116642_All//7.00E-11//ES845934				
Unigene11665_All	4684	77.27	186.32	118.29	20.75	14.32	13.52	9.07	10.23	4.55	1.27E+00	0.00E+00	Up	6.14E-01	6.11E-167		-5.35E-01	4.78E-29		-6.18E-01	2.08E-33		1.73E-01	1.68E-02		-9.95E-01	8.41E-39		Unigene11665_All//gi|75755655|ref|YP_319761.1|//0.00E+00//photosystem II 44 kDa protein [Acorus calamus] >gi|74381705|emb|CAI53790.1| PSII 43 KDa protein [Acorus calamus]	Unigene11665_All//0.00E+00//ATCG00280| chloroplast gene encoding a CP43 subunit of the photosystem II reaction center. promoter contains a blue-light responsive element.	Unigene11665_All//0.00E+00//TC129711		ko00195|Photosynthesis	GO:0006464//GO:0009055//GO:0009521//GO:0009534//GO:0009767//GO:0031224//GO:0042716//GO:0043169//GO:0046906//GO:0051234	nr -
Unigene116653_All	244	0.00	0.00	0.00	0.00	0.00	0.00	0.00	2.23	0.00	null	null		null	null		null	null		null	null		1.11E+01	5.36E-04	Up	null	null		Unigene116653_All//gi|298708397|emb|CBJ48460.1|//2.00E-32//Molecular chaperones GRP78/BiP/KAR2, HSP70 superfamily [Ectocarpus siliculosus]	Unigene116653_All//2.00E-24//AT5G28540| BIP1; ATP binding			ko03040|Spliceosome//ko04141|Protein processing in endoplasmic reticulum//ko04144|Endocytosis//ko03060|Protein export	GO:0032559//GO:0050896	nr -
Unigene116660_All	244	0.00	0.00	0.00	0.00	0.00	0.00	0.00	4.45	1.31	null	null		null	null		null	null		null	null		1.21E+01	1.04E-07	Up	1.04E+01	1.34E-02								ESTscan -
Unigene116664_All	244	10.72	2.49	5.67	21.84	11.85	13.88	3.81	5.31	7.67	-2.11E+00	2.26E-06	Down	-9.18E-01	2.50E-02		-8.82E-01	2.40E-04		-6.54E-01	1.09E-02		4.78E-01	3.95E-01		1.01E+00	1.46E-02		Unigene116664_All//gi|119888002|gb|ABM05953.1|//2.00E-07//proline-rich protein [Gossypium hirsutum]		Unigene116664_All//2.00E-88//gi|109838020|gb|DW236581.1|DW236581				nr +
Unigene11667_All	2100	3.64	1.95	5.71	2.13	1.18	0.84	0.58	0.52	0.17	-8.96E-01	2.44E-05		6.49E-01	1.25E-04		-8.55E-01	1.33E-03		-1.35E+00	3.28E-06	Down	-1.74E-01	7.49E-01		-1.77E+00	1.72E-03		Unigene11667_All//gi|168012500|ref|XP_001758940.1|//6.00E-69//plastid sigma facor SIG2 [Physcomitrella patens subsp. patens] >gi|12697780|dbj|BAB21618.1| PpSIG2 [Physcomitrella patens] >gi|14669832|dbj|BAB62008.1| PpSIG2 [Physcomitrella patens] >gi|162690077|gb|EDQ76446.1| plastid sigma facor SIG2 [Physcomitrella patens subsp. patens]	Unigene11667_All//7.00E-59//AT2G36990| SIGF (RNA POLYMERASE SIGMA-SUBUNIT F); DNA binding / DNA-directed RNA polymerase/ sigma factor/ transcription factor	Unigene11667_All//0.00E+00//DR452614	Sigma70-like		GO:0000996//GO:0001071//GO:0006351//GO:0009536//GO:0009639//GO:0009791//GO:0010557//GO:0034062	nr -
Unigene116672_All	245	0.00	0.62	1.30	0.81	0.00	0.74	176.02	156.78	1371.75	9.28E+00	2.36E-01		1.03E+01	4.53E-02		-9.67E+00	8.46E-02		-1.41E-01	9.35E-01		-1.67E-01	3.06E-02		2.96E+00	0.00E+00	Up	Unigene116672_All//gi|3970689|emb|CAA77191.1|//9.00E-39//(+)-delta-cadinene synthase [Gossypium arboreum]	Unigene116672_All//5.00E-16//AT5G23960| TPS21 (TERPENE SYNTHASE 21); (-)-E-beta-caryophyllene synthase/ alpha-humulene synthase	Unigene116672_All//4.00E-136//TC145282		ko00904|Diterpenoid biosynthesis	GO:0010334//GO:0046872	nr -
Unigene116677_All	245	0.00	0.00	0.00	0.00	0.00	0.00	1.55	15.18	4.88	null	null		null	null		null	null		null	null		3.29E+00	9.25E-17	Up	1.65E+00	3.87E-03				Unigene116677_All//3.00E-07//TC144453				
Unigene116687_All	246	0.00	0.00	0.00	0.00	0.00	0.00	0.69	10.36	0.00	null	null		null	null		null	null		null	null		3.91E+00	2.40E-13	Up	-9.43E+00	1.08E-01		Unigene116687_All//gi|1545805|dbj|BAA10929.1|//3.00E-11//cytochrome P450 like_TBP [Nicotiana tabacum]		Unigene116687_All//8.00E-20//gi|73861424|gb|DT464163.1|DT464163				nr -
Unigene116690_All	246	0.00	0.00	0.00	0.00	0.00	0.00	0.00	6.80	0.00	null	null		null	null		null	null		null	null		1.27E+01	8.76E-12	Up	null	null		Unigene116690_All//gi|168068200|ref|XP_001785975.1|//5.00E-11//predicted protein [Physcomitrella patens subsp. patens] >gi|162662338|gb|EDQ49215.1| predicted protein [Physcomitrella patens subsp. patens]		Unigene116690_All//2.00E-14//TC155558				nr -
Unigene116701_All	247	0.00	0.00	0.00	0.81	0.00	0.00	0.34	2.37	3.71	null	null		null	null		-9.66E+00	8.45E-02		-9.66E+00	1.34E-01		2.79E+00	8.26E-03		3.44E+00	7.53E-05	Up	Unigene116701_All//gi|255568217|ref|XP_002525084.1|//5.00E-06//ATP-binding cassette transporter, putative [Ricinus communis] >gi|223535665|gb|EEF37331.1| ATP-binding cassette transporter, putative [Ricinus communis]	Unigene116701_All//9.00E-05//AT3G55090| ATPase, coupled to transmembrane movement of substances	Unigene116701_All//5.00E-18//gi|78349095|gb|DT569369.1|DT569369			GO:0016787	nr +
Unigene116704_All	247	35.37	11.69	20.05	51.43	45.59	47.72	160.05	67.35	47.10	-1.60E+00	1.09E-13	Down	-8.19E-01	5.58E-05		-1.74E-01	3.19E-01		-1.08E-01	5.90E-01		-1.25E+00	2.67E-50	Down	-1.76E+00	4.36E-86	Down	Unigene116704_All//gi|195658145|gb|ACG48540.1|//6.00E-09//60S ribosomal protein L23 [Zea mays]	Unigene116704_All//2.00E-10//AT3G04400| emb2171 (embryo defective 2171); structural constituent of ribosome	Unigene116704_All//5.00E-123//gi|13246858|gb|BE055726.2|BE055726		ko03010|Ribosome		nr +
Unigene116705_All	247	0.00	0.00	0.00	0.00	0.00	0.00	9.59	38.07	0.16	null	null		null	null		null	null		null	null		1.99E+00	7.31E-24	Up	-5.89E+00	5.63E-16	Down			Unigene116705_All//1.00E-09//TC146753				
Unigene116708_All	247	40.46	18.67	15.09	131.29	92.15	173.34	115.54	55.33	88.23	-1.12E+00	2.65E-09	Down	-1.42E+00	1.25E-12	Down	-5.11E-01	3.20E-09		4.01E-01	3.93E-07		-1.06E+00	2.21E-28	Down	-3.89E-01	1.05E-05		Unigene116708_All//gi|183393002|gb|ACC61674.1|//2.00E-30//mitochondrial cytochrome c [Vitis vinifera]	Unigene116708_All//4.00E-31//AT4G10040| CYTC-2 (cytochrome c-2); electron carrier/ heme binding / iron ion binding	Unigene116708_All//1.00E-108//gi|18099811|gb|BM359065.1|BM359065			GO:0005506//GO:0005743//GO:0006091//GO:0044425//GO:0051234	nr +
Unigene116714_All	247	136.84	90.47	137.54	124.43	120.59	162.00	284.83	84.10	158.06	-5.97E-01	1.14E-10		7.40E-03	9.64E-01		-4.52E-02	7.28E-01		3.81E-01	3.69E-06		-1.76E+00	1.09E-147	Down	-8.50E-01	5.75E-49				Unigene116714_All//2.00E-70//gi|109836664|gb|DW235225.1|DW235225				
Unigene11672_All	3206	4.70	1.53	3.95	15.54	9.60	8.95	8.39	2.65	3.27	-1.62E+00	1.82E-23	Down	-2.50E-01	1.17E-01		-6.94E-01	6.94E-24		-7.97E-01	7.31E-27		-1.66E+00	1.31E-52	Down	-1.36E+00	2.17E-40	Down	Unigene11672_All//gi|300143215|gb|EFJ09908.1|//0.00E+00//trehalose phosphate synthase [Selaginella moellendorffii]	Unigene11672_All//0.00E+00//AT4G17770| ATTPS5; protein binding / transferase, transferring glycosyl groups / trehalose-phosphatase	Unigene11672_All//0.00E+00//TC150954		ko00500|Starch and sucrose metabolism	GO:0005488//GO:0005991//GO:0019203//GO:0035251	nr -
Unigene116722_All	248	0.00	0.00	0.00	0.00	0.00	0.00	1.53	5.56	0.00	null	null		null	null		null	null		null	null		1.86E+00	7.81E-04	Up	-1.06E+01	4.20E-03		Unigene116722_All//gi|299470138|emb|CBN78167.1|//2.00E-18//ribosomal protein L34 [Ectocarpus siliculosus]	Unigene116722_All//3.00E-15//AT1G26880| 60S ribosomal protein L34 (RPL34A)			ko03010|Ribosome	GO:0043231	nr +
Unigene11674_All	944	1.72	2.04	2.31	2.16	1.61	1.58	1.75	2.97	3.88	2.48E-01	6.22E-01		4.29E-01	3.62E-01		-4.31E-01	3.48E-01		-4.55E-01	3.46E-01		7.64E-01	2.26E-02		1.15E+00	5.48E-05	Up	Unigene11674_All//gi|10177638|dbj|BAB10911.1|//1.00E-90//receptor protein kinase [Arabidopsis thaliana] >gi|224589711|gb|ACN59387.1| leucine-rich repeat receptor-like protein kinase [Arabidopsis thaliana]	Unigene11674_All//3.00E-92//AT5G49660| leucine-rich repeat transmembrane protein kinase, putative	Unigene11674_All//0.00E+00//DW508005			GO:0004674//GO:0004871//GO:0006464//GO:0016491//GO:0031224//GO:0031410//GO:0032559	nr +
Unigene116745_All	251	0.00	0.00	0.00	0.00	0.00	0.00	2.53	2.33	0.00	null	null		null	null		null	null		null	null		-1.16E-01	8.88E-01		-1.13E+01	7.11E-05	Down	Unigene116745_All//gi|300261783|gb|EFJ45994.1|//1.00E-19//component of cytosolic 80S ribosome and 40S small subunit [Volvox carteri f. nagariensis]	Unigene116745_All//7.00E-20//AT4G39200| 40S ribosomal protein S25 (RPS25E)			ko03010|Ribosome	GO:0010467//GO:0015935//GO:0016020	nr -
Unigene116747_All	252	0.00	0.00	0.00	0.00	0.00	0.00	2.35	1.82	0.00	null	null		null	null		null	null		null	null		-3.65E-01	6.49E-01		-1.12E+01	1.41E-04	Down							
Unigene11675_All	3370	1.75	1.56	2.35	20.19	11.98	9.82	56.68	26.55	11.30	-1.66E-01	5.31E-01		4.24E-01	5.22E-02		-7.54E-01	1.66E-37		-1.04E+00	6.43E-57	Down	-1.09E+00	1.11E-194	Down	-2.33E+00	0.00E+00	Down	Unigene11675_All//gi|10177638|dbj|BAB10911.1|//0.00E+00//receptor protein kinase [Arabidopsis thaliana] >gi|224589711|gb|ACN59387.1| leucine-rich repeat receptor-like protein kinase [Arabidopsis thaliana]	Unigene11675_All//0.00E+00//AT5G49660| leucine-rich repeat transmembrane protein kinase, putative	Unigene11675_All//0.00E+00//TC142851			GO:0004702//GO:0006464//GO:0016628//GO:0031224//GO:0031410//GO:0032559	nr -
Unigene116765_All	253	0.00	0.00	0.00	0.00	0.00	0.00	2.17	1.49	0.00	null	null		null	null		null	null		null	null		-5.47E-01	5.52E-01		-1.11E+01	2.81E-04	Down							
Unigene11678_All	844	0.12	0.00	0.13	1.89	0.69	1.34	1.70	2.87	4.48	-6.95E+00	3.75E-01		2.54E-02	1.00E+00		-1.45E+00	4.07E-03		-4.98E-01	3.77E-01		7.54E-01	3.93E-02		1.40E+00	1.36E-06	Up	Unigene11678_All//gi|33350934|gb|AAK39643.3|//1.00E-115//ATP-binding cassette transporter AtABCA1 [Arabidopsis thaliana]	Unigene11678_All//3.00E-109//AT2G41700| ATPase, coupled to transmembrane movement of substances / amino acid transmembrane transporter				GO:0017111//GO:0032559	nr -
Unigene116792_All	255	0.00	0.00	0.00	0.00	0.00	0.00	0.66	3.93	4.69	null	null		null	null		null	null		null	null		2.57E+00	4.79E-04	Up	2.82E+00	3.02E-05	Up							
Unigene116794_All	255	0.00	0.00	0.00	0.00	0.00	0.00	0.00	2.13	0.00	null	null		null	null		null	null		null	null		1.11E+01	5.36E-04	Up	null	null		Unigene116794_All//gi|159477663|ref|XP_001696928.1|//1.00E-24//aldehyde dehydrogenase [Chlamydomonas reinhardtii] >gi|158274840|gb|EDP00620.1| aldehyde dehydrogenase [Chlamydomonas reinhardtii]	Unigene116794_All//2.00E-16//AT5G62530| ALDH12A1; 1-pyrroline-5-carboxylate dehydrogenase/ 3-chloroallyl aldehyde dehydrogenase			ko00250|Alanine, aspartate and glutamate metabolism//ko00330|Arginine and proline metabolism	GO:0004030//GO:0008152//GO:0043231	nr -
Unigene116804_All	256	0.00	0.00	0.00	0.00	0.00	0.00	0.00	2.45	0.31	null	null		null	null		null	null		null	null		1.13E+01	1.47E-04	Up	8.28E+00	4.05E-01		Unigene116804_All//gi|193878342|gb|ACF25911.1|//1.00E-20//MLO-like protein 6 [Vitis vinifera]	Unigene116804_All//5.00E-13//AT2G39200| MLO12 (MILDEW RESISTANCE LOCUS O 12); calmodulin binding				GO:0009987//GO:0031224	nr +
Unigene116807_All	257	0.00	0.00	0.00	0.19	0.27	0.18	3.62	1.95	0.47	null	null		null	null		4.68E-01	9.60E-01		-1.42E-01	9.55E-01		-8.91E-01	1.72E-01		-2.96E+00	1.72E-04	Down							
Unigene11681_All	2500	2.66	0.45	1.79	3.91	3.07	3.92	4.75	2.62	2.71	-2.58E+00	4.97E-19	Down	-5.71E-01	1.67E-02		-3.47E-01	5.84E-02		5.50E-03	9.78E-01		-8.56E-01	7.83E-09		-8.11E-01	2.14E-08		Unigene11681_All//gi|124360024|gb|ABN08040.1|//0.00E+00//Acyl-coA-binding protein, ACBP; Serine/threonine protein phosphatase, BSU1 [Medicago truncatula]	Unigene11681_All//0.00E+00//AT3G05420| ACBP4 (ACYL-COA BINDING PROTEIN 4); acyl-CoA binding	Unigene11681_All//0.00E+00//gi|164247899|gb|ES796003.1|ES796003		ko04712|Circadian rhythm - plant	GO:0005504//GO:0006810//GO:0009314//GO:0009725//GO:0043231//GO:0044444	nr +
Unigene116817_All	257	0.00	0.00	0.00	1.94	0.00	0.35	0.82	1.46	0.00	null	null		null	null		-1.09E+01	8.99E-04	Down	-2.46E+00	4.83E-02		8.31E-01	4.67E-01		-9.68E+00	5.80E-02		Unigene116817_All//gi|297596372|ref|NP_001042475.2|//3.00E-15//Os01g0228200 [Oryza sativa Japonica Group] >gi|255673021|dbj|BAF04389.2| Os01g0228200 [Oryza sativa Japonica Group]	Unigene116817_All//7.00E-12//AT1G17240| AtRLP2 (Receptor Like Protein 2); protein binding / protein kinase	Unigene116817_All//2.00E-08//ES834884		ko04626|Plant-pathogen interaction	GO:0004672//GO:0006464//GO:0016628//GO:0031224//GO:0032559	nr -
Unigene11682_All	2293	5.50	6.23	3.86	12.93	12.70	11.19	8.28	4.32	3.96	1.80E-01	2.62E-01		-5.12E-01	1.96E-03		-2.49E-02	8.29E-01		-2.08E-01	4.75E-02		-9.39E-01	9.31E-16		-1.06E+00	8.83E-20	Down	Unigene11682_All//gi|124360024|gb|ABN08040.1|//0.00E+00//Acyl-coA-binding protein, ACBP; Serine/threonine protein phosphatase, BSU1 [Medicago truncatula]	Unigene11682_All//0.00E+00//AT3G05420| ACBP4 (ACYL-COA BINDING PROTEIN 4); acyl-CoA binding	Unigene11682_All//0.00E+00//ES825853		ko04712|Circadian rhythm - plant	GO:0005504//GO:0006810//GO:0009314//GO:0009725//GO:0043231//GO:0044444	nr -
Unigene116824_All	258	0.00	0.00	0.00	0.00	0.00	0.00	0.66	3.56	5.25	null	null		null	null		null	null		null	null		2.44E+00	1.33E-03		3.00E+00	3.58E-06	Up							
Unigene116832_All	258	306.22	470.36	779.14	610.51	533.21	995.52	55.72	127.17	132.02	6.19E-01	1.38E-38		1.35E+00	3.04E-226	Up	-1.95E-01	2.24E-07		7.05E-01	1.26E-109		1.19E+00	7.74E-39	Up	1.24E+00	3.97E-44	Up	Unigene116832_All//gi|23506611|gb|AAN37904.1|//1.00E-19//histone H1D [Nicotiana tabacum]	Unigene116832_All//4.00E-12//AT2G18050| HIS1-3 (HISTONE H1-3); DNA binding / nucleosomal DNA binding	Unigene116832_All//5.00E-126//EV498964			GO:0000785//GO:0003676//GO:0034728//GO:0043231	nr -
Unigene116843_All	259	0.00	0.00	0.00	1.15	0.00	0.00	0.65	4.68	1.08	null	null		null	null		-1.02E+01	2.02E-02		-1.02E+01	4.00E-02		2.84E+00	3.39E-05	Up	7.22E-01	5.97E-01								ESTscan +

Unigene116849_All	260	0.00	0.97	0.00	0.00	0.00	0.00	57.08	31.35	302.93	9.93E+00	7.88E-02		null	null		null	null		null	null		-8.65E-01	5.42E-11		2.41E+00	8.71E-252	Up	Unigene116849_All//gi|11132838|sp|064967.1|HMDH2_GOSHI//9.00E-42//RecName: Full=3-hydroxy-3-methylglutaryl-coenzyme A reductase 2; Short=HMG-CoA reductase 2 >gi|2935300|gb|AAC05089.1| 3-hydroxy-3-methylglutaryl-coenzyme A reductase 2 [Gossypium hirsutum]	Unigene116849_All//1.00E-37//AT1G76490| HMG1 (HYDROXY METHYLGLUTARYL COA REDUCTASE 1); hydroxymethylglutaryl-CoA reductase	Unigene116849_All//1.00E-135//TC162385		ko00900|Terpenoid backbone biosynthesis	GO:0000166//GO:0005740//GO:0006720//GO:0006732//GO:0009526//GO:0016616//GO:0031090//GO:0031224//GO:0048037	nr +
Unigene116857_All	260	0.60	0.00	0.82	2.30	1.46	1.74	0.33	2.89	0.15	-9.24E+00	2.16E-01		4.40E-01	7.76E-01		-6.58E-01	4.78E-01		-4.04E-01	6.73E-01		3.15E+00	9.54E-04	Up	-1.09E+00	6.77E-01								
Unigene116858_All	260	0.20	1.36	0.00	0.00	0.00	0.00	1.14	5.30	1.38	2.76E+00	9.60E-02		-7.65E+00	6.28E-01		null	null		null	null		2.22E+00	1.31E-04	Up	2.77E-01	7.87E-01								
Unigene11686_All	968	1.84	3.87	4.29	4.89	5.48	4.01	3.32	7.82	4.16	1.08E+00	8.32E-04	Up	1.22E+00	1.23E-04	Up	1.64E-01	5.91E-01		-2.85E-01	3.57E-01		1.24E+00	4.29E-10	Up	3.25E-01	2.41E-01		Unigene11686_All//gi|242390089|dbj|BAH80551.1|//8.00E-37//transcription factor squamosa promoter binding protein-like [Eucalyptus grandis]	Unigene11686_All//2.00E-35//AT5G43270| SPL2 (SQUAMOSA PROMOTER BINDING PROTEIN-LIKE 2); DNA binding / transcription factor	Unigene11686_All//0.00E+00//TC150184	SBP		GO:0050789	nr -
Unigene116868_All	261	0.00	0.00	0.00	0.00	0.00	0.00	1.46	5.93	0.00	null	null		null	null		null	null		null	null		2.02E+00	1.30E-04	Up	-1.05E+01	4.20E-03								
Unigene11687_All	1671	2.41	4.88	2.26	2.86	4.31	2.86	2.99	4.10	2.86	1.02E+00	9.45E-07	Up	-9.17E-02	7.74E-01		5.90E-01	5.07E-03		1.50E-03	9.98E-01		4.58E-01	2.36E-02		-6.16E-02	8.06E-01		Unigene11687_All//gi|242390089|dbj|BAH80551.1|//4.00E-53//transcription factor squamosa promoter binding protein-like [Eucalyptus grandis]	Unigene11687_All//3.00E-38//AT5G43270| SPL2 (SQUAMOSA PROMOTER BINDING PROTEIN-LIKE 2); DNA binding / transcription factor	Unigene11687_All//0.00E+00//TC167346	SBP		GO:0048506	nr -
Unigene116875_All	262	0.00	0.00	0.00	0.00	0.00	0.00	1.45	5.42	0.00	null	null		null	null		null	null		null	null		1.90E+00	5.03E-04	Up	-1.05E+01	4.20E-03		Unigene116875_All//gi|19908703|gb|AAM02974.1|AF421541_1//3.00E-25//Hsp90 [Crypthecodinium cohnii]	Unigene116875_All//3.00E-22//AT5G56010| HSP81-3; ATP binding / unfolded protein binding			ko04626|Plant-pathogen interaction//ko04141|Protein processing in endoplasmic reticulum	GO:0005515//GO:0032559//GO:0044267//GO:0044424//GO:0050896	nr -
Unigene11688_All	1783	0.65	1.68	1.25	1.20	1.47	1.29	1.14	1.97	0.98	1.38E+00	2.60E-04	Up	9.58E-01	3.31E-02		2.89E-01	5.28E-01		1.05E-01	8.18E-01		7.91E-01	7.01E-03		-2.11E-01	6.05E-01		Unigene11688_All//gi|242390089|dbj|BAH80551.1|//2.00E-92//transcription factor squamosa promoter binding protein-like [Eucalyptus grandis]	Unigene11688_All//8.00E-69//AT5G43270| SPL2 (SQUAMOSA PROMOTER BINDING PROTEIN-LIKE 2); DNA binding / transcription factor	Unigene11688_All//0.00E+00//TC167346	SBP		GO:0050789	nr +
Unigene116880_All	262	0.00	0.00	0.00	0.00	0.00	0.00	0.00	2.39	0.61	null	null		null	null		null	null		null	null		1.12E+01	1.47E-04	Up	9.25E+00	1.40E-01								
Unigene116898_All	264	0.00	0.00	0.00	0.00	0.00	0.00	0.64	4.75	0.00	null	null		null	null		null	null		null	null		2.89E+00	1.97E-05	Up	-9.32E+00	1.08E-01				Unigene116898_All//2.00E-17//FL576877				
Unigene116899_All	264	3.17	0.00	1.41	0.94	1.43	1.20	1.12	1.58	0.15	-1.16E+01	5.57E-05	Down	-1.17E+00	1.52E-01		6.05E-01	6.59E-01		3.44E-01	7.97E-01		4.98E-01	6.33E-01		-2.89E+00	6.77E-02								
Unigene116902_All	265	0.00	0.00	0.00	0.00	0.00	0.00	0.00	5.68	0.00	null	null		null	null		null	null		null	null		1.25E+01	1.29E-10	Up	null	null				Unigene116902_All//8.00E-11//TC167383				
Unigene116903_All	265	0.00	0.00	0.00	0.19	0.00	0.00	0.00	2.68	0.30	null	null		null	null		-7.55E+00	5.59E-01		-7.55E+00	6.40E-01		1.14E+01	3.98E-05	Up	8.23E+00	4.05E-01				Unigene116903_All//3.00E-07//gi|73863255|gb|DT465994.1|DT465994				
Unigene116912_All	266	0.00	0.00	0.00	0.00	0.00	0.00	0.00	2.20	0.00	null	null		null	null		null	null		null	null		1.11E+01	2.81E-04	Up	null	null		Unigene116912_All//gi|219120887|ref|XP_002185675.1|//6.00E-06//predicted protein [Phaeodactylum tricornutum CCAP 1055/1] >gi|209582524|gb|ACI65145.1| predicted protein [Phaeodactylum tricornutum CCAP 1055/1]						nr -
Unigene116918_All	266	21.44	6.86	10.41	42.89	46.86	34.47	50.55	9.90	22.02	-1.64E+00	1.49E-09	Down	-1.04E+00	7.12E-05	Down	1.28E-01	5.21E-01		-3.15E-01	7.26E-02		-2.35E+00	1.13E-41	Down	-1.20E+00	4.05E-17	Down	Unigene116918_All//gi|225463470|ref|XP_002276512.1|//2.00E-16//PREDICTED: similar to RPL18AA (60S RIBOSOMAL PROTEIN L18A-1); structural constituent of ribosome isoform 1 [Vitis vinifera]	Unigene116918_All//5.00E-15//AT2G34480| 60S ribosomal protein L18A (RPL18aB)	Unigene116918_All//2.00E-85//gi|164262579|gb|EV487043.1|EV487043		ko03010|Ribosome	GO:0005198//GO:0010467//GO:0030529	nr -
Unigene11692_All	270	5.04	1.13	4.73	3.32	1.79	2.68	5.95	2.79	2.07	-2.16E+00	9.05E-04	Down	-9.01E-02	8.77E-01		-8.95E-01	2.14E-01		-3.11E-01	6.88E-01		-1.09E+00	1.97E-02		-1.53E+00	1.16E-03		Unigene11692_All//gi|240255277|ref|NP_187084.6|//1.00E-42//emb2458 (embryo defective 2458); ATP binding / ATPase/ metalloendopeptidase/ nucleoside-triphosphatase/ nucleotide binding [Arabidopsis thaliana]	Unigene11692_All//4.00E-44//AT3G04340| emb2458 (embryo defective 2458); ATP binding / ATPase/ metalloendopeptidase/ nucleoside-triphosphatase/ nucleotide binding	Unigene11692_All//2.00E-141//gi|48748088|gb|CO078607.1|CO078607			GO:0004175//GO:0019538//GO:0032559//GO:0042623	nr -
Unigene116920_All	267	0.00	0.00	0.00	0.00	0.00	0.00	3.48	5.64	0.15	null	null		null	null		null	null		null	null		6.94E-01	1.55E-01		-4.55E+00	6.41E-06	Down	Unigene116920_All//gi|4139170|gb|AAD03711.1|//8.00E-35//elongation translation factor 1 alpha [Cyanophora paradoxa]	Unigene116920_All//1.00E-31//AT5G60390| elongation factor 1-alpha / EF-1-alpha	Unigene116920_All//1.00E-25//CD809343			GO:0006412//GO:0008135//GO:0017111//GO:0032561//GO:0044424	nr -
Unigene116921_All	267	0.00	0.00	0.00	0.00	0.00	0.00	0.00	2.35	1.04	null	null		null	null		null	null		null	null		1.12E+01	1.47E-04	Up	1.00E+01	2.44E-02								
Unigene116928_All	267	0.00	1.33	1.00	0.93	0.13	0.51	0.16	2.97	1.04	1.04E+01	2.46E-02		9.96E+00	8.03E-02		-2.85E+00	1.37E-01		-8.78E-01	6.16E-01		4.23E+00	1.05E-04	Up	2.72E+00	9.95E-02				Unigene116928_All//8.00E-11//CO114754				
Unigene116930_All	268	0.00	0.00	0.00	0.00	0.00	0.00	0.00	2.18	0.00	null	null		null	null		null	null		null	null		1.11E+01	2.81E-04	Up	null	null				Unigene116930_All//1.00E-21//ES790885				
Unigene116936_All	268	0.00	0.00	0.00	0.00	0.00	0.00	1.89	2.65	0.00	null	null		null	null		null	null		null	null		4.86E-01	5.42E-01		-1.09E+01	5.54E-04	Down	Unigene116936_All//gi|300264012|gb|EFJ48210.1|//2.00E-38//component of cytosolic 80S ribosome and 60S large subunit [Volvox carteri f. nagariensis]	Unigene116936_All//8.00E-40//AT2G18020| EMB2296 (embryo defective 2296); structural constituent of ribosome	Unigene116936_All//9.00E-17//ES821617		ko03010|Ribosome	GO:0003006//GO:0005198//GO:0005488//GO:0009536//GO:0010467//GO:0015934//GO:0016020//GO:0031981	nr +
Unigene116940_All	269	0.00	2.45	0.20	0.74	0.51	0.17	1.10	3.11	1.04	1.13E+01	6.26E-04	Up	7.63E+00	6.38E-01		-5.33E-01	7.49E-01		-2.14E+00	3.47E-01		1.50E+00	3.73E-02		-8.58E-02	9.52E-01								
Unigene116942_All	269	0.00	0.00	0.00	0.00	0.00	0.00	1.41	5.28	0.00	null	null		null	null		null	null		null	null		1.90E+00	5.03E-04	Up	-1.05E+01	4.20E-03								
Unigene116949_All	270	0.00	0.00	0.00	0.74	0.26	0.00	158.94	21.98	32.90	null	null		null	null		-1.53E+00	4.16E-01		-9.53E+00	1.34E-01		-2.85E+00	1.42E-163	Down	-2.27E+00	4.79E-130	Down			Unigene116949_All//9.00E-134//gi|13245508|gb|BE052503.2|BE052503				
Unigene11695_All	1295	1.82	0.63	1.40	4.92	3.94	2.79	2.12	1.10	2.31	-1.54E+00	4.45E-04	Down	-3.79E-01	3.92E-01		-3.23E-01	1.80E-01		-8.20E-01	3.47E-04		-9.52E-01	5.03E-03		1.21E-01	7.13E-01		Unigene11695_All//gi|195605628|gb|ACG24644.1|//3.00E-58//protein SYS1 [Zea mays]	Unigene11695_All//1.00E-57//AT1G79990| LOCATED IN: endomembrane system, COPI vesicle coat, Golgi membrane; EXPRESSED IN: 25 plant structures; EXPRESSED DURING: 15 growth stages; CONTAINS InterPro DOMAIN/s: SYS1 homologue (InterPro:IPR016973); Has 55556 Blast hits to 24059 proteins in 620 species: Archae - 38; Bacteria - 5697; Metazoa - 25539; Fungi - 10898; Plants - 5309; Viruses - 0; Other Eukaryotes - 8075 (source: NCBI BLink).	Unigene11695_All//0.00E+00//TC174885			GO:0015031//GO:0030120	nr -
Unigene116955_All	271	8.69	5.42	5.89	34.74	23.38	25.66	105.62	85.29	27.49	-6.80E-01	1.01E-01		-5.60E-01	2.06E-01		-5.71E-01	1.09E-03		-4.37E-01	2.10E-02		-3.09E-01	7.34E-04		-1.94E+00	3.41E-71	Down	Unigene116955_All//gi|15241844|ref|NP_198206.1|//7.00E-19//BIP1; ATP binding [Arabidopsis thaliana] >gi|18206379|sp|Q9LKR3.1|BIP1_ARATH RecName: Full=Luminal-binding protein 1; Short=BiP1; Short=AtBP1; Flags: Precursor >gi|9502169|gb|AAF88019.1| Hypothetical protein T26D3.10 [Arabidopsis thaliana] >gi|23306406|gb|AAN17430.1| Unknown protein [Arabidopsis thaliana] >gi|24899769|gb|AAN65099.1| Unknown protein [Arabidopsis thaliana]	Unigene116955_All//3.00E-20//AT5G28540| BIP1; ATP binding	Unigene116955_All//7.00E-147//TC179555		ko04141|Protein processing in endoplasmic reticulum//ko03060|Protein export	GO:0000741//GO:0006950//GO:0009536//GO:0016020//GO:0016628//GO:0030312//GO:0031410//GO:0032559//GO:0043161//GO:0044432	nr +
Unigene116964_All	271	0.00	1.12	1.96	0.00	1.02	2.67	0.94	1.39	0.74	1.01E+01	4.44E-02		1.09E+01	4.05E-03		9.99E+00	7.02E-02		1.14E+01	2.31E-04	Up	5.68E-01	6.38E-01		-3.49E-01	7.67E-01				Unigene116964_All//6.00E-21//BG444481				
Unigene116968_All	272	21.93	38.75	13.12	41.57	27.98	41.84	21.61	31.19	10.11	8.21E-01	3.58E-06		-7.41E-01	3.34E-03		-5.71E-01	2.71E-04		9.30E-03	9.57E-01		5.30E-01	2.83E-03		-1.10E+00	4.95E-07	Down			Unigene116968_All//2.00E-57//gi|72280248|gb|DT052441.1|DT052441				
Unigene116973_All	272	1.54	1.86	1.37	3.30	1.65	0.33	1.55	5.53	1.46	2.76E-01	7.64E-01		-1.67E-01	8.79E-01		-1.00E+00	1.61E-01		-3.31E+00	5.80E-04	Down	1.83E+00	4.72E-04	Up	-8.58E-02	9.39E-01		Unigene116973_All//gi|297799832|ref|XP_002867800.1|//8.00E-17//PRH26 protein [Arabidopsis lyrata subsp. lyrata] >gi|297313636|gb|EFH44059.1| PRH26 protein [Arabidopsis lyrata subsp. lyrata]	Unigene116973_All//7.00E-18//AT4G21990| APR3 (APS REDUCTASE 3); adenylyl-sulfate reductase	Unigene116973_All//8.00E-79//TC149017			GO:0000097//GO:0009532//GO:0016671//GO:0019725//GO:0031976	nr -
Unigene116979_All	273	0.00	0.00	0.00	0.00	0.00	0.00	1.08	4.44	0.00	null	null		null	null		null	null		null	null		2.03E+00	8.61E-04	Up	-1.01E+01	1.58E-02		Unigene116979_All//gi|255076213|ref|XP_002501781.1|//2.00E-26//histone H2B [Micromonas sp. RCC299] >gi|226517045|gb|AC063039.1| histone H2B [Micromonas sp. RCC299]	Unigene116979_All//1.00E-27//AT3G09480| histone H2B, putative				GO:0000785//GO:0003676//GO:0034728//GO:0043231	nr +
Unigene11698_All	600	1.05	1.35	1.69	2.74	2.47	2.86	5.64	6.06	11.69	3.69E-01	6.22E-01		6.88E-01	3.42E-01		-1.51E-01	8.02E-01		6.21E-02	9.25E-01		1.04E-01	7.34E-01		1.05E+00	1.11E-07	Up	Unigene11698_All//gi|297601401|ref|NP_001050782.2|//3.00E-24//Os03g0649000 [Oryza sativa Japonica Group] >gi|255674749|dbj|BAF12696.2| Os03g0649000 [Oryza sativa Japonica Group]	Unigene11698_All//1.00E-50//AT3G11760| unknown protein	Unigene11698_All//0.00E+00//TC166259			GO:0043231	nr -
Unigene116987_All	274	0.00	0.00	0.00	0.36	0.00	0.82	0.62	6.41	1.16	null	null		null	null		-8.51E+00	3.15E-01		1.18E+00	5.50E-01		3.38E+00	2.17E-08	Up	9.14E-01	4.53E-01				Unigene116987_All//2.00E-104//CO101692				
Unigene116998_All	275	0.00	0.18	0.58	0.72	0.75	0.16	0.00	4.56	0.87	7.53E+00	6.19E-01		9.18E+00	2.39E-01		5.22E-02	9.89E-01		-2.14E+00	3.47E-01		1.22E+01	7.17E-09	Up	9.76E+00	4.40E-02				Unigene116998_All//2.00E-11//TC142238				
Unigene1170_All	1751	18.23	14.93	12.47	1.48	4.72	4.85	2.54	21.08	127.27	-2.88E-01	2.99E-03		-5.48E-01	2.26E-08		1.67E+00	1.13E-15	Up	1.71E+00	2.17E-15	Up	3.06E+00	2.45E-150	Up	5.65E+00	0.00E+00	Up	Unigene1170_All//gi|297827611|ref|XP_002881688.1|//1.00E-166//transferase family protein [Arabidopsis lyrata subsp. lyrata] >gi|297327527|gb|EFH57947.1| transferase family protein [Arabidopsis lyrata subsp. lyrata]	Unigene1170_All//1.00E-160//AT2G39980| transferase family protein	Unigene1170_All//4.00E-94//DR461983		ko00940|Phenylpropanoid biosynthesis//ko00945|Stilbenoid, diarylheptanoid and gingerol biosynthesis//ko00941|Flavonoid biosynthesis		nr -
Unigene117002_All	276	0.00	2.57	0.00	0.72	0.12	0.65	2.30	3.94	1.59	1.13E+01	3.33E-04	Up	null	null		-2.53E+00	2.34E-01		-1.41E-01	9.35E-01		7.77E-01	1.94E-01		-5.33E-01	4.94E-01				Unigene117002_All//3.00E-07//ES842675				
Unigene117003_All	276	0.00	0.00	0.00	0.00	0.00	0.00	2.14	1.82	0.00	null	null		null	null		null	null		null	null		-2.39E-01	7.65E-01		-1.11E+01	1.41E-04	Down	Unigene117003_All//gi|254933823|gb|ACT87561.1|//1.00E-12//60S ribosomal protein L14 [Amoebophrya sp. ex Karlodinium veneficum]	Unigene117003_All//1.00E-07//AT4G27090| 60S ribosomal protein L14 (RPL14B)			ko03010|Ribosome	GO:0009536//GO:0015934//GO:0016020//GO:0031981	nr -
Unigene117008_All	276	0.00	0.00	0.00	0.00	0.00	0.00	0.00	1.97	0.00	null	null		null	null		null	null		null	null		1.09E+01	5.36E-04	Up	null	null		Unigene117008_All//gi|298714497|emb|CBJ27519.1|//1.00E-06//eukaryotic elongation factor-1B gamma [Ectocarpus siliculosus]	Unigene117008_All//1.00E-05//AT1G09640| elongation factor 1B-gamma, putative / eEF-1B gamma, putative					nr -
Unigene117010_All	277	0.00	0.00	0.00	0.00	0.00	0.00	0.00	2.26	0.00	null	null		null	null		null	null		null	null		1.11E+01	1.47E-04	Up	null	null		Unigene117010_All//gi|38325811|gb|AAR17078.1|//3.00E-43//heat shock protein 70-1 [Nicotiana tabacum]	Unigene117010_All//1.00E-34//AT3G12580| HSP70 (heat shock protein 70); ATP binding	Unigene117010_All//1.00E-12//TC166610		ko03040|Spliceosome//ko04141|Protein processing in endoplasmic reticulum//ko04144|Endocytosis	GO:0008152//GO:0016628//GO:0032559//GO:0050896	nr +
Unigene117011_All	277	0.00	0.00	0.00	0.36	0.37	0.00	0.00	2.41	0.29	null	null		null	null		5.20E-02	9.67E-01		-8.49E+00	4.03E-01		1.12E+01	7.64E-05	Up	8.17E+00	4.05E-01				Unigene117011_All//8.00E-153//ES809411				
Unigene117016_All	277	0.94	0.00	0.38	0.90	0.62	0.65	1.07	6.04	1.73	-9.88E+00	6.72E-02		-1.30E+00	4.37E-01		-5.33E-01	7.13E-01		-4.63E-01	7.45E-01		2.50E+00	3.97E-06	Up	6.92E-01	4.63E-01				Unigene117016_All//5.00E-40//TC154162				
Unigene117017_All	277	0.00	0.00	0.00	0.00	0.00	0.00	1.83	1.21	0.00	null	null		null	null		null	null		null	null		-6.02E-01	5.32E-01		-1.08E+01	5.54E-04	Down	Unigene117017_All//gi|32394540|gb|AAM93968.1|//1.00E-15//Gbp1 [Griffithsia japonica]	Unigene117017_All//1.00E-09//AT1G02840| SR1; RNA binding / nucleic acid binding / nucleotide binding			ko03040|Spliceosome	GO:0046872	nr -
Unigene117024_All	278	0.00	0.00	0.00	0.00	0.00	0.00	20.08	16.69	0.00	null	null		null	null		null	null		null	null		-2.67E-01	2.76E-01		-1.43E+01	4.80E-41	Down	Unigene117024_All//gi|168068200|ref|XP_001785975.1|//6.00E-14//predicted protein [Physcomitrella patens subsp. patens] >gi|162662338|gb|EDQ49215.1| predicted protein [Physcomitrella patens subsp. patens]		Unigene117024_All//1.00E-28//gi|78348172|gb|DT568446.1|DT568446				nr -
Unigene117026_All	278	0.38	0.00	0.00	0.36	0.00	0.00	1.37	5.41	3.01	-8.56E+00	3.75E-01		-8.56E+00	3.96E-01		-8.49E+00	3.15E-01		-8.49E+00	4.03E-01		1.98E+00	2.06E-04	Up	1.14E+00	9.20E-02				Unigene117026_All//3.00E-07//TC154702				
Unigene117027_All	278	0.00	0.73	0.00	0.00	0.00	0.00	0.30	6.92	0.72	9.51E+00	1.37E-01		null	null		null	null		null	null		4.51E+00	4.15E-11	Up	1.24E+00	4.69E-01		Unigene117027_All//gi|3341464|emb|CAA12387.1|//8.00E-19//Hsp20.1 protein [Solanum peruvianum]	Unigene117027_All//4.00E-17//AT2G29500| 17.6 kDa class I small heat shock protein (HSP17.6B-CI)	Unigene117027_All//1.00E-108//TC145915				nr +

Unigene117028_All	278	0.00	0.00	0.00	0.00	0.00	0.00	0.00	2.56	0.00	null	null		null	null		null	null		null	null		1.13E+01	3.98E-05	Up	null	null		Unigene117028_All//gi|38325811|gb|AAR17078.1|//1.00E-39//heat shock protein 70-1 [Nicotiana tabacum]	Unigene117028_All//5.00E-32//AT3G09440| heat shock cognate 70 kDa protein 3 (HSC70-3) (HSP70-3)	Unigene117028_All//5.00E-12//EX170481		ko03040|Spliceosome//ko04141|Protein processing in endoplasmic reticulum//ko04144|Endocytosis		nr +
Unigene11703_All	1944	15.29	8.78	10.71	3.84	6.59	7.99	1.35	2.19	1.23	-7.99E-01	2.65E-15		-5.13E-01	4.73E-07		7.78E-01	1.50E-07		1.06E+00	1.39E-13	Up	7.02E-01	7.42E-03		-1.33E-01	6.99E-01		Unigene11703_All//gi|297796055|ref|XP_002865912.1|//1.00E-132//hydroxyproline-rich glycoprotein family protein [Arabidopsis lyrata subsp. lyrata] >gi|297311747|gb|EFH42171.1| hydroxyproline-rich glycoprotein family protein [Arabidopsis lyrata subsp. lyrata]	Unigene11703_All//5.00E-118//AT4G25620| hydroxyproline-rich glycoprotein family protein	Unigene11703_All//0.00E+00//TC144318				nr +
Unigene11704_All	2079	2.24	10.87	3.59	2.68	3.89	4.00	3.13	3.32	4.73	2.28E+00	1.92E-54	Up	6.79E-01	2.20E-03		5.37E-01	6.61E-03		5.75E-01	4.02E-03		8.29E-02	7.20E-01		5.96E-01	1.73E-04		Unigene11704_All//gi|224067156|ref|XP_002302383.1|//1.00E-173//amino acid transporter [Populus trichocarpa] >gi|222844109|gb|EEE81656.1| amino acid transporter [Populus trichocarpa]	Unigene11704_All//6.00E-132//AT3G30390| amino acid transporter family protein	Unigene11704_All//0.00E+00//gi|109861836|gb|DW490813.1|DW490813			GO:0031224	nr -
Unigene117042_All	280	0.00	0.00	0.00	0.00	0.00	0.00	0.00	5.22	0.00	null	null		null	null		null	null		null	null		1.24E+01	2.52E-10	Up	null	null		Unigene117042_All//gi|42565109|ref|NP_188851.2|//4.00E-08//CWLP (CELL WALL-PLASMA MEMBRANE LINKER PROTEIN); lipid binding [Arabidopsis thaliana] >gi|124301006|gb|ABN04755.1| At3g22120 [Arabidopsis thaliana]						nr +
Unigene117045_All	281	0.00	0.00	0.00	1.77	0.37	0.16	0.30	34.36	0.14	null	null		null	null		-2.27E+00	4.22E-02		-3.46E+00	1.61E-02		6.84E+00	2.12E-64	Up	-1.09E+00	6.77E-01		Unigene117045_All//gi|224073330|ref|XP_002304078.1|//1.00E-12//NAC domain protein, IPR003441 [Populus trichocarpa] >gi|222841510|gb|EEE79057.1| NAC domain protein, IPR003441 [Populus trichocarpa]	Unigene117045_All//8.00E-14//AT5G64530| XND1; transcription factor		NAC			nr +
Unigene117046_All	281	0.00	0.00	0.00	0.53	0.37	0.00	1.20	5.50	0.14	null	null		null	null		-5.33E-01	7.85E-01		-9.05E+00	2.34E-01		2.19E+00	5.11E-05	Up	-3.09E+00	3.92E-02				Unigene117046_All//4.00E-68//DW508429				
Unigene117051_All	281	0.56	6.31	1.14	0.00	0.00	0.32	0.15	1.19	0.43	3.50E+00	3.83E-07	Up	1.03E+00	4.71E-01		null	null		8.33E+00	4.79E-01		2.98E+00	5.74E-02		1.50E+00	5.58E-01				Unigene117051_All//3.00E-38//TC173468				
Unigene117053_All	281	0.00	0.36	0.00	0.00	0.00	0.00	293.55	57.71	76.70	8.49E+00	3.89E-01		null	null		null	null		null	null		-2.35E+00	9.02E-252	Down	-1.94E+00	5.33E-203	Down	Unigene117053_All//gi|255045895|gb|ACU00104.1|//2.00E-15//plasma membrane intrinsic protein PIP2.2 [Populus trichocarpa x Populus deltoides]	Unigene117053_All//2.00E-13//AT3G54820| PIP2;5 (PLASMA MEMBRANE INTRINSIC PROTEIN 2;5); water channel	Unigene117053_All//2.00E-98//gi|31407371|gb|CD486406.1|CD486406				nr +
Unigene117060_All	282	0.00	0.00	0.00	0.00	0.24	0.00	0.30	2.82	0.71	null	null		null	null		7.93E+00	6.13E-01		null	null		3.23E+00	5.44E-04	Up	1.24E+00	4.69E-01		Unigene117060_All//gi|92886073|gb|ABE88083.1|//1.00E-07//Protein kinase [Medicago truncatula]	Unigene117060_All//4.00E-08//AT4G05200| protein kinase family protein					nr -
Unigene117061_All	283	0.00	0.00	0.00	0.00	0.00	0.00	0.00	2.07	0.00	null	null		null	null		null	null		null	null		1.10E+01	2.81E-04	Up	null	null		Unigene117061_All//gi|38325811|gb|AAR17078.1|//7.00E-44//heat shock protein 70-1 [Nicotiana tabacum]	Unigene117061_All//3.00E-34//AT1G09080| BIP3; ATP binding	Unigene117061_All//1.00E-12//gi|78350754|gb|DT571028.1|DT571028		ko03040|Spliceosome//ko04141|Protein processing in endoplasmic reticulum//ko04144|Endocytosis//ko03060|Protein export		nr -
Unigene117068_All	284	4539.38	2046.05	4863.94	698.80	824.75	499.18	0.45	1.91	0.84	-1.15E+00	0.00E+00	Down	9.96E-02	1.44E-13		2.39E-01	2.07E-15		-4.85E-01	7.76E-44		2.10E+00	3.71E-02		9.14E-01	5.42E-01		Unigene117068_All//gi|231610|sp|P29790.1|ATPG_TOBAC//3.00E-24//RecName: Full=ATP synthase gamma chain, chloroplastic; AltName: Full=F-ATPase gamma subunit; Flags: Precursor >gi|19785|emb|CAA45152.1| ATP synthase (gamma subunit) [Nicotiana tabacum]		Unigene117068_All//2.00E-159//gi|164250171|gb|EX167616.1|EX167616		ko00190|Oxidative phosphorylation//ko00195|Photosynthesis		nr +
Unigene11707_All	1201	14.72	34.55	17.56	28.04	30.48	42.87	35.87	57.91	72.78	1.23E+00	4.92E-42	Up	2.54E-01	5.05E-02		1.20E-01	2.34E-01		6.13E-01	1.48E-17		6.91E-01	4.12E-33		1.02E+00	1.35E-81	Up	Unigene11707_All//gi|42570941|ref|NP_973544.1|//1.00E-120//OTU-like cysteine protease family protein [Arabidopsis thaliana] >gi|79602271|ref|NP_973546.2| OTU-like cysteine protease family protein [Arabidopsis thaliana] >gi|186503523|ref|NP_973545.2| OTU-like cysteine protease family protein [Arabidopsis thaliana] >gi|110735090|gb|ABG89115.1| OTU-containing protein 6 [synthetic construct]	Unigene11707_All//3.00E-109//AT2G27350| OTU-like cysteine protease family protein	Unigene11707_All//0.00E+00//TC136519			GO:0043231	nr -
Unigene117079_All	286	0.00	0.00	0.00	0.00	0.00	0.00	0.00	2.34	0.00	null	null		null	null		null	null		null	null		1.12E+01	7.64E-05	Up	null	null		Unigene117079_All//gi|50843988|gb|AAT84169.1|//1.00E-26//60S ribosomal protein L27 [Chara globularis]	Unigene117079_All//5.00E-24//AT3G22230| 60S ribosomal protein L27 (RPL27B)			ko03010|Ribosome		nr -
Unigene11708_All	2215	4.84	7.87	5.12	7.74	8.05	9.36	9.07	13.62	23.40	7.01E-01	1.52E-07		8.06E-02	6.63E-01		5.79E-02	7.49E-01		2.75E-01	2.86E-02		5.87E-01	2.48E-11		1.37E+00	5.01E-77	Up	Unigene11708_All//gi|226494281|ref|NP_001151795.1|//1.00E-141//cysteine-type peptidase [Zea mays] >gi|195649739|gb|ACG44337.1| cysteine-type peptidase [Zea mays]	Unigene11708_All//1.00E-111//AT2G27350| OTU-like cysteine protease family protein	Unigene11708_All//0.00E+00//TC153990			GO:0043231	nr +
Unigene117083_All	286	0.00	0.00	0.00	0.00	0.24	0.00	0.15	3.07	0.14	null	null		null	null		7.91E+00	6.12E-01		null	null		4.38E+00	3.08E-05	Up	-8.55E-02	9.73E-01				Unigene117083_All//3.00E-66//DT467436				
Unigene117089_All	287	0.18	0.00	0.00	0.00	0.00	0.16	1.18	1.60	5.27	-7.51E+00	6.16E-01		-7.51E+00	6.28E-01		null	null		7.30E+00	6.99E-01		4.43E-01	6.32E-01		2.16E+00	5.30E-05	Up	Unigene117089_All//gi|164604966|dbj|BAF98279.1|//5.00E-31//hydroxymethylglutaryl-CoA synthase [Hevea brasiliensis]	Unigene117089_All//7.00E-28//AT4G11820| MVA1; acetyl-CoA C-acetyltransferase/ hydroxymethylglutaryl-CoA synthase	Unigene117089_All//3.00E-118//gi|13248420|gb|BF275790.2|BF275790		ko00280|Valine, leucine and isoleucine degradation//ko00650|Butanoate metabolism//ko00900|Terpenoid backbone biosynthesis//ko00072|Synthesis and degradation of ketone bodies		nr +
Unigene11709_All	490	12.71	29.88	27.60	21.15	25.72	23.69	19.59	23.46	21.55	1.23E+00	2.30E-15	Up	1.12E+00	7.68E-12	Up	2.83E-01	8.61E-02		1.64E-01	4.00E-01		2.60E-01	1.02E-01		1.38E-01	4.23E-01		Unigene11709_All//gi|89257511|gb|ABD65001.1|//5.00E-41//Mo25 family protein [Brassica oleracea]	Unigene11709_All//3.00E-40//AT5G47540| binding	Unigene11709_All//0.00E+00//gi|78351728|gb|DT572002.1|DT572002			GO:0016020	nr +
Unigene117091_All	287	77.84	31.60	48.24	41.83	31.20	22.66	1.47	2.33	0.69	-1.30E+00	4.49E-25	Down	-6.90E-01	8.24E-09		-4.23E-01	6.77E-03		-8.84E-01	3.54E-08		6.61E-01	4.02E-01		-1.09E+00	2.82E-01								ESTscan -
Unigene117096_All	288	0.00	0.00	0.00	0.17	0.00	0.16	0.00	1.89	0.55	null	null		null	null		-7.43E+00	5.59E-01		-1.42E-01	9.55E-01		1.09E+01	5.36E-04	Up	9.11E+00	1.40E-01		Unigene117096_All//gi|46931304|gb|AAT06456.1|//1.00E-26//At5g50410 [Arabidopsis thaliana]	Unigene117096_All//4.00E-28//AT5G50410| unknown protein	Unigene117096_All//2.00E-51//DR453384				nr -
Unigene117097_All	288	0.18	2.29	0.00	0.35	0.84	0.31	0.15	2.61	0.42	3.65E+00	4.10E-03		-7.51E+00	6.28E-01		1.27E+00	5.32E-01		-1.41E-01	9.51E-01		4.15E+00	1.92E-04	Up	1.50E+00	5.58E-01				Unigene117097_All//2.00E-110//TC147893				
Unigene117099_All	288	881.82	376.15	293.80	799.97	847.37	509.81	329.14	134.97	200.99	-1.23E+00	8.52E-255	Down	-1.59E+00	0.00E+00	Down	8.31E-02	1.31E-02		-6.50E-01	7.69E-86		-1.29E+00	3.65E-125	Down	-7.12E-01	1.56E-48		Unigene117099_All//gi|7387726|sp|O22582.3|H2B_GOSHI//2.00E-29//RecName: Full=Histone H2B >gi|2558962|gb|AAB97163.1| histone H2B1 [Gossypium hirsutum]		Unigene117099_All//4.00E-142//TC144873				nr -
Unigene1171_All	1560	2.52	3.96	3.14	3.54	7.90	25.94	8.10	8.63	35.04	6.56E-01	5.80E-03		3.20E-01	2.79E-01		1.16E+00	1.47E-13	Up	2.87E+00	4.99E-135	Up	9.03E-02	6.09E-01		2.11E+00	5.54E-149	Up	Unigene1171_All//gi|297827611|ref|XP_002881688.1|//1.00E-178//transferase family protein [Arabidopsis lyrata subsp. lyrata] >gi|297327527|gb|EFH57947.1| transferase family protein [Arabidopsis lyrata subsp. lyrata]	Unigene1171_All//4.00E-174//AT2G39980| transferase family protein	Unigene1171_All//0.00E+00//TC162451		ko00940|Phenylpropanoid biosynthesis//ko00945|Stilbenoid, diarylheptanoid and gingerol biosynthesis//ko00941|Flavonoid biosynthesis	GO:0016747	nr +
Unigene11710_All	715	27.44	30.54	34.71	26.06	25.67	27.29	22.77	17.54	9.36	1.55E-01	2.31E-01		3.39E-01	3.22E-03		-2.16E-02	8.88E-01		6.66E-02	6.74E-01		-3.77E-01	2.46E-03		-1.28E+00	1.00E-22	Down	Unigene11710_All//gi|240256410|ref|NP_199565.4|//5.00E-86//binding [Arabidopsis thaliana] >gi|15214076|sp|Q9FGK3.1|MO25N_ARATH RecName: Full=Putative MO25-like protein At5g47540 >gi|16226301|gb|AAL16128.1|AF428296_1 AT5g47540/MNJ7_13 [Arabidopsis thaliana] >gi|193211497|gb|ACF16168.1| At5g47540 [Arabidopsis thaliana]	Unigene11710_All//3.00E-87//AT5G47540| binding	Unigene11710_All//0.00E+00//TC136454			GO:0016020	nr +
Unigene117104_All	289	0.00	0.00	0.00	0.00	0.00	0.00	0.15	2.89	0.14	null	null		null	null		null	null		null	null		4.31E+00	5.67E-05	Up	-8.64E-02	9.73E-01		Unigene117104_All//COG0438//6.00E-12//Glycosyltransferase						cog +
Unigene117106_All	289	0.00	0.00	0.00	0.00	0.00	0.00	2.78	7.38	0.00	null	null		null	null		null	null		null	null		1.41E+00	5.52E-04	Up	-1.14E+01	4.43E-06	Down							ESTscan +
Unigene117108_All	290	0.00	0.00	0.00	0.00	0.00	0.00	0.00	2.31	0.00	null	null		null	null		null	null		null	null		1.12E+01	7.64E-05	Up	null	null		Unigene117108_All//gi|3107919|dbj|BAA25911.1|//2.00E-09//actin [Nannochloris bacillaris]	Unigene117108_All//2.00E-10//AT5G09810| ACT7 (ACTIN 7); structural constituent of cytoskeleton	Unigene117108_All//4.00E-13//TC129604		ko04145|Phagosome		nr +
Unigene117112_All	290	0.00	0.00	0.00	0.00	0.00	0.00	2.33	2.74	0.14	null	null		null	null		null	null		null	null		2.31E-01	7.35E-01		-4.09E+00	3.10E-04	Down	Unigene117112_All//gi|112253381|gb|ABI14278.1|//2.00E-29//translation elongation factor P [Pfiesteria piscicida] >gi|112253383|gb|ABI14279.1| translation initiation factor 5A [Pfiesteria piscicida]	Unigene117112_All//4.00E-28//AT1G26630| FBR12 (FUMONISIN B1-RESISTANT12); translation initiation factor					nr +
Unigene117120_All	291	0.00	0.00	0.00	0.00	0.00	0.00	1.89	4.60	0.00	null	null		null	null		null	null		null	null		1.28E+00	1.56E-02		-1.09E+01	2.81E-04	Down	Unigene117120_All//gi|44917163|dbj|BAD12182.1|//1.00E-32//14-3-3 i-1 protein [Nicotiana tabacum]	Unigene117120_All//2.00E-32//AT1G26480| GRF12 (GENERAL REGULATORY FACTOR 12); protein binding / protein phosphorylated amino acid binding	Unigene117120_All//6.00E-21//gi|5046419|gb|AI727567.1|AI727567				nr -
Unigene117122_All	291	221.87	201.98	223.80	341.51	392.87	388.00	74.54	118.92	160.45	-1.36E-01	5.28E-02		1.25E-02	8.78E-01		2.02E-01	8.66E-06		1.84E-01	1.43E-04		6.74E-01	3.29E-16		1.11E+00	4.96E-50	Up	Unigene117122_All//gi|300510880|gb|ADK25058.1|//2.00E-10//AN1-like transcription factor [Glycine max]	Unigene117122_All//1.00E-08//AT3G12630| zinc finger (AN1-like) family protein	Unigene117122_All//1.00E-120//gi|164266342|gb|ES822249.1|ES822249				nr -
Unigene117124_All	292	0.00	0.00	0.00	0.00	0.00	0.00	0.00	1.86	0.00	null	null		null	null		null	null		null	null		1.09E+01	5.36E-04	Up	null	null		Unigene117124_All//gi|32401377|gb|AAP80858.1|//5.00E-39//ribosomal protein L19 [Triticum aestivum]	Unigene117124_All//3.00E-22//AT1G02780| emb2386 (embryo defective 2386); structural constituent of ribosome	Unigene117124_All//3.00E-07//gi|11200775|gb|BF269780.1|BF269780		ko03010|Ribosome		nr -
Unigene117126_All	292	0.00	0.00	0.00	0.00	0.00	0.00	2.32	2.86	0.00	null	null		null	null		null	null		null	null		3.05E-01	6.47E-01		-1.12E+01	3.56E-05	Down	Unigene117126_All//gi|195618310|gb|ACG30985.1|//2.00E-22//60S acidic ribosomal protein P1 [Zea mays] >gi|195625300|gb|ACG34480.1| 60S acidic ribosomal protein P1 [Zea mays]	Unigene117126_All//1.00E-08//AT5G24510| 60s acidic ribosomal protein P1, putative			ko03010|Ribosome		nr +
Unigene11713_All	425	23.76	17.41	7.52	46.88	32.90	37.83	40.59	35.90	21.47	-4.49E-01	1.33E-02		-1.66E+00	7.79E-16	Down	-5.11E-01	6.76E-06		-3.10E-01	1.38E-02		-1.77E-01	1.80E-01		-9.19E-01	2.29E-14				Unigene11713_All//0.00E+00//TC139822				
Unigene117134_All	293	0.00	0.00	0.00	0.00	0.00	0.00	1.44	5.42	0.00	null	null		null	null		null	null		null	null		1.91E+00	1.97E-04	Up	-1.05E+01	2.14E-03								ESTscan -
Unigene117142_All	295	0.00	0.00	0.00	0.00	0.00	0.00	0.00	6.38	0.00	null	null		null	null		null	null		null	null		1.26E+01	3.04E-13	Up	null	null		Unigene117142_All//sp|Q99334|MT1_COLGL//1.00E-06//Metallothionein-like protein CAP3 OS=Colletotrichum gloeosporioides GN=CAP3 PE=2 SV=1						swissprot +
Unigene117147_All	296	0.00	0.00	0.00	0.17	0.00	0.00	0.00	1.84	0.27	null	null		null	null		-7.39E+00	5.59E-01		-7.39E+00	6.40E-01		1.08E+01	5.36E-04	Up	8.07E+00	4.05E-01		Unigene117147_All//gi|21311555|gb|AAM46778.1|AF466103_1//1.00E-41//15.8 kDa oleosin [Theobroma cacao]	Unigene117147_All//9.00E-18//AT2G25890| glycine-rich protein / oleosin	Unigene117147_All//4.00E-164//TC144426				nr +
Unigene11715_All	345	12.74	5.73	6.17	11.55	6.99	10.47	16.79	8.60	4.85	-1.15E+00	9.42E-05	Down	-1.05E+00	6.04E-04	Down	-7.25E-01	1.20E-02		-1.41E-01	6.92E-01		-9.65E-01	1.38E-05		-1.79E+00	1.35E-13	Down	Unigene11715_All//gi|86611379|gb|ABD14373.1|//2.00E-34//cyclin-dependent kinase type A [Prunus dulcis]	Unigene11715_All//2.00E-34//AT3G48750| CDC2 (CELL DIVISION CONTROL 2); cyclin-dependent protein kinase/ kinase/ protein binding / protein kinase	Unigene11715_All//2.00E-175//gi|164338395|gb|ES829952.1|ES829952			GO:0000087//GO:0004674//GO:0006464//GO:0032559	nr +

<table>
<tr><td>Unigene117157_All</td><td>297</td><td>0.00</td><td>0.00</td><td>0.00</td><td>0.00</td><td>0.00</td><td>0.00</td><td>0.57</td><td>3.52</td><td>0.00</td><td>null</td><td>null</td><td></td><td>null</td><td>null</td><td></td><td>null</td><td>null</td><td></td><td>null</td><td>null</td><td></td><td>2.63E+00</td><td>2.86E-04</td><td>Up</td><td>-9.15E+00</td><td>1.08E-01</td><td></td><td>Unigene117157_All//gi|18403247|ref|NP_566697.1|//9.00E-13//receptor protein kinase-related [Arabidopsis thaliana] >gi|75273758|sp|Q9LRJ9.1|CRR38_ARATH RecName: Full=Cysteine-rich repeat secretory protein 38; Flags: Precursor >gi|90186230|gb|ABD91491.1| At3g22060 [Arabidopsis thaliana]</td><td>Unigene117157_All//3.00E-14//AT3G22060| receptor protein kinase-related</td><td></td><td></td><td></td><td></td><td>nr -</td></tr>
<tr><td>Unigene11716_All</td><td>1617</td><td>8.61</td><td>6.77</td><td>7.74</td><td>16.97</td><td>15.35</td><td>15.67</td><td>17.36</td><td>10.68</td><td>7.86</td><td>-3.47E-01</td><td>2.39E-02</td><td></td><td>-1.53E-01</td><td>3.92E-01</td><td></td><td>-1.45E-01</td><td>2.09E-01</td><td></td><td>-1.16E-01</td><td>3.54E-01</td><td></td><td>-7.02E-01</td><td>3.12E-14</td><td></td><td>-1.14E+00</td><td>2.66E-32</td><td>Down</td><td>Unigene11716_All//gi|157683271|gb|ABV64386.1|//1.00E-161//cyclin-dependent kinase A [Gossypium hirsutum]</td><td>Unigene11716_All//2.00E-143//AT3G48750| CDC2 (CELL DIVISION CONTROL 2); cyclin-dependent protein kinase/ kinase/ protein binding / protein kinase</td><td>Unigene11716_All//0.00E+00//gi|164248123|gb|ES806283.1|ES806283</td><td></td><td></td><td></td><td>nr +</td></tr>
<tr><td>Unigene117162_All</td><td>299</td><td>0.00</td><td>0.34</td><td>0.18</td><td>0.50</td><td>0.23</td><td>0.00</td><td>0.00</td><td>1.96</td><td>0.67</td><td>8.40E+00</td><td>3.89E-01</td><td></td><td>7.48E+00</td><td>6.38E-01</td><td></td><td>-1.12E+00</td><td>5.84E-01</td><td></td><td>-8.97E+00</td><td>2.34E-01</td><td></td><td>1.09E+01</td><td>2.81E-04</td><td>Up</td><td>9.38E+00</td><td>7.90E-02</td><td></td><td></td><td></td><td></td><td></td><td></td><td></td><td></td></tr>
<tr><td>Unigene117163_All</td><td>299</td><td>0.00</td><td>0.00</td><td>0.00</td><td>0.00</td><td>0.00</td><td>0.00</td><td>0.00</td><td>1.82</td><td>1.07</td><td>null</td><td>null</td><td></td><td>null</td><td>null</td><td></td><td>null</td><td>null</td><td></td><td>null</td><td>null</td><td></td><td>1.08E+01</td><td>5.36E-04</td><td>Up</td><td>1.01E+01</td><td>1.34E-02</td><td></td><td></td><td></td><td></td><td></td><td></td><td></td><td></td></tr>
<tr><td>Unigene117166_All</td><td>300</td><td>0.00</td><td>0.00</td><td>0.00</td><td>0.00</td><td>0.00</td><td>0.00</td><td>2.11</td><td>1.53</td><td>0.00</td><td>null</td><td>null</td><td></td><td>null</td><td>null</td><td></td><td>null</td><td>null</td><td></td><td>null</td><td>null</td><td></td><td>-4.64E-01</td><td>5.94E-01</td><td></td><td>-1.10E+01</td><td>7.11E-05</td><td>Down</td><td>Unigene117166_All//gi|255087726|ref|XP_002505786.1|//1.00E-18//histone chaperone [Micromonas sp. RCC299] >gi|226521056|gb|ACO67044.1| histone chaperone [Micromonas sp. RCC299]</td><td>Unigene117166_All//5.00E-19//AT3G28730| ATHMG (ARABIDOPSIS THALIANA HIGH MOBILITY GROUP); transcription factor</td><td></td><td></td><td>ko03410|Base excision repair</td><td></td><td>nr -</td></tr>
<tr><td>Unigene117168_All</td><td>300</td><td>0.00</td><td>0.00</td><td>0.00</td><td>0.00</td><td>0.00</td><td>0.00</td><td>0.00</td><td>6.97</td><td>0.00</td><td>null</td><td>null</td><td></td><td>null</td><td>null</td><td></td><td>null</td><td>null</td><td></td><td>null</td><td>null</td><td></td><td>1.28E+01</td><td>1.05E-14</td><td>Up</td><td>null</td><td>null</td><td></td><td>Unigene117168_All//gi|1655699|emb|CAA69032.1|//4.00E-13//pherophorin-S [Volvox carteri f. nagariensis]</td><td></td><td></td><td></td><td></td><td></td><td>nr +</td></tr>
<tr><td>Unigene11717_All</td><td>1804</td><td>11.60</td><td>6.40</td><td>7.91</td><td>17.40</td><td>14.07</td><td>10.87</td><td>15.16</td><td>7.32</td><td>4.59</td><td>-8.57E-01</td><td>2.62E-12</td><td></td><td>-5.52E-01</td><td>8.15E-06</td><td></td><td>-3.06E-01</td><td>8.08E-04</td><td></td><td>-6.79E-01</td><td>4.13E-13</td><td></td><td>-1.05E+00</td><td>9.23E-27</td><td>Down</td><td>-1.72E+00</td><td>7.20E-58</td><td>Down</td><td>Unigene11717_All//gi|17224978|gb|AAL37195.1|AF321361_1//1.00E-158//cyclin dependent kinase [Helianthus annuus]</td><td>Unigene11717_All//1.00E-145//AT3G48750| CDC2 (CELL DIVISION CONTROL 2); cyclin-dependent protein kinase/ kinase/ protein binding / protein kinase</td><td>Unigene11717_All//0.00E+00//TC151725</td><td></td><td></td><td></td><td>nr +</td></tr>
<tr><td>Unigene117171_All</td><td>301</td><td>0.00</td><td>0.00</td><td>0.00</td><td>0.00</td><td>0.11</td><td>0.15</td><td>0.00</td><td>2.22</td><td>0.00</td><td>null</td><td>null</td><td></td><td>null</td><td>null</td><td></td><td>6.84E+00</td><td>8.34E-01</td><td></td><td>7.23E+00</td><td>6.99E-01</td><td></td><td>1.11E+01</td><td>7.64E-05</td><td>Up</td><td>null</td><td>null</td><td></td><td></td><td></td><td></td><td></td><td></td><td></td><td></td></tr>
<tr><td>Unigene117176_All</td><td>301</td><td>0.00</td><td>0.00</td><td>0.00</td><td>0.00</td><td>0.00</td><td>0.00</td><td>3.65</td><td>1.81</td><td>0.26</td><td>null</td><td>null</td><td></td><td>null</td><td>null</td><td></td><td>null</td><td>null</td><td></td><td>null</td><td>null</td><td></td><td>-1.02E+00</td><td>8.43E-02</td><td></td><td>-3.79E+00</td><td>3.27E-06</td><td>Down</td><td></td><td></td><td></td><td></td><td></td><td></td><td>ESTscan -</td></tr>
<tr><td>Unigene117180_All</td><td>302</td><td>0.00</td><td>0.00</td><td>0.00</td><td>0.00</td><td>0.00</td><td>0.00</td><td>0.00</td><td>1.80</td><td>0.00</td><td>null</td><td>null</td><td></td><td>null</td><td>null</td><td></td><td>null</td><td>null</td><td></td><td>null</td><td>null</td><td></td><td>1.08E+01</td><td>5.36E-04</td><td>Up</td><td>null</td><td>null</td><td></td><td>Unigene117180_All//gi|17367847|sp|Q9XF97.1|RL4_PRUAR//2.00E-25//RecName: Full=60S ribosomal protein L4; AltName: Full=L1 >gi|4887131|gb|AAD32206.1|AF134732_1 60S ribosomal protein L1 [Prunus armeniaca]</td><td>Unigene117180_All//4.00E-24//AT3G09630| 60S ribosomal protein L4/L1 (RPL4A)</td><td></td><td></td><td>ko03010|Ribosome</td><td></td><td>nr -</td></tr>
<tr><td>Unigene11719_All</td><td>1677</td><td>582.95</td><td>50.70</td><td>194.01</td><td>323.54</td><td>312.05</td><td>125.63</td><td>96.84</td><td>7.03</td><td>35.75</td><td>-3.52E+00</td><td>0.00E+00</td><td>Down</td><td>-1.59E+00</td><td>0.00E+00</td><td>Down</td><td>-5.22E-02</td><td>2.09E-02</td><td></td><td>-1.36E+00</td><td>0.00E+00</td><td>Down</td><td>-3.78E+00</td><td>0.00E+00</td><td>Down</td><td>-1.44E+00</td><td>6.93E-261</td><td>Down</td><td>Unigene11719_All//gi|23452315|gb|AAN32988.1|//0.00E+00//beta-tubulin 1 [Gossypium hirsutum]</td><td>Unigene11719_All//0.00E+00//AT5G12250| TUB6 (BETA-6 TUBULIN); structural constituent of cytoskeleton</td><td>Unigene11719_All//0.00E+00//TC155561</td><td></td><td>ko04145|Phagosome</td><td>GO:0006970//GO:0007017//GO:0015630//GO:0016020//GO:0017111//GO:0032561//GO:0032991//GO:0043623</td><td>nr +</td></tr>
<tr><td>Unigene117190_All</td><td>304</td><td>0.00</td><td>0.00</td><td>0.00</td><td>0.00</td><td>0.00</td><td>0.00</td><td>0.28</td><td>3.02</td><td>0.00</td><td>null</td><td>null</td><td></td><td>null</td><td>null</td><td></td><td>null</td><td>null</td><td></td><td>null</td><td>null</td><td></td><td>3.44E+00</td><td>9.84E-05</td><td>Up</td><td>-8.12E+00</td><td>3.59E-01</td><td></td><td></td><td></td><td></td><td></td><td></td><td></td><td>ESTscan -</td></tr>
<tr><td>Unigene117195_All</td><td>305</td><td>0.00</td><td>0.00</td><td>0.00</td><td>0.82</td><td>0.79</td><td>0.30</td><td>8.87</td><td>2.33</td><td>2.87</td><td>null</td><td>null</td><td></td><td>null</td><td>null</td><td></td><td>-4.72E-02</td><td>9.52E-01</td><td></td><td>-1.46E+00</td><td>4.07E-01</td><td></td><td>-1.93E+00</td><td>3.86E-07</td><td>Down</td><td>-1.63E+00</td><td>4.80E-06</td><td>Down</td><td></td><td></td><td>Unigene117195_All//3.00E-07//DW509255</td><td></td><td></td><td></td><td></td></tr>
<tr><td>Unigene117197_All</td><td>305</td><td>0.00</td><td>0.00</td><td>0.35</td><td>0.65</td><td>0.79</td><td>0.74</td><td>15.66</td><td>5.34</td><td>1.44</td><td>null</td><td>null</td><td></td><td>8.45E+00</td><td>4.01E-01</td><td></td><td>2.75E-01</td><td>8.95E-01</td><td></td><td>1.81E-01</td><td>9.35E-01</td><td></td><td>-1.55E+00</td><td>4.27E-09</td><td>Down</td><td>-3.45E+00</td><td>4.00E-23</td><td>Down</td><td></td><td></td><td>Unigene117197_All//3.00E-07//FL576500</td><td></td><td></td><td></td><td></td></tr>
<tr><td>Unigene117198_All</td><td>306</td><td>0.00</td><td>0.83</td><td>0.00</td><td>1.63</td><td>0.00</td><td>0.00</td><td>1.38</td><td>2.32</td><td>7.03</td><td>9.69E+00</td><td>7.87E-02</td><td></td><td>null</td><td>null</td><td></td><td>-1.07E+01</td><td>8.98E-04</td><td>Down</td><td>-1.07E+01</td><td>2.98E-03</td><td></td><td>7.49E-01</td><td>3.41E-01</td><td></td><td>2.35E+00</td><td>2.10E-07</td><td>Up</td><td></td><td></td><td>Unigene117198_All//3.00E-66//DW485235</td><td></td><td></td><td></td><td></td></tr>
<tr><td>Unigene11720_All</td><td>1754</td><td>42.48</td><td>14.82</td><td>29.60</td><td>322.43</td><td>241.30</td><td>150.99</td><td>20.25</td><td>16.92</td><td>8.18</td><td>-1.52E+00</td><td>1.15E-103</td><td>Down</td><td>-5.21E-01</td><td>4.20E-17</td><td></td><td>-4.18E-01</td><td>1.23E-106</td><td></td><td>-1.09E+00</td><td>0.00E+00</td><td>Down</td><td>-2.59E-01</td><td>1.57E-03</td><td></td><td>-1.31E+00</td><td>4.82E-50</td><td>Down</td><td>Unigene11720_All//gi|54036488|sp|Q6VAF5.1|TBB7_GOSHI//0.00E+00//RecName: Full=Tubulin beta-7 chain; AltName: Full=Beta-7-tubulin >gi|37529498|gb|AAQ92667.1| beta-tubulin 7 [Gossypium hirsutum]</td><td>Unigene11720_All//0.00E+00//AT5G12250| TUB6 (BETA-6 TUBULIN); structural constituent of cytoskeleton</td><td>Unigene11720_All//0.00E+00//TC153064</td><td></td><td>ko04145|Phagosome</td><td>GO:0007017//GO:0015630//GO:0017111//GO:0032561//GO:0032991//GO:0043623</td><td>nr +</td></tr>
<tr><td>Unigene117201_All</td><td>306</td><td>0.00</td><td>0.00</td><td>0.00</td><td>0.00</td><td>0.00</td><td>0.00</td><td>0.00</td><td>3.14</td><td>0.00</td><td>null</td><td>null</td><td></td><td>null</td><td>null</td><td></td><td>null</td><td>null</td><td></td><td>null</td><td>null</td><td></td><td>1.16E+01</td><td>7.61E-07</td><td>Up</td><td>null</td><td>null</td><td></td><td>Unigene117201_All//gi|38325813|gb|AAR17079.1|//4.00E-31//heat shock protein 70-2 [Nicotiana tabacum]</td><td>Unigene117201_All//6.00E-30//AT3G12580| HSP70 (heat shock protein 70); ATP binding</td><td>Unigene117201_All//1.00E-22//gi|84149718|gb|DN781018.1|DN781018</td><td></td><td>ko03040|Spliceosome//ko04141|Protein processing in endoplasmic reticulum//ko04144|Endocytosis</td><td></td><td>nr -</td></tr>
<tr><td>Unigene117208_All</td><td>307</td><td>0.00</td><td>0.00</td><td>0.00</td><td>0.00</td><td>0.00</td><td>0.00</td><td>2.34</td><td>1.63</td><td>0.00</td><td>null</td><td>null</td><td></td><td>null</td><td>null</td><td></td><td>null</td><td>null</td><td></td><td>null</td><td>null</td><td></td><td>-5.19E-01</td><td>5.08E-01</td><td></td><td>-1.12E+01</td><td>1.78E-05</td><td>Down</td><td>Unigene117208_All//sp|Q9ERH8|S28A3_MOUSE//8.00E-21//Solute carrier family 28 member 3 OS=Mus musculus GN=Slc28a3 PE=2 SV=1</td><td></td><td></td><td></td><td></td><td></td><td>swissprot +</td></tr>
<tr><td>Unigene117209_All</td><td>308</td><td>0.00</td><td>0.00</td><td>0.00</td><td>0.00</td><td>0.00</td><td>0.00</td><td>0.14</td><td>2.58</td><td>0.65</td><td>null</td><td>null</td><td></td><td>null</td><td>null</td><td></td><td>null</td><td>null</td><td></td><td>null</td><td>null</td><td></td><td>4.23E+00</td><td>1.05E-04</td><td>Up</td><td>2.24E+00</td><td>2.49E-01</td><td></td><td>Unigene117209_All//gi|255603215|ref|XP_002538008.1|//3.00E-06//conserved hypothetical protein [Ricinus communis] >gi|223514214|gb|EEF24374.1| conserved hypothetical protein [Ricinus communis]</td><td></td><td></td><td></td><td></td><td></td><td>nr +</td></tr>
<tr><td>Unigene11721_All</td><td>743</td><td>2.46</td><td>11.66</td><td>4.37</td><td>4.96</td><td>4.96</td><td>4.26</td><td>2.90</td><td>2.81</td><td>1.45</td><td>2.24E+00</td><td>7.62E-21</td><td>Up</td><td>8.27E-01</td><td>2.17E-02</td><td></td><td>-6.00E-04</td><td>9.87E-01</td><td></td><td>-2.22E-01</td><td>5.48E-01</td><td></td><td>-4.52E-02</td><td>9.27E-01</td><td></td><td>-1.00E+00</td><td>7.92E-03</td><td></td><td>Unigene11721_All//gi|20141056|sp|Q9M2D2.2|YU88_ARATH//2.00E-74//RecName: Full=UPF0187 protein At3g61320, chloroplastic; Flags: Precursor >gi|16604549|gb|AAL24280.1| AT3g61320/T20K12_220 [Arabidopsis thaliana] >gi|23296291|gb|AAN12915.1| At3g61320/T20K12_220 [Arabidopsis thaliana]</td><td>Unigene11721_All//1.00E-75//AT3G61320| unknown protein</td><td>Unigene11721_All//0.00E+00//DW508764</td><td></td><td></td><td></td><td>nr -</td></tr>
<tr><td>Unigene117210_All</td><td>308</td><td>0.00</td><td>0.00</td><td>0.00</td><td>0.00</td><td>0.00</td><td>0.00</td><td>0.00</td><td>2.31</td><td>0.26</td><td>null</td><td>null</td><td></td><td>null</td><td>null</td><td></td><td>null</td><td>null</td><td></td><td>null</td><td>null</td><td></td><td>1.12E+01</td><td>3.98E-05</td><td>Up</td><td>8.02E+00</td><td>4.05E-01</td><td></td><td></td><td></td><td></td><td></td><td></td><td></td><td></td></tr>
<tr><td>Unigene117213_All</td><td>309</td><td>0.00</td><td>0.98</td><td>3.45</td><td>1.45</td><td>0.22</td><td>0.88</td><td>1.23</td><td>1.89</td><td>0.52</td><td>9.94E+00</td><td>4.44E-02</td><td></td><td>1.18E+01</td><td>6.63E-06</td><td>Up</td><td>-2.70E+00</td><td>3.24E-02</td><td></td><td>-7.26E-01</td><td>5.32E-01</td><td></td><td>6.21E-01</td><td>4.78E-01</td><td></td><td>-1.26E+00</td><td>2.48E-01</td><td></td><td></td><td></td><td></td><td></td><td></td><td></td><td></td></tr>
<tr><td>Unigene117216_All</td><td>310</td><td>0.00</td><td>1.96</td><td>0.00</td><td>0.96</td><td>0.67</td><td>0.44</td><td>0.00</td><td>2.02</td><td>0.26</td><td>1.09E+01</td><td>1.17E-03</td><td></td><td>null</td><td>null</td><td></td><td>-5.33E-01</td><td>6.79E-01</td><td></td><td>-1.14E+00</td><td>4.58E-01</td><td></td><td>1.10E+01</td><td>1.47E-04</td><td>Up</td><td>8.01E+00</td><td>4.05E-01</td><td></td><td></td><td></td><td>Unigene117216_All//4.00E-07//ES806034</td><td></td><td></td><td></td><td></td></tr>
<tr><td>Unigene117218_All</td><td>310</td><td>0.00</td><td>0.17</td><td>0.00</td><td>0.32</td><td>0.00</td><td>0.00</td><td>2.32</td><td>27.91</td><td>1.29</td><td>-7.40E+00</td><td>6.16E-01</td><td></td><td>-7.40E+00</td><td>6.28E-01</td><td></td><td>-8.33E+00</td><td>3.15E-01</td><td></td><td>-8.33E+00</td><td>4.02E-01</td><td></td><td>3.59E+00</td><td>3.32E-41</td><td>Up</td><td>-8.51E-01</td><td>2.39E-01</td><td></td><td></td><td></td><td>Unigene117218_All//4.00E-07//DW507929</td><td></td><td></td><td></td><td></td></tr>
<tr><td>Unigene117234_All</td><td>313</td><td>0.00</td><td>0.00</td><td>0.00</td><td>0.00</td><td>0.00</td><td>0.00</td><td>0.41</td><td>3.47</td><td>0.00</td><td>null</td><td>null</td><td></td><td>null</td><td>null</td><td></td><td>null</td><td>null</td><td></td><td>null</td><td>null</td><td></td><td>3.10E+00</td><td>4.52E-05</td><td>Up</td><td>-8.66E+00</td><td>2.00E-01</td><td></td><td>Unigene117234_All//sp|Q54LY1|Y6311_DICDI//9.00E-06//G8 domain-containing protein DDB_G0286311 OS=Dictyostelium discoideum GN=DDB_G0286311 PE=3 SV=1</td><td></td><td></td><td></td><td></td><td></td><td>swissprot +</td></tr>
<tr><td>Unigene117236_All</td><td>313</td><td>0.00</td><td>0.00</td><td>0.68</td><td>1.27</td><td>0.00</td><td>0.00</td><td>0.00</td><td>4.94</td><td>1.02</td><td>null</td><td>null</td><td></td><td>9.41E+00</td><td>1.41E-01</td><td></td><td>-1.03E+01</td><td>4.39E-03</td><td></td><td>-1.03E+01</td><td>1.11E-02</td><td></td><td>1.23E+01</td><td>6.59E-11</td><td>Up</td><td>9.99E+00</td><td>1.34E-02</td><td></td><td></td><td></td><td></td><td></td><td></td><td></td><td></td></tr>
<tr><td>Unigene117237_All</td><td>314</td><td>0.50</td><td>0.00</td><td>0.00</td><td>0.00</td><td>0.00</td><td>0.00</td><td>0.54</td><td>3.86</td><td>0.76</td><td>-8.97E+00</td><td>2.16E-01</td><td></td><td>-8.97E+00</td><td>2.46E-01</td><td></td><td>null</td><td>null</td><td></td><td>null</td><td>null</td><td></td><td>2.84E+00</td><td>3.39E-05</td><td>Up</td><td>4.99E-01</td><td>7.08E-01</td><td></td><td></td><td></td><td>Unigene117237_All//4.00E-07//TC172964</td><td></td><td></td><td></td><td></td></tr>
<tr><td>Unigene117238_All</td><td>314</td><td>0.00</td><td>0.00</td><td>0.00</td><td>0.00</td><td>0.00</td><td>0.00</td><td>2.02</td><td>6.92</td><td>1.14</td><td>null</td><td>null</td><td></td><td>null</td><td>null</td><td></td><td>null</td><td>null</td><td></td><td>null</td><td>null</td><td></td><td>1.78E+00</td><td>2.47E-05</td><td>Up</td><td>-8.23E-01</td><td>2.93E-01</td><td></td><td></td><td></td><td>Unigene117238_All//4.00E-07//DT046802</td><td></td><td></td><td></td><td></td></tr>
<tr><td>Unigene11724_All</td><td>1171</td><td>13.99</td><td>2.77</td><td>3.09</td><td>23.06</td><td>18.76</td><td>29.00</td><td>26.14</td><td>7.32</td><td>12.35</td><td>-2.34E+00</td><td>4.00E-41</td><td>Down</td><td>-2.18E+00</td><td>2.80E-36</td><td>Down</td><td>-2.97E-01</td><td>3.10E-03</td><td></td><td>3.31E-01</td><td>2.86E-04</td><td></td><td>-1.84E+00</td><td>1.55E-68</td><td>Down</td><td>-1.08E+00</td><td>3.31E-32</td><td>Down</td><td>Unigene11724_All//gi|15229546|ref|NP_189036.1|//1.00E-159//dehydratase family [Arabidopsis thaliana] >gi|17933301|gb|AAL48233.1|AF446360_1 AT3g23940/F14013_13 [Arabidopsis thaliana] >gi|9294662|dbj|BAB03011.1| dihydroxy-acid dehydratase [Arabidopsis thaliana]</td><td>Unigene11724_All//1.00E-157//AT3G23940| dehydratase family</td><td>Unigene11724_All//0.00E+00//TC143517</td><td></td><td>ko00290|Valine, leucine and isoleucine biosynthesis//ko00770|Pantothenate and CoA biosynthesis</td><td></td><td>nr -</td></tr>
<tr><td>Unigene117245_All</td><td>317</td><td>0.00</td><td>0.00</td><td>0.00</td><td>0.00</td><td>0.00</td><td>0.00</td><td>0.00</td><td>4.75</td><td>0.13</td><td>null</td><td>null</td><td></td><td>null</td><td>null</td><td></td><td>null</td><td>null</td><td></td><td>null</td><td>null</td><td></td><td>1.22E+01</td><td>1.29E-10</td><td>Up</td><td>6.97E+00</td><td>6.46E-01</td><td></td><td></td><td></td><td>Unigene117245_All//6.00E-12//ES807146</td><td></td><td></td><td></td><td></td></tr>
<tr><td>Unigene117246_All</td><td>318</td><td>0.00</td><td>0.00</td><td>0.00</td><td>0.00</td><td>0.00</td><td>0.00</td><td>0.00</td><td>8.81</td><td>0.00</td><td>null</td><td>null</td><td></td><td>null</td><td>null</td><td></td><td>null</td><td>null</td><td></td><td>null</td><td>null</td><td></td><td>1.31E+01</td><td>1.07E-19</td><td>Up</td><td>null</td><td>null</td><td></td><td></td><td></td><td></td><td></td><td></td><td></td><td></td></tr>
<tr><td>Unigene11725_All</td><td>2528</td><td>19.52</td><td>7.10</td><td>7.77</td><td>26.60</td><td>29.82</td><td>42.68</td><td>35.57</td><td>23.16</td><td>41.48</td><td>-1.46E+00</td><td>6.39E-65</td><td>Down</td><td>-1.33E+00</td><td>2.30E-54</td><td>Down</td><td>1.65E-01</td><td>6.14E-03</td><td></td><td>6.82E-01</td><td>1.15E-43</td><td></td><td>-6.19E-01</td><td>3.24E-35</td><td></td><td>2.22E-01</td><td>6.01E-07</td><td></td><td>Unigene11725_All//gi|255671803|gb|ACU26534.1|//0.00E+00//dihydroxyacid dehydratase [Glycine max]</td><td>Unigene11725_All//0.00E+00//AT3G23940| dehydratase family</td><td>Unigene11725_All//0.00E+00//TC136783</td><td></td><td>ko00290|Valine, leucine and isoleucine biosynthesis//ko00770|Pantothenate and CoA biosynthesis</td><td>GO:0006549//GO:0006573//GO:0008652//GO:0009532//GO:0016836//GO:0046914//GO:0048229</td><td>nr -</td></tr>
<tr><td>Unigene117251_All</td><td>320</td><td>0.00</td><td>0.00</td><td>0.00</td><td>0.00</td><td>0.00</td><td>0.00</td><td>2.64</td><td>3.53</td><td>0.00</td><td>null</td><td>null</td><td></td><td>null</td><td>null</td><td></td><td>null</td><td>null</td><td></td><td>null</td><td>null</td><td></td><td>4.16E-01</td><td>4.92E-01</td><td></td><td>-1.14E+01</td><td>2.21E-06</td><td>Down</td><td>Unigene117251_All//gi|145343549|ref|XP_001416382.1|//6.00E-38//ribosomal protein L19 [Ostreococcus lucimarinus CCE9901] >gi|144576607|gb|ABO94675.1| ribosomal protein L19 [Ostreococcus lucimarinus CCE9901]</td><td>Unigene117251_All//2.00E-37//AT4G02230| 60S ribosomal protein L19 (RPL19C)</td><td></td><td></td><td>ko03010|Ribosome</td><td></td><td>nr +</td></tr>
<tr><td>Unigene117254_All</td><td>322</td><td>0.00</td><td>0.00</td><td>0.00</td><td>0.00</td><td>0.00</td><td>0.00</td><td>0.26</td><td>3.12</td><td>0.12</td><td>null</td><td>null</td><td></td><td>null</td><td>null</td><td></td><td>null</td><td>null</td><td></td><td>null</td><td>null</td><td></td><td>3.57E+00</td><td>3.08E-05</td><td>Up</td><td>-1.09E+00</td><td>6.77E-01</td><td></td><td></td><td></td><td></td><td></td><td></td><td></td><td></td></tr>
<tr><td>Unigene117255_All</td><td>322</td><td>0.00</td><td>0.00</td><td>0.00</td><td>0.00</td><td>0.00</td><td>0.00</td><td>0.00</td><td>2.73</td><td>0.00</td><td>null</td><td>null</td><td></td><td>null</td><td>null</td><td></td><td>null</td><td>null</td><td></td><td>null</td><td>null</td><td></td><td>1.14E+01</td><td>2.86E-06</td><td>Up</td><td>null</td><td>null</td><td></td><td>Unigene117255_All//gi|32400971|gb|AAP80691.1|//3.00E-46//ubiquitin-conjugating enzyme [Griffithsia japonica]</td><td>Unigene117255_All//8.00E-46//AT3G08690| UBC11 (UBIQUITIN-CONJUGATING ENZYME 11); ubiquitin-protein ligase</td><td>Unigene117255_All//1.00E-10//EX170326</td><td></td><td>ko04141|Protein processing in endoplasmic reticulum//ko04120|Ubiquitin mediated proteolysis</td><td></td><td>nr +</td></tr>
<tr><td>Unigene117256_All</td><td>322</td><td>0.00</td><td>0.00</td><td>0.00</td><td>0.00</td><td>0.00</td><td>0.00</td><td>0.39</td><td>3.76</td><td>0.00</td><td>null</td><td>null</td><td></td><td>null</td><td>null</td><td></td><td>null</td><td>null</td><td></td><td>null</td><td>null</td><td></td><td>3.26E+00</td><td>8.29E-06</td><td>Up</td><td>-8.62E+00</td><td>2.00E-01</td><td></td><td></td><td></td><td></td><td></td><td></td><td></td><td>ESTscan +</td></tr>
<tr><td>Unigene117259_All</td><td>323</td><td>0.00</td><td>0.00</td><td>0.00</td><td>0.00</td><td>0.00</td><td>0.00</td><td>0.00</td><td>2.07</td><td>0.00</td><td>null</td><td>null</td><td></td><td>null</td><td>null</td><td></td><td>null</td><td>null</td><td></td><td>null</td><td>null</td><td></td><td>1.10E+01</td><td>7.64E-05</td><td>Up</td><td>null</td><td>null</td><td></td><td></td><td></td><td></td><td></td><td></td><td></td><td>ESTscan +</td></tr>
<tr><td>Unigene11726_All</td><td>1506</td><td>14.87</td><td>5.79</td><td>7.85</td><td>9.20</td><td>6.63</td><td>4.89</td><td>4.46</td><td>1.42</td><td>1.35</td><td>-1.36E+00</td><td>6.53E-27</td><td>Down</td><td>-9.22E-01</td><td>4.32E-14</td><td></td><td>-4.72E-01</td><td>8.57E-04</td><td></td><td>-9.12E-01</td><td>8.65E-10</td><td></td><td>-1.66E+00</td><td>1.20E-13</td><td>Down</td><td>-1.73E+00</td><td>6.72E-15</td><td>Down</td><td>Unigene11726_All//gi|82569446|gb|ABB83365.1|//1.00E-174//enoyl-ACP reductase [Malus x domestica]</td><td>Unigene11726_All//2.00E-154//AT2G05990| MOD1 (MOSAIC DEATH 1); enoyl-[acyl-carrier-protein] reductase (NADH)/ enoyl-[acyl-carrier-protein] reductase/ oxidoreductase</td><td>Unigene11726_All//0.00E+00//gi|109852131|gb|DW481110.1|DW481110</td><td></td><td>ko00061|Fatty acid biosynthesis</td><td>GO:0004312//GO:0006631</td><td>nr -</td></tr>
<tr><td>Unigene117262_All</td><td>323</td><td>4.70</td><td>5.65</td><td>6.59</td><td>6.48</td><td>2.35</td><td>3.22</td><td>2.23</td><td>5.82</td><td>1.48</td><td>2.66E-01</td><td>6.07E-01</td><td></td><td>4.89E-01</td><td>2.95E-01</td><td></td><td>-1.47E+00</td><td>6.01E-04</td><td>Down</td><td>-1.01E+00</td><td>2.37E-02</td><td></td><td>1.39E+00</td><td>1.52E-03</td><td></td><td>-5.88E-01</td><td>4.09E-01</td><td></td><td></td><td></td><td>Unigene117262_All//4.00E-07//TC143316</td><td></td><td></td><td></td><td></td></tr>
<tr><td>Unigene117263_All</td><td>324</td><td>0.00</td><td>0.00</td><td>0.00</td><td>0.00</td><td>0.00</td><td>0.00</td><td>0.65</td><td>3.23</td><td>0.00</td><td>null</td><td>null</td><td></td><td>null</td><td>null</td><td></td><td>null</td><td>null</td><td></td><td>null</td><td>null</td><td></td><td>2.31E+00</td><td>8.56E-04</td><td>Up</td><td>-9.35E+00</td><td>5.80E-02</td><td></td><td></td><td></td><td></td><td></td><td></td><td></td><td>ESTscan +</td></tr>
<tr><td>Unigene117266_All</td><td>325</td><td>0.00</td><td>0.00</td><td>0.00</td><td>0.00</td><td>0.00</td><td>0.00</td><td>0.00</td><td>1.93</td><td>0.00</td><td>null</td><td>null</td><td></td><td>null</td><td>null</td><td></td><td>null</td><td>null</td><td></td><td>null</td><td>null</td><td></td><td>1.09E+01</td><td>1.47E-04</td><td>Up</td><td>null</td><td>null</td><td></td><td>Unigene117266_All//COG0778//2.00E-06//Nitroreductase</td><td></td><td></td><td></td><td></td><td></td><td>cog +</td></tr>
<tr><td>Unigene11727_All</td><td>1767</td><td>19.31</td><td>8.17</td><td>6.12</td><td>19.11</td><td>15.90</td><td>15.21</td><td>21.63</td><td>5.65</td><td>6.45</td><td>-1.24E+00</td><td>2.96E-35</td><td>Down</td><td>-1.66E+00</td><td>2.92E-52</td><td>Down</td><td>-2.65E-01</td><td>2.99E-03</td><td></td><td>-3.30E-01</td><td>3.03E-04</td><td></td><td>-1.94E+00</td><td>1.22E-91</td><td>Down</td><td>-1.75E+00</td><td>4.88E-82</td><td>Down</td><td>Unigene11727_All//gi|82569446|gb|ABB83365.1|//0.00E+00//enoyl-ACP reductase [Malus x domestica]</td><td>Unigene11727_All//6.00E-149//AT2G05990| MOD1 (MOSAIC DEATH 1); enoyl-[acyl-carrier-protein] reductase (NADH)/ enoyl-[acyl-carrier-protein] reductase/ oxidoreductase</td><td>Unigene11727_All//0.00E+00//TC155088</td><td></td><td>ko00061|Fatty acid biosynthesis</td><td>GO:0004312//GO:0006631</td><td>nr +</td></tr>
<tr><td>Unigene117279_All</td><td>329</td><td>0.00</td><td>0.00</td><td>0.00</td><td>0.00</td><td>0.21</td><td>0.55</td><td>0.00</td><td>1.65</td><td>1.09</td><td>null</td><td>null</td><td></td><td>null</td><td>null</td><td></td><td>7.71E+00</td><td>6.12E-01</td><td></td><td>9.10E+00</td><td>1.95E-01</td><td></td><td>1.07E+01</td><td>5.36E-04</td><td>Up</td><td>1.01E+01</td><td>7.30E-03</td><td></td><td>Unigene117279_All//gi|18405611|ref|NP_566829.1|//1.00E-28//nodulin MtN3 family protein [Arabidopsis thaliana] >gi|25090096|gb|AAN72227.1| At3g28008/At3g28008 [Arabidopsis thaliana]</td><td>Unigene117279_All//6.00E-30//AT3G28007| nodulin MtN3 family protein</td><td></td><td></td><td></td><td></td><td>nr -</td></tr>
<tr><td>Unigene11728_All</td><td>724</td><td>1.37</td><td>0.00</td><td>1.54</td><td>2.13</td><td>2.09</td><td>0.50</td><td>1.40</td><td>0.35</td><td>0.88</td><td>-1.04E+01</td><td>7.46E-06</td><td>Down</td><td>1.70E-01</td><td>7.78E-01</td><td></td><td>-2.74E-02</td><td>9.58E-01</td><td></td><td>-2.10E+00</td><td>3.41E-04</td><td>Down</td><td>-2.02E+00</td><td>2.83E-03</td><td></td><td>-6.71E-01</td><td>2.53E-01</td><td></td><td>Unigene11728_All//gi|15217667|ref|NP_174087.1|//6.00E-45//ethylene-responsive protein -related [Arabidopsis thaliana] >gi|218563530|sp|Q9SFZ3.2|BH110_ARATH RecName: Full=Transcription factor bHLH110; AltName: Full=Transcription factor EN 59; AltName: Full=bHLH transcription factor bHLH110; AltName: Full=Basic helix-loop-helix protein 110; Short=bHLH 110; Short=AtbHLH110</td><td>Unigene11728_All//6.00E-37//AT1G27660| ethylene-responsive protein -related</td><td>Unigene11728_All//3.00E-93//CO085880</td><td>bHLH</td><td></td><td>GO:0010468//GO:0043231</td><td>nr -</td></tr>
<tr><td>Unigene117286_All</td><td>331</td><td>0.00</td><td>0.00</td><td>0.00</td><td>0.00</td><td>0.00</td><td>0.00</td><td>0.00</td><td>2.90</td><td>0.00</td><td>null</td><td>null</td><td></td><td>null</td><td>null</td><td></td><td>null</td><td>null</td><td></td><td>null</td><td>null</td><td></td><td>1.15E+01</td><td>7.61E-07</td><td>Up</td><td>null</td><td>null</td><td></td><td>Unigene117286_All//sp|Q8KU07|AZOB_XENAZ//3.00E-06//NAD(P)H azoreductase OS=Xenophilus azovorans GN=azoB PE=1 SV=2</td><td></td><td></td><td></td><td></td><td></td><td>swissprot +</td></tr>
</table>

<table>
<tr><td>Unigene117289_All</td><td>332</td><td>0.00</td><td>0.00</td><td>0.00</td><td>0.00</td><td>0.00</td><td>0.00</td><td>1.91</td><td>3.15</td><td>0.00</td><td>null</td><td>null</td><td></td><td>null</td><td>null</td><td></td><td>null</td><td>null</td><td></td><td>null</td><td>null</td><td></td><td>7.20E-01</td><td>2.38E-01</td><td></td><td>-1.09E+01</td><td>7.11E-05</td><td>Down</td><td>Unigene117289_All//gi|13661265|gb|AAK37834.1|AF182558_1//2.00E-58//beta-tubulin [Euglena gracilis] >gi|13661269|gb|AAK37836.1|AF182554_1 beta-tubulin [Euglena gracilis] >gi|13661271|gb|AAK37837.1|AF182555_1 beta-tubulin [Euglena gracilis] >gi|13661273|gb|AAK37838.1|AF182759_1 beta-tubulin [Euglena gracilis]</td><td>Unigene117289_All//4.00E-57//AT1G20010| TUB5; structural constituent of cytoskeleton</td><td>Unigene117289_All//1.00E-22//TC129598</td><td></td><td>ko04145|Phagosome</td><td></td><td>nr -</td></tr>
<tr><td>Unigene11729_All</td><td>323</td><td>0.00</td><td>0.78</td><td>0.49</td><td>0.77</td><td>0.43</td><td>0.00</td><td>3.93</td><td>0.39</td><td>0.37</td><td>9.62E+00</td><td>7.87E-02</td><td></td><td>8.95E+00</td><td>2.39E-01</td><td></td><td>-8.55E-01</td><td>5.74E-01</td><td></td><td>-9.59E+00</td><td>7.35E-02</td><td></td><td>-3.34E+00</td><td>3.42E-06</td><td>Down</td><td>-3.41E+00</td><td>1.45E-06</td><td>Down</td><td>Unigene11729_All//gi|15217667|ref|NP_174087.1|//2.00E-21//ethylene-responsive protein -related [Arabidopsis thaliana] >gi|218563530|sp|Q9SFZ3.2|BH110_ARATH RecName: Full=Transcription factor bHLH110; AltName: Full=Transcription factor EN 59; AltName: Full=bHLH transcription factor bHLH110; AltName: Full=Basic helix-loop-helix protein 110; Short=bHLH 110; Short=AtbHLH110</td><td>Unigene11729_All//9.00E-23//AT1G27660| ethylene-responsive protein -related</td><td>Unigene11729_All//6.00E-83//DW507031</td><td>bHLH</td><td></td><td>GO:0001071//GO:0010468//GO:0043231</td><td>nr +</td></tr>
<tr><td>Unigene117292_All</td><td>333</td><td>0.00</td><td>0.00</td><td>0.64</td><td>0.00</td><td>0.00</td><td>0.00</td><td>0.13</td><td>2.26</td><td>0.24</td><td>null</td><td>null</td><td></td><td>9.32E+00</td><td>1.41E-01</td><td></td><td>null</td><td>null</td><td></td><td>null</td><td>null</td><td></td><td>4.15E+00</td><td>1.92E-04</td><td>Up</td><td>9.14E-01</td><td>7.50E-01</td><td></td><td>Unigene117292_All//gi|294514718|gb|ADE95828.1|//2.00E-31//4-coumarate:CoA ligase 1 [Corchorus capsularis]</td><td>Unigene117292_All//7.00E-30//AT1G51680| 4CL1 (4-COUMARATE:COA LIGASE 1); 4-coumarate-CoA ligase</td><td>Unigene117292_All//2.00E-95//gi|82756624|gb|DV850186.1|DV850186</td><td></td><td>ko00940|Phenylpropanoid biosynthesis//ko00360|Phenylalanine metabolism//ko00130|Ubiquinone and other terpenoid-quinone biosynthesis</td><td></td><td>nr -</td></tr>
<tr><td>Unigene1173_All</td><td>3567</td><td>3.55</td><td>16.05</td><td>3.58</td><td>8.60</td><td>8.83</td><td>9.08</td><td>10.69</td><td>8.25</td><td>11.07</td><td>2.18E+00</td><td>1.34E-129</td><td>Up</td><td>1.34E-02</td><td>9.57E-01</td><td></td><td>3.82E-02</td><td>7.84E-01</td><td></td><td>7.76E-02</td><td>5.11E-01</td><td></td><td>-3.74E-01</td><td>1.24E-06</td><td></td><td>4.99E-02</td><td>5.99E-01</td><td></td><td>Unigene1173_All//gi|115478695|ref|NP_001062941.1|//0.00E+00//Os09g0346700 [Oryza sativa Japonica Group] >gi|113631174|dbj|BAF24855.1| Os09g0346700 [Oryza sativa Japonica Group]</td><td>Unigene1173_All//0.00E+00//AT2G25800| INVOLVED IN: biological_process unknown; EXPRESSED IN: 23 plant structures; EXPRESSED DURING: 13 growth stages; CONTAINS InterPro DOMAIN/s: Munc13 homology 1 (InterPro:IPR014770), Protein of unknown function DUF810 (InterPro:IPR008528), Munc13 homology 2 (InterPro:IPR014772); BEST Arabidopsis thaliana protein match is: unknown protein (TAIR:AT2G20010.2); Has 242 Blast hits to 156 proteins in 35 species: Archae - 0; Bacteria - 8; Metazoa - 36; Fungi - 18; Plants - 94; Viruses - 0; Other Eukaryotes - 86 (source: NCBI BLink).</td><td>Unigene1173_All//0.00E+00//TC162496</td><td></td><td></td><td></td><td>nr -</td></tr>
<tr><td>Unigene11730_All</td><td>2100</td><td>1.59</td><td>3.31</td><td>2.76</td><td>15.56</td><td>13.61</td><td>7.61</td><td>7.79</td><td>4.94</td><td>5.86</td><td>1.05E+00</td><td>3.80E-06</td><td>Up</td><td>7.94E-01</td><td>1.96E-03</td><td></td><td>-1.93E-01</td><td>4.54E-02</td><td></td><td>-1.03E+00</td><td>2.99E-27</td><td>Down</td><td>-6.59E-01</td><td>8.18E-08</td><td></td><td>-4.11E-01</td><td>6.10E-04</td><td></td><td>Unigene11730_All//gi|15217667|ref|NP_174087.1|//3.00E-46//ethylene-responsive protein -related [Arabidopsis thaliana] >gi|218563530|sp|Q9SFZ3.2|BH110_ARATH RecName: Full=Transcription factor bHLH110; AltName: Full=Transcription factor EN 59; AltName: Full=bHLH transcription factor bHLH110; AltName: Full=Basic helix-loop-helix protein 110; Short=bHLH 110; Short=AtbHLH110</td><td>Unigene11730_All//1.00E-35//AT1G27660| ethylene-responsive protein -related</td><td>Unigene11730_All//0.00E+00//TC162179</td><td>bHLH</td><td></td><td></td><td>nr -</td></tr>
<tr><td>Unigene117301_All</td><td>339</td><td>0.00</td><td>0.00</td><td>0.00</td><td>0.00</td><td>0.00</td><td>0.00</td><td>1.00</td><td>5.79</td><td>1.53</td><td>null</td><td>null</td><td></td><td>null</td><td>null</td><td></td><td>null</td><td>null</td><td></td><td>null</td><td>null</td><td></td><td>2.54E+00</td><td>3.39E-07</td><td>Up</td><td>6.15E-01</td><td>4.94E-01</td><td></td><td></td><td></td><td>Unigene117301_All//6.00E-145//CO125364</td><td></td><td></td><td></td><td></td></tr>
<tr><td>Unigene117305_All</td><td>341</td><td>0.00</td><td>0.00</td><td>0.00</td><td>0.15</td><td>0.10</td><td>0.00</td><td>0.62</td><td>3.80</td><td>1.99</td><td>null</td><td>null</td><td></td><td>null</td><td>null</td><td></td><td>-5.33E-01</td><td>8.27E-01</td><td></td><td>-7.19E+00</td><td>6.40E-01</td><td></td><td>2.62E+00</td><td>4.08E-05</td><td>Up</td><td>1.68E+00</td><td>3.70E-02</td><td></td><td></td><td></td><td></td><td></td><td></td><td></td><td></td></tr>
<tr><td>Unigene117308_All</td><td>343</td><td>0.00</td><td>0.00</td><td>0.00</td><td>0.00</td><td>0.00</td><td>0.00</td><td>0.74</td><td>4.02</td><td>0.00</td><td>null</td><td>null</td><td></td><td>null</td><td>null</td><td></td><td>null</td><td>null</td><td></td><td>null</td><td>null</td><td></td><td>2.44E+00</td><td>4.59E-05</td><td>Up</td><td>-9.53E+00</td><td>3.04E-02</td><td></td><td>Unigene117308_All//sp|Q02817|MUC2_HUMAN//7.00E-06//Mucin-2 OS=Homo sapiens GN=MUC2 PE=1 SV=2</td><td></td><td></td><td></td><td></td><td></td><td>swissprot +</td></tr>
<tr><td>Unigene11731_All</td><td>1226</td><td>2.60</td><td>0.41</td><td>4.21</td><td>4.23</td><td>4.07</td><td>2.14</td><td>1.79</td><td>0.17</td><td>1.23</td><td>-2.66E+00</td><td>8.99E-10</td><td>Down</td><td>6.95E-01</td><td>1.14E-02</td><td></td><td>-5.32E-02</td><td>8.47E-01</td><td></td><td>-9.84E-01</td><td>1.49E-04</td><td></td><td>-3.39E+00</td><td>1.88E-10</td><td>Down</td><td>-5.38E-01</td><td>1.51E-01</td><td></td><td>Unigene11731_All//gi|15217667|ref|NP_174087.1|//4.00E-56//ethylene-responsive protein -related [Arabidopsis thaliana] >gi|218563530|sp|Q9SFZ3.2|BH110_ARATH RecName: Full=Transcription factor bHLH110; AltName: Full=Transcription factor EN 59; AltName: Full=bHLH transcription factor bHLH110; AltName: Full=Basic helix-loop-helix protein 110; Short=bHLH 110; Short=AtbHLH110</td><td>Unigene11731_All//4.00E-48//AT1G27660| ethylene-responsive protein -related</td><td>Unigene11731_All//0.00E+00//gi|78352935|gb|DT573209.1|DT573209</td><td>bHLH</td><td></td><td>GO:0010468//GO:0043231</td><td>nr +</td></tr>
<tr><td>Unigene117312_All</td><td>346</td><td>0.00</td><td>0.00</td><td>0.00</td><td>0.00</td><td>0.00</td><td>0.26</td><td>0.00</td><td>9.30</td><td>1.38</td><td>null</td><td>null</td><td></td><td>null</td><td>null</td><td></td><td>null</td><td>null</td><td></td><td>8.03E+00</td><td>4.79E-01</td><td></td><td>1.32E+01</td><td>1.23E-22</td><td>Up</td><td>1.04E+01</td><td>1.15E-03</td><td></td><td></td><td></td><td>Unigene117312_All//4.00E-38//TC137853</td><td></td><td></td><td></td><td></td></tr>
<tr><td>Unigene117316_All</td><td>349</td><td>0.00</td><td>0.00</td><td>0.00</td><td>0.00</td><td>0.00</td><td>0.00</td><td>0.00</td><td>3.71</td><td>0.00</td><td>null</td><td>null</td><td></td><td>null</td><td>null</td><td></td><td>null</td><td>null</td><td></td><td>null</td><td>null</td><td></td><td>1.19E+01</td><td>3.67E-09</td><td>Up</td><td>null</td><td>null</td><td></td><td>Unigene117316_All//sp|O94564|YGD6_SCHPO//2.00E-06//Zinc-type alcohol dehydrogenase-like protein C1773.06c OS=Schizosaccharomyces pombe GN=SPBC1773.06c PE=2 SV=1</td><td></td><td></td><td></td><td></td><td></td><td>swissprot -</td></tr>
<tr><td>Unigene117318_All</td><td>349</td><td>0.00</td><td>0.00</td><td>0.00</td><td>0.00</td><td>0.00</td><td>0.00</td><td>2.06</td><td>2.40</td><td>0.00</td><td>null</td><td>null</td><td></td><td>null</td><td>null</td><td></td><td>null</td><td>null</td><td></td><td>null</td><td>null</td><td></td><td>2.18E-01</td><td>7.44E-01</td><td></td><td>-1.10E+01</td><td>1.78E-05</td><td>Down</td><td></td><td></td><td></td><td></td><td></td><td></td><td>ESTscan +</td></tr>
<tr><td>Unigene117326_All</td><td>354</td><td>0.00</td><td>0.00</td><td>0.00</td><td>0.00</td><td>0.00</td><td>0.00</td><td>2.39</td><td>5.90</td><td>0.00</td><td>null</td><td>null</td><td></td><td>null</td><td>null</td><td></td><td>null</td><td>null</td><td></td><td>null</td><td>null</td><td></td><td>1.31E+00</td><td>1.39E-03</td><td></td><td>-1.12E+01</td><td>2.21E-06</td><td>Down</td><td>Unigene117326_All//gi|161701447|gb|ABX75541.1|//3.00E-49//60S ribosomal protein L17-like protein [Panax ginseng]</td><td>Unigene117326_All//4.00E-48//AT3G04400| emb2171 (embryo defective 2171); structural constituent of ribosome</td><td>Unigene117326_All//1.00E-10//TC153743</td><td></td><td>ko03010|Ribosome</td><td></td><td>nr +</td></tr>
<tr><td>Unigene117329_All</td><td>355</td><td>0.00</td><td>0.00</td><td>0.00</td><td>0.00</td><td>0.00</td><td>0.00</td><td>2.50</td><td>5.77</td><td>0.00</td><td>null</td><td>null</td><td></td><td>null</td><td>null</td><td></td><td>null</td><td>null</td><td></td><td>null</td><td>null</td><td></td><td>1.21E+00</td><td>3.25E-03</td><td></td><td>-1.13E+01</td><td>1.10E-06</td><td>Down</td><td>Unigene117329_All//gi|14423905|sp|Q9XGL4.1|RL31_CYAPA//7.00E-31//RecName: Full=60S ribosomal protein L31 >gi|5123727|emb|CAB45375.1| ribosomal protein L31 [Cyanophora paradoxa]</td><td>Unigene117329_All//1.00E-25//AT4G26230| 60S ribosomal protein L31 (RPL31B)</td><td></td><td></td><td>ko03010|Ribosome</td><td></td><td>nr +</td></tr>
<tr><td>Unigene11733_All</td><td>1591</td><td>2.66</td><td>3.09</td><td>5.72</td><td>3.79</td><td>4.11</td><td>4.43</td><td>0.58</td><td>0.53</td><td>0.30</td><td>2.14E-01</td><td>4.53E-01</td><td></td><td>1.10E+00</td><td>5.14E-08</td><td>Up</td><td>1.18E-01</td><td>6.68E-01</td><td></td><td>2.25E-01</td><td>3.81E-01</td><td></td><td>-1.54E-01</td><td>8.08E-01</td><td></td><td>-9.60E-01</td><td>1.20E-01</td><td></td><td>Unigene11733_All//gi|7767672|gb|AAF69169.1|AC007915_21//4.00E-67//F27F5.21 [Arabidopsis thaliana]</td><td>Unigene11733_All//1.00E-35//AT4G29090| reverse transcriptase, putative / RNA-dependent DNA polymerase, putative</td><td>Unigene11733_All//5.00E-07//TC133829</td><td></td><td></td><td>GO:0003676//GO:0016772//GO:0043231//GO:0044238//GO:0044260</td><td>nr -</td></tr>
<tr><td>Unigene117335_All</td><td>362</td><td>0.43</td><td>0.28</td><td>0.59</td><td>0.00</td><td>0.00</td><td>0.25</td><td>0.00</td><td>8.89</td><td>0.00</td><td>-6.32E-01</td><td>7.28E-01</td><td></td><td>4.40E-01</td><td>7.76E-01</td><td></td><td>null</td><td>null</td><td></td><td>7.96E+00</td><td>4.79E-01</td><td></td><td>1.31E+01</td><td>1.22E-22</td><td>Up</td><td>null</td><td>null</td><td></td><td></td><td></td><td>Unigene117335_All//1.00E-50//CO118951</td><td></td><td></td><td></td><td></td></tr>
<tr><td>Unigene11734_All</td><td>1920</td><td>3.27</td><td>4.35</td><td>7.16</td><td>4.64</td><td>5.47</td><td>5.57</td><td>2.49</td><td>1.72</td><td>1.56</td><td>4.13E-01</td><td>4.25E-02</td><td></td><td>1.13E+00</td><td>3.43E-12</td><td>Up</td><td>2.36E-01</td><td>2.23E-01</td><td></td><td>2.64E-01</td><td>1.66E-01</td><td></td><td>-5.33E-01</td><td>3.16E-02</td><td></td><td>-6.77E-01</td><td>4.26E-03</td><td></td><td>Unigene11734_All//gi|38344244|emb|CAD41337.2|//9.00E-26//OJ991113_30.21 [Oryza sativa (japonica cultivar-group)]</td><td>Unigene11734_All//7.00E-27//AT2G18410| unknown protein</td><td>Unigene11734_All//0.00E+00//TC178231</td><td></td><td></td><td>GO:0043231</td><td>nr -</td></tr>
<tr><td>Unigene117344_All</td><td>371</td><td>0.00</td><td>0.00</td><td>0.00</td><td>0.00</td><td>0.00</td><td>0.00</td><td>0.80</td><td>4.06</td><td>0.00</td><td>null</td><td>null</td><td></td><td>null</td><td>null</td><td></td><td>null</td><td>null</td><td></td><td>null</td><td>null</td><td></td><td>2.35E+00</td><td>3.01E-05</td><td>Up</td><td>-9.64E+00</td><td>1.58E-02</td><td></td><td>Unigene117344_All//gi|255078736|ref|XP_002502948.1|//7.00E-39//translation elongation factor EF-1alpha/Tu [Micromonas sp. RCC299] >gi|226518214|gb|AC064206.1| translation elongation factor EF-1alpha/Tu [Micromonas sp. RCC299]</td><td>Unigene117344_All//6.00E-20//AT5G60390| elongation factor 1-alpha / EF-1-alpha</td><td></td><td></td><td></td><td></td><td>nr +</td></tr>
<tr><td>Unigene117345_All</td><td>371</td><td>0.00</td><td>0.00</td><td>0.00</td><td>0.54</td><td>0.00</td><td>0.00</td><td>0.34</td><td>5.07</td><td>0.21</td><td>null</td><td>null</td><td></td><td>null</td><td>null</td><td></td><td>-9.07E+00</td><td>8.45E-02</td><td></td><td>-9.07E+00</td><td>1.34E-01</td><td></td><td>3.89E+00</td><td>6.17E-10</td><td>Up</td><td>-6.71E-01</td><td>7.20E-01</td><td></td><td></td><td></td><td></td><td></td><td></td><td></td><td></td></tr>
<tr><td>Unigene117354_All</td><td>379</td><td>0.28</td><td>0.00</td><td>0.00</td><td>0.39</td><td>0.18</td><td>0.00</td><td>0.11</td><td>3.97</td><td>0.63</td><td>-8.11E+00</td><td>3.74E-01</td><td></td><td>-8.11E+00</td><td>3.96E-01</td><td></td><td>-1.12E+00</td><td>5.84E-01</td><td></td><td>-8.62E+00</td><td>2.34E-01</td><td></td><td>5.15E+00</td><td>2.32E-09</td><td>Up</td><td>2.50E+00</td><td>1.59E-01</td><td></td><td></td><td></td><td></td><td></td><td></td><td></td><td></td></tr>
<tr><td>Unigene117355_All</td><td>379</td><td>0.00</td><td>0.00</td><td>0.00</td><td>0.00</td><td>0.00</td><td>0.00</td><td>2.57</td><td>4.30</td><td>0.00</td><td>null</td><td>null</td><td></td><td>null</td><td>null</td><td></td><td>null</td><td>null</td><td></td><td>null</td><td>null</td><td></td><td>7.45E-01</td><td>1.10E-01</td><td></td><td>-1.13E+01</td><td>2.72E-07</td><td>Down</td><td>Unigene117355_All//gi|116056034|emb|CAL58567.1|//6.00E-11//Nucleolar GTPase/ATPase p130 (ISS) [Ostreococcus tauri]</td><td></td><td></td><td></td><td>ko03018|RNA degradation</td><td></td><td>nr -</td></tr>
<tr><td>Unigene117359_All</td><td>383</td><td>0.68</td><td>0.26</td><td>0.00</td><td>0.13</td><td>0.45</td><td>0.47</td><td>1.21</td><td>16.26</td><td>0.94</td><td>-1.37E+00</td><td>3.83E-01</td><td></td><td>-9.42E+00</td><td>8.59E-02</td><td></td><td>1.79E+00</td><td>5.21E-01</td><td></td><td>1.86E+00</td><td>4.59E-01</td><td></td><td>3.74E+00</td><td>8.12E-31</td><td>Up</td><td>-3.75E-01</td><td>6.65E-01</td><td></td><td>Unigene117359_All//gi|115469074|ref|NP_001058136.1|//8.00E-06//Os06g0634300 [Oryza sativa Japonica Group] >gi|51535777|dbj|BAD37816.1| MutT domain protein-like [Oryza sativa Japonica Group] >gi|51535894|dbj|BAD37977.1| MutT domain protein-like [Oryza sativa Japonica Group] >gi|113596176|dbj|BAF20050.1| Os06g0634300 [Oryza sativa Japonica Group]</td><td>Unigene117359_All//3.00E-06//AT4G25434| ATNUDT10 (Arabidopsis thaliana Nudix hydrolase homolog 10); ADP-ribose diphosphatase/ NAD or NADH binding / catalytic</td><td>Unigene117359_All//9.00E-135//DR458609</td><td></td><td></td><td></td><td>nr -</td></tr>
<tr><td>Unigene117362_All</td><td>390</td><td>0.00</td><td>0.00</td><td>0.00</td><td>0.00</td><td>0.00</td><td>0.00</td><td>0.43</td><td>3.11</td><td>0.20</td><td>null</td><td>null</td><td></td><td>null</td><td>null</td><td></td><td>null</td><td>null</td><td></td><td>null</td><td>null</td><td></td><td>2.84E+00</td><td>3.39E-05</td><td>Up</td><td>-1.09E+00</td><td>5.53E-01</td><td></td><td></td><td></td><td></td><td></td><td></td><td></td><td></td></tr>
<tr><td>Unigene117365_All</td><td>392</td><td>0.13</td><td>0.26</td><td>0.00</td><td>0.89</td><td>1.32</td><td>0.92</td><td>0.54</td><td>99.26</td><td>1.52</td><td>9.53E-01</td><td>7.27E-01</td><td></td><td>-7.06E+00</td><td>6.28E-01</td><td></td><td>5.67E-01</td><td>6.04E-01</td><td></td><td>5.13E-02</td><td>9.69E-01</td><td></td><td>7.52E+00</td><td>2.68E-265</td><td>Up</td><td>1.50E+00</td><td>7.88E-02</td><td></td><td></td><td></td><td>Unigene117365_All//3.00E-08//TC148689</td><td></td><td></td><td></td><td>ESTscan -</td></tr>
<tr><td>Unigene117366_All</td><td>392</td><td>0.00</td><td>0.00</td><td>0.00</td><td>0.00</td><td>0.00</td><td>0.00</td><td>0.00</td><td>1.71</td><td>0.00</td><td>null</td><td>null</td><td></td><td>null</td><td>null</td><td></td><td>null</td><td>null</td><td></td><td>null</td><td>null</td><td></td><td>1.07E+01</td><td>7.64E-05</td><td>Up</td><td>null</td><td>null</td><td></td><td>Unigene117366_All//gi|1743354|emb|CAA71003.1|//5.00E-40//aldehyde dehydrogenase (NAD+) [Nicotiana tabacum]</td><td>Unigene117366_All//1.00E-40//AT3G48000| ALDH2B4 (ALDEHYDE DEHYDROGENASE 2B4); 3-chloroallyl aldehyde dehydrogenase/ ATP binding / aldehyde dehydrogenase (NAD)</td><td></td><td></td><td>ko00940|Phenylpropanoid biosynthesis//ko00010|Glycolysis / Gluconeogenesis//ko00903|Limonene and pinene degradation//ko00620|Pyruvate metabolism//ko00380|Tryptophan metabolism//ko00071|Fatty acid metabolism//ko00053|Ascorbate and aldarate metabolism//ko00330|Arginine and proline metabolism//ko00280|Valine, leucine and isoleucine degradation//ko00640|Propanoate metabolism//ko00561|Glycerolipid metabolism//ko00310|Lysine degradation//ko00410|beta-Alanine metabolism//ko00340|Histidine metabolism</td><td></td><td>nr -</td></tr>
<tr><td>Unigene117369_All</td><td>394</td><td>0.00</td><td>0.00</td><td>0.00</td><td>0.00</td><td>0.00</td><td>0.00</td><td>0.21</td><td>4.67</td><td>0.10</td><td>null</td><td>null</td><td></td><td>null</td><td>null</td><td></td><td>null</td><td>null</td><td></td><td>null</td><td>null</td><td></td><td>4.44E+00</td><td>1.47E-10</td><td>Up</td><td>-1.09E+00</td><td>6.77E-01</td><td></td><td></td><td></td><td></td><td></td><td></td><td></td><td></td></tr>
<tr><td>Unigene11737_All</td><td>1646</td><td>29.02</td><td>17.12</td><td>25.91</td><td>11.92</td><td>12.24</td><td>10.59</td><td>7.55</td><td>4.88</td><td>3.27</td><td>-7.62E-01</td><td>2.95E-22</td><td></td><td>-1.63E-01</td><td>5.55E-02</td><td></td><td>3.76E-02</td><td>8.39E-01</td><td></td><td>-1.71E-01</td><td>2.26E-01</td><td></td><td>-6.31E-01</td><td>9.49E-06</td><td></td><td>-1.21E+00</td><td>4.38E-16</td><td>Down</td><td>Unigene11737_All//gi|58760232|gb|AAW82030.1|//9.00E-93//inositol monophosphatase [Chlamydomonas incerta]</td><td>Unigene11737_All//4.00E-82//AT1G31190| IMPL1 (MYO-INOSITOL MONOPHOSPHATASE LIKE 1); 3’(2’),5’-bisphosphate nucleotidase/ inositol or phosphatidylinositol phosphatase/ inositol-1(or 4)-monophosphatase</td><td>Unigene11737_All//0.00E+00//CO132745</td><td></td><td>ko04070|Phosphatidylinositol signaling system//ko00562|Inositol phosphate metabolism//ko00053|Ascorbate and aldarate metabolism</td><td>GO:0004437//GO:0008252//GO:0009532//GO:0044237</td><td>nr +</td></tr>
<tr><td>Unigene117371_All</td><td>394</td><td>0.27</td><td>0.00</td><td>0.00</td><td>0.00</td><td>0.17</td><td>0.46</td><td>0.64</td><td>8.27</td><td>1.21</td><td>-8.05E+00</td><td>3.74E-01</td><td></td><td>-8.05E+00</td><td>3.96E-01</td><td></td><td>7.45E+00</td><td>6.12E-01</td><td></td><td>8.84E+00</td><td>1.95E-01</td><td></td><td>3.68E+00</td><td>3.48E-16</td><td>Up</td><td>9.14E-01</td><td>3.38E-01</td><td></td><td></td><td></td><td></td><td></td><td></td><td></td><td></td></tr>
</table>

Unigene117372_All	395	0.00	0.00	0.00	0.00	0.00	0.00	0.00	2.86	0.00	null	null		null	null		null	null		null	null		1.15E+01	5.32E-08	Up	null	null		Unigene117372_All//gi|145344436|ref|XP_001416738.1|//6.00E-56//Peptidyl-prolyl cis-trans isomerase, cyclophilin-type [Ostreococcus lucimarinus CCE9901] >gi|144576964|gb|ABO95031.1| Peptidyl-prolyl cis-trans isomerase, cyclophilin-type [Ostreococcus lucimarinus CCE9901]	Unigene117372_All//2.00E-45//AT3G56070| ROC2 (ROTAMASE CYCLOPHILIN 2); cyclosporin A binding / peptidyl-prolyl cis-trans isomerase	Unigene117372_All//3.00E-11//gi|48796304|gb|CO097618.1|CO097618				nr +
Unigene117373_All	396	0.26	0.00	0.54	0.00	0.00	0.00	0.00	11.93	0.10	-8.05E+00	3.74E-01		1.03E+00	5.92E-01		null	null		null	null		1.35E+01	2.92E-33	Up	6.65E+00	6.46E-01				Unigene117373_All//5.00E-07//TC173397				
Unigene117374_All	398	0.00	0.00	0.00	0.00	0.00	0.00	0.00	2.10	0.00	null	null		null	null		null	null		null	null		1.10E+01	5.54E-06	Up	null	null		Unigene117374_All//gi|293336034|ref|NP_001168895.1|//4.00E-11//hypothetical protein LOC100382700 [Zea mays] >gi|223973529|gb|ACN30952.1| unknown [Zea mays]						nr -
Unigene117375_All	398	0.00	0.00	0.00	0.00	0.09	0.00	0.00	4.20	0.00	null	null		null	null		6.43E+00	8.34E-01		null	null		1.20E+01	8.75E-12	Up	null	null								
Unigene117376_All	402	0.00	0.00	0.00	0.00	0.00	0.00	0.00	3.22	0.00	null	null		null	null		null	null		null	null		1.17E+01	3.67E-09	Up	null	null								
Unigene117378_All	403	0.00	0.00	0.00	0.00	0.00	0.00	0.73	4.36	0.00	null	null		null	null		null	null		null	null		2.57E+00	1.41E-06	Up	-9.52E+00	1.58E-02				Unigene117378_All//2.00E-09//TC137772				ESTscan +
Unigene117379_All	403	0.00	0.00	0.00	0.00	0.00	0.00	0.21	5.70	0.00	null	null		null	null		null	null		null	null		4.76E+00	1.36E-13	Up	-7.71E+00	3.59E-01		Unigene117379_All//gi|226458329|gb|EEH55626.1|//8.00E-09//predicted protein [Micromonas pusilla CCMP1545]	Unigene117379_All//9.00E-05//AT2G02380| ATGSTZ2; catalytic/ glutathione transferase					nr -
Unigene11738_All	1911	1.51	2.23	2.73	21.22	18.07	16.61	13.41	3.15	5.23	5.65E-01	5.65E-02		8.59E-01	1.65E-03		-2.31E-01	4.68E-03		-3.53E-01	1.57E-05		-2.09E+00	4.25E-68	Down	-1.36E+00	1.99E-38	Down	Unigene11738_All//gi|297805050|ref|XP_002870409.1|//0.00E+00//glycosyl hydrolase family 79 N-terminal domain-containing protein [Arabidopsis lyrata subsp. lyrata] >gi|297316245|gb|EFH46668.1| glycosyl hydrolase family 79 N-terminal domain-containing protein [Arabidopsis lyrata subsp. lyrata]	Unigene11738_All//0.00E+00//AT5G34940| AtGUS3 (Arabidopsis thaliana glucuronidase 3); beta-glucuronidase	Unigene11738_All//0.00E+00//TC157182			GO:0005618//GO:0016787//GO:0031410//GO:0043167//GO:0044238	nr +
Unigene117381_All	408	0.00	0.00	0.00	0.00	0.00	0.00	2.07	4.92	0.10	null	null		null	null		null	null		null	null		1.25E+00	2.74E-03		-4.41E+00	2.37E-05	Down	Unigene117381_All//gi|55168340|gb|AAV44205.1|//1.00E-06//unknow protein [Oryza sativa Japonica Group]		Unigene117381_All//9.00E-21//TC139311				nr +
Unigene117384_All	413	0.00	0.00	0.00	0.00	0.00	0.00	0.00	1.52	0.00	null	null		null	null		null	null		null	null		1.06E+01	1.47E-04	Up	null	null		Unigene117384_All//gi|297242517|gb|ADI24922.1|//5.00E-39//pirin [Carica papaya]	Unigene117384_All//3.00E-37//AT2G43120| pirin, putative					nr +
Unigene117386_All	413	1.01	0.00	0.77	0.36	0.50	0.66	0.31	2.33	0.77	-9.99E+00	1.05E-02		-3.90E-01	7.17E-01		4.68E-01	8.25E-01		8.59E-01	6.31E-01		2.92E+00	2.38E-04	Up	1.33E+00	2.93E-01				Unigene117386_All//5.00E-07//TC177933				
Unigene11739_All	1948	8.70	6.74	9.35	25.83	22.04	32.44	29.04	13.50	15.75	-3.69E-01	6.79E-03		1.03E-01	5.02E-01		-2.29E-01	1.49E-03		3.29E-01	3.02E-07		-1.11E+00	3.85E-59	Down	-8.83E-01	3.61E-42		Unigene11739_All//gi|297805050|ref|XP_002870409.1|//0.00E+00//glycosyl hydrolase family 79 N-terminal domain-containing protein [Arabidopsis lyrata subsp. lyrata] >gi|297316245|gb|EFH46668.1| glycosyl hydrolase family 79 N-terminal domain-containing protein [Arabidopsis lyrata subsp. lyrata]	Unigene11739_All//0.00E+00//AT5G34940| AtGUS3 (Arabidopsis thaliana glucuronidase 3); beta-glucuronidase	Unigene11739_All//0.00E+00//TC141405			GO:0005618//GO:0016787//GO:0031410//GO:0043167//GO:0044238	nr +
Unigene117390_All	420	0.00	0.00	0.00	0.00	0.00	0.00	0.00	3.48	0.47	null	null		null	null		null	null		null	null		1.18E+01	2.52E-10	Up	8.89E+00	7.90E-02								
Unigene117391_All	421	0.00	0.00	0.00	0.00	0.00	0.00	1.51	1.69	0.00	null	null		null	null		null	null		null	null		1.64E-01	8.24E-01		-1.06E+01	7.11E-05	Down	Unigene117391_All//gi|8050572|gb|AAF71706.1|AF213663_1//9.00E-50//elongation factor 2 [Euglena gracilis]	Unigene117391_All//2.00E-48//AT1G56070| LOS1; copper ion binding / translation elongation factor/ translation factor, nucleic acid binding	Unigene117391_All//3.00E-08//ES844303				nr -
Unigene117392_All	422	0.00	0.00	0.00	0.00	0.00	0.00	1.30	7.43	0.00	null	null		null	null		null	null		null	null		2.51E+00	5.72E-11	Up	-1.03E+01	2.80E-04	Down	Unigene117392_All//gi|115435924|ref|NP_001042720.1|//5.00E-06//Os01g0274800 [Oryza sativa Japonica Group] >gi|113532251|dbj|BAF04634.1| Os01g0274800 [Oryza sativa Japonica Group]						nr -
Unigene117393_All	422	0.00	2.52	0.25	0.35	0.16	0.75	2.00	2.48	0.28	1.13E+01	3.82E-06	Up	7.98E+00	4.01E-01		-1.12E+00	5.84E-01		1.08E+00	4.76E-01		3.05E-01	6.58E-01		-2.82E+00	5.38E-04	Down							
Unigene117397_All	425	1115.60	789.61	1219.11	227.86	327.18	190.96	1.19	4.03	1.50	-4.99E-01	5.20E-101		1.28E-01	1.40E-08		5.22E-01	9.93E-39		-2.55E-01	6.45E-07		1.76E+00	2.64E-04	Up	3.29E-01	6.60E-01		Unigene117397_All//gi|62910995|gb|AAY21210.1|//1.00E-36//ultraviolet-B-repressible protein [Gossypium hirsutum]		Unigene117397_All//0.00E+00//gi|84148317|gb|DN779617.1|DN779617				nr -
Unigene117398_All	426	0.00	0.00	0.00	0.00	0.00	0.00	0.00	1.37	0.00	null	null		null	null		null	null		null	null		1.04E+01	2.81E-04	Up	null	null		Unigene117398_All//sp|P19752|HSP30_NEUCR//8.00E-16//30 kDa heat shock protein OS=Neurospora crassa GN=hsp30 PE=3 SV=1						swissprot +
Unigene1174_All	476	4.29	8.73	3.24	3.87	4.12	10.15	3.64	4.92	1.84	1.03E+00	6.93E-04	Up	-4.02E-01	3.83E-01		9.08E-02	8.64E-01		1.39E+00	8.19E-07	Up	4.33E-01	2.64E-01		-9.84E-01	2.16E-02		Unigene1174_All//gi|1839022|emb|CAA72006.1|//2.00E-34//amino acid carrier [Ricinus communis]	Unigene1174_All//3.00E-35//AT5G49630| AAP6 (AMINO ACID PERMEASE 6); acidic amino acid transmembrane transporter/ amino acid transmembrane transporter/ neutral amino acid transmembrane transporter	Unigene1174_All//6.00E-121//TC145723			GO:0015171//GO:0015711//GO:0015801//GO:0031224//GO:0051707	nr +
Unigene11740_All	577	12.42	5.71	6.00	22.19	20.53	18.86	18.76	8.62	10.77	-1.12E+00	5.92E-07	Down	-1.05E+00	5.32E-06	Down	-1.12E-01	5.50E-01		-2.34E-01	1.77E-01		-1.12E+00	3.10E-12	Down	-8.00E-01	1.41E-07		Unigene11740_All//gi|15234416|ref|NP_192948.1|//3.00E-80//SMO1-1 (STEROL-4ALPHA-METHYL OXIDASE 1-1); 4,4-dimethyl-9beta,19-cyclopropylsterol-4alpha-methyl oxidase/ C-4 methylsterol oxidase/ catalytic [Arabidopsis thaliana] >gi|21928127|gb|AAM78091.1| AT4g12110/F16J13_180 [Arabidopsis thaliana] >gi|23308291|gb|AAN18115.1| At4g12110/F16J13_180 [Arabidopsis thaliana]	Unigene11740_All//2.00E-69//AT4G12110| SMO1-1 (STEROL-4ALPHA-METHYL OXIDASE 1-1); 4,4-dimethyl-9beta,19-cyclopropylsterol-4alpha-methyl oxidase/ C-4 methylsterol oxidase/ catalytic	Unigene11740_All//0.00E+00//TC163490		ko00100|Steroid biosynthesis	GO:0000254//GO:0006631//GO:0006694//GO:0031224//GO:0043231//GO:0046914	nr +
Unigene117400_All	426	0.00	0.00	0.00	0.00	0.00	0.00	0.00	4.12	1.22	null	null		null	null		null	null		null	null		1.20E+01	2.29E-12	Up	1.02E+01	6.14E-04	Up			Unigene117400_All//1.00E-07//NP12958110				
Unigene117401_All	428	0.00	0.00	0.00	0.00	0.00	0.00	1.09	3.71	0.19	null	null		null	null		null	null		null	null		1.77E+00	4.38E-04	Up	-2.54E+00	2.35E-02		Unigene117401_All//gi|192913002|gb|ACF06609.1|//1.00E-51//ribosomal protein L19 [Elaeis guineensis]	Unigene117401_All//4.00E-37//AT3G16780| 60S ribosomal protein L19 (RPL19B)	Unigene117401_All//1.00E-07//ES804152		ko03010|Ribosome		nr +
Unigene117402_All	431	0.00	0.00	0.00	0.00	0.00	0.00	2.26	3.01	0.00	null	null		null	null		null	null		null	null		4.14E-01	4.51E-01		-1.11E+01	2.72E-07	Down	Unigene117402_All//gi|109391829|gb|ABG33906.1|//3.00E-26//Ole e 2 allergen [Olea europaea]	Unigene117402_All//3.00E-18//AT2G19770| PRF5 (PROFILIN5); actin binding / actin monomer binding					nr +
Unigene117405_All	434	0.00	0.00	0.00	0.00	0.00	0.00	0.29	5.01	0.00	null	null		null	null		null	null		null	null		4.10E+00	8.27E-12	Up	-8.19E+00	2.00E-01								ESTscan -
Unigene117406_All	435	0.00	0.00	0.00	0.00	0.00	0.00	0.10	2.21	0.00	null	null		null	null		null	null		null	null		4.51E+00	8.92E-06	Up	-6.60E+00	6.14E-01		Unigene117406_All//sp|P48060|GLIP1_HUMAN//3.00E-07//Glioma pathogenesis-related protein 1 OS=Homo sapiens GN=GLIPR1 PE=1 SV=3	Unigene117406_All//1.00E-05//AT2G14610| PR1 (PATHOGENESIS-RELATED GENE 1)			ko04626|Plant-pathogen interaction		swissprot +
Unigene117407_All	443	0.00	0.00	0.00	0.00	0.00	0.00	0.38	2.36	0.00	null	null		null	null		null	null		null	null		2.63E+00	2.86E-04	Up	-8.58E+00	1.08E-01								ESTscan -
Unigene117408_All	444	0.00	0.00	0.00	0.00	0.00	0.00	0.19	2.54	0.00	null	null		null	null		null	null		null	null		3.74E+00	5.20E-06	Up	-7.57E+00	3.59E-01		Unigene117408_All//gi|53801490|gb|AAU93947.1|//1.00E-19//thioredoxin H [Helicosporidium sp. ex Simulium jonesi]	Unigene117408_All//2.00E-17//AT1G19730| ATTRX4; oxidoreductase, acting on sulfur group of donors, disulfide as acceptor					nr +
Unigene11741_All	1452	20.58	8.41	11.52	13.52	11.12	12.47	12.26	6.22	8.42	-1.29E+00	6.56E-33	Down	-8.37E-01	6.73E-16		-2.81E-01	2.25E-02		-1.16E-01	4.43E-01		-9.79E-01	7.37E-16		-5.41E-01	2.12E-06		Unigene11741_All//gi|15234416|ref|NP_192948.1|//1.00E-140//SMO1-1 (STEROL-4ALPHA-METHYL OXIDASE 1-1); 4,4-dimethyl-9beta,19-cyclopropylsterol-4alpha-methyl oxidase/ C-4 methylsterol oxidase/ catalytic [Arabidopsis thaliana] >gi|21928127|gb|AAM78091.1| AT4g12110/F16J13_180 [Arabidopsis thaliana] >gi|23308291|gb|AAN18115.1| At4g12110/F16J13_180 [Arabidopsis thaliana]	Unigene11741_All//3.00E-134//AT4G12110| SMO1-1 (STEROL-4ALPHA-METHYL OXIDASE 1-1); 4,4-dimethyl-9beta,19-cyclopropylsterol-4alpha-methyl oxidase/ C-4 methylsterol oxidase/ catalytic	Unigene11741_All//0.00E+00//gi|164310790|gb|EX171182.1|EX171182		ko00100|Steroid biosynthesis		nr +
Unigene117412_All	449	0.00	0.00	0.00	0.00	0.00	0.00	1.70	4.19	0.00	null	null		null	null		null	null		null	null		1.31E+00	2.64E-03		-1.07E+01	8.87E-06	Down	Unigene117412_All//gi|224006369|ref|XP_002292145.1|//4.00E-41//sulfolipid biosynthesis protein [Thalassiosira pseudonana CCMP1335] >gi|220972664|gb|EED90996.1| sulfolipid biosynthesis protein [Thalassiosira pseudonana CCMP1335]	Unigene117412_All//1.00E-27//AT4G33030| SQD1; UDPsulfoquinovose synthase/ sulfotransferase			ko00520|Amino sugar and nucleotide sugar metabolism//ko00561|Glycerolipid metabolism		nr -
Unigene117413_All	450	0.00	0.00	0.00	0.00	0.00	0.00	555.84	214.27	797.22	null	null		null	null		null	null		null	null		-1.38E+00	0.00E+00	Down	5.20E-01	1.80E-103		Unigene117413_All//gi|124365601|gb|AAK50778.4|AF361893_1//7.00E-14//bacterial-induced lipoxygenase [Gossypium hirsutum]	Unigene117413_All//2.00E-11//AT3G22400| LOX5; electron carrier/ iron ion binding / lipoxygenase/ metal ion binding / oxidoreductase, acting on single donors with incorporation of molecular oxygen, incorporation of two atoms of oxygen	Unigene117413_All//8.00E-43//TC147960		ko00592|alpha-Linolenic acid metabolism//ko00591|Linoleic acid metabolism		nr +
Unigene117414_All	456	0.00	0.00	0.00	0.00	0.00	0.00	2.32	3.21	0.00	null	null		null	null		null	null		null	null		4.69E-01	3.70E-01		-1.12E+01	6.72E-08	Down	Unigene117414_All//gi|298705540|emb|CBJ28807.1|//5.00E-06//conserved unknown protein [Ectocarpus siliculosus]						nr -
Unigene117415_All	457	0.00	0.00	0.00	0.00	0.00	0.00	0.65	3.48	0.00	null	null		null	null		null	null		null	null		2.42E+00	1.10E-05	Up	-9.34E+00	1.58E-02		Unigene117415_All//gi|18406066|ref|NP_565985.1|//9.00E-59//PP5.2 (PROTEIN PHOSPHATASE 5.2); phosphoprotein phosphatase/ protein binding / protein serine/threonine phosphatase [Arabidopsis thaliana] >gi|16930441|gb|AAL31906.1|AF419574_1 At2g42810/F7D19.19 [Arabidopsis thaliana] >gi|33589766|gb|AAQ22649.1| At2g42810/F7D19.19 [Arabidopsis thaliana]	Unigene117415_All//6.00E-60//AT2G42810| PP5.2 (PROTEIN PHOSPHATASE 5.2); phosphoprotein phosphatase/ protein binding / protein serine/threonine phosphatase					nr -
Unigene117416_All	458	0.00	0.00	0.00	0.00	0.00	0.00	0.18	2.19	0.00	null	null		null	null		null	null		null	null		3.57E+00	3.08E-05	Up	-7.53E+00	3.59E-01								ESTscan +
Unigene117417_All	459	0.00	0.00	0.00	0.00	0.00	0.00	6.26	8.83	0.43	null	null		null	null		null	null		null	null		4.96E-01	7.25E-02		-3.85E+00	1.93E-15	Down							
Unigene117418_All	459	0.00	0.00	0.00	0.00	0.00	0.00	0.00	2.64	0.00	null	null		null	null		null	null		null	null		1.14E+01	1.40E-08	Up	null	null								ESTscan -
Unigene11742_All	728	3.45	29.44	8.19	4.93	21.29	33.81	22.07	46.33	69.12	3.09E+00	4.02E-72	Up	1.25E+00	1.62E-06	Up	2.11E+00	1.45E-39	Up	2.78E+00	1.99E-79	Up	1.07E+00	9.78E-34	Up	1.65E+00	3.65E-98	Up	Unigene11742_All//gi|20197500|gb|AAD03372.2|//2.00E-46//expressed protein [Arabidopsis thaliana]	Unigene11742_All//1.00E-47//AT2G24100| unknown protein	Unigene11742_All//0.00E+00//TC140257				nr -
Unigene117420_All	465	0.00	0.00	0.00	0.00	0.00	0.00	5.55	2.88	0.34	null	null		null	null		null	null		null	null		-9.47E-01	7.11E-03		-4.02E+00	2.52E-14	Down	Unigene117420_All//gi|226461892|gb|EEH59185.1|//7.00E-11//transcription elongation-nucleosome displacement protein Spt6 [Micromonas pusilla CCMP1545]						nr +

Unigene117421_All	468	0.00	0.00	0.00	0.00	0.00	0.00	0.00	2.95	0.00	null	null		null	null		null	null		null	null		1.15E+01	9.64E-10	Up	null	null		Unigene117421_All//gi|5002232|gb|AAD37373.1|AF145234_1//3.00E-43//NADPH:quinone oxidoreductase [Arabidopsis thaliana]	Unigene117421_All//9.00E-38//AT3G27890| NQR (NADPH:QUINONE OXIDOREDUCTASE); FMN reductase					nr +
Unigene117422_All	468	0.00	0.00	0.00	0.00	0.00	0.00	0.90	3.48	0.09	null	null		null	null		null	null		null	null		1.95E+00	1.26E-04	Up	-3.41E+00	1.24E-02		Unigene117422_All//gi|159483497|ref|XP_001699797.1|//1.00E-45//ribosomal protein S3, component of cytosolic 80S ribosome and 40S small subunit [Chlamydomonas reinhardtii] >gi|158281739|gb|EDP07493.1| ribosomal protein S3, component of cytosolic 80S ribosome and 40S small subunit [Chlamydomonas reinhardtii]	Unigene117422_All//8.00E-47//AT2G31610| 40S ribosomal protein S3 (RPS3A)			ko03010|Ribosome		nr +
Unigene117423_All	469	0.00	0.00	0.00	0.00	0.00	0.00	1.62	3.74	0.00	null	null		null	null		null	null		null	null		1.21E+00	7.16E-03		-1.07E+01	8.87E-06	Down							ESTscan +
Unigene11743_All	927	2.71	21.32	9.02	4.62	25.00	32.45	12.95	32.33	47.40	2.98E+00	4.72E-64	Up	1.74E+00	4.58E-14	Up	2.44E+00	3.63E-70	Up	2.81E+00	3.12E-98	Up	1.32E+00	1.32E-41	Up	1.87E+00	2.15E-102	Up	Unigene11743_All//gi|20197500|gb|AAD03372.2|//5.00E-13//expressed protein [Arabidopsis thaliana]	Unigene11743_All//1.00E-14//AT4G30780| unknown protein	Unigene11743_All//4.00E-176//gi|73864036|gb|DT466775.1|DT466775				nr +
Unigene117431_All	477	0.00	0.00	0.00	0.00	0.00	0.00	0.00	3.24	0.00	null	null		null	null		null	null		null	null		1.17E+01	6.59E-11	Up	null	null		Unigene117431_All//gi|18408550|ref|NP_564878.1|//1.00E-55//60S ribosomal protein L10 (RPL10C) [Arabidopsis thaliana] >gi|297838747|ref|XP_002887255.1| 60S ribosomal protein L10 [Arabidopsis lyrata subsp. lyrata] >gi|75249285|sp|Q93W22.1|RL103_ARATH RecName: Full=60S ribosomal protein L10-3 >gi|14194141|gb|AAK56265.1|AF367276_1 At1g66580/T12I7_3 [Arabidopsis thaliana] >gi|15777885|gb|AAL05903.1| At1g66580/T12I7_3 [Arabidopsis thaliana] >gi|297333096|gb|EFH63514.1| 60S ribosomal protein L10 [Arabidopsis lyrata subsp. lyrata]	Unigene117431_All//9.00E-57//AT1G66580| 60S ribosomal protein L10 (RPL10C)	Unigene117431_All//4.00E-14//CD486423		ko03010|Ribosome		nr +
Unigene117432_All	479	0.00	0.00	0.00	0.00	0.00	0.00	1.15	1.92	0.00	null	null		null	null		null	null		null	null		7.42E-01	2.60E-01		-1.02E+01	2.80E-04	Down	Unigene117432_All//gi|145344432|ref|XP_001416736.1|//2.00E-47//Ribosomal protein L10, component of cytosolic 80S ribosome and 60S large subunit [Ostreococcus lucimarinus CCE9901] >gi|144576962|gb|ABO95029.1| Ribosomal protein L10, component of cytosolic 80S ribosome and 60S large subunit [Ostreococcus lucimarinus CCE9901]	Unigene117432_All//8.00E-48//AT1G26910| 60S ribosomal protein L10 (RPL10B)	Unigene117432_All//2.00E-15//CD485839		ko03010|Ribosome		nr +
Unigene117433_All	480	0.00	0.00	0.00	0.00	0.00	0.00	2.11	11.15	0.75	null	null		null	null		null	null		null	null		2.40E+00	2.50E-17	Up	-1.50E+00	1.41E-02								
Unigene117434_All	481	0.00	0.00	0.00	0.00	0.00	0.00	0.53	5.39	0.00	null	null		null	null		null	null		null	null		3.35E+00	4.80E-12	Up	-9.04E+00	3.04E-02								ESTscan +
Unigene117437_All	492	0.43	0.21	0.43	0.20	0.28	0.00	0.60	4.33	1.86	-1.05E+00	5.65E-01		2.52E-02	1.00E+00		4.68E-01	8.77E-01		-7.66E+00	4.02E-01		2.85E+00	1.12E-08	Up	1.63E+00	1.45E-02								
Unigene117438_All	493	0.00	0.21	0.00	0.10	0.14	0.00	0.51	2.80	0.00	7.68E+00	3.89E-01		null	null		4.68E-01	9.60E-01		-6.66E+00	6.40E-01		2.44E+00	4.59E-05	Up	-9.01E+00	3.04E-02		Unigene117438_All//gi|297720735|ref|NP_001172729.1|//5.00E-44//Os01g0935200 [Oryza sativa Japonica Group] >gi|148921414|dbj|BAF64446.1| HAP3 subunit of HAP complex [Oryza sativa Japonica Group] >gi|255674039|dbj|BAH91459.1| Os01g0935200 [Oryza sativa Japonica Group]	Unigene117438_All//3.00E-41//AT2G47810| NF-YB5 (NUCLEAR FACTOR Y, SUBUNIT B5); transcription factor		CCAAT	ko00230|Purine metabolism//ko00240|Pyrimidine metabolism//ko03420|Nucleotide excision repair//ko03410|Base excision repair//ko03030|DNA replication		nr -
Unigene11744_All	1983	12.66	72.16	30.80	18.97	89.26	125.29	40.57	110.80	183.76	2.51E+00	0.00E+00	Up	1.28E+00	7.88E-64	Up	2.23E+00	0.00E+00	Up	2.72E+00	0.00E+00	Up	1.45E+00	0.00E+00	Up	2.18E+00	0.00E+00	Up	Unigene11744_All//gi|115466824|ref|NP_001057011.1|//1.00E-116//Os06g0186600 [Oryza sativa Japonica Group] >gi|113595051|dbj|BAF18925.1| Os06g0186600 [Oryza sativa Japonica Group]	Unigene11744_All//2.00E-110//AT2G24100| unknown protein	Unigene11744_All//0.00E+00//TC138776				nr -
Unigene117440_All	506	0.21	0.00	0.00	0.10	0.00	0.00	0.58	3.80	0.24	-7.69E+00	3.74E-01		-7.69E+00	3.96E-01		-6.62E+00	5.59E-01		-6.62E+00	6.40E-01		2.70E+00	1.69E-07	Up	-1.31E+00	3.13E-01		Unigene117440_All//gi|95117792|gb|ABF57084.1|//1.00E-60//GDHB glutamate dehydrogenase [Vitis vinifera] >gi|95117794|gb|ABF57085.1| GDHB glutamate dehydrogenase [Vitis vinifera]	Unigene117440_All//2.00E-58//AT5G18170| GDH1 (GLUTAMATE DEHYDROGENASE 1); ATP binding / glutamate dehydrogenase [NAD(P)+]/ oxidoreductase	Unigene117440_All//2.00E-80//TC143512		ko00910|Nitrogen metabolism//ko00250|Alanine, aspartate and glutamate metabolism//ko00330|Arginine and proline metabolism		nr +
Unigene117441_All	516	0.00	0.00	0.00	0.00	0.00	0.00	0.90	3.00	0.00	null	null		null	null		null	null		null	null		1.73E+00	6.66E-04	Up	-9.82E+00	1.09E-03		Unigene117441_All//gi|192910906|gb|ACF06561.1|//2.00E-49//ribosomal protein L18a [Elaeis guineensis]	Unigene117441_All//1.00E-45//AT1G29970| RPL18AA (60S RIBOSOMAL PROTEIN L18A-1)			ko03010|Ribosome		nr -
Unigene117442_All	519	0.00	0.00	0.00	0.00	0.00	0.00	1.47	4.03	0.00	null	null		null	null		null	null		null	null		1.46E+00	4.39E-04	Up	-1.05E+01	8.87E-06	Down							ESTscan -
Unigene117443_All	521	0.00	0.00	0.00	0.00	0.00	0.00	0.73	3.05	0.00	null	null		null	null		null	null		null	null		2.06E+00	8.20E-05	Up	-9.51E+00	4.19E-03		Unigene117443_All//gi|300262824|gb|EFJ47028.1|//3.00E-10//transcription factor jumonji domain-containing protein [Volvox carteri f. nagariensis]				ko04144|Endocytosis		nr -
Unigene117444_All	530	0.00	0.57	0.00	0.38	0.26	0.09	0.32	3.39	1.20	9.16E+00	4.44E-02		null	null		-5.33E-01	7.49E-01		-2.14E+00	3.47E-01		3.41E+00	1.21E-08	Up	1.91E+00	2.60E-02								
Unigene117446_All	544	0.00	0.00	0.00	0.00	0.00	0.00	1.01	3.61	0.29	null	null		null	null		null	null		null	null		1.84E+00	4.40E-05	Up	-1.79E+00	5.07E-02								
Unigene117447_All	544	0.00	0.00	0.00	0.00	0.00	0.00	0.78	2.84	0.00	null	null		null	null		null	null		null	null		1.87E+00	3.06E-04	Up	-9.60E+00	2.14E-03		Unigene117447_All//gi|2500261|sp|P93099.1|RL13A_CYAPA//4.00E-52//RecName: Full=60S ribosomal protein L13a >gi|1743007|emb|CAA71090.1| ribosomal protein L13a [Cyanophora paradoxa]	Unigene117447_All//2.00E-46//AT5G48760| 60S ribosomal protein L13A (RPL13aD)			ko03010|Ribosome		nr +
Unigene117448_All	552	0.00	0.00	0.00	0.00	0.00	0.00	1.46	1.89	0.07	null	null		null	null		null	null		null	null		3.79E-01	5.56E-01		-4.33E+00	4.54E-05	Down	Unigene117448_All//gi|160330969|ref|XP_001712192.1|//6.00E-81//ef2 [Hemiselmis andersenii] >gi|159765639|gb|ABW97867.1| ef2 [Hemiselmis andersenii]	Unigene117448_All//5.00E-73//AT1G56070| LOS1; copper ion binding / translation elongation factor/ translation factor, nucleic acid binding	Unigene117448_All//7.00E-07//BF277406				nr -
Unigene117449_All	556	0.00	0.00	0.00	0.00	0.00	0.00	0.00	3.38	0.00	null	null		null	null		null	null		null	null		1.17E+01	3.04E-13	Up	null	null								ESTscan -
Unigene11745_All	2188	2.80	1.41	1.44	2.28	3.76	2.97	8.14	21.83	30.86	-9.86E-01	4.56E-05		-9.62E-01	1.24E-04		7.24E-01	1.56E-04		3.85E-01	1.15E-01		1.42E+00	6.48E-74	Up	1.92E+00	7.97E-163	Up	Unigene11745_All//gi|115469978|ref|NP_001058588.1|//1.00E-149//Os06g0716000 [Oryza sativa Japonica Group] >gi|53791786|dbj|BAD53580.1| receptor-like protein kinase-like [Oryza sativa Japonica Group] >gi|113596628|dbj|BAF20502.1| Os06g0716000 [Oryza sativa Japonica Group]	Unigene11745_All//6.00E-168//AT1G30755| unknown protein	Unigene11745_All//4.00E-178//DT047820				nr +
Unigene117450_All	557	0.00	0.00	0.00	0.00	0.00	0.00	1.06	4.13	0.79	null	null		null	null		null	null		null	null		1.96E+00	2.90E-06	Up	-4.34E-01	6.08E-01								
Unigene117451_All	558	0.00	0.00	0.00	0.00	0.00	0.00	0.30	3.22	0.00	null	null		null	null		null	null		null	null		3.41E+00	1.21E-08	Up	-8.24E+00	1.08E-01								
Unigene117452_All	559	0.19	0.00	0.00	0.00	0.00	0.16	1.29	4.64	2.28	-7.55E+00	3.74E-01		-7.55E+00	3.96E-01		null	null		7.34E+00	4.79E-01		1.85E+00	1.60E-06	Up	8.27E-01	1.08E-01				Unigene117452_All//1.00E-35//DN800918				
Unigene117453_All	564	0.00	0.00	0.00	0.00	0.00	0.00	0.22	2.82	0.35	null	null		null	null		null	null		null	null		3.65E+00	4.24E-08	Up	6.51E-01	6.66E-01		Unigene117453_All//gi|21593366|gb|AAM65315.1|//2.00E-28//ethylene-forming-enzyme-like dioxygenase-like protein [Arabidopsis thaliana]	Unigene117453_All//1.00E-29//AT5G20400| oxidoreductase, 2OG-Fe(II) oxygenase family protein	Unigene117453_All//0.00E+00//CO125364		ko00270|Cysteine and methionine metabolism//ko00904|Diterpenoid biosynthesis		nr -
Unigene117455_All	569	0.00	0.00	0.00	0.00	0.00	0.00	0.07	2.79	0.14	null	null		null	null		null	null		null	null		5.23E+00	6.39E-10	Up	9.14E-01	7.50E-01		Unigene117455_All//gi|55168340|gb|AAV44205.1|//2.00E-07//unknow protein [Oryza sativa Japonica Group]		Unigene117455_All//7.00E-07//EX171356				nr +
Unigene117459_All	586	0.00	0.00	0.00	0.00	0.00	0.00	0.07	0.21	1.50	null	null		null	null		null	null		null	null		1.57E+00	5.46E-01		4.37E+00	2.26E-05	Up	Unigene117459_All//gi|75321780|sp|Q5W274.1|PDR3_TOBAC//4.00E-78//RecName: Full=Pleiotropic drug resistance protein 3; AltName: Full=NtPDR3 >gi|55056942|emb|CAH39853.1| PDR-like ABC transporter [Nicotiana tabacum]	Unigene117459_All//2.00E-76//AT3G53480| PDR9 (PLEIOTROPIC DRUG RESISTANCE 9); ATPase, coupled to transmembrane movement of substances			ko03040|Spliceosome//ko02010|ABC transporters		nr +
Unigene11746_All	2426	1.62	13.18	3.58	1.19	2.90	2.72	17.57	36.02	47.95	3.03E+00	4.19E-105	Up	1.15E+00	4.19E-08	Up	1.28E+00	3.41E-09	Up	1.19E+00	2.81E-07	Up	1.04E+00	8.89E-82	Up	1.45E+00	1.94E-187	Up	Unigene11746_All//gi|115469978|ref|NP_001058588.1|//1.00E-142//Os06g0716000 [Oryza sativa Japonica Group] >gi|53791786|dbj|BAD53580.1| receptor-like protein kinase-like [Oryza sativa Japonica Group] >gi|113596628|dbj|BAF20502.1| Os06g0716000 [Oryza sativa Japonica Group]	Unigene11746_All//2.00E-163//AT1G30755| unknown protein	Unigene11746_All//0.00E+00//DT464104				nr +
Unigene117460_All	591	0.27	0.34	0.45	0.76	0.00	0.84	0.64	2.62	0.81	3.69E-01	8.17E-01		7.62E-01	6.44E-01		-9.57E+00	2.00E-03		1.48E-01	9.06E-01		2.02E+00	1.30E-04	Up	3.29E-01	7.08E-01		Unigene117460_All//gi|223452528|gb|ACM89591.1|//2.00E-50//leucine-rich repeat family protein / protein kinase family protein [Glycine max]	Unigene117460_All//3.00E-44//AT4G08850| kinase	Unigene117460_All//6.00E-140//gi|48777745|gb|CO087111.1|CO087111		ko04626|Plant-pathogen interaction		nr -
Unigene117461_All	603	0.00	0.00	0.00	0.00	0.00	0.00	1.47	4.23	0.00	null	null		null	null		null	null		null	null		1.52E+00	4.46E-05	Up	-1.05E+01	1.10E-06	Down	Unigene117461_All//gi|6093872|sp|O65082.1|RL15B_PICMA//5.00E-80//RecName: Full=60S ribosomal protein L15-2 >gi|2982318|gb|AAC32144.1| probable 60S ribosomal protein L15 [Picea mariana]	Unigene117461_All//6.00E-68//AT4G17390| 60S ribosomal protein L15 (RPL15B)			ko03010|Ribosome		nr -
Unigene117462_All	613	0.09	0.00	0.17	0.16	0.45	0.07	2.76	6.00	51.54	-6.42E+00	6.16E-01		1.02E+00	7.04E-01		1.47E+00	4.20E-01		-1.14E+00	6.78E-01		1.12E+00	1.14E-04	Up	4.22E+00	1.30E-171	Up	Unigene117462_All//gi|297801996|ref|XP_002868882.1|//4.00E-46//zinc finger (B-box type) family protein [Arabidopsis lyrata subsp. lyrata] >gi|297314718|gb|EFH45141.1| zinc finger (B-box type) family protein [Arabidopsis lyrata subsp. lyrata]	Unigene117462_All//3.00E-45//AT4G39070| zinc finger (B-box type) family protein		DBB	ko04712|Circadian rhythm - plant		nr +
Unigene117463_All	613	0.00	0.00	0.00	0.00	0.00	0.00	0.48	3.34	0.00	null	null		null	null		null	null		null	null		2.79E+00	3.35E-08	Up	-8.92E+00	1.58E-02		Unigene117463_All//gi|1208549|gb|AAC49455.1|//8.00E-44//Pseudotzain [Pseudotsuga menziesii]	Unigene117463_All//3.00E-42//AT4G36880| CP1 (CYSTEINE PROTEINASE1); cysteine-type endopeptidase/ cysteine-type peptidase					nr +

Unigene117464_All	625	0.00	0.00	0.00	0.00	0.00	0.00	0.27	2.81	0.00	null	null		null	null		null	null		null	null		3.38E+00	2.17E-08	Up	-8.08E+00	1.08E-01		Unigene117464_All//gi|224101487|ref|XP_002312301.1|//1.00E-26//1-cys peroxiredoxin [Populus trichocarpa] >gi|222852121|gb|EEE89668.1| 1-cys peroxiredoxin [Populus trichocarpa]	Unigene117464_All//4.00E-21//AT1G48130| ATPER1; antioxidant/ thioredoxin peroxidase			ko00940|Phenylpropanoid biosynthesis//ko00360|Phenylalanine metabolism		nr +
Unigene117465_All	641	0.00	0.00	0.00	0.00	0.21	0.14	483.62	201.81	1138.17	null	null		null	null		7.75E+00	3.19E-01		7.14E+00	4.79E-01		-1.26E+00	0.00E+00	Down	1.23E+00	0.00E+00	Up	Unigene117465_All//gi|124365601|gb|AAK50778.4|AF361893_1//9.00E-94//bacterial-induced lipoxygenase [Gossypium hirsutum]	Unigene117465_All//8.00E-82//AT3G22400| LOX5; electron carrier/ iron ion binding / lipoxygenase/ metal ion binding / oxidoreductase, acting on single donors with incorporation of molecular oxygen, incorporation of two atoms of oxygen	Unigene117465_All//0.00E+00//gi|164249251|gb|ES810220.1|ES810220		ko00592|alpha-Linolenic acid metabolism//ko00591|Linoleic acid metabolism		nr -
Unigene117466_All	650	0.16	0.00	0.00	0.00	0.00	0.00	0.13	2.96	0.12	-7.33E+00	3.74E-01		-7.33E+00	3.96E-01		null	null		null	null		4.51E+00	4.15E-11	Up	-8.57E-02	9.73E-01		Unigene117466_All//gi|4887010|gb|AAD32141.1|AF123503_1//5.00E-96//Nt-gh3 deduced protein [Nicotiana tabacum]	Unigene117466_All//2.00E-93//AT2G14960| GH3.1	Unigene117466_All//0.00E+00//gi|78352188|gb|DT572462.1|DT572462				nr +
Unigene117467_All	652	0.00	0.00	0.00	0.00	0.00	0.00	0.71	3.27	0.00	null	null		null	null		null	null		null	null		2.20E+00	1.16E-06	Up	-9.48E+00	1.09E-03		Unigene117467_All//gi|70663909|emb|CAE01636.3|//1.00E-43//OSJNBa0029H02.21 [Oryza sativa (japonica cultivar-group)]	Unigene117467_All//2.00E-38//AT3G55280| RPL23AB (RIBOSOMAL PROTEIN L23AB); RNA binding / nucleotide binding / structural constituent of ribosome	Unigene117467_All//5.00E-08//DW519718		ko03010|Ribosome		nr +
Unigene117468_All	657	0.00	0.31	0.00	0.38	0.00	0.14	0.00	7.32	0.12	8.27E+00	1.37E-01		null	null		-8.57E+00	4.19E-02		-1.46E+00	4.07E-01		1.28E+01	7.48E-34	Up	6.92E+00	4.05E-01		Unigene117468_All//gi|125620180|gb|ABN46982.1|//1.00E-32//small molecular heat shock protein 19 [Nelumbo nucifera]	Unigene117468_All//1.00E-17//AT1G59860| 17.6 kDa class I heat shock protein (HSP17.6A-CI)					nr -
Unigene117469_All	699	0.00	0.00	0.00	0.00	0.00	0.00	0.73	2.51	0.23	null	null		null	null		null	null		null	null		1.79E+00	1.73E-04	Up	-1.67E+00	7.78E-02		Unigene117469_All//gi|18421592|ref|NP_568543.1|//7.00E-75//DegP10 (DegP protease 10); catalytic/ protein binding / serine-type endopeptidase/ serine-type peptidase [Arabidopsis thaliana] >gi|75262529|sp|Q9FIV6.1|DGP10_ARATH RecName: Full=Protease Do-like 10, mitochondrial; Flags: Precursor >gi|10178214|dbj|BAB11638.1| serine protease-like protein [Arabidopsis thaliana] >gi|17978951|gb|AAL47441.1| AT5g36950/MLF18_70 [Arabidopsis thaliana] >gi|50897262|gb|AAT85770.1| At5g36950 [Arabidopsis thaliana]	Unigene117469_All//3.00E-76//AT5G36950| DegP10 (DegP protease 10); catalytic/ protein binding / serine-type endopeptidase/ serine-type peptidase					nr +
Unigene117470_All	705	0.00	0.00	0.00	0.00	0.00	0.00	0.30	3.26	0.00	null	null		null	null		null	null		null	null		3.44E+00	5.53E-11	Up	-8.23E+00	5.80E-02		Unigene117470_All//gi|28558781|gb|AAO45752.1|//6.00E-39//pol protein [Cucumis melo]		Unigene117470_All//6.00E-48//FL577265				nr -
Unigene117471_All	729	0.43	2.02	0.51	1.44	3.35	3.59	4.93	19.09	14.59	2.23E+00	4.29E-04	Up	2.48E-01	8.23E-01		1.22E+00	2.37E-03		1.32E+00	9.26E-04	Up	1.95E+00	3.84E-34	Up	1.57E+00	5.80E-20	Up	Unigene117471_All//gi|224082488|ref|XP_002306712.1|//3.00E-54//AP2/ERF domain-containing transcription factor [Populus trichocarpa] >gi|222856161|gb|EEE93708.1| AP2/ERF domain-containing transcription factor [Populus trichocarpa]	Unigene117471_All//5.00E-20//AT5G50080| DNA binding / transcription factor	Unigene117471_All//0.00E+00//ES831270	AP2-EREBP			nr -
Unigene117472_All	754	0.00	0.00	0.00	0.00	0.00	0.00	0.56	4.32	0.00	null	null		null	null		null	null		null	null		2.95E+00	2.30E-13	Up	-9.13E+00	2.14E-03		Unigene117472_All//gi|300268606|gb|EFJ52786.1|//5.00E-63//eukaryotic translation initiation factor 1 alpha 1 [Volvox carteri f. nagariensis]	Unigene117472_All//3.00E-22//AT5G60390| elongation factor 1-alpha / EF-1-alpha					nr -
Unigene117473_All	790	0.00	0.00	0.00	0.00	0.00	0.00	0.11	2.75	0.00	null	null		null	null		null	null		null	null		4.68E+00	9.19E-13	Up	-6.74E+00	3.59E-01		Unigene117473_All//gi|600118|emb|CAA84230.1|//2.00E-10//extensin-like protein [Zea mays] >gi|1096557|prf||2111476A extensin-like domain						nr -
Unigene117474_All	809	0.00	0.00	0.13	0.49	0.17	0.11	1.31	4.34	0.39	null	null		7.04E+00	4.01E-01		-1.53E+00	2.06E-01		-2.14E+00	1.26E-01		1.73E+00	6.71E-08	Up	-1.73E+00	4.76E-03		Unigene117474_All//gi|240256087|ref|NP_194459.4|//7.00E-77//ATP binding / protein kinase/ protein serine/threonine kinase/ protein tyrosine kinase/ sugar binding [Arabidopsis thaliana]	Unigene117474_All//4.00E-78//AT4G27290| ATP binding / protein kinase/ protein serine/threonine kinase/ protein tyrosine kinase/ sugar binding					nr -
Unigene117475_All	839	0.00	0.00	0.00	0.00	0.00	0.00	0.00	9.51	0.00	null	null		null	null		null	null		null	null		1.32E+01	2.39E-56	Up	null	null		Unigene117475_All//gi|115468044|ref|NP_001057621.1|//3.00E-10//Os06g0472200 [Oryza sativa Japonica Group] >gi|113595661|dbj|BAF19535.1| Os06g0472200 [Oryza sativa Japonica Group]	Unigene117475_All//2.00E-11//AT1G75280| isoflavone reductase, putative			ko00640|Propanoate metabolism		nr +
Unigene117476_All	857	0.00	0.00	0.00	0.12	0.00	0.00	0.39	5.36	0.09	null	null		null	null		-6.86E+00	3.15E-01		-6.86E+00	4.02E-01		3.76E+00	5.49E-23	Up	-2.09E+00	1.07E-01								
Unigene117477_All	1186	0.00	0.00	0.00	0.00	0.17	0.00	453.39	146.71	1033.55	null	null		null	null		7.44E+00	1.57E-01		null	null		-1.63E+00	0.00E+00	Down	1.19E+00	0.00E+00	Up	Unigene117477_All//gi|124365601|gb|AAK50778.4|AF361893_1//1.00E-142//bacterial-induced lipoxygenase [Gossypium hirsutum]	Unigene117477_All//5.00E-125//AT1G55020| LOX1; lipoxygenase	Unigene117477_All//4.00E-167//DR463563		ko00592|alpha-Linolenic acid metabolism//ko00591|Linoleic acid metabolism		nr -
Unigene117478_All	892	0.00	0.00	0.00	0.00	0.00	0.00	0.43	0.61	18.53	null	null		null	null		null	null		null	null		5.14E-01	5.89E-01		5.44E+00	1.35E-103	Up	Unigene117478_All//gi|22218276|gb|AAM94617.1|//4.00E-14//PR10-like protein [Glycine max]		Unigene117478_All//4.00E-74//DR454610				nr -
Unigene117479_All	373	0.00	0.14	0.00	0.00	0.00	0.00	0.23	0.45	2.03	7.09E+00	6.19E-01		null	null		null	null		null	null		9.83E-01	6.34E-01		3.16E+00	6.83E-04	Up	Unigene117479_All//gi|115475257|ref|NP_001061225.1|//3.00E-14//Os08g0203700 [Oryza sativa Japonica Group] >gi|113623194|dbj|BAF23139.1| Os08g0203700 [Oryza sativa Japonica Group]	Unigene117479_All//2.00E-11//AT3G11080| AtRLP35 (Receptor Like Protein 35); kinase/ protein binding	Unigene117479_All//6.00E-108//gi|48789604|gb|CO090918.1|CO090918				nr -
Unigene117481_All	610	0.00	0.00	0.00	0.00	0.00	0.00	1.04	0.14	4.96	null	null		null	null		null	null		null	null		-2.92E+00	4.20E-03		2.26E+00	1.11E-09	Up	Unigene117481_All//gi|224057862|ref|XP_002299361.1|//2.00E-30//GH3 family protein [Populus trichocarpa] >gi|222846619|gb|EEE84166.1| GH3 family protein [Populus trichocarpa]	Unigene117481_All//4.00E-29//AT1G28130| GH3.17; indole-3-acetic acid amido synthetase	Unigene117481_All//2.00E-10//DT467060				nr +
Unigene117482_All	298	0.00	0.00	0.00	0.00	0.00	0.00	2.13	17.95	3.88	null	null		null	null		null	null		null	null		3.08E+00	1.95E-22	Up	8.65E-01	1.14E-01		Unigene117482_All//gi|33636090|emb|CAD91338.1|//5.00E-16//beta-fructofuranosidase [Glycine max]	Unigene117482_All//4.00E-16//AT3G52600| AtcwINV2 (Arabidopsis thaliana cell wall invertase 2); hydrolase, hydrolyzing O-glycosyl compounds	Unigene117482_All//5.00E-74//TC171440		ko00500|Starch and sucrose metabolism//ko00052|Galactose metabolism		nr +
Unigene117483_All	474	0.55	1.50	0.67	1.05	1.16	2.57	2.32	2.82	7.23	1.44E+00	1.06E-01		2.89E-01	8.10E-01		1.46E-01	9.05E-01		1.29E+00	4.33E-02		2.83E-01	6.34E-01		1.64E+00	2.03E-07	Up	Unigene117483_All//gi|224106844|ref|XP_002333623.1|//2.00E-33//cc-nbs-lrr resistance protein [Populus trichocarpa] >gi|222837867|gb|EEE76232.1| cc-nbs-lrr resistance protein [Populus trichocarpa]	Unigene117483_All//2.00E-13//AT3G14470| disease resistance protein (NBS-LRR class), putative	Unigene117483_All//7.00E-167//gi|109856029|gb|DW485008.1|DW485008		ko04626|Plant-pathogen interaction		nr -
Unigene117488_All	344	9.28	7.81	3.56	9.56	10.31	15.10	9.34	13.85	12.51	-2.49E-01	4.89E-01		-1.38E+00	2.46E-04	Down	1.09E-01	8.02E-01		6.60E-01	1.20E-02		5.68E-01	2.17E-02		4.21E-01	1.04E-01		Unigene117488_All//gi|115187640|gb|ABI84264.1|//5.00E-19//ubiquitin-conjugating enzyme [Arachis hypogaea]	Unigene117488_All//3.00E-19//AT3G46460| UBC13 (ubiquitin-conjugating enzyme 13); ubiquitin-protein ligase	Unigene117488_All//1.00E-56//TC161010		ko04141|Protein processing in endoplasmic reticulum//ko04120|Ubiquitin mediated proteolysis		nr +
Unigene117489_All	581	1.08	0.00	0.46	1.11	1.66	2.18	1.31	1.94	3.09	-1.01E+01	7.92E-04	Down	-1.24E+00	2.13E-01		5.74E-01	4.52E-01		9.66E-01	1.22E-01		5.68E-01	3.44E-01		1.24E+00	4.17E-03				Unigene117489_All//3.00E-55//TC173528				
Unigene11749_All	2361	3.68	3.43	2.41	5.36	4.96	5.83	5.21	2.18	8.39	-9.94E-02	6.21E-01		-6.08E-01	2.84E-03		-1.12E-01	5.54E-01		1.23E-01	4.99E-01		-1.26E+00	2.11E-16	Down	6.86E-01	3.38E-10		Unigene11749_All//gi|12043533|emb|CAC19789.1|//1.00E-163//MYB-like DNA-binding protein [Catharanthus roseus]	Unigene11749_All//2.00E-139//AT5G13820| TBP1 (TELOMERIC DNA BINDING PROTEIN 1); DNA binding / double-stranded telomeric DNA binding	Unigene11749_All//0.00E+00//TC158069	MYB-related			nr +
Unigene117491_All	663	0.00	0.00	0.24	0.15	0.10	0.07	1.66	2.27	7.69	null	null		7.91E+00	2.39E-01		-5.33E-01	8.18E-01		-1.14E+00	6.78E-01		4.53E-01	3.78E-01		2.21E+00	1.94E-15	Up	Unigene117491_All//gi|133925821|gb|ABO43663.1|//5.00E-12//calcineurin B-like protein 4-1 [Populus trichocarpa]	Unigene117491_All//1.00E-10//AT5G24270| SOS3 (SALT OVERLY SENSITIVE 3); calcium ion binding / calcium-dependent protein serine/threonine phosphatase	Unigene117491_All//4.00E-64//gi|73851161|gb|DT460181.1|DT460181		ko04650|Natural killer cell mediated cytotoxicity		nr -
Unigene117493_All	1629	0.00	0.00	0.00	0.03	0.00	0.00	1.25	0.33	1.59	null	null		null	null		-4.94E+00	5.59E-01		-4.94E+00	6.40E-01		-1.90E+00	1.90E-05	Down	3.52E-01	3.26E-01		Unigene117493_All//gi|3152598|gb|AAC17079.1|//1.00E-160//Contains similarity to C2-HC type zinc finger protein C.e-MyT1 gb|U67079 from C. elegans and to hypersensitivity-related gene 201 isolog T28M21.14 from A. thaliana BAC gb|AF002109 [Arabidopsis thaliana]	Unigene117493_All//8.00E-153//AT1G78990| transferase family protein	Unigene117493_All//0.00E+00//DW488553		ko00940|Phenylpropanoid biosynthesis//ko00945|Stilbenoid, diarylheptanoid and gingerol biosynthesis//ko00941|Flavonoid biosynthesis		nr -
Unigene117496_All	871	0.00	0.00	0.00	0.00	0.00	0.00	2.48	0.53	12.21	null	null		null	null		null	null		null	null		-2.23E+00	7.33E-07	Down	2.30E+00	7.31E-33	Up	Unigene117496_All//gi|171452356|dbj|BAG15869.1|//8.00E-45//ankyrin repeat protein [Bruguiera gymnorhiza]	Unigene117496_All//2.00E-39//AT4G10720| ankyrin repeat family protein	Unigene117496_All//0.00E+00//TC175880				nr -
Unigene117498_All	255	21.54	36.56	43.44	26.96	50.10	39.50	7.30	4.75	9.37	7.63E-01	5.32E-05		1.01E+00	1.99E-08	Up	8.94E-01	2.15E-09		5.51E-01	2.04E-03		-6.18E-01	1.52E-01		3.62E-01	3.36E-01		Unigene117498_All//gi|124360233|gb|ABN08246.1|//3.00E-20//Solute carrier family 35 member B3, related [Medicago truncatula]	Unigene117498_All//5.00E-21//AT1G12600| FUNCTIONS IN: molecular_function unknown; INVOLVED IN: biological_process unknown; LOCATED IN: cellular_component unknown; EXPRESSED IN: leaf whorl, petal, male gametophyte, flower, pollen tube; EXPRESSED DURING: L mature pollen stage, 4 anthesis, petal differentiation and expansion stage; CONTAINS InterPro DOMAIN/s: UAA transporter (InterPro:IPR013657); BEST Arabidopsis thaliana protein match is: UDP-galactose transporter-related (TAIR:AT4G23010.1); Has 943 Blast hits to 943 proteins in 196 species: Archae - 2; Bacteria - 55; Metazoa - 506; Fungi - 148; Plants - 112; Viruses - 0; Other Eukaryotes - 120 (source: NCBI BLink).	Unigene117498_All//7.00E-73//BQ403172				nr -

Unigene117499_All	335	0.31	0.00	0.00	0.30	0.00	0.00	0.13	0.25	3.69	-8.29E+00	3.74E-01		-8.29E+00	3.96E-01		-8.22E+00	3.15E-01		-8.22E+00	4.02E-01		9.83E-01	7.33E-01		4.87E+00	9.59E-08	Up	Unigene117499_All//gi|115441637|ref|NP_001045098.1|//1.00E-26//Os01g0899000 [Oryza sativa Japonica Group]<br>>gi|113534629|dbj|BAF07012.1|Os01g0899000 [Oryza sativa Japonica Group]	Unigene117499_All//1.00E-27//AT3G59350| serine/threonine protein kinase, putative	Unigene117499_All//5.00E-99//TC146290		ko04626|Plant-pathogen interaction		nr +
Unigene1175_All	1936	26.89	94.56	58.37	79.02	63.37	103.37	18.43	22.39	35.17	1.81E+00	0.00E+00	Up	1.12E+00	7.10E-95	Up	-3.18E-01	1.59E-17		3.88E-01	3.46E-29		2.81E-01	1.17E-04		9.32E-01	5.83E-55		Unigene1175_All//gi|15240523|ref|NP_199774.1|//0.00E+00//AAP6 (AMINO ACID PERMEASE 6); acidic amino acid transmembrane transporter/ amino acid transmembrane transporter/ neutral amino acid transmembrane transporter [Arabidopsis thaliana]<br>>gi|75220393|sp|P92934.1|AAP6_ARATH RecName: Full=Amino acid permease 6; AltName: Full=Amino acid transporter AAP6<br>>gi|1769887|emb|CAA65051.1| amino acid permease 6 [Arabidopsis thaliana]<br>>gi|8809686|dbj|BAA97227.1| amino acid permease 6 [Arabidopsis thaliana]<br>>gi|110738094|dbj|BAF00980.1| amino acid permease 6 [Arabidopsis thaliana]	Unigene1175_All//0.00E+00//AT5G49630| AAP6 (AMINO ACID PERMEASE 6); acidic amino acid transmembrane transporter/ amino acid transmembrane transporter/ neutral amino acid transmembrane transporter	Unigene1175_All//0.00E+00//TC145723			GO:0015171//GO:0015711//GO:0015801//GO:0031224//GO:0051707	nr +
Unigene11750_All	1324	20.43	5.13	6.92	26.90	12.51	5.15	19.48	1.96	13.45	-1.99E+00	2.12E-55	Down	-1.56E+00	3.86E-38	Down	-1.10E+00	1.30E-37	Down	-2.38E+00	4.01E-99	Down	-3.32E+00	4.63E-114	Down	-5.34E-01	1.11E-08		Unigene11750_All//gi|260424374|gb|ACX37656.1|//1.00E-108//homeodomain associated leucine zipper protein [Gossypium arboreum]	Unigene11750_All//3.00E-73//AT4G16780| ATHB-2 (ARABIDOPSIS THALIANA HOMEOBOX PROTEIN 2); DNA binding / protein homodimerization/ sequence-specific DNA binding / transcription factor	Unigene11750_All//0.00E+00//TC151568	HB		GO:0000902//GO:0001071//GO:0003677//GO:0006351//GO:0009639//GO:0009755//GO:0010468//GO:0030528//GO:0042802//GO:0043231//GO:0048513	nr +
Unigene117500_All	418	0.13	0.36	0.25	0.24	0.33	0.65	1.42	0.50	6.58	1.54E+00	5.32E-01		1.03E+00	7.04E-01		4.68E-01	8.77E-01		1.44E+00	4.09E-01		-1.50E+00	9.13E-02		2.22E+00	1.10E-08	Up	Unigene117500_All//gi|15919089|emb|CAC86996.1|//4.00E-15//ATP citrate lyase b-subunit [Lupinus albus]	Unigene117500_All//4.00E-15//AT1G10670| ACLA-1; ATP citrate synthase	Unigene117500_All//1.00E-60//BF274632		ko00020|Citrate cycle (TCA cycle)		nr +
Unigene117513_All	535	0.00	0.00	0.20	0.00	0.26	0.00	0.16	0.39	3.20	null	null		7.64E+00	4.01E-01		8.01E+00	3.19E-01		null	null		1.30E+00	4.48E-01		4.34E+00	5.93E-10	Up			Unigene117513_All//6.00E-07//DN817073				ESTscan +
Unigene117520_All	337	0.00	2.26	0.00	0.00	0.92	0.54	0.13	1.49	1.77	1.11E+01	1.77E-04	Up	null	null		9.84E+00	4.55E-02		9.07E+00	1.95E-01		3.57E+00	6.53E-03		3.82E+00	1.35E-03								
Unigene117526_All	276	0.00	0.00	0.00	0.00	0.00	0.00	0.00	0.00	2.17	null	null		null	null		null	null		null	null		null	null		1.11E+01	1.76E-04	Up	Unigene117526_All//gi|66271061|gb|AAY43808.1|//3.00E-09//Fb17 [Gossypium hirsutum]		Unigene117526_All//1.00E-49//gi|109890392|gb|DW519356.1|DW519356				nr +
Unigene117533_All	400	0.26	0.89	1.33	0.12	0.17	0.23	0.42	1.46	6.27	1.76E+00	2.18E-01		2.35E+00	5.88E-02		4.68E-01	9.60E-01		8.59E-01	7.95E-01		1.79E+00	5.30E-02		3.89E+00	2.29E-13	Up	Unigene117533_All//gi|297843964|ref|XP_002889863.1|//6.00E-24//S-locus lectin protein kinase family protein [Arabidopsis lyrata subsp. lyrata]<br>>gi|297335705|gb|EFH66122.1| S-locus lectin protein kinase family protein [Arabidopsis lyrata subsp. lyrata]	Unigene117533_All//1.00E-24//AT1G11300| ATP binding / carbohydrate binding / kinase/ protein kinase/ protein serine/threonine kinase/ protein tyrosine kinase/ sugar binding	Unigene117533_All//8.00E-160//gi|109861376|gb|DW490353.1|DW490353				nr -
Unigene117542_All	328	0.00	0.00	0.49	0.00	0.00	0.00	0.00	0.00	1.94	null	null		8.93E+00	2.39E-01		null	null		null	null		null	null		1.09E+01	9.35E-05	Up							
Unigene117544_All	304	0.00	0.00	0.00	0.00	0.00	0.00	0.42	5.36	0.79	null	null		null	null		null	null		null	null		3.68E+00	2.33E-08	Up	9.14E-01	5.42E-01								
Unigene117545_All	1458	0.00	0.00	0.00	0.07	0.02	0.12	0.17	0.20	8.69	null	null		null	null		-1.53E+00	5.87E-01		8.59E-01	6.96E-01		2.06E-01	8.68E-01		5.64E+00	1.18E-80	Up	Unigene117545_All//gi|12322008|gb|AAG51046.1|AC069473_8//2.00E-34//gypsy/Ty-3 retroelement polyprotein; 69905-74404 [Arabidopsis thaliana]<br>>gi|10998138|dbj|BAB03109.1| retroelement pol polyprotein [Arabidopsis thaliana]						nr -
Unigene11756_All	595	4.48	1.36	3.58	9.29	6.54	4.63	0.92	0.98	0.47	-1.72E+00	3.82E-05	Down	-3.25E-01	4.20E-01		-5.07E-01	3.93E-02		-1.01E+00	5.86E-05	Down	9.02E-02	9.27E-01		-9.79E-01	2.50E-01		Unigene11756_All//gi|82880418|gb|ABB92748.1|//2.00E-44//UDP-glucose glucosyltransferase [Fragaria x ananassa]	Unigene11756_All//2.00E-34//AT3G21790| UDP-glucoronosyl/UDP-glucosyl transferase family protein	Unigene11756_All//0.00E+00//gi|78337285|gb|DT557559.1|DT557559			GO:0016758	nr +
Unigene117560_All	371	0.00	0.27	0.00	0.00	0.00	0.00	0.34	0.45	5.91	8.09E+00	3.89E-01		null	null		null	null		null	null		3.98E-01	8.15E-01		4.11E+00	3.56E-12	Up							
Unigene117563_All	487	0.00	0.10	0.00	0.00	0.00	0.00	0.26	0.00	2.86	6.70E+00	6.19E-01		null	null		null	null		null	null		-8.03E+00	2.36E-01		3.46E+00	4.41E-07	Up	Unigene117563_All//gi|255560201|ref|XP_002521118.1|//4.00E-07//conserved hypothetical protein [Ricinus communis]<br>>gi|223539687|gb|EEF41269.1| conserved hypothetical protein [Ricinus communis]						nr +
Unigene117569_All	605	0.00	0.00	0.00	0.00	0.00	0.00	0.14	0.14	2.44	null	null		null	null		null	null		null	null		-1.66E-02	1.01E+00		4.12E+00	2.19E-08	Up							
Unigene11757_All	1766	28.59	18.99	20.96	19.04	19.13	15.34	5.70	6.22	1.62	-5.90E-01	3.11E-15		-4.48E-01	4.13E-09		6.70E-03	9.56E-01		-3.11E-01	7.20E-04		1.28E-01	4.81E-01		-1.81E+00	2.50E-23	Down	Unigene11757_All//gi|283362116|dbj|BAI65911.1|//1.00E-120//UDP-sugar:glycosyltransferase [Forsythia x intermedia]	Unigene11757_All//8.00E-101//AT3G21780| UGT71B6 (UDP-glucosyl transferase 71B6); UDP-glycosyltransferase/ abscisic acid glucosyltransferase/ transferase, transferring glycosyl groups	Unigene11757_All//0.00E+00//gi|109871404|gb|DW500380.1|DW500380			GO:0035251	nr +
Unigene117575_All	219	0.00	0.00	0.00	0.00	0.00	0.00	12.36	9.54	0.73	null	null		null	null		null	null		null	null		-3.73E-01	3.08E-01		-4.09E+00	3.55E-15	Down	Unigene117575_All//gi|242070905|ref|XP_002450729.1|//1.00E-10//hypothetical protein SORBIDRAFT_05g016460 [Sorghum bicolor]<br>>gi|241936572|gb|EES09717.1| hypothetical protein SORBIDRAFT_05g016460 [Sorghum bicolor]		Unigene117575_All//2.00E-32//TC146753				nr +
Unigene11758_All	2012	3.41	2.42	1.48	10.67	5.77	3.28	1.37	0.62	0.53	-4.95E-01	2.79E-02		-1.20E+00	5.60E-07	Down	-8.88E-01	6.19E-16		-1.70E+00	1.34E-38	Down	-1.13E+00	9.60E-04	Down	-1.35E+00	7.11E-05	Down	Unigene11758_All//gi|283362116|dbj|BAI65911.1|//1.00E-118//UDP-sugar:glycosyltransferase [Forsythia x intermedia]	Unigene11758_All//2.00E-99//AT3G21780| UGT71B6 (UDP-glucosyl transferase 71B6); UDP-glycosyltransferase/ abscisic acid glucosyltransferase/ transferase, transferring glycosyl groups	Unigene11758_All//0.00E+00//TC170176			GO:0035251	nr -
Unigene117582_All	287	0.00	0.00	0.00	0.00	0.00	0.00	0.00	0.73	1.80	null	null		null	null		null	null		null	null		9.51E+00	7.86E-02		1.08E+01	6.14E-04	Up							
Unigene117584_All	279	0.00	0.00	0.00	0.00	0.00	0.00	0.00	0.00	3.14	null	null		null	null		null	null		null	null		null	null		1.16E+01	2.05E-06	Up							
Unigene117585_All	511	0.00	0.10	0.00	0.10	0.40	0.35	0.00	0.00	2.81	6.63E+00	6.19E-01		null	null		2.05E+00	3.95E-01		1.86E+00	4.58E-01		null	null		1.15E+01	2.48E-10	Up	Unigene117585_All//gi|15242647|ref|NP_198848.1|//1.00E-12//DC1 domain-containing protein [Arabidopsis thaliana]<br>>gi|10178142|dbj|BAB11587.1| CHP-rich zinc finger protein-like [Arabidopsis thaliana]	Unigene117585_All//1.00E-14//AT1G58037| FUNCTIONS IN: molecular_function unknown; INVOLVED IN: intracellular signaling cascade; LOCATED IN: cellular_component unknown; CONTAINS InterPro DOMAIN/s: Protein kinase C, phorbol ester/diacylglycerol binding (InterPro:IPR002219), DC1 (InterPro:IPR004146), C1-like (InterPro:IPR011424); BEST Arabidopsis thaliana protein match is: DC1 domain-containing protein (TAIR:AT5G45730.1); Has 1201 Blast hits to 455 proteins in 13 species: Archae - 0; Bacteria - 0; Metazoa - 3; Fungi - 0; Plants - 1193; Viruses - 0; Other Eukaryotes - 5 (source: NCBI BLink).	Unigene117585_All//7.00E-16//ES842244				nr -
Unigene117587_All	303	1.38	0.67	0.00	1.32	0.91	0.30	0.42	0.00	3.68	-1.05E+00	3.66E-01		-1.04E+01	1.56E-02		-5.33E-01	6.16E-01		-2.14E+00	1.26E-01		-8.71E+00	2.36E-01		3.14E+00	2.19E-05	Up							
Unigene117588_All	710	0.00	0.00	0.00	0.77	0.00	0.13	3.57	3.30	2.24	null	null		null	null		-9.59E+00	4.01E-04	Down	-2.60E+00	2.93E-02		-1.16E-01	7.57E-01		-6.71E-01	4.94E-02		Unigene117588_All//gi|224114718|ref|XP_002332307.1|//4.00E-08//predicted protein [Populus trichocarpa]<br>>gi|222832306|gb|EEE70783.1| predicted protein [Populus trichocarpa]						nr +
Unigene117590_All	276	215.92	673.39	296.93	627.37	535.74	692.34	204.97	120.84	196.45	1.64E+00	7.74E-287	Up	4.60E-01	4.79E-15		-2.28E-01	1.20E-10		1.42E-01	1.16E-04		-7.62E-01	4.85E-32		-6.12E-02	4.03E-01		Unigene117590_All//gi|56121699|gb|AAV74186.1|//2.00E-13//metallothionein-like protein [Gossypium hirsutum]	Unigene117590_All//4.00E-05//AT3G09390| MT2A (METALLOTHIONEIN 2A); copper ion binding	Unigene117590_All//7.00E-67//TC156098			GO:0043169	nr +
Unigene117603_All	457	0.00	0.78	0.12	0.22	0.00	0.20	0.37	1.10	5.32	9.60E+00	2.46E-02		6.86E+00	6.38E-01		-7.77E+00	3.15E-01		-1.41E-01	9.51E-01		1.57E+00	1.18E-01		3.84E+00	7.47E-13	Up	Unigene117603_All//gi|18411945|ref|NP_565179.1|//4.00E-06//VQ motif-containing protein [Arabidopsis thaliana]<br>>gi|17979514|gb|AAL50092.1| At1g78410/F3F9_27 [Arabidopsis thaliana]<br>>gi|20857141|gb|AAM26702.1| At1g78410/F3F9_27 [Arabidopsis thaliana]<br>>gi|55982672|gb|AAV69753.1| tobacco rattle virus-induced protein variant 1 [Arabidopsis thaliana]	Unigene117603_All//1.00E-07//AT1G78410| VQ motif-containing protein	Unigene117603_All//0.00E+00//TC165064			GO:0006950	nr -
Unigene117604_All	621	0.00	0.00	0.00	0.00	0.11	0.00	0.75	0.61	3.34	null	null		null	null		6.79E+00	6.12E-01		null	null		-3.06E-01	7.36E-01		2.16E+00	1.54E-06	Up	Unigene117604_All//gi|82568711|dbj|BAE48668.1|//3.00E-45//Alcohol acyl-transferase [Prunus mume]	Unigene117604_All//5.00E-44//AT5G48930| HCT (HYDROXYCINNAMOYL-COA SHIKIMATE/QUINATE HYDROXYCINNAMOYL TRANSFERASE); quinate O-hydroxycinnamoyltransferase/ shikimate O-hydroxycinnamoyltransferase/ transferase	Unigene117604_All//4.00E-150//TC146659		ko00940|Phenylpropanoid biosynthesis//ko00945|Stilbenoid, diarylheptanoid and gingerol biosynthesis//ko00941|Flavonoid biosynthesis	GO:0016747	nr +
Unigene117608_All	375	0.00	0.41	0.00	0.13	0.00	0.12	0.00	1.23	2.44	8.66E+00	2.36E-01		null	null		-7.05E+00	5.59E-01		-1.41E-01	9.55E-01		1.03E+01	1.94E-03		1.13E+01	1.08E-06	Up							
Unigene11761_All	862	3.76	34.86	2.10	10.17	33.92	5.71	12.80	10.57	170.72	3.21E+00	1.28E-104	Up	-8.41E-01	1.97E-02		1.74E+00	8.49E-58	Up	-8.33E-01	1.27E-05		-2.76E-01	8.75E-02		3.74E+00	0.00E+00	Up	Unigene11761_All//gi|171452368|dbj|BAG15875.1|//4.00E-52//WRKY transcription factor [Bruguiera gymnorhiza]	Unigene11761_All//2.00E-31//AT4G23810| WRKY53; DNA binding / protein binding / transcription activator/ transcription factor	Unigene11761_All//0.00E+00//gi|73864993|gb|DT467732.1|DT467732	WRKY		GO:0001071//GO:0003677//GO:0010468//GO:0043231	nr -
Unigene117610_All	336	0.16	0.00	0.00	2.08	1.64	2.15	7.80	2.49	5.69	-7.28E+00	6.16E-01		-7.28E+00	6.28E-01		-3.40E-01	6.80E-01		5.12E-02	9.48E-01		-1.65E+00	9.48E-06	Down	-4.55E-01	1.85E-01		Unigene117610_All//gi|224113571|ref|XP_002332539.1|//4.00E-15//BED finger-nbs-lrr resistance protein [Populus trichocarpa]<br>>gi|222832683|gb|EEE71160.1| BED finger-nbs-lrr resistance protein [Populus trichocarpa]	Unigene117610_All//8.00E-10//AT1G12220| RPS5 (RESISTANT TO P. SYRINGAE 5); nucleotide binding	Unigene117610_All//6.00E-74//DT463318		ko04626|Plant-pathogen interaction	GO:0005488	nr +

<table>
<tr><td>Unigene117614_All</td><td>252</td><td>0.00</td><td>0.20</td><td>0.00</td><td>0.79</td><td>1.09</td><td>0.36</td><td>1.17</td><td>1.00</td><td>8.06</td><td>7.65E+00</td><td>6.19E-01</td><td></td><td>null</td><td>null</td><td></td><td>4.67E-01</td><td>8.17E-01</td><td></td><td>-1.14E+00</td><td>5.69E-01</td><td></td><td>-2.39E-01</td><td>8.40E-01</td><td></td><td>2.78E+00</td><td>2.59E-08</td><td>Up</td><td>Unigene117614_All//gi|217070972|gb|ACJ83846.1|//5.00E-10//unknown [Medicago truncatula]</td><td></td><td>Unigene117614_All//7.00E-76//TC162083</td><td></td><td></td><td></td><td>nr -</td></tr>
<tr><td>Unigene117615_All</td><td>219</td><td>0.00</td><td>0.00</td><td>0.00</td><td>0.00</td><td>0.00</td><td>0.00</td><td>9.65</td><td>0.57</td><td>3.27</td><td>null</td><td>null</td><td></td><td>null</td><td>null</td><td></td><td>null</td><td>null</td><td></td><td>null</td><td>null</td><td></td><td>-4.08E+00</td><td>1.66E-11</td><td>Down</td><td>-1.56E+00</td><td>1.14E-04</td><td>Down</td><td></td><td></td><td>Unigene117615_All//4.00E-37//gi|73850352|gb|DT459372.1|DT459372</td><td></td><td></td><td></td><td></td></tr>
<tr><td>Unigene117617_All</td><td>841</td><td>0.31</td><td>0.24</td><td>0.76</td><td>0.53</td><td>0.57</td><td>0.75</td><td>0.50</td><td>0.00</td><td>7.06</td><td>-3.68E-01</td><td>7.79E-01</td><td></td><td>1.29E+00</td><td>1.87E-01</td><td></td><td>1.05E-01</td><td>9.55E-01</td><td></td><td>4.96E-01</td><td>6.37E-01</td><td></td><td>-8.97E+00</td><td>3.25E-03</td><td></td><td>3.81E+00</td><td>2.30E-30</td><td>Up</td><td>Unigene117617_All//gi|56121882|gb|AAV74222.1|//9.00E-19//At5g40700 [Arabidopsis thaliana] >gi|63003888|gb|AAY25473.1|At5g40700 [Arabidopsis thaliana]</td><td>Unigene117617_All//3.00E-21//AT3G27350| unknown protein</td><td>Unigene117617_All//4.00E-86//DT462327</td><td></td><td></td><td>GO:0016020</td><td>nr +</td></tr>
<tr><td>Unigene11762_All</td><td>439</td><td>0.60</td><td>4.16</td><td>0.00</td><td>3.74</td><td>6.67</td><td>4.73</td><td>2.50</td><td>1.62</td><td>17.70</td><td>2.80E+00</td><td>4.13E-06</td><td>Up</td><td>-9.22E+00</td><td>8.58E-02</td><td></td><td>8.32E-01</td><td>2.07E-02</td><td></td><td>3.38E-01</td><td>4.92E-01</td><td></td><td>-6.30E-01</td><td>2.93E-01</td><td></td><td>2.82E+00</td><td>3.07E-30</td><td>Up</td><td>Unigene11762_All//gi|171452368|dbj|BAG15875.1|//6.00E-30//WRKY transcription factor [Bruguiera gymnorhiza]</td><td>Unigene11762_All//3.00E-26//AT4G11070| WRKY41; transcription factor</td><td>Unigene11762_All//2.00E-65//gi|73864993|gb|DT467732.1|DT467732</td><td>WRKY</td><td>ko04626|Plant-pathogen interaction</td><td>GO:0001071//GO:0003677//GO:0006351//GO:0009536//GO:0009743//GO:0010260//GO:0010468//GO:0010557//GO:0030528//GO:0042742</td><td>nr -</td></tr>
<tr><td>Unigene117620_All</td><td>237</td><td>0.00</td><td>0.00</td><td>0.00</td><td>0.00</td><td>0.00</td><td>0.00</td><td>0.00</td><td>0.00</td><td>2.19</td><td>null</td><td>null</td><td></td><td>null</td><td>null</td><td></td><td>null</td><td>null</td><td></td><td>null</td><td>null</td><td></td><td>null</td><td>null</td><td></td><td>1.11E+01</td><td>6.14E-04</td><td>Up</td><td></td><td></td><td></td><td></td><td></td><td></td><td></td></tr>
<tr><td>Unigene117629_All</td><td>237</td><td>0.00</td><td>0.00</td><td>0.00</td><td>0.00</td><td>0.00</td><td>0.00</td><td>0.00</td><td>0.00</td><td>2.86</td><td>null</td><td>null</td><td></td><td>null</td><td>null</td><td></td><td>null</td><td>null</td><td></td><td>null</td><td>null</td><td></td><td>null</td><td>null</td><td></td><td>1.15E+01</td><td>4.97E-05</td><td>Up</td><td></td><td></td><td></td><td></td><td></td><td></td><td></td></tr>
<tr><td>Unigene11763_All</td><td>1029</td><td>30.05</td><td>21.62</td><td>22.05</td><td>13.36</td><td>19.98</td><td>14.05</td><td>5.10</td><td>2.88</td><td>13.63</td><td>-4.75E-01</td><td>8.90E-07</td><td></td><td>-4.47E-01</td><td>7.79E-06</td><td></td><td>5.80E-01</td><td>2.74E-07</td><td></td><td>7.20E-02</td><td>7.01E-01</td><td></td><td>-8.21E-01</td><td>4.17E-04</td><td></td><td>1.42E+00</td><td>1.17E-22</td><td>Up</td><td>Unigene11763_All//gi|18700139|gb|AAL77681.1|//2.00E-52//At1g64350/F15H21_2 [Arabidopsis thaliana] >gi|88193800|gb|ABD42989.1|At1g64355 [Arabidopsis thaliana]</td><td>Unigene11763_All//1.00E-53//AT1G64355| unknown protein</td><td>Unigene11763_All//0.00E+00//TC140916</td><td></td><td></td><td>GO:0009536</td><td>nr -</td></tr>
<tr><td>Unigene117635_All</td><td>230</td><td>0.00</td><td>33.93</td><td>0.69</td><td>0.00</td><td>0.00</td><td>0.20</td><td>0.00</td><td>0.00</td><td>0.35</td><td>1.51E+01</td><td>1.26E-44</td><td>Up</td><td>9.44E+00</td><td>2.39E-01</td><td></td><td>null</td><td>null</td><td></td><td>7.62E+00</td><td>6.99E-01</td><td></td><td>null</td><td>null</td><td></td><td>8.44E+00</td><td>4.05E-01</td><td></td><td></td><td></td><td>Unigene117635_All//2.00E-29//DN800060</td><td></td><td></td><td></td><td>ESTscan +</td></tr>
<tr><td>Unigene117637_All</td><td>764</td><td>0.00</td><td>0.00</td><td>0.00</td><td>0.00</td><td>0.00</td><td>0.00</td><td>0.66</td><td>0.27</td><td>21.43</td><td>null</td><td>null</td><td></td><td>null</td><td>null</td><td></td><td>null</td><td>null</td><td></td><td>null</td><td>null</td><td></td><td>-1.28E+00</td><td>1.86E-01</td><td></td><td>5.01E+00</td><td>1.11E-98</td><td>Up</td><td></td><td></td><td>Unigene117637_All//2.00E-60//TC141760</td><td></td><td></td><td></td><td></td></tr>
<tr><td>Unigene11764_All</td><td>3750</td><td>3.36</td><td>35.18</td><td>3.45</td><td>7.44</td><td>21.97</td><td>8.05</td><td>8.17</td><td>8.46</td><td>78.23</td><td>3.39E+00</td><td>0.00E+00</td><td>Up</td><td>3.73E-02</td><td>8.31E-01</td><td></td><td>1.56E+00</td><td>2.95E-140</td><td>Up</td><td>1.13E-01</td><td>3.40E-01</td><td></td><td>4.95E-02</td><td>6.21E-01</td><td></td><td>3.26E+00</td><td>0.00E+00</td><td>Up</td><td>Unigene11764_All//gi|259121389|gb|ACV92014.1|//1.00E-125//WRKY transcription factor 12 [(Populus tomentosa x P. bolleana) x P. tomentosa]</td><td>Unigene11764_All//3.00E-60//AT4G11070| WRKY41; transcription factor</td><td>Unigene11764_All//0.00E+00//TC150705</td><td>WRKY</td><td>ko04626|Plant-pathogen interaction</td><td>GO:0003677//GO:0010467</td><td>nr +</td></tr>
<tr><td>Unigene117640_All</td><td>960</td><td>0.00</td><td>0.21</td><td>0.22</td><td>0.00</td><td>0.00</td><td>0.00</td><td>0.97</td><td>0.96</td><td>5.81</td><td>7.72E+00</td><td>1.37E-01</td><td></td><td>7.79E+00</td><td>1.41E-01</td><td></td><td>null</td><td>null</td><td></td><td>null</td><td>null</td><td></td><td>-1.66E-02</td><td>1.02E+00</td><td></td><td>2.58E+00</td><td>5.31E-20</td><td>Up</td><td></td><td></td><td></td><td></td><td></td><td></td><td></td></tr>
<tr><td>Unigene117654_All</td><td>247</td><td>0.00</td><td>0.41</td><td>0.00</td><td>0.00</td><td>0.00</td><td>0.00</td><td>0.51</td><td>0.17</td><td>4.19</td><td>8.68E+00</td><td>3.89E-01</td><td></td><td>null</td><td>null</td><td></td><td>null</td><td>null</td><td></td><td>null</td><td>null</td><td></td><td>-1.60E+00</td><td>5.35E-01</td><td></td><td>3.03E+00</td><td>6.50E-05</td><td>Up</td><td></td><td></td><td>Unigene117654_All//5.00E-123//FL577603</td><td></td><td></td><td></td><td></td></tr>
<tr><td>Unigene11767_All</td><td>2648</td><td>6.82</td><td>14.79</td><td>7.26</td><td>13.54</td><td>10.61</td><td>20.89</td><td>16.81</td><td>37.49</td><td>30.06</td><td>1.12E+00</td><td>3.60E-34</td><td>Up</td><td>9.08E-02</td><td>5.52E-01</td><td></td><td>-3.52E-01</td><td>2.32E-05</td><td></td><td>6.25E-01</td><td>1.66E-19</td><td></td><td>1.16E+00</td><td>8.73E-111</td><td>Up</td><td>8.38E-01</td><td>9.88E-54</td><td></td><td>Unigene11767_All//gi|15229641|ref|NP_188473.1|//0.00E+00//AtALMT9 (aluminum-activated malate transporter 9); anion channel [Arabidopsis thaliana]</td><td>Unigene11767_All//6.00E-178//AT3G18440| AtALMT9 (aluminum-activated malate transporter 9); anion channel</td><td>Unigene11767_All//0.00E+00//TC154916</td><td></td><td></td><td>GO:0000325//GO:0010038//GO:0015740</td><td>nr +</td></tr>
<tr><td>Unigene117671_All</td><td>512</td><td>32.90</td><td>43.15</td><td>36.82</td><td>10.90</td><td>14.19</td><td>24.43</td><td>11.40</td><td>9.80</td><td>3.19</td><td>3.91E-01</td><td>8.43E-04</td><td></td><td>1.62E-01</td><td>2.64E-01</td><td></td><td>3.81E-01</td><td>8.47E-02</td><td></td><td>1.17E+00</td><td>7.71E-13</td><td>Up</td><td>-2.18E-01</td><td>3.80E-01</td><td></td><td>-1.84E+00</td><td>3.90E-14</td><td>Down</td><td>Unigene117671_All//gi|161778780|gb|ABX79341.1|//1.00E-59//cysteine protease [Vitis vinifera]</td><td>Unigene117671_All//5.00E-54//AT4G39090| RD19 (RESPONSIVE TO DEHYDRATION 19); cysteine-type endopeptidase/ cysteine-type peptidase</td><td>Unigene117671_All//1.00E-146//gi|164285445|gb|ES796421.1|ES796421</td><td></td><td></td><td>GO:0070011</td><td>nr -</td></tr>
<tr><td>Unigene117681_All</td><td>269</td><td>0.00</td><td>0.00</td><td>0.00</td><td>0.00</td><td>0.00</td><td>0.00</td><td>0.00</td><td>0.16</td><td>7.70</td><td>null</td><td>null</td><td></td><td>null</td><td>null</td><td></td><td>null</td><td>null</td><td></td><td>null</td><td>null</td><td></td><td>7.28E+00</td><td>6.37E-01</td><td></td><td>1.29E+01</td><td>7.51E-15</td><td>Up</td><td></td><td></td><td></td><td></td><td></td><td></td><td></td></tr>
<tr><td>Unigene117687_All</td><td>245</td><td>0.00</td><td>0.00</td><td>0.00</td><td>0.00</td><td>0.00</td><td>0.00</td><td>0.17</td><td>0.00</td><td>2.93</td><td>null</td><td>null</td><td></td><td>null</td><td>null</td><td></td><td>null</td><td>null</td><td></td><td>null</td><td>null</td><td></td><td>-7.43E+00</td><td>6.61E-01</td><td></td><td>4.08E+00</td><td>2.41E-04</td><td>Up</td><td>Unigene117687_All//gi|242070905|ref|XP_002450729.1|//3.00E-08//hypothetical protein SORBIDRAFT_05g016460 [Sorghum bicolor] >gi|241936572|gb|EES09717.1|hypothetical protein SORBIDRAFT_05g016460 [Sorghum bicolor]</td><td></td><td>Unigene117687_All//3.00E-22//TC146753</td><td></td><td></td><td></td><td>nr +</td></tr>
<tr><td>Unigene11770_All</td><td>1286</td><td>6.47</td><td>6.90</td><td>11.43</td><td>16.19</td><td>16.39</td><td>21.35</td><td>21.63</td><td>9.07</td><td>12.73</td><td>9.21E-02</td><td>6.48E-01</td><td></td><td>8.21E-01</td><td>6.37E-08</td><td></td><td>1.74E-02</td><td>9.11E-01</td><td></td><td>3.99E-01</td><td>8.56E-05</td><td></td><td>-1.25E+00</td><td>7.02E-36</td><td>Down</td><td>-7.65E-01</td><td>9.19E-17</td><td></td><td>Unigene11770_All//gi|209978718|gb|ACJ04704.1|//1.00E-91//hexokinase 1 [Cucumis melo]</td><td>Unigene11770_All//1.00E-74//AT4G29130| HXK1 (HEXOKINASE 1); ATP binding / fructokinase/ glucokinase/ hexokinase</td><td>Unigene11770_All//0.00E+00//gi|78324449|gb|DT544723.1|DT544723</td><td></td><td>ko00500|Starch and sucrose metabolism//ko00010|Glycolysis / Gluconeogenesis//ko00520|Amino sugar and nucleotide sugar metabolism//ko00051|Fructose and mannose metabolism//ko00052|Galactose metabolism</td><td>GO:0004396//GO:0006007//GO:0008219//GO:0009527//GO:0009757//GO:0031224//GO:0032559//GO:0046914</td><td>nr -</td></tr>
<tr><td>Unigene117701_All</td><td>228</td><td>0.00</td><td>0.00</td><td>0.00</td><td>0.00</td><td>0.30</td><td>0.20</td><td>0.00</td><td>2.57</td><td>4.72</td><td>null</td><td>null</td><td></td><td>null</td><td>null</td><td></td><td>8.24E+00</td><td>6.12E-01</td><td></td><td>7.63E+00</td><td>6.99E-01</td><td></td><td>1.13E+01</td><td>2.81E-04</td><td>Up</td><td>1.22E+01</td><td>8.30E-08</td><td>Up</td><td></td><td></td><td></td><td></td><td></td><td></td><td></td></tr>
<tr><td>Unigene11771_All</td><td>837</td><td>17.19</td><td>9.20</td><td>8.14</td><td>20.35</td><td>12.67</td><td>18.13</td><td>29.25</td><td>17.98</td><td>9.23</td><td>-9.02E-01</td><td>2.04E-09</td><td></td><td>-1.08E+00</td><td>7.98E-12</td><td>Down</td><td>-6.84E-01</td><td>2.86E-08</td><td></td><td>-1.67E-01</td><td>2.82E-01</td><td></td><td>-7.02E-01</td><td>1.56E-12</td><td></td><td>-1.66E+00</td><td>2.20E-49</td><td>Down</td><td>Unigene11771_All//gi|297828127|ref|XP_002881946.1|//6.00E-66//dihydrodipicolinate reductase family protein [Arabidopsis lyrata subsp. lyrata] >gi|297327785|gb|EFH58205.1|dihydrodipicolinate reductase family protein [Arabidopsis lyrata subsp. lyrata]</td><td>Unigene11771_All//3.00E-67//AT2G44040| dihydrodipicolinate reductase family protein</td><td>Unigene11771_All//0.00E+00//TC135930</td><td></td><td>ko00300|Lysine biosynthesis</td><td>GO:0009089//GO:0009536//GO:0016628</td><td>nr -</td></tr>
<tr><td>Unigene117718_All</td><td>256</td><td>0.00</td><td>0.00</td><td>0.83</td><td>0.19</td><td>0.40</td><td>0.18</td><td>1.49</td><td>3.92</td><td>6.69</td><td>null</td><td>null</td><td></td><td>9.70E+00</td><td>1.41E-01</td><td></td><td>1.05E+00</td><td>8.09E-01</td><td></td><td>-1.42E-01</td><td>9.55E-01</td><td></td><td>1.40E+00</td><td>2.82E-02</td><td></td><td>2.17E+00</td><td>1.39E-05</td><td>Up</td><td></td><td></td><td>Unigene117718_All//7.00E-42//gi|109849493|gb|DW478472.1|DW478472</td><td></td><td></td><td></td><td></td></tr>
<tr><td>Unigene117719_All</td><td>676</td><td>0.00</td><td>0.15</td><td>0.00</td><td>0.15</td><td>0.00</td><td>0.00</td><td>0.38</td><td>0.06</td><td>5.07</td><td>7.23E+00</td><td>3.89E-01</td><td></td><td>null</td><td>null</td><td></td><td>-7.20E+00</td><td>3.15E-01</td><td></td><td>-7.20E+00</td><td>4.02E-01</td><td></td><td>-2.60E+00</td><td>1.44E-01</td><td></td><td>3.76E+00</td><td>1.41E-17</td><td>Up</td><td>Unigene117719_All//gi|224069196|ref|XP_002326298.1|//7.00E-45//AP2/ERF domain-containing transcription factor [Populus trichocarpa] >gi|148372128|gb|ABQ62995.1| RAP2-like protein [Populus trichocarpa] >gi|222833491|gb|EEE71968.1|AP2/ERF domain-containing transcription factor [Populus trichocarpa]</td><td>Unigene117719_All//2.00E-36//AT1G19210| AP2 domain-containing transcription factor, putative</td><td>Unigene117719_All//6.00E-20//gi|78324292|gb|DT544566.1|DT544566</td><td>AP2-EREBP</td><td></td><td>GO:0001071//GO:0006351//GO:0006950//GO:0010468//GO:0043231</td><td>nr +</td></tr>
<tr><td>Unigene11773_All</td><td>1016</td><td>1.60</td><td>1.90</td><td>5.50</td><td>4.51</td><td>5.05</td><td>5.16</td><td>4.04</td><td>5.14</td><td>1.49</td><td>2.48E-01</td><td>6.22E-01</td><td></td><td>1.79E+00</td><td>5.79E-10</td><td>Up</td><td>1.63E-01</td><td>6.04E-01</td><td></td><td>1.93E-01</td><td>5.28E-01</td><td></td><td>3.49E-01</td><td>1.53E-01</td><td></td><td>-1.44E+00</td><td>1.99E-07</td><td>Down</td><td>Unigene11773_All//gi|124359162|gb|ABN05685.1|//5.00E-12//RNA-directed DNA polymerase (Reverse transcriptase) [Medicago truncatula]</td><td></td><td>Unigene11773_All//0.00E+00//gi|21095789|gb|BQ408102.1|BQ408102</td><td></td><td></td><td></td><td>nr -</td></tr>
<tr><td>Unigene117736_All</td><td>476</td><td>0.44</td><td>1.49</td><td>0.22</td><td>1.36</td><td>1.01</td><td>1.33</td><td>1.07</td><td>2.72</td><td>4.60</td><td>1.76E+00</td><td>5.61E-02</td><td></td><td>-9.75E-01</td><td>6.21E-01</td><td></td><td>-4.26E-01</td><td>6.09E-01</td><td></td><td>-3.46E-02</td><td>9.53E-01</td><td></td><td>1.35E+00</td><td>1.28E-02</td><td></td><td>2.11E+00</td><td>1.02E-06</td><td>Up</td><td>Unigene117736_All//gi|145335397|ref|NP_172597.2|//3.00E-33//ATP binding / carbohydrate binding / kinase/ protein kinase/ protein serine/threonine kinase/ protein tyrosine kinase/ sugar binding [Arabidopsis thaliana]</td><td>Unigene117736_All//1.00E-30//AT1G11300| ATP binding / carbohydrate binding / kinase/ protein kinase/ protein serine/threonine kinase/ protein tyrosine kinase/ sugar binding</td><td>Unigene117736_All//0.00E+00//CO081719</td><td></td><td>ko04626|Plant-pathogen interaction</td><td></td><td>nr +</td></tr>
<tr><td>Unigene117740_All</td><td>505</td><td>0.73</td><td>0.80</td><td>0.00</td><td>0.20</td><td>0.41</td><td>0.18</td><td>0.50</td><td>0.83</td><td>6.63</td><td>1.47E-01</td><td>8.98E-01</td><td></td><td>-9.50E+00</td><td>2.79E-02</td><td></td><td>1.05E+00</td><td>6.13E-01</td><td></td><td>-1.41E-01</td><td>9.51E-01</td><td></td><td>7.20E-01</td><td>5.07E-01</td><td></td><td>3.72E+00</td><td>4.55E-17</td><td>Up</td><td></td><td></td><td></td><td></td><td></td><td></td><td></td></tr>
<tr><td>Unigene117745_All</td><td>1036</td><td>0.00</td><td>0.00</td><td>0.00</td><td>0.10</td><td>0.07</td><td>0.17</td><td>1.43</td><td>0.16</td><td>3.65</td><td>null</td><td>null</td><td></td><td>null</td><td>null</td><td></td><td>-5.33E-01</td><td>8.18E-01</td><td></td><td>8.58E-01</td><td>6.96E-01</td><td></td><td>-3.15E+00</td><td>8.54E-07</td><td>Down</td><td>1.35E+00</td><td>2.45E-06</td><td>Up</td><td>Unigene117745_All//gi|226507536|ref|NP_001148133.1|//1.00E-07//WAK53a - OsWAK receptor-like protein kinase [Zea mays] >gi|195616018|gb|ACG29839.1|WAK53a - OsWAK receptor-like protein kinase [Zea mays]</td><td>Unigene117745_All//1.00E-08//AT1G16090| WAKL7 (wall associated kinase-like 7); protein serine/threonine kinase</td><td>Unigene117745_All//7.00E-178//TC169886</td><td></td><td></td><td>GO:0016301</td><td>nr +</td></tr>
<tr><td>Unigene117749_All</td><td>1580</td><td>0.00</td><td>0.00</td><td>0.00</td><td>0.00</td><td>0.00</td><td>0.00</td><td>0.00</td><td>0.05</td><td>3.05</td><td>null</td><td>null</td><td></td><td>null</td><td>null</td><td></td><td>null</td><td>null</td><td></td><td>null</td><td>null</td><td></td><td>5.73E+00</td><td>4.03E-01</td><td></td><td>1.16E+01</td><td>1.69E-34</td><td>Up</td><td>Unigene117749_All//gi|38344889|emb|CAD41912.2|//1.00E-133//OSJNBa0033G05.13 [Oryza sativa (japonica cultivar-group)]</td><td>Unigene117749_All//2.00E-21//ATMG00300| hypothetical protein</td><td>Unigene117749_All//3.00E-48//FL576972</td><td></td><td></td><td></td><td>nr +</td></tr>
<tr><td>Unigene117750_All</td><td>238</td><td>11.21</td><td>1.70</td><td>2.01</td><td>10.47</td><td>10.85</td><td>6.07</td><td>0.18</td><td>1.05</td><td>0.67</td><td>-2.72E+00</td><td>1.79E-08</td><td>Down</td><td>-2.48E+00</td><td>2.65E-07</td><td>Down</td><td>5.23E-02</td><td>9.16E-01</td><td></td><td>-7.85E-01</td><td>5.15E-02</td><td></td><td>2.57E+00</td><td>1.53E-01</td><td></td><td>1.91E+00</td><td>3.78E-01</td><td></td><td>Unigene117750_All//gi|48310189|gb|AAT41771.1|//1.00E-09//At5g42680 [Arabidopsis thaliana] >gi|52218794|gb|AAU29467.1|At5g42680 [Arabidopsis thaliana]</td><td>Unigene117750_All//5.00E-11//AT5G42680| unknown protein</td><td>Unigene117750_All//8.00E-57//ES820721</td><td></td><td></td><td></td><td>nr +</td></tr>
<tr><td>Unigene117751_All</td><td>868</td><td>0.12</td><td>0.00</td><td>0.00</td><td>0.00</td><td>0.08</td><td>0.00</td><td>0.54</td><td>0.34</td><td>2.16</td><td>-6.91E+00</td><td>3.74E-01</td><td></td><td>-6.91E+00</td><td>3.96E-01</td><td></td><td>6.31E+00</td><td>6.12E-01</td><td></td><td>null</td><td>null</td><td></td><td>-6.69E-01</td><td>5.09E-01</td><td></td><td>2.01E+00</td><td>1.50E-05</td><td>Up</td><td>Unigene117751_All//gi|15236937|ref|NP_195254.1|//2.00E-46//zinc finger (C2H2 type) family protein [Arabidopsis thaliana] >gi|91806770|gb|ABE66112.1| zinc finger family protein [Arabidopsis thaliana]</td><td>Unigene117751_All//6.00E-42//AT4G35280| zinc finger (C2H2 type) family protein</td><td></td><td>C2H2</td><td></td><td>GO:0005488//GO:0010468</td><td>nr -</td></tr>
<tr><td>Unigene117758_All</td><td>708</td><td>0.00</td><td>0.00</td><td>0.00</td><td>0.00</td><td>0.00</td><td>0.00</td><td>0.06</td><td>0.06</td><td>0.90</td><td>null</td><td>null</td><td></td><td>null</td><td>null</td><td></td><td>null</td><td>null</td><td></td><td>null</td><td>null</td><td></td><td>-1.70E-02</td><td>1.01E+00</td><td></td><td>3.91E+00</td><td>7.66E-04</td><td>Up</td><td>Unigene117758_All//gi|224074401|ref|XP_002304364.1|//3.00E-81//4-coumarate-coa ligase [Populus trichocarpa] >gi|222841796|gb|EEE79343.1| 4-coumarate-coa ligase [Populus trichocarpa]</td><td>Unigene117758_All//1.00E-65//AT4G19010| 4-coumarate--CoA ligase family protein / 4-coumaroyl-CoA synthase family protein</td><td>Unigene117758_All//0.00E+00//BQ402740</td><td></td><td>ko00940|Phenylpropanoid biosynthesis//ko00360|Phenylalanine metabolism//ko00592|alpha-Linolenic acid metabolism//ko00130|Ubiquinone and other terpenoid-quinone biosynthesis</td><td>GO:0008152//GO:0016405//GO:0016491//GO:0031410</td><td>nr -</td></tr>
<tr><td>Unigene11776_All</td><td>1450</td><td>4.19</td><td>1.71</td><td>1.98</td><td>3.26</td><td>3.75</td><td>5.79</td><td>1.98</td><td>0.81</td><td>1.48</td><td>-1.29E+00</td><td>2.76E-07</td><td>Down</td><td>-1.08E+00</td><td>2.21E-05</td><td>Down</td><td>2.01E-01</td><td>5.12E-01</td><td></td><td>8.28E-01</td><td>2.59E-05</td><td></td><td>-1.30E+00</td><td>1.31E-04</td><td>Down</td><td>-4.18E-01</td><td>1.98E-01</td><td></td><td>Unigene11776_All//gi|224059034|ref|XP_002299685.1|//8.00E-49//heavy metal ATPase [Populus trichocarpa] >gi|222846943|gb|EEE84490.1| heavy metal ATPase [Populus trichocarpa]</td><td>Unigene11776_All//4.00E-33//AT4G33520| PAA1 (P-TYPE ATP-ASE 1); ATPase, coupled to transmembrane movement of ions, phosphorylative mechanism / copper ion transmembrane transporter</td><td>Unigene11776_All//6.00E-176//gi|164350307|gb|ES831374.1|ES831374</td><td></td><td></td><td>GO:0000166//GO:0000502//GO:0005375//GO:0009526//GO:0009532//GO:0010038//GO:0015662//GO:0016020//GO:0019941//GO:0022900</td><td>nr +</td></tr>
<tr><td>Unigene117769_All</td><td>453</td><td>0.92</td><td>4.81</td><td>1.76</td><td>1.65</td><td>3.72</td><td>3.59</td><td>1.03</td><td>0.46</td><td>2.29</td><td>2.38E+00</td><td>4.02E-06</td><td>Up</td><td>9.32E-01</td><td>2.56E-01</td><td></td><td>1.18E+00</td><td>2.09E-02</td><td></td><td>1.12E+00</td><td>3.47E-02</td><td></td><td>-1.15E+00</td><td>2.53E-01</td><td></td><td>1.16E+00</td><td>5.12E-02</td><td></td><td></td><td></td><td>Unigene117769_All//1.00E-97//TC169302</td><td></td><td></td><td></td><td></td></tr>
<tr><td>Unigene11779_All</td><td>3470</td><td>1.07</td><td>1.11</td><td>1.06</td><td>2.44</td><td>2.50</td><td>3.36</td><td>2.44</td><td>1.72</td><td>5.01</td><td>5.20E-02</td><td>8.75E-01</td><td></td><td>-1.57E-02</td><td>9.85E-01</td><td></td><td>3.52E-02</td><td>8.85E-01</td><td></td><td>4.60E-01</td><td>5.63E-03</td><td></td><td>-5.01E-01</td><td>4.93E-03</td><td></td><td>1.04E+00</td><td>2.18E-17</td><td>Up</td><td>Unigene11779_All//gi|108708012|gb|ABF95807.1|//1.00E-160//Lung seven transmembrane receptor family protein, expressed [Oryza sativa (japonica cultivar-group)]</td><td>Unigene11779_All//5.00E-140//AT1G61670| FUNCTIONS IN: molecular_function unknown; LOCATED IN: endomembrane system, integral to membrane; CONTAINS InterPro DOMAIN/s: Transmembrane receptor, eukaryota (InterPro:IPR009637); BEST Arabidopsis thaliana protein match is: unknown protein (TAIR:AT1G10980.1); Has 473 Blast hits to 471 proteins in 125 species: Archae - 0; Bacteria - 2; Metazoa - 242; Fungi - 99; Plants - 92; Viruses - 0; Other Eukaryotes - 38 (source: NCBI BLink).</td><td>Unigene11779_All//0.00E+00//DR454835</td><td></td><td></td><td></td><td>nr +</td></tr>
<tr><td>Unigene117794_All</td><td>282</td><td>0.00</td><td>0.18</td><td>0.76</td><td>0.18</td><td>0.00</td><td>0.00</td><td>0.15</td><td>0.59</td><td>3.25</td><td>7.49E+00</td><td>6.19E-01</td><td></td><td>9.56E+00</td><td>1.41E-01</td><td></td><td>-7.46E+00</td><td>5.59E-01</td><td></td><td>-7.46E+00</td><td>6.40E-01</td><td></td><td>1.98E+00</td><td>3.80E-01</td><td></td><td>4.44E+00</td><td>1.24E-05</td><td>Up</td><td></td><td></td><td>Unigene117794_All//8.00E-82//DW225774</td><td></td><td></td><td></td><td></td></tr>
<tr><td>Unigene117796_All</td><td>251</td><td>0.00</td><td>0.00</td><td>0.00</td><td>0.00</td><td>0.00</td><td>0.00</td><td>0.00</td><td>0.17</td><td>2.54</td><td>null</td><td>null</td><td></td><td>null</td><td>null</td><td></td><td>null</td><td>null</td><td></td><td>null</td><td>null</td><td></td><td>7.38E+00</td><td>6.37E-01</td><td></td><td>1.13E+01</td><td>9.35E-05</td><td>Up</td><td></td><td></td><td>Unigene117796_All//3.00E-07//BE055792</td><td></td><td></td><td></td><td></td></tr>
<tr><td>Unigene117808_All</td><td>601</td><td>0.52</td><td>0.00</td><td>0.00</td><td>0.08</td><td>0.00</td><td>0.00</td><td>0.63</td><td>0.14</td><td>4.97</td><td>-9.03E+00</td><td>3.66E-02</td><td></td><td>-9.03E+00</td><td>4.92E-02</td><td></td><td>-6.37E+00</td><td>5.59E-01</td><td></td><td>-6.37E+00</td><td>6.40E-01</td><td></td><td>-2.19E+00</td><td>8.76E-02</td><td></td><td>2.97E+00</td><td>1.11E-12</td><td>Up</td><td>Unigene117808_All//gi|224060299|ref|XP_002300130.1|//4.00E-26//glutaredoxin [Populus trichocarpa] >gi|222847388|gb|EEE84935.1|glutaredoxin [Populus trichocarpa]</td><td>Unigene117808_All//1.00E-18//AT5G14070| ROXY2; electron carrier/ protein disulfide oxidoreductase</td><td>Unigene117808_All//2.00E-164//DW503331</td><td></td><td></td><td>GO:0005488//GO:0006350//GO:0015036//GO:0019725//GO:0031410//GO:0043231//GO:0048449</td><td>nr +</td></tr>
<tr><td>Unigene117823_All</td><td>345</td><td>2.12</td><td>0.29</td><td>0.31</td><td>11.26</td><td>3.19</td><td>2.75</td><td>3.19</td><td>2.18</td><td>1.96</td><td>-2.85E+00</td><td>6.26E-03</td><td></td><td>-2.78E+00</td><td>1.05E-02</td><td></td><td>-1.82E+00</td><td>4.05E-09</td><td>Down</td><td>-2.03E+00</td><td>2.68E-09</td><td>Down</td><td>-5.47E-01</td><td>3.72E-01</td><td></td><td>-6.99E-01</td><td>2.11E-01</td><td></td><td></td><td></td><td>Unigene117823_All//4.00E-07//TC136732</td><td></td><td></td><td></td><td></td></tr>
<tr><td>Unigene117824_All</td><td>395</td><td>0.00</td><td>0.00</td><td>0.00</td><td>0.25</td><td>0.17</td><td>0.23</td><td>0.43</td><td>0.74</td><td>4.54</td><td>null</td><td>null</td><td></td><td>null</td><td>null</td><td></td><td>-5.32E-01</td><td>8.18E-01</td><td></td><td>-1.41E-01</td><td>9.51E-01</td><td></td><td>7.91E-01</td><td>5.63E-01</td><td></td><td>3.41E+00</td><td>8.04E-09</td><td>Up</td><td>Unigene117824_All//gi|224055915|ref|XP_002298700.1|//3.00E-29//cc-nbs-lrr resistance protein [Populus trichocarpa] >gi|222845958|gb|EEE83505.1| cc-nbs-lrr resistance protein [Populus trichocarpa]</td><td>Unigene117824_All//2.00E-22//AT1G12210| RFL1 (RPS5-like 1); ATP binding / protein binding</td><td></td><td></td><td>ko04626|Plant-pathogen interaction</td><td>GO:0000166//GO:0006950//GO:0016787//GO:0051707</td><td>nr -</td></tr>
</table>

Unigene11783_All	1785	0.85	1.87	0.92	2.79	5.15	4.76	11.04	2.41	6.14	1.14E+00	9.68E-04	Up	1.22E-01	8.08E-01		8.84E-01	8.77E-07		7.69E-01	8.21E-05		-2.19E+00	7.77E-56	Down	-8.47E-01	2.75E-14		Unigene11783_All//gi|22330658|ref|NP_177758.2|//1.00E-150//CYCB2;4 (CYCLIN B2;4); cyclin-dependent protein kinase regulator [Arabidopsis thaliana] >gi|147636286|sp|Q9SFW6.2|CCB24_ARATH RecName: Full=Cyclin-B2-4; AltName: Full=G2/mitotic-specific cyclin-B2-4; Short=CycB2;4	Unigene11783_All//6.00E-145//AT1G20610| CYCB2;3 (Cyclin B2;3); cyclin-dependent protein kinase regulator	Unigene11783_All//0.00E+00//DW501540			GO:0009987//GO:0043231	nr -
Unigene117831_All	352	0.45	0.00	0.00	0.14	0.39	0.00	0.48	0.59	3.06	-8.80E+00	2.16E-01		-8.80E+00	2.45E-01		1.47E+00	6.19E-01		-7.14E+00	6.40E-01		3.05E-01	8.36E-01		2.67E+00	1.42E-04	Up	Unigene117831_All//gi|10177558|dbj|BAB10837.1|//1.00E-13//retroelement pol polyprotein-like [Arabidopsis thaliana]					GO:0003676//GO:0004518//GO:0006260//GO:0034061//GO:0042254//GO:0044424//GO:0046914	nr -
Unigene117838_All	604	0.00	0.00	0.09	0.00	0.00	0.00	0.28	1.11	7.65	null	null		6.46E+00	6.38E-01		null	null		null	null		1.98E+00	2.26E-02		4.77E+00	1.93E-27	Up	Unigene117838_All//gi|147860110|emb|CAN82922.1|//9.00E-08//hypothetical protein [Vitis vinifera]		Unigene117838_All//6.00E-60//gi|109849827|gb|DW478806.1|DW478806			GO:0005488//GO:0016020//GO:0044237//GO:0044424	nr +
Unigene11784_All	2101	5.08	4.90	2.84	14.25	14.75	12.25	16.52	5.57	10.20	-5.34E-02	7.73E-01		-8.40E-01	3.87E-06		4.99E-02	6.95E-01		-2.18E-01	3.62E-02		-1.57E+00	3.26E-62	Down	-6.96E-01	1.14E-17		Unigene11784_All//gi|22330658|ref|NP_177758.2|//1.00E-160//CYCB2;4 (CYCLIN B2;4); cyclin-dependent protein kinase regulator [Arabidopsis thaliana] >gi|147636286|sp|Q9SFW6.2|CCB24_ARATH RecName: Full=Cyclin-B2-4; AltName: Full=G2/mitotic-specific cyclin-B2-4; Short=CycB2;4	Unigene11784_All//1.00E-147//AT1G76310| CYCB2;4 (CYCLIN B2;4); cyclin-dependent protein kinase regulator	Unigene11784_All//0.00E+00//TC175569			GO:0009987//GO:0043231	nr +
Unigene11785_All	934	3.14	0.33	0.34	20.69	11.61	4.21	10.32	3.54	1.62	-3.27E+00	6.04E-11	Down	-3.20E+00	3.48E-10	Down	-8.33E-01	5.85E-13		-2.30E+00	1.38E-51	Down	-1.55E+00	1.79E-17	Down	-2.67E+00	7.63E-36	Down	Unigene11785_All//gi|75100616|sp|O82318.1|Y2579_ARATH//8.00E-53//RecName: Full=Probably inactive leucine-rich repeat receptor-like protein kinase At2g25790; Flags: Precursor >gi|224589523|gb|ACN59295.1| leucine-rich repeat receptor-like protein kinase [Arabidopsis thaliana]	Unigene11785_All//6.00E-54//AT2G25790| leucine-rich repeat transmembrane protein kinase, putative	Unigene11785_All//0.00E+00//CO121142		ko04626|Plant-pathogen interaction	GO:0000166//GO:0004871//GO:0008152//GO:0016020//GO:0016301//GO:0016491	nr -
Unigene117851_All	822	0.13	0.12	0.26	0.18	0.42	0.11	1.59	0.10	0.34	-4.61E-02	9.90E-01		1.03E+00	5.92E-01		1.20E+00	4.26E-01		-7.26E-01	7.16E-01		-3.97E+00	3.35E-07	Down	-2.23E+00	1.19E-04	Down	Unigene117851_All//gi|18076679|emb|CAC84774.1|//9.00E-35//P70 protein [Nicotiana tabacum]	Unigene117851_All//2.00E-20//AT5G60210| LOCATED IN: plasma membrane; EXPRESSED IN: 22 plant structures; EXPRESSED DURING: 12 growth stages; BEST Arabidopsis thaliana protein match is: myosin heavy chain-related (TAIR:AT2G37080.1); Has 77946 Blast hits to 39215 proteins in 1793 species: Archae - 845; Bacteria - 8877; Metazoa - 39997; Fungi - 6049; Plants - 2988; Viruses - 248; Other Eukaryotes - 18942 (source: NCBI BLink).	Unigene117851_All//0.00E+00//DW225237			GO:0005515	nr +
Unigene117852_All	241	14.33	4.84	14.36	12.61	11.72	5.81	0.35	0.35	1.32	-1.57E+00	9.76E-06	Down	3.40E-03	9.90E-01		-1.06E-01	8.10E-01		-1.12E+00	1.74E-03		-1.65E-02	1.01E+00		1.91E+00	1.54E-01				Unigene117852_All//4.00E-43//TC139629				
Unigene117857_All	353	0.00	0.00	0.45	0.00	0.10	0.00	0.12	0.47	1.92	null	null		8.82E+00	2.39E-01		6.61E+00	8.34E-01		null	null		1.98E+00	3.80E-01		4.00E+00	4.30E-04	Up			Unigene117857_All//4.00E-47//gi|164335631|gb|ES806224.1|ES806224				
Unigene11786_All	3146	1.31	0.18	0.14	4.09	3.61	1.88	7.12	3.96	1.80	-2.89E+00	1.41E-13	Down	-3.28E+00	1.62E-14	Down	-1.78E-01	3.07E-01		-1.12E+00	1.46E-12	Down	-8.47E-01	1.17E-15		-1.99E+00	1.40E-57	Down	Unigene11786_All//gi|75100616|sp|O82318.1|Y2579_ARATH//0.00E+00//RecName: Full=Probably inactive leucine-rich repeat receptor-like protein kinase At2g25790; Flags: Precursor >gi|224589523|gb|ACN59295.1| leucine-rich repeat receptor-like protein kinase [Arabidopsis thaliana]	Unigene11786_All//0.00E+00//AT2G25790| leucine-rich repeat transmembrane protein kinase, putative	Unigene11786_All//0.00E+00//CO121141			GO:0003824//GO:0016020	nr -
Unigene11787_All	3239	3.18	1.56	2.15	21.07	18.69	19.84	28.63	49.72	10.54	-1.02E+00	1.97E-08	Down	-5.63E-01	2.33E-03		-1.73E-01	5.88E-03		-8.66E-02	2.46E-01		7.96E-01	1.22E-96		-1.44E+00	7.89E-150	Down	Unigene11787_All//gi|75100616|sp|O82318.1|Y2579_ARATH//0.00E+00//RecName: Full=Probably inactive leucine-rich repeat receptor-like protein kinase At2g25790; Flags: Precursor >gi|224589523|gb|ACN59295.1| leucine-rich repeat receptor-like protein kinase [Arabidopsis thaliana]	Unigene11787_All//0.00E+00//AT2G25790| leucine-rich repeat transmembrane protein kinase, putative	Unigene11787_All//0.00E+00//TC133977			GO:0004871//GO:0006464//GO:0007167//GO:0016301//GO:0016628//GO:0031224//GO:0032559	nr +
Unigene117872_All	505	0.00	0.00	0.00	0.00	0.00	0.00	0.08	0.00	1.42	null	null		null	null		null	null		null	null		-6.39E+00	6.61E-01		4.08E+00	2.41E-04	Up	Unigene117872_All//gi|76559894|tpe|CAI56334.1|//6.00E-13//TPA: isoflavone reductase-like protein 5 [Vitis vinifera]	Unigene117872_All//1.00E-09//AT4G39230| isoflavone reductase, putative			ko00640|Propanoate metabolism	GO:0043231	nr +
Unigene117873_All	575	0.18	0.00	0.00	1.21	0.84	0.16	0.44	0.73	2.98	-7.51E+00	3.74E-01		-7.51E+00	3.96E-01		-5.33E-01	5.45E-01		-2.95E+00	5.84E-03		7.20E-01	5.07E-01		2.76E+00	4.59E-07	Up	Unigene117873_All//gi|255584614|ref|XP_002533031.1|//3.00E-14//conserved hypothetical protein [Ricinus communis] >gi|223527193|gb|EEF29362.1| conserved hypothetical protein [Ricinus communis]						nr -
Unigene117881_All	979	0.00	0.00	0.00	0.20	0.04	0.00	0.60	1.15	1.79	null	null		null	null		-2.53E+00	2.34E-01		-7.67E+00	1.34E-01		9.31E-01	1.09E-01		1.57E+00	5.53E-04	Up	Unigene117881_All//gi|210063111|gb|ACJ06506.1|//9.00E-80//polygalacturonase [Diospyros kaki]	Unigene117881_All//3.00E-74//AT3G07970| QRT2 (QUARTET 2); polygalacturonase	Unigene117881_All//0.00E+00//gi|3325965|gb|AI054851.1|AI054851		ko00500|Starch and sucrose metabolism//ko00040|Pentose and glucuronate interconversions	GO:0004553//GO:0008152	nr -
Unigene117882_All	286	0.00	0.00	0.00	0.00	0.00	0.00	0.00	0.00	2.79	null	null		null	null		null	null		null	null		null	null		1.14E+01	7.36E-06	Up							
Unigene117898_All	236	0.00	0.00	0.00	0.00	0.00	0.00	0.36	0.35	4.73	null	null		null	null		null	null		null	null		-1.66E-02	1.01E+00		3.72E+00	4.35E-06	Up			Unigene117898_All//5.00E-18//gi|109859340|gb|DW488319.1|DW488319				ESTscan -
Unigene117903_All	223	0.00	0.00	0.00	0.00	0.00	0.00	1.14	0.75	5.36	null	null		null	null		null	null		null	null		-6.02E-01	6.48E-01		2.24E+00	3.02E-04	Up			Unigene117903_All//2.00E-14//TC134559				
Unigene117909_All	477	287.82	242.19	153.16	160.62	256.28	253.56	358.10	630.18	954.21	-2.49E-01	1.13E-08		-9.10E-01	5.87E-82		6.74E-01	1.27E-53		6.59E-01	5.24E-46		8.15E-01	3.32E-187		1.41E+00	0.00E+00	Up	Unigene117909_All//gi|298706692|emb|CBJ34222.1|//1.00E-18//conserved unknown protein [Ectocarpus siliculosus] >gi|298713886|emb|CBJ33762.1| conserved unknown protein [Ectocarpus siliculosus]		Unigene117909_All//3.00E-110//gi|13351010|gb|BG441370.1|BG441370				nr +
Unigene11791_All	2806	18.94	15.58	19.20	22.92	32.64	26.93	23.69	26.17	9.81	-2.82E-01	1.10E-04		1.97E-02	8.16E-01		5.10E-01	1.33E-24		2.33E-01	9.29E-05		1.44E-01	1.26E-02		-1.27E+00	6.33E-89	Down	Unigene11791_All//gi|18396647|ref|NP_564298.1|//0.00E+00//SCPL45 (SERINE CARBOXYPEPTIDASE-LIKE 45 PRECURSOR); serine-type carboxypeptidase [Arabidopsis thaliana] >gi|42571671|ref|NP_973926.1| SCPL45 (SERINE CARBOXYPEPTIDASE-LIKE 45 PRECURSOR); serine-type carboxypeptidase [Arabidopsis thaliana] >gi|75331738|sp|Q93Y09.1|SCP45_ARATH RecName: Full=Serine carboxypeptidase-like 45; Flags: Precursor >gi|31376397|gb|AAP49525.1| At1g28110 [Arabidopsis thaliana]	Unigene11791_All//0.00E+00//AT1G28110| SCPL45 (SERINE CARBOXYPEPTIDASE-LIKE 45 PRECURSOR); serine-type carboxypeptidase	Unigene11791_All//0.00E+00//TC171096			GO:0004180//GO:0005618//GO:0019538//GO:0031410	nr -
Unigene117917_All	442	0.36	0.46	0.48	0.90	0.23	0.20	0.29	0.00	2.16	3.69E-01	8.17E-01		4.41E-01	7.76E-01		-1.95E+00	1.19E-01		-2.14E+00	1.26E-01		-8.16E+00	2.36E-01		2.91E+00	1.88E-04	Up	Unigene117917_All//gi|115439195|ref|NP_001043877.1|//2.00E-09//Os01g0681000 [Oryza sativa Japonica Group] >gi|113533408|dbj|BAF05791.1| Os01g0681000 [Oryza sativa Japonica Group]	Unigene117917_All//2.00E-08//AT3G49190| condensation domain-containing protein	Unigene117917_All//6.00E-13//TC157940				nr +
Unigene117945_All	752	0.00	0.13	0.07	0.00	0.00	0.00	1.01	1.72	11.76	7.07E+00	3.89E-01		6.15E+00	6.38E-01		null	null		null	null		7.68E-01	1.50E-01		3.54E+00	2.24E-42	Up			Unigene117945_All//5.00E-76//TC147375				
Unigene117951_All	305	9.09	1.83	4.71	8.17	2.60	1.33	5.82	47.00	2.61	-2.31E+00	1.92E-07	Down	-9.48E-01	1.68E-02		-1.65E+00	2.41E-05	Down	-2.62E+00	3.98E-08	Down	3.01E+00	6.39E-58	Up	-1.16E+00	6.84E-03				Unigene117951_All//1.00E-96//BQ409813				
Unigene117958_All	821	0.00	0.00	0.00	0.00	0.00	0.00	0.21	0.00	2.86	null	null		null	null		null	null		null	null		-7.69E+00	1.35E-01		3.80E+00	2.43E-12	Up	Unigene117958_All//gi|224132258|ref|XP_002328224.1|//1.00E-33//cc-nbs-lrr resistance protein [Populus trichocarpa] >gi|222837739|gb|EEE76104.1| cc-nbs-lrr resistance protein [Populus trichocarpa]	Unigene117958_All//5.00E-25//AT3G14470| disease resistance protein (NBS-LRR class), putative			ko04626|Plant-pathogen interaction	GO:0005488	nr +
Unigene117972_All	474	0.00	0.00	0.11	0.00	0.22	0.57	0.18	1.23	1.77	null	null		6.81E+00	6.38E-01		7.77E+00	4.57E-01		9.16E+00	7.04E-02		2.79E+00	8.26E-03		3.31E+00	2.30E-04	Up	Unigene117972_All//gi|188509950|gb|ACD56636.1|//6.00E-06//disease resistance related protein [Gossypium raimondii]		Unigene117972_All//4.00E-91//TC176729				nr -
Unigene117973_All	231	0.00	9.21	8.53	0.43	0.00	0.00	1.46	3.62	18.28	1.32E+01	4.10E-12	Up	1.31E+01	7.46E-11	Up	-8.75E+00	3.15E-01		-8.75E+00	4.02E-01		1.31E+00	6.56E-02		3.64E+00	4.95E-21	Up	Unigene117973_All//gi|145408581|ref|YP_001152205.1|//5.00E-08//ORF58e [Pinus koraiensis] >gi|145048830|gb|ABP35448.1| ORF58e [Pinus koraiensis]		Unigene117973_All//2.00E-48//gi|109859341|gb|DW488320.1|DW488320			GO:0009536	nr -
Unigene117980_All	698	0.00	0.15	0.00	0.36	0.10	0.00	0.48	1.08	1.94	7.18E+00	3.89E-01		null	null		-1.85E+00	2.75E-01		-8.48E+00	7.34E-02		1.15E+00	1.26E-01		2.00E+00	3.24E-04	Up	Unigene117980_All//gi|79339171|ref|NP_172362.3|//4.00E-30//BON3 (BONZAI 3); calcium-dependent phospholipid binding [Arabidopsis thaliana] >gi|53636240|gb|AAU89273.1| BON3 [Arabidopsis thaliana]	Unigene117980_All//9.00E-27//AT1G08860| BON3 (BONZAI 3); calcium-dependent phospholipid binding				GO:0006968//GO:0010941	nr +
Unigene117989_All	720	0.00	0.00	0.00	0.00	0.00	0.00	0.06	0.64	8.19	null	null		null	null		null	null		null	null		3.44E+00	1.16E-02		7.12E+00	2.30E-40	Up	Unigene117989_All//gi|301016726|dbj|BAJ11784.1|//2.00E-19//dehydration responsive protein [Corchorus olitorius]		Unigene117989_All//2.00E-13//TC152783				nr -
Unigene117994_All	242	0.00	0.42	0.00	0.00	0.00	0.56	0.17	0.86	2.96	8.71E+00	3.89E-01		null	null		null	null		9.13E+00	3.12E-01		2.31E+00	2.40E-01		4.08E+00	2.41E-04	Up							
Unigene117998_All	920	0.00	0.00	0.00	0.00	0.00	0.00	0.00	0.18	6.80	null	null		null	null		null	null		null	null		7.51E+00	1.39E-01		1.27E+01	8.68E-45	Up	Unigene117998_All//gi|225455127|ref|XP_002267039.1|//2.00E-12//PREDICTED: hypothetical protein [Vitis vinifera]					GO:0003676//GO:0006259//GO:0046914	nr -
Unigene118002_All	363	0.00	0.00	0.00	0.00	0.00	0.00	0.00	0.00	2.09	null	null		null	null		null	null		null	null		null	null		1.10E+01	1.39E-05	Up	Unigene118002_All//gi|124359276|gb|ABN05778.1|//7.00E-10//Polynucleotidyl transferase, Ribonuclease H fold [Medicago truncatula]	Unigene118002_All//7.00E-07//AT4G29090| reverse transcriptase, putative / RNA-dependent DNA polymerase, putative				GO:0003824	nr -
Unigene118005_All	414	0.51	0.24	0.64	0.96	0.33	0.22	0.20	0.50	2.02	-1.05E+00	5.65E-01		3.47E-01	7.97E-01		-1.53E+00	2.06E-01		-2.14E+00	1.26E-01		1.31E+00	4.47E-01		3.31E+00	2.30E-04	Up							
Unigene11801_All	2412	9.72	28.91	21.37	18.13	21.69	27.49	16.35	26.77	28.82	1.57E+00	1.66E-101	Up	1.14E+00	1.19E-44	Up	2.58E-01	2.13E-04		6.00E-01	1.16E-21		7.11E-01	2.35E-32		8.18E-01	4.19E-45				Unigene11801_All//0.00E+00//TC174620				
Unigene118012_All	229	0.00	0.00	0.00	0.00	0.30	0.99	0.00	2.37	4.52	null	null		null	null		8.23E+00	6.12E-01		9.95E+00	1.19E-01		1.12E+01	5.36E-04	Up	1.21E+01	1.58E-07	Up	Unigene118012_All//gi|1769897|emb|CAA69271.1|//7.00E-12//lectin receptor kinase [Arabidopsis thaliana]	Unigene118012_All//2.00E-11//AT1G48720| unknown protein	Unigene118012_All//3.00E-16//TC178414			GO:0005488	nr -

Unigene11802_All	706	21.79	2.44	15.01	12.49	13.12	5.50	1.38	0.83	0.17	-3.16E+00	1.89E-53	Down	-5.38E-01	2.67E-04		7.12E-02	7.85E-01		-1.18E+00	1.44E-09	Down	-7.33E-01	2.48E-01		-3.02E+00	9.60E-05	Down	Unigene11802_All//gi|163889378|gb|ABY48148.1|//4.00E-18//lectin [Medicago truncatula]	Unigene11802_All//6.00E-07//AT5G10530| lectin protein kinase, putative	Unigene11802_All//9.00E-90//TC144439			GO:0004672//GO:0005488	nr +
Unigene118020_All	224	0.93	1.13	4.28	5.11	4.77	0.00	1.51	1.31	1.42	2.76E-01	8.43E-01		2.20E+00	9.10E-03		-1.02E-01	8.56E-01		-1.23E+01	3.58E-07	Down	-2.09E-01	8.50E-01		-8.58E-02	9.47E-01		Unigene118020_All//gi|297739057|emb|CBI28546.3|//7.00E-07//unnamed protein product [Vitis vinifera]	Unigene118020_All//3.00E-06//AT1G60460| unknown protein	Unigene118020_All//1.00E-40//BG445931				nr -
Unigene118023_All	294	0.00	0.00	0.36	0.00	0.00	0.00	6.62	0.43	1.22	null	null		8.50E+00	4.00E-01		null	null		null	null		-3.96E+00	2.04E-10	Down	-2.44E+00	3.73E-07	Down	Unigene118023_All//gi|253759874|ref|XP_002488951.1|//6.00E-11//hypothetical protein SORBIDRAFT_1292s002010 [Sorghum bicolor] >gi|241947058|gb|EES20203.1| hypothetical protein SORBIDRAFT_1292s002010 [Sorghum bicolor]		Unigene118023_All//3.00E-23//TC148521				nr -
Unigene118024_All	262	6.99	29.78	14.43	10.08	12.62	36.37	12.75	9.57	6.39	2.09E+00	2.53E-17	Up	1.05E+00	1.60E-03		3.24E-01	3.92E-01		1.85E+00	4.23E-19	Up	-4.14E-01	1.91E-01		-9.97E-01	7.10E-04		Unigene118024_All//gi|2764806|emb|CAA55294.1|//1.00E-27//epoxide hydrolase [Glycine max]	Unigene118024_All//4.00E-26//AT3G05600| epoxide hydrolase, putative	Unigene118024_All//3.00E-115//gi|48753192|gb|CO083711.1|CO083711			GO:0004713	nr -
Unigene118026_All	598	0.09	0.17	0.36	0.08	0.29	0.15	1.06	0.14	0.07	9.54E-01	7.27E-01		2.03E+00	3.62E-01		1.79E+00	5.21E-01		8.58E-01	7.95E-01		-2.92E+00	4.20E-03		-3.99E+00	5.82E-04	Down	Unigene118026_All//gi|15218183|ref|NP_175630.1|//2.00E-41//proton-dependent oligopeptide transport (POT) family protein [Arabidopsis thaliana] >gi|75186219|sp|Q9M817.1|PTR2X_ARATH RecName: Full=Probable peptide transporter At1g52190	Unigene118026_All//6.00E-36//AT1G52190| proton-dependent oligopeptide transport (POT) family protein				GO:0015833//GO:0044464	nr -
Unigene118028_All	323	0.00	0.00	0.00	0.31	0.11	0.14	0.26	0.00	2.84	null	null		null	null		-1.53E+00	5.87E-01		-1.14E+00	6.78E-01		-8.03E+00	4.05E-01		3.44E+00	7.53E-05	Up	Unigene118028_All//gi|3894393|gb|AAC78596.1|//4.00E-18//Hcr2-5D [Solanum lycopersicum var. cerasiforme]	Unigene118028_All//2.00E-13//AT2G01950| BRL2 (BRI1-LIKE 2); ATP binding / protein serine/threonine kinase/ transmembrane receptor protein serine/threonine kinase				GO:0003824	nr -
Unigene11803_All	497	13.48	2.85	5.57	8.12	6.65	3.27	0.94	4.29	0.56	-2.24E+00	2.32E-16	Down	-1.27E+00	1.97E-07	Down	-2.88E-01	3.81E-01		-1.31E+00	1.89E-05	Down	2.20E+00	1.16E-06	Up	-7.38E-01	4.26E-01		Unigene11803_All//gi|115440305|ref|NP_001044432.1|//3.00E-18//Os01g0779300 [Oryza sativa Japonica Group] >gi|113533963|dbj|BAF06346.1| Os01g0779300 [Oryza sativa Japonica Group]	Unigene11803_All//6.00E-11//AT5G42120| lectin protein kinase family protein	Unigene11803_All//0.00E+00//TC164982			GO:0004672//GO:0006464//GO:0031410//GO:0032559	nr +
Unigene118037_All	904	0.06	0.00	0.06	0.00	0.00	0.00	0.23	1.20	1.06	-5.86E+00	6.15E-01		2.47E-02	9.96E-01		null	null		null	null		2.36E+00	5.23E-04	Up	2.18E+00	1.83E-03				Unigene118037_All//1.00E-15//TC149689				
Unigene11804_All	2310	0.91	0.22	0.69	7.14	7.51	5.32	3.73	9.14	3.83	-2.05E+00	4.33E-05	Down	-3.90E-01	3.94E-01		7.39E-02	6.52E-01		-4.25E-01	1.74E-03		1.29E+00	1.38E-28	Up	3.62E-02	8.54E-01		Unigene11804_All//gi|115440305|ref|NP_001044432.1|//1.00E-127//Os01g0779300 [Oryza sativa Japonica Group] >gi|113533963|dbj|BAF06346.1| Os01g0779300 [Oryza sativa Japonica Group]	Unigene11804_All//2.00E-124//AT5G10530| lectin protein kinase, putative	Unigene11804_All//0.00E+00//DW242638		ko04626|Plant-pathogen interaction		nr -
Unigene118044_All	335	0.00	0.00	0.00	0.00	0.00	0.00	0.50	0.37	3.57	null	null		null	null		null	null		null	null		-4.32E-01	7.93E-01		2.82E+00	3.02E-05	Up	Unigene118044_All//gi|253721991|gb|ACT34060.1|//4.00E-19//Cf2/Cf5-like disease resistance protein [Aegilops tauschii]	Unigene118044_All//1.00E-13//AT2G34930| disease resistance family protein			ko04626|Plant-pathogen interaction	GO:0016491	nr -
Unigene118049_All	403	8.44	14.46	5.02	13.72	19.05	18.38	19.30	22.30	9.19	7.77E-01	1.60E-03		-7.49E-01	3.29E-02		4.74E-01	2.21E-02		4.22E-01	5.69E-02		2.08E-01	2.75E-01		-1.07E+00	1.04E-08	Down	Unigene118049_All//gi|18423628|ref|NP_568808.1|//2.00E-06//universal stress protein (USP) family protein [Arabidopsis thaliana]		Unigene118049_All//2.00E-101//gi|78351714|gb|DT571988.1|DT571988				nr -
Unigene11805_All	2393	4.72	1.74	4.87	6.87	7.18	4.17	4.06	6.13	1.95	-1.44E+00	3.56E-15	Down	4.53E-02	8.06E-01		6.39E-02	7.18E-01		-7.20E-01	7.03E-08		5.93E-01	5.08E-06		-1.06E+00	1.58E-10	Down	Unigene11805_All//gi|226498624|ref|NP_001147903.1|//1.00E-125//protein kinase [Zea mays] >gi|195614480|gb|ACG29070.1| protein kinase [Zea mays]	Unigene11805_All//1.00E-86//AT5G10530| lectin protein kinase, putative	Unigene11805_All//0.00E+00//ES817348		ko04626|Plant-pathogen interaction		nr -
Unigene118056_All	463	0.11	0.00	0.23	0.65	0.37	0.29	1.19	0.00	0.34	-6.82E+00	6.15E-01		1.03E+00	7.04E-01		-7.96E-01	5.57E-01		-1.14E+00	4.58E-01		-1.02E+01	4.61E-04	Down	-1.79E+00	5.07E-02		Unigene118056_All//gi|224056507|ref|XP_002298888.1|//2.00E-15//predicted protein [Populus trichocarpa] >gi|118485886|gb|ABK94789.1| unknown [Populus trichocarpa] >gi|222846146|gb|EEE83693.1| predicted protein [Populus trichocarpa]		Unigene118056_All//6.00E-47//TC168043				nr -
Unigene118059_All	617	0.00	0.00	0.00	0.00	0.00	0.00	0.69	0.27	5.04	null	null		null	null		null	null		null	null		-1.34E+00	2.24E-01		2.88E+00	9.23E-13	Up			Unigene118059_All//8.00E-84//gi|73859503|gb|DT462242.1|DT462242				
Unigene11806_All	981	0.11	0.10	0.43	1.12	1.79	2.35	7.93	3.11	60.43	-4.67E-02	9.90E-01		2.03E+00	1.40E-01		6.80E-01	1.82E-01		1.07E+00	1.20E-02		-1.35E+00	8.60E-12	Down	2.93E+00	2.49E-235	Up	Unigene11806_All//gi|124359440|gb|ABN05887.1|//1.00E-149//[2Fe-2S]-binding [Medicago truncatula]	Unigene11806_All//2.00E-142//AT3G62010| unknown protein	Unigene11806_All//2.00E-140//gi|54117136|gb|CO492645.1|CO492645				nr -
Unigene11807_All	1602	0.62	1.17	1.13	3.54	4.51	4.96	9.79	5.37	25.56	9.15E-01	5.49E-02		8.65E-01	8.81E-02		3.49E-01	1.23E-01		4.85E-01	2.00E-02		-8.65E-01	1.37E-11		1.38E+00	3.66E-62	Up	Unigene11807_All//gi|297817536|ref|XP_002876651.1|//0.00E+00//metal ion binding protein [Arabidopsis lyrata subsp. lyrata] >gi|297322489|gb|EFH52910.1| metal ion binding protein [Arabidopsis lyrata subsp. lyrata]	Unigene11807_All//0.00E+00//AT3G62010| unknown protein	Unigene11807_All//0.00E+00//gi|54117136|gb|CO492645.1|CO492645				nr +
Unigene118070_All	463	0.00	0.00	0.00	0.11	0.00	0.00	0.00	0.00	1.55	null	null		null	null		-6.75E+00	5.59E-01		-6.75E+00	6.40E-01		null	null		1.06E+01	2.63E-05	Up							
Unigene118076_All	384	0.00	0.00	0.00	0.00	0.00	0.00	0.77	0.22	3.53	null	null		null	null		null	null		null	null		-1.82E+00	2.11E-01		2.19E+00	1.27E-04	Up	Unigene118076_All//gi|55168340|gb|AAV44205.1|//1.00E-08//unknow protein [Oryza sativa Japonica Group]		Unigene118076_All//2.00E-12//TC139311				nr +
Unigene118079_All	407	0.00	0.00	0.00	0.00	0.00	0.00	0.62	1.13	5.68	null	null		null	null		null	null		null	null		8.58E-01	3.96E-01		3.19E+00	1.37E-10	Up	Unigene118079_All//gi|147860110|emb|CAN82922.1|//2.00E-07//hypothetical protein [Vitis vinifera]		Unigene118079_All//4.00E-38//gi|109849827|gb|DW478806.1|DW478806			GO:0005488//GO:0016020//GO:0044237//GO:0044424	nr +
Unigene11808_All	853	1.35	1.31	4.74	3.62	4.32	5.61	6.49	3.82	4.48	-4.63E-02	9.53E-01		1.81E+00	1.57E-07	Up	2.55E-01	4.94E-01		6.32E-01	2.25E-02		-7.65E-01	6.81E-04		-5.34E-01	1.46E-02		Unigene11808_All//gi|297817536|ref|XP_002876651.1|//1.00E-125//metal ion binding protein [Arabidopsis lyrata subsp. lyrata] >gi|297322489|gb|EFH52910.1| metal ion binding protein [Arabidopsis lyrata subsp. lyrata]	Unigene11808_All//3.00E-126//AT3G62010| unknown protein	Unigene11808_All//9.00E-177//gi|54117136|gb|CO492645.1|CO492645				nr -
Unigene118086_All	577	0.18	0.35	0.00	0.26	0.66	0.39	0.29	0.94	3.25	9.54E-01	6.22E-01		-7.50E+00	3.96E-01		1.34E+00	3.40E-01		5.96E-01	7.22E-01		1.68E+00	7.92E-02		3.47E+00	2.57E-09	Up							
Unigene11809_All	1369	2.14	34.20	6.07	11.03	19.80	20.95	13.03	29.31	47.67	4.00E+00	8.96E-196	Up	1.50E+00	1.73E-11	Up	8.44E-01	1.03E-17		9.26E-01	6.40E-20		1.17E+00	5.35E-46	Up	1.87E+00	1.30E-151	Up	Unigene11809_All//gi|206584341|gb|ACI15343.1|//0.00E+00//NAC domain protein NAC6 [Gossypium hirsutum] >gi|206584359|gb|ACI15352.1| NAC domain protein NAC6 [Gossypium hirsutum]	Unigene11809_All//4.00E-94//AT5G18270| ANAC087; transcription factor	Unigene11809_All//0.00E+00//TC145720	NAC		GO:0003676//GO:0010468	nr -
Unigene118095_All	229	0.00	0.00	0.23	0.22	0.45	0.00	0.00	0.00	4.70	null	null		7.86E+00	6.38E-01		1.05E+00	8.09E-01		-7.76E+00	6.40E-01		null	null		1.22E+01	8.30E-08	Up			Unigene118095_All//1.00E-46//gi|78346504|gb|DT566778.1|DT566778				
Unigene1181_All	1246	3.07	10.70	4.49	7.00	11.58	11.89	7.13	8.76	6.84	1.80E+00	4.96E-24	Up	5.50E-01	3.77E-02		7.27E-01	1.42E-07		7.65E-01	8.13E-08		2.97E-01	6.56E-02		-5.86E-02	7.49E-01		Unigene1181_All//gi|225442186|ref|XP_002276676.1|//1.00E-135//PREDICTED: similar to mitochondrial substrate carrier family protein isoform 1 [Vitis vinifera]	Unigene1181_All//9.00E-115//AT1G72820| mitochondrial substrate carrier family protein	Unigene1181_All//0.00E+00//gi|164250315|gb|ES810237.1|ES810237				nr -
Unigene11810_All	1577	2.39	4.88	3.61	8.78	4.98	8.31	45.52	134.77	184.95	1.03E+00	1.52E-06	Up	5.97E-01	2.21E-02		-8.19E-01	3.68E-09		-8.04E-02	6.64E-01		1.57E+00	0.00E+00	Up	2.02E+00	0.00E+00	Up	Unigene11810_All//gi|206584341|gb|ACI15343.1|//1.00E-126//NAC domain protein NAC6 [Gossypium hirsutum] >gi|206584359|gb|ACI15352.1| NAC domain protein NAC6 [Gossypium hirsutum]	Unigene11810_All//1.00E-97//AT5G18270| ANAC087; transcription factor	Unigene11810_All//0.00E+00//TC171573	NAC			nr +
Unigene118100_All	602	0.00	0.00	0.00	0.00	0.00	0.00	0.00	0.00	1.52	null	null		null	null		null	null		null	null		null	null		1.06E+01	1.08E-06	Up	Unigene118100_All//gi|224061413|ref|XP_002300467.1|//8.00E-06//predicted protein [Populus trichocarpa] >gi|222847725|gb|EEE85272.1| predicted protein [Populus trichocarpa]						nr +
Unigene118102_All	236	0.89	0.00	0.00	0.00	0.00	0.00	0.90	2.30	6.75	-9.79E+00	1.22E-01		-9.79E+00	1.46E-01		null	null		null	null		1.36E+00	1.44E-01		2.91E+00	5.82E-07	Up			Unigene118102_All//7.00E-14//TC157689				
Unigene118109_All	240	0.00	0.00	0.00	0.00	0.00	0.00	0.00	0.87	2.49	null	null		null	null		null	null		null	null		9.77E+00	7.86E-02		1.13E+01	1.76E-04	Up							
Unigene118119_All	500	0.10	0.00	0.21	0.20	0.00	0.18	0.00	0.17	2.71	-6.71E+00	6.15E-01		1.03E+00	7.04E-01		-7.64E+00	3.15E-01		-1.41E-01	9.51E-01		7.39E+00	4.03E-01		1.14E+01	9.04E-10	Up	Unigene118119_All//gi|171988286|gb|ACB59355.1|//7.00E-35//peroxisome biogenesis factor 13 [Nicotiana tabacum]	Unigene118119_All//3.00E-12//AT3G07560| PEX13 (PEROXIN 13); protein binding	Unigene118119_All//5.00E-32//gi|78334975|gb|DT555249.1|DT555249		ko03040|Spliceosome	GO:0006625//GO:0009062//GO:0044439	nr -
Unigene118139_All	477	0.00	0.00	0.00	0.00	0.07	0.00	0.44	0.88	4.18	null	null		null	null		6.17E+00	8.33E-01		null	null		9.83E-01	3.73E-01		3.24E+00	2.44E-09	Up			Unigene118139_All//5.00E-35//BQ407647				
Unigene118148_All	250	0.84	6.08	2.77	9.37	3.72	6.14	0.34	1.50	0.64	2.86E+00	2.69E-05	Up	1.73E+00	7.68E-02		-1.33E+00	7.69E-04	Down	-6.09E-01	1.55E-01		2.15E+00	9.45E-02		9.14E-01	6.25E-01				Unigene118148_All//9.00E-60//TC157852				
Unigene118163_All	238	10.77	5.54	2.68	7.33	12.01	9.87	35.35	10.71	13.39	-9.61E-01	1.52E-02		-2.00E+00	1.34E-05	Down	7.13E-01	5.38E-02		4.30E-01	3.46E-01		-1.72E+00	8.53E-18	Down	-1.40E+00	8.29E-14	Down	Unigene118163_All//gi|255581663|ref|XP_002531635.1|//3.00E-09//alpha-glucosidase, putative [Ricinus communis] >gi|223528753|gb|EEF30763.1| alpha-glucosidase, putative [Ricinus communis]	Unigene118163_All//3.00E-06//AT1G68560| XYL1 (ALPHA-XYLOSIDASE 1); alpha-N-arabinofuranosidase/ hydrolase, hydrolyzing O-glycosyl compounds / xylan 1,4-beta-xylosidase	Unigene118163_All//3.00E-84//BQ414225				nr -
Unigene118166_All	543	0.00	0.00	0.00	0.00	0.06	0.00	0.31	0.92	16.80	null	null		null	null		5.99E+00	8.33E-01		null	null		1.57E+00	1.18E-01		5.75E+00	1.32E-58	Up	Unigene118166_All//gi|145408582|ref|YP_001152206.1|//1.00E-07//ORF137 [Pinus koraiensis] >gi|145048831|gb|ABP35449.1| ORF137 [Pinus koraiensis]		Unigene118166_All//2.00E-44//gi|109823694|gb|DW008546.1|DW008546			GO:0009536	nr +
Unigene118167_All	733	0.14	0.00	0.00	0.14	0.09	0.00	0.46	0.40	2.28	-7.16E+00	3.74E-01		-7.16E+00	3.96E-01		-5.32E-01	8.17E-01		-7.09E+00	4.02E-01		-2.09E-01	8.50E-01		2.31E+00	8.00E-06	Up	Unigene118167_All//gi|224071947|ref|XP_002303599.1|//5.00E-80//tir-nbs resistance protein [Populus trichocarpa] >gi|222841031|gb|EEE78578.1| tir-nbs resistance protein [Populus trichocarpa]	Unigene118167_All//4.00E-67//AT4G23440| nucleoside-triphosphatase/ nucleotide binding / transmembrane receptor				GO:0004872//GO:0006952//GO:0012501//GO:0016462//GO:0023060//GO:0032559//GO:0043231//GO:0044425	nr -
Unigene118177_All	503	0.00	0.00	0.00	0.00	0.00	0.00	0.00	0.25	2.06	null	null		null	null		null	null		null	null		7.96E+00	2.41E-01		1.10E+01	1.58E-07	Up			Unigene118177_All//2.00E-161//CO112141				ESTscan +
Unigene11818_All	540	3.88	4.32	3.06	6.64	4.97	4.43	6.50	2.63	6.12	1.55E-01	7.01E-01		-3.42E-01	4.64E-01		-4.17E-01	2.08E-01		-5.83E-01	7.68E-02		-1.30E+00	1.72E-05	Down	-8.58E-02	7.71E-01		Unigene11818_All//gi|30695999|ref|NP_199921.2|//6.00E-26//RNase H domain-containing protein [Arabidopsis thaliana] >gi|79330542|ref|NP_001032053.1| RNase H domain-containing protein [Arabidopsis thaliana] >gi|29028872|gb|AAO64815.1| At5g51080 [Arabidopsis thaliana] >gi|222424417|dbj|BAH20164.1| AT5G51080 [Arabidopsis thaliana]	Unigene11818_All//1.00E-23//AT1G24090| RNase H domain-containing protein	Unigene11818_All//0.00E+00//TC177117			GO:0005488//GO:0016891	nr +
Unigene118181_All	310	0.00	0.00	0.00	0.00	0.00	0.00	0.14	0.00	2.06	null	null		null	null		null	null		null	null		-7.09E+00	6.60E-01		3.91E+00	7.66E-04	Up							ESTscan +
Unigene11820_All	706	5.85	2.30	3.85	4.73	5.95	4.67	1.44	1.95	1.86	-1.35E+00	1.53E-05	Down	-6.06E-01	5.38E-02		3.32E-01	3.12E-01		-1.77E-02	9.50E-01		4.43E-01	3.96E-01		3.74E-01	4.74E-01		Unigene11820_All//gi|9369399|gb|AAF87147.1|AC002423_12//1.00E-25//T23E23.24 [Arabidopsis thaliana]	Unigene11820_All//2.00E-23//AT1G24090| RNase H domain-containing protein	Unigene11820_All//0.00E+00//TC177117			GO:0005488//GO:0016891	nr +

Unigene118202_All	355	0.29	0.14	0.15	0.14	0.10	0.00	0.24	0.00	2.81	-1.05E+00	6.85E-01		-9.75E-01	7.21E-01		-5.33E-01	8.27E-01		-7.13E+00	6.39E-01		-7.90E+00	4.05E-01		3.56E+00	2.43E-05	Up							
Unigene118204_All	447	0.00	0.00	0.00	0.00	0.23	0.00	0.00	0.19	1.52	null	null		null	null		7.85E+00	4.57E-01		null	null		7.55E+00	4.03E-01		1.06E+01	4.97E-05	Up			Unigene118204_All//2.00E-34//TC177455				
Unigene118205_All	313	1.00	1.30	2.04	1.59	1.21	1.01	0.95	0.93	4.96	3.69E-01	7.24E-01		1.03E+00	2.81E-01		-3.95E-01	6.89E-01		-6.56E-01	5.50E-01		-1.67E-02	1.02E+00		2.39E+00	1.16E-05	Up			Unigene118205_All//4.00E-47//TC174278				
Unigene118208_All	392	0.00	0.00	0.00	0.00	0.00	0.00	0.43	0.00	2.74	null	null		null	null		null	null		null	null		-8.75E+00	1.35E-01		2.67E+00	1.42E-04	Up							
Unigene118215_All	239	0.00	0.00	0.22	0.00	0.43	0.38	0.00	0.70	2.17	null	null		7.80E+00	6.38E-01		8.76E+00	4.57E-01		8.56E+00	4.79E-01		9.45E+00	1.39E-01		1.11E+01	6.14E-04	Up							
Unigene11822_All	503	0.42	0.40	0.42	4.16	3.70	3.77	6.14	4.82	2.93	-4.65E-02	9.89E-01		2.54E-02	1.00E+00		-1.70E-01	7.32E-01		-1.41E-01	7.55E-01		-3.49E-01	3.05E-01		-1.07E+00	5.76E-04	Down	Unigene11822_All//gi|224064438|ref|XP_002301476.1|//6.00E-72//multidrug resistance protein ABC transporter family [Populus trichocarpa] >gi|222843202|gb|EEE80749.1| multidrug resistance protein ABC transporter family [Populus trichocarpa]	Unigene11822_All//4.00E-30//AT2G47800| ATMRP4 (ARABIDOPSIS THALIANA MULTIDRUG RESISTANCE-ASSOCIATED PROTEIN 4); ATPase, coupled to transmembrane movement of substances / folic acid transporter	Unigene11822_All//0.00E+00//ES834598		ko02010|ABC transporters	GO:0005773//GO:0006950//GO:0008152//GO:0009987//GO:0015405//GO:0015893//GO:0016628//GO:0017111//GO:0031090//GO:0031224//GO:0032559//GO:0051183//GO:0051707	nr +
Unigene118223_All	978	21.93	13.57	16.72	6.11	6.27	2.72	0.43	0.64	0.65	-6.92E-01	5.96E-09		-3.92E-01	1.53E-03		3.62E-02	9.05E-01		-1.17E+00	1.48E-06	Down	5.68E-01	5.04E-01		5.92E-01	4.53E-01		Unigene118223_All//gi|240256095|ref|NP_194537.6|//3.00E-24//binding [Arabidopsis thaliana]	Unigene118223_All//3.00E-20//AT4G28080| binding	Unigene118223_All//4.00E-99//DN800287			GO:0005488	nr -
Unigene118226_All	431	0.00	0.24	0.12	0.00	0.16	0.21	0.69	0.39	4.44	7.88E+00	3.89E-01		6.95E+00	6.38E-01		7.32E+00	6.12E-01		7.71E+00	4.79E-01		-8.24E-01	5.40E-01		2.69E+00	1.24E-07	Up							
Unigene11823_All	295	1.42	0.34	2.53	4.39	1.05	2.76	5.30	5.24	2.97	-2.05E+00	1.21E-01		8.33E-01	3.39E-01		-2.06E+00	6.01E-04	Down	-6.72E-01	2.74E-01		-1.66E-02	1.01E+00		-8.36E-01	6.46E-02		Unigene11823_All//gi|297596742|ref|NP_001043000.2|//2.00E-37//Os01g0356000 [Oryza sativa Japonica Group] >gi|255673217|dbj|BAF04914.2| Os01g0356000 [Oryza sativa Japonica Group]	Unigene11823_All//5.00E-38//AT2G47800| ATMRP4 (ARABIDOPSIS THALIANA MULTIDRUG RESISTANCE-ASSOCIATED PROTEIN 4); ATPase, coupled to transmembrane movement of substances / folic acid transporter	Unigene11823_All//1.00E-71//CO127490		ko02010|ABC transporters	GO:0000166//GO:0005773//GO:0006950//GO:0009987//GO:0015405//GO:0015893//GO:0016491//GO:0017111//GO:0031090//GO:0031410//GO:0051183//GO:0051707	nr -
Unigene118235_All	227	0.00	0.00	3.05	0.44	0.46	0.20	0.93	0.00	1.05	null	null		1.16E+01	6.17E-04	Up	5.23E-02	9.67E-01		-1.14E+00	6.77E-01		-9.86E+00	7.45E-02		1.77E-01	9.09E-01								
Unigene11824_All	1315	7.60	4.55	6.36	26.74	21.63	25.62	9.32	19.29	12.97	-7.41E-01	4.30E-05		-2.57E-01	2.03E-01		-3.06E-01	3.39E-04		-6.19E-02	6.13E-01		1.05E+00	8.67E-25	Up	4.76E-01	4.84E-05		Unigene11824_All//gi|186478074|ref|NP_001117218.1|//1.00E-111//NLA (nitrogen limitation adaptation); ubiquitin-protein ligase [Arabidopsis thaliana]	Unigene11824_All//5.00E-113//AT1G02860| NLA (nitrogen limitation adaptation); ubiquitin-protein ligase				GO:0046872	nr -
Unigene118243_All	295	0.18	0.00	0.00	0.00	0.00	0.00	1.58	0.71	6.89	-7.47E+00	6.15E-01		-7.47E+00	6.28E-01		null	null		null	null		-1.15E+00	2.53E-01		2.13E+00	2.44E-06	Up	Unigene118243_All//gi|224116168|ref|XP_002331978.1|//2.00E-17//tir-nbs-lrr resistance protein [Populus trichocarpa] >gi|222832102|gb|EEE70579.1| tir-nbs-lrr resistance protein [Populus trichocarpa]	Unigene118243_All//2.00E-15//AT5G17680| disease resistance protein (TIR-NBS-LRR class), putative				GO:0005488	nr +
Unigene118247_All	593	0.00	0.00	0.27	0.25	0.00	0.00	0.00	0.07	1.88	null	null		8.07E+00	2.39E-01		-7.98E+00	1.66E-01		-7.98E+00	2.34E-01		6.14E+00	6.37E-01		1.09E+01	4.36E-08	Up							
Unigene118252_All	381	0.27	0.00	0.00	0.00	0.00	0.24	0.33	0.99	12.02	-8.10E+00	3.74E-01		-8.10E+00	3.96E-01		null	null		7.89E+00	4.79E-01		1.57E+00	1.95E-01		5.17E+00	2.53E-28	Up	Unigene118252_All//gi|151301818|gb|ABN50365.2|//5.00E-31//ethylene responsive element binding protein 1 [Gossypium barbadense]	Unigene118252_All//1.00E-19//AT5G47220| ERF2 (ETHYLENE RESPONSIVE ELEMENT BINDING FACTOR 2); DNA binding / transcription activator/ transcription factor	Unigene118252_All//3.00E-45//gi|109825974|gb|DW224695.1|DW224695	AP2-EREBP		GO:0000160//GO:0001071//GO:0002218//GO:0003676//GO:0006351//GO:0009743//GO:0010468//GO:0010557//GO:0030528//GO:0043231	nr +
Unigene118256_All	354	0.00	0.00	0.00	0.00	0.00	0.00	0.00	0.00	1.80	null	null		null	null		null	null		null	null		null	null		1.08E+01	9.35E-05	Up							
Unigene11826_All	3420	12.50	5.38	9.26	17.77	14.05	12.41	58.05	134.27	99.68	-1.22E+00	1.04E-42	Down	-4.32E-01	2.34E-07		-3.39E-01	3.92E-08		-5.18E-01	1.79E-15		1.21E+00	0.00E+00	Up	7.80E-01	5.73E-201		Unigene11826_All//gi|30695748|ref|NP_199787.2|//0.00E+00//leucine-rich repeat family protein / protein kinase family protein [Arabidopsis thaliana] >gi|30102752|gb|AAP21294.1| At5g49760 [Arabidopsis thaliana] >gi|224589713|gb|ACN59388.1| leucine-rich repeat receptor-like protein kinase [Arabidopsis thaliana]	Unigene11826_All//0.00E+00//AT5G49760| leucine-rich repeat family protein / protein kinase family protein	Unigene11826_All//0.00E+00//gi|109879548|gb|DW508518.1|DW508518			GO:0004674//GO:0004871//GO:0006464//GO:0007167//GO:0016020//GO:0016491//GO:0031410//GO:0032559//GO:0043231	nr -
Unigene118267_All	397	0.00	0.00	0.00	0.00	0.09	0.00	0.11	0.74	2.31	null	null		null	null		6.44E+00	8.33E-01		null	null		2.79E+00	9.48E-02		4.44E+00	1.24E-05	Up			Unigene118267_All//5.00E-07//DR455508				
Unigene118268_All	237	0.00	0.00	0.00	0.00	0.00	0.00	1.25	0.88	7.73	null	null		null	null		null	null		null	null		-5.02E-01	6.78E-01		2.63E+00	3.47E-07	Up			Unigene118268_All//9.00E-103//CO132424				ESTscan -
Unigene118269_All	525	0.00	0.00	0.00	0.00	0.00	0.17	0.48	0.72	2.81	null	null		null	null		null	null		7.43E+00	4.79E-01		5.68E-01	6.38E-01		2.54E+00	9.95E-06	Up			Unigene118269_All//6.00E-07//gi|164269365|gb|ES827929.1|ES827929				
Unigene11828_All	4184	2.01	0.46	0.52	4.74	8.55	8.32	3.63	7.31	5.47	-2.13E+00	3.31E-19	Down	-1.95E+00	2.00E-16	Down	8.52E-01	1.29E-23		8.13E-01	2.11E-19		1.01E+00	4.89E-28	Up	5.91E-01	3.86E-09		Unigene11828_All//gi|224122448|ref|XP_002318839.1|//0.00E+00//tir-nbs-lrr resistance protein [Populus trichocarpa] >gi|222859512|gb|EEE97059.1| tir-nbs-lrr resistance protein [Populus trichocarpa]	Unigene11828_All//2.00E-126//AT5G36930| disease resistance protein (TIR-NBS-LRR class), putative	Unigene11828_All//6.00E-166//TC167883		ko04626|Plant-pathogen interaction		nr -
Unigene118280_All	699	0.00	0.00	0.15	0.00	0.00	0.00	0.12	1.08	0.46	null	null		7.25E+00	4.00E-01		null	null		null	null		3.15E+00	9.54E-04	Up	1.91E+00	1.54E-01				Unigene118280_All//2.00E-16//gi|109878436|gb|DW507406.1|DW507406				
Unigene118284_All	233	0.00	0.00	0.00	0.00	0.00	0.00	0.00	0.54	5.47	null	null		null	null		null	null		null	null		9.07E+00	2.41E-01		1.24E+01	3.29E-09	Up							
Unigene118285_All	364	0.00	0.00	0.00	0.27	0.00	0.12	7.20	0.92	0.44	null	null		null	null		-8.10E+00	3.15E-01		-1.14E+00	6.77E-01		-2.97E+00	5.45E-11	Down	-4.04E+00	1.31E-14	Down			Unigene118285_All//3.00E-122//DW510669				
Unigene118286_All	232	0.00	0.00	0.00	0.00	0.00	0.00	0.36	0.36	3.78	null	null		null	null		null	null		null	null		-1.67E-02	1.01E+00		3.37E+00	1.32E-04	Up							
Unigene11829_All	1463	1.50	2.42	1.78	3.10	2.85	2.41	18.61	23.63	91.74	6.91E-01	3.52E-02		2.48E-01	5.65E-01		-1.22E-01	7.14E-01		-3.64E-01	2.34E-01		3.44E-01	2.52E-05		2.30E+00	0.00E+00	Up	Unigene11829_All//gi|195618208|gb|ACG30934.1|//2.00E-34//60S ribosomal protein L19-3 [Zea mays] >gi|195635187|gb|ACG37062.1| 60S ribosomal protein L19-3 [Zea mays]	Unigene11829_All//2.00E-26//AT1G18270| ketose-bisphosphate aldolase class-II family protein	Unigene11829_All//0.00E+00//TC143184		ko03010|Ribosome	GO:0005622	nr -
Unigene118295_All	259	0.00	0.00	0.00	0.00	0.00	0.00	0.33	0.16	3.38	null	null		null	null		null	null		null	null		-1.02E+00	7.26E-01		3.37E+00	1.32E-04	Up			Unigene118295_All//2.00E-29//DT461892				
Unigene118296_All	240	0.44	0.00	0.00	0.00	0.00	0.19	0.18	2.79	1.00	-8.77E+00	3.74E-01		-8.77E+00	3.96E-01		null	null		7.56E+00	6.99E-01		3.98E+00	6.38E-04	Up	2.50E+00	1.59E-01				Unigene118296_All//2.00E-35//gi|73863313|gb|DT466052.1|DT466052				
Unigene118297_All	351	0.00	0.00	0.00	0.00	0.00	0.00	0.00	0.00	2.27	null	null		null	null		null	null		null	null		null	null		1.11E+01	7.36E-06	Up							
Unigene1183_All	742	2.26	3.48	4.31	4.16	5.43	5.23	29.35	78.97	85.69	6.26E-01	1.14E-01		9.32E-01	1.04E-02		3.84E-01	2.34E-01		3.31E-01	3.38E-01		1.43E+00	7.14E-91	Up	1.55E+00	4.39E-113	Up	Unigene1183_All//gi|4530126|gb|AAD21872.1|//8.00E-48//receptor-like protein kinase homolog RK20-1 [Phaseolus vulgaris]	Unigene1183_All//7.00E-39//AT4G21410| protein kinase family protein	Unigene1183_All//0.00E+00//TC153623		ko04626|Plant-pathogen interaction	GO:0004713//GO:0004871//GO:0006464//GO:0032559	nr -
Unigene118304_All	582	0.09	0.00	0.27	0.09	0.36	0.39	0.29	0.07	1.64	-6.49E+00	6.15E-01		1.61E+00	5.11E-01		2.05E+00	3.95E-01		2.18E+00	3.23E-01		-2.02E+00	3.68E-01		2.50E+00	6.42E-04	Up	Unigene118304_All//gi|15237223|ref|NP_197702.1|//5.00E-31//zinc finger (C3HC4-type RING finger) family protein [Arabidopsis thaliana]	Unigene118304_All//3.00E-32//AT5G23110| zinc finger (C3HC4-type RING finger) family protein				GO:0046872	nr -
Unigene118315_All	331	0.16	1.38	0.97	4.06	2.91	3.55	0.64	2.40	6.50	3.12E+00	3.58E-02		2.61E+00	1.46E-01		-4.80E-01	4.15E-01		-1.96E-01	7.30E-01		1.91E+00	1.37E-02		3.35E+00	2.59E-10	Up	Unigene118315_All//gi|24461861|gb|AAN62348.1|AF506028_15//7.00E-18//NBS-LRR type disease resistance protein [Citrus trifoliata]	Unigene118315_All//1.00E-12//AT1G61310| ATP binding / protein binding	Unigene118315_All//5.00E-170//DR461626		ko04626|Plant-pathogen interaction		nr +
Unigene118325_All	233	0.00	1.74	0.00	0.64	0.30	1.55	0.00	0.90	2.74	1.08E+01	1.36E-02		null	null		-1.12E+00	5.84E-01		1.27E+00	3.82E-01		9.81E+00	7.86E-02		1.14E+01	9.34E-05	Up			Unigene118325_All//3.00E-07//DW478609				
Unigene118331_All	668	0.24	0.91	0.56	0.52	0.72	0.27	0.13	0.69	4.71	1.95E+00	6.04E-02		1.25E+00	3.66E-01		4.67E-01	7.04E-01		-9.49E-01	4.81E-01		2.44E+00	3.74E-02		5.22E+00	1.18E-19	Up			Unigene118331_All//9.00E-16//BF276866				
Unigene11834_All	1223	16.86	17.90	21.51	14.01	10.19	6.61	1.21	1.64	0.75	8.66E-02	5.25E-01		3.52E-01	1.51E-03		-4.59E-01	2.40E-04		-1.08E+00	9.61E-16	Down	4.39E-01	3.08E-01		-6.92E-01	1.40E-01		Unigene11834_All//gi|297829562|ref|XP_002882663.1|//1.00E-133//anion-transporting ATPase family protein [Arabidopsis lyrata subsp. lyrata] >gi|297328503|gb|EFH58922.1| anion-transporting ATPase family protein [Arabidopsis lyrata subsp. lyrata]	Unigene11834_All//9.00E-120//AT3G10350| anion-transporting ATPase family protein	Unigene11834_All//0.00E+00//CO081126				nr +
Unigene118341_All	475	4.74	4.16	5.94	9.02	10.80	7.13	10.41	5.10	6.79	-1.87E-01	6.44E-01		3.27E-01	4.00E-01		2.60E-01	3.85E-01		-3.39E-01	2.89E-01		-1.03E+00	2.41E-05	Down	-6.16E-01	8.02E-03		Unigene118341_All//gi|34481803|emb|CAC87837.1|//2.00E-74//cullin 1C [Nicotiana tabacum]	Unigene118341_All//2.00E-73//AT4G02570| ATCUL1 (ARABIDOPSIS THALIANA CULLIN 1); protein binding	Unigene118341_All//0.00E+00//gi|13355430|gb|BG445778.1|BG445778		ko04120|Ubiquitin mediated proteolysis//ko03420|Nucleotide excision repair	GO:0000151//GO:0019899//GO:0019941	nr +
Unigene118347_All	246	0.00	0.00	0.00	0.00	0.00	0.00	0.00	0.34	2.11	null	null		null	null		null	null		null	null		8.41E+00	4.03E-01		1.10E+01	6.14E-04	Up							
Unigene118351_All	418	0.00	0.61	0.51	0.12	0.25	0.00	0.10	0.10	2.00	9.24E+00	7.87E-02		8.99E+00	1.41E-01		1.05E+00	8.09E-01		-6.90E+00	6.39E-01		-1.58E-02	1.01E+00		4.31E+00	4.11E-05	Up	Unigene118351_All//gi|15239544|ref|NP_200218.1|//7.00E-23//AT-HSFA9; DNA binding / transcription factor [Arabidopsis thaliana] >gi|75264295|sp|Q9LVW2.1|HSFA9_ARATH RecName: Full=Heat stress transcription factor A-9; Short=AtHsfA9; AltName: Full=AtHsf-21	Unigene118351_All//4.00E-24//AT5G54070| AT-HSFA9; DNA binding / transcription factor	Unigene118351_All//2.00E-25//TC158707	HSF		GO:0001071//GO:0003677//GO:0006350//GO:0009408//GO:0010557//GO:0030528//GO:0043231	nr -
Unigene11837_All	3115	0.10	0.49	0.27	0.37	0.45	0.67	0.45	0.50	0.77	2.28E+00	2.68E-04	Up	1.44E+00	8.23E-02		3.01E-01	6.16E-01		8.59E-01	6.02E-02		1.49E-01	7.61E-01		7.77E-01	2.89E-02		Unigene11837_All//gi|75336836|sp|Q9S7I6.1|RPK2_ARATH//0.00E+00//RecName: Full=LRR receptor-like serine/threonine-protein kinase RPK2; AltName: Full=Receptor-like protein kinase 2; AltName: Full=Protein TOADSTOOL 2; Flags: Precursor	Unigene11837_All//0.00E+00//AT3G02130| RPK2 (RECEPTOR-LIKE PROTEIN KINASE 2); ATP binding / kinase/ protein serine/threonine kinase	Unigene11837_All//0.00E+00//TC164113		ko04626|Plant-pathogen interaction	GO:0000578//GO:0003006//GO:0005488//GO:0006950//GO:0009555//GO:0009698//GO:0009856//GO:0016020//GO:0016772//GO:0043231//GO:0044444	nr -
Unigene118373_All	255	0.00	0.00	0.00	0.00	0.00	0.00	9.45	2.13	0.78	null	null		null	null		null	null		null	null		-2.15E+00	2.78E-07	Down	-3.60E+00	2.13E-12	Down	Unigene118373_All//gi|75858833|gb|ABA28993.1|//4.00E-37//polyubiquitin [Symbiodinium sp. C3]	Unigene118373_All//5.00E-37//AT5G03240| UBQ3 (POLYUBIQUITIN 3); protein binding	Unigene118373_All//2.00E-30//TC134297			GO:0043231	nr +
Unigene118374_All	341	1.07	1.19	0.78	0.00	0.61	0.00	0.25	0.25	2.80	1.46E-01	8.98E-01		-4.60E-01	6.97E-01		9.24E+00	1.57E-01		null	null		-1.70E-02	1.01E+00		3.50E+00	4.29E-05	Up			Unigene118374_All//1.00E-47//TC170579				
Unigene118378_All	249	0.21	1.42	0.00	0.20	0.69	0.00	0.00	0.17	2.56	2.76E+00	9.60E-02		-7.71E+00	6.28E-01		1.79E+00	5.21E-01		-7.64E+00	6.39E-01		7.39E+00	6.37E-01		1.13E+01	9.34E-05	Up							
Unigene118384_All	1391	0.00	0.07	0.00	0.07	0.10	0.13	0.52	0.18	4.87	6.19E+00	3.89E-01		null	null		4.68E-01	8.77E-01		8.59E-01	6.96E-01		-1.52E+00	5.42E-02		3.24E+00	3.56E-30	Up	Unigene118384_All//gi|115467526|ref|NP_001057362.1|//5.00E-12//Os06g0271600 [Oryza sativa Japonica Group] >gi|113595402|dbj|BAF19276.1| Os06g0271600 [Oryza sativa Japonica Group]	Unigene118384_All//4.00E-13//AT5G57750| zinc finger (C3HC4-type RING finger) family protein	Unigene118384_All//3.00E-60//TC152784			GO:0003006//GO:0031410//GO:0046872	nr +
Unigene118388_All	330	0.00	0.00	0.00	0.00	0.00	0.00	0.64	1.90	3.50	null	null		null	null		null	null		null	null		1.57E+00	7.10E-02		2.45E+00	1.69E-04	Up							ESTscan +
Unigene11839_All	1307	5.52	15.97	6.76	2.67	3.50	7.33	32.28	94.14	92.24	1.53E+00	1.07E-29	Up	2.92E-01	1.68E-01		3.93E-01	1.84E-01		1.46E+00	8.26E-14	Up	1.54E+00	1.96E-213	Up	1.51E+00	2.00E-207	Up	Unigene11839_All//gi|42572503|ref|NP_974347.1|//1.00E-170//NADK1 (NAD KINASE 1); NAD+ kinase/ NADH kinase/ calmodulin binding [Arabidopsis thaliana] >gi|94717660|sp|Q56YN3.2|NADK1_ARATH RecName: Full=NAD(H) kinase 1; Short=AtNADK-1 >gi|39843359|gb|AAR32133.1| NADK1 [Arabidopsis thaliana]	Unigene11839_All//1.00E-171//AT3G21070| NADK1 (NAD KINASE 1); NAD+ kinase/ NADH kinase/ calmodulin binding	Unigene11839_All//0.00E+00//gi|164361092|gb|ES819069.1|ES819069		ko00760|Nicotinate and nicotinamide metabolism	GO:0016301//GO:0043169//GO:0044424	nr +
Unigene118395_All	302	0.00	0.00	0.00	0.00	0.00	0.30	0.00	0.00	5.01	null	null		null	null		null	null		8.22E+00	4.79E-01		null	null		1.23E+01	6.77E-11	Up							
Unigene118396_All	241	0.00	0.00	0.00	0.00	0.00	0.00	6.84	2.60	0.99	null	null		null	null		null	null		null	null		-1.40E+00	3.12E-03		-2.79E+00	5.06E-07	Down	Unigene118396_All//gi|148524155|gb|ABQ81936.1|//8.00E-20//elongation factor 1-alpha [Chara australis]	Unigene118396_All//3.00E-18//AT5G60390| elongation factor 1-alpha / EF-1-alpha				GO:0004779//GO:0006412//GO:0008135//GO:0017111//GO:0032561//GO:0044424	nr +
Unigene1184_All	2369	1.59	3.57	2.74	3.45	4.59	9.21	24.08	50.76	73.22	1.17E+00	1.68E-08	Up	7.86E-01	1.06E-03		4.14E-01	1.49E-02		1.42E+00	1.46E-29	Up	1.08E+00	2.29E-119	Up	1.60E+00	0.00E+00	Up	Unigene1184_All//gi|4530126|gb|AAD21872.1|//0.00E+00//receptor-like protein kinase homolog RK20-1 [Phaseolus vulgaris]	Unigene1184_All//1.00E-157//AT4G23180| CRK10 (CYSTEINE-RICH RLK10); ATP binding / kinase/ protein kinase/ protein serine/threonine kinase/ protein tyrosine kinase	Unigene1184_All//0.00E+00//TC153623		ko04626|Plant-pathogen interaction	GO:0000166//GO:0004672//GO:0004871	nr +

Unigene11840_All	1851	0.88	0.96	1.32	1.05	1.15	0.68	5.09	2.73	1.64	1.29E-01	7.79E-01		5.95E-01	1.60E-01		1.36E-01	8.19E-01		-6.20E-01	1.97E-01		-8.99E-01	1.03E-07		-1.64E+00	3.75E-19	Down	Unigene11840_All//gi|42572503|ref|NP_974347.1|//0.00E+00//NADK1 (NAD KINASE 1); NAD+ kinase/ NADH kinase/ calmodulin binding [Arabidopsis thaliana] >gi|94717660|sp|Q56YN3.2|NADK1_ARATH RecName: Full=NAD(H) kinase 1; Short=AtNADK-1 >gi|39843359|gb|AAR32133.1| NADK1 [Arabidopsis thaliana]	Unigene11840_All//0.00E+00//AT3G21070| NADK1 (NAD KINASE 1); NAD+ kinase/ NADH kinase/ calmodulin binding	Unigene11840_All//0.00E+00//gi|164361092|gb|ES819069.1|ES819069		ko00760|Nicotinate and nicotinamide metabolism	GO:0005515//GO:0006739//GO:0009108//GO:0016301//GO:0043169//GO:0044424	nr +
Unigene118405_All	664	0.00	0.00	0.00	0.00	0.00	0.95	1.46	1.32	5.46	null	null		null	null		null	null		9.90E+00	7.60E-04	Up	-1.48E-01	8.13E-01		1.90E+00	2.37E-09	Up							
Unigene11841_All	1466	2.75	3.97	1.63	3.13	6.93	10.01	3.20	3.48	3.37	5.32E-01	3.13E-02		-7.50E-01	1.82E-02		1.15E+00	4.06E-11	Up	1.68E+00	1.52E-25	Up	1.20E-01	6.40E-01		7.40E-02	7.68E-01		Unigene11841_All//gi|124360132|gb|ABN08148.1|//3.00E-72//tRNA intron endonuclease, catalytic C-terminal [Medicago truncatula]	Unigene11841_All//6.00E-71//AT3G45590| ATSEN1 (SPLICING ENDONUCLEASE 1); nuclease/ nucleic acid binding / tRNA-intron endonuclease	Unigene11841_All//0.00E+00//TC170905			GO:0000394//GO:0004549//GO:0005488//GO:0043234	nr +
Unigene118412_All	522	0.00	1.07	0.00	0.00	0.00	0.00	0.24	1.12	2.59	1.01E+01	2.19E-03		null	null		null	null		null	null		2.21E+00	2.34E-02		3.42E+00	7.76E-07	Up			Unigene118412_All//5.00E-97//DW491233				
Unigene118421_All	231	0.91	0.66	0.69	37.52	34.44	9.58	3.84	1.81	5.86	-4.61E-01	7.58E-01		-3.90E-01	8.08E-01		-1.24E-01	5.86E-01		-1.97E+00	2.82E-19	Down	-1.09E+00	1.07E-01		6.09E-01	2.25E-01		Unigene118421_All//gi|40217878|gb|AAR82850.1|//8.00E-24//expansin-2 [Petunia x hybrida]	Unigene118421_All//1.00E-22//AT2G39700| ATEXPA4 (ARABIDOPSIS THALIANA EXPANSIN A4)	Unigene118421_All//4.00E-105//TC139820			GO:0007047//GO:0044464	nr +
Unigene118422_All	360	0.15	0.99	0.00	0.28	0.67	0.75	1.41	0.12	8.08	2.76E+00	9.60E-02		-7.18E+00	6.28E-01		1.28E+00	5.32E-01		1.44E+00	4.09E-01		-3.60E+00	5.77E-03		2.52E+00	1.68E-10	Up	Unigene118422_All//gi|225451968|ref|XP_002279750.1|//7.00E-15//PREDICTED: similar to mangrin [Vitis vinifera]	Unigene118422_All//6.00E-09//AT3G25780| AOC3 (ALLENE OXIDE CYCLASE 3); allene-oxide cyclase	Unigene118422_All//5.00E-176//gi|78325914|gb|DT546188.1|DT546188		ko00592|alpha-Linolenic acid metabolism	GO:0009536//GO:0009975	nr -
Unigene118432_All	418	0.25	0.00	0.00	0.00	0.00	0.00	0.00	0.00	2.10	-7.97E+00	3.74E-01		-7.97E+00	3.96E-01		null	null		null	null		null	null		1.10E+01	2.05E-06	Up	Unigene118432_All//gi|170660047|gb|ACB28472.1|//1.00E-46//polyprotein [Ananas comosus]		Unigene118432_All//3.00E-11//CK640452			GO:0003676//GO:0006260//GO:0034061//GO:0043229	nr -
Unigene118434_All	239	0.00	2.12	0.00	0.21	0.00	0.00	0.00	1.40	5.00	1.10E+01	4.05E-03		null	null		-7.70E+00	5.58E-01		-7.70E+00	6.39E-01		1.05E+01	1.27E-02		1.23E+01	1.20E-08	Up							
Unigene118435_All	345	0.00	0.00	0.00	0.00	0.00	0.00	0.12	0.00	2.77	null	null		null	null		null	null		null	null		-6.94E+00	6.60E-01		4.50E+00	6.81E-06	Up			Unigene118435_All//1.00E-37//TC178834				
Unigene118436_All	622	0.00	0.00	0.00	0.00	0.00	0.00	0.00	0.00	1.73	null	null		null	null		null	null		null	null		null	null		1.08E+01	8.30E-08	Up	Unigene118436_All//gi|115473321|ref|NP_001060259.1|//3.00E-09//Os07g0613900 [Oryza sativa Japonica Group] >gi|113611795|dbj|BAF22173.1| Os07g0613900 [Oryza sativa Japonica Group]						nr -
Unigene118443_All	749	0.00	0.00	0.00	0.00	0.00	0.12	0.00	0.11	3.35	null	null		null	null		null	null		6.91E+00	4.79E-01		6.80E+00	4.03E-01		1.17E+01	5.71E-18	Up							
Unigene118447_All	248	20.68	4.09	11.17	3.21	3.61	0.91	0.00	1.35	0.64	-2.34E+00	2.46E-13	Down	-8.89E-01	1.29E-03		1.68E-01	8.32E-01		-1.82E+00	3.32E-02		1.04E+01	1.27E-02		9.33E+00	1.40E-01		Unigene118447_All//gi|255543699|ref|XP_002512912.1|//3.00E-18//conserved hypothetical protein [Ricinus communis] >gi|223547923|gb|EEF49415.1| conserved hypothetical protein [Ricinus communis]		Unigene118447_All//3.00E-53//TC141372				nr +
Unigene11846_All	728	1.80	5.78	4.54	1.03	3.31	2.42	0.81	0.98	2.24	1.68E+00	2.01E-07	Up	1.34E+00	2.75E-04	Up	1.69E+00	6.31E-05	Up	1.24E+00	1.46E-02		2.63E-01	7.21E-01		1.46E+00	1.69E-03		Unigene11846_All//gi|6635256|dbj|BAA88627.1|//4.00E-22//tRNA intron endonuclease [Arabidopsis thaliana] >gi|6635258|dbj|BAA88628.1| tRNA intron endonuclease [Arabidopsis thaliana]	Unigene11846_All//4.00E-23//AT3G45590| ATSEN1 (SPLICING ENDONUCLEASE 1); nuclease/ nucleic acid binding / tRNA-intron endonuclease	Unigene11846_All//0.00E+00//gi|54115565|gb|CO491074.1|CO491074			GO:0000394//GO:0004549//GO:0005488//GO:0043234	nr -
Unigene118463_All	917	0.00	0.00	0.00	0.11	0.23	0.20	0.37	0.18	14.34	null	null		null	null		1.05E+00	6.13E-01		8.59E-01	6.96E-01		-1.02E+00	4.00E-01		5.28E+00	2.49E-81	Up			Unigene118463_All//2.00E-72//TC173686				
Unigene118465_All	1427	0.00	0.00	0.00	0.00	0.00	0.00	0.39	0.00	5.81	null	null		null	null		null	null		null	null		-8.59E+00	4.61E-04	Down	3.91E+00	5.23E-43	Up	Unigene118465_All//gi|224107585|ref|XP_002314526.1|//0.00E+00//flavine-containing monoxygenase [Populus trichocarpa] >gi|222863566|gb|EEF00697.1| flavine-containing monoxygenase [Populus trichocarpa]	Unigene118465_All//2.00E-173//AT1G04610| flavin-containing monooxygenase / FMO (YUCCA3)	Unigene118465_All//5.00E-13//gi|78348897|gb|DT569171.1|DT569171		ko00380|Tryptophan metabolism	GO:0008152//GO:0016709//GO:0030554	nr +
Unigene118467_All	497	0.74	1.02	1.07	1.30	0.97	0.55	2.89	1.77	0.64	4.68E-01	6.11E-01		5.40E-01	6.10E-01		-4.26E-01	6.09E-01		-1.26E+00	1.83E-01		-7.12E-01	1.55E-01		-2.17E+00	6.89E-05	Down	Unigene118467_All//gi|42567229|ref|NP_194611.2|//3.00E-10//zinc finger (HIT type) family protein [Arabidopsis thaliana] >gi|92856590|gb|ABE77404.1| At4g28820 [Arabidopsis thaliana]	Unigene118467_All//2.00E-11//AT4G28820| zinc finger (HIT type) family protein	Unigene118467_All//1.00E-85//TC148315				nr +
Unigene118472_All	252	0.00	0.00	0.00	0.20	0.27	0.00	0.67	0.33	9.49	null	null		null	null		4.67E-01	9.59E-01		-7.63E+00	6.39E-01		-1.02E+00	6.15E-01		3.82E+00	1.35E-12	Up			Unigene118472_All//6.00E-64//FL576842				
Unigene118486_All	489	0.00	0.00	0.00	0.00	0.00	0.00	0.52	0.68	2.53	null	null		null	null		null	null		null	null		3.98E-01	7.24E-01		2.28E+00	1.88E-04	Up							
Unigene118488_All	289	44.36	113.79	63.75	61.02	37.53	71.89	93.34	211.87	87.67	1.36E+00	6.63E-39	Up	5.23E-01	8.43E-05		-7.01E-01	6.63E-09		2.37E-01	6.64E-02		1.18E+00	4.75E-71	Up	-9.03E-02	3.95E-01		Unigene118488_All//gi|4150974|emb|CAA11899.1|//3.00E-08//cystatin [Castanea sativa]	Unigene118488_All//3.00E-07//AT5G12140| ATCYS1 (A. thaliana cystatin-1); cysteine-type endopeptidase inhibitor	Unigene118488_All//2.00E-58//gi|13247918|gb|BF273082.2|BF273082			GO:0004866//GO:0016787	nr -
Unigene11849_All	2156	16.67	14.22	17.83	19.29	23.02	22.58	18.45	9.73	7.65	-2.30E-01	1.29E-02		9.71E-02	3.56E-01		2.55E-01	4.13E-04		2.27E-01	3.25E-03		-9.23E-01	2.82E-31		-1.27E+00	1.71E-53	Down	Unigene11849_All//gi|81074221|gb|ABB55364.1|//0.00E+00//aspartate aminotransferase-like [Solanum tuberosum]	Unigene11849_All//0.00E+00//AT4G31990| ASP5 (ASPARTATE AMINOTRANSFERASE 5); L-aspartate:2-oxoglutarate aminotransferase	Unigene11849_All//0.00E+00//TC144385		ko00270|Cysteine and methionine metabolism//ko00360|Phenylalanine metabolism//ko00710|Carbon fixation in photosynthetic organisms//ko00250|Alanine, aspartate and glutamate metabolism//ko00330|Arginine and proline metabolism//ko00350|Tyrosine metabolism//ko00400|Phenylalanine, tyrosine and tryptophan biosynthesis//ko00960|Tropane, piperidine and pyridine alkaloid biosynthesis//ko00950|Isoquinoline alkaloid biosynthesis	GO:0005576//GO:0006950//GO:0008483//GO:0009064//GO:0009066//GO:0009526//GO:0009532//GO:0010038//GO:0031980//GO:0043648//GO:0048037	nr -
Unigene118491_All	232	0.00	0.00	0.00	0.21	0.15	0.19	2.55	3.60	13.57	null	null		null	null		-5.33E-01	8.27E-01		-1.41E-01	9.54E-01		4.98E-01	4.86E-01		2.41E+00	8.65E-11	Up			Unigene118491_All//2.00E-69//gi|109868468|gb|DW497444.1|DW497444				
Unigene118493_All	464	0.00	0.00	0.00	0.00	0.00	0.00	0.46	2.97	4.38	null	null		null	null		null	null		null	null		2.71E+00	1.42E-05	Up	3.26E+00	1.40E-09	Up			Unigene118493_All//5.00E-07//CO074079				
Unigene118494_All	445	0.00	0.00	0.00	0.00	0.00	0.00	0.00	0.19	2.60	null	null		null	null		null	null		null	null		7.55E+00	4.03E-01		1.13E+01	2.28E-08	Up							
Unigene1185_All	6346	1.10	2.47	2.01	6.04	3.89	5.28	4.10	1.96	3.87	1.17E+00	4.05E-15	Up	8.71E-01	1.29E-07		-6.38E-01	6.19E-16		-1.95E-01	3.24E-02		-1.06E+00	6.43E-26	Down	-8.35E-02	4.45E-01		Unigene1185_All//gi|124360779|gb|ABN08751.1|//0.00E+00//Protein kinase [Medicago truncatula]	Unigene1185_All//0.00E+00//AT5G56890| protein kinase family protein	Unigene1185_All//0.00E+00//TC177762		ko04626|Plant-pathogen interaction	GO:0004672//GO:0006464//GO:0016020//GO:0032559//GO:0043231	nr +
Unigene118509_All	261	0.00	0.00	0.20	0.00	0.00	0.17	2.43	5.12	23.96	null	null		7.67E+00	6.38E-01		null	null		7.43E+00	6.99E-01		1.08E+00	4.02E-02		3.30E+00	2.02E-28	Up	Unigene118509_All//gi|224143055|ref|XP_002324836.1|//5.00E-20//2-oxoglutarate-dependent dioxygenase [Populus trichocarpa] >gi|222866270|gb|EEF03401.1| 2-oxoglutarate-dependent dioxygenase [Populus trichocarpa]	Unigene118509_All//2.00E-15//AT1G52820| 2-oxoglutarate-dependent dioxygenase, putative	Unigene118509_All//5.00E-83//TC142413		ko00270|Cysteine and methionine metabolism//ko00904|Diterpenoid biosynthesis	GO:0008152//GO:0016701//GO:0016706	nr -
Unigene11851_All	3301	9.05	13.42	10.55	14.46	13.67	14.84	13.64	20.83	28.83	5.68E-01	1.37E-12		2.21E-01	2.49E-02		-8.12E-02	3.82E-01		3.82E-02	6.77E-01		6.11E-01	1.83E-26		1.08E+00	2.98E-97	Up	Unigene11851_All//gi|42567457|ref|NP_568003.2|//0.00E+00//FAD linked oxidase family protein [Arabidopsis thaliana] >gi|42573191|ref|NP_974692.1| FAD linked oxidase family protein [Arabidopsis thaliana] >gi|294956519|sp|O23240.3|D2HDH_ARATH RecName: Full=D-2-hydroxyglutarate dehydrogenase, mitochondrial; Short=AtD-2HGDH; Flags: Precursor	Unigene11851_All//0.00E+00//AT4G36400| FAD linked oxidase family protein	Unigene11851_All//0.00E+00//TC170837		ko04146|Peroxisome//ko00620|Pyruvate metabolism	GO:0004457//GO:0008152//GO:0030554//GO:0043231	nr -
Unigene118512_All	275	0.00	0.00	0.97	0.00	0.13	0.16	0.00	0.00	2.32	null	null		9.92E+00	8.03E-02		6.97E+00	8.33E-01		7.36E+00	6.99E-01		null	null		1.12E+01	9.34E-05	Up							
Unigene118513_All	245	0.00	0.00	0.00	0.00	0.00	0.00	0.00	0.00	2.28	null	null		null	null		null	null		null	null		null	null		1.12E+01	3.28E-04	Up							
Unigene11852_All	505	6.94	11.34	11.39	10.26	16.23	14.49	4.35	9.44	6.31	7.08E-01	4.33E-03		7.14E-01	5.56E-03		6.62E-01	5.94E-04		4.98E-01	2.27E-02		1.12E+00	8.85E-06	Up	5.36E-01	7.73E-02		Unigene11852_All//gi|297806241|ref|XP_002871004.1|//1.00E-16//hydrolase, alpha/beta fold family protein [Arabidopsis lyrata subsp. lyrata] >gi|297316841|gb|EFH47263.1| hydrolase, alpha/beta fold family protein [Arabidopsis lyrata subsp. lyrata]	Unigene11852_All//7.00E-18//AT5G02970| hydrolase, alpha/beta fold family protein	Unigene11852_All//0.00E+00//gi|78351500|gb|DT571774.1|DT571774				nr +
Unigene118529_All	223	0.00	0.00	0.00	0.00	0.00	0.00	0.19	0.19	2.86	null	null		null	null		null	null		null	null		-1.68E-02	1.01E+00		3.91E+00	7.66E-04	Up							
Unigene118533_All	458	0.00	0.00	0.00	0.00	0.00	0.20	0.18	0.37	2.17	null	null		null	null		null	null		7.62E+00	4.79E-01		9.84E-01	6.34E-01		3.56E+00	2.43E-05	Up	Unigene118533_All//gi|297838715|ref|XP_002887239.1|//6.00E-34//oxygen binding protein [Arabidopsis lyrata subsp. lyrata] >gi|297333080|gb|EFH63498.1| oxygen binding protein [Arabidopsis lyrata subsp. lyrata]	Unigene118533_All//6.00E-31//AT1G69500| electron carrier/ heme binding / iron ion binding / monooxygenase/ oxygen binding			ko00945|Stilbenoid, diarylheptanoid and gingerol biosynthesis//ko00903|Limonene and pinene degradation	GO:0005506//GO:0006091//GO:0016712//GO:0031410	nr +
Unigene118545_All	243	28.42	11.68	20.16	7.99	5.38	9.48	0.00	0.00	0.00	-1.28E+00	3.99E-08	Down	-4.95E-01	3.54E-02		-5.70E-01	2.20E-01		2.46E-01	6.31E-01		null	null		null	null		Unigene118545_All//gi|115485721|ref|NP_001068004.1|//4.00E-17//Os11g0528500 [Oryza sativa Japonica Group] >gi|77551234|gb|ABA94031.1| Rubredoxin family protein, expressed [Oryza sativa (japonica cultivar-group)] >gi|113645226|dbj|BAF28367.1| Os11g0528500 [Oryza sativa Japonica Group]	Unigene118545_All//6.00E-16//AT5G51010| rubredoxin family protein	Unigene118545_All//6.00E-107//gi|109886952|gb|DW515921.1|DW515921			GO:0006810//GO:0043169//GO:0043231	nr +
Unigene11856_All	1276	2.75	6.19	4.26	12.92	11.85	13.77	32.67	77.30	27.29	1.17E+00	4.78E-08	Up	6.32E-01	1.80E-02		-1.25E-01	4.37E-01		9.15E-02	5.93E-01		1.24E+00	3.26E-123	Up	-2.60E-01	3.60E-04		Unigene11856_All//gi|297812691|ref|XP_002874229.1|//3.00E-34//zinc finger family protein [Arabidopsis lyrata subsp. lyrata] >gi|297320066|gb|EFH50488.1| zinc finger family protein [Arabidopsis lyrata subsp. lyrata]	Unigene11856_All//7.00E-13//AT5G24870| zinc finger (C3HC4-type RING finger) family protein	Unigene11856_All//0.00E+00//TC145984				nr +
Unigene118561_All	722	0.00	0.84	0.00	0.00	0.00	0.13	0.29	0.41	6.73	9.72E+00	1.17E-03		null	null		null	null		6.97E+00	4.79E-01		4.69E-01	7.06E-01		4.52E+00	5.55E-28	Up	Unigene118561_All//gi|224071451|ref|XP_002303466.1|//3.00E-10//predicted protein [Populus trichocarpa] >gi|222840898|gb|EEE78445.1| predicted protein [Populus trichocarpa]		Unigene118561_All//0.00E+00//gi|109885675|gb|DW514645.1|DW514645				nr -
Unigene118562_All	779	0.00	0.00	0.00	0.00	0.00	0.00	2.71	0.43	0.51	null	null		null	null		null	null		null	null		-2.66E+00	4.31E-08	Down	-2.41E+00	1.32E-07	Down	Unigene118562_All//gi|224127106|ref|XP_002329398.1|//1.00E-15//predicted protein [Populus trichocarpa] >gi|222870448|gb|EEF07579.1| predicted protein [Populus trichocarpa]		Unigene118562_All//6.00E-45//TC175720				nr +
Unigene118573_All	490	0.21	0.21	0.43	0.00	0.00	0.00	0.09	0.51	1.79	-4.67E-02	9.90E-01		1.03E+00	5.92E-01		null	null		null	null		2.57E+00	1.53E-01		4.37E+00	2.26E-05	Up	Unigene118573_All//gi|21553445|gb|AAM62538.1|//3.00E-18//bZIP transcription factor-like protein [Arabidopsis thaliana]	Unigene118573_All//1.00E-13//AT4G37730| AtbZIP7 (Arabidopsis thaliana basic leucine-zipper 7); DNA binding / transcription factor		bZIP	ko04626|Plant-pathogen interaction//ko04650|Natural killer cell mediated cytotoxicity	GO:0001071//GO:0003677//GO:0005515//GO:0006351//GO:0043231	nr +

Unigene118576_All	470	0.00	0.00	0.00	0.00	0.00	0.00	0.00	0.27	2.29	null	null		null	null		null	null		null	null		8.06E+00	2.41E-01		1.12E+01	8.30E-08	Up	Unigene118576_All//gi|302142601|emb|CBI19804.3|//6.00E-09//unnamed protein product [Vitis vinifera]	Unigene118576_All//7.00E-06//AT4G16310| LDL3 (LSD1-LIKE3); amine oxidase/ electron carrier/ oxidoreductase	Unigene118576_All//2.00E-16//TC165986			GO:0008152//GO:0016641//GO:0016741	nr +
Unigene118578_All	254	0.00	0.00	0.00	0.20	0.00	0.00	0.00	0.16	2.04	null	null		null	null		-7.62E+00	5.58E-01		-7.62E+00	6.39E-01		7.36E+00	6.37E-01		1.10E+01	6.14E-04	Up							
Unigene11858_All	2809	11.98	16.54	13.29	30.56	24.98	21.38	29.88	65.20	25.02	4.66E-01	1.98E-09		1.50E-01	1.36E-01		-2.90E-01	1.59E-08		-5.15E-01	2.06E-21		1.13E+00	3.23E-195	Up	-2.56E-01	2.65E-07		Unigene11858_All//gi|297812691|ref|XP_002874229.1|//5.00E-86//zinc finger family protein [Arabidopsis lyrata subsp. lyrata] >gi|297320066|gb|EFH50488.1| zinc finger family protein [Arabidopsis lyrata subsp. lyrata]	Unigene11858_All//1.00E-64//AT5G24870| zinc finger (C3HC4-type RING finger) family protein	Unigene11858_All//0.00E+00//TC145984		ko00190|Oxidative phosphorylation	GO:0005488	nr -
Unigene118584_All	349	0.00	0.00	0.00	0.57	0.10	0.52	4.36	0.72	3.88	null	null		null	null		-2.53E+00	2.34E-01		-1.41E-01	9.35E-01		-2.60E+00	6.96E-06	Down	-1.68E-01	7.13E-01				Unigene118584_All//5.00E-28//CO080430				ESTscan +
Unigene1186_All	2238	24.03	11.84	10.87	65.80	59.50	40.66	33.31	20.45	20.60	-1.02E+00	1.52E-40	Down	-1.14E+00	6.13E-47	Down	-1.45E-01	3.68E-04		-6.94E-01	8.83E-62		-7.04E-01	1.33E-36		-6.94E-01	1.06E-36		Unigene1186_All//gi|42571735|ref|NP_973958.1|//1.00E-74//RNA recognition motif (RRM)-containing protein [Arabidopsis thaliana] >gi|14532532|gb|AAK63994.1| At1g33470/F10C21_14 [Arabidopsis thaliana] >gi|18655391|gb|AAL76151.1| At1g33470/F10C21_14 [Arabidopsis thaliana]	Unigene1186_All//5.00E-52//AT1G33470| RNA recognition motif (RRM)-containing protein	Unigene1186_All//0.00E+00//TC167468		ko03040|Spliceosome	GO:0003676	nr -
Unigene118605_All	438	0.12	0.00	0.24	0.00	0.00	0.10	0.10	0.29	1.91	-6.90E+00	6.15E-01		1.03E+00	7.04E-01		null	null		6.69E+00	6.99E-01		1.57E+00	5.46E-01		4.31E+00	4.11E-05	Up	Unigene118605_All//gi|226290819|gb|ACO40485.1|//3.00E-37//terpene synthase [Actinidia deliciosa]	Unigene118605_All//1.00E-19//AT1G79460| GA2 (GA REQUIRING 2); ent-kaurene synthase			ko00904|Diterpenoid biosynthesis	GO:0016838	nr +
Unigene118615_All	483	0.22	15.95	0.00	0.00	0.21	0.47	0.00	0.00	0.49	6.20E+00	1.33E-40	Up	-7.76E+00	3.96E-01		7.74E+00	4.57E-01		8.87E+00	1.19E-01		null	null		8.95E+00	4.40E-02		Unigene118615_All//gi|295393494|gb|ADG03603.1|//7.00E-28//galactinol synthase [Brassica napus]	Unigene118615_All//2.00E-28//AT2G47180| AtGolS1 (Arabidopsis thaliana galactinol synthase 1); transferase, transferring glycosyl groups / transferase, transferring hexosyl groups	Unigene118615_All//1.00E-109//TC156180			GO:0000302//GO:0005975//GO:0009642//GO:0035250	nr +
Unigene11862_All	3083	68.92	176.05	108.56	82.71	180.83	255.19	42.92	143.63	50.82	1.35E+00	0.00E+00	Up	6.56E-01	2.86E-113		1.13E+00	0.00E+00	Up	1.63E+00	0.00E+00	Up	1.74E+00	0.00E+00	Up	2.44E-01	9.73E-12		Unigene11862_All//gi|6682841|dbj|BAA88904.1|//0.00E+00//sucrose synthase [Citrus unshiu]	Unigene11862_All//0.00E+00//AT4G02280| SUS3 (sucrose synthase 3); UDP-glycosyltransferase/ sucrose synthase/ transferase, transferring glycosyl groups	Unigene11862_All//0.00E+00//TC147396		ko00500|Starch and sucrose metabolism	GO:0005985//GO:0006073//GO:0035251//GO:0048316	nr -
Unigene11863_All	1205	3.34	1.68	1.24	7.28	7.00	3.79	7.96	2.25	3.34	-9.91E-01	1.19E-03		-1.43E+00	1.53E-05	Down	-5.55E-02	8.33E-01		-9.43E-01	8.97E-07		-1.82E+00	9.61E-22	Down	-1.25E+00	2.54E-13	Down	Unigene11863_All//gi|54035683|sp|O81221.1|ACT_GOSHI//0.00E+00//RecName: Full=Actin >gi|3420239|gb|AAC31886.1| actin [Gossypium hirsutum]	Unigene11863_All//0.00E+00//AT3G53750| ACT3 (actin 3); structural constituent of cytoskeleton	Unigene11863_All//0.00E+00//gi|48801570|gb|CO102884.1|CO102884		ko04145|Phagosome	GO:0006996//GO:0016020//GO:0030312//GO:0032559//GO:0043231//GO:0043232//GO:0048588	nr +
Unigene118634_All	399	0.00	0.00	0.00	0.00	0.00	0.00	0.00	0.52	6.49	null	null		null	null		null	null		null	null		9.03E+00	7.86E-02		1.27E+01	1.54E-18	Up							
Unigene11864_All	1227	2.09	1.69	1.30	6.74	6.88	3.13	8.20	1.60	2.11	-3.03E-01	4.47E-01		-6.82E-01	1.00E-01		2.89E-02	9.11E-01		-1.11E+00	3.60E-08	Down	-2.36E+00	1.96E-31	Down	-1.96E+00	7.61E-26	Down	Unigene11864_All//gi|54035683|sp|O81221.1|ACT_GOSHI//0.00E+00//RecName: Full=Actin >gi|3420239|gb|AAC31886.1| actin [Gossypium hirsutum]	Unigene11864_All//0.00E+00//AT3G53750| ACT3 (actin 3); structural constituent of cytoskeleton	Unigene11864_All//0.00E+00//TC140956		ko04145|Phagosome	GO:0006996//GO:0016020//GO:0030312//GO:0032559//GO:0043231//GO:0043232//GO:0048588	nr +
Unigene118647_All	609	0.00	0.00	0.00	0.00	0.00	0.00	0.14	0.27	1.64	null	null		null	null		null	null		null	null		9.83E-01	6.34E-01		3.56E+00	2.43E-05	Up	Unigene118647_All//gi|53749404|gb|AAU90262.1|//5.00E-12//integrase core domain containing protein [Oryza sativa Japonica Group]		Unigene118647_All//4.00E-24//gi|195983411|gb|FL576905.1|FL576905			GO:0005488	nr -
Unigene11865_All	1442	5.77	4.50	2.84	19.03	17.00	12.84	15.98	5.88	6.66	-3.59E-01	7.97E-02		-1.02E+00	1.43E-06	Down	-1.63E-01	1.47E-01		-5.68E-01	1.46E-08		-1.44E+00	9.31E-37	Down	-1.26E+00	4.44E-31	Down	Unigene11865_All//gi|54035683|sp|O81221.1|ACT_GOSHI//0.00E+00//RecName: Full=Actin >gi|3420239|gb|AAC31886.1| actin [Gossypium hirsutum]	Unigene11865_All//0.00E+00//AT3G53750| ACT3 (actin 3); structural constituent of cytoskeleton	Unigene11865_All//0.00E+00//TC165291		ko04145|Phagosome	GO:0007010//GO:0016020//GO:0030312//GO:0032559//GO:0043231//GO:0043232//GO:0048588	nr +
Unigene118659_All	246	0.00	0.00	0.00	0.00	0.00	0.00	0.00	0.34	2.11	null	null		null	null		null	null		null	null		8.41E+00	4.03E-01		1.10E+01	6.14E-04	Up							
Unigene118661_All	587	0.00	0.00	0.00	0.00	0.00	0.00	0.22	0.00	3.26	null	null		null	null		null	null		null	null		-7.76E+00	2.36E-01		3.91E+00	2.32E-10	Up	Unigene118661_All//gi|15214248|sp|Q9Y068.1|RIC1_PHYIN//2.00E-10//RecName: Full=Protein Ric1 >gi|5531402|emb|CAB50926.1| RIC1 protein [Phytophthora infestans]	Unigene118661_All//2.00E-07//AT4G30660| hydrophobic protein, putative / low temperature and salt responsive protein, putative				GO:0016020//GO:0031410	nr -
Unigene11867_All	986	9.23	11.36	3.35	13.44	15.02	17.82	28.82	20.64	17.13	2.99E-01	9.02E-02		-1.46E+00	4.98E-12	Down	1.60E-01	3.33E-01		4.07E-01	2.00E-03		-4.81E-01	1.02E-07		-7.50E-01	1.43E-16		Unigene11867_All//gi|226497034|ref|NP_001149714.1|//7.00E-78//LOC100283341 [Zea mays] >gi|195629710|gb|ACG36496.1| adenylate kinase [Zea mays]	Unigene11867_All//2.00E-77//AT2G39270| adenylate kinase family protein	Unigene11867_All//0.00E+00//TC160571		ko00230|Purine metabolism	GO:0006520//GO:0009117//GO:0009532//GO:0016776//GO:0019205//GO:0032559//GO:0048513	nr +
Unigene118670_All	270	0.97	0.38	0.00	0.00	0.26	0.00	0.31	0.15	6.79	-1.37E+00	3.83E-01		-9.92E+00	8.58E-02		7.99E+00	6.12E-01		null	null		-1.02E+00	7.26E-01		4.44E+00	9.60E-11	Up			Unigene118670_All//5.00E-89//CA992796				
Unigene118673_All	342	363.95	527.44	666.40	416.28	467.59	859.42	257.64	317.50	529.67	5.35E-01	4.99E-44		8.73E-01	8.65E-127		1.68E-01	1.16E-05		1.05E+00	2.92E-244	Up	3.01E-01	7.97E-12		1.04E+00	1.92E-173	Up	Unigene118673_All//gi|115460714|ref|NP_001053957.1|//2.00E-37//Os04g0628100 [Oryza sativa Japonica Group] >gi|113565528|dbj|BAF15871.1| Os04g0628100 [Oryza sativa Japonica Group]	Unigene118673_All//6.00E-38//AT5G03240| UBQ3 (POLYUBIQUITIN 3); protein binding	Unigene118673_All//3.00E-177//gi|13352460|gb|BG442808.1|BG442808			GO:0031410	nr -
Unigene118683_All	976	0.11	0.16	0.00	0.00	0.00	0.00	0.00	0.00	3.84	5.39E-01	7.79E-01		-6.74E+00	3.96E-01		null	null		null	null		null	null		1.19E+01	8.55E-27	Up							
Unigene118690_All	349	0.00	0.00	0.00	0.00	0.00	0.00	0.00	0.00	1.94	null	null		null	null		null	null		null	null		null	null		1.09E+01	4.97E-05	Up	Unigene118690_All//gi|113129056|gb|ABI30334.1|//1.00E-08//Cys-3-His zinc finger protein [Capsicum annuum]	Unigene118690_All//2.00E-06//AT2G40140| CZF1; transcription factor	Unigene118690_All//1.00E-07//DW226743	C3H		GO:0046872	nr +
Unigene118692_All	1753	0.00	0.00	0.00	0.00	0.00	0.00	0.34	0.00	0.23	null	null		null	null		null	null		null	null		-8.40E+00	2.39E-04	Down	-5.71E-01	4.78E-01		Unigene118692_All//gi|224139828|ref|XP_002323296.1|//1.00E-129//cytochrome P450 [Populus trichocarpa] >gi|222867926|gb|EEF05057.1| cytochrome P450 [Populus trichocarpa]	Unigene118692_All//1.00E-119//AT2G42250| CYP712A1; electron carrier/ heme binding / iron ion binding / monooxygenase/ oxygen binding			ko00945|Stilbenoid, diarylheptanoid and gingerol biosynthesis//ko00903|Limonene and pinene degradation//ko00941|Flavonoid biosynthesis//ko00944|Flavone and flavonol biosynthesis		nr -
Unigene118697_All	236	0.00	0.00	0.00	0.00	0.00	0.00	0.00	0.18	3.38	null	null		null	null		null	null		null	null		7.47E+00	6.37E-01		1.17E+01	7.36E-06	Up	Unigene118697_All//gi|297830676|ref|XP_002883220.1|//1.00E-07//binding protein [Arabidopsis lyrata subsp. lyrata] >gi|297329060|gb|EFH59479.1| binding protein [Arabidopsis lyrata subsp. lyrata]	Unigene118697_All//6.00E-09//AT3G19990| unknown protein				GO:0009532	nr -
Unigene1187_All	1501	4.88	2.77	2.09	7.60	6.58	5.33	9.30	4.09	10.80	-8.18E-01	1.55E-04		-1.22E+00	1.35E-07	Down	-2.07E-01	2.62E-01		-5.13E-01	1.98E-03		-1.18E+00	7.59E-17	Down	2.17E-01	8.87E-02		Unigene1187_All//gi|115436734|ref|NP_001043123.1|//1.00E-168//Os01g0496900 [Oryza sativa Japonica Group] >gi|113532654|dbj|BAF05037.1| Os01g0496900 [Oryza sativa Japonica Group]	Unigene1187_All//0.00E+00//AT3G16200| unknown protein	Unigene1187_All//0.00E+00//gi|78330964|gb|DT551238.1|DT551238			GO:0043231	nr +
Unigene118700_All	477	0.00	0.42	0.00	0.00	0.00	0.00	0.00	0.18	1.42	8.73E+00	1.37E-01		null	null		null	null		null	null		7.45E+00	4.03E-01		1.05E+01	4.97E-05	Up	Unigene118700_All//gi|297725715|ref|NP_001175221.1|//1.00E-19//Os07g0513300 [Oryza sativa Japonica Group] >gi|255677803|dbj|BAH93949.1| Os07g0513300 [Oryza sativa Japonica Group]	Unigene118700_All//6.00E-19//AT1G48120| calcineurin-like phosphoesterase family protein	Unigene118700_All//6.00E-07//gi|78334205|gb|DT554479.1|DT554479				nr +
Unigene118706_All	1361	0.00	0.00	0.00	0.00	0.00	0.00	0.00	0.03	5.77	null	null		null	null		null	null		null	null		4.94E+00	6.37E-01		1.25E+01	3.11E-56	Up	Unigene118706_All//gi|297793719|ref|XP_002864744.1|//2.00E-45//myb family transcription factor [Arabidopsis lyrata subsp. lyrata] >gi|297310579|gb|EFH41003.1| myb family transcription factor [Arabidopsis lyrata subsp. lyrata]	Unigene118706_All//1.00E-46//AT5G61620| myb family transcription factor		MYB-related	ko04712|Circadian rhythm - plant	GO:0005488	nr +
Unigene118711_All	258	0.00	0.00	0.00	0.39	0.27	1.40	0.00	0.65	2.32	null	null		null	null		-5.33E-01	8.17E-01		1.86E+00	2.20E-01		9.34E+00	1.39E-01		1.12E+01	1.76E-04	Up							
Unigene118723_All	1147	0.00	0.00	0.00	0.00	0.00	0.00	0.04	0.15	2.54	null	null		null	null		null	null		null	null		1.98E+00	3.79E-01		6.10E+00	2.88E-19	Up							
Unigene118724_All	399	0.00	0.00	0.00	0.00	0.00	0.00	0.74	0.73	4.39	null	null		null	null		null	null		null	null		-1.66E-02	1.02E+00		2.57E+00	9.62E-07	Up							
Unigene11873_All	810	7.30	6.82	5.19	9.84	4.76	4.85	5.17	4.64	2.85	-9.83E-02	6.91E-01		-4.91E-01	5.68E-02		-1.05E+00	4.49E-08	Down	-1.02E+00	5.35E-07	Down	-1.54E-01	6.33E-01		-8.57E-01	8.55E-04		Unigene11873_All//gi|145322873|ref|NP_001030648.2|//3.00E-22//zinc finger (C3HC4-type RING finger) family protein [Arabidopsis thaliana]	Unigene11873_All//2.00E-17//AT3G06330| zinc finger (C3HC4-type RING finger) family protein	Unigene11873_All//5.00E-138//gi|73852414|gb|DT461434.1|DT461434			GO:0009536//GO:0019787//GO:0046914	nr -
Unigene118730_All	409	0.13	0.25	0.52	0.85	0.25	0.22	0.10	0.31	1.85	9.54E-01	7.27E-01		2.03E+00	3.62E-01		-1.76E+00	1.90E-01		-1.95E+00	1.96E-01		1.57E+00	5.46E-01		4.16E+00	1.34E-04	Up	Unigene118730_All//gi|224126033|ref|XP_002319739.1|//8.00E-19//chromatin remodeling complex subunit [Populus trichocarpa] >gi|222858115|gb|EEE95662.1| chromatin remodeling complex subunit [Populus trichocarpa]	Unigene118730_All//8.00E-20//AT3G54280| RGD3 (ROOT GROWTH DEFECTIVE 3); ATP binding / DNA binding / binding / helicase/ nucleic acid binding	Unigene118730_All//2.00E-105//DW484463			GO:0003676//GO:0010467//GO:0017111//GO:0032559//GO:0034062//GO:0043231	nr -
Unigene118733_All	683	0.08	0.45	0.55	0.36	0.96	0.20	0.87	1.53	2.68	2.54E+00	1.51E-01		2.83E+00	9.31E-02		1.39E+00	1.51E-01		-8.78E-01	6.16E-01		8.20E-01	1.79E-01		1.63E+00	2.55E-04	Up			Unigene118733_All//8.00E-44//CO110587				
Unigene118744_All	400	0.00	0.25	0.00	0.00	0.17	0.00	0.11	0.31	5.98	7.98E+00	3.89E-01		null	null		7.43E+00	6.12E-01		null	null		1.57E+00	5.46E-01		5.82E+00	1.17E-15	Up			Unigene118744_All//7.00E-40//TC162362				
Unigene118748_All	499	0.00	0.00	0.00	0.10	0.00	0.00	0.00	0.34	1.60	null	null		null	null		-6.64E+00	5.58E-01		-6.64E+00	6.39E-01		8.39E+00	1.39E-01		1.06E+01	7.36E-06	Up	Unigene118748_All//gi|115485947|ref|NP_001068117.1|//4.00E-28//Os11g0569500 [Oryza sativa Japonica Group] >gi|113645339|dbj|BAF28480.1| Os11g0569500 [Oryza sativa Japonica Group]	Unigene118748_All//3.00E-13//AT3G47570| leucine-rich repeat transmembrane protein kinase, putative			ko04626|Plant-pathogen interaction	GO:0004672//GO:0005488//GO:0044464	nr +
Unigene11875_All	341	0.31	0.89	0.00	0.58	1.72	0.00	0.99	3.80	0.93	1.54E+00	3.23E-01		-8.26E+00	3.96E-01		1.55E+00	1.41E-01		-9.19E+00	1.34E-01		1.94E+00	8.28E-04	Up	-8.58E-02	9.47E-01			Unigene11875_All//2.00E-05//AT5G09660| PMDH2 (peroxisomal NAD-malate dehydrogenase 2); malate dehydrogenase	Unigene11875_All//7.00E-12//TC164570				
Unigene118754_All	991	0.32	0.26	0.75	0.20	0.14	0.73	0.94	0.00	0.24	-3.10E-01	7.89E-01		1.25E+00	1.54E-01		-5.33E-01	7.49E-01		1.86E+00	4.71E-02		-9.87E+00	1.14E-06	Down	-1.96E+00	3.98E-03		Unigene118754_All//gi|30697152|ref|NP_851217.1|//5.00E-95//GIL1 (GRAVITROPIC IN THE LIGHT) [Arabidopsis thaliana]	Unigene118754_All//5.00E-93//AT5G58960| GIL1 (GRAVITROPIC IN THE LIGHT)	Unigene118754_All//0.00E+00//TC146888			GO:0009416//GO:0009630	nr +
Unigene118758_All	373	0.00	0.00	0.00	0.00	0.00	0.00	0.23	0.34	2.99	null	null		null	null		null	null		null	null		5.69E-01	7.83E-01		3.72E+00	4.35E-06	Up			Unigene118758_All//5.00E-25//gi|78330320|gb|DT550594.1|DT550594				
Unigene118767_All	491	0.00	0.00	0.00	0.00	0.14	1.47	0.00	0.85	6.00	null	null		null	null		7.13E+00	6.12E-01		1.05E+01	2.31E-04	Up	9.73E+00	3.65E-03		1.26E+01	4.24E-21	Up							
Unigene118772_All	680	0.00	0.00	0.00	0.00	0.00	0.00	0.00	0.37	2.23	null	null		null	null		null	null		null	null		8.53E+00	4.31E-02		1.11E+01	6.77E-11	Up	Unigene118772_All//gi|297843370|ref|XP_002889566.1|//1.00E-60//lipase class 3 family protein [Arabidopsis lyrata subsp. lyrata] >gi|297335408|gb|EFH65825.1| lipase class 3 family protein [Arabidopsis lyrata subsp. lyrata]	Unigene118772_All//2.00E-61//AT1G05800| DGL (DONGLE); triacylglycerol lipase				GO:0004091//GO:0006641//GO:0006952//GO:0009532//GO:0009668//GO:0009694//GO:0010260//GO:0016049	nr -
Unigene118775_All	289	0.00	0.00	0.37	0.00	0.60	0.16	0.15	0.14	10.06	null	null		8.53E+00	4.00E-01		9.22E+00	2.27E-01		7.29E+00	6.99E-01		-1.69E-02	1.01E+00		6.10E+00	2.88E-19	Up			Unigene118775_All//7.00E-30//DR463937				
Unigene118790_All	274	0.00	0.00	0.00	0.00	0.00	0.00	0.00	0.61	2.04	null	null		null	null		null	null		null	null		9.25E+00	1.39E-01		1.10E+01	3.28E-04	Up							
Unigene118799_All	996	0.16	0.00	0.00	0.15	0.17	0.09	0.08	0.21	2.72	-7.30E+00	2.16E-01		-7.30E+00	2.45E-01		2.05E-01	9.50E-01		-7.26E-01	7.16E-01		1.31E+00	4.47E-01		5.00E+00	1.18E-16	Up	Unigene118799_All//gi|33465891|gb|AAQ19327.1|//4.00E-11//bZIP-like protein [Oryza sativa Japonica Group]	Unigene118799_All//4.00E-06//AT2G01050| nucleic acid binding / zinc ion binding				GO:0005488	nr -

Unigene1188_All	1563	16.03	8.33	8.89	21.86	18.86	17.74	10.87	4.25	6.14	-9.45E-01	4.86E-17		-8.51E-01	8.77E-14		-2.13E-01	2.07E-02		-3.01E-01	9.88E-04		-1.35E+00	6.13E-25	Down	-8.24E-01	6.34E-12		Unigene1188_All//gi|115436734|ref|NP_001043123.1|//1.00E-170//Os01g0496900 [Oryza sativa Japonica Group] >gi|113532654|dbj|BAF05037.1| Os01g0496900 [Oryza sativa Japonica Group]	Unigene1188_All//0.00E+00//AT3G16200| unknown protein	Unigene1188_All//0.00E+00//TC132326			GO:0043231	nr +
Unigene118804_All	1392	0.04	0.11	0.00	0.07	0.02	0.00	0.21	0.27	11.33	1.54E+00	5.32E-01		-5.23E+00	6.27E-01		-1.54E+00	5.87E-01		-6.16E+00	4.02E-01		3.46E-01	7.38E-01		5.74E+00	4.83E-101	Up	Unigene118804_All//gi|224079880|ref|XP_002305957.1|//5.00E-08//histone acetyltransferase [Populus trichocarpa] >gi|222848921|gb|EEE86468.1| histone acetyltransferase [Populus trichocarpa]						nr +
Unigene11882_All	799	1.64	0.63	0.53	2.31	3.79	1.36	2.59	2.09	0.80	-1.37E+00	2.40E-02		-1.62E+00	1.33E-02		7.17E-01	4.36E-02		-7.66E-01	1.18E-01		-3.09E-01	4.71E-01		-1.70E+00	4.43E-05	Down	Unigene11882_All//gi|22326882|ref|NP_197349.2|//1.00E-70//PGSIP6 (PLANT GLYCOGENIN-LIKE STARCH INITIATION PROTEIN 6); transferase, transferring glycosyl groups / transferase, transferring hexosyl groups [Arabidopsis thaliana]	Unigene11882_All//4.00E-65//AT5G18480| PGSIP6 (PLANT GLYCOGENIN-LIKE STARCH INITIATION PROTEIN 6); transferase, transferring glycosyl groups / transferase, transferring hexosyl groups	Unigene11882_All//0.00E+00//EE593156			GO:0005622//GO:0016758//GO:0044238	nr -
Unigene11883_All	1311	1.20	0.39	1.10	2.28	3.15	2.62	3.58	1.28	0.94	-1.63E+00	3.58E-03		-1.27E-01	8.07E-01		4.67E-01	1.30E-01		2.00E-01	6.28E-01		-1.49E+00	1.80E-08	Down	-1.93E+00	3.07E-12	Down	Unigene11883_All//gi|22326882|ref|NP_197349.2|//1.00E-147//PGSIP6 (PLANT GLYCOGENIN-LIKE STARCH INITIATION PROTEIN 6); transferase, transferring glycosyl groups / transferase, transferring hexosyl groups [Arabidopsis thaliana]	Unigene11883_All//3.00E-133//AT5G18480| PGSIP6 (PLANT GLYCOGENIN-LIKE STARCH INITIATION PROTEIN 6); transferase, transferring glycosyl groups / transferase, transferring hexosyl groups	Unigene11883_All//0.00E+00//EE593156			GO:0031410	nr -
Unigene118835_All	519	0.00	0.00	0.00	0.00	0.00	0.00	0.73	0.89	4.45	null	null		null	null		null	null		null	null		2.73E-01	7.69E-01		2.60E+00	8.88E-09	Up	Unigene118835_All//gi|15241735|ref|NP_198755.1|//1.00E-19//leucine-rich repeat family protein / protein kinase family protein [Arabidopsis thaliana]	Unigene118835_All//3.00E-21//AT5G39390| leucine-rich repeat family protein / protein kinase family protein	Unigene118835_All//2.00E-44//TC151383		ko04626|Plant-pathogen interaction	GO:0004672//GO:0006464//GO:0016491//GO:0031224//GO:0032559	nr -
Unigene118837_All	430	0.00	0.00	0.00	0.00	0.00	0.00	0.00	0.00	1.48	null	null		null	null		null	null		null	null		null	null		1.05E+01	9.34E-05	Up							
Unigene118841_All	845	0.12	0.00	0.00	0.24	0.00	0.00	1.20	0.49	3.54	-6.95E+00	3.74E-01		-6.95E+00	3.96E-01		-7.88E+00	8.45E-02		-7.88E+00	1.34E-01		-1.28E+00	4.06E-02		1.56E+00	3.58E-06	Up			Unigene118841_All//2.00E-76//gi|31072987|gb|CA993266.1|CA993266				
Unigene118847_All	531	0.30	1.34	0.40	0.47	0.19	0.34	0.40	0.47	2.10	2.18E+00	2.50E-02		4.40E-01	7.76E-01		-1.27E+00	4.24E-01		-4.64E-01	7.45E-01		2.47E-01	8.53E-01		2.40E+00	2.76E-04	Up			Unigene118847_All//3.00E-46//DT048530				
Unigene11885_All	787	6.71	7.92	4.80	25.26	27.30	16.87	14.67	4.89	5.72	2.38E-01	3.55E-01		-4.83E-01	7.93E-02		1.13E-01	4.29E-01		-5.82E-01	1.20E-06		-1.59E+00	1.58E-21	Down	-1.36E+00	8.22E-18	Down	Unigene11885_All//gi|16226792|gb|AAL16263.1|AF428333_1//1.00E-66//At1g35780/F14D7_9 [Arabidopsis thaliana]	Unigene11885_All//4.00E-67//AT1G35780| unknown protein	Unigene11885_All//0.00E+00//gi|78332729|gb|DT553003.1|DT553003			GO:0003676	nr +
Unigene11886_All	1504	4.52	2.43	2.09	13.94	13.71	10.93	12.17	4.36	2.78	-8.99E-01	7.23E-05		-1.11E+00	3.12E-06	Down	-2.39E-02	8.67E-01		-3.51E-01	3.40E-03		-1.48E+00	1.61E-30	Down	-2.13E+00	1.30E-51	Down	Unigene11886_All//gi|227202770|dbj|BAH56858.1|//3.00E-99//AT1G78150 [Arabidopsis thaliana]	Unigene11886_All//6.00E-100//AT1G78150| unknown protein	Unigene11886_All//0.00E+00//TC163710				nr -
Unigene118860_All	1081	0.15	0.00	0.25	0.00	0.13	0.08	0.70	0.31	2.95	-7.18E+00	2.16E-01		7.62E-01	6.44E-01		6.99E+00	3.19E-01		6.39E+00	4.79E-01		-1.19E+00	1.12E-01		2.07E+00	3.44E-09	Up	Unigene118860_All//gi|399213|sp|P31542.1|CLPAB_SOLLC//1.00E-117//RecName: Full=ATP-dependent Clp protease ATP-binding subunit clpA homolog CD4B, chloroplastic; Flags: Precursor >gi|170435|gb|AAA34161.1| ATP-dependent protease (CD4B) [Solanum lycopersicum]	Unigene118860_All//5.00E-111//AT5G50920| CLPC1; ATP binding / ATP-dependent peptidase/ ATPase	Unigene118860_All//3.00E-152//CK640493				nr +
Unigene118864_All	377	0.14	0.13	0.00	0.66	0.18	0.12	0.34	1.00	2.32	-4.65E-02	9.86E-01		-7.12E+00	6.27E-01		-1.85E+00	2.75E-01		-2.46E+00	2.24E-01		1.57E+00	1.95E-01		2.79E+00	5.37E-04	Up							
Unigene11887_All	1630	15.60	9.01	8.40	23.99	24.91	17.12	29.78	7.95	9.34	-7.91E-01	5.49E-13		-8.94E-01	3.81E-15		5.41E-02	5.98E-01		-4.87E-01	3.70E-09		-1.91E+00	9.25E-114	Down	-1.67E+00	6.29E-98	Down	Unigene11887_All//gi|227202770|dbj|BAH56858.1|//2.00E-90//AT1G78150 [Arabidopsis thaliana]	Unigene11887_All//1.00E-91//AT1G78150| unknown protein	Unigene11887_All//0.00E+00//gi|78323239|gb|DT543513.1|DT543513				nr -
Unigene11888_All	1277	1.27	4.48	2.67	4.02	4.37	4.49	3.71	2.52	2.28	1.82E+00	7.41E-11	Up	1.07E+00	2.37E-03		1.21E-01	6.96E-01		1.61E-01	6.01E-01		-5.57E-01	2.48E-02		-7.03E-01	3.17E-03		Unigene11888_All//gi|9454542|gb|AAF87865.1|AC022520_9//1.00E-146//Unknown protein [Arabidopsis thaliana]	Unigene11888_All//5.00E-141//AT1G53040| unknown protein	Unigene11888_All//0.00E+00//ES797722				nr -
Unigene118886_All	311	0.00	0.00	0.00	0.00	0.00	0.00	0.00	0.13	3.71	null	null		null	null		null	null		null	null		7.07E+00	6.37E-01		1.19E+01	2.28E-08	Up			Unigene118886_All//4.00E-07//TC171906				
Unigene11889_All	2064	0.94	1.74	2.01	5.16	5.14	5.91	5.98	4.66	4.19	8.94E-01	6.08E-03		1.10E+00	4.78E-04	Up	-7.30E-03	9.67E-01		1.94E-01	2.99E-01		-3.61E-01	1.37E-02		-5.14E-01	2.27E-04		Unigene11889_All//gi|9454542|gb|AAF87865.1|AC022520_9//1.00E-179//Unknown protein [Arabidopsis thaliana]	Unigene11889_All//1.00E-170//AT1G53040| unknown protein	Unigene11889_All//0.00E+00//gi|78344783|gb|DT565057.1|DT565057				nr -
Unigene118894_All	857	0.00	0.00	0.00	0.00	0.00	0.32	0.20	0.83	3.07	null	null		null	null		null	null		8.30E+00	7.04E-02		2.07E+00	1.45E-02		3.96E+00	3.85E-14	Up	Unigene118894_All//gi|297722269|ref|NP_001173498.1|//3.00E-08//Os03g0565600 [Oryza sativa Japonica Group] >gi|255674640|dbj|BAH92226.1| Os03g0565600 [Oryza sativa Japonica Group]	Unigene118894_All//2.00E-08//AT4G21790| TOM1 (TOBAMOVIRUS MULTIPLICATION 1); protein binding				GO:0044464	nr +
Unigene118896_All	293	0.00	0.00	0.00	0.00	0.00	0.00	0.00	0.71	2.04	null	null		null	null		null	null		null	null		9.48E+00	7.86E-02		1.10E+01	1.76E-04	Up							
Unigene118898_All	505	0.00	0.00	0.42	0.00	0.41	0.18	0.08	0.25	1.81	null	null		8.72E+00	1.41E-01		8.68E+00	1.57E-01		7.48E+00	4.79E-01		1.57E+00	5.46E-01		4.44E+00	1.24E-05	Up	Unigene118898_All//gi|32492359|emb|CAE05990.1|//3.00E-28//OSJNBa0004L19.22 [Oryza sativa (japonica cultivar-group)]					GO:0003676//GO:0009987//GO:0043170//GO:0044238	nr +
Unigene118902_All	385	0.00	0.00	0.00	0.00	0.00	0.00	0.00	0.00	1.66	null	null		null	null		null	null		null	null		null	null		1.07E+01	9.34E-05	Up	Unigene118902_All//gi|15225286|ref|NP_180201.1|//7.00E-09//ER (ERECTA); transmembrane receptor protein kinase [Arabidopsis thaliana] >gi|75319658|sp|Q42371.1|ERECT_ARATH RecName: Full=LRR receptor-like serine/threonine-protein kinase ERECTA; AltName: Full=Protein QUANTITATIVE RESISTANCE TO PLECTOSPHAERELLA 1; AltName: Full=Protein QUANTITATIVE RESISTANCE TO RALSTONIA SOLANACEARUM 1; AltName: Full=Protein TRANSPIRATION EFFICIENCY 1; Flags: Precursor >gi|1345132|gb|AAC49302.1| ERECTA [Arabidopsis thaliana] >gi|1389566|dbj|BAA11869.1| receptor protein kinase [Arabidopsis thaliana] >gi|224589525|gb|ACN59296.1| leucine-rich repeat receptor-like protein kinase [Arabidopsis thaliana]	Unigene118902_All//4.00E-10//AT2G26330| ER (ERECTA); transmembrane receptor protein kinase			ko04626|Plant-pathogen interaction	GO:0004672//GO:0006952//GO:0009886//GO:0009887//GO:0016043//GO:0043231//GO:0050794	nr -
Unigene118904_All	1164	0.00	0.00	0.00	0.00	0.00	0.00	0.29	0.25	3.46	null	null		null	null		null	null		null	null		-2.10E-01	8.50E-01		3.57E+00	9.17E-20	Up	Unigene118904_All//gi|209970600|gb|ACJ03063.1|//6.00E-78//M18-6p [Malus floribunda]	Unigene118904_All//1.00E-34//AT5G27060| AtRLP53 (Receptor Like Protein 53); kinase/ protein binding			ko04626|Plant-pathogen interaction		nr +
Unigene118905_All	318	0.00	0.00	0.00	0.00	0.00	0.85	0.13	1.45	3.38	null	null		null	null		null	null		9.73E+00	7.04E-02		3.44E+00	1.16E-02		4.67E+00	1.11E-06	Up							
Unigene118907_All	468	0.00	0.00	0.00	0.11	0.00	0.00	0.36	0.09	4.43	null	null		null	null		-6.73E+00	5.58E-01		-6.73E+00	6.39E-01		-2.02E+00	3.68E-01		3.61E+00	1.45E-10	Up							
Unigene118910_All	587	0.00	0.00	0.00	0.00	0.00	0.00	0.00	0.07	1.49	null	null		null	null		null	null		null	null		6.15E+00	6.37E-01		1.05E+01	2.05E-06	Up							
Unigene118914_All	427	0.00	0.00	0.00	0.00	0.00	0.00	0.79	0.59	4.29	null	null		null	null		null	null		null	null		-4.32E-01	7.03E-01		2.44E+00	1.14E-06	Up							
Unigene11892_All	1966	4.39	2.19	2.55	9.91	9.77	10.04	13.72	6.36	8.04	-1.00E+00	5.57E-07	Down	-7.86E-01	1.20E-04		-1.96E-02	8.97E-01		1.91E-02	9.16E-01		-1.11E+00	8.45E-29	Down	-7.70E-01	1.79E-16		Unigene11892_All//gi|297820766|ref|XP_002878266.1|//0.00E+00//UDP-galactose transporter 6 [Arabidopsis lyrata subsp. lyrata] >gi|297324104|gb|EFH54525.1| UDP-galactose transporter 6 [Arabidopsis lyrata subsp. lyrata]	Unigene11892_All//3.00E-180//AT2G43240| nucleotide-sugar transmembrane transporter/ sugar:hydrogen symporter	Unigene11892_All//0.00E+00//TC154914			GO:0005402//GO:0008643//GO:0012505//GO:0015144//GO:0031224	nr -
Unigene118935_All	426	0.00	0.00	0.00	0.00	0.00	0.00	0.00	0.00	2.06	null	null		null	null		null	null		null	null		null	null		1.10E+01	2.05E-06	Up	Unigene118935_All//gi|4996363|dbj|BAA78424.1|//6.00E-19//polyprotein [Arabidopsis thaliana]	Unigene118935_All//6.00E-11//AT4G23160| protein kinase family protein	Unigene118935_All//3.00E-30//ES812448			GO:0003676	nr -
Unigene118936_All	228	0.00	0.00	0.47	0.44	0.15	0.20	0.93	2.20	4.54	null	null		8.87E+00	4.00E-01		-1.53E+00	5.87E-01		-1.14E+00	6.77E-01		1.25E+00	2.04E-01		2.29E+00	7.17E-04	Up			Unigene118936_All//1.00E-30//CO113944				
Unigene11894_All	620	24.73	15.85	12.28	11.89	13.16	4.66	2.86	1.89	1.99	-6.41E-01	6.77E-06		-1.01E+00	2.39E-11	Down	1.47E-01	5.42E-01		-1.35E+00	7.94E-10	Down	-6.02E-01	1.78E-01		-5.24E-01	2.19E-01		Unigene11894_All//gi|297852222|ref|XP_002893992.1|//6.00E-39//universal stress protein family protein [Arabidopsis lyrata subsp. lyrata] >gi|297339834|gb|EFH70251.1| universal stress protein family protein [Arabidopsis lyrata subsp. lyrata]	Unigene11894_All//3.00E-38//AT1G44760| universal stress protein (USP) family protein	Unigene11894_All//0.00E+00//TC176468				nr +
Unigene118940_All	534	0.00	0.00	0.00	0.09	0.00	0.00	0.08	0.00	2.98	null	null		null	null		-6.54E+00	5.58E-01		-6.54E+00	6.39E-01		-6.31E+00	6.60E-01		5.24E+00	3.60E-10	Up							
Unigene118948_All	403	0.00	0.00	0.00	0.00	0.00	0.00	0.21	0.93	2.67	null	null		null	null		null	null		null	null		2.15E+00	9.45E-02		3.67E+00	7.76E-06	Up	Unigene118948_All//gi|297601110|ref|NP_001050390.2|//8.00E-25//Os03g0422800 [Oryza sativa Japonica Group] >gi|108708887|gb|ABF96682.1| D-mannose binding lectin family protein, expressed [Oryza sativa (japonica cultivar-group)] >gi|255674599|dbj|BAF12304.2| Os03g0422800 [Oryza sativa Japonica Group]	Unigene118948_All//1.00E-17//AT5G24080| protein kinase family protein	Unigene118948_All//1.00E-10//CO079258			GO:0004672//GO:0006464//GO:0008037//GO:0030246//GO:0032559	nr +
Unigene11895_All	461	11.58	8.46	5.20	3.35	7.84	1.37	3.76	1.45	4.06	-4.52E-01	8.33E-02		-1.16E+00	2.65E-05	Down	1.23E+00	1.09E-04	Up	-1.29E+00	1.75E-02		-1.37E+00	2.67E-03		1.11E-01	7.90E-01		Unigene11895_All//gi|157849662|gb|ABV89614.1|//8.00E-16//universal stress protein family protein [Brassica rapa]	Unigene11895_All//9.00E-11//AT1G44760| universal stress protein (USP) family protein	Unigene11895_All//0.00E+00//ES792017			GO:0050896	nr -
Unigene118958_All	977	0.00	0.00	0.00	0.00	0.00	0.05	0.82	0.94	2.69	null	null		null	null		null	null		5.53E+00	6.98E-01		1.95E-01	7.62E-01		1.71E+00	3.42E-06	Up			Unigene118958_All//3.00E-72//ES836194				
Unigene118959_All	277	0.00	0.00	0.00	0.00	0.00	0.00	0.46	3.17	0.86	null	null		null	null		null	null		null	null		2.79E+00	6.97E-04	Up	9.14E-01	5.42E-01		Unigene118959_All//gi|229893769|gb|ACQ90242.1|//6.00E-20//cinnamyl alcohol dehydrogenase [Glycine max]	Unigene118959_All//1.00E-18//AT4G37980| ELI3-1 (ELICITOR-ACTIVATED GENE 3-1); binding / catalytic/ oxidoreductase/ zinc ion binding	Unigene118959_All//1.00E-31//TC179502		ko00940|Phenylpropanoid biosynthesis	GO:0008152//GO:0016616//GO:0046914	nr +
Unigene11896_All	741	5.15	4.10	2.52	3.09	1.77	2.93	3.31	2.59	1.45	-3.29E-01	3.15E-01		-1.04E+00	1.71E-03		-8.08E-01	4.44E-02		-8.00E-02	8.60E-01		-3.51E-01	3.73E-01		-1.19E+00	8.55E-04	Down	Unigene11896_All//gi|38603890|gb|AAR24690.1|//1.00E-23//At1g48200 [Arabidopsis thaliana] >gi|38603976|gb|AAR24731.1| At1g48200 [Arabidopsis thaliana]	Unigene11896_All//1.00E-20//AT1G48200| unknown protein	Unigene11896_All//0.00E+00//gi|109872156|gb|DW501132.1|DW501132				nr +
Unigene118960_All	231	0.00	0.00	0.92	0.22	0.15	0.00	0.18	1.09	3.10	null	null		9.85E+00	1.41E-01		-5.32E-01	8.26E-01		-7.75E+00	6.39E-01		2.57E+00	1.53E-01		4.08E+00	2.41E-04	Up			Unigene118960_All//1.00E-27//CO108330				
Unigene118961_All	312	0.00	0.00	0.00	0.32	0.00	0.43	0.95	3.88	7.41	null	null		null	null		-8.32E+00	3.15E-01		4.44E-01	8.62E-01		2.03E+00	8.61E-04	Up	2.96E+00	6.16E-10	Up	Unigene118961_All//gi|225452181|ref|XP_002265308.1|//4.00E-11//PREDICTED: similar to LHT1 (LYSINE HISTIDINE TRANSPORTER 1); amino acid transmembrane transporter [Vitis vinifera]	Unigene118961_All//1.00E-12//AT5G40780| LHT1; amino acid transmembrane transporter	Unigene118961_All//7.00E-55//gi|48778405|gb|CO087771.1|CO087771			GO:0006865//GO:0015171//GO:0031224//GO:0043231	nr -
Unigene118962_All	225	0.00	0.00	1.42	2.21	3.21	1.81	4.70	0.74	5.49	null	null		1.05E+01	4.53E-02		5.38E-01	5.64E-01		-2.94E-01	7.57E-01		-2.66E+00	2.25E-04	Down	2.25E-01	6.66E-01				Unigene118962_All//7.00E-91//CO094931				

Unigene11897_All	880	7.02	6.56	3.45	5.55	4.62	4.36	1.63	0.85	1.72	-9.60E-02	6.91E-01		-1.02E+00	4.34E-05	Down	-2.65E-01	3.69E-01		-3.47E-01	2.31E-01		-9.34E-01	6.12E-02		7.46E-02	8.85E-01		Unigene11897_All//gi|115464025|ref|NP_001055612.1|//5.00E-50//Os05g0428400 [Oryza sativa Japonica Group] >gi|113579163|dbj|BAF17526.1| Os05g0428400 [Oryza sativa Japonica Group]	Unigene11897_All//1.00E-46//AT1G44760| universal stress protein (USP) family protein	Unigene11897_All//0.00E+00//gi|164275001|gb|ES823111.1|ES823111			GO:0050896	nr -
Unigene118982_All	601	2.87	13.49	8.51	0.83	3.09	2.03	0.98	0.56	1.86	2.23E+00	1.97E-19	Up	1.57E+00	1.01E-07	Up	1.90E+00	1.74E-04	Up	1.29E+00	4.33E-02		-8.24E-01	3.48E-01		9.14E-01	1.03E-01								
Unigene118984_All	207	3.79	5.63	3.86	18.77	10.15	3.49	2.45	2.42	5.20	5.70E-01	3.77E-01		2.54E-02	9.94E-01		-8.87E-01	2.22E-03		-2.43E+00	2.00E-11	Down	-1.66E-02	1.02E+00		1.08E+00	5.98E-02				Unigene118984_All//2.00E-07//BF278120				
Unigene118985_All	348	0.00	0.00	0.00	0.00	0.00	0.00	0.00	0.00	1.49	null	null		null	null		null	null		null	null		null	null		1.05E+01	6.14E-04	Up							
Unigene118989_All	248	0.42	0.41	0.86	0.00	0.69	0.00	1.70	2.36	6.75	-4.62E-02	9.90E-01		1.03E+00	5.92E-01		9.44E+00	2.27E-01		null	null		4.69E-01	6.13E-01		1.98E+00	5.62E-05	Up			Unigene118989_All//2.00E-54//TC130711				
Unigene118996_All	397	0.00	0.00	0.00	0.00	0.00	0.00	0.00	0.95	6.32	null	null		null	null		null	null		null	null		9.89E+00	6.86E-03		1.26E+01	5.71E-18	Up			Unigene118996_All//5.00E-07//TC153841				
Unigene118999_All	361	0.00	0.28	1.03	0.28	0.00	0.00	0.00	0.81	4.64	8.13E+00	3.89E-01		1.00E+01	2.50E-02		-8.11E+00	3.15E-01		-8.11E+00	4.02E-01		9.66E+00	2.35E-02		1.22E+01	5.04E-12	Up							
Unigene119_All	519	0.40	1.86	0.00	4.22	2.26	2.09	4.81	1.53	1.23	2.20E+00	6.58E-03		-8.66E+00	1.46E-01		-9.05E-01	2.79E-02		-1.02E+00	1.93E-02		-1.65E+00	1.62E-05	Down	-1.97E+00	4.57E-07	Down	Unigene119_All//gi|60327635|gb|AAX19057.1|//3.00E-84//class III HD-Zip protein 8 [Populus trichocarpa]	Unigene119_All//1.00E-82//AT4G32880| ATHB-8 (HOMEOBOX GENE 8); DNA binding / transcription factor	Unigene119_All//0.00E+00//TC178623	HB			nr -
Unigene1190_All	3046	2.08	4.76	2.60	2.91	2.87	2.49	2.75	4.67	26.11	1.19E+00	1.69E-14	Up	3.26E-01	1.46E-01		-1.97E-02	9.32E-01		-2.25E-01	3.07E-01		7.63E-01	1.12E-08		3.25E+00	0.00E+00	Up	Unigene1190_All//gi|75170629|sp|Q9FHK4.1|Y5519_ARATH//1.00E-22//RecName: Full=Uncharacterized protein At5g05190 >gi|30725434|gb|AAP37739.1| At5g05190 [Arabidopsis thaliana]	Unigene1190_All//2.00E-17//AT5G05190| unknown protein	Unigene1190_All//0.00E+00//DT465398				nr +
Unigene11900_All	1916	11.80	9.18	7.87	1.35	1.91	2.62	14.85	20.00	47.39	-3.62E-01	1.78E-03		-5.85E-01	8.23E-07		4.95E-01	1.36E-01		9.53E-01	3.53E-04		4.30E-01	2.27E-08		1.67E+00	1.17E-180	Up	Unigene11900_All//gi|240256105|ref|NP_567845.4|//0.00E+00//ATDGK7 (Diacylglycerol kinase 7); diacylglycerol kinase [Arabidopsis thaliana]	Unigene11900_All//0.00E+00//AT4G30340| ATDGK7 (Diacylglycerol kinase 7); diacylglycerol kinase	Unigene11900_All//0.00E+00//TC155811		ko04070|Phosphatidylinositol signaling system//ko00564|Glycerophospholipid metabolism//ko00561|Glycerolipid metabolism	GO:0007186//GO:0016301	nr -
Unigene119002_All	236	0.00	0.00	0.00	0.00	0.00	0.00	10.75	1.42	11.31	null	null		null	null		null	null		null	null		-2.92E+00	1.70E-10	Down	7.34E-02	8.38E-01		Unigene119002_All//gi|1181331|emb|CAA54678.1|//7.00E-07//calnexin [Zea mays]		Unigene119002_All//1.00E-40//TC158845		ko04141|Protein processing in endoplasmic reticulum//ko04145|Phagosome		nr -
Unigene119003_All	232	2.26	2.18	2.30	6.23	3.41	0.58	0.36	0.18	1.72	-4.63E-02	9.76E-01		2.54E-02	9.99E-01		-8.67E-01	1.04E-01		-3.41E+00	3.16E-06	Down	-1.02E+00	7.26E-01		2.24E+00	6.43E-02								
Unigene119005_All	428	0.00	0.00	0.00	0.00	0.00	0.00	0.00	0.00	1.86	null	null		null	null		null	null		null	null		null	null		1.09E+01	7.36E-06	Up	Unigene119005_All//gi|115473125|ref|NP_001060161.1|//4.00E-09//Os07g0592200 [Oryza sativa Japonica Group] >gi|113611697|dbj|BAF22075.1| Os07g0592200 [Oryza sativa Japonica Group]	Unigene119005_All//4.00E-09//AT5G43100| aspartyl protease family protein					nr -
Unigene119015_All	389	0.00	0.00	0.00	0.26	0.00	0.00	0.43	0.21	2.87	null	null		null	null		-8.00E+00	3.15E-01		-8.00E+00	4.02E-01		-1.02E+00	6.15E-01		2.72E+00	8.54E-05	Up			Unigene119015_All//3.00E-73//DW485235				
Unigene119027_All	328	1.28	6.49	1.62	0.00	0.00	0.00	0.00	0.00	0.00	2.35E+00	6.59E-06	Up	3.47E-01	7.06E-01		null	null		null	null		null	null		null	null		Unigene119027_All//gi|148608655|gb|ABQ95657.1|//2.00E-07//auxin hydrogen symporter [Malus x domestica]	Unigene119027_All//2.00E-08//AT5G65980| auxin efflux carrier family protein	Unigene119027_All//4.00E-149//CO124843				nr +
Unigene119030_All	339	0.00	0.60	0.16	0.15	0.20	0.00	1.12	0.99	4.11	9.22E+00	1.37E-01		7.30E+00	6.38E-01		4.68E-01	9.59E-01		-7.20E+00	6.39E-01		-1.87E-01	8.57E-01		1.87E+00	4.97E-04	Up							
Unigene119032_All	428	0.00	0.12	0.12	0.00	0.00	0.00	0.10	0.10	2.70	6.89E+00	6.19E-01		6.96E+00	6.38E-01		null	null		null	null		-1.62E-02	1.01E+00		4.77E+00	3.27E-07	Up			Unigene119032_All//3.00E-27//gi|109875078|gb|DW504052.1|DW504052				ESTscan +
Unigene119047_All	946	0.00	0.00	0.00	0.05	0.00	0.00	0.27	0.04	4.46	null	null		null	null		-5.72E+00	5.58E-01		-5.72E+00	6.39E-01		-2.60E+00	1.44E-01		4.06E+00	9.27E-23	Up	Unigene119047_All//gi|18025342|gb|AAK38482.1|//2.00E-63//beta-D-xylosidase [Hordeum vulgare]	Unigene119047_All//4.00E-59//AT3G19620| glycosyl hydrolase family 3 protein	Unigene119047_All//4.00E-80//gi|48751990|gb|CO082509.1|CO082509		ko00500|Starch and sucrose metabolism//ko00940|Phenylpropanoid biosynthesis//ko00460|Cyanoamino acid metabolism		nr +
Unigene119068_All	258	0.41	4.32	0.83	1.74	2.67	3.50	0.98	0.32	1.54	3.41E+00	1.24E-04	Up	1.03E+00	5.92E-01		6.19E-01	5.30E-01		1.01E+00	1.96E-01		-1.60E+00	3.11E-01		6.51E-01	5.45E-01								
[truncated: 1,513,319 more chars]
